# Analysis of Argonaute Complex Bound mRNAs in DU145 Prostate Carcinoma Cells Reveals New miRNA Target Genes

**DOI:** 10.1155/2017/4893921

**Published:** 2017-01-05

**Authors:** Jaroslaw Szczyrba, Volker Jung, Michaela Beitzinger, Elke Nolte, Sven Wach, Martin Hart, Sandra Sapich, Marc Wiesehöfer, Helge Taubert, Gunther Wennemuth, Norbert Eichner, Thomas Stempfl, Bernd Wullich, Gunter Meister, Friedrich A. Grässer

**Affiliations:** ^1^Institute of Virology, Saarland University Medical School, Kirrbergerstrasse, Haus 47, 66421 Homburg/Saar, Germany; ^2^Institute of Anatomy, University Hospital Essen, Hufelandstrasse 55, 45147 Essen, Germany; ^3^University Clinic of Urology, Saarland University Medical School, Kirrbergerstrasse, 66421 Homburg/Saar, Germany; ^4^Biochemistry Center Regensburg (BZR), Laboratory for RNA Biology, 93053 Regensburg, Germany; ^5^University Clinic of Urology, Friedrich-Alexander-University Erlangen-Nürnberg, Krankenhausstrasse 12, 91054 Erlangen, Germany; ^6^Center of Excellence for Fluorescent Bioanalytics (KFB), University of Regensburg, 93053 Regensburg, Germany

## Abstract

Posttranscriptional gene regulation by microRNAs (miRNAs) contributes to the induction and maintenance of prostate carcinoma (PCa). To identify mRNAs enriched or removed from Ago2-containing RISC complexes, these complexes were immunoprecipitated from normal prostate fibroblasts (PNFs) and the PCa line DU145 and the bound mRNAs were quantified by microarray. The analysis of Ago complexes derived from PNFs or DU145 confirmed the enrichment or depletion of a variety of mRNAs already known from the literature to be deregulated. Novel potential targets were analyzed by luciferase assays with miRNAs known to be deregulated in PCa. We demonstrate that the mRNAs of the death effector domain-containing protein (DEDD), the tumor necrosis factor receptor superfamily, member 10b protein (TNFRSF10B), the tumor protein p53 inducible nuclear protein 1 (TP53INP1), and the secreted protein, acidic, cysteine-rich (SPARC; osteonectin) are regulated by miRNAs miR-148a, miR-20a, miR-24, and miR-29a/b, respectively. Therefore, these miRNAs represent potential targets for therapy. Surprisingly, overexpression of miR-24 induced focus formation and proliferation of DU145 cells, while miR-29b reduced proliferation. The study confirms genes deregulated in PCa by virtue of their presence/absence in the Ago2-complex. In conjunction with the already published miRNA profiles of PCa, the data can be used to identify miRNA-regulated mRNAs.

## 1. Introduction

Prostate carcinoma (PCa) is a leading cause of cancer morbidity and mortality in men worldwide [1]. The etiology of PCa is complex and numerous genetic [2] and epigenetic alterations [3] have previously been reported.

The elucidation of possible functional consequences of genetic or epigenetic alterations is still a great challenge. MicroRNAs (miRNAs) are short noncoding RNAs of 18–23 nt length that posttranscriptionally regulate gene expression by binding to mRNA targets to inhibit protein synthesis (reviewed in [4]). In the context of tumorigenesis, miRNAs can function both as tumor suppressors and as oncogenes [5]. They are bound to their target mRNAs in the RISC complex, which contains an Argonaute (Ago) protein that is tethered to the mRNA by the miRNA. Binding of the miRNA to the target initially inhibits protein synthesis by reducing translation but eventually leads to degradation of the mRNA target [6].

The expression of miRNAs in PCa has been studied extensively (reviewed in [7]). We had previously described the miRNA profile of PCa at different stages of malignancy and could identify novel targets of miRNAs deregulated in PCa [8–12]. Although there has been substantial progress in understanding the way how miRNAs are processed and loaded into Ago protein complexes (reviewed in [6, 13]), it is still not fully understood if or how pre-miRNAs are selected for loading into Ago complexes and if a deregulated miRNA expression ultimately leads to an altered abundance of this miRNA in the active miRISC complexes. After construction of the active miRISC, the miRNA component guides the active miRISC complex to its cognate mRNA target. There are numerous additional factors that may influence the final interaction of miRISC with the target mRNA (reviewed in [14]). miRNA-regulated mRNAs can be isolated by immunoprecipitation of the Ago protein(s) [15–18]. Therefore, the aim of our study was to investigate whether the altered miRNA expression in PCa leads to an altered spectrum of miRISC-associated mRNAs in cancer cells.

## 2. Materials and Methods

### 2.1. Cell Lines

The human PCa cell lines DU145 and LNCaP as well as HEK293T cells were purchased from the German collection of microorganisms and cell cultures (DSMZ, Braunschweig, Germany). Primary human normal prostate fibroblasts (PNF-08) were kindly provided by Prof. Gerhard Unteregger (Dept. of Urology, University of Saarland Medical School). All cells were cultured as previously described [19]. For precipitation of the Ago2 containing RISC complex, extract of approx. 2 × 10^8^ PNF-08 and DU145 cells were used (see below).

### 2.2. RNA Extraction

Extracts from cell lines were generated using TRIzol (Life Technologies, Darmstadt, Germany). Extraction of total RNA and protein was carried out according to the TRIzol manual.

### 2.3. Analysis of Ago2-Bound mRNAs

The Ago2-containing RISC complexes were isolated from the DU145 and PNF cells as previously described [20]. Shortly, the DU145 and PNF-08 cells were lysed in a buffer consisting of 0.5% NP40, 150 mM KCl, 25 mM Tris pH 7.5, 2 mM EDTA, and 0.5 mM DTT. After centrifugation at 15.000 ×g at 4°C for 20 min, the supernatant was incubated for 4 h at 4°C with protein G Sepharose (GE Healthcare, Solingen, Germany) that was previously saturated with Ago2-specific antibody 11A9 [16]. The beads were washed repeatedly with a buffer containing 300 mM NaCl, 50 mM Tris pH 7.5, 5 mM MgCl_2_, and 0.05% NP40. The beads were then incubated for 15 min at 65°C with Proteinase K digestion buffer (300 mM NaCl, 200 mM Tris-HCl, pH 7.5, 25 mM EDTA, 2% SDS, 20 mg/mL Proteinase K). The RNA was then extracted with TRIzol according to the TRIzol manual.

### 2.4. Microarray Analysis

Sample preparation for microarray hybridization was carried out as described in the Ambion WT Expression Kit Protocol (Thermo Scientific) and the Affymetrix WT Terminal Labeling and Hybridization User Manual (Affymetrix, Inc., Santa Clara, CA, USA).  In brief, 200–300 ng of extracted RNA were reverse transcribed, using an rRNA-depleted random primer mix, as provided in the Ambion WT Expression Kit, followed by an in vitro transcription reaction. 8–12 *μ*g of in vitro transcribed antisense RNA were purified and reverse transcribed into dUTP-containing sense-strand- (ss-) cDNA. Purified ss-cDNA was fragmented using a combination of uracil DNA glycosylase (UDG) and apurinic/apyrimidinic endonuclease 1 (APE 1), followed by terminal labeling with biotin. This process yielded between 1 and 3 *μ*g fragmented and labeled ss-cDNA, which were hybridized to Affymetrix Human Gene 1.1 ST PEG plate arrays. Hybridization, washing, staining, and array scanning were performed with a GeneTitan® instrument (Affymetrix).

Summarized probe set signals were calculated by applying the RMA [21] algorithm as implemented in the Affymetrix GeneChip Expression Console Software. The result file was exported in  .txt format, and fold change/significance calculations were done in Microsoft Excel.

### 2.5. Data Analysis

First, a literature research has been performed for mRNAs which were enriched or depleted at least threefold in the Ago2 complexes of DU145 in comparison to PNF-08 to identify genes involved in prostate tumorigenesis or in cancer development and progression in general. The genes found were checked for their expression in prostatic normal and tumor tissue using an online database for mRNA expression from micro arrays* oncomine.org*. The next step was to analyze the 3′ untranslated regions (3′UTRs) of selected genes for putative miRNA target sites (Supplementary Table S2 in Supplementary Material available online at https://doi.org/10.1155/2017/4893921). Prediction of miRNA target sites in the 3′UTRs was performed by online algorithms* targetscan* (http://www.targetscan.org/) and microRNA.org using binding of the “seed sequence,” binding energy, and conservation of binding sites as criteria. For further analysis, putative binding miRNAs were selected which have been previously published as deregulated in PCa.

### 2.6. Quantitative Real-Time PCR (qRT-PCR) Analysis of mRNA Expression

cDNA synthesis was performed with the DyNAmo cDNA Synthesis Kit (Finnzymes Oy, Vantaa, Finland) using 200 ng of total RNA and random hexamer primers. Real-time PCRs were performed in triplicate using TaqMan gene expression assays and TaqMan reagents (Applied Biosystems, Foster City, CA, USA) according to the manufacturer's protocols with the StepOnePlus Real-Time PCR System (Life Technologies, Darmstadt, Germany). Sequence-specific primers and fluorescence-labeled probes complementary to EI24, YWHAE, TFRC, CORO1C, S100A16, NLK, RAB1B, DEDD, CUL5, and PRDX3 and the endogenous reference GAPDH were used. All PCRs were measured in triplicate in a final volume of 10 *μ*L containing 1x TaqMan Universal PCR Master Mix (No Amperase UNG) and 50 ng cDNA. Thermal cycling conditions were chosen according to the manufacturer's recommendations. As a reference sample cDNA prepared from DU145 cells was included in every reaction plate. Calculation of relative RNA expression levels by applying the ΔΔCt method [22] was performed using the StepOne software v2.0 (Applied Biosystems, Foster City, CA, USA).

### 2.7. Dual Luciferase Assays

Dual luciferase assays employing 3′UTR reporters in pMIR-RNLTK (a dual firefly and renilla luciferase vector) were carried out in HEK293T cells as described [11]. A total of 10^5^ HEK293T cells were seeded in each cavity of a 24-well tissue culture dish and transfected using Nanofectin transfection reagent (GE Healthcare, Solingen, Germany) with 0.2 *μ*g/well reporter vector and a total of 0.8 *μ*g pSG5-miRNA effector plasmid(s). Luciferase assays were performed 48 hours after transfection using the Dual Luciferase Reporter Assay System according to the manufacturer's instructions (Promega, Madison, WI, USA). Assays were conducted in duplicate and carried out at least four times for each effector/reporter pair.

### 2.8. Plasmids

The pSG5 plasmids for the expression of miRNAs miR-24 and -29b [11], miR-148a [10], miR-200c and -375 [12] have been described previously. For the present study, miRNAs miR-15b, 20a, -21, and -29a were PCR amplified from human genomic DNA and inserted into the vector pSG5 (Agilent Technologies) using the primers shown in Supplementary Table S3. The 3′UTRs of the genes of the death effector domain-containing protein (DEDD), the tumor necrosis factor receptor superfamily, member 10b protein (TNFRSF10B), the tumor protein p53 inducible nuclear protein 1 (TP53INP1), the secreted protein, acidic, cysteine-rich (SPARC; osteonectin), the Abelson interactor 2 protein (ABI2), and the periredoxin 3 (PRDX3) protein were PCR amplified and inserted into the pMIR-RNLTK vector using the primers shown in Supplementary Table  S3. When necessary, the potential binding sites for miRNAs were mutated by PCR; the primers used for the mutations are also shown in Supplementary Table S3.

### 2.9. Western Blotting

For western blotting, 2 × 10^5^DU145 cells grown in 10 cm dishes were transfected with 10 *μ*g of plasmid DNA using jetPRIME (Polyplus transfection, Sélestat, France). After 48 h cells were lysed with 2-fold concentrated lysis buffer (130 mM Tris/HCl, 6% SDS, 10% 3-mercapto-1,2-propandiol, 10% glycerol) and 30 *μ*g of extracted proteins were separated on 9% tricine gels and transferred to nitrocellulose membranes (Invitrogen, Carlsbad, CA, USA). Membranes were blocked with TBST/5% BSA for 30 min and incubated with primary antibodies over night at 4°C. After washing three times with TBST, the membranes were incubated with HRP-conjugated secondary antibodies for 1 h at room temperature. Protein bands were detected with ECL reagent (GE Healthcare) using FujiFilm LAS-3000 imaging system (FujiFilm, Tokyo, Japan). Primary rabbit anti-SPARC antibody was purchased from Cell Signaling Technology (#D10F10, Danvers, MA, USA), rat anti-TP53INP1 antibody was a generous gift from Dr. Alice Carrier (Centre de Recherche en Cancérologie de Marseille, France), and secondary HRP-conjugated goat anti-rabbit IgG (#31460) and goat anti-rat IgG (#31470) antibodies were purchased from Thermo Scientific Pierce (Fisher Scientific, Schwerte, Germany).

### 2.10. Colony Formation Assay

2 × 10^6^ DU145 cells were seeded in 10 cm dishes and transfected with 10 *μ*g plasmid DNA using jetPRIME (Polyplus transfection, Sélestat, France). 24 hours after transfection, the cells were detached by trypsin, resuspended in 10 mL medium, seeded in 6-well plates (2000 cells/well), and cultured for 8 days. After medium replacement, cultures were stained with 0.4% cristal violet, fixed with 4% paraformaldehyde for 30 minutes, and washed 3 times with PBS. Wells were photographed and densitometrically analysed with ImageJ 1.48v (National Institute of Health, USA).

### 2.11. Cell Proliferation Assay

1.5 × 10^5^ DU145 cells were seeded in 6-well plates, transfected with 2 *μ*g plasmid DNA using jetPRIME (Polyplus transfection, Sélestat, France) and cultivated for 24–72 h. For measuring cell numbers on days 0 to 3 after transfection, cells were detached with trypsin and resuspended in 1 mL medium. Cell numbers were determined with CASY 1 cell counter (Schärfe System, Reutlingen, Germany) in cells/mL.

### 2.12. Statistics

Statistical evaluation of the luciferase assays was performed with SigmaPlot 10 (Systat) using Student's *t*-test analysis. All statistical tests were performed as two-sided and *p* values of <0.05 were considered as significant.

## 3. Results

To identify mRNAs differentially enriched or excluded from the RISC complex, Ago-2 was immunoprecipitated from extracts derived from normal prostate fibroblasts (PNF-08) and from the PCa cell line DU145 using the previously described Ago-2 antibody 11A9 and an appropriate isotype control [15, 16]. The mRNAs present in the Ago-precipitate were quantitated by microarray analysis. The relative abundance of RISC-associated mRNAs in the two cell lines are presented in Supplementary Table S1. Twenty-four mRNAs that exhibited a pronounced enrichment or depletion in the Ago complexes of DU145 cell are presented in Table 1. Supplementary Table S4 lists the functions (if known) of the genes in Table 1 and if they are potential or known targets of miRNAs deregulated in PCa. For instance, the highest abundance in the Ago complexes of DU145 cells was observed for laminin alpha 3 (LAMA3). Expression of this gene is, among other members in its family, significantly reduced in prostate carcinoma [23] in line with being a potential target for miRNAs miR-20a and miR-106a (http://www.targetscan.org/), both of which are up-regulated in prostate carcinoma [8, 19]. In addition to the LAMA3 mRNA, the highly RISC-enriched adenylate cyclase 3 (ADCY3) mRNA is a potential target for the upregulated miRNAs miR-25 and miR-27a/b. Conversely, the Thy-1 cell surface antigen (THY1) has been shown to be upregulated in PCa [24]. Accordingly, the THY1 mRNA level was reduced in the Ago complex of the DU145 cells.

**Figure 1:**
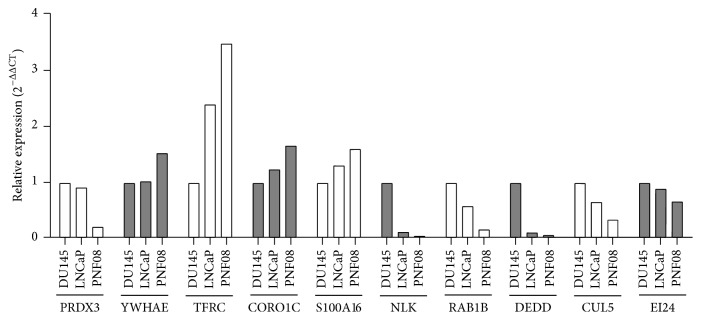
Quantification of mRNA expression in primary normal prostate (PNF-08) cells and the DU145 and LNCaP PCa cell lines. The expression of ten mRNAs that were assumed to be elevated or reduced according to their presence or absence in the Ago complex was assessed by qRT-PCR. YWHAE, TRFC, CORO1C, and PRDX3 were predicted to be elevated while, NLK, RAB1B, DEDD, CUL5, EI24, and S100A16 were predicted to be reduced in PCa cells as compared to PNF-08 cells.

We then selected 10 mRNAs that, based on our results, suggest a possible deregulation in PCa. We analyzed by qRT-PCR the relative expression of these genes in PNF-08 cells in comparison with DU145 and LNCaP cells. Six mRNAs were selected that exhibited an enrichment in the Ago complexes from PCa cells and were therefore potentially downregulated in cell lines and tumors: Etoposide induced 2.4 mRNA (EI24, alias p53-induced gene 8 protein), S100 calcium binding protein A16 (S100A16), Nemo-like kinase (NLK), RAS oncogene family member 1B (RAB1B), death effector domain-containing protein (DEDD), and cullin 5 (CUL5). On the other end, we selected mRNAs that showed depletion from Ago complexes of PCa cells and therefore are potentially overexpressed in PCa cells and tumors. These were tyrosine 3-Monooxygenase (YWHAE, aka 14-3-3 protein epsilon), transferrin receptor (TRFC), coronin, actin binding protein 1C (CORO1C), and periredoxin 3 (PRDX3). The results of the analysis are shown in Figure 1.

We initially expected that mRNAs enriched in Ago complexes undergo degradation by deadenylation or by 5′-decapping which would result in a reduced overall mRNA content in whole-cell RNA preparations. Interestingly, the results of quantitative PCR analyses demonstrated that mRNAs enriched in the Ago complexes of PCa cells were present at higher amounts in whole-cell RNA and mRNAs depleted from Ago complexes of PCa cells exhibited a reduced amount. Only two out of the selected 10 mRNAs (S100A16 and PRDX3) followed our initial expectation. These results demonstrate that an enrichment of mRNAs in Ago complexes does not necessarily lead to an enhanced degradation of the respective mRNA.

EI24, as an Etoposide-inducible gene, was chosen because Etoposide has recently been suggested as one component of a combination chemotherapy treatment of advanced PCa [25]. YWHAE is among the 14-3-3 proteins that regulate the ETV1 transcription factor in PCa [26]. TRFC was shown originally to be significantly induced in DU145 cells [27], CORO1C/coronin was found to be induced in androgen-insensitive PCa [28] and S100A16 appears to be upregulated only in metastatic tumor cells [29].

NLK acts as a *β*-catenin pathway inhibitor through phosphorylation and degradation of TCF/LEF transcription factors [30]. NLK and DEDD are suggested to induce apoptosis [31, 32] and loss of RAB1B was shown to enhance the aggressiveness of breast carcinoma [33]. A reduction of CUL5 expression was found, for instance, in breast cancer [34], but so far not in prostate carcinoma. Finally, PRDX3 was chosen as it was previously found to be upregulated in primary PCa [35]. For this gene, our initial assumption holds true, where a reduced abundance of the PRDX3 mRNA in Ago complexes of PCa cells results in an elevated mRNA content in whole cells. In addition, the 3′UTRs of DEDD and PRDX3 were analyzed as potential targets for miRNAs known to be deregulated in PCa (see below).

To gain additional insight into the miRNA-based regulation of tumor relevant genes, we selected genes form the list of Ago-associated mRNAs for their potential regulation by miRNAs (refer to Supplementary Table S2). For this analysis, we chose DEDD, the tumor necrosis factor receptor superfamily, member 10b protein (TNFRSF10B), the tumor protein p53 inducible nuclear protein 1 (TP53INP1), the secreted protein, acidic, cysteine-rich (SPARC; osteonectin), the Abelson interactor 2 protein (ABI2) and the periredoxin 3 (PRDX3) gene.

**Figure 2:**
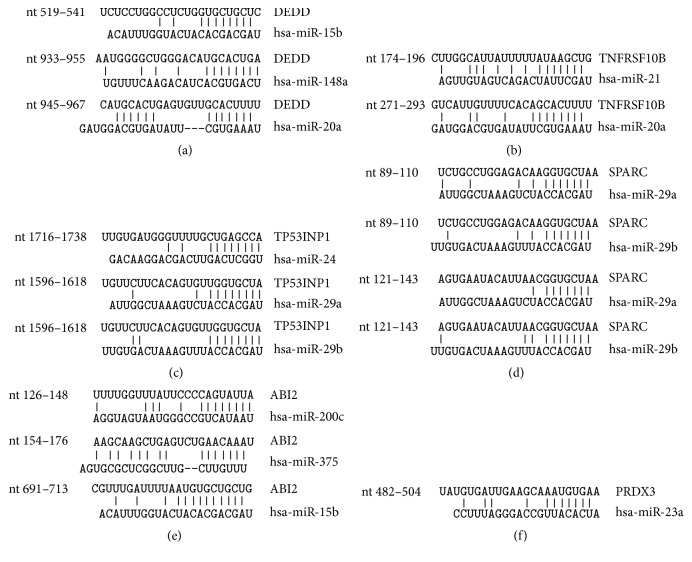
Predicted miRNA target sites in 3′UTRs. The 3′UTR regions of DEDD (a), TNFRSF10B (b), TP53INP1 (c), SPARC (d), ABI2 (e), and PRDX3 (f) are depicted. A schematic representation of the predicted miRNAs and their interaction site(s) is shown.

**Figure 3:**
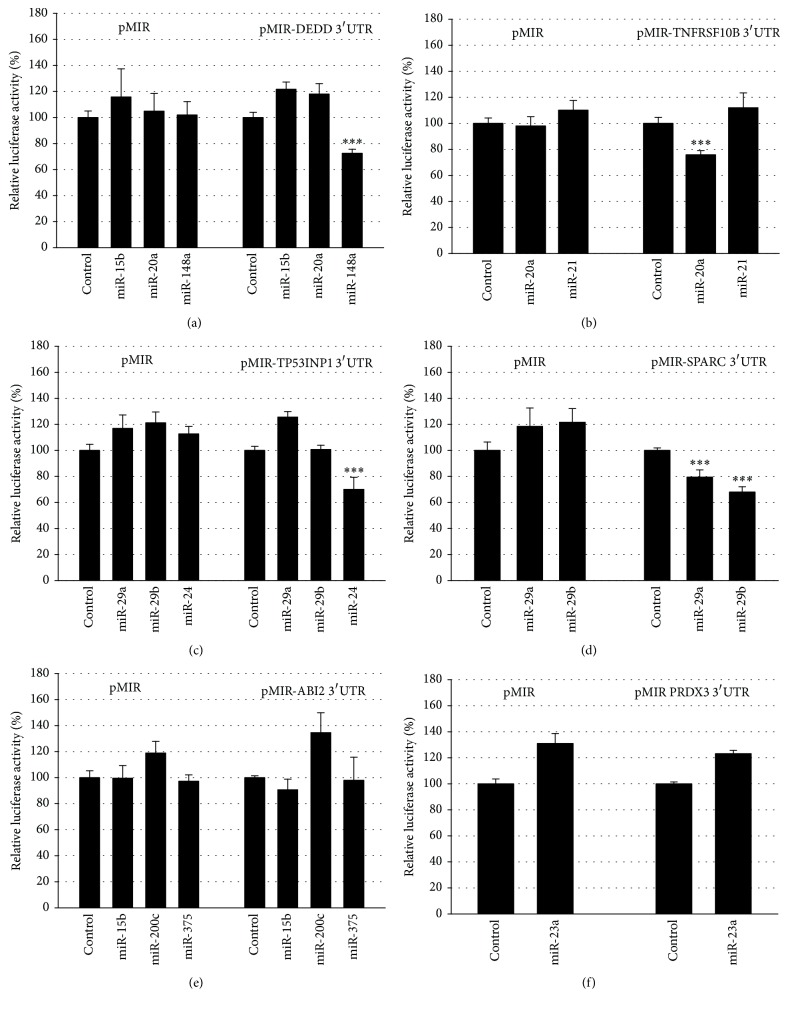
Response of 3′UTRs towards miRNAs. The DEDD (a), TNFRSF10B (b), TP53INP1 (c), SPARC (d), ABI2 (e), and PRDX3 (f) 3′UTRs were cloned behind the luciferase reporter gene of the pMIR vector. The reporter gene constructs were expressed with the miRNA expression construct or with the empty pSG5 vector in the indicated combinations. Results represent the mean of 4 independent experiments performed in duplicate. ^*∗∗∗*^ *p* < 0.001.

These mRNAs are potential targets for previously identified PCa-deregulated miRNAs miR-15b, miR-20a, and miR-148a (DEDD), miR-20a and miR-21 (TNFRSF10B), miR-24, and -29a/b (TP53INP1), miR-29a/b (SPARC), miR-15b, miR-200c, and miR-375 (ABI2), and miR-23a (PRDX3). The potential binding sites of the miRNAs in the 3′UTRs of analyzed mRNAs are depicted in Figure 2.  Figure 3 shows the negative regulatory effects of the miRNAs towards the reporter gene under the regulatory control of their responsive 3′UTRs. We could demonstrate negative regulatory effects of miR-148a towards the DEDD 3′UTR (Figure 3(a)), of miR-20a towards the TNFRSF10B 3′UTR (Figure 3(b)), of miR-24 towards the TP53INP1 3′UTR (Figure 3(c)), and of both miR-29a and miR -29b towards the SPARC 3′UTR (Figure 3(d)). The 3′UTRs of ABI2 and PRDX3 were not responsive towards miRNAs miR-375, miR-200c, miR-15b, and miR-23a, respectively, and were thus not analyzed further (Figures 3(e) and 3(f)).

**Figure 4:**
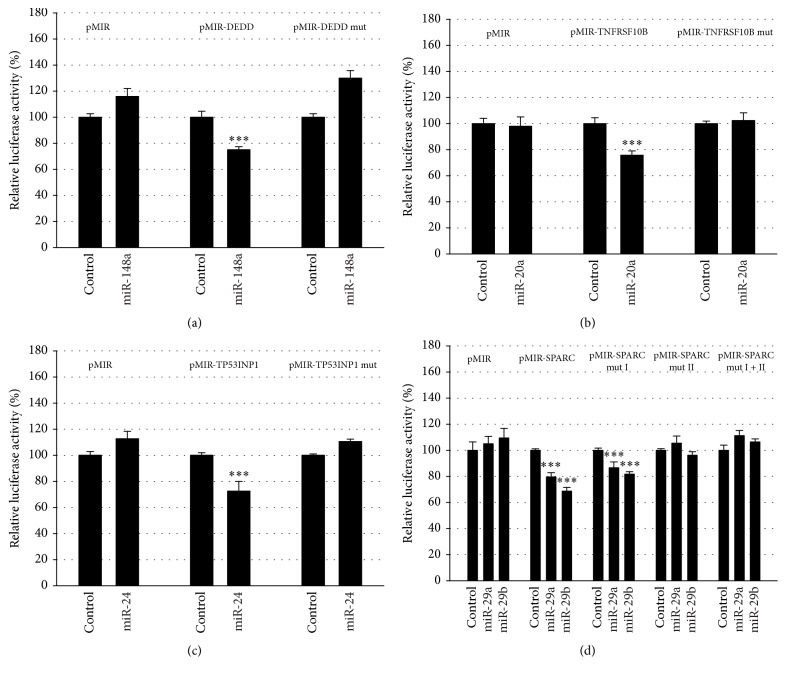
Identification of miRNA binding sites. The potential binding sites for the indicated miRNAs in the 3′UTRs of DEDD (a), TNFRSF10B (b), TP53INP1 (c), and SPARC (d) were mutated by site directed mutagenesis (mut) and coexpressed with the miRNA expression construct or with the empty pSG5 vector in the indicated combinations. Results represent the mean of 4 independent experiments performed in duplicate. ^*∗∗∗*^ *p* < 0.001.

To further validate the results of the negative regulation of the reporter gene by miRNA responsive 3′UTRs, the predicted binding sites for the miRNAs were mutated and analyzed in parallel with the wild-type-constructs. As shown in Figure 4, the mutation of the seed-sequences for miR-148a in the DEDD 3′UTR (Figure 4(a)), for miR-20a in the TNFRSF10B 3′UTR (Figure 4(b)), and miR-24 in the TP53INP1 3′UTR (Figure 4(c)) resulted in loss of responsiveness establishing these mRNAs as targets for the respective miRNAs. In the SPARC 3′UTR (Figure 4(d)), two potential binding sites are present and both of them can potentially be targeted by miR-29a and miR-29b. Mutation of the first binding site (nt 89–110) had no effect on responsiveness, while the mutation of the second binding site (nt 121–143) or of both binding sites abrogated responsiveness to both miR-29a and miR-29b. This demonstrated that the second binding site in the SPARC 3′UTR is a target for miRNAs miR-29a and miR-29b.

**Figure 5:**
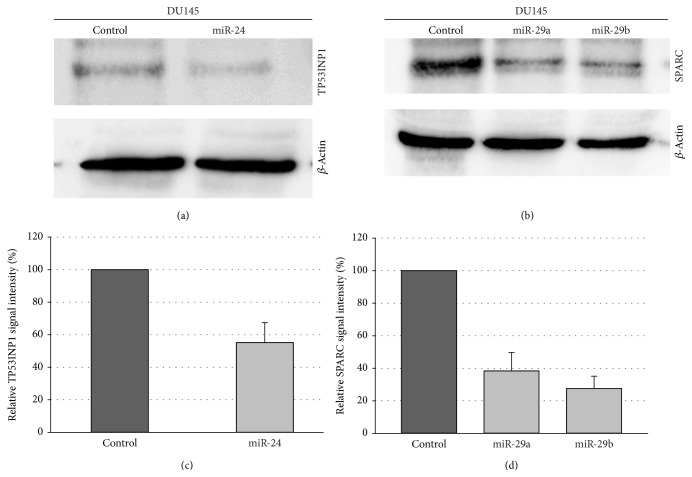
Regulation of TP53INP1 and SPARC protein expression by miR-24 and 29a/b. DU145 cells were transfected either with control vectors or miRNA expression vectors. 48 hours after transfection, the protein expression of (a) TP53INP1 and (b) SPARC was determined by western blot using *β*-actin as loading control. The quantification of the western blots in (c) and (d) represents the relative downregulation as determined in three independent experiments using *β*-actin as loading control.

**Figure 6:**
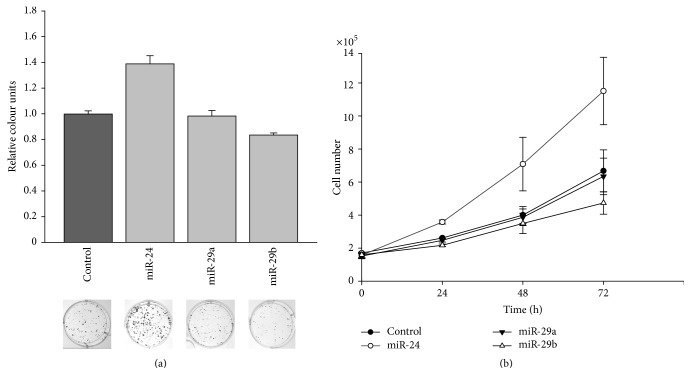
Effect of miR-24 and miR-29a/b on cell growth. DU145 cells were transfected with miRNA expression vectors or control vectors and seeded in a limited cell number. Eight days after seeding, colonies were stained with crystal violet (a). Colony formation was quantified by densitometry analyses. Data show the mean and ± SEM of the densitometry analysis of CFA (*p* < 0.001) of three independent experiments. Cell growth was determined by automated cell counting of parallel experiments at 24, 48, and 72 h after transfection (b).

We then assayed the effect of overexpression of miR-24 and miR-29a/b on the protein levels of the TP53INP1 and SPARC protein, respectively. As shown in Figure 5(a), the TP53INP1 protein was downregulated in DU145 cells after overexpression of miR-24. Likewise, both miRNAs miR-29a and miR-29b reduced the SPARC protein levels (Figure 5(b)). The determination of the relative downregulation is shown in Figures 5(c) and 5(d). TP53INP1 protein was downregulated by about 40%, and reduction of SPARC by miR-29a and miR-29b was approx. 60% and 70%, respectively. Next we tested the effect of the miRNAs on the growth behavior of DU145 cells. We previously showed that these miRNAs are downregulated in primary PCa tissue as compared to nonmalignant prostatic tissue [11, 19]. First, we analyzed the colony formation capacity of DU145 cells after overexpression of the three miRNAs. Interestingly, miR-24 significantly increased both the number of colonies (Figure 6(a)) as well as the colony size. In contrast, miR-29a showed no effect, while miR-29b significantly decreased both the number as well as the size of DU145 cell colonies (Figures 6(a) and 6(b)).

## 4. Discussion

We could demonstrate that a large amount of the mRNAs that we identified as differentially represented in the Ago complexes of DU145 cells have already been described as differentially expressed in primary PCa. Although miRNAs act as the guiding element for the RISC factor, it is still not fully understood how the fate of a targeted mRNA is decided. The binding of miRISC complexes can equally affect only protein translation without measurably altering the mRNA content of the cell. Another source of variability exists in the fact that the level of a given miRNA does not necessarily reflect its presence in the RISC complexes [36]. Furthermore, La Rocca and coworkers have demonstrated that miRNAs in resting tissues are predominantly found in low-molecular weight complexes not associated with mRNA while in growing tissues the majority of miRNAs are found in high molecular weight complexes bound to mRNAs [37]. Here, we used growing cells where this effect might be disregarded.

In addition to the already mentioned LAMA3 mRNA, the highly RISC-enriched adenylate cyclase 3 (ADCY3) mRNA is a potential target for the upregulated miRNAs miR-25 and miR-27a/b [8, 19]. In contrast, overexpression of MICB, the likewise highly enriched mRNA in the Ago complex, is considered to have a tumor-promoting rather than suppressing function [38]. The also enriched succinate dehydrogenase complex, subunit A, flavoprotein (SDHA), a potential tumor suppressor [39], was not found to be differentially expressed in normal versus PCa tissue [40]. However, its presence in the Ago complex might indicate that SDHA is reduced at the protein level in PCa. In line with this assumption, it has been demonstrated, that loss of SDH expression leads to a pseudohypoxia signaling [41]. As further examples, the Ago-IP enriched mRNAs ATM interactor (ATMIN), considered to be a tumor suppressor [42], and the adiponectin receptor 2 (ADIPOR2), a known inhibitor of proliferation [43], are both potential targets for the upregulated miR-200c. However, a recent study described a strong correlation of increased ADIPOR2 expression with poor prognosis of PCa [44]. Of the genes found to be present at reduced levels in the Ago complex (Table 1), the Thy-1 cell surface antigen (THY1) [24], chemokine (C-C motif ligand 2 (CCL2, MCP1)) [45], matrix Gla protein (MGP) [46], periostin (POSTN) [47, 48], secreted protein, acidic, cysteine-rich (SPARC, osteonectin) [49], or cathepsin K (CSTK) [50] have been found to be overexpressed in PCa.

We demonstrate that the 3′UTRs of the DEDD, TNFRSF10B/TRAIL, TP53INP1, and SPARC/osteonectin mRNAs are targets for miR-148a, miR-20a, miR-24, miR-29a, and miR-29b, respectively. In our previous analysis, miR-148a and miR-20a/b were among the strongest induced miRNAs in primary PCa versus normal tissue [8, 19] in line with the high abundance of the DEDD and TNFRSF10B/TRAIL mRNAs in the RISC complexes (Table 1). So far, no association of DEDD with prostate carcinoma has been described but a reduction of this protein might protect PCa cells from undergoing apoptosis. DEDD can attenuate epithelial-mesenchymal transition and acts as an endogenous suppressor of tumor growth and metastasis in human breast cancer [51]. However, overexpression of DEDD was observed in HP16/18 transformed cervical carcinoma cells [52]. Also copy number gain of 1q23.3 which included amplification of DEDD in urothelial tumors was recently described [53] as well as increased DEDD expression in nasopharyngeal carcinoma [54]. A function for DEDD in transcriptional activation via NFKB in addition to its role in apoptosis has been described [55]. The importance of DEDD in PCa should thus to be explored further. Likewise, our results suggest a role for cullin 5 (CUL5) in prostate carcinoma. CUL5 (VACM-1) is located on chromosome 11q22-q23 [56], a genomic region associated with loss of heterozygosity in breast cancer [57], and might therefore be considered as a tumor suppressor. For instance, it inhibits the degradation of tumor suppressor proteins and is present at reduced levels in cell lines derived from breast, colon, melanoma, and non-small lung cancer cell lines; its reduced levels might also desensitize cancer cells during chemotherapy [58].

The downregulation of tumor necrosis factor receptor superfamily, member 10b protein (TNFRSF10B/TRAIL) by upregulated miR-20a [8, 19] is in line with the resistance of the PCa cell lines LNCaP, DU145, or PC3 towards TRAIL/TNFRSF10B-induced apoptosis [59] and its downregulation in PCa [60]. We further show that SPARC/osteonectin and TP53INP1 are targets for miRNAs miR-29a/b and miR-24, respectively. All three miRNAs are downregulated in PCa. In line with these observations, we found reduced protein levels of TP53INP1 and SPARC/osteonectin after overexpression of the respective targeting miRNAs in DU145 cells (Figure 5). SPARC/osteonectin is upregulated and promotes the malignant behavior of PCa [49]. These findings are corroborated by the known function of miRNAs miR-29a/b/c in inhibiting the growth and invasiveness of PCa [61] and are high-lighted by the inhibition of cell growth by miR-29b. Overexpression of TP53INP1 has been observed in PCa and is predictive of biological cancer relapse [62]. Interestingly, overexpression of miR-24 exerted a positive effect on the growth behavior of DU145 cells as determined by colony formation assays (Figure 6). It is possible that the downregulation of miR-24 and its possible tumor-promoting effect works only in cooperation with other factors such as miRNAs. In mammary carcinoma, overexpression of miR-24 enhances proliferation and metastatic potential [63]. Likewise, miR-24 overexpression increases cell proliferation in non-small cell lung cancer [64]. Furthermore, miR-24 targets PTEN in human tongue squamous cell carcinoma which could also explain the increase in growth rate [65]. Conversely, miR-24 functions as a tumor suppressor in nasopharyngeal carcinoma [66].

The data by Flores et al. [36] and La Rocca et al. [37] show that mRNAs are differentially associated with the RISC complex and that the majority of Ago complexes are not associated with mRNAs in resting tissues. The present approach using growing cell lines was chosen to avoid the latter problem. In conjunction with our previously established miRNA expression data, we were able to mechanistically confirm the regulation of four out of six selected genes by miRNAs deregulated in PCa. Our data might be useful for the detection of novel deregulated mRNAs as we provide a list of mRNAs highly enriched in the Ago complexes or those that are only presented at low levels. Our experimental data, however, does not fully support our initial hypothesis that the presence or absence of a mRNA in these complexes affects the expression of the corresponding gene product.

## Supplementary Material

Supplementary Table 1: Relative representation of Ago2-associated mRNAs in DU145 tumor cells as compared to PNF-8 normal fibroblasts. Supplementary Table 2: potential 3'UTR targets for the indicated miRNAs. For tested miRNAs, see Figures 2, 3, and 4. Supplementary Table 3: Primers used for amplification and cloning of the indicted miRNAs and the indicated 3'UTRs as well as primers used for mutation of potential miRNA binding sites. Supplementary Table 4: Genes selected from suplementary Table 1 for further analysis. Shown are a possible functions in (prostae) cancer and potential miRNA that target their respective 3'UTRs.

## Figures and Tables

**Table 1:** Abundance of selected mRNAs in AGO complexes of DU145 PCa cells in comparison to prostate normal fibroblasts (PNF08).

Gene title	Gene symbol	Enrichment
Thy-1 cell surface antigen	THY1	<0.01
Matrix Gla protein	MGP	<0.01
Periostin, osteoblast specific factor	POSTN	<0.01
Secreted protein, acidic, cysteine-rich (osteonectin)	SPARC	<0.01
Chemokine (C-C motif) ligand 2	CCL2	<0.01
Cathepsin K	CTSK	0.059
Tyrosine 3-monooxygenase/tryptophan 5-monooxygenase activation protein, epsilon polypeptide	YWHAE	0.093
Peroxiredoxin 3	PRDX3	0.158
Coronin, actin binding protein, 1C	CORO1C	0.187
Tumor protein p53 inducible nuclear protein 1	TP53INP1	0.214
Transferrin receptor (p90, CD71)	TFRC	0.278

Laminin, alpha 3	LAMA3	6,891
Etoposide induced 2.4 mRNA	EI24	6,813
Adenylate cyclase 3	ADCY3	6,001
Succinate dehydrogenase complex, subunit A	SDHA	5,973
Adiponectin receptor 2	ADIPOR2	5,187
Nemo-like kinase	NLK	4,582
ATM interactor	ATMIN	4,430
RAB1B, member RAS oncogene family	RAB1B	4,293
Death effector domain containing	DEDD	4,019
Tumor necrosis factor receptor superfamily, member 10b	TNFRSF10B	3,635
abl-interactor 2	ABI2	3,267
cullin 5	CUL5	3,144
S100 calcium binding protein A16	S100A16	3,069
