# Supplementary material for: Analysis of Argonaute Complex Bound mRNAs in DU145 Prostate Carcinoma Cells Reveals New miRNA Target Genes
Source: Prostate Cancer. 2017 Jan 5;2017:4893921. doi: 10.1155/2017/4893921 (PMC5253174; doi:10.1155/2017/4893921)
Supplement: Supplementary file 1 — Supplementary Table 1: Relative representation of Ago2-associated mRNAs in DU145 tumor cells as compared to PNF-8 normal fibroblasts. Supplementary Table 2: potential 3'UTR targets for the indicated miRNAs. For tested miRNAs, see Figures 2, 3, and 4. Supplementary Table 3: Primers used for amplification and cloning of the indicted miRNAs and the indicated 3'UTRs as well as primers used for mutation of potential miRNA binding sites. Supplementary Table 4: Genes selected from suplementary Table 1 for further analysis. Shown are a possible functions in (prostae) cancer and potential miRNA that target their respective 3'UTRs. [file 4893921.f1.pdf]

Supplementary Table S2: Potential 3'UTR targets for miRNAs

Target gene	miRNA
DEDD	miR-15b, miR-20a, miR-148a
TNFRSF10B	miR-20, miR-21
TP53INP1	miR-24, miR-29a, miR-29b
SPARC	miR-29a, miR-29b
ABI2	miR-15b, miR-200c, miR-375
PRDX3	miR-23a

Supplementary Table S3

Gene	Forward Primer	Reverse Primer
hsa-miR-20a	ggaattctgcaaaactgatggtggcctg	cgggatccccacagtcagttttgcatgg
hsa-miR-15b	ggaattcgccatggaattgacttggacc	cgggatccgttgctgtatccctgtcacac
hsa-miR-21	gcggatccgaattcaacaggccagaaatgcctggg	agcggatccaagtgccaccagacagaag
hsa-miR-29a	cgaattcAgagacttgagcatctgtgga	cgggatccacatgcaattcaggtcagtga
ABI2	gactagtcctgggaattacgttgagtc	cgagctcatctgggtacactggccat
PRDX3	gactagtagatcacccatgtgtatctg	cgagctctgcaatccctggatttgat
SPARC	gactagtctccttccacagtaccggattc	cgagctcgctgaccacttcccagagaac
SPARC mut1	ctgcctggagacaa**TCGTCGAC**catagatttaagtg	cacttaaatctatg**GTCGACGA**ttgtctccaggcag
SPARC mut2	gtgaatacattaac**CCCACGTG**aaatgaaaattctaacc	ggttagaattttcattt**CACGTGGG**gttaatgtattcac
TNFRSF10B	ggactagtcagactctgccatgtccta	ggactagtcagactctgccatgtccta
TNFRSF10B mut	gtcattgttttcact**CACGTG**gtttatcctaatgtaaatg	catttacattaggataaac**CACGTG**agtgaaaacaatgac
TP53INP1	ggactagtctgccaagtggcaagagtgtggg	cgagctcgacgtcttccactccaaacgctc
TP53INP1 mut1	gttcttcacagtgtc**CACGTG**catgaggccagggtgcag	ctgcaccctggcctcatg**CACGTG**gacactgtgaagaac
TP53INP1 mut2	gttgtgatgggttttgac**GTCGAC**tccactctgactgat	atcagtcagagtgga**GTCGAC**gtcaaaacccatcacaac

Supplementary Table S4

Gene symbol	Gene title	Enrichment	Function in Cancer	References	Function in PCa	References	validated miRNAs	References	predicted miRNAs (target scan: conserved conserved among vertebrates)
LAMA3	laminin, alpha 3	6.891	tumor invasion and metastasis(1, 2,3)	(1) Choi et al. 2014, (2) Tanis et al. 2014, (3) Moller-Levet et al. 2009	tumor invasion and metastasis (1)	(1) Sathyanarayana et al. 2013	miR-17/20 (1), miR-218 (2)	(1) Chang et al. 2013, (2) Kinoshita et al. 2012	miR-17/17-5p/20ab/20b-5p/93/106ab/427/518a-3p/519d, miR-93/93a/105/106a/291a-3p/294/295/302abcde/372/373/428/519a/520be/520acd-3p/1378/1420ac, miR-217, miR-148ab-3p/152, miR-33ab/33-5p
EI24	etoposide induced 2.4 mRNA	6.813	tumor suppressor (1)	(1) Mork et al. 2007	n.a.	n.a.	miR-290/-295 (1)	(1) Zheng et al. 2011	miR-22/22-3p miR-34ac/34bc-5p/449abc/449c-5p miR-183 miR-103a/107/107ab miR-129-5p/129ab-5p miR-142-3p miR-27abc/27a-3p miR-490-3p miR-203
ADCY3	adenylate cyclase 3	6.001	cell migration, invasion, proliferation (1)	(1) Hong et al. 2013	n.a.	n.a.	n.a.	n.a.	miR-25/32/92abc/363/363-3p/367 miR-132/212/212-3p miR-128/128ab miR-205/205ab miR-26ab/1297/4465 miR-383 miR-27abc/27a-3p
SDHA	succinate dehydrogenase complex, subunit A	5.973	invasion (1)	(1) Kim et al. 2014	n.a.	n.a.	n.a.	n.a.	miR-375,miR-31,miR-425/425-5p/489,miR-93/93a/105/106a/291a-3p/294/295/302abcde/372/373/428/519a/520be/520acd-3p/1378/1420ac
ADIPOR2	adiponectin receptor 2	5.187	metastasis (1)	(1) Hiyoshi et al. 2012	PCa progression (1,2)	(1) Rider et al. 2015, (2) Gao Q. and Zheng J. 2015	miR-218 (1) miR-423-3p (2)	(1) Du et al. 2015, (2) Guan et al. 2014	miR-150/5127 miR-218/218a miR-383 miR-19ab miR-200bc/429/548a miR-34ac/34bc-5p/449abc/449c-5p miR-490-3plet-7/98/4458/4500 miR-193/193b/193a-3p miR-214/761/3619-5p miR-190/190ab miR-139-5p miR-29abcd miR-124/124ab/506 miR-223 miR-425/425-5p/489 miR-499-5p miR-17/17-5p/20ab/20b-5p/93/106ab/427/518a-3p/519d miR-375 miR-122/122a/1352 miR-338/338-3p miR-216a miR-26ab/1297/4465 miR-181abcd/4262

NLK	nemo-like kinase	4.582	cancer progression (1)	(1) Li et al. 2013	cancer progression (1)	(1) Emami et al. 2009	miR-199a-3p (1), miR-101 (2), miR-197 (3), miR-92b (4)	(1) Han et al. 2014, (2) Shen et al. 2014, (3) Zou et al. 2015, (4) Wang et al. 2013	miR-199ab-5p miR-101/101ab miR-140/140-5p/876-3p/1244 miR-25/32/92abc/363/363-3p/367iR-208ab/208ab-3p miR-455-5p miR-221/222/222ab/1928 miR-150/5127 miR-23abc/23b-3pmiR-19abmiR-27abc/27a-3pmiR-26ab/1297/4465miR-24/24ab/24-3pmiR-203let-7/98/4458/4500miR-144miR-132/212/212-3pmiR-216amiR-148ab-3p/152miR-499-5pmiR-181abcd/4262miR-375miR-30abcdef/30abe-5p/384-5pmiR-129-5p/129ab-5p
ATMIN	ATM interactor	4.430	tumor suppressor (1)	(1) Loizou et al. 2011	n.a.	n.a.	n.a.	n.a.	miR-124/124ab/506 miR-135ab/135a-5p miR-217 miR-34ac/34bc-5p/449abc/449c-5p miR-138/138ab miR-200bc/429/548a miR-490-3p miR-23abc/23b-3p miR-17/17-5p/20ab/20b-5p/93/106ab/427/518a-3p/519d miR-455-5p miR-130ac/301ab/301b/301b-3p/454/721/4295/3666 miR-93/93a/105/106a/291a3p/294/295/302abcde/372/373/428/519a/20be/520acd-3p/1378/1420ac miR-194 miR-214/761/3619-5p miR-190/190ab miR-181abcd/4262 miR-146ac/146b-5p miR-103a/107/107ab miR-25/32/92abc/363/363-3p/367 miR-19ab miR150/5127 miR-24/24ab/24-3p miR-129-5p/129ab-5p
RAB1B	RAB1B, member RAS oncogene family	4.293	metastasis (1)	(1) Jiang et al. 2015	tumor progression (1)	(1) Abd Elmageed et al. 2014	miR-502 (1)	(1) Zhai et al. 2012	miR-214/761/3619-5p miR-103a/107/107ab miR-184 miR-24/24ab/24-3p miR-125a-5p/125b-5p/351/670/4319 miR-31 miR-18ab/4735-3p miR-33a-3p/365/365-3p miR-148ab-3p/152 miR-138/138ab miR-122/122a/1352 miR-425/425-5p/489 miR-135ab/135a-5p miR-34ac/34bc-5p/449abc/449c-5p miR-490-3p miR-129-5p/129ab-5p miR-455-5p

DEDD	death effector domain containing	4.019	tumor invasion and metastasis(1, 2,3,4)	(1) Lv et al. 2012, (2) Lv et al. 2012, (3) Lv et al. 2012, (4) Stegh et al. 1998	n.a.	n.a.	n.a.	n.a.	miR-24/24ab/24-3p miR-148ab-3p/152 miR-130ac/301ab/301b/301b-3p/454/721/4295/3666 miR-17/17-5p/20ab/20b-5p/93/106ab/427/518a-3p/519d miR-150/5127 miR-15abc/16/16abc/195/322/424/497/1907 miR-122/122a/1352 miR-9/9ab miR-455-5p miR-10abc/10a-5p miR-7/7ab miR-145 miR-132/212/212-3p miR-23abc/23b-3p miR-1ab/206/613 miR-29abcd
TNFRSF10B	tumor necrosis factor receptor superfamily, member 10b	3.635	tumor suppressor (1)	(1) Li et al. 2015	tumor suppressor (1,2)	(1) Van Ophoven et al. 1999, (2) Modur et al. 2002	n.a.	n.a.	miR-145 miR-21/590-5p miR-191 miR-30abcdef/30abe-5p/384-5p miR-218/218a miR-17/17-5p/20ab/20b-5p/93/106ab/427/518a-3p/519d miR-125a-5p/125b-5p/351/670/4319 miR-93/93a/105/106a/291a-3p/294/295/302abcde/372/373/428/519a/520be/520acd-3p/1378/1420ac miR-141/200a miR-193/193b/193a-3p miR-33a-3p/365/365-3p miR-25/32/92abc/363/363-3p/367 miR-23abc/23b-3p miR-216a miR-135ab/135a-5p miR-138/138ab miR-181abcd/4262 miR-130ac/301ab/301b/301b-3p/454/721/4295/3666 miR-24/24ab/24-3p miR-29abcd miR-132/212/212-3p miR-19ab miR-143/1721/4770 miR-128/128ab miR-9/9ab miR-10abc/10a-5p miR-15abc/16/16abc/195/322/424/497/1907 miR-26ab/1297/4465 miR-203 miR-425/425-5p/489 miR-155 miR-200bc/429/548a miR-1ab/206/613
ABI2	abl-interactor 2	3.267	tumor suppressor (1,2)	(1) Dai et al. 1998, (2) Kano et al. 2008	n.a.	n.a.	n.a.	n.a.	miR-193/193b/193a-3p miR-205/205ab miR-187 miR-15abc/16/16abc/195/322/424/497/1907 miR-183 miR-200bc/429/548a miR-7/7ab miR-144 miR-223 miR-122/122a/1352 miR-199ab-5p miR-25/32/92abc/363/363-3p/367 miR-196abc miR-181abcd/4262 miR-217 miR-93/93a/105/106a/291a-3p/294/295/302abcde/372/373/428/519a/520be/520acd-3p/1378/1420ac miR-103a/107/107ab miR-96/507/1271 miR-23abc/23b-3p miR-375 miR-192/215 miR-150/5127 miR-139-5p miR-101/101ab miR-146ac/146b-5p miR-17/17-5p/20ab/20b-5p/93/106ab/427/518a-3p/519d miR-24/24ab/24-3p miR-155 miR-128/128ab miR-1ab/206/613 miR-214/761/3619-5p miR-129-5p/129ab-5p miR-208ab/208ab-3p miR-153 miR-216b/216b-5p miR-27abc/27a-3p miR-203 miR-499-5p miR-503 miR-142-3p miR-143/1721/4770 miR-140/140-5p/876-3p/1244 miR-145 miR-26ab/1297/4465 miR-137/137ab miR-425/425-5p/489 miR-21/590-5p

CUL5	cullin 5	3.144	tumor suppressor (1)	(1) Burnatowska-Hledin et al. 2004	n.a.	n.a.	miR-19a/b (1)	(1) Xu et al. 2012	miR-217 miR-7/7ab miR-148ab-3p/152 miR-182 miR-142-3p miR-33ab/33-5p miR-19ab miR-145 miR-338/338-3p miR-184 miR-124/124ab/506 miR-181abcd/4262 miR-192/215 miR-183 miR-146ac/146b-5p miR-375 miR-30abcdef/30abe-5p/384-5p miR-130ac/301ab/301b/301b-3p/454/721/4295/3666 miR-31 miR-138/138ab miR-221/222/222ab/1928 miR-96/507/1271 miR-214/761/3619-5p miR-205/205ab miR-144 miR-200bc/429/548a miR-132/212/212-3p miR-137/137ab miR-101/101ab miR-26ab/1297/4465 miR-17/17-5p/20ab/20b-5p/93/106ab/427/518a-3p/519d miR-194 miR-129-5p/129ab-5p
S100A16	S100 calcium binding protein A16	3.069	invasion (1) , EMT (2)	(1) Tanaka et al. 2015, (2) Zhou et al. 2014	n.a.	n.a.	n.a.	n.a.	miR-29abcd miR-184 miR-24/24ab/24-3p miR-125a-5p/125b-5p/351/670/4319 miR-96/507/1271 miR-182 miR-17/17-5p/20ab/20b-5p/93/106ab/427/518a-3p/519d

THY1	Thy-1 cell surface antigen	< 0.01	tumor supressor (1,3), apoptosis (2)	(1)Abeysinghe et al. 2003, (2) Fujita et al. 1997, (3) Lung et al. 2005	overexpressed (1)	(1) True et al. 2010	n.a.	n.a.	miR-103a/107/107ab, miR-125a-5p/125b-5p/351/670/4319
MGP	matrix Gla protein	< 0.01	evtl. tumor supressor (1)	(1) Levedakou et al. 1992	evtl. tumor supressor (1)	(1) Levedakou et al. 1992	n.a.	n.a.	miR-143/1721/4770 miR-148ab-3p/152 miR-122/122a/1352 miR-221/222/222ab/1928 miR-135ab/135a-5p miR-33ab/33-5p miR-155 miR-18ab/4735-3p miR-27abc/27a-3p miR-129-5p/129ab-5p miR-200bc/429/548a
POSTN	periostin, osteoblast specific factor	< 0.01	carcinogenesis, invasion, metastasis (1)	(1) Kim et al. 2012	invasion (1)	(1) Kim et al. 2012	n.a.	n.a.	miR-19ab miR-33ab/33-5p miR-218/218a miR-18ab/4735-3p miR-135ab/135a-5p miR-101/101ab miR-144 miR-425/425-5p/489 miR-132/212/212-3p

SPARC	secreted protein, acidic, cysteine-rich (osteonectin)	< 0.01	carcinogenesis (1), metastasis (2), migration (3)	(1) Yamada et al. 2014, (2) Botti et al. 2014, (3) Yusuf et al. 2014	metastasis (1), migration and invasion (2)	(1) Derosa et al. 2012, (2) Jacob et al. 1999	miR-29a/b (1)	(1) Qiu et al. 2014	miR-29abcd miR-31 miR-192/215 miR-455-5p miR-203 miR-17/17-5p/20ab/20b-5p/93/106ab/427/518a-3p/519d miR-150/5127 miR-93/93a/105/106a/291a-3p/294/295/302abcde/372/373/428/519a/520be/520acd-3p/1378/1420ac miR-155 miR-125a-5p/125b-5p/351/670/4319 miR-204/204b/211 miR-219-5p/508/508-3p/4782-3p miR-22/22-3p miR-101/101ab miR-19ab miR-124/124ab/506 miR-205/205ab miR-18ab/4735-3p miR-34ac/34bc-5p/449abc/449c-5p miR-499-5p miR-425/425-5p/489 miR-146ac/146b-5p miR-23abc/23b-3p miR-33ab/33-5p
CCL2	chemokine (C-C motif) ligand 2	< 0.01	metastasis (1), invasion (2), cancer progression (3)	(1) Kitamura et al. 2015, (2) He et al. 2015, (3) Li et al. 2013	migration (1), tumorigenesis (2), metastasis (2)	(1) Lin et al. 2013, (2) Zhang et al. 2010	miR-495 (1), miR-126	(1) Liu et al. 2015, (2) Arner et al. 2012	miR-1ab/206/613 miR-33ab/33-5p miR-22/22-3p miR-124/124ab/506 miR-23abc/23b-3p miR-122/122a/1352
CTSK	cathepsin K	0.059	tumor progression (1,2)	(1) Kleer et al. 2008,(2) Herroon et al. 2013	metastasis progression (1), tumorigenesis (2)	(1) Brubaker et al. 2003, (2) Nägler et al. 2004	n.a.	n.a.	miR-7/7ab miR-17/17-5p/20ab/20b-5p/93/106ab/427/518a-3p/519d miR-130ac/301ab/301b/301b-3p/454/721/4295/3666 miR-19ab miR-128/128ab miR-23abc/23b-3p miR-216a miR-30abcdef/30abe-5p/384-5p
YWHAE	tyrosine 3-monooxygenase/tryptophan 5-monooxygenase activation protein, epsilon polypeptide	0,093	cell survival (1), translocation (2), EMT (3)	(1) Liou et al. 2007, (2) Gremel et al. 2015, (3) Liu et al. 2013	cell survival (1)	(1) Kim et al. 2012	n.a.	n.a.	miR-31 miR-135ab/135a-5p miR-29abcd miR-141/200a let-7/98/4458/4500 miR-490-3p miR-26ab/1297/4465 miR-155
PRDX3	peroxiredoxin 3	0.158	carcinogenesis (1)	(1) Kim et al. 2009	proliferation (1), cell survival (2)	(1) Ummanni et al. 2012, (2) Whitaker et al. 2013	miR-383(1), miR-23b (2)	(1) Li et al. 2013, (2) He et al. 2012	miR-383 miR-31 miR-181abcd/4262 miR-18ab/4735-3p miR-26ab/1297/4465 miR-23abc/23b-3p miR-21/590-5p miR-200bc/429/548a

CORO1C	coronin, actin binding protein, 1C	0.183	migration (1)	(1) Luan et al. 2010	n.a.	n.a.	miR-206 (1), miR-1/133a (2)	(1) Wang et al. 2014, (2) Mataki et al. 2015	miR-1ab/206/613 miR-34ac/34bc-5p/449abc/449c-5p miR-182 miR-204/204b/211 miR-383 miR-133abc miR-135ab/135a-5p miR-128/128ab miR-183 miR-96/507/1271 miR-217 miR-210 miR-214/761/3619-5p miR-150/5127 miR-26ab/1297/4465 miR-29abcd miR-141/200a miR-218/218a miR-31 miR-200bc/429/548a miR-203 miR-181abcd/4262 miR-219-5p/508/508-3p/4782-3p miR-19ab miR-125a-5p/125b-5p/351/670/4319 miR-17/17-5p/20ab/20b-5p/93/106ab/427/518a-3p/519d miR-194 miR-21/590-5p miR-129-5p/129ab-5p
TP53INP1	tumor protein p53 inducible nuclear protein 1	0.214	tumor suppressor (1,2)	(1) Saadi et al. 2015, (2) Shahbazi et al. 2013	proliferation (1), cell survival (1)	(1) Giusiano et al. 2012	miR-155 (1), miR-125b (2), miR-17-5p (3), miR-182 (4), miR-96 (5)	(1) Gironella et al, 2007, (2) Jiang et al. 2011, (3) Wie et al. 2012, (4) Qin et al. 2014, (5) Gao et al. 2015	miR-22/22-3p miR-130ac/301ab/301b/301b-3p/454/721/4295/3666 miR-190/190ab miR-182 miR-217 miR-125a-5p/125b-5p/351/670/4319 miR-34ac/34bc-5p/449abc/449c-5p miR-155 miR-26ab/1297/4465 miR-124/124ab/506 miR-19ab miR-153 miR-141/200a miR-196abc miR-93/93a/105/106a/291a-3p/294/295/302abcde/372/373/428/519a/520be/520acd-3p/1378/1420ac miR-204/204b/211 miR-17/17-5p/20ab/20b-5p/93/106ab/427/518a-3p/519d miR-221/222/222ab/1928 miR-24/24ab/24-3p miR-96/507/1271 miR-193/193b/193a-3p miR-199ab-5p miR-216a miR-216b/216b-5p miR-129-5p/129ab-5p miR-455-5p miR-29abcd miR-200bc/429/548a miR-144 miR-31 miR-150/5127 miR-101/101ab miR-30abcdef/30abe-5p/384-5p miR-148ab-3p/152 miR-145 miR-205/205ab miR-128/128ab miR-33ab/33-5p miR-140/140-5p/876-3p/1244 miR-23abc/23b-3p miR-191 miR-27abc/27a-3p let-7/98/4458/4500 miR-203 miR-223 miR-132/212/212-3p
TFRC	transferrin receptor (p90, CD71)	0.279	carcinogenesis (1), proliferation (1)	(1) O'Donnell et al. 2006	n.a.	n.a.	n.a.	n.a.	miR-10abc/10a-5p miR-140/140-5p/876-3p/1244 miR-31 miR-490-3p miR-210 miR-181abcd/4262 miR-338/338-3p miR-103a/107/107ab miR-193/193b/193a-3p miR-182 miR-7/7ab miR-218/218a miR-221/222/222ab/1928 miR-425/425-5p/489 miR-148ab-3p/152 miR-223 miR-124/124ab/506 miR-217 miR-96/507/1271 miR-214/761/3619-5p miR-23abc/23b-3p miR-141/200a miR-145 miR-9/9ab miR-26ab/1297/4465 miR-144 miR-216a miR-194

Supplementary Table S1

Probe Set ID	Sig log ratio_Ago2_IP_PNF8 vs ctrl._PNF8	Fold Change_Ago2_IP_PNF8 vs ctrl._PNF8	p-Value_Ago2_IP_PNF8 vs ctrl._PNF8	Sig log ratio_Ago2_IP_DU145 vs ctrl._DU145	Fold Change_Ago2_IP_DU145 vs ctrl._DU145	p-Value_Ago2_IP_DU145 vs ctrl._DU145	Gene Accession Number	Gene Symbol
8055476	8,29	313,26	0,00000	4,87	29,26	0,02603	NM_006761	YWHAE
7965403	7,58	191,79	0,00001	-1,45	-2,73	0,00042	NM_002345	LUM
8146863	7,46	175,83	0,00000	-0,83	-1,78	0,05130	NM_001128205	SULF1
8050548	7,43	172,44	0,00008	5,05	33,21	0,03101	NM_014713	LAPTM4A
8112139	7,38	166,85	0,00000	3,96	15,61	0,02853	NM_002184	IL6ST
8115327	7,26	153,36	0,00002	-1,44	-2,71	0,02283	NM_003118	SPARC
8146216	7,23	150,04	0,00003	5,67	51,06	0,00052	NM_001135694	VDAC3
8154305	7,16	143,50	0,00028	4,61	24,40	0,08064	NM_016275	SELT
7971077	7,14	141,37	0,00000	-0,68	-1,61	0,04170	NM_006475	POSTN
7981978	6,99	127,02	0,00057	-1,24	-2,37	0,58671	NR_003330	SNORD116-15
7965410	6,98	126,18	0,00001	-0,90	-1,87	0,00737	NM_001920	DCN
8130499	6,90	119,32	0,00000	4,06	16,72	0,00135	NM_006519	DYNLT1
7961514	6,89	118,98	0,00008	-2,00	-4,01	0,00926	NM_000900	MGP
8046333	6,74	107,14	0,00002	4,25	19,06	0,00006	NM_024843	CYBRD1
8067007	6,68	102,82	0,00014	2,67	6,36	0,01082	NM_021109	TMSB4X
8167185	6,68	102,78	0,00028	3,90	14,96	0,00256	NM_003254	TIMP1
8042788	6,62	98,55	0,00029	-1,70	-3,26	0,00688	NM_001615	ACTG2
7977775	6,62	98,21	0,00011	6,03	65,40	0,00041	NM_001344	DAD1
8106986	6,61	97,69	0,00002	3,86	14,49	0,00077	NM_014899	RHOBTB3
7953603	6,59	96,57	0,00005	0,67	1,59	0,09890	NM_201442	C1S
8152512	6,56	94,46	0,00022	-0,22	-1,17	0,36992	NM_002546	TNFRSF11B
7939137	6,54	93,33	0,00001	5,32	39,91	0,00179	NM_006360	EIF3M
7982597	6,54	93,16	0,00001	2,84	7,17	0,00008	NM_003246	THBS1
7958019	6,52	92,06	0,00003	4,42	21,38	0,00062	NM_018370	DRAM1
8008870	6,45	87,27	0,00000	4,02	16,18	0,03023	NM_030938	TMEM49
8139087	6,43	86,14	0,00002	-0,85	-1,81	0,01160	NM_003014	SFRP4
8113073	6,38	83,01	0,00000	5,42	42,87	0,00001	NM_020801	ARRDC3
7938291	6,34	81,24	0,00064	1,89	3,71	0,11699	NR_002580	SNORA3
8093053	6,34	81,15	0,00000	4,50	22,60	0,00008	NM_003234	TFRC
8056201	6,32	79,62	0,00012	5,42	42,68	0,00006	NM_016836	RBMS1
8098758	6,27	77,16	0,00007	3,23	9,37	0,03083	NM_133474	ZNF721
7966135	6,25	76,07	0,00018	3,83	14,24	0,01548	NM_014325	CORO1C
8052680	6,25	75,92	0,00000	5,34	40,61	0,00000	NM_004161	RAB1A
7959023	6,19	72,79	0,00011	4,29	19,62	0,00005	NM_001085481	MAP1LC3B2
8154295	6,19	72,78	0,00005	-1,21	-2,31	0,00226	NM_033439	IL33
7933084	6,18	72,71	0,00035	4,97	31,41	0,00515	NM_005746	NAMPT
8178050	6,17	72,18	0,00146	4,90	29,83	0,00126	NM_001320	CSNK2B
8072407	6,16	71,39	0,00000	4,24	18,96	0,00364	NR_002323	TUG1
7962537	6,12	69,43	0,00003	3,84	14,36	0,07746	NM_018976	SLC38A2
7919815	6,11	69,19	0,00000	2,04	4,10	0,00201	NM_000396	CTSK
7979864	6,11	69,17	0,00311	5,29	39,06	0,01907	NM_004450	ERH
7964757	6,11	68,91	0,00024	4,60	24,29	0,00067	NM_016836	RBMS1
7989224	6,11	68,90	0,00047	4,03	16,30	0,02242	NM_001110	ADAM10
7917472	6,11	68,90	0,00001	4,82	28,34	0,00005	NM_001514	GTF2B
8073015	6,07	67,19	0,00013	4,26	19,22	0,00559	NM_006855	KDELR3
8091715	6,07	67,13	0,00001	-0,59	-1,51	0,01144	NM_020169	LXN
8155849	6,06	66,90	0,00327	3,67	12,76	0,01822	NM_000700	ANXA1
7902771	6,04	65,77	0,00000	1,84	3,59	0,01468	NM_016009	SH3GLB1

8140828	6,03	65,39	0,00008	4,72	26,27	0,00435	NM_003130	SRI
8102532	6,02	65,10	0,00001	0,02	1,02	0,93876	NM_001083	PDE5A
8065325	6,02	64,97	0,00066	5,62	49,33	0,00127	---	TMEM30A
8127637	6,02	64,86	0,00006	5,16	35,79	0,00346	NM_018247	TXNIP
7904726	6,01	64,37	0,00051	0,73	1,65	0,15404	NM_006472	DYNLL1
7980535	6,00	64,16	0,00005	5,10	34,40	0,00509	NM_001037494	ACTA2
7934906	5,99	63,62	0,00002	0,15	1,11	0,59950	NM_001141945	DAB2
8111772	5,98	63,16	0,00000	1,57	2,97	0,00176	NM_001343	NPC2
7980146	5,98	63,05	0,00031	4,21	18,54	0,00185	NM_006432	RND3
8055688	5,97	62,78	0,00002	3,07	8,37	0,01985	NM_005168	MYL6
7956211	5,97	62,65	0,00004	5,06	33,45	0,00018	NM_021019	ITGB1
7932966	5,96	62,29	0,00088	2,00	4,01	0,33091	NM_033666	CCL2
8006433	5,95	61,81	0,00009	-2,04	-4,12	0,00011	NM_002982	MED21
7954503	5,95	61,74	0,00000	4,98	31,60	0,00235	NM_004264	STRADB
7901336	5,94	61,56	0,00007	4,31	19,86	0,00145	NM_018571	DPP4
8056222	5,94	61,18	0,00001	1,65	3,14	0,00733	NM_001935	SEC23A
7978718	5,93	60,93	0,00001	3,93	15,21	0,03383	NM_006364	COX16
7979906	5,92	60,62	0,00240	5,89	59,19	0,00002	NM_016468	ZFAND5
8161747	5,90	59,87	0,00021	5,18	36,32	0,00005	NM_001102420	RPS25
7952129	5,90	59,84	0,00140	1,93	3,82	0,14723	NM_001028	RARRES1
8091723	5,88	58,77	0,00000	0,72	1,65	0,01830	NM_206963	CCDC80
8089544	5,85	57,84	0,00002	0,80	1,74	0,02695	NM_199511	KIF5B
7932911	5,85	57,65	0,00036	4,10	17,19	0,02376	NM_004521	RPN2
8062349	5,85	57,58	0,00003	4,89	29,68	0,00020	NM_002951	TMEM50A
7899005	5,84	57,47	0,00029	5,19	36,59	0,00417	NM_014313	STT3B
8078360	5,84	57,18	0,00026	3,83	14,26	0,01878	NM_178862	MINPP1
7928937	5,83	56,99	0,00002	3,90	14,89	0,03496	NM_004897	ASB5
8103812	5,83	56,96	0,00009	-0,49	-1,41	0,17975	NM_080874	HGF
8140556	5,83	56,76	0,00012	-0,61	-1,53	0,01599	NM_000601	IL6
8131803	5,82	56,60	0,00014	3,48	11,17	0,00229	NM_000600	FN1
8058765	5,82	56,43	0,00014	1,25	2,38	0,00266	NM_212482	SMPDL3A
8121794	5,82	56,38	0,00013	1,76	3,38	0,01420	NM_006714	PRDX3
7936661	5,82	56,31	0,00016	3,16	8,95	0,04830	NM_006793	PICALM
7950838	5,79	55,36	0,00001	4,50	22,63	0,00184	NM_007166	SERINC1
8129317	5,78	55,04	0,00006	4,67	25,48	0,00196	NM_020755	FSTL1
8089835	5,78	55,04	0,00001	1,82	3,53	0,01161	NM_007085	ANXA5
8102619	5,78	54,92	0,00002	4,68	25,56	0,00000	NM_001154	KDELR2
8138108	5,76	54,34	0,00005	4,64	24,94	0,00026	ENST00258739	NOP10
7987225	5,75	53,96	0,00001	3,78	13,73	0,00218	NM_018648	UBE2L3
7968331	5,75	53,84	0,00002	1,33	2,51	0,33839	NM_003347	GDI2
7931873	5,75	53,63	0,00133	4,63	24,73	0,00040	NM_001494	CST1
8065412	5,74	53,61	0,00010	-1,47	-2,76	0,05031	NM_001898	B2M
7983360	5,74	53,30	0,00000	3,76	13,54	0,00009	NM_004048	SMURF2
8017651	5,73	53,24	0,00004	3,35	10,20	0,01596	NM_022739	CDH11
8001800	5,73	52,99	0,00003	-1,04	-2,05	0,00150	NM_001797	SLC35F5
8054771	5,72	52,85	0,00002	5,24	37,87	0,00023	NM_025181	TRAPPC1
8012304	5,72	52,71	0,00139	4,85	28,86	0,00025	NM_021210	MOXD1
8129573	5,72	52,70	0,00007	2,04	4,12	0,00352	NM_015529	SERPINE2
8059376	5,72	52,53	0,00150	3,07	8,42	0,00156	NM_001136529	PLIN2
8160297	5,71	52,44	0,00001	4,58	23,94	0,00117	NM_001122	VPS35
8001149	5,71	52,39	0,00000	4,20	18,35	0,00031	NM_018206	SAR1A
7934122	5,68	51,35	0,00005	4,45	21,91	0,00001	NM_001142648	TFDP1
7970317	5,68	51,12	0,00020	5,35	40,88	0,00229	NM_007111	MYL12B
8019930	5,67	51,00	0,00283	5,12	34,78	0,02058	NM_033546	RPLP0
8109750	5,66	50,48	0,04419	4,97	31,31	0,02327	NM_001002	NAMPT
8142120	5,66	50,39	0,00013	4,54	23,21	0,01081	NM_005746	C6orf72
8122689	5,65	50,07	0,00001	3,48	11,12	0,00648	AY358952 CSDE1	
7918825	5,63	49,38	0,00000	2,67	6,38	0,09014	NM_001007553	DSTN
8061114	5,63	49,36	0,00014	3,94	15,33	0,00049	NM_001011546	TGFBI
8108217	5,61	48,79	0,00007	2,91	7,51	0,00011	NM_000358	CHP
7982878	5,61	48,73	0,00044	5,19	36,39	0,00190	NM_007236	WTAP
8126952	5,60	48,67	0,00086	3,91	15,00	0,00847	NM_004906	CALU
8135955	5,60	48,39	0,00003	4,31	19,89	0,00082	NM_001219	SH3BGRL
8168557	5,59	48,23	0,00011	4,15	17,79	0,00205	NM_003022	SERPINE1
8135069	5,59	48,18	0,00035	-0,91	-1,88	0,02458	NM_000602	ARPC2
8048234	5,59	48,14	0,00279	4,47	22,20	0,02089	NM_152862	PBX3
8157890	5,59	48,07	0,00000	3,10	8,56	0,01906	NM_006195	TMEM47
8172022	5,58	47,81	0,00004	-2,06	-4,17	0,00002	NM_031442	ARPC3
7966315	5,58	47,80	0,00020	4,51	22,76	0,00037	NM_005719	MMD
8016832	5,57	47,59	0,00052	5,57	47,51	0,00020	NM_012329	TMBIM6
7955277	5,57	47,44	0,00224	5,48	44,49	0,00268	NM_003217	THY1
7952268	5,57	47,39	0,00002	-1,91	-3,77	0,00063	NM_006288	CLTC
8008834	5,56	47,21	0,00075	2,73	6,64	0,06836	NM_004859	CD59

7947425	5,56	47,18	0,00049	3,98	15,83	0,00007	NM_203330	IGFBP5
8058857	5,56	47,17	0,00007	-0,38	-1,31	0,07184	NM_000599	SDCBP
8146550	5,56	47,02	0,00002	4,41	21,33	0,00036	NM_005625	HSP90B1
7958130	5,55	46,86	0,00019	3,57	11,86	0,05324	NM_003299	TMBIM1
8058927	5,55	46,82	0,00014	4,78	27,39	0,00116	NM_022152	RLIM
8173613	5,55	46,77	0,00105	3,25	9,49	0,00628	NM_016120	TMSB4X
8166072	5,55	46,70	0,00029	1,80	3,49	0,02279	NM_021109	LPHN2
7902565	5,54	46,59	0,00011	-0,50	-1,42	0,11679	NM_012302	MRPL45
8006762	5,53	46,25	0,00448	6,97	125,16	0,00016	NM_032351	PCMTD1
8150714	5,52	45,90	0,00041	4,78	27,47	0,00314	NM_052937	TAF9B
8173732	5,52	45,87	0,00036	3,38	10,43	0,00700	NM_015975	TAF9B
8176263	5,52	45,87	0,00036	3,38	10,43	0,00700	NM_015975	CLDN11
8083887	5,50	45,27	0,00053	1,28	2,42	0,05238	NM_005602	WARS
7981290	5,49	44,95	0,00046	4,67	25,50	0,00055	NM_004184	SUZ12
8006325	5,48	44,66	0,00015	3,73	13,25	0,00231	NM_015355	LOX
8113709	5,48	44,66	0,00002	-1,12	-2,17	0,05231	NM_002317	NT5E
8120967	5,48	44,65	0,00033	4,15	17,77	0,00028	NM_002526	HSPA8
7952325	5,48	44,64	0,01023	3,34	10,10	0,05482	NM_006597	EDIL3
8112980	5,47	44,37	0,00023	5,15	35,46	0,00541	NM_005711	SEMA3A
8140668	5,46	44,12	0,00005	0,58	1,50	0,15589	NM_006080	SEC63
8128650	5,46	44,12	0,00010	4,84	28,58	0,00007	NM_007214	SC5DL
7944656	5,45	43,73	0,00188	4,82	28,32	0,00118	NM_006918	TIMP3
8075635	5,44	43,54	0,00021	-0,29	-1,23	0,22797	NM_000362	LDHA
7938777	5,44	43,52	0,00107	5,21	36,91	0,00000	NM_005566	ELOVL5
8127145	5,44	43,34	0,00085	4,93	30,46	0,00030	NM_021814	SOD2
8130556	5,44	43,33	0,00025	2,34	5,06	0,01029	NM_001024465	---
7974471	5,43	43,07	0,00020	1,75	3,36	0,17921	---	COPA
7921583	5,43	43,06	0,00026	4,06	16,65	0,00605	NM_001098398	OSMR
8105040	5,43	43,01	0,00041	3,77	13,62	0,03171	NM_003999	ZNF737
8035793	5,43	43,01	0,00674	2,68	6,40	0,01509	NM_001159293	STAM2
8055890	5,42	42,91	0,00003	4,44	21,72	0,00025	NM_005843	TMSB4X
8158240	5,42	42,91	0,00006	1,56	2,95	0,00328	NM_021109	RAB7A
8082431	5,42	42,75	0,00269	5,19	36,46	0,00047	NM_004637	MTCH2
7947934	5,42	42,72	0,00029	5,01	32,24	0,00180	NM_014342	ZNF253
8035779	5,41	42,48	0,00499	3,51	11,39	0,00323	NM_021047	PLAT
8150509	5,40	42,34	0,00003	-1,29	-2,44	0,00109	NM_000930	CSNK2B
8118218	5,40	42,25	0,00262	4,26	19,18	0,00028	NM_001320	RSU1
7932311	5,40	42,15	0,00106	3,93	15,27	0,00329	NM_012425	STRADB
8047443	5,40	42,13	0,00001	4,45	21,88	0,00010	NM_018571	RBBP7
8171516	5,40	42,10	0,00072	5,39	42,04	0,00560	NM_002893	CRYZ
7917037	5,39	42,05	0,00005	4,58	23,90	0,00050	NM_001130042	F
7938313	5,39	42,02	0,00242	3,37	10,31	0,07729	NM_006391	TXN
8163185	5,38	41,72	0,00090	4,54	23,22	0,00000	NM_003329	HIF1A
7974851	5,38	41,69	0,00014	3,60	12,13	0,02619	NM_001530	FNDC3B
8083901	5,38	41,62	0,00008	2,85	7,21	0,01342	NM_022763	KIAA1715
8056943	5,37	41,34	0,00019	3,87	14,64	0,00024	NM_030650	GLIPR1
7957260	5,36	41,20	0,00002	-0,22	-1,17	0,43382	NM_006851	PTGIS
8066925	5,36	41,07	0,00022	0,45	1,36	0,31474	NM_000961	ADAM9
8146000	5,36	41,06	0,00008	4,28	19,48	0,00042	NM_003816	SSB
8046201	5,36	40,99	0,00050	2,11	4,32	0,14063	NM_003142	GNG10
7946559	5,36	40,98	0,00099	5,33	40,18	0,00008	NM_001017998	MRPL50
8162880	5,36	40,96	0,00258	3,72	13,14	0,00752	NM_019051	NDUFB4
8081945	5,35	40,74	0,00008	3,40	10,56	0,00398	NM_004547	NDUFB4
8093314	5,35	40,74	0,00008	3,40	10,56	0,00398	NM_004547	PCDH18
8102792	5,34	40,63	0,00069	-0,70	-1,63	0,06795	NM_019035	SERINC3
8066417	5,34	40,59	0,00073	4,81	28,11	0,01519	NM_006811	MMADHC
8055672	5,33	40,18	0,00005	4,99	31,81	0,00010	NM_015702	PPP6C
8164155	5,32	39,85	0,00012	4,85	28,89	0,01318	NM_001123355	IARS
8162313	5,31	39,70	0,00010	3,97	15,72	0,06240	NM_013417	PMP22
8012896	5,31	39,64	0,00131	3,56	11,82	0,00000	NM_000304	PTX3
8083594	5,31	39,53	0,00008	-0,10	-1,07	0,43973	NM_002852	C14orf156
7975989	5,30	39,42	0,00050	5,38	41,74	0,00001	NM_031210	MDM2
7956989	5,30	39,40	0,00033	3,59	12,08	0,01435	NM_002392	GNG12
7916843	5,30	39,37	0,00006	3,72	13,16	0,00339	NM_018841	TFPI
8057599	5,30	39,35	0,00024	3,96	15,54	0,00222	NM_006287	GLRX
8113214	5,30	39,35	0,00042	2,96	7,76	0,00424	NM_002064	NDUFA2
8114618	5,30	39,34	0,00087	4,65	25,13	0,00012	NM_002488	CAV1
8135594	5,30	39,29	0,00003	4,51	22,78	0,00032	NM_001753	ZNF91
8035842	5,29	39,16	0,00020	2,43	5,38	0,01102	NM_003430	A2M
7960947	5,29	39,08	0,00013	-1,30	-2,47	0,03658	NM_000014	SNORD116-24
7981996	5,29	39,08	0,00270	-1,58	-2,99	0,53280	NR_003338	CLDN12
8134091	5,28	38,88	0,00033	4,87	29,17	0,00055	NM_012129	VCAN
8106743	5,28	38,82	0,00092	-1,16	-2,23	0,00734	NM_004385	NDUFB5

8084092	5,28	38,74	0,00059	4,52	22,97	0,00029	NM_002492	FAM48A
7971039	5,27	38,50	0,00004	3,99	15,84	0,00596	NM_001014286	TES
8135576	5,26	38,39	0,00030	5,18	36,32	0,00005	NM_015641	VAMP7
8171041	5,25	38,18	0,00016	4,69	25,73	0,00223	NM_005638	VAMP7
8176962	5,25	38,18	0,00016	4,69	25,73	0,00223	NM_005638	LPAR1
8163257	5,25	38,18	0,00001	3,19	9,10	0,00033	NM_057159	TMTC3
7957478	5,25	38,09	0,00001	3,60	12,09	0,00486	NM_181783	SLAIN2
8094948	5,25	37,98	0,00034	3,20	9,16	0,03267	NM_020846	CFH
7908459	5,25	37,97	0,00000	-0,80	-1,74	0,01860	NM_000186	---
8100125	5,25	37,97	0,00050	4,46	22,06	0,00004	---	MRPL20
7911559	5,23	37,49	0,00086	5,32	39,87	0,00002	NM_017971	MRPL20
8039939	5,23	37,49	0,00086	5,32	39,87	0,00002	NM_017971	GNS
7964701	5,23	37,48	0,00008	5,30	39,28	0,00015	NM_002076	BZW1L1
8047339	5,22	37,26	0,00003	4,51	22,78	0,00490	NR_026584	MID1
8171297	5,21	37,13	0,00010	4,14	17,58	0,00082	NM_000381	SNORD49B
8005200	5,21	37,06	0,00311	2,38	5,19	0,14211	NR_003043	CD164
8128716	5,21	37,01	0,00043	4,84	28,58	0,00073	NM_006016	PAPOLA
7976598	5,21	36,95	0,00102	4,91	30,00	0,01483	NM_032632	EIF3H
8152465	5,20	36,83	0,00002	2,74	6,69	0,01123	NM_003756	RPF2
8121510	5,20	36,79	0,00171	3,44	10,84	0,04782	NM_032194	PLS3
8169473	5,20	36,66	0,00002	4,13	17,57	0,00207	NM_005032	ARCN1
7944285	5,20	36,64	0,00113	3,72	13,18	0,00236	NM_001655	MMP2
7995681	5,19	36,62	0,00002	-1,47	-2,77	0,02427	NM_004530	WBP11
7961489	5,19	36,42	0,00037	4,15	17,72	0,00018	NM_016312	RAB23
8127346	5,18	36,37	0,00003	3,40	10,53	0,00080	NM_016277	ATP6V0E1
8110022	5,18	36,36	0,00020	5,29	39,22	0,00017	NM_003945	SEC24B
8096781	5,18	36,29	0,00011	2,91	7,51	0,08840	NM_006323	HIST1H2BK
8124492	5,18	36,25	0,00134	3,26	9,57	0,06596	NM_080593	NCRNA00152
8054611	5,18	36,15	0,00291	4,59	24,17	0,00144	NR_024204	LPCAT3
7960730	5,17	36,03	0,00060	5,72	52,77	0,00002	NM_005768	MAGED1
8167656	5,17	35,93	0,00002	3,95	15,46	0,00008	NM_001005333	CD302
8056102	5,16	35,86	0,00020	2,53	5,76	0,00982	NM_014880	NDUFA12
7965515	5,16	35,73	0,00729	2,40	5,26	0,11301	NM_018838	TMED10
7980296	5,16	35,71	0,00000	4,95	30,98	0,00323	NM_006827	GBP1
7917516	5,16	35,70	0,00009	-0,23	-1,17	0,62753	NM_002053	GABARAP
8012110	5,15	35,63	0,00758	5,96	62,06	0,00076	NM_007278	SPPL2A
7988753	5,15	35,61	0,00025	5,34	40,40	0,00072	NM_032802	TMSL3
8101774	5,15	35,55	0,00034	1,61	3,06	0,04978	NM_183049	SPCS2
7942553	5,15	35,52	0,00016	3,64	12,43	0,00107	NM_014752	DDR2
7906878	5,15	35,50	0,00041	0,28	1,21	0,33561	NM_001014796	GPR137B
7910680	5,15	35,46	0,00005	4,22	18,60	0,00002	NM_003272	SEC31A
8101376	5,14	35,29	0,00011	3,43	10,75	0,03804	NM_014933	ERLIN1
7935692	5,14	35,27	0,00033	3,93	15,22	0,01877	NM_001100626	WIPI1
8017850	5,14	35,20	0,00014	3,63	12,40	0,00008	NM_017983	RGS2
7908409	5,13	35,09	0,00123	3,90	14,95	0,00003	NM_002923	USO1
8095773	5,13	35,07	0,00005	4,05	16,55	0,00002	NM_003715	---
8163714	5,13	35,02	0,00024	2,15	4,42	0,01467	--- VDAC2	
8042335	5,12	34,86	0,00008	5,27	38,58	0,00034	NM_003375	
8045919	5,12	34,79	0,00042	3,88	14,73	0,00094	NM_022826	CNOT7
8149475	5,12	34,77	0,00138	4,88	29,37	0,00393	NM_013354	AP3M1
7934513	5,12	34,75	0,00016	4,16	17,86	0,00536	NM_207012	EIF3D
8075798	5,12	34,73	0,00029	3,68	12,79	0,02495	NM_003753	PAFAH1B2
7944042	5,12	34,72	0,00068	2,65	6,28	0,00091	NM_002572	CAP1
7900382	5,10	34,31	0,00020	3,71	13,13	0,00227	NM_006367	NME2P1
7959146	5,10	34,27	0,00003	4,89	29,73	0,00189	NR_001577	SDHB
7912928	5,10	34,20	0,00005	3,31	9,91	0,00052	NM_003000	STOM
8157605	5,09	34,17	0,00011	-0,28	-1,22	0,61064	NM_004099	RAB14
8163882	5,09	34,07	0,00029	3,79	13,86	0,02367	NM_016322	CCNG1
8109697	5,09	34,01	0,00004	3,73	13,29	0,00012	NM_004060	RAP1B
8112728	5,09	33,97	0,00002	3,84	14,34	0,00182	NM_015646	CDC42EP3
8051573	5,08	33,86	0,00033	2,77	6,84	0,00277	NM_006449	REEP5
8113542	5,07	33,70	0,00003	4,54	23,21	0,00897	NM_005669	RPL24
8126450	5,07	33,68	0,00297	4,28	19,49	0,00193	NM_000986	C7orf23
8140730	5,07	33,52	0,00100	3,65	12,55	0,00027	NM_024315	SUMO2
8174636	5,06	33,41	0,00075	4,56	23,58	0,00033	NM_006937	UXT
8172358	5,06	33,36	0,00005	4,42	21,36	0,00190	NM_153477	PIAS1
7984453	5,06	33,26	0,00004	4,00	16,03	0,03053	NM_016166	TUBA1A
7963054	5,06	33,25	0,00094	2,78	6,86	0,00872	NM_006009	SOS2
7978932	5,05	33,24	0,00037	3,71	13,07	0,01947	NM_006939	ACO1
8154733	5,05	33,15	0,00006	3,96	15,53	0,00153	NM_002197	CD46
7909400	5,04	32,91	0,00048	3,58	11,94	0,03890	NM_002389	SNORD116-14
7981976	5,04	32,90	0,00185	-1,56	-2,94	0,49142	NR_003329	SHOC2
7930470	5,04	32,87	0,00053	4,67	25,41	0,00350	NM_007373	GTF2A2

7989315	5,04	32,85	0,00025	4,06	16,64	0,00064	NM_004492	C11orf10
7948606	5,03	32,64	0,00126	3,95	15,42	0,01054	NM_014206	FKBP1A
8120428	5,03	32,63	0,00302	3,20	9,21	0,01587	NM_000801	NPEPPS
8007976	5,02	32,47	0,00002	3,83	14,25	0,00299	NM_006310	OSBPL8
7965064	5,02	32,43	0,00003	3,45	10,95	0,00342	NM_020841	ATP6V0D1
8002041	5,01	32,31	0,00278	4,57	23,69	0,00001	NM_004691	FERMT2
7979204	5,01	32,29	0,00002	3,76	13,51	0,00007	NM_006832	TMEM181
8123062	5,01	32,17	0,00014	3,85	14,38	0,00004	NM_020823	PPP1CC
7966368	5,00	32,10	0,00015	5,75	53,98	0,00049	NM_002710	LMAN1
8023561	5,00	31,92	0,00030	4,47	22,18	0,00015	NM_005570	PDIA6
8050278	4,99	31,81	0,00152	4,09	17,04	0,00424	NM_005742	PSMB2
7914834	4,99	31,77	0,00169	4,02	16,20	0,00071	NM_002794	GNB2L1
8116520	4,99	31,74	0,02416	3,82	14,14	0,06583	NM_006098	CAST
8107005	4,99	31,68	0,00010	2,38	5,22	0,05201	NM_001750	SLC39A9
7975344	4,98	31,66	0,00054	5,12	34,68	0,00000	NM_018375	PXDN
8050007	4,98	31,49	0,00020	-0,08	-1,06	0,36729	NM_012293	GTF2H1
7938762	4,97	31,43	0,00007	3,44	10,84	0,00037	NM_005316	PCYOX1
8042519	4,97	31,35	0,00013	3,65	12,57	0,00001	NM_016297	CRYAB
7951662	4,97	31,25	0,00006	-1,32	-2,50	0,10600	NM_001885	TNFAIP1
8005847	4,96	31,20	0,00002	5,31	39,63	0,00001	NM_021137	UTP11L
7900201	4,96	31,07	0,00005	3,67	12,69	0,00050	NM_016037	SUMO2
8018315	4,96	31,07	0,00100	4,02	16,22	0,00140	NM_006937	TRAM1
8151281	4,96	31,04	0,00012	3,82	14,09	0,00004	NM_014294	CANX
8110522	4,96	31,04	0,00114	3,14	8,83	0,02348	NM_001746	YWHAE
8011011	4,95	30,93	0,00520	4,36	20,47	0,03386	NR_024058	USP53
8097098	4,95	30,92	0,00002	3,05	8,27	0,00035	NM_019050	CSE1L
8063283	4,95	30,91	0,00004	3,77	13,62	0,00252	NM_001316	PPP1R12A
7965123	4,95	30,90	0,00001	3,36	10,30	0,03800	NM_001143885	MRPL47
8092265	4,94	30,79	0,00000	2,73	6,64	0,01365	NM_020409	XPOT
7956785	4,94	30,78	0,00019	3,88	14,72	0,00340	NM_007235	PSMD7
7997230	4,94	30,74	0,00031	4,73	26,45	0,00006	NM_002811	PIGY
8101718	4,94	30,72	0,00005	3,62	12,32	0,00091	NM_032906	SLIT2
8094301	4,94	30,69	0,00005	0,28	1,21	0,16770	NM_004787	GPX8
8105348	4,93	30,59	0,00051	2,80	6,97	0,00063	NM_001008397	GARS
8132070	4,93	30,51	0,00026	5,05	33,15	0,00632	NM_002047	CYP1B1
8051583	4,93	30,45	0,00005	3,70	12,96	0,00019	NM_000104	ITM2B
7969003	4,93	30,45	0,00030	3,06	8,35	0,00882	NM_021999	C1orf152
7904974	4,93	30,41	0,00654	1,08	2,11	0,04470	NR_003242	RB1
7969017	4,93	30,41	0,00002	4,15	17,71	0,00225	NM_000321	NARS
8023481	4,93	30,38	0,00052	4,48	22,29	0,00137	NM_004539	ITGA2
8105267	4,92	30,33	0,00000	3,18	9,05	0,05443	NM_002203	NCOA4
7927536	4,92	30,29	0,00019	4,43	21,51	0,00035	NM_005437	COPS4
8096098	4,90	29,88	0,00149	5,02	32,39	0,00011	NM_016129	DNAJC15
7968872	4,90	29,83	0,01580	-0,90	-1,86	0,12409	NM_013238	SLC38A1
7962516	4,90	29,76	0,00035	3,26	9,59	0,06614	NM_030674	AGFG1
8048847	4,89	29,73	0,00047	4,07	16,79	0,08689	NM_001135187	AKT3
7925531	4,89	29,71	0,00002	2,81	7,00	0,01148	NM_181690	STOM
8163896	4,89	29,71	0,00020	3,26	9,55	0,00019	NM_004099	EIF4A1
8004497	4,89	29,59	0,00052	3,96	15,59	0,00008	NM_001416	HSPA9
8114455	4,88	29,45	0,00075	4,67	25,54	0,00143	NM_004134	SLC30A7
7903369	4,88	29,43	0,00001	4,11	17,30	0,00037	NM_133496	COPS2
7988605	4,88	29,40	0,00083	4,73	26,46	0,00014	NM_004236	C7orf42
8133192	4,88	29,35	0,00002	3,62	12,31	0,01369	NM_017994	C9orf102
8156601	4,87	29,33	0,00055	3,61	12,19	0,04439	AL133663 CDC123	
7926207	4,87	29,31	0,00009	4,82	28,19	0,00028	NM_006023	ATP6V1C1
8147724	4,87	29,22	0,00006	4,46	21,97	0,00008	NM_001695	NEK7
7908543	4,87	29,15	0,00098	4,48	22,32	0,00003	NM_133494	CSRP1
7923378	4,86	29,07	0,00074	4,74	26,72	0,00110	NM_004078	CSNK2B
8179298	4,86	29,00	0,00371	3,68	12,81	0,00069	NM_001320	COQ10B
8047217	4,85	28,94	0,00291	3,55	11,74	0,00094	NM_025147	NUP88
8011832	4,85	28,89	0,00001	4,15	17,75	0,00627	NM_002532	RPS3
7942586	4,85	28,85	0,01141	2,98	7,90	0,07938	NM_001005	FARSB
8059319	4,85	28,82	0,00152	4,29	19,60	0,00484	NM_005687	ARF4
8088339	4,85	28,80	0,00001	3,30	9,82	0,01243	NM_001660	FOXO3B
8013307	4,85	28,74	0,00251	4,44	21,75	0,00494	NR_026718	ALG8
7950654	4,84	28,70	0,00055	4,94	30,63	0,00109	NM_024079	FBXO32
8152703	4,84	28,66	0,00036	3,41	10,66	0,00061	NM_058229	---
8139879	4,84	28,62	0,00496	4,04	16,46	0,00050	--- RPS20	
8150872	4,84	28,62	0,00481	2,04	4,10	0,25910	NM_001023	MOSPD1
8175288	4,84	28,59	0,00019	1,60	3,03	0,09790	NM_019556	WASF1
8128818	4,84	28,58	0,00009	3,66	12,61	0,01497	NM_003931	ABI3BP
8089145	4,83	28,51	0,00005	-0,11	-1,08	0,71291	NM_015429	MGST1
7954196	4,83	28,45	0,00285	2,26	4,77	0,16060	NM_145792	ATP2B1

7965359	4,83	28,45	0,00030	3,39	10,48	0,02758	NM_001001323	EFEMP1
8052355	4,83	28,44	0,00021	3,05	8,31	0,01018	NM_004105	SEC24A
8108099	4,83	28,41	0,00051	3,90	14,91	0,00082	NM_021982	RARB
8078286	4,83	28,39	0,00051	-0,96	-1,94	0,00884	NM_000965	DKK1
7927631	4,83	28,38	0,00157	4,30	19,73	0,00035	NM_012242	HLA-B
8179731	4,83	28,37	0,00083	5,63	49,54	0,00000	BC013187 BAT1	
8178476	4,82	28,18	0,00002	5,26	38,33	0,00662	NM_004640	GREM1
7982377	4,82	28,17	0,00027	-1,48	-2,78	0,00024	NM_013372	OSTC
8096771	4,81	28,15	0,00042	4,75	26,95	0,00005	NM_021227	PDIA3P
7904881	4,81	28,10	0,00014	4,31	19,90	0,00012	NR_002305	NMD3
8083757	4,81	28,05	0,00008	4,07	16,77	0,00021	NM_015938	YME1L1
7932678	4,81	27,99	0,00081	3,86	14,54	0,00055	NM_139312	ERP44
8162833	4,80	27,91	0,00015	3,51	11,41	0,00082	NM_015051	NUCB2
7938687	4,80	27,89	0,00034	2,27	4,83	0,00022	NM_005013	TMSB10
8043100	4,80	27,87	0,00008	3,57	11,91	0,00138	NM_021103	RPL30
8151935	4,79	27,75	0,00022	3,86	14,47	0,01725	NM_000989	DDX5
8017634	4,78	27,48	0,00001	4,87	29,32	0,00028	NM_004396	CAPZA2
8135625	4,78	27,48	0,00210	2,64	6,25	0,00455	NM_006136	ITFG1
8001211	4,78	27,42	0,00004	3,76	13,52	0,00525	NM_030790	ATL3
7948995	4,77	27,33	0,00060	3,55	11,71	0,01506	ENST00000398868	PSMD1
8049016	4,77	27,28	0,00312	3,75	13,44	0,00083	NM_002807	C3orf64
8088680	4,77	27,27	0,00090	3,38	10,40	0,00000	NM_173654	RDH10
8146921	4,77	27,24	0,00134	3,79	13,79	0,00307	NM_172037	RIPK2
8147206	4,77	27,20	0,00009	4,62	24,57	0,00166	NM_003821	EFR3A
8148333	4,76	27,14	0,00022	3,65	12,59	0,01017	NM_015137	SLFN5
8006531	4,76	27,13	0,00054	2,50	5,68	0,00112	NM_144975	IREB2
7985166	4,76	27,10	0,00038	4,09	17,09	0,00118	NM_004136	CETN2
8175755	4,76	27,09	0,00008	2,66	6,33	0,00658	NM_004344	RPL26
8012469	4,76	27,07	0,00046	1,77	3,42	0,10709	NM_000987	EIF4EBP2
7928119	4,76	27,00	0,00037	4,41	21,29	0,00141	NM_004096	---
8162531	4,75	26,91	0,04223	3,00	7,98	0,03500	--- PIK3R1	
8105778	4,75	26,83	0,00006	2,47	5,55	0,00057	NM_181523	CKS2
8156290	4,74	26,81	0,00180	4,74	26,64	0,01831	NM_001827	PGRMC1
8169617	4,74	26,77	0,00028	4,42	21,39	0,00041	NM_006667	UBA2
8027650	4,74	26,75	0,00062	2,49	5,63	0,12454	NM_005499	TMEM59
7916372	4,74	26,74	0,00071	4,47	22,23	0,00146	NM_004872	ERGIC2
7962013	4,74	26,72	0,00083	3,70	13,03	0,00249	NM_016570	GNG11
8134257	4,74	26,65	0,00044	1,39	2,63	0,03572	NM_004126	BAT1
8179750	4,74	26,65	0,00010	5,43	43,08	0,00659	NM_004640	RARS
8109802	4,73	26,60	0,00011	3,79	13,80	0,00051	NM_002887	SDHC
8011212	4,73	26,58	0,00122	5,25	38,05	0,00000	NM_003001	CPD
8006123	4,73	26,48	0,00042	2,88	7,38	0,06440	NM_001304	TFG
8081316	4,73	26,48	0,00129	3,71	13,06	0,00316	NM_006070	SCFD1
7973770	4,72	26,40	0,00006	3,95	15,44	0,00081	NM_016106	CAP2
8117054	4,72	26,39	0,00032	3,51	11,37	0,00409	NM_006366	COL3A1
8046922	4,72	26,30	0,00048	-1,29	-2,45	0,00941	NM_000090	EIF3I
7899737	4,71	26,26	0,00547	4,93	30,45	0,00002	NM_003757	PAPSS2
7928944	4,71	26,25	0,00086	2,58	5,97	0,00067	NM_004670	GNB1
7911619	4,71	26,23	0,01273	4,15	17,72	0,00553	NM_002074	FAM114A1
8094609	4,71	26,15	0,00010	2,19	4,56	0,00393	NM_138389	PDLIM1
7935180	4,71	26,11	0,00002	3,84	14,36	0,00019	NM_020992	PSMA1
7946728	4,71	26,10	0,00109	4,70	25,96	0,00223	NM_148976	RPS3A
7988031	4,70	25,99	0,01081	3,64	12,44	0,00639	NM_001006	LYRM2
7999752	4,70	25,93	0,00390	3,92	15,15	0,00203	NR_028493	REEP3
7927786	4,69	25,89	0,00001	3,67	12,77	0,00037	NM_001001330	UBC
7967563	4,69	25,88	0,00897	2,47	5,52	0,09939	NM_021009	RAP1B
7956937	4,69	25,87	0,00006	3,30	9,82	0,00559	NM_015646	MCFD2
8051998	4,69	25,87	0,00004	5,02	32,41	0,00002	NM_139279	---
8084715	4,69	25,82	0,00169	1,79	3,45	0,20044	--- NDUFS5	
7900228	4,68	25,70	0,00006	3,06	8,36	0,00183	NM_004552	OAT
7936871	4,68	25,63	0,00004	3,36	10,25	0,02237	NM_000274	TP53INP1
8151890	4,68	25,56	0,00013	2,45	5,48	0,00371	NM_033285	TUSC3
8144726	4,68	25,56	0,00014	4,36	20,48	0,00235	NM_006765	LRRC58
8089830	4,67	25,53	0,00006	1,99	3,98	0,03722	NM_001099678	COL1A2
8134263	4,67	25,52	0,00019	-1,78	-3,43	0,00002	NM_000089	HSP90AB1
8119993	4,67	25,50	0,00430	3,89	14,78	0,02620	NM_007355	AHR
8131614	4,67	25,48	0,00004	3,31	9,94	0,00558	NM_001621	PPA1
7934133	4,67	25,45	0,00419	1,41	2,65	0,09144	NM_021129	IRF2
8103911	4,67	25,38	0,00030	2,83	7,12	0,00057	NM_002199	MME
8083494	4,67	25,38	0,00132	-0,56	-1,47	0,10085	NM_007288	PLOD2
8091283	4,67	25,37	0,00012	4,59	24,14	0,00007	NM_182943	DCTN4
8115181	4,66	25,26	0,00033	4,02	16,19	0,00130	NM_001135643	UBQLN1
8161988	4,65	25,18	0,00138	3,51	11,40	0,02747	NM_013438	PARM1

8095751	4,65	25,12	0,00026	1,78	3,42	0,00006	NM_015393	PSMA6
7973936	4,65	25,10	0,00006	4,45	21,83	0,00563	NM_002791	METAP1
8096556	4,65	25,09	0,00053	5,44	43,40	0,00010	NM_015143	ANP32B
8156750	4,65	25,07	0,00101	2,70	6,48	0,09418	NM_006401	CHIC2
8100382	4,65	25,07	0,00143	3,42	10,69	0,00706	NM_012110	TBP
8123507	4,65	25,07	0,00042	4,60	24,29	0,00014	NM_003194	CYB5A
8023855	4,65	25,02	0,00105	4,40	21,08	0,00037	NM_148923	PSMA3
7974603	4,64	25,01	0,00337	5,29	38,99	0,00022	NM_002788	APBB2
8099982	4,64	24,94	0,00067	4,19	18,30	0,00926	NM_004307	TBC1D19
8094476	4,64	24,90	0,00089	2,49	5,62	0,00386	NM_018317	ANKIB1
8134180	4,64	24,87	0,00012	4,36	20,59	0,00197	NM_019004	UBE2K
8094704	4,64	24,86	0,00016	4,96	31,09	0,00001	NM_005339	SRP14
7987449	4,63	24,85	0,00346	1,69	3,22	0,10685	NM_003134	NDUFA6
8076417	4,63	24,80	0,00003	3,50	11,33	0,00220	NM_002490	SUMO2
8178322	4,63	24,78	0,00141	3,72	13,17	0,00146	NM_006937	PTAR1
8161632	4,63	24,71	0,00078	4,25	19,00	0,00525	NM_001099666	DCTN2
7964466	4,63	24,69	0,00018	4,10	17,11	0,00014	NM_006400	CMPK1
7901299	4,62	24,61	0,00009	3,82	14,10	0,00075	NM_016308	ZMPSTE24
7900413	4,62	24,60	0,00067	3,94	15,36	0,00148	NM_005857	CDV3
8082788	4,62	24,60	0,00029	3,57	11,91	0,05237	NM_001134422	APP
8069644	4,62	24,56	0,00264	3,51	11,37	0,01835	NM_000484	PSMC2
8135250	4,62	24,51	0,00044	4,59	24,02	0,00034	NM_002803	SSFA2
8046726	4,61	24,39	0,00026	2,37	5,16	0,02767	NM_001130445	FBXO25
8049961	4,61	24,37	0,00758	5,58	47,82	0,00003	NM_183421	PATL1
7948399	4,61	24,37	0,00041	4,43	21,52	0,00596	NM_152716	ANO6
7954997	4,61	24,36	0,00007	3,73	13,25	0,00206	NM_001025356	GIT2
7966268	4,60	24,31	0,00050	4,15	17,71	0,00212	NM_057169	ATXN10
8073799	4,60	24,26	0,00035	4,74	26,75	0,00529	NM_013236	ATF2
8056909	4,60	24,23	0,00022	3,45	10,95	0,02554	NM_001880	CEP170
7925525	4,60	24,22	0,00286	3,95	15,42	0,01536	NM_014812	YTHDF3
8146637	4,59	24,16	0,00001	3,22	9,29	0,00844	NM_152758	EPRS
7924351	4,59	24,15	0,00094	2,63	6,19	0,05338	NM_004446	APOBEC3C
8073068	4,59	24,15	0,00063	4,21	18,54	0,00043	NM_014508	KCNJ2
8009502	4,59	24,14	0,00463	-0,60	-1,51	0,03582	NM_000891	TCP1
8130567	4,59	24,13	0,00002	5,54	46,46	0,00035	NM_030752	DCTN5
7994102	4,59	24,13	0,00285	5,22	37,28	0,00017	NM_032486	SLC14A1
8021081	4,59	24,12	0,00619	-0,99	-1,98	0,00304	NM_001128588	LAMP2
8174779	4,59	24,12	0,00007	3,52	11,44	0,00038	NM_013995	AIDA
7917148	4,59	24,10	0,00043	4,06	16,69	0,00045	NM_022831	PAPD4
8106534	4,59	24,10	0,00025	3,45	10,96	0,00037	NM_001114394	SCARB2
8101158	4,59	24,08	0,00057	2,84	7,16	0,00008	NM_005506	DAZAP2
7955464	4,59	24,06	0,00259	5,64	49,85	0,00006	NM_014764	FBN2
8113800	4,58	24,00	0,00052	-1,12	-2,17	0,00320	NM_001999	FBXO3
7947434	4,58	23,98	0,00035	4,02	16,27	0,00093	NM_033406	SECISBP2L
7988581	4,58	23,96	0,00018	4,10	17,14	0,00006	NM_014701	TMEM131
8054092	4,58	23,93	0,00029	3,36	10,29	0,00259	NM_015348	IDE
7935027	4,58	23,92	0,00032	3,08	8,48	0,01693	NM_004969	RCN1
7939120	4,58	23,86	0,00001	2,99	7,93	0,00309	NM_002901	MAPK8
7927389	4,57	23,82	0,00030	3,64	12,47	0,00326	NM_002750	CREB1
8047839	4,57	23,82	0,00029	3,64	12,44	0,00191	NM_004379	LY96
8146934	4,57	23,79	0,00003	0,27	1,21	0,54193	NM_015364	SSR3
8091648	4,57	23,79	0,00137	4,01	16,12	0,00002	NM_007107	AP1G1
8002592	4,57	23,77	0,00021	5,10	34,41	0,00113	NM_001030007	SLC40A1
8057677	4,57	23,75	0,00018	-0,12	-1,09	0,37140	NM_014585	SPCS2
7914180	4,57	23,72	0,00005	3,22	9,33	0,00062	NM_014752	OGFRL1
8120602	4,57	23,70	0,00023	3,20	9,18	0,02392	NM_024576	F2RL2
8112731	4,56	23,61	0,00393	0,31	1,24	0,36907	NM_004101	S100A10
7920123	4,56	23,59	0,00005	4,02	16,27	0,00042	NM_002966	PITRM1
7931778	4,56	23,59	0,00208	3,95	15,45	0,00941	NM_014889	KLHL5
8094625	4,56	23,58	0,00013	4,74	26,81	0,00005	NM_015990	SMG1
8000217	4,56	23,53	0,00009	2,96	7,80	0,02061	NM_015092	KIF2A
8105523	4,56	23,51	0,00031	3,98	15,78	0,01305	NM_004520	FBLN5
7980908	4,56	23,51	0,00013	2,42	5,34	0,00025	NM_006329	PMS2L1
8141490	4,55	23,50	0,00195	6,00	63,87	0,00009	NR_003613	NFE2L2
8056977	4,55	23,48	0,00127	3,87	14,59	0,00384	NM_006164	STAT3
8015607	4,55	23,46	0,00270	4,08	16,94	0,00172	NM_139276	TMEM167A
8112967	4,55	23,43	0,00041	4,01	16,08	0,00096	NM_174909	COPG
8082478	4,55	23,41	0,00436	4,25	19,04	0,00235	NM_016128	IL13RA2
8174598	4,55	23,35	0,00086	-1,13	-2,19	0,01525	NM_000640	MT1L
7995793	4,55	23,35	0,00107	4,02	16,20	0,00040	NR_001447	FAM35B2
7927288	4,55	23,34	0,00413	2,79	6,90	0,00696	NR_027634	CNOT1
8001693	4,54	23,31	0,00046	3,81	14,06	0,01779	NM_016284	AFF4
8114083	4,54	23,22	0,00075	2,75	6,72	0,00528	NM_014423	TGFBR2

8078350	4,54	23,18	0,00004	4,15	17,73	0,00018	NM_001024847	SCYL2
7957806	4,53	23,18	0,00130	3,28	9,69	0,04808	NM_017988	SAMD9L
8140971	4,53	23,13	0,00006	-1,04	-2,05	0,01678	NM_152703	ARL6IP1
7999834	4,53	23,13	0,01139	5,29	39,02	0,00224	NM_015161	TNFAIP6
8045688	4,53	23,11	0,00069	-0,97	-1,96	0,00771	NM_007115	PLA1A
8081890	4,53	23,07	0,00158	-0,51	-1,43	0,14185	NM_015900	MT1X
7995838	4,53	23,06	0,02089	3,03	8,19	0,01651	NM_005952	PRNP
8060758	4,53	23,04	0,00082	4,46	21,99	0,00226	NM_000311	PGK1
8168500	4,52	23,00	0,00092	3,68	12,79	0,00160	NM_000291	BAT1
8124926	4,52	22,96	0,00007	4,88	29,54	0,00705	NM_004640	CD44
7939341	4,52	22,91	0,00043	3,63	12,41	0,00131	NM_000610	PSD3
8149555	4,52	22,91	0,00004	3,53	11,55	0,00587	NM_015310	LIFR
8111677	4,52	22,90	0,00003	4,14	17,67	0,00146	NM_002310	TM9SF2
7969796	4,52	22,90	0,00058	4,16	17,83	0,00332	NM_004800	GTF3C6
8121502	4,52	22,90	0,00139	4,00	15,97	0,00107	NM_138408	PPAP2A
8112107	4,52	22,87	0,00081	3,89	14,79	0,00011	NM_003711	HAT1
8046346	4,52	22,87	0,00048	4,85	28,89	0,00014	NM_003642	TM7SF3
7961983	4,51	22,85	0,00094	5,40	42,32	0,00008	NM_016551	C3
8033257	4,51	22,82	0,00155	3,15	8,88	0,00039	NM_000064	MORF4L2
8174253	4,51	22,81	0,00002	2,87	7,30	0,00150	NM_001142418	VIM
7926368	4,51	22,80	0,00032	3,04	8,23	0,00056	NM_003380	PTENP1
8160718	4,51	22,76	0,00004	1,75	3,36	0,06676	NR_023917	RAB11A
7984289	4,50	22,70	0,00010	3,12	8,68	0,00463	NM_004663	ATP6V1G1
8157454	4,50	22,66	0,00061	3,13	8,73	0,00103	NM_004888	ATF6
7906819	4,50	22,63	0,00006	3,93	15,25	0,00010	NM_007348	PRPS1
8169240	4,50	22,62	0,00146	4,22	18,62	0,00021	NM_002764	ZBTB38
8083090	4,50	22,61	0,00014	3,56	11,79	0,06133	NM_001080412	CTSB
8149330	4,49	22,53	0,00127	4,27	19,27	0,00137	NM_147780	USP10
7997633	4,49	22,51	0,00209	4,71	26,13	0,00142	NM_005153	GORASP2
8046306	4,49	22,50	0,00024	2,72	6,61	0,01825	NM_015530	TMEM123
7951207	4,49	22,48	0,00032	3,12	8,71	0,00226	NM_052932	CXCL12
7933194	4,49	22,48	0,00200	-0,86	-1,81	0,01923	NM_000609	PAPSS1
8102214	4,49	22,47	0,00130	3,45	10,91	0,00004	NM_005443	EIF3L
8072946	4,49	22,44	0,00044	3,73	13,24	0,00806	NM_016091	PDIA3
7983274	4,49	22,44	0,00004	3,95	15,47	0,00004	NM_005313	TOR1AIP2
7922637	4,49	22,43	0,00045	3,83	14,18	0,00046	NM_145034	HNRNPC
8047059	4,49	22,42	0,00005	2,74	6,68	0,00005	NM_031314	NQO1
8002303	4,48	22,36	0,00066	3,92	15,15	0,00835	NM_000903	U2AF1
8070655	4,48	22,34	0,00079	3,80	13,94	0,00527	NM_001025204	RPL14
7956743	4,48	22,30	0,00546	3,12	8,69	0,00001	NM_001034996	DENND4A
7989849	4,48	22,24	0,00069	3,48	11,19	0,00110	NM_001144823	CAPN2
7909967	4,47	22,23	0,00204	3,84	14,33	0,00016	NM_001748	PDCD1LG2
8154245	4,47	22,21	0,00143	-1,08	-2,11	0,01037	NM_025239	DYM
8023228	4,47	22,21	0,00226	3,65	12,57	0,00071	NM_017653	RBMS2
7956261	4,47	22,21	0,00030	2,49	5,60	0,06281	NM_002898	TIMM23
7927519	4,47	22,19	0,00026	2,93	7,62	0,00002	NM_006327	CCNI
8101212	4,47	22,18	0,00116	4,56	23,57	0,00010	NM_006835	ITGAV
8046861	4,47	22,17	0,00087	3,90	14,91	0,00004	NM_002210	ADAM12
7936968	4,47	22,15	0,00122	-0,62	-1,54	0,07221	NM_003474	RPS3A
8097782	4,47	22,14	0,00412	3,82	14,14	0,00181	NM_001006	CDH6
8104663	4,47	22,14	0,00070	-2,07	-4,19	0,00047	NM_004932	GOLGA5
7976307	4,47	22,12	0,00025	3,50	11,28	0,00247	NM_005113	COL12A1
8127563	4,47	22,11	0,00054	1,55	2,92	0,00719	NM_004370	TTC17
7939434	4,47	22,09	0,00018	4,28	19,38	0,00400	NM_018259	PGM3
8127841	4,46	22,08	0,00242	3,16	8,93	0,00020	NM_015599	ZSWIM6
8105506	4,46	22,07	0,00046	3,53	11,56	0,01444	NM_020928	SNORD116-17
7981982	4,46	22,00	0,00024	-1,18	-2,27	0,56422	NR_003332	SNORD116-17
7981986	4,46	22,00	0,00024	-1,18	-2,27	0,56422	NR_003332	ILF2
7920317	4,46	21,96	0,00084	3,80	13,93	0,00516	NM_004515	GRAMD3
8107673	4,46	21,95	0,00031	3,68	12,84	0,00045	NM_023927	THOC7
8088526	4,45	21,92	0,00001	2,13	4,37	0,00318	NM_025075	XPO6
8000482	4,45	21,88	0,00012	5,25	38,09	0,00077	NM_015171	MRPL35
8043278	4,45	21,87	0,00510	3,66	12,65	0,00058	NM_016622	ATP13A3
8092849	4,45	21,85	0,00032	4,93	30,48	0,00019	NM_024524	WAC
7926851	4,45	21,84	0,00019	2,48	5,58	0,04078	NR_024557	CTNNB1
8079021	4,44	21,76	0,00088	3,66	12,61	0,01667	NM_001904	---
7917530	4,44	21,68	0,00024	0,34	1,26	0,63700	--- NCRNA00152	
8043363	4,44	21,63	0,00158	4,28	19,46	0,00367	NR_024204	ARHGEF12
7944560	4,43	21,59	0,00094	4,29	19,53	0,00878	NM_015313	TMEM167B
7903586	4,43	21,58	0,00046	3,75	13,43	0,00098	NM_020141	TMEM167B
8039905	4,43	21,58	0,00046	3,75	13,43	0,00098	NM_020141	HAUS1
8021101	4,43	21,55	0,00006	2,21	4,63	0,06985	NR_026978	POLR2J4
8139264	4,43	21,55	0,00030	3,26	9,58	0,00818	NR_003655	PHC3

8092035	4,43	21,54	0,00045	3,30	9,82	0,03948	NM_024947	IAH1
8040163	4,42	21,47	0,00115	4,74	26,79	0,00028	NM_001039613	LARP4
7955361	4,42	21,46	0,00233	3,36	10,24	0,00768	NM_052879	ZNF43
8035813	4,42	21,42	0,00009	0,41	1,33	0,14378	NM_003423	RAB5B
7956088	4,42	21,41	0,00007	3,87	14,63	0,00058	NM_002868	FAF2
8110169	4,42	21,38	0,00037	4,39	21,01	0,00217	NM_014613	UBXN7
8093112	4,42	21,38	0,00027	3,97	15,69	0,00377	NM_015562	BMPR2
8047538	4,41	21,33	0,00333	3,07	8,38	0,01173	NM_001204	DCTD
8103859	4,41	21,27	0,00017	4,51	22,78	0,00008	NM_001012732	MBNL2
7969677	4,41	21,24	0,00006	2,26	4,79	0,00200	NM_144778	ATF7IP
7954104	4,41	21,24	0,00017	2,02	4,05	0,17558	NM_018179	MOSPD2
8166140	4,41	21,19	0,00040	3,39	10,46	0,00522	NM_152581	TMED2
7959597	4,40	21,18	0,00016	2,42	5,35	0,04798	NM_006815	NUFIP2
8013908	4,40	21,13	0,00009	3,38	10,42	0,02871	NM_020772	RAB8B
7984112	4,40	21,08	0,00113	2,17	4,50	0,00090	NM_016530	TIMM23
7933582	4,40	21,05	0,00013	3,37	10,37	0,00069	NM_006327	TMED9
8110392	4,40	21,05	0,00338	3,02	8,12	0,00053	NM_017510	ATP1A1
7904254	4,39	21,02	0,00044	3,30	9,88	0,01911	NM_000701	PLP2
8167449	4,39	21,01	0,00056	4,09	17,03	0,00003	NM_002668	OCIAD1
8094974	4,39	20,98	0,00023	3,52	11,48	0,01308	NM_017830	B4GALT5
8066939	4,39	20,96	0,00343	4,37	20,62	0,00003	NM_004776	VTA1
8122348	4,39	20,94	0,00117	3,76	13,54	0,00092	NM_016485	HADHB
8040639	4,38	20,83	0,00088	4,38	20,80	0,00001	NM_000183	SNAPIN
7905598	4,38	20,83	0,00023	4,10	17,19	0,00300	NM_012437	LAMC1
7908041	4,38	20,79	0,00003	3,15	8,86	0,00223	NM_002293	CBLL1
8135458	4,37	20,70	0,00014	3,01	8,07	0,00207	NM_024814	---
8099631	4,37	20,69	0,00005	3,37	10,31	0,00045	--- MPV17	
8051066	4,37	20,65	0,00334	4,89	29,67	0,00000	NM_002437	MFSD1
8083656	4,37	20,64	0,00618	4,15	17,69	0,00001	NM_022736	STX12
7899361	4,36	20,60	0,00018	3,40	10,53	0,00012	NM_177424	TTC37
8113157	4,36	20,58	0,00063	2,92	7,57	0,00125	NM_014639	FAR2
7954631	4,36	20,58	0,00320	-0,66	-1,58	0,00391	NM_018099	RHOT1
8006345	4,36	20,57	0,00007	3,79	13,84	0,00758	NM_001033568	IFT20
8013581	4,36	20,53	0,00146	3,28	9,70	0,00170	NM_174887	CAPRIN1
7939242	4,35	20,45	0,00058	3,64	12,49	0,00031	NM_005898	GJA1
8121749	4,35	20,42	0,00079	1,00	2,00	0,03523	NM_000165	COPZ2
8016390	4,35	20,38	0,00027	0,68	1,60	0,18244	NM_016429	MFGE8
7991234	4,35	20,34	0,00102	3,28	9,69	0,00928	NM_005928	PSMB6
8003953	4,35	20,34	0,00280	4,51	22,75	0,00023	NM_002798	DSCR3
8070269	4,34	20,31	0,00157	3,97	15,71	0,00002	NM_006052	LRRC17
8135218	4,34	20,25	0,00850	-0,96	-1,94	0,02853	NM_005824	SMNDC1
7936307	4,34	20,25	0,00002	3,39	10,47	0,00360	NM_005871	TNRC6A
7994187	4,34	20,25	0,00011	3,72	13,17	0,00001	NM_014494	SBDS
8139891	4,34	20,24	0,00092	3,03	8,18	0,00554	NM_016038	UCHL1
8094778	4,34	20,23	0,00172	4,16	17,93	0,00074	NM_004181	NNT
8105153	4,34	20,21	0,00151	4,90	29,78	0,00001	NM_012343	HLA-A
8179041	4,34	20,21	0,00249	4,15	17,80	0,00014	D32129 EPS15	
7916045	4,34	20,21	0,00139	2,69	6,46	0,00327	NM_001981	FYTTD1
8084986	4,34	20,18	0,00009	3,29	9,79	0,00179	NM_032288	CFB
8178115	4,33	20,18	0,00013	1,48	2,78	0,00589	NM_001710	SSR1
8123767	4,33	20,17	0,00039	3,91	15,02	0,00003	NM_003144	DNAJC19
7974190	4,33	20,12	0,00980	3,76	13,56	0,00028	NM_145261	RPLP2
7937476	4,33	20,08	0,00733	3,78	13,75	0,00012	NM_001004	UQCRFS1
8073192	4,33	20,07	0,00021	3,86	14,54	0,00004	NM_006003	CFB
8118345	4,32	20,01	0,00008	1,23	2,35	0,01699	NM_001710	PITPNA
8011077	4,32	20,01	0,00055	3,53	11,56	0,01146	NM_006224	RPL35
8164100	4,32	19,99	0,00290	2,92	7,57	0,01601	NM_007209	XRN2
8061324	4,32	19,95	0,00001	3,65	12,54	0,00096	NM_012255	AP2B1
8006542	4,32	19,92	0,00000	4,25	19,08	0,00146	NM_001030006	NPEPPS
8014650	4,31	19,84	0,00008	3,39	10,49	0,01253	NM_006310	---
8103222	4,31	19,84	0,01679	3,32	10,00	0,03240	--- RNFT1	
8017162	4,31	19,83	0,00289	4,73	26,46	0,00032	NM_016125	DRAM2
7918474	4,31	19,82	0,00036	4,37	20,65	0,00016	NM_178454	EIF4G2
7946610	4,31	19,82	0,00068	4,27	19,26	0,00089	NM_001418	SAT1
8166469	4,31	19,81	0,00090	4,15	17,80	0,00543	NR_027783	TXNRD1
7958174	4,31	19,80	0,00575	3,52	11,50	0,02968	NM_003330	FAM103A1
8130765	4,31	19,77	0,00016	4,60	24,21	0,00021	BC112329 IL1R1	
8043995	4,31	19,77	0,00005	0,90	1,86	0,08727	NM_000877	RRM1
7937915	4,30	19,71	0,00029	3,35	10,20	0,00670	NM_001033	AKIRIN2
8128034	4,30	19,67	0,00083	3,55	11,68	0,00015	NM_018064	---
7910385	4,30	19,67	0,02344	2,08	4,24	0,18994	--- PABPC1	
8152079	4,30	19,66	0,01628	3,82	14,13	0,00750	NM_002568	MYBL1
8151101	4,29	19,60	0,00039	4,38	20,78	0,01229	NM_001080416	---

8064857	4,29	19,60	0,00101	2,89	7,42	0,00480	---	MAT2A
8043187	4,29	19,60	0,00557	3,81	13,99	0,00172	NM_005911	COPS3
8013094	4,29	19,56	0,00061	4,42	21,47	0,00148	NM_003653	UBB
8005166	4,29	19,56	0,00403	2,75	6,74	0,02493	NM_018955	STMN2
8147030	4,29	19,55	0,00028	-1,17	-2,25	0,11070	NM_007029	GNPTAB
7965812	4,29	19,51	0,00012	3,38	10,43	0,00470	NM_024312	EIF2S3
8166493	4,28	19,49	0,00004	3,47	11,11	0,00545	NM_001415	DNAJC10
8046759	4,28	19,48	0,00142	4,64	24,86	0,00010	NM_018981	UGP2
8042270	4,28	19,47	0,00017	3,14	8,81	0,00136	NM_001001521	SLC7A11
8102800	4,28	19,42	0,00047	4,42	21,34	0,00027	NM_014331	DPM1
8067017	4,28	19,42	0,00038	4,92	30,21	0,00029	NM_003859	CAMK2D
8102415	4,28	19,37	0,00267	3,26	9,55	0,00068	NM_001221	MRPS11
7985757	4,27	19,36	0,00448	4,10	17,18	0,00195	NM_022839	NXF1
7948839	4,27	19,34	0,00099	2,56	5,88	0,00060	NM_006362	FOS
7975779	4,27	19,30	0,00012	2,07	4,20	0,00803	NM_005252	SULF2
8066822	4,27	19,29	0,00005	-1,05	-2,07	0,03626	NM_018837	EIF4A3
8019046	4,27	19,28	0,00083	3,67	12,69	0,00013	NM_014740	POLR3C
7919226	4,27	19,28	0,00049	4,83	28,49	0,00009	NM_006468	DICER1
7981111	4,27	19,28	0,00013	4,23	18,80	0,00066	NM_177438	CHI3L1
7923547	4,27	19,28	0,00074	-1,11	-2,16	0,02452	NM_001276	ORMDL2
7956031	4,27	19,24	0,00021	5,47	44,26	0,00023	NM_014182	C12orf4
7960411	4,27	19,24	0,00026	2,51	5,69	0,00533	NM_020374	LGALS1
8072876	4,26	19,22	0,00537	3,17	9,03	0,02485	NM_002305	PLEKHA1
7931081	4,26	19,18	0,00137	4,67	25,41	0,00086	NM_021622	ATG3
8089527	4,26	19,16	0,00048	3,85	14,40	0,00149	NM_022488	LUZP6
8143132	4,26	19,16	0,00467	4,76	27,04	0,00013	NM_001128619	C14orf2
7981481	4,26	19,16	0,00052	5,28	38,92	0,00012	NM_004894	PXK
8080781	4,26	19,14	0,00164	3,24	9,46	0,00012	NM_017771	RNF38
8161192	4,26	19,12	0,00033	3,91	15,00	0,00643	NM_194328	COL14A1
8148070	4,26	19,10	0,00022	0,90	1,86	0,00271	NM_021110	HERPUD1
7995895	4,26	19,10	0,00062	3,01	8,07	0,00957	NM_014685	MAN2A1
8107234	4,26	19,09	0,00001	3,05	8,27	0,00483	NM_002372	MBNL1
8083429	4,25	19,06	0,00006	2,76	6,77	0,02022	NM_021038	MYOF
7935058	4,25	19,04	0,00047	3,16	8,96	0,03490	NM_013451	SLC17A5
8127549	4,25	19,03	0,00089	3,92	15,09	0,00038	NM_012434	UGDH
8099897	4,25	19,03	0,00269	3,70	12,97	0,03265	NM_003359	YWHAB
8062880	4,25	19,01	0,00130	4,72	26,44	0,00401	NM_003404	ARIH1
7984641	4,25	19,01	0,00017	3,86	14,51	0,00416	NM_005744	CHCHD3
8143028	4,25	19,01	0,00001	3,50	11,33	0,00148	NM_017812	RPS27
8079019	4,25	18,99	0,01096	2,78	6,85	0,00225	NM_001030	C19orf2
8027416	4,25	18,99	0,00002	3,61	12,22	0,01555	NM_003796	ADH5
8101844	4,24	18,92	0,00168	2,72	6,58	0,07774	NM_000671	ATP5EP2 // ATP5EP2 // ATP5EP2 // ATP5EP2 // ATP5EP2 // ATP5EP2 // ATP5EP2
7968270	4,24	18,91	0,00369	2,49	5,62	0,01940	NR_002162 // NR_002162 // NR_002162 // NR_002162 // NR_002162 // NR_002162 // NR_002162	CNN3
7917885	4,24	18,90	0,00209	2,43	5,39	0,01241	NM_001839	CLIC4
7898988	4,24	18,88	0,00077	2,64	6,24	0,00257	NM_013943	P4HB
8019762	4,24	18,88	0,00261	3,73	13,25	0,00012	NM_000918	EIF1
8007141	4,24	18,87	0,01356	2,20	4,60	0,11421	NM_005801	ZNF292
8120992	4,22	18,69	0,00065	3,24	9,46	0,00303	NM_015021	NAE1
8001876	4,22	18,67	0,00128	4,73	26,58	0,00132	NM_001018159	ATXN7L1
8142100	4,22	18,66	0,00054	2,52	5,72	0,00305	NM_020725	SENP1
7962760	4,22	18,60	0,00576	4,34	20,27	0,01644	NM_014554	PRKAR2B
8135378	4,22	18,59	0,00277	1,00	2,00	0,07569	NM_002736	MAPK1IP1L
7974455	4,21	18,57	0,00327	3,84	14,34	0,00128	NM_144578	PIGU
8065762	4,21	18,57	0,00011	4,79	27,67	0,00011	NM_080476	WDR26
7924582	4,21	18,57	0,00072	4,87	29,27	0,00040	NM_025160	MED14
8172119	4,21	18,53	0,00052	4,31	19,88	0,00024	NM_004229	P4HB
8019250	4,21	18,52	0,00084	3,78	13,69	0,00068	NM_000918	LTA4H
7965627	4,21	18,51	0,00029	3,81	13,99	0,00637	NM_000895	ENTPD7
7929750	4,21	18,47	0,00047	2,47	5,55	0,00540	NM_020354	TMEM209
8142912	4,21	18,47	0,00047	3,64	12,50	0,01399	NM_032842	NID2
7979133	4,20	18,44	0,00247	0,19	1,14	0,40438	NM_007361	PEA15
7906564	4,20	18,44	0,00014	3,58	12,00	0,01041	NM_003768	XRN1
8091141	4,20	18,41	0,00066	3,18	9,04	0,00639	NM_019001	HMGCS1
8111941	4,20	18,39	0,00029	4,69	25,88	0,00002	NM_001098272	STAM
7926385	4,20	18,39	0,00186	4,04	16,42	0,00007	NM_003473	AMZ2
8009432	4,20	18,36	0,00098	3,25	9,53	0,00433	NM_016627	RBM9
8075673	4,20	18,33	0,00267	3,93	15,21	0,00002	NM_001082578	CAPZB
7913169	4,19	18,31	0,00218	2,69	6,47	0,00090	NM_004930	TNPO3
8142852	4,19	18,28	0,00218	4,32	19,94	0,00000	NM_012470	UBE2Q1
7920515	4,19	18,27	0,00332	5,48	44,78	0,00040	NM_017582	SERPING1
7940028	4,19	18,26	0,00109	1,44	2,71	0,09416	NM_000062	FAM103A1

7985488	4,19	18,23	0,00023	4,33	20,13	0,00051	BC112329 EIF4A2	
8084694	4,19	18,21	0,00417	4,90	29,93	0,00509	NM_001967	RBPMS
8145669	4,18	18,17	0,00126	1,74	3,34	0,00702	NM_001008711	GABARAPL2
7997272	4,18	18,10	0,00127	4,74	26,67	0,00005	NM_007285	PSMA4
7985202	4,18	18,07	0,00237	4,71	26,17	0,00005	NM_002789	ANKRD13A
7958600	4,17	18,06	0,00113	3,25	9,54	0,02313	NM_033121	---
8118667	4,17	18,05	0,00294	3,25	9,52	0,01473	--- C14orf135	
7974771	4,17	18,01	0,00003	4,25	18,97	0,00652	NM_022495	CDC26
8163481	4,17	17,99	0,00140	3,01	8,06	0,00092	NM_139286	ITGB8
8131666	4,17	17,97	0,00167	0,36	1,29	0,51638	NM_002214	BAG2
8120402	4,17	17,94	0,00451	3,38	10,45	0,00554	NM_004282	ABHD5
8079153	4,16	17,91	0,00008	3,01	8,08	0,00026	NM_016006	ATOX1
8115346	4,16	17,91	0,00115	3,61	12,19	0,01111	NM_004045	ARF3
7962904	4,16	17,87	0,00044	4,28	19,41	0,00009	NM_001659	IFITM2
7937330	4,16	17,86	0,00049	2,42	5,36	0,00841	NM_006435	RANBP9
8123989	4,16	17,85	0,00076	3,68	12,83	0,00031	NM_005493	PRRX1
7907222	4,16	17,85	0,00714	-1,78	-3,43	0,00100	NM_006902	KIAA2026
8160011	4,16	17,83	0,00051	3,25	9,53	0,00043	NM_001017969	EEA1
7965436	4,16	17,83	0,00017	1,71	3,28	0,00410	NM_003566	PARK7
7897404	4,16	17,82	0,00069	3,77	13,64	0,00125	NM_007262	RPL41
7965467	4,15	17,80	0,06662	2,54	5,82	0,05601	NM_021104	RPL41
7982129	4,15	17,80	0,06662	2,54	5,82	0,05601	NM_021104	CFL2
7978586	4,15	17,80	0,00042	3,44	10,88	0,00031	NM_021914	TRIM33
7918725	4,15	17,79	0,00064	3,24	9,47	0,00623	NM_015906	SPATS2L
8047272	4,15	17,76	0,00228	3,02	8,11	0,00050	NM_015535	EXOSC10
7912385	4,15	17,74	0,00026	2,66	6,34	0,00129	NM_001001998	SEC23B
8061186	4,15	17,73	0,00060	3,44	10,82	0,00253	NM_006363	RPL10A
8118974	4,15	17,72	0,00393	4,41	21,28	0,00104	NM_007104	SLC39A6
8022927	4,15	17,72	0,00061	3,65	12,57	0,00675	NM_012319	ITGA5
7963786	4,15	17,71	0,00062	3,39	10,49	0,00053	NM_002205	C11orf51
7950128	4,14	17,68	0,00319	2,88	7,35	0,01109	NM_014042	QRICH1
8087271	4,14	17,67	0,00123	3,54	11,60	0,00686	NM_017730	KARS
8002919	4,14	17,67	0,00039	3,54	11,61	0,00024	NM_001130089	06. Mrz
8104463	4,14	17,63	0,00188	4,49	22,40	0,00600	NM_005885	ARV1
7910494	4,14	17,62	0,00022	4,17	17,99	0,00013	NM_022786	GPNMB
8131844	4,14	17,62	0,00051	-0,85	-1,81	0,04590	NM_001005340	ENY2
8147883	4,14	17,58	0,00008	4,33	20,10	0,00108	NM_020189	CAND1
7956910	4,14	17,58	0,00081	3,63	12,36	0,01158	NM_018448	CUL3
8059393	4,13	17,55	0,00093	2,67	6,37	0,05879	NM_003590	USP14
8019807	4,13	17,55	0,00031	3,05	8,31	0,02715	NM_005151	C6orf125
8125775	4,13	17,53	0,00069	4,04	16,48	0,00221	AY568085 DDHD1	
7979223	4,13	17,51	0,00048	3,67	12,74	0,00017	NM_001160148	ATP2A2
7958644	4,13	17,51	0,00016	3,85	14,44	0,02184	NM_170665	PDGFRA
8095080	4,13	17,51	0,00019	-1,10	-2,15	0,02417	NM_006206	C6orf62
8124273	4,13	17,51	0,00049	4,36	20,47	0,00153	NM_030939	SPA17
7944869	4,13	17,50	0,00126	2,72	6,58	0,00793	NM_017425	PTPLB
8090091	4,13	17,49	0,00128	3,85	14,43	0,01741	NM_198402	CHMP2A
8039796	4,13	17,47	0,00375	4,14	17,67	0,00011	NM_014453	SLC35D2
8162586	4,13	17,46	0,00065	3,96	15,57	0,00014	NM_007001	AK2
7916590	4,12	17,45	0,00119	4,07	16,81	0,00224	NM_001625	HSD17B11
8101648	4,12	17,43	0,00457	4,81	28,10	0,00037	NM_016245	PCBP2
7955817	4,12	17,38	0,00156	4,29	19,57	0,00192	NM_005016	S100A11
7920128	4,12	17,35	0,06438	3,58	11,94	0,02611	NM_005620	PARVA
7938528	4,12	17,34	0,00440	2,07	4,20	0,00150	NM_018222	SAMD9
8140967	4,11	17,32	0,00078	0,15	1,11	0,68809	NM_017654	CNIH
7979250	4,11	17,30	0,00038	4,34	20,24	0,00008	NM_005776	CRBN
8085081	4,11	17,30	0,00081	2,38	5,21	0,00291	NM_016302	XRCC5
8048146	4,11	17,29	0,00003	3,57	11,90	0,00103	NM_021141	TRAPPC4
7944375	4,11	17,29	0,00383	4,77	27,37	0,00002	NM_016146	---
8111210	4,11	17,23	0,06733	2,65	6,28	0,00098	--- LARS	
8114861	4,11	17,23	0,00014	3,84	14,34	0,00655	NM_020117	TM9SF3
7935320	4,10	17,20	0,00113	3,69	12,89	0,01480	NM_020123	GTF2H5
8123038	4,10	17,20	0,00044	2,21	4,64	0,00814	NM_207118	VAMP3
7897370	4,10	17,18	0,00218	3,85	14,44	0,00010	NM_004781	ATP6V1B2
8144931	4,10	17,17	0,00159	4,01	16,07	0,00036	NM_001693	TUBG1
8007302	4,10	17,16	0,00597	4,94	30,77	0,00126	NM_001070	IFITM1
7937335	4,10	17,15	0,00042	1,93	3,80	0,00972	NM_003641	KLHL28
7978760	4,10	17,13	0,00148	2,20	4,58	0,02616	NM_017658	GBAS
8132929	4,10	17,12	0,00630	4,64	24,98	0,00110	NM_001483	CKS1B
8112327	4,09	17,06	0,00007	2,48	5,59	0,09908	NM_001826	IPMK
7933723	4,09	17,02	0,00024	3,06	8,33	0,00947	NM_152230	REXO2
7944011	4,09	17,00	0,00221	2,69	6,44	0,00023	NM_015523	CTSA
8063078	4,08	16,94	0,00329	3,34	10,13	0,00000	NM_001127695	RPL28

8031483	4,08	16,93	0,00448	2,91	7,52	0,00082	NM_001136134	C20orf30
8064833	4,08	16,88	0,00293	3,71	13,08	0,00001	NM_001009924	NSF
8007885	4,08	16,87	0,00016	4,49	22,42	0,00307	NM_006178	PAMR1
7947512	4,08	16,86	0,00561	-1,21	-2,32	0,00075	NM_015430	SLC2A3
7960865	4,08	16,86	0,00013	3,07	8,42	0,05699	NM_006931	STXBP1
8158059	4,08	16,86	0,00125	4,80	27,82	0,00098	NM_003165	MTHFD2
8042830	4,08	16,86	0,00149	3,65	12,58	0,00092	NR_027405	DCBLD2
8089082	4,08	16,86	0,00139	3,47	11,05	0,02541	NM_080927	PRRC1
8107750	4,07	16,85	0,00039	2,96	7,77	0,00376	NM_130809	RNF115
7904830	4,07	16,83	0,00117	4,59	24,02	0,00157	NM_014455	HEXB
8106252	4,07	16,82	0,00112	2,97	7,83	0,00982	NM_000521	TMEM106B
8131539	4,07	16,80	0,00245	2,69	6,47	0,00319	NM_018374	MRPL51
7960553	4,07	16,79	0,00032	4,57	23,80	0,00127	NM_016497	PDS5A
8099926	4,07	16,76	0,00014	3,36	10,26	0,02217	NM_001100399	ZDHHC13
7938816	4,06	16,73	0,00091	4,14	17,61	0,00118	NM_019028	SNAI2
8150698	4,06	16,73	0,00018	2,93	7,64	0,00301	NM_003068	PAFAH1B1
8003758	4,06	16,71	0,00021	4,01	16,09	0,00031	NM_000430	FZD6
8147766	4,06	16,70	0,00140	3,57	11,84	0,03238	NM_003506	ANKRD12
8020068	4,06	16,69	0,00008	1,84	3,58	0,01125	NM_015208	ENPP1
8122099	4,06	16,66	0,00023	3,46	11,00	0,00047	NM_006208	FBXO30
8130032	4,05	16,62	0,00093	2,71	6,54	0,02369	NM_032145	TMEM14C
8116859	4,05	16,62	0,02273	6,91	120,48	0,00001	NM_001165258	TMEM184C
8097704	4,05	16,60	0,00303	4,90	29,85	0,00000	NM_018241	DUSP11
8053046	4,05	16,59	0,00234	3,11	8,61	0,06026	NM_003584	CWC15
7951068	4,05	16,53	0,00026	2,56	5,92	0,00467	NM_016403	ERCC3
8054978	4,05	16,52	0,00029	3,98	15,77	0,00145	NM_000122	TCF12
7983843	4,04	16,50	0,00160	3,14	8,84	0,00544	NM_207036	RQCD1
8048340	4,04	16,48	0,00007	2,82	7,05	0,00189	NM_005444	DNAJC13
8082688	4,04	16,48	0,00009	3,25	9,53	0,00255	NM_015268	SRP72
8095230	4,04	16,48	0,00166	4,27	19,28	0,00522	NM_006947	EPS8
7961546	4,04	16,46	0,00033	3,25	9,50	0,00078	NM_004447	CCNK
7976648	4,04	16,46	0,00117	2,14	4,41	0,03332	NM_001099402	CD151
7937508	4,04	16,46	0,00061	3,94	15,35	0,00350	NM_004357	FGF7
7983630	4,04	16,43	0,00005	-1,30	-2,46	0,00151	NM_002009	C12orf24
7958666	4,04	16,41	0,00101	4,58	23,97	0,00062	NM_013300	MRPS18A
8126646	4,04	16,40	0,00391	4,94	30,63	0,00087	NM_018135	YWHAZ
8152096	4,04	16,40	0,00230	2,92	7,56	0,01482	NM_145690	ASAH1
8149534	4,04	16,39	0,00144	3,47	11,08	0,00018	NM_004315	RBBP4
7899813	4,03	16,38	0,00826	3,99	15,91	0,01208	NM_005610	UFC1
7906662	4,03	16,37	0,00143	4,36	20,59	0,00877	NM_016406	HAS2
8152617	4,03	16,36	0,00352	3,26	9,55	0,00003	NM_005328	HDLBP
8060225	4,03	16,35	0,00010	2,74	6,66	0,00004	NM_203346	RPL39 // RPL39
8127526	4,03	16,34	0,00018	0,47	1,39	0,26406	NM_001000 // NM_001000	PPIB
7989619	4,03	16,34	0,00230	3,44	10,87	0,00275	NM_000942	PRKD1
7978407	4,03	16,33	0,00125	3,12	8,70	0,00019	NM_002742	GSTP1
7941936	4,03	16,32	0,00624	3,50	11,35	0,00009	NM_000852	EIF3E
8152323	4,03	16,29	0,00323	4,21	18,54	0,00043	NM_001568	11. Sep
8095854	4,03	16,29	0,00229	3,49	11,26	0,01004	NM_018243	CCDC53
7965846	4,02	16,25	0,00118	2,66	6,32	0,01601	NM_016053	TRAK2
8058221	4,02	16,24	0,00013	2,47	5,54	0,00012	NM_015049	PAM
8107133	4,02	16,23	0,00103	2,90	7,49	0,00146	NM_000919	FDFT1
8144669	4,02	16,22	0,00322	4,19	18,20	0,00057	NM_004462	WWP1
8147156	4,02	16,21	0,00124	3,17	8,99	0,00213	NM_007013	SEL1L3
8099721	4,02	16,20	0,00033	4,53	23,04	0,00027	NM_015187	TIMP2
8018966	4,02	16,20	0,00073	4,01	16,14	0,00033	NM_003255	ARRDC4
7986350	4,02	16,19	0,01146	-0,43	-1,35	0,17410	NM_183376	SLC39A10
8047174	4,02	16,18	0,00265	3,63	12,34	0,00050	NM_001127257	ALDH1L2
7965979	4,02	16,18	0,00143	2,70	6,49	0,00052	NM_001034173	KIAA0368
8163275	4,02	16,18	0,00022	4,16	17,91	0,00277	NM_001080398	PTPN12
8133788	4,01	16,13	0,00073	3,15	8,87	0,00045	NM_002835	VCAM1
7903358	4,01	16,12	0,00133	-1,34	-2,53	0,00454	NM_001078	PTK2
8153223	4,01	16,11	0,00003	3,70	13,00	0,01021	NM_153831	PGCP
8147469	4,01	16,11	0,00140	1,43	2,69	0,00635	NM_016134	EIF4E2
8049180	4,01	16,10	0,00214	3,42	10,72	0,00013	NM_004846	RPL24
8089249	4,01	16,10	0,00206	3,84	14,31	0,00049	NM_000986	SNORD116-20
7981988	4,01	16,09	0,00139	-1,17	-2,24	0,55311	NR_003334	GDF15
8027002	4,01	16,09	0,00083	5,42	42,87	0,00013	NM_004864	PAIP2
8108424	4,01	16,09	0,00088	2,82	7,06	0,00345	NM_001033112	FAM35A
7928909	4,01	16,08	0,00025	2,48	5,58	0,00760	NM_019054	TNPO1
8106122	4,01	16,08	0,00332	2,66	6,32	0,02965	NM_002270	ATP5E
8067288	4,01	16,07	0,00643	1,45	2,72	0,00388	NM_001001977	GUCY1B3
8097973	4,01	16,07	0,00144	-0,53	-1,45	0,06996	NM_000857	KIAA1033
7958216	4,01	16,07	0,00149	3,61	12,17	0,00120	NM_015275	CDH2

8022674	4,00	16,02	0,00083	-0,78	-1,72	0,07049	NM_001792	CD68
8004510	4,00	16,00	0,00045	2,63	6,18	0,00519	NM_001251	SLC35B1
8016578	4,00	15,99	0,00070	5,08	33,75	0,00000	NM_005827	GNA13
8017711	4,00	15,98	0,00018	3,18	9,09	0,00111	NM_006572	CHRM2
8136448	4,00	15,98	0,00713	-1,18	-2,27	0,03671	NM_001006630	C18orf21
8020919	4,00	15,96	0,00076	4,08	16,96	0,00150	NM_031446	SUB1
8104738	4,00	15,95	0,00486	3,97	15,65	0,00420	NM_006713	PPP1R3C
7934997	3,99	15,94	0,00550	1,03	2,04	0,02462	NM_005398	PAPPA
8157487	3,99	15,94	0,00064	-0,01	-1,01	0,95862	NM_002581	ZNF644
7917604	3,99	15,93	0,00158	3,31	9,92	0,01109	NM_201269	AKR1B1
8143054	3,99	15,92	0,01092	3,47	11,07	0,00660	NM_001628	MUTED
8123802	3,99	15,91	0,00056	3,70	12,98	0,00086	NM_201280	ZNF486
8027260	3,99	15,90	0,00159	3,31	9,92	0,00037	NM_052852	BAT2L2
7907310	3,99	15,89	0,00065	2,06	4,17	0,01864	NM_015172	USP4
8087380	3,99	15,88	0,00108	4,01	16,14	0,00074	NM_003363	FAM45A
7930882	3,99	15,88	0,00055	4,49	22,40	0,00005	NM_207009	HLA-B
8178498	3,99	15,87	0,00437	4,75	26,95	0,00005	NM_005514	HNRNPH2
8168852	3,99	15,87	0,00212	2,40	5,28	0,00041	NM_019597	PSEN1
7975545	3,99	15,85	0,00082	4,68	25,58	0,00144	NM_000021	USP9X
8166826	3,99	15,83	0,00021	3,10	8,56	0,01366	NM_001039590	ETFA
7990566	3,98	15,83	0,00147	3,54	11,63	0,00324	NM_000126	DSE
8121588	3,98	15,83	0,00087	3,64	12,50	0,00036	NM_013352	C3orf17
8089584	3,98	15,82	0,00251	3,86	14,52	0,00366	NM_015412	SF3B14
8050695	3,98	15,82	0,00006	2,42	5,35	0,01498	NM_016047	GLUD1
7934852	3,98	15,80	0,00055	4,42	21,41	0,00004	NM_005271	ZNF675
8035847	3,98	15,80	0,00245	3,03	8,18	0,00592	NM_138330	PRKCI
8083854	3,98	15,78	0,00089	4,55	23,41	0,00640	NM_002740	SETD5
8077528	3,98	15,76	0,00148	3,06	8,34	0,02103	NM_001080517	RAB11B
8025414	3,98	15,74	0,00062	5,68	51,17	0,00001	NM_004218	SF3B3
7997099	3,98	15,74	0,00015	4,16	17,87	0,00025	NM_012426	PNRC2
7898910	3,98	15,74	0,00120	4,33	20,04	0,00011	NM_017761	PNRC2
8039933	3,98	15,74	0,00120	4,33	20,04	0,00011	NM_017761	LANCL1
8058614	3,98	15,73	0,00392	4,43	21,52	0,00026	NM_006055	PSMD10
8174379	3,97	15,71	0,00323	4,48	22,33	0,00040	NM_002814	RAI14
8104788	3,97	15,71	0,00066	1,79	3,45	0,04323	NM_001145525	HPS3
8083282	3,97	15,70	0,00128	4,47	22,12	0,00502	NM_032383	PSAP
7934196	3,97	15,70	0,00128	3,40	10,57	0,00092	NM_002778	USP25
8067903	3,97	15,69	0,00051	3,28	9,68	0,01999	NM_013396	IQGAP1
7986010	3,97	15,69	0,00239	2,87	7,31	0,06797	NM_003870	RPL23A
8005943	3,97	15,68	0,02977	1,49	2,80	0,31836	NM_000984	GYG1
8083272	3,97	15,67	0,00015	1,76	3,38	0,05221	NM_004130	ALDH1A1
8161755	3,97	15,65	0,00007	-1,32	-2,50	0,00565	NM_000689	MED13
8017312	3,97	15,63	0,00032	3,25	9,52	0,06120	NM_005121	SLPI
8066493	3,96	15,61	0,00996	3,92	15,13	0,00084	NM_003064	CRIPT
8041813	3,96	15,61	0,00095	3,61	12,24	0,00040	NM_014171	SOD1
8068168	3,96	15,60	0,00002	3,12	8,69	0,00004	NM_000454	SC4MOL
8098195	3,96	15,60	0,00151	4,82	28,21	0,00001	NM_006745	BNIP3L
8145454	3,96	15,59	0,00100	2,16	4,48	0,05206	NM_004331	TMEM111
8085263	3,96	15,57	0,00023	2,22	4,66	0,00007	NM_018447	LAMA4
8128991	3,96	15,54	0,00040	-1,17	-2,25	0,00026	NM_001105206	IDH1
8058552	3,96	15,51	0,00699	5,40	42,31	0,00059	NM_005896	ARHGAP1
7947681	3,96	15,51	0,00659	2,42	5,36	0,00005	NM_004308	NAP1L1
7965048	3,95	15,47	0,00225	3,64	12,46	0,00099	NM_004537	TMEM66
8150089	3,95	15,47	0,00080	3,85	14,41	0,00079	NM_016127	CLINT1
8115524	3,95	15,47	0,00154	2,87	7,33	0,00707	NM_014666	KRR1
7965022	3,95	15,47	0,00016	3,04	8,20	0,00134	NM_007043	RPS4Y1
8176375	3,95	15,45	0,00010	3,31	9,94	0,00338	NM_001008	ANO10
8086467	3,95	15,44	0,00172	2,54	5,80	0,00026	NM_018075	WDR41
8112746	3,95	15,43	0,00181	4,06	16,69	0,00001	NM_018268	FNDC3A
7969060	3,95	15,42	0,00046	2,48	5,57	0,02309	NM_001079673	ATP6AP2
8166805	3,94	15,40	0,00075	3,19	9,13	0,00074	NM_005765	ATP5C1
7926084	3,94	15,39	0,00036	4,05	16,58	0,00170	NM_001001973	SLC38A9
8112121	3,94	15,37	0,00211	4,51	22,82	0,00141	NM_173514	MSL1
8007023	3,94	15,36	0,00571	3,09	8,53	0,00663	NM_001012241	KDELC2
7951535	3,94	15,35	0,00190	3,75	13,47	0,00008	NM_153705	ARID5B
7927732	3,94	15,34	0,00055	1,18	2,27	0,01335	NM_032199	RAN
8117888	3,94	15,34	0,00105	4,35	20,38	0,00067	NM_006325	STK38L
7954511	3,94	15,33	0,00027	3,00	7,98	0,00048	NM_015000	CUL2
7933047	3,94	15,33	0,01372	3,75	13,46	0,00459	NM_003591	FOXN3
7980680	3,94	15,32	0,00783	2,97	7,83	0,00715	NM_001085471	HLA-B
8124911	3,94	15,31	0,00565	4,83	28,50	0,00003	NM_005514	KLHL24
8084219	3,94	15,31	0,00021	3,28	9,71	0,00392	NM_017644	VPS26A
7927972	3,94	15,31	0,00013	2,87	7,31	0,00310	NM_004896	CTNNA1

8108378	3,94	15,30	0,00045	3,20	9,21	0,00093	NM_001903	SCRN1
8138824	3,94	15,30	0,00221	4,67	25,47	0,00041	NM_014766	ACSL4
8174474	3,94	15,30	0,00001	2,42	5,35	0,00024	NM_022977	ERBB2IP
8105681	3,93	15,29	0,00028	2,74	6,69	0,07916	NM_018695	ORC3L
8121043	3,93	15,28	0,00022	2,69	6,46	0,00304	NM_181837	RPS3A
8141222	3,93	15,26	0,00106	3,66	12,66	0,00180	NM_001006	ARL2BP
7996012	3,93	15,24	0,00006	3,86	14,48	0,00016	NM_012106	HEG1
8090193	3,93	15,23	0,00466	2,09	4,26	0,01216	NM_020733	ARL6IP5
8080926	3,93	15,23	0,00106	3,04	8,25	0,00105	NM_006407	CASK
8172158	3,93	15,23	0,00038	3,72	13,17	0,00331	NM_003688	C5orf13
8113504	3,93	15,22	0,01154	2,85	7,20	0,00015	NM_004772	TCTN3
7935251	3,93	15,22	0,00150	4,43	21,57	0,00037	NM_015631	IPO5
7969703	3,93	15,20	0,00039	3,46	11,02	0,00957	NM_002271	DPF2
7941243	3,92	15,19	0,00079	4,50	22,63	0,00462	NM_006268	MED8
7915516	3,92	15,18	0,00037	2,82	7,05	0,00010	NM_201542	CCNT2
8045381	3,92	15,18	0,00022	2,81	7,01	0,00871	NM_058241	HBP1
8135392	3,92	15,17	0,00291	3,41	10,60	0,00020	NM_012257	---
8102787	3,92	15,17	0,00282	-1,23	-2,35	0,16665	--- SVEP1	
8163202	3,92	15,17	0,00317	-0,29	-1,22	0,20289	NM_153366	RPL7
8109222	3,92	15,16	0,00251	2,74	6,69	0,00244	NM_000971	SAR1B
8114193	3,92	15,16	0,00099	4,29	19,55	0,00096	NM_001033503	FAM57A
8003611	3,92	15,15	0,00057	3,57	11,84	0,00004	NM_024792	TBK1
7956795	3,92	15,13	0,00317	4,00	15,98	0,00086	NM_013254	SNORD41
8034512	3,92	15,11	0,00422	-0,64	-1,56	0,74474	NR_002751	BMS1
8071649	3,92	15,09	0,00131	2,91	7,52	0,05984	BC043345 15. Sep	
7917455	3,91	15,07	0,01208	4,29	19,54	0,00000	NM_004261	ELF1
7971197	3,91	15,07	0,00172	4,20	18,36	0,00022	NM_172373	GPBP1
8105463	3,91	15,06	0,00105	3,86	14,51	0,00103	NM_022913	TRPM7
7988713	3,91	15,06	0,00009	3,92	15,17	0,00223	NM_017672	VPS39
7987840	3,91	15,05	0,00180	3,71	13,05	0,00003	NM_015289	NCKAP1
8057517	3,91	15,03	0,00043	3,36	10,28	0,03329	NM_013436	CASP4
7951372	3,91	15,03	0,00029	1,94	3,84	0,00166	NM_033306	UBE4A
7944195	3,91	15,02	0,00221	3,13	8,74	0,01114	NM_004788	PTP4A2
7914489	3,91	15,01	0,00010	4,26	19,22	0,00283	NM_080391	BNIP3
7937079	3,91	15,00	0,00098	3,82	14,16	0,00000	NM_004052	ATP5J2
8141295	3,91	15,00	0,00008	3,53	11,56	0,00780	NM_004889	CNOT4
8143088	3,90	14,96	0,00297	2,99	7,95	0,00486	NM_013316	RPS27
7900157	3,90	14,95	0,01482	2,73	6,64	0,00279	NM_001030	ACTN1
7979824	3,90	14,95	0,00515	2,92	7,59	0,00592	NM_001130004	TLK1
8056734	3,90	14,94	0,00623	3,44	10,86	0,00375	NM_012290	ERLEC1
8041967	3,90	14,93	0,00003	1,84	3,57	0,03069	NM_015701	RHOQ
8045289	3,90	14,92	0,00073	3,15	8,90	0,00130	NM_012249	SF3B1
8058024	3,90	14,92	0,00078	2,94	7,69	0,03317	NM_012433	CDV3
8081135	3,90	14,88	0,00017	2,02	4,05	0,09044	NM_001134422	C7orf44
8139244	3,89	14,88	0,01799	5,12	34,85	0,00004	NM_018224	GALNT12
8156770	3,89	14,86	0,00046	3,04	8,21	0,00015	NM_024642	UBAP2L
7905700	3,89	14,85	0,00070	3,89	14,79	0,00088	NM_014847	ABHD13
7969979	3,89	14,83	0,00207	3,54	11,59	0,00588	NM_032859	DPY30
8051387	3,89	14,83	0,00311	3,87	14,59	0,00026	NM_032574	HNRNPC
7977657	3,89	14,82	0,00010	3,00	7,99	0,00002	NM_031314	ACTR2
8042337	3,89	14,81	0,00029	3,31	9,91	0,00175	NM_001005386	PDPK1
7998825	3,89	14,79	0,00191	4,72	26,42	0,00064	NM_002613	RAB10
8040618	3,89	14,79	0,00298	4,17	18,01	0,00245	NM_016131	KPNA2
8009417	3,89	14,78	0,00061	4,66	25,24	0,00035	NM_002266	PTPRM
8019988	3,89	14,78	0,00032	4,38	20,88	0,00231	NM_001105244	ASAP1
8152867	3,89	14,78	0,00060	3,54	11,66	0,00015	NM_018482	DNTTIP2
7917771	3,88	14,75	0,00011	3,04	8,20	0,00338	NM_014597	CCDC72
8079630	3,88	14,73	0,00478	4,35	20,43	0,00002	NM_015933	MSL2
8090893	3,88	14,71	0,01063	3,30	9,82	0,03527	NM_018133	ZBTB38
8083092	3,88	14,71	0,00335	3,13	8,73	0,00117	NM_001080412	DCAF6
7907104	3,88	14,68	0,00069	3,17	9,02	0,00143	NM_018442	MKLN1
8136259	3,87	14,65	0,00053	3,46	11,00	0,00014	NM_013255	GLB1
8086028	3,87	14,65	0,00127	4,52	22,95	0,00000	NM_000404	ZMYM2
7967900	3,87	14,64	0,00005	2,05	4,13	0,19068	NM_003453	PSD3
8149551	3,87	14,63	0,01595	3,87	14,66	0,00061	NM_015310	REPS1
8129901	3,87	14,58	0,00036	3,78	13,72	0,00314	NM_031922	AK3L1
7962183	3,87	14,57	0,00022	2,66	6,32	0,01775	NM_001005353	APLP2
7945182	3,86	14,57	0,00429	3,76	13,54	0,02335	NM_001642	IMPAD1
8150906	3,86	14,56	0,01852	4,79	27,74	0,00388	NM_017813	SNAPC3
8154394	3,86	14,56	0,00069	3,35	10,22	0,01499	NM_001039697	SOS1
8051670	3,86	14,55	0,00056	3,26	9,58	0,00813	NM_005633	DRG1
8072488	3,86	14,51	0,00030	3,13	8,75	0,00274	NM_004147	GNG2
7974341	3,86	14,49	0,00016	-0,41	-1,33	0,14472	NM_053064	NRBP1

8040927	3,86	14,49	0,00460	4,23	18,78	0,00003	NM_013392	TMED4
8139411	3,86	14,48	0,00374	4,85	28,94	0,00102	NM_182547	MNAT1
7974799	3,86	14,48	0,00067	3,38	10,40	0,00146	NM_002431	COPS6
8134699	3,86	14,48	0,00329	2,60	6,06	0,00167	NM_006833	TMCO1
7921987	3,85	14,47	0,00533	4,82	28,27	0,00012	NM_019026	FBN1
7988467	3,85	14,47	0,00073	-0,06	-1,04	0,81852	NM_000138	POLR2K
8147654	3,85	14,45	0,00019	2,24	4,72	0,00082	NM_005034	RPL39 // RPL39
8174710	3,85	14,45	0,00017	0,41	1,32	0,31408	NM_001000 // NM_001000	MYLK
8090098	3,85	14,44	0,00054	0,38	1,30	0,05824	NM_053025	GLT8D2
7965941	3,85	14,42	0,00188	-0,85	-1,81	0,10937	NM_031302	ANXA6
8115234	3,85	14,42	0,00055	2,90	7,49	0,00228	NM_001155	STK38
8126018	3,85	14,40	0,00024	3,77	13,63	0,00014	NM_007271	ANXA2
7989335	3,85	14,40	0,02711	1,89	3,71	0,06280	NM_001002858	FAM198B
8103415	3,85	14,37	0,00021	-1,34	-2,54	0,00075	NM_001128424	CTDSPL2
7983335	3,84	14,36	0,00006	2,19	4,56	0,02612	NM_016396	GLO1
8126135	3,84	14,35	0,00030	2,80	6,98	0,01628	NM_006708	MTF2
7903032	3,84	14,32	0,00011	4,76	27,17	0,00008	NM_007358	COPB1
7946703	3,84	14,31	0,00185	3,22	9,33	0,00908	NM_016451	NSMAF
8150928	3,84	14,30	0,00033	4,50	22,63	0,00196	NM_003580	SNX13
8138401	3,84	14,29	0,00089	3,00	8,01	0,00900	NM_015132	NRD1
7916077	3,84	14,28	0,00055	4,06	16,64	0,00003	NM_002525	FAM105A
8104570	3,84	14,27	0,00017	1,22	2,34	0,03857	NM_019018	KIAA1109
8097148	3,83	14,23	0,00003	3,37	10,35	0,01791	NM_015312	ARFGEF1
8151149	3,83	14,22	0,00021	3,56	11,80	0,01005	NM_006421	PSMB7
8164067	3,83	14,19	0,00478	3,53	11,58	0,00008	NM_002799	RC3H2
8163972	3,83	14,19	0,00345	3,91	14,99	0,00420	NM_001100588	C7orf36
8132399	3,83	14,19	0,00325	3,46	10,98	0,00482	NM_020192	ZEB1
7926916	3,82	14,17	0,00266	2,43	5,40	0,00147	NM_030751	RPL5
8076511	3,82	14,16	0,00400	3,03	8,15	0,05556	NM_000969	VPS45
7905099	3,82	14,15	0,00012	4,89	29,73	0,00001	NM_007259	FABP3
7914342	3,82	14,13	0,00074	-1,18	-2,27	0,01328	NM_004102	B4GALT1
8160637	3,82	14,13	0,00311	4,35	20,34	0,00226	NM_001497	PIGK
7917088	3,82	14,12	0,00294	3,65	12,54	0,00068	NM_005482	PIK3C2A
7946815	3,82	14,11	0,00093	3,55	11,72	0,00941	NM_002645	GCSH
8114778	3,82	14,11	0,01016	2,81	7,00	0,00422	NM_004483	RPL31
8043932	3,82	14,11	0,00002	3,02	8,13	0,00310	NM_001099693	VCP
8160914	3,82	14,10	0,00114	3,98	15,75	0,00211	NM_007126	CNBP
8090546	3,82	14,10	0,00015	4,20	18,43	0,00033	NM_001127192	ANKRD28
8085628	3,82	14,08	0,00028	4,06	16,66	0,00010	NM_015199	PLK2
8112202	3,81	14,05	0,00018	3,74	13,33	0,00006	NM_006622	COG5
8142143	3,81	14,03	0,00015	3,96	15,57	0,00055	NM_006348	LAMB1
8142194	3,81	14,03	0,00079	2,77	6,80	0,00948	NM_002291	SCD
7929816	3,81	14,02	0,00011	3,13	8,73	0,02537	NM_005063	HNRNPK
8162047	3,81	14,01	0,00549	4,39	21,00	0,00133	NM_002140	NONO
8168280	3,81	13,99	0,02414	3,07	8,41	0,00035	NM_001145408	GFPT1
8052762	3,81	13,98	0,00019	3,48	11,18	0,00690	NM_002056	---
8132589	3,80	13,97	0,00941	0,77	1,71	0,14534	---	SRPX
8172043	3,80	13,97	0,00037	1,99	3,96	0,00733	NM_006307	FAM127A
8170009	3,80	13,97	0,00863	1,83	3,55	0,00234	NM_001078171	RPS4X
8173513	3,80	13,95	0,00004	3,63	12,35	0,00003	NM_001007	MSN
8167965	3,80	13,95	0,00269	4,78	27,41	0,00000	NM_002444	UROS
7936937	3,80	13,92	0,00029	2,56	5,90	0,00021	NM_000375	CSNK2A2
8001666	3,80	13,92	0,00291	3,86	14,54	0,01066	NM_001896	C11orf75
7951004	3,80	13,90	0,01226	3,89	14,78	0,00007	NM_020179	HSPA4L
8097335	3,80	13,90	0,00155	3,03	8,15	0,00060	NM_014278	TMEM147
8027876	3,80	13,90	0,00481	5,08	33,88	0,00191	NM_032635	ZNF207
8006377	3,80	13,88	0,00079	3,71	13,06	0,00039	NM_001098507	TMEM60
8140500	3,80	13,88	0,00000	3,06	8,31	0,00016	NM_032936	PIK3C3
8021015	3,79	13,88	0,00061	2,10	4,30	0,02930	NM_002647	CDC16
7970347	3,79	13,88	0,00026	2,99	7,97	0,00034	NM_001078645	NOMO3
7993371	3,79	13,88	0,00073	3,54	11,60	0,00649	NM_001004067	NOMO3
7993511	3,79	13,88	0,00073	3,54	11,60	0,00649	NM_001004067	NOMO3
7999791	3,79	13,88	0,00073	3,54	11,60	0,00649	NM_001004067	EIF3C
7994415	3,79	13,87	0,00099	3,63	12,36	0,00045	NM_001037808	EIF3C
8000507	3,79	13,87	0,00099	3,63	12,36	0,00045	NM_001037808	HEXA
7990211	3,79	13,87	0,00543	3,28	9,73	0,00002	NM_000520	SPIN1
8156263	3,79	13,86	0,00897	4,70	25,95	0,00027	NM_006717	BRAF
8143417	3,79	13,86	0,00896	2,82	7,06	0,00127	NM_004333	HEBP2
8122317	3,79	13,86	0,00007	2,20	4,60	0,00218	NM_014320	H3F3B
8018439	3,79	13,85	0,00047	3,89	14,83	0,00003	NM_005324	SNX6
7978570	3,79	13,84	0,00045	3,76	13,55	0,03107	NM_021249	OXSR1
8078738	3,79	13,83	0,00009	2,92	7,57	0,00001	NM_005109	NRAS
7918813	3,79	13,83	0,00031	2,71	6,55	0,00334	NM_002524	RPL9

7990949	3,79	13,82	0,00071	3,18	9,09	0,00123	NM_000661	TRIP12
8059596	3,79	13,82	0,00229	3,86	14,51	0,00118	NM_004238	ID2
8040103	3,79	13,81	0,00111	0,22	1,17	0,52758	NM_002166	RBMS3
8078330	3,79	13,81	0,00013	2,84	7,17	0,00008	NM_001003793	SEMA3C
8140534	3,79	13,80	0,00202	3,78	13,78	0,00021	NM_006379	SURF4
8164883	3,79	13,79	0,00076	3,91	15,00	0,00005	NM_033161	YIPF5
8114829	3,78	13,78	0,00139	2,48	5,57	0,00148	NM_001024947	PCMT1
8122705	3,78	13,77	0,00445	4,89	29,60	0,00006	NM_005389	C1orf27
7908330	3,78	13,77	0,00086	2,30	4,94	0,01684	NM_017847	STARD3NL
8132376	3,78	13,74	0,00006	3,05	8,31	0,00134	NM_032016	ZMYM4
7899957	3,78	13,74	0,00036	3,39	10,50	0,01278	NM_005095	PTMA
8061136	3,78	13,73	0,01131	2,39	5,22	0,04645	NM_002823	CKS1B
7926896	3,78	13,73	0,00005	2,20	4,59	0,10129	NM_001826	TWSG1
8020090	3,78	13,72	0,00299	3,57	11,90	0,00032	NM_020648	TMBIM4
7964745	3,78	13,71	0,00041	3,56	11,76	0,00188	NM_016056	SNORD113-4
7976812	3,78	13,71	0,02377	-0,78	-1,71	0,00103	NR_003232	GMPR
8117034	3,78	13,70	0,00193	4,04	16,43	0,00006	NM_006877	ELL2
7921344	3,78	13,70	0,00005	2,02	4,05	0,04009	NM_012081	MTHFD2
8084064	3,78	13,70	0,00710	3,78	13,74	0,00001	NM_006636	C1orf58
7909931	3,78	13,70	0,01194	4,57	23,67	0,00023	NM_144695	RPS6KA3
8171762	3,78	13,69	0,00012	3,84	14,30	0,00020	NM_004586	EXOC6B
8052956	3,77	13,68	0,01007	3,37	10,35	0,00819	NM_015189	RAPH1
8058390	3,77	13,68	0,00295	3,37	10,34	0,00012	NM_213589	PPP3CA
8101971	3,77	13,68	0,00192	3,60	12,12	0,00014	NM_000944	BTG1
7965423	3,77	13,67	0,00136	3,60	12,09	0,00176	NM_001731	RPL15
8078270	3,77	13,67	0,00163	3,38	10,43	0,00681	NM_002948	ZNF525
8030980	3,77	13,67	0,03717	1,89	3,72	0,12771	NR_003699	C7orf58
8135734	3,77	13,64	0,00052	-0,50	-1,41	0,00544	NM_024913	BICC1
7927681	3,77	13,63	0,00233	2,39	5,26	0,00161	NM_001080512	ERGIC3
8062174	3,77	13,62	0,00029	3,76	13,58	0,00038	NM_198398	C1R
7960744	3,77	13,62	0,00379	1,56	2,95	0,01061	NM_001733	MOBKL1B
8053064	3,77	13,61	0,00524	5,26	38,21	0,00002	NM_018221	CTSC
7950906	3,76	13,57	0,00154	2,87	7,29	0,00018	NM_001814	FBLN1
8073775	3,76	13,56	0,00086	0,10	1,07	0,87878	NM_006486	GNAQ
8161906	3,76	13,56	0,00702	3,91	15,00	0,00019	NM_002072	CDR1
8175531	3,76	13,56	0,00313	-1,01	-2,01	0,01914	NM_004065	NFAT5
7996954	3,76	13,56	0,00135	1,87	3,66	0,07537	NM_138714	MMP1
7951271	3,76	13,55	0,00271	-0,18	-1,14	0,50708	NM_002421	ATP6V1E1
8074251	3,76	13,55	0,00031	1,20	2,30	0,17334	NM_001696	SLC35A5
8081667	3,76	13,55	0,00080	3,64	12,47	0,00205	NM_017945	KIAA0174
7997168	3,76	13,54	0,00608	5,14	35,18	0,00019	NM_014761	YPEL5
8041197	3,76	13,53	0,00017	3,13	8,76	0,00338	NM_001127401	GHITM
7928752	3,76	13,52	0,00249	3,61	12,23	0,00081	NM_014394	RPS15A
7999827	3,76	13,52	0,00107	3,73	13,23	0,00035	NM_001019	C5orf22
8104680	3,76	13,51	0,00034	4,31	19,82	0,00026	NM_018356	DCUN1D4
8095009	3,76	13,51	0,00216	3,54	11,66	0,00006	NM_001040402	NSA2
8106271	3,75	13,50	0,00002	2,62	6,16	0,00173	NM_014886	TNFSF10
8092169	3,75	13,49	0,00102	4,58	23,98	0,00054	NM_003810	JAK1
7916747	3,75	13,47	0,00259	3,93	15,27	0,00034	NM_002227	RPL7
8151376	3,75	13,47	0,00258	2,24	4,71	0,00810	NM_000971	RPS6
8160308	3,75	13,47	0,00074	1,77	3,41	0,09005	NM_001010	NDUFV2
8020058	3,75	13,46	0,00061	3,29	9,79	0,00249	NM_021074	ATP5L
7944216	3,75	13,46	0,00117	2,89	7,40	0,00053	NM_006476	MFF
8048835	3,75	13,45	0,00484	4,38	20,86	0,02911	NM_020194	CCDC92
7967486	3,75	13,44	0,00171	2,85	7,20	0,00069	NM_025140	IL13RA1
8169580	3,75	13,43	0,00003	2,74	6,68	0,00027	NM_001560	TSG101
7947015	3,75	13,42	0,00080	4,10	17,17	0,00012	NM_006292	RPL9
8099887	3,75	13,41	0,00094	3,14	8,83	0,00065	NM_001024921	DENND4C
8154531	3,75	13,41	0,00033	2,31	4,96	0,16573	NM_017925	ZNF765
8039010	3,75	13,41	0,00002	3,61	12,24	0,00067	NM_001040185	UBLCP1
8109597	3,74	13,38	0,00234	3,90	14,94	0,00004	NM_145049	CNIH4
7910014	3,74	13,37	0,00481	4,80	27,87	0,00039	NM_014184	RNASEK
8004237	3,74	13,37	0,00312	4,19	18,21	0,00001	NM_001004333	TMEM48
7916316	3,74	13,36	0,00267	4,64	24,94	0,00060	NM_018087	CRABP2
7921099	3,74	13,36	0,00120	1,75	3,36	0,00551	NM_001878	RNF146
8121927	3,74	13,36	0,00004	1,74	3,34	0,02185	NM_030963	COX5B
8043718	3,74	13,36	0,01322	5,52	45,92	0,00029	NM_001862	FRMD6
7974316	3,74	13,35	0,00042	1,56	2,96	0,02589	NM_001042481	RPL23A
8026440	3,74	13,35	0,05879	0,87	1,83	0,52266	NM_000984	FCF1
7975713	3,74	13,35	0,00016	2,85	7,22	0,00114	NM_015962	SEC61A1
8082408	3,74	13,33	0,00043	3,92	15,14	0,00023	NM_013336	ATIC
8048120	3,74	13,32	0,00249	3,62	12,33	0,00455	NM_004044	FAM98A
8051413	3,74	13,32	0,00108	3,99	15,92	0,00002	NM_015475	KPNA6

7899688	3,74	13,32	0,00468	5,80	55,82	0,00009	NM_012316	RAB7A
8090509	3,73	13,30	0,00225	1,30	2,47	0,10603	NM_004637	PSMG2
8020241	3,73	13,29	0,00101	4,91	30,03	0,00039	NM_020232	PMPCB
8135235	3,73	13,29	0,00183	4,92	30,28	0,00005	NM_004279	PTP4A1
8120431	3,73	13,25	0,00020	3,15	8,87	0,02530	NM_003463	USMG5
8168416	3,73	13,24	0,02277	4,73	26,52	0,00068	NM_032747	RFX7
7989132	3,73	13,22	0,00147	3,21	9,29	0,00805	NM_022841	ACSL1
8103951	3,72	13,19	0,00254	3,34	10,13	0,00046	NM_001995	RBM39
8066009	3,72	13,18	0,00655	3,74	13,37	0,00093	NM_184234	ARPC1A
8134581	3,72	13,18	0,00298	2,80	6,98	0,00677	NM_006409	GNG5
8174509	3,72	13,18	0,00020	3,66	12,63	0,00025	NM_005274	TMEM9B
7946439	3,72	13,18	0,00448	4,59	24,09	0,00020	NM_020644	C15orf41
7982535	3,72	13,15	0,00123	4,52	22,88	0,00232	NM_032499	FAM127B
8175302	3,71	13,11	0,00079	2,72	6,57	0,00082	NM_001078172	RPL7A
8022972	3,71	13,11	0,00377	1,84	3,57	0,03457	NM_000972	USP44
7965565	3,71	13,10	0,00440	3,33	10,02	0,00159	NM_032147	STAT1
8057744	3,71	13,09	0,00096	2,77	6,82	0,00243	NM_007315	TBC1D22B
8119169	3,71	13,08	0,01057	4,31	19,79	0,00067	NM_017772	DLG1
8093191	3,71	13,08	0,00015	2,74	6,66	0,08590	NM_001098424	PBRM1
8087951	3,71	13,08	0,00205	3,05	8,30	0,00084	NM_018165	OBFC2A
8047161	3,71	13,05	0,00734	3,11	8,64	0,00015	NM_001031716	---
7916564	3,71	13,05	0,00115	1,78	3,44	0,17544	--- ESYT2	
8144184	3,71	13,05	0,00020	3,77	13,62	0,00145	NM_020728	RBMS1
8056206	3,71	13,04	0,00525	1,90	3,73	0,02918	NM_016836	ZNF493
8027304	3,70	13,04	0,00016	1,47	2,77	0,05861	NM_001076678	RRN3
7999608	3,70	13,03	0,00388	3,40	10,55	0,00112	NM_018427	RPS3A
8141050	3,70	13,02	0,00047	1,94	3,83	0,01064	NM_001006	ASB3
8052125	3,70	13,02	0,00380	4,29	19,52	0,00072	NM_016115	GUSBP1
8111455	3,70	13,02	0,00106	4,38	20,82	0,00025	NR_027026	CAT
7939298	3,70	13,02	0,00121	3,42	10,73	0,00056	NM_001752	CD2AP
8120102	3,70	13,02	0,00065	3,38	10,39	0,01350	NM_012120	SLC25A36
8083063	3,70	13,00	0,00368	4,97	31,36	0,00146	NM_001104647	FAM38B
8022295	3,70	12,99	0,01180	1,58	3,00	0,00061	NM_022068	NOL7
8116969	3,70	12,98	0,00048	3,27	9,65	0,00075	NM_016167	DERL1
8152628	3,70	12,97	0,02917	5,14	35,22	0,00001	NM_024295	C9orf21
8162624	3,70	12,95	0,00180	2,84	7,16	0,00247	NM_153698	PTPRK
8129418	3,69	12,95	0,00037	2,65	6,26	0,00224	NM_001135648	KPNB1
8007992	3,69	12,94	0,00482	3,73	13,28	0,01107	NM_002265	DPH3
8085660	3,69	12,94	0,00055	2,89	7,39	0,00024	NM_206831	ATP5G1
8008132	3,69	12,94	0,00648	4,23	18,83	0,00005	NM_005175	FAH
7985268	3,69	12,92	0,00449	2,44	5,43	0,00993	NM_000137	SQLE
8148280	3,69	12,92	0,00168	4,07	16,76	0,00005	NM_003129	RAB3GAP2
7924405	3,69	12,92	0,00034	2,30	4,92	0,00312	NM_012414	CD81
7937802	3,69	12,91	0,01867	4,24	18,91	0,00037	NM_004356	FAM126A
8138553	3,69	12,91	0,00081	3,86	14,55	0,00146	NM_032581	NUDT21
8001496	3,69	12,90	0,02535	4,81	28,05	0,00195	NM_007006	RAB13
7963986	3,69	12,90	0,00030	3,48	11,13	0,00001	NM_002870	JUNB
8026047	3,69	12,90	0,00009	2,98	7,87	0,00017	NM_002229	DENND1B
7923131	3,69	12,90	0,00088	3,83	14,21	0,00399	NM_001142795	ZNF253
8027241	3,69	12,89	0,00009	2,98	7,90	0,00116	NM_021047	ZNF480
8030908	3,69	12,88	0,00068	3,07	8,41	0,00208	NM_144684	C19orf53
8026193	3,69	12,88	0,00080	2,10	4,27	0,03126	NM_014047	LLPH
8048703	3,69	12,86	0,00254	3,73	13,29	0,00014	NM_032338	C11orf58
7978754	3,68	12,83	0,02570	3,28	9,70	0,00097	NM_014267	STT3A
7944991	3,68	12,82	0,00079	3,65	12,56	0,00598	NM_152713	CIRH1A
7996891	3,68	12,82	0,00884	5,90	59,91	0,00007	NM_032830	CYB5R3
8076481	3,68	12,81	0,00077	2,75	6,74	0,00012	NM_000398	CSNK2A1
8064351	3,68	12,81	0,00068	3,60	12,15	0,00133	NM_177559	MALAT1
7941272	3,68	12,80	0,00173	1,26	2,39	0,42317	NR_002819	GLG1
8002729	3,68	12,79	0,00822	3,04	8,23	0,02794	NM_001145667	MAP3K1
8105436	3,68	12,78	0,00955	3,03	8,15	0,01521	NM_005921	WDR45L
8019523	3,68	12,77	0,00053	5,38	41,60	0,00055	NM_019613	CPNE3
8147172	3,67	12,77	0,00117	3,55	11,72	0,00406	NM_003909	C5orf51
8105104	3,67	12,77	0,00448	3,89	14,82	0,00182	NM_175921	FAM171B
8046895	3,67	12,74	0,00799	3,27	9,64	0,00091	NM_177454	RMND5A
8043310	3,67	12,73	0,00273	4,41	21,22	0,00003	NM_022780	SGK3
8146717	3,67	12,71	0,01129	4,87	29,21	0,00164	NM_013257	LDB1
7935968	3,67	12,69	0,00089	3,94	15,33	0,00040	NM_003893	HIPK2
8143307	3,67	12,69	0,00052	3,12	8,71	0,02778	NM_022740	GUSBL1
8112558	3,66	12,68	0,00255	4,44	21,69	0,00039	NR_003504	TOR1AIP1
7907773	3,66	12,68	0,00069	3,09	8,53	0,01652	NM_015602	PRKAG1
7962935	3,66	12,68	0,00028	3,02	8,09	0,00179	NM_212461	CDK8
7968199	3,66	12,67	0,00741	3,88	14,74	0,00059	NM_001260	PEX11B

7904755	3,66	12,65	0,00233	3,75	13,48	0,00000	NM_003846	CDC27
8016324	3,66	12,64	0,00025	2,53	5,79	0,00580	NM_001114091	SET
8158372	3,66	12,63	0,00822	4,87	29,34	0,00007	NM_003011	MAPK1
8074791	3,66	12,61	0,00048	4,28	19,42	0,00012	NM_002745	SLC31A1
8157270	3,66	12,61	0,00174	4,33	20,10	0,00250	NM_001859	NECAP1
7953715	3,66	12,61	0,00360	2,69	6,46	0,00016	NM_015509	IGBP1
8168087	3,66	12,60	0,00819	2,69	6,46	0,00006	NM_001551	LOC91316
8074925	3,65	12,57	0,00218	4,48	22,31	0,00065	NR_024448	SEC24D
8102482	3,65	12,57	0,00152	2,14	4,39	0,00022	NM_014822	CCDC72
7899346	3,65	12,57	0,01148	3,75	13,50	0,00235	NM_015933	MGC57346
8007799	3,65	12,55	0,00794	6,35	81,82	0,00001	NR_026680	PSMC4
8028756	3,65	12,54	0,00391	4,14	17,69	0,00060	NM_006503	RPL41
8075691	3,65	12,54	0,08043	1,89	3,71	0,05087	NM_021104	GPX3
8109333	3,65	12,53	0,00292	2,72	6,60	0,00007	NM_002084	BTBD10
7946680	3,65	12,51	0,00190	3,28	9,70	0,00059	NM_032320	FAM69A
7917728	3,64	12,50	0,00003	2,12	4,36	0,00534	NM_001006605	TUBB2C
8159642	3,64	12,50	0,00527	4,39	21,02	0,00649	NM_006088	---
8005736	3,64	12,49	0,00567	2,63	6,18	0,03285	--- FEZ1	
7952490	3,64	12,48	0,00090	0,79	1,73	0,03908	NM_005103	KAT2B
8078227	3,64	12,48	0,00035	2,23	4,71	0,00364	NM_003884	QSER1
7939158	3,64	12,47	0,00019	3,24	9,47	0,00151	NM_001076786	MCL1
7919751	3,64	12,46	0,00090	2,21	4,63	0,06959	NM_021960	CLDND1
8089062	3,64	12,45	0,00028	4,75	26,90	0,00134	NM_019895	LDB2
8099524	3,64	12,43	0,00043	-1,71	-3,26	0,00047	NM_001130834	BOLA2
8000692	3,64	12,42	0,00046	2,54	5,80	0,00612	NM_001031827	UBXN8
8145691	3,63	12,42	0,00268	3,49	11,26	0,00078	NM_005671	THBS2
8130867	3,63	12,41	0,00397	-0,56	-1,48	0,01959	NM_003247	RPS27
7905691	3,63	12,40	0,00618	2,46	5,50	0,00309	NM_001030	CCT6A
8132943	3,63	12,38	0,00062	3,99	15,84	0,00062	NM_001762	SLC16A4
7918426	3,63	12,38	0,00329	-0,68	-1,60	0,01386	NM_004696	HECTD1
7978449	3,63	12,37	0,00033	3,36	10,24	0,01976	NM_015382	LIMA1
7963187	3,63	12,35	0,00148	3,51	11,36	0,00074	NM_001113546	APPL2
7966003	3,63	12,34	0,00001	2,66	6,34	0,00657	NM_018171	HIPK3
7939197	3,62	12,34	0,00138	3,16	8,95	0,00265	NM_005734	ATP1B3
8083136	3,62	12,34	0,00162	3,94	15,34	0,00125	NM_001679	ROCK1
8022441	3,62	12,33	0,00214	3,22	9,32	0,01380	NM_005406	STXBP3
7903541	3,62	12,33	0,00008	2,03	4,08	0,00203	NM_007269	IFNGR1
8129861	3,62	12,32	0,00194	2,92	7,56	0,00058	NM_000416	ATP11B
8084173	3,62	12,31	0,00312	3,62	12,31	0,00021	NM_014616	GOLGA7
8146122	3,62	12,31	0,00907	3,12	8,68	0,00005	NM_016099	RECK
8155169	3,62	12,30	0,00047	2,29	4,88	0,00178	NM_021111	SGK1
8129677	3,62	12,28	0,00015	1,22	2,33	0,00312	NM_001143676	KIAA1430
8103979	3,62	12,27	0,00031	1,36	2,56	0,14924	NM_020827	NOTCH2
7919095	3,62	12,26	0,00310	3,25	9,53	0,00635	NM_024408	RPS24
7928551	3,61	12,25	0,00022	2,01	4,02	0,08389	NM_033022	GABARAPL1
7953943	3,61	12,25	0,00219	3,98	15,76	0,00018	NM_031412	RGPD5
8054676	3,61	12,24	0,00118	2,40	5,30	0,03212	NM_005054	NPTN
7990253	3,61	12,24	0,00192	2,46	5,50	0,00387	NM_012428	CNN2
8024111	3,61	12,23	0,00114	3,93	15,27	0,00124	NM_004368	SETD7
8102848	3,61	12,22	0,00003	3,47	11,09	0,00016	NM_030648	ADSL
8073242	3,61	12,22	0,00165	4,66	25,23	0,00000	NM_000026	RAB6C
8055261	3,61	12,21	0,00128	4,52	22,98	0,00187	NM_032144	PRPF38A
7901447	3,61	12,21	0,00070	3,81	14,02	0,00079	NM_032864	ITGB5
8090162	3,61	12,20	0,00020	2,52	5,73	0,00102	NM_002213	RPL41
8061364	3,61	12,20	0,06778	1,83	3,56	0,03590	NM_021104	TEAD1
7938544	3,61	12,19	0,00076	3,88	14,69	0,00055	NM_021961	AGA
8103834	3,61	12,18	0,00692	2,00	3,99	0,00378	NM_000027	UAP1
7906863	3,60	12,15	0,00123	3,66	12,66	0,00016	NM_003115	VIPAR
7980403	3,60	12,12	0,00237	4,03	16,32	0,00004	NM_022067	ABCC4
7972297	3,60	12,11	0,00096	3,76	13,53	0,00164	NM_005845	HIAT1
7903294	3,60	12,11	0,00046	5,10	34,21	0,00475	NM_033055	ZNF141
8093336	3,60	12,10	0,00008	2,41	5,33	0,00234	NM_003441	TRPC4AP
8065832	3,60	12,09	0,00023	2,01	4,03	0,00672	NM_015638	POLR2B
8095269	3,60	12,09	0,00108	2,88	7,38	0,00006	NM_000938	UQCR10
8072274	3,59	12,07	0,01677	3,76	13,59	0,00044	NM_001003684	DGUOK
8042801	3,59	12,07	0,02252	3,84	14,34	0,00217	NM_080916	VEZT
7957613	3,59	12,07	0,00054	3,59	12,05	0,00002	NM_017599	VKORC1
8000998	3,59	12,06	0,00210	4,36	20,53	0,00009	NM_024006	SNORD53
8041168	3,59	12,04	0,00556	-0,49	-1,40	0,60021	NR_002741	ACSL3
8048733	3,59	12,01	0,00704	3,29	9,78	0,00107	NM_004457	PPIC
8113726	3,59	12,00	0,05715	2,77	6,81	0,00029	NM_000943	GDI1
8170891	3,58	11,99	0,00299	3,72	13,18	0,00423	NM_001493	TPM2
8161044	3,58	11,98	0,00677	-0,41	-1,33	0,31024	NM_003289	PSMD8

8028380	3,58	11,97	0,00041	3,33	10,04	0,00426	NM_002812	SERPINH1
7942596	3,58	11,93	0,00054	3,00	7,99	0,00176	NM_001235	DDAH1
7917347	3,58	11,93	0,00397	3,70	13,01	0,00588	NM_012137	EGR1
8108370	3,57	11,90	0,00066	3,25	9,53	0,00472	NM_001964	RPL9
7990898	3,57	11,89	0,00076	3,06	8,36	0,00112	NM_000661	RPN1
8090490	3,57	11,89	0,01278	3,39	10,51	0,00015	NM_002950	JAZF1
8138789	3,57	11,88	0,00034	2,13	4,39	0,00003	NM_175061	C9orf150
8154381	3,57	11,88	0,00178	3,01	8,03	0,01782	NM_203403	RGPD5
8054532	3,57	11,87	0,00077	2,33	5,03	0,03689	NM_005054	DIRC2
8082120	3,57	11,85	0,00442	4,17	18,04	0,00417	NM_032839	CAV2
8135587	3,57	11,85	0,00329	2,92	7,57	0,00003	NM_001233	UBE2J1
8128111	3,56	11,83	0,00463	4,10	17,20	0,00102	NM_016021	RPL41
8105432	3,56	11,83	0,07983	1,83	3,56	0,04008	NM_021104	AP3S2
7991357	3,56	11,82	0,00073	5,53	46,16	0,00000	NM_005829	RGPD5
8054557	3,56	11,82	0,00232	2,18	4,52	0,06266	NM_032260	NEO1
7984704	3,56	11,77	0,00091	3,86	14,54	0,00106	NM_002499	ASPH
8150988	3,56	11,77	0,00023	1,60	3,03	0,10866	NM_001164750	IFITM3
7945371	3,55	11,74	0,00129	-0,82	-1,77	0,09631	NM_021034	LGALS3BP
8018975	3,55	11,73	0,00007	1,12	2,17	0,07614	NM_005567	MAPK10
8101587	3,55	11,72	0,00217	-0,76	-1,69	0,03598	NM_138982	PPAP2B
7916493	3,55	11,71	0,01807	4,14	17,69	0,00092	NM_003713	UCHL3
7969428	3,55	11,70	0,00012	2,03	4,07	0,00025	NM_006002	FAM102B
7903507	3,55	11,69	0,00637	2,72	6,61	0,00067	NM_001010883	RPL7
7917906	3,55	11,69	0,01148	2,39	5,23	0,00343	NM_000971	C13orf33
7968351	3,55	11,69	0,01505	-0,73	-1,65	0,00972	NM_032849	GPCPD1
8064868	3,55	11,68	0,00354	4,61	24,44	0,00081	NM_019593	---
8143035	3,55	11,68	0,01327	2,96	7,76	0,00287	--- HNRNPA1	
8022814	3,55	11,67	0,02250	1,33	2,51	0,00039	NM_002136	RPS28
8025395	3,54	11,67	0,03811	3,96	15,61	0,00010	NM_001031	ZBTB20
8089701	3,54	11,67	0,00090	1,44	2,71	0,00366	NM_015642	COIL
8016858	3,54	11,66	0,00004	3,51	11,38	0,00325	NM_004645	JAM3
7945262	3,54	11,65	0,00434	2,28	4,84	0,01441	NM_032801	CLN5
7969488	3,54	11,64	0,00259	2,74	6,69	0,00825	NM_006493	ESYT1
7956166	3,54	11,64	0,01016	5,08	33,74	0,00000	NM_015292	PSMC1
8080923	3,54	11,64	0,00233	2,18	4,54	0,00498	NM_002802	CA12
7989501	3,54	11,63	0,00517	0,93	1,90	0,00236	NM_001218	NIPAL2
8151952	3,54	11,63	0,00322	3,23	9,39	0,00048	NM_024759	API5
7939424	3,54	11,61	0,00628	4,92	30,20	0,00011	NM_001142930	C14orf166
7974352	3,54	11,61	0,00127	2,72	6,57	0,00454	NM_016039	ATP5O
8070160	3,54	11,60	0,00137	2,97	7,84	0,00861	NM_001697	KIAA1143
7978542	3,53	11,59	0,00131	2,08	4,24	0,00953	NM_020696	CUTC
7929768	3,53	11,59	0,00415	4,77	27,29	0,00019	NM_015960	LIPA
7934920	3,53	11,58	0,00577	3,81	14,06	0,00003	NM_001127605	LGMN
7980958	3,53	11,57	0,00079	4,02	16,20	0,00359	NM_005606	SCAMP1
8106479	3,53	11,56	0,00246	3,03	8,14	0,00070	NM_004866	DCP1B
7960320	3,53	11,55	0,00628	2,56	5,90	0,00077	NM_152640	NUP155
8111629	3,53	11,54	0,00290	4,29	19,57	0,01563	NM_153485	SYPL1
8142110	3,53	11,53	0,01022	3,46	11,02	0,00055	NM_006754	TGFBR3
7917649	3,53	11,52	0,00347	0,63	1,55	0,17815	NM_003243	NDST1
8109283	3,53	11,51	0,00319	2,68	6,41	0,00534	NM_001543	EEF2
8032730	3,52	11,51	0,00490	3,88	14,76	0,00178	NM_001961	TRPA1
8151341	3,52	11,50	0,00075	-1,13	-2,19	0,01806	NM_007332	FADS2
7940565	3,52	11,50	0,00207	4,24	18,84	0,00222	NM_004265	NDUFV2
8039068	3,52	11,49	0,00041	3,09	8,54	0,00966	NM_021074	BCAT1
7961829	3,52	11,49	0,00256	3,66	12,64	0,00000	NM_005504	PHF10
8123463	3,52	11,48	0,00121	3,60	12,10	0,00252	NM_018288	DDT
8074969	3,52	11,47	0,01213	4,06	16,65	0,00038	NM_001355	PKN2
7902822	3,52	11,47	0,00351	4,71	26,24	0,00095	NM_006256	RHOA
8087409	3,52	11,47	0,00818	3,62	12,26	0,00039	NM_001664	ACOT13
8117219	3,52	11,46	0,00457	3,44	10,87	0,00032	NM_018473	ELMO2
8066716	3,52	11,44	0,00368	3,99	15,93	0,00000	NM_133171	MRPS24
8139250	3,52	11,43	0,03950	5,67	50,90	0,00001	NM_032014	TAOK1
8006030	3,51	11,42	0,00074	3,71	13,09	0,00295	NM_020791	FBXO7
8072610	3,51	11,42	0,00953	3,81	14,03	0,00101	NM_012179	SBNO1
7967420	3,51	11,42	0,00686	3,95	15,45	0,00004	NM_001167856	PMS2L2
8140239	3,51	11,41	0,00216	4,42	21,46	0,00003	NR_003614	KPNA2
8019737	3,51	11,41	0,00045	4,35	20,43	0,00050	NM_002266	SLC16A7
7956658	3,51	11,41	0,00553	-0,97	-1,96	0,01277	NM_004731	NF1
8006239	3,51	11,40	0,00017	3,58	11,94	0,02225	NM_001042492	C12orf35
7954711	3,51	11,40	0,00006	2,01	4,03	0,00560	NM_018169	UBE2G1
8011626	3,51	11,39	0,01033	4,12	17,34	0,01255	NM_003342	JKAMP
7974725	3,51	11,37	0,00177	4,79	27,60	0,00012	NM_016475	ATXN7L1
8142102	3,51	11,35	0,00062	2,63	6,20	0,00237	NM_020725	NAA25

7966462	3,50	11,35	0,01238	4,12	17,38	0,00016	NM_024953	ZFAND2A
8137709	3,50	11,35	0,00204	3,93	15,29	0,00384	NM_182491	ZNF654
8081069	3,50	11,34	0,00030	1,86	3,62	0,03629	NM_018293	NNMT
7943998	3,50	11,34	0,00246	2,68	6,41	0,00611	NM_006169	KLHDC3
8119648	3,50	11,33	0,00122	4,01	16,17	0,00012	NM_057161	PAF1
8036720	3,50	11,33	0,00004	2,73	6,62	0,00213	NM_019088	KCMF1
8043105	3,50	11,32	0,01399	4,93	30,49	0,00089	NM_020122	COPB2
8091048	3,50	11,32	0,00027	2,99	7,97	0,00361	NM_004766	ZNF717
8088911	3,50	11,32	0,00023	5,30	39,47	0,00026	NM_001128223	PSMC1
7976189	3,50	11,31	0,00194	2,17	4,49	0,00752	NM_002802	GDAP2
7918955	3,50	11,30	0,00055	4,06	16,67	0,00017	NM_001135589	NEU1
8178676	3,50	11,29	0,00407	2,69	6,47	0,00286	NM_000434	SKIV2L2
8105353	3,50	11,28	0,00127	3,22	9,35	0,00016	NM_015360	VKORC1L1
8133114	3,50	11,28	0,00087	3,51	11,42	0,00019	NM_173517	TMOD2
7983734	3,50	11,28	0,00131	1,45	2,73	0,01216	NM_014548	SRP54
7973902	3,49	11,27	0,00351	4,30	19,75	0,00068	NM_003136	COL1A1
8016646	3,49	11,27	0,00015	-0,67	-1,59	0,19746	NM_000088	MPHOSPH9
7967386	3,49	11,26	0,00017	3,55	11,74	0,00007	NM_022782	LRRC42
7901577	3,49	11,23	0,00035	2,18	4,53	0,00019	NM_052940	RALGAPB
8062492	3,49	11,23	0,00026	2,95	7,74	0,00048	NM_020336	NDUFA4
8138224	3,49	11,22	0,00033	1,67	3,19	0,03341	NM_002489	ATP2B4
7908940	3,49	11,21	0,00490	2,38	5,20	0,00074	NM_001001396	SNORD5
7951036	3,49	11,21	0,00356	1,53	2,88	0,20256	NR_003033	TMEM62
7983157	3,49	11,20	0,01286	4,12	17,45	0,00004	NM_024956	SCML1
8166278	3,49	11,20	0,00717	3,55	11,71	0,00096	NM_001037540	RPL9
8171834	3,48	11,19	0,00063	3,01	8,05	0,00125	NM_000661	ANTXR2
8101260	3,48	11,19	0,01349	2,26	4,80	0,00328	NM_058172	TXNL1
8023450	3,48	11,17	0,00118	1,73	3,33	0,01073	NR_024546	SGCE
8141035	3,48	11,16	0,00187	4,36	20,47	0,00053	NM_001099401	HMGCR
8106280	3,48	11,16	0,00282	2,83	7,13	0,00914	NM_000859	SLC10A7
8103043	3,48	11,15	0,00079	3,09	8,49	0,00287	NM_001029998	RAB27B
8021301	3,48	11,14	0,00085	1,52	2,88	0,09729	NM_004163	LRRC28
7986394	3,48	11,12	0,01957	4,45	21,88	0,00105	NM_144598	MORC4
8174322	3,48	11,12	0,00227	2,31	4,97	0,01372	NM_024657	RAB31
8020110	3,47	11,11	0,01214	4,88	29,54	0,00413	NM_006868	PDGFRL
8144802	3,47	11,11	0,00177	1,36	2,57	0,00491	NM_006207	MBTPS1
8003089	3,47	11,10	0,00525	3,40	10,57	0,00021	NM_003791	NEDD8
7978201	3,47	11,09	0,02912	3,72	13,21	0,00080	NM_006156	SPG20
7970999	3,47	11,08	0,01300	2,30	4,94	0,00200	NM_015087	NIT2
8081277	3,47	11,07	0,00382	4,81	28,02	0,00072	NM_020202	MYO1D
8014115	3,46	11,04	0,00244	3,09	8,52	0,00282	NM_015194	MLXIP
7959361	3,46	11,01	0,01111	4,37	20,74	0,00002	NM_014938	TMEM183A
7908867	3,46	11,00	0,00595	4,10	17,18	0,00000	NM_138391	KCTD3
7909745	3,46	11,00	0,00275	3,60	12,17	0,00013	NM_016121	CCAR1
7927889	3,46	10,99	0,00256	2,66	6,33	0,03857	NM_018237	DOCK7
7916669	3,46	10,99	0,00023	2,93	7,62	0,00098	NM_033407	RAB5C
8015545	3,46	10,97	0,01605	5,63	49,48	0,00019	NM_201434	VAMP4
7922309	3,46	10,97	0,00679	3,47	11,07	0,00116	NM_003762	EMP1
7954090	3,45	10,96	0,00321	-0,05	-1,04	0,83130	NM_001423	PSMD11
8006392	3,45	10,96	0,00021	3,19	9,10	0,00060	NM_002815	GXYLT2
8080964	3,45	10,96	0,01461	1,17	2,25	0,02036	NM_001080393	TET2
8096675	3,45	10,95	0,00667	2,98	7,86	0,00783	NM_001127208	RPS28
7942824	3,45	10,94	0,04093	3,83	14,25	0,00007	NM_001031	ALCAM
8081431	3,45	10,93	0,00038	2,80	6,96	0,03080	NM_001627	PCMTD2
8097064	3,45	10,93	0,03833	2,81	7,02	0,18171	BC032332 PHF3	
8120441	3,45	10,92	0,00235	1,66	3,16	0,00098	NM_015153	CAMK2N1
7913237	3,45	10,92	0,00028	2,81	7,01	0,00320	NM_018584	YIPF6
8168018	3,45	10,91	0,00294	3,92	15,15	0,00127	NM_173834	PLCB1
8060854	3,45	10,90	0,00308	2,02	4,05	0,00543	NM_182734	STRN
8051443	3,45	10,90	0,00083	3,67	12,70	0,00202	NM_003162	H3F3A
8169740	3,45	10,90	0,03740	2,17	4,52	0,00027	NM_002107	PMS2L5
8140269	3,45	10,90	0,00169	4,26	19,17	0,00006	NR_027775	CCDC6
7933760	3,45	10,89	0,00418	2,73	6,61	0,02328	NM_005436	ETV5
8092578	3,45	10,89	0,00018	3,88	14,74	0,00000	NM_004454	ENAH
7924619	3,44	10,89	0,00057	1,77	3,40	0,01038	NM_001008493	TGFB2
7909789	3,44	10,88	0,00220	3,10	8,58	0,00187	NM_001135599	FAM38B
8022283	3,44	10,88	0,00847	0,21	1,16	0,41244	NM_022068	EIF5AL1
7928630	3,44	10,87	0,00123	3,68	12,80	0,00104	NM_001099692	SP100
8048940	3,44	10,86	0,00067	1,58	2,99	0,00432	NM_001080391	HLA-J
8179049	3,44	10,85	0,00340	4,53	23,10	0,00049	NR_024240	UBE2R2
8154856	3,44	10,85	0,00026	3,00	7,98	0,00550	NM_017811	GALNT1
8020903	3,44	10,85	0,00039	2,67	6,34	0,00003	NM_020474	SMG1
7994006	3,44	10,85	0,00034	3,06	8,36	0,00945	NM_015092	AHCYL1

7903803	3,44	10,84	0,00022	3,78	13,78	0,00050	NM_006621	METTL14
8097066	3,44	10,83	0,00170	2,40	5,28	0,00325	NM_020961	ANTXR1
8042439	3,44	10,83	0,00062	4,39	20,91	0,00004	NM_032208	C5orf15
8114138	3,44	10,83	0,00871	2,54	5,81	0,00036	NM_020199	GMPS
8083523	3,43	10,80	0,00045	3,32	10,00	0,00609	NM_003875	TMEM41B
7946504	3,43	10,80	0,00095	3,27	9,64	0,00680	NM_015012	02. Sep
8049827	3,43	10,79	0,00235	3,55	11,68	0,00227	NM_001008492	STEAP2
8134036	3,43	10,78	0,00217	2,18	4,52	0,07323	NM_152999	RLF
7900395	3,43	10,78	0,00845	2,97	7,83	0,00208	NM_012421	GRN
8007620	3,43	10,78	0,00213	4,39	20,99	0,00222	NM_002087	PITPNB
8075130	3,43	10,75	0,00081	2,54	5,80	0,00314	NM_012399	QARS
8087283	3,43	10,75	0,00743	5,16	35,66	0,00003	NM_005051	MTMR2
7951112	3,42	10,74	0,00223	3,44	10,85	0,00065	NM_201278	HNRPA1L-2
8034313	3,42	10,73	0,02058	1,71	3,28	0,00026	NR_002944	MT2A
7995783	3,42	10,73	0,02369	2,38	5,19	0,03707	NM_005953	PHACTR4
7899455	3,42	10,72	0,03364	4,97	31,26	0,00022	NM_001048183	COL6A3
8059905	3,42	10,71	0,00101	-0,95	-1,93	0,00872	NM_004369	SF3A3
7915130	3,42	10,71	0,00079	3,99	15,93	0,00025	NM_006802	LEPROTL1
8145652	3,42	10,70	0,01007	3,38	10,41	0,00013	NM_015344	COMMD2
8091432	3,42	10,69	0,02829	4,86	28,96	0,00087	NM_016094	PRDX1
7915733	3,42	10,68	0,00199	3,85	14,43	0,00009	NM_002574	BMI1
7926609	3,42	10,68	0,00057	3,95	15,45	0,00087	NM_005180	CFB
8179351	3,42	10,67	0,00823	0,21	1,16	0,53956	NM_001710	MT2A
8095362	3,42	10,67	0,02216	2,62	6,16	0,00159	NM_005953	MAPK9
8116402	3,41	10,66	0,00073	4,12	17,43	0,00036	NM_139068	SMCHD1
8019885	3,41	10,66	0,00051	3,54	11,59	0,01799	NM_015295	RNU1-1
7919269	3,41	10,66	0,03562	-0,63	-1,55	0,39247	NR_004430	RNU1-1
7919349	3,41	10,66	0,03562	-0,63	-1,55	0,39247	NR_004430	ATXN7L1
8142087	3,41	10,66	0,00071	2,39	5,24	0,00958	NM_020725	WRB
8068612	3,41	10,65	0,00886	3,22	9,29	0,00155	NM_004627	HERPUD2
8139003	3,41	10,63	0,00909	2,81	7,03	0,00015	NM_022373	PMS2L4
8139896	3,41	10,62	0,00252	5,52	45,89	0,00008	NR_022007	ORC5L
8142019	3,41	10,62	0,00174	3,32	9,97	0,00065	NM_002553	CNOT2
7957106	3,41	10,61	0,00083	3,24	9,47	0,00070	NM_014515	ST13
8076272	3,40	10,59	0,01154	2,02	4,06	0,00859	NM_003932	NT5C2
7936064	3,40	10,56	0,01816	4,16	17,87	0,00184	NM_012229	SMA5
8105995	3,40	10,56	0,00238	4,51	22,83	0,00067	AK289851 REV3L	
8128894	3,40	10,55	0,00054	2,71	6,53	0,00680	NM_002912	PGD
7897620	3,40	10,55	0,00935	4,73	26,60	0,00087	NM_002631	SCUBE3
8118890	3,40	10,54	0,00186	-0,77	-1,70	0,24746	NM_152753	FAM3C
8166442	3,40	10,54	0,00109	3,45	10,94	0,00003	NM_014888	KIAA0494
7915882	3,40	10,53	0,00398	3,19	9,10	0,00271	NM_014774	FKBP10
8007154	3,39	10,51	0,00284	3,17	8,99	0,00047	NM_021939	MGST3
7906978	3,39	10,50	0,00040	3,95	15,42	0,00019	NM_004528	GALNT5
8045835	3,39	10,49	0,00047	-1,03	-2,05	0,01957	NM_014568	TOX4
7973214	3,39	10,49	0,00054	4,13	17,56	0,00001	NM_014828	PAIP1
8111974	3,39	10,49	0,00005	2,70	6,50	0,00421	NM_006451	ERO1L
7979179	3,39	10,49	0,00441	4,03	16,29	0,00034	NM_014584	TRAM2
8127051	3,39	10,48	0,00654	3,62	12,26	0,00266	NM_012288	METTL9
7993833	3,39	10,48	0,00037	2,26	4,78	0,00004	NM_016025	RNF130
8116372	3,39	10,47	0,00069	3,36	10,27	0,00025	NM_018434	TMTC1
7962058	3,39	10,47	0,00891	-0,89	-1,85	0,00702	NM_175861	PSME2
7978123	3,39	10,47	0,01545	3,06	8,33	0,00595	NM_002818	EIF2AK1
8138045	3,39	10,46	0,00355	4,42	21,40	0,00023	NM_014413	CBLB
8089261	3,39	10,46	0,00162	2,24	4,72	0,00123	NM_170662	EML4
8041592	3,39	10,46	0,01650	3,71	13,11	0,01944	NM_019063	AKAP11
7968835	3,39	10,45	0,00157	2,00	4,00	0,01088	NM_016248	DDOST
7913256	3,38	10,44	0,00117	3,81	14,02	0,00205	NM_005216	EXOC5
7979367	3,38	10,44	0,00199	4,00	16,05	0,00022	NM_006544	---
7907859	3,38	10,44	0,00266	2,07	4,20	0,00582	--- XPO1	
8052526	3,38	10,44	0,00265	3,59	12,08	0,00199	NM_003400	HMG20A
7985119	3,38	10,44	0,00174	3,15	8,86	0,00194	NM_018200	HEATR5A
7978492	3,38	10,44	0,00859	3,87	14,61	0,00275	NM_015473	ARNT
7919825	3,38	10,43	0,00289	3,54	11,64	0,00011	NM_001668	TCP11L2
7958262	3,38	10,42	0,01456	3,75	13,47	0,00066	NM_152772	ENPP5
8126750	3,38	10,42	0,00160	3,88	14,69	0,00694	NM_021572	TMEM106C
7955063	3,38	10,41	0,00367	4,27	19,31	0,00090	NM_001143842	SENP6
8120758	3,38	10,40	0,00114	2,92	7,56	0,00665	NM_015571	KRIT1
8140878	3,38	10,40	0,00114	3,65	12,51	0,00058	NM_194455	EFNA5
8113433	3,38	10,39	0,00105	2,23	4,71	0,01847	NM_001962	RPS27
7923438	3,38	10,39	0,00542	2,47	5,53	0,00059	NM_001030	C5orf41
8110032	3,38	10,38	0,01194	2,28	4,84	0,00597	NM_153607	CALM3
8029831	3,38	10,38	0,00171	3,39	10,48	0,00010	NM_005184	---

8047763	3,37	10,37	0,02766	-0,15	-1,11	0,61385	--- TROVE2	
7908421	3,37	10,36	0,00143	3,34	10,11	0,00661	NM_004600	CCDC99
8109830	3,37	10,36	0,00185	4,39	20,95	0,00011	NM_017785	ZNF626
8035795	3,37	10,35	0,00016	-0,41	-1,33	0,26461	NM_145297	ATM
7943620	3,37	10,35	0,00041	2,65	6,26	0,01044	NM_000051	DSEL
8023727	3,37	10,34	0,00409	-1,64	-3,13	0,05880	NM_032160	RPS26
8007797	3,37	10,33	0,03571	1,73	3,31	0,04452	NM_001029	MFAP4
8013341	3,37	10,32	0,04371	-1,24	-2,37	0,04183	NM_002404	UBA3
8088718	3,37	10,31	0,00326	3,22	9,32	0,00136	NM_003968	ACBD3
7924701	3,37	10,31	0,00082	3,60	12,11	0,00001	NM_022735	EIF5AL1
7934753	3,36	10,30	0,00247	3,65	12,56	0,00070	NM_001099692	SLC38A6
7974816	3,36	10,29	0,00339	3,28	9,73	0,00148	NM_001172702	ERRFI1
7912157	3,36	10,28	0,01380	4,33	20,17	0,00621	NM_018948	PA2G4
7956152	3,36	10,28	0,00886	4,15	17,81	0,00122	NM_006191	PYGL
7979085	3,36	10,27	0,00119	3,82	14,15	0,00730	NM_002863	ZNF138
8133057	3,36	10,27	0,00010	2,73	6,61	0,00185	NR_027660	PRKACB
7902594	3,36	10,27	0,00102	2,89	7,39	0,00057	NM_182948	PRRG1
8166705	3,36	10,26	0,00785	2,21	4,61	0,00744	NM_000950	KDM5B
7923453	3,36	10,24	0,00112	3,07	8,39	0,00060	NM_006618	PMS2L5
8140258	3,36	10,24	0,00282	4,29	19,63	0,00008	NR_027775	STAG2
8169750	3,36	10,24	0,00095	3,15	8,89	0,00064	NM_001042750	GSPT1
7999532	3,35	10,22	0,00038	2,43	5,40	0,00758	NM_002094	CPA4
8136200	3,35	10,22	0,00715	-0,28	-1,21	0,02259	NM_016352	HIGD1A
8086451	3,35	10,20	0,00271	2,57	5,93	0,00046	NM_001099669	HSP90AA6P
8103722	3,35	10,20	0,00864	2,77	6,83	0,02204	AY956762 SAP18	
7967976	3,35	10,20	0,00268	2,01	4,03	0,00613	NM_005870	PRCP
7950731	3,35	10,19	0,03171	2,76	6,78	0,00197	NM_199418	ITM2C
8048995	3,35	10,18	0,00146	3,17	9,01	0,00141	NM_030926	C4orf34
8099912	3,35	10,17	0,00177	4,11	17,31	0,00088	BC008502 HOOK3	
8146243	3,35	10,17	0,00073	2,74	6,66	0,00652	NM_032410	UGGT1
8045090	3,35	10,17	0,00311	3,84	14,34	0,00278	NM_020120	ZNF521
8022612	3,35	10,17	0,00261	-0,89	-1,85	0,01221	NM_015461	YWHAH
8072577	3,35	10,16	0,00483	2,79	6,92	0,00254	NM_003405	FAM160B1
7930682	3,34	10,16	0,00042	2,35	5,10	0,08995	NM_020940	CCNC
8128429	3,34	10,15	0,00344	3,68	12,86	0,00313	NM_005190	NRIP1
8069553	3,34	10,15	0,00148	3,69	12,94	0,01998	NM_003489	SPCS1
8080438	3,34	10,13	0,00574	2,98	7,87	0,00041	NM_014041	IMPACT
8020668	3,34	10,11	0,00040	3,42	10,70	0,00334	NM_018439	PCMTD2
8176865	3,34	10,10	0,02974	2,45	5,45	0,20057	BC032332 PCMTD2	
8177460	3,34	10,10	0,02974	2,45	5,45	0,20057	BC032332 ADH1C	
8101893	3,34	10,10	0,01727	-0,43	-1,35	0,28376	NM_000669	MAPRE1
8061772	3,33	10,09	0,00162	5,25	38,15	0,00026	NM_012325	DR1
7903079	3,33	10,09	0,00061	4,00	15,99	0,00275	NM_001938	SERPINF1
8003667	3,33	10,08	0,00319	0,32	1,25	0,43217	NM_002615	TM4SF1
8091411	3,33	10,08	0,02122	5,42	42,76	0,00166	NM_014220	ATP7A
8168472	3,33	10,08	0,00145	1,92	3,79	0,01001	NM_000052	MDH1
8042259	3,33	10,06	0,00095	3,46	10,97	0,00054	NM_005917	CCNT1
7962831	3,33	10,05	0,00477	3,76	13,58	0,00088	NM_001240	EIF2B1
7967473	3,33	10,05	0,00137	4,42	21,35	0,00021	NM_001414	SRPRB
8082816	3,33	10,05	0,00917	4,31	19,82	0,00001	NM_021203	EFEMP2
7949503	3,33	10,04	0,00063	-0,36	-1,29	0,16967	NM_016938	SDAD1
8101099	3,33	10,03	0,00209	3,81	14,00	0,00018	NM_018115	EBPL
7971615	3,33	10,03	0,00109	2,17	4,50	0,08372	NM_032565	CISD1
7927649	3,32	10,02	0,00382	3,80	13,98	0,00053	NM_018464	PSENEN
8027996	3,32	10,01	0,00861	4,42	21,41	0,00007	NM_172341	MIA3
7909898	3,32	10,00	0,00559	2,75	6,74	0,00126	NM_198551	LSM12
8150206	3,32	9,98	0,00143	4,20	18,44	0,00029	NM_152344	AZI2
8085954	3,32	9,98	0,00376	3,02	8,10	0,00097	NM_022461	HP1BP3
7913300	3,32	9,98	0,00439	3,90	14,95	0,00139	NM_016287	EPHA7
8128284	3,32	9,97	0,00166	-1,11	-2,16	0,00497	NM_004440	CCND2
7953200	3,32	9,97	0,01046	-1,50	-2,84	0,01491	NM_001759	LASP1
8006820	3,32	9,97	0,00152	3,56	11,83	0,00012	NM_006148	NUP153
8124059	3,32	9,97	0,00216	3,67	12,70	0,00228	NM_005124	NAA35
8156167	3,32	9,97	0,00435	4,33	20,08	0,00039	NM_024635	NSL1
7924172	3,32	9,96	0,00108	3,17	9,00	0,00110	NM_015471	RAD21
8152477	3,32	9,95	0,00370	3,64	12,42	0,00006	NM_006265	EPAS1
8041781	3,32	9,95	0,00100	3,44	10,84	0,00107	NM_001430	KPNA3
7971620	3,31	9,95	0,00275	3,72	13,21	0,00095	NM_002267	MAP4K4
8043945	3,31	9,94	0,00189	3,47	11,11	0,00011	NM_145686	PUM2
8050565	3,31	9,92	0,00013	3,25	9,49	0,01067	NM_015317	IFNAR1
8068266	3,31	9,91	0,00072	2,50	5,64	0,00424	NM_000629	ZBTB34
8157947	3,31	9,90	0,00406	2,80	6,98	0,00591	NM_001099270	EPHA4
8059279	3,31	9,89	0,00333	0,66	1,58	0,07161	NM_004438	CBX5

7963750	3,31	9,89	0,00435	3,84	14,36	0,02535	NM_012117	TNC
8163637	3,31	9,89	0,00137	2,07	4,21	0,01831	NM_002160	LOC400590
8006237	3,31	9,88	0,00051	2,25	4,77	0,00087	ENST00000433145	KIAA0776
8121161	3,30	9,87	0,00292	2,41	5,32	0,00090	NM_015323	PBX1
7906954	3,30	9,87	0,00078	-0,04	-1,03	0,90810	NM_002585	ABCE1
8097647	3,30	9,87	0,00182	4,19	18,22	0,00059	NM_002940	VPS41
8139165	3,30	9,87	0,01698	3,15	8,89	0,00015	NM_014396	PGM2
8094556	3,30	9,85	0,00528	3,89	14,85	0,00158	NM_018290	ZNF254
8027368	3,30	9,84	0,00188	2,70	6,50	0,00325	NM_203282	MEAF6
7914996	3,30	9,82	0,00984	3,56	11,78	0,00090	NM_022756	SNX2
8107613	3,30	9,82	0,00114	2,72	6,61	0,01162	NM_003100	RPL5
7986765	3,29	9,81	0,01522	2,25	4,74	0,01469	NM_000969	PMS2L5
8133531	3,29	9,81	0,00228	4,41	21,26	0,00003	NR_027775	ILK
7938154	3,29	9,80	0,00208	3,17	9,00	0,00010	NM_004517	C19orf42
8035193	3,29	9,80	0,01083	3,42	10,71	0,00417	NM_024104	ZNF518B
8099364	3,29	9,80	0,01286	-1,21	-2,31	0,01314	NM_053042	UBE2Z
8008139	3,29	9,80	0,00334	4,36	20,54	0,00138	NM_023079	---
8042107	3,29	9,79	0,00465	3,88	14,74	0,00064	--- SLFN11	
8014233	3,29	9,79	0,00696	4,42	21,37	0,00054	NM_001104587	CACNA2D1
8140579	3,29	9,78	0,00580	-0,27	-1,21	0,46214	NM_000722	RPS28
8005471	3,29	9,77	0,03400	3,77	13,66	0,00004	NM_001031	DST
8127234	3,29	9,77	0,00026	1,91	3,76	0,04126	NM_015548	G3BP1
8109368	3,29	9,76	0,00590	3,86	14,48	0,00011	NM_005754	SLK
7930276	3,29	9,75	0,00213	2,92	7,55	0,08947	NM_014720	LRP6
7961339	3,29	9,75	0,00075	2,95	7,75	0,01216	NM_002336	KIFAP3
7922268	3,28	9,73	0,00264	3,39	10,45	0,00028	NM_014970	MAP1B
8106098	3,28	9,73	0,00284	2,28	4,84	0,00089	NM_005909	ARHGEF3
8088247	3,28	9,73	0,00026	1,39	2,63	0,00150	NM_001128615	PAPD5
7995479	3,28	9,72	0,00324	3,54	11,60	0,00430	NM_001040284	CCNB2
7983969	3,28	9,72	0,01289	4,10	17,11	0,00095	NM_004701	NHP2L1
8076374	3,28	9,72	0,00130	4,03	16,35	0,00042	NM_005008	HOXA9
8138749	3,28	9,71	0,00441	-0,46	-1,37	0,03931	NM_152739	ALKBH1
7980454	3,28	9,71	0,00286	2,14	4,41	0,00152	NM_006020	KLF3
8094599	3,28	9,70	0,00212	3,07	8,41	0,00006	NM_016531	ATXN2
7966397	3,28	9,69	0,00086	2,99	7,94	0,01590	NM_002973	TULP3
7953135	3,28	9,69	0,00002	3,81	14,02	0,00016	NM_003324	USP34
8052443	3,28	9,68	0,00085	3,48	11,13	0,01234	NM_014709	SGMS2
8096733	3,27	9,68	0,00612	1,68	3,20	0,02453	NM_001136258	ARHGAP21
7932554	3,27	9,68	0,00243	3,30	9,85	0,00077	NM_020824	PARP4
7970602	3,27	9,67	0,00195	3,15	8,91	0,03878	NM_006437	DDR2
7906900	3,27	9,66	0,01567	-0,46	-1,38	0,00191	AY423733 MBIP	
7978666	3,27	9,65	0,00542	3,57	11,91	0,00206	NM_016586	MBD4
8090577	3,27	9,65	0,00075	2,30	4,91	0,00168	NM_003925	TPST1
8133155	3,27	9,65	0,00334	2,77	6,82	0,00072	NM_003596	PHF21A
7947624	3,27	9,64	0,00041	3,56	11,78	0,00406	NM_001101802	LPP
8084742	3,27	9,63	0,00243	2,94	7,66	0,00315	NM_005578	HBS1L
8129706	3,27	9,63	0,00968	3,61	12,19	0,00118	NM_006620	ANGPTL2
8164200	3,27	9,63	0,00311	-1,60	-3,03	0,00822	NM_012098	C22orf28
8075585	3,27	9,62	0,02077	4,23	18,82	0,00065	NM_014306	---
8171846	3,26	9,60	0,01853	1,58	2,99	0,07086	--- DUSP1	
8115831	3,26	9,60	0,00761	4,24	18,90	0,00223	NM_004417	10. Sep
8054467	3,26	9,60	0,00066	4,09	17,01	0,00077	NM_144710	SEC22B
7904695	3,26	9,60	0,00061	1,57	2,98	0,01115	NM_004892	ATF1
7955425	3,26	9,59	0,00469	3,21	9,22	0,00164	NM_005171	NFIA
7901788	3,26	9,59	0,02212	3,66	12,63	0,00415	NM_001134673	SUCLA2
7971541	3,26	9,58	0,02350	3,75	13,42	0,02004	NM_003850	TALDO1
7937465	3,26	9,57	0,00048	1,94	3,83	0,01270	NM_006755	PEBP1
7959070	3,26	9,56	0,00118	3,57	11,84	0,00057	NM_002567	NDRG3
8066051	3,26	9,55	0,00635	4,52	22,89	0,00003	NM_032013	MAP4K5
7978997	3,25	9,54	0,00254	3,67	12,77	0,00092	NM_198794	CSDA
7961230	3,25	9,54	0,00617	3,07	8,42	0,00023	NM_003651	PTPLAD1
7984263	3,25	9,54	0,00761	4,45	21,81	0,00037	NM_016395	DDX1
8040386	3,25	9,53	0,00361	3,75	13,49	0,00000	NM_004939	---
8139889	3,25	9,53	0,00057	2,49	5,63	0,00026	--- MYH10	
8012475	3,25	9,52	0,00973	3,24	9,48	0,00284	NM_005964	NCEH1
8092177	3,25	9,52	0,00483	4,09	17,08	0,00079	NM_001146276	ZFP106
7987892	3,25	9,52	0,00130	3,32	9,97	0,00213	NM_022473	XRCC6
8073457	3,25	9,50	0,00906	1,61	3,06	0,04230	NM_001469	RPF2
8062211	3,25	9,50	0,00166	3,79	13,81	0,00043	NM_032194	RPL6
8022170	3,25	9,49	0,03582	2,42	5,33	0,00026	NM_001024662	SPAG9
8016745	3,25	9,48	0,00238	2,45	5,48	0,03188	NM_001130528	GULP1
8046906	3,25	9,48	0,02431	2,43	5,41	0,02804	NM_016315	EIF4B
7955646	3,24	9,48	0,00440	2,95	7,70	0,00029	NM_001417	PPARG

8077899	3,24	9,47	0,00077	2,69	6,43	0,00164	NM_138712	FAM73A
7902476	3,24	9,47	0,00051	3,14	8,79	0,00006	BX537792 ZNF680	
8139820	3,24	9,47	0,00243	3,16	8,95	0,00415	NM_178558	TBL1XR1
8092201	3,24	9,46	0,00709	4,29	19,61	0,00137	NM_024665	SPIRE1
8022356	3,24	9,46	0,00740	4,09	17,08	0,00104	NM_001128626	COX6A1
7959153	3,24	9,46	0,00222	3,66	12,61	0,00002	NM_004373	PRDX6
7907439	3,24	9,45	0,01766	3,15	8,87	0,00049	NM_004905	RSPRY1
7995994	3,24	9,45	0,00539	3,03	8,18	0,00086	NM_133368	ZNF148
8090237	3,24	9,42	0,00072	2,54	5,81	0,01118	NM_021964	RPAIN
8004133	3,24	9,42	0,00631	3,85	14,40	0,00008	NR_027680	FRY
7968417	3,23	9,41	0,00097	0,38	1,30	0,02157	NM_023037	SAV1
7979033	3,23	9,41	0,00024	3,60	12,16	0,00672	NM_021818	RAF1
8085374	3,23	9,41	0,01468	4,67	25,54	0,00001	NM_002880	YWHAQ
8050215	3,23	9,40	0,00619	2,08	4,22	0,10544	NM_006826	NIP7
7996934	3,23	9,40	0,00007	3,09	8,53	0,00149	NM_016101	NAA50
8089652	3,23	9,39	0,03880	3,94	15,30	0,00018	NM_025146	PRDX4
8166455	3,23	9,39	0,00019	1,60	3,03	0,01027	NM_006406	NBAS
8050367	3,23	9,39	0,00089	2,52	5,75	0,00375	NM_015909	LOC100287552
8136177	3,23	9,39	0,01159	3,27	9,66	0,00275	AF277177 EIF3A	
7936614	3,23	9,39	0,00119	2,85	7,19	0,09185	NM_003750	RPLP0
7966996	3,23	9,39	0,05379	3,53	11,54	0,00899	NM_053275	EIF3K
8028514	3,23	9,38	0,00068	4,05	16,57	0,00004	NM_013234	ADNP
8067011	3,23	9,38	0,00069	3,13	8,76	0,01045	NM_015339	FRG1
8161509	3,23	9,37	0,00017	2,44	5,41	0,00649	NM_004477	DCAF12
8160756	3,23	9,37	0,00527	3,73	13,30	0,00001	NM_015397	CAPZA1
7904036	3,23	9,37	0,00130	1,81	3,50	0,03961	NM_006135	BCL7B
8140061	3,23	9,36	0,00006	4,48	22,26	0,00003	NM_001707	MYO1B
8047127	3,22	9,34	0,00330	3,25	9,51	0,00025	NM_001130158	PTPN13
8096176	3,22	9,34	0,00037	2,14	4,42	0,00176	NM_080683	EIF4H
8138773	3,22	9,34	0,00484	3,23	9,37	0,00279	NM_022170	JMY
8106516	3,22	9,34	0,00051	3,83	14,23	0,00175	NM_152405	RPS17
7990916	3,22	9,33	0,00375	3,24	9,42	0,02522	NM_001021	RPS17
7990965	3,22	9,33	0,00375	3,24	9,42	0,02522	NM_001021	IRF1
8114010	3,22	9,33	0,00258	2,88	7,39	0,00014	NM_002198	IFI16
7906400	3,22	9,33	0,00334	1,07	2,10	0,03454	NM_005531	BRE
8041031	3,22	9,32	0,00013	2,70	6,49	0,01153	NM_199193	PRR13
8036808	3,22	9,32	0,00089	2,64	6,24	0,00421	NM_018457	TM9SF4
8061685	3,22	9,31	0,00104	2,93	7,60	0,00090	NM_014742	SNAP23
7983111	3,22	9,30	0,01418	3,91	15,02	0,00278	NM_003825	RPL6
7966534	3,22	9,30	0,03669	2,32	4,99	0,00039	NM_001024662	THUMPD3
8077513	3,22	9,30	0,00432	3,43	10,78	0,00051	NM_015453	MTFR1
8146649	3,22	9,29	0,00300	4,22	18,62	0,00026	NM_014637	ME1
8127854	3,22	9,29	0,01170	4,40	21,07	0,00010	NM_002395	SFRS11
7902269	3,22	9,29	0,00362	2,82	7,05	0,03006	NM_004768	PTEN
7928959	3,21	9,28	0,00660	1,06	2,08	0,12229	NM_000314	RPS10
8125835	3,21	9,28	0,00291	1,61	3,04	0,08484	NM_001014	HEBP1
7961390	3,21	9,28	0,00860	2,81	7,03	0,00017	NM_015987	FST
8105302	3,21	9,27	0,01007	0,06	1,04	0,88054	NM_006350	ANXA4
8042468	3,21	9,27	0,00272	3,63	12,35	0,00003	NM_001153	MAML2
7951133	3,21	9,27	0,00474	0,72	1,64	0,04439	NM_032427	LRIG3
7964602	3,21	9,27	0,00480	3,07	8,39	0,00110	NM_153377	SLC41A2
7965964	3,21	9,27	0,00642	3,62	12,26	0,00009	NM_032148	IGF2BP3
8131860	3,21	9,26	0,00271	3,26	9,61	0,00439	NM_006547	ZC3H7A
7999496	3,21	9,26	0,00010	3,32	9,98	0,00078	NM_014153	ZNF117
8139832	3,21	9,26	0,00051	1,51	2,84	0,04889	NM_015852	AK3
8159945	3,21	9,26	0,00260	2,87	7,32	0,00003	NM_016282	RAB11FIP2
7936567	3,21	9,25	0,00087	2,72	6,59	0,00373	NM_014904	BFAR
7993341	3,21	9,25	0,00127	3,31	9,90	0,00034	NM_016561	EEF1A1
8138531	3,21	9,25	0,03098	1,25	2,38	0,01140	NM_001402	RPL34
8035829	3,21	9,25	0,01303	3,45	10,90	0,00210	NM_000995	HLA-G
8177725	3,21	9,25	0,04407	3,24	9,47	0,00481	NM_002127	HLA-G
8179034	3,21	9,25	0,04407	3,24	9,47	0,00481	NM_002127	ERC1
7952988	3,21	9,25	0,00198	3,58	11,93	0,00122	NR_027948	PTBP1
8024019	3,21	9,24	0,00598	4,23	18,73	0,00009	NM_002819	EIF2AK4
7982620	3,21	9,24	0,00280	4,16	17,84	0,00008	NM_001013703	STARD5
7990839	3,21	9,24	0,01222	2,65	6,26	0,00485	NM_181900	GTF2A1
7980523	3,21	9,23	0,00504	2,79	6,91	0,00250	NM_015859	VPS4B
8023672	3,21	9,23	0,00074	1,70	3,24	0,00482	NM_004869	TCF4
8023415	3,21	9,23	0,00662	-0,77	-1,71	0,02144	NM_001083962	FAS
7929032	3,21	9,22	0,00008	0,93	1,90	0,05230	NM_000043	ZFR
8111360	3,20	9,21	0,01302	4,21	18,51	0,00040	NM_016107	CCPG1
7989037	3,20	9,20	0,00453	2,46	5,50	0,00069	NM_004748	ROD1
8163402	3,20	9,20	0,00474	3,52	11,46	0,00612	NM_005156	RARS2

8128013	3,20	9,19	0,00060	2,13	4,38	0,00967	NM_020320	COMMD1
8042207	3,20	9,19	0,00085	1,90	3,73	0,01826	NM_152516	RNF114
8063369	3,20	9,19	0,00143	4,72	26,32	0,00009	NM_018683	VCL
7928444	3,20	9,18	0,00364	2,86	7,26	0,00008	NM_014000	MAPK6
7983763	3,20	9,18	0,02936	3,98	15,83	0,00095	NM_002748	WASL
8142671	3,20	9,18	0,00142	3,16	8,93	0,00145	NM_003941	PDGFD
7951351	3,20	9,18	0,00649	1,37	2,58	0,00538	NM_025208	PION
8140478	3,20	9,17	0,00268	3,08	8,48	0,00358	NM_017439	MYL9
8062312	3,20	9,17	0,00005	1,00	2,00	0,01851	NM_006097	LAMA2
8121949	3,20	9,17	0,00225	0,00	1,00	0,98559	NM_000426	PTTG1IP
8070819	3,20	9,16	0,01843	4,07	16,77	0,00100	NM_004339	C6orf211
8122818	3,20	9,16	0,00038	1,77	3,41	0,04527	BC011348 CUL4B	
8174794	3,20	9,16	0,00202	1,90	3,74	0,01725	NM_001079872	C12orf32
7953130	3,20	9,16	0,00124	3,11	8,66	0,00369	NR_027363	C14orf126
7978527	3,19	9,16	0,00029	2,99	7,95	0,00262	NM_080664	PGAM1
7965480	3,19	9,16	0,01018	4,09	16,98	0,00002	NM_002629	KPNA1
8089999	3,19	9,15	0,00236	3,92	15,11	0,00045	NR_026698	C4orf41
8098512	3,19	9,15	0,00250	2,51	5,71	0,00597	NM_021942	PHB2
7960716	3,19	9,15	0,00987	3,51	11,36	0,00154	NM_001144831	SRPX2
8168749	3,19	9,15	0,00920	-0,37	-1,29	0,15793	NM_014467	THAP1
8150559	3,19	9,15	0,00192	2,19	4,57	0,00219	NM_018105	RASA1
8106784	3,19	9,13	0,00092	4,04	16,42	0,00009	NM_002890	RPS5
8031933	3,19	9,13	0,00329	3,57	11,89	0,00016	NM_001009	CDK7
8105862	3,19	9,12	0,00071	1,59	3,01	0,00977	NM_001799	CDK7
8177462	3,19	9,12	0,00071	1,59	3,01	0,00977	NM_001799	TAB3
8171901	3,19	9,11	0,00019	3,27	9,64	0,00115	NM_152787	DCAF10
8155312	3,19	9,11	0,00144	3,85	14,46	0,00040	NM_024345	CCNY
7927008	3,19	9,11	0,00015	2,20	4,59	0,00098	NM_181698	C14orf118
7975863	3,18	9,09	0,01628	4,00	16,01	0,00041	NM_017926	SRPK1
8125941	3,18	9,09	0,00570	3,68	12,80	0,00054	NM_003137	PRKRIR
7950544	3,18	9,08	0,00123	3,25	9,49	0,00263	NM_004705	CDC26
8142878	3,18	9,08	0,00014	1,45	2,73	0,04804	NM_139286	C15orf24
7987172	3,18	9,08	0,00391	3,80	13,90	0,00004	NM_020154	HLA-A
8177732	3,18	9,08	0,00570	3,57	11,87	0,00264	NM_002116	RAB18
7926836	3,18	9,07	0,00423	3,84	14,28	0,00013	NM_021252	HPRT1
8169984	3,18	9,07	0,01033	3,12	8,71	0,00135	NM_000194	C16orf63
7999718	3,18	9,07	0,00041	3,25	9,48	0,00063	NM_144600	RYK
8090840	3,18	9,07	0,00133	3,27	9,64	0,00431	NM_001005861	TMEM183B
8091452	3,18	9,07	0,00602	3,42	10,72	0,00040	NM_001079809	GLT8D1
8087985	3,18	9,07	0,00106	3,62	12,28	0,00098	NM_001010983	ESD
7971513	3,18	9,07	0,00484	3,22	9,33	0,00037	NM_001984	TSPAN3
7990620	3,18	9,06	0,00089	3,87	14,61	0,00124	NM_005724	TSC22D1
7971350	3,18	9,06	0,00329	3,06	8,33	0,00002	NM_183422	CLNS1A
7950597	3,18	9,04	0,00217	4,65	25,08	0,00071	NM_001293	DLD
8135464	3,18	9,03	0,00858	3,09	8,50	0,00362	NM_000108	DHX8
8007505	3,17	9,02	0,00075	2,80	6,96	0,00206	NM_004941	COX6B1
8027932	3,17	9,02	0,00072	3,77	13,66	0,00003	NM_001863	DHX15
8099649	3,17	9,01	0,00405	3,36	10,25	0,00300	NM_001358	TMEM194A
7964347	3,17	9,01	0,00166	4,14	17,65	0,00008	NM_001130963	USP22
8013486	3,17	9,01	0,00127	2,58	5,96	0,00052	NM_015276	FECH
8023466	3,17	9,01	0,00168	2,87	7,30	0,00154	NM_001012515	ALG3
8092457	3,17	9,00	0,00571	4,42	21,35	0,00059	NM_005787	SECISBP2
8156295	3,17	8,99	0,00524	3,10	8,57	0,00309	NM_024077	LMAN2
8116020	3,17	8,99	0,00303	4,28	19,38	0,00001	NM_006816	FAM35B
7927267	3,17	8,99	0,00069	2,42	5,35	0,00222	NR_027632	FOXJ3
7915408	3,17	8,99	0,00190	2,62	6,14	0,02761	NM_014947	TGFBR1
8156826	3,17	8,99	0,00189	1,97	3,92	0,00479	NM_004612	RAB5A
8078214	3,17	8,97	0,00017	2,36	5,14	0,00168	NM_004162	ECD
7934301	3,17	8,97	0,00330	2,96	7,79	0,00049	NM_007265	RNF14
8108847	3,16	8,97	0,01203	3,09	8,54	0,00095	NM_183401	EEF1A1
8158952	3,16	8,97	0,04783	1,30	2,47	0,01300	NM_001402	C14orf101
7974542	3,16	8,97	0,01037	3,53	11,58	0,00128	NM_017799	ADD3
7930380	3,16	8,96	0,00130	2,65	6,28	0,00016	NM_016824	MLLT10
7926565	3,16	8,94	0,00510	3,69	12,87	0,00012	NM_004641	CBFB
7996393	3,16	8,94	0,00069	3,63	12,35	0,00000	NM_001755	RYBP
8088820	3,16	8,94	0,00095	2,44	5,43	0,00002	NM_012234	ODC1
8050240	3,16	8,94	0,00273	4,60	24,30	0,00040	NM_002539	PPP1R10
8124756	3,16	8,93	0,00121	2,49	5,63	0,05988	NM_002714	PPP1R10
8178358	3,16	8,93	0,00121	2,49	5,63	0,05988	NM_002714	PPP1R10
8179664	3,16	8,93	0,00121	2,49	5,63	0,05988	NM_002714	GSR
8150112	3,16	8,91	0,00630	4,88	29,44	0,00004	NM_000637	LSM12
8015908	3,15	8,91	0,00226	4,17	17,98	0,00035	NM_152344	EXOSC2
8158714	3,15	8,89	0,05198	5,18	36,31	0,00030	NM_014285	PIGB

7983811	3,15	8,89	0,00753	5,35	40,92	0,00002	NM_004855	BTF3L4
7901418	3,15	8,88	0,00911	4,38	20,88	0,00001	NM_152265	TPP1
7946228	3,15	8,88	0,00113	2,74	6,70	0,00266	NM_000391	C2orf28
8040831	3,15	8,88	0,00064	2,46	5,51	0,00105	NM_080592	ATP6V1F
8136039	3,15	8,87	0,00092	2,97	7,81	0,00175	NM_004231	YES1
8021984	3,15	8,87	0,01155	1,87	3,65	0,00204	NM_005433	C16orf61
8002987	3,15	8,86	0,04885	5,13	35,04	0,00007	NM_020188	ADH1B
8101881	3,15	8,86	0,00909	-0,95	-1,94	0,01556	NM_000668	TWF2
8087860	3,15	8,85	0,00614	3,60	12,11	0,00013	NM_007284	NUCKS1
7923798	3,14	8,84	0,00081	2,29	4,87	0,01082	NM_022731	EXT1
8152491	3,14	8,83	0,00712	3,44	10,88	0,00019	NM_000127	MAN1A2
7904340	3,14	8,83	0,00240	2,96	7,76	0,00031	NM_006699	AHSA1
7975976	3,14	8,82	0,00071	2,55	5,84	0,00035	NM_012111	PPP2CB
8150126	3,14	8,82	0,00154	3,53	11,51	0,00162	NM_001009552	UQCRC2
7993872	3,14	8,82	0,00226	3,65	12,55	0,00441	NM_003366	TBPL1
8122182	3,14	8,82	0,01067	2,27	4,82	0,01977	NM_004865	REST
8095262	3,14	8,81	0,00009	2,06	4,17	0,00912	NM_005612	CHD9
7995583	3,14	8,81	0,00198	2,43	5,40	0,00342	NM_025134	MEIS1
8042356	3,14	8,81	0,00263	-0,63	-1,54	0,21696	NM_002398	ACVR2A
8045587	3,14	8,81	0,03053	2,18	4,53	0,00061	NM_001616	PLDN
7983502	3,14	8,80	0,00141	3,17	8,97	0,00268	NM_012388	GOLGA4
8078569	3,14	8,80	0,00111	1,99	3,98	0,02318	NM_002078	C3orf1
8081867	3,14	8,80	0,03739	5,56	47,04	0,00002	NM_016589	CALCOCO2
8008113	3,14	8,79	0,00027	2,92	7,59	0,00007	NM_005831	ALS2CR4
8058258	3,14	8,79	0,00543	2,90	7,47	0,00790	NM_152388	PLAUR
8037374	3,14	8,79	0,00556	4,26	19,14	0,00012	NM_002659	CCDC91
7954613	3,13	8,78	0,00104	2,04	4,12	0,00556	NM_018318	ZDHHC2
8144758	3,13	8,77	0,00577	3,01	8,03	0,00043	NM_016353	KIAA2018
8089647	3,13	8,76	0,00370	2,64	6,25	0,00301	NM_001009899	PPP2R1A
8030881	3,13	8,76	0,00116	3,16	8,95	0,00026	NM_014225	SNRPE
8160033	3,13	8,76	0,00012	4,21	18,55	0,00017	NM_003094	USP30
7958439	3,13	8,75	0,00462	3,37	10,33	0,00235	NM_032663	FAM175B
7931216	3,13	8,75	0,00115	2,96	7,77	0,00145	NM_032182	BTRC
7929958	3,13	8,75	0,00042	2,57	5,95	0,00693	NM_033637	IGF2
7937772	3,13	8,73	0,00359	-0,54	-1,45	0,12548	NM_000612	SFXN1
8110090	3,13	8,73	0,00072	3,46	10,98	0,00002	NM_022754	OCRL
8169811	3,12	8,72	0,00209	4,01	16,06	0,00002	NM_000276	FAM3C
8142540	3,12	8,72	0,00241	3,31	9,90	0,00002	NM_014888	SERPINB1
8123598	3,12	8,72	0,00593	4,76	27,16	0,00000	NM_030666	LOXL2
8149774	3,12	8,71	0,00192	3,77	13,60	0,00111	NM_002318	CEPT1
7903908	3,12	8,71	0,00246	2,84	7,14	0,00141	NM_006090	DOCK11
8169541	3,12	8,71	0,00052	0,97	1,96	0,12552	NM_144658	LRP1
7956301	3,12	8,71	0,00205	2,81	7,03	0,00072	NM_002332	PPP2R1B
7951614	3,12	8,71	0,00692	2,83	7,10	0,00314	NM_002716	DNAJC1
7932512	3,12	8,70	0,00019	1,76	3,39	0,00910	NM_022365	SETX
8164701	3,12	8,70	0,00118	2,90	7,47	0,01936	NM_015046	ISOC1
8107814	3,12	8,70	0,01143	1,86	3,63	0,00304	NM_016048	TBCK
8102171	3,12	8,69	0,00596	3,68	12,82	0,00083	NM_001163435	MEST
8136248	3,12	8,69	0,00162	2,38	5,21	0,00020	NM_002402	---
8000480	3,12	8,69	0,00069	0,50	1,42	0,29712	--- NDUFAF4	
8128322	3,12	8,68	0,00033	2,64	6,23	0,01640	NM_014165	ZNF761
8030993	3,12	8,68	0,00039	1,78	3,44	0,01361	NM_001008401	MAP3K7
8128260	3,12	8,67	0,00268	2,43	5,39	0,01521	NM_145331	EIF5AL1
7928600	3,12	8,67	0,00290	3,72	13,17	0,00038	NM_001099692	---
7916016	3,12	8,66	0,00190	0,08	1,06	0,89571	--- LRRC40	
7916910	3,11	8,65	0,00091	2,78	6,86	0,00306	NM_017768	CHAC1
7982868	3,11	8,65	0,00027	3,72	13,20	0,00174	NM_024111	PRPF8
8011141	3,11	8,65	0,00057	3,88	14,69	0,00069	NM_006445	PDGFC
8103399	3,11	8,64	0,00167	0,28	1,21	0,27827	NM_016205	OPA1
8084844	3,11	8,64	0,00113	2,79	6,94	0,01636	NM_130837	NGDN
7973414	3,11	8,64	0,00001	3,65	12,53	0,00014	NM_015514	RPS27
7960359	3,11	8,63	0,00829	2,92	7,55	0,00326	ENST00000447487	POMP
7968297	3,11	8,63	0,00026	1,39	2,63	0,05513	NM_015932	PDCD6IP
8078479	3,11	8,61	0,00683	2,08	4,22	0,12615	NM_013374	AP3B1
8112772	3,11	8,61	0,00039	3,28	9,68	0,00275	NM_003664	NLK
8005814	3,11	8,61	0,01346	5,30	39,43	0,00087	NM_016231	PCTP
8008598	3,10	8,60	0,00853	3,50	11,33	0,00035	NM_001102402	SNRNP27
8042495	3,10	8,59	0,01509	3,24	9,47	0,00096	NM_006857	ZNF724P
8035838	3,10	8,57	0,01078	2,46	5,51	0,01402	AK301230 CLOCK	
8100428	3,10	8,57	0,01381	3,56	11,82	0,00011	NM_004898	MAPKAPK5
7958800	3,10	8,57	0,00046	3,78	13,70	0,00024	NM_003668	FMOD
7923578	3,10	8,56	0,01037	-1,53	-2,89	0,00383	NM_002023	TFAM
7927669	3,10	8,56	0,00651	2,56	5,92	0,00252	NM_003201	SNX7

7903203	3,10	8,55	0,00012	1,43	2,70	0,00562	NM_015976	CCNDBP1
7983173	3,09	8,54	0,00823	2,89	7,43	0,00052	NM_012142	RIT1
7920839	3,09	8,54	0,00328	3,02	8,14	0,00028	NM_006912	P4HA1
7934278	3,09	8,54	0,00142	2,30	4,91	0,00188	NM_000917	FHL1
8170119	3,09	8,53	0,00324	2,78	6,87	0,00072	NM_001159702	CDS2
8060772	3,09	8,53	0,00284	3,15	8,88	0,00011	NM_003818	POM121
8133326	3,09	8,52	0,06560	5,01	32,14	0,00028	NM_172020	APOOL
8168580	3,09	8,52	0,00148	3,10	8,56	0,00217	NM_198450	C17orf63
8013776	3,09	8,51	0,01218	3,10	8,57	0,00015	NM_018182	PTGR1
8163328	3,09	8,49	0,00405	3,55	11,69	0,00065	NM_001146108	SNORD4B
8005957	3,09	8,49	0,02617	0,42	1,33	0,73747	NR_000009	TMEM165
8095148	3,09	8,49	0,00490	3,20	9,22	0,00001	NM_018475	HLA-DPA1
8125556	3,09	8,49	0,00125	1,37	2,59	0,00967	NM_033554	HLA-DPA1
8178891	3,09	8,49	0,00125	1,37	2,59	0,00967	NM_033554	NR2C2
8078091	3,09	8,49	0,00262	2,81	7,03	0,01464	NM_003298	GPX7
7901460	3,08	8,48	0,00939	2,32	5,00	0,00034	NM_015696	ZNF273
8133062	3,08	8,48	0,00070	3,08	8,45	0,00252	NM_021148	LAMP1
7970287	3,08	8,48	0,00364	1,69	3,22	0,00213	NM_005561	TOMM70A
8089128	3,08	8,48	0,00054	2,45	5,47	0,00041	NM_014820	TMEM87A
7987869	3,08	8,47	0,00715	3,62	12,29	0,00102	NM_015497	KRTAP1-5
8015179	3,08	8,47	0,00291	-1,06	-2,08	0,02614	NM_031957	KRTAP1-5
8019588	3,08	8,47	0,00291	-1,06	-2,08	0,02614	NM_031957	AGPS
8046604	3,08	8,46	0,00103	3,41	10,62	0,00106	NM_003659	HEATR1
7925364	3,08	8,46	0,00072	3,29	9,81	0,00464	NM_018072	HERC4
7933947	3,08	8,46	0,01842	3,75	13,43	0,00008	NM_022079	TSPAN31
7956613	3,08	8,45	0,01258	1,36	2,57	0,03109	NM_005981	CTSL1
8156228	3,08	8,45	0,00064	3,53	11,58	0,00383	NM_001912	C14orf1
7980309	3,08	8,45	0,00504	4,62	24,64	0,00005	NM_007176	USP3
7984132	3,08	8,44	0,00947	3,79	13,82	0,00043	NM_006537	STK17A
8132503	3,08	8,44	0,00069	3,12	8,67	0,00009	NM_004760	FNIP1
8113914	3,08	8,44	0,00767	3,24	9,48	0,00045	NM_133372	08. Mrz
7933228	3,08	8,44	0,00215	2,93	7,60	0,00006	NM_001002265	RPL23A
8091806	3,08	8,43	0,03617	0,86	1,81	0,51905	NM_000984	LTBP1
8041383	3,08	8,43	0,00032	1,69	3,23	0,00104	NM_206943	PHACTR2
8122426	3,08	8,43	0,00212	1,66	3,16	0,02019	NM_014721	SNX3
8128683	3,08	8,43	0,00108	2,52	5,75	0,00293	NM_003795	MDH2
8133690	3,07	8,42	0,00355	3,57	11,86	0,00107	NM_005918	USP7
7999326	3,07	8,42	0,00061	3,50	11,30	0,02157	NM_003470	PDSS1
7926807	3,07	8,42	0,00288	4,04	16,42	0,00057	NM_014317	DUSP10
7924450	3,07	8,42	0,03670	1,96	3,90	0,00082	NM_007207	RAB3GAP1
8045398	3,07	8,42	0,01264	2,39	5,24	0,00023	NM_001172435	C1orf85
7920971	3,07	8,42	0,00925	5,03	32,73	0,00006	NM_144580	CYB5B
7996947	3,07	8,41	0,00327	3,88	14,68	0,00095	NM_030579	PHTF2
8133818	3,07	8,41	0,00638	1,96	3,89	0,00025	NM_001127358	TTLL4
8048411	3,07	8,40	0,00101	3,48	11,19	0,00055	NM_014640	ANKRD17
8100902	3,07	8,40	0,00078	3,62	12,27	0,00008	NM_032217	---
7960131	3,07	8,40	0,02174	1,76	3,39	0,02477	--- ATXN3	
7980940	3,07	8,40	0,00012	2,51	5,68	0,00049	NR_028459	MRPS23
8016870	3,07	8,39	0,00559	3,58	11,99	0,00019	NM_016070	SMAD4
8021228	3,07	8,39	0,02017	1,01	2,01	0,03735	NM_005359	SYNRG
8014551	3,07	8,39	0,01502	3,90	14,91	0,00003	NM_007247	CCT3
7920984	3,07	8,38	0,00252	3,73	13,30	0,00156	NM_005998	PCBD1
7934178	3,07	8,37	0,01153	3,91	15,04	0,00086	NM_000281	FRS2
7957043	3,07	8,37	0,00120	2,94	7,66	0,00154	NM_006654	RPL11
7898875	3,06	8,36	0,01449	1,39	2,61	0,31723	NM_000975	PLEKHB2
8045247	3,06	8,35	0,01086	3,06	8,34	0,00251	NM_017958	RAG1AP1
7905938	3,06	8,33	0,01613	5,72	52,70	0,00006	NM_018845	SNX12
8173430	3,06	8,33	0,00514	2,50	5,64	0,00664	NM_013346	C1orf43
7920492	3,06	8,33	0,01268	4,90	29,82	0,00017	NM_001098616	EXOSC1
7935462	3,06	8,32	0,00906	3,34	10,16	0,00044	NM_016046	MT2A
8095376	3,06	8,31	0,01456	2,19	4,57	0,00799	NM_005953	ZZZ3
7917103	3,05	8,31	0,00243	2,58	5,96	0,00654	NM_015534	TMEM39A
8089759	3,05	8,30	0,00319	4,15	17,74	0,00007	NM_018266	PGAM1
7958152	3,05	8,30	0,01199	3,76	13,57	0,00023	NM_002629	XPO4
7970473	3,05	8,29	0,00150	3,29	9,77	0,00052	NM_022459	GRINA
8148694	3,05	8,28	0,00179	2,45	5,48	0,00044	NM_000837	ROCK2
8050302	3,05	8,28	0,00024	3,21	9,28	0,00648	NM_004850	LYSMD3
8113064	3,05	8,28	0,00664	1,82	3,53	0,03160	NM_198273	M6PR
7960933	3,05	8,27	0,02315	4,98	31,57	0,00045	NM_002355	GAB1
8097586	3,05	8,27	0,00451	2,89	7,40	0,00052	NM_207123	ATXN7L1
8142096	3,05	8,27	0,01776	3,22	9,32	0,00252	NM_020725	UBE2E2
8078252	3,05	8,26	0,00774	4,00	16,02	0,00009	NM_152653	03. Mrz
8113790	3,05	8,26	0,01290	2,36	5,14	0,00800	NM_178450	KIAA1107

7902977	3,04	8,25	0,00159	1,49	2,80	0,05165	NM_015237	ACTR3
8044686	3,04	8,25	0,00555	3,11	8,64	0,00074	NM_005721	CDKN1B
7954029	3,04	8,24	0,00057	3,06	8,36	0,00200	NM_004064	KIT
8095110	3,04	8,24	0,01213	-0,64	-1,56	0,09967	NM_000222	ZNF280C
8175076	3,04	8,24	0,00316	3,90	14,92	0,00071	NM_017666	SLC4A4
8095585	3,04	8,24	0,00176	2,42	5,36	0,00552	NM_001098484	DNAJC11
7912112	3,04	8,23	0,00520	2,47	5,56	0,00609	NM_018198	PSMC6
8150870	3,04	8,23	0,00717	3,31	9,89	0,00450	NM_002806	---
8121212	3,04	8,23	0,00049	2,39	5,25	0,01286	--- BBX	
8081465	3,04	8,22	0,00530	2,94	7,66	0,00452	NM_001142568	PMS2L2
8140280	3,04	8,22	0,00270	4,05	16,58	0,00007	NR_003614	SLC44A2
8025672	3,04	8,22	0,00022	3,66	12,64	0,00028	NM_020428	ARID2
7955019	3,04	8,22	0,00067	3,56	11,76	0,00348	NM_152641	PRICKLE1
7962375	3,04	8,21	0,00035	2,75	6,73	0,00062	NM_153026	PGAM4
8173729	3,04	8,21	0,01138	3,32	9,98	0,00021	NM_001029891	PGAM4
8176282	3,04	8,21	0,01138	3,32	9,98	0,00021	NM_001029891	GPR107
8158597	3,04	8,21	0,00210	3,89	14,88	0,00006	NM_001136557	GMFB
7979260	3,04	8,20	0,00259	1,97	3,93	0,00531	NM_004124	RGPD6
8044304	3,04	8,20	0,00091	2,05	4,15	0,03813	NM_001123363	07. Sep
8132292	3,03	8,19	0,00010	1,43	2,70	0,02380	NM_001788	SPG21
7989696	3,03	8,19	0,00015	3,76	13,53	0,00001	NM_001127889	CNN1
8025918	3,03	8,19	0,00442	-1,29	-2,45	0,02055	NM_001299	NSDHL
8170590	3,03	8,18	0,02002	3,27	9,65	0,00020	NM_015922	RGPD3
8054414	3,03	8,18	0,00351	1,84	3,58	0,04623	NM_001144013	CNOT6
8110589	3,03	8,17	0,00175	2,88	7,37	0,00237	NM_015455	MARS
7956443	3,03	8,16	0,00565	3,57	11,84	0,00001	NM_004990	ZNF528
8030931	3,03	8,16	0,00130	1,96	3,88	0,01697	NM_032423	MEIS2
7987385	3,03	8,15	0,00088	1,73	3,33	0,00073	NM_172316	ACP1
8039961	3,03	8,15	0,02002	3,72	13,18	0,00436	NR_024080	C4orf46
7991777	3,03	8,14	0,00369	2,72	6,58	0,00205	NM_001008393	WSB2
7966829	3,03	8,14	0,01193	3,78	13,70	0,00006	NM_018639	PFN2
8091446	3,03	8,14	0,01336	5,34	40,62	0,00019	NM_053024	ATP5H
8018288	3,02	8,13	0,00113	2,87	7,29	0,00075	NM_006356	CYR61
7902687	3,02	8,12	0,00139	2,16	4,46	0,02400	NM_001554	HLTF
8091354	3,02	8,12	0,00426	3,56	11,75	0,02072	NM_003071	FAM10A5
7938756	3,02	8,12	0,01090	1,43	2,69	0,07702	AF512499 RPL36AL	
7978833	3,02	8,11	0,00446	3,28	9,71	0,00041	NM_001001	GBA
7920697	3,02	8,11	0,04441	3,03	8,18	0,00270	NM_000157	ZNF805
8031712	3,02	8,11	0,00650	2,54	5,80	0,00299	NM_001145078	B3GNT5
8084206	3,02	8,11	0,00465	3,27	9,66	0,00114	NM_032047	STK39
8056545	3,02	8,10	0,00563	2,80	6,96	0,00100	NM_013233	ISCA1
8112331	3,02	8,09	0,05837	4,02	16,23	0,00054	NM_030940	HLA-C
8178489	3,02	8,09	0,00297	3,70	13,00	0,00195	NM_002117	IGFBP3
8139488	3,02	8,09	0,00007	1,06	2,08	0,16805	NM_001013398	CREG1
7922051	3,01	8,08	0,00020	3,77	13,61	0,00015	NM_003851	H3F3A
8102860	3,01	8,08	0,05496	3,38	10,41	0,00080	NM_002107	LRPPRC
8051882	3,01	8,07	0,00597	3,44	10,82	0,01774	NM_133259	BTBD3
8060988	3,01	8,06	0,00740	1,16	2,23	0,00991	NM_014962	HNRNPA1
7995574	3,01	8,06	0,05108	1,63	3,10	0,00243	NM_002136	HIST1H2BK
8068898	3,01	8,05	0,07875	3,67	12,74	0,00005	NM_080593	TMEM161B
8113023	3,01	8,04	0,02232	4,21	18,55	0,00365	NM_153354	TOB1
8016739	3,01	8,04	0,00049	2,03	4,09	0,01858	NM_005749	DNAJC3
7969651	3,01	8,03	0,00186	3,52	11,48	0,00008	NM_006260	PLD3
8028791	3,00	8,02	0,04111	5,34	40,56	0,00014	NM_012268	CD99
8165794	3,00	8,02	0,00007	1,01	2,01	0,02360	NM_002414	CD99
8176360	3,00	8,02	0,00007	1,01	2,01	0,02360	NM_002414	SFRS1
8016898	3,00	8,01	0,00632	3,74	13,34	0,00019	NM_001078166	TBCEL
7944623	3,00	8,01	0,00021	3,03	8,14	0,00235	NM_152715	UGCG
8157216	3,00	8,01	0,01203	4,72	26,44	0,00014	NM_003358	APOO
8171823	3,00	8,01	0,01836	3,82	14,09	0,00070	NM_024122	CRYBG3
8081171	3,00	8,01	0,00143	1,96	3,89	0,02092	NM_153605	UBE2H
8142886	3,00	8,00	0,00499	3,35	10,22	0,00124	NM_003344	SNCA
8101762	3,00	8,00	0,00871	-0,59	-1,50	0,08097	NM_000345	MRPS35
7954591	3,00	8,00	0,00113	2,99	7,96	0,00107	NM_021821	SMARCA5
8097600	3,00	8,00	0,00122	1,73	3,32	0,07579	NM_003601	DDB1
7948534	3,00	8,00	0,01572	3,67	12,73	0,00111	NM_001923	ANXA2P2 // ANXA2P2 // ANXA2P2
8154836	3,00	8,00	0,00686	2,13	4,38	0,00895	NR_003573 // NR_003573 // NR_003573	SNORD13
8086752	3,00	7,99	0,05439	-2,25	-4,74	0,33652	NR_003041	ATP5I
8098762	3,00	7,98	0,00422	2,31	4,97	0,00039	NM_007100	IFIT1
7929065	3,00	7,97	0,00031	0,16	1,12	0,51008	NM_001548	SYT11
7906061	2,99	7,97	0,01327	-0,31	-1,24	0,11773	NM_152280	LETMD1

7955450	2,99	7,97	0,00719	4,84	28,60	0,00041	NM_015416	EMP3
8030007	2,99	7,96	0,00099	2,29	4,88	0,00061	NM_001425	KIAA1199
7985317	2,99	7,96	0,00608	-0,34	-1,26	0,02388	NM_018689	DEGS1
7910001	2,99	7,96	0,00770	3,00	8,01	0,00012	NM_003676	OXR1
8147848	2,99	7,96	0,00988	3,25	9,54	0,00030	NM_181354	RPS25
7939068	2,99	7,95	0,00009	2,59	6,04	0,00004	NM_001028	FAM135A
8120552	2,99	7,95	0,00706	4,00	15,99	0,00013	NM_001105531	AMZ2P1
8017704	2,99	7,95	0,01241	2,79	6,94	0,03321	NR_026903	CASC4
7983321	2,99	7,95	0,00048	1,49	2,80	0,05996	NM_138423	C18orf10
8022941	2,99	7,93	0,00177	2,44	5,41	0,02554	NM_015476	FILIP1L
8089112	2,99	7,93	0,00039	-0,71	-1,64	0,02373	NM_182909	ZNF506
8035773	2,99	7,92	0,00344	2,49	5,60	0,00126	NM_001099269	HIGD1A
7955719	2,99	7,92	0,00842	1,50	2,83	0,01583	NM_001099668	FAF1
7915995	2,98	7,92	0,00359	4,47	22,21	0,00111	NM_007051	SLC6A15
7965206	2,98	7,91	0,00349	-0,99	-1,99	0,00064	NM_182767	DPAGT1
7952185	2,98	7,91	0,00059	4,31	19,84	0,00106	NM_001382	TFDP2
8091103	2,98	7,91	0,00237	2,50	5,66	0,00144	NM_006286	TAF2
8152553	2,98	7,91	0,00839	3,52	11,45	0,00186	NM_003184	CCT5
8104449	2,98	7,91	0,00316	4,04	16,43	0,00006	NM_012073	NOTCH2NL
7904702	2,98	7,90	0,01658	2,42	5,37	0,00530	NM_203458	SLC30A6
8041257	2,98	7,90	0,02798	4,54	23,27	0,00007	NM_017964	KLHL9
8160405	2,98	7,90	0,00360	2,90	7,45	0,00024	NM_018847	C10orf57
7928671	2,98	7,89	0,03228	6,80	111,53	0,00003	NM_025125	ANAPC7
7966301	2,98	7,89	0,00070	3,85	14,40	0,00020	NM_016238	GBP2
7917532	2,98	7,89	0,00578	0,11	1,08	0,57247	NM_004120	PLXDC2
7926545	2,98	7,89	0,01376	-0,72	-1,65	0,02702	NM_032812	SLC30A9
8094848	2,98	7,88	0,00181	3,61	12,25	0,00061	NM_006345	DTX3L
8082075	2,98	7,87	0,00686	2,37	5,17	0,00304	NM_138287	RHOC
7918593	2,98	7,87	0,00074	2,65	6,26	0,00368	NM_175744	AP3S1
8107421	2,98	7,86	0,00001	2,61	6,11	0,01209	NM_001284	HDHD2
8023165	2,98	7,86	0,01946	3,54	11,63	0,00389	NM_032124	CCT2
7957052	2,98	7,86	0,00540	3,22	9,34	0,00011	NM_006431	IL10RB
8068254	2,97	7,86	0,02789	3,56	11,75	0,00061	NM_000628	ARHGAP42
7943349	2,97	7,85	0,00074	2,69	6,45	0,00243	NM_152432	CCNA2
8102643	2,97	7,85	0,00150	2,93	7,61	0,00007	NM_001237	MRPL42
7957540	2,97	7,84	0,00873	4,30	19,76	0,00306	NM_014050	IPO8
7962085	2,97	7,84	0,00197	3,46	11,03	0,00042	NM_006390	AEBP2
7954279	2,97	7,84	0,00188	2,46	5,52	0,00212	NM_153207	TNFRSF10D
8149749	2,97	7,83	0,01759	4,06	16,74	0,00009	NM_003840	SDCCAG1
7978866	2,97	7,83	0,00185	2,64	6,25	0,00484	NM_004713	BNC2
8160260	2,97	7,83	0,00140	-1,59	-3,00	0,00577	NM_017637	TMEM106A
8019622	2,97	7,83	0,01019	1,94	3,83	0,00299	NM_145041	DDX42
8009205	2,97	7,81	0,00051	2,34	5,07	0,02135	NM_007372	NUP43
8130129	2,96	7,81	0,00457	3,43	10,79	0,00034	NM_198887	MKI67IP
8054930	2,96	7,80	0,03668	3,58	11,92	0,00058	NM_032390	GOT1
7935627	2,96	7,80	0,02354	4,05	16,60	0,00025	NM_002079	TNFRSF1A
7960518	2,96	7,80	0,01297	2,92	7,56	0,00610	NM_001065	PLAGL1
8129985	2,96	7,78	0,00605	3,04	8,21	0,00026	NM_006718	SDHAF2
7940486	2,96	7,78	0,00042	3,18	9,03	0,00106	NM_017841	MRPS21
7905163	2,96	7,78	0,04127	4,20	18,37	0,00050	NM_018997	GCNT1
8155930	2,96	7,78	0,00160	1,24	2,37	0,03319	NM_001490	SEL1L
7980547	2,96	7,78	0,00252	2,88	7,37	0,00127	NM_005065	SMARCA1
8174985	2,96	7,77	0,00748	2,89	7,43	0,00139	NM_003069	CLU
8149927	2,96	7,77	0,00500	1,41	2,65	0,00023	NM_001831	PERP
8129880	2,96	7,76	0,01242	2,32	5,01	0,00037	NM_022121	UBR3
8046213	2,96	7,75	0,01451	2,22	4,65	0,11420	NM_172070	CDK14
8134098	2,95	7,75	0,00279	2,42	5,36	0,00144	NM_012395	RPAP3
7962590	2,95	7,75	0,00106	2,40	5,29	0,02970	NM_024604	POLR2L
7945573	2,95	7,75	0,00553	3,40	10,53	0,00287	NM_021128	FCHSD2
7950248	2,95	7,74	0,00247	3,90	14,93	0,00023	NM_014824	RAB1B
7941583	2,95	7,73	0,01573	5,05	33,19	0,00011	NM_030981	PDIA4
8143684	2,95	7,73	0,00109	2,82	7,04	0,00188	NM_004911	TIPARP
8083569	2,95	7,73	0,01520	4,09	17,00	0,00745	NM_015508	POP4
8027390	2,94	7,70	0,00904	3,54	11,64	0,00021	NM_006627	ZFAND3
8119227	2,94	7,68	0,00021	3,29	9,81	0,00261	NM_021943	RCHY1
8101013	2,94	7,68	0,00414	2,93	7,60	0,00027	NM_015436	PAWR
7965112	2,94	7,67	0,00045	3,33	10,05	0,02884	NM_002583	BNIP2
7989323	2,94	7,67	0,00791	3,43	10,81	0,00003	NM_004330	TSPAN8
7964927	2,94	7,67	0,02421	-0,52	-1,43	0,09915	NM_004616	PHLDB2
8081590	2,94	7,65	0,00150	3,09	8,53	0,00130	NM_001134439	CHORDC1
7950983	2,94	7,65	0,00215	2,67	6,35	0,00039	NM_012124	PGAM1
7929624	2,94	7,65	0,01407	3,68	12,79	0,00026	NM_002629	ARL8B
8077450	2,93	7,63	0,04162	3,21	9,25	0,00122	NM_018184	RBMX

8175420	2,93	7,63	0,01005	2,40	5,27	0,00090	NM_002139	ELTD1
7917182	2,93	7,62	0,00963	-0,76	-1,70	0,00643	NM_022159	C11orf17
7938301	2,93	7,62	0,02652	4,51	22,71	0,00007	NM_020642	B4GALT4
8089743	2,93	7,62	0,00320	1,55	2,93	0,00369	NM_212543	DDX17
8076077	2,93	7,62	0,00200	3,95	15,48	0,00333	NM_006386	MLLT11
7905329	2,93	7,61	0,00629	2,32	5,01	0,00022	NM_006818	GOLIM4
8091954	2,93	7,61	0,00209	3,07	8,40	0,00057	NM_014498	RPS13
7946812	2,93	7,61	0,00270	2,92	7,58	0,01229	NM_001017	ENO1
7912198	2,93	7,61	0,00239	4,33	20,09	0,00033	NM_001428	MLL3
8143988	2,93	7,60	0,00207	2,90	7,48	0,00161	NM_170606	KIAA1279
7927955	2,93	7,60	0,00402	2,69	6,46	0,00056	NM_015634	CD109
8120719	2,93	7,60	0,00550	3,14	8,80	0,01189	NM_133493	AMFR
8001477	2,92	7,59	0,00761	3,57	11,91	0,00036	NM_001144	OXA1L
7973314	2,92	7,59	0,00924	2,67	6,36	0,00000	NM_005015	UBR2
8119529	2,92	7,59	0,00289	3,41	10,59	0,00031	NM_015255	SNX5
8065165	2,92	7,58	0,00110	2,50	5,65	0,00209	NM_152227	APPL1
8080645	2,92	7,58	0,00045	3,21	9,28	0,00210	NM_012096	PMS2L2
8133300	2,92	7,57	0,00581	4,21	18,49	0,00004	NR_003614	USMG5
7936091	2,92	7,56	0,00175	4,50	22,70	0,00205	NM_032747	SMYD2
7909689	2,92	7,55	0,00162	3,67	12,75	0,00022	NM_020197	RALGPS2
7907657	2,92	7,55	0,00618	2,80	6,98	0,00070	NM_152663	ACADM
7902367	2,92	7,54	0,00691	3,73	13,31	0,00116	NM_000016	PMAIP1
8021470	2,91	7,53	0,00979	4,07	16,81	0,00006	NM_021127	CPSF2
7976243	2,91	7,53	0,00629	3,70	13,03	0,00002	NM_017437	EZH1
8015685	2,91	7,53	0,00132	3,25	9,53	0,00014	NM_001991	THOC2
8174893	2,91	7,53	0,00623	3,31	9,93	0,00127	NM_001081550	SNX1
7984155	2,91	7,53	0,00214	2,91	7,52	0,00053	NM_003099	PTGER2
7974366	2,91	7,52	0,00100	0,39	1,31	0,07994	NM_000956	---
8111099	2,91	7,51	0,00336	1,98	3,94	0,01346	--- NDUFC2	
7950644	2,91	7,51	0,00339	3,85	14,38	0,00149	NM_004549	C12orf75
7958253	2,91	7,51	0,00035	2,95	7,72	0,00055	NM_001145199	CNOT6L
7989128	2,91	7,50	0,00568	2,80	6,98	0,00298	NM_144571	CLPX
7989735	2,91	7,50	0,00430	2,97	7,86	0,00008	NM_006660	WTAP
8123129	2,91	7,49	0,03714	1,93	3,82	0,02075	NM_004906	GNB4
8092251	2,91	7,49	0,01252	3,09	8,50	0,00028	NM_021629	SNX25
8098581	2,91	7,49	0,00278	1,61	3,06	0,00601	NM_031953	DFNA5
8138602	2,90	7,49	0,00402	2,58	5,96	0,00040	NM_004403	MET
8135601	2,90	7,48	0,00844	2,91	7,51	0,01907	NM_001127500	OPTN
7926239	2,90	7,48	0,00242	1,44	2,71	0,00653	NM_001008211	C1orf174
7911897	2,90	7,47	0,00279	3,96	15,61	0,00050	NM_207356	DNAJB9
8135480	2,90	7,47	0,00022	3,06	8,37	0,00129	NM_012328	---
8097655	2,90	7,47	0,00254	2,90	7,44	0,00909	--- XPO7	
8144953	2,90	7,47	0,00342	4,17	17,99	0,00009	NM_001100161	MED6
7979931	2,90	7,46	0,00749	3,62	12,27	0,00389	NM_005466	TIMM8B
7951679	2,90	7,46	0,00113	2,65	6,29	0,00125	NR_028383	PPP2R5C
7976863	2,90	7,46	0,00199	3,83	14,22	0,00000	NM_001161725	ACVR1
8056005	2,90	7,46	0,00778	3,20	9,17	0,00520	NM_001105	DCTN6
8145660	2,90	7,45	0,00402	2,46	5,50	0,04367	NM_006571	BCLAF1
8129773	2,90	7,45	0,00318	2,90	7,44	0,00163	NM_014739	STK4
8062908	2,90	7,45	0,02183	2,64	6,21	0,00038	NM_006282	FRYL
8100251	2,90	7,44	0,00615	2,93	7,64	0,00070	NM_015030	ZDHHC3
8086505	2,89	7,43	0,00816	2,87	7,30	0,00005	NM_016598	RNF25
8058985	2,89	7,43	0,01108	3,67	12,71	0,00011	NM_022453	ZNF430
8027279	2,89	7,43	0,01173	2,66	6,31	0,00456	NM_025189	NDUFA9
7953243	2,89	7,42	0,00324	4,12	17,43	0,00030	NM_005002	TMEM106A
8007483	2,89	7,42	0,00753	1,57	2,96	0,01359	NM_145041	XPR1
7907861	2,89	7,42	0,01430	4,18	18,08	0,00001	NM_004736	RHBDD1
8048772	2,89	7,42	0,01207	2,73	6,66	0,00313	NM_032276	ASAH2
7927599	2,89	7,42	0,00211	4,19	18,26	0,00120	NM_019893	TMEM116
7966448	2,89	7,42	0,00143	2,56	5,88	0,00354	NM_138341	GINS1
8061471	2,89	7,41	0,00547	4,87	29,22	0,00006	NM_021067	GBE1
8088958	2,89	7,41	0,01502	1,40	2,64	0,00578	NM_000158	USPL1
7968333	2,89	7,41	0,01996	2,57	5,95	0,00042	NM_005800	FAP
8056257	2,89	7,41	0,02043	-1,54	-2,91	0,00249	NM_004460	FAM164A
8147019	2,89	7,41	0,00044	2,68	6,42	0,00818	NM_016010	SMU1
8160622	2,89	7,41	0,00025	3,15	8,85	0,00218	NM_018225	TWF1
8013906	2,89	7,41	0,00151	1,85	3,60	0,00044	NM_002822	PIK3R4
8090639	2,89	7,40	0,01219	3,12	8,67	0,00407	NM_014602	PEX13
8042161	2,89	7,39	0,01247	3,19	9,12	0,00087	NM_002618	ME2
8021208	2,89	7,39	0,01259	3,23	9,36	0,00032	NM_002396	HAUS2
7983123	2,89	7,39	0,00500	2,15	4,45	0,16323	NM_018097	NGLY1
8085852	2,88	7,39	0,00284	2,98	7,89	0,00003	NM_018297	CPSF3
8040142	2,88	7,39	0,00154	3,63	12,39	0,00022	NM_016207	KIAA0317

7980189	2,88	7,38	0,00806	3,94	15,34	0,00146	NM_001039479	WLS
7916862	2,88	7,38	0,00201	2,65	6,28	0,00450	NM_024911	GOLM1
8162117	2,88	7,37	0,00276	3,12	8,71	0,00124	NM_016548	DDX47
7954036	2,88	7,37	0,00927	2,91	7,54	0,00045	NM_016355	ANP32A // ANP32A
7982531	2,88	7,37	0,00785	2,79	6,92	0,00436	NM_006305 // NM_006305	SUPT7L
8051204	2,88	7,37	0,00126	4,47	22,24	0,00023	NM_014860	MYADM
8031047	2,88	7,37	0,00247	1,65	3,14	0,00030	NM_001020818	NPAT
7951497	2,88	7,37	0,00599	1,72	3,28	0,03811	NM_002519	NUP98
7945902	2,88	7,36	0,00290	3,71	13,10	0,00003	NM_016320	IGFBP4
8007100	2,88	7,36	0,03614	1,87	3,65	0,02457	NM_001552	HLA-A
8117800	2,88	7,35	0,01112	3,08	8,43	0,00153	NM_002116	UBA6
8100615	2,88	7,35	0,00043	2,07	4,20	0,01895	NM_018227	MYC
8148317	2,88	7,34	0,00199	3,61	12,25	0,00155	NM_002467	FAM13B
8114326	2,87	7,34	0,00034	2,12	4,34	0,01226	NM_016603	CPEB2
8094169	2,87	7,34	0,00121	1,96	3,88	0,01181	NM_182485	RAP2A
7969693	2,87	7,33	0,00526	1,78	3,44	0,00774	NM_021033	STX3
7940191	2,87	7,33	0,00106	3,09	8,53	0,00001	NM_004177	UACA
7990054	2,87	7,33	0,00458	0,64	1,56	0,12877	NM_018003	NDUFB10
7992402	2,87	7,32	0,00751	2,95	7,75	0,00343	NM_004548	---
7964828	2,87	7,32	0,01067	2,50	5,66	0,00171	--- RAB3B	
7916112	2,87	7,32	0,02098	3,68	12,79	0,00035	NM_002867	ABCD3
7903119	2,87	7,31	0,01067	3,95	15,50	0,00002	NM_002858	---
8145134	2,87	7,31	0,00623	0,08	1,06	0,82487	--- PDIA5	
8082133	2,87	7,31	0,00553	2,59	6,02	0,00083	NM_006810	NOLC1
7930008	2,87	7,30	0,03465	3,55	11,71	0,00469	NM_004741	SNORD116-8
7981964	2,87	7,30	0,01190	-2,18	-4,54	0,11817	NR_003323	BRAP
7966427	2,87	7,30	0,00019	2,81	7,01	0,00691	NM_006768	ZCCHC9
8106702	2,87	7,30	0,00614	2,63	6,20	0,00270	NM_032280	H3F3C
7962194	2,87	7,29	0,08550	2,69	6,44	0,01155	NM_001013699	CTGF
8129562	2,87	7,29	0,00484	1,13	2,18	0,07931	NM_001901	STK17B
8057887	2,87	7,29	0,04242	2,08	4,23	0,04482	NM_004226	C5orf24
8108163	2,87	7,29	0,00312	3,07	8,40	0,02153	NM_152409	WBP2
8018482	2,87	7,29	0,00311	3,77	13,68	0,00142	NM_012478	ACTG1
8019183	2,87	7,29	0,01262	3,30	9,86	0,00001	NM_001614	BLMH
8014008	2,87	7,29	0,01581	2,84	7,16	0,00018	NM_000386	MAP4K3
8051707	2,86	7,28	0,01938	2,38	5,22	0,07543	NM_003618	RNF2
7908169	2,86	7,28	0,00153	2,55	5,87	0,00205	NM_007212	SERTAD2
8052669	2,86	7,28	0,00155	2,85	7,22	0,00068	NM_014755	DCP1A
8088128	2,86	7,28	0,00084	3,47	11,09	0,00208	NM_018403	DNAJC7
8015490	2,86	7,28	0,00071	3,27	9,63	0,00249	NM_003315	GLS
8047097	2,86	7,27	0,00231	2,90	7,46	0,00173	NM_014905	SNRK
8079140	2,86	7,27	0,00748	2,61	6,13	0,00092	NM_017719	AQR
7987325	2,86	7,27	0,00027	3,22	9,33	0,00256	NM_014691	VAT1
8015759	2,86	7,27	0,00116	3,17	9,00	0,00481	NM_006373	BIRC2
7943424	2,86	7,27	0,00565	3,61	12,21	0,00057	NM_001166	SFRS2IP
7962499	2,86	7,26	0,00143	2,15	4,44	0,00500	NM_004719	KPNA4
8091764	2,86	7,26	0,01840	3,79	13,83	0,00022	NM_002268	ANPEP
7991335	2,86	7,26	0,00939	-0,80	-1,74	0,06033	NM_001150	DAP3
7906041	2,86	7,26	0,00216	3,19	9,10	0,00001	NM_033657	NUP93
7995843	2,86	7,26	0,00614	3,42	10,73	0,00114	NM_014669	LAPTM4B
8147503	2,86	7,26	0,02384	4,53	23,16	0,00756	NM_018407	DKK3
7946661	2,86	7,25	0,00018	-0,53	-1,44	0,07865	NM_015881	ZNF259
7951838	2,86	7,25	0,00684	3,40	10,52	0,00002	NM_003904	PCM1
8144812	2,86	7,25	0,00188	3,09	8,51	0,00528	NM_006197	KDELR1
8038078	2,86	7,25	0,02623	3,18	9,07	0,00015	NM_006801	PJA2
8113469	2,86	7,25	0,00566	2,75	6,74	0,00057	NM_014819	SFT2D2
7907135	2,86	7,24	0,01040	3,29	9,80	0,00143	NM_199344	RAB21
7957177	2,86	7,24	0,02291	3,41	10,60	0,00051	NM_014999	POLDIP2
8013588	2,85	7,23	0,01602	3,99	15,92	0,00113	NM_015584	MFSD6
8047078	2,85	7,23	0,00111	2,55	5,84	0,00052	NM_017694	SPTAN1
8158317	2,85	7,23	0,00325	2,93	7,61	0,00225	NM_001130438	TMEM2
8161701	2,85	7,23	0,02287	4,09	17,05	0,00075	NM_013390	ELP2
8020930	2,85	7,23	0,00655	3,24	9,45	0,00001	NM_018255	PKM2
7990151	2,85	7,22	0,00008	2,62	6,15	0,00015	NM_182470	DDX24
7981032	2,85	7,22	0,00057	3,26	9,56	0,00006	NM_020414	MFAP3
8109403	2,85	7,21	0,01085	3,96	15,54	0,00177	NR_024152	EIF4H
8133434	2,85	7,20	0,00765	2,86	7,25	0,00095	NM_022170	ELL2
8113220	2,85	7,19	0,00028	1,82	3,53	0,01196	NM_012081	HUWE1
8172914	2,85	7,19	0,00645	3,32	10,01	0,01502	NM_031407	GLCCI1
8131496	2,84	7,18	0,01993	2,95	7,70	0,00434	NM_138426	HMGA2
7956867	2,84	7,18	0,00054	0,23	1,17	0,19155	NM_003483	GK
8174103	2,84	7,18	0,00779	2,88	7,38	0,01759	NM_001128127	ATE1
7936762	2,84	7,18	0,00344	4,22	18,64	0,00001	NM_001001976	INTS4L1

8133074	2,84	7,17	0,01098	2,58	5,96	0,00387	NR_027393	ZNF708
8035803	2,84	7,17	0,00017	1,87	3,64	0,00886	NM_021269	PIK3CA
8084016	2,84	7,17	0,00588	3,30	9,86	0,00028	NM_006218	ATL1
7974270	2,84	7,16	0,00312	1,01	2,01	0,06024	NM_015915	TMEM192
8103524	2,84	7,16	0,00549	1,94	3,85	0,00213	NM_001100389	NUPR1
8000574	2,84	7,16	0,00050	0,44	1,35	0,07095	NM_001042483	MRPL18
8123148	2,84	7,16	0,02010	2,66	6,34	0,00128	NM_014161	EYA1
8151310	2,84	7,15	0,00913	-0,57	-1,49	0,08420	NM_000503	C11orf58
7938669	2,84	7,14	0,02419	3,15	8,89	0,00266	NM_014267	ASH2L
8145894	2,84	7,14	0,01058	3,98	15,74	0,00092	NM_004674	LPAR6
7971565	2,84	7,14	0,01364	-1,40	-2,64	0,02407	NM_005767	RBBP8
8020468	2,83	7,13	0,00451	3,96	15,57	0,00103	NM_002894	CSNK1G3
8107655	2,83	7,13	0,00368	3,17	9,02	0,00019	NM_004384	CLCC1
7918255	2,83	7,12	0,00444	2,73	6,64	0,00446	NM_001048210	TMEM170A
8002878	2,83	7,12	0,00897	4,77	27,38	0,00009	NM_145254	COBLL1
8056343	2,83	7,12	0,00080	1,09	2,12	0,00943	NM_014900	C12orf62
7955358	2,83	7,12	0,00126	2,01	4,02	0,00029	BC007849 AKAP10	
8013431	2,83	7,11	0,00876	3,18	9,04	0,00105	NM_007202	MMAA
8097670	2,83	7,11	0,00220	1,17	2,25	0,02596	NM_172250	GTF2H3
7959623	2,83	7,11	0,00243	3,34	10,13	0,00183	NM_001516	TMEM179B
7940711	2,83	7,10	0,00026	3,16	8,93	0,00087	NM_199337	ADIPOR1
7923503	2,83	7,10	0,00801	4,45	21,86	0,01214	NM_015999	SMAD2
8023195	2,83	7,09	0,00959	3,45	10,95	0,00007	NM_005901	CYP2R1
7946742	2,83	7,09	0,01744	4,15	17,76	0,00010	NM_024514	PSMB3
8006812	2,83	7,09	0,02112	2,08	4,24	0,00628	NM_002795	TMOD1
8156706	2,83	7,09	0,03126	1,95	3,86	0,01110	NM_003275	NUP133
7924923	2,83	7,09	0,00285	4,06	16,67	0,00016	NM_018230	REEP3
7927799	2,82	7,08	0,00026	2,53	5,79	0,00150	NM_001001330	ARMCX6
8174086	2,82	7,08	0,00072	3,10	8,56	0,00054	NM_019007	VPS11
7944382	2,82	7,08	0,00151	2,79	6,93	0,00005	NM_021729	TMED5
7917741	2,82	7,08	0,00454	2,52	5,75	0,00999	NM_016040	TINF2
7978208	2,82	7,08	0,00611	3,19	9,11	0,00521	NM_012461	---
8133902	2,82	7,08	0,00201	1,95	3,85	0,03737	--- USP46	
8100328	2,82	7,08	0,01244	2,44	5,42	0,00060	NM_022832	EIF2A
8083333	2,82	7,07	0,00130	2,57	5,94	0,00288	NM_032025	---
8138545	2,82	7,06	0,00991	1,70	3,25	0,00826	--- APPBP2	
8017235	2,82	7,06	0,00527	3,56	11,82	0,00058	NM_006380	METAP2
7957633	2,82	7,06	0,00094	3,19	9,10	0,00502	NM_006838	SPRED1
7982564	2,82	7,05	0,00116	2,62	6,16	0,00119	NM_152594	SRGAP2
7904469	2,82	7,05	0,00722	3,57	11,87	0,00027	NM_015326	MR1
7907893	2,81	7,04	0,00226	2,64	6,22	0,00025	NM_001531	RABL2A
8044669	2,81	7,03	0,01029	3,18	9,09	0,00187	NM_013412	YY1AP1
7920799	2,81	7,03	0,00005	1,97	3,91	0,01141	NM_139118	TIA1
8052845	2,81	7,02	0,00352	4,09	17,00	0,00029	NM_022173	GTPBP8
8081676	2,81	7,02	0,00164	1,99	3,98	0,01367	NM_014170	SRFBP1
8107578	2,81	7,01	0,01137	2,31	4,95	0,03214	NM_152546	STRAP
7954173	2,81	7,01	0,01445	3,71	13,12	0,00179	NM_007178	EPHX1
7910111	2,81	7,01	0,00058	3,08	8,45	0,00314	NM_000120	ATP6V1A
8081740	2,81	7,01	0,01626	2,63	6,18	0,00035	NM_001690	YTHDF2
7899519	2,81	7,01	0,00229	2,63	6,20	0,00040	NM_016258	FADS1
7948612	2,81	7,00	0,00191	4,62	24,54	0,00055	NM_013402	FRRS1
7917954	2,81	7,00	0,02064	1,96	3,90	0,01143	NM_001013660	DONSON
8070129	2,81	7,00	0,01573	3,59	12,03	0,00056	NM_017613	MORN2
8041561	2,81	7,00	0,00883	2,07	4,19	0,01580	NM_001145450	MAPK14
8119000	2,81	7,00	0,00072	3,74	13,33	0,00003	NM_001315	PARN
7999562	2,81	6,99	0,00429	2,51	5,69	0,02372	NM_002582	SSR2
7920903	2,81	6,99	0,00382	3,88	14,72	0,00005	NM_003145	PREPL
8051928	2,80	6,99	0,01088	2,90	7,47	0,00095	NM_006036	TBC1D15
7957186	2,80	6,99	0,00744	3,22	9,33	0,01371	NM_022771	MTMR9
8144586	2,80	6,99	0,01908	3,63	12,34	0,00056	NM_015458	C1orf198
7924996	2,80	6,98	0,00529	2,10	4,28	0,00013	NM_032800	PCGF3
8093398	2,80	6,97	0,00170	3,40	10,57	0,00010	NM_006315	MT1E
7995797	2,80	6,97	0,02184	3,57	11,88	0,00041	NM_175617	GNPDA2
8100085	2,80	6,97	0,00165	3,11	8,63	0,00610	NM_138335	KCNRG
7969166	2,80	6,97	0,00205	4,21	18,45	0,00389	NM_173605	SLC7A6
7996772	2,80	6,97	0,01835	3,65	12,53	0,00143	NM_003983	SLC37A3
8143367	2,80	6,97	0,00447	2,38	5,22	0,00068	NM_207113	TFPI2
8141016	2,80	6,96	0,01420	3,73	13,30	0,00012	NM_006528	ARMC8
8082940	2,80	6,95	0,01922	3,75	13,47	0,00065	NM_015396	AKR1C2
7931832	2,80	6,95	0,00238	1,78	3,44	0,05273	NM_001354	PSME4
8052149	2,79	6,94	0,02816	2,96	7,76	0,00398	NM_014614	ZFHX4
8147000	2,79	6,94	0,00385	0,66	1,58	0,01835	NM_024721	APOBEC3B
8073062	2,79	6,94	0,00515	5,13	34,97	0,00004	NM_004900	LOC402778

7945663	2,79	6,94	0,00528	1,76	3,40	0,00053	NM_001170820	PMS2L11
8133754	2,79	6,93	0,00188	3,97	15,63	0,00002	NR_023383	GSTK1
8136849	2,79	6,93	0,00278	1,71	3,26	0,02047	NM_001143679	NPIP
7999634	2,79	6,92	0,02179	3,74	13,35	0,00029	NM_006985	RIN2
8061247	2,79	6,92	0,00764	1,35	2,55	0,00180	NM_018993	LCMT1
7994237	2,79	6,92	0,01641	3,78	13,72	0,00027	NM_016309	CTR9
7938422	2,79	6,92	0,00565	2,92	7,55	0,00790	NM_014633	ZNF146
8028186	2,79	6,91	0,00342	5,13	35,05	0,00004	NM_007145	RPS11
8030368	2,79	6,91	0,00092	1,62	3,08	0,00237	NM_001015	PROS1
8089015	2,79	6,91	0,00693	2,71	6,54	0,00838	NM_000313	PHGDH
7904433	2,79	6,91	0,01303	3,49	11,25	0,00079	NM_006623	SDHD
7899016	2,79	6,91	0,00488	4,66	25,32	0,00786	NM_003002	SNW1
7980463	2,79	6,90	0,00031	2,65	6,28	0,00598	NM_012245	BCL6
8092691	2,79	6,90	0,01802	1,86	3,63	0,00364	NM_001706	PCGF5
7929132	2,79	6,90	0,00350	3,59	12,05	0,00010	NM_032373	HLA-C
8124901	2,79	6,90	0,00347	3,41	10,61	0,00193	NM_002117	ITGBL1
7969861	2,79	6,90	0,01643	-0,20	-1,15	0,08436	NM_004791	MANSC1
7961365	2,79	6,90	0,01728	1,25	2,37	0,03169	NM_018050	CCDC50
8084818	2,78	6,89	0,00085	2,71	6,55	0,00029	NM_178335	MPDZ
8160088	2,78	6,89	0,00983	2,80	6,95	0,00007	NM_003829	GLI3
8139212	2,78	6,89	0,01145	-0,20	-1,15	0,33907	NM_000168	PCNP
7971563	2,78	6,88	0,00579	1,99	3,97	0,00051	NM_020357	NDFIP1
8108861	2,78	6,88	0,00822	1,84	3,58	0,00182	NM_030571	TTLL5
7975815	2,78	6,88	0,00275	3,16	8,92	0,00122	NM_015072	SPON2
8098870	2,78	6,88	0,00013	-1,13	-2,19	0,03551	NM_012445	EPB41L2
8129497	2,78	6,88	0,00382	3,06	8,37	0,00001	NM_001431	FBXL4
8128356	2,78	6,87	0,00099	2,65	6,26	0,00082	NM_012160	HADHA
8050908	2,78	6,87	0,01398	4,21	18,46	0,00011	NM_000182	RPS23
8112961	2,78	6,86	0,00154	3,50	11,30	0,00136	NM_001025	HDDC2
8129363	2,78	6,86	0,00114	2,11	4,32	0,00323	NM_016063	DDX3X
8166876	2,78	6,85	0,00842	3,04	8,22	0,00003	NM_001356	PGGT1B
8113591	2,78	6,85	0,01612	2,92	7,57	0,00277	NM_005023	SNORA23
7938329	2,78	6,85	0,00714	1,18	2,27	0,07149	NR_002962	MLL5
8135277	2,78	6,85	0,00232	2,54	5,82	0,00024	NM_182931	BUB3
7931187	2,78	6,85	0,01725	4,44	21,65	0,00074	NM_004725	PLRG1
8103289	2,77	6,84	0,00155	2,79	6,92	0,00028	NM_002669	PSME3
8007397	2,77	6,83	0,01264	3,49	11,25	0,00003	NM_176863	LAYN
7943749	2,77	6,83	0,00039	-0,61	-1,52	0,00552	NM_178834	PCNP
8081348	2,77	6,83	0,02085	2,80	6,94	0,00464	NM_020357	C1orf21
7908161	2,77	6,83	0,01209	1,82	3,52	0,00116	NM_030806	PAK2
8084963	2,77	6,83	0,02254	4,59	24,07	0,00032	NM_002577	NRBF2
7927775	2,77	6,82	0,00565	0,39	1,31	0,04329	NM_030759	SMAP1
8120585	2,77	6,82	0,00237	2,32	5,01	0,00500	NM_001044305	PDHX
7939329	2,77	6,82	0,02577	2,51	5,70	0,00843	NM_003477	TM2D2
8150364	2,77	6,82	0,00568	3,71	13,11	0,00086	NM_031940	DPYSL2
8145470	2,77	6,81	0,00747	3,13	8,78	0,00338	NM_001386	EAF1
8078138	2,77	6,81	0,00959	2,90	7,48	0,00208	NM_033083	RRN3
7994565	2,77	6,81	0,01231	3,72	13,21	0,00376	NM_018427	GBF1
7930031	2,77	6,81	0,00136	3,08	8,43	0,00048	NM_004193	C6orf203
8121312	2,77	6,81	0,00049	0,80	1,75	0,19813	NM_016487	ATP5A1
8023063	2,77	6,80	0,00130	2,20	4,60	0,00704	NM_001001937	CSNK1D
8019463	2,77	6,80	0,00557	2,72	6,60	0,00046	NM_139062	LRBA
8103106	2,77	6,80	0,00556	3,60	12,10	0,00108	NM_006726	LRRC8D
7902883	2,76	6,79	0,00957	3,04	8,23	0,00002	NM_001134479	TAF1D
7951008	2,76	6,79	0,00059	1,94	3,83	0,03363	NM_024116	HCFC2
7958158	2,76	6,79	0,01584	2,19	4,57	0,00314	NM_013320	GPN1
8041000	2,76	6,77	0,01682	3,11	8,63	0,00011	NM_007266	TTC33
8111788	2,76	6,76	0,00800	1,53	2,89	0,00661	NM_012382	KIAA1191
8115895	2,76	6,76	0,00302	3,86	14,50	0,00073	NM_020444	YEATS4
7957032	2,76	6,76	0,01522	2,00	3,99	0,00878	NM_006530	PAN3
7968274	2,76	6,76	0,00258	3,49	11,22	0,00102	NM_175854	HERC3
8096385	2,76	6,76	0,02491	1,21	2,31	0,00577	NM_014606	PACSIN2
8076533	2,76	6,76	0,00390	2,37	5,18	0,00267	NM_007229	CIAPIN1
8001552	2,76	6,76	0,00013	3,31	9,88	0,00614	NM_020313	SLIT3
8115691	2,76	6,75	0,02695	-1,55	-2,93	0,00252	NM_003062	ABHD10
8081612	2,75	6,75	0,02954	3,16	8,92	0,00061	NM_018394	USP8
7983663	2,75	6,75	0,01123	3,50	11,30	0,00326	NM_005154	RARRES3
7940775	2,75	6,74	0,00782	0,21	1,16	0,40963	NM_004585	ZC3HAV1
8143279	2,75	6,73	0,00897	2,78	6,86	0,00265	NM_020119	CAMSAP1L1
7908614	2,75	6,72	0,00138	4,02	16,18	0,00222	NM_203459	PMS2L5
8133309	2,75	6,72	0,04889	4,12	17,41	0,00021	NR_027776	SDHD
7943853	2,75	6,72	0,00022	3,63	12,39	0,00078	NM_003002	CKAP2
7969243	2,75	6,71	0,00153	3,07	8,41	0,00819	NM_018204	SMAD3

7984364	2,74	6,69	0,02062	3,60	12,14	0,00012	NM_005902	TMEM188
7995448	2,74	6,69	0,01049	4,54	23,26	0,00008	NM_153261	TMEM14B
8116867	2,74	6,69	0,09022	5,61	48,76	0,00002	NM_030969	PUM1
7914296	2,74	6,69	0,01513	4,10	17,21	0,00039	NM_001020658	IFIT2
7929047	2,74	6,69	0,00319	-0,27	-1,20	0,02025	NM_001547	PTDSS1
8147447	2,74	6,68	0,00704	4,57	23,68	0,00006	NM_014754	ALG9
7951633	2,74	6,68	0,00331	3,22	9,32	0,00033	NM_024740	CH25H
7934916	2,74	6,67	0,05538	-0,45	-1,36	0,00463	NM_003956	CADPS2
8142585	2,74	6,67	0,01736	1,23	2,35	0,00259	NM_017954	CCT7
8042720	2,74	6,67	0,01084	4,24	18,89	0,00005	NM_006429	CDH13
7997504	2,74	6,67	0,00381	-1,39	-2,62	0,00957	NM_001257	ERAP1
8113250	2,74	6,67	0,00863	3,75	13,46	0,00072	NM_001040458	ZNF121
8033789	2,74	6,67	0,00130	2,75	6,71	0,00118	NM_001008727	C6orf89
8119109	2,74	6,66	0,00395	2,44	5,41	0,00470	NM_152734	DDX52
8014574	2,74	6,66	0,01383	4,30	19,70	0,00002	NM_007010	PIK3CB
8091009	2,73	6,65	0,00624	3,93	15,29	0,00079	NM_006219	DYNLL1
7959164	2,73	6,65	0,00601	3,09	8,54	0,00496	NM_001037495	GANAB
7948744	2,73	6,64	0,00195	4,02	16,27	0,00063	NM_198335	LMBRD2
8111533	2,73	6,64	0,00339	3,51	11,42	0,00210	NM_001007527	ASAP2
8040113	2,73	6,64	0,01154	4,77	27,28	0,00000	NM_003887	KIAA1429
8151842	2,73	6,63	0,00190	2,86	7,28	0,00237	NM_015496	EIF2S1
7975224	2,73	6,63	0,01113	4,60	24,18	0,00206	NM_004094	PPP1R15B
7923659	2,73	6,63	0,00468	3,74	13,33	0,00006	NM_032833	RWDD2B
8069744	2,73	6,63	0,05660	3,32	9,98	0,00087	NM_016940	CDK12
8006850	2,73	6,62	0,00027	2,78	6,87	0,01088	NM_016507	LARP4B
7931728	2,73	6,62	0,00054	3,62	12,33	0,00054	NM_015155	CALD1
8136347	2,73	6,61	0,00080	-0,10	-1,07	0,55008	NM_033138	C1orf9
7907404	2,73	6,61	0,01544	3,49	11,22	0,00245	NM_014283	AARS
8002347	2,72	6,61	0,00338	4,34	20,21	0,00390	NM_001605	NOC3L
7935146	2,72	6,61	0,01260	2,79	6,93	0,00066	NM_022451	RB1CC1
8150757	2,72	6,61	0,00597	2,73	6,64	0,00037	NM_014781	PSMD2
8084423	2,72	6,60	0,00684	3,19	9,10	0,00103	NM_002808	ANLN
8132318	2,72	6,60	0,00051	3,05	8,29	0,03007	NM_018685	C9orf102
8156604	2,72	6,59	0,00047	1,25	2,37	0,13229	BC022957 CLASP1	
8054888	2,72	6,59	0,00179	3,27	9,66	0,00020	NM_015282	ATP8B1
8023497	2,72	6,58	0,00326	2,17	4,49	0,00045	NM_005603	TGOLN2
8053388	2,72	6,58	0,00069	1,67	3,19	0,00246	NM_006464	C7orf60
8142415	2,72	6,58	0,02216	3,30	9,87	0,00073	NM_152556	PCMTD2
7927095	2,72	6,58	0,07823	2,32	4,99	0,19754	BC032332 CRTAP	
8078450	2,72	6,58	0,00227	3,24	9,46	0,00061	NM_006371	ARHGAP29
7917850	2,72	6,57	0,00766	3,75	13,44	0,00028	NM_004815	BBS9
8132218	2,72	6,57	0,01389	2,07	4,20	0,01254	NM_198428	PCNX
7975416	2,72	6,57	0,00746	3,62	12,31	0,00032	NM_014982	SAMHD1
8066117	2,72	6,57	0,00531	2,44	5,42	0,00009	NM_015474	ARMCX6
8168873	2,71	6,57	0,00743	2,18	4,53	0,02010	NM_019007	LSM3
8078008	2,71	6,56	0,00449	3,34	10,15	0,00019	NM_014463	BPTF
8009382	2,71	6,56	0,00186	2,66	6,31	0,00835	NM_004459	CD63
7963911	2,71	6,56	0,00095	1,33	2,51	0,02114	NM_001780	NR1D2
8078272	2,71	6,56	0,02796	3,25	9,52	0,00390	NM_005126	NCSTN
7906576	2,71	6,55	0,00871	3,65	12,59	0,00116	NM_015331	GDE1
7999889	2,71	6,55	0,00285	3,16	8,92	0,00479	NM_016641	SKAP2
8138689	2,71	6,55	0,00244	4,03	16,35	0,00028	NM_003930	IRAK3
7956878	2,71	6,55	0,01833	3,01	8,06	0,00060	NM_007199	HIST1H2BF
8117395	2,71	6,54	0,01171	2,81	7,02	0,00595	NM_003522	ZADH2
8023871	2,71	6,54	0,00048	0,28	1,21	0,23841	NM_175907	QSOX1
7907830	2,71	6,54	0,01207	2,35	5,08	0,00795	NM_002826	KTN1
7974483	2,71	6,54	0,00162	2,34	5,06	0,00204	NM_182926	MLH1
8078544	2,71	6,54	0,01474	2,85	7,23	0,00135	NM_000249	MIER3
8112182	2,71	6,53	0,00117	1,42	2,68	0,02529	NM_152622	C19orf56
8034448	2,71	6,52	0,01233	2,69	6,46	0,00023	NM_016145	MRPL15
8146448	2,71	6,52	0,02059	3,46	10,97	0,00231	NM_014175	WDFY1
8059361	2,71	6,52	0,01118	2,03	4,10	0,00073	NM_020830	FOXP2
8135544	2,70	6,52	0,03352	0,55	1,46	0,16379	NM_148898	MSL3L2 // MSL3L2 // MSL3L2 // MSL3L2 // MSL3L2
8059852	2,70	6,51	0,00128	-0,11	-1,08	0,81040	NM_001166217 // NM_001166217 // NM_001166217 // NM_001166217 // NM_001166217	GRB10
8139656	2,70	6,51	0,00161	3,91	15,07	0,00001	NM_001001555	KCTD20
8119067	2,70	6,51	0,01013	4,66	25,27	0,00097	NM_173562	RPS25
8113660	2,70	6,51	0,00153	1,93	3,80	0,07940	NM_001028	NEU1
8125139	2,70	6,51	0,00155	2,20	4,59	0,00502	NM_000434	NEU1
8179851	2,70	6,51	0,00155	2,20	4,59	0,00502	NM_000434	HLA-DPA1
8180100	2,70	6,51	0,00125	1,44	2,72	0,00120	NM_033554	P4HA2
8113981	2,70	6,50	0,00459	2,58	5,96	0,00001	NM_004199	UBR5

8152148	2,70	6,50	0,00201	3,47	11,07	0,00741	NM_015902	MEA1
8126474	2,70	6,50	0,00207	1,72	3,29	0,00385	NM_014623	KIAA0196
8152782	2,70	6,50	0,00858	3,36	10,30	0,00291	NM_014846	GALNT13
8045776	2,70	6,50	0,04157	-0,99	-1,99	0,00001	NM_052917	CAB39
8048980	2,70	6,50	0,00426	2,77	6,83	0,00184	NM_016289	YTHDF1
8067593	2,70	6,50	0,00053	4,30	19,71	0,00041	NM_017798	DDX58
8160559	2,70	6,50	0,01331	1,61	3,05	0,01350	NM_014314	ROBO1
8088919	2,70	6,50	0,02231	-1,53	-2,88	0,00001	NM_133631	MAPKAP1
8164177	2,70	6,49	0,00191	2,95	7,71	0,00015	NM_001006617	BMPR1A
7928855	2,70	6,49	0,00476	3,69	12,89	0,00082	NM_004329	BDKRB1
7976567	2,70	6,49	0,00351	-0,47	-1,39	0,10756	NM_000710	ASCC2
8075285	2,70	6,48	0,00569	3,34	10,13	0,00085	NM_032204	STAMBP
8042772	2,70	6,48	0,00240	2,64	6,25	0,00092	NM_213622	CDK17
7965652	2,70	6,48	0,00264	3,87	14,63	0,00014	NM_002595	C9orf30
8156897	2,70	6,48	0,01356	3,00	8,02	0,00181	BC008993 TAB2	
8122672	2,69	6,47	0,00253	2,82	7,08	0,00692	NM_015093	ABCB7
8173629	2,69	6,47	0,02577	3,77	13,62	0,00002	NM_004299	G3BP2
8101043	2,69	6,46	0,00403	2,63	6,21	0,00012	NM_203505	BVES
8128553	2,69	6,45	0,02050	3,08	8,44	0,00134	NM_147147	NDUFS4
8105311	2,69	6,45	0,05362	2,39	5,25	0,00040	NM_002495	GCSH
7922104	2,69	6,45	0,00885	2,90	7,47	0,00684	NM_004483	C11orf57
7943842	2,69	6,45	0,00716	2,57	5,92	0,00303	NM_018195	LMO7
7969438	2,69	6,45	0,00838	2,46	5,51	0,00267	NM_005358	SQRDL
7983512	2,69	6,44	0,00056	3,12	8,71	0,00115	NM_021199	FAM104A
8018097	2,69	6,44	0,00103	3,81	14,01	0,00002	NM_001098832	ATP11C
8175492	2,69	6,44	0,01416	2,72	6,58	0,00055	NM_173694	VGLL3
8088979	2,69	6,43	0,00510	-0,90	-1,87	0,00013	NM_016206	UHMK1
7906852	2,68	6,43	0,00663	2,35	5,09	0,00047	NM_175866	XPNPEP1
7936284	2,68	6,43	0,00915	2,99	7,94	0,00000	NM_020383	TMX1
7974303	2,68	6,43	0,01680	3,32	9,96	0,00439	NM_030755	MRPL48
7942465	2,68	6,43	0,02104	2,41	5,32	0,00624	NM_016055	TBRG1
7944850	2,68	6,43	0,02125	3,50	11,28	0,00062	NM_032811	NPR3
8104746	2,68	6,42	0,00679	0,21	1,16	0,28189	NM_000908	PPT1
7915286	2,68	6,42	0,02806	4,59	24,15	0,00000	NM_000310	SMG7
7908097	2,68	6,42	0,01342	3,21	9,23	0,00112	NM_173156	FNIP2
8098103	2,68	6,42	0,00125	1,63	3,10	0,03204	NM_020840	SGCB
8100318	2,68	6,41	0,06306	2,24	4,72	0,00045	NM_000232	ZRANB2
7916969	2,68	6,41	0,00465	2,54	5,80	0,00153	NM_005455	SKP2
8104912	2,68	6,41	0,00593	3,93	15,29	0,00597	NM_005983	WEE1
7938348	2,68	6,41	0,00052	1,75	3,36	0,01754	NM_003390	DYNC1H1
7976876	2,68	6,41	0,01361	2,87	7,32	0,01261	NM_001376	SMAD2
8023191	2,68	6,40	0,00996	2,86	7,28	0,00818	NM_005901	GLUL
8154951	2,68	6,40	0,01582	1,65	3,14	0,05886	NM_002065	MYST2
8008213	2,68	6,40	0,00099	3,57	11,89	0,00230	NM_007067	RPL18
8038086	2,68	6,39	0,01791	3,62	12,33	0,00220	NM_000979	TWF1
7962441	2,67	6,39	0,00373	1,63	3,10	0,00099	NM_002822	BTG2
7908917	2,67	6,38	0,02009	1,62	3,08	0,00753	NM_006763	COMMD3
7926596	2,67	6,38	0,00356	1,10	2,15	0,05093	NM_012071	ZNF302
8027674	2,67	6,38	0,00066	2,14	4,40	0,00479	NM_018443	FAM120B
8123494	2,67	6,38	0,00088	1,50	2,83	0,00696	NM_032448	ORMDL1
8057700	2,67	6,38	0,00903	3,40	10,52	0,00041	NM_016467	CSGALNACT2
7927146	2,67	6,38	0,00801	2,53	5,76	0,00183	NM_018590	PODN
7901535	2,67	6,38	0,02205	-0,60	-1,51	0,00189	NM_153703	HSPA4
8108015	2,67	6,37	0,02524	2,55	5,85	0,00010	NM_002154	RAB35
7966929	2,67	6,37	0,00050	2,90	7,47	0,00174	NM_006861	LOC100132992
8023526	2,67	6,37	0,04546	3,43	10,80	0,00078	ENST00000446244	DEK
8124144	2,67	6,36	0,00006	3,10	8,55	0,01201	NM_003472	NCBP1
8156718	2,67	6,36	0,01488	3,88	14,77	0,00020	NM_002486	LIN52
7975687	2,66	6,34	0,01084	3,12	8,69	0,00278	NM_001024674	RGPD4
8044161	2,66	6,34	0,00272	1,59	3,01	0,03902	NM_182588	SPCS3
8098414	2,66	6,34	0,02278	2,45	5,46	0,00062	NM_021928	ASAM
7952341	2,66	6,34	0,00539	0,90	1,87	0,00286	NM_024769	ATP2C1
8082607	2,66	6,34	0,00518	2,15	4,44	0,00028	NM_014382	ASNA1
8026024	2,66	6,34	0,02190	3,46	11,00	0,00004	NM_004317	VPS13C
7989387	2,66	6,33	0,00330	2,48	5,57	0,00140	NM_020821	UFSP2
8103998	2,66	6,33	0,02947	2,22	4,64	0,00337	NM_018359	DHCR24
7916432	2,66	6,33	0,01707	4,90	29,96	0,00008	NM_014762	LIN7C
7947221	2,66	6,33	0,01210	1,50	2,84	0,02706	NM_018362	CRIM1
8041447	2,66	6,32	0,00043	2,94	7,66	0,02427	NM_016441	SLBP
8098904	2,66	6,32	0,00779	1,88	3,67	0,00491	NM_006527	SKA2
8157691	2,66	6,32	0,02414	4,44	21,72	0,00185	NM_182620	C10orf46
7936596	2,66	6,31	0,00046	2,31	4,96	0,00241	NM_153810	TLR3
8098611	2,66	6,31	0,00424	0,33	1,26	0,38407	NM_003265	TAF11

8125859	2,66	6,31	0,03188	4,06	16,71	0,00033		NM_005643	LPCAT2
7995697	2,66	6,31	0,00695	0,69	1,62	0,18866		NM_017839	ATP10D
8094911	2,66	6,31	0,01478	2,45	5,46	0,00484		NM_020453	RRN3P1
8000200	2,66	6,30	0,01755	3,66	12,64	0,00746		NR_003370	PDE1A
8057486	2,66	6,30	0,00228	-0,95	-1,94	0,00084		NM_005019	ZYG11B
7901479	2,65	6,30	0,03293	3,48	11,13	0,00060		NM_024646	PPIP5K2
8107164	2,65	6,30	0,02555	2,54	5,83	0,00712		NM_015216	C5orf44
8105647	2,65	6,29	0,03907	3,10	8,54	0,00336		NM_001093755	VPS36
7971820	2,65	6,28	0,00405	1,90	3,73	0,00061		NM_016075	PCMTD2
7924549	2,65	6,28	0,02423	2,47	5,53	0,18487		BC032332 PCMTD2	
8049963	2,65	6,28	0,02423	2,47	5,53	0,18487		BC032332 MRPL3	
8090678	2,65	6,28	0,00053	3,44	10,86	0,00001		NM_007208	NACA
7964262	2,65	6,27	0,04345	1,69	3,23	0,02489		NM_001113201	---
8058333	2,65	6,27	0,00899	3,75	13,48	0,00106		--- ZNF828	
7970376	2,65	6,27	0,05263	-1,10	-2,15	0,00021		NM_032436	GCNT4
8112668	2,65	6,27	0,00735	0,20	1,15	0,41594		NM_016591	HUS1
8139592	2,65	6,27	0,00039	3,59	12,05	0,00163		NM_004507	FAIM2
7963142	2,65	6,27	0,03492	-1,71	-3,28	0,00437		NM_012306	NFKBIA
7978644	2,65	6,26	0,04978	3,92	15,12	0,00083		NM_020529	TTC27
8041360	2,65	6,26	0,00392	3,51	11,43	0,00016		NM_017735	SACS
7970569	2,65	6,26	0,03987	3,07	8,38	0,00040		NM_014363	DYNC1LI2
8001841	2,65	6,26	0,01113	3,06	8,36	0,00041		NM_006141	AFTPH
8042291	2,65	6,26	0,00165	2,48	5,56	0,00367		NM_203437	ATP5B
7964234	2,65	6,26	0,01145	4,19	18,30	0,00240		NM_001686	SLC25A13
8141107	2,65	6,26	0,01507	2,79	6,91	0,00083		NM_001160210	SYNPO2
8097080	2,64	6,25	0,00560	-1,44	-2,72	0,00134		NM_133477	ANKMY2
8138370	2,64	6,25	0,04786	2,85	7,19	0,00310		NM_020319	RPS16
8036737	2,64	6,25	0,02582	3,11	8,65	0,00141		NM_001020	PSMA2
8139232	2,64	6,24	0,00888	3,56	11,76	0,00426		NM_002787	TAGLN
7944082	2,64	6,24	0,02363	-1,52	-2,86	0,00153		NM_001001522	TFB2M
7925611	2,64	6,24	0,01260	2,20	4,59	0,04134		NM_022366	HDAC2
8129045	2,64	6,24	0,01524	2,91	7,50	0,00722		NM_001527	DYNLT3
8172035	2,64	6,24	0,02129	1,88	3,69	0,00026		NM_006520	SFRS7
8051622	2,64	6,24	0,01113	3,04	8,22	0,00043		NM_001031684	MED13L
7966706	2,64	6,24	0,00947	4,60	24,17	0,00054		NM_015335	TMEM138
7940473	2,64	6,23	0,04608	5,21	36,94	0,00027		NM_016464	CLPTM1
8029560	2,64	6,22	0,00378	3,99	15,84	0,00099		NM_001294	EIF2C3
7900051	2,64	6,22	0,00535	3,15	8,85	0,00010		NM_024852	SDF2
8013741	2,64	6,22	0,01513	4,01	16,09	0,00520		NM_006923	LASS2
7919856	2,64	6,22	0,00781	3,96	15,58	0,00034		NM_181746	MFSD8
8102730	2,64	6,22	0,01497	3,82	14,14	0,00021		NM_152778	DHRS3
7912537	2,63	6,21	0,00650	0,98	1,98	0,04975		NM_004753	NQO2
8116610	2,63	6,21	0,00422	3,83	14,21	0,00205		NM_000904	PPP4R2
8080973	2,63	6,21	0,00086	1,43	2,70	0,01101		NM_174907	NUDT5
7932069	2,63	6,20	0,04111	4,18	18,12	0,00007		NM_014142	SMAD5
8108238	2,63	6,20	0,01976	4,07	16,82	0,00030		NM_001001419	CEP350
7907790	2,63	6,20	0,00082	2,77	6,81	0,01051		NM_014810	CCDC88A
8052269	2,63	6,20	0,00954	2,78	6,89	0,00196		NM_001135597	HIST1H2BH
8117426	2,63	6,20	0,02987	-0,96	-1,95	0,18146		NM_003524	HSPB1
8133721	2,63	6,20	0,03743	2,99	7,94	0,00438		NM_001540	---
8150204	2,63	6,19	0,00219	2,18	4,53	0,00378		--- PRMT5	
7977820	2,63	6,19	0,01186	5,17	35,89	0,00042		NM_001039619	EHBP1
8042223	2,63	6,18	0,01033	4,19	18,28	0,00048		NM_015252	SRGN
7927964	2,63	6,18	0,01754	0,14	1,10	0,68941		NM_002727	CHPT1
7958000	2,63	6,18	0,00031	3,25	9,54	0,00379		NM_020244	SNRPD1
8020411	2,63	6,18	0,03022	3,30	9,88	0,00162		NM_006938	EPC1
7932938	2,63	6,17	0,00854	3,09	8,49	0,00316		NM_025209	FTH1
8170360	2,63	6,17	0,07281	2,14	4,41	0,00732		NM_002032	KIAA0430
7999642	2,63	6,17	0,00856	2,69	6,43	0,00468		NM_014647	RPL35A
8085026	2,62	6,16	0,00710	3,42	10,69	0,00385		NM_000996	PRKAG2
8143961	2,62	6,16	0,00536	2,36	5,14	0,00660		NM_016203	STAU1
8066889	2,62	6,16	0,00435	3,84	14,36	0,00137		NM_017453	TARS
8104760	2,62	6,16	0,00389	3,19	9,12	0,00174		NM_152295	SLC25A38
8078898	2,62	6,15	0,04230	2,73	6,64	0,00025		NM_017875	RFC1
8099860	2,62	6,15	0,00755	2,65	6,28	0,00029		NM_002913	SMARCC1
8086810	2,62	6,15	0,00134	2,86	7,27	0,01728		NM_003074	NYNRIN
7973727	2,62	6,14	0,01887	-1,36	-2,57	0,13705		NM_025081	FTL
8030171	2,62	6,14	0,01001	3,17	8,97	0,02479		NM_000146	IDS
8170390	2,62	6,14	0,00575	2,43	5,38	0,00315		NM_000202	SCO1
8012823	2,62	6,13	0,01085	3,20	9,17	0,00510		NM_004589	ASH1L
7920766	2,62	6,13	0,00787	3,21	9,23	0,02958		NM_018489	C1orf25
7922870	2,62	6,13	0,00211	2,61	6,12	0,00269		NM_030934	STEAP1
8134030	2,62	6,13	0,01257	2,51	5,71	0,00201		NM_012449	ASCC3

8128472	2,62	6,13	0,01568	2,12	4,35	0,00102	NM_006828	TMEM63B
8119926	2,62	6,13	0,00688	4,15	17,70	0,00026	NM_018426	MBOAT2
8050160	2,62	6,13	0,00116	3,56	11,78	0,00061	NM_138799	RAVER2
7902023	2,61	6,13	0,00427	3,62	12,28	0,00060	NM_018211	SR140
8083183	2,61	6,12	0,00196	3,10	8,56	0,00347	NM_001080415	SPATA6
7915955	2,61	6,11	0,02221	2,75	6,74	0,00422	NM_019073	ZNF295
8070557	2,61	6,11	0,01390	2,90	7,44	0,00021	NM_001098402	ZBTB34
8157941	2,61	6,11	0,00581	2,02	4,06	0,00050	NM_001099270	CALM1
7976200	2,61	6,11	0,00955	3,24	9,48	0,00084	NM_006888	CKAP5
7947694	2,61	6,11	0,00337	3,09	8,51	0,00025	NM_001008938	PIGN
8023605	2,61	6,11	0,02136	2,43	5,39	0,00161	NM_176787	FAM134C
8015655	2,61	6,11	0,00684	3,42	10,74	0,00581	NM_178126	SLC9A6
8170097	2,61	6,10	0,00598	3,57	11,88	0,00023	NM_001042537	SMG1
7999841	2,61	6,10	0,00332	3,47	11,08	0,00296	NM_015092	MPP1
8176174	2,61	6,10	0,01072	3,15	8,88	0,00007	NM_002436	MED31
8011968	2,61	6,09	0,03171	3,59	12,03	0,00068	NM_016060	EDEM3
7922823	2,61	6,09	0,00453	3,53	11,59	0,00005	NM_025191	BZW2
8131583	2,61	6,09	0,00405	3,33	10,04	0,00009	NM_001159767	COLEC10
8148029	2,61	6,09	0,00112	-1,65	-3,14	0,00043	NM_006438	CASP3
8103922	2,61	6,09	0,00047	1,57	2,97	0,00015	NM_004346	DDX6
7952103	2,60	6,08	0,00733	2,33	5,04	0,00795	NM_004397	ACTN4
8028524	2,60	6,08	0,06366	3,80	13,91	0,00236	NM_004924	FAM120A
8162462	2,60	6,08	0,01930	3,16	8,91	0,00685	NM_014612	ECE1
7913357	2,60	6,07	0,00393	3,95	15,45	0,00269	NM_001397	FLOT1
8124828	2,60	6,07	0,00564	3,15	8,89	0,00081	NM_005803	FLOT1
8178419	2,60	6,07	0,00564	3,15	8,89	0,00081	NM_005803	FLOT1
8179688	2,60	6,07	0,00564	3,15	8,89	0,00081	NM_005803	SCHIP1
8083677	2,60	6,07	0,01034	1,81	3,51	0,02206	NM_014575	H3F3A
8046515	2,60	6,07	0,01888	2,30	4,94	0,00024	NM_002107	C20orf11
8064007	2,60	6,06	0,00979	2,91	7,49	0,00041	NM_017896	ST3GAL5
8053484	2,60	6,06	0,01030	0,83	1,78	0,05697	NM_003896	SAPS3
7942032	2,60	6,06	0,01004	2,98	7,90	0,01128	NM_001164162	ZYX
8136918	2,60	6,06	0,01921	3,64	12,47	0,00002	NM_003461	ADAR
7920531	2,60	6,06	0,00057	3,15	8,85	0,00157	NM_001111	NUP205
8136401	2,60	6,06	0,00764	2,93	7,64	0,00121	NM_015135	RGS4
7906919	2,60	6,06	0,00100	-1,74	-3,35	0,00009	NM_001102445	PCOLCE
8134869	2,60	6,05	0,00114	-0,19	-1,14	0,26473	NM_002593	PPP2R3C
7978628	2,60	6,05	0,01259	3,14	8,83	0,00082	NM_017917	EPC2
8045619	2,60	6,05	0,00187	2,25	4,76	0,00384	NM_015630	TRA2B
8092564	2,60	6,05	0,00973	2,51	5,70	0,00106	NM_004593	GIGYF2
8049199	2,60	6,05	0,00949	2,65	6,30	0,00342	NM_001103147	RPSA
8078905	2,60	6,05	0,03309	2,13	4,39	0,00020	NM_002295	MRPS27
8112570	2,60	6,04	0,00833	2,92	7,55	0,00201	NM_015084	CTPS2
8171493	2,59	6,04	0,00437	2,24	4,71	0,00164	NM_175859	PRKAA1
8111796	2,59	6,04	0,01737	2,70	6,51	0,00587	NM_206907	NDRG1
8153002	2,59	6,04	0,00208	2,79	6,90	0,00201	NM_001135242	C15orf44
7989834	2,59	6,04	0,01814	4,12	17,34	0,00001	AK296134 C9orf5	
8163086	2,59	6,03	0,01987	4,24	18,87	0,00022	NM_032012	MLEC
7959205	2,59	6,03	0,00146	3,15	8,90	0,00004	NM_014730	RPS27A
8001782	2,59	6,03	0,00099	1,71	3,27	0,01501	NM_002954	PPP3CB
7934393	2,59	6,03	0,02608	2,90	7,47	0,00045	NM_001142353	GSK3B
8089801	2,59	6,03	0,02420	3,78	13,75	0,00000	NM_002093	HMGN1
8176191	2,59	6,03	0,10825	2,03	4,07	0,00416	NM_004965	PON2
8141076	2,59	6,03	0,01042	3,96	15,54	0,00022	NM_000305	SPTBN1
8041995	2,59	6,03	0,00771	2,52	5,74	0,00224	NM_003128	CD200
8081657	2,59	6,03	0,04016	-0,89	-1,85	0,00022	NM_001004196	HIATL1
8156538	2,59	6,02	0,01607	4,27	19,27	0,00039	NM_032558	CDC42BPA
7924773	2,59	6,02	0,00541	2,75	6,71	0,00799	NM_003607	TMEM87B
8044417	2,59	6,02	0,02238	2,35	5,09	0,00177	NM_032824	YKT6
8132580	2,59	6,02	0,00158	3,43	10,81	0,00374	NM_006555	RPL41
7957530	2,59	6,02	0,21461	-1,34	-2,53	0,00124	NM_021104	TGM2
8066214	2,59	6,01	0,00375	3,51	11,39	0,00031	NM_004613	SNORD116-1
7981949	2,59	6,01	0,01178	-2,00	-4,00	0,01432	NR_003316	TOMM20
7925174	2,59	6,01	0,04376	3,13	8,77	0,00468	NM_014765	TYW3
7902345	2,59	6,00	0,00083	1,76	3,40	0,00019	NM_138467	HGSNAT
8146285	2,59	6,00	0,00028	3,29	9,75	0,00004	NM_152419	PLAU
7928429	2,58	5,99	0,07589	4,78	27,57	0,00016	NM_002658	PLEKHA2
8145977	2,58	5,98	0,00170	0,78	1,72	0,09413	NM_021623	PLEKHM3
8058512	2,58	5,98	0,00250	3,22	9,29	0,00002	NM_001080475	SENP5
8084971	2,58	5,98	0,00730	3,94	15,36	0,00003	NM_152699	HTRA1
7931097	2,58	5,97	0,00211	1,12	2,18	0,00323	NM_002775	PLTP
8066619	2,58	5,97	0,00189	-1,03	-2,04	0,00207	NM_006227	PEPD
8036010	2,58	5,97	0,02433	3,42	10,72	0,00005	NM_000285	ACAT1

7943605	2,58	5,97	0,01916	3,01	8,07	0,00251	NM_000019	PTCD3
8043251	2,58	5,96	0,01403	3,19	9,13	0,00002	NM_017952	AAK1
8052798	2,58	5,96	0,00899	1,92	3,77	0,00061	NM_014911	GM2A
8109344	2,58	5,96	0,00760	2,58	5,99	0,00066	NM_000405	NUDT4P1
7919193	2,58	5,96	0,00427	3,48	11,13	0,00414	NR_002212	C20orf177
8063755	2,58	5,96	0,00419	2,41	5,30	0,00461	BC054002 SNX30	
8157253	2,58	5,96	0,00695	3,21	9,26	0,00022	NM_001012994	COX5A
7990436	2,58	5,96	0,00329	3,72	13,15	0,00172	NM_004255	C18orf32
8023246	2,57	5,96	0,00026	0,67	1,59	0,30630	BC093004 PELO	
8105229	2,57	5,95	0,00815	1,08	2,11	0,01532	NM_015946	---
7904967	2,57	5,95	0,00109	-0,76	-1,70	0,12622	--- CSF1	
7903786	2,57	5,95	0,00427	1,72	3,29	0,00400	NM_000757	OBFC2B
7956220	2,57	5,95	0,00170	2,66	6,31	0,00096	NM_024068	TBC1D2B
7990657	2,57	5,94	0,01379	2,96	7,77	0,00055	NM_144572	ID3
7913655	2,57	5,94	0,02707	2,38	5,21	0,00021	NM_002167	SCRN3
8046502	2,57	5,94	0,00093	2,29	4,89	0,00909	NM_024583	MED15
8071392	2,57	5,94	0,00877	4,30	19,72	0,00075	NM_001003891	CCBL2
7917482	2,57	5,94	0,00874	3,29	9,76	0,00113	NM_001008661	ADAM19
8115490	2,57	5,93	0,00623	0,71	1,64	0,13995	NM_033274	ANGPTL1
7922598	2,57	5,93	0,02632	-0,97	-1,96	0,02306	NM_004673	RPL3
8076209	2,57	5,92	0,04068	2,69	6,46	0,01286	NM_000967	C1orf144
7898328	2,57	5,92	0,01493	3,39	10,49	0,00069	NM_001114600	RPSA
7918050	2,57	5,92	0,02211	2,10	4,29	0,00027	NM_002295	AKR1A1
7901110	2,57	5,92	0,00523	3,99	15,85	0,00006	NM_006066	ITSN2
8050719	2,56	5,92	0,00149	2,84	7,18	0,01344	NM_006277	WDR36
8107282	2,56	5,92	0,00160	2,90	7,48	0,00199	NM_139281	EYA3
7914153	2,56	5,91	0,00040	1,74	3,35	0,00099	NM_001990	WDR43
8041149	2,56	5,91	0,02480	3,54	11,65	0,00044	NM_015131	ARHGAP18
8129458	2,56	5,91	0,01054	2,18	4,52	0,00057	NM_033515	BIRC6
8041283	2,56	5,91	0,00567	3,02	8,12	0,00680	NM_016252	SLMO2
8067295	2,56	5,91	0,00274	2,73	6,62	0,00016	NM_016045	MED28
8094271	2,56	5,90	0,11329	2,88	7,37	0,00000	NM_025205	NAGK
8042576	2,56	5,90	0,02095	3,55	11,73	0,00074	NM_017567	MMGT1
8175360	2,56	5,90	0,00283	3,06	8,34	0,00280	NM_173470	ZNF12
8138116	2,56	5,89	0,00059	1,88	3,68	0,00496	NM_016265	ZNF267
7995258	2,56	5,89	0,00347	2,07	4,20	0,03089	NM_003414	SIKE1
7918847	2,56	5,89	0,01099	2,66	6,31	0,00155	NM_001102396	MPP5
7975203	2,56	5,89	0,00163	2,96	7,80	0,00499	NM_022474	MRPS18B
8117955	2,56	5,89	0,00336	2,90	7,48	0,00855	NM_014046	MRPS18B
8177824	2,56	5,89	0,00336	2,90	7,48	0,00855	NM_014046	MRPS18B
8179139	2,56	5,89	0,00336	2,90	7,48	0,00855	NM_014046	FAM129A
7922846	2,56	5,89	0,00117	2,92	7,55	0,00029	NM_052966	SERP1
8091458	2,56	5,88	0,00736	3,09	8,52	0,00054	NM_014445	TMEM98
8006415	2,56	5,88	0,01992	2,69	6,46	0,00094	NM_015544	TERF1
8146914	2,56	5,88	0,02424	3,21	9,25	0,00619	NM_017489	CCDC117
8072153	2,55	5,88	0,00120	1,44	2,71	0,00384	NM_173510	ADAM23
8047788	2,55	5,87	0,00322	0,97	1,96	0,13391	NM_003812	ZHX1
8152656	2,55	5,87	0,00420	3,02	8,12	0,00286	NM_001017926	TOP1
8062603	2,55	5,87	0,00335	3,37	10,35	0,00081	NM_003286	RHEB
8143957	2,55	5,87	0,02373	2,69	6,45	0,00101	NM_005614	SMPD4
8055183	2,55	5,86	0,03426	4,05	16,55	0,00193	NM_017751	TAF13
7918284	2,55	5,86	0,00588	1,57	2,97	0,05705	NM_005645	RPS8
7901038	2,55	5,86	0,03355	2,39	5,23	0,00025	NM_001012	NME1
8008517	2,55	5,86	0,00511	4,06	16,64	0,00011	NM_198175	UBR4
7913001	2,55	5,86	0,00219	3,05	8,25	0,01236	NM_020765	UBP1
8086057	2,55	5,86	0,01939	2,51	5,71	0,00025	NM_014517	IPO11
8105545	2,55	5,86	0,00796	3,16	8,94	0,00396	NM_016338	DYNC2H1
7943442	2,55	5,86	0,01486	1,29	2,45	0,03943	NM_001377	MORC4
8174313	2,55	5,85	0,00504	1,48	2,78	0,02159	NM_024657	CD47
8089299	2,55	5,85	0,02701	3,74	13,33	0,00276	NM_001777	---
8106475	2,55	5,85	0,00668	1,13	2,19	0,02857	--- SGPL1	
7928171	2,55	5,85	0,00728	4,27	19,32	0,00002	NM_003901	HBXIP
7918437	2,55	5,85	0,00736	4,31	19,80	0,00004	NM_006402	SRPR
7952557	2,55	5,85	0,00685	3,03	8,15	0,00091	NM_003139	PSME1
7973564	2,55	5,84	0,00285	2,61	6,09	0,00004	NM_176783	CPT2
7916262	2,55	5,84	0,00375	4,56	23,57	0,00009	NM_000098	LEPR
7902074	2,55	5,84	0,01250	2,04	4,12	0,01035	NM_002303	MT1A
7995806	2,55	5,84	0,03765	3,29	9,81	0,00123	NM_005946	LDHB
7961693	2,55	5,84	0,01034	4,46	21,98	0,00220	NM_002300	ARL8A
7923394	2,55	5,84	0,00636	3,32	9,98	0,00001	NM_138795	PQLC3
8040278	2,54	5,83	0,00039	3,85	14,42	0,00004	NM_152391	MPP6
8131927	2,54	5,83	0,00943	2,85	7,20	0,00315	NM_016447	TMEM85
7982507	2,54	5,83	0,05825	4,06	16,71	0,00021	NM_016454	KLHDC2

7974229	2,54	5,83	0,02404	5,04	32,84	0,00037	NM_014315	ADAMTS5
8069689	2,54	5,82	0,00502	-1,10	-2,15	0,00022	NM_007038	GUSBP1
8104621	2,54	5,82	0,05430	1,69	3,22	0,05724	NR_027028	CTSD
7945666	2,54	5,81	0,00533	2,43	5,38	0,00378	NM_001909	ARHGAP5
7973840	2,54	5,81	0,00231	1,49	2,81	0,06977	NM_001030055	CENPN
7997381	2,54	5,81	0,00916	3,44	10,85	0,00043	NM_001100624	PFDN6
8118661	2,54	5,81	0,00889	4,08	16,90	0,00267	NM_014260	PFDN6
8178270	2,54	5,81	0,00889	4,08	16,90	0,00267	NM_014260	PFDN6
8179559	2,54	5,81	0,00889	4,08	16,90	0,00267	NM_014260	C8orf40
8146225	2,54	5,81	0,00644	3,54	11,67	0,00014	NM_001135674	CNPY3
8119609	2,54	5,80	0,00173	2,13	4,37	0,08418	NM_006586	EXOC1
8095163	2,54	5,80	0,00351	1,78	3,43	0,00257	NM_001024924	---
7915594	2,54	5,80	0,01237	1,73	3,32	0,00644	---	DNAJC18
8114526	2,54	5,80	0,00369	2,40	5,27	0,00224	NM_152686	GAPVD1
8157858	2,54	5,80	0,01379	3,63	12,35	0,00026	NM_015635	KBTBD2
8138922	2,54	5,80	0,00480	3,11	8,61	0,00004	NM_015483	OAZ1
8024436	2,54	5,80	0,01219	4,84	28,66	0,00447	NM_004152	C5orf23
8104758	2,53	5,80	0,08478	0,63	1,55	0,37858	BC022250	---
8106761	2,53	5,79	0,02050	-0,08	-1,06	0,88263	---	OSBPL9
7901385	2,53	5,79	0,03818	3,11	8,65	0,00052	NM_024586	CACHD1
7901993	2,53	5,79	0,00687	2,33	5,02	0,00065	NM_020925	ATG2B
7981217	2,53	5,79	0,00282	3,32	9,98	0,00959	NM_018036	C11orf54
7943162	2,53	5,78	0,00137	4,04	16,48	0,00030	NM_014039	PPP2R2A
8145440	2,53	5,78	0,01620	1,71	3,28	0,02264	NM_002717	COX6C
8151993	2,53	5,78	0,00429	1,69	3,22	0,02856	NM_004374	ZC3H14
7976101	2,53	5,78	0,00077	2,00	3,99	0,00252	NM_024824	TAX1BP1
8131975	2,53	5,78	0,00105	1,64	3,12	0,01774	NM_006024	PFDN1
8114567	2,53	5,77	0,09185	3,93	15,28	0,00035	NM_002622	KIAA0922
8097867	2,53	5,77	0,00242	2,20	4,58	0,00279	NM_001131007	BRD7
8001350	2,53	5,77	0,00287	2,14	4,42	0,00219	NM_001173984	DHRS7B
8005699	2,53	5,77	0,00025	2,64	6,25	0,00459	NM_015510	RPL36A
8138361	2,53	5,76	0,08338	2,59	6,03	0,00836	NM_021029	FBL
8036777	2,53	5,76	0,00537	3,68	12,81	0,00026	NM_001436	YAP1
7943398	2,53	5,76	0,01447	1,03	2,04	0,00579	NM_001130145	TRPS1
8152453	2,53	5,76	0,00790	-0,64	-1,56	0,01135	NM_014112	PRKAA2
7901720	2,52	5,76	0,01714	3,47	11,09	0,00099	NM_006252	FAM82B
8151644	2,52	5,75	0,02402	2,71	6,55	0,00035	NM_016033	NUPL1
7968132	2,52	5,75	0,00310	3,61	12,19	0,00011	NM_014089	EIF2B3
7915640	2,52	5,75	0,02374	2,92	7,55	0,00210	NM_020365	RNF111
7983953	2,52	5,75	0,02252	2,86	7,27	0,00866	NM_017610	SPRED2
8052689	2,52	5,74	0,00626	2,98	7,91	0,00233	NM_181784	PYROXD1
7954382	2,52	5,74	0,03547	0,77	1,71	0,24139	NM_024854	NEXN
7902495	2,52	5,74	0,00140	0,04	1,03	0,80997	NM_144573	ZNF195
7945864	2,52	5,74	0,01872	3,95	15,50	0,00003	NM_001130520	PNPLA8
8142307	2,52	5,73	0,02703	3,11	8,61	0,00022	NM_015723	EIF2AK2
8051501	2,52	5,73	0,02241	3,25	9,50	0,00074	NM_002759	GPI
8027621	2,52	5,72	0,04106	3,58	11,94	0,00030	NM_000175	NFIB
8160138	2,51	5,72	0,09441	0,25	1,19	0,52302	NM_005596	HELZ
8017776	2,51	5,71	0,01565	2,72	6,61	0,00000	NM_014877	ARL2
7941104	2,51	5,71	0,01007	2,75	6,71	0,01462	NM_001667	COG6
7968711	2,51	5,71	0,01367	2,13	4,37	0,00038	NM_020751	GCSH
8002999	2,51	5,71	0,01225	2,85	7,21	0,00498	NM_004483	C11orf59
7950119	2,51	5,71	0,04163	3,43	10,75	0,00000	NM_017907	NHP2
7929243	2,51	5,70	0,00042	1,98	3,94	0,00706	NM_017838	PPP1CA
7949765	2,51	5,70	0,00241	4,40	21,09	0,00253	NM_001008709	MAGT1
8173713	2,51	5,70	0,00197	2,77	6,84	0,00120	NM_032121	DEDD
7921793	2,51	5,70	0,01096	4,52	22,91	0,00003	NM_032998	PDE6D
8059731	2,51	5,70	0,04041	4,43	21,54	0,00010	NM_002601	STYXL1
8140386	2,51	5,69	0,00492	2,35	5,10	0,00378	NM_016086	SMG1
8000638	2,51	5,69	0,00733	3,06	8,35	0,01459	NM_015092	MEF2A
7986411	2,51	5,68	0,00580	1,43	2,70	0,02046	NM_005587	LNPEP
8107066	2,50	5,67	0,00509	2,33	5,04	0,02098	NM_005575	PUS7L
7962427	2,50	5,66	0,01262	1,52	2,86	0,01972	NM_031292	S100A16
7920291	2,50	5,66	0,00156	4,12	17,38	0,00003	NM_080388	ARL1
7965789	2,50	5,66	0,03564	2,24	4,73	0,00827	NM_001177	NID1
7925320	2,50	5,66	0,01284	1,90	3,73	0,00352	NM_002508	SON
8068289	2,50	5,66	0,00057	2,85	7,19	0,01758	NM_138927	RABL2B
8077171	2,50	5,65	0,01082	2,84	7,14	0,00094	NM_001130921	FAM32A
8026541	2,50	5,65	0,03056	2,13	4,37	0,02671	AF151902	RICTOR
8111698	2,50	5,65	0,02743	3,30	9,85	0,01149	NM_152756	SGK269
7990632	2,50	5,65	0,05428	2,92	7,58	0,01420	NM_024776	ATMIN
7997396	2,50	5,65	0,00125	2,23	4,69	0,05672	NM_015251	MFAP5
7960919	2,50	5,65	0,02607	-0,15	-1,11	0,78216	NM_003480	MYSM1

7916592	2,50	5,64	0,00495	2,60	6,05	0,00269	NM_001085487	TXNL4A
8023920	2,50	5,64	0,03103	2,48	5,57	0,02244	NM_006701	MFAP1
7988245	2,50	5,64	0,00058	1,81	3,51	0,00789	NM_005926	ADD1
8093643	2,49	5,64	0,00012	2,07	4,21	0,00060	NM_176801	BCAP31
8175835	2,49	5,63	0,02433	2,42	5,35	0,00080	NM_001139457	SMAP2
7900426	2,49	5,63	0,02394	1,50	2,84	0,02567	NM_022733	USP28
7951752	2,49	5,62	0,00699	2,95	7,73	0,00000	NM_020886	UBE2D1
7927658	2,49	5,62	0,00270	2,72	6,57	0,00105	NM_003338	ZMAT3
8092230	2,49	5,62	0,03369	2,24	4,73	0,00020	NM_022470	DPP8
7989806	2,49	5,62	0,00142	3,64	12,47	0,00044	NM_197960	ELOVL4
8127767	2,49	5,62	0,00063	1,58	2,99	0,01511	NM_022726	ADK
7928471	2,49	5,62	0,00489	3,58	11,98	0,00037	NM_006721	AHNAK
7948667	2,49	5,62	0,00302	1,02	2,03	0,11603	NM_001620	HIVEP2
8129953	2,49	5,61	0,00845	2,99	7,95	0,01277	NM_006734	---
8060738	2,49	5,61	0,01087	1,12	2,18	0,09322	---	RHOBTB1
7933821	2,49	5,61	0,04488	1,67	3,17	0,00334	NR_024556	SLC9A8
8063351	2,49	5,61	0,00770	2,35	5,11	0,00155	NM_015266	MT1DP
7995813	2,49	5,61	0,00782	2,03	4,09	0,00427	NR_027781	RBPJ
8094460	2,49	5,61	0,00541	3,48	11,13	0,00216	NM_005349	ALAS1
8080184	2,49	5,61	0,00627	3,35	10,18	0,00053	NM_000688	NUB1
8137414	2,49	5,60	0,00127	2,51	5,69	0,00023	NM_016118	VPS29
7966332	2,49	5,60	0,00895	2,02	4,06	0,00902	NM_016226	MYH9
8075728	2,49	5,60	0,00556	3,19	9,12	0,00167	NM_002473	FKBP7
8057045	2,49	5,60	0,00123	-0,25	-1,19	0,15966	NM_181342	SH3D19
8103166	2,48	5,60	0,02911	1,70	3,24	0,05184	NM_001009555	LOC728613
8110858	2,48	5,59	0,00230	2,12	4,35	0,01683	NR_003713	RTN4
8052204	2,48	5,59	0,00474	1,16	2,23	0,04622	NM_020532	PRKRA
8057034	2,48	5,59	0,00099	1,92	3,79	0,02352	NM_003690	MRPL46
7991209	2,48	5,59	0,03174	3,25	9,50	0,00166	NM_022163	ARFGEF2
8063242	2,48	5,59	0,00591	3,88	14,75	0,00011	NM_006420	GOPC
8129181	2,48	5,58	0,00594	2,41	5,31	0,00315	NM_020399	PIGT
8062981	2,48	5,58	0,01040	1,81	3,52	0,00479	NM_015937	---
8151540	2,48	5,58	0,08632	2,79	6,93	0,00417	---	FAM91A1
8148208	2,48	5,58	0,02242	4,63	24,83	0,00039	NM_144963	C10orf116
7928882	2,48	5,57	0,00350	-1,06	-2,08	0,19218	NM_006829	TTC3
8068522	2,48	5,57	0,02575	3,52	11,50	0,00079	NM_003316	ZDHHC7
8003180	2,48	5,57	0,00575	3,53	11,52	0,00041	NM_001145548	MRS2
8117194	2,48	5,57	0,00804	3,51	11,37	0,00078	NM_020662	RRAS
8038407	2,48	5,56	0,00812	3,19	9,10	0,00329	NM_006270	CROT
8133938	2,48	5,56	0,07603	1,14	2,21	0,02898	NM_001143935	SETD2
8086706	2,47	5,56	0,00898	3,81	14,02	0,00091	NM_014159	ELOVL2
8123920	2,47	5,56	0,00355	1,79	3,47	0,00035	NM_017770	TTC23
7991485	2,47	5,56	0,00072	1,37	2,59	0,01220	NM_001040655	CNTN4
8077323	2,47	5,55	0,02667	-1,48	-2,79	0,00292	NM_175607	VOPP1
7904969	2,47	5,55	0,00331	1,43	2,69	0,00040	NM_030796	PLA2G4A
7908351	2,47	5,55	0,00037	2,08	4,23	0,00867	NM_024420	SNORA8
7951034	2,47	5,55	0,00665	2,92	7,57	0,03865	NR_002920	SFRS3
8119080	2,47	5,55	0,02892	3,74	13,32	0,00067	NM_003017	KLHL20
7907445	2,47	5,54	0,03310	1,96	3,90	0,00014	NM_014458	ZNF100
8035808	2,47	5,54	0,00821	3,25	9,54	0,00162	NM_173531	IFRD1
8135514	2,47	5,54	0,00074	2,63	6,21	0,00588	NM_001550	CTDSP2
7964579	2,47	5,54	0,01209	2,49	5,62	0,02001	NM_005730	IGF2R
8123181	2,47	5,53	0,00411	2,43	5,37	0,00186	NM_000876	GOT2
8001764	2,47	5,53	0,00640	3,75	13,43	0,00029	NM_002080	SMG1
8000156	2,47	5,53	0,00596	3,18	9,04	0,00674	NM_015092	WDR82
8087874	2,47	5,53	0,02678	3,22	9,33	0,00311	NM_025222	AHI1
8129728	2,47	5,53	0,00490	1,82	3,54	0,00644	NM_001134831	GTF2I
8133504	2,47	5,53	0,04260	3,51	11,40	0,01355	NM_032999	CCT4
8052562	2,47	5,53	0,00464	3,75	13,50	0,00055	NM_006430	IGF1R
7986383	2,47	5,53	0,00942	3,57	11,91	0,00029	NM_000875	ATP6AP1
8170882	2,47	5,53	0,00174	3,15	8,85	0,00625	NM_001183	SCP2
7901513	2,46	5,52	0,03888	2,72	6,59	0,00043	NM_002979	EIF2C1
7900030	2,46	5,52	0,00587	2,64	6,25	0,00806	NM_012199	FLRT3
8065071	2,46	5,52	0,00959	1,71	3,27	0,01154	NM_198391	RBM27
8108927	2,46	5,52	0,00055	3,42	10,71	0,00041	NM_018989	NAA15
8097480	2,46	5,52	0,01795	2,27	4,83	0,00393	NM_057175	TXN2
8075778	2,46	5,51	0,02279	2,20	4,59	0,00797	NM_012473	AGPAT6
8146142	2,46	5,51	0,01704	3,28	9,71	0,00039	NM_178819	TSC1
8164781	2,46	5,51	0,00365	2,61	6,11	0,01911	NM_000368	ISCU
7958414	2,46	5,51	0,00636	2,83	7,11	0,00001	NM_014301	FBXO8
8103755	2,46	5,51	0,02462	1,45	2,73	0,00763	NM_012180	HLA-J
8117813	2,46	5,51	0,00730	3,61	12,21	0,00012	NR_024240	COG1
8009533	2,46	5,51	0,00053	1,81	3,51	0,00002	NM_018714	GBP3

7917503	2,46	5,50	0,00645	-0,74	-1,67	0,00636	NM_018284	ZFP62
8116494	2,46	5,50	0,01337	2,53	5,77	0,00021	NM_152283	DUSP18
8075423	2,46	5,50	0,00582	2,63	6,19	0,00192	NM_152511	DYNC2LI1
8041676	2,46	5,50	0,00084	1,51	2,85	0,03183	NM_016008	HNRNPD
8101324	2,46	5,50	0,01078	3,59	12,02	0,00041	NM_031370	LOC554202
8160431	2,46	5,50	0,01557	1,87	3,65	0,00824	NR_027054	RNASEK
8012000	2,46	5,50	0,00002	1,46	2,74	0,02291	NM_001004333	DCAF17
8046318	2,46	5,49	0,01896	4,03	16,39	0,00133	NR_028482	RPP21
8177782	2,46	5,49	0,11188	4,68	25,63	0,00002	NM_024839	RPP21
8179097	2,46	5,49	0,11188	4,68	25,63	0,00002	NM_024839	NPC1
8022531	2,46	5,49	0,02775	3,50	11,32	0,01737	NM_000271	TXLNG
8166230	2,46	5,48	0,02232	3,94	15,37	0,00044	NM_018360	SCYL3
7922250	2,46	5,48	0,02842	3,12	8,68	0,00015	NM_181093	PLD1
8092134	2,45	5,48	0,00265	2,73	6,63	0,00623	NM_002662	FLII
8013191	2,45	5,48	0,00821	3,80	13,89	0,00066	NM_002018	NHLRC2
7930614	2,45	5,48	0,00166	3,37	10,32	0,00764	NM_198514	PDP1
8147344	2,45	5,48	0,06391	4,30	19,67	0,00429	NM_001161778	GNAI2
8079950	2,45	5,47	0,00827	2,70	6,48	0,00021	NM_002070	BOD1
8115865	2,45	5,47	0,00012	1,33	2,51	0,11395	NM_138369	PRKAR1A
8009457	2,45	5,46	0,00632	2,12	4,34	0,01125	NM_212472	SSBP2
8112940	2,45	5,45	0,00983	-0,75	-1,68	0,04464	NM_012446	DDX56
8139392	2,45	5,45	0,00375	3,34	10,13	0,00097	NM_019082	TIMM17A
7908766	2,45	5,45	0,00909	3,19	9,11	0,00079	NM_006335	RBX1
8073334	2,44	5,44	0,01921	4,61	24,50	0,00001	NM_014248	CTNS
8003824	2,44	5,44	0,01118	3,07	8,41	0,00005	NM_004937	ACTL6A
8084067	2,44	5,44	0,01915	2,99	7,97	0,00071	NM_178042	ASNSD1
8046997	2,44	5,44	0,01651	2,65	6,28	0,00142	NM_019048	RABGAP1
8157700	2,44	5,44	0,01222	3,39	10,51	0,00003	NM_012197	FBXO28
7909992	2,44	5,44	0,00679	2,52	5,73	0,00066	NM_015176	FPGT
7902308	2,44	5,44	0,03853	1,97	3,93	0,00337	NM_003838	VAPA
8020129	2,44	5,44	0,01775	2,89	7,42	0,00059	NM_003574	C16orf87
8001178	2,44	5,44	0,00011	1,49	2,81	0,00954	BC056676 ZNF561	
8033795	2,44	5,43	0,00854	1,15	2,22	0,03355	NM_152289	TRHDE
7957221	2,44	5,43	0,02094	-0,91	-1,87	0,00039	NM_013381	KRTCAP2
7920659	2,44	5,43	0,03730	2,36	5,15	0,00927	NM_173852	INTS9
8150014	2,44	5,43	0,00968	2,48	5,57	0,00454	NM_018250	DIMT1L
8112312	2,44	5,43	0,00951	3,24	9,47	0,00030	NM_014473	ANXA11
7934733	2,44	5,42	0,00880	2,05	4,13	0,00105	NM_145869	PPHLN1
7954940	2,44	5,42	0,00233	2,17	4,51	0,00204	NM_016488	FBXL2
8078461	2,44	5,42	0,00368	2,47	5,53	0,00027	NM_012157	MOBKL1A
8095566	2,44	5,42	0,01351	2,63	6,18	0,00087	NM_173468	MTDH
8147483	2,44	5,42	0,00171	2,39	5,24	0,02032	NM_178812	WARS2
7919038	2,44	5,42	0,02861	2,04	4,11	0,01507	NM_201263	ZNF570
8028248	2,44	5,42	0,01133	2,05	4,14	0,01045	NM_144694	RPL36A
7946807	2,44	5,41	0,08752	1,25	2,38	0,05552	NM_021029	CREB3L1
7939642	2,44	5,41	0,02597	0,10	1,07	0,32035	NM_052854	DYRK1A
8068551	2,44	5,41	0,01595	2,74	6,66	0,01785	NM_101395	ADAMTS1
8069676	2,44	5,41	0,01473	-1,47	-2,77	0,00361	NM_006988	NIPBL
8104944	2,44	5,41	0,00978	3,09	8,50	0,01660	NM_015384	EEF1G
7948679	2,44	5,41	0,02244	2,36	5,15	0,05888	NM_001404	TMEM14A
8120239	2,43	5,39	0,05005	3,00	7,98	0,00012	NM_014051	SEC13
8085300	2,43	5,39	0,00069	1,54	2,91	0,09852	NR_024272	WDR44
8169519	2,43	5,39	0,00075	1,81	3,50	0,00374	NM_019045	RAB2A
8146564	2,43	5,39	0,00248	2,46	5,49	0,00602	NM_002865	RPRD2
7905185	2,43	5,39	0,00672	3,95	15,48	0,00820	NM_015203	ADHFE1
8146687	2,43	5,38	0,02717	-0,18	-1,14	0,18707	NM_144650	ZNF429
8027312	2,43	5,38	0,02572	1,01	2,01	0,12157	NM_001001415	STOML2
8160968	2,43	5,38	0,00106	3,73	13,27	0,00073	NM_013442	TTC39C
8020630	2,43	5,38	0,01174	3,29	9,80	0,00008	NM_153211	NMI
8055702	2,43	5,38	0,01123	0,43	1,35	0,13120	NM_004688	TMEM126A
7942889	2,43	5,38	0,00256	1,47	2,76	0,02143	NM_032273	MRPL39
8069620	2,43	5,38	0,02647	3,90	14,97	0,00003	NM_080794	ZNF107
8133049	2,43	5,37	0,00212	1,67	3,18	0,00798	NM_016220	FBXL5
8099449	2,43	5,37	0,02046	4,58	23,92	0,00020	NM_033535	SOCS5
8041820	2,42	5,37	0,00927	1,08	2,11	0,01086	NM_014011	SRP9
7935002	2,42	5,37	0,00138	2,39	5,26	0,00118	NM_003133	RALY
8061966	2,42	5,34	0,01171	1,91	3,76	0,00325	NM_016732	SLC4A1AP
8041015	2,42	5,34	0,00414	3,25	9,51	0,00259	NM_018158	ZFYVE16
8106602	2,42	5,34	0,00446	1,79	3,45	0,00747	NM_014733	SYVN1
7949383	2,42	5,34	0,00574	2,83	7,13	0,00019	NM_032431	FAM179B
7974125	2,42	5,34	0,00487	2,18	4,53	0,00051	NM_015091	THOC4
8019772	2,42	5,33	0,01101	3,65	12,58	0,00036	NM_005782	RPS26
8154363	2,41	5,33	0,05994	0,44	1,36	0,64775	NM_001029	SNX17

8040908	2,41	5,33	0,01678	4,15	17,77	0,00027	NM_014748	PCF11
7942839	2,41	5,33	0,00342	2,68	6,40	0,00158	NM_015885	HNRNPL
8036613	2,41	5,33	0,02925	4,95	30,93	0,00112	NM_001533	LY6E
8148572	2,41	5,33	0,00046	2,63	6,19	0,00822	NM_002346	RAD50
8107942	2,41	5,33	0,00207	1,65	3,14	0,00680	NM_005732	CXorf26
8168447	2,41	5,32	0,00643	2,31	4,96	0,01214	BC001220 FAM175A	
8101489	2,41	5,32	0,02348	0,52	1,44	0,14098	NM_139076	KLHDC10
8136163	2,41	5,32	0,00516	3,51	11,38	0,00358	NM_014997	PTPN11
7958846	2,41	5,32	0,01586	2,66	6,32	0,00136	NM_002834	FGFR1
8150318	2,41	5,32	0,00161	0,68	1,60	0,00177	NM_023110	HNRNPA1
7916562	2,41	5,32	0,10454	1,38	2,60	0,00223	NM_002136	RDX
7951554	2,41	5,32	0,01643	2,92	7,56	0,00006	NM_002906	SLC15A4
7967624	2,41	5,31	0,02629	3,68	12,85	0,00003	NM_145648	---
8176253	2,41	5,31	0,03851	2,35	5,10	0,03256	--- ZNF92	
8133089	2,41	5,31	0,03284	3,60	12,13	0,00023	NM_152626	RNASEN
8111286	2,41	5,31	0,00443	3,54	11,65	0,00048	NM_013235	CDC73
7908437	2,41	5,31	0,03495	2,93	7,63	0,00047	NM_024529	CCDC25
8149942	2,41	5,31	0,00470	2,44	5,42	0,00102	NM_018246	ZFP36L2
8051814	2,41	5,31	0,01629	1,28	2,43	0,09412	NM_006887	C2orf29
8043937	2,41	5,30	0,00133	3,09	8,50	0,00260	NM_017546	CCDC59
7965200	2,41	5,30	0,01021	2,50	5,65	0,01001	NR_033192	EIF5A2
8092073	2,41	5,30	0,00655	-0,16	-1,11	0,60387	NM_020390	USP38
8097570	2,40	5,29	0,00371	2,91	7,53	0,00580	NM_032557	---
8172028	2,40	5,29	0,01293	2,99	7,93	0,00024	--- ATAD1	
7934870	2,40	5,29	0,01980	2,22	4,64	0,00786	NM_032810	PSMD14
8045946	2,40	5,29	0,01587	2,66	6,30	0,00158	NM_005805	POLR1D
7968254	2,40	5,29	0,02275	1,92	3,78	0,01349	NM_152705	JMJD1C
7933877	2,40	5,28	0,00810	3,16	8,92	0,01226	NM_004241	SACM1L
8079346	2,40	5,28	0,01543	2,27	4,82	0,00010	NM_014016	RNF216L
8131286	2,40	5,28	0,00035	2,92	7,56	0,00103	NR_023384	SELT
8083352	2,40	5,28	0,12099	5,15	35,42	0,00067	NM_016275	FIGN
8056323	2,40	5,28	0,00621	3,13	8,75	0,00017	NM_018086	OSGIN2
8147221	2,40	5,27	0,00145	2,57	5,93	0,00481	NM_004337	POLK
8106303	2,40	5,27	0,00548	1,70	3,25	0,00411	NM_016218	HLCS
8070239	2,40	5,27	0,01138	3,33	10,03	0,00693	NM_000411	SS18
8022625	2,40	5,26	0,00197	1,87	3,66	0,00495	NM_001007559	C19orf10
8032863	2,40	5,26	0,00506	1,42	2,68	0,03024	NM_019107	FKTN
8157074	2,40	5,26	0,02072	4,00	15,99	0,00010	NM_001079802	KIRREL
7906307	2,39	5,26	0,00152	2,46	5,49	0,00303	NM_018240	ANAPC1
8053610	2,39	5,25	0,04875	3,24	9,43	0,00001	NM_022662	PRELID1
8110318	2,39	5,25	0,02665	1,97	3,91	0,00431	NM_013237	ATXN7
8080878	2,39	5,25	0,00423	3,22	9,29	0,00045	NM_000333	ACIN1
7973371	2,39	5,24	0,01014	3,09	8,50	0,00040	NM_001164814	TARDBP
7897663	2,39	5,24	0,02657	3,16	8,95	0,00009	NM_007375	SMARCC2
7963988	2,39	5,24	0,00031	3,23	9,36	0,00098	NM_003075	UBA1
8167069	2,39	5,24	0,00058	3,09	8,54	0,00156	NM_003334	TLN1
8161056	2,39	5,24	0,01077	2,11	4,32	0,01202	NM_006289	SEMA5A
8110932	2,39	5,24	0,01416	-1,32	-2,49	0,08679	NM_003966	FAM18B2
8005501	2,39	5,24	0,00735	2,13	4,39	0,00027	NM_001135036	14. Sep
7911329	2,39	5,24	0,11015	1,60	3,03	0,35347	NM_207366	PWP1
7958379	2,39	5,23	0,00656	2,83	7,12	0,00050	NM_007062	ACLY
8015460	2,39	5,23	0,01353	3,58	11,94	0,00054	NM_001096	IFT80
8091737	2,39	5,23	0,02821	3,23	9,35	0,00086	NM_020800	GATAD2B
7920341	2,39	5,23	0,00145	4,56	23,61	0,00142	NM_020699	SIDT2
7944049	2,39	5,23	0,00966	2,04	4,11	0,00975	NM_001040455	MRPL30
8043848	2,39	5,22	0,01166	2,33	5,05	0,00267	NM_145212	ZFP36
8028652	2,38	5,22	0,01403	1,52	2,87	0,09568	NM_003407	COX6A1
8119153	2,38	5,22	0,00105	2,60	6,06	0,00060	NM_004373	C2orf43
8050608	2,38	5,22	0,00481	3,20	9,20	0,00106	BC017473 YBX1	
7900585	2,38	5,22	0,03637	0,68	1,60	0,05773	NM_004559	PALLD
8098263	2,38	5,22	0,00297	2,16	4,46	0,01160	NM_001166108	YIPF1
7916343	2,38	5,22	0,00766	1,87	3,65	0,00054	NM_018982	CSTF1
8063484	2,38	5,21	0,00325	3,75	13,49	0,00100	NM_001033522	PLAG1
8150881	2,38	5,21	0,03297	4,16	17,82	0,00004	NM_002655	USP24
7916443	2,38	5,21	0,00671	3,79	13,81	0,00152	NM_015306	BLOC1S1
7956013	2,38	5,21	0,02636	1,84	3,57	0,00010	NM_001487	SLC2A10
8063177	2,38	5,21	0,00602	1,15	2,22	0,03165	NM_030777	FBXO10
8161229	2,38	5,21	0,01796	3,65	12,54	0,00008	NM_012166	MED1
8014841	2,38	5,21	0,02385	3,88	14,76	0,00038	NM_004774	TMEM133
7943369	2,38	5,21	0,00261	2,56	5,90	0,00299	NM_032021	CHURC1
7975113	2,38	5,20	0,01879	3,09	8,51	0,01043	NM_145165	OXCT1
8111892	2,38	5,20	0,01642	3,22	9,32	0,00011	NM_000436	WNK1
7952953	2,38	5,20	0,00040	2,44	5,42	0,01610	NM_018979	VAPB

8063620	2,38	5,20	0,01144	2,87	7,33	0,00003	NM_004738	XBP1
8075182	2,38	5,20	0,00747	2,37	5,15	0,00876	NM_005080	IARS2
7909815	2,38	5,20	0,00605	3,24	9,44	0,00253	NM_018060	BACH1
8068105	2,38	5,20	0,01766	1,56	2,94	0,01086	NR_027655	HSD17B4
8107532	2,38	5,19	0,04671	3,56	11,78	0,00005	NM_000414	TBC1D7
8123961	2,38	5,19	0,00320	2,57	5,93	0,00025	NM_016495	RBM16
8122909	2,38	5,19	0,00384	2,20	4,59	0,00347	NM_014892	SPRY4
8114797	2,38	5,19	0,03881	0,81	1,76	0,04392	NM_030964	FTO
7995655	2,38	5,19	0,01568	2,57	5,93	0,00004	NM_001080432	TRIB1
8148304	2,37	5,19	0,01006	2,76	6,76	0,00020	NM_025195	RPF1
7902634	2,37	5,19	0,00329	1,92	3,78	0,04348	NM_025065	DCBLD1
8121685	2,37	5,19	0,00995	2,11	4,32	0,00027	NM_173674	LRRC16A
8117243	2,37	5,19	0,00165	2,74	6,67	0,00161	NM_017640	LITAF
7999468	2,37	5,18	0,02549	2,85	7,20	0,00284	NM_004862	FAM92A2
7982876	2,37	5,18	0,01346	0,82	1,76	0,48756	ENST00000287660	SUCLG1
8053366	2,37	5,18	0,02071	2,22	4,66	0,00119	NM_003849	STX8
8012581	2,37	5,17	0,00908	1,70	3,26	0,01864	NM_004853	C8orf59
8151587	2,37	5,17	0,01533	1,77	3,41	0,00762	NM_001099670	CTBS
7917240	2,37	5,17	0,03777	2,66	6,31	0,00139	NM_004388	VPS25
8007355	2,37	5,17	0,00502	3,20	9,19	0,00005	NM_032353	TRIO
8104506	2,37	5,17	0,00279	3,23	9,37	0,00011	NM_007118	SLC30A5
8105801	2,37	5,16	0,00223	2,92	7,58	0,00016	NM_022902	IMPAD1
8150908	2,37	5,16	0,01038	3,04	8,24	0,00057	NM_017813	RCOR3
7909529	2,37	5,16	0,00358	2,26	4,78	0,00247	NM_001136223	YEATS2
8084232	2,37	5,16	0,01388	3,36	10,30	0,00015	NM_018023	BAZ1A
7978595	2,37	5,16	0,00027	3,23	9,40	0,01310	NM_013448	C9orf78
8164596	2,37	5,15	0,00482	2,69	6,46	0,00107	NM_016520	CAPN7
8078110	2,36	5,15	0,00488	3,13	8,76	0,01271	NM_014296	GALNT2
7910427	2,36	5,15	0,03300	2,45	5,48	0,01378	NM_004481	IVNS1ABP
7922889	2,36	5,14	0,02432	3,83	14,22	0,00079	NM_006469	SLC1A1
8154135	2,36	5,14	0,04513	1,14	2,21	0,07830	NM_004170	C5orf32
8108478	2,36	5,14	0,01510	2,94	7,69	0,00100	NM_032412	SRP68
8018600	2,36	5,13	0,00595	3,35	10,22	0,00008	NM_014230	ATP6V0A1
8007228	2,36	5,13	0,02733	4,43	21,62	0,00072	NM_001130020	ING4
7960654	2,36	5,12	0,00131	2,51	5,71	0,00039	NM_016162	TDP1
7976160	2,36	5,12	0,00747	3,58	11,93	0,00098	NM_018319	RPL26L1
7945740	2,36	5,12	0,00329	1,08	2,12	0,03790	NM_016093	ANXA7
7934367	2,36	5,12	0,02596	3,12	8,69	0,00305	NM_004034	LONP2
7995421	2,36	5,12	0,01153	2,05	4,13	0,00132	NM_031490	PURA
8108472	2,36	5,12	0,01064	2,82	7,05	0,00079	NM_005859	DNAJC21
8104838	2,36	5,12	0,00829	1,97	3,91	0,00674	NM_194283	FARP1
7969736	2,35	5,11	0,00560	1,95	3,87	0,00007	NM_005766	C6orf145
8123678	2,35	5,11	0,00490	3,92	15,09	0,00004	NM_183373	MON2
7956697	2,35	5,11	0,01968	2,38	5,21	0,00683	NM_015026	PDE7B
8122222	2,35	5,11	0,01237	-0,96	-1,95	0,00570	NM_018945	EIF4G3
7913319	2,35	5,11	0,00656	2,08	4,23	0,00009	NM_003760	PTBP2
7903188	2,35	5,11	0,00201	2,13	4,37	0,00658	NM_021190	UBE2E1
8078262	2,35	5,11	0,00814	2,88	7,36	0,00011	NM_003341	TCF21
8122176	2,35	5,11	0,02217	-1,35	-2,54	0,01787	NM_003206	G2E3
7973756	2,35	5,10	0,01675	3,13	8,77	0,00093	NM_017769	MAP3K12
7963670	2,35	5,10	0,00789	1,76	3,38	0,00056	NM_006301	WASF2
7914094	2,35	5,10	0,04795	3,67	12,72	0,00414	NM_006990	SPOCK1
8114287	2,35	5,10	0,01427	-1,04	-2,06	0,06449	NM_004598	RNF181
8043209	2,35	5,10	0,00691	0,83	1,78	0,05265	NM_016494	OS9
7956593	2,35	5,10	0,00643	3,59	12,01	0,00021	NM_006812	ZBTB33
8169683	2,35	5,10	0,04634	2,26	4,80	0,00464	NM_006777	C16orf62
7993680	2,35	5,10	0,03108	3,09	8,51	0,00002	BC050464 NBEAL1	
8047606	2,35	5,10	0,00187	2,97	7,86	0,00011	NM_001114132	SNRPA
8028916	2,35	5,09	0,00267	2,60	6,06	0,00006	NM_004596	WDR1
8099340	2,35	5,09	0,00544	3,52	11,51	0,00033	NM_017491	TEX10
8162850	2,35	5,09	0,00355	2,43	5,37	0,00161	NM_017746	FBXW2
8163784	2,35	5,09	0,00738	3,57	11,84	0,00311	NM_012164	PRUNE2
8161865	2,35	5,09	0,05364	-0,39	-1,31	0,15247	NM_015225	EIF6
8065868	2,35	5,09	0,01249	2,68	6,42	0,00036	NM_002212	HSD11B1
7909446	2,35	5,08	0,03170	-0,68	-1,60	0,02711	NM_005525	MBD2
8023382	2,35	5,08	0,01005	1,41	2,66	0,02573	NM_003927	ADAM17
8050190	2,35	5,08	0,01389	4,01	16,07	0,00021	NM_003183	RDH11
7979743	2,35	5,08	0,01368	3,58	11,95	0,00016	NM_016026	ABCC1
7993478	2,34	5,08	0,00339	3,43	10,79	0,00150	NM_004996	PFDN5
7955768	2,34	5,08	0,01223	2,56	5,90	0,00200	NM_002624	HIPK1
7904137	2,34	5,07	0,00220	2,75	6,73	0,00821	NM_198268	H3F3A
7910124	2,34	5,07	0,02995	2,07	4,19	0,00015	NM_002107	LHFPL2
8112803	2,34	5,07	0,00399	1,34	2,52	0,00023	NM_005779	LOC441722

7982529	2,34	5,07	0,00276	1,56	2,96	0,01480	ENST00000454093	TTPAL
8062844	2,34	5,07	0,01170	3,07	8,38	0,00004	NM_024331	FXR1
8084146	2,34	5,07	0,01149	2,65	6,28	0,00097	NM_001013439	PGAP2
7937892	2,34	5,07	0,01601	5,08	33,74	0,00002	NR_027015	TANK
8045933	2,34	5,06	0,00815	1,17	2,25	0,01105	NM_004180	ZNFX1
8066905	2,34	5,06	0,00115	3,45	10,92	0,00013	NM_021035	POLH
8119858	2,34	5,06	0,01374	2,51	5,69	0,00017	NM_006502	PSMA5
7918345	2,34	5,06	0,00335	1,99	3,98	0,00534	NM_002790	RASGRF2
8106660	2,34	5,06	0,00369	-0,16	-1,12	0,69890	NM_006909	IBTK
8127787	2,34	5,06	0,01874	3,19	9,13	0,00288	NM_015525	MTA2
7948696	2,34	5,06	0,00715	3,58	11,95	0,00271	NM_004739	CCNB1
8105828	2,34	5,06	0,02698	3,25	9,54	0,00628	NM_031966	SLC39A8
8101992	2,34	5,05	0,00042	2,97	7,82	0,00325	NM_022154	ZFPM2
8147837	2,34	5,05	0,00231	-0,22	-1,17	0,52731	NM_012082	TNS3
8139500	2,34	5,05	0,00372	2,37	5,17	0,00015	NM_022748	SP1
7955787	2,34	5,05	0,04126	3,98	15,74	0,00005	NM_138473	RPL15
8116929	2,33	5,04	0,08150	3,65	12,54	0,00002	NM_002948	RAB4A
7910398	2,33	5,04	0,00741	2,89	7,40	0,00020	NM_004578	CLIP4
8041179	2,33	5,04	0,01179	3,67	12,70	0,00010	NM_024692	PTGES3
7964250	2,33	5,03	0,02661	4,36	20,60	0,00036	NM_006601	NCAPD2
7953351	2,33	5,03	0,00177	3,47	11,10	0,00093	NM_014865	C10orf18
7925978	2,33	5,03	0,00991	3,51	11,36	0,00646	NM_017782	ZBTB11
8089234	2,33	5,03	0,00386	2,37	5,18	0,00840	NM_014415	TSPAN5
8101828	2,33	5,03	0,07199	3,07	8,41	0,00031	NM_005723	C13orf23
7971134	2,33	5,03	0,00789	3,28	9,69	0,01082	NM_025138	ZNF415
8039044	2,33	5,02	0,00913	-0,79	-1,73	0,00355	NR_028343	TMEM177
8044844	2,33	5,02	0,08710	2,73	6,62	0,00252	NM_001105198	SLMAP
8080685	2,33	5,02	0,00892	2,30	4,92	0,00059	NM_007159	ZRANB1
7931226	2,33	5,02	0,02256	3,03	8,19	0,00143	NM_017580	PPAPDC1B
8150276	2,33	5,02	0,00527	2,19	4,56	0,01586	NM_001102559	EIF4B
8135268	2,33	5,02	0,00326	1,70	3,25	0,21867	NM_001417	ZNF652
8016546	2,33	5,02	0,03319	2,11	4,33	0,00008	NM_014897	EML1
7976698	2,33	5,01	0,02247	1,17	2,25	0,01539	NM_001008707	NCBP2
8093156	2,33	5,01	0,05542	3,28	9,72	0,00036	NM_007362	IDH3A
7985134	2,33	5,01	0,01568	4,53	23,10	0,00082	NM_005530	HINT2
8161147	2,33	5,01	0,01435	3,31	9,94	0,00016	NM_032593	GBAP1
7920687	2,33	5,01	0,02147	2,48	5,59	0,00472	NR_002188	GOSR1
8006148	2,32	5,01	0,00525	3,28	9,73	0,00183	NM_004871	PIR
8171435	2,32	5,01	0,01781	2,53	5,79	0,00320	NM_003662	TSC22D2
8083324	2,32	5,01	0,00051	1,25	2,37	0,13731	NM_014779	CSTB
8070701	2,32	5,01	0,02129	2,33	5,04	0,01045	NM_000100	PIKFYVE
8047865	2,32	5,00	0,00336	2,28	4,85	0,02018	NM_015040	CELF1
7947894	2,32	5,00	0,02982	2,87	7,32	0,00343	NM_001172639	ASTE1
8090664	2,32	5,00	0,01887	1,80	3,48	0,00220	NM_014065	GTPBP10
8134079	2,32	5,00	0,00608	3,60	12,13	0,00059	NM_033107	TOPBP1
8090772	2,32	5,00	0,00620	3,42	10,73	0,00144	NM_007027	PSMD5
8163795	2,32	4,99	0,00640	3,09	8,49	0,00194	NM_005047	PLXNB2
8076998	2,32	4,98	0,00250	2,31	4,96	0,00901	NM_012401	ATP6V1H
8150797	2,32	4,98	0,00807	1,92	3,79	0,00253	NM_015941	OSBPL10
8085984	2,31	4,97	0,00204	2,47	5,53	0,00605	NM_017784	BMPR1A
7952305	2,31	4,97	0,01836	4,10	17,09	0,00134	NM_004329	C11orf30
7942650	2,31	4,97	0,01273	2,86	7,24	0,00801	NM_020193	KIAA1143
8086498	2,31	4,97	0,00410	1,81	3,51	0,00850	NM_020696	NOL9
7912086	2,31	4,96	0,00792	2,05	4,15	0,02428	NM_024654	GSTM3
7918379	2,31	4,96	0,01740	1,17	2,25	0,01094	NM_000849	RPA2
7914141	2,31	4,95	0,01758	3,13	8,78	0,00005	NM_002946	MRPL17
7946267	2,31	4,95	0,02662	2,93	7,60	0,01431	NM_022061	SLC43A3
7948229	2,31	4,95	0,00666	1,64	3,13	0,00306	NM_017611	MFSD5
7955729	2,31	4,94	0,02141	3,48	11,14	0,00005	NM_001170790	---
8122198	2,31	4,94	0,30409	-0,68	-1,60	0,28259	--- SELM	
8075462	2,30	4,94	0,04288	0,63	1,55	0,27948	NM_080430	TSPAN4
7937518	2,30	4,94	0,00504	2,41	5,32	0,00510	NM_001025237	SUSD1
8163383	2,30	4,94	0,00842	3,63	12,36	0,00001	NM_022486	RNF41
7964021	2,30	4,94	0,00429	2,92	7,56	0,00085	NM_194358	SLC2A12
8129666	2,30	4,93	0,00135	0,99	1,99	0,00797	NM_145176	EIF5B
8043861	2,30	4,93	0,00284	1,48	2,79	0,08708	NM_015904	TIPRL
7907124	2,30	4,93	0,01363	2,81	7,01	0,00072	NM_152902	PRKAB2
7919305	2,30	4,92	0,02314	2,88	7,37	0,00003	NM_005399	TCEB3
7898881	2,30	4,92	0,00271	2,90	7,46	0,00001	NM_003198	ACTB
8137979	2,30	4,91	0,04507	1,54	2,90	0,00071	NM_001101	RCBTB1
7971602	2,30	4,91	0,01621	2,55	5,84	0,00244	NM_018191	VPS37A
8144774	2,30	4,91	0,01001	0,79	1,73	0,13989	NM_152415	DMXL2
7988789	2,30	4,91	0,00447	3,24	9,42	0,01688	NM_001174116	STAT2

7964119	2,30	4,91	0,04589	2,39	5,24	0,00244	NM_005419	NIPAL3
7898939	2,29	4,91	0,00904	2,61	6,10	0,00021	NM_020448	TEX261
8052925	2,29	4,90	0,00566	2,97	7,86	0,00141	NM_144582	RPS27A
8042052	2,29	4,90	0,00191	1,64	3,12	0,00526	NM_002954	TAF1B
8040173	2,29	4,90	0,03334	3,30	9,86	0,00055	NM_005680	ASCC1
7934228	2,29	4,90	0,00445	1,81	3,52	0,00767	NM_015947	CCDC109A
7928318	2,29	4,90	0,01787	3,20	9,18	0,00685	NM_138357	SNX4
8090256	2,29	4,89	0,00627	2,81	7,02	0,00461	NM_003794	EI24
7944978	2,29	4,89	0,01030	5,06	33,34	0,00109	NM_004879	POLR2D
8055089	2,29	4,89	0,02334	2,08	4,23	0,00181	NM_004805	ATG7
8077858	2,29	4,89	0,00083	1,42	2,68	0,00066	NM_006395	ABHD4
7973306	2,29	4,89	0,01395	4,19	18,27	0,00124	NM_022060	COX7A2L
8051777	2,29	4,89	0,00685	1,25	2,37	0,02620	NM_004718	UTRN
8122464	2,29	4,89	0,00099	1,67	3,19	0,03225	NM_007124	ACTR8
8088151	2,29	4,89	0,00302	2,91	7,53	0,00006	NM_022899	ABCF2
8143905	2,29	4,89	0,03652	1,83	3,55	0,00585	NM_007189	PROS1
8089011	2,29	4,88	0,02363	1,90	3,72	0,01327	NM_000313	BTN3A1
8117458	2,29	4,88	0,00029	2,04	4,12	0,00028	NM_001145009	GMPPA
8048595	2,29	4,88	0,01043	2,46	5,51	0,00129	NM_013335	AMMECR1
8174496	2,29	4,88	0,01255	1,52	2,87	0,00216	NM_015365	MRPS14
7922462	2,29	4,88	0,06335	2,71	6,54	0,00347	NM_022100	MT1F
7995825	2,29	4,88	0,01767	4,38	20,81	0,00014	NM_005949	NCOA1
8040552	2,29	4,87	0,01209	2,59	6,01	0,00211	NM_147223	USP15
7956670	2,29	4,87	0,00563	2,40	5,26	0,00871	NM_006313	ACTR1A
7936028	2,28	4,87	0,00686	2,19	4,56	0,00171	NM_005736	TRIM24
8136473	2,28	4,87	0,00116	1,64	3,11	0,06927	NM_015905	ERAL1
8005994	2,28	4,87	0,04666	3,71	13,10	0,00039	NM_005702	MED27
8112890	2,28	4,87	0,00736	1,79	3,45	0,00579	NM_004269	TOM1L2
8013159	2,28	4,87	0,01856	2,60	6,07	0,00018	NM_001082968	TCEA1
8078600	2,28	4,86	0,00633	3,69	12,90	0,00379	NM_006756	SMN1
8106006	2,28	4,86	0,00032	2,90	7,45	0,00015	NM_000344	LASS5
7963174	2,28	4,86	0,03210	4,07	16,82	0,00004	NM_147190	RCN1
7947358	2,28	4,86	0,01375	1,50	2,82	0,02077	NM_002901	SOAT1
7907702	2,28	4,86	0,00910	2,85	7,23	0,00011	NM_003101	PSMD13
7937287	2,28	4,85	0,03419	2,36	5,12	0,00176	NM_002817	AUH
8162264	2,28	4,85	0,00128	2,70	6,49	0,00004	NM_001698	CCDC90A
8124008	2,28	4,85	0,00712	2,31	4,96	0,00348	NM_001031713	ABCF1
8117929	2,28	4,85	0,01768	2,49	5,63	0,00035	NM_001025091	ABCF1
8177797	2,28	4,85	0,01768	2,49	5,63	0,00035	NM_001025091	ABCF1
8179112	2,28	4,85	0,01768	2,49	5,63	0,00035	NM_001025091	TAF5L
7924969	2,28	4,85	0,01319	2,98	7,89	0,00664	NM_014409	PHF6
8169969	2,28	4,85	0,00695	2,75	6,72	0,00057	NM_032458	ZNF766
8030899	2,28	4,84	0,01023	1,92	3,79	0,01747	NM_001010851	BBS10
7965060	2,28	4,84	0,00938	1,82	3,52	0,04058	NM_024685	---
7991835	2,28	4,84	0,21024	0,14	1,10	0,86524	--- MACF1	
7900235	2,27	4,84	0,00713	2,42	5,34	0,00423	NM_012090	MT1G
8001531	2,27	4,83	0,02984	1,72	3,30	0,00352	NM_005950	CAB39L
7971590	2,27	4,83	0,00121	0,63	1,54	0,24237	NM_030925	DAP
8110982	2,27	4,83	0,00807	2,98	7,87	0,00153	NM_004394	UEVLD
7947027	2,27	4,83	0,02906	2,54	5,83	0,00106	NM_001040697	POM121C
8140297	2,27	4,83	0,00181	3,10	8,56	0,00055	NM_001099415	INHBA
8139207	2,27	4,82	0,01637	-0,68	-1,60	0,02456	NM_002192	SMPD1
7938100	2,27	4,82	0,00659	0,72	1,64	0,02930	NM_000543	C5orf30
8107194	2,27	4,82	0,01371	3,13	8,75	0,00104	BC009203 RPL21	
7999360	2,27	4,82	0,07025	0,67	1,59	0,12941	NM_000982	NBR1
8007471	2,27	4,82	0,05299	3,45	10,96	0,00220	NM_031858	SLC5A3
8068361	2,27	4,81	0,00871	3,89	14,78	0,00034	NM_006933	SDC2
8147461	2,27	4,81	0,04423	1,35	2,55	0,00002	NM_002998	RPS27L
7989493	2,27	4,81	0,02666	1,22	2,33	0,08645	NM_015920	KDM1A
7898833	2,27	4,81	0,00375	3,74	13,37	0,00047	NM_001009999	NUP107
7956949	2,26	4,80	0,00645	3,11	8,65	0,00035	NM_020401	SNORD13
8145793	2,26	4,80	0,20420	-2,01	-4,03	0,44537	NR_003041	UBE2D4
8132523	2,26	4,80	0,02816	2,75	6,75	0,00264	NM_015983	ITGA4
8046695	2,26	4,80	0,00897	-0,51	-1,42	0,00315	NM_000885	FHL2
8054377	2,26	4,80	0,02976	2,62	6,15	0,00925	NM_201555	INPP5A
7931479	2,26	4,80	0,02407	2,49	5,63	0,00026	NM_005539	SSX2IP
7917255	2,26	4,79	0,01326	1,57	2,97	0,01915	NM_014021	SYT14
7909494	2,26	4,79	0,01624	-1,24	-2,36	0,01000	NR_027458	HSPA13
8069532	2,26	4,79	0,04336	3,65	12,57	0,00005	NM_006948	FLJ44606
8113796	2,26	4,79	0,00884	3,07	8,42	0,00659	NM_001164478	CTNNAL1
8163063	2,26	4,78	0,00380	3,17	9,01	0,00026	NM_003798	LPIN1
8040340	2,26	4,78	0,00457	2,91	7,53	0,00010	NM_145693	PARP8
8105191	2,26	4,78	0,00037	2,45	5,46	0,00122	NM_001178055	DDX46

8108134	2,26	4,78	0,00126	2,43	5,37	0,00031	NM_014829	KCTD21
7950669	2,26	4,78	0,03755	1,39	2,62	0,02011	NM_001029859	ALG13
8169412	2,26	4,78	0,04203	2,48	5,59	0,01598	NM_018466	ATF6B
8125295	2,26	4,78	0,01950	3,28	9,71	0,00026	NM_004381	ACSS3
7957386	2,26	4,77	0,01489	-0,87	-1,82	0,04195	NM_024560	LRRC1
8120279	2,25	4,77	0,01887	2,29	4,90	0,01744	NM_018214	TMEM117
7954985	2,25	4,77	0,06038	3,40	10,54	0,00028	NM_032256	ALDH1B1
8155327	2,25	4,77	0,01032	1,54	2,91	0,02461	NM_000692	KGFLP1
8067839	2,25	4,76	0,00009	-0,18	-1,13	0,66070	NR_003674	PIGP
8070257	2,25	4,76	0,00734	2,01	4,02	0,00387	NR_028352	TMEM69
7901135	2,25	4,76	0,01395	1,70	3,25	0,02843	NM_016486	SMC3
7930422	2,25	4,76	0,00369	3,62	12,32	0,00199	NM_005445	ADAT1
8002904	2,25	4,76	0,01498	3,69	12,87	0,00001	NM_012091	SERPINA3
7976496	2,25	4,76	0,00230	-0,77	-1,70	0,05602	NM_001085	ANG
7973084	2,25	4,75	0,02196	0,96	1,94	0,01184	NM_001145	MRPS7
8009784	2,25	4,75	0,00485	2,81	7,01	0,00453	NM_015971	ARPP19
7988963	2,25	4,75	0,00775	2,26	4,80	0,02822	NM_006628	FLNA
8176026	2,25	4,74	0,01778	3,10	8,57	0,00824	NM_001456	CAMLG
8108127	2,25	4,74	0,01205	2,50	5,64	0,00033	NM_001745	AASDHPPT
7943552	2,24	4,74	0,01329	2,22	4,65	0,01323	NM_015423	MYO10
8111153	2,24	4,74	0,00294	2,83	7,11	0,00000	NM_012334	DNAJC16
7898192	2,24	4,74	0,01590	2,86	7,28	0,00001	NM_015291	FAR1
7938592	2,24	4,73	0,00253	2,26	4,80	0,00115	NM_032228	STK16
8048505	2,24	4,73	0,05217	1,52	2,87	0,01165	NR_026909	MAN1A1
8129254	2,24	4,73	0,01343	-0,45	-1,36	0,17505	NM_005907	CHEK1
7945014	2,24	4,73	0,02442	3,45	10,96	0,00129	NM_001274	FAM49A
8050427	2,24	4,72	0,00650	-0,75	-1,68	0,02730	NM_030797	GAPDH
7953385	2,24	4,72	0,20242	1,14	2,20	0,00289	NM_002046	VTI1B
7979732	2,24	4,72	0,03092	3,59	12,03	0,00229	NM_006370	KIF20A
8108301	2,24	4,72	0,01028	3,54	11,64	0,00032	NM_005733	CCDC45
8009255	2,24	4,71	0,00430	2,73	6,61	0,02981	NM_138363	CDC5L
8120022	2,24	4,71	0,00151	2,07	4,20	0,02796	NM_001253	AAK1
8052803	2,24	4,71	0,00269	1,62	3,08	0,00364	NM_014911	MUT
8126860	2,24	4,71	0,00475	3,30	9,85	0,00092	NM_000255	ZNF595
8093320	2,24	4,71	0,00961	1,44	2,71	0,03352	NM_182524	ZEB2
8055624	2,23	4,71	0,00227	-0,58	-1,49	0,28959	NM_014795	BTN3A3
8117476	2,23	4,70	0,01495	1,42	2,67	0,07393	NM_006994	PION
8140468	2,23	4,70	0,06614	2,31	4,97	0,00831	NM_017439	STARD7
8053890	2,23	4,70	0,03061	3,48	11,12	0,00002	NM_020151	RRN3P1
8000192	2,23	4,70	0,01415	3,48	11,15	0,00041	NR_003370	DARS
8055445	2,23	4,70	0,02125	3,20	9,18	0,00184	NM_001349	ST5
7946401	2,23	4,70	0,03593	0,44	1,35	0,05056	NM_005418	CDKN1A
8119088	2,23	4,70	0,00071	1,37	2,59	0,01126	NM_078467	CYP4V2
8098637	2,23	4,70	0,00496	2,01	4,04	0,01984	NM_207352	SNRPN
7981919	2,23	4,70	0,02413	2,18	4,53	0,00292	NM_022807	RHOJ
7974902	2,23	4,70	0,02997	-1,47	-2,76	0,00008	NM_020663	CLCN3
8098291	2,23	4,69	0,00610	2,39	5,25	0,00001	NM_173872	LCLAT1
8041212	2,23	4,69	0,01880	3,32	9,99	0,00003	NM_182551	FKBP15
8163452	2,23	4,69	0,00971	3,01	8,07	0,00109	NM_015258	KIAA1586
8120378	2,23	4,69	0,04157	0,39	1,31	0,02644	NM_020931	CCNA1
7968637	2,23	4,69	0,01067	1,95	3,87	0,00017	NM_003914	ATXN1
8124040	2,23	4,69	0,00534	2,92	7,55	0,00104	NM_000332	UGGT2
7972369	2,23	4,69	0,01583	1,89	3,72	0,04046	NM_020121	MRPS15
7914940	2,23	4,69	0,03509	4,34	20,31	0,00048	NM_031280	C18orf8
8020527	2,23	4,69	0,00554	3,02	8,12	0,00002	NM_013326	GNPDA1
8114787	2,23	4,69	0,01117	1,14	2,20	0,02106	NM_005471	LASS6
8046086	2,23	4,68	0,00477	3,88	14,69	0,00090	NM_203463	STX2
7967685	2,23	4,68	0,00363	2,68	6,40	0,00352	NM_194356	RASSF3
7956819	2,23	4,68	0,02038	3,63	12,34	0,00002	NM_178169	GNE
8161174	2,23	4,68	0,01806	3,03	8,17	0,00178	NM_005476	RPL10
8109821	2,23	4,68	0,11236	4,46	21,93	0,00039	NM_006013	TNKS
8144528	2,23	4,68	0,08499	3,20	9,18	0,00014	NM_003747	LYPLA1
8150830	2,23	4,68	0,02365	5,21	36,91	0,00273	NM_006330	PHAX
8107691	2,22	4,67	0,03711	2,08	4,24	0,01111	NM_032177	DOCK10
8059413	2,22	4,67	0,00590	2,29	4,90	0,00219	NM_014689	KIAA1370
7988970	2,22	4,67	0,01884	1,58	2,98	0,01598	NM_019600	HSPA5
8164165	2,22	4,66	0,00384	2,51	5,71	0,00095	NM_005347	CLASP2
8086077	2,22	4,66	0,00779	3,30	9,84	0,00017	NM_015097	MFN1
8084045	2,22	4,66	0,01639	3,38	10,42	0,00275	NM_033540	PGM2L1
7950391	2,22	4,66	0,00960	1,50	2,82	0,02263	NM_173582	SH3PXD2B
8115814	2,22	4,66	0,00421	2,02	4,05	0,00044	NM_001017995	CAPNS1
8028172	2,22	4,65	0,02870	3,51	11,42	0,00088	NM_001749	STS
8165866	2,22	4,65	0,01141	1,51	2,85	0,00182	NM_000351	RUFY3

8095545	2,22	4,65	0,00130	1,18	2,26	0,00100	NM_001037442	DHX40
8008825	2,22	4,65	0,01211	3,19	9,10	0,00207	NM_024612	LZTFL1
8086555	2,22	4,65	0,02206	1,05	2,06	0,06709	NM_020347	NDUFV3
8068857	2,22	4,64	0,01658	2,69	6,45	0,01510	NM_021075	HHLA3
7902282	2,22	4,64	0,06220	3,23	9,41	0,00026	NM_001036645	ASPA
8003814	2,21	4,64	0,04934	-0,92	-1,89	0,03418	NM_000049	FAT1
8104079	2,21	4,64	0,00929	1,25	2,38	0,00967	NM_005245	DNAJC8
7899422	2,21	4,64	0,02593	0,56	1,47	0,47539	NM_014280	PECI
8123695	2,21	4,64	0,01999	3,04	8,24	0,00077	NR_028588	TTC35
8147864	2,21	4,64	0,00942	2,65	6,26	0,00000	NM_014673	LSP1
7905028	2,21	4,64	0,07055	2,70	6,52	0,00103	NM_002339	TRIM38
8117321	2,21	4,64	0,02883	2,38	5,20	0,00093	NM_006355	PPP1R14B // PPP1R14B // PPP1R14B
7949060	2,21	4,63	0,01627	2,19	4,57	0,00089	NM_138689 // NM_138689 // NM_138689	STARD4
8113491	2,21	4,63	0,01352	1,71	3,28	0,00701	NM_139164	RWDD4A
8139198	2,21	4,63	0,00119	2,07	4,19	0,00139	NM_152682	NSUN3
8081128	2,21	4,63	0,01039	2,20	4,59	0,00085	NM_022072	SESTD1
8057394	2,21	4,63	0,03036	1,90	3,73	0,00066	NM_178123	C20orf24
8062326	2,21	4,63	0,01030	3,24	9,44	0,00739	NR_026562	RC3H1
7922432	2,21	4,63	0,01075	2,69	6,46	0,00007	NM_172071	FXYD5
8027778	2,21	4,63	0,02350	0,77	1,71	0,22754	NR_028406	ZNF93
8027247	2,21	4,62	0,02459	1,72	3,29	0,02094	NM_031218	TMEM189
8066964	2,21	4,62	0,01279	3,54	11,60	0,00020	NM_199129	ZNF281
7923183	2,21	4,62	0,02535	1,94	3,83	0,00112	NM_012482	NSMCE1
8000413	2,21	4,62	0,03165	1,40	2,64	0,00283	NM_145080	AP1M1
8026548	2,21	4,62	0,01966	2,89	7,41	0,00167	NM_001130524	EIF2S2
8065730	2,21	4,62	0,00076	2,45	5,45	0,01033	NM_003908	TP53BP2
7924526	2,21	4,62	0,00540	2,37	5,18	0,00595	NM_005426	CLIC1
8179827	2,21	4,62	0,01304	2,73	6,62	0,00055	NM_001288	WDR45
8172538	2,21	4,62	0,01295	2,25	4,75	0,04218	NM_007075	C8orf84
8151369	2,21	4,61	0,04766	-1,69	-3,22	0,01678	NM_153225	CALM2
8052010	2,21	4,61	0,00412	1,27	2,41	0,04838	NM_001743	EIF5
7977058	2,20	4,61	0,03848	4,71	26,21	0,00048	NM_001969	ZNF507
8027439	2,20	4,61	0,01127	2,74	6,66	0,00303	NM_001136156	MAP3K8
7926900	2,20	4,61	0,00790	0,57	1,49	0,36386	NM_005204	PLA2R1
8056151	2,20	4,60	0,00913	0,43	1,35	0,13216	NM_007366	NRP1
7932985	2,20	4,60	0,00030	1,85	3,60	0,00875	NM_003873	YWHAG
8140398	2,20	4,60	0,00656	2,18	4,53	0,00814	NM_012479	PHKB
7995382	2,20	4,60	0,01267	3,36	10,26	0,00414	NM_001031835	PTPN1
8063394	2,20	4,60	0,03466	2,10	4,29	0,00128	NM_002827	SLC25A46
8107259	2,20	4,60	0,03463	1,85	3,60	0,01431	NM_138773	ETFDH
8098084	2,20	4,60	0,03072	1,05	2,07	0,00250	NM_004453	HARS
8114628	2,20	4,60	0,02747	2,87	7,33	0,00074	NM_002109	SUV420H1
7949931	2,20	4,59	0,02153	2,31	4,97	0,00628	NM_017635	---
8092532	2,20	4,59	0,03473	1,65	3,13	0,05707	---	RIOK3
8020508	2,20	4,59	0,00531	1,96	3,89	0,00128	NM_003831	RWDD1
8121613	2,20	4,58	0,00553	1,40	2,64	0,03459	NM_016104	NTAN1
7999598	2,20	4,58	0,05045	1,40	2,64	0,00721	NM_173474	GPR180
7969626	2,20	4,58	0,03809	2,63	6,17	0,00022	NM_180989	RER1
7897078	2,20	4,58	0,00874	2,60	6,07	0,00009	NM_007033	PDLIM5
8096489	2,20	4,58	0,00951	2,92	7,56	0,00001	NM_006457	USP32
8017212	2,20	4,58	0,04745	3,19	9,16	0,00078	NM_032582	ACAP2
8092933	2,19	4,58	0,00185	2,63	6,20	0,00557	NM_012287	UBXN4
8045455	2,19	4,58	0,00205	2,82	7,05	0,00339	NM_014607	TMEM222
7899310	2,19	4,58	0,04551	2,57	5,94	0,00021	NM_032125	ZDHHC9
8175023	2,19	4,57	0,00839	4,46	22,02	0,00005	NM_016032	LOC100132288
8071044	2,19	4,57	0,00851	2,49	5,62	0,00042	NM_001033515	POLR2H
8084488	2,19	4,57	0,02676	6,00	63,87	0,00003	NM_006232	SGTA
8032530	2,19	4,57	0,00540	2,75	6,75	0,00494	NM_003021	RNF19A
8152041	2,19	4,57	0,00519	3,37	10,36	0,00005	NM_183419	---
8136159	2,19	4,57	0,00220	0,65	1,56	0,25345	---	PKD1
7999614	2,19	4,56	0,01544	3,40	10,55	0,00008	NM_001009944	PPP1R2
8092959	2,19	4,56	0,00927	2,99	7,95	0,00316	NM_006241	ACOX1
8018558	2,19	4,56	0,01331	2,92	7,56	0,00363	NM_004035	FAM55C
8081375	2,19	4,56	0,02014	1,81	3,51	0,00224	NM_145037	RABGEF1
8133176	2,19	4,56	0,07482	1,68	3,20	0,00339	NM_014504	MED10
8110886	2,19	4,56	0,01053	3,69	12,94	0,00110	NM_032286	NDUFA3
8031097	2,19	4,56	0,02856	1,25	2,37	0,01054	NM_004542	PLN
8121729	2,19	4,56	0,00012	-0,71	-1,64	0,05360	NM_002667	ENOX2
8175102	2,19	4,56	0,04317	3,10	8,57	0,00076	NM_182314	PRDX5
7940996	2,19	4,56	0,02851	3,58	11,93	0,00003	NM_012094	TUBA1C
7955179	2,19	4,56	0,03279	3,21	9,28	0,00248	NM_032704	TBC1D23

8081256	2,19	4,55	0,01102	2,29	4,91	0,00223	NM_018309	KDM3A
8043283	2,19	4,55	0,01769	2,44	5,42	0,00040	NM_018433	FMO4
7907297	2,19	4,55	0,00705	1,74	3,35	0,00627	NM_002022	EXT2
7939524	2,19	4,55	0,02143	1,77	3,40	0,00576	NM_000401	SUPV3L1
7927981	2,19	4,55	0,00452	3,16	8,94	0,00035	NM_003171	POC1B
7965343	2,19	4,55	0,01597	2,92	7,58	0,00174	NM_172240	PPP1R11
8117834	2,18	4,55	0,02864	3,90	14,90	0,00068	NM_021959	PPP1R11
8177744	2,18	4,55	0,02864	3,90	14,90	0,00068	NM_021959	R3HDM2
7964413	2,18	4,55	0,01064	3,13	8,73	0,01089	NM_014925	DMXL1
8107474	2,18	4,55	0,00920	2,95	7,72	0,00153	NM_005509	ELOVL1
7915504	2,18	4,55	0,01600	2,56	5,88	0,00028	NM_022821	SF3B5
8130003	2,18	4,54	0,02490	2,50	5,64	0,00091	NM_031287	EAPP
7978558	2,18	4,54	0,02312	3,94	15,36	0,00163	NM_018453	PPP1R7
8049784	2,18	4,54	0,00054	1,89	3,72	0,00057	NM_002712	PPWD1
8105633	2,18	4,54	0,00660	1,92	3,78	0,00503	NM_015342	HSPD1
8058052	2,18	4,54	0,00112	1,76	3,40	0,09151	NM_002156	NEDD4
7989094	2,18	4,54	0,01992	4,45	21,87	0,00022	NM_006154	ZBTB39
7964300	2,18	4,54	0,00179	2,06	4,18	0,00423	NM_014830	DDX60L
8103601	2,18	4,53	0,03183	1,76	3,39	0,00833	NM_001012967	C2orf47
8047265	2,18	4,53	0,00315	2,13	4,38	0,00190	NM_024520	NR1H4
7957835	2,18	4,53	0,00547	-0,67	-1,59	0,03787	NM_005123	KIAA1737
7975926	2,18	4,53	0,00291	2,73	6,63	0,00809	NM_033426	PDLIM3
8104022	2,18	4,53	0,01383	0,00	-1,00	0,97299	NM_014476	UTP6
8014081	2,18	4,53	0,00778	2,79	6,90	0,00033	NM_018428	PRPF4
8157283	2,18	4,53	0,01487	3,00	8,00	0,00173	NM_004697	CTSO
8103389	2,18	4,52	0,01944	-0,38	-1,30	0,22910	NM_001334	POLR2J3
8141795	2,18	4,52	0,00102	1,28	2,42	0,01841	NM_001097615	DPY19L4
8147375	2,18	4,52	0,02633	3,21	9,27	0,00475	NM_181787	---
7908635	2,18	4,52	0,00197	1,42	2,68	0,10933	--- MAT2B	
8109732	2,18	4,52	0,00306	2,38	5,21	0,00313	NM_013283	MRPL16
7948424	2,17	4,51	0,00111	2,20	4,60	0,00994	NM_017840	RCOR1
7977003	2,17	4,51	0,02174	2,61	6,10	0,01239	NM_015156	SLC4A7
8085914	2,17	4,51	0,00565	2,32	4,98	0,00061	NM_003615	CYB5R1
7923516	2,17	4,51	0,03039	1,76	3,38	0,00699	NM_016243	SRP9
7910099	2,17	4,51	0,00093	1,29	2,44	0,00702	NM_001130440	SMC4
8083709	2,17	4,51	0,02008	1,52	2,87	0,09050	NM_005496	NFYB
7965956	2,17	4,51	0,00769	1,24	2,37	0,01119	NM_006166	RSRC1
8083605	2,17	4,51	0,02560	2,07	4,19	0,00286	NM_016625	TRA2A
8138581	2,17	4,50	0,02431	3,20	9,17	0,00306	NM_013293	OTUB1
7940840	2,17	4,50	0,07352	3,23	9,37	0,00059	NM_017670	FMNL2
8045736	2,17	4,50	0,01434	3,09	8,53	0,00063	NM_052905	RNF34
7959282	2,17	4,50	0,04246	3,76	13,53	0,00035	NM_194271	VPS24
8053562	2,17	4,50	0,00363	1,59	3,01	0,00123	NM_016079	PPPDE1
7911078	2,17	4,49	0,01886	1,96	3,88	0,00536	NM_016076	TCF7L2
7930537	2,17	4,49	0,01487	2,62	6,16	0,01274	NM_001146274	UBE4B
7897527	2,17	4,49	0,00377	2,40	5,28	0,00151	NM_001105562	HSPC159
8042283	2,17	4,49	0,05035	3,72	13,21	0,00000	NM_014181	PDHA1
8166335	2,17	4,49	0,01095	3,09	8,49	0,00012	NM_000284	SMARCD1
7955331	2,17	4,49	0,01347	3,74	13,32	0,00010	NM_003076	DERA
7954185	2,17	4,49	0,04265	1,56	2,95	0,00231	NM_015954	TLR4
8157524	2,16	4,48	0,00323	-1,41	-2,66	0,02286	NR_024168	TAPBPL
7953341	2,16	4,48	0,01714	2,34	5,07	0,01259	NM_018009	NOL11
8009366	2,16	4,48	0,01006	2,11	4,33	0,04363	NM_015462	MYL12A
8019924	2,16	4,48	0,00176	0,64	1,56	0,08715	NM_006471	FXC1
7938111	2,16	4,48	0,03191	2,44	5,43	0,00175	NM_012192	CLTA
8155203	2,16	4,48	0,00072	0,99	1,98	0,05843	NM_007096	C3orf55
8083599	2,16	4,47	0,00233	2,20	4,60	0,00028	NM_001130002	PARP14
8082100	2,16	4,47	0,00581	2,15	4,45	0,00879	NM_017554	TRIM13
7969153	2,16	4,47	0,01223	1,83	3,56	0,04351	NM_213590	ZDHHC16
7929634	2,16	4,47	0,04286	4,31	19,86	0,00003	NM_198046	TERF1
8102789	2,16	4,47	0,00724	1,21	2,31	0,04953	NM_003218	VRK3
8038515	2,16	4,47	0,03194	2,67	6,37	0,00034	NM_016440	TBC1D20
8064336	2,16	4,47	0,00090	3,59	12,01	0,00100	NM_144628	SLC9A7
8172280	2,16	4,46	0,00921	2,29	4,87	0,00022	NM_032591	ITCH
8061986	2,16	4,46	0,00230	2,02	4,06	0,01199	NM_031483	C12orf23
7958346	2,16	4,46	0,00244	1,53	2,89	0,02543	NM_152261	BTF3L4
8069561	2,16	4,46	0,02651	3,83	14,24	0,00001	NM_152265	BTBD1
7991057	2,16	4,46	0,01540	3,75	13,46	0,00049	NM_025238	GPBP1L1
7915758	2,16	4,46	0,00245	2,61	6,11	0,00097	NM_021639	IGF2BP2
8092552	2,16	4,45	0,05311	2,51	5,71	0,00017	NM_006548	RRAGC
7915160	2,15	4,45	0,03478	3,44	10,84	0,00000	NM_022157	RPP30
7929116	2,15	4,45	0,01060	2,97	7,84	0,00034	NM_006413	SNORA50
8001748	2,15	4,45	0,04057	2,21	4,64	0,13224	NR_002980	EXOC2

8123524	2,15	4,45	0,00075	2,51	5,70	0,00423	NM_018303	RAN
7959882	2,15	4,45	0,00813	2,24	4,72	0,01869	NM_006325	SLC44A1
8157038	2,15	4,45	0,01226	3,35	10,17	0,00013	NM_080546	SURF1
8164896	2,15	4,45	0,00711	1,72	3,29	0,01527	NM_003172	SERINC5
8112865	2,15	4,45	0,04533	2,68	6,43	0,00002	NM_178276	PSMD6
8088535	2,15	4,44	0,00422	0,84	1,79	0,05309	NM_014814	PRR16
8107563	2,15	4,44	0,00843	-0,32	-1,24	0,37357	NM_016644	MEF2C
8113039	2,15	4,44	0,04844	0,44	1,35	0,26141	NM_002397	DHRS7
7979473	2,15	4,44	0,02271	3,12	8,72	0,00021	NM_016029	SPON1
7938608	2,15	4,44	0,04275	-1,05	-2,06	0,10165	NM_006108	TNKS2
7929168	2,15	4,44	0,00165	2,92	7,58	0,00315	NM_025235	TTL
8044462	2,15	4,43	0,00673	2,50	5,67	0,00001	NM_153712	COX10
8005089	2,15	4,43	0,00992	1,95	3,87	0,00270	NM_001303	SNORD116-23
7981994	2,15	4,43	0,05979	-1,90	-3,73	0,25395	NR_003337	IPP
7915775	2,15	4,43	0,01494	1,75	3,36	0,00366	NM_005897	JUN
7916609	2,15	4,43	0,00335	1,73	3,32	0,00773	NM_002228	GNAI3
7903703	2,15	4,43	0,01392	1,76	3,40	0,00292	NM_006496	ZNF260
8036324	2,15	4,42	0,00936	3,45	10,92	0,00075	NM_001012756	ARNTL
7938563	2,15	4,42	0,00915	3,07	8,41	0,00011	NM_001178	ZNF362
7899870	2,15	4,42	0,00289	3,05	8,31	0,00222	NM_152493	CTNND1
7940079	2,15	4,42	0,02428	2,68	6,40	0,00038	NM_001085458	FKBP14
8138834	2,14	4,42	0,06100	3,34	10,11	0,00022	NM_017946	ARIH2
8079662	2,14	4,42	0,02736	3,57	11,90	0,00023	NM_006321	---
7964832	2,14	4,42	0,01431	1,49	2,82	0,02669	--- RAB22A	
8063607	2,14	4,42	0,01062	2,38	5,19	0,00002	NM_020673	RAB28
8099395	2,14	4,42	0,07211	3,74	13,37	0,00098	NM_004249	BAMBI
7926875	2,14	4,42	0,00938	0,11	1,08	0,72838	NM_012342	PARP1
7924733	2,14	4,42	0,04187	3,70	13,00	0,00005	NM_001618	PRPF18
7926283	2,14	4,41	0,02380	1,66	3,16	0,01934	NM_003675	SNORA73A
7899480	2,14	4,41	0,08351	-0,70	-1,63	0,58484	NR_002907	SHQ1
8088830	2,14	4,41	0,00199	1,96	3,89	0,04539	NM_018130	SETD8
8053733	2,14	4,41	0,07170	3,54	11,67	0,00051	NM_020382	RPL23AP82
8074157	2,14	4,41	0,00533	1,08	2,11	0,05200	NR_026982	ABI1
7932616	2,14	4,41	0,00936	2,27	4,81	0,00367	NM_005470	RBM5
8079869	2,14	4,40	0,02225	1,41	2,66	0,02858	NM_005778	C17orf49
8004247	2,14	4,40	0,05020	4,63	24,69	0,00030	NM_001142798	SORT1
7918323	2,14	4,40	0,01461	3,42	10,73	0,00004	NM_002959	VPS53
8010924	2,14	4,40	0,03065	2,81	7,00	0,00087	NM_001128159	ST3GAL1
8153021	2,14	4,40	0,05462	0,54	1,45	0,13383	NM_003033	MPDU1
8004521	2,14	4,40	0,00160	2,50	5,66	0,03915	NM_004870	NEK6
8157761	2,14	4,39	0,00843	2,76	6,77	0,00013	NM_001145001	ZNF468
8038998	2,14	4,39	0,00018	3,07	8,42	0,00050	NM_199132	MLL
7944223	2,14	4,39	0,01641	2,43	5,40	0,00034	NM_005933	E2F5
8147101	2,13	4,39	0,04926	4,32	19,92	0,00012	NM_001951	CLTB
8115918	2,13	4,39	0,01549	1,63	3,09	0,00148	NM_007097	IRAK4
7954969	2,13	4,39	0,01102	2,34	5,07	0,00152	NM_001114182	FIP1L1
8095048	2,13	4,38	0,01756	2,72	6,60	0,00116	NM_030917	PPP4R1
8022251	2,13	4,38	0,03228	4,56	23,57	0,00001	NM_001042388	OGN
8162373	2,13	4,38	0,00266	-1,04	-2,06	0,00417	NM_033014	TMEM45A
8081288	2,13	4,38	0,08051	0,41	1,33	0,18815	NM_018004	SERBP1
7916836	2,13	4,37	0,08733	3,08	8,46	0,00163	NM_001018067	LDLR
8025828	2,13	4,37	0,00255	3,42	10,71	0,00033	NM_000527	CEBPD
8150592	2,13	4,37	0,00378	1,95	3,87	0,01446	NM_005195	MTRR
8104422	2,13	4,37	0,02221	4,67	25,49	0,00047	NM_002454	NDUFS2
7906703	2,13	4,37	0,02300	3,80	13,97	0,00137	NM_004550	C9orf41
8161829	2,13	4,37	0,00225	2,11	4,33	0,00259	BC034033 PLSCR1	
8091327	2,13	4,37	0,00805	1,24	2,36	0,03018	NM_021105	RPL17
7903582	2,12	4,36	0,00283	1,28	2,42	0,00353	NM_001035006	ADM
7938390	2,12	4,36	0,03550	0,88	1,84	0,25951	NM_001124	MTUS1
8149500	2,12	4,36	0,00081	2,02	4,05	0,01077	NM_001001924	INTS3
7905631	2,12	4,36	0,00243	3,84	14,33	0,00015	NM_023015	DBI
8044804	2,12	4,36	0,00909	1,85	3,61	0,05319	NM_020548	TH1L
8063697	2,12	4,35	0,01106	3,79	13,85	0,00002	NM_198976	MAP2K4
8005029	2,12	4,35	0,02198	2,71	6,52	0,00054	NM_003010	SNORD57
8060503	2,12	4,35	0,06958	-2,69	-6,45	0,20697	NR_002738	TPCN1
7958960	2,12	4,35	0,00459	4,09	16,99	0,00001	NM_001143819	RIOK2
8113286	2,12	4,35	0,02215	2,00	4,01	0,00116	NM_018343	SFRS6
8062695	2,12	4,35	0,02227	2,54	5,81	0,03864	NM_006275	PCOTH
7968029	2,12	4,35	0,01303	-0,68	-1,61	0,00268	NM_001014442	PIGS
8013641	2,12	4,34	0,01824	3,87	14,67	0,00007	NM_033198	GABPB1
7988687	2,12	4,34	0,01395	3,02	8,10	0,00000	NM_005254	TRIM37
8017106	2,12	4,34	0,01997	3,95	15,51	0,00796	NM_015294	FAM96B
8001924	2,12	4,34	0,00649	3,36	10,24	0,00169	NR_024525	RCAN1

8070182	2,12	4,34	0,00145	0,36	1,28	0,21624	NM_004414	TMEM56
7903162	2,12	4,33	0,02788	2,69	6,47	0,00061	NM_152487	C16orf80
8001658	2,12	4,33	0,02531	4,97	31,32	0,00008	NM_013242	TYMS
8019842	2,12	4,33	0,00346	3,86	14,49	0,00008	NM_001071	SPG11
7988286	2,11	4,33	0,00468	2,46	5,52	0,00475	NM_025137	DOPEY1
8120883	2,11	4,32	0,00078	0,92	1,89	0,04096	NM_015018	---
8094874	2,11	4,32	0,00740	1,45	2,73	0,06813	--- ZNF473	
8030569	2,11	4,32	0,00709	2,42	5,36	0,00029	NM_015428	CLIC1
8178598	2,11	4,32	0,02233	2,47	5,53	0,00134	NM_001288	HRSP12
8151942	2,11	4,32	0,02714	2,83	7,13	0,00079	NM_005836	HSP90AB3P
8096296	2,11	4,32	0,04885	3,04	8,25	0,01167	AY956764	EIF3J
7983350	2,11	4,32	0,00637	3,00	7,97	0,00637	NM_003758	BLZF1
7907171	2,11	4,32	0,05751	1,98	3,93	0,02274	NM_003666	RPP40
8123717	2,11	4,32	0,03672	2,27	4,82	0,00412	NM_006638	OGFOD1
7995755	2,11	4,31	0,02054	2,67	6,38	0,00026	NM_018233	GUSBP3
8112560	2,11	4,31	0,12997	1,55	2,93	0,02161	NR_027386	PEX19
7921571	2,11	4,31	0,01293	2,54	5,80	0,00005	NM_002857	NPIPL3
8000636	2,11	4,31	0,07239	3,43	10,81	0,00844	ENST00000446615	C19orf50
8027018	2,11	4,31	0,04544	2,62	6,13	0,00079	NM_001171948	ATL3
7948997	2,11	4,31	0,02400	3,10	8,56	0,00001	NM_015459	RBM18
8163948	2,11	4,31	0,03110	2,76	6,79	0,00041	NM_033117	USP16
8068062	2,11	4,30	0,02775	2,96	7,80	0,00393	NM_006447	GRLF1
8029856	2,11	4,30	0,00543	3,18	9,07	0,00716	NM_004491	MAP3K2
8054997	2,11	4,30	0,00350	1,29	2,45	0,12464	NM_006609	OTUD4
8103011	2,11	4,30	0,00718	2,26	4,80	0,03690	NM_199324	SMC5
8155770	2,11	4,30	0,02366	3,45	10,92	0,00032	NM_015110	LYRM1
7993800	2,11	4,30	0,02236	2,84	7,18	0,00308	NM_001128301	TUBB
8117995	2,10	4,30	0,06166	3,97	15,63	0,00120	NM_178014	TUBB
8177858	2,10	4,30	0,06166	3,97	15,63	0,00120	NM_178014	TUBB
8179174	2,10	4,30	0,06166	3,97	15,63	0,00120	NM_178014	SESN1
8128698	2,10	4,30	0,03187	0,23	1,17	0,50702	NM_014454	RNF160
8069711	2,10	4,30	0,02784	2,92	7,58	0,00241	NM_015565	NMT1
8007715	2,10	4,30	0,00502	2,84	7,15	0,00019	NM_021079	C6orf153
8119661	2,10	4,30	0,00520	2,00	4,01	0,01105	NM_033112	APOL6
8072710	2,10	4,30	0,03186	0,69	1,61	0,03667	NM_030641	IFNGR2
8068280	2,10	4,29	0,01385	2,66	6,30	0,00091	NM_005534	YARS
7914563	2,10	4,29	0,00814	3,81	13,98	0,00044	NM_003680	LSM14A
8027592	2,10	4,29	0,00409	2,77	6,80	0,00000	NM_001114093	DEXI
7982185	2,10	4,29	0,04681	3,75	13,49	0,00030	NM_014015	DEXI
7986685	2,10	4,29	0,04681	3,75	13,49	0,00030	NM_014015	SEMA3D
8140686	2,10	4,29	0,00158	-0,99	-1,99	0,03649	NM_152754	MATR3
8108403	2,10	4,29	0,04269	2,27	4,83	0,03164	NM_199189	RPS26
7972577	2,10	4,28	0,00331	0,87	1,83	0,07894	NM_001029	PRUNE
7905299	2,10	4,28	0,02689	3,89	14,79	0,00159	NM_021222	TLE4
8156060	2,10	4,28	0,02130	2,36	5,15	0,00014	NM_007005	WDR48
8078834	2,10	4,28	0,01351	2,16	4,46	0,00789	NM_020839	MYCBP
7915170	2,10	4,28	0,04786	1,08	2,11	0,05016	NM_012333	COPZ1
7955896	2,10	4,27	0,01746	3,65	12,55	0,00048	NM_016057	GOSR2
8007904	2,10	4,27	0,05234	1,99	3,97	0,00342	NM_004287	AMMECR1L
8055096	2,10	4,27	0,00625	3,88	14,69	0,00006	NM_031445	IFT57
8089314	2,09	4,27	0,02483	1,13	2,18	0,00958	NM_018010	PRKAR2A
8087210	2,09	4,27	0,02630	2,55	5,84	0,00045	NM_004157	DCAF16
8099570	2,09	4,27	0,01582	3,65	12,51	0,00157	NM_017741	ISG20L2
7921110	2,09	4,27	0,01577	3,67	12,72	0,00000	NM_030980	DBT
7918008	2,09	4,27	0,02362	2,43	5,37	0,00328	NM_001918	BSG
8023955	2,09	4,27	0,00113	2,20	4,60	0,00842	NM_001728	STX7
8129590	2,09	4,26	0,00188	1,73	3,32	0,00261	NM_003569	PPIA
8067978	2,09	4,25	0,21338	1,97	3,92	0,00086	NM_021130	ARHGAP17
8000375	2,09	4,25	0,01696	2,43	5,40	0,00073	NM_001006634	IWS1
8055021	2,09	4,25	0,00195	2,39	5,22	0,00296	NM_017969	HMGXB4
8072645	2,09	4,25	0,00065	2,33	5,02	0,00113	NR_027780	CDC42SE2
8107868	2,09	4,24	0,04032	3,93	15,24	0,00108	NM_020240	GUSBP3
8105991	2,08	4,24	0,12382	1,48	2,80	0,02889	NR_027386	USP40
8059801	2,08	4,24	0,00301	1,77	3,40	0,00014	NM_018218	ARHGEF7
7970111	2,08	4,24	0,00355	0,61	1,52	0,23704	NM_003899	NIPSNAP3A
8157021	2,08	4,23	0,02554	3,20	9,21	0,00069	NM_015469	ZNHIT6
7917359	2,08	4,23	0,00520	1,35	2,55	0,10645	NM_017953	KCNT2
7923043	2,08	4,23	0,06191	0,26	1,20	0,53496	NM_198503	IFT52
8062748	2,08	4,23	0,00147	1,77	3,40	0,00032	NM_016004	AP2M1
8084345	2,08	4,23	0,03429	1,70	3,24	0,00029	NM_004068	ZBTB8OS
7914550	2,08	4,23	0,02532	1,96	3,90	0,00676	NM_178547	LRCH1
7968976	2,08	4,22	0,00974	1,82	3,53	0,02145	NM_001164211	TMOD3
7983744	2,08	4,22	0,00122	2,69	6,45	0,00193	NM_014547	RFWD3

8002762	2,08	4,22	0,03145	3,96	15,53	0,00001	NM_018124	ADSS
7925550	2,08	4,22	0,00485	2,33	5,03	0,00178	NM_001126	C7orf28A
8138128	2,08	4,22	0,05616	3,12	8,67	0,00079	NM_015622	EMD
8170850	2,08	4,22	0,01676	4,11	17,26	0,00074	NM_000117	OCIAD2
8100298	2,08	4,22	0,00368	1,27	2,41	0,02835	NM_001014446	CBX1
8016402	2,08	4,22	0,00795	1,37	2,58	0,00555	NM_001127228	SSH2
8013965	2,07	4,21	0,00121	2,03	4,09	0,00271	NM_033389	CHMP5
8154785	2,07	4,21	0,03359	3,33	10,08	0,00330	NM_016410	KDELC1
7972682	2,07	4,21	0,02781	2,77	6,80	0,00164	NM_024089	LGALS8
7910706	2,07	4,21	0,01781	2,09	4,26	0,00159	NM_006499	IPPK
8162421	2,07	4,21	0,02797	3,26	9,56	0,00536	NM_022755	AP3D1
8032418	2,07	4,21	0,02511	3,11	8,61	0,00245	NM_003938	ASXL2
8050875	2,07	4,20	0,03257	2,98	7,90	0,03147	NM_018263	NHEJ1
8059071	2,07	4,20	0,01110	2,20	4,59	0,00669	NM_024782	SIPA1L1
7975459	2,07	4,20	0,05872	4,18	18,10	0,00200	NM_015556	INTS4
7950628	2,07	4,20	0,00977	2,91	7,52	0,00318	NM_033547	ACIN1
7977906	2,07	4,20	0,00178	2,45	5,46	0,00007	NM_014977	ATF6B
8178727	2,07	4,20	0,03258	3,11	8,64	0,00021	NM_004381	PPP1CB
8041122	2,07	4,20	0,00805	2,70	6,48	0,00120	NM_002709	HSBP1
7997520	2,07	4,20	0,00880	1,51	2,85	0,00538	NM_001537	SGMS1
7933619	2,07	4,20	0,01467	2,05	4,15	0,00454	NM_147156	SUCLG2
8088664	2,07	4,20	0,00747	1,41	2,65	0,00855	NM_003848	SNAP29
8071426	2,07	4,20	0,00209	3,35	10,20	0,00006	NM_004782	PIP4K2A
7932530	2,07	4,19	0,02220	2,34	5,06	0,00116	NM_005028	PTRH2
8017143	2,07	4,19	0,00151	3,25	9,54	0,00068	NM_016077	GLE1
8158298	2,07	4,19	0,00361	2,72	6,58	0,00006	NM_001003722	EXOC6
7929288	2,07	4,19	0,01703	2,34	5,07	0,01955	NM_019053	TMEM217
8126086	2,07	4,19	0,00322	0,21	1,16	0,37388	NM_145316	EXOSC9
8097128	2,07	4,19	0,02222	3,29	9,81	0,00232	NM_001034194	TOM1L1
8008547	2,07	4,19	0,00954	4,46	22,05	0,00005	NM_005486	THADA
8051820	2,07	4,19	0,00258	2,50	5,67	0,00074	NM_022065	ZNF451
8120382	2,07	4,19	0,01121	2,68	6,42	0,01109	NM_001031623	FNTB
7975121	2,07	4,19	0,02782	3,13	8,77	0,00061	NM_002028	C8orf76
8152648	2,07	4,18	0,00151	2,14	4,40	0,01603	BC067796 TOP2A	
8014974	2,06	4,18	0,00172	2,16	4,46	0,11466	NM_001067	ZNF512
8040985	2,06	4,18	0,00418	2,67	6,36	0,00123	NM_032434	NME4
7991837	2,06	4,17	0,01409	2,63	6,19	0,00223	NM_005009	TTC9C
7940679	2,06	4,17	0,03977	2,92	7,58	0,00029	NM_173810	C17orf39
8005305	2,06	4,17	0,00537	1,52	2,87	0,00366	NM_024052	UBE2F
8049574	2,06	4,17	0,03135	2,15	4,43	0,00217	NM_080678	ANAPC5
7967149	2,06	4,17	0,00596	3,66	12,67	0,00017	NM_016237	SLC7A2
8144786	2,06	4,17	0,00391	3,36	10,24	0,00154	NM_003046	NPLOC4
8019211	2,06	4,17	0,00185	3,48	11,17	0,00050	NM_017921	C2orf49
8044127	2,06	4,17	0,05292	3,53	11,54	0,00072	NM_024093	DHX32
7936949	2,06	4,16	0,00393	2,77	6,80	0,00000	NM_018180	GEM
8151816	2,06	4,16	0,02777	1,03	2,04	0,03357	NM_005261	NKRF
8174684	2,06	4,16	0,00742	2,11	4,33	0,01491	NM_001173487	CDH10
8111255	2,06	4,16	0,00364	-0,80	-1,74	0,02774	NM_006727	TMEM110
8088054	2,06	4,16	0,01194	1,45	2,73	0,00101	NM_198563	VEZF1
8016891	2,06	4,16	0,01253	2,73	6,64	0,00769	NM_007146	METTL3
7977749	2,06	4,16	0,05305	4,17	17,97	0,00030	NM_019852	RBL2
7995631	2,05	4,15	0,00938	2,94	7,66	0,00047	NM_005611	TNFAIP3
8122265	2,05	4,15	0,01753	2,11	4,32	0,00494	NM_006290	THRA
8007008	2,05	4,15	0,00702	1,89	3,70	0,00469	NM_003250	PPP2R2D
7931405	2,05	4,15	0,01648	3,37	10,33	0,00042	NM_018461	CIZ1
8164373	2,05	4,15	0,01774	2,88	7,37	0,00699	NM_012127	RSL24D1
7989013	2,05	4,15	0,01090	3,20	9,20	0,00014	NM_016304	ITGB3
8007931	2,05	4,15	0,03517	0,95	1,93	0,00823	NM_000212	GALNT11
8137448	2,05	4,15	0,00291	2,13	4,37	0,00141	NM_022087	PCMTD2
8064302	2,05	4,15	0,01436	3,61	12,25	0,00078	NM_018257	SUGT1
7969271	2,05	4,14	0,07098	2,49	5,62	0,00017	NM_001130912	SAMD8
7928516	2,05	4,14	0,00828	1,80	3,47	0,00279	NM_144660	NCRNA00188
8005191	2,05	4,14	0,00357	1,53	2,88	0,00379	BC027986 TAX1BP3	
8011407	2,05	4,14	0,02179	1,55	2,93	0,00195	NM_014604	FAM50A
8170906	2,05	4,14	0,05993	3,74	13,34	0,00018	NM_004699	SGIP1
7902127	2,05	4,14	0,00248	-2,04	-4,11	0,00014	NM_032291	DPYSL3
8114920	2,05	4,14	0,00937	2,65	6,30	0,00009	NM_001387	SRR
8003722	2,05	4,13	0,00636	3,09	8,52	0,00127	NM_021947	MYO6
8120783	2,05	4,13	0,00331	1,84	3,59	0,00360	NM_004999	LIG4
7972737	2,05	4,13	0,06685	2,62	6,15	0,00401	NM_002312	FAM107B
7932160	2,04	4,13	0,02475	0,80	1,74	0,03416	BC072452 COX7A2	
8127629	2,04	4,13	0,00201	1,17	2,25	0,06241	NM_001865	C4orf14
8100532	2,04	4,13	0,00902	2,95	7,74	0,00066	NM_032313	TTC19

8005141	2,04	4,12	0,01630	3,19	9,10	0,00003	NM_017775	BCCIP
7931268	2,04	4,12	0,00546	2,02	4,05	0,00717	NM_016567	ACOT9
8171802	2,04	4,12	0,06473	3,55	11,73	0,00052	NM_001037171	MARVELD1
7929689	2,04	4,12	0,00026	2,62	6,13	0,00281	NM_031484	MICB
8118116	2,04	4,12	0,00953	4,68	25,65	0,00003	NM_005931	BRIX1
8104825	2,04	4,12	0,00255	1,34	2,52	0,01426	NM_018321	FNBP1
8164607	2,04	4,12	0,01944	2,32	4,98	0,00065	NM_015033	RAB13
7920422	2,04	4,12	0,05108	2,89	7,42	0,00314	NM_002870	LMBR1L
7963024	2,04	4,12	0,00171	1,99	3,98	0,02064	NM_018113	---
8127419	2,04	4,12	0,02126	1,12	2,18	0,01069	---	POTEF
8055151	2,04	4,12	0,04218	0,28	1,22	0,35742	NM_001099771	HSDL2
8157233	2,04	4,11	0,07915	1,31	2,48	0,06690	NM_032303	RABEP1
8004111	2,04	4,11	0,05953	3,05	8,31	0,00025	NM_004703	UBN2
8136516	2,04	4,11	0,00734	2,25	4,76	0,01264	NM_173569	MAP2
8047926	2,04	4,11	0,06556	2,02	4,06	0,00865	NM_002374	ATMIN
8156575	2,04	4,11	0,03469	4,19	18,20	0,00246	NM_015251	RNF217
8121825	2,04	4,11	0,03344	1,33	2,52	0,01111	NM_152553	WIPF1
8056860	2,04	4,10	0,03903	0,83	1,77	0,15269	NM_003387	ETV6
7953981	2,04	4,10	0,00722	2,88	7,38	0,00526	NM_001987	KIAA1468
8021496	2,04	4,10	0,00628	1,43	2,70	0,04039	NM_020854	MED23
8129522	2,04	4,10	0,01482	2,41	5,33	0,00203	NM_004830	LIMS3
8044333	2,03	4,10	0,00539	1,98	3,95	0,01563	NR_027467	LIMS3
8054519	2,03	4,10	0,00539	1,98	3,95	0,01563	NR_027467	FBXO22
7985053	2,03	4,10	0,01495	1,75	3,35	0,00333	NM_147188	LATS1
8130116	2,03	4,10	0,00144	1,69	3,22	0,00470	NM_004690	ARNTL2
7954527	2,03	4,09	0,06205	2,25	4,76	0,00439	NM_020183	GPR155
8056837	2,03	4,09	0,08045	2,07	4,20	0,00193	NM_001033045	KGFLP1
8155487	2,03	4,09	0,00215	-0,03	-1,02	0,94076	NR_003674	KGFLP1
8161423	2,03	4,09	0,00215	-0,03	-1,02	0,94076	NR_003674	KGFLP1
8161455	2,03	4,09	0,00215	-0,03	-1,02	0,94076	NR_003674	DIAPH2
8168691	2,03	4,09	0,00901	2,25	4,77	0,00164	NM_006729	---
7926670	2,03	4,09	0,00142	1,33	2,51	0,09331	---	ETV1
8138289	2,03	4,09	0,03167	0,11	1,08	0,77490	NM_004956	IL15RA
7931899	2,03	4,08	0,00164	1,97	3,92	0,04665	NM_002189	FOXP1
8088776	2,03	4,08	0,00690	1,60	3,04	0,00062	NM_032682	---
8129095	2,03	4,08	0,01669	2,33	5,04	0,01087	---	SMAGP
7963280	2,03	4,08	0,05492	2,67	6,36	0,00050	NM_001033873	SARS
7903619	2,03	4,08	0,02043	3,56	11,79	0,00091	NM_006513	GPC6
7969613	2,03	4,07	0,08934	-1,54	-2,90	0,00026	NM_005708	SCPEP1
8008646	2,03	4,07	0,01112	2,40	5,27	0,00241	NM_021626	COG3
7968931	2,03	4,07	0,04800	2,96	7,76	0,00147	NM_031431	HAX1
7905733	2,03	4,07	0,15097	2,62	6,13	0,00325	NM_006118	ATP8B4
7988644	2,02	4,07	0,02100	-0,50	-1,42	0,14600	NM_024837	RPAP2
7902992	2,02	4,07	0,00866	1,01	2,02	0,06997	NM_024813	PINK1
7913252	2,02	4,06	0,00210	1,68	3,21	0,00052	NM_032409	RASAL2
7907611	2,02	4,06	0,02533	3,93	15,24	0,00000	NM_170692	GCC2
8044236	2,02	4,06	0,04772	1,24	2,36	0,10517	NM_181453	SLC20A1
8044499	2,02	4,06	0,04077	3,15	8,88	0,00041	NM_005415	ZNF143
7938331	2,02	4,06	0,03145	2,82	7,08	0,00040	NM_003442	UBA52
8027024	2,02	4,06	0,00010	1,54	2,92	0,00557	NM_001033930	PVRL3
8081548	2,02	4,06	0,01192	1,91	3,75	0,01184	NM_015480	P2RX4
7959267	2,02	4,06	0,05233	3,79	13,79	0,00050	NM_002560	RASSF2
8064790	2,02	4,06	0,03710	1,53	2,89	0,02281	NM_014737	DPY19L1
8138977	2,02	4,05	0,12171	3,78	13,71	0,00001	NM_015283	PDXDC1
7993433	2,02	4,05	0,00655	2,41	5,32	0,02159	NM_015027	IDS
8175593	2,02	4,05	0,02218	2,86	7,26	0,00035	NM_000202	HMGB1
8078248	2,02	4,05	0,09579	2,22	4,67	0,00224	NM_002128	MAPKSP1
8101925	2,02	4,05	0,04251	2,83	7,13	0,00183	NM_021970	RFC1
8165672	2,02	4,05	0,20700	-4,56	-23,63	0,03126	L23320	OSBPL1A
8022572	2,02	4,04	0,01043	0,63	1,55	0,01966	NM_080597	BPNT1
7924388	2,02	4,04	0,00021	2,36	5,12	0,01522	NM_006085	FAM118B
7945058	2,02	4,04	0,03277	2,88	7,34	0,00075	NM_024556	CPPED1
7999553	2,01	4,04	0,01209	2,30	4,92	0,00043	NM_018340	SNRPC
8118826	2,01	4,04	0,02239	0,39	1,31	0,40108	NM_003093	TJP1
7986977	2,01	4,04	0,01436	2,26	4,80	0,08068	NM_003257	HIST1H4H
8124448	2,01	4,04	0,12393	4,30	19,64	0,00028	NM_003543	ZNF791
8026007	2,01	4,04	0,15907	3,85	14,45	0,00065	NM_153358	CREBBP
7999044	2,01	4,04	0,01424	3,23	9,36	0,00045	NM_004380	SSNA1
8159609	2,01	4,03	0,04638	2,59	6,01	0,00204	NM_003731	CPNE1
8065963	2,01	4,03	0,01954	3,08	8,43	0,00062	NM_152930	HIBADH
8138776	2,01	4,03	0,08421	4,14	17,60	0,00013	NM_152740	SRD5A3
8095139	2,01	4,02	0,03700	3,32	10,01	0,00108	NM_024592	BBS2
8001507	2,01	4,02	0,01654	4,02	16,20	0,00011	NM_031885	NPAS2

8043909	2,01	4,02	0,00346	2,54	5,82	0,00813	NM_002518	BAZ2A
7964203	2,01	4,02	0,00857	3,70	12,99	0,00023	NM_013449	---
7988281	2,01	4,02	0,07124	0,46	1,37	0,06366	---	FAM76A
7899350	2,01	4,02	0,00528	1,04	2,06	0,01892	NM_001143912	EIF1AX
7912852	2,01	4,02	0,02527	2,57	5,94	0,02261	NM_001412	C3orf38
8081073	2,00	4,01	0,01058	1,25	2,38	0,00153	NM_173824	DHDDS
7899173	2,00	4,01	0,01545	2,15	4,43	0,00110	NM_024887	WDR19
8094638	2,00	4,01	0,02937	2,20	4,59	0,00130	NM_025132	TMLHE
8176255	2,00	4,01	0,02122	2,06	4,16	0,00288	NM_018196	GSTT1
8074980	2,00	4,01	0,02243	2,63	6,19	0,00438	NM_000853	IDI1
7931754	2,00	4,00	0,04148	2,39	5,24	0,00300	NM_004508	EPDR1
8132369	2,00	4,00	0,00444	1,04	2,05	0,08003	NM_017549	PPFIA1
7942174	2,00	4,00	0,06043	3,30	9,82	0,00046	NM_003626	AGPAT5
8144378	2,00	4,00	0,00068	4,07	16,84	0,00116	NM_018361	RAD23B
8157125	2,00	4,00	0,00285	2,31	4,97	0,00127	NM_002874	SMG1
8000834	2,00	4,00	0,00519	2,37	5,19	0,00208	NM_015092	ARPP19
8110618	2,00	3,99	0,12856	2,17	4,50	0,02449	NM_006628	GPR176
7987439	2,00	3,99	0,02530	0,47	1,38	0,06704	NM_007223	TPP2
7969881	2,00	3,99	0,00385	2,36	5,13	0,00148	NM_003291	ARMC1
8151066	2,00	3,99	0,06057	3,91	15,01	0,00007	NM_018120	PSMB4
7905395	2,00	3,99	0,01211	2,62	6,14	0,00300	NM_002796	GTF3A
7968242	2,00	3,99	0,04528	1,74	3,34	0,00224	NM_002097	CBWD1
8044613	1,99	3,99	0,00250	1,77	3,40	0,00158	NM_001145355	PDHB
8088384	1,99	3,99	0,05184	2,45	5,46	0,00049	NM_000925	DUSP22
8116548	1,99	3,98	0,00423	1,40	2,65	0,05341	NM_020185	STX17
8156861	1,99	3,98	0,01813	2,88	7,38	0,00004	NM_017919	WRN
8145702	1,99	3,98	0,01336	2,64	6,25	0,00448	NM_000553	FAM38B
8022310	1,99	3,98	0,02721	0,67	1,59	0,04033	NM_022068	MKRN1
8143387	1,99	3,98	0,01046	2,72	6,58	0,00069	NM_013446	VPS13B
8147580	1,99	3,98	0,02398	3,08	8,46	0,00164	NM_017890	DPY19L3
8027448	1,99	3,98	0,01694	1,27	2,41	0,03516	NM_001172774	---
7903474	1,99	3,98	0,00020	0,86	1,81	0,10489	---	MAP7D3
8175369	1,99	3,97	0,01028	1,59	3,01	0,03156	NM_024597	PACS1
7941537	1,99	3,97	0,05434	2,99	7,96	0,00069	NM_018026	MADD
7939767	1,99	3,97	0,00015	2,69	6,44	0,00094	NM_003682	CALR
8026106	1,99	3,97	0,02559	3,17	9,00	0,00516	NM_004343	SAMM50
8073645	1,99	3,97	0,02684	4,60	24,22	0,00001	NM_015380	BBS4
7984686	1,99	3,96	0,05335	1,21	2,31	0,00547	NM_033028	MASTL
7926821	1,99	3,96	0,00869	2,90	7,44	0,00182	NM_001172303	HSP90AA1
7981335	1,99	3,96	0,02472	2,05	4,14	0,02418	NM_001017963	TAP1
8125512	1,99	3,96	0,00423	1,87	3,67	0,01792	NM_000593	TAP1
8178867	1,99	3,96	0,00423	1,87	3,67	0,01792	NM_000593	TAP1
8180061	1,99	3,96	0,00423	1,87	3,67	0,01792	NM_000593	ZNF614
8038933	1,99	3,96	0,01097	1,80	3,49	0,00411	NM_025040	DPYD
7917912	1,99	3,96	0,12893	-0,05	-1,04	0,72188	NM_000110	GPR126
8122365	1,98	3,96	0,00741	3,57	11,84	0,02845	NM_020455	LRCH2
8174610	1,98	3,96	0,01339	0,12	1,08	0,68436	NM_020871	PPARA
8073826	1,98	3,96	0,00583	1,50	2,82	0,12505	NM_005036	RNF170
8150565	1,98	3,95	0,00953	2,28	4,87	0,00235	NM_001160223	DDX19B
7997048	1,98	3,95	0,04688	2,28	4,86	0,00076	NM_007242	KCTD10
7966202	1,98	3,95	0,00874	1,76	3,39	0,00630	NM_031954	ABCF3
8084360	1,98	3,95	0,00219	2,30	4,91	0,02578	NM_018358	SMG1
8000651	1,98	3,95	0,00005	2,34	5,07	0,00077	NM_015092	NUDCD1
8152340	1,98	3,95	0,02038	2,37	5,16	0,00097	NM_001128211	SEC23IP
7930956	1,98	3,95	0,06988	2,52	5,75	0,00146	NM_007190	IER3
8178435	1,98	3,95	0,05185	2,77	6,83	0,00015	NM_003897	TSPAN6
8173941	1,98	3,95	0,01568	3,17	8,99	0,00005	NM_003270	MT1P3 // MT1P3
8062119	1,98	3,95	0,02192	2,01	4,02	0,00442	BC133653 // BC133653	BTBD7
7980998	1,98	3,95	0,02580	3,36	10,24	0,00004	NM_001002860	VEGFC
8103822	1,98	3,94	0,07105	1,44	2,71	0,06367	NM_005429	FZD7
8047487	1,98	3,94	0,00216	0,40	1,32	0,53878	NM_003507	---
8043743	1,98	3,94	0,05551	-0,48	-1,40	0,41134	---	TCTN1
7958692	1,98	3,94	0,00903	2,18	4,53	0,02999	NM_001173975	ARF1
7910229	1,98	3,94	0,00121	2,53	5,79	0,00143	NM_001024226	MTX2
8046573	1,98	3,94	0,07725	3,60	12,13	0,00064	NR_027850	FUCA2
8129974	1,98	3,94	0,02929	2,29	4,88	0,00308	NM_032020	FBXO11
8052024	1,98	3,93	0,02817	3,21	9,23	0,00087	NM_025133	HIST2H2BE
7919625	1,98	3,93	0,03419	2,71	6,52	0,00101	NM_003528	WDR75
8046975	1,97	3,93	0,04469	3,38	10,39	0,00597	NM_032168	ZCCHC6
8162147	1,97	3,93	0,03326	2,72	6,61	0,00589	NM_024617	AFAP1
8099259	1,97	3,93	0,02425	2,49	5,62	0,00098	NM_198595	CDC42SE1
7919888	1,97	3,93	0,01042	2,58	5,98	0,01213	NM_001038707	DYNLRB1
8062016	1,97	3,93	0,00037	1,30	2,45	0,04186	NM_014183	SLC25A3

7957746	1,97	3,93	0,02035	2,38	5,21	0,00169	NM_213611	HS2ST1
7902789	1,97	3,92	0,02914	1,97	3,93	0,00108	NM_012262	WAPAL
7934812	1,97	3,92	0,00259	2,64	6,23	0,00671	NM_015045	ATRX
8173673	1,97	3,92	0,01686	1,32	2,50	0,01385	NM_000489	PRR13
7955810	1,97	3,92	0,00243	2,03	4,08	0,00025	NM_018457	MAGEH1
8167887	1,97	3,92	0,02149	0,81	1,76	0,00173	NM_014061	SNTB2
7996908	1,97	3,92	0,00399	2,06	4,17	0,02875	NM_006750	TPM1
7984079	1,97	3,91	0,00503	2,00	4,00	0,01004	NM_000366	FAM49B
8152845	1,97	3,91	0,00835	2,66	6,32	0,00105	BC017297 USP39	
8043218	1,97	3,91	0,01089	2,72	6,58	0,00044	NM_006590	SRGAP1
7956759	1,97	3,91	0,00873	4,05	16,60	0,00006	NM_020762	PFDN2
7921786	1,97	3,91	0,09684	3,64	12,48	0,00093	NM_012394	FIG4
8121429	1,97	3,91	0,03347	2,46	5,51	0,00021	NM_014845	---
8032972	1,97	3,91	0,00711	1,51	2,86	0,00460	--- TSEN15	
7908147	1,96	3,90	0,02862	3,70	13,01	0,00008	NM_052965	KIAA1432
8154254	1,96	3,90	0,05913	2,65	6,28	0,00037	NM_020829	GPR89B
7904930	1,96	3,90	0,01068	3,08	8,45	0,00115	NM_016334	C10orf137
7931239	1,96	3,90	0,01838	3,06	8,33	0,00114	NM_015608	NCOR1
8012961	1,96	3,89	0,00376	1,76	3,39	0,00411	NM_006311	UBE2L6
7948274	1,96	3,89	0,00331	0,33	1,26	0,53668	NM_004223	CHD1
8113305	1,96	3,89	0,02224	2,69	6,44	0,00617	NM_001270	CUL1
8137054	1,96	3,89	0,03855	3,14	8,84	0,00077	NM_003592	ZNF90
8027254	1,96	3,89	0,00103	1,16	2,24	0,14243	NM_007138	UBE2D2
8108435	1,96	3,89	0,03490	2,37	5,17	0,00655	NM_181838	AKTIP
8001410	1,96	3,89	0,00984	2,63	6,21	0,00352	NM_001012398	DTWD1
7983638	1,96	3,89	0,02996	1,20	2,30	0,21862	NM_020234	RPL41
7956159	1,96	3,89	0,01255	0,84	1,79	0,29495	NM_001035267	KIAA0319L
7914809	1,96	3,89	0,02487	2,21	4,62	0,00131	NM_024874	HMGB2
8103728	1,96	3,89	0,00384	0,93	1,91	0,20266	NM_001130688	PGM1
7901951	1,96	3,89	0,00114	1,71	3,28	0,00037	NM_002633	C2orf42
8052834	1,96	3,89	0,04461	3,80	13,88	0,00010	BC005079 FUT11	
7928395	1,96	3,89	0,00217	2,92	7,59	0,00701	NM_173540	---
8128695	1,96	3,89	0,01083	0,78	1,72	0,08435	--- ARHGAP28	
8019964	1,96	3,89	0,08306	0,36	1,28	0,18929	NM_001010000	SIAE
7952408	1,96	3,88	0,02000	1,64	3,11	0,00013	NM_170601	EVI5
7917707	1,96	3,88	0,01966	2,44	5,42	0,00175	NM_005665	FAM54B
7899057	1,96	3,88	0,00081	1,69	3,22	0,01426	NM_019557	PAK1
7950578	1,96	3,88	0,01952	4,24	18,85	0,00007	NM_001128620	PLOD1
7897803	1,96	3,88	0,02258	2,87	7,32	0,00078	NM_000302	ST20
7990815	1,96	3,88	0,04716	4,14	17,68	0,00176	NM_001100880	DDX18
8044745	1,96	3,88	0,07467	3,03	8,19	0,00174	NM_006773	GPR89B
7919251	1,96	3,88	0,01141	3,03	8,19	0,00096	NM_016334	DNAJC19
8092314	1,95	3,87	0,00118	0,89	1,86	0,11481	NM_145261	GIT1
8013923	1,95	3,87	0,04135	2,92	7,56	0,00104	NM_001085454	GABPA
8133030	1,95	3,87	0,01298	2,05	4,13	0,00284	NM_002040	RAP1A
7903988	1,95	3,87	0,00532	1,40	2,63	0,02416	NM_001010935	CBARA1
7934255	1,95	3,87	0,01906	2,80	6,98	0,00006	NM_006077	MED20
8126360	1,95	3,87	0,01427	2,90	7,48	0,00183	NM_004275	MPI
7984922	1,95	3,87	0,02638	3,27	9,62	0,00187	NM_002435	BTG3
8069565	1,95	3,87	0,00746	2,83	7,12	0,00245	NM_001130914	IL18
7951686	1,95	3,87	0,00710	3,78	13,71	0,00331	NM_001562	AGTPBP1
8162086	1,95	3,86	0,03772	3,52	11,47	0,00098	NM_015239	CPSF7
7948574	1,95	3,86	0,02321	3,31	9,93	0,00065	NM_024811	TMEM184B
8076046	1,95	3,86	0,00430	3,65	12,54	0,00001	NM_012264	SUMO1
8058335	1,95	3,86	0,08765	2,82	7,07	0,00235	NM_003352	SNORD116-3
7981953	1,95	3,86	0,10189	-2,76	-6,78	0,01769	NR_003318	SNORD116-3
7981966	1,95	3,86	0,10189	-2,76	-6,78	0,01769	NR_003318	UBE3B
7958532	1,95	3,86	0,02760	3,62	12,28	0,00128	NM_130466	DDHD2
8145922	1,95	3,86	0,02437	2,99	7,92	0,00217	NM_015214	VGLL4
8085340	1,95	3,86	0,00408	1,84	3,58	0,00040	NM_014667	IFIT3
7929052	1,95	3,86	0,06559	-0,29	-1,22	0,10732	NM_001031683	FLJ44253
8071051	1,95	3,85	0,02193	2,41	5,33	0,00064	AK126241	RANBP6
8160016	1,95	3,85	0,02656	2,03	4,08	0,00236	NM_012416	SLC25A39
8016018	1,95	3,85	0,03037	3,53	11,59	0,00053	NM_001143780	ZFC3H1
7964937	1,94	3,85	0,01418	2,10	4,29	0,00222	NM_144982	RANBP2
8044263	1,94	3,85	0,04297	3,01	8,07	0,00045	NM_006267	FIBP
7949518	1,94	3,85	0,01162	2,55	5,87	0,00088	NM_198897	RAB2B
7977736	1,94	3,85	0,00615	2,36	5,14	0,00489	NM_032846	ZNF217
8067113	1,94	3,84	0,00177	2,50	5,64	0,00012	NM_006526	RBM22
8115168	1,94	3,84	0,01671	3,48	11,15	0,00022	NM_018047	TIMM9
7979416	1,94	3,84	0,00491	2,25	4,75	0,00479	NM_012460	SEPP1
8111915	1,94	3,84	0,05379	1,76	3,40	0,00309	NM_005410	VAMP2
8012376	1,94	3,84	0,00656	3,33	10,06	0,00012	NM_014232	C4orf3

8102518	1,94	3,84	0,00803	1,02	2,02	0,04453	NM_001170330	AOX1
8047300	1,94	3,84	0,02508	-1,51	-2,84	0,00242	NM_001159	FBXO34
7974473	1,94	3,84	0,06652	3,64	12,45	0,00069	NM_017943	OLFML2B
7921882	1,94	3,84	0,00810	-0,32	-1,25	0,06321	NM_015441	FAM63B
7983940	1,94	3,84	0,09038	1,98	3,94	0,00050	NM_001040450	HSD17B12
7939465	1,94	3,83	0,01129	3,63	12,36	0,00034	NM_016142	SEC22C
8086406	1,94	3,83	0,14446	3,33	10,07	0,00058	NM_004206	STAT5B
8015590	1,94	3,83	0,02010	2,32	4,99	0,01459	NM_012448	---
8056966	1,94	3,83	0,00496	1,16	2,23	0,03425	---	HNRNPM
8025429	1,94	3,83	0,00218	2,00	4,00	0,00056	NM_005968	ZC3H15
8046848	1,94	3,83	0,01530	2,52	5,75	0,00143	NM_018471	DDX21
7927936	1,94	3,83	0,05057	3,26	9,60	0,00005	NM_004728	NDFIP2
7969544	1,94	3,83	0,09859	2,85	7,19	0,00003	NM_019080	USP42
8131387	1,94	3,83	0,04752	3,17	8,99	0,00006	NM_032172	DNM1L
7954752	1,94	3,83	0,00959	3,07	8,40	0,00006	NM_012062	ZNF3
8141380	1,94	3,83	0,01296	2,02	4,04	0,00838	NM_032924	IFNAR2
8068238	1,94	3,83	0,00268	2,42	5,34	0,00434	NM_207585	THOC5
8075239	1,94	3,82	0,03417	2,76	6,79	0,00512	NM_001002878	UBR1
7987981	1,94	3,82	0,04863	2,88	7,38	0,00561	NM_174916	COMT
8071289	1,93	3,82	0,00503	2,45	5,45	0,00828	NM_000754	MTM1
8170428	1,93	3,82	0,02046	1,27	2,41	0,00752	NM_000252	MGA
7982957	1,93	3,82	0,00761	2,66	6,30	0,00211	NM_001164273	CSNK1G1
7989628	1,93	3,82	0,00594	2,09	4,27	0,00194	NM_022048	RGL1
7908125	1,93	3,82	0,02332	-0,17	-1,13	0,74641	NM_015149	ENTPD4
8149793	1,93	3,82	0,02484	2,79	6,90	0,00007	NM_004901	SRXN1
8064375	1,93	3,82	0,04999	3,76	13,58	0,00011	NM_080725	RNF139
8148265	1,93	3,82	0,01740	2,56	5,91	0,00145	NM_007218	CCND1
7942123	1,93	3,82	0,00292	2,14	4,40	0,00046	NM_053056	MAPKAPK3
8080013	1,93	3,82	0,01749	0,87	1,83	0,01751	NM_004635	C5orf36
8113097	1,93	3,81	0,02239	-0,14	-1,10	0,68256	NM_001145678	AMD1
8121489	1,93	3,81	0,02785	2,78	6,86	0,00212	NM_001634	C10orf119
7936706	1,93	3,81	0,01163	3,15	8,89	0,01159	NM_024834	LRRFIP2
8086150	1,93	3,81	0,00119	1,51	2,85	0,01740	NM_006309	MIER1
7902166	1,93	3,81	0,03851	1,47	2,78	0,04345	NM_020948	PHYH
7932094	1,93	3,81	0,01952	0,72	1,64	0,13990	NM_006214	ACTR10
7974587	1,93	3,81	0,05876	4,25	19,00	0,00015	NM_018477	CEBPG
8027566	1,93	3,81	0,00175	2,83	7,12	0,00039	NM_001806	ABHD2
7985809	1,93	3,81	0,00980	2,37	5,18	0,00032	NM_007011	BOLA3
8053059	1,93	3,81	0,00610	1,13	2,19	0,11571	NM_212552	ZFAND1
8151561	1,93	3,81	0,06209	4,93	30,45	0,00002	NM_024699	PMS2
8138030	1,93	3,80	0,01852	2,83	7,12	0,01166	NM_000535	INTS2
8017283	1,93	3,80	0,03500	3,18	9,07	0,00118	NR_026641	EYA4
8122150	1,93	3,80	0,00858	0,41	1,33	0,26336	NM_004100	RRAGB
8167897	1,93	3,80	0,08240	2,55	5,87	0,00104	NM_016656	FBXO38
8109062	1,93	3,80	0,02521	2,06	4,16	0,02083	NM_205836	LRIG2
7904086	1,93	3,80	0,02663	3,00	8,01	0,00037	NM_014813	CARM1
8025766	1,93	3,80	0,04177	3,69	12,91	0,00461	NM_199141	CBR1
8068401	1,93	3,80	0,03751	2,52	5,72	0,00000	NM_001757	IP6K2
8087201	1,92	3,80	0,01552	2,74	6,70	0,00381	NM_016291	POLR2G
7940688	1,92	3,79	0,02920	2,37	5,17	0,00166	NM_002696	CDK16
8167103	1,92	3,79	0,00747	3,88	14,76	0,00038	NM_006201	EXOC4
8136293	1,92	3,79	0,00988	2,06	4,16	0,00249	NM_021807	CST3
8065403	1,92	3,79	0,00709	-0,55	-1,46	0,22545	NM_000099	SFT2D1
8130720	1,92	3,78	0,01737	2,28	4,85	0,00196	NM_145169	ALDH18A1
7935230	1,92	3,78	0,01006	2,75	6,73	0,00103	NM_002860	SMS
8166402	1,92	3,78	0,05402	0,07	1,05	0,90061	NM_004595	BACE1
7951910	1,92	3,78	0,01932	2,37	5,18	0,00016	NM_012104	N4BP2L2
7970907	1,92	3,78	0,01289	2,34	5,06	0,00120	NM_014887	LTBR
7953321	1,92	3,78	0,01166	2,76	6,77	0,00263	NM_002342	FBXL3
7972062	1,92	3,77	0,00313	1,72	3,30	0,01290	NM_012158	NARF
8010804	1,92	3,77	0,01069	3,23	9,38	0,00112	NM_001038618	VLDLR
8154100	1,92	3,77	0,03505	3,31	9,95	0,00001	NM_003383	POLR1E
8155268	1,91	3,77	0,03168	3,04	8,23	0,00001	NM_022490	GNPAT
7910520	1,91	3,77	0,00475	3,50	11,35	0,00067	NM_014236	PPME1
7942503	1,91	3,77	0,03563	3,17	8,98	0,00017	NM_016147	DISP1
7909954	1,91	3,77	0,00388	2,18	4,54	0,00526	NM_032890	SBF2
7946516	1,91	3,77	0,00542	2,28	4,86	0,00062	NM_030962	INTS4L2
8133095	1,91	3,76	0,02359	2,51	5,69	0,00328	NR_027392	CASD1
8134318	1,91	3,76	0,07284	4,14	17,58	0,00011	NM_022900	GPRC5A
7954065	1,91	3,76	0,06524	3,65	12,58	0,00013	NM_003979	RFTN2
8058063	1,91	3,76	0,00412	-0,03	-1,02	0,94909	NM_144629	CEP57
7943297	1,91	3,76	0,02164	3,22	9,34	0,00031	NM_014679	LHFP
7971150	1,91	3,76	0,04114	2,63	6,18	0,00520	NM_005780	ZNHIT3

8006627	1,91	3,76	0,00143	2,58	5,96	0,00082	NM_004773	MRPL37
7901601	1,91	3,76	0,00692	2,07	4,20	0,00584	NM_016491	TRIM16
8012953	1,91	3,76	0,00260	3,14	8,83	0,00056	NM_006470	RNF11
7901376	1,91	3,76	0,00125	1,07	2,10	0,04331	NM_014372	05. Mrz
7929247	1,91	3,76	0,01512	3,53	11,52	0,00003	NM_017824	SCRG1
8103736	1,91	3,75	0,00758	-1,18	-2,27	0,00379	NM_007281	OTUD6B
8147262	1,91	3,75	0,07662	4,05	16,60	0,00003	NM_016023	CACNB3
7955142	1,91	3,75	0,02519	1,63	3,09	0,00324	NM_000725	HMCN1
7908204	1,91	3,75	0,03046	-0,90	-1,86	0,00117	NM_031935	SLC29A1
8119974	1,91	3,75	0,00656	2,67	6,34	0,00170	NM_001078175	LACTB2
8151296	1,91	3,75	0,01478	3,04	8,23	0,00373	NM_016027	AHCTF1
8056220	1,91	3,75	0,05400	3,48	11,15	0,00286	NM_015446	MIOS
8131479	1,91	3,75	0,00730	3,53	11,56	0,00003	NM_019005	ZBTB44
7952739	1,91	3,75	0,02346	2,32	4,99	0,06116	NM_014155	CBL
7944493	1,91	3,75	0,04013	3,15	8,87	0,00139	NM_005188	TM2D3
7991630	1,91	3,75	0,01290	2,17	4,51	0,00133	NM_078474	BTAF1
7929201	1,91	3,75	0,01246	2,74	6,66	0,01244	NM_003972	SNX27
7905444	1,90	3,74	0,00150	3,32	9,96	0,00000	NM_030918	---
8088478	1,90	3,74	0,08550	3,05	8,31	0,00039	--- INTS7	
7924119	1,90	3,74	0,04081	4,30	19,73	0,00001	NM_015434	ALDH3A2
8005638	1,90	3,74	0,00133	2,75	6,73	0,00022	NM_001031806	---
8083032	1,90	3,74	0,07442	0,05	1,04	0,79656	--- SETD3	
7981249	1,90	3,74	0,00770	3,10	8,57	0,00025	NM_032233	HDAC1
7899774	1,90	3,74	0,01829	3,42	10,73	0,00172	NM_004964	LRP10
7973352	1,90	3,74	0,00359	1,59	3,01	0,00299	NM_014045	CCRL1
8122334	1,90	3,74	0,01084	-0,90	-1,87	0,02554	NM_178445	DYNLL2
8008706	1,90	3,73	0,03543	2,01	4,03	0,00187	NM_080677	MKRN2
8077931	1,90	3,73	0,04813	2,52	5,74	0,00240	NM_014160	AKAP9
8134122	1,90	3,73	0,00501	0,86	1,82	0,04445	NM_005751	UBL5
8025500	1,90	3,72	0,03437	0,73	1,65	0,34963	NM_024292	---
8117237	1,90	3,72	0,02338	2,21	4,62	0,06723	--- SNX24	
8107632	1,90	3,72	0,05859	2,28	4,87	0,00383	NM_014035	PI4KA
8074647	1,90	3,72	0,02011	3,43	10,78	0,00103	NM_058004	ARFGAP3
8076515	1,90	3,72	0,05777	1,86	3,64	0,00264	NM_014570	CYLD
7995552	1,89	3,72	0,02006	1,16	2,23	0,02029	NM_015247	SWAP70
7938370	1,89	3,72	0,00350	2,53	5,76	0,00019	NM_015055	SLC9A9
8091260	1,89	3,72	0,02538	0,25	1,19	0,62762	NM_173653	BAZ2B
8056060	1,89	3,72	0,00177	1,61	3,05	0,00189	NM_013450	C12orf49
7966738	1,89	3,72	0,02804	2,85	7,21	0,00179	BC019843 FAM162A	
8082066	1,89	3,71	0,00059	0,47	1,39	0,16589	NM_014367	GUSB
8139859	1,89	3,71	0,00311	1,65	3,14	0,01316	NM_000181	CACYBP
8057554	1,89	3,71	0,03553	3,21	9,25	0,00072	NM_014412	LGR4
7947199	1,89	3,71	0,00443	3,52	11,49	0,00001	NM_018490	KLHL13
8174654	1,89	3,71	0,02480	0,94	1,92	0,01265	NM_033495	DHX29
8112081	1,89	3,71	0,00667	2,16	4,46	0,00511	NM_019030	ASPN
8162394	1,89	3,71	0,00822	-0,84	-1,79	0,00400	NM_017680	CTTNBP2NL
7904018	1,89	3,71	0,04175	1,47	2,76	0,01433	NM_018704	GFM1
8083630	1,89	3,70	0,02784	2,55	5,87	0,00302	NM_024996	TTLL7
7917199	1,89	3,70	0,00722	2,09	4,26	0,00116	NM_024686	SGCD
8109490	1,89	3,70	0,05770	-0,83	-1,78	0,00320	NM_000337	MAGI1
8088602	1,89	3,70	0,02623	1,32	2,50	0,00482	NM_015520	ABR
8010983	1,89	3,70	0,05391	2,90	7,47	0,00616	NM_021962	---
7957271	1,89	3,70	0,00018	0,42	1,34	0,33625	--- BUD31	
8134589	1,89	3,70	0,00609	1,31	2,47	0,00265	NM_003910	UBE2A
8169645	1,89	3,70	0,01789	3,20	9,16	0,00213	NM_003336	SPPL3
7967091	1,89	3,70	0,06210	4,42	21,41	0,00009	NM_139015	GOLGB1
8089930	1,89	3,70	0,01269	1,61	3,05	0,00545	NM_004487	MID2
8169249	1,89	3,70	0,01777	1,48	2,80	0,00421	NM_012216	HIGD2A
8110166	1,89	3,70	0,06939	3,56	11,80	0,00085	NM_138820	LRRN3
8135488	1,89	3,70	0,02445	-0,70	-1,63	0,00458	NM_001099660	CABLES1
8020495	1,88	3,69	0,03940	2,54	5,82	0,00295	NM_138375	---
7960516	1,88	3,69	0,00035	0,88	1,84	0,01488	--- ERCC4	
7993298	1,88	3,69	0,04496	3,08	8,44	0,00073	NM_005236	THYN1
7952869	1,88	3,69	0,01284	1,62	3,07	0,00624	NM_014174	SLC12A2
8107769	1,88	3,69	0,01003	1,75	3,35	0,00035	NM_001046	TXNDC15
8108166	1,88	3,69	0,00470	0,75	1,68	0,27719	NM_024715	DKFZp686O24166
7938702	1,88	3,69	0,04830	3,05	8,31	0,00047	NR_026750	HNRNPR
7913571	1,88	3,68	0,03094	3,25	9,50	0,00284	NM_001102398	RPL27A
7938286	1,88	3,68	0,00472	2,06	4,16	0,00083	NM_000990	SLC25A32
8152255	1,88	3,68	0,03246	4,83	28,40	0,00042	NM_030780	SRF
8119712	1,88	3,68	0,01006	3,43	10,77	0,00084	NM_003131	PRPSAP2
8005512	1,88	3,68	0,02205	3,88	14,77	0,00011	NM_002767	SMEK2
8052307	1,88	3,68	0,01444	2,52	5,74	0,00017	NM_001122964	ARRB1

7950473	1,88	3,68	0,01722	3,72	13,19	0,00021	NM_004041	RPL27
8007441	1,88	3,68	0,06132	0,70	1,63	0,10875	NM_000988	HDAC3
8114691	1,88	3,68	0,00053	3,26	9,55	0,00008	NM_003883	DHX9
7908022	1,88	3,68	0,03582	3,85	14,41	0,00013	NR_033302	CS
7964064	1,88	3,68	0,05524	3,84	14,33	0,00011	NM_004077	IDH2
7991374	1,88	3,67	0,02783	3,04	8,23	0,00654	NM_002168	CRTC3
7986049	1,88	3,67	0,03500	3,00	8,01	0,00002	NM_022769	MLLT3
8160332	1,87	3,67	0,02475	1,84	3,58	0,00912	NM_004529	WDR11
7930996	1,87	3,67	0,02960	3,50	11,32	0,00109	NM_018117	DYNC1LI1
8086008	1,87	3,67	0,06545	2,63	6,19	0,00195	NM_016141	C9orf123
8160036	1,87	3,66	0,00523	1,22	2,33	0,00674	NM_033428	FAM10A4
7969177	1,87	3,66	0,00034	0,05	1,04	0,88695	NR_002183	FASTKD5
8064581	1,87	3,66	0,02062	2,21	4,62	0,00367	NM_021826	STXBP5
8122600	1,87	3,66	0,04279	2,72	6,59	0,00383	NM_001127715	DYNLL1
7967067	1,87	3,66	0,01983	-0,33	-1,26	0,72774	NM_001037495	GUF1
8094876	1,87	3,66	0,05688	3,04	8,25	0,00179	NM_021927	---
7942779	1,87	3,66	0,10424	-0,86	-1,82	0,34269	--- TRAFD1	
7958828	1,87	3,66	0,04730	2,67	6,36	0,00002	NM_001143906	C1orf124
7910539	1,87	3,65	0,02119	2,13	4,37	0,01129	NM_001010984	ARMC9
8049044	1,87	3,65	0,01607	0,55	1,46	0,28136	NM_025139	C17orf37
8014882	1,87	3,65	0,14750	3,60	12,17	0,00328	NM_032339	KIAA0090
7913110	1,87	3,65	0,03352	2,98	7,91	0,00210	BC034589 C3orf10	
8077770	1,87	3,65	0,00989	2,99	7,96	0,00154	NM_018462	ZNF532
8021442	1,87	3,65	0,00257	1,52	2,86	0,00265	NM_018181	ITGA11
7989985	1,86	3,64	0,01652	-0,78	-1,72	0,15748	NM_001004439	GRSF1
8100834	1,86	3,64	0,00694	1,66	3,17	0,00452	NM_002092	EBAG9
8147970	1,86	3,64	0,00926	1,81	3,51	0,00835	NM_198120	EDEM1
8077458	1,86	3,64	0,09330	3,39	10,50	0,00248	NM_014674	MIB1
8020423	1,86	3,64	0,02028	3,62	12,26	0,00082	NM_020774	MRPL22
8109475	1,86	3,63	0,00964	1,74	3,35	0,00564	NM_014180	GNL2
7915015	1,86	3,63	0,00211	2,06	4,17	0,00001	NM_013285	---
7971858	1,86	3,63	0,07808	3,53	11,55	0,00501	--- TMEM43	
8077993	1,86	3,63	0,05193	2,06	4,18	0,01125	NM_024334	ABL1
8158725	1,86	3,63	0,03986	3,37	10,36	0,00082	NM_005157	TOR1AIP2
7922646	1,86	3,63	0,23220	4,94	30,75	0,00014	NM_022347	TCTN2
7959638	1,86	3,62	0,00085	2,07	4,21	0,01019	NM_024809	NAT15
7992905	1,86	3,62	0,02137	1,26	2,39	0,05356	NM_001083601	EIF2C4
7900009	1,86	3,62	0,03550	2,41	5,30	0,00688	NM_017629	KIAA1598
7936529	1,86	3,62	0,02206	1,79	3,47	0,14448	NM_001127211	METTL6
8085571	1,86	3,62	0,02917	3,09	8,54	0,00222	NM_152396	MTMR3
8072279	1,86	3,62	0,04192	2,68	6,42	0,00348	NM_153050	E2F4
7996455	1,86	3,62	0,00270	3,78	13,78	0,00005	NM_001950	---
8165665	1,86	3,62	0,16850	-0,61	-1,53	0,01845	--- RTCD1	
7903321	1,86	3,62	0,02734	2,73	6,63	0,00005	NM_001130841	RABL5
8141728	1,86	3,62	0,01041	1,38	2,60	0,00599	NM_022777	MRAS
8082965	1,86	3,62	0,00301	0,62	1,54	0,15815	NM_012219	MIPEP
7970577	1,86	3,62	0,01572	0,42	1,33	0,07525	NM_005932	POPDC3
8128565	1,85	3,62	0,00577	3,53	11,56	0,00106	NM_022361	ZBTB24
8128767	1,85	3,62	0,00558	1,83	3,56	0,00315	NM_014797	MTFMT
7989708	1,85	3,61	0,05923	2,89	7,42	0,00075	NM_139242	C11orf1
7943779	1,85	3,61	0,00253	2,22	4,66	0,00648	NM_022761	TCF7L1
8043114	1,85	3,61	0,00554	1,68	3,20	0,00038	NM_031283	---
7936833	1,85	3,61	0,05902	0,88	1,84	0,10931	--- EMG1	
7953594	1,85	3,61	0,01003	2,48	5,58	0,00117	NM_006331	---
8084630	1,85	3,61	0,05303	3,86	14,50	0,00085	--- CEP55	
7929334	1,85	3,61	0,00279	3,93	15,28	0,00011	NM_018131	VEPH1
8091678	1,85	3,61	0,02314	-0,18	-1,13	0,52194	NM_024621	ZWILCH
7984330	1,85	3,61	0,05622	3,12	8,68	0,00116	NR_003105	C5orf36
8113103	1,85	3,61	0,05159	0,00	1,00	0,99931	NM_001145678	ZNF222
8029353	1,85	3,61	0,06199	0,87	1,83	0,09127	NM_001129996	CSNK2A1P
7946655	1,85	3,60	0,02600	1,11	2,17	0,13043	NR_002207	DAB1
7916541	1,85	3,60	0,01333	-1,51	-2,84	0,00451	NM_021080	KIF13A
8124088	1,85	3,60	0,00865	2,96	7,79	0,00021	NM_022113	ASF1A
8121734	1,85	3,60	0,03568	2,26	4,79	0,02563	NM_014034	CCDC127
8110678	1,85	3,60	0,01926	1,07	2,10	0,10654	NM_145265	HLA-H
8117777	1,85	3,60	0,01757	2,40	5,28	0,00354	NR_001434	SLC25A1
8074388	1,85	3,59	0,00660	3,04	8,25	0,00124	NM_005984	C2CD3
7950336	1,85	3,59	0,00935	2,45	5,45	0,00049	NM_015531	FUS
7995069	1,85	3,59	0,02454	2,88	7,38	0,00215	NM_004960	C3orf31
8085350	1,84	3,59	0,10113	2,47	5,56	0,00650	NM_138807	CHMP2B
8081055	1,84	3,59	0,08997	2,50	5,65	0,00121	NM_014043	ZNF616
8038952	1,84	3,59	0,00178	0,47	1,38	0,20177	BC032805 CD9	
7953291	1,84	3,59	0,00432	2,59	6,03	0,00096	NM_001769	PSMF1

8060379	1,84	3,59	0,03356	3,42	10,70	0,00410	NM_178578	COMMD10
8107458	1,84	3,59	0,00170	1,10	2,15	0,00901	NM_016144	HSPH1
7970864	1,84	3,59	0,01829	1,25	2,39	0,01637	NM_006644	MMP3
7951284	1,84	3,59	0,01392	-0,61	-1,53	0,00572	NM_002422	NT5C3L
8015445	1,84	3,58	0,00652	2,62	6,14	0,00244	NR_033465	IFI30
8026971	1,84	3,58	0,01431	3,55	11,69	0,00007	NM_006332	MITF
8080938	1,84	3,58	0,03546	1,65	3,15	0,00054	NM_198159	AHCTF1
7925622	1,84	3,58	0,01418	2,91	7,54	0,00241	NM_015446	TRANK1
8086125	1,84	3,58	0,02553	1,80	3,49	0,00113	NM_014831	CST2
8065416	1,84	3,58	0,00741	-1,87	-3,65	0,00110	NM_001322	PIP4K2B
8014723	1,84	3,58	0,01911	3,02	8,12	0,00025	NM_003559	GGNBP2
8006638	1,84	3,58	0,01140	3,00	8,02	0,00086	NM_024835	ZMYND8
8066786	1,84	3,58	0,00014	2,32	5,01	0,01010	NM_183047	---
8165644	1,84	3,57	0,14027	-1,78	-3,43	0,00052	--- RAD18	
8085145	1,84	3,57	0,00264	2,38	5,21	0,00387	NM_020165	PARP6
7990165	1,84	3,57	0,02345	4,13	17,56	0,00009	NM_020214	ERAP2
8107044	1,84	3,57	0,08668	1,93	3,80	0,00769	NM_022350	DDIT4
7928308	1,83	3,57	0,01028	1,50	2,83	0,03635	NM_019058	SCFD2
8100347	1,83	3,57	0,00453	1,46	2,75	0,02502	NM_152540	MFN2
7897824	1,83	3,56	0,00262	2,28	4,87	0,00021	NM_014874	SLC30A4
7988426	1,83	3,56	0,03504	2,47	5,53	0,00606	NM_013309	RPL23AP7
8054762	1,83	3,56	0,00746	0,53	1,45	0,08764	NR_024528	NIPA2
7986665	1,83	3,56	0,00206	3,41	10,62	0,00039	NM_030922	ATF7
7963698	1,83	3,56	0,00410	1,84	3,59	0,00167	NM_001130059	PAIP1
8005473	1,83	3,56	0,06720	1,45	2,73	0,02398	NM_006451	USP31
8000244	1,83	3,56	0,10548	2,17	4,50	0,00194	NM_020718	RNPEP
7908779	1,83	3,55	0,00808	3,02	8,08	0,00342	NM_020216	RPL13A
8030351	1,83	3,55	0,07564	2,65	6,29	0,01163	NM_012423	TOX
8150962	1,83	3,55	0,01278	-0,33	-1,26	0,06792	NM_014729	STON1-GTF2A1L
8041940	1,83	3,55	0,01547	-0,21	-1,16	0,29963	NM_172311	ARHGAP12
7932885	1,83	3,55	0,01369	2,51	5,70	0,00104	NM_018287	TMED7
8113623	1,83	3,55	0,01642	1,55	2,93	0,00495	NM_181836	CDK4
7964522	1,83	3,55	0,13221	4,34	20,27	0,00039	NM_000075	IMPDH2
8087254	1,83	3,55	0,02894	2,26	4,80	0,00589	NM_000884	COL4A3BP
8112687	1,83	3,55	0,03809	1,66	3,17	0,01521	NM_005713	GTF2E1
8081953	1,83	3,55	0,02034	1,24	2,37	0,03495	NM_005513	ALDH7A1
8113773	1,83	3,55	0,08207	2,31	4,95	0,02822	NM_001182	SHISA5
8086953	1,83	3,55	0,00546	2,36	5,15	0,00663	NM_016479	SMC1A
8172876	1,83	3,54	0,00280	2,66	6,30	0,00000	NM_006306	DCLK1
7970954	1,83	3,54	0,07875	-0,60	-1,52	0,10409	NM_004734	PALB2
8000329	1,82	3,54	0,00897	2,38	5,20	0,00938	NM_024675	ANKRD27
8035905	1,82	3,54	0,00455	2,77	6,83	0,00144	NM_032139	POLE2
7978846	1,82	3,54	0,00774	4,12	17,40	0,00051	NM_002692	IK
8108568	1,82	3,54	0,00688	1,33	2,52	0,00022	NM_006083	PHLDA1
7965040	1,82	3,54	0,01932	2,32	4,98	0,00261	NM_007350	NEK9
7980271	1,82	3,54	0,03186	3,63	12,34	0,00050	NM_033116	SLC25A40
8140814	1,82	3,54	0,03581	3,97	15,65	0,00004	NM_018843	ECM1
7905220	1,82	3,54	0,06762	0,83	1,77	0,10452	NM_004425	SLC35B3
8123825	1,82	3,54	0,01613	1,43	2,70	0,04249	NM_015948	MORF4L1
7985226	1,82	3,53	0,00206	3,16	8,96	0,00612	NM_206839	PPAT
8100495	1,82	3,53	0,03809	3,43	10,79	0,00024	NM_002703	IPO9
7908732	1,82	3,53	0,02353	3,29	9,75	0,00009	NM_018085	CTTN
7942204	1,82	3,53	0,01440	3,25	9,51	0,00342	NM_005231	HNRPLL
8051605	1,82	3,53	0,01508	2,01	4,02	0,01661	NM_138394	COMMD8
8100145	1,82	3,53	0,05835	1,99	3,98	0,01064	NM_017845	RIPK1
8116622	1,82	3,53	0,01994	1,65	3,14	0,00205	NM_003804	NT5DC1
8121578	1,82	3,53	0,01334	1,60	3,04	0,00026	NM_152729	MARK3
7977077	1,82	3,53	0,02667	2,94	7,70	0,00011	NM_001128918	CEP170L
8097058	1,82	3,52	0,07359	1,64	3,13	0,01407	NR_003135	ABCC5
8092418	1,82	3,52	0,00488	2,69	6,44	0,00012	NM_005688	C6orf225
8121547	1,82	3,52	0,07578	0,16	1,12	0,52836	NM_001033564	MAP9
8103341	1,82	3,52	0,00090	-0,61	-1,53	0,06759	NM_001039580	PHF5A
8076339	1,81	3,52	0,01114	3,00	8,01	0,00582	NM_032758	ARF5
8135856	1,81	3,52	0,05509	2,65	6,30	0,00500	NM_001662	ZNF462
8157105	1,81	3,52	0,01350	2,66	6,32	0,00003	NM_021224	TRIM22
7938035	1,81	3,51	0,00609	-0,70	-1,63	0,02133	NM_006074	PDS5B
7968516	1,81	3,51	0,00591	2,04	4,10	0,00520	NM_015032	CTPS
7900510	1,81	3,51	0,00853	1,75	3,37	0,00082	NM_001905	SNORD80
7922404	1,81	3,51	0,00173	-2,79	-6,91	0,21371	NR_003940	TOMM34
8066461	1,81	3,51	0,00260	3,24	9,47	0,00003	NM_006809	SCMH1
7915363	1,81	3,51	0,02670	2,65	6,26	0,00039	NM_001031694	RPS18
8118644	1,81	3,51	0,05926	0,83	1,77	0,00513	NM_022551	RPS18
8178253	1,81	3,51	0,05926	0,83	1,77	0,00513	NM_022551	RPS18

8179544	1,81	3,51	0,05926	0,83	1,77	0,00513	NM_022551	INPP5K
8011062	1,81	3,51	0,00383	1,98	3,94	0,00004	NM_130766	RPL21P28
7924144	1,81	3,51	0,15554	0,59	1,50	0,12216	NR_026911	FAM116A
8088348	1,81	3,50	0,00058	2,38	5,19	0,00009	NM_152678	DDX3Y
8176624	1,81	3,50	0,01847	2,42	5,35	0,00001	NM_001122665	C1orf103
7918467	1,81	3,50	0,02250	3,14	8,84	0,00280	NM_018372	C17orf75
8014100	1,81	3,50	0,01265	2,88	7,34	0,00293	NM_022344	NIPSNAP1
8075263	1,81	3,50	0,01084	3,30	9,82	0,00021	NM_003634	SPATS2
7955217	1,81	3,50	0,00608	4,17	18,04	0,00001	NM_023071	BCL9
7904907	1,80	3,49	0,00475	1,90	3,74	0,00149	NM_004326	GTPBP2
8126629	1,80	3,49	0,03200	5,10	34,30	0,00010	NM_019096	---
8097443	1,80	3,49	0,00225	0,79	1,72	0,13249	--- RPS6KA6	
8173825	1,80	3,49	0,01702	2,53	5,77	0,00078	NM_014496	CENPF
7909708	1,80	3,49	0,00395	1,66	3,16	0,11135	NM_016343	ACAT2
8123137	1,80	3,49	0,00960	1,96	3,88	0,00587	NM_005891	NUP37
7965855	1,80	3,49	0,02003	2,76	6,78	0,00074	NM_024057	PANK3
8115681	1,80	3,49	0,03976	2,19	4,57	0,00278	NM_024594	ADNP2
8021914	1,80	3,48	0,04978	3,00	8,00	0,00227	NM_014913	TFB1M
8130438	1,80	3,48	0,02519	1,78	3,43	0,00158	NM_016020	MKNK2
8032392	1,80	3,48	0,00743	3,24	9,43	0,00250	NM_199054	SMN1
8105958	1,80	3,48	0,00102	2,50	5,67	0,00059	NM_000344	SMN1
8177647	1,80	3,48	0,00102	2,50	5,67	0,00059	NM_000344	ZNF800
8142730	1,80	3,48	0,03792	0,05	1,04	0,82926	NM_176814	NANS
8156761	1,80	3,48	0,01131	3,60	12,09	0,00018	NM_018946	CREB5
8131996	1,80	3,48	0,02569	1,35	2,54	0,01540	NM_182898	RASA2
8083094	1,80	3,48	0,08558	2,88	7,35	0,00119	NM_006506	FAM26E
8121601	1,80	3,48	0,08856	-0,39	-1,31	0,27893	NM_153711	VOPP1
8139712	1,80	3,47	0,00905	1,16	2,23	0,01181	NM_030796	MTF1
7915091	1,80	3,47	0,03529	2,06	4,17	0,00065	NM_005955	PKD2
8096314	1,80	3,47	0,02344	2,16	4,48	0,00003	NM_000297	EBNA1BP2
7915485	1,80	3,47	0,06644	3,10	8,55	0,00004	NM_006824	SNORD33
8030362	1,80	3,47	0,01905	-2,80	-6,94	0,15326	NR_000020	LMO3
7961580	1,80	3,47	0,01066	4,01	16,15	0,00516	NM_018640	C5orf28
8111952	1,79	3,47	0,01007	2,47	5,54	0,00298	NM_022483	H2AFY
8114225	1,79	3,47	0,00372	2,83	7,12	0,00001	NM_004893	FBXL20
8014825	1,79	3,46	0,04114	3,99	15,86	0,00001	NM_032875	FAM117B
8047565	1,79	3,46	0,05586	1,98	3,94	0,00428	NM_173511	EPT1
8040655	1,79	3,46	0,02688	3,65	12,59	0,00018	NM_033505	ZDHHC17
7957277	1,79	3,46	0,05009	2,26	4,78	0,00450	NM_015336	KIDINS220
8050128	1,79	3,46	0,05340	2,70	6,50	0,00011	NM_020738	MRPL28
7998222	1,79	3,46	0,01006	2,06	4,16	0,00213	NM_006428	IFI6
7914127	1,79	3,46	0,00171	1,89	3,71	0,01234	NM_002038	CHM
8173892	1,79	3,46	0,00622	1,39	2,62	0,01767	NM_000390	RPRD1A
8022914	1,79	3,45	0,06136	3,98	15,73	0,00010	NM_018170	AAGAB
7989953	1,79	3,45	0,06193	2,53	5,77	0,00201	NM_024666	SLC25A24
7918188	1,79	3,45	0,01082	1,50	2,83	0,00053	NM_013386	---
8166948	1,79	3,45	0,02557	0,45	1,36	0,33466	--- NAPA	
8037913	1,79	3,45	0,00492	0,68	1,60	0,01342	NM_003827	LEPREL1
8092707	1,78	3,45	0,01225	2,85	7,20	0,01253	NM_018192	PIGM
7921526	1,78	3,45	0,01974	2,73	6,65	0,00004	NM_145167	KPNA5
8121632	1,78	3,44	0,12547	3,18	9,09	0,00312	NM_002269	DNTTIP1
8063028	1,78	3,44	0,03675	2,98	7,89	0,00154	NM_052951	TMEM51
7898115	1,78	3,44	0,02793	2,39	5,23	0,00088	NM_001136218	UCK1
8164653	1,78	3,44	0,03343	4,30	19,63	0,00009	NM_031432	LACTB
7984103	1,78	3,44	0,03511	1,61	3,05	0,00005	NM_032857	HEATR6
8017186	1,78	3,44	0,10840	2,96	7,80	0,00075	NM_022070	TMEM120A
8140371	1,78	3,44	0,02251	1,03	2,05	0,05597	NM_031925	C10orf118
7936419	1,78	3,44	0,00676	2,20	4,59	0,00737	NM_018017	SCAMP2
7990417	1,78	3,44	0,03179	3,59	12,00	0,00164	NM_005697	HIVEP1
8116910	1,78	3,44	0,00577	2,06	4,17	0,00879	NM_002114	EBF1
8115543	1,78	3,44	0,05436	-1,09	-2,13	0,01002	NM_024007	YIF1A
7949577	1,78	3,44	0,11122	3,75	13,42	0,00394	NM_020470	CAPRIN2
7962112	1,78	3,44	0,01292	1,37	2,58	0,00146	NM_001002259	GYG2
8165817	1,78	3,44	0,00175	0,30	1,23	0,63744	NM_001079855	NCOA7
8121861	1,78	3,44	0,01523	2,33	5,02	0,00769	NM_181782	---
8165663	1,78	3,43	0,09660	-1,41	-2,66	0,24169	--- DOK5	
8063458	1,78	3,43	0,01034	-0,91	-1,87	0,00245	NM_018431	WWTR1
8091422	1,78	3,43	0,01730	1,58	2,99	0,00312	NM_001168278	COL6A1
8069269	1,78	3,43	0,00219	1,16	2,24	0,05417	NM_001848	CIRBP
8024238	1,78	3,42	0,04978	2,68	6,43	0,00089	NM_001280	ANAPC1
8043349	1,77	3,42	0,00119	3,51	11,36	0,00574	NM_022662	TLK2
8009014	1,77	3,42	0,00337	1,75	3,37	0,00000	NM_006852	GLIS3
8159900	1,77	3,42	0,00753	2,45	5,47	0,00036	NM_152629	TRERF1

8126428	1,77	3,42	0,05554	1,70	3,24	0,02718	NM_033502	LAMB2
8087337	1,77	3,42	0,00465	2,82	7,05	0,00090	NM_002292	SNRNP200
8053909	1,77	3,42	0,02576	2,87	7,33	0,00057	NM_014014	C3orf23
8079170	1,77	3,42	0,00173	1,31	2,47	0,01019	NM_173826	ASB8
7962783	1,77	3,41	0,06477	2,34	5,07	0,00101	NM_024095	USP18
8074606	1,77	3,41	0,05682	2,77	6,84	0,00182	NM_017414	EIF2AK3
8053668	1,77	3,41	0,02426	1,49	2,82	0,00386	NM_004836	TPM3
8142468	1,77	3,41	0,19784	2,88	7,34	0,01602	NM_153649	BLOC1S2
7935746	1,77	3,41	0,08141	1,52	2,87	0,03943	NM_001001342	MAPKAPK2
7909236	1,77	3,41	0,02252	2,24	4,73	0,00098	NM_004759	DUSP3
8015835	1,77	3,41	0,07243	3,13	8,74	0,00035	NM_004090	TADA2B
8093993	1,77	3,41	0,05822	3,35	10,21	0,00000	NM_152293	LRRFIP1
8049544	1,77	3,41	0,00782	0,27	1,21	0,25084	NM_001137550	KDM4A
7900833	1,77	3,40	0,09021	3,31	9,91	0,00025	NM_014663	PLK3
7901054	1,77	3,40	0,04164	0,65	1,56	0,13271	NM_004073	RNLS
7934885	1,77	3,40	0,00443	3,83	14,17	0,00017	NM_018363	ZNF845
8030978	1,76	3,40	0,00493	2,98	7,89	0,00005	NM_138374	TMEM159
7993807	1,76	3,39	0,00594	1,57	2,97	0,00234	NM_020422	ACAD11
8090715	1,76	3,39	0,01938	1,81	3,50	0,02256	NM_032169	PPIL2
8071566	1,76	3,39	0,02189	2,67	6,36	0,00008	NM_014337	TDRD7
8156688	1,76	3,39	0,00819	2,85	7,19	0,00052	NM_014290	SCOC
8097521	1,76	3,39	0,03251	1,97	3,91	0,02528	NM_032547	CLEC16A
7993223	1,76	3,39	0,01525	2,65	6,28	0,00670	NM_015226	TMEM50B
8070083	1,76	3,39	0,01358	3,19	9,14	0,00059	NM_006134	NUCB1
8030133	1,76	3,39	0,00180	1,38	2,61	0,06943	NM_006184	SFRS12IP1
8112337	1,76	3,39	0,05623	1,68	3,21	0,01834	NM_173829	PDK2
8008263	1,76	3,39	0,02003	2,78	6,88	0,00373	NM_002611	PCGF6
8027330	1,76	3,39	0,04123	2,99	7,96	0,00016	NM_001011663	C10orf84
7936578	1,76	3,39	0,01315	1,90	3,73	0,01205	NM_022063	EIF4G1
8084439	1,76	3,39	0,00700	2,13	4,38	0,00689	NM_182917	TAPBP
8180166	1,76	3,38	0,00052	1,76	3,38	0,02256	NM_003190	RBBP5
7923712	1,76	3,38	0,01521	1,89	3,72	0,00027	NM_005057	COX15
7935647	1,76	3,38	0,06571	3,12	8,68	0,00006	NM_078470	UBAP2
8160722	1,76	3,38	0,02715	2,91	7,49	0,00176	NM_018449	ANGEL2
7924190	1,76	3,38	0,00604	2,23	4,68	0,00036	NM_144567	ARHGAP23
8006779	1,76	3,38	0,00450	1,29	2,45	0,06019	NM_020876	SLC35A1
8121031	1,76	3,38	0,01526	2,84	7,16	0,00042	NM_006416	AADAT
8103706	1,75	3,37	0,00900	2,49	5,63	0,00143	NM_016228	PEX3
8122409	1,75	3,37	0,04115	1,40	2,63	0,02512	NM_003630	CCDC55
8006112	1,75	3,37	0,02568	2,56	5,91	0,00468	NM_032141	NUP214
8158783	1,75	3,37	0,03784	3,38	10,42	0,00002	NM_005085	ZNF625
8034342	1,75	3,37	0,16356	1,85	3,60	0,01759	AK292455 MEGF9	
8163775	1,75	3,37	0,01170	3,65	12,59	0,00015	NM_001080497	PKDCC
8041582	1,75	3,37	0,07283	0,44	1,35	0,44033	NM_138370	MYLIP
8117020	1,75	3,37	0,03281	3,51	11,41	0,00048	NM_013262	RPL17
7902448	1,75	3,37	0,00453	1,01	2,02	0,04945	NM_000985	PTPRJ
7939839	1,75	3,37	0,05200	2,13	4,38	0,00049	NM_002843	CYP20A1
8047641	1,75	3,37	0,01959	1,37	2,59	0,01584	NM_177538	CBWD3
8161537	1,75	3,37	0,00215	1,91	3,76	0,00087	NM_201453	TCEA1
8150818	1,75	3,37	0,02079	3,20	9,20	0,00589	NM_006756	TRIM32
8157516	1,75	3,36	0,00963	1,86	3,62	0,01811	NM_012210	ABI2
8047659	1,75	3,36	0,01232	3,46	10,99	0,00035	NM_005759	RINT1
8135323	1,75	3,36	0,06817	2,44	5,41	0,00023	NM_021930	CABYR
8020653	1,75	3,36	0,01587	2,61	6,10	0,00076	NM_012189	BRWD3
8173766	1,75	3,36	0,01088	3,04	8,20	0,00001	NM_153252	NARS2
7950683	1,75	3,36	0,01867	3,94	15,31	0,00034	NM_024678	FAM190B
7928800	1,75	3,36	0,01271	2,53	5,78	0,00187	NM_018999	PAXIP1
8144047	1,75	3,36	0,00101	2,46	5,49	0,00324	NM_007349	FADS3
7948630	1,75	3,36	0,01413	3,52	11,46	0,00122	NM_021727	FGD4
7954729	1,75	3,36	0,00171	1,67	3,19	0,00237	NM_139241	TOR3A
7907690	1,75	3,36	0,06225	3,12	8,69	0,00000	NM_022371	CCDC93
8054804	1,75	3,36	0,00384	2,82	7,04	0,00024	NM_019044	GCLC
8127158	1,75	3,35	0,08269	4,03	16,37	0,00019	NM_001498	HNRPDL
8101340	1,75	3,35	0,01694	1,89	3,71	0,00170	NR_003249	SNUPN
7990528	1,75	3,35	0,01506	3,42	10,67	0,00010	NM_005701	CAMK2G
7934477	1,75	3,35	0,00669	1,65	3,15	0,00233	NM_172171	FNBP4
7947969	1,75	3,35	0,04400	2,83	7,10	0,00021	NM_015308	CDK6
8140955	1,75	3,35	0,05161	0,33	1,25	0,03075	NM_001259	TRPC1
8083166	1,75	3,35	0,01782	2,74	6,70	0,00125	NM_003304	SDHA
8104166	1,75	3,35	0,03442	4,32	20,03	0,00046	NM_004168	HMOX2
7992987	1,74	3,35	0,01364	0,95	1,93	0,14103	NM_001127204	F2R
8106393	1,74	3,35	0,06097	1,18	2,27	0,01508	NM_001992	CDK2AP1
7967412	1,74	3,35	0,00442	2,53	5,76	0,00119	NM_004642	SEPHS1

7932109	1,74	3,35	0,20569	2,96	7,79	0,00907	NM_012247	TMF1
8088700	1,74	3,35	0,00556	1,36	2,56	0,00086	NM_007114	CAMSAP1
8165046	1,74	3,35	0,01937	2,83	7,11	0,00066	NM_015447	NDUFS1
8058428	1,74	3,35	0,05778	2,52	5,75	0,00012	NM_005006	C20orf194
8064637	1,74	3,34	0,05714	1,39	2,62	0,00418	NM_001009984	DHX36
8091562	1,74	3,34	0,05408	3,88	14,69	0,00019	NM_020865	SREBF2
8073522	1,74	3,34	0,01278	3,65	12,59	0,00193	NM_004599	UBAP1
8154872	1,74	3,33	0,06368	3,19	9,10	0,00046	NM_016525	INO80
7987584	1,74	3,33	0,02596	3,05	8,30	0,00010	NM_017553	ACSS2
8062041	1,74	3,33	0,00790	0,93	1,91	0,00784	NM_018677	NUDT7
7997332	1,74	3,33	0,05054	1,16	2,24	0,02374	NM_001105663	METTL4
8022009	1,73	3,33	0,09908	4,58	24,00	0,00015	NM_022840	HIST1H4E
8117402	1,73	3,33	0,14534	-0,63	-1,55	0,02196	NM_003545	FAM122C
8169995	1,73	3,33	0,03655	1,67	3,18	0,00530	NM_001170779	PRKCSH
8025895	1,73	3,33	0,01867	2,90	7,45	0,00208	NM_002743	MYCBP2
7972069	1,73	3,33	0,01086	2,55	5,86	0,00036	NM_015057	RGPD1
8043324	1,73	3,32	0,01340	0,87	1,82	0,17831	NM_001024457	RGPD1
8053622	1,73	3,32	0,01340	0,87	1,82	0,17831	NM_001024457	RNF6
7970681	1,73	3,32	0,06914	2,03	4,08	0,00114	NM_005977	KIAA0652
7939676	1,73	3,32	0,01370	2,75	6,73	0,00008	NR_024587	PRDM4
7966072	1,73	3,32	0,02017	2,24	4,72	0,00078	NM_012406	SFRS15
8069910	1,73	3,32	0,02333	3,59	12,04	0,00042	NM_020706	MUDENG
7974566	1,73	3,32	0,01229	2,03	4,10	0,00772	NM_018229	MYO5A
7988921	1,73	3,32	0,01223	2,68	6,40	0,00114	NM_000259	DTNA
8020847	1,73	3,32	0,00285	0,55	1,46	0,14031	NM_001390	14. Sep
7896742	1,73	3,31	0,15309	1,43	2,69	0,40078	NM_207366	14. Sep
7911347	1,73	3,31	0,15309	1,43	2,69	0,40078	NM_207366	14. Sep
7945342	1,73	3,31	0,15309	1,43	2,69	0,40078	NM_207366	14. Sep
8031990	1,73	3,31	0,15309	1,43	2,69	0,40078	NM_207366	14. Sep
8085054	1,73	3,31	0,15309	1,43	2,69	0,40078	NM_207366	14. Sep
8110670	1,73	3,31	0,15309	1,43	2,69	0,40078	NM_207366	14. Sep
8137666	1,73	3,31	0,15309	1,43	2,69	0,40078	NM_207366	14. Sep
8148964	1,73	3,31	0,15309	1,43	2,69	0,40078	NM_207366	BMP2K
8096004	1,73	3,31	0,03088	1,34	2,53	0,00363	NM_198892	CLIP1
7967255	1,73	3,31	0,01951	2,09	4,27	0,00104	NM_002956	CD55
7909332	1,73	3,31	0,00614	2,25	4,75	0,00178	NM_001114752	UQCRH
7912670	1,73	3,31	0,04788	2,54	5,83	0,00360	NM_006004	NAV1
7908694	1,73	3,31	0,01523	2,29	4,89	0,00012	NM_020443	SHFM1
8141133	1,73	3,31	0,01014	1,28	2,43	0,04475	NM_006304	EIF1AY
8176719	1,73	3,31	0,06066	2,59	6,04	0,00450	NM_004681	LMBRD1
8127425	1,72	3,31	0,09030	3,24	9,42	0,00064	NM_018368	SLC16A2
8168424	1,72	3,31	0,03673	0,95	1,93	0,01900	NM_006517	ATP5F1
7903972	1,72	3,30	0,00085	0,82	1,76	0,00436	NM_001688	KIAA0564
7971246	1,72	3,30	0,00375	2,67	6,36	0,00044	NM_015058	ZNF490
8034408	1,72	3,30	0,05850	1,39	2,61	0,00037	NM_020714	AKAP13
7985695	1,72	3,30	0,00203	2,12	4,34	0,00229	NM_006738	KATNAL1
7970844	1,72	3,30	0,04842	1,77	3,40	0,00366	NM_001014380	ATG9A
8059139	1,72	3,30	0,01882	2,46	5,51	0,00133	NM_001077198	VWA5A
7944803	1,72	3,29	0,00866	0,15	1,11	0,68031	NM_001130142	SPAST
8041236	1,72	3,29	0,01630	2,59	6,03	0,00333	NM_014946	TBCCD1
8092627	1,72	3,29	0,03467	2,36	5,13	0,02183	NM_018138	TOPORS
8160581	1,72	3,29	0,00175	1,28	2,42	0,01528	NM_005802	VPS54
8052626	1,72	3,29	0,03075	2,57	5,93	0,00042	NM_016516	APIP
7947490	1,72	3,29	0,00261	-0,27	-1,20	0,26531	NM_015957	CRISPLD1
8146967	1,72	3,29	0,02105	2,00	3,99	0,00550	NM_031461	KIAA1632
8022996	1,72	3,29	0,05008	1,78	3,43	0,00107	NM_020964	GTF2H2
8105970	1,72	3,29	0,00283	2,25	4,77	0,00121	NM_001515	RPP21
8117881	1,72	3,28	0,18776	3,98	15,74	0,00013	NM_024839	PRUNE2
8161884	1,71	3,28	0,00446	-0,30	-1,23	0,32014	NM_015225	PRC1
7991406	1,71	3,28	0,02205	3,49	11,24	0,00006	NM_003981	C13orf1
7971644	1,71	3,28	0,00200	2,16	4,46	0,00046	NM_020456	BBS7
8102655	1,71	3,28	0,05050	2,80	6,95	0,00032	NM_176824	G6PD
8176133	1,71	3,28	0,00865	2,74	6,68	0,00020	NM_000402	CIAO1
8043585	1,71	3,28	0,08000	3,54	11,65	0,00378	NM_004804	OSTM1
8128669	1,71	3,28	0,01423	1,15	2,22	0,02796	NM_014028	CLIC1
8125059	1,71	3,28	0,02133	2,07	4,19	0,00423	NM_001288	C5orf25
8110147	1,71	3,28	0,00796	1,07	2,10	0,02793	NM_198567	TSPYL1
8129099	1,71	3,28	0,01113	2,81	7,03	0,00053	NM_003309	SDHAP2
8084912	1,71	3,27	0,00066	2,95	7,71	0,00034	NR_003265	NEGR1
7916986	1,71	3,27	0,00208	-0,94	-1,91	0,00610	NM_173808	GNPNAT1
7979196	1,71	3,27	0,04280	2,54	5,82	0,00367	NM_198066	CCDC76
7903308	1,71	3,27	0,00116	1,47	2,78	0,04658	NM_019083	LARP6
7990080	1,71	3,27	0,06635	2,62	6,15	0,00085	NM_018357	PFKP

7925876	1,71	3,27	0,01751	3,19	9,14	0,00008	NM_002627	TTC39B
8160213	1,71	3,27	0,02226	1,71	3,27	0,01174	NM_152574	IL8
8095680	1,71	3,27	0,06582	0,58	1,50	0,68863	NM_000584	MXRA5
8171172	1,71	3,27	0,07083	-0,70	-1,62	0,14920	NM_015419	SESN3
7951077	1,71	3,27	0,00982	-0,13	-1,10	0,47374	NM_144665	H19
7945680	1,71	3,26	0,00879	0,34	1,27	0,46865	NR_002196	POLB
8146198	1,71	3,26	0,03664	4,26	19,19	0,00005	NM_002690	RPS6KC1
7909661	1,70	3,26	0,01646	2,78	6,89	0,00026	NM_012424	---
8044189	1,70	3,26	0,00593	0,39	1,31	0,06502	---	PPP2CA
8114158	1,70	3,26	0,07176	3,16	8,92	0,00111	NM_002715	SNRPD2
8037642	1,70	3,26	0,01607	1,18	2,27	0,05639	NM_177542	STIP1
7940857	1,70	3,26	0,03282	2,70	6,51	0,00517	NM_006819	TTC8
7976128	1,70	3,26	0,05427	1,97	3,92	0,00646	NM_144596	EDNRA
8097692	1,70	3,26	0,06579	-0,49	-1,40	0,13440	NM_001957	ZFP1
7997257	1,70	3,26	0,05138	1,61	3,05	0,02581	NM_153688	DNAJB1
8034837	1,70	3,26	0,02740	4,17	18,01	0,00259	NM_006145	FAM86B1 // FAM86B1 // FAM86B1
8149389	1,70	3,25	0,00722	3,45	10,96	0,00098	NR_003494 // NR_003494 // NR_003494	DOK4
8001564	1,70	3,25	0,00380	2,29	4,91	0,00497	NM_018110	---
7903183	1,70	3,25	0,00195	0,75	1,68	0,00659	---	POM121
8133275	1,70	3,25	0,00553	2,93	7,60	0,00264	NM_172020	GUSBP3
8177669	1,70	3,25	0,14161	1,73	3,32	0,01271	NR_027386	PPP3R1
8052721	1,70	3,25	0,13059	3,08	8,46	0,00002	NM_000945	SSBP1
8136631	1,70	3,25	0,02664	0,98	1,97	0,01928	NM_003143	POLD3
7942527	1,70	3,25	0,07656	3,34	10,11	0,00003	NM_006591	ACAD9
8082444	1,70	3,25	0,05971	2,94	7,66	0,00170	NM_014049	---
8103413	1,70	3,24	0,14485	-0,72	-1,64	0,51483	---	MRFAP1
8093936	1,70	3,24	0,15810	1,16	2,24	0,00013	NM_033296	PLCB4
8060897	1,70	3,24	0,00459	1,38	2,61	0,00417	NM_001172646	RPL21
7968226	1,70	3,24	0,22257	0,91	1,88	0,06376	NM_000982	ATL2
8051589	1,70	3,24	0,03737	2,70	6,50	0,00144	NM_022374	MLX
8007290	1,69	3,24	0,00054	2,14	4,41	0,00068	NM_170607	NAV2
7938834	1,69	3,24	0,03127	0,16	1,12	0,68451	NM_182964	WHSC1L1
8150287	1,69	3,24	0,01802	3,23	9,35	0,00399	NM_023034	WDYHV1
8148198	1,69	3,24	0,06442	3,28	9,75	0,00024	NM_018024	GLYR1
7999233	1,69	3,24	0,00653	1,79	3,45	0,00907	NM_032569	AFF1
8096224	1,69	3,23	0,03791	2,54	5,80	0,00495	NM_001166693	ERI3
7915567	1,69	3,23	0,02123	1,99	3,98	0,00012	NM_024066	EID1
7983606	1,69	3,23	0,06902	1,69	3,22	0,00087	NM_014335	KIAA1147
8143441	1,69	3,23	0,01210	3,25	9,49	0,00006	NM_001080392	C1orf52
7917331	1,69	3,23	0,02161	1,63	3,10	0,00631	NR_024113	SNX14
8127943	1,69	3,23	0,03887	3,46	11,00	0,00111	NM_153816	TSKU
7942674	1,69	3,23	0,01441	2,36	5,13	0,00628	NM_015516	GFPT2
8116418	1,69	3,23	0,01273	2,03	4,07	0,01536	NM_005110	MRPL21
7949995	1,69	3,23	0,00158	2,13	4,37	0,01902	NM_181515	RAB3A
8035398	1,69	3,23	0,05561	3,08	8,46	0,00142	NM_002866	DNAJC8
7914194	1,69	3,23	0,06810	3,13	8,74	0,00265	NM_014280	SUPT4H1
8016982	1,69	3,23	0,05541	2,62	6,16	0,00102	NM_003168	NPR2
8155121	1,69	3,23	0,00175	-0,68	-1,61	0,00495	NM_003995	---
8101210	1,69	3,22	0,00170	0,45	1,37	0,17655	---	RFWD2
7922504	1,69	3,22	0,14200	3,88	14,73	0,00010	NM_022457	ERCC6
7933509	1,69	3,22	0,00425	0,98	1,98	0,01056	NM_000124	RPL21
8131661	1,69	3,22	0,19904	0,52	1,43	0,16676	NM_000982	ZNF827
8103025	1,69	3,22	0,01532	1,86	3,64	0,01688	NM_178835	PRR11
8008784	1,69	3,22	0,01922	3,74	13,32	0,00014	NM_018304	AIMP2
8131374	1,69	3,22	0,01596	3,26	9,58	0,00008	NM_006303	UBE2G2
8070799	1,69	3,22	0,06043	2,88	7,38	0,00111	NM_182688	DENND5A
7946478	1,68	3,21	0,02408	2,48	5,59	0,00017	NM_015213	CCDC14
8090133	1,68	3,21	0,05432	1,82	3,52	0,00618	NM_022757	TIMM50
8028705	1,68	3,21	0,01054	2,52	5,73	0,00472	NM_001001563	CCDC43
8016088	1,68	3,21	0,03776	3,38	10,42	0,00003	NM_144609	DDI2
7898211	1,68	3,21	0,01567	2,59	6,03	0,00448	NM_032341	RPL21
7936727	1,68	3,21	0,21168	0,51	1,42	0,10385	NM_000982	ANAPC1
8054614	1,68	3,21	0,05806	3,67	12,71	0,00136	NM_022662	AASDH
8100478	1,68	3,21	0,03957	2,62	6,13	0,00150	NM_181806	CTCFL
8067206	1,68	3,21	0,02478	0,36	1,28	0,67145	BC137482	ABCA8
8017885	1,68	3,20	0,05979	-0,98	-1,97	0,06407	NM_007168	KITLG
7965322	1,68	3,20	0,12469	1,02	2,02	0,04260	NM_000899	RORB
8155864	1,68	3,20	0,03984	-1,07	-2,10	0,00086	NM_006914	ZNF346
8110253	1,68	3,20	0,03030	2,26	4,78	0,00133	NM_012279	ASPM
7923086	1,68	3,20	0,01579	2,40	5,29	0,01098	NM_018136	SQSTM1
8110569	1,68	3,20	0,00476	2,58	5,99	0,00131	NM_003900	FAM21B

7927323	1,68	3,20	0,01705	1,81	3,51	0,00109	NM_018232	ZNF330
8097543	1,67	3,19	0,04679	2,24	4,71	0,00035	NM_014487	GPAM
7936322	1,67	3,19	0,06137	2,54	5,81	0,00099	NM_020918	DUSP7
8087833	1,67	3,19	0,07836	2,67	6,35	0,00397	NM_001947	09. Sep
8010161	1,67	3,19	0,00989	2,63	6,19	0,00129	NM_006640	ABHD6
8080762	1,67	3,19	0,05930	2,80	6,97	0,00195	NM_020676	POLR2J3
8141791	1,67	3,19	0,03373	3,41	10,64	0,00296	NM_001097615	GPATCH8
8016077	1,67	3,19	0,00397	2,58	5,98	0,00003	NM_001002909	LBH
8041206	1,67	3,19	0,00060	-0,88	-1,84	0,00034	NM_030915	ZNF234
8029392	1,67	3,19	0,03496	2,48	5,58	0,00337	NM_006630	ELF4
8175039	1,67	3,19	0,07093	2,42	5,34	0,00478	NM_001421	TRIM8
7930139	1,67	3,19	0,07369	2,81	7,03	0,00217	NM_030912	---
8107096	1,67	3,18	0,06290	0,17	1,13	0,56976	--- FER	
8107208	1,67	3,18	0,00144	0,98	1,97	0,04798	NM_005246	PPM1A
7974781	1,67	3,18	0,00138	1,91	3,76	0,01022	NM_021003	EIF2B2
7975760	1,67	3,18	0,04654	2,98	7,89	0,00629	NM_014239	C20orf20
8063949	1,67	3,18	0,07957	2,26	4,79	0,00104	NM_018270	TMEM33
8094830	1,67	3,18	0,04508	3,13	8,77	0,00432	NM_018126	GOLPH3L
7919780	1,67	3,18	0,07623	3,76	13,57	0,00008	NM_018178	MFAP2
7912887	1,67	3,18	0,05712	2,36	5,12	0,00784	NM_002403	HDHD1A
8171222	1,67	3,18	0,03458	2,45	5,45	0,00021	NM_001135565	UBXN2B
8146544	1,67	3,18	0,10039	2,75	6,75	0,00226	NM_001077619	TTC14
8084128	1,67	3,18	0,05195	1,01	2,02	0,04171	NM_133462	FAM127C
8175299	1,67	3,17	0,06209	-1,13	-2,19	0,02681	NM_001078173	COMMD7
8065637	1,67	3,17	0,01508	1,72	3,30	0,00065	NM_053041	PPM1B
8041713	1,67	3,17	0,05729	2,62	6,16	0,00812	NM_177968	MMS19
7935474	1,67	3,17	0,02439	3,13	8,75	0,00002	NM_022362	TRIP11
7980923	1,66	3,17	0,06119	1,80	3,49	0,04049	NM_004239	HN1
8018305	1,66	3,17	0,02055	2,95	7,71	0,00109	NM_016185	---
7896746	1,66	3,17	0,03845	-1,86	-3,62	0,01605	--- NDUFA8	
8163930	1,66	3,17	0,03679	2,19	4,57	0,00008	NM_014222	RPL36A
8168843	1,66	3,17	0,04511	1,39	2,62	0,02623	NM_021029	PCID2
7972867	1,66	3,17	0,01230	1,59	3,02	0,02703	NM_001127202	KIAA1797
8154574	1,66	3,17	0,01903	2,88	7,35	0,00399	NM_017794	RNF213
8010454	1,66	3,17	0,02499	2,87	7,32	0,00015	NM_020914	CDC25B
8060675	1,66	3,16	0,02366	2,04	4,11	0,00222	NM_021873	ATG2B
7981192	1,66	3,16	0,04667	3,30	9,88	0,00008	NM_018036	KIAA1826
7951422	1,66	3,16	0,08238	2,19	4,56	0,01239	NM_032424	EDNRB
7972157	1,66	3,16	0,01224	-0,58	-1,50	0,00651	NM_001122659	KIAA0528
7961767	1,66	3,16	0,01569	3,04	8,21	0,00069	AK295862 JTB	
7920409	1,66	3,16	0,00092	2,80	6,96	0,00005	NM_006694	ZNF850P
8036351	1,66	3,16	0,01804	1,42	2,68	0,02415	BC052603 LDOC1	
8175539	1,66	3,16	0,04726	3,21	9,23	0,00037	NM_012317	HMBOX1
8145636	1,66	3,16	0,05803	2,91	7,52	0,00054	NM_024567	TRIM25
8016847	1,66	3,16	0,02272	3,23	9,35	0,00044	NM_005082	C1GALT1
8131475	1,66	3,16	0,01505	2,63	6,19	0,00987	NM_020156	NSUN2
8110894	1,66	3,15	0,03720	2,91	7,49	0,00020	NM_017755	SUMO3
8070811	1,66	3,15	0,07002	3,16	8,95	0,00021	NM_006936	---
7974481	1,66	3,15	0,25749	0,51	1,43	0,25252	--- MRPS16	
7934326	1,66	3,15	0,00465	1,72	3,29	0,00905	NM_016065	LIN54
8101411	1,66	3,15	0,00273	1,07	2,10	0,04401	NM_194282	TEX264
8080121	1,66	3,15	0,00038	1,40	2,65	0,02142	NM_015926	C15orf23
7982712	1,66	3,15	0,01007	2,79	6,90	0,00055	NM_033286	PRKG2
8101284	1,65	3,15	0,06779	0,45	1,37	0,22926	NM_006259	LCOR
7929596	1,65	3,14	0,09295	2,12	4,34	0,01895	NM_032440	TNFRSF21
8126839	1,65	3,14	0,01136	2,65	6,27	0,00279	NM_014452	CDKAL1
8117140	1,65	3,14	0,04051	2,84	7,16	0,00030	NM_017774	SAP130
8055104	1,65	3,14	0,04866	3,43	10,79	0,00021	NM_001145928	ADA
8066431	1,65	3,14	0,00009	2,68	6,41	0,00306	NM_000022	SNX9
8122986	1,65	3,14	0,05548	2,78	6,85	0,00029	NM_016224	MEIS3P1
8005132	1,65	3,14	0,13743	3,83	14,21	0,00003	NR_002211	ZCCHC11
7916185	1,65	3,14	0,01926	3,03	8,15	0,00226	NM_001009881	PLAGL2
8065607	1,65	3,13	0,08773	3,00	8,01	0,00089	NM_002657	TSHZ3
8035896	1,65	3,13	0,11926	-1,45	-2,74	0,00055	NM_020856	IL4R
7994280	1,65	3,13	0,01591	1,69	3,23	0,01535	NM_000418	C6orf138
8126853	1,65	3,13	0,05064	-1,69	-3,23	0,06543	NM_001013732	AKT2
8036840	1,65	3,13	0,00386	3,03	8,15	0,00091	NM_001626	LOC171220
7953518	1,65	3,13	0,01075	1,62	3,06	0,00711	U72518 DTWD2	
8113691	1,65	3,13	0,02553	2,28	4,85	0,00043	NM_173666	MMP19
7956038	1,65	3,13	0,06271	2,07	4,19	0,00359	NM_002429	WASF3
7968212	1,64	3,13	0,00725	1,99	3,98	0,00089	NM_006646	ZNF583
8031640	1,64	3,13	0,04518	0,37	1,30	0,52666	NM_152478	SNHG12
7914202	1,64	3,13	0,01144	4,69	25,87	0,00001	AY277594 RPS6KA2	

8130739	1,64	3,13	0,00185	2,59	6,03	0,00258	NM_021135	DAAM1
7974697	1,64	3,12	0,00781	2,24	4,71	0,00420	NM_014992	RGMB
8107100	1,64	3,12	0,01043	1,22	2,32	0,00051	NM_001012761	ZNF738
8027297	1,64	3,12	0,09817	2,82	7,07	0,00247	NR_027130	DPCD
7929976	1,64	3,12	0,08046	1,11	2,16	0,08107	NM_015448	DNAJB6
8137627	1,64	3,12	0,04456	0,64	1,56	0,39405	NM_058246	DDX19A
7997059	1,64	3,12	0,00668	0,92	1,90	0,02956	NM_018332	ARSJ
8102440	1,64	3,12	0,00505	2,80	6,96	0,00137	NM_024590	PRKDC
8150599	1,64	3,12	0,01264	2,54	5,82	0,01382	NM_006904	CCDC56
8015712	1,64	3,12	0,01934	2,32	5,00	0,00102	NM_001040431	ASNS
8141150	1,64	3,12	0,04128	2,69	6,47	0,00081	NM_133436	ISCA1
8162142	1,64	3,12	0,07051	0,66	1,59	0,24661	NM_030940	NAV3
7957298	1,64	3,12	0,03393	0,45	1,37	0,04803	NM_014903	CD97
8026300	1,64	3,12	0,06426	2,57	5,94	0,02952	NM_078481	CHST15
7936856	1,64	3,12	0,03502	1,82	3,53	0,02273	NM_015892	CRISPLD2
7997642	1,64	3,11	0,00521	0,03	1,02	0,87528	NM_031476	WDFY3
8101511	1,64	3,11	0,01438	3,37	10,32	0,00015	NM_014991	COL15A1
8156783	1,64	3,11	0,01945	-1,73	-3,33	0,00898	NM_001855	PEG10
8134339	1,64	3,11	0,07792	4,09	17,00	0,00485	NM_001040152	VPS52
8180123	1,64	3,11	0,01671	3,02	8,13	0,00054	NM_022553	ZCCHC7
8155234	1,63	3,10	0,01601	0,80	1,75	0,21454	NM_032226	PEX12
8014264	1,63	3,10	0,12172	2,53	5,77	0,00003	NM_000286	PCGF2
8014706	1,63	3,10	0,00217	1,98	3,94	0,00064	NM_007144	C14orf129
7976571	1,63	3,10	0,03006	2,91	7,53	0,00291	NM_016472	VPS28
8153776	1,63	3,10	0,08811	2,72	6,58	0,00098	NM_183057	CSNK1A1
8115022	1,63	3,10	0,10477	3,46	10,99	0,00066	NM_001025105	TPR
7922912	1,63	3,10	0,01342	2,27	4,81	0,00271	NM_003292	COX7B
8168470	1,63	3,10	0,02726	-0,37	-1,29	0,38587	NM_001866	CFI
8102328	1,63	3,10	0,04137	1,00	1,99	0,00480	NM_000204	UVRAG
7942626	1,63	3,10	0,02641	4,04	16,46	0,00006	NM_003369	TECR
8101622	1,63	3,10	0,09035	3,79	13,80	0,00050	NM_138501	RABL3
8089867	1,63	3,10	0,06900	3,04	8,24	0,00457	NM_173825	RAD51C
8008754	1,63	3,10	0,00577	2,18	4,54	0,00315	NM_058216	ZNF28
8038993	1,63	3,10	0,03902	2,49	5,63	0,00313	NM_006969	C22orf13
8075009	1,63	3,10	0,01003	2,37	5,18	0,00012	NM_031444	NFS1
8065992	1,63	3,09	0,01520	3,93	15,26	0,00067	NM_021100	AGK
8136614	1,63	3,09	0,00067	2,59	6,02	0,00057	NM_018238	SNRNP25
7991735	1,63	3,09	0,02455	3,27	9,62	0,00180	NM_024571	ANKRD50
8102720	1,63	3,09	0,03448	2,15	4,43	0,02003	NM_020337	AGL
7903239	1,63	3,09	0,00680	2,82	7,07	0,00153	NM_000028	IKBKAP
8163023	1,63	3,09	0,02430	3,53	11,52	0,00000	NM_003640	ZNF286B
8013305	1,63	3,09	0,00955	2,34	5,07	0,00942	NM_001145045	CD276
7984743	1,63	3,09	0,02799	1,57	2,96	0,01035	NM_001024736	ZNF682
8035782	1,63	3,09	0,06173	-0,29	-1,22	0,14631	NM_033196	GBA2
8161114	1,63	3,09	0,09616	3,32	10,01	0,00007	NM_020944	CCDC47
8017421	1,63	3,09	0,03882	2,68	6,41	0,00012	NM_020198	ZNF25
7933105	1,62	3,08	0,12952	-0,29	-1,23	0,44004	NM_145011	SEC61G
8139706	1,62	3,08	0,01658	0,13	1,10	0,48891	NM_014302	ZNF655
8134631	1,62	3,08	0,01911	1,18	2,26	0,02730	NM_138494	TUBB6
8020220	1,62	3,08	0,02867	0,62	1,53	0,37710	NM_032525	ATXN7L1
8142098	1,62	3,08	0,06448	2,48	5,60	0,02387	NM_020725	ZMYM3
8173457	1,62	3,08	0,00507	2,83	7,13	0,00004	NM_201599	STAT6
7964360	1,62	3,08	0,00077	1,91	3,76	0,01116	NM_003153	TP53BP1
7988093	1,62	3,08	0,06530	2,47	5,53	0,00027	NM_001141980	PMS1
8047038	1,62	3,08	0,05799	3,01	8,04	0,00054	NM_000534	TBCA
8112767	1,62	3,08	0,00558	0,60	1,51	0,21409	NM_004607	BRWD1
8070341	1,62	3,08	0,03353	2,54	5,80	0,01268	NM_033656	GPR89A
7904853	1,62	3,08	0,01860	2,96	7,79	0,00303	NM_001097612	TKT
8088106	1,62	3,08	0,02753	2,68	6,43	0,00017	NM_001135055	FCHO2
8106141	1,62	3,08	0,05486	1,59	3,01	0,00173	NM_138782	JRKL
7943314	1,62	3,07	0,08790	1,21	2,31	0,03471	NM_003772	RCN1
7968928	1,62	3,07	0,08899	2,15	4,45	0,00136	NM_002901	PHLPP2
8002571	1,62	3,07	0,02646	2,71	6,54	0,00081	NM_015020	RTF1
7982904	1,62	3,07	0,05809	2,81	7,00	0,00087	NM_015138	GNA14
8161892	1,62	3,07	0,03204	0,63	1,55	0,14354	NM_004297	USF1
7921738	1,62	3,07	0,02071	1,23	2,34	0,03131	NM_007122	PPP1R8
7899377	1,62	3,07	0,03003	1,96	3,89	0,01741	NM_138558	CFLAR
8047381	1,62	3,07	0,00976	2,09	4,24	0,00015	NM_003879	MTMR1
8170443	1,62	3,07	0,00476	2,08	4,24	0,00962	NM_003828	GMEB1
7899504	1,62	3,07	0,02918	2,97	7,86	0,00007	NM_006582	---
8093256	1,62	3,07	0,02444	1,61	3,05	0,02111	--- OSBP	
7948379	1,62	3,07	0,01225	1,12	2,18	0,00215	NM_002556	UPRT
8168438	1,62	3,07	0,02074	1,15	2,22	0,00070	NM_145052	HSPB8

7959102	1,62	3,07	0,02415	0,09	1,06	0,53779	NM_014365	RNF103
8053576	1,62	3,07	0,00252	1,74	3,35	0,00327	NM_005667	FOLH1
7939897	1,62	3,06	0,33262	1,00	2,00	0,05777	NM_004476	ARHGAP24
8096160	1,62	3,06	0,02259	-0,36	-1,29	0,44387	NM_001025616	ATG10
8106709	1,62	3,06	0,01493	1,27	2,41	0,06630	NM_031482	FAM21A
7927560	1,62	3,06	0,02274	1,89	3,72	0,00027	NM_001005751	NEDD1
7957715	1,61	3,06	0,00959	3,19	9,16	0,00146	NM_001135175	VPS52
8125649	1,61	3,06	0,01301	2,83	7,13	0,00044	NM_022553	VPS52
8178917	1,61	3,06	0,01301	2,83	7,13	0,00044	NM_022553	ELOF1
8034299	1,61	3,06	0,00519	1,20	2,30	0,08613	NM_032377	MAP1A
7983228	1,61	3,06	0,01527	-1,57	-2,97	0,02207	NM_002373	NOL8
8162352	1,61	3,06	0,01562	1,79	3,45	0,00358	NR_024020	---
8171024	1,61	3,06	0,04157	1,68	3,21	0,01019	---	CCNH
8113010	1,61	3,06	0,02898	1,46	2,74	0,02117	NM_001239	RPLP1
7984562	1,61	3,05	0,01759	2,43	5,37	0,00075	NM_001003	AIFM1
8175052	1,61	3,05	0,00853	2,80	6,94	0,00008	NM_001130847	SMARCE1
8015039	1,61	3,05	0,00972	1,64	3,12	0,00653	NM_003079	CEP120
8113733	1,61	3,05	0,01559	1,33	2,51	0,03694	NM_153223	FAM86B1
8149387	1,61	3,05	0,11716	2,31	4,97	0,04878	NR_003494	ZAK
8046461	1,61	3,05	0,04611	2,67	6,36	0,00046	NM_133646	GNPTG
7992255	1,61	3,05	0,03131	1,12	2,17	0,08133	NM_032520	ABCA1
8162940	1,61	3,05	0,01517	1,58	2,99	0,00305	NM_005502	UFM1
7968670	1,61	3,04	0,06782	1,26	2,39	0,05920	NM_016617	DRAP1
7941460	1,61	3,04	0,04269	2,20	4,61	0,02446	NM_006442	CHMP1B
8020179	1,61	3,04	0,05935	2,82	7,08	0,00141	NM_020412	ELF2
8102817	1,61	3,04	0,09237	2,16	4,46	0,00077	NM_201999	NETO2
8001197	1,61	3,04	0,08188	4,44	21,67	0,00547	NM_018092	ZFAND6
7985259	1,60	3,04	0,09348	3,20	9,19	0,00281	NM_019006	MST131
8145766	1,60	3,04	0,03787	-0,48	-1,40	0,44635	ENST00000423322	CDK19
8128867	1,60	3,04	0,03893	1,58	2,98	0,01706	NM_015076	FAM86B1
8149365	1,60	3,03	0,01845	3,27	9,63	0,00279	NR_003494	TANC1
8045889	1,60	3,03	0,02153	2,69	6,47	0,01273	NM_033394	ANKRD13C
7916928	1,60	3,03	0,05201	2,23	4,70	0,00227	NM_030816	S100A4
7920271	1,60	3,03	0,04957	-0,45	-1,36	0,47423	NM_019554	ATRN
8060627	1,60	3,03	0,02113	2,94	7,66	0,00006	NM_139321	ALDH2
7958784	1,60	3,03	0,03945	3,19	9,13	0,00443	NM_000690	PPIL3
8058147	1,60	3,03	0,08165	3,52	11,50	0,00116	NM_130906	VCPIP1
8151118	1,60	3,03	0,06523	0,89	1,85	0,00014	NM_025054	TAF15
8006573	1,60	3,03	0,00297	2,84	7,15	0,00007	NM_139215	PCNA
8064844	1,60	3,03	0,03020	1,86	3,63	0,00495	NM_002592	RPL18AP3
7958197	1,60	3,03	0,00495	1,20	2,29	0,00557	NR_001593	APLF
8042402	1,60	3,02	0,04580	1,29	2,45	0,02190	NM_173545	PLA2G2A
7913216	1,60	3,02	0,10555	-1,66	-3,16	0,00565	NM_000300	UBQLN2
8167924	1,60	3,02	0,02005	1,65	3,14	0,00384	NM_013444	MICB
8177955	1,60	3,02	0,03996	4,22	18,65	0,00001	NM_005931	TMEM54
7914592	1,60	3,02	0,00032	2,37	5,17	0,00503	NM_033504	RSL1D1
7999520	1,59	3,02	0,04419	2,96	7,78	0,00060	NM_015659	RNF10
7959173	1,59	3,02	0,03065	2,71	6,52	0,00209	NM_014868	PTGS1
8157650	1,59	3,02	0,00023	-0,60	-1,51	0,03289	NM_000962	ZNF518A
7929562	1,59	3,02	0,00994	3,25	9,49	0,00073	NM_014803	ALKBH3
7939477	1,59	3,02	0,01272	2,26	4,78	0,00384	NM_139178	SFPQ
7914791	1,59	3,02	0,03299	2,19	4,58	0,02557	NM_005066	MAPK3
8000811	1,59	3,02	0,04919	2,61	6,11	0,00078	NM_001040056	PYGO1
7989069	1,59	3,02	0,02295	0,99	1,99	0,01322	NM_015617	TXNDC12
7916120	1,59	3,01	0,04388	3,40	10,58	0,00246	NM_015913	WDR33
8055060	1,59	3,01	0,00589	2,27	4,82	0,00029	NM_018383	ZNF765
8030982	1,59	3,01	0,00530	0,61	1,52	0,01531	NM_001040185	NFX1
8154793	1,59	3,01	0,00176	2,26	4,80	0,00123	NM_002504	RASSF8
7954469	1,59	3,01	0,00419	-0,19	-1,14	0,26900	NM_007211	MED17
7943176	1,59	3,01	0,02982	2,94	7,65	0,00104	NM_004268	SCNM1
7905355	1,59	3,01	0,00955	1,78	3,44	0,00003	NM_024041	TMEM154
8103226	1,59	3,01	0,00801	0,61	1,52	0,03978	NM_152680	IGFBP6
7955694	1,59	3,01	0,00089	-1,01	-2,02	0,01366	NM_002178	LRIG1
8088642	1,59	3,01	0,08691	3,02	8,12	0,00215	NM_015541	SPOPL
8045514	1,59	3,01	0,05987	2,30	4,92	0,00020	NM_001001664	ENG
8164269	1,59	3,01	0,00834	-1,12	-2,17	0,00553	NM_000118	MTOR
7912412	1,59	3,01	0,04833	3,22	9,32	0,00014	NM_004958	YTHDC1
8100714	1,59	3,01	0,02271	2,92	7,58	0,00087	NM_001031732	GRPEL1
8099246	1,59	3,00	0,01218	3,97	15,62	0,00007	NM_025196	RWDD3
7903171	1,59	3,00	0,07623	0,11	1,08	0,55803	NM_015485	ZDHHC6
7936346	1,59	3,00	0,09784	3,75	13,49	0,00018	NM_022494	BNIP1
8110043	1,58	3,00	0,02838	1,28	2,42	0,00606	NM_013979	EIF1B
8078962	1,58	3,00	0,01499	0,77	1,71	0,18967	NM_005875	PHB

8016552	1,58	3,00	0,00126	2,55	5,84	0,00161	NM_002634	RAB6C
8045136	1,58	3,00	0,01513	1,51	2,86	0,02692	NM_032144	ZBTB41
7923119	1,58	3,00	0,03382	3,02	8,11	0,00488	NM_194314	IER3IP1
8023175	1,58	3,00	0,03440	1,52	2,88	0,00002	NM_016097	LRRC37B2
8006170	1,58	2,99	0,02708	3,38	10,42	0,00033	NR_015341	RIF1
8045697	1,58	2,99	0,04887	3,02	8,13	0,01065	NM_018151	ZNF354A
8116247	1,58	2,99	0,00526	2,16	4,47	0,02965	NM_005649	FBXW7
8103206	1,58	2,99	0,04743	1,32	2,49	0,00496	NM_033632	IDH3G
8175844	1,58	2,99	0,01407	2,10	4,29	0,01341	NM_174869	SLC39A1
7920401	1,58	2,99	0,03932	3,38	10,41	0,00007	NM_014437	SLC25A5
8169640	1,58	2,99	0,04540	4,21	18,54	0,00017	NM_001152	SFRS13A
7913712	1,58	2,98	0,00341	1,00	2,00	0,02633	NM_006625	NOL10
8050255	1,58	2,98	0,01164	2,13	4,37	0,00557	NM_024894	NAF1
8103485	1,58	2,98	0,04055	1,78	3,44	0,00209	NM_138386	C7orf59
8134740	1,58	2,98	0,02191	1,86	3,62	0,00386	NM_001008395	NECAP2
7898337	1,58	2,98	0,03580	2,49	5,63	0,00081	NM_018090	TRIT1
7915261	1,58	2,98	0,04243	2,89	7,41	0,00040	NM_017646	TSGA14
8142930	1,58	2,98	0,04282	1,55	2,93	0,00477	NM_018718	LEMD3
7956842	1,58	2,98	0,02762	2,60	6,06	0,00256	NM_014319	RPS26
7956114	1,57	2,98	0,01295	0,55	1,46	0,09105	NM_001029	STYX
7974387	1,57	2,98	0,04907	2,63	6,18	0,00144	NM_145251	TBC1D13
8158406	1,57	2,98	0,00690	2,16	4,46	0,00694	NM_018201	FGD6
7965541	1,57	2,97	0,02038	2,84	7,16	0,01204	NM_018351	MORC3
8068460	1,57	2,97	0,05135	2,34	5,05	0,00594	NM_015358	HERC2P2
7986701	1,57	2,97	0,01134	2,98	7,89	0,00676	NR_002824	MAK16
8145782	1,57	2,97	0,09546	3,20	9,17	0,00002	NM_032509	RPS26
8102410	1,57	2,97	0,00970	0,43	1,34	0,15657	NM_001029	C6orf129
8126095	1,57	2,97	0,06491	2,73	6,64	0,00076	NM_138493	SEC11A
7991143	1,57	2,97	0,00809	1,03	2,04	0,15122	NM_014300	TGFBRAP1
8054364	1,57	2,97	0,03324	3,47	11,06	0,00087	NM_004257	YBX1P2
8142084	1,57	2,97	0,00701	1,05	2,07	0,09409	CR601484 RFT1	
8088092	1,57	2,97	0,03016	3,05	8,26	0,00002	NM_052859	UBL7
7990361	1,57	2,97	0,00162	1,56	2,96	0,00180	NM_032907	---
7947423	1,57	2,97	0,37607	-1,78	-3,43	0,30213	--- CEP192	
8020267	1,57	2,97	0,02095	2,89	7,42	0,00850	NM_032142	SNRNP40
7914334	1,57	2,97	0,15940	2,33	5,04	0,06312	NM_004814	CDIPT
8000706	1,57	2,97	0,00459	2,02	4,05	0,02533	NM_006319	---
8110914	1,57	2,97	0,08350	1,12	2,17	0,00281	--- SPRY2	
7972217	1,57	2,96	0,01486	2,70	6,51	0,00162	NM_005842	---
8161169	1,57	2,96	0,00156	1,16	2,23	0,03780	--- PLEKHM3	
8058509	1,57	2,96	0,02733	2,11	4,31	0,00336	NM_001080475	C3orf26
8081241	1,57	2,96	0,07381	3,55	11,74	0,00080	NM_032359	DENND1B
7923141	1,57	2,96	0,00953	3,05	8,28	0,00089	NM_144977	COG4
8002381	1,57	2,96	0,01437	1,98	3,94	0,00023	NM_015386	HERC2P2
7986569	1,57	2,96	0,02184	2,75	6,73	0,00752	NR_002824	CFTR
8135661	1,57	2,96	0,05251	-1,03	-2,04	0,01572	NM_000492	NCOA3
8063211	1,57	2,96	0,00874	3,80	13,93	0,00019	NM_181659	HLA-E
8177788	1,57	2,96	0,00776	2,76	6,79	0,00521	NM_005516	HLA-E
8179103	1,57	2,96	0,00776	2,76	6,79	0,00521	NM_005516	COQ5
7967072	1,56	2,96	0,06758	3,31	9,92	0,00003	NM_032314	GK
8166632	1,56	2,96	0,05745	1,79	3,45	0,00298	NM_001128127	SUPT6H
8005903	1,56	2,95	0,01892	2,29	4,89	0,00069	NM_003170	TNPO2
8034482	1,56	2,95	0,03278	2,98	7,87	0,00109	NM_001136196	PSAT1
8156043	1,56	2,95	0,01915	4,74	26,73	0,00019	NM_058179	MAN2B2
8093916	1,56	2,95	0,00109	1,49	2,81	0,01474	NM_015274	TMEM126B
7942879	1,56	2,95	0,02464	1,75	3,35	0,00928	NM_018480	HERC1
7989516	1,56	2,95	0,01331	1,64	3,12	0,00035	NM_003922	C4orf27
8103684	1,56	2,95	0,00106	0,15	1,11	0,83763	NM_017867	DENND2C
7918749	1,56	2,95	0,06661	-0,46	-1,37	0,06751	BC063894 HSD17B6	
7956271	1,56	2,95	0,02637	1,58	2,98	0,00016	NM_003725	RPL12
8063473	1,56	2,95	0,05346	1,35	2,55	0,00022	NM_000976	METTL1
8171876	1,56	2,95	0,11489	1,02	2,03	0,07094	NM_005371	PDPR
7997025	1,56	2,95	0,01162	1,65	3,13	0,10829	NM_017990	PTGS2
7922976	1,56	2,95	0,02053	0,47	1,39	0,29579	NM_000963	MAD2L1BP
8119874	1,56	2,94	0,00527	2,93	7,60	0,00277	NM_014628	PSTPIP2
8023043	1,56	2,94	0,02207	1,01	2,01	0,01244	NM_024430	GON4L
7920810	1,56	2,94	0,05049	2,36	5,13	0,00409	NM_001037533	PTPLAD2
8160346	1,56	2,94	0,04313	0,39	1,31	0,06425	NM_001010915	PDE8A
7985662	1,56	2,94	0,05782	4,65	25,07	0,00011	NM_002605	---
8106473	1,56	2,94	0,01253	-0,15	-1,11	0,80893	--- SLC39A14	
8145122	1,56	2,94	0,01651	2,70	6,51	0,00035	NM_001128431	HNRNPUL1
8029029	1,56	2,94	0,03204	3,04	8,25	0,00171	NM_007040	KIAA0947
8104350	1,56	2,94	0,00137	2,35	5,09	0,00383	NM_015325	WWC2

8098470	1,56	2,94	0,01125	1,93	3,81	0,00726	NM_024949	BRD2
8118580	1,55	2,94	0,08311	2,67	6,37	0,00354	NM_005104	ELK3
7957665	1,55	2,94	0,09376	2,59	6,01	0,00192	NM_005230	SMG1
8000823	1,55	2,93	0,01612	2,98	7,91	0,00921	NM_015092	FARS2
8116724	1,55	2,93	0,04307	2,34	5,05	0,00199	NM_006567	GORAB
7907213	1,55	2,93	0,12297	1,71	3,27	0,00200	NM_152281	NUP54
8101143	1,55	2,93	0,00598	2,08	4,23	0,00148	NM_017426	TMCC1
8090630	1,55	2,93	0,01012	1,93	3,80	0,03581	NM_001017395	NUMB
7980005	1,55	2,93	0,01640	2,62	6,14	0,00043	NM_001005743	MXRA8
7911529	1,55	2,93	0,21298	-2,34	-5,07	0,00023	NM_032348	ATPBD4
7987369	1,55	2,93	0,05632	2,96	7,77	0,00300	NM_080650	PLEKHM1P
8017675	1,55	2,93	0,02194	2,91	7,54	0,00425	NR_024386	USP45
8128409	1,55	2,93	0,03040	2,38	5,22	0,00532	NM_001080481	PGAP1
8057959	1,55	2,93	0,02351	1,82	3,54	0,01429	NM_024989	SPATA5
8097262	1,55	2,93	0,05733	2,30	4,92	0,00117	NM_145207	PRAF2
8172531	1,55	2,93	0,11491	0,88	1,84	0,02861	NM_007213	TRIM23
8112388	1,55	2,93	0,03140	0,84	1,79	0,10611	NM_001656	TAOK3
7966851	1,55	2,93	0,01858	2,24	4,73	0,00307	NM_016281	YIPF3
8126574	1,55	2,92	0,02835	2,03	4,08	0,00040	NM_015388	MKX
7932733	1,55	2,92	0,00690	-0,26	-1,20	0,25382	NM_173576	LRRC59
8016708	1,55	2,92	0,01249	2,27	4,84	0,00737	NM_018509	NFKB1
8096635	1,55	2,92	0,07908	3,81	14,06	0,00017	NM_003998	PAPOLG
8042119	1,54	2,92	0,08589	3,06	8,34	0,00220	NM_022894	MSRB3
7956856	1,54	2,91	0,18562	0,84	1,79	0,10776	NM_001031679	EPM2AIP1
8086141	1,54	2,91	0,09650	0,73	1,66	0,13341	NM_014805	SEC11C
8021453	1,54	2,91	0,02467	2,23	4,69	0,00492	NM_033280	SERF2
7983290	1,54	2,91	0,00062	0,42	1,34	0,11401	NM_001018108	ZNF627
8025927	1,54	2,91	0,04947	2,04	4,11	0,00716	NM_145295	NR3C1
8114814	1,54	2,91	0,01005	2,79	6,93	0,00035	NM_000176	RPS27A
7908407	1,54	2,91	0,01884	1,17	2,25	0,00775	NM_002954	SLC5A3
8068353	1,54	2,91	0,01051	2,23	4,71	0,00183	NM_006933	CITED2
8129937	1,54	2,91	0,04470	2,55	5,87	0,00169	NM_006079	FMR1
8170326	1,54	2,91	0,00718	2,47	5,55	0,00125	NM_002024	KIAA1467
7954077	1,54	2,90	0,04258	3,66	12,66	0,00001	NM_020853	HMGN3
8127743	1,54	2,90	0,00350	1,76	3,38	0,00872	NM_004242	ZNF343
8064514	1,54	2,90	0,00679	0,50	1,42	0,11441	NM_024325	PREB
8051012	1,54	2,90	0,04456	2,32	4,99	0,00065	NM_013388	CHD4
7960594	1,54	2,90	0,00138	1,75	3,36	0,00078	NM_001273	HNRNPH1
8116297	1,54	2,90	0,09066	3,58	11,99	0,00001	NM_005520	ZNF426
8033780	1,54	2,90	0,17633	3,00	7,98	0,00023	NM_024106	PHIP
8127698	1,53	2,90	0,00069	1,38	2,60	0,03315	NM_017934	PSG5
8037272	1,53	2,90	0,07141	-1,83	-3,54	0,00638	NM_002781	MAPRE3
8040742	1,53	2,89	0,08460	2,28	4,87	0,00600	NM_012326	C10orf88
7936817	1,53	2,89	0,07911	2,18	4,54	0,01758	NM_024942	ATG5
8128592	1,53	2,89	0,04699	1,76	3,38	0,00779	NM_004849	POGZ
7920000	1,53	2,89	0,00099	2,18	4,54	0,00818	NM_015100	BRCC3
8171006	1,53	2,89	0,03249	2,04	4,12	0,00760	NM_024332	SHMT2
7956401	1,53	2,89	0,02449	4,46	21,94	0,00064	NM_005412	FUT8
7975136	1,53	2,89	0,01597	2,56	5,90	0,00074	NM_178155	BOD1L
8099410	1,53	2,89	0,00756	1,50	2,83	0,00322	NM_148894	SMAD1
8097657	1,53	2,89	0,08241	1,56	2,95	0,01290	NM_005900	GSPT2
8167654	1,53	2,89	0,01996	1,36	2,57	0,00755	NM_018094	KIAA0391
7973924	1,53	2,88	0,10782	2,83	7,13	0,00214	NM_014672	GGPS1
7910640	1,53	2,88	0,00162	0,76	1,69	0,26101	NM_004837	H1F0
8072926	1,53	2,88	0,01073	1,18	2,27	0,15997	NM_005318	CERCAM
8158250	1,53	2,88	0,00947	2,63	6,20	0,00355	NM_016174	RBM26
7972190	1,53	2,88	0,00461	2,06	4,18	0,01415	NM_022118	AGPHD1
7985192	1,53	2,88	0,00539	2,99	7,96	0,00289	NM_001013619	ITGB1BP1
8050176	1,53	2,88	0,14354	3,49	11,27	0,00082	NM_004763	GNL3
8080419	1,53	2,88	0,05944	1,83	3,55	0,00881	NM_206825	WDR61
7990700	1,53	2,88	0,03409	1,27	2,42	0,00816	NM_025234	CUL5
7943580	1,53	2,88	0,03931	3,18	9,05	0,00006	NM_003478	ECT2
8083941	1,52	2,88	0,01287	3,44	10,87	0,00929	NM_018098	TAF12
7914218	1,52	2,88	0,02565	1,96	3,88	0,00478	NM_001135218	HSPA1A
8118310	1,52	2,88	0,01262	2,88	7,36	0,00230	NM_005345	ALDH1A3
7986446	1,52	2,88	0,04785	0,56	1,47	0,18581	NM_000693	HNRNPA2B1
8138670	1,52	2,87	0,03360	1,92	3,78	0,00013	NM_031243	RBM24
8117045	1,52	2,87	0,03756	1,79	3,46	0,00012	NM_001143942	RPS26P11
8168357	1,52	2,87	0,00451	-0,34	-1,27	0,25039	NR_002309	MMP16
8151684	1,52	2,87	0,05007	-2,16	-4,47	0,00377	AL136588 LRRC49	
7984569	1,52	2,87	0,09243	1,49	2,81	0,03133	NM_017691	FBXO5
8130374	1,52	2,87	0,03616	1,88	3,68	0,02038	NM_012177	PKNOX1
8068866	1,52	2,87	0,03004	1,60	3,04	0,02493	NM_004571	GLCE

7984517	1,52	2,87	0,06077	2,83	7,09	0,00197	NM_015554	UBFD1
7994095	1,52	2,87	0,03423	1,41	2,66	0,00401	NM_019116	PISD
8075529	1,52	2,87	0,00013	1,78	3,45	0,02632	NM_014338	PTPRA
8060539	1,52	2,87	0,00354	0,61	1,53	0,02462	NM_002836	---
7902306	1,52	2,87	0,02444	0,31	1,24	0,62522	--- MYD88	
8078729	1,52	2,87	0,07371	3,03	8,16	0,00040	NM_001172567	LYRM4
8123728	1,52	2,87	0,03317	2,47	5,55	0,00014	NM_020408	AKT1
7981494	1,52	2,87	0,02210	3,37	10,36	0,00182	NM_001014432	GUSBP3
8124469	1,52	2,86	0,01812	2,57	5,93	0,00085	NR_027386	CNPY4
8134730	1,52	2,86	0,10371	0,59	1,50	0,24328	NM_152755	C10orf28
7929719	1,52	2,86	0,03783	1,31	2,49	0,01622	NM_014472	POM121
8133331	1,52	2,86	0,00009	2,05	4,14	0,00023	NM_172020	SEC24C
7928369	1,52	2,86	0,03513	2,16	4,47	0,00003	NM_004922	TPT1
7971375	1,52	2,86	0,00162	1,70	3,25	0,03075	NM_003295	KIAA0232
8093961	1,51	2,86	0,01453	1,25	2,38	0,00104	NM_014743	CNOT6L
8101228	1,51	2,86	0,14798	2,82	7,05	0,00202	NM_144571	ZNF397
8020889	1,51	2,86	0,00776	0,74	1,67	0,16294	NM_001135178	ZNF638
8042601	1,51	2,86	0,02201	2,25	4,76	0,00224	NM_014497	ZNF398
8137091	1,51	2,86	0,01117	1,41	2,65	0,00041	NM_170686	JMJD6
8018793	1,51	2,85	0,03146	2,07	4,19	0,00261	NM_001081461	ZNF56
8027239	1,51	2,85	0,01289	0,95	1,93	0,06095	ENST00000341262	PTPN2
8022393	1,51	2,85	0,01975	2,07	4,19	0,02537	NM_002828	MPZL1
7907092	1,51	2,85	0,00659	2,44	5,42	0,00001	NM_003953	EPB41
7899534	1,51	2,85	0,05643	2,03	4,09	0,00038	NM_001166005	POTEF
8045321	1,51	2,85	0,03877	0,45	1,37	0,10741	NM_001099771	ALG5
7971027	1,51	2,85	0,07764	1,67	3,18	0,00116	NM_013338	IQCB1
8089954	1,51	2,85	0,05579	2,41	5,33	0,00070	NM_001023570	NCRNA00219
8107321	1,51	2,85	0,01587	2,48	5,58	0,00718	NR_015370	PLAA
8160487	1,51	2,85	0,00078	2,42	5,37	0,00082	NM_001031689	SRRM2
7992692	1,51	2,85	0,04165	2,22	4,65	0,02173	NM_016333	MYO9A
7990092	1,51	2,85	0,04467	3,15	8,87	0,00088	NM_006901	GSTO1
7930304	1,51	2,85	0,05737	1,67	3,18	0,00608	NM_004832	---
8127423	1,51	2,85	0,22301	-3,53	-11,54	0,05426	--- ---	
7932209	1,51	2,85	0,12340	1,20	2,30	0,10247	--- CDK13	
8132417	1,51	2,85	0,01938	2,89	7,41	0,00001	NM_003718	HSPA1A
8179322	1,51	2,84	0,01298	2,72	6,59	0,00196	NM_005345	CTH
7902290	1,51	2,84	0,12376	4,93	30,47	0,00048	NM_001902	ETNK2
7923596	1,51	2,84	0,13394	1,87	3,66	0,00020	NM_018208	LARP1
8109438	1,51	2,84	0,01174	2,12	4,34	0,00357	NM_015315	ATR
8091190	1,51	2,84	0,01395	2,77	6,83	0,00721	NM_001184	CFHR1
7908488	1,50	2,84	0,00061	-0,90	-1,87	0,08900	NM_002113	RFTN1
8085665	1,50	2,84	0,22085	0,16	1,11	0,54801	NM_015150	MTA3
8041617	1,50	2,84	0,02217	2,11	4,32	0,00771	NM_020744	C10orf76
7935951	1,50	2,84	0,00491	2,60	6,07	0,00053	NM_024541	NPIP
7993349	1,50	2,84	0,15020	2,53	5,79	0,00006	NM_006985	SKA2
8017133	1,50	2,83	0,03867	3,39	10,50	0,00039	NM_182620	VPS13D
7897890	1,50	2,83	0,01867	1,80	3,49	0,00016	NM_015378	KIF3A
8114030	1,50	2,83	0,00971	0,77	1,71	0,03938	NM_007054	NT5DC2
8087935	1,50	2,83	0,01418	2,43	5,37	0,00120	NM_022908	C11orf24
7949948	1,50	2,83	0,03145	2,49	5,60	0,00003	NM_022338	ZNF876P
8093332	1,50	2,83	0,06426	0,02	1,01	0,97033	NR_027481	TMX2
7940066	1,50	2,83	0,01980	3,20	9,20	0,00088	NM_015959	DDIT3
7964460	1,50	2,83	0,02106	3,48	11,13	0,00378	NM_004083	---
7978905	1,50	2,83	0,43674	-1,09	-2,13	0,01498	--- HMGCL	
7913682	1,50	2,83	0,03650	1,78	3,42	0,00036	NM_000191	PIGX
8084955	1,50	2,83	0,06992	2,61	6,10	0,00762	NM_001166304	CLK1
8058127	1,50	2,82	0,00799	1,13	2,19	0,07022	NR_027856	TRMT6
8064879	1,50	2,82	0,01167	2,23	4,70	0,00033	NM_015939	HEATR5B
8051464	1,50	2,82	0,02651	2,53	5,78	0,00124	NM_019024	SPATA18
8095021	1,50	2,82	0,05696	-1,73	-3,31	0,00284	NM_145263	COMMD6
7972044	1,50	2,82	0,00796	0,84	1,80	0,05483	NM_203497	JOSD1
8076128	1,50	2,82	0,11059	4,02	16,20	0,00045	NM_014876	---
8059672	1,50	2,82	0,24709	3,58	11,97	0,00003	--- KRCC1	
8053648	1,50	2,82	0,03153	1,58	2,99	0,02784	NM_016618	FAM120AOS
8162466	1,50	2,82	0,02449	2,46	5,52	0,00097	NM_198841	CMTM7
8078405	1,50	2,82	0,00440	2,87	7,29	0,00540	NM_138410	DNAJB14
7989885	1,50	2,82	0,02855	1,13	2,19	0,00108	NM_001031723	ZNF431
8027292	1,49	2,82	0,01330	2,31	4,95	0,00083	NM_133473	SMG1
8000167	1,49	2,82	0,01292	2,44	5,41	0,00092	NM_015092	ELAVL1
8033479	1,49	2,82	0,01667	3,08	8,43	0,00010	NM_001419	CADPS
8088491	1,49	2,82	0,03908	-1,08	-2,12	0,00439	NM_003716	RHBDD2
8133662	1,49	2,81	0,07261	2,42	5,36	0,00138	NM_001040457	WBSCR22
8133345	1,49	2,81	0,00133	2,56	5,88	0,00188	NM_017528	SNRPB2

8061075	1,49	2,81	0,10049	2,77	6,84	0,00311	NM_003092	CCDC71
8087372	1,49	2,81	0,21303	3,02	8,09	0,00004	NM_022903	FAM122B
8175269	1,49	2,81	0,03514	1,36	2,56	0,00210	NM_001166599	C3orf10
8085287	1,49	2,81	0,01102	0,64	1,56	0,20130	NM_018462	MRPL44
8048752	1,49	2,81	0,07196	1,86	3,62	0,00970	NM_022915	ZNF681
8035855	1,49	2,81	0,00037	1,59	3,01	0,02628	NM_138286	SLC12A6
7987192	1,49	2,81	0,00725	1,80	3,48	0,00019	NM_001042496	CDC37
8034021	1,49	2,81	0,01029	3,62	12,32	0,00175	NM_007065	SCYL1
7941274	1,49	2,81	0,00753	2,30	4,91	0,01226	NM_020680	POLR3K
7998129	1,49	2,81	0,07139	1,80	3,48	0,00068	NM_016310	HOXA6
8138741	1,49	2,81	0,00876	2,50	5,66	0,00952	NM_024014	PGBD4
7982504	1,49	2,80	0,04442	2,43	5,37	0,00199	NM_152595	C6orf70
8123467	1,49	2,80	0,05526	2,37	5,18	0,00570	NM_018341	SCAI
8164131	1,49	2,80	0,02867	1,89	3,71	0,01751	NM_173690	THOC4
8019273	1,49	2,80	0,02749	2,90	7,48	0,00122	NM_005782	LOC388022
7977270	1,49	2,80	0,19787	2,61	6,09	0,02897	AK131040 ALG11	
7969228	1,49	2,80	0,03432	2,48	5,59	0,00145	NM_001004127	HNRNPA1L2
7969263	1,49	2,80	0,04857	2,77	6,84	0,00095	NM_001011724	EIF4B
7963575	1,49	2,80	0,05185	0,41	1,33	0,31704	NM_001417	ALPL
7898693	1,48	2,80	0,14901	-1,27	-2,42	0,04675	NM_000478	PAK1IP1
8116848	1,48	2,80	0,04712	3,28	9,73	0,00036	NM_017906	ZFP36L1
7979813	1,48	2,80	0,03709	2,31	4,96	0,00094	NM_004926	HNRNPU
7925565	1,48	2,80	0,01539	1,40	2,64	0,00907	NM_031844	MXRA7
8056217	1,48	2,80	0,24284	3,32	9,97	0,00167	NM_198530	ATP5G2
7963713	1,48	2,80	0,04106	1,81	3,50	0,00888	NM_005176	TMEM214
8040753	1,48	2,80	0,06185	2,50	5,65	0,00012	NM_017727	WNT2
8142471	1,48	2,80	0,08615	-0,79	-1,72	0,00422	NM_003391	ZNF605
7967863	1,48	2,80	0,03495	1,59	3,00	0,03796	NM_183238	HINT1
8113873	1,48	2,80	0,01371	1,44	2,72	0,00485	NM_005340	PDE4DIP
7904965	1,48	2,80	0,06380	-1,77	-3,42	0,04957	AB042555 FBXW11	
8115765	1,48	2,79	0,02559	2,64	6,22	0,00005	NM_033644	ZNF75A
7992877	1,48	2,79	0,04388	2,25	4,75	0,00429	NM_153028	ARPC1B
8134552	1,48	2,79	0,02120	4,38	20,82	0,00079	NM_005720	SH3KBP1
8171684	1,48	2,79	0,02390	2,75	6,72	0,00834	NM_031892	SRPK2
8142036	1,48	2,79	0,01375	1,76	3,38	0,00044	NM_182691	SHC3
8162216	1,48	2,79	0,01749	-0,19	-1,14	0,19044	NM_016848	TBC1D5
8085689	1,48	2,79	0,02124	2,02	4,06	0,00917	NM_014744	GALNT7
8098328	1,48	2,79	0,03168	2,16	4,47	0,00060	NM_017423	---
8056728	1,48	2,79	0,06985	0,64	1,56	0,36081	--- POTEE	
8055220	1,48	2,79	0,07146	-0,37	-1,29	0,04719	NM_001083538	KDSR
8023656	1,48	2,79	0,03627	1,23	2,35	0,01026	NM_002035	---
8031899	1,48	2,79	0,07169	3,91	15,04	0,00003	--- OSBPL11	
8090277	1,48	2,78	0,05354	3,22	9,30	0,00031	NM_022776	KLF7
8058477	1,48	2,78	0,02822	1,39	2,62	0,00607	NM_003709	FAM126B
8058182	1,48	2,78	0,01250	2,48	5,57	0,00022	NM_173822	SLC25A6
8171111	1,48	2,78	0,03535	3,31	9,90	0,00093	NM_001636	SLC25A6
8177003	1,48	2,78	0,03535	3,31	9,90	0,00093	NM_001636	KIAA1267
8016266	1,48	2,78	0,05255	2,05	4,15	0,00016	NM_015443	CA5B
8166184	1,47	2,78	0,08185	1,57	2,96	0,04420	NM_007220	TMEM205
8034210	1,47	2,78	0,03505	3,76	13,54	0,00091	NM_198536	PLEKHA5
7954245	1,47	2,78	0,00391	1,76	3,39	0,00059	NM_019012	GPRC5B
7999909	1,47	2,78	0,01483	2,49	5,64	0,00780	NM_016235	STK24
7972444	1,47	2,78	0,07489	2,59	6,03	0,00038	NM_003576	NLGN1
8083968	1,47	2,78	0,04013	-1,12	-2,17	0,03907	NM_014932	CBWD1
8159815	1,47	2,78	0,00269	1,39	2,62	0,00935	NM_001145355	ATP6V1D
7979698	1,47	2,77	0,05228	2,41	5,30	0,00035	NM_015994	AGTRAP
7897745	1,47	2,77	0,00510	2,58	5,97	0,00501	NM_020350	NUDT3
8125825	1,47	2,77	0,00633	2,98	7,89	0,00019	NM_006703	CHUK
7935707	1,47	2,77	0,04117	3,07	8,42	0,00028	NM_001278	CLUL1
8019831	1,47	2,77	0,00432	0,07	1,05	0,76401	NM_014410	MRPL9
7920047	1,47	2,77	0,00100	3,30	9,88	0,00062	NM_031420	WDR7
8021312	1,47	2,77	0,02496	1,62	3,07	0,00079	NM_015285	EXOSC4
8148710	1,47	2,77	0,06961	2,43	5,40	0,00082	NM_019037	PNPO
8008064	1,47	2,77	0,07679	3,45	10,90	0,00383	NM_018129	RALA
8132406	1,47	2,77	0,02528	1,41	2,65	0,00080	NM_005402	PART1
8105495	1,47	2,77	0,06240	-1,37	-2,59	0,00447	NR_028508	FAM173B
8110966	1,47	2,77	0,06660	2,14	4,40	0,00224	NM_199133	NUDC
7899273	1,47	2,77	0,00756	1,98	3,93	0,00777	NM_006600	LIN7A
7965156	1,47	2,77	0,08562	-1,65	-3,13	0,01770	NM_004664	STARD3
8006877	1,47	2,77	0,01891	3,14	8,81	0,01086	NM_006804	GHR
8105121	1,47	2,77	0,10542	-0,83	-1,78	0,12646	NM_000163	ABL2
7922610	1,47	2,77	0,02233	2,09	4,25	0,00017	NM_007314	VANGL1
7904211	1,47	2,77	0,00830	2,31	4,96	0,00003	NM_138959	RNF26

7944510	1,47	2,76	0,09210	3,59	12,04	0,00042	NM_032015	ZC3H11A
7908978	1,47	2,76	0,29211	4,09	17,02	0,00015	NM_014827	TLE1
8161919	1,47	2,76	0,07528	3,45	10,89	0,00051	NM_005077	C10orf10
7933204	1,46	2,76	0,06841	-0,44	-1,36	0,01213	NM_007021	FUBP1
7917156	1,46	2,76	0,05304	2,25	4,75	0,00166	NM_003902	EPS15L1
8035120	1,46	2,76	0,01365	2,76	6,79	0,02751	NM_021235	USP33
7917120	1,46	2,76	0,08136	1,64	3,12	0,00281	NM_015017	AP1S2
8017210	1,46	2,76	0,02734	2,06	4,18	0,00021	NM_003916	IQCG
8093258	1,46	2,76	0,02506	1,49	2,80	0,02396	NM_032263	ZNF160
8039034	1,46	2,76	0,06526	1,35	2,56	0,00251	NM_001102603	KCTD9
8149857	1,46	2,76	0,22920	0,32	1,25	0,51203	NM_017634	BCL2L1
8065569	1,46	2,75	0,02820	2,46	5,52	0,00430	NM_138578	LMBR1
8144089	1,46	2,75	0,20269	4,03	16,29	0,00002	NM_022458	COX8A
7940835	1,46	2,75	0,00083	1,66	3,17	0,04780	NM_004074	PACRGL
8094342	1,46	2,75	0,03139	3,23	9,40	0,00154	NM_145048	TMSL2
8050089	1,46	2,75	0,01086	-1,00	-2,00	0,25032	BC104197 NFYC	
7900468	1,46	2,75	0,01400	2,47	5,53	0,00004	NM_001142588	NCOA2
8151254	1,46	2,75	0,04669	2,83	7,14	0,00101	NM_006540	CHD8
7977693	1,46	2,75	0,07534	3,04	8,25	0,00044	NM_020920	TBCE
7910651	1,46	2,74	0,05056	2,58	5,97	0,00072	NM_001079515	PDE1C
8138888	1,46	2,74	0,00930	-0,87	-1,82	0,01465	NM_005020	RAB30
7950743	1,45	2,74	0,03638	0,50	1,41	0,22027	NM_014488	SRGAP2
7904482	1,45	2,74	0,12484	2,50	5,65	0,00051	NM_015326	EFHA1
7970546	1,45	2,74	0,04888	2,64	6,21	0,00322	NM_152726	MOCOS
8020955	1,45	2,74	0,05298	3,28	9,70	0,00000	NM_017947	DUSP12
7906810	1,45	2,74	0,02544	3,65	12,53	0,00006	NM_007240	NPNT
8096704	1,45	2,74	0,07986	-0,58	-1,49	0,02911	NM_001033047	AUP1
8053214	1,45	2,74	0,01408	3,05	8,25	0,00202	NM_181575	UQCRH
7901212	1,45	2,74	0,02859	1,09	2,12	0,14350	NM_006004	XPNPEP3
8073311	1,45	2,74	0,03761	1,19	2,29	0,00564	NM_022098	FUNDC2
8171001	1,45	2,74	0,15155	2,64	6,22	0,01500	NM_023934	LRP12
8152280	1,45	2,73	0,00930	3,53	11,58	0,00005	NM_013437	---
8170418	1,45	2,73	0,04745	1,60	3,04	0,05378	--- ELAC2	
8012856	1,45	2,73	0,00537	2,34	5,05	0,00086	NM_018127	CORO2B
7984475	1,45	2,73	0,03031	-0,02	-1,01	0,94328	NM_006091	HIST1H3E
8117415	1,45	2,73	0,08231	-0,79	-1,73	0,00975	NM_003532	FAM40A
7903827	1,45	2,73	0,05833	2,87	7,30	0,00138	NM_033088	ATXN7L3
8015955	1,45	2,73	0,04061	2,41	5,32	0,00038	NM_020218	RPA1
8003679	1,45	2,73	0,00888	2,37	5,18	0,00007	NM_002945	MGC72080
8045887	1,45	2,73	0,38080	0,94	1,91	0,27589	NR_002822	NPIP
7993359	1,45	2,73	0,16863	2,48	5,59	0,00005	NM_006985	TMTC4
7972579	1,45	2,73	0,06262	4,30	19,63	0,00012	NM_032813	REV1
8054227	1,45	2,73	0,00716	3,29	9,78	0,00051	NM_016316	NEK1
8103646	1,45	2,73	0,00433	0,47	1,38	0,31594	NM_012224	TACC1
8145954	1,45	2,73	0,01870	1,88	3,67	0,01160	NM_006283	PTPN14
7924207	1,45	2,72	0,05297	2,75	6,71	0,00006	NM_005401	EFHC1
8120222	1,45	2,72	0,02575	1,20	2,30	0,00931	NR_033327	RWDD2A
8120927	1,45	2,72	0,08438	1,33	2,52	0,01104	NM_033411	SIRT1
7927814	1,45	2,72	0,04197	2,09	4,27	0,00248	NM_012238	CARS2
7972810	1,45	2,72	0,01184	0,87	1,82	0,06416	NM_024537	MST4
8169949	1,44	2,72	0,00271	2,66	6,34	0,00081	NM_016542	SMG1
8000687	1,44	2,72	0,10336	1,80	3,48	0,11295	NM_015092	KDM6A
8166956	1,44	2,72	0,03407	2,73	6,62	0,00014	NM_021140	CRK
8011018	1,44	2,72	0,07683	3,07	8,40	0,00009	NM_016823	HNMT
8045499	1,44	2,72	0,05016	-0,55	-1,46	0,00172	NM_006895	FNBP1L
7903092	1,44	2,72	0,01970	3,14	8,79	0,00014	NM_001024948	C20orf94
8060977	1,44	2,72	0,00456	1,03	2,04	0,04809	NM_001009608	TMEM164
8169365	1,44	2,72	0,00125	2,80	6,95	0,00084	NM_032227	SNAPC1
7974870	1,44	2,72	0,02624	3,09	8,53	0,00447	NM_003082	C16orf70
7996403	1,44	2,71	0,09432	3,13	8,74	0,00027	NM_025187	ARSK
8106962	1,44	2,71	0,09335	2,19	4,58	0,00643	NM_198150	PPP1R12B
7908841	1,44	2,71	0,08387	0,71	1,64	0,06536	NM_032105	EP300
8073345	1,44	2,71	0,04099	3,25	9,54	0,00083	NM_001429	KRTAP1-1
8015187	1,44	2,71	0,02969	-2,70	-6,50	0,00624	NM_030967	KLF10
8152215	1,44	2,71	0,00432	0,11	1,08	0,46092	NM_005655	CDC42BPB
7981387	1,44	2,71	0,02867	4,04	16,49	0,00057	NM_006035	MGC72080
7971241	1,44	2,71	0,38388	0,89	1,85	0,27917	NR_002822	PSG4
8037283	1,44	2,71	0,13979	-2,51	-5,68	0,01103	NM_002780	WDR70
8104998	1,44	2,71	0,03243	1,40	2,64	0,01110	NM_018034	CENPE
8102076	1,44	2,71	0,00578	0,81	1,76	0,12178	NM_001813	SLC35A4
8108558	1,44	2,71	0,17516	3,07	8,41	0,00422	NM_080670	UBE2N
7965471	1,44	2,71	0,16052	1,37	2,59	0,02259	NM_003348	UTP15
8106193	1,44	2,71	0,12402	2,63	6,18	0,00071	NM_032175	PLCE1

7929388	1,44	2,71	0,01508	1,24	2,37	0,00050	NM_016341	GSTA4
8127094	1,44	2,71	0,06501	1,89	3,70	0,00760	NM_001512	KIAA0100
8013696	1,44	2,70	0,03837	3,15	8,88	0,00007	NM_014680	SNX19
7952768	1,44	2,70	0,00758	2,41	5,33	0,00379	NM_014758	---
8090507	1,44	2,70	0,11851	1,14	2,20	0,00974	--- PLSCR3	
8012197	1,43	2,70	0,01726	2,50	5,67	0,00625	NM_020360	HLA-E
8117890	1,43	2,70	0,00864	2,68	6,40	0,00544	NM_005516	CYTH2
8030049	1,43	2,70	0,06151	3,04	8,25	0,00097	NM_017457	RPS7
8040036	1,43	2,70	0,00357	1,29	2,45	0,03727	NM_001011	GEMIN5
8115410	1,43	2,70	0,00534	2,17	4,51	0,00606	NM_015465	ZNF37A
7927071	1,43	2,70	0,01079	2,52	5,72	0,00131	NM_001007094	TUBD1
8017150	1,43	2,70	0,09319	2,57	5,95	0,00044	NM_016261	ZNF317
8025458	1,43	2,70	0,01752	3,24	9,47	0,00009	NM_020933	LRCH3
8085000	1,43	2,69	0,08230	1,83	3,56	0,00408	NM_032773	MTHFD1L
8155630	1,43	2,69	0,03180	0,59	1,50	0,13039	NM_015440	MTHFD1L
8161377	1,43	2,69	0,03180	0,59	1,50	0,13039	NM_015440	RPL12
8047635	1,43	2,69	0,06066	1,20	2,30	0,00103	NM_000976	KIAA1012
8022767	1,43	2,69	0,08706	3,17	9,00	0,00032	NM_014939	TOR1A
8164587	1,43	2,69	0,00739	1,81	3,51	0,00000	NM_000113	SALL1
8001387	1,43	2,69	0,06094	-0,52	-1,43	0,04503	NM_002968	NAPEPLD
8141872	1,43	2,69	0,03384	2,68	6,42	0,00016	NM_001122838	RCN2
7985089	1,43	2,69	0,04456	2,35	5,11	0,00234	NM_002902	RNF141
7946569	1,43	2,69	0,04853	2,96	7,79	0,00000	NM_016422	HADH
8096753	1,43	2,69	0,13974	2,62	6,16	0,00049	NM_005327	NF1
8022424	1,43	2,69	0,09462	3,98	15,80	0,00040	NM_001128147	NF1
8067862	1,43	2,69	0,09462	3,98	15,80	0,00040	NM_001128147	MANEA
8121144	1,43	2,69	0,03601	2,76	6,79	0,01180	NM_024641	PARP16
7989759	1,43	2,69	0,01544	3,67	12,77	0,00008	NM_017851	ALDH9A1
7921970	1,43	2,69	0,07014	3,28	9,72	0,00005	NM_000696	DENND2D
7918487	1,42	2,68	0,00066	2,76	6,79	0,00143	NM_024901	AOC3
8007420	1,42	2,68	0,01522	1,26	2,39	0,00450	NM_003734	C20orf43
8063509	1,42	2,68	0,01036	1,27	2,42	0,00344	NM_016407	FTH1
7948656	1,42	2,68	0,01235	1,25	2,37	0,05001	NM_002032	CLK2
7920737	1,42	2,68	0,02554	3,25	9,52	0,00485	NM_003993	MEX3C
8023377	1,42	2,68	0,10502	2,22	4,67	0,01434	NM_016626	HACE1
8128522	1,42	2,68	0,07248	3,48	11,17	0,00005	NM_020771	LAMA1
8022176	1,42	2,68	0,00410	0,14	1,10	0,26572	NM_005559	BRP44L
8130732	1,42	2,68	0,12958	2,79	6,91	0,00007	NM_016098	C7orf26
8131427	1,42	2,68	0,03692	2,41	5,33	0,00024	BC001076 USP11	
8167125	1,42	2,68	0,18347	3,27	9,67	0,00294	NM_004651	S100PBP
7899829	1,42	2,67	0,00986	2,47	5,56	0,00521	NM_022753	DNMBP
7935660	1,42	2,67	0,00109	1,53	2,88	0,00034	NM_015221	PRPF38B
7903519	1,42	2,67	0,02209	2,30	4,92	0,01032	NM_018061	MORC2
8075430	1,42	2,67	0,00665	2,39	5,24	0,00017	NM_014941	CBX3
8131949	1,42	2,67	0,03681	1,73	3,32	0,06712	NM_016587	ECH1
8036602	1,42	2,67	0,02025	3,80	13,93	0,01826	NM_001398	C15orf29
8130071	1,42	2,67	0,12294	2,61	6,09	0,08355	NM_024713	SEMA6D
7983527	1,42	2,67	0,01931	-1,77	-3,41	0,00900	NM_153618	PFN1
8011759	1,42	2,67	0,02675	1,31	2,48	0,00205	NM_005022	WDR89
7979565	1,42	2,67	0,02066	1,53	2,88	0,01816	NM_080666	MGC72080
7968787	1,42	2,67	0,38136	0,97	1,96	0,24454	NR_002822	R3HDM1
8045425	1,42	2,67	0,01306	2,35	5,11	0,00785	NM_015361	BEND6
8120362	1,42	2,67	0,04384	0,94	1,92	0,06442	NM_152731	---
8166580	1,42	2,67	0,00420	2,43	5,39	0,00406	--- STXBP4	
8008566	1,41	2,67	0,01364	2,07	4,19	0,00267	NM_178509	---
7905731	1,41	2,67	0,10205	2,50	5,64	0,01544	--- AZIN1	
8152222	1,41	2,67	0,03225	2,49	5,60	0,00016	NM_015878	TOR1B
8158560	1,41	2,67	0,05999	3,26	9,61	0,00009	NM_014506	CDC14B
8162610	1,41	2,66	0,01673	2,17	4,49	0,00022	NM_033331	CYP27A1
8048432	1,41	2,66	0,20903	2,27	4,83	0,00307	NM_000784	FAM149B1
7928354	1,41	2,66	0,11118	2,22	4,66	0,01265	NM_173348	STAU2
8151384	1,41	2,66	0,04854	1,80	3,49	0,00635	NM_014393	TMEM141
8159379	1,41	2,66	0,06872	1,40	2,64	0,09722	NM_032928	COX4I1
7997702	1,41	2,66	0,00088	1,02	2,02	0,01173	NM_001861	ARFIP1
8097813	1,41	2,66	0,04002	1,06	2,08	0,05811	NM_001025595	ZNF585A
8036373	1,41	2,66	0,00810	1,41	2,66	0,08186	NM_152655	SLC7A1
7970810	1,41	2,66	0,05003	4,03	16,37	0,00097	NM_003045	NUS1
8121704	1,41	2,66	0,04826	2,11	4,32	0,02893	NM_138459	TXLNA
7899703	1,41	2,66	0,00359	2,49	5,63	0,00007	NM_175852	PRKCD
8080487	1,41	2,66	0,04694	2,64	6,24	0,00572	NM_006254	ACP2
7947815	1,41	2,66	0,08184	2,83	7,12	0,01126	NM_001610	NPIP
7993404	1,41	2,66	0,07810	2,76	6,78	0,00008	NM_006985	MFHAS1
8149258	1,41	2,66	0,07963	3,69	12,95	0,00034	NM_004225	POU2F1

7907058	1,41	2,66	0,00184	2,27	4,83	0,00045	NM_002697	KRT8
7963567	1,41	2,66	0,26281	3,22	9,29	0,00029	NM_002273	TMEM199
8005857	1,41	2,66	0,11814	3,46	11,00	0,00067	NM_152464	CBWD3
8161587	1,41	2,66	0,00390	1,72	3,31	0,00174	NM_201453	MBOAT1
8124166	1,41	2,66	0,07504	4,66	25,28	0,00000	NM_001080480	DZIP1
7972336	1,41	2,66	0,04688	-0,64	-1,56	0,02079	NM_198968	WSB1
8005765	1,41	2,66	0,04466	3,22	9,29	0,00302	NM_015626	ALDH6A1
7980098	1,41	2,66	0,18853	2,83	7,12	0,00475	NM_005589	GFM2
8112622	1,41	2,65	0,09647	3,07	8,39	0,00049	NM_032380	FAT4
8097288	1,41	2,65	0,04400	1,87	3,66	0,02917	NM_024582	KLF12
7972003	1,41	2,65	0,01172	2,36	5,12	0,00356	NM_007249	NCK1
8082911	1,41	2,65	0,02984	2,82	7,05	0,00014	NM_006153	E2F6
8050336	1,41	2,65	0,01342	2,87	7,32	0,00004	NR_003094	---
8144667	1,41	2,65	0,01719	0,33	1,26	0,29035	--- SEC61B	
8156838	1,41	2,65	0,00171	0,99	1,98	0,00462	NM_006808	SLC35C2
8066697	1,41	2,65	0,00897	2,61	6,09	0,00189	NM_173179	SF3A1
8075343	1,40	2,65	0,06352	2,10	4,30	0,00050	NM_005877	FLJ35776
8019954	1,40	2,65	0,06704	2,01	4,03	0,00031	NR_024101	MAP3K5
8129804	1,40	2,65	0,06236	0,73	1,66	0,00753	NM_005923	USE1
8026679	1,40	2,65	0,04754	1,67	3,18	0,00050	NM_018467	VEGFA
8119898	1,40	2,65	0,07548	3,54	11,65	0,00324	NM_001025366	MRFAP1L1
8099235	1,40	2,65	0,03949	2,40	5,29	0,00260	NM_203462	FMO1
7907286	1,40	2,64	0,12514	-0,65	-1,56	0,01198	NM_002021	RFC3
7968563	1,40	2,64	0,15120	2,67	6,37	0,00367	NM_002915	PSMC5
8009227	1,40	2,64	0,00997	2,61	6,09	0,00012	NM_002805	LOC654433
8044605	1,40	2,64	0,06369	-0,80	-1,74	0,01794	NR_015377	TRIM4
8141363	1,40	2,64	0,10223	1,68	3,20	0,00062	NM_033017	C7orf68
8135915	1,40	2,64	0,06267	0,88	1,84	0,08818	NM_013332	PPDPF
8064100	1,40	2,64	0,04366	-0,02	-1,02	0,94388	NM_024299	TADA2A
8006715	1,40	2,64	0,02240	2,37	5,18	0,00060	NM_001488	DKC1
8170971	1,40	2,64	0,01906	3,22	9,33	0,00015	NM_001363	C13orf15
7968789	1,40	2,64	0,07424	0,38	1,30	0,32060	NM_014059	RUNX1
8070194	1,40	2,64	0,01317	1,65	3,14	0,00657	NM_001001890	EYA2
8063187	1,40	2,64	0,03851	-1,55	-2,92	0,00081	NM_005244	SNORA71D
8066262	1,40	2,64	0,20153	-2,11	-4,32	0,12672	NR_003018	SFRS2B
7943288	1,40	2,64	0,00881	1,66	3,15	0,01810	NM_032102	TGFB1
8037005	1,40	2,64	0,00765	1,76	3,40	0,00093	NM_000660	ELP3
8145586	1,40	2,64	0,00566	1,99	3,96	0,00278	NM_018091	SLC39A7
8118613	1,40	2,64	0,04108	1,36	2,57	0,01877	NM_006979	SLC39A7
8178225	1,40	2,64	0,04108	1,36	2,57	0,01877	NM_006979	SLC39A7
8179525	1,40	2,64	0,04108	1,36	2,57	0,01877	NM_006979	KLHDC5
7954604	1,40	2,64	0,03371	1,72	3,30	0,00043	NM_020782	TSR1
8011245	1,40	2,64	0,02278	3,57	11,91	0,00002	NM_018128	CDC23
8114396	1,40	2,63	0,10745	3,87	14,67	0,00004	NM_004661	UCHL5
7923007	1,40	2,63	0,00680	1,37	2,59	0,02069	NM_015984	CTTNBP2
8142497	1,40	2,63	0,04017	-0,60	-1,51	0,14541	NM_033427	PSMG1
8070330	1,40	2,63	0,09761	3,87	14,63	0,00001	NM_003720	HECTD2
7929145	1,40	2,63	0,01734	0,93	1,90	0,00261	NM_182765	NDUFA10
8060063	1,40	2,63	0,00940	1,58	3,00	0,00018	NM_004544	FAM65B
8124280	1,40	2,63	0,00829	-1,02	-2,03	0,00396	NM_014722	RPL29
8086148	1,40	2,63	0,02407	0,74	1,67	0,23839	NM_000992	TMEM97
8005839	1,39	2,63	0,00548	2,41	5,31	0,00337	NM_014573	SERTAD4
7909503	1,39	2,63	0,10638	-0,29	-1,22	0,31984	NM_019605	MASP1
8092661	1,39	2,63	0,08717	-1,27	-2,42	0,02456	NM_001879	---
8171865	1,39	2,63	0,08195	2,58	5,99	0,02829	--- DLAT	
7943827	1,39	2,63	0,02322	2,91	7,50	0,00135	NM_001931	CYB5D1
8004694	1,39	2,62	0,02651	2,85	7,19	0,00283	NM_144607	C18orf19
8022404	1,39	2,62	0,07150	3,12	8,68	0,00004	NM_001098801	THUMPD1
8000003	1,39	2,62	0,02524	1,64	3,12	0,01159	NM_017736	ST6GALNAC6
8164304	1,39	2,62	0,00024	0,89	1,85	0,00107	NM_013443	SRM
7912374	1,39	2,62	0,10965	2,55	5,84	0,01741	NM_003132	C8orf41
8150175	1,39	2,62	0,02709	1,90	3,72	0,00491	NM_025115	HOXA7
8138745	1,39	2,62	0,10419	0,09	1,06	0,77759	NM_006896	MGAT4B
8116316	1,39	2,62	0,00922	2,22	4,67	0,00165	NM_014275	NGF
7918869	1,39	2,62	0,03254	0,04	1,03	0,86240	NM_002506	PRDM2
7898070	1,39	2,62	0,06664	1,68	3,21	0,00261	NM_012231	SFXN3
7929919	1,39	2,62	0,12842	1,53	2,88	0,00558	NM_030971	SCG5
7982366	1,39	2,62	0,10868	-0,77	-1,70	0,11192	NM_001144757	FAM21C
7927233	1,39	2,62	0,05713	1,76	3,39	0,00026	NM_015262	MGEA5
7935910	1,39	2,62	0,13858	2,53	5,78	0,00032	NM_012215	PPP2R5E
7979551	1,39	2,61	0,04242	1,58	2,98	0,01764	NM_006246	MYO1E
7989277	1,39	2,61	0,00889	3,34	10,15	0,00116	NM_004998	SFRP1
8150428	1,39	2,61	0,08454	-0,71	-1,63	0,24873	NM_003012	ZNF136

8025998	1,38	2,61	0,01815	1,23	2,35	0,01490	NM_003437	TNIK
8092095	1,38	2,61	0,01085	1,36	2,56	0,00236	NM_015028	EIF1AX
8171747	1,38	2,61	0,04015	2,05	4,14	0,03160	NM_001412	SHKBP1
8028851	1,38	2,61	0,00590	2,03	4,09	0,00739	NM_138392	UHRF2
8154316	1,38	2,61	0,08564	2,60	6,05	0,00078	NM_152896	ARL15
8112033	1,38	2,61	0,11031	2,01	4,03	0,01206	NM_019087	BDH2
8102065	1,38	2,61	0,02945	0,77	1,71	0,09399	NM_020139	STK19
8118395	1,38	2,61	0,01699	1,68	3,20	0,00059	NR_026717	STK19
8178164	1,38	2,61	0,01699	1,68	3,20	0,00059	NR_026717	TUBGCP5
7981798	1,38	2,61	0,02823	2,09	4,26	0,00433	NM_052903	SKA1
8021187	1,38	2,61	0,02592	0,68	1,60	0,05632	NM_001039535	GMDS
8123562	1,38	2,61	0,03693	3,37	10,37	0,00075	NM_001500	TOB2
8076331	1,38	2,61	0,00898	2,35	5,09	0,00827	NM_016272	MED29
8028645	1,38	2,60	0,02194	1,98	3,94	0,00044	NM_017592	EPB41L5
8044882	1,38	2,60	0,01758	1,99	3,97	0,00129	NM_020909	UBE3A
7986769	1,38	2,60	0,00089	2,24	4,73	0,00008	NM_130839	TRAPPC2L
7997852	1,38	2,60	0,00863	0,62	1,53	0,21231	NM_016209	RNF122
8150186	1,38	2,60	0,06238	2,14	4,41	0,00695	NM_024787	HMGN4
8117510	1,38	2,60	0,09100	2,55	5,86	0,00032	NM_006353	CNOT10
8078412	1,38	2,60	0,00113	2,47	5,56	0,00010	NM_015442	SMURF1
8141241	1,38	2,60	0,02950	2,74	6,68	0,01023	NM_020429	SYDE2
7917322	1,38	2,60	0,02576	1,59	3,01	0,00044	NM_032184	UBE3C
8137596	1,38	2,60	0,02880	2,94	7,65	0,00014	NM_014671	ZNF804A
8046815	1,38	2,59	0,07376	-1,16	-2,24	0,00975	NM_194250	LGTN
7923889	1,38	2,59	0,06469	2,54	5,83	0,00066	NM_006893	PIGW
8006634	1,37	2,59	0,05361	2,67	6,36	0,00116	NM_178517	HIST1H2BM
8117594	1,37	2,59	0,05526	1,06	2,08	0,01835	NM_003521	SATB2
8058091	1,37	2,59	0,06140	3,03	8,18	0,00018	NM_015265	UQCRQ
8107998	1,37	2,59	0,05762	1,72	3,29	0,00091	NM_014402	PTCD2
8106107	1,37	2,59	0,01497	1,16	2,23	0,01176	NM_024754	FLOT2
8013788	1,37	2,59	0,02850	2,57	5,94	0,00066	NM_004475	---
7965152	1,37	2,59	0,03073	-0,03	-1,02	0,92473	--- FAM63A	
7919872	1,37	2,59	0,06675	2,40	5,30	0,00024	NM_018379	SMARCAL1
8048175	1,37	2,59	0,02838	1,72	3,30	0,00354	NM_014140	DIP2B
7955376	1,37	2,59	0,01145	1,63	3,10	0,00493	NM_173602	PTMA
8107470	1,37	2,59	0,18918	1,60	3,03	0,00076	NM_002823	KIAA0406
8066200	1,37	2,59	0,03291	2,07	4,21	0,00116	BC013755 CDC34	
8023968	1,37	2,59	0,04293	1,97	3,91	0,00521	NM_004359	MAPK7
8005576	1,37	2,59	0,01951	2,28	4,86	0,00391	NM_139033	NBPF12
7904500	1,37	2,58	0,58148	-0,85	-1,80	0,00729	ENST00000436832	IL1RAP
8084794	1,37	2,58	0,01631	1,81	3,50	0,00253	NM_002182	PDPN
7898057	1,37	2,58	0,00606	-1,44	-2,71	0,01181	NM_006474	LRRC37A4
8007919	1,37	2,58	0,00509	3,06	8,36	0,00082	NR_002940	ZNF547
8031732	1,37	2,58	0,10270	0,86	1,82	0,07825	NM_173631	SLC26A2
8109194	1,37	2,58	0,05095	1,56	2,95	0,00353	NM_000112	PLSCR4
8091306	1,37	2,58	0,06006	0,83	1,78	0,02355	NR_033438	MKNK1
7915846	1,37	2,58	0,07870	2,45	5,46	0,00130	NM_003684	GPX4
8024194	1,37	2,58	0,06691	2,11	4,33	0,00852	NM_002085	SDHAP1
8093039	1,37	2,58	0,00535	2,27	4,82	0,00446	NR_003264	USP9Y
8176578	1,37	2,58	0,10535	2,01	4,04	0,00102	NM_004654	ANXA10
8098246	1,37	2,58	0,08471	0,03	1,02	0,77644	NM_007193	RBP1
8091078	1,37	2,58	0,00457	-1,09	-2,12	0,02772	NM_002899	STMN1
7913869	1,37	2,58	0,02758	2,34	5,05	0,00323	NM_203401	MRPS22
8083011	1,37	2,58	0,04831	0,43	1,35	0,04832	NM_020191	HOMER1
8112841	1,37	2,58	0,01783	1,37	2,59	0,01167	NM_004272	TM9SF1
7978166	1,36	2,57	0,01409	2,21	4,62	0,00125	NM_006405	FRG1
8098714	1,36	2,57	0,05023	0,17	1,12	0,49461	NM_004477	XRCC1
8037331	1,36	2,57	0,00635	2,65	6,29	0,00033	NM_006297	ROR1
7901969	1,36	2,57	0,02835	1,27	2,41	0,00814	NM_005012	DIO2
7980485	1,36	2,57	0,10245	-1,23	-2,35	0,01536	NM_013989	RABIF
7923483	1,36	2,57	0,04569	2,62	6,15	0,00469	NM_002871	ZNF597
7998978	1,36	2,57	0,15160	-1,03	-2,04	0,01730	NM_152457	STK19
8179391	1,36	2,57	0,01684	1,54	2,91	0,00444	NR_026717	MANBA
8102006	1,36	2,57	0,03430	0,93	1,90	0,00410	NM_005908	PCGF1
8053187	1,36	2,57	0,03569	2,63	6,20	0,00121	NM_032673	HNRNPH3
7927854	1,36	2,57	0,10819	2,65	6,29	0,00087	NM_012207	PPFIBP1
7954559	1,36	2,56	0,01606	1,56	2,94	0,00052	NM_003622	DNAJC12
7933933	1,36	2,56	0,10634	1,53	2,88	0,00888	NM_021800	JAK2
8154178	1,36	2,56	0,00044	1,17	2,26	0,02704	NM_004972	PCGF6
7936083	1,36	2,56	0,32341	1,77	3,40	0,03964	NM_001011663	PSG7
8037251	1,36	2,56	0,04771	-1,16	-2,24	0,11785	NM_002783	HDGFRP3
7991070	1,36	2,56	0,02063	2,32	5,00	0,01288	NM_016073	GGH
8151032	1,36	2,56	0,02951	2,84	7,16	0,00102	NM_003878	KANK1

8154012	1,36	2,56	0,00644	0,83	1,78	0,00124	NM_153186	FAM21C
7927513	1,36	2,56	0,07288	1,78	3,42	0,00057	NM_015262	ST7
8135638	1,36	2,56	0,04736	2,22	4,66	0,00210	NM_018412	RAD54L2
8080100	1,36	2,56	0,01483	2,50	5,64	0,00016	NM_015106	RABAC1
8037071	1,36	2,56	0,10929	0,91	1,88	0,01060	NM_006423	RNF216
8137986	1,36	2,56	0,05121	2,33	5,01	0,00022	NM_207111	SLC2A14
7960850	1,36	2,56	0,00389	0,98	1,97	0,02140	NM_153449	RPP14
8080773	1,36	2,56	0,13459	2,69	6,45	0,00258	NM_001098783	SMARCB1
8071768	1,35	2,56	0,03639	2,21	4,62	0,00007	NM_003073	ANO4
7957861	1,35	2,56	0,01986	0,06	1,04	0,74229	NM_178826	GMPR2
7973660	1,35	2,56	0,03349	1,60	3,04	0,00103	NM_016576	DTL
7909568	1,35	2,56	0,05283	3,24	9,46	0,00473	NM_016448	IMPA1
8151549	1,35	2,55	0,11636	3,94	15,37	0,00010	NM_005536	C14orf104
7978838	1,35	2,55	0,00953	1,54	2,90	0,08922	NM_018139	RSRC2
7967304	1,35	2,55	0,05026	1,92	3,77	0,00190	NM_198261	GZF1
8061373	1,35	2,55	0,00329	1,08	2,11	0,02141	NM_022482	PHF1
8118682	1,35	2,55	0,01881	1,79	3,45	0,00438	NR_027692	RNU1-1
7898375	1,35	2,55	0,01467	-1,19	-2,29	0,07854	NR_004430	RNU1-1
7898411	1,35	2,55	0,01467	-1,19	-2,29	0,07854	NR_004430	RNU1-1
7912800	1,35	2,55	0,01467	-1,19	-2,29	0,07854	NR_004430	RNU1-1
7912850	1,35	2,55	0,01467	-1,19	-2,29	0,07854	NR_004430	RNU1-1
7919576	1,35	2,55	0,01467	-1,19	-2,29	0,07854	NR_004430	EPHB2
7898809	1,35	2,55	0,02992	0,54	1,45	0,40621	NM_017449	USP37
8058940	1,35	2,55	0,08922	2,28	4,86	0,00107	NM_020935	PSIP1
8160238	1,35	2,55	0,03291	0,64	1,55	0,12414	NM_033222	AKAP12
8122807	1,35	2,55	0,02489	1,01	2,01	0,00137	NM_005100	ALPK1
8096919	1,35	2,55	0,02017	1,60	3,04	0,00181	NM_025144	NCL
8059689	1,35	2,55	0,00089	2,66	6,31	0,00020	NM_005381	PSMB1
8130952	1,35	2,55	0,00918	1,94	3,84	0,00044	NM_002793	ZFYVE9
7901426	1,35	2,55	0,04167	1,41	2,66	0,00492	NM_004799	NSMCE2
8148293	1,35	2,55	0,01275	1,29	2,44	0,01434	NM_173685	ZDHHC21
8160151	1,35	2,54	0,08134	2,48	5,56	0,00239	NM_178566	KLF11
8040211	1,35	2,54	0,00499	2,33	5,03	0,00312	NM_003597	PDXDC2
8002322	1,35	2,54	0,02049	2,25	4,75	0,00607	NR_003610	SNORD74
7922418	1,35	2,54	0,04696	2,59	6,02	0,02473	NR_002579	BBS1
7941639	1,35	2,54	0,10993	2,60	6,05	0,00761	NM_024649	BEND7
7932118	1,35	2,54	0,04111	1,97	3,91	0,00583	NM_152751	EXTL2
7918026	1,34	2,54	0,00622	0,74	1,67	0,00582	NM_001439	PRMT1
8030437	1,34	2,54	0,05845	2,25	4,74	0,00486	NR_033397	NOB1
8002312	1,34	2,54	0,24085	4,66	25,36	0,00002	NM_014062	AP4E1
7983679	1,34	2,54	0,04140	2,48	5,57	0,00079	NM_007347	MAP4
8086842	1,34	2,54	0,00122	0,55	1,47	0,12797	NM_002375	ATP1B1
7907160	1,34	2,54	0,10962	3,58	11,92	0,00801	NM_001677	---
7910790	1,34	2,53	0,01615	1,43	2,70	0,00605	--- MATN2	
8147516	1,34	2,53	0,00106	-0,64	-1,55	0,20066	NM_002380	ST3GAL2
8002370	1,34	2,53	0,06627	1,98	3,95	0,04523	NM_006927	C7orf28A
8131356	1,34	2,53	0,07013	1,56	2,94	0,02226	NM_015622	LOC138412
8154727	1,34	2,53	0,10966	2,62	6,14	0,00775	AF091236 ST6GALNAC5	
7902441	1,34	2,53	0,00289	-0,14	-1,10	0,36289	NM_030965	ARHGAP19
7935403	1,34	2,53	0,04047	2,53	5,76	0,00120	NM_032900	PLEKHM2
7898227	1,34	2,52	0,05890	3,29	9,75	0,00095	NM_015164	RPL13AP20
7954063	1,33	2,52	0,12710	3,01	8,06	0,00014	NR_003932	SPRY1
8097282	1,33	2,52	0,09582	0,79	1,73	0,00655	NM_005841	SNORD116-6
7981960	1,33	2,52	0,07722	-1,15	-2,22	0,10820	NR_003321	TMEM140
8136388	1,33	2,52	0,09786	-1,31	-2,47	0,00004	NM_018295	EYS
8127396	1,33	2,52	0,09966	-1,06	-2,08	0,35513	NM_001142800	DNAJA1
8154765	1,33	2,52	0,00545	0,86	1,81	0,05184	NM_001539	PRPSAP1
8018694	1,33	2,52	0,01702	2,16	4,48	0,00201	NM_002766	ZNF22
7927202	1,33	2,52	0,00690	2,08	4,24	0,00159	NM_006963	MINK1
8003991	1,33	2,52	0,00899	2,87	7,31	0,00098	NM_153827	C10orf12
7929609	1,33	2,52	0,00768	2,75	6,75	0,00078	BC024315 POTEE	
8045208	1,33	2,52	0,07578	-0,74	-1,68	0,07304	NM_001083538	C1D
8052698	1,33	2,51	0,05062	2,15	4,45	0,00048	NM_006333	BCAP29
8135422	1,33	2,51	0,01376	1,25	2,38	0,00822	NM_001008405	PABPC4
7915207	1,33	2,51	0,00191	2,29	4,89	0,00066	NM_001135653	PNN
7974066	1,33	2,51	0,00854	0,70	1,62	0,04518	NM_002687	MMP14
7973336	1,33	2,51	0,00461	0,16	1,12	0,79675	NM_004995	LMAN2L
8053975	1,33	2,51	0,01760	1,47	2,77	0,04027	NM_001142292	ANKRD49
7943231	1,33	2,51	0,08959	2,30	4,92	0,00886	NM_017704	TMEM55A
8151756	1,33	2,51	0,04385	1,70	3,25	0,00042	NM_018710	SLC19A2
7922162	1,33	2,51	0,12356	3,21	9,23	0,00060	NM_006996	SIX1
7979505	1,33	2,51	0,04647	0,97	1,96	0,13138	NM_005982	AHNAK2
7981514	1,33	2,51	0,04436	2,07	4,21	0,04863	NM_138420	BDP1

8177560	1,33	2,51	0,01751	1,63	3,10	0,00256	NM_018429	ALDOC	
8013660	1,32	2,51	0,06589	0,30	1,23	0,17623	NM_005165	ZNF266	
8033754	1,32	2,50	0,03703	3,09	8,53	0,00411	NM_006631	PTPN4	
8044849	1,32	2,50	0,01732	1,85	3,60	0,01823	NM_002830	ANKFY1	
8011599	1,32	2,50	0,01711	3,10	8,60	0,00019	NM_016376	KYNU	
8045539	1,32	2,50	0,00864	4,01	16,14	0,00049	NM_003937	PCMTD1	
8146427	1,32	2,50	0,01781	-0,05	-1,03	0,87844	NM_052937	SSU72	
7911568	1,32	2,50	0,01959	1,72	3,30	0,01180	NM_014188	CACNB2	
7926506	1,32	2,50	0,01022	-0,62	-1,53	0,14714	NM_201596	MICA	
8179238	1,32	2,50	0,10232	2,31	4,96	0,00130	NM_000247	PSPC1	
7970413	1,32	2,50	0,01905	1,81	3,50	0,00736	NR_003272	MTMR4	
8017019	1,32	2,50	0,19083	4,99	31,82	0,00037	NM_004687	ALPK2	
8023528	1,32	2,50	0,01774	-0,24	-1,18	0,31094	NM_052947	KIAA0586	
7974653	1,32	2,50	0,02348	2,19	4,58	0,00363	NM_014749	POLR2J2	
8141829	1,32	2,50	0,01788	1,64	3,11	0,00047	NM_032959	CCNL1	
8091658	1,32	2,50	0,11263	2,49	5,61	0,00208	NM_020307	PGM5P2	
8161513	1,32	2,49	0,05932	0,62	1,54	0,28779	NR_002836	BEX4	
8169009	1,32	2,49	0,13216	0,25	1,19	0,00395	NM_001080425	VPS33A	
7967240	1,32	2,49	0,05063	2,01	4,03	0,00085	NM_022916	PPIF	
7928589	1,32	2,49	0,09356	3,60	12,13	0,00032	NM_005729	PRSS23	
7942957	1,32	2,49	0,01078	1,51	2,85	0,00093	NM_007173	TRAK1	
8079037	1,32	2,49	0,04188	2,24	4,72	0,00023	NM_001042646	SPATA7	
7976084	1,32	2,49	0,03550	0,21	1,16	0,34119	NM_018418	FANCI	
7985829	1,32	2,49	0,02189	3,86	14,57	0,00292	NM_001113378	EXOSC8	
7968658	1,32	2,49	0,00292	2,24	4,72	0,00307	NM_181503	INMT	
8132092	1,32	2,49	0,03086	-1,71	-3,27	0,00467	NM_006774	ANKRD29	
8022559	1,32	2,49	0,01942	3,72	13,18	0,00029	NM_173505	PRKD3	
8051547	1,32	2,49	0,02896	0,97	1,95	0,04057	NM_005813	KIAA1731	
7943126	1,32	2,49	0,00396	1,07	2,10	0,01858	NM_033395	TUBA1B	
7963046	1,32	2,49	0,00369	1,03	2,04	0,04495	NM_006082	STAMBPL1	
7929012	1,31	2,49	0,14170	2,20	4,61	0,00419	NM_020799	ING3	
8135718	1,31	2,49	0,11338	1,80	3,49	0,00612	NM_019071	FEZ2	
8051427	1,31	2,49	0,00899	1,15	2,22	0,00355	NM_005102	ARID1A	
7899220	1,31	2,49	0,00326	2,42	5,37	0,00101	NM_006015	SETD8	
7959574	1,31	2,49	0,05178	2,75	6,74	0,00021	NM_020382	YTHDC2	
8107375	1,31	2,49	0,00977	2,08	4,24	0,00141	NM_022828	SNORD15A	
7942592	1,31	2,48	0,10400	0,06	1,05	0,91984	NR_000005	STMN3	
8067722	1,31	2,48	0,04126	0,11	1,08	0,78707	NM_015894	BRD2	
8179504	1,31	2,48	0,09788	2,70	6,50	0,00492	NM_005104	TRIM5	
7946089	1,31	2,48	0,08391	2,10	4,28	0,00025	NM_033092	C20orf4	
8062286	1,31	2,48	0,01445	1,75	3,37	0,00067	BC019311 RABGGTB		
7902382	1,31	2,48	0,08586	4,07	16,77	0,00002	NM_004582	ZFAND2B	
8048478	1,31	2,48	0,02430	1,05	2,08	0,09111	NM_138802	LRRC41	
7915827	1,31	2,48	0,06578	2,33	5,01	0,00777	NM_006369	IGF1R	
7986359	1,31	2,48	0,09215	2,97	7,83	0,01519	NM_000875	APEX1	
7973056	1,31	2,48	0,17224	3,31	9,89	0,00035	NM_001641	COQ6	
7975661	1,31	2,48	0,02340	2,76	6,78	0,00178	NM_182476	PRIM2	
8120411	1,31	2,48	0,04227	1,24	2,37	0,04815	NM_000947	MGC72080	
8082244	1,31	2,48	0,34025	0,37	1,30	0,52602	NR_002822	---	
7984567	1,31	2,48	0,01451	0,31	1,24	0,12327	--- ZNF268		
7960165	1,31	2,48	0,07246	2,18	4,52	0,00532	NM_003415	UBE2T	
7923426	1,31	2,48	0,01642	2,71	6,54	0,00041	NM_014176	RUVBL1	
8090448	1,31	2,48	0,13694	3,54	11,61	0,00001	NM_003707	GLMN	
7917676	1,31	2,48	0,05560	1,97	3,92	0,00868	NM_053274	DECR1	
8147228	1,31	2,48	0,16382	1,40	2,64	0,03187	NM_001359	GPSM2	
7903565	1,31	2,48	0,09810	3,64	12,51	0,00469	NM_013296	TMEM194B	
8057732	1,31	2,47	0,03949	2,40	5,27	0,00010	NM_001142645	FAM168B	
8055249	1,30	2,47	0,13325	1,07	2,11	0,01522	NM_001009993	TXNL4B	
8002660	1,30	2,47	0,00851	2,69	6,47	0,00043	NM_017853	GPD2	
8045816	1,30	2,47	0,02854	2,92	7,54	0,00013	NM_001083112	BHLHE40	
8077441	1,30	2,47	0,02365	1,75	3,35	0,00154	NM_003670	TMEM8A	
7998233	1,30	2,47	0,02493	2,22	4,66	0,00539	NM_021259	SIN3A	
7990487	1,30	2,46	0,03275	2,62	6,14	0,00625	NM_015477	FAM86B1 //	
FAM86B1 // FAM86B1 // FAM86B1 // FAM86B1 // FAM86B1									
7945875	1,30	2,46	0,02755	1,99	3,96	0,01035	NR_003494 // NR_003494 // NR_003494		
// NR_003494 // NR_003494 // NR_003494				SMC6					
8050443	1,30	2,46	0,02439	2,25	4,75	0,00041	NM_001142286	DEPDC5	
8072529	1,30	2,46	0,02201	2,13	4,39	0,00100	NM_014662	MCOLN3	
7917304	1,30	2,46	0,01723	3,07	8,43	0,00623	NM_018298	CRNKL1	
8065256	1,30	2,46	0,12152	1,11	2,16	0,00323	NM_016652	SUN1	
8131013	1,30	2,46	0,03387	2,61	6,11	0,00001	NM_001130965	HTATIP2	
7938880	1,30	2,46	0,04124	4,14	17,60	0,00016	NM_001098520	THOC1	
8021924	1,30	2,46	0,15837	2,73	6,63	0,00346	NM_005131	FH	

7925480	1,30	2,46	0,06684	1,06	2,09	0,00379	NM_000143	RNASEH2B
7969179	1,30	2,46	0,12434	2,11	4,33	0,00675	NM_024570	URM1
8158242	1,30	2,46	0,09305	2,70	6,51	0,00037	NM_030914	ARPC4
8077652	1,30	2,46	0,03358	0,96	1,94	0,00724	NM_005718	ATP6V0C
7992646	1,30	2,46	0,01771	2,12	4,34	0,00901	NM_001694	VAC14
8002421	1,30	2,46	0,22320	2,20	4,58	0,01474	NM_018052	MTMR10
7987048	1,30	2,46	0,02154	1,21	2,31	0,06194	NM_017762	PCDH10
8097449	1,30	2,46	0,03282	-0,89	-1,85	0,00370	NM_032961	FAM178A
7929858	1,30	2,46	0,05650	1,20	2,30	0,00502	NM_018121	PRPS2
8166049	1,30	2,46	0,00262	3,28	9,70	0,00214	NM_001039091	BCL10
7917338	1,30	2,45	0,06227	0,75	1,69	0,16041	NM_003921	ADAL
7983191	1,30	2,45	0,01162	2,99	7,93	0,00162	NM_001159280	WBP4
7968746	1,30	2,45	0,00761	0,52	1,43	0,27708	NM_007187	GNAI1
8133860	1,29	2,45	0,02191	1,38	2,60	0,01390	NM_002069	OGT
8168316	1,29	2,45	0,04947	2,11	4,31	0,00119	NM_181672	ECHS1
7937217	1,29	2,45	0,00356	1,48	2,78	0,00227	NM_004092	DDX23
7962869	1,29	2,45	0,18772	3,59	12,08	0,00016	NM_004818	LARGE
8075637	1,29	2,45	0,14959	-1,43	-2,69	0,00615	NM_004737	SDCCAG8
7911017	1,29	2,45	0,03600	1,03	2,04	0,02199	NM_006642	BAP1
8087885	1,29	2,45	0,00570	3,15	8,85	0,00060	NM_004656	SFXN4
7936641	1,29	2,45	0,00179	1,22	2,33	0,00488	NM_213649	ATPAF2
8013179	1,29	2,45	0,00835	2,37	5,16	0,00062	NM_145691	SMARCAD1
8096463	1,29	2,45	0,13316	2,87	7,33	0,00053	NM_001128429	PIBF1
7969390	1,29	2,45	0,11157	0,35	1,28	0,08880	NM_006346	MOV10
7904050	1,29	2,45	0,15962	2,29	4,89	0,00289	NM_020963	TRAF6
7947540	1,29	2,45	0,02873	2,11	4,32	0,00992	NM_145803	VPRBP
8087748	1,29	2,45	0,02602	2,98	7,91	0,00004	NM_014703	AP2A1
8030470	1,29	2,45	0,03626	2,16	4,48	0,00432	NM_014203	NIN
7979044	1,29	2,44	0,02439	2,17	4,50	0,00241	NM_020921	GPAM
8113356	1,29	2,44	0,35430	-1,22	-2,32	0,05706	AK172782 ZNF558	
8033667	1,29	2,44	0,00251	2,73	6,62	0,00203	NM_144693	MRRF
8157638	1,29	2,44	0,10637	2,71	6,56	0,00015	NM_138777	CBWD3
8155636	1,29	2,44	0,00283	1,61	3,05	0,00267	NM_201453	UNC50
8043820	1,29	2,44	0,00095	1,85	3,60	0,00368	NM_014044	---
8150844	1,29	2,44	0,00415	0,50	1,41	0,00301	--- FRK	
8129071	1,29	2,44	0,04303	1,03	2,05	0,00400	NM_002031	PDP2
7996341	1,28	2,44	0,06333	2,32	4,99	0,00009	NM_020786	---
8165656	1,28	2,44	0,05440	-1,62	-3,08	0,00747	--- ELP4	
7939102	1,28	2,43	0,08423	1,90	3,73	0,00585	NM_019040	RHOF
7967202	1,28	2,43	0,01140	3,59	12,03	0,00034	NM_019034	FAM188A
7932285	1,28	2,43	0,15262	1,78	3,43	0,01852	NM_024948	TSPYL5
8151931	1,28	2,43	0,12682	-2,60	-6,07	0,01131	NM_033512	MAGED2
8167815	1,28	2,43	0,04795	1,58	2,98	0,04408	NM_014599	SPTLC1
8162294	1,28	2,43	0,04698	2,69	6,45	0,00073	NM_006415	C6orf186
8128837	1,28	2,43	0,03853	-0,28	-1,22	0,38688	NM_001123364	MT1P2
7925413	1,28	2,43	0,04351	0,27	1,20	0,75129	AF333388 ---	
7965508	1,28	2,43	0,00968	0,15	1,11	0,78454	--- RPL10	
8034416	1,28	2,43	0,22248	2,45	5,48	0,00369	NM_006013	MTX3
8112857	1,28	2,43	0,04032	0,83	1,78	0,24751	NM_001010891	UPF2
7932041	1,28	2,43	0,00424	2,05	4,14	0,01058	NM_080599	UHRF1BP1L
7965723	1,28	2,42	0,15359	0,96	1,95	0,02986	NM_015054	---
7928489	1,28	2,42	0,29564	-0,23	-1,17	0,72857	--- SEH1L	
8020254	1,28	2,42	0,10863	3,49	11,24	0,00027	NM_031216	NENF
7909603	1,28	2,42	0,06860	1,05	2,07	0,00316	NM_013349	SELS
7991587	1,27	2,42	0,08498	2,12	4,34	0,00032	NM_203472	TMEM203
8165642	1,27	2,42	0,02377	3,40	10,55	0,00011	NM_053045	RPL38
8009561	1,27	2,42	0,00355	2,00	4,01	0,00160	NM_000999	CCNJ
7929550	1,27	2,42	0,10077	0,84	1,79	0,04814	NM_001134375	NCS1
8158627	1,27	2,42	0,02883	2,59	6,02	0,00007	NM_014286	SLC38A4
7962559	1,27	2,42	0,04637	-1,24	-2,36	0,03153	NM_018018	COX7C
8106776	1,27	2,42	0,00332	1,10	2,15	0,02102	NM_001867	CSPP1
8146756	1,27	2,41	0,03769	1,82	3,52	0,02341	NM_001077204	PDCD4
7930454	1,27	2,41	0,03922	1,91	3,77	0,00495	NM_145341	ANKRD40
8016725	1,27	2,41	0,03140	3,25	9,50	0,00310	NM_052855	BAK1
8125766	1,27	2,41	0,25851	2,19	4,56	0,02825	NM_001188	RCAN2
8126760	1,27	2,41	0,13993	-0,81	-1,75	0,01886	NM_005822	DSN1
8066074	1,27	2,41	0,09515	2,99	7,92	0,00387	NM_001145316	ACBD5
7932703	1,27	2,41	0,00657	2,79	6,92	0,00011	NM_145698	ARHGDIB
7961532	1,27	2,41	0,17220	-0,52	-1,43	0,36918	NM_001175	---
8165661	1,27	2,41	0,14936	-0,80	-1,74	0,00873	--- IL1B	
8054722	1,27	2,41	0,03905	-0,51	-1,42	0,08431	NM_000576	NEFL
8149835	1,27	2,41	0,05673	-0,57	-1,49	0,02143	NM_006158	SH3BGRL2
8120833	1,27	2,41	0,01514	1,76	3,39	0,00884	NM_031469	FAM20B

7907680	1,27	2,41	0,05681	1,97	3,92	0,00044	NM_014864	ETNK1
7954419	1,27	2,40	0,00479	2,53	5,79	0,00017	NM_018638	KIAA0101
7989647	1,27	2,40	0,09813	2,95	7,73	0,00781	NM_014736	ALMS1
8042737	1,27	2,40	0,01118	2,20	4,60	0,00828	NM_015120	LRPAP1
8099073	1,26	2,40	0,11395	1,25	2,38	0,08337	NM_002337	FASTKD3
8110920	1,26	2,40	0,10220	2,77	6,80	0,00302	NM_024091	C1orf57
7910591	1,26	2,40	0,13627	2,47	5,54	0,00224	NM_032324	NFE2L1
8008087	1,26	2,40	0,05992	2,95	7,75	0,00304	NM_003204	RANBP1
8071332	1,26	2,40	0,06188	2,41	5,32	0,02350	NM_002882	BPGM
8136341	1,26	2,40	0,03776	1,16	2,24	0,00011	NM_199186	RLIM
8173615	1,26	2,40	0,03581	2,15	4,43	0,00036	NM_183353	SERTAD3
8036908	1,26	2,40	0,03823	2,28	4,84	0,00039	NM_013368	FASTKD2
8047815	1,26	2,40	0,08268	2,20	4,60	0,00237	NM_001136194	UQCR11
8032284	1,26	2,40	0,03502	2,49	5,63	0,00183	NM_006830	PITPNC1
8009353	1,26	2,40	0,08778	4,42	21,42	0,00038	NM_181671	RAC1
8131406	1,26	2,40	0,03896	2,21	4,63	0,01713	NM_018890	ZNF714
8027285	1,26	2,40	0,02882	3,09	8,54	0,02331	NM_182515	LAMA3
8020551	1,26	2,40	0,00521	4,05	16,52	0,00001	NM_198129	PRPF40A
8055913	1,26	2,40	0,10522	2,27	4,83	0,00104	NM_017892	NBEA
7968577	1,26	2,40	0,01137	1,90	3,73	0,00141	NM_015678	NGRN
7985983	1,26	2,40	0,05551	1,97	3,91	0,01250	NR_028052	USP13
8084100	1,26	2,40	0,03597	2,74	6,67	0,00001	NM_003940	PARP9
8090018	1,26	2,40	0,02974	0,13	1,09	0,49784	NM_031458	NR2C1
7965523	1,26	2,40	0,03009	2,18	4,52	0,00346	NM_003297	RUFY1
8110499	1,26	2,40	0,00016	2,87	7,33	0,00000	NM_025158	TMEM136
7944554	1,26	2,39	0,01673	2,00	3,99	0,00243	NM_174926	SUMF2
8132964	1,26	2,39	0,11829	2,96	7,76	0,00040	NM_015411	PPIAL4A
7919436	1,26	2,39	0,44381	0,52	1,44	0,05472	NM_178230	COPS7B
8049088	1,26	2,39	0,12957	2,90	7,48	0,00005	NM_022730	RRP1B
8068902	1,26	2,39	0,00016	3,06	8,32	0,00043	NM_015056	SLC43A1
7948249	1,26	2,39	0,00923	2,41	5,31	0,00012	NM_003627	DUSP16
7961371	1,26	2,39	0,02917	2,08	4,23	0,00032	NM_030640	TMEM41A
8092534	1,26	2,39	0,21465	4,11	17,24	0,00007	NM_080652	MIR622
7969574	1,26	2,39	0,18285	4,25	19,04	0,00181	NR_030754	MRPS31
7971184	1,26	2,39	0,04946	1,67	3,18	0,00866	NM_005830	JAG1
8064978	1,26	2,39	0,05372	1,06	2,08	0,06422	NM_000214	HRH1
8077851	1,26	2,39	0,12896	0,10	1,07	0,68769	NM_001098213	MGC72080
7945873	1,26	2,39	0,46051	0,61	1,53	0,47315	NR_002822	LMOD1
7923386	1,26	2,39	0,11253	-1,30	-2,46	0,00628	NM_012134	GADD45A
7902227	1,26	2,39	0,01271	1,82	3,53	0,00122	NM_001924	TGFB3
7980316	1,25	2,38	0,03232	-0,34	-1,27	0,13200	NM_003239	BRD8
8114365	1,25	2,38	0,02275	2,17	4,50	0,00337	NM_139199	SRBD1
8051963	1,25	2,38	0,17580	2,80	6,98	0,01058	NM_018079	ZCCHC8
7967287	1,25	2,38	0,15184	3,50	11,29	0,00018	NM_017612	MT1H
7995829	1,25	2,38	0,07289	0,09	1,07	0,82512	NM_005951	MRPL1
8095894	1,25	2,38	0,00898	0,94	1,92	0,06270	NM_020236	DCAF5
7979849	1,25	2,38	0,08096	2,13	4,38	0,00131	NM_003861	SERPINB9
8123609	1,25	2,38	0,00771	2,07	4,21	0,00203	NM_004155	PLK1S1
8061305	1,25	2,38	0,00391	0,42	1,34	0,09835	NM_018474	MCOLN1
8025183	1,25	2,38	0,02550	1,62	3,08	0,01369	NM_020533	C11orf48
7948775	1,25	2,38	0,15540	1,79	3,47	0,00493	NM_024099	KIAA0020
8159854	1,25	2,38	0,08301	1,85	3,61	0,00344	NM_014878	KHDRBS1
7899654	1,25	2,38	0,02223	1,72	3,28	0,00041	NM_006559	NME6
8086899	1,25	2,38	0,10710	1,37	2,58	0,01067	NM_005793	ST3GAL6
8081219	1,25	2,37	0,04506	1,38	2,60	0,00826	NM_006100	FAM188B
8132097	1,25	2,37	0,00320	1,19	2,29	0,00558	NM_032222	MGC72080
8141169	1,25	2,37	0,12908	1,18	2,27	0,14844	NR_002822	QKI
8123315	1,25	2,37	0,00944	1,00	2,00	0,00374	NM_206855	RRS1
8146685	1,25	2,37	0,03245	2,15	4,43	0,00850	NM_015169	TAPT1
8099506	1,25	2,37	0,07481	2,75	6,75	0,00498	NM_153365	ENDOD1
7943293	1,25	2,37	0,04576	0,46	1,38	0,09193	NM_015036	NFYA
8119408	1,25	2,37	0,04938	2,93	7,64	0,00463	NM_002505	C1orf107
7909478	1,25	2,37	0,02354	2,50	5,65	0,00026	NM_014388	SEC62
8083826	1,25	2,37	0,03107	0,86	1,82	0,02903	NM_003262	CYB5R2
7946292	1,24	2,37	0,02982	1,53	2,88	0,00773	NM_016229	POFUT2
8070876	1,24	2,37	0,06674	0,60	1,52	0,03276	NM_015227	AGGF1
8106429	1,24	2,37	0,00764	1,68	3,21	0,00284	NM_018046	NKIRAS2
8007197	1,24	2,37	0,08403	3,01	8,03	0,00160	NM_017595	RUSC2
8155048	1,24	2,37	0,06725	1,30	2,45	0,01101	NM_014806	---
8130403	1,24	2,37	0,12311	0,62	1,53	0,25493	---	EXOC5
8095333	1,24	2,37	0,07519	1,41	2,65	0,03435	NM_006544	ERGIC1
8109999	1,24	2,37	0,01296	2,40	5,28	0,00197	NM_001031711	PDE9A
8068833	1,24	2,37	0,03101	2,20	4,59	0,00942	NM_002606	RSBN1

7918681	1,24	2,37	0,01111	1,96	3,88	0,00241	NM_018364	TBC1D1
8094574	1,24	2,37	0,00944	1,62	3,07	0,00154	NM_015173	LAP3
8094259	1,24	2,37	0,02382	1,53	2,88	0,00185	NM_015907	C9orf72
8160531	1,24	2,37	0,10058	2,18	4,52	0,00618	NM_018325	SLC35B4
8143040	1,24	2,36	0,07121	2,65	6,28	0,00015	NM_032826	DET1
7991216	1,24	2,36	0,07306	2,17	4,51	0,00037	NM_017996	MRTO4
7898549	1,24	2,36	0,07159	2,06	4,18	0,00097	NM_016183	CDC14A
7903334	1,24	2,36	0,13488	1,86	3,64	0,00171	NM_003672	RPS6KB1
8008887	1,24	2,36	0,11738	2,22	4,65	0,00027	NM_003161	POC5
8112709	1,24	2,36	0,02539	3,25	9,48	0,00095	NM_001099271	TMEM70
8146930	1,24	2,36	0,00230	2,49	5,61	0,00132	NM_017866	GLA
8174076	1,24	2,36	0,02480	2,72	6,60	0,00012	NM_000169	BCAS2
7918759	1,24	2,36	0,02739	2,14	4,40	0,00264	NM_005872	ARMCX2
8174092	1,24	2,36	0,01476	-0,04	-1,03	0,64856	NM_014782	NBN
8151711	1,24	2,36	0,10367	2,36	5,13	0,00801	NM_002485	SLTM
7989253	1,24	2,36	0,02614	2,74	6,67	0,00015	NM_024755	SH2D4A
8144880	1,24	2,36	0,07256	1,92	3,78	0,00174	NM_022071	SKP1
8114152	1,24	2,36	0,06399	0,64	1,55	0,01955	NM_006930	RPL18A
8154359	1,24	2,36	0,01767	0,76	1,69	0,02488	AY779046 NF2	
8072242	1,24	2,36	0,10397	3,02	8,14	0,00073	NM_016418	POLR3B
7958275	1,24	2,36	0,07632	1,81	3,51	0,00055	NM_018082	ADI1
8050071	1,24	2,36	0,01492	2,09	4,26	0,00409	NM_018269	MOCS2
8112020	1,24	2,36	0,08620	2,22	4,67	0,00019	NM_176806	FKBP11
7962895	1,24	2,36	0,01001	1,00	2,01	0,06217	NM_016594	POLR1B
8044473	1,24	2,36	0,05461	2,05	4,14	0,00178	NM_019014	---
8149953	1,23	2,35	0,07547	0,36	1,28	0,21140	--- RCL1	
8154163	1,23	2,35	0,04023	3,30	9,83	0,00018	NM_005772	ZFPL1
7941127	1,23	2,35	0,03688	2,51	5,69	0,01967	NM_006782	HELQ
8101467	1,23	2,35	0,06602	2,00	3,99	0,00332	NM_133636	JHDM1D
8143341	1,23	2,35	0,07254	1,29	2,44	0,08130	NM_030647	SASS6
7917976	1,23	2,35	0,04120	2,30	4,91	0,00218	NM_194292	RWDD4A
8103881	1,23	2,35	0,02151	1,83	3,56	0,00233	NM_152682	NAP1L4
7945786	1,23	2,35	0,00152	2,29	4,88	0,00147	NM_005969	TPD52L1
8121838	1,23	2,35	0,01042	1,06	2,09	0,02093	NM_001003395	SAFB
8024936	1,23	2,35	0,01067	1,60	3,03	0,00734	NM_002967	NPIP
7993546	1,23	2,35	0,14734	1,93	3,80	0,00025	NM_006985	MTERFD1
8151917	1,23	2,35	0,00271	2,10	4,28	0,00275	NM_015942	PIP4K2C
7956524	1,23	2,35	0,03805	3,52	11,50	0,00007	NM_024779	RN7SK // RN7SK
8120249	1,23	2,35	0,11104	1,48	2,78	0,00471	NR_001445 // NR_001445	UXS1
8054395	1,23	2,34	0,06064	2,47	5,52	0,00442	NM_025076	ZBTB1
7975068	1,23	2,34	0,07502	2,66	6,30	0,00324	NM_001123329	FAHD1
7992396	1,23	2,34	0,04484	1,30	2,45	0,05603	NM_031208	FBXO18
7925996	1,23	2,34	0,07531	2,45	5,46	0,00845	NM_032807	ULK2
8013399	1,23	2,34	0,06419	2,52	5,73	0,00263	NM_014683	RNF169
7942544	1,23	2,34	0,02699	1,76	3,39	0,00713	NM_001098638	NT5DC3
7965918	1,23	2,34	0,05054	2,36	5,14	0,00340	NM_001031701	KDM2A
7941769	1,23	2,34	0,02726	2,75	6,71	0,00036	NM_012308	DERL2
8011875	1,23	2,34	0,08446	3,81	14,05	0,00000	NM_016041	BDP1
8106025	1,22	2,34	0,02814	1,54	2,90	0,00336	NM_018429	TNFAIP8
8107520	1,22	2,34	0,00041	0,07	1,05	0,76497	NM_014350	LRRC37A4
8016245	1,22	2,34	0,00484	3,02	8,10	0,00024	NR_002940	FAM58A
7967879	1,22	2,33	0,07556	-0,14	-1,10	0,73339	NM_152274	TBC1D14
8093976	1,22	2,33	0,00745	3,29	9,77	0,00002	NM_020773	RPL19
8006845	1,22	2,33	0,04286	0,25	1,19	0,45547	NM_000981	SCCPDH
7911155	1,22	2,33	0,07827	1,74	3,34	0,00073	NM_016002	GNAS
8063668	1,22	2,33	0,00060	1,44	2,71	0,00441	NM_000516	C21orf63
8068202	1,22	2,33	0,00767	0,89	1,85	0,02907	NM_058187	DDA1
8026729	1,22	2,33	0,06464	1,34	2,52	0,00065	NM_024050	XYLT2
8008297	1,22	2,33	0,07208	2,32	5,00	0,00486	NM_022167	ERLIN2
8145835	1,22	2,33	0,01321	1,27	2,42	0,00105	NM_007175	EZR
8130505	1,22	2,33	0,00979	2,33	5,02	0,00095	NM_003379	ANKRD46
8152053	1,22	2,33	0,11240	2,98	7,91	0,00009	NM_198401	---
7896750	1,22	2,33	0,17832	-1,28	-2,43	0,21586	--- DAPK1	
8156199	1,22	2,33	0,12982	1,52	2,87	0,00166	NM_004938	ITGB3BP
7916727	1,22	2,33	0,02181	1,94	3,84	0,00282	NM_014288	HSDL1
8003116	1,22	2,33	0,05518	2,57	5,95	0,00207	NM_031463	SASH1
8122637	1,22	2,33	0,08193	1,30	2,47	0,00599	NM_015278	ARHGAP26
8108873	1,22	2,33	0,00573	1,67	3,18	0,00269	NM_015071	LOC342541
8009094	1,22	2,33	0,05712	1,06	2,09	0,05880	ENST00000416490	ADCY9
7999079	1,22	2,33	0,00772	0,97	1,96	0,01331	NM_001116	LGALS3
7974461	1,22	2,33	0,12158	0,29	1,22	0,43838	NR_003225	TPRA1
8090420	1,22	2,32	0,01051	3,09	8,54	0,00037	NM_016372	AKAP6
7973850	1,22	2,32	0,13113	1,21	2,31	0,06507	NM_004274	---

7997489	1,22	2,32	0,06329	2,45	5,45	0,00004	--- BCAS3	
8008933	1,22	2,32	0,00806	2,38	5,20	0,00187	NM_001099432	VPS13A
8155946	1,22	2,32	0,04123	2,66	6,34	0,00204	NM_033305	PDE3A
7954293	1,22	2,32	0,02983	-1,45	-2,74	0,02273	NM_000921	ZNF416
8039664	1,22	2,32	0,10545	1,20	2,29	0,01318	NM_017879	UBE2S
8012958	1,22	2,32	0,01822	1,68	3,22	0,00806	NM_014501	ADAMTS19
8107823	1,21	2,32	0,08454	-0,83	-1,78	0,05170	NM_133638	GJC1
8016094	1,21	2,32	0,06918	2,48	5,59	0,00493	NM_005497	TFE3
8172520	1,21	2,32	0,14987	2,35	5,10	0,01161	NM_006521	NDUFB8
7935810	1,21	2,32	0,00547	1,80	3,49	0,00548	NM_005004	CCNB1IP1
7977497	1,21	2,32	0,05597	0,46	1,38	0,07418	NM_021178	SIK3
7951873	1,21	2,32	0,07279	4,09	16,97	0,00003	NM_025164	NRXN3
7976012	1,21	2,32	0,07015	-1,12	-2,18	0,00035	NM_004796	BMS1
7927108	1,21	2,32	0,00331	1,16	2,24	0,08755	NM_014753	CYTH1
8018922	1,21	2,32	0,01180	3,06	8,35	0,00001	NM_004762	DNAJB4
7902512	1,21	2,32	0,14149	1,47	2,77	0,00517	NM_007034	MTCH1
8126066	1,21	2,31	0,04223	2,17	4,51	0,03415	NM_014341	C5orf33
8111552	1,21	2,31	0,11965	2,31	4,97	0,00155	NM_001085411	ZNF174
7992897	1,21	2,31	0,02144	1,93	3,81	0,00263	NM_003450	BRCA2
7968484	1,21	2,31	0,02186	1,96	3,90	0,00204	NM_000059	VAT1L
7997336	1,21	2,31	0,05437	-1,89	-3,72	0,00306	NM_020927	TBCB
8028162	1,21	2,31	0,04049	2,85	7,20	0,00567	NM_001281	ADAMTSL1
8154491	1,21	2,31	0,00281	-0,89	-1,85	0,11935	NM_001040272	KIAA0562
7911870	1,21	2,31	0,00583	0,02	1,01	0,94630	NM_014704	CREBL2
7954021	1,21	2,31	0,07847	1,69	3,22	0,00146	NM_001310	CKAP2L
8054702	1,21	2,31	0,04470	1,34	2,53	0,02929	NM_152515	MALL
8054479	1,21	2,31	0,00388	1,12	2,18	0,01812	NM_005434	PTMA // PTMA
7961022	1,21	2,31	0,12502	2,47	5,52	0,00015	NM_002823 // NM_002823 MED4	
7971550	1,21	2,31	0,11563	0,71	1,64	0,09129	NM_014166	IP6K1
8087473	1,21	2,31	0,01194	2,02	4,05	0,01153	NM_153273	DNASE2
8034565	1,21	2,31	0,00316	1,21	2,31	0,00735	NM_001375	SNRNP48
8116807	1,20	2,31	0,11335	3,41	10,65	0,00066	NM_152551	ZNF770
7987361	1,20	2,30	0,05359	2,56	5,89	0,00139	NM_014106	C12orf11
7961964	1,20	2,30	0,02277	2,14	4,39	0,00300	NM_018164	MAPRE2
8020878	1,20	2,30	0,02004	1,90	3,73	0,00019	NM_014268	GSTM4
7903742	1,20	2,30	0,08311	3,81	14,03	0,00027	NM_000850	SLC35C1
7939590	1,20	2,30	0,07863	1,60	3,03	0,00845	NM_018389	PRPF4B
8116664	1,20	2,30	0,02978	1,86	3,64	0,00290	NM_003913	CSRNP2
7963235	1,20	2,30	0,04821	2,49	5,62	0,00204	NM_030809	NARG2
7989347	1,20	2,30	0,12906	2,81	7,02	0,00108	NM_024611	LOC100130331
7910901	1,20	2,30	0,17513	0,50	1,41	0,73304	NR_027247	TAF10
7946221	1,20	2,30	0,01349	1,53	2,88	0,02254	NM_006284	MICA
8118100	1,20	2,30	0,03981	2,22	4,66	0,00121	NM_001177519	HIST1H1C
8124397	1,20	2,30	0,01361	1,08	2,12	0,01110	NM_005319	RPL7L1
8119595	1,20	2,30	0,04689	2,62	6,14	0,00197	NM_198486	COX4NB
8003217	1,20	2,29	0,05047	2,25	4,75	0,00016	NM_006067	IPO13
7900888	1,20	2,29	0,02899	2,07	4,20	0,00298	NM_014652	ALDOA
7994737	1,20	2,29	0,05158	1,72	3,29	0,00106	NM_000034	ZNF621
8079005	1,20	2,29	0,00715	1,02	2,03	0,00820	NM_198484	PSMB9
8118571	1,20	2,29	0,01365	0,24	1,18	0,27028	NM_002800	PSMB9
8178211	1,20	2,29	0,01365	0,24	1,18	0,27028	NM_002800	PSMB9
8179495	1,20	2,29	0,01365	0,24	1,18	0,27028	NM_002800	LRRC57
7987916	1,20	2,29	0,11427	1,51	2,86	0,00488	NM_153260	PGM5P2
8161520	1,19	2,29	0,08852	0,38	1,30	0,47198	NR_002836	GSTM2
7903753	1,19	2,29	0,02125	1,69	3,22	0,04557	NM_000848	PARP12
8143327	1,19	2,29	0,03597	2,29	4,88	0,00305	NM_022750	AP3M2
8146159	1,19	2,29	0,04576	1,99	3,98	0,00046	NM_001134296	SERPINB6
8123621	1,19	2,29	0,03001	1,92	3,79	0,00668	NM_004568	C17orf80
8009552	1,19	2,29	0,03122	2,15	4,43	0,00073	NM_017941	SNORD116-5
7981958	1,19	2,29	0,22058	-2,83	-7,11	0,01296	NR_003320	SNORD116-5
7981962	1,19	2,29	0,22058	-2,83	-7,11	0,01296	NR_003320	TIMP3
8072626	1,19	2,28	0,16304	-1,18	-2,26	0,00878	NM_000362	RPS19
8029129	1,19	2,28	0,06059	1,70	3,24	0,00429	NM_001022	RNF20
8156945	1,19	2,28	0,03477	2,68	6,42	0,00046	NM_019592	RBM4
7941709	1,19	2,28	0,10174	2,73	6,65	0,00106	NM_002896	TXNDC17
8004175	1,19	2,28	0,00293	1,15	2,22	0,02365	NM_032731	SMEK1
7980833	1,19	2,28	0,09434	2,57	5,92	0,00064	NM_032560	MYOCD
8005048	1,19	2,28	0,10544	-1,35	-2,54	0,00181	NM_001146312	---
8089230	1,19	2,28	0,20145	2,45	5,47	0,03964	--- QRSL1	
8121300	1,19	2,28	0,11319	2,02	4,06	0,00123	NM_018292	IGFBP7
8100541	1,19	2,28	0,03077	-1,67	-3,18	0,00015	NM_001553	MDC1
8124813	1,19	2,28	0,00854	2,16	4,47	0,00116	NM_014641	MDC1
8178404	1,19	2,28	0,00854	2,16	4,47	0,00116	NM_014641	PRPF19

7948476	1,19	2,28	0,02811	4,10	17,14	0,00011	NM_014502	NUP160
7947991	1,19	2,28	0,05407	3,06	8,32	0,00029	NM_015231	KIAA1462
7932826	1,19	2,28	0,05851	-0,07	-1,05	0,73656	NM_020848	---
8072122	1,19	2,28	0,47233	0,49	1,40	0,57062	--- YLPM1	
7975725	1,19	2,28	0,06031	2,81	7,01	0,00043	NM_019589	FAM102A
8164343	1,19	2,27	0,04202	3,11	8,61	0,00384	NM_001035254	TMEM135
7942964	1,19	2,27	0,02165	1,40	2,64	0,00182	NM_022918	SMG1
8002333	1,19	2,27	0,04138	2,37	5,17	0,00462	NM_015092	SP110
8059650	1,18	2,27	0,11530	0,01	1,01	0,95549	NM_080424	TUBE1
8128977	1,18	2,27	0,19001	3,86	14,50	0,00090	NM_016262	TMEFF1
8156905	1,18	2,27	0,16030	1,86	3,62	0,02730	NM_003692	UTP20
7957890	1,18	2,27	0,06549	2,46	5,49	0,00670	NM_014503	PLOD3
8141688	1,18	2,27	0,01547	0,81	1,75	0,20218	NM_001084	MPPE1
8022326	1,18	2,27	0,05507	3,80	13,92	0,00001	NM_023075	GALC
7980580	1,18	2,27	0,04187	2,46	5,52	0,00229	NM_000153	ATAD2B
8050658	1,18	2,27	0,02486	2,33	5,03	0,00321	NM_017552	PARD3
7933010	1,18	2,27	0,02338	1,34	2,54	0,00306	NM_019619	ARFIP2
7946201	1,18	2,27	0,08934	2,59	6,04	0,00061	NM_012402	CMBL
8110971	1,18	2,27	0,16407	2,22	4,66	0,00257	NM_138809	HIST2H2BA
7904465	1,18	2,26	0,13762	1,86	3,62	0,01605	NR_027337	MCTP1
8113130	1,18	2,26	0,09500	0,41	1,33	0,08588	NM_024717	PHF19
8163807	1,18	2,26	0,04327	3,21	9,22	0,00049	NM_015651	CC2D2A
8094190	1,18	2,26	0,02829	1,69	3,23	0,00453	NM_001080522	TBC1D8B
8169186	1,18	2,26	0,04534	-0,21	-1,15	0,21396	NM_017752	PTMA // PTMA
7954006	1,18	2,26	0,06396	2,56	5,90	0,00016	NM_002823 // NM_002823	C17orf48
8004940	1,18	2,26	0,06214	0,55	1,46	0,08839	NM_020233	ANXA2P1
8103240	1,18	2,26	0,05495	0,58	1,50	0,00104	NR_001562	TTLL1
8076547	1,18	2,26	0,04411	1,28	2,43	0,02106	NR_027779	ZNF383
8028227	1,17	2,26	0,04933	2,11	4,30	0,00235	NM_152604	IER3
8124848	1,17	2,26	0,06729	2,25	4,77	0,00014	NM_003897	IER3
8179704	1,17	2,26	0,06729	2,25	4,77	0,00014	NM_003897	ZNF382
8028194	1,17	2,26	0,07135	2,92	7,59	0,00088	NM_032825	PDZRN3
8088848	1,17	2,26	0,05999	-1,31	-2,48	0,00432	NM_015009	SKA3
7970513	1,17	2,26	0,10646	1,97	3,92	0,01605	NM_145061	ZNF347
8039054	1,17	2,26	0,05605	0,72	1,65	0,09390	NM_001172674	CREB3L2
8143188	1,17	2,26	0,03755	1,84	3,58	0,00175	NM_194071	NDUFAF3
8079707	1,17	2,26	0,07691	2,06	4,16	0,00619	NM_199069	AS3MT
7930181	1,17	2,25	0,04521	2,22	4,66	0,00311	NM_020682	CCDC109B
8096808	1,17	2,25	0,11482	1,90	3,72	0,00078	NM_017918	EDA2R
8173299	1,17	2,25	0,20020	-1,09	-2,12	0,00019	NM_021783	CBWD5
8155422	1,17	2,25	0,02312	1,71	3,27	0,01116	BC043420 FLRT2	
7976073	1,17	2,25	0,06668	-0,92	-1,90	0,00122	NM_013231	ZDHHC4
8131414	1,17	2,25	0,03676	2,94	7,69	0,00346	NM_001134387	MKL2
7993310	1,17	2,25	0,05092	1,72	3,29	0,03473	NM_014048	PLBD1
7961440	1,17	2,25	0,01140	1,91	3,75	0,00174	NM_024829	FAM172A
8113083	1,17	2,25	0,06636	0,66	1,57	0,03547	NM_032042	BIVM
7969916	1,17	2,25	0,00339	1,34	2,54	0,00547	NM_017693	PLEKHO1
7905116	1,17	2,25	0,00242	-0,38	-1,30	0,21927	NM_016274	POMZP3
8140433	1,17	2,25	0,02470	1,28	2,42	0,00343	NM_012230	TSLP
8107270	1,17	2,25	0,00538	-1,38	-2,59	0,01969	NM_033035	ZMAT2
8108620	1,17	2,25	0,04131	2,32	4,99	0,00420	NM_144723	RAP2C
8175169	1,17	2,25	0,01427	-0,14	-1,11	0,52136	NM_021183	HERC2P2
7982154	1,17	2,25	0,00349	2,85	7,22	0,00500	NR_002824	ZMYM6
7914764	1,17	2,25	0,07100	1,86	3,64	0,00171	NM_007167	COL5A2
8057620	1,17	2,25	0,02066	-0,39	-1,31	0,00302	NM_000393	HTR2B
8059680	1,17	2,25	0,14117	-0,66	-1,58	0,05741	NM_000867	C22orf30
8075542	1,17	2,25	0,00258	1,82	3,54	0,00018	NM_173566	MRPS9
8044111	1,17	2,25	0,00972	1,40	2,65	0,00604	NM_182640	TDP2
8124262	1,17	2,25	0,14068	2,09	4,27	0,00375	NM_016614	RBBP6
7994161	1,17	2,25	0,02905	2,12	4,35	0,00037	NM_006910	TNFRSF10B
8149733	1,17	2,25	0,04373	3,03	8,17	0,00260	NM_003842	ZNF808
8030946	1,17	2,25	0,01939	0,66	1,58	0,04003	NM_001039886	MRPS10
8126394	1,17	2,25	0,19732	1,96	3,88	0,00043	NM_018141	ANKHD1-EIF4EBP3
8108510	1,17	2,25	0,00401	2,01	4,03	0,00015	NM_020690	NSD1
8110289	1,17	2,25	0,00748	1,89	3,72	0,00091	NM_022455	RERE
7912166	1,17	2,25	0,05493	1,56	2,94	0,00284	NM_012102	CLK3
7984846	1,17	2,24	0,02286	0,94	1,92	0,00075	NM_001130028	DCAF13
8147785	1,17	2,24	0,00886	0,84	1,79	0,07671	NM_015420	RAD17
8177478	1,17	2,24	0,05785	2,56	5,89	0,01063	NM_133338	PIAS2
8023133	1,16	2,24	0,06970	1,23	2,34	0,01571	NM_004671	MYST3
8150491	1,16	2,24	0,12465	2,16	4,47	0,00345	NM_001099412	PHTF1
7918634	1,16	2,24	0,10354	1,48	2,78	0,00114	NM_006608	ADIPOR2
7953021	1,16	2,24	0,01400	3,54	11,61	0,00001	NM_024551	KCTD7

8133167	1,16	2,24	0,05181	2,03	4,08	0,00024	NM_153033	MORC4
8174338	1,16	2,24	0,00312	1,42	2,68	0,02085	NM_024657	RANBP17
8109938	1,16	2,23	0,00943	3,19	9,13	0,00077	NM_022897	MAP3K4
8123274	1,16	2,23	0,00726	1,99	3,97	0,00017	NM_005922	P704P
7977454	1,16	2,23	0,07573	0,21	1,15	0,21955	NM_001145442	GTF2IRD2B
8133549	1,16	2,23	0,03393	1,59	3,00	0,00053	NM_001003795	FIBIN
7939052	1,16	2,23	0,05086	-0,74	-1,67	0,03701	NM_203371	CD99L2
8175647	1,16	2,23	0,00973	1,51	2,86	0,00315	NM_031462	PSMA7
8067382	1,16	2,23	0,08023	1,75	3,36	0,00041	NM_002792	ACADSB
7931168	1,16	2,23	0,05363	2,00	4,00	0,00163	NM_001609	SORBS2
8104035	1,16	2,23	0,03746	-1,01	-2,01	0,00025	NM_021069	RETSAT
8053406	1,16	2,23	0,04009	1,95	3,87	0,01309	NM_017750	LPGAT1
7924107	1,15	2,23	0,27218	3,31	9,92	0,00016	NM_014873	VPS8
8084541	1,15	2,23	0,04639	2,65	6,26	0,00014	NM_001009921	TAPBP
8178977	1,15	2,23	0,00730	1,73	3,32	0,02217	NM_172208	MED19
7948293	1,15	2,23	0,01381	0,39	1,31	0,11258	NM_153450	ZNF880
8030925	1,15	2,22	0,07440	0,03	1,02	0,87495	NM_001145434	HSP90AB2P
8094165	1,15	2,22	0,02448	1,17	2,26	0,02556	NR_003132	KLC1
7977127	1,15	2,22	0,07542	3,15	8,90	0,00142	NM_001130107	TEAD3
8125887	1,15	2,22	0,08516	0,59	1,51	0,15989	NM_003214	---
8045088	1,15	2,22	0,37398	-3,25	-9,49	0,00049	--- SDHAP2	
8084904	1,15	2,22	0,10844	2,62	6,16	0,00246	NR_003265	---
7946567	1,15	2,22	0,09503	-2,97	-7,84	0,00056	--- ALG10B	
7954789	1,15	2,22	0,05114	2,42	5,35	0,00099	NM_001013620	LIMCH1
8094789	1,15	2,22	0,02719	0,29	1,23	0,19735	NM_014988	GTF2IRD2
8140170	1,15	2,22	0,01840	1,55	2,92	0,00065	NM_173537	NFIX
8026139	1,15	2,22	0,00982	0,22	1,17	0,37007	NM_002501	MCPH1
8144361	1,15	2,22	0,00254	1,27	2,41	0,13627	NM_024596	PLXNA1
8082314	1,15	2,22	0,01329	3,31	9,93	0,00109	NM_032242	RIOK1
8116760	1,15	2,22	0,00335	2,67	6,35	0,00111	NM_031480	TSN
8044965	1,15	2,22	0,09241	1,58	2,98	0,00046	NM_004622	FLNC
8135990	1,15	2,22	0,04682	1,02	2,03	0,11732	NM_001458	GPATCH2
7924327	1,15	2,22	0,15091	1,19	2,28	0,03186	NM_018040	GUSBP3
8112469	1,15	2,22	0,16706	1,88	3,67	0,00555	NR_027386	ECHDC1
8129379	1,15	2,22	0,10809	2,06	4,16	0,00762	NM_001002030	XPO5
8126588	1,15	2,22	0,15776	3,55	11,73	0,00043	NM_020750	CDKN2AIP
8098500	1,15	2,22	0,12553	0,83	1,77	0,03422	NM_017632	KCTD18
8058118	1,15	2,21	0,13660	1,03	2,04	0,00259	NM_152387	CASC2
7930861	1,15	2,21	0,03153	-0,20	-1,15	0,43427	NR_026939	AK5
7902452	1,15	2,21	0,09080	-0,73	-1,66	0,01805	NM_174858	RNU1-1
7973896	1,15	2,21	0,05881	-1,21	-2,32	0,10090	NR_004430	RNU1-1
7978568	1,15	2,21	0,05881	-1,21	-2,32	0,10090	NR_004430	RAD23A
8026122	1,14	2,21	0,12559	2,01	4,02	0,00991	NM_005053	SLC9A1
7914021	1,14	2,21	0,04729	2,34	5,07	0,00044	NM_003047	HIST2H2BF
7919606	1,14	2,21	0,06149	1,11	2,16	0,01910	NM_001024599	PSMC6
7974380	1,14	2,21	0,01192	2,06	4,18	0,00028	NM_002806	TOMM22
7930870	1,14	2,21	0,54245	3,19	9,14	0,01258	NM_020243	AQP3
8160670	1,14	2,21	0,03428	2,65	6,27	0,00215	NM_004925	WDR47
7918275	1,14	2,21	0,17666	1,82	3,54	0,00405	NM_001142550	TMEM144
8098041	1,14	2,21	0,02906	1,70	3,26	0,02765	NM_018342	TRIM16L
8005475	1,14	2,21	0,11041	2,84	7,18	0,00071	NM_001037330	BTD
8078147	1,14	2,21	0,23357	1,41	2,65	0,02627	NM_000060	PLK1
7994109	1,14	2,21	0,05231	2,38	5,19	0,00509	NM_005030	TK2
8001818	1,14	2,21	0,16250	2,40	5,27	0,00054	NM_004614	LRP8
7916282	1,14	2,21	0,01680	3,57	11,86	0,00042	NM_004631	PIP5K1A
7905365	1,14	2,21	0,25888	4,79	27,68	0,00036	NM_001135638	CCDC132
8134219	1,14	2,21	0,02330	1,97	3,91	0,01154	NM_017667	MLH3
7980246	1,14	2,20	0,02325	2,45	5,46	0,00232	NM_001040108	SORD
7983393	1,14	2,20	0,07501	3,30	9,82	0,00024	NM_003104	HLA-F
8177717	1,14	2,20	0,04822	2,27	4,82	0,00982	NM_018950	FITM2
8066407	1,14	2,20	0,03591	1,84	3,57	0,00541	NM_001080472	BST1
8094228	1,14	2,20	0,14158	1,04	2,05	0,05396	NM_004334	CUTA
8125752	1,14	2,20	0,04954	2,82	7,08	0,00024	NM_015921	SLC5A6
8051030	1,14	2,20	0,02410	3,47	11,06	0,00234	NM_021095	POT1
8142697	1,14	2,20	0,14630	1,91	3,76	0,00180	NM_015450	CHEK2
8075164	1,14	2,20	0,02314	2,29	4,88	0,00020	NM_001005735	CASP1
7951397	1,14	2,20	0,09528	-1,42	-2,67	0,01767	NM_033292	GXYLT1
7962349	1,14	2,20	0,29258	2,30	4,91	0,00399	NM_173601	---
8165646	1,14	2,20	0,14656	-1,09	-2,12	0,03737	--- PEX1	
8140915	1,14	2,20	0,09497	1,85	3,61	0,01563	NM_000466	GTF2H2
8112538	1,14	2,20	0,00360	1,85	3,59	0,00236	NM_001515	GTF2H2
8177507	1,14	2,20	0,00360	1,85	3,59	0,00236	NM_001515	DIS3
7971967	1,14	2,20	0,03881	1,53	2,89	0,01885	NM_014953	SYTL2

7950810	1,13	2,20	0,03023	-0,06	-1,04	0,80471	NM_206927	AATF
8006690	1,13	2,20	0,10014	2,60	6,05	0,00006	NM_012138	GUSBP3
8112564	1,13	2,19	0,17270	1,91	3,76	0,00553	NR_027386	TRMT112
7949075	1,13	2,19	0,17292	4,45	21,83	0,00059	NM_016404	NLN
8105663	1,13	2,19	0,02556	2,03	4,09	0,00101	NM_020726	MTMR6
7970655	1,13	2,19	0,01038	2,19	4,56	0,00324	NM_004685	MTHFD1L
8161484	1,13	2,19	0,02239	0,87	1,82	0,01305	NM_015440	MRPL32
8132458	1,13	2,19	0,13569	0,47	1,39	0,08092	NM_031903	TRAPPC10
8068974	1,13	2,19	0,00328	2,30	4,93	0,00495	NM_003274	NUSAP1
7982889	1,13	2,19	0,05798	2,27	4,82	0,08701	NM_016359	PLEKHH2
8041644	1,13	2,19	0,06402	1,14	2,20	0,00304	NM_172069	DBNL
8132539	1,13	2,19	0,00328	1,01	2,02	0,15058	NM_014063	CCT8
8069753	1,13	2,19	0,09915	1,18	2,27	0,06326	NM_006585	ETS2
8068593	1,13	2,19	0,09460	3,46	10,99	0,00020	NM_005239	RPL5
7903010	1,13	2,19	0,00043	0,86	1,81	0,00269	NM_000969	C11orf63
7944751	1,13	2,19	0,03673	0,08	1,05	0,76605	NM_024806	GRIK2
8121225	1,13	2,19	0,06741	-0,26	-1,20	0,20038	NM_175768	MRPL43
7935819	1,13	2,19	0,02345	2,42	5,35	0,00042	NM_032112	TSC22D3
8174361	1,13	2,19	0,02302	2,57	5,94	0,00232	NM_198057	TPRKB
8053036	1,13	2,19	0,01389	1,26	2,39	0,06329	NM_016058	AP2S1
8037847	1,13	2,19	0,02410	0,86	1,81	0,11407	NM_004069	HNRNPA3
8080991	1,13	2,19	0,19626	1,14	2,20	0,03155	NM_194247	PRTFDC1
7932584	1,13	2,19	0,04104	2,57	5,95	0,00402	NM_020200	ADCY3
8050766	1,13	2,19	0,00291	3,71	13,12	0,00258	NM_004036	POLI
8021275	1,13	2,18	0,07831	0,91	1,88	0,07149	NM_007195	MBTPS2
8166382	1,13	2,18	0,01013	1,46	2,75	0,00266	NM_015884	DCAF7
8009164	1,13	2,18	0,06344	3,38	10,40	0,00061	NM_005828	KHDRBS3
8148448	1,13	2,18	0,05113	-1,16	-2,23	0,01096	NM_006558	---
8165650	1,13	2,18	0,42116	-1,49	-2,81	0,00112	--- RASA4	
8141843	1,13	2,18	0,11836	1,48	2,79	0,30055	NM_006989	NDEL1
8004867	1,13	2,18	0,04632	3,14	8,79	0,00004	NM_001025579	SFMBT2
7931951	1,13	2,18	0,08007	3,45	10,92	0,00042	NM_001029880	RAB7L1
7923812	1,12	2,18	0,04568	2,03	4,07	0,00088	NM_003929	MT1B
7995820	1,12	2,18	0,00102	0,77	1,70	0,01858	NM_005947	FRYL
8100292	1,12	2,18	0,05644	0,58	1,49	0,30339	NM_015030	FAM111A
7940153	1,12	2,18	0,03936	1,60	3,02	0,00918	NM_022074	RPL10
8170859	1,12	2,18	0,03661	3,03	8,14	0,04881	NR_026898	PFKFB3
7926037	1,12	2,18	0,00344	2,60	6,07	0,00042	NM_004566	ZBTB26
8164002	1,12	2,18	0,11779	1,12	2,17	0,08501	NM_020924	FOSL2
8041048	1,12	2,18	0,00006	1,71	3,26	0,00954	NM_005253	UBE2E3
8046685	1,12	2,18	0,29734	1,99	3,98	0,00484	NM_182678	MRPS5
8053763	1,12	2,18	0,00390	1,74	3,33	0,00567	NM_031902	CPT2
7901549	1,12	2,18	0,01490	1,64	3,12	0,01413	NM_000098	ICK
8127109	1,12	2,18	0,02841	2,34	5,08	0,00788	NM_016513	GLI2
8044933	1,12	2,18	0,05989	0,75	1,68	0,01792	NM_005270	SMYD5
8042705	1,12	2,18	0,02264	2,72	6,57	0,00054	NM_006062	DGAT1
8153684	1,12	2,18	0,09428	2,77	6,83	0,00191	NM_012079	DGAT1
8153939	1,12	2,18	0,09428	2,77	6,83	0,00191	NM_012079	ZNF763
8025978	1,12	2,18	0,25554	-0,54	-1,45	0,05088	NM_001012753	CTSZ
8067279	1,12	2,18	0,00019	1,11	2,15	0,07384	NM_001336	HERC6
8096335	1,12	2,18	0,13914	2,55	5,85	0,00071	NM_017912	RBM14
7941694	1,12	2,18	0,03516	1,62	3,08	0,00554	NM_006328	TIMM10
7948267	1,12	2,17	0,05064	2,67	6,38	0,00166	NM_012456	PID1
8059565	1,12	2,17	0,03493	-0,74	-1,68	0,02363	NM_017933	MTR
7910752	1,12	2,17	0,00143	1,95	3,87	0,00261	NM_000254	GTF3C3
8057933	1,12	2,17	0,09919	3,16	8,93	0,00309	NM_012086	ST3GAL3
7900857	1,12	2,17	0,02290	1,63	3,09	0,00205	NM_174963	SLC11A2
7963212	1,12	2,17	0,07465	3,31	9,94	0,00104	NM_001174126	AMAC1
8014230	1,12	2,17	0,07451	-1,02	-2,03	0,15906	NM_152462	MGC72080
8082248	1,12	2,17	0,42443	0,90	1,87	0,35217	NR_002822	PSPH
8137464	1,12	2,17	0,07842	3,56	11,81	0,00067	NM_004577	WBP5
8169022	1,12	2,17	0,04742	-0,34	-1,27	0,18536	NM_016303	GUSBP3
8112491	1,12	2,17	0,01359	2,48	5,60	0,00104	NR_027386	PAPPA
8163672	1,12	2,17	0,14282	-0,57	-1,49	0,04914	NM_002581	FKBP4
7953100	1,12	2,17	0,10473	1,94	3,83	0,05958	NM_002014	STAG1
8090898	1,12	2,17	0,13326	1,13	2,19	0,02349	NM_005862	SCD
7935776	1,12	2,17	0,11378	0,16	1,12	0,74478	NM_005063	SCAP
8086754	1,12	2,17	0,09437	1,45	2,74	0,04154	NM_012235	SFRS13A
8039947	1,11	2,17	0,02400	1,31	2,48	0,00115	NM_006625	UTP23
8147994	1,11	2,16	0,13491	2,55	5,86	0,00071	NM_032334	UBE2D3
8102024	1,11	2,16	0,08889	2,21	4,63	0,00450	NM_181886	CFDP1
8002865	1,11	2,16	0,09991	0,98	1,97	0,03271	NM_006324	CRKL
8071434	1,11	2,16	0,00525	3,11	8,65	0,00247	NM_005207	PDK1

8046408	1,11	2,16	0,03298	2,08	4,24	0,00051	NM_002610	---
7942520	1,11	2,16	0,03524	1,08	2,12	0,01230	--- TRNT1	
8077353	1,11	2,16	0,02124	0,89	1,86	0,09191	NM_182916	SYNJ1
8070010	1,11	2,16	0,03253	1,41	2,65	0,00137	NM_003895	NFU1
8052784	1,11	2,16	0,02136	0,52	1,43	0,06960	NM_015700	LONRF1
8149399	1,11	2,16	0,00841	1,60	3,03	0,00703	NM_152271	ZNF788
8025992	1,11	2,16	0,01458	-0,62	-1,54	0,27103	NR_027049	H2AFV
8139421	1,11	2,16	0,13877	3,22	9,31	0,00031	NM_012412	NOP58
8047518	1,11	2,16	0,01719	3,03	8,20	0,00012	NM_015934	DHX57
8051637	1,11	2,16	0,05176	0,73	1,66	0,13764	NM_198963	CUL4A
7970262	1,11	2,16	0,04576	2,48	5,56	0,00003	NM_001008895	ANKRD10
7972828	1,11	2,15	0,01495	2,09	4,26	0,00008	NM_017664	CLGN
8102877	1,11	2,15	0,01710	1,67	3,19	0,00960	NM_004362	GTF3C2
8051075	1,11	2,15	0,12507	3,60	12,09	0,00103	NM_001521	NPIP
7999769	1,11	2,15	0,22296	1,67	3,17	0,00014	NM_006985	SLC25A43
8169624	1,11	2,15	0,02563	0,90	1,87	0,06271	NM_145305	C9orf85
8155794	1,11	2,15	0,23819	1,94	3,83	0,01706	NM_182505	ZNF702P
8039025	1,11	2,15	0,06183	0,29	1,22	0,04922	NR_003578	NUP188
8158446	1,11	2,15	0,16437	3,59	12,05	0,00010	NM_015354	CHERP
8035156	1,11	2,15	0,06404	1,74	3,34	0,00084	NM_006387	TAF1
8168303	1,11	2,15	0,05356	1,61	3,06	0,04024	NM_004606	JMJD8
7998267	1,11	2,15	0,03423	2,43	5,38	0,01503	NM_001005920	TRAF5
7909545	1,11	2,15	0,02800	1,63	3,09	0,00351	NM_145759	STRN3
7978428	1,10	2,15	0,15014	2,23	4,68	0,00471	NM_001083893	RPS15
8024299	1,10	2,15	0,23753	3,13	8,73	0,04385	NM_001018	KCTD13
8000738	1,10	2,15	0,11873	3,38	10,43	0,00147	NM_178863	OTUD1
7926677	1,10	2,15	0,12804	3,21	9,23	0,00124	NM_001145373	CIB1
7991386	1,10	2,15	0,12301	1,94	3,84	0,00322	NM_006384	GCLM
7917779	1,10	2,15	0,03557	1,09	2,12	0,01062	NM_002061	SH3RF1
8103630	1,10	2,15	0,02035	1,28	2,43	0,00275	NM_020870	KCTD14
7950641	1,10	2,15	0,08421	2,20	4,60	0,00079	NM_023930	IKBIP
7965681	1,10	2,15	0,07214	0,26	1,20	0,45645	NM_153687	MED27
8164698	1,10	2,15	0,24647	0,55	1,46	0,28833	NM_004269	EIF3F
7938263	1,10	2,14	0,17814	0,65	1,57	0,35730	NM_003754	NET1
7925954	1,10	2,14	0,09091	2,62	6,16	0,00025	NM_001047160	CENPV
8013015	1,10	2,14	0,07152	2,13	4,39	0,00238	NM_181716	RNGTT
8128052	1,10	2,14	0,03344	2,76	6,76	0,00072	NM_003800	DCTN1
8053107	1,10	2,14	0,02233	1,99	3,96	0,00022	NM_004082	RTKN
8053142	1,10	2,14	0,01724	3,09	8,49	0,00001	NM_033046	EHD2
8029950	1,10	2,14	0,02924	0,57	1,49	0,30868	NM_014601	GAS6
7970329	1,10	2,14	0,07844	-1,06	-2,08	0,01550	NM_000820	ZNF384
7960666	1,10	2,14	0,00792	1,75	3,36	0,00992	NM_133476	FAM185A
8135211	1,10	2,14	0,23305	2,83	7,09	0,00803	NR_026879	KLHL7
8131815	1,10	2,14	0,03056	2,10	4,29	0,00169	NR_033328	TECR
8026341	1,09	2,14	0,06271	2,60	6,05	0,00079	NM_138501	DHFR
8022640	1,09	2,14	0,18630	2,42	5,34	0,00288	NM_000791	C14orf45
7975676	1,09	2,14	0,04481	0,08	1,05	0,61770	NM_025057	DCTN3
8160839	1,09	2,13	0,20595	2,62	6,13	0,00548	NM_024348	OLA1
8056812	1,09	2,13	0,17363	1,32	2,50	0,00540	NM_013341	OGDH
8132592	1,09	2,13	0,10447	3,12	8,67	0,00261	NM_002541	ZW10
7951734	1,09	2,13	0,09528	1,57	2,98	0,00773	NM_004724	ZBTB37
7907486	1,09	2,13	0,03482	2,00	4,01	0,01470	NM_032522	DHFR
8112902	1,09	2,13	0,09640	2,18	4,54	0,00381	NM_000791	SH3KBP1
8165674	1,09	2,13	0,27510	-1,73	-3,32	0,00525	AY423734 MALT1	
8021418	1,09	2,13	0,18178	2,13	4,39	0,01013	NM_006785	C2orf34
8041745	1,09	2,13	0,08356	3,32	9,99	0,00015	NM_024766	ADAMTS9
8088560	1,09	2,13	0,06023	-0,88	-1,84	0,01288	NM_182920	KIAA0146
8146336	1,09	2,13	0,09585	2,04	4,11	0,00014	AK301677 MTERF	
8140859	1,09	2,13	0,05143	0,91	1,88	0,07407	NM_006980	LYN
8146500	1,09	2,13	0,11560	2,86	7,27	0,00131	NM_002350	SLC31A2
8157264	1,09	2,13	0,09893	1,70	3,25	0,00442	NM_001860	DLGAP5
7979307	1,09	2,12	0,02094	2,79	6,93	0,00063	NM_014750	RECQL
7961654	1,09	2,12	0,14467	0,54	1,45	0,25322	NM_002907	RBM23
7977803	1,09	2,12	0,01340	2,69	6,44	0,00204	NM_001077351	ZFP37
8163444	1,09	2,12	0,00503	1,28	2,43	0,06890	NM_003408	C5orf62
8109326	1,09	2,12	0,06082	-0,58	-1,50	0,14877	NM_032947	PBX2
8083221	1,09	2,12	0,00388	2,27	4,83	0,00119	NM_002586	PRKG1
7927606	1,09	2,12	0,11214	-0,95	-1,93	0,00288	NM_001098512	CALCOCO1
7963721	1,09	2,12	0,01621	1,59	3,02	0,00900	NM_020898	FBXO25
8144239	1,09	2,12	0,05189	0,37	1,29	0,41136	NM_183421	ABLIM3
8109093	1,08	2,12	0,10159	1,45	2,73	0,00143	NM_014945	---
7904569	1,08	2,12	0,58467	-0,54	-1,45	0,02514	--- PPP4C	
7994756	1,08	2,12	0,03214	2,87	7,29	0,00321	NM_002720	KIAA1328

8091550	1,08	2,12	0,05046	1,93	3,82	0,03109	NM_020776	SAP30BP
8009932	1,08	2,12	0,01825	1,02	2,03	0,02372	NM_013260	SFRS12
8105714	1,08	2,12	0,02156	0,86	1,81	0,01659	NM_139168	GTF2E2
8150103	1,08	2,12	0,14093	2,59	6,04	0,00300	NM_002095	DCAF4
7975506	1,08	2,11	0,05223	0,77	1,71	0,01202	NM_015604	HTT
8093685	1,08	2,11	0,06218	2,67	6,35	0,00372	NM_002111	PPP3CC
8145136	1,08	2,11	0,01126	1,97	3,92	0,00781	NM_005605	C20orf69
8123520	1,08	2,11	0,18285	1,62	3,08	0,04226	BC118988 KGFLP2	
8161362	1,08	2,11	0,04585	0,23	1,17	0,32363	NR_003670	SNRPB
8064502	1,08	2,11	0,02444	2,94	7,66	0,00126	NM_003091	FLNB
8080714	1,08	2,11	0,02279	2,75	6,74	0,02699	NM_001164317	C6orf204
8129193	1,08	2,11	0,01206	-0,11	-1,08	0,44735	NM_001178035	PHF2
8156476	1,08	2,11	0,04537	2,94	7,67	0,00091	NM_005392	CHI3L2
7903920	1,08	2,11	0,00078	-0,63	-1,55	0,00292	NM_001025199	FGF2
8097256	1,08	2,11	0,06748	0,45	1,37	0,33718	NM_002006	CSRP2
7965090	1,08	2,11	0,59362	-0,85	-1,80	0,37470	NM_001321	ATP8B2
7905754	1,08	2,11	0,00430	1,19	2,28	0,02456	NM_020452	PPID
8103437	1,08	2,11	0,04403	0,59	1,51	0,07726	NM_005038	CAMK1
8085206	1,08	2,11	0,03374	0,50	1,42	0,37775	NM_003656	CCDC104
8042066	1,08	2,11	0,00881	0,91	1,88	0,06068	NM_080667	MMP16
8151686	1,08	2,11	0,02572	-1,31	-2,47	0,01138	NM_005941	GLTP
7966259	1,08	2,11	0,02696	0,78	1,72	0,04672	NM_016433	SPOCK3
8103544	1,08	2,11	0,01059	-0,70	-1,63	0,00546	NM_001040159	DMTF1
8133914	1,08	2,11	0,00262	1,96	3,89	0,00456	NR_024549	VTN
8013606	1,08	2,11	0,14415	-1,85	-3,60	0,00037	NM_000638	DNAJB11
8084634	1,08	2,11	0,03155	2,30	4,93	0,00318	NM_016306	INSIG1
8137526	1,07	2,11	0,12413	0,74	1,67	0,02043	NM_005542	NBPF16
7904874	1,07	2,11	0,56524	-0,25	-1,19	0,32577	NM_001102663	PPIE
7900354	1,07	2,11	0,04977	0,98	1,97	0,02877	NM_006112	UBR7
7976336	1,07	2,11	0,00397	2,28	4,84	0,00232	NM_175748	CHID1
7945579	1,07	2,11	0,13281	1,85	3,60	0,01354	NM_001142674	GTF2H2D
8177674	1,07	2,10	0,00053	1,80	3,47	0,00246	NM_001042490	C17orf53
8007569	1,07	2,10	0,02378	2,63	6,21	0,00349	NM_024032	NAIP
8112478	1,07	2,10	0,01188	1,66	3,16	0,00650	NM_004536	TSNAX
7910550	1,07	2,10	0,09196	2,13	4,38	0,00599	NM_005999	CCDC102B
8021685	1,07	2,10	0,03455	-0,73	-1,66	0,04943	NM_001093729	BAZ1B
8140037	1,07	2,10	0,02192	2,18	4,53	0,00268	NM_032408	GUSBP3
8105937	1,07	2,10	0,01112	2,21	4,62	0,00093	BT006760 RNPC3	
7903404	1,07	2,10	0,21947	-0,88	-1,84	0,22777	NM_017619	NAALADL2
8083978	1,07	2,10	0,24951	-0,27	-1,21	0,37367	NM_207015	IL17RA
8071069	1,07	2,10	0,05784	1,89	3,69	0,00029	NM_014339	ATG12
8113651	1,07	2,10	0,07556	1,88	3,68	0,00387	NR_033362	TRRAP
8134470	1,07	2,10	0,05954	2,82	7,05	0,00033	NM_003496	C14orf179
7975851	1,07	2,10	0,07709	1,63	3,09	0,00727	NM_001102564	MED24
8014925	1,07	2,10	0,03253	1,88	3,68	0,01497	NM_014815	PDK4
8141094	1,07	2,10	0,17376	0,89	1,85	0,06191	NM_002612	WDR37
7925851	1,07	2,10	0,00647	1,95	3,86	0,00025	NM_014023	PDCL3
8043902	1,07	2,10	0,13050	2,15	4,43	0,00772	NM_024065	HNRNPF
7933164	1,07	2,10	0,04514	2,41	5,32	0,00262	NM_004966	PGRMC2
8102745	1,07	2,10	0,01111	0,55	1,46	0,09034	NM_006320	DNAJB5
8154962	1,07	2,09	0,01528	2,48	5,56	0,00216	NM_001135004	MBTD1
8016789	1,07	2,09	0,03873	2,13	4,37	0,00165	NM_017643	ZFYVE26
7979757	1,07	2,09	0,00361	2,80	6,97	0,00026	NM_015346	FAM46A
8127778	1,07	2,09	0,03756	0,35	1,28	0,00676	NM_017633	C6orf48
8178090	1,06	2,09	0,04392	5,02	32,34	0,00111	NM_001040437	C6orf48
8179326	1,06	2,09	0,04392	5,02	32,34	0,00111	NM_001040437	PI4KB
7919950	1,06	2,09	0,07914	2,36	5,15	0,00063	NM_002651	---
8089038	1,06	2,09	0,26747	-3,09	-8,53	0,00133	--- ---	
8112914	1,06	2,09	0,26747	-3,09	-8,53	0,00133	--- MTMR12	
8111339	1,06	2,09	0,07606	3,89	14,84	0,00146	NM_001040446	BAG3
7930921	1,06	2,09	0,08450	0,86	1,82	0,07631	NM_004281	PPP2R3A
8082869	1,06	2,09	0,11819	1,86	3,63	0,00546	NM_002718	ELOVL6
8102342	1,06	2,09	0,10347	3,09	8,53	0,00005	NM_024090	ZFX
8166500	1,06	2,09	0,12272	3,30	9,84	0,00004	NM_003410	SLC25A30
7971388	1,06	2,09	0,15389	1,41	2,66	0,00584	NM_001010875	RPL27A
7938295	1,06	2,09	0,01064	0,80	1,74	0,08840	NM_000990	ANKRD42
7942858	1,06	2,09	0,01721	0,66	1,58	0,02030	NM_182603	CENPK
8112376	1,06	2,09	0,05517	4,20	18,37	0,00006	NM_022145	ZNF585B
8036389	1,06	2,09	0,07645	3,09	8,50	0,00043	NM_152279	---
7950444	1,06	2,09	0,05466	-1,60	-3,02	0,01626	--- KIAA0907	
7920852	1,06	2,09	0,00144	2,49	5,63	0,00012	BC062637 CCNG2	
8095870	1,06	2,09	0,01190	0,71	1,63	0,04462	NM_004354	SSBP3
7916403	1,06	2,09	0,10556	0,83	1,77	0,11639	NM_145716	MSH6

8041888	1,06	2,09	0,03552	3,05	8,26	0,00017	NM_000179	MINA
8089040	1,06	2,08	0,03604	2,02	4,07	0,00173	NM_001042533	RNF13
8083310	1,06	2,08	0,32103	3,43	10,79	0,00003	NM_007282	SLC41A3
8090295	1,06	2,08	0,14206	1,92	3,77	0,01606	NM_017836	SEC14L1
8010139	1,06	2,08	0,08720	2,79	6,93	0,00095	NM_001143998	NBPF10
7904506	1,06	2,08	0,62307	-0,87	-1,83	0,00787	NM_001039703	NBPF3
7913380	1,06	2,08	0,57354	-0,88	-1,85	0,01028	NM_032264	DHRS1
7978260	1,06	2,08	0,11877	3,33	10,04	0,00007	NM_001136050	LYST
7925257	1,06	2,08	0,12564	0,92	1,90	0,00563	NM_000081	CMIP
7997427	1,06	2,08	0,10613	4,80	27,86	0,00016	NM_198390	BHLHE41
7961891	1,06	2,08	0,00149	-1,78	-3,43	0,00010	NM_030762	HEXIM1
8007745	1,06	2,08	0,12099	2,32	5,00	0,00067	NM_006460	CDC40
8121461	1,06	2,08	0,06785	2,29	4,88	0,00064	NM_015891	AXL
8029006	1,05	2,08	0,01304	2,91	7,51	0,00704	NM_021913	C15orf40
7991049	1,05	2,08	0,08611	1,87	3,66	0,00837	NM_144597	SLC25A28
7935639	1,05	2,08	0,22323	2,68	6,39	0,00001	NM_031212	GSS
8065817	1,05	2,08	0,21293	3,25	9,55	0,00123	NM_000178	---
8085114	1,05	2,08	0,10731	-2,37	-5,16	0,08934	--- USP6NL	
7932023	1,05	2,08	0,01179	3,17	8,97	0,00002	NM_014688	APAF1
7957759	1,05	2,08	0,05680	0,83	1,78	0,11332	NM_181861	RAB27A
7989023	1,05	2,08	0,00528	0,85	1,81	0,03791	NM_004580	GPR133
7959893	1,05	2,07	0,03860	-1,19	-2,28	0,05733	NM_198827	FBXO36
8048889	1,05	2,07	0,07779	0,86	1,82	0,08146	NM_174899	CHML
7925500	1,05	2,07	0,08984	1,92	3,78	0,00234	NM_001821	08. Sep
8114050	1,05	2,07	0,05726	2,07	4,19	0,00043	NM_015146	PKIA
8147012	1,05	2,07	0,02734	-0,56	-1,47	0,12886	NM_006823	ORMDL3
8014916	1,05	2,07	0,02486	0,82	1,77	0,01868	NM_139280	DCAKD
8016159	1,05	2,07	0,10920	3,81	14,05	0,00001	NM_024819	EFNA1
7905929	1,05	2,07	0,00021	3,03	8,15	0,00205	NM_004428	RGS10
7936673	1,05	2,07	0,15579	1,24	2,36	0,02679	NM_001005339	GPHN
7975167	1,05	2,07	0,05777	2,13	4,39	0,00060	NM_020806	RAPGEF2
8098121	1,05	2,07	0,02545	0,47	1,39	0,14981	NM_014247	C14orf153
7977119	1,05	2,07	0,00020	2,08	4,22	0,00268	NM_032374	MIS12
8004144	1,05	2,06	0,14092	1,98	3,95	0,00212	NM_024039	ALG6
7901915	1,05	2,06	0,09091	1,74	3,33	0,00272	NM_013339	GLB1L
8059158	1,05	2,06	0,00781	1,82	3,54	0,00040	NM_024506	U2AF2
8031536	1,04	2,06	0,04083	1,67	3,19	0,00787	NM_007279	MGC72080
8156319	1,04	2,06	0,37155	0,21	1,16	0,73951	NR_002822	FAM192A
8001537	1,04	2,06	0,02012	1,62	3,08	0,01052	NM_024946	GTF3C4
8158918	1,04	2,06	0,03285	1,97	3,93	0,00148	NM_012204	SECTM1
8019486	1,04	2,06	0,02441	1,14	2,20	0,00538	NM_003004	---
8112959	1,04	2,06	0,05810	1,06	2,09	0,00488	--- RAB8A	
8026520	1,04	2,06	0,06779	1,16	2,24	0,00581	NM_005370	MVP
7994659	1,04	2,06	0,04630	0,29	1,22	0,55797	NM_017458	RPL12
8125750	1,04	2,06	0,03081	1,51	2,85	0,00134	NM_000976	FKBP1A
8064432	1,04	2,06	0,32651	2,23	4,69	0,01743	NM_000801	INTS12
8102162	1,04	2,06	0,09092	1,79	3,46	0,02161	NM_020395	HNRNPAB
8110450	1,04	2,06	0,00436	2,48	5,58	0,00872	NM_031266	YOD1
7923967	1,04	2,06	0,18083	1,36	2,58	0,02904	NM_018566	SNORA20
8130578	1,04	2,06	0,03208	-0,69	-1,61	0,00902	NR_002960	FAM193A
8093601	1,04	2,05	0,03210	2,06	4,18	0,00750	NM_003704	FBXO21
7966760	1,04	2,05	0,01490	2,54	5,83	0,00050	NM_033624	TMEM194B
8057736	1,04	2,05	0,08427	1,39	2,63	0,05203	NM_001142645	ABHD12
8065469	1,04	2,05	0,01091	0,84	1,79	0,05637	NM_001042472	CYP2U1
8096744	1,04	2,05	0,07843	1,08	2,11	0,03662	NM_183075	USP48
7913415	1,04	2,05	0,04697	2,09	4,27	0,00166	NM_032236	PYGB
8061447	1,04	2,05	0,06723	2,89	7,42	0,00046	NM_002862	BLVRB
8036913	1,04	2,05	0,17802	2,06	4,17	0,00138	NM_000713	MED12
8168215	1,04	2,05	0,08561	2,75	6,71	0,00047	NM_005120	PARP2
7973036	1,04	2,05	0,14319	2,77	6,82	0,00063	NM_005484	BAT2
8118174	1,04	2,05	0,02885	1,74	3,33	0,00486	NM_080686	DDX26B
8170027	1,04	2,05	0,06117	1,96	3,90	0,00595	NM_182540	SCAMP3
7920725	1,03	2,05	0,07069	2,10	4,28	0,00028	NM_005698	SFRS18
8128394	1,03	2,05	0,02677	2,58	5,98	0,00451	NM_032870	NTN4
7965573	1,03	2,05	0,14617	2,79	6,94	0,00024	NM_021229	SYDE1
8026407	1,03	2,05	0,09426	1,69	3,23	0,00299	NM_033025	RBM7
7944006	1,03	2,05	0,04289	1,49	2,81	0,02548	NM_016090	SLCO3A1
7986214	1,03	2,05	0,09267	-0,99	-1,98	0,00230	NM_013272	IFI35
8007446	1,03	2,05	0,00271	0,60	1,51	0,04569	NM_005533	POMT2
7980358	1,03	2,05	0,03039	3,30	9,86	0,00040	NM_013382	ADH1A
8101874	1,03	2,04	0,05314	-0,68	-1,60	0,01001	NM_000667	SYMPK
8037695	1,03	2,04	0,07095	2,56	5,88	0,00004	NM_004819	YPEL2
8008819	1,03	2,04	0,07622	1,67	3,18	0,01227	NM_001005404	MEIS3P1

8005695	1,03	2,04	0,06870	2,69	6,47	0,00002	NR_002211	UBE2W
8151401	1,03	2,04	0,14969	2,47	5,54	0,01132	NM_001001481	ZNF844
8025984	1,03	2,04	0,03376	0,05	1,03	0,87410	NM_001136501	ATG4A
8169272	1,03	2,04	0,07546	1,56	2,95	0,00372	NM_052936	TM2D1
7916643	1,03	2,04	0,07436	1,59	3,02	0,02760	NM_032027	DHX30
8079563	1,03	2,04	0,02623	1,98	3,96	0,00008	NM_138615	STK3
8151967	1,03	2,04	0,07332	1,18	2,27	0,01032	NM_006281	AMOTL2
8090852	1,03	2,04	0,04259	2,12	4,36	0,02006	NM_016201	KBTBD4
7947917	1,03	2,04	0,13138	1,11	2,16	0,07899	NM_016506	ABLIM1
7936463	1,02	2,03	0,11293	3,23	9,40	0,00117	NM_002313	KLHDC1
7974214	1,02	2,03	0,09035	1,31	2,48	0,11250	NM_172193	CEP135
8095187	1,02	2,03	0,14109	1,68	3,21	0,01313	NM_025009	SYNC
7914557	1,02	2,03	0,17483	-0,55	-1,47	0,13918	NM_030786	F3
7917875	1,02	2,03	0,00279	0,95	1,93	0,00487	NM_001993	KIF1B
7897561	1,02	2,03	0,02648	2,27	4,83	0,00066	NM_015074	ZNF622
8111129	1,02	2,03	0,16576	1,22	2,33	0,03162	NM_033414	SNRPE
7908988	1,02	2,03	0,00968	0,71	1,64	0,01227	NM_003094	MTHFD1L
8155563	1,02	2,03	0,02203	0,79	1,73	0,01170	NM_015440	GINS2
8003204	1,02	2,03	0,03899	3,05	8,30	0,00261	NM_016095	NDUFAF1
7987642	1,02	2,03	0,06543	2,26	4,78	0,00172	NM_016013	MAX
7979671	1,02	2,03	0,00356	0,71	1,63	0,10123	NM_145113	EIF4EBP1
8145889	1,02	2,03	0,25072	1,88	3,69	0,00212	NM_004095	ZNF548
8031737	1,02	2,03	0,03639	-0,52	-1,43	0,02610	NM_001172773	ZFP91-CNTF
7940118	1,02	2,03	0,03860	0,44	1,36	0,23579	NR_024091	CRLF3
8014037	1,02	2,03	0,05962	2,17	4,49	0,00130	NM_015986	SIAE
7944867	1,02	2,03	0,55891	-3,58	-11,99	0,01323	NM_170601	SND1
8135876	1,02	2,03	0,03764	1,17	2,24	0,13316	NM_014390	FUT10
8150165	1,02	2,02	0,02056	1,41	2,65	0,02417	NM_032664	ZMYND11
7925792	1,02	2,02	0,04210	2,34	5,05	0,00182	NM_006624	WDR67
8148158	1,02	2,02	0,10596	2,14	4,40	0,01227	NM_145647	CCDC28A
8122327	1,02	2,02	0,04213	1,39	2,62	0,00307	NM_015439	FLJ43681
7911333	1,02	2,02	0,03648	0,26	1,20	0,60780	NR_029406	RPL23AP82
8069470	1,02	2,02	0,03648	0,26	1,20	0,60780	NR_026981	NAA10
8175924	1,02	2,02	0,01857	3,35	10,21	0,00220	NM_003491	CCDC46
8017736	1,02	2,02	0,04607	0,36	1,28	0,40109	NM_145036	NDUFB9
8148270	1,01	2,02	0,01696	2,16	4,47	0,03572	NM_005005	SGOL2
8047288	1,01	2,02	0,13792	1,76	3,38	0,02819	NM_152524	---
7922523	1,01	2,02	0,10492	2,89	7,43	0,00081	--- PRKCE	
8041763	1,01	2,02	0,00956	2,65	6,27	0,00007	NM_005400	---
7915563	1,01	2,02	0,03202	1,54	2,90	0,01770	--- RBM4B	
7949674	1,01	2,02	0,21018	2,31	4,94	0,00896	NM_031492	TTC28
8075142	1,01	2,02	0,06908	2,57	5,94	0,00122	NM_001145418	ACER3
7942679	1,01	2,02	0,20742	3,80	13,90	0,00342	NM_018367	ZBED5
7946635	1,01	2,02	0,03710	1,13	2,18	0,00428	NM_021211	---
8065254	1,01	2,02	0,05919	2,68	6,43	0,00015	--- TRIP13	
8104234	1,01	2,02	0,06874	3,63	12,36	0,00071	NM_004237	SENP8
7984626	1,01	2,02	0,07631	0,22	1,17	0,47258	NM_145204	ARHGEF9
8173217	1,01	2,02	0,08716	2,27	4,82	0,00028	NM_015185	TRPC4
7971104	1,01	2,02	0,01644	-0,77	-1,71	0,01133	NM_016179	FEM1C
8113616	1,01	2,02	0,05880	1,63	3,09	0,00057	NM_020177	NAPB
8065372	1,01	2,02	0,14220	1,56	2,94	0,00267	NM_022080	DNAJA2
8001185	1,01	2,02	0,08395	3,08	8,47	0,00040	NM_005880	MRPL19
8042962	1,01	2,01	0,07491	1,10	2,15	0,00752	NM_014763	THAP2
7957161	1,01	2,01	0,01884	0,68	1,60	0,23767	NM_031435	SVIP
7947147	1,01	2,01	0,19850	1,26	2,39	0,04687	NM_148893	ABCC9
7961710	1,01	2,01	0,19571	-0,75	-1,68	0,00391	NM_005691	ZNF24
8022882	1,01	2,01	0,30781	3,19	9,10	0,00047	NM_006965	COPE
8035553	1,01	2,01	0,06604	1,76	3,38	0,03308	NM_007263	CTDSPL
8078650	1,01	2,01	0,15717	3,23	9,35	0,00000	NM_001008392	XAF1
8004184	1,01	2,01	0,04608	-0,93	-1,91	0,00678	NM_017523	MAGOHB
7961208	1,01	2,01	0,15158	1,48	2,78	0,00866	NM_018048	IFI44
7902553	1,01	2,01	0,17806	-0,74	-1,67	0,03927	NM_006417	IL17RD
8088264	1,01	2,01	0,12933	0,94	1,92	0,00514	NM_017563	ZNF460
8031714	1,01	2,01	0,25229	1,79	3,46	0,00080	NM_006635	ALG13
8169419	1,01	2,01	0,01131	0,75	1,69	0,01344	NM_001099922	HNRNPA0
8114320	1,01	2,01	0,03206	2,72	6,57	0,00494	NM_006805	WDR59
8002802	1,01	2,01	0,02995	2,97	7,81	0,00042	NM_030581	GGCX
8053429	1,01	2,01	0,11069	1,92	3,80	0,00037	NM_000821	RPL18A
8026868	1,01	2,01	0,02316	0,87	1,83	0,00133	NM_000980	DNAJC5
8064208	1,01	2,01	0,08756	0,52	1,43	0,04032	NM_025219	TMEM175
8093413	1,01	2,01	0,11044	0,83	1,77	0,07090	NM_032326	DDX20
7904000	1,01	2,01	0,13049	1,65	3,13	0,00558	NM_007204	MRPL33
8041027	1,01	2,01	0,14502	1,93	3,81	0,01441	NM_004891	---

7954243	1,01	2,01	0,11096	0,11	1,08	0,67639	---	TUBGCP4
7983206	1,00	2,01	0,07000	4,13	17,52	0,00082	NM_014444	DDB2
7939738	1,00	2,01	0,02465	0,86	1,82	0,00685	NM_000107	RNMT
8020323	1,00	2,01	0,05271	1,66	3,16	0,01203	NM_003799	GMCL1
8042487	1,00	2,00	0,04305	2,32	4,99	0,01148	NM_178439	MRPS18C
8096109	1,00	2,00	0,12438	0,82	1,76	0,11333	NM_016067	AKAP1
8008664	1,00	2,00	0,08485	3,35	10,17	0,00001	NM_003488	RAP1GDS1
8096538	1,00	2,00	0,07935	2,17	4,49	0,00041	NM_001100426	CYFIP1
7981824	1,00	2,00	0,03486	1,10	2,15	0,01219	NM_014608	GALK2
7983616	1,00	2,00	0,03655	2,96	7,79	0,00007	NM_001001556	LUC7L2
8136539	1,00	2,00	0,09015	2,58	5,97	0,00496	NM_016019	CTNNBIP1
7912283	1,00	2,00	0,13611	1,57	2,98	0,00227	NM_020248	SENP2
8084607	1,00	2,00	0,07387	2,48	5,57	0,00006	NM_021627	SLC7A5
8003298	1,00	2,00	0,12729	3,74	13,36	0,00026	NM_003486	LRRC37A2
8007867	1,00	2,00	0,00391	2,66	6,31	0,00007	NM_001006607	AKR1C1
7925918	1,00	2,00	0,17531	-0,20	-1,15	0,80630	NM_001353	MTO1
8120698	1,00	2,00	0,05306	1,68	3,20	0,00031	NM_133645	SLC20A2
8150537	1,00	2,00	0,00851	1,87	3,65	0,00394	NM_006749	UBE2S
8039491	1,00	2,00	0,01458	1,26	2,39	0,00605	NM_014501	COX17
7968972	1,00	2,00	0,03220	0,36	1,28	0,28585	NM_005694	LIMK1
8133413	1,00	2,00	0,03267	1,76	3,40	0,01678	NM_002314	ZNF611
8038981	1,00	2,00	0,00609	0,35	1,28	0,03005	NM_030972	GALM
8041542	1,00	2,00	0,11549	2,62	6,15	0,00069	NM_138801	KRT18
7955637	1,00	2,00	0,38744	3,01	8,06	0,01046	NM_199187	FBXW8
7959039	1,00	2,00	0,08981	1,67	3,18	0,00133	NM_153348	PER3
7897378	1,00	2,00	0,06192	0,29	1,23	0,22677	NM_016831	BDNF
7947230	1,00	2,00	0,12554	0,74	1,67	0,07049	NM_170732	---
8131867	1,00	2,00	0,32268	1,16	2,23	0,24326	---	ALOX5AP
7968344	1,00	2,00	0,17721	2,90	7,49	0,00636	NM_001629	B3GALNT2
7925229	1,00	2,00	0,01344	3,67	12,71	0,00008	NM_152490	CDON
7952526	1,00	2,00	0,04410	2,64	6,24	0,00003	NM_016952	C13orf27
7972674	1,00	2,00	0,11989	2,05	4,13	0,00234	NM_138779	C20orf3
8065433	1,00	2,00	0,06570	1,53	2,89	0,00255	NM_020531	GLIPR2
8155192	1,00	1,99	0,24298	0,48	1,39	0,02617	NM_022343	TRIP10
8025058	1,00	1,99	0,06113	3,51	11,43	0,00066	NM_004240	LPIN2
8022022	1,00	1,99	0,09213	0,99	1,98	0,03623	NM_014646	ZSWIM1
8063074	1,00	1,99	0,04674	1,48	2,79	0,02518	NM_080603	C1orf163
7916219	1,00	1,99	0,20266	3,07	8,40	0,00012	NM_023077	INTS8
8147396	1,00	1,99	0,03627	3,03	8,17	0,00026	NM_017864	SETDB1
7905258	1,00	1,99	0,12185	2,25	4,76	0,00049	NM_001145415	MCCC1
8092328	1,00	1,99	0,01616	1,59	3,01	0,00255	NM_020166	ZNF134
8031784	1,00	1,99	0,24205	2,44	5,43	0,00201	NM_003435	STEAP4
8140840	0,99	1,99	0,19480	0,92	1,89	0,29530	NM_024636	GGA2
8000284	0,99	1,99	0,15043	2,07	4,19	0,00109	NM_015044	PIK3R3
7915787	0,99	1,99	0,10114	1,51	2,85	0,03180	NM_003629	HNRNPA3
8046590	0,99	1,99	0,09256	1,02	2,03	0,06677	NM_194247	DRG2
8005313	0,99	1,99	0,04456	1,47	2,77	0,01108	NM_001388	GUSBP3
8177544	0,99	1,99	0,16697	2,24	4,71	0,00179	NR_027386	ORC2L
8058161	0,99	1,99	0,07081	2,34	5,05	0,00183	NM_006190	ZER1
8164464	0,99	1,99	0,00757	1,99	3,97	0,00713	NM_006336	TRAF3IP2
8128939	0,99	1,99	0,01557	1,53	2,89	0,00157	NR_028338	ZNF836 // ZNF836 // ZNF836
8038962	0,99	1,99	0,13076	0,79	1,73	0,10881	NM_001102657 // NM_001102657 // NM_001102657	FTSJD2
8119198	0,99	1,99	0,24956	2,36	5,14	0,00136	NM_015050	PCBP1
8042515	0,99	1,99	0,13211	2,24	4,72	0,00078	NM_006196	FAM114A2
8115375	0,99	1,99	0,07330	1,10	2,14	0,03379	NM_018691	NEDD4L
8021376	0,99	1,99	0,11489	3,21	9,25	0,00006	NM_001144967	NHEDC2
8102050	0,99	1,99	0,10186	2,12	4,34	0,00007	NM_178833	TRIM44
7939368	0,99	1,99	0,14575	2,15	4,44	0,01328	NM_017583	ACSL5
7930498	0,99	1,99	0,02759	-0,49	-1,40	0,05747	NM_016234	SNF8
8016508	0,99	1,99	0,22164	3,33	10,06	0,00000	NM_007241	PHF20L1
8148358	0,99	1,99	0,12360	2,13	4,37	0,00142	NM_016018	ARSB
8112807	0,99	1,99	0,03243	0,53	1,45	0,00654	NM_000046	DPM2
8164336	0,99	1,98	0,00837	2,56	5,90	0,00060	NM_003863	ARL17A
8016285	0,99	1,98	0,01664	1,70	3,26	0,00780	NM_001113738	AES
8032576	0,99	1,98	0,01092	1,26	2,39	0,07556	NM_198969	PCCA
7969835	0,99	1,98	0,09727	1,81	3,50	0,00050	NM_000282	HLA-F
8117760	0,99	1,98	0,05216	1,91	3,75	0,00965	NM_001098479	ALG10
7954777	0,99	1,98	0,07445	2,47	5,55	0,00409	NM_032834	ZNF826
8035789	0,99	1,98	0,00543	1,89	3,71	0,04234	NM_001039884	RHOU
7910387	0,98	1,98	0,10487	1,32	2,49	0,10117	NM_021205	STX18
8099121	0,98	1,98	0,10930	1,04	2,06	0,01731	NM_016930	CGGBP1

8089000	0,98	1,98	0,08655	2,51	5,69	0,00010	NM_001008390	UBA7
8087485	0,98	1,98	0,05814	-0,58	-1,50	0,02531	NM_003335	C5orf42
8111584	0,98	1,97	0,02087	1,14	2,20	0,00997	BC144069 CASP2	
8136869	0,98	1,97	0,01709	2,65	6,26	0,00219	NM_032982	CPM
7964834	0,98	1,97	0,15460	4,24	18,93	0,00016	NM_001874	C11orf46
7939087	0,98	1,97	0,12521	2,03	4,08	0,00177	BC047775 HINFP	
7944435	0,98	1,97	0,13898	2,88	7,37	0,00169	NM_015517	RFC2
8140151	0,98	1,97	0,08651	2,46	5,49	0,00028	NM_181471	HERC2P4
8001099	0,98	1,97	0,14854	1,75	3,36	0,06412	NR_002827	TMEM120B
7959298	0,98	1,97	0,04685	2,76	6,76	0,01120	NM_001080825	CHCHD2
8139758	0,98	1,97	0,06501	0,20	1,15	0,62132	NM_016139	TDG
7958147	0,98	1,97	0,23579	1,32	2,49	0,05171	NM_003211	PPTC7
7966345	0,98	1,97	0,24255	2,63	6,17	0,00115	NM_139283	GSK3A
8037152	0,98	1,97	0,03425	2,49	5,61	0,00003	NM_019884	DLST
7975747	0,98	1,97	0,19319	3,46	10,97	0,00009	NM_001933	PBK
8149955	0,98	1,97	0,00201	2,99	7,96	0,00541	NM_018492	POLR2I
8036276	0,98	1,97	0,08019	2,19	4,57	0,01443	NM_006233	TRIM68
7945979	0,98	1,97	0,11520	2,40	5,28	0,00001	NM_018073	TXNDC9
8054217	0,98	1,97	0,09242	1,27	2,42	0,06419	NM_005783	ATP6V1G2
8124942	0,98	1,97	0,15684	1,29	2,45	0,01032	NM_130463	ATP6V1G2
8179762	0,98	1,97	0,15684	1,29	2,45	0,01032	NM_130463	FIS1
8141717	0,97	1,97	0,27476	1,15	2,22	0,00867	NM_016068	ITPR1
8077376	0,97	1,96	0,01339	0,00	-1,00	0,99387	NM_001168272	UBE2NL // UBE2NL
8170298	0,97	1,96	0,00500	0,50	1,42	0,11254	NM_001012989 // NM_001012989	
	WIPI2							
8131303	0,97	1,96	0,01298	1,14	2,20	0,01101	NM_015610	HLA-F
8179019	0,97	1,96	0,08018	2,08	4,24	0,00926	NM_018950	DENND1A
8164034	0,97	1,96	0,04998	1,59	3,02	0,00750	NM_020946	STX10
8034631	0,97	1,96	0,16682	1,53	2,88	0,14474	NM_003765	ARAF
8167165	0,97	1,96	0,10101	2,04	4,12	0,00162	NM_001654	SLC35D1
7916808	0,97	1,96	0,09791	1,53	2,89	0,00063	NM_015139	PRDM5
8102567	0,97	1,96	0,01705	0,47	1,38	0,13839	NM_018699	RRM2B
8152133	0,97	1,96	0,05887	1,12	2,18	0,00261	NM_015713	TPK1
8143643	0,97	1,96	0,18194	0,75	1,68	0,06517	NM_022445	TDRD3
7969341	0,97	1,96	0,00731	1,39	2,62	0,01453	NM_030794	THG1L
8109576	0,97	1,96	0,03085	1,82	3,53	0,00045	NM_017872	C2orf18
8040698	0,97	1,96	0,13555	2,59	6,01	0,00059	NM_017877	C7orf11
8137008	0,97	1,96	0,43952	-2,66	-6,32	0,00144	AK290098 ---	
8132290	0,97	1,96	0,26768	0,54	1,45	0,43882	--- ZNF66	
8027268	0,97	1,96	0,00237	0,50	1,41	0,09469	BC067843 ---	
8081233	0,97	1,96	0,24302	0,41	1,33	0,72472	--- SHC1	
7920600	0,97	1,96	0,07969	1,90	3,72	0,09101	NM_183001	TSPAN9
7953166	0,97	1,96	0,05938	1,04	2,06	0,03044	NM_006675	ICT1
8009727	0,97	1,96	0,07014	2,84	7,18	0,00158	NM_001545	CYB5R4
8120943	0,97	1,95	0,18156	1,67	3,18	0,00102	NM_016230	ZNF277
8135497	0,97	1,95	0,02140	1,35	2,54	0,00774	NM_021994	FAM89B
7941302	0,97	1,95	0,00912	2,47	5,52	0,02083	NM_001098785	UNK
8009995	0,97	1,95	0,01308	1,74	3,34	0,01368	NM_001080419	NR2F1
8106923	0,97	1,95	0,04223	-0,76	-1,69	0,00105	NM_005654	NAP1L5
8101723	0,97	1,95	0,09868	-0,14	-1,10	0,56050	NM_153757	UST
8122660	0,96	1,95	0,14796	-0,30	-1,23	0,27810	NM_005715	PNPT1
8052331	0,96	1,95	0,16020	2,55	5,87	0,00101	NM_033109	SMC2
8156982	0,96	1,95	0,06580	2,86	7,28	0,00018	NM_001042551	AP1S1
8135080	0,96	1,95	0,03545	2,54	5,83	0,00334	NM_001283	FYCO1
8086572	0,96	1,95	0,05177	1,13	2,19	0,02831	NM_024513	SERAC1
8130474	0,96	1,95	0,03595	1,93	3,82	0,00104	NM_032861	LNX2
7970716	0,96	1,95	0,02476	3,36	10,27	0,00047	NM_153371	TRAPPC3
7914887	0,96	1,95	0,20078	2,58	5,99	0,00082	NM_014408	GSN
8157582	0,96	1,95	0,03016	0,71	1,63	0,05713	NM_000177	C21orf7
8068083	0,96	1,95	0,06089	1,59	3,01	0,00271	NM_020152	FOXN2
8041902	0,96	1,95	0,05913	0,91	1,88	0,05296	NM_002158	NPHP3
8090737	0,96	1,95	0,08713	0,77	1,71	0,03827	NM_153240	KRT10
8015104	0,96	1,94	0,01541	-0,21	-1,16	0,16077	NM_000421	CYC1
8148728	0,96	1,94	0,10676	3,72	13,21	0,00207	NM_001916	ODZ3
8098441	0,96	1,94	0,02224	-0,98	-1,97	0,00133	NM_001080477	MAP3K11
7949454	0,96	1,94	0,03231	1,32	2,49	0,04375	NM_002419	MTA1
7977373	0,96	1,94	0,04414	3,59	12,07	0,00007	NM_004689	HIBCH
8057719	0,96	1,94	0,04118	1,25	2,38	0,02042	NM_014362	PTER
7926356	0,96	1,94	0,11566	2,45	5,47	0,00666	NM_001001484	MOSC1
7909877	0,96	1,94	0,00205	2,44	5,43	0,00044	NM_022746	CCNE2
8151871	0,96	1,94	0,08067	2,59	6,02	0,00020	NM_057749	DUSP14
8006736	0,96	1,94	0,30406	1,97	3,92	0,01082	NM_007026	COX17
8089795	0,96	1,94	0,02485	0,60	1,52	0,11537	NM_005694	FSCN1

8131339	0,96	1,94	0,20271	2,26	4,80	0,02991	NM_003088	BUB1
8054580	0,96	1,94	0,03719	3,54	11,65	0,00412	NM_004336	SHPRH
8130038	0,96	1,94	0,06364	2,03	4,08	0,00312	NM_001042683	DOCK5
8145365	0,96	1,94	0,02828	3,56	11,81	0,00013	NM_024940	PTPLA
7932420	0,95	1,94	0,07671	1,34	2,53	0,03219	NM_014241	CNDP2
8021727	0,95	1,94	0,02444	1,26	2,39	0,02282	NM_018235	ZMYM5
7970428	0,95	1,94	0,17982	1,11	2,16	0,02908	NM_001039650	LRRC37A3
8017688	0,95	1,94	0,00832	2,64	6,25	0,00023	NM_199340	NDE1
7993467	0,95	1,93	0,11468	1,75	3,36	0,00046	NM_001143979	NSUN4
7901219	0,95	1,93	0,18200	3,06	8,34	0,00004	NM_199044	CASC3
8007028	0,95	1,93	0,05851	3,28	9,74	0,00085	NM_007359	ACN9
8134415	0,95	1,93	0,00462	0,59	1,51	0,08097	NM_020186	INO80D
8058415	0,95	1,93	0,12895	1,70	3,25	0,01892	NM_017759	---
8165669	0,95	1,93	0,28025	-1,79	-3,45	0,00301	---	MAST2
7901140	0,95	1,93	0,02307	1,97	3,92	0,00398	NM_015112	LRRC37A2
8007850	0,95	1,93	0,00960	2,59	6,02	0,00014	NM_001006607	PIGA
8171418	0,95	1,93	0,04554	1,73	3,31	0,02699	NM_002641	TSPAN7
8166784	0,95	1,93	0,02350	1,14	2,21	0,00809	NM_004615	DPP7
8165438	0,95	1,93	0,09720	2,11	4,32	0,00319	NM_013379	---
8057613	0,95	1,93	0,37495	-1,00	-2,00	0,45741	---	ANGPT1
8152297	0,95	1,93	0,03997	-0,97	-1,96	0,02342	NM_001146	C15orf57
7987526	0,95	1,93	0,07946	2,24	4,74	0,00073	NM_001080791	POLD2
8139299	0,95	1,93	0,10208	2,70	6,48	0,00331	NM_001127218	NBPF9
7904486	0,94	1,92	0,66695	-1,01	-2,01	0,00379	AK294414	C6orf99
8123101	0,94	1,92	0,08309	-0,66	-1,58	0,28898	ENST00000367073	APH1B
7984124	0,94	1,92	0,25266	0,95	1,94	0,00805	NM_031301	FGF5
8096050	0,94	1,92	0,18502	-1,84	-3,57	0,00003	NM_004464	RORA
7989365	0,94	1,92	0,00856	0,63	1,55	0,01700	NM_134260	SIL1
8114476	0,94	1,92	0,06734	0,47	1,38	0,17516	NM_001037633	DVL3
8084323	0,94	1,92	0,08713	2,16	4,48	0,01345	NM_004423	SUPT16H
7977674	0,94	1,92	0,14827	2,60	6,05	0,00008	NM_007192	SIAH1
8001306	0,94	1,92	0,01291	0,92	1,89	0,02169	NM_001006610	RGS9
8009277	0,94	1,92	0,03168	1,32	2,50	0,01266	NM_003835	C6orf130
8126259	0,94	1,92	0,19447	1,46	2,74	0,00412	AJ420538	CRY2
7939595	0,94	1,92	0,06155	0,10	1,08	0,78701	NM_021117	TOP2B
8085815	0,94	1,91	0,17792	2,37	5,17	0,00033	NM_001068	PVR
8029437	0,94	1,91	0,00371	2,98	7,87	0,00083	NM_006505	STIM2
8094501	0,94	1,91	0,03032	1,01	2,01	0,02255	NM_001169118	TMC7
7993588	0,94	1,91	0,03523	3,14	8,82	0,00018	NM_024847	---
8009513	0,94	1,91	0,11451	-0,18	-1,14	0,77341	---	LUZP1
7913558	0,94	1,91	0,16409	1,21	2,31	0,00199	NM_033631	SCG2
8059345	0,94	1,91	0,24163	-0,49	-1,41	0,03192	NM_003469	SPRYD4
7956257	0,94	1,91	0,00567	1,11	2,16	0,00482	NM_207344	CLK2P // CLK2P // CLK2P // CLK2P // CLK2P // CLK2P // CLK2P
8138596	0,94	1,91	0,21966	2,66	6,31	0,00021	NR_002711 // NR_002711 // NR_002711 // NR_002711 // NR_002711 // NR_002711 // NR_002711	---
7946563	0,93	1,91	0,47756	-2,61	-6,10	0,00256	---	NFXL1
8100179	0,93	1,91	0,08831	2,73	6,65	0,00253	NM_152995	FLVCR1
7909628	0,93	1,91	0,08929	3,77	13,62	0,00063	NM_014053	ZDHHC5
7940051	0,93	1,91	0,15946	2,62	6,16	0,00122	NM_015457	PTRF
8015635	0,93	1,91	0,03075	0,90	1,86	0,07135	NM_012232	FAM151B
8106626	0,93	1,91	0,14485	0,28	1,21	0,32091	AY358256	MTMR14
8077550	0,93	1,91	0,02751	1,34	2,53	0,00301	NM_001077525	ZC3H7B
8073397	0,93	1,91	0,03430	1,47	2,77	0,00460	NM_017590	ZHX2
8148149	0,93	1,91	0,21417	2,80	6,95	0,00001	NM_014943	SNX16
8151572	0,93	1,91	0,29628	2,34	5,05	0,00157	NM_022133	SNRPG
8026339	0,93	1,91	0,05058	2,53	5,78	0,00611	NM_003096	ENOPH1
8096081	0,93	1,91	0,19435	1,42	2,68	0,01090	NM_021204	HERC2P4
8001067	0,93	1,91	0,15073	1,68	3,22	0,02409	NR_002827	NBPF16
7904999	0,93	1,91	0,56038	-0,29	-1,22	0,37906	NM_001102663	CRYBB2P1
8072009	0,93	1,91	0,19201	2,00	4,01	0,01477	BC047380	MYNN
8083794	0,93	1,91	0,10825	1,50	2,82	0,01954	NM_018657	KIAA0247
7975361	0,93	1,91	0,01317	2,50	5,67	0,00369	NM_014734	SOCS4
7974447	0,93	1,91	0,08688	1,98	3,96	0,00110	NM_199421	ISYNA1
8035465	0,93	1,91	0,10790	2,61	6,09	0,00300	NM_016368	HAGH
7998604	0,93	1,90	0,08208	1,63	3,09	0,00041	NM_005326	SEC22A
8082153	0,93	1,90	0,24216	1,14	2,21	0,00984	NM_012430	CLEC2B
7961083	0,93	1,90	0,15694	-0,75	-1,69	0,02340	NM_005127	CTCF
7996593	0,93	1,90	0,03066	2,09	4,27	0,00173	NM_006565	C20orf112
8065612	0,93	1,90	0,05804	0,81	1,76	0,00209	NM_080616	HIST1H1E
8117377	0,93	1,90	0,31296	2,61	6,09	0,17456	NM_005321	C19orf62
8026698	0,93	1,90	0,08028	1,42	2,67	0,07051	NM_001033549	LOC220594
8005689	0,93	1,90	0,09286	2,38	5,19	0,00432	NR_003554	CCDC123

8035958	0,93	1,90	0,02166	1,13	2,19	0,00717	NM_032816	NPY
8131919	0,93	1,90	0,08005	-1,64	-3,12	0,00120	NM_000905	CD82
7939546	0,93	1,90	0,00880	-0,51	-1,43	0,35278	NM_002231	MGAT5
8045349	0,93	1,90	0,02275	1,91	3,77	0,00090	NM_002410	TMEM38B
8157092	0,93	1,90	0,25792	3,22	9,32	0,00185	NM_018112	TP53TG1
8140739	0,93	1,90	0,21113	-0,78	-1,72	0,03118	AB007455 TRMT5	
7979516	0,92	1,90	0,11671	1,78	3,44	0,00152	NM_020810	TNFRSF12A
7992789	0,92	1,90	0,35998	3,29	9,79	0,01934	NM_016639	FAM120A
8156452	0,92	1,90	0,01080	1,88	3,68	0,00577	NM_014612	HSF2
8121757	0,92	1,90	0,00520	0,57	1,48	0,00747	NM_004506	---
8056763	0,92	1,90	0,19250	3,59	12,04	0,00290	--- ZNF593	
7899096	0,92	1,90	0,02355	0,86	1,82	0,21652	NM_015871	MXD1
8042503	0,92	1,90	0,00070	2,05	4,15	0,00324	NM_002357	B3GAT3
7948741	0,92	1,90	0,43066	3,92	15,13	0,00181	NM_012200	NCRNA00171
8178324	0,92	1,90	0,04580	0,72	1,64	0,12288	AF032109 CPEB1	
7990971	0,92	1,89	0,04275	0,03	1,02	0,83543	NM_030594	BAT3
8178526	0,92	1,89	0,04388	2,83	7,09	0,00264	NM_004639	BAT3
8179782	0,92	1,89	0,04388	2,83	7,09	0,00264	NM_004639	ZNF639
8084035	0,92	1,89	0,15204	2,21	4,64	0,00007	NM_016331	PINK1
7898663	0,92	1,89	0,00348	1,31	2,48	0,04017	NM_032409	NFATC3
7996744	0,92	1,89	0,06376	3,13	8,75	0,00008	NM_173163	PHF16
8167013	0,92	1,89	0,03435	1,31	2,47	0,00086	NM_014735	SIK2
7943760	0,92	1,89	0,00108	1,47	2,78	0,01501	NM_015191	FLJ16171
8115871	0,92	1,89	0,38082	-1,54	-2,92	0,01654	AK131247 INPP5F	
7930927	0,92	1,89	0,16070	1,72	3,30	0,00026	NM_014937	MANF
8080084	0,92	1,89	0,01830	2,00	4,01	0,01192	NM_006010	ZC3HAV1L
8143270	0,92	1,89	0,07305	2,79	6,91	0,00010	NM_080660	---
8112918	0,92	1,89	0,25103	-2,16	-4,47	0,00348	--- MLLT1	
8033118	0,92	1,89	0,02759	0,92	1,89	0,04050	NM_005934	TIMM22
8003627	0,92	1,89	0,16539	2,45	5,48	0,00486	NM_013337	CEP78
8156026	0,92	1,89	0,04162	3,27	9,62	0,00040	NM_001098802	SEPN1
7899043	0,92	1,89	0,06852	2,13	4,38	0,01797	NM_020451	ZC3H13
7971422	0,92	1,89	0,03970	0,36	1,28	0,17110	NM_015070	TMEM67
8147313	0,92	1,89	0,23035	3,19	9,15	0,00058	NM_153704	NFKBIZ
8081386	0,92	1,89	0,07245	0,79	1,73	0,05404	NM_031419	SVIL
7932796	0,92	1,89	0,01943	0,56	1,48	0,01496	NM_021738	HAUS6
8160284	0,91	1,89	0,20441	2,65	6,28	0,02060	NM_017645	ADAMTS3
8100870	0,91	1,88	0,02752	-0,53	-1,45	0,22806	NM_014243	SLC35B2
8126658	0,91	1,88	0,16873	3,72	13,16	0,00215	NM_178148	---
8130495	0,91	1,88	0,14284	0,71	1,64	0,14085	--- PPIL4	
8130087	0,91	1,88	0,02971	1,99	3,96	0,00374	NM_139126	FAM20A
8017867	0,91	1,88	0,09517	-1,98	-3,95	0,00136	NM_017565	ZFYVE1
7979984	0,91	1,88	0,10626	1,33	2,52	0,01456	NM_021260	GSTM5
7903777	0,91	1,88	0,10374	-0,14	-1,10	0,15353	NM_000851	TMEM63A
7924636	0,91	1,88	0,09495	3,11	8,63	0,00902	NM_014698	NBPF15
7904976	0,91	1,88	0,56363	-0,29	-1,23	0,33686	NM_001170755	CFL1
7949496	0,91	1,88	0,10692	2,57	5,92	0,02825	NM_005507	TMTC2
7957417	0,91	1,88	0,24960	1,05	2,07	0,04098	NM_152588	IFI44L
7902541	0,91	1,88	0,13416	-1,00	-2,00	0,02956	NM_006820	KIAA0831
7979328	0,91	1,88	0,14346	2,11	4,30	0,00038	NM_014924	PNPLA4
8171229	0,91	1,88	0,24327	1,53	2,89	0,01241	NM_004650	C17orf85
8011462	0,91	1,88	0,06732	2,18	4,54	0,00721	NM_018553	---
8160258	0,91	1,88	0,33911	0,39	1,31	0,18003	--- GPR108	
8033300	0,91	1,88	0,17244	3,14	8,79	0,00022	NM_001080452	ARHGAP11B
7982287	0,91	1,88	0,35521	2,55	5,86	0,00054	NM_001039841	BAT2
8178011	0,91	1,88	0,02571	1,58	2,98	0,00367	NM_080686	F8A1
8170998	0,91	1,87	0,06224	1,61	3,05	0,00488	NM_012151	F8A1
8171029	0,91	1,87	0,06224	1,61	3,05	0,00488	NM_012151	ACOX2
8088397	0,91	1,87	0,14485	0,39	1,31	0,07137	NM_003500	NUBP1
7993185	0,91	1,87	0,18438	3,24	9,46	0,00130	NM_002484	UBA5
8082750	0,91	1,87	0,04558	1,79	3,46	0,00004	NM_024818	GNA11
8024557	0,91	1,87	0,24154	2,96	7,81	0,00087	NM_002067	ZNF780B
8036813	0,91	1,87	0,00441	2,00	3,99	0,00917	NM_001005851	ZMIZ1
7928558	0,91	1,87	0,24838	2,25	4,77	0,00289	NM_020338	RALB
8044919	0,90	1,87	0,09112	1,42	2,67	0,01214	NM_002881	GTF2H2
8105918	0,90	1,87	0,00104	1,63	3,09	0,00515	NM_001515	MCCC2
8106068	0,90	1,87	0,04677	3,62	12,29	0,00016	NM_022132	MCCC2
8177601	0,90	1,87	0,04677	3,62	12,29	0,00016	NM_022132	ZKSCAN3
8117685	0,90	1,87	0,02282	0,13	1,09	0,52773	NM_024493	C10orf78
7930299	0,90	1,87	0,16031	0,62	1,54	0,34103	NM_145247	PMM2
7993148	0,90	1,87	0,04236	1,19	2,27	0,04193	NM_000303	NKIRAS1
8085788	0,90	1,87	0,27102	2,70	6,49	0,00418	NM_020345	PHC2
7914648	0,90	1,87	0,00295	0,09	1,06	0,67595	NM_198040	PIGV

7899244	0,90	1,87	0,01793	2,91	7,50	0,00042	NM_017837	INPP4B
8102950	0,90	1,87	0,04530	0,32	1,25	0,09479	NM_003866	TACO1
8009176	0,90	1,87	0,05677	1,31	2,47	0,02389	NM_016360	GATAD2A
8027169	0,90	1,87	0,04321	1,38	2,60	0,02099	NM_017660	MCM7
8141395	0,90	1,87	0,04146	3,04	8,25	0,00040	NM_005916	FAM160A1
8097801	0,90	1,86	0,24453	2,70	6,51	0,00009	NM_001109977	AKAP8L
8035023	0,90	1,86	0,16182	2,47	5,53	0,00024	NM_014371	C16orf72
7993159	0,90	1,86	0,35093	1,04	2,05	0,01610	NM_014117	SEMA6A
8113666	0,90	1,86	0,13031	-0,09	-1,06	0,62024	NM_020796	GLRB
8098006	0,90	1,86	0,05697	-0,15	-1,11	0,66529	NM_000824	BMP1
8145055	0,90	1,86	0,01232	1,14	2,21	0,08290	NM_006129	CEP76
8022380	0,90	1,86	0,18676	2,18	4,54	0,00383	NM_024899	KIF20B
7929078	0,90	1,86	0,06267	1,33	2,51	0,02758	NM_016195	TST
8075857	0,90	1,86	0,20413	2,36	5,12	0,00063	NM_003312	ZDHHC12
8164452	0,90	1,86	0,04674	3,54	11,59	0,00195	NM_032799	HYOU1
7952145	0,90	1,86	0,05099	2,69	6,47	0,00286	NM_006389	UBQLN4
7920912	0,90	1,86	0,18677	2,28	4,87	0,00530	NM_020131	NBPF15
7919412	0,90	1,86	0,56665	-0,32	-1,25	0,29485	NM_001170755	TMEM206
7924150	0,90	1,86	0,03610	2,63	6,19	0,00217	NM_018252	---
7969559	0,90	1,86	0,08343	1,27	2,41	0,00112	--- AGPAT1	
8125328	0,90	1,86	0,20319	2,06	4,18	0,00180	NM_006411	AGPAT1
8178762	0,90	1,86	0,20319	2,06	4,18	0,00180	NM_006411	AGPAT1
8179958	0,90	1,86	0,20319	2,06	4,18	0,00180	NM_006411	RNH1
7945420	0,89	1,86	0,10410	2,06	4,18	0,00084	NM_002939	CDC6
8007071	0,89	1,86	0,08719	2,95	7,72	0,00088	NM_001254	SLFN12L
8014257	0,89	1,86	0,39104	-0,45	-1,37	0,48816	NM_001145027	XPA
8162696	0,89	1,86	0,03361	0,62	1,54	0,19445	NM_000380	C9orf80
8163428	0,89	1,86	0,20143	1,69	3,23	0,00044	NM_021218	ARMCX1
8168868	0,89	1,86	0,00939	-0,01	-1,01	0,93794	NM_016608	TUBG2
8007312	0,89	1,86	0,22257	2,70	6,52	0,00367	NM_016437	ZNF484
8162449	0,89	1,86	0,01325	1,21	2,31	0,00347	NM_031486	DEGS1
7924553	0,89	1,86	0,15998	1,69	3,24	0,04528	ENST00000415210	TBC1D4
7972021	0,89	1,86	0,13918	1,30	2,46	0,02222	NM_014832	NBPF10
7904562	0,89	1,86	0,65733	-0,91	-1,88	0,00628	NM_001039703	C5orf4
8115397	0,89	1,86	0,11267	-0,26	-1,20	0,17625	NM_032385	STX6
7922669	0,89	1,85	0,05203	2,05	4,13	0,00376	NM_005819	KRT33A
8015257	0,89	1,85	0,09435	-1,38	-2,60	0,02732	NM_004138	TCTA
8079746	0,89	1,85	0,00238	1,35	2,55	0,03090	NM_022171	WDR76
7983306	0,89	1,85	0,11382	2,07	4,19	0,03780	NM_024908	---
8098344	0,89	1,85	0,17109	-0,50	-1,42	0,03183	--- FAM125B	
8157905	0,89	1,85	0,13742	2,00	3,99	0,00578	NM_033446	PDZD8
7936559	0,89	1,85	0,03577	2,08	4,23	0,00258	NM_173791	PTPRG
8080810	0,89	1,85	0,15420	1,38	2,61	0,00028	NM_002841	SNX33
7985016	0,89	1,85	0,04408	1,65	3,14	0,00624	NM_153271	PFKM
7955078	0,89	1,85	0,05386	1,89	3,70	0,00272	NM_001166686	CDK15
8047467	0,89	1,85	0,17649	-0,86	-1,81	0,00052	NM_139158	RPL13AP5
7929593	0,89	1,85	0,31458	2,00	4,01	0,01089	NR_026712	TRAP1
7999025	0,89	1,85	0,03839	2,84	7,17	0,00097	NM_016292	MTIF3
7970704	0,89	1,85	0,01387	0,56	1,47	0,18393	NM_001166263	CERK
8076792	0,89	1,85	0,08453	2,69	6,47	0,00055	NM_022766	C9orf23
8160835	0,89	1,85	0,00865	2,31	4,98	0,00020	NM_148179	CLIC2
8176234	0,89	1,85	0,04598	-0,23	-1,17	0,46041	NM_001289	UCK2
7906995	0,89	1,85	0,05372	1,48	2,79	0,00015	NM_012474	C17orf95
8010086	0,89	1,85	0,16915	3,11	8,62	0,00219	ENST00000341249	CCL4
8006602	0,89	1,85	0,08692	-0,99	-1,98	0,20419	NM_002984	STIL
7915926	0,89	1,85	0,05194	2,97	7,86	0,00105	NM_001048166	SARNP
7963923	0,88	1,85	0,00641	3,03	8,15	0,00037	NR_026723	FGL2
8140463	0,88	1,84	0,47838	-1,15	-2,22	0,07124	NM_006682	ZNF700
8025973	0,88	1,84	0,03873	0,59	1,50	0,33473	NM_144566	RPL23P8
8131705	0,88	1,84	0,01959	0,12	1,09	0,85980	NR_026673	AGAP1
8049448	0,88	1,84	0,03312	1,83	3,56	0,02994	NM_001037131	BAT3
8124967	0,88	1,84	0,01693	2,69	6,46	0,00346	NM_004639	MLF1IP
8103932	0,88	1,84	0,09001	1,30	2,46	0,03722	NM_024629	RAB34
8013753	0,88	1,84	0,09788	2,65	6,26	0,00452	NM_031934	TRIM27
8124610	0,88	1,84	0,11835	2,15	4,44	0,01243	NM_006510	TRIM27
8179575	0,88	1,84	0,11835	2,15	4,44	0,01243	NM_006510	RANBP10
8002087	0,88	1,84	0,03746	1,63	3,09	0,00236	NM_020850	IMP4
8045171	0,88	1,84	0,29262	1,83	3,54	0,02890	NM_033416	ANP32E
7919715	0,88	1,84	0,03497	2,05	4,14	0,00923	NM_030920	ZSWIM3
8063071	0,88	1,84	0,11312	0,90	1,87	0,01293	NM_080752	PDLIM7
8116070	0,88	1,84	0,03635	1,38	2,61	0,05967	NM_005451	N4BP2L1
7970892	0,88	1,84	0,04869	0,34	1,27	0,21332	NM_052818	STK40
7914904	0,88	1,84	0,10680	2,28	4,84	0,00325	NM_032017	UBE2B

8108066	0,88	1,84	0,09741	0,76	1,69	0,01865	NM_003337	SNORD76
7922414	0,88	1,84	0,21608	0,42	1,34	0,75001	NR_003942	UBTF
8015969	0,88	1,84	0,15675	1,33	2,52	0,00194	NM_014233	CNTNAP1
8007323	0,88	1,84	0,03633	1,45	2,73	0,01215	NM_003632	TP53
8012257	0,88	1,84	0,61001	4,12	17,41	0,00016	NM_000546	TRUB1
7930703	0,88	1,84	0,11883	2,27	4,81	0,00059	NM_139169	NBEAL1
8047596	0,88	1,84	0,12037	1,91	3,75	0,00047	NM_001114132	C16orf52
7993889	0,88	1,84	0,33275	3,10	8,58	0,00173	NM_001164579	C7orf11
8165648	0,88	1,84	0,50158	-2,22	-4,67	0,00104	AK290098 ANAPC13	
8090866	0,88	1,84	0,03683	2,98	7,89	0,00046	NR_024400	EFCAB7
7901931	0,88	1,84	0,03434	1,11	2,16	0,07335	NM_032437	PDCD5
8027473	0,88	1,84	0,05493	1,05	2,07	0,14664	NM_004708	C4orf49
8102831	0,88	1,84	0,12332	-0,32	-1,25	0,14002	NM_032623	06. Sep
8174692	0,88	1,83	0,01836	2,97	7,86	0,00053	NM_145799	MFSD10
8099037	0,88	1,83	0,17334	1,86	3,64	0,04883	NM_001146069	---
7986246	0,87	1,83	0,14960	3,09	8,50	0,02781	--- ZCCHC10	
8114113	0,87	1,83	0,07968	-0,27	-1,21	0,30489	NM_017665	PHF20
8062213	0,87	1,83	0,06056	2,28	4,84	0,00116	NM_016436	ANKRD44
8057990	0,87	1,83	0,17644	1,70	3,26	0,01206	NM_153697	MT1IP
7995834	0,87	1,83	0,00758	-0,64	-1,55	0,06267	NR_003669	COPS7A
7953395	0,87	1,83	0,02072	1,85	3,60	0,00172	NM_001164094	KIAA0495
7911854	0,87	1,83	0,13454	1,29	2,45	0,01307	NM_207306	NACA2
8017259	0,87	1,83	0,04940	-0,22	-1,17	0,50599	NM_199290	RAB6A
7950284	0,87	1,83	0,12907	3,51	11,37	0,00194	NM_002869	---
8165686	0,87	1,83	0,29926	-1,52	-2,88	0,00049	--- YPEL3	
8000791	0,87	1,83	0,15001	0,80	1,74	0,23515	NM_031477	WDR5
8159111	0,87	1,83	0,02156	2,37	5,17	0,00167	NM_017588	NMT2
7932227	0,87	1,83	0,09437	1,82	3,53	0,00496	NM_004808	HCCS
8165995	0,87	1,83	0,16931	2,65	6,27	0,00503	NM_005333	NME5
8114354	0,87	1,83	0,14034	-1,30	-2,46	0,00588	NM_003551	GATA6
8020455	0,87	1,83	0,12059	-0,98	-1,97	0,12297	NM_005257	PREP
8128572	0,87	1,83	0,13926	2,38	5,22	0,00047	NM_002726	VN1R1
8039642	0,87	1,83	0,00660	0,04	1,03	0,82832	NM_020633	RTN3
7940781	0,87	1,83	0,05043	0,51	1,43	0,41077	NM_201428	CPEB4
8110055	0,87	1,83	0,11687	0,47	1,38	0,11333	NM_030627	ROMO1
8062206	0,87	1,82	0,07680	1,12	2,18	0,00732	NM_080748	SDC4
8066513	0,87	1,82	0,17546	1,80	3,49	0,01623	NM_002999	IQCK
7993713	0,87	1,82	0,05719	1,09	2,13	0,00009	NM_153208	CARS
7945803	0,87	1,82	0,09162	2,24	4,72	0,00009	NM_001014438	MTHFD2L
8095705	0,87	1,82	0,04688	1,73	3,31	0,02823	NM_001144978	FAM53C
8108321	0,86	1,82	0,08533	1,55	2,94	0,00100	NM_001135647	EEF1E1
8123819	0,86	1,82	0,14777	1,58	2,99	0,04971	NM_004280	NPIPL3
8022320	0,86	1,82	0,45469	0,70	1,63	0,04213	NM_130464	HNRNPA3
7971920	0,86	1,82	0,24747	1,22	2,34	0,04242	NM_194247	ZNF320
8039006	0,86	1,82	0,03779	1,13	2,18	0,05262	NM_207333	SGSH
8019061	0,86	1,82	0,01634	1,24	2,36	0,04825	NM_000199	AIP
7941927	0,86	1,82	0,11803	1,11	2,16	0,00208	NM_003977	BCL2A1
7990818	0,86	1,82	0,16955	0,13	1,09	0,69823	NM_001114735	LPAR3
7917276	0,86	1,82	0,28695	1,10	2,14	0,05270	NM_012152	COX11
8016825	0,86	1,82	0,00129	2,25	4,75	0,00100	NM_004375	RTKN2
7933855	0,86	1,82	0,21968	-0,33	-1,26	0,19477	NM_145307	SCML2
8171561	0,86	1,82	0,08403	2,80	6,95	0,00014	NM_006089	NAIP
8112521	0,86	1,82	0,06695	1,22	2,33	0,02562	NM_004536	NAIP
8177527	0,86	1,82	0,06695	1,22	2,33	0,02562	NM_004536	ZNF14
8035765	0,86	1,82	0,10168	1,18	2,26	0,04433	NM_021030	C1orf55
7924686	0,86	1,82	0,24131	2,40	5,26	0,00003	NM_152608	---
8063313	0,86	1,82	0,09990	-0,72	-1,65	0,19431	--- OLFML1	
7938225	0,86	1,81	0,15908	-1,35	-2,54	0,01048	NM_198474	THEM4
7920114	0,86	1,81	0,15064	3,27	9,68	0,00076	NM_053055	TCEB1
8151413	0,86	1,81	0,03654	1,70	3,24	0,02504	NM_005648	CPNE2
7995976	0,86	1,81	0,05992	2,61	6,12	0,00007	NM_152727	BIN3
8149707	0,86	1,81	0,05210	0,59	1,51	0,00644	NM_018688	E2F3
8117128	0,86	1,81	0,06035	0,80	1,74	0,00851	NM_001949	PBX2
8125360	0,86	1,81	0,00087	1,51	2,86	0,00966	NM_002586	AIG1
8122396	0,86	1,81	0,22950	1,37	2,59	0,01163	NM_016108	OSBPL3
8138613	0,86	1,81	0,03281	1,18	2,26	0,02938	NM_015550	RNF24
8064766	0,86	1,81	0,10258	0,11	1,08	0,41814	NM_001134338	NUFIP1
7971361	0,86	1,81	0,21262	1,93	3,81	0,02691	NM_012345	SLC19A3
8059538	0,86	1,81	0,20077	0,63	1,55	0,01409	NM_025243	USP47
7938448	0,86	1,81	0,08065	1,79	3,47	0,00897	NM_017944	PLCG1
8062623	0,86	1,81	0,02867	1,20	2,30	0,00511	NM_002660	AMOTL1
7943263	0,86	1,81	0,03275	0,10	1,07	0,75594	NM_130847	CXXC5
8108447	0,86	1,81	0,12663	0,37	1,29	0,08881	NM_016463	KLHL18

8079517	0,85	1,81	0,02543	1,36	2,57	0,00044	NM_025010	RPUSD4
7952549	0,85	1,81	0,01103	1,27	2,41	0,00152	NM_032795	CREB3
8155096	0,85	1,81	0,20590	1,54	2,91	0,03428	NM_006368	GLRX3
7931393	0,85	1,81	0,12018	2,19	4,56	0,00083	NM_006541	KIAA0355
8027604	0,85	1,81	0,13501	2,08	4,24	0,00072	NM_014686	PELI1
8052654	0,85	1,81	0,21677	3,47	11,10	0,00028	NM_020651	TPI1
7902435	0,85	1,81	0,26304	2,37	5,16	0,02604	NM_000365	ZNF98
8035831	0,85	1,81	0,00637	0,18	1,13	0,35612	NM_001098626	DNAJC24
7939093	0,85	1,81	0,01975	0,66	1,58	0,28193	NM_181706	CCDC52
8089627	0,85	1,80	0,05633	1,85	3,59	0,01419	NM_144718	C22orf9
8076690	0,85	1,80	0,20384	1,79	3,46	0,03365	NM_001009880	NACC1
8026155	0,85	1,80	0,05166	1,42	2,67	0,01511	NM_052876	ATG4C
7901895	0,85	1,80	0,14575	1,85	3,61	0,00246	NM_032852	PKP4
8045860	0,85	1,80	0,01224	2,84	7,15	0,00003	NM_003628	KLHL8
8101624	0,85	1,80	0,03694	1,10	2,15	0,02072	NM_020803	ZNF678
7910190	0,85	1,80	0,04208	1,80	3,49	0,00769	NR_033184	USP21
7906671	0,85	1,80	0,04561	3,54	11,66	0,00039	NM_001014443	SNTB1
8152606	0,85	1,80	0,05011	0,67	1,59	0,22796	NM_021021	LRRFIP1
8049512	0,85	1,80	0,18953	1,98	3,94	0,01123	NM_001137552	C11orf49
7939723	0,85	1,80	0,03757	0,91	1,88	0,18534	NM_001003676	USP32
8013262	0,85	1,80	0,05016	1,98	3,94	0,00047	ENST00000300896	CWC27
8105612	0,85	1,80	0,04763	1,32	2,50	0,00012	NM_005869	C1D
7932964	0,85	1,80	0,24686	2,48	5,58	0,00035	NM_006333	PPA2
8102141	0,85	1,80	0,18734	1,97	3,92	0,02117	NM_176869	FXN
8155699	0,85	1,80	0,34267	2,63	6,19	0,00172	NM_000144	SEPSECS
8099696	0,85	1,80	0,13985	2,55	5,84	0,00002	NM_016955	SNORA70 // SNORA70
8025498	0,85	1,80	0,13055	5,06	33,41	0,00003	NR_000011 // NR_000011	C3orf63
8088219	0,85	1,80	0,09236	1,39	2,62	0,00841	NM_001112736	RAD54B
8151824	0,85	1,80	0,05234	2,28	4,87	0,00478	NM_012415	TMEM19
7957167	0,84	1,80	0,14182	3,48	11,17	0,00024	NM_018279	THBS3
7920664	0,84	1,79	0,07843	1,58	2,98	0,01687	NM_007112	C12orf41
7962811	0,84	1,79	0,00193	1,83	3,54	0,00843	NM_017822	LLPH
7956876	0,84	1,79	0,10315	-0,94	-1,92	0,04207	NM_032338	PTPN9
7990511	0,84	1,79	0,22618	3,65	12,54	0,00003	NM_002833	FAM183A
7900635	0,84	1,79	0,00282	-0,52	-1,44	0,02702	NM_001101376	---
8016806	0,84	1,79	0,04447	-0,13	-1,09	0,80842	---	IMMT
8053533	0,84	1,79	0,19132	3,02	8,14	0,00039	NM_006839	XG
8165808	0,84	1,79	0,25427	-1,38	-2,60	0,04741	NM_001141919	RP2
8167006	0,84	1,79	0,05824	0,35	1,28	0,05747	NM_006915	LRRC37B
8006336	0,84	1,79	0,06334	3,24	9,44	0,00333	NM_052888	WDR12
8058373	0,84	1,79	0,26778	2,33	5,04	0,00061	NM_018256	FAM92A1
8147307	0,84	1,79	0,35103	1,08	2,11	0,24576	BC014598	EIF2B4
8051097	0,84	1,79	0,22389	1,90	3,72	0,00621	NM_001034116	DCP2
8107356	0,84	1,79	0,22099	1,50	2,83	0,00036	NM_152624	CPSF6
7957008	0,84	1,79	0,02709	1,95	3,86	0,00035	NM_007007	SLC36A4
7950990	0,84	1,79	0,29789	2,26	4,78	0,00541	NM_152313	RACGAP1
7963157	0,84	1,79	0,13185	3,69	12,93	0,00198	NM_013277	---
7990564	0,84	1,79	0,46547	-0,38	-1,30	0,18714	---	LOC441453 // LOC441453 // LOC441453 // LOC441453
7938683	0,84	1,79	0,56176	0,32	1,24	0,61691	BC008657 // BC008657 // BC008657 // BC008657	C18orf55
8021716	0,84	1,79	0,16373	0,84	1,80	0,07422	NM_014177	C8orf45
8146738	0,84	1,79	0,02741	2,97	7,85	0,00124	NM_001136161	GGA3
8018324	0,84	1,79	0,02157	2,92	7,59	0,00014	NR_033345	ZCCHC14
8003263	0,84	1,79	0,22299	2,82	7,04	0,00068	NM_015144	THAP6
8095760	0,84	1,79	0,25840	1,96	3,90	0,00400	NM_144721	ENOX1
7971311	0,84	1,79	0,04177	0,13	1,09	0,74621	NM_017993	HMGN2
7982204	0,84	1,79	0,58324	0,50	1,41	0,32001	NM_005517	BGN
8170648	0,84	1,79	0,22816	-1,34	-2,53	0,01812	NM_001711	SLC1A4
8042310	0,84	1,79	0,05294	2,35	5,09	0,00293	NM_003038	PAPD7
8104378	0,84	1,78	0,09865	3,63	12,42	0,00024	NM_006999	ZNF280B
8074845	0,84	1,78	0,07837	2,58	5,98	0,00070	NM_080764	SNORD14C
7952339	0,84	1,78	0,23960	0,64	1,56	0,16415	NR_001453	RAD1
8111457	0,83	1,78	0,09106	0,90	1,86	0,02697	NM_002853	BAG4
8145914	0,83	1,78	0,19313	0,80	1,74	0,15792	NM_004874	ZNF616
8038954	0,83	1,78	0,07276	0,46	1,38	0,01171	NM_178523	CHST11
7958202	0,83	1,78	0,03003	1,84	3,58	0,00022	NM_018413	FABP4
8151532	0,83	1,78	0,01067	-1,05	-2,07	0,03697	NM_001442	SLC38A5
8172425	0,83	1,78	0,05673	-1,14	-2,20	0,00300	NM_033518	PRKAB1
7959123	0,83	1,78	0,01271	1,32	2,50	0,01574	NM_006253	DDX41
8116096	0,83	1,78	0,14896	3,00	7,98	0,00078	NM_016222	SEPT7L
7933115	0,83	1,78	0,00515	0,65	1,57	0,05523	NR_027269	CEP70

8090988	0,83	1,78	0,11636	2,64	6,24	0,00351	NM_024491	XRCC6BP1
7956648	0,83	1,78	0,06986	1,51	2,84	0,00599	NM_033276	LIG3
8006479	0,83	1,78	0,12515	1,16	2,23	0,01068	NM_013975	SFRS8
7959927	0,83	1,78	0,21027	1,42	2,68	0,00163	NM_004592	C3orf59
8092765	0,83	1,78	0,18108	2,39	5,26	0,00028	NM_178496	---
7980096	0,83	1,78	0,25857	2,08	4,22	0,02912	---	LSM1
8150266	0,83	1,78	0,24416	0,70	1,63	0,06000	NM_014462	NUDT13
7934299	0,83	1,78	0,15760	1,03	2,05	0,04981	NM_015901	ARMC10
8089034	0,83	1,78	0,14457	2,18	4,54	0,00009	NM_001161009	AARSD1
8015741	0,83	1,77	0,00519	1,66	3,15	0,00050	NM_001136042	RNF219
7972180	0,83	1,77	0,03056	0,44	1,36	0,25352	NM_024546	DDX60
8103563	0,83	1,77	0,19037	0,23	1,17	0,41175	NM_017631	UBXN6
8032815	0,83	1,77	0,03853	1,06	2,08	0,01199	NM_025241	RUFY2
7933999	0,82	1,77	0,00231	1,26	2,39	0,01951	NM_017987	SYNGR1
8073104	0,82	1,77	0,05888	1,27	2,40	0,10662	NM_004711	---
8040463	0,82	1,77	0,00272	0,02	1,01	0,93019	---	CSTF2T
7933659	0,82	1,77	0,12299	0,41	1,33	0,12474	NM_015235	SGTB
8112409	0,82	1,77	0,21759	1,80	3,47	0,00288	NM_019072	ZNF555
8024518	0,82	1,77	0,06557	1,74	3,35	0,01429	NM_152791	DHX33
8011861	0,82	1,77	0,27266	3,81	14,00	0,00020	NM_020162	SPTLC3
8060997	0,82	1,77	0,16518	-0,79	-1,73	0,00521	NM_018327	LARP1B
8097388	0,82	1,77	0,00049	1,16	2,24	0,10714	NM_018078	ZNF410
7975645	0,82	1,77	0,07185	2,62	6,15	0,00058	NM_021188	C18orf54
8021286	0,82	1,77	0,08563	0,88	1,85	0,06195	NM_173529	PHF12
8013812	0,82	1,77	0,07982	2,89	7,43	0,00098	NM_001033561	PDE4D
8112220	0,82	1,76	0,00747	-0,26	-1,20	0,05501	NM_001165899	UGT2B7
8100758	0,82	1,76	0,61323	-3,31	-9,93	0,00809	NM_001074	NEK3
7971780	0,82	1,76	0,04117	2,52	5,75	0,00104	NM_002498	TIMM23
7927548	0,82	1,76	0,24536	0,00	-1,00	0,99948	NM_006327	NCOA6
8065776	0,82	1,76	0,36027	3,13	8,76	0,00110	NM_014071	TTC21B
8056426	0,82	1,76	0,22494	1,98	3,96	0,00131	NM_024753	PTS
7943882	0,82	1,76	0,12336	1,94	3,84	0,00606	NM_000317	INTS10
8144894	0,82	1,76	0,10843	2,66	6,31	0,00012	NM_018142	NCKAP5
8055323	0,82	1,76	0,09923	-1,35	-2,54	0,00419	NM_207363	---
8165692	0,82	1,76	0,59763	-3,22	-9,33	0,00477	---	ZNF543
8031720	0,82	1,76	0,32234	1,63	3,11	0,02648	NM_213598	GAN
7997414	0,82	1,76	0,10889	2,87	7,30	0,00349	NM_022041	SLC7A6OS
8002237	0,82	1,76	0,08616	2,85	7,19	0,00036	NM_032178	CRY1
7966052	0,81	1,76	0,05033	0,54	1,45	0,00139	NM_004075	MSRB2
7926661	0,81	1,76	0,07592	0,92	1,90	0,03078	NM_012228	CCBE1
8023575	0,81	1,76	0,01650	0,72	1,65	0,01460	NM_133459	BACE2
8068671	0,81	1,76	0,07924	2,44	5,42	0,00119	NM_012105	ZCRB1
7962367	0,81	1,76	0,05823	1,24	2,36	0,03961	NM_033114	CLCN5
8167603	0,81	1,76	0,00083	1,00	2,00	0,00438	NM_001127899	DUS2L
7996725	0,81	1,76	0,15547	4,12	17,43	0,00011	NM_017803	CPSF4
8134599	0,81	1,76	0,06668	3,68	12,84	0,00149	NM_006693	FUCA1
7913694	0,81	1,76	0,16712	3,16	8,91	0,00033	NM_000147	USP36
8018937	0,81	1,76	0,04320	1,24	2,36	0,01645	NM_025090	---
8095341	0,81	1,76	0,28387	-0,66	-1,58	0,02584	---	---
7962479	0,81	1,76	0,22559	-2,70	-6,50	0,18793	---	C20orf72
8061129	0,81	1,76	0,08590	2,32	4,98	0,01395	BC016869 AEBP1	
8132557	0,81	1,76	0,01532	-1,14	-2,20	0,00591	NM_001129	ZNF720
7995252	0,81	1,76	0,39580	4,25	19,09	0,00090	NM_001130913	---
8146955	0,81	1,76	0,57031	-2,72	-6,59	0,00118	---	ZNF44
8034349	0,81	1,75	0,17011	-0,12	-1,09	0,71917	NM_001164276	NUAK2
7923753	0,81	1,75	0,10187	3,47	11,06	0,00057	NM_030952	MIR21
8008885	0,81	1,75	0,15202	1,12	2,18	0,05062	NR_029493	LRRC32
7950555	0,81	1,75	0,07708	-2,41	-5,33	0,00015	NM_001128922	PSG3
8037231	0,81	1,75	0,14995	-0,96	-1,94	0,07981	NM_021016	MOBKL3
8047228	0,81	1,75	0,25885	2,46	5,50	0,00092	NM_199482	ETS1
7952601	0,81	1,75	0,06734	1,42	2,68	0,03555	NM_001143820	SLC35F2
7951485	0,81	1,75	0,01348	3,61	12,24	0,00259	NM_017515	NPIP
7993580	0,81	1,75	0,49024	0,02	1,02	0,91972	NM_006985	PTHLH
7962000	0,81	1,75	0,04080	-0,37	-1,30	0,03354	NM_198965	SNAP47
7910200	0,80	1,75	0,04613	0,79	1,73	0,03421	NM_053052	EPCAM
8098439	0,80	1,75	0,40833	3,93	15,26	0,08258	NM_002354	ZBTB34
8157945	0,80	1,75	0,28577	1,05	2,07	0,06594	NM_001099270	GYS1
8038261	0,80	1,75	0,15647	2,02	4,04	0,00053	NM_002103	ZNF571
8036406	0,80	1,75	0,00129	-0,08	-1,06	0,83565	NM_016536	WBP1
8053599	0,80	1,74	0,06048	2,82	7,07	0,00025	NM_012477	ZNF251
8153890	0,80	1,74	0,03061	2,05	4,14	0,00300	NM_138367	JAM2
8068024	0,80	1,74	0,08490	-0,97	-1,96	0,03069	NM_021219	CYTSA
8071881	0,80	1,74	0,02975	1,78	3,44	0,00696	NM_015330	BCKDHA

8029065	0,80	1,74	0,08821	2,33	5,01	0,02009	NM_000709	PLEKHM1
8016239	0,80	1,74	0,13063	0,15	1,11	0,81976	NR_027774	C12orf57
7953564	0,80	1,74	0,21194	3,37	10,34	0,00002	NM_138425	ZNF329
8039740	0,80	1,74	0,19002	0,62	1,53	0,07865	NM_024620	CXADR
8067955	0,80	1,74	0,25397	3,04	8,25	0,00625	NM_001338	ZNF559
8025478	0,80	1,74	0,05517	2,29	4,91	0,00164	NM_032497	FBXO42
7912750	0,80	1,74	0,16530	3,02	8,11	0,00199	NM_018994	MRPS36
8105852	0,80	1,74	0,10143	0,43	1,35	0,09064	NM_033281	KATNA1
8130102	0,80	1,74	0,32407	2,21	4,62	0,02407	NM_007044	---
7907970	0,80	1,74	0,12535	1,55	2,93	0,01635	--- VHL	
8077781	0,80	1,74	0,18565	1,95	3,87	0,00023	NM_000551	---
7898276	0,80	1,74	0,02978	1,44	2,71	0,01506	--- INPP1	
8047069	0,80	1,74	0,17694	1,23	2,35	0,01384	NM_001128928	GATSL1
8133540	0,80	1,74	0,29544	2,70	6,49	0,00184	NM_001145063	GATSL1
8140249	0,80	1,74	0,29544	2,70	6,49	0,00184	NM_001145063	LBR
7924603	0,80	1,74	0,11747	2,86	7,24	0,00130	NM_002296	RPL8
8153903	0,80	1,74	0,19970	3,05	8,30	0,00480	NM_000973	SRD5A1
8104369	0,80	1,74	0,06978	1,58	2,99	0,00268	NM_001047	ALG1
7993071	0,80	1,74	0,03924	1,49	2,82	0,06823	NM_019109	ARPC5
7922793	0,80	1,74	0,10454	1,76	3,38	0,00722	NM_005717	XAB2
8033392	0,80	1,74	0,23208	1,73	3,31	0,00083	NM_020196	TBX18
8127932	0,79	1,73	0,23534	-1,32	-2,49	0,02069	NM_001080508	TIAL1
7936683	0,79	1,73	0,07273	1,16	2,24	0,06924	NM_001033925	KCNMA1
7934570	0,79	1,73	0,07094	0,61	1,53	0,00124	NM_001014797	GLUD1
7928907	0,79	1,73	0,29515	-1,42	-2,67	0,21441	NM_005271	LTV1
8122440	0,79	1,73	0,23712	1,57	2,97	0,00675	NM_032860	SLC25A17
8076260	0,79	1,73	0,22919	3,02	8,13	0,00232	NM_006358	SET
7917674	0,79	1,73	0,53702	5,12	34,79	0,00008	NM_001122821	CDCA2
8145418	0,79	1,73	0,06451	2,13	4,37	0,00771	NM_152562	RBPJ
8155458	0,79	1,73	0,18872	0,81	1,75	0,04430	D14041 TSPAN2	
7918857	0,79	1,73	0,02079	0,50	1,42	0,07405	NM_005725	ELMOD2
8097529	0,79	1,73	0,10475	1,59	3,01	0,01212	NM_153702	ARMCX3
8168875	0,79	1,73	0,06694	0,41	1,33	0,22650	NM_016607	KCNIP3
8043552	0,79	1,73	0,28919	0,84	1,80	0,06197	NM_013434	RDBP
8125204	0,79	1,73	0,04205	2,16	4,48	0,00392	NM_002904	RDBP
8178686	0,79	1,73	0,04205	2,16	4,48	0,00392	NM_002904	RDBP
8179913	0,79	1,73	0,04205	2,16	4,48	0,00392	NM_002904	OSR2
8147573	0,79	1,73	0,25666	0,14	1,10	0,67407	NM_001142462	HMGN2
8000409	0,79	1,73	0,56793	0,40	1,32	0,44822	NM_005517	ARHGAP20
7951565	0,79	1,73	0,12159	-1,02	-2,03	0,00130	NM_020809	ZNF730
8027348	0,79	1,73	0,43217	-1,27	-2,41	0,05772	ENST00000327867	DPT
7922130	0,79	1,73	0,02152	-1,78	-3,44	0,03754	NM_001937	DOCK4
8142345	0,79	1,73	0,01259	2,01	4,02	0,00026	NM_014705	C14orf132
7976556	0,79	1,73	0,16097	-0,61	-1,53	0,01261	NR_023938	SAP30L
8109428	0,79	1,73	0,11422	1,97	3,92	0,00004	NR_024084	SNRPD3
8071920	0,79	1,73	0,09064	0,77	1,71	0,00568	NM_004175	TNS1
8058869	0,79	1,73	0,20067	-0,91	-1,88	0,00606	NM_022648	---
8162260	0,79	1,73	0,32849	0,39	1,31	0,51971	--- SLC41A1	
7923824	0,79	1,73	0,04467	2,18	4,54	0,00574	NM_173854	NDNL2
7986969	0,79	1,72	0,03224	-0,50	-1,41	0,10180	NM_138704	HLA-L
8117861	0,79	1,72	0,15608	1,32	2,50	0,00932	NR_027822	HLA-L
8179080	0,79	1,72	0,15608	1,32	2,50	0,00932	NR_027822	RBM6
8079842	0,79	1,72	0,04020	1,81	3,52	0,01588	NM_005777	ACACA
8014487	0,79	1,72	0,04302	2,30	4,91	0,00132	NM_198839	HIF1AN
7929831	0,79	1,72	0,10292	1,63	3,09	0,00032	NM_017902	ZNF391
8117572	0,79	1,72	0,08504	1,05	2,08	0,04006	NM_001076781	AP1AR
8096905	0,78	1,72	0,05412	1,86	3,64	0,00198	NM_018569	ZFP3
8004081	0,78	1,72	0,08444	0,51	1,43	0,16591	NM_153018	RAPGEF6
8113881	0,78	1,72	0,09298	1,26	2,40	0,00517	NM_001164386	LYPLA2P1
8125748	0,78	1,72	0,13590	1,29	2,44	0,10825	NR_001444	SYAP1
8166219	0,78	1,72	0,04160	1,22	2,33	0,00016	NM_032796	SIRPA
8060418	0,78	1,72	0,06041	0,84	1,79	0,03091	NM_001040022	NDUFAB1
8000323	0,78	1,72	0,13879	1,92	3,80	0,00015	NM_005003	FMO2
7907271	0,78	1,72	0,01452	-0,58	-1,49	0,00066	NM_001460	ZFP64
8067094	0,78	1,72	0,08614	2,75	6,73	0,00081	NM_199427	CHN1
8056890	0,78	1,72	0,04569	1,76	3,40	0,01460	NM_001822	POLR2F
8072979	0,78	1,72	0,13786	1,64	3,12	0,04617	NM_021974	ZNF287
8013026	0,78	1,72	0,20011	0,54	1,46	0,10560	NM_020653	MSH3
8106633	0,78	1,72	0,09515	0,56	1,47	0,06319	NM_002439	UQCRC1
8087100	0,78	1,71	0,19978	1,84	3,57	0,00177	NM_003365	SGK196
8146278	0,78	1,71	0,32737	3,69	12,93	0,00001	NM_032237	WBP1
8043377	0,78	1,71	0,05962	2,78	6,85	0,00016	NM_012477	ZMYND19
8165622	0,78	1,71	0,23736	2,35	5,11	0,02779	NM_138462	ACVR1B

7955535	0,78	1,71	0,01570	1,19	2,28	0,00489	NM_004302	CPE
8098204	0,78	1,71	0,02619	0,92	1,89	0,00931	NM_001873	SP3
8056798	0,78	1,71	0,21535	2,62	6,13	0,00174	NM_003111	ADRBK1
7941797	0,78	1,71	0,07389	3,05	8,26	0,00240	NM_001619	RIMS1
8120613	0,77	1,71	0,02250	-1,33	-2,51	0,00467	NM_014989	BCL2L13
8071119	0,77	1,71	0,29132	2,82	7,07	0,00062	NM_015367	NRP2
8047738	0,77	1,71	0,19131	-1,10	-2,14	0,01413	NM_201266	RNF121
7942289	0,77	1,71	0,04365	0,85	1,81	0,04642	NM_018320	RAB9A
8166098	0,77	1,71	0,01902	1,44	2,71	0,01013	NM_004251	NUBPL
7973826	0,77	1,71	0,15666	1,94	3,84	0,01232	NM_025152	JAGN1
8077663	0,77	1,71	0,35508	3,20	9,21	0,00093	NM_032492	DOCK1
7931293	0,77	1,71	0,19771	2,58	5,96	0,00030	NM_001380	---
8165676	0,77	1,71	0,18707	-1,32	-2,49	0,00252	--- VRK2	
8042086	0,77	1,71	0,13229	2,07	4,21	0,00128	NM_006296	CPZ
8094101	0,77	1,71	0,09631	-1,04	-2,05	0,02241	NM_001014448	C17orf71
8008795	0,77	1,71	0,12457	2,12	4,35	0,00549	NM_018149	---
8123800	0,77	1,71	0,14791	0,74	1,67	0,07587	--- ---	
7932960	0,77	1,71	0,26335	0,13	1,09	0,78130	--- ZNF608	
8113761	0,77	1,71	0,28506	-0,63	-1,55	0,00271	NM_020747	ALS2CR8
8047577	0,77	1,71	0,00253	-0,31	-1,24	0,61599	NM_024744	MGC72080
8042574	0,77	1,71	0,48651	0,25	1,19	0,70015	NR_002822	BOC
8081686	0,77	1,71	0,07691	-0,10	-1,07	0,55291	NM_033254	SCN9A
8056491	0,77	1,71	0,00626	0,23	1,17	0,44450	NM_002977	MKS1
8016909	0,77	1,71	0,11650	1,68	3,21	0,00060	NM_017777	BRP44
7922095	0,77	1,70	0,03212	1,39	2,62	0,00045	NR_026550	FLJ44635
8168362	0,77	1,70	0,07043	-0,08	-1,06	0,77500	NM_207422	ARL17A
8007794	0,77	1,70	0,19580	1,32	2,50	0,05998	NM_001113738	PSMD12
8017810	0,77	1,70	0,05701	1,59	3,01	0,03223	NM_002816	CHRDL2
7950425	0,77	1,70	0,32730	-0,93	-1,90	0,08091	NM_015424	C1orf128
7898894	0,77	1,70	0,24984	1,82	3,53	0,00086	NM_020362	ZNF823
8034315	0,77	1,70	0,22745	1,18	2,27	0,04845	NM_001080493	SELENBP1
7919984	0,77	1,70	0,06141	-0,90	-1,86	0,09051	NM_003944	C16orf68
7993114	0,77	1,70	0,09212	0,96	1,94	0,00108	NM_024109	CINP
7981364	0,77	1,70	0,14840	1,29	2,44	0,00018	NM_032630	RPE
8048014	0,77	1,70	0,25227	0,80	1,74	0,02680	NM_199229	---
8161444	0,77	1,70	0,65529	-5,25	-38,05	0,00190	--- IL1A	
8054712	0,77	1,70	0,22414	-0,82	-1,77	0,02102	NM_000575	ITSN1
8068305	0,76	1,70	0,03395	2,00	3,99	0,00002	NM_003024	SIX4
7979510	0,76	1,70	0,09858	0,15	1,11	0,51913	NM_017420	---
7946565	0,76	1,70	0,07887	-0,72	-1,65	0,09870	--- SPRYD3	
7963577	0,76	1,70	0,12077	1,97	3,92	0,02662	NM_032840	TTF2
7904314	0,76	1,70	0,13975	1,95	3,86	0,00175	NM_003594	SMARCA2
8154059	0,76	1,70	0,06695	0,57	1,48	0,16371	NM_003070	ANP32A
7990020	0,76	1,70	0,03244	2,71	6,54	0,01402	NM_006305	FAM162B
8129120	0,76	1,70	0,04094	-0,57	-1,49	0,01083	NM_001085480	PEX2
8151436	0,76	1,70	0,21846	2,05	4,15	0,00151	NM_000318	ZNF592
7985605	0,76	1,70	0,40925	3,47	11,04	0,00004	NM_014630	---
7949894	0,76	1,69	0,00872	0,25	1,19	0,09536	--- ERMP1	
8159992	0,76	1,69	0,11864	2,58	5,98	0,00071	NM_024896	PPIL6
8128726	0,76	1,69	0,29437	2,12	4,36	0,01344	NM_173672	PPIH
7900576	0,76	1,69	0,15689	1,49	2,82	0,16955	NM_006347	WDR3
7904364	0,76	1,69	0,17070	2,32	4,98	0,00021	NM_006784	EFTUD2
8016099	0,76	1,69	0,22793	3,36	10,28	0,00026	NM_004247	NBPF15
7919271	0,76	1,69	0,55305	-0,07	-1,05	0,82564	NM_173638	TRAPPC6B
7978739	0,76	1,69	0,17139	2,78	6,86	0,00026	NM_001079537	EPAG
8173930	0,76	1,69	0,01198	-0,64	-1,56	0,01272	L22650 DNPEP	
8059222	0,76	1,69	0,26447	1,48	2,79	0,00080	NM_012100	MAGI2
8140504	0,75	1,69	0,21109	-0,87	-1,83	0,04483	NM_012301	MYO9B
8026638	0,75	1,69	0,01167	1,47	2,77	0,04784	NM_004145	BRD7
8083704	0,75	1,69	0,28470	1,32	2,50	0,09380	NM_001173984	---
7934531	0,75	1,69	0,17059	0,76	1,70	0,02652	--- MLF2	
7960689	0,75	1,69	0,26734	3,24	9,45	0,00858	NR_026581	TAPBP
8125713	0,75	1,69	0,01685	0,84	1,79	0,05557	NM_003190	EXOC7
8018620	0,75	1,69	0,02318	2,10	4,30	0,00833	NM_001145297	COG2
7910446	0,75	1,69	0,20064	2,17	4,50	0,00233	NM_007357	DENND2C
7918768	0,75	1,69	0,11056	0,54	1,45	0,01112	NM_198459	ZNF280D
7989159	0,75	1,68	0,12031	1,24	2,36	0,02043	NM_017661	SPOP
8016562	0,75	1,68	0,01916	1,28	2,42	0,00189	NM_001007230	SMG6
8011222	0,75	1,68	0,00210	1,04	2,05	0,01705	NM_017575	BTN3A2
8117435	0,75	1,68	0,39368	1,38	2,60	0,00283	NM_007047	KIF23
7984540	0,75	1,68	0,11629	2,55	5,85	0,00235	NM_138555	NAPG
8020149	0,75	1,68	0,15128	2,22	4,66	0,00024	NM_003826	RPS2
8172154	0,75	1,68	0,28135	0,98	1,98	0,01757	NM_002952	MYO1C

8011027	0,75	1,68	0,19819	3,25	9,52	0,00270	NM_001080779	---
8112916	0,75	1,68	0,07079	-0,74	-1,67	0,09488	---	---
8174970	0,75	1,68	0,07079	-0,74	-1,67	0,09488	---	CYFIP2
8109528	0,75	1,68	0,26094	1,66	3,16	0,01227	NM_001037332	ATG16L1
8049271	0,75	1,68	0,09685	2,37	5,16	0,00073	NM_030803	FAHD2B
8054045	0,75	1,68	0,17202	2,42	5,36	0,00047	NM_199336	ARMCX5
8168958	0,75	1,68	0,04436	1,93	3,80	0,00271	NM_001168479	B4GALT2
7900931	0,75	1,68	0,02588	1,48	2,80	0,03303	NM_003780	APOBEC3F
8073081	0,75	1,68	0,02009	1,48	2,78	0,00824	NM_145298	APOA1BP
7906185	0,75	1,68	0,29913	3,64	12,48	0,00001	NM_144772	ZNF449
8170015	0,75	1,68	0,30023	1,36	2,56	0,00435	NM_152695	B3GALNT1
8091780	0,75	1,68	0,03345	1,35	2,56	0,08554	NM_001038628	NBPF15
7919351	0,75	1,68	0,55949	-0,06	-1,04	0,83973	NM_173638	GPR1
8058450	0,75	1,68	0,11301	-0,44	-1,36	0,12216	NM_005279	PDE3B
7938629	0,75	1,68	0,19375	1,81	3,51	0,02517	NM_000922	TTC7B
7980720	0,74	1,68	0,26154	2,55	5,84	0,00204	NM_001010854	ZNF224
8029377	0,74	1,68	0,31749	1,35	2,56	0,00268	NM_013398	SLC33A1
8091637	0,74	1,68	0,26584	3,03	8,17	0,00206	NM_004733	ANKRD52
7964033	0,74	1,67	0,17911	2,61	6,10	0,00109	NM_173595	LOC100132288
8177120	0,74	1,67	0,07758	1,27	2,41	0,00548	NM_001033515	TMEM109
7940372	0,74	1,67	0,00557	2,87	7,31	0,04628	NM_024092	---
8058411	0,74	1,67	0,09608	-0,19	-1,14	0,68934	---	CPSF3L
7911486	0,74	1,67	0,17774	2,55	5,87	0,00083	NM_017871	DHCR7
7950067	0,74	1,67	0,07617	3,46	11,03	0,00071	NM_001360	STRBP
8164013	0,74	1,67	0,10193	4,03	16,38	0,00014	NM_001171137	PARL
8092409	0,74	1,67	0,07430	1,30	2,47	0,11078	NM_018622	KCTD5
7992685	0,74	1,67	0,07290	1,55	2,92	0,00392	NM_018992	RNF207
7897263	0,74	1,67	0,09441	0,15	1,11	0,70829	NM_207396	PDXDC2
7994559	0,74	1,67	0,31050	1,60	3,04	0,01577	NR_003610	TCIRG1
7941985	0,74	1,67	0,08933	2,14	4,40	0,01031	NM_006019	KDM4C
8154333	0,74	1,67	0,06858	1,54	2,91	0,00009	NM_015061	TEX2
8017582	0,74	1,67	0,06169	1,91	3,75	0,00192	NM_018469	POLR2A
8004431	0,74	1,67	0,05070	2,52	5,73	0,00177	NM_000937	NDN
7986757	0,74	1,67	0,24099	-1,13	-2,19	0,07758	NM_002487	FAM20C
8130993	0,74	1,67	0,03184	1,55	2,93	0,04660	NM_020223	APBB1
7946149	0,74	1,67	0,08601	0,32	1,25	0,39929	NM_001164	KIF3B
8061715	0,74	1,67	0,01329	0,94	1,91	0,10987	NM_004798	PHC1
7963869	0,74	1,67	0,15772	2,32	4,98	0,00004	NM_004426	IFT46
7952069	0,74	1,67	0,20402	1,56	2,95	0,00983	NM_020153	AP4B1
7918703	0,74	1,67	0,07348	1,00	1,99	0,09023	NM_006594	BRI3
8134454	0,74	1,67	0,18622	-0,15	-1,11	0,68811	NM_015379	SLC1A5
8037835	0,74	1,67	0,05219	2,26	4,78	0,03416	NM_005628	PAG1
8151512	0,74	1,67	0,19116	0,63	1,54	0,18793	NM_018440	SLC46A3
7970793	0,74	1,67	0,12336	2,76	6,79	0,00122	NM_181785	14. Sep
7998117	0,74	1,66	0,46510	0,93	1,91	0,60005	NM_207366	---
8053761	0,73	1,66	0,06239	0,67	1,59	0,00494	---	EBF3
7937039	0,73	1,66	0,22174	-1,08	-2,11	0,02196	NM_001005463	PMF1
7906133	0,73	1,66	0,05655	2,63	6,21	0,00219	NM_007221	ERCC5
7969935	0,73	1,66	0,03606	0,94	1,92	0,00412	NM_000123	TUBGCP3
7972840	0,73	1,66	0,12657	2,45	5,48	0,00023	NM_006322	TNXB
8178712	0,73	1,66	0,13067	0,41	1,32	0,29535	NM_032470	TNXB
8179935	0,73	1,66	0,13067	0,41	1,32	0,29535	NM_032470	MX1
8068713	0,73	1,66	0,10706	-0,26	-1,20	0,05958	NM_002462	CCDC66
8080621	0,73	1,66	0,12284	0,55	1,47	0,41453	NR_024460	ZNF706
8152111	0,73	1,66	0,04639	1,85	3,61	0,02989	NM_001042510	SLC16A14
8059642	0,73	1,66	0,12621	0,62	1,54	0,02517	NM_152527	RMRP
8161024	0,73	1,66	0,14597	-1,20	-2,29	0,04174	NR_003051	GLRX2
7923027	0,73	1,66	0,06871	0,43	1,35	0,16678	NM_197962	PRKACA
8034762	0,73	1,66	0,09650	1,98	3,95	0,00088	NM_002730	KCNQ5
8120654	0,73	1,66	0,07137	1,41	2,65	0,00068	NM_001160133	TPST2
8075106	0,73	1,66	0,00982	0,54	1,46	0,08125	NM_003595	ANAPC4
8094408	0,73	1,66	0,05498	2,87	7,31	0,00264	NM_013367	MPP7
7932765	0,73	1,66	0,00943	2,36	5,13	0,00018	NM_173496	ZNF227
8029413	0,73	1,66	0,10028	0,13	1,09	0,40113	NM_182490	NCAPG
8094278	0,73	1,66	0,01590	2,32	4,98	0,00067	NM_022346	CEP290
7965264	0,73	1,66	0,13878	0,57	1,49	0,09418	NM_025114	LYRM2
8128133	0,73	1,66	0,38491	0,22	1,16	0,35727	NM_020466	PCDHB7
8108708	0,73	1,66	0,05281	-0,42	-1,34	0,09298	NM_018940	SEMA4C
8054004	0,73	1,66	0,09426	2,59	6,04	0,00449	NM_017789	NDUFB3
8047372	0,73	1,66	0,21133	1,01	2,01	0,05336	NM_002491	CCDC125
8112439	0,73	1,66	0,06849	0,88	1,84	0,04225	NM_176816	CCDC125
8177628	0,73	1,66	0,24832	1,26	2,40	0,00386	NM_176816	UBE2I
7992205	0,73	1,66	0,02940	1,52	2,87	0,00695	NM_194259	ANUBL1

7933237	0,73	1,66	0,15242	1,46	2,75	0,03089	NM_174890	---
7924758	0,73	1,66	0,21767	0,91	1,89	0,28103	--- SKIL	
8083876	0,73	1,66	0,11978	0,94	1,91	0,00322	NM_005414	NBR2
8007462	0,73	1,66	0,10052	2,36	5,12	0,00169	NR_003108	LOC100129033
8116722	0,73	1,65	0,13819	1,27	2,40	0,06475	AY358807 TCERG1	
8108954	0,73	1,65	0,17093	2,95	7,75	0,00001	NM_006706	HMGB1
7970858	0,72	1,65	0,07960	0,96	1,94	0,05643	NM_002128	KDM5A
7960221	0,72	1,65	0,01921	1,52	2,87	0,00017	NM_001042603	MEMO1
8051372	0,72	1,65	0,13727	0,85	1,81	0,00995	NM_015955	TXNDC9
8100519	0,72	1,65	0,23452	0,69	1,61	0,13037	NM_005783	PLIN3
8032909	0,72	1,65	0,15080	2,14	4,39	0,00483	NM_005817	PPIP5K1
7988124	0,72	1,65	0,36611	1,66	3,15	0,00047	NM_014659	SLC39A13
7939805	0,72	1,65	0,02385	1,89	3,70	0,03729	NM_001128225	CRYZL1
8070141	0,72	1,65	0,00194	1,65	3,13	0,02448	NM_145858	LSAMP
8089714	0,72	1,65	0,23122	-1,83	-3,55	0,02376	NM_002338	LYRM5
7954460	0,72	1,65	0,15816	1,36	2,58	0,02531	NM_001001660	RALBP1
8020100	0,72	1,65	0,03701	2,50	5,67	0,00144	NM_006788	CCDC58
8089988	0,72	1,65	0,21420	1,53	2,89	0,05270	NM_001017928	TUBB2A
8116649	0,72	1,65	0,59680	-1,55	-2,93	0,02273	NM_001069	TUBB2A
8116653	0,72	1,65	0,59680	-1,55	-2,93	0,02273	NM_001069	PTPN21
7980616	0,72	1,65	0,12531	2,50	5,64	0,00428	NM_007039	DEPDC6
8148059	0,72	1,65	0,21021	0,79	1,73	0,08869	NM_022783	LEPRE1
7915444	0,72	1,65	0,23122	0,47	1,38	0,20625	NM_022356	FZD4
7950885	0,72	1,65	0,08962	-0,33	-1,26	0,33502	NM_012193	SYF2
7913814	0,72	1,65	0,10254	0,54	1,45	0,11597	NM_015484	AVL9
8132188	0,72	1,65	0,10669	2,74	6,69	0,00297	NM_015060	JARID2
8116998	0,72	1,65	0,22878	2,92	7,55	0,00018	NM_004973	APC
8107330	0,72	1,65	0,15130	0,75	1,68	0,03355	NM_001127511	NAA16
7968761	0,72	1,65	0,04479	2,08	4,23	0,00428	NM_024561	OSCP1
7914923	0,72	1,65	0,28589	-0,26	-1,20	0,59602	NM_145047	PRIM1
7964271	0,72	1,65	0,19217	4,27	19,33	0,00091	NM_000946	C17orf65
8015946	0,72	1,64	0,08302	2,16	4,47	0,00148	NM_178542	EEF1B2
8047771	0,72	1,64	0,27695	0,71	1,63	0,19193	NM_001959	PMS2L3
8140311	0,72	1,64	0,49658	1,50	2,82	0,24405	NR_028059	HIST1H4K
8124521	0,72	1,64	0,05594	2,03	4,09	0,00124	NM_003541	ADARB1
8069178	0,72	1,64	0,06291	1,16	2,23	0,02626	NR_027673	LOC388692
7905058	0,72	1,64	0,49287	-0,21	-1,16	0,72923	NR_027002	YAF2
7962358	0,72	1,64	0,01768	0,49	1,40	0,20183	NM_005748	RAD51
7982792	0,72	1,64	0,10907	3,20	9,19	0,00050	NM_002875	APOD
8092970	0,72	1,64	0,14300	-1,43	-2,70	0,01777	NM_001647	AHCY
8065738	0,72	1,64	0,12750	2,69	6,44	0,01564	NM_000687	NR2F2
7986329	0,72	1,64	0,05617	-0,02	-1,02	0,91869	NM_021005	ITGA6
8046380	0,71	1,64	0,12932	3,32	10,02	0,00038	NM_000210	RFC5
7959052	0,71	1,64	0,04052	2,86	7,26	0,00040	NM_181578	CNST
7911138	0,71	1,64	0,06874	0,77	1,70	0,01711	NM_152609	KIF16B
8065089	0,71	1,64	0,17046	1,18	2,26	0,00293	NM_024704	STC1
8149825	0,71	1,64	0,01114	0,34	1,27	0,10334	NM_003155	ELK4
7923778	0,71	1,64	0,05062	0,48	1,40	0,13359	NM_001973	HSD17B7
7906904	0,71	1,64	0,15697	1,74	3,35	0,00044	NM_016371	ZFYVE20
8085537	0,71	1,64	0,10690	1,17	2,26	0,01795	NM_022340	MCM9
8129214	0,71	1,64	0,18533	2,65	6,28	0,00054	AK299076 PARG	
7933561	0,71	1,64	0,04022	1,91	3,75	0,03677	NM_003631	BCKDK
7995040	0,71	1,64	0,10241	2,24	4,74	0,00314	NM_001122957	LRRC23
7953520	0,71	1,64	0,02768	0,49	1,41	0,44191	NM_201650	SYNCRIP
8127977	0,71	1,64	0,46877	0,96	1,94	0,12886	NM_001159677	P4HA3
7950374	0,71	1,64	0,14788	-0,22	-1,17	0,16497	NM_182904	SLC23A2
8064808	0,71	1,64	0,06342	2,65	6,27	0,00357	NM_005116	TRIP4
7984190	0,71	1,63	0,23474	2,82	7,08	0,00009	NM_016213	L3MBTL3
8122013	0,71	1,63	0,22201	1,14	2,20	0,01421	NM_032438	H2AFY2
7928107	0,71	1,63	0,06078	2,07	4,19	0,01447	NM_018649	TIMM44
8033465	0,71	1,63	0,10402	2,09	4,26	0,01095	NM_006351	PI4K2A
7929677	0,71	1,63	0,11126	2,19	4,57	0,00236	NM_018425	PRKCA
8009301	0,71	1,63	0,11295	3,63	12,37	0,00002	NM_002737	SERINC2
7899615	0,71	1,63	0,09875	1,25	2,37	0,11588	NM_178865	LOXL1
7984771	0,71	1,63	0,26365	-0,64	-1,56	0,14215	NM_005576	C14orf149
7979437	0,71	1,63	0,07747	2,38	5,21	0,00114	NM_144581	F8A1
8176245	0,71	1,63	0,09636	1,38	2,60	0,00834	NM_012151	TBC1D25
8167314	0,71	1,63	0,02618	1,73	3,32	0,03616	NM_002536	DGKA
7956046	0,71	1,63	0,38212	2,22	4,67	0,00411	NM_201444	PAQR5
7984524	0,71	1,63	0,01237	1,49	2,81	0,00663	NM_001104554	EIF2C2
8153201	0,71	1,63	0,31171	1,85	3,60	0,02057	NM_012154	NUMA1
7950086	0,71	1,63	0,20453	1,90	3,74	0,01901	NM_006185	ZNF177
8025488	0,71	1,63	0,02762	0,66	1,58	0,13628	NM_003451	PCDHB9

8108720	0,71	1,63	0,11044	0,02	1,01	0,92664	NM_019119	EZH2
8143663	0,70	1,63	0,30502	3,68	12,79	0,00014	NM_004456	BTBD2
8032380	0,70	1,63	0,09018	3,19	9,13	0,00690	NM_017797	DNAJA3
7992973	0,70	1,63	0,02715	2,57	5,94	0,00056	NM_005147	DCLK2
8097753	0,70	1,63	0,18071	0,02	1,01	0,94913	NM_001040260	ZNF624
8013035	0,70	1,63	0,14763	-0,64	-1,56	0,27483	NM_020787	DOPEY2
8068422	0,70	1,63	0,03780	2,30	4,91	0,00048	NM_005128	WDR25
7976766	0,70	1,63	0,00923	0,56	1,47	0,21847	NM_024515	PCIF1
8063097	0,70	1,62	0,13242	3,08	8,46	0,00472	NM_022104	CSGALNACT1
8149574	0,70	1,62	0,30516	2,57	5,94	0,01319	NM_018371	ZCCHC4
8094393	0,70	1,62	0,23812	2,53	5,79	0,00185	NM_024936	CASC5
7982757	0,70	1,62	0,06560	1,31	2,48	0,14875	NM_170589	RPP38
7926345	0,70	1,62	0,04087	3,32	9,98	0,00061	NM_183005	AMN1
7962185	0,70	1,62	0,22162	1,77	3,41	0,01269	NM_001113402	PDCD2
8130962	0,70	1,62	0,18927	0,89	1,86	0,06030	NM_002598	ID1
8061564	0,70	1,62	0,15495	2,78	6,85	0,00083	NM_181353	PLEKHA3
8046680	0,70	1,62	0,38467	0,26	1,20	0,59309	NM_019091	BDKRB2
7976560	0,70	1,62	0,34142	-1,35	-2,55	0,00073	NM_000623	ANKRD39
8053998	0,70	1,62	0,38942	1,24	2,37	0,01926	NM_016466	TPI1P2
8136061	0,70	1,62	0,28680	1,14	2,20	0,02120	NR_002187	RELN
8141950	0,70	1,62	0,00456	-1,04	-2,05	0,00139	NM_005045	LOC729992
8040362	0,70	1,62	0,10398	0,45	1,37	0,21888	XR_038318	EXOSC3
8161242	0,70	1,62	0,16259	1,35	2,54	0,04353	NM_016042	TET1
7927876	0,70	1,62	0,28426	2,31	4,95	0,00288	NM_030625	EPHA2
7912706	0,69	1,62	0,04759	3,06	8,36	0,00006	NM_004431	ATAD2
8152668	0,69	1,62	0,06116	2,18	4,52	0,01110	NM_014109	---
7982269	0,69	1,62	0,65555	-2,56	-5,88	0,01252	--- ---	
7987025	0,69	1,62	0,65555	-2,56	-5,88	0,01252	--- TMCO7	
7996860	0,69	1,62	0,30588	1,77	3,41	0,00021	NM_024562	DDAH2
8178590	0,69	1,62	0,12551	0,75	1,69	0,13582	NM_013974	DDAH2
8179819	0,69	1,62	0,12551	0,75	1,69	0,13582	NM_013974	NKAP
8174737	0,69	1,62	0,06896	0,55	1,46	0,12061	NM_024528	SLC25A37
8145291	0,69	1,62	0,25618	4,06	16,66	0,00027	AF495725 KLHL2	
8098177	0,69	1,62	0,11984	1,92	3,79	0,00490	NM_007246	---
8002711	0,69	1,62	0,25874	1,67	3,18	0,00191	--- BSDC1	
7914530	0,69	1,62	0,18968	1,32	2,50	0,01303	NM_001143888	STK36
8048381	0,69	1,61	0,16162	1,22	2,33	0,00073	NM_015690	PIK3IP1
8075483	0,69	1,61	0,13602	-1,03	-2,04	0,05146	NM_052880	OLFML3
7904158	0,69	1,61	0,17180	-1,89	-3,71	0,00540	NM_020190	DIRAS2
8162254	0,69	1,61	0,05094	-0,75	-1,68	0,02889	NM_017594	ZNF470
8031669	0,69	1,61	0,15722	0,13	1,09	0,78486	NM_001001668	UTY
8177137	0,69	1,61	0,13634	2,29	4,89	0,00030	NM_007125	DGKH
7968800	0,69	1,61	0,26679	2,90	7,46	0,00008	NM_178009	DOCK9
7972487	0,69	1,61	0,05870	3,05	8,28	0,00019	NM_015296	HSPA12A
7936516	0,69	1,61	0,02768	0,60	1,51	0,09751	NM_025015	PGPEP1
8026991	0,69	1,61	0,05440	0,42	1,34	0,09844	NM_017712	POLDIP3
8076465	0,69	1,61	0,00666	2,11	4,33	0,00231	NM_032311	NUP35
8046804	0,69	1,61	0,06849	1,75	3,36	0,00087	NM_138285	---
8052438	0,69	1,61	0,00929	1,73	3,32	0,00316	--- RAD17	
8105878	0,69	1,61	0,20675	2,01	4,04	0,00979	NM_133338	GABPB2
7905339	0,69	1,61	0,15844	2,10	4,29	0,00177	NM_144618	WDHD1
7979281	0,69	1,61	0,20101	2,84	7,14	0,00255	NM_007086	ANKRD36
8053801	0,69	1,61	0,24719	1,16	2,24	0,06038	NM_001164315	---
8043375	0,68	1,61	0,64760	-3,14	-8,83	0,00244	--- ---	
8097827	0,68	1,61	0,20531	0,43	1,34	0,23426	--- INVS	
8156873	0,68	1,61	0,01567	2,10	4,30	0,00078	NM_014425	---
8133856	0,68	1,61	0,46460	-0,37	-1,29	0,49157	--- KGFLP2	
8155530	0,68	1,60	0,09068	0,09	1,07	0,78841	NR_003670	PPM1D
8008922	0,68	1,60	0,07229	1,39	2,63	0,01292	NM_003620	TMEM101
8015900	0,68	1,60	0,37148	2,01	4,04	0,00027	NM_032376	KIAA0913
7928411	0,68	1,60	0,21030	1,45	2,73	0,00690	NM_015037	MDM4
7909011	0,68	1,60	0,29605	2,32	5,01	0,00217	NM_002393	ZNF350
8038919	0,68	1,60	0,24479	0,58	1,50	0,07690	NM_021632	NIT1
7906652	0,68	1,60	0,09778	2,39	5,24	0,00051	NM_005600	TMEM5
7956749	0,68	1,60	0,07346	1,64	3,11	0,01056	NM_014254	REL
8042144	0,68	1,60	0,34379	1,57	2,98	0,00002	NM_002908	CEBPB
8063386	0,68	1,60	0,21192	1,29	2,44	0,15217	NM_005194	C14orf167
7973446	0,68	1,60	0,09376	-0,54	-1,45	0,25753	ENST00000354854	MAN2B1
8034420	0,68	1,60	0,05037	1,55	2,94	0,02243	NM_000528	C19orf61
8037389	0,68	1,60	0,06162	1,31	2,48	0,00090	NM_019108	BANF1
7941501	0,68	1,60	0,39206	-0,52	-1,43	0,13938	NM_003860	TRIAP1
7967056	0,67	1,60	0,11224	1,05	2,07	0,02058	NM_016399	BECN1
8015715	0,67	1,60	0,30894	1,97	3,91	0,00322	NM_003766	DCPS

7945101	0,67	1,60	0,21423	2,16	4,47	0,00011	NM_014026	LRRFIP1
8049528	0,67	1,60	0,37857	0,10	1,07	0,85749	NM_001137550	ZCCHC17
7899604	0,67	1,59	0,08421	0,89	1,86	0,01576	NM_016505	WHAMM
7985482	0,67	1,59	0,08545	1,35	2,55	0,02823	NM_001080435	PDCD10
8091941	0,67	1,59	0,10987	1,08	2,11	0,00695	NM_007217	TTC12
7943919	0,67	1,59	0,12252	0,52	1,43	0,02449	NM_017868	RPS29
7978824	0,67	1,59	0,06897	1,84	3,59	0,00068	NM_001030001	TPRG1L
7897172	0,67	1,59	0,17381	1,60	3,02	0,00732	NM_182752	MRPL49
7941164	0,67	1,59	0,04586	0,99	1,98	0,14948	NM_004927	TMX3
8023735	0,67	1,59	0,24689	0,20	1,15	0,19794	NM_019022	---
8136889	0,67	1,59	0,19557	-1,03	-2,04	0,10648	--- FBXW5	
8165319	0,67	1,59	0,00887	1,13	2,18	0,09879	NM_018998	MSX2
8110084	0,67	1,59	0,31561	0,32	1,25	0,21673	NM_002449	---
8058518	0,67	1,59	0,19305	-0,35	-1,27	0,11770	--- APOL1	
8072735	0,67	1,59	0,25168	-1,30	-2,46	0,05119	NM_145343	XPNPEP2
8169836	0,67	1,59	0,42303	-0,83	-1,78	0,38176	NM_003399	GALNT10
8109407	0,67	1,59	0,05626	0,95	1,93	0,00291	NM_198321	CLUAP1
7992917	0,67	1,59	0,19362	0,34	1,26	0,17244	NM_015041	EDC4
7996685	0,67	1,59	0,03485	1,83	3,56	0,03175	NM_014329	TMEM150A
8053449	0,67	1,59	0,14031	1,35	2,54	0,08819	NM_001031738	TMPPE
8086048	0,67	1,59	0,11505	1,03	2,04	0,01187	NM_001039770	DNAJC27
8050790	0,67	1,59	0,01723	1,89	3,72	0,02663	NM_016544	CD74
8115147	0,67	1,59	0,19874	1,44	2,71	0,03443	NM_001025159	PAICS
8095221	0,67	1,59	0,06426	1,72	3,28	0,00635	NM_001079525	EIF3G
8033899	0,67	1,59	0,07963	2,68	6,41	0,00080	NM_003755	CPT1A
7949971	0,66	1,59	0,02202	0,96	1,94	0,00487	NM_001876	---
7986601	0,66	1,58	0,27327	-0,80	-1,75	0,02372	--- ---	
7986739	0,66	1,58	0,27327	-0,80	-1,75	0,02372	--- ZFHX3	
8002692	0,66	1,58	0,00200	1,13	2,19	0,03453	NM_006885	PLCXD3
8111887	0,66	1,58	0,12421	-1,09	-2,13	0,00689	NM_001005473	---
7954061	0,66	1,58	0,01378	0,12	1,09	0,79979	--- C9orf46	
8159984	0,66	1,58	0,39658	0,45	1,37	0,19433	NM_018465	AGBL5
8040774	0,66	1,58	0,00010	2,31	4,97	0,00107	NM_021831	TSTD2
8162676	0,66	1,58	0,05145	2,39	5,25	0,00067	NM_139246	ENOSF1
8021966	0,66	1,58	0,03878	2,56	5,91	0,00003	NM_017512	CENPO
8050763	0,66	1,58	0,21595	3,35	10,17	0,00099	ENST00000380834	SLC25A16
7934050	0,66	1,58	0,10165	0,60	1,51	0,00481	NM_152707	MED7
8115476	0,66	1,58	0,21185	1,05	2,07	0,02210	NM_004270	GAB2
7950671	0,66	1,58	0,14338	0,95	1,94	0,12290	NM_080491	ZNF252
8153935	0,66	1,58	0,05711	3,18	9,08	0,00232	NR_023392	COQ2
8101437	0,66	1,58	0,17690	1,59	3,01	0,00002	NM_015697	CCDC77
7952914	0,66	1,58	0,09815	1,40	2,64	0,00596	NM_032358	FICD
7958410	0,66	1,58	0,13476	2,54	5,80	0,00148	NM_007076	PSMB5
7977879	0,66	1,58	0,23369	1,37	2,58	0,00624	NM_001144932	MICAL2
7938485	0,66	1,58	0,07560	1,48	2,78	0,00190	NM_014632	PPP2R2B
8114900	0,66	1,58	0,18247	-0,42	-1,33	0,17662	NM_004576	IGF1
7965873	0,66	1,58	0,10978	-1,11	-2,17	0,02967	NM_001111283	ZNF192
8117646	0,66	1,58	0,13371	1,16	2,24	0,01609	NM_006298	CADM1
7951807	0,66	1,58	0,01155	0,77	1,71	0,02805	NM_014333	C7orf70
8155332	0,66	1,58	0,30981	2,94	7,65	0,00195	NM_001037163	FAM189B
7920707	0,66	1,58	0,34849	1,26	2,39	0,04241	NM_006589	TMEM185B
8054862	0,66	1,58	0,28295	1,29	2,45	0,15147	NR_000034	C3orf37
8082504	0,66	1,58	0,03078	1,61	3,06	0,07128	NM_001006109	MFSD11
8010092	0,66	1,57	0,15712	2,55	5,86	0,00070	NM_024311	ATPAF1
7915870	0,65	1,57	0,04530	0,65	1,57	0,02113	NM_022745	SCARB1
7967544	0,65	1,57	0,08408	2,80	6,98	0,00016	NM_005505	CYBASC3
7948565	0,65	1,57	0,04808	1,72	3,29	0,03774	NM_001161454	NUTF2
7996677	0,65	1,57	0,37118	1,54	2,90	0,01641	NM_005796	C1orf56
7905324	0,65	1,57	0,02708	2,97	7,86	0,00541	BC002469 CENPO	
8040578	0,65	1,57	0,22941	1,70	3,24	0,01391	NM_024322	STC2
8115851	0,65	1,57	0,21484	2,22	4,67	0,00368	NM_003714	CYCS
8138640	0,65	1,57	0,18825	1,50	2,82	0,01549	NM_018947	MSI2
8008682	0,65	1,57	0,00529	0,99	1,98	0,00700	NM_138962	PANX1
7943218	0,65	1,57	0,17077	1,47	2,77	0,00416	NM_015368	ORC4L
8055645	0,65	1,57	0,28593	1,52	2,88	0,00292	NM_002552	KIAA0141
8108832	0,65	1,57	0,14956	1,46	2,75	0,02720	NM_014773	KIAA0753
8011945	0,65	1,57	0,06852	1,22	2,33	0,07731	NM_014804	CHKA
7949916	0,65	1,57	0,07167	2,61	6,10	0,00799	NM_001277	GUCY1A3
8097957	0,65	1,57	0,16709	-1,24	-2,36	0,00001	NM_000856	AGPAT9
8096116	0,65	1,57	0,15614	3,92	15,14	0,00001	NM_032717	CASP8
8047419	0,65	1,57	0,03223	1,66	3,16	0,00003	NM_001228	PECR
8058837	0,65	1,57	0,10595	2,83	7,09	0,00025	NM_018441	PET112L
8103188	0,65	1,57	0,12115	0,01	1,01	0,96281	NM_004564	---

8150087	0,65	1,57	0,23429	-0,31	-1,24	0,21964	--- SLU7	
8115606	0,65	1,57	0,04594	0,81	1,76	0,06814	NM_006425	EDC3
7990379	0,65	1,57	0,09773	2,20	4,59	0,00283	NM_001142443	C22orf32
8073578	0,65	1,57	0,03346	1,98	3,95	0,00050	BC024237 SF3B2	
7941511	0,65	1,57	0,26422	1,08	2,11	0,10179	NM_006842	SYS1
8062964	0,65	1,57	0,22183	1,18	2,27	0,03044	NM_033542	LANCL2
8132897	0,65	1,57	0,35123	1,42	2,68	0,00192	NM_018697	PABPN1
7973384	0,65	1,57	0,14489	2,07	4,20	0,00492	NM_004643	ZNF83
8038967	0,65	1,57	0,06434	1,14	2,21	0,01109	NM_001105549	OAS2
7958913	0,65	1,57	0,20301	-1,20	-2,29	0,00043	NM_002535	SOX5
7961798	0,65	1,57	0,24316	-1,06	-2,08	0,02521	NM_152989	RNF135
8006229	0,65	1,56	0,20997	2,59	6,00	0,00130	NM_032322	IFT74
8154670	0,65	1,56	0,23322	0,77	1,70	0,07891	NM_025103	SRRD
8072113	0,65	1,56	0,28911	2,23	4,69	0,00170	NM_001013694	GPR125
8022338	0,64	1,56	0,08917	2,51	5,68	0,00237	NM_145290	PACS2
7977344	0,64	1,56	0,31716	2,46	5,52	0,00099	NM_001100913	MIR147
8163729	0,64	1,56	0,43889	-0,90	-1,87	0,00471	NR_029604	FYN
8128956	0,64	1,56	0,00743	0,65	1,57	0,02118	NM_002037	ZDBF2
8047784	0,64	1,56	0,36365	3,07	8,37	0,00360	NM_020923	PLBD2
7958989	0,64	1,56	0,20557	0,86	1,81	0,17910	NM_173542	S100A6
7920258	0,64	1,56	0,43591	-0,23	-1,17	0,69562	NM_014624	C21orf119
8068200	0,64	1,56	0,09094	2,16	4,46	0,00074	NR_026845	NUCKS1
7909142	0,64	1,56	0,14409	-0,38	-1,30	0,47287	NM_022731	VAV2
8164967	0,64	1,56	0,03629	2,40	5,28	0,00088	NM_001134398	ZNF275
8170635	0,64	1,56	0,16955	1,03	2,05	0,00652	NM_001080485	KRAS
7961865	0,64	1,56	0,23033	1,78	3,42	0,00159	NM_033360	PDGFRB
8115099	0,64	1,56	0,08115	-0,81	-1,75	0,01665	NM_002609	RRAGD
8128123	0,64	1,56	0,14857	2,72	6,57	0,00003	NM_021244	SLC35A3
7903281	0,64	1,56	0,05613	2,34	5,08	0,00084	NM_012243	WDR77
7918517	0,64	1,56	0,28581	2,07	4,21	0,01631	NM_024102	---
8116651	0,64	1,56	0,31319	-0,44	-1,36	0,35942	--- CWC22	
8057441	0,64	1,56	0,02585	1,15	2,22	0,02461	NM_020943	DCUN1D5
7951325	0,64	1,56	0,32579	2,96	7,78	0,00069	NM_032299	ALKBH8
7951467	0,64	1,56	0,01397	1,34	2,54	0,00265	NM_138775	MAP7
8129783	0,64	1,56	0,08734	1,82	3,53	0,00317	NM_003980	MYST4
7928491	0,64	1,56	0,17071	2,57	5,94	0,00117	NM_012330	GIN1
8113403	0,64	1,56	0,12083	0,73	1,65	0,15654	NM_017676	TTC4
7901662	0,64	1,56	0,08404	0,03	1,02	0,83477	NM_004623	CMTM3
7996318	0,64	1,55	0,40482	1,98	3,93	0,06382	NM_144601	PUS3
7952522	0,64	1,55	0,12107	0,80	1,74	0,12314	NM_031307	RFX3
8159876	0,64	1,55	0,17718	1,15	2,22	0,03058	NM_134428	ABCA9
8017927	0,64	1,55	0,25796	-0,72	-1,65	0,00196	NM_080283	KIF11
7929258	0,64	1,55	0,09194	3,22	9,32	0,00017	NM_004523	ERO1LB
7925342	0,64	1,55	0,14033	3,10	8,57	0,00058	NM_019891	MRPS25
8085531	0,63	1,55	0,20502	1,53	2,88	0,00390	NM_022497	IL7R
8104901	0,63	1,55	0,17366	-1,03	-2,04	0,05074	NM_002185	ZNF772
8039645	0,63	1,55	0,09875	0,42	1,34	0,01888	NM_001024596	PCOLCE2
8091243	0,63	1,55	0,18845	0,93	1,90	0,05212	NM_013363	ITFG3
7991779	0,63	1,55	0,05372	1,64	3,11	0,01935	NM_032039	INPP5B
7915101	0,63	1,55	0,08500	1,67	3,19	0,00175	NM_005540	CLN6
7989975	0,63	1,55	0,05218	1,62	3,07	0,01219	NM_017882	BAT2L1
8158829	0,63	1,55	0,31552	3,58	11,99	0,00300	NM_013318	PRSS12
8102468	0,63	1,55	0,06393	-0,59	-1,50	0,21593	NM_003619	IFT81
7958620	0,63	1,55	0,18321	0,82	1,77	0,10495	NM_014055	GSTT2
8071809	0,63	1,55	0,27697	0,64	1,55	0,17141	NM_000854	GSTT2
8074962	0,63	1,55	0,27697	0,64	1,55	0,17141	NM_000854	BASP1
8104601	0,63	1,55	0,02895	1,07	2,09	0,04250	NM_006317	KLHL15
8171837	0,63	1,55	0,17992	2,55	5,87	0,00028	NM_030624	ACYP2
8041982	0,63	1,55	0,13686	0,55	1,46	0,11169	NM_138448	VMA21
8170479	0,63	1,55	0,03509	0,86	1,82	0,05289	NM_001017980	CASP6
8102311	0,63	1,55	0,27260	1,72	3,30	0,04713	NM_001226	PDE4DIP
7919168	0,63	1,55	0,19169	-1,47	-2,77	0,00214	NM_022359	CNP
8007188	0,63	1,55	0,26233	2,43	5,39	0,00551	NM_033133	RAC2
8075910	0,63	1,55	0,13977	0,00	1,00	0,98789	NM_002872	C9orf37
8165630	0,63	1,55	0,10176	3,01	8,08	0,00199	NM_032937	WIZ
8035040	0,63	1,55	0,12155	0,57	1,49	0,09105	NM_021241	AREG
8095744	0,63	1,55	0,05685	5,68	51,12	0,00074	NM_001657	TADA3
8085220	0,63	1,55	0,26489	2,51	5,70	0,00073	NM_133480	TSC2
7992474	0,63	1,55	0,05873	2,48	5,58	0,00388	NM_000548	TEK
8154692	0,63	1,55	0,24377	-0,85	-1,80	0,00543	NM_000459	MEF2D
7921014	0,63	1,55	0,24873	1,97	3,92	0,00269	NM_005920	ARMC10
8135229	0,63	1,55	0,49108	1,61	3,05	0,03052	NM_031905	PEMT
8013120	0,63	1,55	0,08218	1,96	3,89	0,00128	NM_148172	NHSL1

8129888	0,63	1,55	0,07214	0,75	1,68	0,03971	NM_001144060	TTC13
7925003	0,63	1,54	0,54357	3,15	8,89	0,00002	NM_024525	CHTF8
8002266	0,63	1,54	0,47335	1,95	3,87	0,00360	NR_033227	UTP18
8008530	0,63	1,54	0,26304	1,50	2,83	0,00085	NM_016001	---
7957611	0,63	1,54	0,04212	-0,29	-1,22	0,25361	--- ZNF18	
8012843	0,63	1,54	0,08211	0,62	1,54	0,05619	NM_144680	DIS3L
7984298	0,63	1,54	0,06774	2,68	6,40	0,00054	NM_133375	VBP1
8171013	0,63	1,54	0,26115	1,60	3,03	0,00703	NM_003372	TRPV2
8005171	0,62	1,54	0,21610	-1,43	-2,69	0,00471	NM_016113	ZNF236
8021783	0,62	1,54	0,08238	2,06	4,16	0,00003	NM_007345	---
8088893	0,62	1,54	0,34637	0,93	1,91	0,05167	--- AASS	
8142554	0,62	1,54	0,04898	0,83	1,77	0,03530	NM_005763	RPL23AP82
7911307	0,62	1,54	0,03927	0,09	1,07	0,80634	NR_026981	GNB2
8134890	0,62	1,54	0,17598	0,68	1,61	0,14494	NM_005273	HSPB7
7912692	0,62	1,54	0,38802	-0,73	-1,66	0,01024	NM_014424	SOCS6
8021707	0,62	1,54	0,23840	0,56	1,48	0,11863	NM_004232	ICMT
7911993	0,62	1,54	0,12378	1,63	3,10	0,00493	NM_012405	SFRS9
7967060	0,62	1,54	0,29702	1,20	2,30	0,00627	NM_003769	LOC149837
8064866	0,62	1,54	0,39221	0,91	1,88	0,15612	NR_015406	GPR63
8128316	0,62	1,54	0,14129	1,04	2,06	0,03322	NM_001143957	TAF6
8141425	0,62	1,54	0,24384	2,05	4,14	0,00138	NM_005641	LSM2
8125125	0,62	1,54	0,17548	1,38	2,61	0,01347	NM_021177	LSM2
8178641	0,62	1,54	0,17548	1,38	2,61	0,01347	NM_021177	LSM2
8179839	0,62	1,54	0,17548	1,38	2,61	0,01347	NM_021177	PAN2
7964089	0,62	1,54	0,18532	2,46	5,49	0,00424	NM_001127460	FMN1
7987145	0,62	1,54	0,07323	-0,71	-1,64	0,02820	NM_001103184	C15orf61
7984405	0,62	1,54	0,31932	2,52	5,73	0,00373	NM_001143936	ZNF238
7911038	0,62	1,54	0,09005	0,39	1,31	0,09333	NM_205768	GCN1L1
7966938	0,62	1,54	0,08471	3,37	10,33	0,00098	NM_006836	SOCS3
8018864	0,62	1,53	0,07220	0,42	1,34	0,10231	NM_003955	LIMK2
8072461	0,62	1,53	0,12316	0,09	1,06	0,84176	NM_016733	ZC3H12C
7943715	0,62	1,53	0,08805	2,20	4,61	0,00284	NM_033390	LIPM
7929003	0,62	1,53	0,07219	-0,90	-1,86	0,05908	NM_001128215	KDM3B
8108330	0,62	1,53	0,15087	2,29	4,89	0,00015	NM_016604	PROCR
8062108	0,62	1,53	0,35849	1,23	2,34	0,00884	NM_006404	KAT5
7941382	0,62	1,53	0,25139	2,33	5,02	0,00799	NM_182710	PHC1
7953812	0,62	1,53	0,29240	2,36	5,13	0,00006	NM_004426	NPIPL3
7994371	0,62	1,53	0,42565	1,88	3,69	0,00198	NM_130464	C6orf136
8177851	0,62	1,53	0,26930	2,98	7,91	0,00024	NM_001161376	C6orf136
8179167	0,62	1,53	0,26930	2,98	7,91	0,00024	NM_001161376	EFTUD1
7990879	0,62	1,53	0,13582	1,16	2,23	0,13046	NM_024580	TFCP2
7963244	0,62	1,53	0,11843	2,05	4,14	0,00142	NM_005653	HDDC3
7991401	0,61	1,53	0,18867	1,14	2,21	0,01123	NM_198527	SLC2A1
7915472	0,61	1,53	0,33010	3,22	9,30	0,00071	NM_006516	KNTC1
7959408	0,61	1,53	0,10777	2,76	6,75	0,00176	NM_014708	ARMC7
8009755	0,61	1,53	0,00801	1,53	2,89	0,01028	NM_024585	GEN1
8040440	0,61	1,53	0,20743	1,81	3,50	0,00703	NM_182625	MGC70870
8019563	0,61	1,53	0,26075	3,26	9,55	0,00777	NR_003682	NADSYN1
7942232	0,61	1,53	0,00523	1,40	2,64	0,04693	NM_018161	NBPF16
7904574	0,61	1,53	0,56032	0,04	1,03	0,90522	NM_001102663	HEATR2
8131000	0,61	1,53	0,18294	2,26	4,79	0,00563	NM_017802	C1orf50
7900597	0,61	1,53	0,20567	0,31	1,24	0,32247	BC001508 CXCL2	
8100994	0,61	1,53	0,00675	0,95	1,93	0,07735	NM_002089	FKBP9
8132214	0,61	1,53	0,14401	0,42	1,33	0,40010	NM_007270	RBBP9
8065230	0,61	1,53	0,15697	1,26	2,40	0,02672	NM_006606	GSTCD
8096688	0,61	1,52	0,27732	1,09	2,13	0,08036	NM_001031720	CHD3
8004699	0,61	1,52	0,25433	1,35	2,55	0,00339	NM_001005273	PDPK1
7998823	0,61	1,52	0,27145	1,02	2,03	0,01443	NM_002613	PDPK1
7998841	0,61	1,52	0,27145	1,02	2,03	0,01443	NM_002613	DIABLO
7967230	0,61	1,52	0,06153	1,85	3,62	0,00150	NM_019887	DNM2
8025741	0,61	1,52	0,41110	2,82	7,05	0,00009	NM_001005360	CNOT8
8109462	0,60	1,52	0,21270	2,62	6,14	0,00009	NM_004779	CSNK1E
8076056	0,60	1,52	0,08924	2,70	6,48	0,00388	NM_001894	PLEKHF2
8147439	0,60	1,52	0,03054	1,94	3,85	0,01912	NM_024613	MPZL3
7952036	0,60	1,52	0,14075	2,93	7,64	0,00112	NM_198275	TBL1X
8165911	0,60	1,52	0,45320	3,33	10,05	0,00022	NM_005647	HINT3
8121886	0,60	1,52	0,25951	1,32	2,49	0,00202	NM_138571	PCYT1A
8093086	0,60	1,52	0,26065	1,42	2,67	0,04457	NM_005017	ZNF142
8058973	0,60	1,52	0,19247	2,23	4,69	0,00030	NM_001105537	RARRES2
8143772	0,60	1,52	0,21480	-2,58	-5,98	0,00477	NM_002889	C15orf17
7990429	0,60	1,52	0,21235	2,97	7,82	0,00316	NM_020447	PAAF1
7942476	0,60	1,52	0,21170	1,31	2,47	0,00313	NM_025155	PRPF6
8064218	0,60	1,52	0,09365	1,03	2,05	0,20471	NM_012469	FAM110B

8146533	0,60	1,52	0,11482	-0,88	-1,84	0,00668	NM_147189	PDE8B
8106448	0,60	1,52	0,27239	1,38	2,60	0,00046	NM_003719	AP1S3
8059350	0,60	1,52	0,17145	0,97	1,96	0,05135	NM_001039569	ZNF85
8027272	0,60	1,52	0,29028	0,10	1,07	0,62215	NM_003429	PCDHB16
8108716	0,60	1,52	0,19353	-0,78	-1,72	0,18001	NM_020957	DCLRE1C
7932186	0,60	1,52	0,06312	1,71	3,28	0,00381	NM_001033858	LOC284751
8063389	0,60	1,52	0,14383	0,29	1,22	0,51897	AK090605 CAMK1D	
7926223	0,60	1,52	0,05569	0,37	1,30	0,21593	NM_153498	MTIF2
8052250	0,60	1,52	0,03105	2,10	4,29	0,00285	NM_001005369	UTS2D
8092741	0,60	1,51	0,08111	-0,18	-1,13	0,25837	NM_198152	C20orf29
8060698	0,60	1,51	0,04546	0,37	1,29	0,11456	BC043344 ---	
8107934	0,60	1,51	0,17027	-0,44	-1,36	0,21310	--- BPHL	
8116635	0,60	1,51	0,17998	3,83	14,23	0,00058	NR_026650	RANBP3
8033075	0,60	1,51	0,32284	1,98	3,94	0,00298	NM_003624	LOC100288114
8031646	0,60	1,51	0,26084	3,77	13,61	0,00305	AK302988 TXNDC11	
7999478	0,60	1,51	0,06429	1,67	3,18	0,00237	NM_015914	ZNF792
8036045	0,60	1,51	0,11725	1,38	2,60	0,05785	NM_175872	ATP6V1G2
8178508	0,60	1,51	0,30272	0,42	1,33	0,26817	NM_130463	EIF3B
8131111	0,60	1,51	0,32410	1,65	3,15	0,00646	NM_001037283	TBC1D2
8162759	0,60	1,51	0,22797	0,42	1,33	0,22670	NM_018421	PRICKLE2
8088550	0,60	1,51	0,09048	-1,21	-2,31	0,06559	NM_198859	TOLLIP
7945620	0,60	1,51	0,04908	0,93	1,90	0,02627	NM_019009	CACNA1C
7953040	0,60	1,51	0,07154	-0,93	-1,90	0,00241	NM_199460	KCTD2
8009737	0,60	1,51	0,01594	1,96	3,90	0,00476	NM_015353	ATP6V0A2
7959657	0,60	1,51	0,08014	3,22	9,35	0,00170	NM_012463	FAM199X
8169085	0,59	1,51	0,01206	1,01	2,01	0,01426	NM_207318	CHD1L
7904883	0,59	1,51	0,01958	1,42	2,68	0,00626	NM_004284	C2orf3
8053288	0,59	1,51	0,27879	1,64	3,12	0,00969	NM_003203	UFD1L
8074464	0,59	1,51	0,45940	1,00	2,00	0,12883	NM_005659	CNTLN
8154449	0,59	1,51	0,01200	0,58	1,49	0,07654	NM_017738	EDEM2
8065855	0,59	1,51	0,17303	0,63	1,55	0,09592	NM_018217	---
8052022	0,59	1,51	0,46036	-1,09	-2,13	0,09768	--- CXorf38	
8172110	0,59	1,51	0,03639	0,80	1,74	0,00219	NM_144970	SCIN
8131550	0,59	1,51	0,12919	-0,58	-1,50	0,01736	NM_001112706	SPTLC2
7980438	0,59	1,51	0,14099	2,33	5,03	0,00015	NM_004863	ZNF232
8011817	0,59	1,51	0,02535	1,34	2,52	0,00446	NM_014519	SLC3A2
7940717	0,59	1,51	0,17122	3,67	12,72	0,00164	NM_001012661	POR
8133670	0,59	1,51	0,30079	1,33	2,51	0,04764	NM_000941	EGFR
8132860	0,59	1,51	0,19871	2,64	6,22	0,00229	NM_005228	SCAPER
7990582	0,59	1,51	0,10303	1,41	2,67	0,00353	NM_020843	C11orf80
7941714	0,59	1,51	0,25133	1,84	3,58	0,00420	NM_024650	MEMO1
8068410	0,59	1,51	0,19460	0,68	1,60	0,01340	NM_015955	C13orf34
7969374	0,59	1,51	0,12607	2,01	4,04	0,00867	NM_024808	TMCO3
7970301	0,59	1,51	0,22425	1,53	2,88	0,00219	NM_017905	SLC25A12
8056766	0,59	1,51	0,24187	2,66	6,32	0,00146	NM_003705	SLC36A1
8109350	0,59	1,51	0,12813	2,31	4,95	0,00008	NM_078483	PRDM1
8121257	0,59	1,51	0,11973	-0,81	-1,75	0,00427	NM_001198	CCDC69
8115261	0,59	1,51	0,13359	-0,59	-1,50	0,15436	NM_015621	PHF8
8173009	0,59	1,51	0,16289	2,24	4,71	0,00140	NM_015107	C6orf35
8130464	0,59	1,51	0,38784	1,24	2,35	0,02086	NM_018452	SLC7A3
8173414	0,59	1,51	0,09898	-0,61	-1,53	0,01868	NM_032803	HLA-DQA1
8178199	0,59	1,50	0,53809	-1,73	-3,31	0,00074	NM_002122	HLA-DQA1
8179489	0,59	1,50	0,53809	-1,73	-3,31	0,00074	NM_002122	RHOD
7941761	0,59	1,50	0,37350	2,31	4,96	0,00080	NM_014578	C19orf6
8032137	0,59	1,50	0,19671	2,97	7,86	0,00230	NM_001033026	ATP11A
7970162	0,59	1,50	0,23986	3,26	9,61	0,00003	NM_015205	---
7987403	0,59	1,50	0,23108	-1,29	-2,45	0,00823	--- NEFM	
8145361	0,59	1,50	0,10768	-0,95	-1,94	0,02763	NM_005382	MALAT1
7949410	0,59	1,50	0,28705	-1,51	-2,85	0,04925	BC018448 CPEB3	
7935011	0,59	1,50	0,00538	0,48	1,40	0,18962	NM_014912	ZNF304
8031726	0,59	1,50	0,17492	1,55	2,94	0,00008	NM_020657	ISLR
7984813	0,59	1,50	0,41135	-1,47	-2,78	0,00125	NM_005545	MRVI1
7946589	0,59	1,50	0,32504	-0,53	-1,45	0,06636	NM_130385	EPN2
8005557	0,59	1,50	0,33438	1,21	2,31	0,06507	NM_014964	ZNF337
8065517	0,59	1,50	0,03092	0,01	1,01	0,84030	NM_015655	LDOC1L
8076673	0,59	1,50	0,12198	1,82	3,52	0,01095	NM_032287	HMGA1
8118794	0,59	1,50	0,41665	1,47	2,76	0,05039	NM_145899	GPN3
7966321	0,59	1,50	0,29300	2,43	5,38	0,00034	NM_001164373	KIAA1324L
8140709	0,59	1,50	0,13036	3,96	15,58	0,00160	NR_030672	MRC2
8009040	0,59	1,50	0,08095	0,43	1,34	0,36122	NM_006039	SLC12A8
8090214	0,58	1,50	0,09972	0,27	1,21	0,08390	NM_024628	ZNF438
7932867	0,58	1,50	0,02804	0,64	1,56	0,16064	NM_001143770	PPARD
8118945	0,58	1,50	0,17974	1,46	2,75	0,05648	NM_001171818	TNFRSF19

7968015	0,58	1,50	0,28653	-0,96	-1,94	0,00234	NM_148957	MRPL27
8016699	0,58	1,50	0,08141	0,26	1,20	0,32336	NM_016504	NDUFA7
8033564	0,58	1,50	0,23036	1,89	3,71	0,00891	NM_005001	C20orf111
8066402	0,58	1,50	0,18494	2,13	4,37	0,00046	NM_016470	TBC1D9B
8116348	0,58	1,50	0,04065	1,85	3,60	0,00800	NM_198868	NPRL3
7998157	0,58	1,50	0,13799	1,73	3,32	0,00415	NM_001077350	SMCR8
8005441	0,58	1,50	0,21874	1,43	2,69	0,01546	NM_144775	DRP2
8168817	0,58	1,50	0,16643	-0,94	-1,92	0,07066	NM_001939	S1PR1
7903393	0,58	1,50	0,44126	-0,53	-1,44	0,04351	NM_001400	RIC8A
7937275	0,58	1,50	0,02633	2,45	5,48	0,04307	NM_021932	---
8051773	0,58	1,49	0,10119	0,90	1,86	0,04336	---	SNORD29
7948902	0,58	1,49	0,17455	1,37	2,58	0,11676	NR_002559	---
8043489	0,58	1,49	0,30199	-0,88	-1,85	0,04435	---	C14orf109
7976333	0,58	1,49	0,00681	0,97	1,96	0,11800	NM_015676	TJP2
8155707	0,58	1,49	0,13062	2,01	4,03	0,00005	NM_004817	DCAF8
7921552	0,58	1,49	0,03775	2,49	5,61	0,00001	NR_028103	---
7982248	0,58	1,49	0,71439	-2,85	-7,19	0,00563	---	ZNF135
8031857	0,58	1,49	0,05785	-0,93	-1,91	0,01083	NM_003436	NSMCE4A
7936777	0,58	1,49	0,03596	0,72	1,65	0,08763	NM_017615	REEP4
8149673	0,58	1,49	0,06700	2,74	6,70	0,00482	NM_025232	NKTR
8079079	0,58	1,49	0,25792	0,76	1,69	0,01002	NM_005385	BET1L
7945349	0,58	1,49	0,34782	1,46	2,74	0,02431	NM_016526	TNRC6B
8073214	0,58	1,49	0,04398	1,57	2,96	0,00612	NM_001024843	LEO1
7988838	0,58	1,49	0,15718	1,25	2,38	0,00382	NM_138792	SEMA7A
7990345	0,58	1,49	0,09507	2,31	4,96	0,01338	NM_003612	SH3BP5
8085556	0,58	1,49	0,00527	-0,24	-1,18	0,47799	NM_004844	PPP2R5A
7909586	0,58	1,49	0,10203	2,38	5,22	0,00127	NM_006243	CDC37L1
8154153	0,58	1,49	0,15044	0,83	1,78	0,00919	NM_017913	ESCO2
8145570	0,58	1,49	0,05125	1,19	2,27	0,07597	NM_001017420	C1orf212
7914750	0,58	1,49	0,22875	0,74	1,67	0,10826	NM_138428	HSPB11
7916356	0,58	1,49	0,13539	1,06	2,09	0,00035	NM_016126	WWP2
7996976	0,57	1,49	0,09021	1,73	3,33	0,01875	NM_007014	SRA1
8114583	0,57	1,49	0,05676	0,16	1,11	0,37117	NM_001035235	ANKS1A
8118863	0,57	1,49	0,14352	2,73	6,62	0,00164	NM_015245	ARHGAP10
8097717	0,57	1,49	0,01415	0,18	1,13	0,33928	NM_024605	DUSP6
7965335	0,57	1,49	0,27093	2,30	4,94	0,00444	NM_001946	DHPS
8034454	0,57	1,49	0,05958	3,22	9,34	0,00300	NM_001930	GLTSCR2
8029958	0,57	1,49	0,01466	0,72	1,64	0,00668	NM_015710	SPATA17
7909768	0,57	1,49	0,30725	0,45	1,37	0,37581	NM_138796	HM13
8061542	0,57	1,49	0,00271	1,85	3,59	0,00294	NM_178580	POLR3A
7934653	0,57	1,49	0,21513	1,79	3,46	0,00274	NM_007055	PIAS3
7904812	0,57	1,49	0,21727	3,28	9,71	0,00079	NM_006099	C6orf136
8117987	0,57	1,49	0,21712	2,69	6,43	0,00050	NM_001161376	MCM6
8055426	0,57	1,49	0,24207	2,04	4,10	0,01846	NM_005915	RBL1
8066136	0,57	1,48	0,21004	3,06	8,34	0,00010	NM_002895	PPIA
8132642	0,57	1,48	0,22751	0,83	1,77	0,13769	NM_021130	GCC1
8142740	0,57	1,48	0,09931	0,49	1,41	0,00054	NM_024523	ST6GALNAC2
8018761	0,57	1,48	0,11244	2,70	6,50	0,00006	NM_006456	PDXDC2
8000537	0,57	1,48	0,49730	1,71	3,28	0,00779	NR_003610	MBOAT7
8039166	0,57	1,48	0,09008	1,29	2,44	0,11242	NM_024298	RNF214
7944096	0,57	1,48	0,19570	2,05	4,13	0,00281	NM_207343	TMED3
7985240	0,57	1,48	0,04048	0,65	1,57	0,17053	NM_007364	TNFSF15
8163618	0,57	1,48	0,25680	-0,75	-1,68	0,01553	NM_005118	NRG1
8145736	0,57	1,48	0,04857	-0,71	-1,64	0,10233	NM_013958	ZNF274
8031871	0,57	1,48	0,15311	0,77	1,71	0,00629	NM_133502	CAPG
8053417	0,57	1,48	0,39664	1,62	3,08	0,00892	NM_001747	ZKSCAN1
8134680	0,57	1,48	0,42969	1,39	2,63	0,00654	NM_003439	DOM3Z
8179926	0,57	1,48	0,30379	0,77	1,71	0,00955	NM_005510	THRB
8085797	0,57	1,48	0,06386	-0,09	-1,07	0,52366	NM_001128176	EXOC8
7925043	0,57	1,48	0,34155	1,86	3,62	0,00357	NM_175876	ARL5B
7926531	0,57	1,48	0,34855	2,94	7,68	0,00052	NM_178815	SEC61A2
7926189	0,57	1,48	0,09547	0,82	1,77	0,09107	NM_018144	SDC3
7914282	0,56	1,48	0,03240	1,04	2,05	0,02171	NM_014654	TGS1
8146482	0,56	1,48	0,21732	1,35	2,55	0,00232	NM_024831	AHCYL2
8136095	0,56	1,48	0,17900	3,86	14,55	0,00015	NM_015328	ITFG2
7953111	0,56	1,48	0,04383	1,28	2,43	0,03390	NM_018463	SALL2
7977761	0,56	1,48	0,13687	0,61	1,53	0,16049	NM_005407	RALGAPA2
8065280	0,56	1,48	0,38509	3,10	8,58	0,00014	NM_020343	---
7918465	0,56	1,48	0,39006	-1,98	-3,96	0,00472	---	SAMD12
8152506	0,56	1,48	0,29880	0,38	1,30	0,15319	NM_207506	SMYD3
7925589	0,56	1,48	0,16903	0,80	1,74	0,05602	NM_001167740	CGRRF1
7974418	0,56	1,48	0,17222	1,28	2,43	0,00961	NM_006568	NCK2
8044133	0,56	1,48	0,20837	2,05	4,13	0,00191	NM_003581	C13orf31

7968883	0,56	1,48	0,27581	0,56	1,48	0,25390	NM_153218	WDR6
8079693	0,56	1,48	0,06671	2,42	5,36	0,00257	NM_018031	GCA
8046003	0,56	1,48	0,25665	1,86	3,62	0,01795	NM_012198	C10orf26
7930162	0,56	1,48	0,17759	0,36	1,29	0,30237	NM_017787	PI4KAP2
8074577	0,56	1,47	0,06934	3,40	10,54	0,00011	NR_003700	NPM3
7935903	0,56	1,47	0,20897	1,68	3,21	0,02493	NM_006993	CC2D1B
7916135	0,56	1,47	0,19174	0,80	1,74	0,00472	NM_032449	CBY1
8073022	0,56	1,47	0,17800	1,78	3,44	0,01603	NM_001002880	FOXJ2
7953699	0,56	1,47	0,04618	1,52	2,87	0,00303	NM_018416	PIGC
7922330	0,56	1,47	0,26951	1,08	2,12	0,00634	NM_153747	---
8118824	0,56	1,47	0,10187	0,62	1,54	0,03587	--- DFFA	
7912303	0,56	1,47	0,13816	2,14	4,40	0,00405	NM_213566	RAB33B
8097507	0,56	1,47	0,10288	1,95	3,85	0,01458	NM_031296	ADRM1
8063893	0,56	1,47	0,44334	3,02	8,12	0,00230	NM_007002	ARFGAP2
7947784	0,56	1,47	0,10216	2,09	4,25	0,00595	NM_032389	PDXDC2
8000501	0,56	1,47	0,52566	1,53	2,89	0,01980	NR_003610	TMEM161A
8035600	0,56	1,47	0,14413	2,41	5,31	0,00181	NM_017814	UTP3
8095539	0,56	1,47	0,08135	0,39	1,31	0,25123	NM_020368	ACPP
8082673	0,56	1,47	0,29265	0,39	1,31	0,28761	NM_001099	SSH1
7966150	0,56	1,47	0,28465	2,03	4,10	0,07097	NM_001161330	G6PC3
8007561	0,56	1,47	0,09800	2,60	6,08	0,00631	NM_138387	FLJ30064
8134460	0,55	1,47	0,23875	0,36	1,28	0,05785	AK054626 CNRIP1	
8052735	0,55	1,47	0,01542	-0,94	-1,92	0,00155	NM_015463	HPS5
7946988	0,55	1,47	0,29689	2,45	5,45	0,00030	NM_181507	LOC554223
8179028	0,55	1,47	0,20847	0,34	1,26	0,69848	AK128290 C2orf69	
8047262	0,55	1,47	0,15322	0,91	1,88	0,14348	NM_153689	RFC4
8092640	0,55	1,47	0,38148	3,99	15,94	0,00080	NM_002916	SETDB2
7969114	0,55	1,47	0,09804	0,62	1,54	0,06036	NM_031915	ZXDC
8090351	0,55	1,47	0,25025	1,61	3,05	0,00135	NM_025112	CASP7
7930577	0,55	1,47	0,15409	0,67	1,59	0,01457	NM_033338	RNF213
8010426	0,55	1,47	0,24815	1,27	2,41	0,00652	NM_020914	SMARCD2
8017460	0,55	1,47	0,10328	0,89	1,85	0,16208	NM_001098426	GLUL
7922689	0,55	1,47	0,10069	0,49	1,41	0,00619	NM_002065	OMA1
7916570	0,55	1,46	0,07930	0,77	1,71	0,04810	NM_145243	HYLS1
7945040	0,55	1,46	0,07051	0,59	1,50	0,21568	NM_145014	STK10
8115783	0,55	1,46	0,31536	1,10	2,14	0,01754	NM_005990	TCTEX1D2
8093096	0,55	1,46	0,16769	1,64	3,12	0,00485	NM_152773	TRIB2
8040365	0,55	1,46	0,47409	-1,07	-2,10	0,07754	NM_021643	GK3P
8103535	0,55	1,46	0,40362	1,95	3,86	0,02984	NR_026575	---
8103904	0,55	1,46	0,26707	0,42	1,34	0,03431	--- RAB33A	
8169898	0,55	1,46	0,06955	0,17	1,13	0,63393	NM_004794	ZNF184
8124502	0,55	1,46	0,04592	2,05	4,15	0,00336	NM_007149	ANKRD13B
8006063	0,55	1,46	0,27367	2,01	4,04	0,00182	NM_152345	ELL
8035477	0,55	1,46	0,04572	0,32	1,25	0,05188	NM_006532	MCC
8113551	0,55	1,46	0,52763	1,08	2,12	0,06255	NM_001085377	MRE11A
7951046	0,55	1,46	0,19783	0,78	1,72	0,04387	NM_005591	---
8165690	0,55	1,46	0,23286	-1,13	-2,18	0,00025	--- RHOBTB2	
8145227	0,55	1,46	0,12379	1,45	2,73	0,00151	NM_001160036	HACL1
8085608	0,54	1,46	0,11449	2,63	6,19	0,00017	NM_012260	ALKBH5
8005399	0,54	1,46	0,22684	2,22	4,64	0,00965	NM_017758	ANK2
8096959	0,54	1,46	0,26914	0,92	1,90	0,00454	NM_001148	CCNYL1
8047854	0,54	1,46	0,28243	1,59	3,00	0,00047	NM_001142300	LOC1720
8043036	0,54	1,46	0,27117	1,90	3,74	0,09673	NR_033423	BCL9L
7952116	0,54	1,46	0,06254	1,92	3,79	0,00091	NM_182557	B3GNT2
8042211	0,54	1,46	0,25987	1,09	2,13	0,01286	NM_006577	ZNF673
8166989	0,54	1,46	0,37283	1,82	3,53	0,00461	NM_001129898	STRN4
8037816	0,54	1,46	0,05581	2,56	5,90	0,00723	NM_013403	C11orf73
7942932	0,54	1,45	0,05418	0,92	1,90	0,02130	NM_016401	LRRC2
8086615	0,54	1,45	0,04247	-0,51	-1,42	0,01274	NM_024750	SUOX
7956097	0,54	1,45	0,26235	1,48	2,79	0,00182	NM_000456	ARL3
7936041	0,54	1,45	0,13244	-0,23	-1,17	0,29533	NM_004311	INPPL1
7942342	0,54	1,45	0,21757	0,66	1,58	0,05976	NM_001567	CARD8
8038029	0,54	1,45	0,11681	2,55	5,86	0,00020	NM_014959	ZNF440
8025958	0,54	1,45	0,03226	0,34	1,27	0,28538	NM_152357	FGFR1OP
8123342	0,54	1,45	0,18218	0,15	1,11	0,45463	NM_007045	ALG14
7917896	0,54	1,45	0,23117	1,60	3,02	0,03206	NM_144988	---
7946426	0,54	1,45	0,78732	-1,19	-2,28	0,36114	--- CCND3	
8126371	0,54	1,45	0,32899	1,22	2,32	0,18800	NM_001760	HNRNPUL2
7948814	0,54	1,45	0,27422	3,40	10,52	0,00016	NM_001079559	TOE1
7901091	0,54	1,45	0,33101	1,98	3,94	0,00032	NM_025077	SYT1
7957338	0,54	1,45	0,19350	2,41	5,30	0,00331	NM_005639	RPS19BP1
8076225	0,54	1,45	0,28009	0,51	1,42	0,18576	NM_194326	APOBEC3G
8073088	0,54	1,45	0,29174	-0,71	-1,63	0,02794	NM_021822	RNF185

8075477	0,54	1,45	0,35034	-1,28	-2,42	0,03106	NR_024209	GOLT1B
7954388	0,54	1,45	0,28347	1,55	2,93	0,00907	NM_016072	SLC29A3
7928208	0,54	1,45	0,36482	0,94	1,92	0,03130	NM_018344	PPCS
7900546	0,53	1,45	0,42933	1,98	3,94	0,00444	NM_024664	ZNF610
8030914	0,53	1,45	0,21258	-0,10	-1,07	0,48362	NM_001161425	RAB12
8020029	0,53	1,45	0,08881	0,71	1,64	0,00457	NM_001025300	HIST2H2BF
7919584	0,53	1,45	0,09589	0,43	1,35	0,03402	NM_001024599	ZNF326
7902891	0,53	1,45	0,22386	0,85	1,80	0,01992	NM_182976	C6orf167
8128329	0,53	1,45	0,28747	2,68	6,39	0,00283	NM_198468	C11orf82
7942832	0,53	1,45	0,11170	2,94	7,70	0,01005	NM_145018	KIAA0240
8119582	0,53	1,45	0,11222	1,04	2,06	0,00973	NM_015349	BNIP3
7973743	0,53	1,45	0,15941	0,29	1,22	0,58316	ENST00000355069	SYNJ2BP
7979916	0,53	1,45	0,06660	2,62	6,16	0,00321	NM_018373	RNASEH1
8050079	0,53	1,45	0,36726	2,12	4,36	0,01101	NM_002936	ZNF816A
8039017	0,53	1,45	0,41034	0,35	1,27	0,44094	NM_001031665	CPSF1
8153727	0,53	1,45	0,16517	1,43	2,70	0,02129	NM_013291	BRPF3
8119034	0,53	1,45	0,29905	2,88	7,35	0,00241	NM_015695	KIF1C
8004057	0,53	1,45	0,18987	1,85	3,60	0,07758	NM_006612	RBM41
8174340	0,53	1,45	0,31036	0,79	1,73	0,04931	NM_018301	CDRT1
8012951	0,53	1,45	0,61170	0,19	1,14	0,90528	NM_006382	QPRT
7994609	0,53	1,44	0,16420	-0,91	-1,88	0,09559	NM_014298	C1orf51
7905154	0,53	1,44	0,43208	-1,28	-2,43	0,04013	BC027999 SPNS1	
7994518	0,53	1,44	0,22185	1,52	2,86	0,02111	NM_032038	HIST1H3A
8117330	0,53	1,44	0,24360	1,00	2,00	0,03428	NM_003529	ARHGAP11A
7982358	0,53	1,44	0,09115	2,40	5,29	0,00163	NM_014783	C6orf35
8130988	0,53	1,44	0,39792	1,21	2,31	0,02239	NM_018452	NAAA
8101086	0,53	1,44	0,22526	3,59	12,01	0,00040	NM_014435	SUDS3
8110415	0,53	1,44	0,18148	1,26	2,39	0,01755	NM_022491	NTNG1
7903461	0,53	1,44	0,20099	-1,30	-2,46	0,00235	NM_001113226	ZRANB3
8055377	0,53	1,44	0,33921	2,07	4,19	0,00764	NM_032143	AURKA
8067167	0,53	1,44	0,08534	2,74	6,70	0,00073	NM_198433	TRERF1
8126402	0,53	1,44	0,18968	0,75	1,69	0,00109	NM_033502	DPH5
7918034	0,53	1,44	0,03273	1,11	2,16	0,06140	NM_001077394	---
8100555	0,53	1,44	0,06108	0,01	1,00	0,98091	--- C8orf48	
8144721	0,53	1,44	0,21564	-0,55	-1,47	0,03070	NM_001007090	---
8155265	0,53	1,44	0,24789	0,22	1,16	0,33206	--- SYPL2	
7903667	0,53	1,44	0,02944	-1,07	-2,10	0,00067	NM_001040709	CHSY1
7991581	0,53	1,44	0,10953	0,33	1,26	0,24539	NM_014918	RPL13AP6
7936320	0,53	1,44	0,07227	0,65	1,57	0,07831	NR_026715	FAM165B
8068375	0,53	1,44	0,37812	2,39	5,25	0,00126	NM_058182	ZKSCAN5
8134621	0,52	1,44	0,24303	1,28	2,43	0,00032	NM_014569	---
7898209	0,52	1,44	0,33956	0,19	1,14	0,58842	--- ---	
8169109	0,52	1,44	0,10314	0,97	1,95	0,01977	--- MCAM	
7952205	0,52	1,44	0,07815	0,94	1,92	0,03244	NM_006500	---
8113443	0,52	1,44	0,22992	-0,82	-1,77	0,09932	--- CAMKK2	
7967127	0,52	1,44	0,36182	2,11	4,33	0,01265	NM_006549	GTF2F2
7968915	0,52	1,44	0,02739	0,66	1,58	0,02539	NM_004128	C3orf19
8078033	0,52	1,44	0,16159	1,40	2,63	0,00725	NM_016474	RASL11A
7968236	0,52	1,44	0,07561	-0,56	-1,47	0,04316	NM_206827	DDAH2
8125048	0,52	1,44	0,10114	0,61	1,53	0,25954	NM_013974	ITPR2
7961900	0,52	1,44	0,31762	0,79	1,73	0,02594	NM_002223	TMEM200A
8122038	0,52	1,44	0,45761	-1,27	-2,41	0,00005	NM_052913	CLDN1
8092726	0,52	1,44	0,36958	4,72	26,31	0,00067	NM_021101	OPHN1
8173310	0,52	1,44	0,26667	0,12	1,09	0,78813	NM_002547	CHD2
7986250	0,52	1,44	0,19759	2,30	4,92	0,00599	NM_001271	DNAJC25
8157203	0,52	1,44	0,16683	1,23	2,35	0,00245	NM_001015882	MAF1
8148737	0,52	1,43	0,11349	1,29	2,45	0,01013	NM_032272	LOC147727
8034097	0,52	1,43	0,36642	1,22	2,33	0,03064	NR_024333	LMLN
8085033	0,52	1,43	0,14839	2,08	4,23	0,00007	NM_001136049	MTRF1L
8130383	0,52	1,43	0,14942	1,07	2,09	0,00164	NM_019041	KIAA1324
7903592	0,52	1,43	0,10606	0,38	1,30	0,20160	NM_020775	RSBN1L
8133809	0,52	1,43	0,05424	0,62	1,53	0,04686	NM_198467	SMPD2
8121418	0,52	1,43	0,15260	2,38	5,19	0,00257	NM_003080	WDR35
8050507	0,52	1,43	0,19204	2,20	4,59	0,00644	NM_001006657	TRMT2B
8174005	0,52	1,43	0,26443	2,00	4,00	0,00021	NM_024917	---
8155451	0,52	1,43	0,31186	-0,86	-1,81	0,06193	--- ---	
8161556	0,52	1,43	0,31186	-0,86	-1,81	0,06193	--- SFMBT1	
8088065	0,52	1,43	0,39017	2,58	5,97	0,00010	NM_001005159	NOVA1
7978391	0,52	1,43	0,10248	0,82	1,77	0,03858	NM_002515	FAM160A2
7946128	0,52	1,43	0,08195	2,25	4,76	0,00173	NM_032127	ERI1
8144516	0,52	1,43	0,31716	1,28	2,43	0,04609	NM_153332	MOAP1
7980983	0,52	1,43	0,42836	1,20	2,29	0,00043	NM_022151	SST
8092682	0,52	1,43	0,34753	-1,66	-3,17	0,00622	NM_001048	ATP5J

8069633	0,52	1,43	0,37618	0,31	1,24	0,40207	NM_001003703	ARHGEF6
8175393	0,52	1,43	0,05505	-0,08	-1,06	0,70756	NM_004840	TSR2
8167790	0,52	1,43	0,42795	1,75	3,37	0,00517	NM_058163	HIST2H2BE
7919637	0,52	1,43	0,14621	2,70	6,51	0,01173	NM_003528	HIST1H2AE
8117408	0,52	1,43	0,03220	0,24	1,18	0,23429	NM_021052	TNXA
8125289	0,52	1,43	0,32255	0,03	1,02	0,95204	NR_001284	RFX5
7919971	0,52	1,43	0,11432	3,27	9,62	0,01463	NM_000449	FBXO9
8120251	0,52	1,43	0,11275	3,01	8,04	0,00003	NM_033480	NAT9
8018209	0,52	1,43	0,29529	1,43	2,70	0,00618	NM_015654	UBTD2
8115806	0,52	1,43	0,50856	2,81	7,01	0,00037	NM_152277	EGLN1
7925048	0,52	1,43	0,27130	0,52	1,43	0,32547	NM_022051	FBXL21
8108205	0,52	1,43	0,09976	-0,90	-1,87	0,00103	NM_012159	ADAMTS2
8116272	0,52	1,43	0,06367	-1,04	-2,06	0,01149	NM_014244	DNAJC9
8064976	0,51	1,43	0,33561	1,67	3,17	0,00445	NM_015190	NDUFS3
7939825	0,51	1,43	0,32535	1,07	2,11	0,25414	NM_004551	TCP11L1
7939184	0,51	1,43	0,26993	1,32	2,50	0,00031	NM_018393	AK3L1
7902038	0,51	1,43	0,64435	-1,02	-2,03	0,41502	NM_001005353	MSTO1
7906021	0,51	1,43	0,10694	1,33	2,52	0,00203	NM_018116	C17orf61
8012207	0,51	1,43	0,28109	2,94	7,65	0,00023	BC030270 LCA5	
8127756	0,51	1,43	0,27847	0,14	1,10	0,59987	NM_181714	MRPL14
8126653	0,51	1,43	0,28879	1,31	2,48	0,00255	NM_032111	TESK1
8155062	0,51	1,43	0,26150	1,29	2,45	0,07602	NM_006285	MTSS1
8152764	0,51	1,43	0,17200	0,58	1,49	0,00572	NM_014751	CDC42EP1
8072817	0,51	1,42	0,10890	1,42	2,67	0,04398	NM_152243	---
7901856	0,51	1,42	0,34753	0,72	1,65	0,01627	--- CDR2	
8000236	0,51	1,42	0,10594	0,33	1,26	0,28390	NM_001802	SLC25A26
8080896	0,51	1,42	0,38995	3,36	10,24	0,00000	NR_028475	FCGRT
8030374	0,51	1,42	0,41166	0,66	1,58	0,23721	NM_004107	BRPF1
8077595	0,51	1,42	0,16423	2,24	4,73	0,02589	NM_001003694	---
8115847	0,51	1,42	0,67357	-0,14	-1,10	0,88688	--- ---	
8050350	0,51	1,42	0,61089	-1,36	-2,58	0,14504	--- FAM100A	
7999173	0,51	1,42	0,55947	2,28	4,85	0,06503	NM_145253	PCCB
8082886	0,51	1,42	0,29833	2,39	5,25	0,00061	NM_000532	C20orf199
8063337	0,51	1,42	0,38622	1,86	3,62	0,00942	NR_003605	DEPDC1
7916898	0,51	1,42	0,16828	1,80	3,49	0,03260	NM_001114120	DTNB
8050846	0,51	1,42	0,33455	1,29	2,44	0,00003	NM_021907	RPS21
8063903	0,51	1,42	0,32219	0,56	1,47	0,13774	NM_001024	---
7896752	0,51	1,42	0,67512	-3,05	-8,29	0,00470	--- ---	
8165667	0,51	1,42	0,67512	-3,05	-8,29	0,00470	--- HIATL1	
8162490	0,51	1,42	0,15357	0,06	1,04	0,90234	NM_032558	C14orf79
7977331	0,51	1,42	0,31157	1,13	2,19	0,02465	NM_174891	OXSM
8078300	0,51	1,42	0,21399	2,75	6,71	0,00062	NM_017897	TNFSF4
7922343	0,51	1,42	0,20784	-0,29	-1,22	0,14971	NM_003326	FANCD2
8077731	0,51	1,42	0,15790	2,11	4,31	0,00158	NM_033084	PRKCH
7974835	0,51	1,42	0,12186	3,22	9,32	0,00002	NM_006255	PPIAL4A
7904997	0,50	1,42	0,55306	-0,42	-1,34	0,21405	NM_178230	PPIAL4A
7905016	0,50	1,42	0,55306	-0,42	-1,34	0,21405	NM_178230	OSGEPL1
8057689	0,50	1,42	0,11065	1,99	3,96	0,00100	NM_022353	ACAA2
8023261	0,50	1,42	0,38317	0,61	1,53	0,03949	NM_006111	TTBK2
7987960	0,50	1,42	0,20178	1,81	3,49	0,00039	NM_173500	FAM122A
8155696	0,50	1,42	0,41057	1,64	3,11	0,06467	NM_138333	MGC87042
8138527	0,50	1,42	0,29822	2,21	4,64	0,00222	NM_207342	SENP7
8089203	0,50	1,42	0,21898	0,96	1,95	0,14188	NM_020654	ZNF567
8028200	0,50	1,42	0,16634	0,92	1,89	0,02478	NM_152603	CRAT
8164535	0,50	1,42	0,01895	1,30	2,46	0,09825	NM_000755	HECTD3
7915659	0,50	1,42	0,21906	2,17	4,50	0,00930	NM_024602	C6orf106
8125850	0,50	1,41	0,23973	1,60	3,04	0,00381	NM_024294	C12orf5
7953211	0,50	1,41	0,40448	1,89	3,71	0,00053	NM_020375	CALCRL
8057578	0,50	1,41	0,09683	0,65	1,57	0,08351	NM_005795	HSPE1
8047223	0,50	1,41	0,02729	0,07	1,05	0,50475	NM_002157	C12orf29
7957467	0,50	1,41	0,39457	1,26	2,40	0,02926	NM_001009894	LSM7
8032480	0,50	1,41	0,41313	0,58	1,50	0,06613	NM_016199	ZNF578
8030944	0,50	1,41	0,06952	-0,67	-1,59	0,09772	NM_001099694	CLPTM1L
8110803	0,50	1,41	0,37404	3,90	14,96	0,00005	NM_030782	KRTAP1-1
8015196	0,50	1,41	0,10177	-0,98	-1,97	0,05531	NM_030967	KRTAP1-1
8019578	0,50	1,41	0,10177	-0,98	-1,97	0,05531	NM_030967	FANCF
7947138	0,50	1,41	0,46574	-0,33	-1,26	0,08191	NM_022725	ADAM32
8146024	0,50	1,41	0,06947	-0,06	-1,04	0,81886	NM_145004	RBMX2
8169920	0,50	1,41	0,24025	1,24	2,35	0,09366	NM_016024	ARHGEF18
8025142	0,50	1,41	0,12353	0,68	1,60	0,27713	NM_001130955	C1orf151
7898574	0,50	1,41	0,17262	0,40	1,32	0,30615	NM_001032363	RSPH3
8130528	0,50	1,41	0,24527	0,71	1,63	0,13770	NM_031924	DEXI
7999419	0,50	1,41	0,50656	3,67	12,69	0,00010	NM_014015	AAMP

8058914	0,50	1,41	0,16580	1,95	3,87	0,00398	NM_001087	UNC13B
8154981	0,50	1,41	0,14552	2,29	4,90	0,00010	NM_006377	PRELP
7908924	0,50	1,41	0,11785	-2,19	-4,55	0,00696	NM_002725	NHLRC3
7968703	0,50	1,41	0,37610	2,71	6,56	0,00030	NM_001012754	RUNX2
8120043	0,50	1,41	0,17617	0,14	1,10	0,40667	NM_001024630	SAAL1
7946957	0,50	1,41	0,19110	2,35	5,09	0,00037	NM_138421	F8
8176193	0,50	1,41	0,27736	-0,21	-1,16	0,39129	NM_000132	PPIG
8046169	0,50	1,41	0,23515	0,63	1,55	0,13214	NM_004792	MTPAP
7932834	0,50	1,41	0,02627	0,69	1,61	0,01225	NM_018109	ABCA6
8017964	0,50	1,41	0,11261	-1,05	-2,07	0,00330	NM_080284	FAM86C
7942279	0,50	1,41	0,29559	1,26	2,40	0,00237	NM_018172	NAA40
7940824	0,50	1,41	0,41968	2,77	6,84	0,00005	NM_024771	PTGFR
7902527	0,50	1,41	0,34376	-0,50	-1,41	0,01603	NM_001039585	CDK10
7997982	0,50	1,41	0,26310	2,65	6,28	0,00005	NM_052988	DEPDC1B
8112260	0,50	1,41	0,07919	2,98	7,90	0,00014	NM_018369	---
8112180	0,50	1,41	0,17951	-1,36	-2,57	0,00005	---	TMEM130
8141228	0,49	1,41	0,14669	-0,45	-1,37	0,30031	NM_001134450	---
8165658	0,49	1,41	0,55807	-2,99	-7,95	0,00745	---	TRIM2
8097841	0,49	1,41	0,25325	1,32	2,49	0,03200	NM_015271	RNF138
8020814	0,49	1,41	0,01914	2,31	4,97	0,00036	NM_016271	N4BP1
8001317	0,49	1,41	0,13631	1,29	2,44	0,01754	NM_153029	ZNF271
8020898	0,49	1,41	0,06353	0,88	1,84	0,01418	NR_024565	FBXL7
8104592	0,49	1,41	0,04648	-2,00	-4,00	0,00049	NM_012304	RSAD2
8040080	0,49	1,41	0,36418	-1,20	-2,30	0,00230	NM_080657	CAPN3
7983054	0,49	1,41	0,16404	0,58	1,50	0,00158	NR_027911	FAM174A
8107119	0,49	1,41	0,07999	1,04	2,06	0,03222	NM_198507	GLOD4
8010949	0,49	1,41	0,19154	2,25	4,77	0,00005	NM_016080	ANKLE2
7967794	0,49	1,41	0,30393	1,83	3,56	0,00495	NM_015114	---
8012218	0,49	1,41	0,84267	-3,07	-8,38	0,02580	---	CHCHD1
7928401	0,49	1,41	0,51875	3,23	9,40	0,00009	NM_203298	DCLRE1A
7936408	0,49	1,41	0,25681	2,74	6,70	0,00063	NM_014881	C2orf60
8058108	0,49	1,41	0,22913	2,03	4,07	0,00673	NR_004862	STX5
7948864	0,49	1,41	0,45717	1,81	3,50	0,00244	NM_003164	RPS2
8013348	0,49	1,41	0,42973	1,94	3,83	0,03100	NM_002952	ZNF594
8011823	0,49	1,41	0,51468	2,19	4,57	0,00250	NM_032530	OAZ2
7989661	0,49	1,41	0,03266	1,08	2,12	0,02925	NM_002537	PPP1R1C
8046746	0,49	1,41	0,30831	1,65	3,15	0,01895	NM_001080545	BCL7A
7959354	0,49	1,41	0,28538	2,08	4,23	0,00687	NM_020993	GSTM1
7903765	0,49	1,41	0,46296	2,25	4,77	0,00086	NM_000561	C3orf58
8083223	0,49	1,40	0,22482	2,05	4,15	0,00752	NM_173552	AMACR
8111430	0,49	1,40	0,32839	1,98	3,95	0,00124	NM_014324	ZNF841
8038949	0,49	1,40	0,34510	0,80	1,74	0,08196	NM_001136499	KANK2
8034130	0,49	1,40	0,04113	0,66	1,58	0,17151	NM_001136191	EDF1
8165309	0,49	1,40	0,32486	2,40	5,29	0,00067	NM_003792	C6orf192
8129649	0,49	1,40	0,11058	3,45	10,90	0,00028	NM_052831	---
8174251	0,49	1,40	0,29327	0,21	1,15	0,47249	---	ZNF577
8038904	0,49	1,40	0,18426	-0,60	-1,51	0,03564	NM_032679	---
7955979	0,49	1,40	0,45563	-1,56	-2,94	0,07104	---	SNX29
7993281	0,49	1,40	0,11082	0,94	1,91	0,01976	NM_001080530	DUSP19
8046792	0,49	1,40	0,11912	-0,20	-1,15	0,37438	NM_080876	RAB24
8115997	0,49	1,40	0,09436	0,55	1,46	0,39887	NM_001031677	C15orf51
7991512	0,49	1,40	0,49384	-1,82	-3,52	0,01716	NR_003260	PALM2-AKAP2
8157153	0,48	1,40	0,27721	1,16	2,23	0,02037	NM_007203	SF3B4
7919659	0,48	1,40	0,15162	1,58	2,98	0,00010	NM_005850	IL7
8151447	0,48	1,40	0,21885	-1,61	-3,06	0,00082	NM_000880	---
7974402	0,48	1,40	0,15522	0,36	1,29	0,03901	---	SYNJ2
8123006	0,48	1,40	0,29132	1,23	2,35	0,00064	NM_003898	ZNF140
7960150	0,48	1,40	0,20938	2,48	5,57	0,00311	NM_003440	IFIH1
8056285	0,48	1,40	0,18670	-0,07	-1,05	0,65091	NM_022168	DDX10
7943690	0,48	1,40	0,14260	2,58	5,99	0,00170	NM_004398	CCDC146
8133770	0,48	1,40	0,23108	1,38	2,61	0,01453	NM_020879	ADO
7927767	0,48	1,40	0,09932	0,81	1,76	0,00723	NM_032804	NAGA
8076403	0,48	1,40	0,46581	1,90	3,72	0,01064	NM_000262	CDC25A
8086880	0,48	1,40	0,07750	1,54	2,92	0,00035	NM_001789	GPR146
8131067	0,48	1,40	0,04152	0,52	1,43	0,07867	NM_138445	AMY2A
7903414	0,48	1,40	0,59629	-1,25	-2,37	0,02412	NM_000699	LOC389834 // LOC389834 // LOC389834 // LOC389834
8104139	0,48	1,39	0,00084	0,07	1,05	0,68815	NR_027420 // NR_027420 // NR_027420 // NR_027420	MLLT4
8123407	0,48	1,39	0,04222	2,89	7,40	0,00171	NM_001040001	BID
8074261	0,48	1,39	0,24004	1,95	3,86	0,00343	NM_197966	FAM96A
7989611	0,48	1,39	0,40918	1,93	3,81	0,00774	NM_032231	RBM45
8046628	0,48	1,39	0,37916	1,06	2,09	0,03455	NM_152945	TSC22D4

8141526	0,48	1,39	0,02852	-0,54	-1,45	0,26260	NM_030935	ADORA2B
8005134	0,48	1,39	0,44689	1,16	2,23	0,01283	NM_000676	NDUFV1
7941946	0,48	1,39	0,09889	1,93	3,80	0,00775	NM_007103	MTHFD1L
8122773	0,48	1,39	0,36281	1,68	3,20	0,01840	NM_015440	GMNN
8117225	0,48	1,39	0,01650	2,82	7,04	0,00342	NM_015895	LOC401397
8142431	0,48	1,39	0,31272	0,81	1,75	0,00502	NR_024412	IGFBP2
8048205	0,48	1,39	0,39284	-0,61	-1,53	0,30164	NM_000597	CCDC124
8026895	0,48	1,39	0,20035	2,48	5,58	0,00712	NM_138442	TMEM25
7944275	0,48	1,39	0,42686	1,19	2,27	0,04619	NM_032780	COX18
8100893	0,48	1,39	0,51694	1,83	3,56	0,00028	NM_173827	SLC12A4
8002152	0,48	1,39	0,06397	1,79	3,46	0,00852	NM_005072	FGFR1OP2
7954492	0,48	1,39	0,16818	0,89	1,85	0,00172	NM_015633	CNNM2
7930194	0,48	1,39	0,04679	1,66	3,16	0,00015	NM_017649	DCTPP1
8000884	0,48	1,39	0,18339	2,60	6,06	0,01029	NM_024096	LARS2
8079311	0,48	1,39	0,43361	1,40	2,64	0,00347	NM_015340	CHST10
8054297	0,48	1,39	0,06412	1,56	2,94	0,00047	NM_004854	C14orf169
7975595	0,47	1,39	0,23641	0,71	1,64	0,06713	NM_024644	FMO3
7907249	0,47	1,39	0,14076	-1,32	-2,50	0,00473	NM_006894	YWHAQP8
8174026	0,47	1,39	0,16447	1,96	3,88	0,01979	DQ145510	TRIM28
8031913	0,47	1,39	0,33087	3,15	8,87	0,00047	NM_005762	---
7922754	0,47	1,39	0,21038	0,68	1,60	0,00658	---	CHPF2
8137404	0,47	1,39	0,38617	1,36	2,57	0,00821	NM_019015	UROD
7901073	0,47	1,39	0,27079	1,36	2,57	0,01490	NM_000374	SSRP1
7948192	0,47	1,39	0,26654	3,27	9,61	0,00015	NM_003146	SCYL3
7907183	0,47	1,39	0,23303	1,05	2,07	0,07220	NM_181093	KAT2A
8015526	0,47	1,39	0,18821	2,64	6,22	0,00463	NM_021078	ULK4
8086352	0,47	1,39	0,16108	-0,01	-1,01	0,94818	NM_017886	C1orf201
7913787	0,47	1,39	0,16251	1,13	2,18	0,00909	BC063891	RUNX1T1
8151768	0,47	1,39	0,23932	-1,23	-2,34	0,00449	NM_175634	FIGNL1
8139632	0,47	1,39	0,17766	1,70	3,26	0,02036	NM_001042762	DMAP1
7900962	0,47	1,39	0,19477	1,43	2,69	0,00330	NM_019100	FBXO27
8036689	0,47	1,39	0,42512	4,02	16,22	0,00002	NM_178820	---
8071804	0,47	1,39	0,53898	0,18	1,13	0,78126	---	CDK5RAP3
8008074	0,47	1,39	0,09957	0,94	1,91	0,09058	NM_176096	---
8151709	0,47	1,39	0,43424	-0,76	-1,70	0,21077	---	STRADA
8017402	0,47	1,38	0,36022	1,05	2,08	0,02006	NM_153335	METTL10
7936891	0,47	1,38	0,38117	2,75	6,74	0,00600	NM_212554	ZC3H6
8044450	0,47	1,38	0,41488	1,72	3,29	0,00103	NM_198581	CABLES2
8067495	0,47	1,38	0,41927	2,64	6,24	0,00265	NM_031215	ZNF658
8161326	0,47	1,38	0,00298	0,29	1,22	0,35315	NM_033160	LRTOMT
7942315	0,47	1,38	0,22344	-0,24	-1,18	0,52192	NM_001145309	VPS53
8010918	0,47	1,38	0,18382	0,82	1,76	0,00722	NM_001128159	C3orf62
8087374	0,47	1,38	0,19313	1,74	3,34	0,00010	NM_198562	ZNF394
8141305	0,47	1,38	0,37164	0,56	1,47	0,14292	NM_032164	PUS10
8052418	0,47	1,38	0,14174	-0,13	-1,10	0,72012	NM_144709	MIDN
8024228	0,47	1,38	0,40321	2,17	4,51	0,00408	NM_177401	PTPDC1
8156506	0,47	1,38	0,20544	2,74	6,68	0,00136	NM_152422	KIAA1377
7943376	0,47	1,38	0,03076	-0,34	-1,26	0,12371	NM_020802	LOXL4
7935553	0,47	1,38	0,01800	-0,21	-1,15	0,47989	NM_032211	CUX1
8135114	0,47	1,38	0,06009	0,78	1,72	0,00251	NM_181552	---
8098506	0,47	1,38	0,13105	0,77	1,71	0,00280	---	KAL1
8171248	0,47	1,38	0,11628	0,83	1,77	0,01991	NM_000216	PROSC
8145854	0,47	1,38	0,16656	1,67	3,19	0,00045	NM_007198	ESF1
8065032	0,46	1,38	0,01319	1,03	2,04	0,05718	NM_016649	NPIPL3
7994026	0,46	1,38	0,66441	1,08	2,11	0,00496	NM_130464	CCDC126
8131871	0,46	1,38	0,42616	1,69	3,23	0,00454	NM_138771	ACO2
8073430	0,46	1,38	0,46380	2,19	4,57	0,00215	NM_001098	H2AFZ
8101945	0,46	1,38	0,08499	1,97	3,91	0,00855	NM_002106	DUT
7983594	0,46	1,38	0,12713	1,17	2,24	0,00855	NM_001025248	TET2
8096669	0,46	1,38	0,13326	0,13	1,09	0,59497	NM_017628	ARRB2
8003903	0,46	1,38	0,24712	2,86	7,26	0,00134	NM_004313	NUDT4
7957536	0,46	1,38	0,41692	-0,75	-1,68	0,00426	NM_019094	ABT1
8117522	0,46	1,38	0,38273	1,57	2,97	0,02007	NM_013375	FAM133B
8055978	0,46	1,38	0,20590	0,38	1,30	0,02300	NM_152789	PRPF3
7905171	0,46	1,38	0,06443	2,58	5,98	0,00332	NM_004698	BMS1P1
7933290	0,46	1,38	0,27784	2,05	4,13	0,00018	NR_026566	FGF1
8114805	0,46	1,38	0,26103	-0,98	-1,98	0,02202	NM_000800	GDPD1
8008802	0,46	1,38	0,18898	1,47	2,77	0,00506	NM_182569	RGNEF
8106210	0,46	1,38	0,15405	2,15	4,45	0,00131	NM_001080479	NPAS3
7973875	0,46	1,38	0,10053	-2,15	-4,45	0,00040	NM_001164749	---
7979694	0,46	1,38	0,35891	0,15	1,11	0,44775	---	PLXND1
8090591	0,46	1,38	0,22386	1,11	2,15	0,05129	NM_015103	SNORA70
8170863	0,46	1,38	0,28322	4,63	24,75	0,00001	NR_000011	---

8104625	0,46	1,38	0,05385	0,75	1,68	0,03313	---	HSPA1B
8118314	0,46	1,38	0,16055	1,88	3,69	0,03988	NM_005346	APTX
8160602	0,46	1,37	0,26881	1,95	3,88	0,00159	NM_175069	ZNF658
8161346	0,46	1,37	0,00461	0,42	1,34	0,21728	NM_033160	TMPO
7957737	0,46	1,37	0,36925	2,56	5,90	0,00031	NM_001032283	ZNF35
8079217	0,46	1,37	0,20480	0,05	1,04	0,85408	NM_003420	STX16
8063636	0,46	1,37	0,33302	1,44	2,71	0,01834	NM_001001433	SIPA1L2
7925062	0,46	1,37	0,02125	0,48	1,39	0,25462	NM_020808	C17orf42
8014047	0,46	1,37	0,11893	0,87	1,83	0,10833	NM_024683	---
7924893	0,46	1,37	0,51161	-0,44	-1,36	0,05228	---	LOC100289668
7986515	0,46	1,37	0,85197	-1,27	-2,41	0,34210	ENST00000417911	LOC100289668
7986525	0,46	1,37	0,85197	-1,27	-2,41	0,34210	ENST00000417911	PYGO2
7920594	0,46	1,37	0,01928	2,27	4,81	0,00475	NM_138300	ANKRD26
7932637	0,46	1,37	0,08913	0,13	1,10	0,48981	NM_014915	BAIAP2
8010562	0,46	1,37	0,41580	0,98	1,97	0,05510	NM_006340	CRYL1
7970455	0,45	1,37	0,23538	0,72	1,65	0,00948	NM_015974	SLC2A13
7962327	0,45	1,37	0,03956	0,62	1,54	0,10333	NM_052885	NPIPL3
8000676	0,45	1,37	0,65853	1,00	2,00	0,00745	NM_130464	SYTL5
8166747	0,45	1,37	0,19390	-0,36	-1,28	0,08048	NM_138780	ABCA5
8018038	0,45	1,37	0,20082	0,00	1,00	0,99414	NM_018672	ARHGAP31
8081838	0,45	1,37	0,29804	-0,84	-1,79	0,06067	NM_020754	PDXDC2
7997239	0,45	1,37	0,59246	1,46	2,75	0,01126	NR_003610	MARK4
8029642	0,45	1,37	0,21031	1,22	2,33	0,02859	NM_031417	ZNF501
8079229	0,45	1,37	0,36357	0,31	1,24	0,49067	NM_145044	C9orf102
8156581	0,45	1,37	0,29473	2,47	5,53	0,00031	NM_001010895	ERCC1
8037579	0,45	1,37	0,29802	0,96	1,94	0,00157	NM_202001	DDX50
7927926	0,45	1,37	0,50806	-0,25	-1,19	0,78007	NM_024045	IDH3B
8064522	0,45	1,37	0,10691	1,95	3,87	0,01176	NM_174856	AMPD3
7938396	0,45	1,37	0,33203	-0,10	-1,07	0,68865	NM_000480	POLR3E
7993973	0,45	1,37	0,13914	2,07	4,19	0,00065	NM_018119	HSPA1B
8178086	0,45	1,37	0,20886	1,71	3,28	0,04410	NM_005346	HSPA1B
8179324	0,45	1,37	0,20886	1,71	3,28	0,04410	NM_005346	ETHE1
8037322	0,45	1,36	0,37938	1,46	2,75	0,01600	NM_014297	---
8125123	0,45	1,36	0,79857	-0,93	-1,91	0,07071	---	RPS12
8122136	0,45	1,36	0,11294	-0,21	-1,15	0,50792	NM_001016	RAE1
8063566	0,45	1,36	0,51300	2,34	5,07	0,00039	NM_003610	PHF11
7969129	0,45	1,36	0,40379	1,09	2,13	0,01086	NM_001040443	MRPS33
8143433	0,45	1,36	0,28053	0,42	1,34	0,12848	NM_016071	LYPD1
8055314	0,45	1,36	0,13136	-0,62	-1,54	0,15321	NM_144586	LCORL
8099581	0,45	1,36	0,11467	1,01	2,02	0,02572	NM_153686	RNU5E
7897801	0,45	1,36	0,14710	0,73	1,66	0,08257	NR_002754	FBXO45
8084947	0,45	1,36	0,28012	1,64	3,12	0,00046	NM_001105573	TMEM218
7952484	0,44	1,36	0,44365	2,73	6,66	0,00226	NM_001080546	NPIPL3
8000205	0,44	1,36	0,66463	0,86	1,82	0,00959	NM_130464	KCTD11
8004360	0,44	1,36	0,12156	0,34	1,27	0,45345	NM_001002914	C1GALT1C1
8174820	0,44	1,36	0,47470	0,08	1,06	0,72909	NM_152692	SPDYE7P
8139943	0,44	1,36	0,50175	-0,37	-1,29	0,52574	NR_003666	SLC35E1
8035177	0,44	1,36	0,06652	2,91	7,52	0,00108	NM_024881	---
7985930	0,44	1,36	0,42753	-1,26	-2,40	0,02804	---	RPGRIP1L
8001423	0,44	1,36	0,47853	0,39	1,31	0,06171	NM_015272	SLC25A44
7906128	0,44	1,36	0,10419	1,53	2,89	0,00012	NM_014655	IKZF2
8058670	0,44	1,36	0,23756	-0,08	-1,06	0,75071	NM_016260	CRCP
8133145	0,44	1,36	0,21456	0,61	1,53	0,00607	NM_014478	ADRB3
8150249	0,44	1,36	0,16218	-1,42	-2,68	0,01195	NM_000025	TGDS
7972269	0,44	1,36	0,20428	0,48	1,40	0,01836	NM_014305	TTC1
8109620	0,44	1,36	0,15569	-0,04	-1,03	0,91284	NM_003314	KDM5C
8172827	0,44	1,36	0,03371	2,17	4,49	0,00070	NM_004187	DIDO1
8067563	0,44	1,36	0,37188	0,64	1,56	0,21028	NM_033081	DLC1
8149413	0,44	1,36	0,23647	-0,33	-1,25	0,05234	NM_182643	DNM3
7907370	0,44	1,36	0,13434	0,24	1,18	0,37091	NM_015569	PSMG4
8116655	0,44	1,36	0,13391	0,53	1,45	0,22044	NM_001128592	---
8078378	0,44	1,36	0,10440	0,17	1,13	0,55439	---	ZC3HC1
8142899	0,44	1,36	0,29117	1,42	2,68	0,02496	NM_016478	SELPLG
7966127	0,44	1,35	0,12122	1,69	3,22	0,00174	NM_003006	CDK5
8143850	0,44	1,35	0,17328	1,85	3,61	0,00049	NM_004935	C18orf25
8021113	0,44	1,35	0,35543	0,35	1,28	0,49808	NM_145055	FRAS1
8095907	0,44	1,35	0,13026	2,61	6,10	0,00039	NM_025074	TNRC6C
8010188	0,44	1,35	0,21435	1,60	3,03	0,00073	NM_001142640	AKIRIN1
7900216	0,44	1,35	0,20031	0,30	1,23	0,21850	NM_024595	---
8071819	0,44	1,35	0,43742	-0,17	-1,12	0,60043	---	PLXNC1
7957570	0,44	1,35	0,04276	-0,16	-1,12	0,66605	NM_005761	TAF9
8112458	0,44	1,35	0,06809	1,26	2,40	0,00892	NM_003187	DHTKD1
7926170	0,43	1,35	0,45925	2,17	4,50	0,00254	NM_018706	IMP3

7990540	0,43	1,35	0,38661	0,30	1,23	0,51103	NM_018285	RTTN
8023766	0,43	1,35	0,39859	1,41	2,65	0,00435	NM_173630	PRKCDBP
7946142	0,43	1,35	0,56641	-0,06	-1,04	0,80821	NM_145040	TMED10P1
8148949	0,43	1,35	0,33739	1,08	2,12	0,02440	NR_002807	BYSL
8119492	0,43	1,35	0,11852	1,53	2,90	0,00052	NM_004053	---
7932794	0,43	1,35	0,24648	-0,91	-1,88	0,28113	---	TBX5
7966668	0,43	1,35	0,29554	-1,64	-3,11	0,04908	NM_181486	TXNRD3
8090395	0,43	1,35	0,26477	1,06	2,09	0,00635	NM_052883	---
8117576	0,43	1,35	0,09282	0,40	1,32	0,18542	---	CDK9
8158112	0,43	1,35	0,21877	3,09	8,50	0,00329	NM_001261	AVPI1
7935521	0,43	1,35	0,31755	2,51	5,69	0,01412	NM_021732	ZNF230
8029347	0,43	1,35	0,04745	0,48	1,39	0,15290	NM_006300	---
8150149	0,43	1,35	0,51035	-0,14	-1,10	0,62454	---	TPI1
7953508	0,43	1,35	0,63964	1,23	2,34	0,16192	NM_000365	ABCC3
8008454	0,43	1,35	0,21218	3,99	15,85	0,00008	NM_003786	DDX49
8027100	0,43	1,35	0,29698	-0,07	-1,05	0,84070	NM_019070	TPX2
8061579	0,43	1,35	0,43311	2,69	6,44	0,00105	NM_012112	GALNTL2
8078155	0,43	1,35	0,03306	-0,47	-1,39	0,04344	NM_054110	NPIP
7999766	0,43	1,35	0,68875	-0,33	-1,26	0,26520	NM_006985	UPF0639
7975354	0,43	1,35	0,52552	-0,31	-1,24	0,22092	NM_001161498	LIN37
8028004	0,43	1,35	0,38194	1,28	2,42	0,02118	NM_019104	ZBTB6
8163999	0,43	1,34	0,10572	2,00	4,00	0,00202	NM_006626	RAB11FIP1
8150225	0,43	1,34	0,09693	0,34	1,26	0,34459	NM_001002814	RBM25
7975521	0,43	1,34	0,28978	0,74	1,68	0,01255	NM_021239	FAM59A
8022803	0,43	1,34	0,38216	0,82	1,77	0,00072	NM_022751	LDLRAD3
7939376	0,43	1,34	0,33823	1,16	2,24	0,06276	NM_174902	AMY1A
7903425	0,42	1,34	0,54571	-0,99	-1,99	0,02784	NM_004038	AMY1A
7903440	0,42	1,34	0,54571	-0,99	-1,99	0,02784	NM_004038	AMY1A
7918134	0,42	1,34	0,54571	-0,99	-1,99	0,02784	NM_004038	ACP6
7919326	0,42	1,34	0,22843	2,69	6,46	0,00037	NM_016361	DPH2
7900911	0,42	1,34	0,11594	1,32	2,50	0,08855	NM_001384	KLRAQ1
8041913	0,42	1,34	0,02764	1,55	2,92	0,00009	NR_024188	CCDC3
7932082	0,42	1,34	0,21312	0,64	1,55	0,13836	NM_031455	LOC147804
8030991	0,42	1,34	0,66021	2,17	4,51	0,02517	NR_003148	ARHGAP32
7952641	0,42	1,34	0,22367	2,67	6,38	0,00024	NM_001142685	DHX58
8015511	0,42	1,34	0,53208	-1,15	-2,21	0,07409	NM_024119	TCEANC
8166096	0,42	1,34	0,40915	-0,42	-1,33	0,31912	NM_152634	GCH1
7979269	0,42	1,34	0,42977	1,06	2,08	0,00375	NM_000161	ANGEL1
7980327	0,42	1,34	0,52057	2,65	6,26	0,00139	NM_015305	TUBGCP2
7937175	0,42	1,34	0,04600	2,19	4,56	0,00095	NM_006659	DAGLB
8138091	0,42	1,34	0,02796	2,31	4,97	0,00364	NM_139179	TBX3
7966690	0,42	1,34	0,23707	-0,37	-1,29	0,30977	NM_016569	TCEB2
7998852	0,42	1,34	0,07497	1,71	3,28	0,00348	NM_007108	ODF2L
7917433	0,42	1,34	0,32134	0,79	1,73	0,05412	NM_020729	ZNF658
8161319	0,42	1,34	0,00429	0,41	1,33	0,21670	NM_033160	COG7
8000263	0,42	1,34	0,51196	1,99	3,98	0,01734	NM_153603	AMIGO2
7962579	0,42	1,34	0,19298	2,25	4,75	0,00636	NM_001143668	PRMT3
7938890	0,42	1,34	0,48465	1,91	3,77	0,00029	NM_005788	NBPF16
7919438	0,42	1,34	0,74127	-0,31	-1,24	0,32175	NM_001102663	RPL37A
8048195	0,42	1,34	0,11864	1,17	2,25	0,00595	NM_000998	NR1H2
8030630	0,42	1,34	0,35589	2,11	4,32	0,01205	NM_007121	NCAM2
8067985	0,42	1,34	0,33324	-0,59	-1,51	0,00114	NM_004540	---
7905481	0,42	1,34	0,29312	0,55	1,47	0,51793	---	ZNF285
8037459	0,42	1,34	0,40922	0,40	1,32	0,36148	NM_152354	PRCC
7906235	0,42	1,34	0,28880	2,10	4,29	0,00385	NM_005973	CISD2
8096663	0,42	1,33	0,32905	2,80	6,97	0,00686	NM_001008388	MYOC
7922301	0,42	1,33	0,44685	-0,41	-1,33	0,19930	NM_000261	PLCD3
8016168	0,42	1,33	0,29588	1,62	3,07	0,01791	NM_133373	MCAT
8076563	0,42	1,33	0,19363	2,31	4,96	0,00187	NM_173467	ATP9A
8067055	0,41	1,33	0,31735	1,79	3,46	0,00084	NM_006045	C1D
7934729	0,41	1,33	0,40216	0,68	1,61	0,47121	NM_006333	GAS1
8162179	0,41	1,33	0,33486	-1,43	-2,69	0,00062	NM_002048	TIMM13
8032484	0,41	1,33	0,27997	2,05	4,14	0,01088	NM_012458	C2orf67
8058570	0,41	1,33	0,08466	-0,15	-1,11	0,45490	NM_152519	PDSS2
8128626	0,41	1,33	0,10670	0,92	1,89	0,03121	NM_020381	PKIB
8121768	0,41	1,33	0,51624	0,43	1,35	0,33288	NM_181794	RHEB
7927285	0,41	1,33	0,40114	2,68	6,41	0,00015	NM_005614	BUB1B
7982663	0,41	1,33	0,25541	3,80	13,96	0,00032	NM_001211	CBWD5
8161575	0,41	1,33	0,24862	0,44	1,36	0,06112	NM_001024916	---
7967870	0,41	1,33	0,39040	1,57	2,97	0,02625	---	PHF14
8131519	0,41	1,33	0,09409	2,17	4,50	0,00085	NM_014660	---
7918900	0,41	1,33	0,43426	0,56	1,48	0,33358	---	RTP4
8084732	0,41	1,33	0,39167	-0,62	-1,54	0,09774	NM_022147	HSPA14

7926299	0,41	1,33	0,03404	1,65	3,14	0,01405	NM_016299	NPIPL3
8000131	0,41	1,33	0,68542	0,87	1,82	0,00896	NM_130464	METT5D1
8091656	0,41	1,33	0,21413	2,95	7,73	0,00010	NM_001113528	SIGLEC9
8030782	0,41	1,33	0,52340	-0,65	-1,57	0,07462	NM_014441	KIAA1549
8143247	0,41	1,33	0,34236	0,62	1,54	0,17808	NM_020910	COLEC12
8021946	0,41	1,33	0,57128	-1,48	-2,79	0,00727	NM_130386	BRCA1
8015769	0,41	1,33	0,37427	2,39	5,25	0,00545	NR_027676	CAPN1
7941179	0,41	1,33	0,24149	2,17	4,51	0,01968	NM_005186	POP1
8147548	0,41	1,33	0,12072	0,13	1,10	0,73998	NM_001145860	RFK
8161857	0,41	1,33	0,40513	2,90	7,49	0,00068	NM_018339	OLR1
7961142	0,41	1,33	0,17287	-1,05	-2,06	0,07816	NM_002543	ECM2
8162404	0,41	1,33	0,35680	-0,67	-1,59	0,00294	NM_001393	SCARNA12
7960728	0,41	1,33	0,45237	1,83	3,55	0,00374	NR_003010	C19orf54
8036956	0,41	1,33	0,33992	0,92	1,89	0,08858	NM_198476	MANBAL
8062371	0,41	1,33	0,28806	1,40	2,64	0,00319	NM_022077	AK1
8164293	0,41	1,33	0,37046	0,75	1,69	0,13110	NM_000476	MRPL53
8053165	0,41	1,33	0,33012	2,07	4,20	0,00554	NM_053050	PSTK
7931159	0,41	1,33	0,24874	0,61	1,53	0,13544	NM_153336	MAFG
8019796	0,41	1,33	0,26617	2,60	6,07	0,00172	NM_002359	GALNS
8003410	0,41	1,33	0,30131	1,08	2,11	0,14263	NM_000512	PXN
7967002	0,41	1,32	0,20419	1,42	2,67	0,01028	NM_001080855	ARID4B
7925201	0,41	1,32	0,16320	1,21	2,31	0,02217	NM_016374	MOSC2
7909866	0,41	1,32	0,19008	1,09	2,14	0,06577	NM_017898	MXI1
7930398	0,41	1,32	0,04955	0,62	1,54	0,06221	NM_005962	CA13
8147112	0,41	1,32	0,20373	1,86	3,62	0,01599	NM_198584	C4orf42
8093456	0,40	1,32	0,43132	2,21	4,62	0,00315	NR_033339	---
7958375	0,40	1,32	0,12807	0,05	1,03	0,86889	--- RBCK1	
8060353	0,40	1,32	0,40609	3,35	10,18	0,01203	NM_031229	ANAPC16
7928300	0,40	1,32	0,08265	2,04	4,13	0,00138	NM_173473	WDSUB1
8056047	0,40	1,32	0,34890	0,69	1,62	0,00651	NM_001128213	OSTF1
8155883	0,40	1,32	0,49588	1,51	2,85	0,00014	NM_012383	ARHGEF2
7920877	0,40	1,32	0,42803	1,56	2,95	0,01361	NM_001162383	LIPT1
8043840	0,40	1,32	0,07325	0,01	1,01	0,89190	NM_145197	MCM3AP
8070988	0,40	1,32	0,13137	2,07	4,21	0,00308	NM_003906	SFT2D3
8045083	0,40	1,32	0,03477	0,89	1,86	0,00927	NM_032740	ZNF300
8115196	0,40	1,32	0,07592	1,02	2,02	0,05540	NM_001172831	MAP2K1
7984319	0,40	1,32	0,52012	2,26	4,79	0,00099	NM_002755	PPIL5
7974198	0,40	1,32	0,28348	2,07	4,19	0,00397	NM_152329	C14orf139
7981157	0,40	1,32	0,30156	0,29	1,22	0,37840	NR_026779	TUFT1
7905428	0,40	1,32	0,08588	3,19	9,11	0,00062	NM_020127	DSCC1
8152582	0,40	1,32	0,01327	2,04	4,11	0,00298	NM_024094	TWISTNB
8138454	0,40	1,32	0,19371	1,07	2,10	0,01897	NM_001002926	SHC4
7988563	0,40	1,32	0,22283	-1,06	-2,08	0,04769	NM_203349	LSS
8070961	0,40	1,32	0,28050	1,51	2,84	0,00556	NM_002340	DGCR8
8071314	0,40	1,32	0,07297	1,26	2,40	0,00008	NM_022720	HSD17B10
8172905	0,40	1,32	0,17611	0,45	1,37	0,06398	NM_004493	KSR1
8005785	0,40	1,32	0,02251	1,01	2,01	0,00398	NM_014238	POGK
7907024	0,40	1,32	0,23333	3,16	8,96	0,00072	NM_017542	SDK1
8131205	0,40	1,32	0,27001	0,88	1,85	0,01293	NM_152744	---
8021150	0,40	1,32	0,02680	-0,11	-1,08	0,63239	--- ZNF563	
8034370	0,40	1,32	0,11020	-0,11	-1,08	0,49264	NM_145276	ADAM15
7905881	0,40	1,32	0,29984	1,84	3,59	0,01490	NM_207196	TOM1
8072659	0,40	1,32	0,53045	1,44	2,71	0,01460	NR_024194	FANCL
8052382	0,39	1,31	0,04701	1,94	3,83	0,00570	NM_001114636	NUDT6
8102713	0,39	1,31	0,34694	0,42	1,34	0,20739	NM_007083	FRMD4A
7932132	0,39	1,31	0,07378	-0,24	-1,18	0,01193	NM_018027	C2orf44
8050689	0,39	1,31	0,29073	2,46	5,51	0,00210	NM_025203	SUMF1
8085094	0,39	1,31	0,36881	0,66	1,58	0,05124	NM_182760	DNASE1L1
8176076	0,39	1,31	0,33593	1,98	3,94	0,00037	NM_001009932	OSBPL2
8063873	0,39	1,31	0,31319	0,82	1,77	0,04406	NM_144498	MGC72080
8138145	0,39	1,31	0,51569	0,54	1,45	0,33977	NR_002822	ZNF441
8025945	0,39	1,31	0,03989	-1,10	-2,14	0,01227	NM_152355	SPATA13
7968035	0,39	1,31	0,38404	0,94	1,92	0,00254	NM_001166271	RASSF4
7927186	0,39	1,31	0,28843	1,35	2,55	0,00041	NM_032023	AKR1C3
7925929	0,39	1,31	0,36457	0,01	1,01	0,99014	NM_003739	CSTF3
7947396	0,39	1,31	0,20849	1,27	2,41	0,00293	NM_001326	SEZ6L2
8000716	0,39	1,31	0,13978	1,14	2,20	0,10603	NM_012410	POP5
7967084	0,39	1,31	0,46104	0,89	1,86	0,02375	NM_015918	MIER2
8032009	0,39	1,31	0,33195	1,27	2,42	0,06637	NM_017550	DYRK4
7953229	0,39	1,31	0,05562	0,20	1,15	0,24268	NM_003845	SH3BP4
8049435	0,39	1,31	0,26383	1,62	3,08	0,00160	NM_014521	BAG5
7981439	0,39	1,31	0,41670	1,19	2,29	0,05513	NM_001015049	MPV17L2
8026982	0,39	1,31	0,31402	2,47	5,55	0,00098	NM_032683	---

8110427	0,39	1,31	0,14964	0,59	1,51	0,18609	---	C12orf66
7964677	0,39	1,31	0,20140	0,73	1,65	0,07821	NM_152440	NEK11
8082643	0,39	1,31	0,32848	-0,13	-1,10	0,43986	NM_024800	MBD5
8045604	0,39	1,31	0,14311	1,39	2,62	0,00276	NM_018328	RRP8
7946211	0,39	1,31	0,34972	0,33	1,25	0,15261	NM_015324	PMPCA
8159337	0,39	1,31	0,44249	2,56	5,89	0,00162	NM_015160	PORCN
8167287	0,39	1,31	0,44760	1,94	3,84	0,00719	NM_022825	PLCL2
8078187	0,39	1,31	0,20599	-0,03	-1,02	0,93627	NM_015184	ZNF679
8133042	0,39	1,31	0,43652	-0,86	-1,81	0,04552	NM_153363	PENK
8150901	0,39	1,31	0,37177	-2,40	-5,29	0,00229	NM_006211	USP2
7952249	0,39	1,31	0,33569	1,63	3,09	0,00280	NM_004205	MSRA
8144557	0,38	1,31	0,28577	0,22	1,16	0,12168	NM_012331	TSPYL2
8167763	0,38	1,31	0,16086	-0,21	-1,16	0,49458	NM_022117	PANK2
8060722	0,38	1,31	0,64912	0,89	1,86	0,08650	NM_153638	ZNF566
8036318	0,38	1,30	0,47509	1,19	2,28	0,12976	NM_001145345	KLHL12
7923489	0,38	1,30	0,12168	2,11	4,33	0,05228	NM_021633	ZNF740
7955721	0,38	1,30	0,19337	2,04	4,11	0,00077	NM_001004304	BATF2
7949340	0,38	1,30	0,24052	0,13	1,10	0,31680	NM_138456	C1QBP
8011850	0,38	1,30	0,53648	1,26	2,40	0,03937	NM_001212	OR7A17
8034901	0,38	1,30	0,03922	-0,87	-1,83	0,00412	NM_030901	PXMP4
8065719	0,38	1,30	0,50537	-1,98	-3,94	0,00205	NM_007238	TCEA3
7913593	0,38	1,30	0,18565	0,44	1,35	0,02861	NM_003196	NALCN
7972601	0,38	1,30	0,36282	1,21	2,31	0,00058	NM_052867	CHL1
8077270	0,38	1,30	0,37225	-0,79	-1,72	0,09616	NM_006614	SNCAIP
8107594	0,38	1,30	0,57805	-1,59	-3,00	0,00089	NM_005460	---
8134429	0,38	1,30	0,41898	-0,53	-1,44	0,49334	---	STARD13
7970924	0,38	1,30	0,12992	0,09	1,07	0,74007	NM_178006	C11orf67
7942783	0,38	1,30	0,00133	0,39	1,31	0,13407	BC002752	HIST1H2BC
8124406	0,38	1,30	0,27517	0,96	1,94	0,15156	NM_003526	SLC27A6
8107798	0,38	1,30	0,43129	-1,19	-2,28	0,02628	NM_001017372	EHD3
8041225	0,38	1,30	0,54291	0,74	1,67	0,04958	NM_014600	CPOX
8089072	0,38	1,30	0,01021	0,46	1,37	0,02356	NM_000097	THSD4
7984588	0,38	1,30	0,23757	2,02	4,05	0,00311	NM_024817	KLF6
7931810	0,38	1,30	0,33628	0,34	1,26	0,13907	NM_001300	POPDC2
8089785	0,38	1,30	0,40163	0,00	1,00	0,98659	NM_022135	TULP4
8123044	0,38	1,30	0,11735	2,24	4,74	0,00078	NM_020245	PRPF39
7974146	0,38	1,30	0,02154	1,25	2,38	0,00062	NM_017922	BICD1
7954717	0,38	1,30	0,13745	0,66	1,58	0,46317	NM_001714	PHF23
8012099	0,38	1,30	0,34831	0,81	1,76	0,15342	NM_024297	GPS1
8010747	0,38	1,30	0,64740	1,36	2,56	0,03173	NM_212492	TRAF3IP1
8049635	0,38	1,30	0,09879	0,69	1,61	0,01637	NM_015650	POLE4
8042953	0,38	1,30	0,44090	2,22	4,64	0,00099	NM_019896	TATDN2
8077804	0,37	1,30	0,42921	2,16	4,48	0,00431	NM_014760	ZNF37B
7933129	0,37	1,30	0,22424	0,51	1,42	0,03917	NR_026777	TSPAN14
7928705	0,37	1,30	0,34728	1,94	3,83	0,01652	NM_030927	SF3A2
8024420	0,37	1,29	0,41139	1,18	2,27	0,18988	NM_007165	ZNF625
8034344	0,37	1,29	0,03919	-0,79	-1,73	0,14593	NM_145233	AURKB
8012403	0,37	1,29	0,27766	2,94	7,66	0,00007	NM_004217	MRPL2
8126512	0,37	1,29	0,26943	2,33	5,03	0,01215	NM_015950	SLC35A2
8172460	0,37	1,29	0,02165	0,85	1,80	0,08617	NM_001042498	HSPB6
8036151	0,37	1,29	0,02696	-0,81	-1,76	0,01675	NM_144617	FAM134A
8048468	0,37	1,29	0,47182	1,80	3,49	0,00442	NM_024293	ZNF257
8027323	0,37	1,29	0,14523	-0,32	-1,25	0,55362	NM_033468	STEAP3
8044793	0,37	1,29	0,31570	0,29	1,22	0,09710	NM_182915	ANKRD36BP1
7922121	0,37	1,29	0,39012	-0,60	-1,51	0,13261	NR_026844	SP140L
8048926	0,37	1,29	0,45695	1,48	2,79	0,01975	NM_138402	SLC25A22
7945539	0,37	1,29	0,41066	0,14	1,10	0,78670	NM_024698	PML
7984779	0,37	1,29	0,21612	0,71	1,63	0,18169	NM_033240	SPINK1
8114964	0,37	1,29	0,18411	0,10	1,07	0,81719	NM_003122	LATS2
7970498	0,37	1,29	0,03667	0,64	1,56	0,03791	NM_014572	ATXN2L
7994386	0,37	1,29	0,44423	2,01	4,03	0,00224	NM_007245	ZMYM1
7899943	0,37	1,29	0,54983	1,79	3,47	0,00621	NM_024772	PPIAL4G
7919157	0,37	1,29	0,68923	-1,63	-3,09	0,00291	NM_001123068	DENND5B
7962151	0,37	1,29	0,10195	2,13	4,37	0,00046	NM_144973	PPPDE2
8076365	0,37	1,29	0,64468	2,23	4,69	0,00617	NM_015704	AP2A2
7937533	0,37	1,29	0,11692	1,18	2,27	0,00552	NM_012305	---
7924819	0,37	1,29	0,19192	-0,11	-1,08	0,53859	---	LOC728024
8150217	0,37	1,29	0,13653	0,10	1,07	0,72039	NR_003671	LRRC6
8152962	0,37	1,29	0,28630	0,67	1,59	0,20085	NM_012472	SF4
8035666	0,37	1,29	0,15595	-0,39	-1,31	0,03916	NM_172231	B4GALT6
8022747	0,37	1,29	0,57392	3,94	15,37	0,00019	NM_004775	ICAM1
8025601	0,37	1,29	0,45410	2,36	5,15	0,05716	NM_000201	ICA1L
8058350	0,37	1,29	0,26439	-0,04	-1,03	0,85863	NM_138468	PRDX2

8034544	0,37	1,29	0,35402	1,02	2,03	0,01595	NM_005809	PEX5
7953651	0,37	1,29	0,44044	1,76	3,39	0,00023	NM_001131023	ZDHHC1
8002029	0,36	1,29	0,31592	1,19	2,28	0,01045	NM_013304	DDX31
8164742	0,36	1,29	0,38382	0,13	1,09	0,54923	NM_022779	---
8166094	0,36	1,29	0,03755	-0,15	-1,11	0,35009	---	NEK4
8088001	0,36	1,29	0,37654	1,19	2,29	0,00224	NM_003157	RICH2
8005064	0,36	1,29	0,18947	1,19	2,27	0,00972	NM_014859	FAM86D
8088895	0,36	1,29	0,11636	0,21	1,16	0,08442	NR_024241	---
8052413	0,36	1,29	0,30212	0,44	1,35	0,10066	---	C2orf76
8054837	0,36	1,29	0,04312	0,35	1,27	0,05154	BC126397	FARSA
8034589	0,36	1,29	0,27021	1,53	2,88	0,00767	NM_004461	GLIS2
7992956	0,36	1,29	0,01055	0,80	1,74	0,03997	NM_032575	DOLK
8164515	0,36	1,29	0,02414	1,33	2,51	0,09970	NM_014908	ZNF623
8148658	0,36	1,29	0,52123	2,19	4,56	0,00098	NM_014789	C17orf58
8017831	0,36	1,29	0,23447	0,50	1,42	0,08288	NM_181656	FAM108C1
7985310	0,36	1,29	0,08693	0,44	1,36	0,17034	NM_021214	CDX4
8168395	0,36	1,29	0,18515	-1,34	-2,53	0,08687	NM_005193	RNASEK
8004241	0,36	1,29	0,11712	0,94	1,92	0,01228	NM_001004333	PCBP4
8087806	0,36	1,29	0,49839	0,40	1,32	0,33672	NM_033010	OSR1
8050497	0,36	1,28	0,44950	-0,62	-1,54	0,17298	NM_145260	DLG5
7934615	0,36	1,28	0,22737	1,77	3,41	0,00752	NM_004747	---
7957606	0,36	1,28	0,50800	-0,16	-1,12	0,34795	---	TRIM21
7945962	0,36	1,28	0,52384	0,72	1,65	0,00354	NM_003141	PABPC3
7968128	0,36	1,28	0,23383	0,04	1,03	0,93773	NM_030979	CD58
7918902	0,36	1,28	0,29381	2,51	5,68	0,00453	NM_001779	FKBP5
8125919	0,36	1,28	0,34623	1,65	3,14	0,00688	NM_001145775	---
8146334	0,36	1,28	0,25061	0,13	1,10	0,61830	---	MAGED4
8167673	0,36	1,28	0,18616	0,72	1,65	0,13416	NM_001098800	MAGED4
8172722	0,36	1,28	0,18616	0,72	1,65	0,13416	NM_001098800	DGKD
8049317	0,36	1,28	0,60628	2,67	6,36	0,00115	NM_152879	EML6
8042040	0,36	1,28	0,35615	-0,41	-1,33	0,21339	NM_001039753	---
8150034	0,36	1,28	0,70842	-2,04	-4,10	0,01443	---	ACY1
8080168	0,36	1,28	0,06929	1,37	2,58	0,02222	NM_000666	IKBKE
7909188	0,36	1,28	0,31890	-0,09	-1,06	0,79655	NM_014002	CDK2
7956076	0,36	1,28	0,44083	2,83	7,09	0,00038	NM_001798	VPS4A
7996919	0,36	1,28	0,46046	1,35	2,55	0,05218	NM_013245	COPS8
8049478	0,36	1,28	0,63205	0,70	1,63	0,13084	NM_198189	YOD1
7909283	0,36	1,28	0,47106	-0,74	-1,67	0,16172	NM_018566	LTBP3
7949412	0,36	1,28	0,23158	0,96	1,94	0,24494	NM_001130144	SIGMAR1
8160857	0,36	1,28	0,27088	2,22	4,64	0,02312	NM_005866	ZDHHC20
7970524	0,36	1,28	0,06955	1,79	3,47	0,02794	NM_153251	C1orf26
7908178	0,36	1,28	0,30596	0,39	1,31	0,12006	NM_017673	WBP11P1 // WBP11P1 // WBP11P1 // WBP11P1
8020842	0,36	1,28	0,28080	0,20	1,15	0,61773	NR_003558 // NR_003558 // NR_003558 // NR_003558	TOX2
8062782	0,36	1,28	0,34027	0,18	1,13	0,56165	NM_032883	PSMD4
7905374	0,36	1,28	0,50809	0,59	1,51	0,05733	NM_002810	PDAP1
8141273	0,36	1,28	0,32733	-0,49	-1,41	0,12398	NM_014891	SLC30A1
7924092	0,35	1,28	0,38934	0,00	1,00	0,99088	NM_021194	ZNF131
8105136	0,35	1,28	0,63062	4,15	17,74	0,00007	NM_003432	NDUFB11
8172296	0,35	1,28	0,30889	1,32	2,50	0,07201	NR_024234	RPL17
8023252	0,35	1,28	0,31809	0,97	1,95	0,02055	NM_000985	TPD52L2
8064191	0,35	1,28	0,47629	2,15	4,44	0,00143	NM_199360	TADA1
7922008	0,35	1,28	0,13759	0,39	1,31	0,13573	NM_053053	FXR2
8012226	0,35	1,28	0,35844	1,12	2,18	0,00035	NM_004860	ATP5G3
8056930	0,35	1,28	0,20076	0,37	1,29	0,16681	NM_001689	GART
8070102	0,35	1,28	0,40019	2,95	7,71	0,00029	NM_000819	LOC100133106
8115732	0,35	1,28	0,48545	-0,36	-1,28	0,06102	AY358216	---
8140707	0,35	1,28	0,38089	-0,28	-1,22	0,62405	---	EIF4ENIF1
8075507	0,35	1,28	0,21494	0,09	1,06	0,87592	NM_019843	NHEDC1
8177130	0,35	1,28	0,48733	-0,27	-1,21	0,57590	NM_139173	HDGF
7921133	0,35	1,28	0,10068	2,62	6,16	0,00101	NM_004494	TENC1
7955663	0,35	1,28	0,48485	0,48	1,39	0,15440	NM_170754	FAM118A
8073752	0,35	1,28	0,27626	1,34	2,54	0,03744	NM_001104595	CMAH
8124307	0,35	1,28	0,50305	-1,03	-2,05	0,02101	NR_002174	IGHD
7981724	0,35	1,28	0,76680	-4,17	-17,95	0,00127	BC021276	HABP4
8156610	0,35	1,28	0,40186	1,41	2,66	0,00017	NM_014282	DNAJC28
8070097	0,35	1,28	0,13549	1,04	2,05	0,12741	NM_017833	PI4KAP2
8074748	0,35	1,28	0,00054	3,52	11,48	0,00022	NR_003700	FNTA
8146268	0,35	1,28	0,43886	1,56	2,94	0,02257	NM_002027	---
7979862	0,35	1,28	0,66942	-1,58	-2,99	0,08155	---	CHMP4B
8061958	0,35	1,28	0,05126	1,14	2,20	0,02917	NM_176812	EVI2B
8014063	0,35	1,28	0,35750	1,73	3,32	0,02873	NM_006495	LIAS

8094688	0,35	1,28	0,27749	1,72	3,29	0,03015	NM_006859	PSG6
8037246	0,35	1,28	0,46573	-1,11	-2,16	0,00457	NM_001031850	TMUB2
8007594	0,35	1,28	0,43946	2,07	4,18	0,00034	NM_177441	LOC442454
8173179	0,35	1,27	0,48230	0,10	1,07	0,51278	NR_002308	TAGLN2
7921487	0,35	1,27	0,28056	0,26	1,20	0,56195	NM_003564	SUPT3H
8126710	0,35	1,27	0,19769	1,33	2,52	0,00176	NM_181356	ZNF630
8172379	0,35	1,27	0,16363	0,76	1,70	0,14008	NM_001037735	CDK5RAP2
8163733	0,35	1,27	0,28701	1,56	2,94	0,00371	NM_018249	FOSL1
7949532	0,35	1,27	0,34256	1,09	2,13	0,08432	NM_005438	---
8030391	0,35	1,27	0,73400	-2,98	-7,88	0,00003	--- ATN1	
7953547	0,35	1,27	0,23085	0,66	1,58	0,06269	NM_001007026	WFDC3
8066579	0,35	1,27	0,37003	0,31	1,24	0,27466	NM_080614	GRAMD1A
8027701	0,35	1,27	0,13411	2,09	4,25	0,00544	NM_020895	DIXDC1
7943803	0,35	1,27	0,40603	0,70	1,62	0,18310	NM_001037954	PPP2R5D
8119627	0,35	1,27	0,18946	1,58	2,99	0,00068	NM_006245	PTGFRN
7904293	0,35	1,27	0,42466	2,80	6,97	0,00301	NM_020440	FLJ16423
8175638	0,35	1,27	0,21329	0,92	1,89	0,01651	ENST00000432041	C10orf134
7930818	0,34	1,27	0,43303	-0,27	-1,21	0,48935	ENST00000341276	IGJ
8100827	0,34	1,27	0,60124	-1,77	-3,41	0,00009	NM_144646	SS18L1
8063839	0,34	1,27	0,06373	1,26	2,39	0,01381	NM_198935	TMSB15B
8169073	0,34	1,27	0,42163	0,13	1,09	0,78485	NM_194324	GATAD1
8134201	0,34	1,27	0,24780	0,48	1,39	0,14560	NM_021167	TEC
8100231	0,34	1,27	0,38774	1,43	2,69	0,05581	NM_003215	PS1TP4
8054769	0,34	1,27	0,25023	0,02	1,01	0,95609	AY427952 SNORD68	
7997940	0,34	1,27	0,26686	-1,23	-2,34	0,37791	NR_002450	EPB41L3
8022118	0,34	1,27	0,36935	-1,35	-2,56	0,00033	NM_012307	PTN
8143144	0,34	1,27	0,41087	-1,39	-2,62	0,00741	NM_002825	NCOR2
7967493	0,34	1,27	0,38305	1,47	2,77	0,00839	NM_006312	MOGS
8053158	0,34	1,27	0,25100	2,52	5,72	0,01515	NM_006302	---
7992516	0,34	1,27	0,56176	-0,62	-1,53	0,28813	--- TMEM18	
8049998	0,34	1,27	0,49273	1,59	3,02	0,01103	NM_152834	NRSN2
8060339	0,34	1,27	0,19828	0,19	1,14	0,71965	NM_024958	MAMLD1
8170420	0,34	1,27	0,37578	0,80	1,74	0,00003	NM_005491	TMEM148
7997676	0,34	1,27	0,22793	-1,33	-2,51	0,01619	BC030801 RFFL	
8014189	0,34	1,27	0,48219	4,04	16,43	0,00120	NM_057178	CYTH3
8138067	0,34	1,27	0,39059	2,80	6,98	0,00001	NM_004227	NUAK1
7966026	0,34	1,26	0,43838	1,40	2,64	0,00017	NM_014840	KIN
7932000	0,34	1,26	0,26486	0,33	1,25	0,31746	NM_012311	SP4
8131709	0,34	1,26	0,50811	2,25	4,75	0,00273	NM_003112	KIF22
8003583	0,34	1,26	0,43267	2,04	4,11	0,00151	NM_007317	MKKS
8064967	0,34	1,26	0,43927	1,74	3,33	0,01645	NM_018848	RPS14P3
7898582	0,34	1,26	0,43496	1,35	2,56	0,03534	BC072682	GPR37
8142687	0,34	1,26	0,36549	-0,96	-1,94	0,00021	NM_005302	LPCAT1
8110841	0,34	1,26	0,30198	2,26	4,79	0,00608	NM_024830	---
7995580	0,34	1,26	0,57951	0,24	1,18	0,48179	--- COPG2	
8142945	0,33	1,26	0,13735	0,79	1,73	0,01443	NM_012133	C15orf29
7987180	0,33	1,26	0,44851	1,15	2,22	0,01844	NM_024713	FCGR2C
7906767	0,33	1,26	0,71098	-0,52	-1,44	0,52470	NM_201563	PANK1
7934945	0,33	1,26	0,28803	0,96	1,95	0,02361	NM_148977	GJD3
8014969	0,33	1,26	0,38725	-0,81	-1,75	0,02455	NM_152219	LIF
8075310	0,33	1,26	0,09342	2,96	7,76	0,00037	NM_002309	CKAP4
7966035	0,33	1,26	0,34291	0,23	1,17	0,57499	NM_006825	ZNF780A
8036820	0,33	1,26	0,36800	1,14	2,20	0,00193	NM_001142577	NUP50
8073733	0,33	1,26	0,44945	1,84	3,58	0,00469	NM_153645	ZNF404
8037430	0,33	1,26	0,41863	1,83	3,56	0,00661	NM_001033719	ADCK1
7976000	0,33	1,26	0,50252	2,59	6,01	0,00494	NM_020421	MRPL40
8071206	0,33	1,26	0,27915	1,51	2,84	0,00318	NM_003776	SCARA3
8145555	0,33	1,26	0,49711	-0,39	-1,31	0,37237	NM_016240	RAB44
8119102	0,33	1,26	0,17983	-1,27	-2,41	0,01596	ENST00000229824	C2orf56
8041495	0,33	1,26	0,47478	1,71	3,27	0,00102	NM_144736	DNMT1
8033912	0,33	1,26	0,27825	1,64	3,11	0,03507	NM_001130823	FAM13A
8101728	0,33	1,26	0,14112	0,58	1,50	0,01036	NM_014883	ZNF420
8028219	0,33	1,26	0,40316	1,05	2,07	0,01273	NM_144689	LOC162632
8005225	0,33	1,26	0,50400	2,75	6,73	0,00407	NR_003190	NBPF10
7912808	0,33	1,26	0,75517	0,09	1,07	0,80438	NM_001039703	LRRCC1
8147079	0,33	1,26	0,22298	2,66	6,34	0,00154	NM_033402	C14orf182
7978911	0,33	1,26	0,40846	-0,69	-1,61	0,32514	NM_001012706	CEP68
8042326	0,33	1,26	0,25843	0,09	1,07	0,75820	NM_015147	SLC8A1
8051762	0,33	1,26	0,36524	-1,06	-2,08	0,00062	NM_021097	RAD51L1
7975292	0,33	1,26	0,48582	1,58	2,98	0,02163	NM_002877	C5orf43
8112302	0,33	1,26	0,26177	1,61	3,05	0,00878	NM_001048249	ARHGDIA
8019263	0,33	1,26	0,02572	0,99	1,98	0,09091	NM_004309	MTX1
7905968	0,33	1,26	0,51648	-0,16	-1,12	0,77926	NM_002455	KCND1

8172493	0,33	1,26	0,10894	0,03	1,02	0,75347	NM_004979	TINAGL1
7899627	0,33	1,26	0,13507	1,87	3,65	0,00756	NM_022164	SEC16A
8165183	0,33	1,26	0,32622	2,23	4,69	0,00252	NM_014866	KIAA1310
8053949	0,33	1,26	0,45753	1,55	2,94	0,02616	NM_017991	APH1A
7919729	0,33	1,26	0,17009	1,56	2,95	0,04488	NM_016022	SYNE1
8130211	0,33	1,26	0,18340	-0,04	-1,03	0,54475	NM_182961	MECR
7914245	0,33	1,26	0,37690	0,95	1,93	0,03227	NM_001024732	NECAB3
8065693	0,33	1,25	0,28114	0,85	1,80	0,12927	NM_031232	KIAA0895
8139021	0,33	1,25	0,45825	2,52	5,73	0,00049	NM_001100425	C12orf51
7966517	0,33	1,25	0,30289	2,59	6,03	0,00009	NM_001109662	GRK5
7930894	0,33	1,25	0,47537	0,17	1,12	0,56866	NM_005308	NDC80
8019857	0,33	1,25	0,05955	2,58	5,99	0,00512	NM_006101	DMGDH
8112822	0,33	1,25	0,10786	1,21	2,31	0,01545	NM_013391	---
7920333	0,33	1,25	0,52613	-1,21	-2,31	0,04659	--- SNORD32A	
8030360	0,33	1,25	0,25089	1,56	2,95	0,10006	NR_000021	C21orf91
8069574	0,33	1,25	0,46134	-0,27	-1,21	0,23512	NM_001100420	---
8090801	0,33	1,25	0,37213	-0,75	-1,68	0,04357	--- IVD	
7982723	0,33	1,25	0,62653	2,18	4,55	0,00083	NM_002225	CTSH
7990757	0,33	1,25	0,39531	1,32	2,50	0,01045	NM_004390	CUEDC2
7936011	0,33	1,25	0,58277	0,80	1,74	0,18514	NM_024040	CDKN3
7974404	0,32	1,25	0,46027	3,26	9,59	0,00039	NM_005192	C1D
7934731	0,32	1,25	0,31963	1,39	2,62	0,02829	NM_006333	LOC100132346
8002760	0,32	1,25	0,33524	-0,83	-1,77	0,06916	ENST00000412576	LOC100132147
7912802	0,32	1,25	0,73698	-0,31	-1,24	0,70983	BC036435 IPO4	
7978132	0,32	1,25	0,38694	2,15	4,45	0,00341	NM_024658	OPN3
7925492	0,32	1,25	0,40462	3,31	9,89	0,00006	NM_014322	CXorf23
8171708	0,32	1,25	0,40841	-0,03	-1,02	0,88473	NM_198279	KDM5D
8177232	0,32	1,25	0,36799	1,24	2,36	0,02080	NM_001146705	AGPAT4
8130628	0,32	1,25	0,12625	0,54	1,45	0,06268	NM_020133	RRP7B
8076449	0,32	1,25	0,73543	-0,51	-1,42	0,37992	NR_002184	TARSL2
7991640	0,32	1,25	0,31065	1,03	2,05	0,02596	NM_152334	P4HA2
8114002	0,32	1,25	0,66697	-0,27	-1,21	0,53702	ENST00000416053	DSG2
8020779	0,32	1,25	0,27093	4,19	18,29	0,00094	NM_001943	LSG1
8092905	0,32	1,25	0,55718	3,08	8,47	0,00143	NM_018385	BMP4
7979241	0,32	1,25	0,51839	0,35	1,27	0,54196	NM_001202	FAM24B
7936809	0,32	1,25	0,42317	2,35	5,09	0,00033	NM_152644	NDUFA13
8027205	0,32	1,25	0,39056	-0,80	-1,74	0,17692	NM_015965	C6orf138
8126855	0,32	1,25	0,20699	-1,49	-2,81	0,01695	NM_001013732	HARS2
8108603	0,32	1,25	0,07186	0,55	1,46	0,14328	NM_012208	PRDM10
7952707	0,32	1,25	0,21119	0,85	1,80	0,02596	NM_020228	SNORA7B
8090565	0,32	1,25	0,44898	-0,09	-1,07	0,40924	NR_002992	BUD13
7951826	0,32	1,25	0,18319	0,58	1,50	0,06145	NM_032725	SDF4
7911422	0,32	1,25	0,30995	0,22	1,16	0,54920	NM_016547	SSPN
7954481	0,32	1,25	0,18833	-0,41	-1,33	0,07736	NM_005086	PURB
8139430	0,32	1,25	0,39202	1,02	2,03	0,00934	NM_033224	C4orf43
8098150	0,32	1,25	0,22915	0,15	1,11	0,48795	NM_018352	---
8165653	0,32	1,24	0,35054	-0,87	-1,82	0,00278	--- NEURL4	
8012166	0,32	1,24	0,42200	0,82	1,76	0,06611	NM_032442	COQ9
7996041	0,32	1,24	0,43142	2,58	5,96	0,00199	NM_020312	GNA12
8137865	0,32	1,24	0,59663	3,23	9,39	0,00638	NM_007353	CENPC1
8100603	0,32	1,24	0,53474	0,36	1,29	0,34358	NM_001812	FN1
8048139	0,31	1,24	0,28550	-1,14	-2,20	0,00582	NM_212482	MSH2
8041867	0,31	1,24	0,28182	2,41	5,31	0,00131	NM_000251	---
8156846	0,31	1,24	0,41918	-0,60	-1,51	0,39936	--- TRAPPC9	
8153175	0,31	1,24	0,63147	2,93	7,64	0,00021	NM_031466	TARBP1
7925130	0,31	1,24	0,46847	2,21	4,62	0,00581	NM_005646	IFT88
7967933	0,31	1,24	0,40382	-0,18	-1,13	0,39609	NM_175605	FAM177A1
7973918	0,31	1,24	0,37413	2,55	5,86	0,00725	NM_173607	WHSC1
8093539	0,31	1,24	0,28229	1,53	2,88	0,00461	NM_133330	ACER2
8154563	0,31	1,24	0,68579	0,84	1,78	0,05031	NM_001010887	NRIP3
7946446	0,31	1,24	0,42323	-0,04	-1,02	0,93078	NM_020645	TSHZ1
8021768	0,31	1,24	0,59078	0,13	1,10	0,40637	NM_005786	DPP3
7941621	0,31	1,24	0,41775	2,65	6,26	0,00683	NM_005700	SFRS4
7914235	0,31	1,24	0,53487	0,98	1,97	0,05150	NM_005626	ZNF321
8039013	0,31	1,24	0,00787	-0,27	-1,20	0,43798	NM_203307	ADAMTSL3
7985522	0,31	1,24	0,34931	0,23	1,17	0,47255	NM_207517	EEF2K
7993946	0,31	1,24	0,34376	0,50	1,41	0,19208	NM_013302	ZNF223
8029360	0,31	1,24	0,17058	0,93	1,91	0,14859	NM_013361	TAF9
8177635	0,31	1,24	0,20928	0,82	1,76	0,02400	NM_003187	FAM171A1
7932243	0,31	1,24	0,46942	1,89	3,70	0,02410	NM_001010924	CDYL
8116710	0,31	1,24	0,51010	1,13	2,18	0,00389	NR_026590	PARD3B
8047709	0,31	1,24	0,43668	-0,10	-1,07	0,84090	NM_152526	GOLGA1
8164105	0,31	1,24	0,29181	1,35	2,56	0,03475	NM_002077	EXOG

8078818	0,31	1,24	0,21404	0,20	1,15	0,39845	NM_005107	STIM1
7937900	0,31	1,24	0,64857	2,98	7,88	0,00383	NM_003156	PHKA1
8173551	0,31	1,24	0,45611	2,90	7,44	0,00022	NM_002637	ANKRD20B
8067864	0,31	1,24	0,65782	0,61	1,53	0,58290	NR_003366	SH2B3
7958749	0,31	1,24	0,39182	0,61	1,53	0,06983	NM_005475	LCA5L
8070398	0,31	1,24	0,29176	-0,34	-1,27	0,29496	NM_152505	ZNF264
8031690	0,31	1,24	0,56632	0,82	1,77	0,10662	NM_003417	SMARCD3
8143919	0,30	1,24	0,09911	-0,41	-1,33	0,13930	NM_001003802	GPR124
8145865	0,30	1,24	0,48963	-0,05	-1,04	0,93495	NM_032777	SUPT5H
8028674	0,30	1,24	0,42689	1,57	2,98	0,01129	NM_003169	LYPD6
8045674	0,30	1,23	0,13556	0,97	1,96	0,06676	NM_194317	RRP15
7909782	0,30	1,23	0,46369	-0,05	-1,03	0,86107	NM_016052	HLA-DMA
8178884	0,30	1,23	0,35918	3,34	10,10	0,00424	NM_006120	HLA-DMA
8180086	0,30	1,23	0,35918	3,34	10,10	0,00424	NM_006120	ANKRA2
8112596	0,30	1,23	0,42565	1,35	2,55	0,01362	NM_023039	APEH
8079772	0,30	1,23	0,59508	1,74	3,34	0,00236	NM_001640	TBC1D22A
8073909	0,30	1,23	0,30939	1,74	3,34	0,01630	NM_014346	ASTN1
7922525	0,30	1,23	0,16337	-0,98	-1,98	0,04096	NM_004319	FAM76B
7951093	0,30	1,23	0,43118	0,66	1,58	0,10889	NM_144664	RGP1
8155110	0,30	1,23	0,16952	1,38	2,61	0,00620	NM_001080496	ABCA13
8132743	0,30	1,23	0,11092	-0,28	-1,21	0,00293	NM_152701	ZNF181
8027685	0,30	1,23	0,27953	0,23	1,18	0,32231	NM_001029997	LALBA
7962804	0,30	1,23	0,42768	-0,10	-1,07	0,46053	NM_002289	---
8078542	0,30	1,23	0,22606	0,14	1,10	0,47154	--- ---	
7979886	0,30	1,23	0,62759	1,28	2,42	0,19812	--- C7orf41	
8132055	0,30	1,23	0,61012	0,39	1,31	0,06007	NM_152793	PIGG
8093343	0,30	1,23	0,28405	0,75	1,68	0,00092	NM_001127178	SCN8A
7955502	0,30	1,23	0,28287	0,39	1,31	0,02114	NM_014191	ZFP90
7996807	0,30	1,23	0,15491	0,98	1,98	0,01228	NM_133458	ZFAT
8153043	0,30	1,23	0,42620	1,31	2,49	0,02295	NM_001029939	RNF167
8004030	0,30	1,23	0,04429	2,33	5,04	0,00184	NM_015528	ZNF609
7984203	0,30	1,23	0,61309	2,58	5,99	0,00039	NM_015042	SMCR7L
8073135	0,30	1,23	0,44571	2,57	5,95	0,00053	NM_019008	---
7924307	0,30	1,23	0,41069	-1,35	-2,54	0,06396	--- AVEN	
7987165	0,30	1,23	0,63541	1,03	2,04	0,00522	NM_020371	BLCAP
8066195	0,30	1,23	0,40141	0,62	1,54	0,07639	NM_006698	NAP1L3
8173917	0,30	1,23	0,35079	-0,99	-1,98	0,04597	NM_004538	CASP8AP2
8121118	0,30	1,23	0,57384	1,61	3,06	0,00233	NM_012115	FAM119A
8058486	0,30	1,23	0,20607	2,24	4,71	0,00004	NM_145280	OR2T2
7911281	0,29	1,23	0,70658	-2,28	-4,86	0,00033	NM_001004136	OR5B2
7948326	0,29	1,23	0,47118	-0,34	-1,27	0,08337	NM_001005566	GPRASP2
8168976	0,29	1,23	0,08339	0,66	1,58	0,00118	NM_001004051	HIST2H4A
7905067	0,29	1,23	0,33972	3,67	12,69	0,00023	NM_003548	HIST2H4A
7919627	0,29	1,23	0,33972	3,67	12,69	0,00023	NM_003548	UQCC
8065889	0,29	1,23	0,40443	1,78	3,44	0,00154	NM_018244	TMEM208
7996490	0,29	1,23	0,70297	0,81	1,75	0,27199	NM_014187	FLJ45950
7952673	0,29	1,23	0,31275	-0,56	-1,48	0,06350	AK127847 HLA-DMA	
8125537	0,29	1,23	0,31805	3,05	8,30	0,00673	NM_006120	C14orf28
7974117	0,29	1,23	0,59131	1,67	3,17	0,00232	NM_001017923	GPR125
8099612	0,29	1,23	0,58312	2,70	6,48	0,00124	NM_145290	MIPOL1
7973985	0,29	1,23	0,14327	1,08	2,11	0,00286	NM_138731	CDKL5
8166289	0,29	1,22	0,50636	0,90	1,87	0,00492	NM_001037343	DAXX
8180179	0,29	1,22	0,39080	-0,17	-1,12	0,61083	NM_001141969	CBR4
8103622	0,29	1,22	0,42818	0,85	1,81	0,04557	NM_032783	DIAPH1
8114658	0,29	1,22	0,52745	2,68	6,41	0,00102	NM_005219	FAM180A
8143127	0,29	1,22	0,60510	-0,74	-1,67	0,06114	NM_205855	CTHRC1
8147777	0,29	1,22	0,29569	0,63	1,54	0,34044	NM_138455	---
8131337	0,29	1,22	0,41968	0,17	1,13	0,52690	--- PVRL2	
8029507	0,29	1,22	0,27111	1,80	3,49	0,03580	NM_002856	RNF5P1
8178188	0,29	1,22	0,44870	1,65	3,14	0,00189	NR_003129	C1orf54
7905147	0,29	1,22	0,33753	-0,99	-1,99	0,01162	NM_024579	HSPA2
7975076	0,29	1,22	0,47914	0,45	1,37	0,17161	NM_021979	ALG2
8162827	0,29	1,22	0,27640	2,43	5,38	0,00215	NR_024532	PTPRF
7900792	0,29	1,22	0,63253	3,49	11,27	0,00158	NM_002840	ZNF226
8029399	0,29	1,22	0,49605	0,65	1,57	0,02182	NM_001032372	FAM133B
8105504	0,29	1,22	0,34282	0,17	1,12	0,45418	NM_152789	CANT1
8018982	0,29	1,22	0,47423	1,87	3,65	0,00456	NM_138793	---
8031906	0,29	1,22	0,00348	-1,02	-2,03	0,13473	--- DGKI	
8143154	0,29	1,22	0,02603	-0,92	-1,89	0,00002	NM_004717	ZNF280A
8074853	0,29	1,22	0,16894	1,12	2,18	0,02044	NM_080740	ZNF773
8031759	0,29	1,22	0,39016	0,45	1,37	0,49002	NM_198542	C14orf106
7978776	0,29	1,22	0,19857	1,39	2,63	0,09944	NM_018353	THUMPD2
8051746	0,29	1,22	0,17118	2,29	4,90	0,00077	NR_028102	ISL1

8105220	0,29	1,22	0,57069	-0,62	-1,54	0,01905	NM_002202	CLSTN1
7912257	0,29	1,22	0,53037	1,59	3,01	0,01377	NM_001009566	AR
8167998	0,29	1,22	0,28889	-0,68	-1,60	0,01130	NM_000044	KIAA0427
8021154	0,29	1,22	0,53022	0,44	1,36	0,33120	NM_001142397	RRBP1
8065136	0,29	1,22	0,24474	0,13	1,10	0,56212	NM_001042576	CCDC111
8098556	0,29	1,22	0,45572	1,51	2,84	0,02283	NM_152683	MTMR15
7982309	0,29	1,22	0,42681	0,62	1,53	0,02978	NM_014967	MAGI3
7904106	0,29	1,22	0,43305	1,94	3,84	0,00136	NM_152900	NUMBL
8036923	0,29	1,22	0,74314	0,29	1,22	0,29958	NM_004756	TMCO4
7913187	0,29	1,22	0,30111	0,53	1,45	0,19413	NM_181719	AGPAT3
8068952	0,29	1,22	0,54738	1,68	3,20	0,00715	NM_020132	UQCRB
8151909	0,29	1,22	0,28084	0,03	1,02	0,83934	NM_006294	ACPL2
8083075	0,29	1,22	0,42116	2,02	4,06	0,00487	NM_152282	THNSL1
7926708	0,29	1,22	0,34923	0,56	1,47	0,08219	NM_024838	WDR55
8108593	0,29	1,22	0,54570	0,70	1,63	0,30477	NM_017706	LOC100131848
7930917	0,29	1,22	0,69231	-0,39	-1,31	0,13257	AK097099 ---	
8139242	0,29	1,22	0,42897	-1,77	-3,40	0,00013	--- TCEAL4	
8169035	0,29	1,22	0,17163	0,33	1,26	0,18097	NM_024863	BAIAP2L1
8141206	0,29	1,22	0,35033	4,18	18,13	0,00189	NM_018842	---
7994265	0,28	1,22	0,66586	-3,00	-7,99	0,00033	--- PPIA	
8124924	0,28	1,22	0,58082	0,34	1,27	0,15887	ENST00000355968	ZBTB5
8161224	0,28	1,22	0,37144	0,84	1,79	0,00598	NM_014872	PDCD2L
8027642	0,28	1,22	0,50424	2,29	4,90	0,00038	NM_032346	SLC45A1
7897416	0,28	1,22	0,39258	0,60	1,52	0,09493	NM_001080397	STRA6
7990309	0,28	1,22	0,45195	-0,96	-1,95	0,03472	NM_022369	C14orf142
7980990	0,28	1,22	0,43775	2,21	4,62	0,00291	NM_032490	BMF
7987454	0,28	1,22	0,10563	1,81	3,51	0,00824	NM_001003940	PHKG2
7994928	0,28	1,22	0,63750	3,05	8,27	0,00598	NM_000294	QDPR
8099541	0,28	1,22	0,66205	1,02	2,03	0,03905	NM_000320	FLAD1
7905831	0,28	1,22	0,06333	1,17	2,24	0,00312	NM_025207	WDR46
8125671	0,28	1,22	0,38830	1,14	2,21	0,01245	NM_005452	WDR46
8178939	0,28	1,22	0,38830	1,14	2,21	0,01245	NM_005452	CDRT4
8012918	0,28	1,22	0,29317	0,69	1,61	0,00411	NM_173622	EMB
8112007	0,28	1,22	0,50815	2,41	5,32	0,00099	NM_198449	TMEM185A
8175621	0,28	1,22	0,63815	1,17	2,25	0,04075	NM_032508	C9orf64
8162039	0,28	1,22	0,62361	3,98	15,73	0,00001	NM_032307	CYHR1
8153819	0,28	1,22	0,61464	1,10	2,14	0,00302	NM_138496	---
8075817	0,28	1,22	0,57262	-0,90	-1,86	0,14755	--- DDX59	
7923221	0,28	1,22	0,17670	0,38	1,30	0,28560	NM_001031725	USP12
7970696	0,28	1,21	0,30352	0,89	1,86	0,00892	NM_182488	POLR2E
8032149	0,28	1,21	0,74463	1,39	2,61	0,06198	NM_002695	ANKRD36B
8043697	0,28	1,21	0,42962	1,35	2,54	0,00561	NM_025190	NYNRIN
7973724	0,28	1,21	0,67916	-1,66	-3,15	0,03358	NM_025081	DIO3
7976858	0,28	1,21	0,67365	-0,72	-1,65	0,05467	NM_001362	ANKS6
8162803	0,28	1,21	0,35025	1,68	3,20	0,01257	NM_173551	PPP5C
8029814	0,28	1,21	0,42416	0,75	1,69	0,00104	NM_006247	RNF149
8054329	0,28	1,21	0,58640	1,39	2,62	0,00446	NM_173647	ARFGAP1
8064042	0,28	1,21	0,37915	0,95	1,93	0,00232	NM_175609	ZKSCAN2
8000399	0,28	1,21	0,33410	1,02	2,03	0,06784	NM_001012981	TRUB2
8164428	0,28	1,21	0,53868	2,04	4,12	0,00337	NM_015679	PTDSS2
7937378	0,28	1,21	0,54990	1,93	3,81	0,00042	NM_030783	FTSJ3
8017437	0,28	1,21	0,23667	0,11	1,08	0,72502	NM_017647	C4orf36
8101616	0,28	1,21	0,49439	0,29	1,22	0,19985	NM_144645	UCKL1
8067756	0,28	1,21	0,48999	2,21	4,61	0,00017	NM_017859	ARID4A
7974621	0,28	1,21	0,64772	1,88	3,67	0,00169	NM_002892	FAM45B
8169928	0,28	1,21	0,81013	-0,18	-1,13	0,67635	NR_027141	FLJ36031
8142136	0,28	1,21	0,40984	-0,36	-1,28	0,46056	NM_175884	ZNF813
8030997	0,28	1,21	0,11685	0,26	1,19	0,25864	NM_001004301	---
8061184	0,28	1,21	0,28551	0,01	1,01	0,94760	--- ---	
7982254	0,28	1,21	0,69601	-0,36	-1,28	0,14939	--- ---	
7987110	0,28	1,21	0,69601	-0,36	-1,28	0,14939	--- FRZB	
8057506	0,28	1,21	0,39835	-0,73	-1,66	0,00707	NM_001463	ARL17A
8016300	0,28	1,21	0,65038	1,27	2,41	0,02370	NM_001113738	OR51E1
7937952	0,28	1,21	0,25618	-1,15	-2,23	0,04299	NM_152430	FAM111B
7940147	0,28	1,21	0,61116	1,09	2,13	0,00279	NM_198947	UBE2V2
8146379	0,28	1,21	0,27907	0,32	1,25	0,08563	NM_003350	ANKRD36B
8054054	0,28	1,21	0,45793	1,47	2,77	0,01229	NM_025190	EEPD1
8132305	0,27	1,21	0,26062	0,05	1,04	0,81941	NM_030636	ZNF84
7960143	0,27	1,21	0,42900	1,72	3,30	0,00766	NM_003428	SMG5
7920944	0,27	1,21	0,43856	2,44	5,41	0,00078	NM_015327	RPS18P9
8122699	0,27	1,21	0,33904	0,33	1,26	0,47797	BC071678 GABARAPL3	
7991395	0,27	1,21	0,58208	-0,73	-1,65	0,24860	NR_028287	DENR
7959473	0,27	1,21	0,19396	1,57	2,98	0,00148	NM_003677	FKBP3

7978766	0,27	1,21	0,22355	0,98	1,98	0,01276	NM_002013	GBP4
7917561	0,27	1,21	0,61333	-0,69	-1,61	0,00146	NM_052941	FAM19A2
7964631	0,27	1,21	0,55957	-0,94	-1,92	0,09255	NM_178539	PODXL2
8082368	0,27	1,21	0,58711	1,57	2,97	0,00914	NM_015720	GEMIN8
8171373	0,27	1,21	0,21479	0,03	1,02	0,94012	NM_017856	ITGA3
8008237	0,27	1,21	0,18154	4,31	19,83	0,00428	NM_002204	---
8081943	0,27	1,21	0,55830	-0,60	-1,52	0,31824	--- TMPRSS6	
8072796	0,27	1,21	0,63752	-0,64	-1,56	0,04708	NM_153609	MATN3
8050537	0,27	1,21	0,53593	1,92	3,78	0,01039	NM_002381	ZNF76
8118915	0,27	1,21	0,23743	1,59	3,01	0,02776	NM_003427	PES1
8075406	0,27	1,20	0,22531	-0,02	-1,02	0,96491	NM_014303	---
7969792	0,27	1,20	0,41475	0,43	1,35	0,00756	--- ---	
8171879	0,27	1,20	0,64766	1,36	2,56	0,00026	--- PDE7A	
8151074	0,27	1,20	0,60573	1,90	3,74	0,00103	NM_002603	C7orf70
8138088	0,27	1,20	0,48080	2,87	7,30	0,00010	NM_001037163	C21orf94
8068052	0,27	1,20	0,45250	-0,86	-1,81	0,20096	NR_027246	ZNF776
8031815	0,27	1,20	0,33270	0,58	1,49	0,02319	NM_173632	ALS2
8058295	0,27	1,20	0,07497	0,08	1,06	0,71058	NM_020919	---
7974255	0,26	1,20	0,88166	-3,11	-8,65	0,00995	--- BCAT2	
8038202	0,26	1,20	0,50447	2,58	6,00	0,00323	NM_001190	ERI2
8000013	0,26	1,20	0,47382	1,84	3,58	0,01007	NM_001142725	TESK2
7915718	0,26	1,20	0,37555	0,15	1,11	0,44918	NM_007170	NXN
8010967	0,26	1,20	0,10083	1,81	3,50	0,02770	NM_022463	ANKH
8111101	0,26	1,20	0,44926	1,40	2,64	0,00801	NM_054027	LOC100130581
8015798	0,26	1,20	0,31363	3,13	8,78	0,00013	NR_027413	C5orf34
8111960	0,26	1,20	0,36602	1,79	3,45	0,02602	BC036867 INSR	
8033362	0,26	1,20	0,62365	1,86	3,63	0,00780	NM_000208	BCS1L
8048370	0,26	1,20	0,47103	2,16	4,47	0,00218	NM_004328	---
8115884	0,26	1,20	0,64011	-0,52	-1,43	0,29775	--- MRPL12	
8010664	0,26	1,20	0,43974	1,67	3,18	0,05946	NM_002949	KBTBD3
7951429	0,26	1,20	0,06423	-0,21	-1,15	0,48486	NM_198439	RNASEH2A
8026051	0,26	1,20	0,51701	1,66	3,16	0,00201	NM_006397	ZBTB43
8157933	0,26	1,20	0,64247	1,24	2,36	0,02154	NM_014007	C15orf42
7985873	0,26	1,20	0,23909	2,03	4,09	0,01911	NM_152259	ZBED4
8073943	0,26	1,20	0,46782	1,79	3,47	0,00017	NM_014838	RNF19B
7914603	0,26	1,20	0,35437	1,91	3,75	0,01862	NM_153341	GNA11
8024566	0,26	1,20	0,30720	1,07	2,10	0,05657	ENST00000433476	IL6
8138542	0,26	1,20	0,50553	0,31	1,24	0,33609	NM_000600	AKR1E2
7925904	0,26	1,20	0,35801	3,51	11,39	0,00042	NM_001040177	TBC1D9
8102912	0,26	1,20	0,53645	1,43	2,69	0,02258	NM_015130	CAPS2
7964997	0,26	1,20	0,19634	0,27	1,21	0,48808	NM_032606	RELL1
8099797	0,26	1,20	0,38087	0,14	1,10	0,82434	NM_001085399	LTBP2
7980152	0,26	1,20	0,57142	1,56	2,96	0,01521	NM_000428	---
8090349	0,26	1,20	0,58655	-0,16	-1,11	0,35527	--- MTHFD1	
7975045	0,26	1,20	0,65974	2,44	5,44	0,00028	NM_005956	PGBD2
7911301	0,26	1,20	0,13088	-0,15	-1,11	0,36022	NM_170725	HSP90AA2
7947245	0,26	1,20	0,60441	-0,29	-1,22	0,29627	M30627 CREM	
7926983	0,26	1,19	0,18502	-0,02	-1,01	0,90506	NM_183013	WDR92
8052703	0,26	1,19	0,14462	0,64	1,55	0,06518	NM_138458	ITGA8
7932254	0,26	1,19	0,68484	-0,98	-1,98	0,02854	NM_003638	RAP2B
8083457	0,26	1,19	0,53009	1,21	2,31	0,04865	NM_002886	PLA2G1B
7967034	0,26	1,19	0,33672	-0,49	-1,41	0,25070	NM_000928	URB1
8069943	0,26	1,19	0,31538	1,93	3,82	0,00149	NM_014825	KIF26B
7911114	0,26	1,19	0,38395	-1,08	-2,11	0,00433	NM_018012	---
8102875	0,26	1,19	0,71328	-0,56	-1,48	0,18669	--- HBEGF	
8114572	0,26	1,19	0,51839	2,08	4,22	0,00222	NM_001945	KIAA0892
8027184	0,26	1,19	0,59906	1,83	3,55	0,02850	NM_015329	NR1D1
8014956	0,26	1,19	0,57201	0,96	1,95	0,01959	NM_021724	DCDC2
8124196	0,25	1,19	0,12124	-0,91	-1,88	0,00665	NM_016356	ENTPD5
7980080	0,25	1,19	0,64146	2,30	4,92	0,00070	NM_001249	KLHL29
8040490	0,25	1,19	0,09943	0,71	1,63	0,07496	NM_052920	PFDN4
8063453	0,25	1,19	0,11321	0,14	1,11	0,45017	NM_002623	C6orf182
8121392	0,25	1,19	0,36063	-0,11	-1,08	0,44836	NM_001083535	SRGAP2
7909175	0,25	1,19	0,56923	3,28	9,69	0,00029	NM_015326	LOC284757
8063793	0,25	1,19	0,53098	-1,16	-2,24	0,00522	AK128288 FAM19A1	
8080918	0,25	1,19	0,65747	-0,97	-1,96	0,03424	NM_213609	HIST4H4
7961483	0,25	1,19	0,15766	0,03	1,02	0,90811	NM_175054	MAST4
8105741	0,25	1,19	0,27797	0,45	1,36	0,05097	NM_001164664	RNF150
8102938	0,25	1,19	0,35939	0,35	1,28	0,03557	NM_020724	TCN2
8072360	0,25	1,19	0,50901	0,49	1,41	0,08317	NM_000355	CDC7
7902913	0,25	1,19	0,39801	1,48	2,79	0,04008	NM_003503	NAB1
8047086	0,25	1,19	0,61834	1,48	2,80	0,00076	NM_005966	LRAT
8097920	0,25	1,19	0,09816	-1,05	-2,07	0,03922	NM_004744	RNF185

8072454	0,25	1,19	0,51771	0,54	1,46	0,23327	NR_024210	CCNE1
8027402	0,25	1,19	0,15031	2,82	7,06	0,00004	NM_001238	THAP11
7996675	0,25	1,19	0,47192	1,74	3,33	0,01434	NM_020457	ZNF600
8038989	0,25	1,19	0,38549	1,27	2,41	0,00664	NM_198457	---
7906305	0,25	1,19	0,57385	1,42	2,67	0,04095	--- INPP4A	
8043791	0,25	1,19	0,67544	1,98	3,94	0,01012	NM_001134225	ZNF286A
8005110	0,25	1,19	0,45488	1,85	3,61	0,00220	NM_020652	WDR63
7902660	0,25	1,19	0,35340	-1,11	-2,15	0,00372	NM_145172	LMO4
7902810	0,25	1,19	0,42761	0,75	1,69	0,01385	NM_006769	LOC441601
7948092	0,25	1,19	0,56644	-0,08	-1,05	0,58658	NR_003034	KIAA1949
8124798	0,25	1,19	0,27772	1,21	2,31	0,02810	NM_133471	PPP1R3B
8149264	0,25	1,19	0,10096	0,95	1,93	0,00350	NM_024607	MYH11
7999674	0,25	1,19	0,43507	-0,95	-1,94	0,02054	NM_022844	C2orf86
8052598	0,25	1,19	0,55600	1,78	3,44	0,02254	NM_015910	HIST1H2AC
8117372	0,25	1,19	0,56576	1,69	3,23	0,00648	NM_003512	SLITRK4
8175574	0,25	1,19	0,30552	-0,69	-1,61	0,16759	NM_173078	ALDH3B1
7941961	0,25	1,19	0,32294	2,09	4,24	0,03497	NM_000694	MCM8
8060813	0,25	1,19	0,63371	2,69	6,46	0,00010	NM_032485	hCG_1990547
8144503	0,25	1,19	0,51716	-0,68	-1,60	0,28605	ENST00000310542	---
7916225	0,25	1,19	0,30285	0,30	1,23	0,20983	--- DAXX	
8125734	0,25	1,19	0,50881	-0,19	-1,14	0,50173	NM_001141969	CNKSR2
8166355	0,25	1,19	0,63306	-1,31	-2,48	0,00016	NM_014927	SSR4
8170775	0,25	1,19	0,60536	0,04	1,03	0,87232	NM_006280	ZNF778
7997904	0,25	1,19	0,91228	0,73	1,65	0,24886	AK295122 TPTE2P3	
7969256	0,25	1,19	0,50356	-0,13	-1,09	0,37748	NR_002793	VDR
7962689	0,25	1,19	0,48362	1,03	2,04	0,03943	NM_001017535	CCDC94
8024798	0,25	1,19	0,59086	1,55	2,93	0,00074	NM_018074	HTR2A
7971526	0,24	1,19	0,22021	-1,17	-2,25	0,06679	NM_000621	C3orf52
8081645	0,24	1,18	0,32915	0,49	1,40	0,10957	NM_024616	STUB1
7992021	0,24	1,18	0,60005	0,65	1,56	0,04702	NM_005861	DTD1
8061211	0,24	1,18	0,54846	0,90	1,86	0,23834	NM_080820	EP400
7959995	0,24	1,18	0,25256	2,49	5,60	0,00004	NM_015409	DIS3L2
8049097	0,24	1,18	0,69745	0,58	1,49	0,23842	NM_152383	IQUB
8142646	0,24	1,18	0,41647	-0,72	-1,64	0,00302	NM_178827	HSPC072
8065242	0,24	1,18	0,30124	-1,53	-2,89	0,10228	AF161557 ZNF215	
7938183	0,24	1,18	0,29727	0,53	1,44	0,20377	NM_013250	GK5
8091120	0,24	1,18	0,40446	1,95	3,86	0,00649	NR_033289	SGCZ
8149438	0,24	1,18	0,14181	-1,06	-2,09	0,01632	NM_139167	FAM129B
8164217	0,24	1,18	0,37040	2,08	4,22	0,00862	NM_022833	NACAD
8139460	0,24	1,18	0,62037	-1,10	-2,14	0,00001	NM_001146334	CHD6
8066303	0,24	1,18	0,50885	1,18	2,26	0,00855	NM_032221	MGC4294
8063634	0,24	1,18	0,63730	-1,27	-2,41	0,01020	ENST00000419204	ABTB2
7947462	0,24	1,18	0,61621	2,50	5,65	0,00182	NM_145804	PPAPDC2
8154151	0,24	1,18	0,54964	1,01	2,01	0,03245	NM_203453	MT4
7995772	0,24	1,18	0,61629	-0,06	-1,05	0,62471	NM_032935	ESM1
8112045	0,24	1,18	0,59201	-0,94	-1,92	0,05097	NM_007036	NCAPD3
7952830	0,24	1,18	0,43672	3,16	8,92	0,00116	NM_015261	FAM124A
7969192	0,24	1,18	0,26751	0,63	1,55	0,00315	BC051771 C21orf128	
8070563	0,24	1,18	0,71533	-0,60	-1,52	0,03565	NR_027243	C6orf170
8129273	0,24	1,18	0,16260	-0,38	-1,30	0,22214	NM_152730	ANO5
7938951	0,24	1,18	0,68780	-1,16	-2,23	0,01401	NM_213599	---
8112666	0,24	1,18	0,67403	-0,09	-1,07	0,79310	--- PTOV1	
8030525	0,24	1,18	0,43802	1,30	2,46	0,02023	NM_017432	SV2A
7919645	0,24	1,18	0,55500	1,71	3,28	0,00952	NM_014849	TMEM64
8151747	0,24	1,18	0,55231	1,64	3,12	0,00098	NM_001008495	BEX1
8174201	0,24	1,18	0,48467	-0,92	-1,90	0,05560	NM_018476	---
8065633	0,24	1,18	0,35920	0,53	1,45	0,08945	--- NOP14	
8099051	0,24	1,18	0,54386	1,71	3,28	0,00087	NM_003703	RPUSD2
7982753	0,24	1,18	0,41931	0,78	1,72	0,31328	NM_152260	GPATCH1
8027521	0,24	1,18	0,19235	0,78	1,72	0,03068	NM_018025	MAP1LC3A
8062023	0,24	1,18	0,64580	-0,29	-1,23	0,48650	NM_032514	LUC7L3
8008493	0,24	1,18	0,55866	1,73	3,31	0,00040	NM_016424	LOC81691
7993776	0,24	1,18	0,28723	0,00	1,00	0,99725	NM_030941	SAT2
8012247	0,24	1,18	0,51441	1,80	3,47	0,00102	NM_133491	---
7913154	0,24	1,18	0,62303	2,12	4,34	0,00097	--- RCN3	
8030383	0,24	1,18	0,49465	0,22	1,16	0,57907	NM_020650	RDH14
8050474	0,24	1,18	0,54681	1,95	3,87	0,00038	NM_020905	HIST1H1A
8124380	0,24	1,18	0,63686	-0,77	-1,71	0,01935	NM_005325	MRI1
8026182	0,24	1,18	0,51453	2,33	5,03	0,00080	NM_001031727	KIAA0513
7997662	0,23	1,18	0,55709	0,16	1,12	0,54632	NM_014732	ST7L
7918569	0,23	1,18	0,44100	0,29	1,22	0,38021	NM_017744	AIMP1
8096718	0,23	1,18	0,34260	0,30	1,24	0,05761	NM_004757	ZNF549
8031762	0,23	1,18	0,45834	0,49	1,40	0,08538	NM_153263	KLHL3

8114300	0,23	1,18	0,55513	-0,16	-1,12	0,80287	NM_017415	XPC
8085486	0,23	1,18	0,26232	0,02	1,01	0,92307	NM_004628	HYDIN
8002481	0,23	1,18	0,68564	-2,33	-5,03	0,00004	NM_032821	TLE2
8032553	0,23	1,18	0,61174	1,54	2,90	0,04949	NM_003260	SPATA20
8008388	0,23	1,18	0,70082	1,92	3,78	0,00708	NM_022827	LOC100129449
8055911	0,23	1,18	0,43406	-0,76	-1,69	0,03812	ENST00000428580	CLN8
8144279	0,23	1,17	0,33928	0,71	1,63	0,00551	NM_018941	OR2T35
7925757	0,23	1,17	0,74412	-1,91	-3,76	0,00077	NM_001001827	ZNF414
8033598	0,23	1,17	0,59638	0,14	1,10	0,73813	NM_032370	IL26
7964795	0,23	1,17	0,53531	-0,35	-1,27	0,30774	NM_018402	GRPEL2
8109141	0,23	1,17	0,68367	1,42	2,67	0,01272	NM_152407	ZNF124
7925691	0,23	1,17	0,38383	0,88	1,85	0,04230	NM_003431	PLD2
8003962	0,23	1,17	0,67651	2,26	4,79	0,00353	NM_002663	RBM3
8167234	0,23	1,17	0,25467	0,00	1,00	0,98676	NM_006743	C22orf42
8075555	0,23	1,17	0,53597	-1,10	-2,14	0,00531	NM_001010859	C1orf91
7914500	0,23	1,17	0,64350	0,08	1,06	0,39566	AF258548 MTSS1L	
8002403	0,23	1,17	0,70600	1,72	3,31	0,01576	NM_138383	LOC644936
8112883	0,23	1,17	0,35751	-0,13	-1,09	0,74381	NR_004845	ZNF187
8117667	0,23	1,17	0,42112	1,93	3,81	0,01254	NM_001023560	WBP1
8042867	0,23	1,17	0,10299	1,98	3,96	0,00739	NM_012477	SNORA61
7914212	0,23	1,17	0,65035	2,14	4,41	0,00534	NR_002987	AK2
7914617	0,23	1,17	0,69688	2,14	4,40	0,00101	NM_013411	NDUFA5
8142663	0,23	1,17	0,65430	-0,31	-1,24	0,35259	NM_005000	C9orf119
8158204	0,23	1,17	0,23187	1,13	2,18	0,05577	NM_001040011	RPL39L
8092654	0,23	1,17	0,19518	0,64	1,55	0,07347	NM_052969	C18orf45
8022514	0,23	1,17	0,57146	1,86	3,64	0,01240	NM_032933	FIGF
8171427	0,23	1,17	0,34087	-0,59	-1,50	0,08488	NM_004469	THOC6
7992795	0,23	1,17	0,70826	2,57	5,93	0,00510	NM_024339	BAT2L1
8158839	0,23	1,17	0,41509	3,10	8,56	0,00518	NM_013318	RREB1
8116740	0,23	1,17	0,35090	2,08	4,23	0,00051	NM_001168344	MTHFSD
8003233	0,23	1,17	0,58403	0,61	1,52	0,05149	NM_001159377	HIST1H4J
8117598	0,23	1,17	0,02333	0,96	1,95	0,02374	NM_021968	RNF168
8093130	0,23	1,17	0,54691	0,91	1,88	0,04941	NM_152617	LRRC47
7911862	0,23	1,17	0,49875	1,07	2,10	0,07948	NM_020710	SENP3
8004485	0,23	1,17	0,10027	1,43	2,70	0,00233	NM_015670	PSMG3
8137798	0,23	1,17	0,76367	2,76	6,80	0,00024	NM_032302	MAD1L1
8137805	0,23	1,17	0,12608	-0,33	-1,26	0,35520	NM_003550	POLA1
8166525	0,23	1,17	0,36258	1,84	3,58	0,00137	NM_016937	DAXX
8178991	0,23	1,17	0,53315	-0,37	-1,29	0,32145	NM_001141969	HMGXB3
8109201	0,23	1,17	0,32164	2,00	3,99	0,00420	NM_014983	KIF18A
7947248	0,23	1,17	0,41241	1,60	3,04	0,00384	NM_031217	ZNF341
8061946	0,23	1,17	0,70309	0,17	1,13	0,14808	NM_032819	GPKOW
8172548	0,23	1,17	0,51663	1,17	2,25	0,06519	NM_015698	MED11
8003922	0,23	1,17	0,33729	1,58	2,99	0,01194	NM_001001683	ST6GAL1
8084717	0,22	1,17	0,68586	2,71	6,54	0,00004	NM_173216	C19orf47
8036867	0,22	1,17	0,52867	0,31	1,24	0,36650	BC027935 AQP4	
8022655	0,22	1,17	0,38216	-0,70	-1,62	0,04038	NM_001650	C7orf64
8134211	0,22	1,17	0,45123	0,84	1,79	0,12035	NM_032120	TRAF4
8005978	0,22	1,17	0,52328	1,96	3,90	0,00019	NM_004295	HDAC7
7962659	0,22	1,17	0,75536	0,45	1,37	0,25267	NM_015401	C16orf45
7993458	0,22	1,17	0,61961	1,04	2,06	0,04343	NM_033201	SHB
8161255	0,22	1,17	0,53355	0,57	1,49	0,11209	NM_003028	CECR1
8074237	0,22	1,17	0,73030	-1,27	-2,42	0,00945	NM_017424	CLEC2D
7953878	0,22	1,17	0,27411	0,09	1,06	0,68630	NM_001004419	SMCHD1
8019877	0,22	1,17	0,51304	2,51	5,71	0,00011	NM_015295	PHF7
8080306	0,22	1,17	0,42780	1,35	2,54	0,00436	NM_016483	MIR330
8037640	0,22	1,17	0,48408	-0,60	-1,52	0,08893	NR_029886	ACSF2
8008321	0,22	1,17	0,62052	1,35	2,54	0,02022	NM_025149	COMMD9
7947531	0,22	1,17	0,64950	1,42	2,68	0,00095	NM_014186	HK1
7928019	0,22	1,17	0,51154	0,07	1,05	0,85724	NM_033500	KIAA0182
7997680	0,22	1,17	0,62452	1,59	3,01	0,00029	NM_014615	EXD2
7975311	0,22	1,17	0,35004	1,63	3,09	0,01455	NM_018199	TMEM134
7949798	0,22	1,17	0,73536	0,66	1,58	0,07959	NM_025124	MKI67
7937020	0,22	1,17	0,04533	1,56	2,96	0,00700	NM_002417	UBIAD1
7897685	0,22	1,17	0,49252	2,63	6,18	0,00084	NM_013319	TMEM203
8165486	0,22	1,17	0,54265	2,87	7,29	0,00039	NM_053045	DSTYK
7923731	0,22	1,17	0,39973	0,48	1,39	0,06679	NM_015375	---
8111913	0,22	1,17	0,69022	-0,94	-1,92	0,01714	--- LARP7	
8096938	0,22	1,17	0,46306	0,79	1,73	0,02841	NM_016648	LYSMD1
7919923	0,22	1,17	0,66645	0,49	1,40	0,23740	NM_212551	EEF1D
8153449	0,22	1,16	0,38213	0,85	1,80	0,06312	NM_032378	ASB13
7931863	0,22	1,16	0,56609	2,17	4,51	0,02772	NM_024701	LIN9
7924712	0,22	1,16	0,48996	1,86	3,62	0,01438	NM_173083	RANGAP1

8076307	0,22	1,16	0,59913	2,06	4,18	0,00583	NM_002883	STK35
8060427	0,22	1,16	0,67804	1,61	3,05	0,00249	NM_080836	NDUFA11
8033069	0,22	1,16	0,53211	0,19	1,14	0,53950	NM_175614	STXBP6
7978376	0,22	1,16	0,37527	-0,77	-1,71	0,00343	NM_014178	CES1
8001457	0,22	1,16	0,79348	-2,21	-4,61	0,00034	NM_001025195	EIF2B5
8084303	0,22	1,16	0,57232	0,86	1,81	0,00948	NM_003907	HCFC1
8175947	0,22	1,16	0,53754	3,21	9,25	0,00263	NM_005334	BCOR
8172088	0,22	1,16	0,34835	2,07	4,20	0,00013	NM_001123385	COPS5
8151136	0,22	1,16	0,37307	1,78	3,44	0,01947	NM_006837	GABRB3
7986822	0,22	1,16	0,36953	-1,11	-2,16	0,05004	NM_000814	GALNT4
7965357	0,22	1,16	0,65259	0,43	1,35	0,08607	NM_003774	PRO2012
7924817	0,22	1,16	0,43168	-0,49	-1,40	0,05970	BC019830 CHRDL1	
8174513	0,22	1,16	0,52588	-1,65	-3,15	0,00416	NM_145234	FAM128B
8045142	0,22	1,16	0,23705	0,00	-1,00	0,99764	NM_025029	USP27X
8167601	0,22	1,16	0,66055	1,17	2,25	0,13028	NM_001145073	PKIG
8062852	0,22	1,16	0,62322	-0,63	-1,55	0,01040	NM_181805	OR4M2
7981781	0,22	1,16	0,76477	-1,35	-2,54	0,12875	NM_001004719	DCUN1D1
8092321	0,22	1,16	0,45665	1,14	2,20	0,01161	NM_020640	---
7924069	0,22	1,16	0,37423	0,00	1,00	0,99948	--- GAL3ST4	
8141459	0,22	1,16	0,66429	-1,30	-2,46	0,05531	NM_024637	---
7955635	0,22	1,16	0,24263	-0,07	-1,05	0,81113	--- DYNC1I1	
8134384	0,21	1,16	0,43678	-0,84	-1,79	0,01718	NM_004411	NR0B1
8171892	0,21	1,16	0,23311	1,73	3,31	0,01091	NM_000475	PHF10
8130916	0,21	1,16	0,53313	1,06	2,09	0,03244	NM_018288	SATB1
8085716	0,21	1,16	0,57866	-0,78	-1,72	0,11217	NM_002971	TUBA4A
8059177	0,21	1,16	0,29439	0,78	1,71	0,02813	NM_006000	ZNF354B
8110463	0,21	1,16	0,44472	1,69	3,23	0,00826	NM_058230	RGS5
7921916	0,21	1,16	0,64828	-0,25	-1,19	0,07184	NM_003617	DLEU1
7969171	0,21	1,16	0,61196	2,71	6,54	0,00039	NR_002605	SMTN
8072413	0,21	1,16	0,41012	0,08	1,06	0,86288	NM_134269	LIPT2
7950420	0,21	1,16	0,69274	1,99	3,97	0,01389	NM_001144869	CXorf19
8170213	0,21	1,16	0,57167	-0,25	-1,19	0,46593	ENST00000309296	ZNF669
7925677	0,21	1,16	0,46675	0,01	1,01	0,93826	NM_024804	IPCEF1
8130408	0,21	1,16	0,51573	-0,73	-1,66	0,00728	NM_001130700	LOC388955
8052622	0,21	1,16	0,70715	0,50	1,42	0,29671	NR_003131	PNPLA6
8025199	0,21	1,16	0,18841	1,04	2,06	0,14233	NM_006702	SHCBP1
8001133	0,21	1,16	0,16190	3,02	8,11	0,00043	NM_024745	FDX1
7943721	0,21	1,16	0,27056	1,90	3,74	0,01062	NM_004109	C21orf94
8022436	0,21	1,16	0,73709	-0,63	-1,55	0,36843	NR_027246	ZNF618
8157381	0,21	1,16	0,61242	2,23	4,70	0,00597	NM_133374	C4orf46
8103431	0,21	1,16	0,53404	0,35	1,27	0,56323	NM_001008393	ZFY
8176384	0,21	1,16	0,48823	0,91	1,88	0,00438	NM_003411	LCMT2
7988077	0,21	1,16	0,12960	0,85	1,80	0,02518	NM_014793	TMEM215
8154760	0,21	1,16	0,59393	-0,39	-1,31	0,21467	NM_212558	GTF3C5
8158961	0,21	1,16	0,63043	1,49	2,81	0,00397	NM_001122823	HSPG2
7913450	0,21	1,16	0,40364	0,79	1,72	0,13585	NM_005529	NBL1
7898585	0,21	1,15	0,28675	-0,31	-1,24	0,50771	NM_182744	POLR1C
8119842	0,21	1,15	0,63818	2,26	4,79	0,00151	NM_203290	---
8041170	0,21	1,15	0,64640	0,42	1,34	0,55078	--- IL32	
7992828	0,21	1,15	0,57696	-0,01	-1,01	0,97373	NM_001012631	CDYL2
8002975	0,21	1,15	0,25832	-0,86	-1,82	0,01490	NM_152342	C17orf60
8009243	0,21	1,15	0,38298	0,36	1,28	0,05752	NM_001085423	---
8144719	0,21	1,15	0,56445	-1,04	-2,05	0,12483	--- KRTAP1-3	
8015189	0,21	1,15	0,47243	-1,78	-3,43	0,00394	NM_030966	KRTAP1-3
8019585	0,21	1,15	0,47243	-1,78	-3,43	0,00394	NM_030966	ZNF57
8024532	0,21	1,15	0,55554	1,21	2,31	0,00031	NM_173480	TDRKH
7920057	0,21	1,15	0,38184	2,64	6,23	0,00007	NM_001083965	FAM200B
8094226	0,21	1,15	0,59282	0,77	1,71	0,06773	NM_001145191	PNO1
8042381	0,21	1,15	0,54189	0,95	1,93	0,02053	NM_020143	THAP4
8060274	0,21	1,15	0,05925	0,24	1,18	0,13548	NM_015963	THAP4
8077250	0,21	1,15	0,05925	0,24	1,18	0,13548	NM_015963	---
8050869	0,21	1,15	0,45878	-0,92	-1,90	0,13047	--- ---	
8127696	0,21	1,15	0,82359	-2,65	-6,29	0,07784	--- ---	
8149200	0,21	1,15	0,82359	-2,65	-6,29	0,07784	--- NPL	
7908003	0,21	1,15	0,41324	0,30	1,23	0,35536	NM_030769	HIST1H4D
8124413	0,20	1,15	0,32156	0,46	1,38	0,17977	NM_003539	ZNF552
8031825	0,20	1,15	0,78939	-0,18	-1,13	0,81214	NM_024762	---
8084945	0,20	1,15	0,38524	-0,43	-1,35	0,20279	--- PHF17	
8097417	0,20	1,15	0,64494	1,12	2,17	0,00077	NM_199320	ZNF189
8156935	0,20	1,15	0,65190	0,74	1,68	0,03594	NM_003452	PFKFB2
7909285	0,20	1,15	0,33404	2,00	3,99	0,00449	NM_006212	KLHL22
8074632	0,20	1,15	0,68944	0,83	1,77	0,05360	NM_032775	MPPED2
7947274	0,20	1,15	0,50827	-1,20	-2,30	0,03312	NM_001584	ZNF235

8037444	0,20	1,15	0,35743	0,19	1,14	0,48972	NM_004234	C22orf24
8075550	0,20	1,15	0,65937	-0,19	-1,14	0,58899	AK294232 NUF2	
7906930	0,20	1,15	0,38634	2,06	4,18	0,00346	NM_145697	B3GALT2
7923034	0,20	1,15	0,47339	-1,07	-2,10	0,01169	NM_003783	OSGEP
7977571	0,20	1,15	0,17469	0,22	1,16	0,19764	NM_017807	DHRS11
8006655	0,20	1,15	0,64157	2,98	7,90	0,00408	NM_024308	C2orf24
8059097	0,20	1,15	0,10198	0,48	1,39	0,09814	NM_015680	---
8009351	0,20	1,15	0,34968	-0,48	-1,40	0,22416	--- GPR85	
8142424	0,20	1,15	0,68374	-0,91	-1,88	0,05564	NM_001146265	CCR10
8015681	0,20	1,15	0,71828	-0,73	-1,66	0,11293	NM_016602	NVL
7924558	0,20	1,15	0,39837	1,60	3,03	0,00419	NM_002533	ENDOU
7962608	0,20	1,15	0,73061	-1,14	-2,20	0,06074	NM_001172439	ALDOAP2
7936923	0,20	1,15	0,69696	0,53	1,44	0,00704	M21191 ZBTB4	
8012212	0,20	1,15	0,16971	0,93	1,91	0,07649	NM_020899	KIF22
7994620	0,20	1,15	0,59262	1,95	3,87	0,00189	NM_007317	---
8148930	0,20	1,15	0,56890	-0,13	-1,10	0,50899	--- CP110	
7993664	0,20	1,15	0,51704	2,39	5,23	0,00172	NM_014711	CPNE8
7962250	0,20	1,15	0,42466	1,92	3,79	0,00051	NM_153634	NDUFA1
8169659	0,20	1,15	0,61193	-0,51	-1,42	0,29534	NM_004541	IRF9
7973618	0,20	1,15	0,43038	0,92	1,90	0,09081	NM_006084	DPY19L2P1
8138988	0,20	1,15	0,71700	0,11	1,08	0,84620	NR_002833	MICAL1
8128737	0,20	1,15	0,28743	0,21	1,16	0,14858	NM_022765	MGC72080
8138151	0,20	1,15	0,73372	-0,14	-1,10	0,79392	NR_002822	PCDHB17
8108706	0,20	1,15	0,69075	-0,68	-1,60	0,03526	NR_001280	CCDC101
7994362	0,20	1,15	0,66124	1,18	2,26	0,00053	NM_138414	---
7995438	0,20	1,15	0,82883	-0,03	-1,02	0,87608	--- CLN8	
8144267	0,20	1,15	0,44530	0,50	1,41	0,00437	NM_018941	FDPSL2A // FDPSL2A // FDPSL2A // FDPSL2A // FDPSL2A // FDPSL2A // FDPSL2A // FDPSL2A
8140443	0,20	1,15	0,34927	0,42	1,34	0,12514	NR_003262 // NR_003262 // NR_003262 // NR_003262 // NR_003262 // NR_003262 // NR_003262 // NR_003262	ELAC1
8021222	0,20	1,15	0,69085	-0,56	-1,48	0,29191	NM_018696	SELK
8088167	0,20	1,15	0,44831	1,48	2,78	0,08761	NM_021237	RAB43
8001147	0,20	1,14	0,55606	1,71	3,27	0,04146	NM_198490	---
7981317	0,19	1,14	0,55290	-0,18	-1,14	0,43067	--- DZIP3	
8081503	0,19	1,14	0,55118	1,11	2,15	0,05414	NM_014648	LOC554223
8117773	0,19	1,14	0,66415	0,09	1,07	0,79199	AK128290 S100A3	
7920278	0,19	1,14	0,71553	0,09	1,06	0,79628	NM_002960	RBKS
8051215	0,19	1,14	0,31504	1,83	3,55	0,00101	NM_022128	HYAL2
8087624	0,19	1,14	0,41359	0,04	1,03	0,80839	NM_033158	GLT25D1
8026806	0,19	1,14	0,54743	1,76	3,38	0,01715	NM_024656	---
8134349	0,19	1,14	0,14224	0,06	1,04	0,87430	--- MRPL24	
7921121	0,19	1,14	0,64129	1,64	3,11	0,01405	NM_145729	NDST2
7934459	0,19	1,14	0,52292	1,46	2,75	0,02000	NM_003635	TTK
8120838	0,19	1,14	0,47945	1,36	2,57	0,03534	NM_003318	SPTY2D1
7947040	0,19	1,14	0,14711	0,10	1,07	0,63414	NM_194285	KCTD21
7942796	0,19	1,14	0,48595	-0,11	-1,08	0,71246	NM_001029859	TBC1D12
7929424	0,19	1,14	0,60151	1,44	2,72	0,00706	NM_015188	---
8009322	0,19	1,14	0,72177	-0,69	-1,61	0,17380	--- FOXRED1	
7945071	0,19	1,14	0,58479	1,91	3,75	0,03175	NM_017547	TCEAL6
8174134	0,19	1,14	0,67045	1,03	2,04	0,00108	NM_001006938	DYNC1I2
8046340	0,19	1,14	0,70166	0,13	1,10	0,66773	NM_001378	MED16
8032106	0,19	1,14	0,62486	0,68	1,60	0,31944	NM_005481	DNAJC2
8141898	0,19	1,14	0,54618	0,85	1,81	0,02503	NM_014377	C20orf12
8065202	0,19	1,14	0,29504	-0,04	-1,03	0,43287	NM_001099407	PLA2G15
7996761	0,19	1,14	0,61204	2,07	4,19	0,04543	NM_012320	TRAF7
7992529	0,19	1,14	0,74878	1,83	3,55	0,00397	NM_032271	SDCCAG3
8165156	0,19	1,14	0,65781	3,11	8,64	0,00006	NM_001039707	SERPINI1
8083779	0,19	1,14	0,57005	0,06	1,04	0,87653	NM_001122752	RSF1
7950606	0,19	1,14	0,67381	1,07	2,10	0,00443	NM_016578	N4BP2
8094719	0,19	1,14	0,47557	1,69	3,22	0,00507	NM_018177	LOC400804
7924445	0,19	1,14	0,59127	-1,22	-2,32	0,03810	AK128488 DPH1	
8003700	0,19	1,14	0,34267	0,60	1,51	0,12243	NM_001383	USP19
8087308	0,19	1,14	0,67264	1,74	3,35	0,02366	NM_006677	ADPRHL2
7900087	0,19	1,14	0,70139	0,09	1,07	0,69740	NM_017825	ZNF433
8034320	0,19	1,14	0,63991	-0,45	-1,37	0,18196	NM_001080411	MAML3
8102862	0,19	1,14	0,40310	0,36	1,28	0,47413	NM_018717	C8orf4
8146115	0,19	1,14	0,26799	-0,15	-1,11	0,14893	NM_020130	NFIL3
8162276	0,19	1,14	0,33337	0,99	1,98	0,02523	NM_005384	ELMOD3
8043131	0,19	1,14	0,59753	0,14	1,10	0,59822	NM_001135021	TUBB8
8021919	0,19	1,14	0,72895	1,03	2,05	0,02009	NM_177987	TSPAN13
8131600	0,19	1,14	0,52512	2,50	5,66	0,00005	NM_014399	ASS1
8158671	0,19	1,14	0,51411	2,64	6,23	0,00467	NM_000050	MX2
8068697	0,19	1,14	0,69448	-1,47	-2,76	0,01864	NM_002463	PHYHD1

8158431	0,19	1,14	0,74560	1,11	2,15	0,00018	NM_001100876	RAD51AP1
7953218	0,19	1,14	0,03572	1,38	2,61	0,00099	NM_001130862	ASXL1
8061725	0,19	1,14	0,40915	2,21	4,62	0,00057	NM_015338	FOXK2
8010820	0,19	1,14	0,70819	2,56	5,88	0,00957	NM_004514	TATDN1
8152759	0,18	1,14	0,30279	1,02	2,02	0,25147	NR_027427	SH3GL1
8032804	0,18	1,14	0,53731	2,39	5,24	0,02300	NM_003025	---
8155248	0,18	1,14	0,57410	-1,18	-2,27	0,21294	---	MAP1LC3B
7997740	0,18	1,14	0,74805	-0,24	-1,18	0,64217	NM_022818	PCDHB14
8108744	0,18	1,14	0,63136	1,64	3,11	0,00962	NM_018934	FAT3
7943075	0,18	1,13	0,61463	-1,50	-2,84	0,00012	NM_001008781	---
7935054	0,18	1,13	0,58459	0,15	1,11	0,52332	---	MESDC2
7990830	0,18	1,13	0,51552	1,62	3,06	0,01391	NM_015154	MAN1C1
7899029	0,18	1,13	0,59784	-1,16	-2,24	0,01694	NM_020379	TNFAIP8L3
7988763	0,18	1,13	0,75201	-1,41	-2,66	0,00384	NM_207381	CWF19L1
7935730	0,18	1,13	0,52053	1,51	2,85	0,00006	NM_018294	ZNF175
8030831	0,18	1,13	0,67487	0,99	1,99	0,05603	NM_007147	SSSCA1
7941296	0,18	1,13	0,62665	2,03	4,09	0,00468	NM_006396	NTM
7945245	0,18	1,13	0,67754	-1,24	-2,36	0,00313	NM_016522	LOC348926
8099084	0,18	1,13	0,80708	0,94	1,92	0,26156	NR_024253	SNORD49A
8005202	0,18	1,13	0,32334	-0,37	-1,30	0,06343	NR_002744	PCSK5
8155898	0,18	1,13	0,49144	-0,09	-1,06	0,73665	NM_006200	---
8082570	0,18	1,13	0,44511	-0,44	-1,36	0,06573	---	SGCG
7968004	0,18	1,13	0,71889	-0,60	-1,52	0,04695	NM_000231	SPAM1
8135835	0,18	1,13	0,31575	-1,25	-2,37	0,00655	NM_003117	PQBP1
8167412	0,18	1,13	0,59965	0,98	1,97	0,02395	NM_005710	---
8132913	0,18	1,13	0,18733	-1,15	-2,22	0,00511	---	MCM3
8127031	0,18	1,13	0,64318	3,87	14,64	0,00013	NM_002388	SAMD4A
7974425	0,18	1,13	0,65795	0,32	1,25	0,26421	NM_015589	ARL13B
8081115	0,18	1,13	0,74853	1,32	2,49	0,00726	NM_001174150	ATP6V0B
7900922	0,18	1,13	0,65671	1,37	2,58	0,02724	NM_004047	RFXANK
8027139	0,18	1,13	0,79901	0,84	1,79	0,01575	NM_003721	GTPBP1
8073039	0,18	1,13	0,77727	0,86	1,82	0,04611	NM_004286	SLC7A14
8092053	0,18	1,13	0,71122	-0,80	-1,74	0,01999	NM_020949	SPRYD5
7939879	0,18	1,13	0,64646	-0,45	-1,37	0,21862	NM_032681	GSTO2
7930311	0,18	1,13	0,73521	2,37	5,15	0,01060	NM_183239	---
8072584	0,18	1,13	0,63126	1,22	2,33	0,01324	---	CCDC112
8113602	0,18	1,13	0,30903	0,45	1,37	0,07345	NM_152549	MEN1
7949206	0,18	1,13	0,55604	1,87	3,64	0,00357	NM_130802	QSOX2
8165094	0,18	1,13	0,20878	2,15	4,45	0,00588	NM_181701	XRRA1
7950447	0,18	1,13	0,25979	0,38	1,30	0,31709	NM_182969	---
8151308	0,18	1,13	0,79034	0,56	1,47	0,12560	---	PEF1
7914354	0,18	1,13	0,76418	0,31	1,24	0,52622	NM_012392	WDR53
8093141	0,18	1,13	0,66559	1,96	3,88	0,00001	NM_182627	VAMP5
8043203	0,17	1,13	0,83658	-1,40	-2,63	0,13050	NM_006634	DNAJB14
8101934	0,17	1,13	0,78251	0,27	1,21	0,65134	NM_001031723	FAM82A2
7987536	0,17	1,13	0,40326	1,87	3,64	0,00523	NM_018145	FAM53B
7936884	0,17	1,13	0,66340	0,39	1,31	0,41031	NM_014661	SMAD9
7971015	0,17	1,13	0,48881	-0,15	-1,11	0,75009	NM_001127217	NDUFB2
8136602	0,17	1,13	0,49696	0,51	1,43	0,08985	NM_004546	LGALS9
8005809	0,17	1,13	0,64619	-0,46	-1,38	0,08142	NM_009587	CNKSR3
8130422	0,17	1,13	0,57588	2,50	5,66	0,00022	NM_173515	LHPP
7931204	0,17	1,13	0,42924	1,84	3,58	0,00142	NM_022126	ARHGDIA
8019765	0,17	1,13	0,78329	2,24	4,73	0,03033	NM_004309	SH3BP5L
7925763	0,17	1,13	0,44353	0,15	1,11	0,78320	NM_030645	ZZEF1
8011542	0,17	1,13	0,33313	2,15	4,45	0,00200	NM_015113	RNMTL1
8003621	0,17	1,13	0,73367	1,21	2,32	0,01592	NM_018146	---
8139031	0,17	1,13	0,70185	-1,84	-3,59	0,00035	---	ARSG
8009443	0,17	1,13	0,27970	0,93	1,91	0,01248	NM_014960	NDUFS7
8032212	0,17	1,13	0,68304	-0,31	-1,24	0,37030	AK127759 ACYP1	
7980265	0,17	1,12	0,45008	1,31	2,47	0,01599	NM_203488	FAM72D
7904452	0,17	1,12	0,61878	2,70	6,49	0,00612	AB096683 ---	
8081217	0,17	1,12	0,56899	0,23	1,17	0,22946	---	APBB3
8114593	0,17	1,12	0,58819	2,51	5,70	0,00443	NM_133174	PPM1G
8051119	0,17	1,12	0,70983	2,03	4,09	0,00191	NM_177983	BMS1P1
7933413	0,17	1,12	0,55607	1,53	2,89	0,00038	NR_026566	C9orf6
8157144	0,17	1,12	0,75975	3,10	8,58	0,00163	NM_017832	ACOT8
8066598	0,17	1,12	0,50792	0,49	1,40	0,14917	NM_005469	FLJ16423
8170400	0,17	1,12	0,44727	0,85	1,81	0,01931	ENST00000432041	RPS2
7998655	0,17	1,12	0,77718	1,70	3,26	0,04245	NM_002952	LIX1L
7904742	0,17	1,12	0,72604	2,55	5,87	0,00423	NM_153713	FAM43A
8084891	0,17	1,12	0,40874	0,25	1,19	0,28605	NM_153690	---
7951589	0,17	1,12	0,74988	0,01	1,01	0,98592	---	DCHS1
7946245	0,17	1,12	0,81535	-1,49	-2,81	0,00846	NM_003737	CLK4

8116227	0,17	1,12	0,45271	0,38	1,30	0,19897	NM_020666	ABHD14B
8087825	0,17	1,12	0,29567	0,37	1,29	0,20368	NM_032750	TEAD2
8038347	0,17	1,12	0,62406	1,33	2,51	0,00208	NM_003598	CTBP1
8098880	0,17	1,12	0,39896	1,37	2,58	0,02061	NM_001012614	RARA
8007084	0,17	1,12	0,70288	-0,26	-1,20	0,37681	NM_000964	BLVRA
8132515	0,17	1,12	0,73495	0,81	1,75	0,08974	NM_000712	CCDC113
7996198	0,17	1,12	0,24161	1,18	2,27	0,03360	NM_014157	TOMM40L
7906728	0,17	1,12	0,21695	-0,02	-1,01	0,96466	NM_032174	INADL
7901804	0,17	1,12	0,65623	3,31	9,89	0,00001	NM_176877	---
8161693	0,16	1,12	0,44275	-0,75	-1,68	0,08838	---	PARP3
8080144	0,16	1,12	0,70322	2,19	4,55	0,00246	NM_001003931	KTELC1
8081853	0,16	1,12	0,72590	2,20	4,59	0,00167	NR_024265	ACAP3
7911458	0,16	1,12	0,61899	0,06	1,04	0,79383	NM_030649	TMEM178
8041570	0,16	1,12	0,69907	-0,71	-1,64	0,07533	NM_152390	ANP32C
8103518	0,16	1,12	0,58298	0,17	1,12	0,50680	NM_012403	GPR115
8120135	0,16	1,12	0,12217	-0,39	-1,31	0,04672	NM_153838	OSBPL5
7945831	0,16	1,12	0,60261	0,61	1,53	0,13981	NM_020896	INF2
7977249	0,16	1,12	0,70676	0,82	1,77	0,29944	NM_022489	ZNF331
8030999	0,16	1,12	0,64787	0,49	1,40	0,01370	NM_018555	ABHD3
8022488	0,16	1,12	0,52175	2,82	7,07	0,00180	NM_138340	ATRX
8176276	0,16	1,12	0,92918	1,83	3,55	0,11281	NM_000489	ENSA
7919763	0,16	1,12	0,47130	1,48	2,80	0,01005	NM_207042	IFI27L2
7981046	0,16	1,12	0,62883	0,27	1,21	0,54699	NM_032036	TUFM
8000603	0,16	1,12	0,74928	1,93	3,82	0,01704	NM_003321	IGF2BP3
8138566	0,16	1,12	0,81343	1,61	3,04	0,01913	NM_006547	MORF4
8103753	0,16	1,12	0,59591	-0,76	-1,69	0,02674	NM_006792	CRELD1
8077712	0,16	1,12	0,43495	0,90	1,86	0,00146	NM_001031717	MAD2L1
8102560	0,16	1,12	0,56256	1,70	3,25	0,00511	NM_002358	---
7942912	0,16	1,12	0,82753	-2,13	-4,38	0,03685	---	RBAK
8131292	0,16	1,12	0,63227	1,34	2,54	0,00819	NM_021163	FEM1B
7984470	0,16	1,12	0,60697	1,57	2,97	0,01512	NM_015322	SMARCA4
8025788	0,16	1,12	0,59820	-0,86	-1,82	0,01674	NM_001128849	MAGT1
8168466	0,16	1,12	0,66921	-0,44	-1,36	0,01731	NM_032121	COTL1
8003171	0,16	1,12	0,86166	0,48	1,39	0,40009	NM_021149	CTDSP1
8048304	0,16	1,12	0,37079	0,80	1,75	0,16642	NM_021198	ANAPC10
8103005	0,16	1,12	0,60259	1,08	2,12	0,05803	NM_014885	VARS
8125091	0,16	1,12	0,54278	2,00	4,00	0,00491	NM_006295	VARS
8178609	0,16	1,12	0,54278	2,00	4,00	0,00491	NM_006295	XIAP
8169742	0,16	1,12	0,48113	0,52	1,44	0,17822	NM_001167	ZNF502
8079224	0,16	1,12	0,57053	0,69	1,61	0,07700	NM_033210	ZNF197
8079204	0,16	1,12	0,21412	0,25	1,19	0,38205	NM_006991	SUV39H2
7926319	0,16	1,12	0,70233	1,01	2,02	0,01344	NM_024670	LRRC37A4
8017840	0,16	1,12	0,36091	0,73	1,66	0,06828	ENST00000443126	ZFP112
8037453	0,16	1,12	0,23921	-0,38	-1,30	0,02682	NM_001083335	CYP51A1
8140864	0,16	1,12	0,52404	0,74	1,67	0,53908	NM_000786	STAT5A
8007212	0,16	1,12	0,63970	-0,12	-1,09	0,45589	NM_003152	TMEM128
8099099	0,16	1,12	0,61690	0,97	1,96	0,01788	NM_032927	HJURP
8059838	0,16	1,12	0,23168	0,82	1,77	0,03048	NM_018410	TMEM182
8044094	0,16	1,12	0,61271	1,53	2,90	0,01140	NM_144632	PFKFB4
8086961	0,16	1,12	0,58922	0,61	1,53	0,25644	NM_004567	IQCH
7984380	0,16	1,12	0,65536	0,12	1,08	0,69830	NM_001031715	ZSCAN29
7988082	0,16	1,11	0,65859	1,23	2,34	0,00269	NM_152455	CPS1
8048026	0,16	1,11	0,80149	-0,93	-1,90	0,03023	NM_001875	PHF15
8108080	0,16	1,11	0,50654	0,71	1,64	0,09914	NM_015288	YARS2
7962203	0,16	1,11	0,66589	0,58	1,49	0,17118	NM_001040436	LMCD1
8077490	0,16	1,11	0,48019	-0,21	-1,16	0,53765	NM_014583	SSTR5
7992145	0,16	1,11	0,67483	-0,17	-1,13	0,59280	NM_001053	POMGNT1
7915801	0,16	1,11	0,73252	1,42	2,68	0,04581	NM_017739	---
8176427	0,16	1,11	0,60914	-0,39	-1,31	0,32259	---	---
8177114	0,16	1,11	0,60914	-0,39	-1,31	0,32259	---	ZNF311
8124622	0,16	1,11	0,59719	0,73	1,66	0,21465	NM_001010877	ZNF311
8178275	0,16	1,11	0,59719	0,73	1,66	0,21465	NM_001010877	ESRRG
7924309	0,15	1,11	0,61644	-1,14	-2,20	0,00450	NM_206594	MYEF2
7988444	0,15	1,11	0,59046	2,44	5,42	0,00464	NM_016132	EGR2
7933872	0,15	1,11	0,75288	0,04	1,03	0,66606	NM_000399	CYGB
8018754	0,15	1,11	0,70708	-1,22	-2,32	0,04929	NM_134268	C22orf25
8071301	0,15	1,11	0,65999	2,09	4,25	0,00359	NM_152906	ASB6
8164572	0,15	1,11	0,64715	1,94	3,84	0,00999	NM_017873	---
8107094	0,15	1,11	0,60281	0,43	1,35	0,45439	---	---
7988342	0,15	1,11	0,74020	0,10	1,07	0,85688	---	CEP97
8081362	0,15	1,11	0,42006	2,15	4,42	0,00109	NM_024548	STK25
8060257	0,15	1,11	0,71187	1,81	3,50	0,00650	NM_006374	PDLIM4
8107897	0,15	1,11	0,64215	-1,12	-2,18	0,07957	NM_003687	SUMO4

8122684	0,15	1,11	0,36237	0,32	1,25	0,36984	NM_001002255	RCC2
7912956	0,15	1,11	0,53341	0,73	1,66	0,23722	NM_001136204	SERGEF
7946933	0,15	1,11	0,69735	0,62	1,53	0,18845	NM_012139	GTPBP4
7925823	0,15	1,11	0,51215	1,89	3,70	0,00003	NM_012341	WDR20
7976957	0,15	1,11	0,73645	0,69	1,62	0,08891	NM_181291	LACE1
8121349	0,15	1,11	0,33375	1,21	2,32	0,00599	NM_145315	PLD5
7925511	0,15	1,11	0,58026	-0,78	-1,71	0,01338	NM_152666	DPY19L2P2
8141882	0,15	1,11	0,75328	0,66	1,58	0,09962	NR_027768	MRP63
7967987	0,15	1,11	0,55766	0,46	1,38	0,08479	NM_024026	KBTBD6
7971208	0,15	1,11	0,60345	1,48	2,79	0,00788	NM_152903	CCDC82
7951144	0,15	1,11	0,71068	0,97	1,96	0,06095	NM_024725	PODNL1
8034712	0,15	1,11	0,73051	-1,69	-3,24	0,01603	NM_024825	MMACHC
7901102	0,15	1,11	0,56955	1,58	3,00	0,00197	NM_015506	STARD10
7950235	0,15	1,11	0,76377	0,39	1,31	0,36207	NM_006645	PDIA6
8040249	0,15	1,11	0,70630	-0,22	-1,17	0,65149	NM_005742	---
8151987	0,15	1,11	0,77590	-1,68	-3,20	0,00375	--- PINX1	
8149296	0,15	1,11	0,41513	0,68	1,60	0,02128	NM_017884	LOC729566
8097780	0,15	1,11	0,41577	-0,44	-1,36	0,08799	ENST00000342688	RNF40
7994939	0,15	1,11	0,52524	1,95	3,88	0,03960	NM_014771	MSL3
8166015	0,15	1,11	0,81346	0,19	1,14	0,58169	NM_006800	TGM5
7988050	0,15	1,11	0,81185	-0,83	-1,77	0,00077	NM_201631	EVC
8093878	0,15	1,11	0,64293	0,48	1,40	0,27125	NM_153717	HIST2H2AB
7919642	0,15	1,11	0,74073	2,21	4,64	0,01670	NM_175065	DEPDC7
7939173	0,15	1,11	0,62026	0,29	1,22	0,30773	NM_001077242	IFNA2
8160422	0,15	1,11	0,32826	-0,63	-1,55	0,00635	NM_000605	NAT10
7939265	0,15	1,11	0,51359	1,62	3,07	0,01036	NM_024662	---
7902611	0,15	1,11	0,75813	-1,19	-2,28	0,00658	--- PHEX	
8166408	0,15	1,11	0,36461	-0,14	-1,10	0,13086	NM_000444	AP4M1
8134712	0,15	1,11	0,57246	0,37	1,29	0,20394	NM_004722	SNORA41
8047780	0,15	1,11	0,29742	-0,51	-1,43	0,10822	NR_002590	CENPJ
7970624	0,15	1,11	0,43216	2,21	4,63	0,03547	NM_018451	TMEM168
8142407	0,14	1,11	0,65653	0,93	1,91	0,03364	NM_022484	SLC25A15
7968734	0,14	1,11	0,80033	1,62	3,07	0,00619	NM_014252	TTC30B
8056995	0,14	1,11	0,92250	0,34	1,26	0,23352	NM_152517	ZC4H2
8173261	0,14	1,10	0,70782	-0,67	-1,59	0,09655	NM_001178032	DAZAP1
8024282	0,14	1,10	0,75342	1,14	2,21	0,00598	NM_170711	TRAPPC2
8171352	0,14	1,10	0,68926	-0,01	-1,01	0,95201	NM_001011658	MMAB
7966213	0,14	1,10	0,61460	1,98	3,94	0,00409	NM_052845	FAM133B
8140942	0,14	1,10	0,69418	0,54	1,45	0,04186	NM_001040057	---
8103725	0,14	1,10	0,67293	-1,18	-2,27	0,04002	--- IQGAP2	
8106354	0,14	1,10	0,64580	1,30	2,46	0,01485	NM_006633	CHRAC1
8148467	0,14	1,10	0,77695	1,64	3,12	0,00010	NM_017444	AACS
7959786	0,14	1,10	0,62179	2,53	5,76	0,00057	NM_023928	NUDT11
8172708	0,14	1,10	0,69676	-0,14	-1,10	0,78938	NM_018159	---
8141533	0,14	1,10	0,43420	0,18	1,13	0,45991	--- METTL8	
8056753	0,14	1,10	0,63439	-0,29	-1,22	0,55398	NM_024770	VSIG10
7966839	0,14	1,10	0,72928	1,30	2,46	0,02290	NM_019086	HAUS4
7977841	0,14	1,10	0,79586	1,77	3,42	0,00023	NM_001166269	TSSC1
8050060	0,14	1,10	0,74622	2,26	4,80	0,00256	NM_003310	TMEM39B
7899675	0,14	1,10	0,65304	1,55	2,92	0,02368	NM_018056	CBX6
8076176	0,14	1,10	0,73788	1,80	3,47	0,02776	NM_014292	ZBTB40
7898750	0,14	1,10	0,73101	2,10	4,30	0,00070	NM_001083621	UBE2Q2
7985044	0,14	1,10	0,84628	1,75	3,35	0,00325	NM_173469	PIGH
7979725	0,14	1,10	0,77946	0,86	1,82	0,02626	NM_004569	XYLB
8078784	0,14	1,10	0,73703	0,54	1,45	0,03928	NM_005108	---
7964983	0,14	1,10	0,76030	-0,41	-1,33	0,29448	--- IKBKB	
8146171	0,14	1,10	0,61952	2,31	4,95	0,00495	NM_001556	---
8140356	0,14	1,10	0,70991	-1,63	-3,10	0,06119	--- PCBP3	
8069252	0,14	1,10	0,64210	-0,77	-1,71	0,08453	NM_020528	SLC16A1
7918622	0,14	1,10	0,70622	1,77	3,41	0,00849	NM_001166496	BAT5
8125017	0,14	1,10	0,05706	1,83	3,56	0,01253	NM_021160	---
7938258	0,14	1,10	0,77978	-0,87	-1,83	0,06794	--- C4orf52	
8094456	0,14	1,10	0,79474	1,76	3,38	0,00056	NM_001145432	TXLNB
8129924	0,14	1,10	0,76128	-0,59	-1,51	0,02465	NM_153235	SOX6
7946757	0,14	1,10	0,73774	-0,46	-1,37	0,30967	NM_017508	FOXK1
8131253	0,14	1,10	0,75722	2,24	4,72	0,00027	NM_001037165	FAM107A
8088415	0,14	1,10	0,76018	-1,41	-2,66	0,03768	NM_007177	SNORD36C
8158998	0,14	1,10	0,82359	0,33	1,26	0,63106	NR_000016	IKZF4
7956105	0,14	1,10	0,78462	0,41	1,33	0,21842	NM_022465	LOC645332
7949898	0,14	1,10	0,85690	1,35	2,55	0,02532	NR_024249	TRIM49 // TRIM49
7939884	0,13	1,10	0,84833	-1,99	-3,97	0,00047	NM_020358 // NM_020358 SYNGR2	
8010243	0,13	1,10	0,77051	3,15	8,86	0,00446	NM_004710	ZNF510
8162631	0,13	1,10	0,80111	1,35	2,55	0,00203	NM_014930	MTMR3

8072302	0,13	1,10	0,69632	1,57	2,97	0,01148	NM_153050	SLC6A9
7915543	0,13	1,10	0,64592	2,34	5,05	0,00189	NM_201649	---
8152092	0,13	1,10	0,75526	-0,36	-1,28	0,45130	---	SAPS2
8074030	0,13	1,10	0,62527	1,42	2,68	0,00886	NM_014678	ZP3
8133728	0,13	1,10	0,79087	2,59	6,02	0,00127	NM_001110354	FKBP9L
8139723	0,13	1,10	0,79070	-0,75	-1,68	0,01062	NR_027342	ARHGEF17
7942417	0,13	1,10	0,66442	1,68	3,19	0,00836	NM_014786	SNORD43
8076223	0,13	1,10	0,80861	-0,17	-1,12	0,09771	NR_002439	NAT1
8144857	0,13	1,10	0,64879	-0,06	-1,04	0,66497	NM_001160170	CCDC138
8044278	0,13	1,10	0,71888	1,35	2,55	0,05482	NM_144978	OTUD3
7898602	0,13	1,10	0,42659	0,89	1,85	0,04595	NM_015207	RGAG4
8173503	0,13	1,10	0,85297	-1,20	-2,30	0,02706	NM_001024455	---
8052667	0,13	1,10	0,56489	-0,14	-1,10	0,63183	---	FAM74A3
8155388	0,13	1,10	0,81400	-1,42	-2,67	0,00076	ENST00000377629	RHBDF1
7998136	0,13	1,10	0,73880	0,48	1,40	0,10196	NM_022450	C4orf29
8097373	0,13	1,10	0,76497	2,54	5,80	0,00077	BC034253	REXO4
8164907	0,13	1,10	0,29540	1,02	2,03	0,01819	NM_020385	FDXACB1
7951654	0,13	1,10	0,80640	0,32	1,25	0,18933	NM_138378	C10orf11
7928534	0,13	1,10	0,70164	1,17	2,25	0,01046	NM_032024	RNPEPL1
8049689	0,13	1,09	0,34484	1,44	2,72	0,03422	NM_018226	ZNF479
8133023	0,13	1,09	0,73917	-0,78	-1,71	0,01313	NM_033273	P2RX5
8011415	0,13	1,09	0,74629	3,08	8,46	0,00114	NM_002561	ST8SIA1
7961757	0,13	1,09	0,79948	-0,96	-1,95	0,19547	NM_003034	MGAT4A
8054135	0,13	1,09	0,68331	2,88	7,37	0,00061	NM_012214	IKZF5
7936826	0,13	1,09	0,72528	2,36	5,12	0,00002	NM_022466	PDCD11
7930226	0,13	1,09	0,70625	2,92	7,56	0,00029	NM_014976	EPHA10
7915056	0,13	1,09	0,33646	-0,17	-1,13	0,36256	NM_001099439	UBXN1
7948782	0,13	1,09	0,31088	0,87	1,83	0,16080	NM_015853	MTERFD2
8060196	0,13	1,09	0,75425	0,35	1,27	0,20375	NR_028049	---
8058458	0,13	1,09	0,93352	0,55	1,46	0,34417	---	PRKY
8176460	0,13	1,09	0,76064	0,17	1,13	0,16027	NR_028062	SMG1
7993999	0,13	1,09	0,55228	1,57	2,98	0,00337	NM_015092	SMG1
8000222	0,13	1,09	0,55228	1,57	2,98	0,00337	NM_015092	HDX
8173848	0,13	1,09	0,34779	-0,54	-1,45	0,32810	NM_001177479	WIF1
7964722	0,13	1,09	0,82529	1,17	2,25	0,00573	NM_007191	GABRE
8175666	0,13	1,09	0,66812	-1,13	-2,18	0,01273	NM_004961	TOM1L2
8013157	0,13	1,09	0,62538	-0,37	-1,29	0,40230	NM_001033551	FDPSL2A // FDPSL2A // FDPSL2A // FDPSL2A // FDPSL2A // FDPSL2A // FDPSL2A // FDPSL2A
8140420	0,13	1,09	0,39597	0,25	1,19	0,30604	NR_003262 // NR_003262 // NR_003262 // NR_003262 // NR_003262 // NR_003262 // NR_003262 // NR_003262	CCDC110
8104014	0,13	1,09	0,69684	-1,14	-2,20	0,01274	NM_152775	WDR83
8026013	0,13	1,09	0,75799	0,06	1,04	0,77128	NR_029375	CACNB4
8055872	0,13	1,09	0,78105	-1,34	-2,53	0,00054	NM_000726	CHN2
8132013	0,13	1,09	0,77901	0,53	1,44	0,52708	NM_004067	STX4
7995017	0,12	1,09	0,76222	1,03	2,04	0,00193	NM_004604	DOM3Z
8125220	0,12	1,09	0,74395	-0,47	-1,38	0,16330	NM_005510	DOM3Z
8178699	0,12	1,09	0,74395	-0,47	-1,38	0,16330	NM_005510	UTP14A
8169868	0,12	1,09	0,61762	-0,02	-1,01	0,95105	NM_006649	TRIM59
8091757	0,12	1,09	0,75772	2,17	4,49	0,00263	NM_173084	PTCH1
8162533	0,12	1,09	0,71002	1,77	3,41	0,02111	NM_001083603	LOC100128830
7937104	0,12	1,09	0,84066	0,33	1,26	0,31308	AK097584	LOC100129118
8026381	0,12	1,09	0,55930	-0,58	-1,50	0,32645	XM_001713875	C10orf58
7928695	0,12	1,09	0,82237	0,99	1,98	0,01930	NM_032333	KIAA1217
7926679	0,12	1,09	0,61621	0,20	1,15	0,43376	NM_019590	LASS4
8025382	0,12	1,09	0,73554	0,70	1,63	0,31125	NM_024552	C3orf14
8080847	0,12	1,09	0,67895	1,30	2,46	0,00719	AF236158	LRRFIP1
8049540	0,12	1,09	0,81226	0,54	1,46	0,03177	NM_001137550	PTPRS
8032926	0,12	1,09	0,72711	3,00	7,99	0,00401	NM_002850	RAB3IL1
7948643	0,12	1,09	0,82104	0,15	1,11	0,65867	NM_013401	FAM98B
7982574	0,12	1,09	0,75713	-0,22	-1,17	0,51767	NM_001042429	THSD1P1
7971800	0,12	1,09	0,54646	0,45	1,37	0,33910	NR_002816	---
8010766	0,12	1,09	0,90961	-1,34	-2,54	0,06510	---	---
8017361	0,12	1,09	0,52176	0,01	1,01	0,96931	---	CLMN
7981142	0,12	1,09	0,78900	3,07	8,42	0,00409	NM_024734	C2CD3
7950332	0,12	1,09	0,81836	0,61	1,52	0,03263	BC035599	FGGY
7901748	0,12	1,09	0,58962	-0,22	-1,16	0,19540	NM_001113411	UNC5C
8101788	0,12	1,09	0,75373	-1,42	-2,67	0,00010	NM_003728	EFHD2
7898161	0,12	1,09	0,56897	2,31	4,95	0,02171	NM_024329	SCLT1
8102751	0,12	1,09	0,76951	1,06	2,09	0,00699	NM_144643	SULT1C4
8044225	0,12	1,09	0,84458	-1,34	-2,54	0,01261	NM_006588	COQ10A
7956242	0,12	1,09	0,80480	3,63	12,42	0,00054	NM_144576	LETM2
8145942	0,12	1,09	0,64274	0,95	1,93	0,00229	NM_144652	RMND1
8130191	0,12	1,09	0,70810	0,50	1,41	0,03157	NM_017909	WDFY2

7969204	0,12	1,09	0,83327	1,41	2,67	0,00051	NM_052950	NME7
7922137	0,12	1,09	0,63523	1,27	2,42	0,00386	NM_013330	RHOG
7945944	0,12	1,09	0,76940	0,28	1,21	0,11884	NM_001665	MAD2L2
7912481	0,12	1,09	0,68915	1,45	2,72	0,00366	NM_001127325	---
8139158	0,12	1,09	0,70518	-1,60	-3,04	0,10305	--- EPSTI1	
7971296	0,12	1,09	0,60678	-0,55	-1,46	0,01394	NM_001002264	SNORA44
7914214	0,12	1,09	0,62347	0,02	1,02	0,96850	NR_002976	C4orf7
8095467	0,12	1,09	0,83893	-1,06	-2,09	0,00056	NM_152997	DUS1L
8019376	0,12	1,09	0,71724	1,26	2,39	0,00622	NM_022156	NUDT12
8113413	0,12	1,09	0,68595	0,73	1,66	0,08305	NM_031438	TRIM52
8110666	0,12	1,09	0,78682	1,45	2,73	0,00251	NM_032765	C9orf91
8157463	0,12	1,09	0,76388	2,89	7,39	0,00089	NM_153045	C15orf51
7986509	0,12	1,08	0,95607	-2,03	-4,07	0,00891	ENST00000423248	LOC100289668
7986512	0,12	1,08	0,95607	-2,03	-4,07	0,00891	ENST00000426730	LOC100289668
7986527	0,12	1,08	0,95607	-2,03	-4,07	0,00891	ENST00000426730	ZNF319
8001651	0,12	1,08	0,80924	1,67	3,19	0,02473	NM_020807	ACBD7
7932214	0,12	1,08	0,58244	-0,14	-1,10	0,72476	NM_001039844	ZFP28
8031659	0,12	1,08	0,86271	0,68	1,61	0,06773	NM_020828	TNFRSF10C
8145244	0,12	1,08	0,55375	-0,17	-1,12	0,27002	NM_003841	ZNF503
7934553	0,12	1,08	0,77042	0,55	1,46	0,28341	NM_032772	DBF4
8133976	0,12	1,08	0,66209	0,42	1,34	0,00176	NM_006716	LRRC37A2
8016259	0,12	1,08	0,71448	1,29	2,45	0,06792	NM_001006607	AFG3L2
8022342	0,12	1,08	0,68620	1,82	3,52	0,00006	NM_006796	NAB2
7956287	0,12	1,08	0,49313	0,19	1,14	0,64522	NM_005967	BAT5
8178561	0,12	1,08	0,23821	1,78	3,43	0,01463	NM_021160	IER2
8026163	0,12	1,08	0,80482	0,54	1,46	0,07301	NM_004907	TRA2A
8138592	0,12	1,08	0,73163	-0,81	-1,76	0,14855	AB052759 SLC25A23	
8033190	0,11	1,08	0,55765	2,31	4,95	0,01133	NM_024103	NCKAP5L
7963134	0,11	1,08	0,65658	-0,10	-1,07	0,78851	NM_001037806	CRTC1
8027053	0,11	1,08	0,63159	0,08	1,06	0,78970	NM_001098482	MPRIP
8005235	0,11	1,08	0,77808	-0,68	-1,60	0,48918	NM_201274	SNORA59A
7897953	0,11	1,08	0,74929	-1,47	-2,77	0,00370	NR_003025	SNORA59A
8005626	0,11	1,08	0,74929	-1,47	-2,77	0,00370	NR_003025	IFIT5
7929072	0,11	1,08	0,60126	0,10	1,07	0,76031	NM_012420	PBRM1
7967896	0,11	1,08	0,53992	-0,55	-1,46	0,00681	ENST00000421695	CSTF2
8168762	0,11	1,08	0,29755	0,87	1,82	0,00902	NM_001325	ZNF239
7933180	0,11	1,08	0,77163	0,27	1,21	0,37793	NM_001099282	GTDC1
8055606	0,11	1,08	0,77156	1,24	2,36	0,00024	NM_001006636	HIST1H4C
8117368	0,11	1,08	0,78228	3,71	13,08	0,00004	NM_003542	CD248
7949588	0,11	1,08	0,76367	-1,20	-2,30	0,06490	NM_020404	---
7968272	0,11	1,08	0,85117	-0,13	-1,09	0,72662	--- TIPIN	
7989915	0,11	1,08	0,84722	1,09	2,13	0,13368	NM_017858	ESCO1
8022473	0,11	1,08	0,70881	1,45	2,72	0,02646	NM_052911	KIF14
7923189	0,11	1,08	0,77411	3,16	8,94	0,00016	NM_014875	ISCA2
7975705	0,11	1,08	0,73190	1,55	2,93	0,01426	NM_194279	FAM72D
8039928	0,11	1,08	0,76831	2,68	6,43	0,00618	AB096683 GALK1	
8018428	0,11	1,08	0,87428	0,92	1,89	0,11418	NM_000154	MCOLN2
7917283	0,11	1,08	0,70252	0,71	1,64	0,02193	NM_153259	NASP
7901123	0,11	1,08	0,53727	1,55	2,93	0,00053	NM_172164	FDPS
7905986	0,11	1,08	0,59743	2,85	7,19	0,00055	NM_002004	SNORD6
7951030	0,11	1,08	0,89284	1,01	2,01	0,11540	NR_003036	ZNF10
7960158	0,11	1,08	0,72549	0,49	1,40	0,09205	NM_015394	PPIL1
8126058	0,11	1,08	0,75264	1,16	2,23	0,09581	NM_016059	TPBG
8120880	0,11	1,08	0,83309	1,65	3,14	0,07648	NM_006670	CWF19L2
7951447	0,11	1,08	0,59575	-0,29	-1,22	0,00323	NM_152434	C6orf150
8127534	0,11	1,08	0,70946	2,92	7,59	0,00352	NM_138441	ZNF568
8028213	0,11	1,08	0,63146	-0,12	-1,08	0,62283	NM_198539	C2orf64
8054131	0,11	1,08	0,60565	1,13	2,19	0,00961	BC047722 CD34	
7923978	0,11	1,08	0,74960	-0,97	-1,95	0,03126	NM_001773	MIR199A1
8034099	0,11	1,08	0,62106	-0,76	-1,69	0,01342	NR_029586	UBASH3B
7944722	0,11	1,08	0,81130	1,91	3,75	0,00129	NM_032873	WNT2B
7904025	0,11	1,08	0,68320	-0,52	-1,44	0,16459	NM_024494	MIR138-1
8079165	0,11	1,08	0,78940	-1,24	-2,37	0,00248	NR_029700	C6orf115
8122336	0,11	1,08	0,86579	1,68	3,20	0,01274	NM_021243	DAG1
8079753	0,11	1,08	0,79687	1,92	3,78	0,00698	NM_001165928	PRMT6
7903457	0,11	1,08	0,79434	-0,52	-1,43	0,07833	NM_018137	CREB3L4
7905677	0,11	1,08	0,66717	0,68	1,60	0,01895	NM_130898	---
8007990	0,11	1,08	0,90346	-3,10	-8,57	0,01747	--- RPL23AP53	
8148966	0,11	1,08	0,85084	-0,42	-1,34	0,29852	NR_003572	ANKRD5
8060949	0,11	1,08	0,73018	2,27	4,82	0,00616	NM_022096	PDCL
8163964	0,11	1,08	0,77446	1,74	3,34	0,00036	NM_005388	NHS
8166266	0,11	1,08	0,57573	-0,23	-1,18	0,56503	NM_198270	ZFP2
8110472	0,11	1,08	0,74412	-0,16	-1,11	0,69115	NM_030613	ZNF208

8035819	0,11	1,08	0,68193	-0,57	-1,48	0,27982	NM_007153	DEFB107A
8144405	0,11	1,08	0,77661	-0,62	-1,53	0,02140	NM_001037668	DEFB107A
8149230	0,11	1,08	0,77661	-0,62	-1,53	0,02140	NM_001037668	RING1
8178244	0,11	1,08	0,88390	1,10	2,15	0,02171	NM_002931	NFRKB
7952677	0,11	1,08	0,75176	1,78	3,44	0,00063	NM_006165	TMEM176A
8137264	0,11	1,08	0,68403	-1,66	-3,16	0,00223	NM_018487	---
8174193	0,11	1,08	0,91294	-2,30	-4,93	0,00028	---	E2F7
7965094	0,10	1,08	0,67382	0,55	1,46	0,01764	NM_203394	SNORA40
7951038	0,10	1,08	0,65364	1,11	2,16	0,03894	NR_002973	UPF3B
8174717	0,10	1,07	0,69579	1,35	2,54	0,01067	NM_080632	ZNF256
8039706	0,10	1,07	0,84629	0,18	1,13	0,67502	NM_005773	RABGGTA
7978239	0,10	1,07	0,84284	1,05	2,08	0,05135	NM_004581	SIRT4
7959148	0,10	1,07	0,75220	1,10	2,14	0,05915	NM_012240	---
8160317	0,10	1,07	0,81674	-0,51	-1,42	0,04458	---	ROBLD3
7906072	0,10	1,07	0,78246	1,37	2,58	0,08272	NM_014017	BET1
8141024	0,10	1,07	0,50800	0,72	1,65	0,17498	NM_005868	---
8094717	0,10	1,07	0,33288	-0,43	-1,35	0,04230	---	FGD1
8173086	0,10	1,07	0,54126	0,28	1,21	0,31852	NM_004463	---
8106250	0,10	1,07	0,74007	-1,35	-2,55	0,02849	---	HHAT
7909510	0,10	1,07	0,69697	0,91	1,88	0,00915	NM_001170580	PRR4
7961252	0,10	1,07	0,76534	0,61	1,52	0,03092	NM_007244	DIRC1
8057615	0,10	1,07	0,91749	-2,21	-4,63	0,01522	NM_052952	GPD1L
8078386	0,10	1,07	0,79329	1,50	2,82	0,00039	NM_015141	ZNF211
8031792	0,10	1,07	0,63847	0,06	1,05	0,80318	NM_006385	HAUS8
8035236	0,10	1,07	0,65595	0,47	1,38	0,04942	NM_033417	HLA-DMB
8125530	0,10	1,07	0,84708	3,67	12,70	0,00074	NM_002118	ZNF442
8034379	0,10	1,07	0,82739	-0,11	-1,08	0,69331	NM_030824	C1orf97
7909561	0,10	1,07	0,77670	1,08	2,11	0,04756	NR_026761	BRF2
8150219	0,10	1,07	0,77009	0,30	1,23	0,15419	NM_018310	GREB1L
8020384	0,10	1,07	0,45165	1,10	2,14	0,00681	NM_001142966	ZBTB49
8093829	0,10	1,07	0,77290	0,53	1,45	0,08405	NM_145291	PNLIPRP1
7930790	0,10	1,07	0,79961	-1,09	-2,13	0,04802	NM_006229	HRASLS2
7948982	0,10	1,07	0,58323	-0,45	-1,37	0,00524	NM_017878	---
7917080	0,10	1,07	0,69832	1,11	2,15	0,00854	---	AKAP8
8035007	0,10	1,07	0,44190	1,31	2,48	0,00952	NM_005858	ANK3
7933772	0,10	1,07	0,64496	0,19	1,14	0,32956	NM_020987	SPESP1
7984488	0,10	1,07	0,80950	-1,13	-2,19	0,00050	NM_145658	ZHX3
8066279	0,10	1,07	0,79399	0,68	1,60	0,00355	NM_015035	ZNF16
8153930	0,10	1,07	0,83080	1,43	2,70	0,00339	NM_001029976	PDPK1
7992670	0,10	1,07	0,88115	0,99	1,98	0,05988	NM_002613	NAA38
8135688	0,10	1,07	0,69958	-0,01	-1,01	0,95818	NM_016200	LOC644714
8117079	0,10	1,07	0,80587	-1,48	-2,79	0,01975	BC047037	QPCTL
8029744	0,10	1,07	0,83129	0,70	1,63	0,09055	NM_017659	KCTD1
8022646	0,10	1,07	0,82933	0,73	1,66	0,10326	NM_001136205	UHRF1BP1
8118833	0,10	1,07	0,77738	2,63	6,20	0,00005	NM_017754	VPS26B
7945275	0,10	1,07	0,69931	2,02	4,07	0,00174	NM_052875	PDPK1
7998820	0,10	1,07	0,90662	1,70	3,25	0,03130	NM_002613	ARL6IP6
8045768	0,10	1,07	0,74969	1,92	3,79	0,02025	NM_152522	SPATA5L1
7983469	0,10	1,07	0,79492	1,92	3,78	0,00172	NM_024063	FTHL5
8126948	0,10	1,07	0,46787	-0,25	-1,19	0,02578	J04755	DCXR
8019357	0,10	1,07	0,72610	4,22	18,63	0,00012	NM_016286	---
8048976	0,10	1,07	0,57440	-0,87	-1,83	0,07932	---	---
8092224	0,10	1,07	0,78189	0,01	1,00	0,98866	---	CPVL
8138805	0,10	1,07	0,70047	0,57	1,49	0,14765	NM_019029	ANKAR
8047006	0,10	1,07	0,54122	0,27	1,21	0,18258	NM_144708	FARP2
8049847	0,10	1,07	0,76354	0,68	1,61	0,12181	NM_014808	PDPK1
7992682	0,10	1,07	0,91132	1,36	2,56	0,06276	NM_002613	RAGE
7981346	0,10	1,07	0,63496	0,75	1,68	0,00376	NM_014226	C4orf21
8102371	0,10	1,07	0,50390	0,44	1,36	0,14325	NM_018392	CDC42
7898739	0,10	1,07	0,82979	-0,09	-1,07	0,77569	NM_044472	---
8146241	0,10	1,07	0,70439	-0,49	-1,40	0,10641	---	KIF27
8156160	0,10	1,07	0,95404	0,26	1,20	0,80845	NM_017576	---
8136179	0,10	1,07	0,87928	0,14	1,10	0,71470	---	HOXA2
8138718	0,10	1,07	0,90832	1,62	3,08	0,00769	NM_006735	ZNF167
8079189	0,10	1,07	0,86745	-0,17	-1,13	0,74266	NM_018651	SCARNA13
7981181	0,10	1,07	0,87002	0,49	1,41	0,33111	NR_003002	MYEOV2
8060086	0,10	1,07	0,80404	0,34	1,27	0,05048	NM_138336	TMEM223
7948836	0,10	1,07	0,74165	3,02	8,10	0,00355	NM_001080501	LOC646982
7971167	0,10	1,07	0,76644	-1,64	-3,11	0,00049	NR_024507	CLSPN
7914851	0,09	1,07	0,66211	0,37	1,29	0,16226	NM_022111	TIMM8A
8174047	0,09	1,07	0,77976	0,65	1,56	0,40174	NM_004085	LAS1L
8173269	0,09	1,07	0,76203	1,29	2,44	0,00591	NM_031206	FGB
8097910	0,09	1,07	0,62283	0,33	1,26	0,17767	NM_005141	DCAF15

8026250	0,09	1,07	0,75301	1,69	3,22	0,00321	NM_138353	MMP13
7951309	0,09	1,07	0,70944	0,04	1,03	0,84673	NM_002427	NRF1
8136140	0,09	1,07	0,77108	0,68	1,60	0,04088	NM_005011	TEX9
7983828	0,09	1,07	0,37301	0,49	1,40	0,21205	NM_198524	TIMP4
8085360	0,09	1,07	0,80917	-1,00	-2,00	0,01101	NM_003256	TMEM55B
7977584	0,09	1,07	0,88391	1,57	2,98	0,03045	NM_001100814	ZNF732
8098745	0,09	1,07	0,74440	-0,23	-1,17	0,41597	NM_001137608	CLEC3A
7997346	0,09	1,07	0,66496	-0,30	-1,23	0,10147	NM_005752	SART3
7966098	0,09	1,07	0,83424	2,16	4,47	0,00044	NM_014706	TOMM22
8073032	0,09	1,07	0,86520	1,66	3,15	0,00080	NM_020243	EED
7942914	0,09	1,07	0,80684	1,66	3,17	0,04651	NM_152991	TAF7
8114653	0,09	1,07	0,68199	1,30	2,47	0,00251	NM_005642	CHMP7
8145259	0,09	1,07	0,58392	1,97	3,91	0,00042	NM_152272	TMEFF2
8057803	0,09	1,07	0,89495	-0,90	-1,87	0,00059	NM_016192	MTAP
8154635	0,09	1,06	0,49487	1,17	2,25	0,01546	NM_002451	C17orf97
8003607	0,09	1,06	0,67571	0,14	1,10	0,56700	NM_001013672	UBE2Q2P1
7991120	0,09	1,06	0,76917	-0,35	-1,28	0,31225	NR_003661	RPL37
8111814	0,09	1,06	0,87278	-0,64	-1,56	0,06491	NM_000997	PHF13
7897322	0,09	1,06	0,84998	0,06	1,04	0,85544	NM_153812	C12orf39
7954398	0,09	1,06	0,60658	0,63	1,55	0,21676	NM_030572	NFIC
8024623	0,09	1,06	0,82006	2,08	4,23	0,00008	NM_205843	OAS3
7958895	0,09	1,06	0,73635	0,73	1,66	0,12402	NM_006187	MAN2A2
7986132	0,09	1,06	0,87039	2,41	5,31	0,00280	NM_006122	SHISA4
7908758	0,09	1,06	0,31455	0,12	1,09	0,82578	NM_198149	PLA2G12A
8102321	0,09	1,06	0,85109	1,71	3,26	0,01135	NM_030821	OR4K15
8020354	0,09	1,06	0,92674	-2,15	-4,43	0,01480	NM_001005486	KTI12
7916130	0,09	1,06	0,85085	3,07	8,38	0,00402	NM_138417	SIN3B
8026610	0,09	1,06	0,66872	1,15	2,22	0,03932	NM_015260	NIPSNAP3B
8157027	0,09	1,06	0,75532	0,32	1,25	0,26142	NM_018376	SNORA4
8084708	0,09	1,06	0,58792	-0,24	-1,18	0,50235	NR_002588	C12orf26
7957404	0,09	1,06	0,87588	0,98	1,97	0,11235	NM_032230	---
7901852	0,09	1,06	0,84925	-1,11	-2,16	0,01829	--- CHAF1B	
8068478	0,09	1,06	0,78435	2,11	4,32	0,00017	NM_005441	RAB3IP
7957072	0,09	1,06	0,74936	2,90	7,45	0,00005	NM_175623	SPAG5
8013671	0,09	1,06	0,65092	3,09	8,54	0,00015	NM_006461	RAB32
8122554	0,09	1,06	0,76759	-0,01	-1,01	0,97832	NM_006834	C3orf75
8086784	0,09	1,06	0,79908	0,90	1,86	0,03744	NM_001031703	MYCT1
8122860	0,09	1,06	0,75836	-0,99	-1,99	0,03054	NM_025107	PFKL
8069057	0,09	1,06	0,79769	1,68	3,21	0,03568	NR_024108	CHMP4A
7978174	0,09	1,06	0,83836	0,68	1,60	0,00879	NM_014169	MRPS17
8132922	0,09	1,06	0,74440	0,34	1,27	0,54332	NM_015969	TNRC18
8137953	0,09	1,06	0,69932	0,08	1,06	0,84030	NM_001080495	DBNDD2
8062971	0,09	1,06	0,88313	1,98	3,95	0,00021	NM_001048223	NAALADL1
7949344	0,09	1,06	0,76606	-0,63	-1,55	0,11255	NM_005468	MGST2
8097513	0,09	1,06	0,65939	1,79	3,46	0,00202	NM_002413	SSRP1
7948211	0,09	1,06	0,79258	-0,92	-1,89	0,00112	AB209132 ZNF835	
8039605	0,09	1,06	0,88613	-1,71	-3,27	0,01001	NM_001005850	LCN8
8165295	0,09	1,06	0,86453	-2,14	-4,42	0,00015	ENST00000371686	RPL26L1
8110018	0,09	1,06	0,87749	-0,45	-1,37	0,27876	NM_016093	CCDC30
7900555	0,09	1,06	0,70005	-0,42	-1,34	0,07513	NM_001080850	GDEP
8096027	0,09	1,06	0,22147	-0,49	-1,41	0,10645	NR_026555	NISCH
8080320	0,09	1,06	0,66999	1,55	2,93	0,02540	NM_007184	---
7945989	0,09	1,06	0,88015	-1,06	-2,09	0,01138	--- SNORA75	
8059708	0,09	1,06	0,81469	-0,81	-1,75	0,00440	NR_002921	RPS6KB2
7941900	0,08	1,06	0,72124	1,65	3,13	0,00121	NM_003952	TANC2
8009075	0,08	1,06	0,79681	2,80	6,98	0,00794	NM_025185	OR2G6
7911287	0,08	1,06	0,88166	-0,89	-1,85	0,01780	NM_001013355	FAM113A
8064557	0,08	1,06	0,57539	0,95	1,93	0,10617	AK293638 ---	
7919854	0,08	1,06	0,93002	-2,22	-4,66	0,01224	--- VAMP1	
7960544	0,08	1,06	0,77627	2,01	4,03	0,00854	NM_199245	C14orf147
7978553	0,08	1,06	0,84758	4,28	19,37	0,00004	NM_138288	ABCB1
8140782	0,08	1,06	0,85595	-0,69	-1,61	0,00675	NM_000927	KRTAP4-3
8015240	0,08	1,06	0,88393	-0,29	-1,22	0,33242	NM_033187	SYTL3
8123080	0,08	1,06	0,84480	-0,23	-1,17	0,49450	NM_001009991	GAS2L3
7957850	0,08	1,06	0,85991	0,47	1,39	0,45841	NM_174942	POLR3D
8145085	0,08	1,06	0,76758	1,13	2,19	0,05845	NM_001722	KRT14
8015366	0,08	1,06	0,88303	-1,17	-2,25	0,02673	NM_000526	HVCN1
7966356	0,08	1,06	0,66069	-0,48	-1,40	0,08281	NM_001040107	PLEKHA6
7923635	0,08	1,06	0,91639	-0,28	-1,21	0,38668	NM_014935	WDR54
8042843	0,08	1,06	0,66585	0,09	1,06	0,83235	NM_032118	ENO2
7953532	0,08	1,06	0,81631	2,04	4,11	0,00264	NM_001975	NOC2L
7911359	0,08	1,06	0,89022	1,99	3,99	0,03245	NM_015658	---
8175490	0,08	1,06	0,78921	-0,88	-1,84	0,02322	--- ZC3H4	

8037856	0,08	1,06	0,67208	0,43	1,34	0,41186	NM_015168	ADCY4
7978285	0,08	1,06	0,80576	-0,36	-1,28	0,48552	NM_139247	PPT2
8179451	0,08	1,06	0,30134	1,06	2,09	0,08992	NM_005155	LOC729020
7930205	0,08	1,06	0,91959	-0,36	-1,28	0,40058	NM_001143909	KIAA1704
7968898	0,08	1,06	0,54794	0,63	1,55	0,32756	BC039586 ---	
8089849	0,08	1,06	0,78348	-0,60	-1,52	0,08247	--- ---	
7984008	0,08	1,06	0,83756	0,46	1,38	0,27592	--- GDF5	
8065905	0,08	1,06	0,75076	-0,50	-1,42	0,06727	NM_000557	ANXA3
8095986	0,08	1,06	0,84700	4,52	22,93	0,00001	NM_005139	RCAN3
7898957	0,08	1,06	0,82269	1,76	3,38	0,00170	NM_013441	ZNF479
8139800	0,08	1,06	0,84242	-0,79	-1,73	0,00628	NM_033273	ZNF471
8031650	0,08	1,06	0,87881	-0,33	-1,26	0,49515	NM_020813	CLTCL1
8074399	0,08	1,06	0,78170	0,43	1,34	0,02034	NM_007098	PKN1
8026315	0,08	1,06	0,86091	1,30	2,47	0,05685	NM_213560	LOC100287852
8081335	0,08	1,05	0,90567	0,40	1,32	0,04162	ENST00000426236	NUPL2
8131831	0,08	1,05	0,87340	1,99	3,96	0,01570	NM_007342	---
8040415	0,08	1,05	0,82646	-0,45	-1,36	0,09677	--- SDSL	
7959002	0,08	1,05	0,79980	0,56	1,48	0,36718	NM_138432	KRTAP5-7
7942255	0,08	1,05	0,94453	-2,41	-5,33	0,02509	NM_001012503	PIH1D1
8038382	0,08	1,05	0,79890	1,47	2,77	0,00014	NM_017916	OR2W1
8124630	0,08	1,05	0,77237	-0,51	-1,42	0,09618	NM_030903	OR2W1
8178283	0,08	1,05	0,77237	-0,51	-1,42	0,09618	NM_030903	OR2W1
8179587	0,08	1,05	0,77237	-0,51	-1,42	0,09618	NM_030903	BCL2L11
8044375	0,08	1,05	0,18709	2,10	4,29	0,00023	NM_138621	PIK3C2B
7923662	0,08	1,05	0,77146	1,81	3,50	0,01065	NM_002646	NACC2
8165077	0,08	1,05	0,88885	0,53	1,44	0,51625	NM_144653	REEP2
8108359	0,08	1,05	0,72257	-0,13	-1,10	0,27480	NM_016606	KHSRP
8033162	0,08	1,05	0,84053	1,55	2,92	0,03437	NM_003685	---
8126033	0,08	1,05	0,74021	-1,09	-2,12	0,18516	--- ZNF710	
7985924	0,07	1,05	0,87013	1,08	2,12	0,00667	NM_198526	---
7967967	0,07	1,05	0,88940	-0,68	-1,60	0,23217	--- GUK1	
7910241	0,07	1,05	0,82826	0,33	1,26	0,27379	NM_000858	FGF14
7972650	0,07	1,05	0,83827	-0,90	-1,87	0,00851	NM_175929	C1orf220
7907649	0,07	1,05	0,76784	-0,04	-1,03	0,72838	NR_033186	C1orf31
7910630	0,07	1,05	0,85565	1,60	3,03	0,00515	BC116455 LPAR4	
8168517	0,07	1,05	0,59218	-1,31	-2,49	0,00190	NM_005296	PPT2
8118509	0,07	1,05	0,28114	1,02	2,03	0,09752	NM_005155	TRAPPC6A
8037505	0,07	1,05	0,78650	0,98	1,97	0,00698	NM_024108	ZNF790
8036357	0,07	1,05	0,73560	0,22	1,17	0,13811	NM_206894	MSC
8151334	0,07	1,05	0,86399	0,00	1,00	0,98668	NM_005098	FAM72D
7909146	0,07	1,05	0,81800	2,70	6,50	0,00751	AB096683 BTF3	
8106181	0,07	1,05	0,84515	0,72	1,65	0,01587	NM_001037637	CKB
7981427	0,07	1,05	0,83695	2,48	5,59	0,00152	NM_001823	WNK3
8173059	0,07	1,05	0,55551	-0,20	-1,15	0,60617	NM_020922	KIF4A
8168146	0,07	1,05	0,88492	2,18	4,53	0,03109	NM_012310	PLEKHB1
7942453	0,07	1,05	0,89434	1,12	2,18	0,15397	NM_021200	FAM119B
7956631	0,07	1,05	0,72870	1,37	2,59	0,00264	NM_206914	NOP56
8060484	0,07	1,05	0,57200	0,29	1,22	0,31583	NR_027700	SORBS1
7935188	0,07	1,05	0,73603	0,40	1,32	0,08406	NM_001034954	MGC39372
8123606	0,07	1,05	0,88004	1,03	2,05	0,01144	BC025340 UBXN4	
8055404	0,07	1,05	0,86198	-1,94	-3,83	0,00379	NM_014607	SLC4A11
8064613	0,07	1,05	0,56550	2,36	5,13	0,00087	NM_032034	RNASET2
8130768	0,07	1,05	0,69053	0,78	1,72	0,00257	NM_003730	ZNF200
7998931	0,07	1,05	0,83147	0,42	1,34	0,04518	NM_198088	HIRA
8074432	0,07	1,05	0,90179	2,27	4,83	0,00016	NM_003325	CCNL2
7911539	0,07	1,05	0,78271	1,38	2,60	0,00004	NM_030937	HSPC157
7898736	0,07	1,05	0,90255	0,76	1,70	0,08738	NR_023918	ZNF436
7913582	0,07	1,05	0,84163	0,75	1,68	0,04350	NM_001077195	HSD17B2
7997491	0,07	1,05	0,66613	-0,72	-1,65	0,01564	NM_002153	OR52W1
7938076	0,07	1,05	0,93989	-1,26	-2,39	0,05462	NM_001005178	MAGEF1
8092514	0,07	1,05	0,80298	0,73	1,66	0,05244	NM_022149	ACCN2
7955317	0,07	1,05	0,80658	2,99	7,95	0,00315	NM_020039	CD36
8133876	0,07	1,05	0,90129	-0,44	-1,36	0,00165	NM_001001548	FMN2
7910923	0,06	1,05	0,77284	-0,23	-1,17	0,41513	NM_020066	ATP5SL
8037037	0,06	1,05	0,87314	3,28	9,73	0,00235	NM_001167867	HFE
8117343	0,06	1,05	0,64098	1,00	2,00	0,00040	NM_000410	EIF1AD
7949545	0,06	1,05	0,90403	2,10	4,29	0,01181	NM_032325	LPPR2
8025877	0,06	1,05	0,85576	0,43	1,35	0,57532	NM_022737	---
8156895	0,06	1,05	0,82890	0,09	1,07	0,65869	--- ---	
7939005	0,06	1,05	0,88893	-1,26	-2,40	0,00857	--- C7orf69	
8132710	0,06	1,05	0,83248	-1,02	-2,03	0,00190	NM_025031	TMEM173
8114536	0,06	1,04	0,88798	-0,68	-1,60	0,29412	NM_198282	LOC441666
8069494	0,06	1,04	0,77547	-0,26	-1,20	0,45696	NR_024380	PI15

8146957	0,06	1,04	0,78834	0,13	1,09	0,75039	NM_015886	SPP1
8096301	0,06	1,04	0,85923	-1,25	-2,37	0,03815	NM_001040058	---
8017253	0,06	1,04	0,77910	-0,19	-1,14	0,10203	--- CSNK1D	
8010778	0,06	1,04	0,91078	0,57	1,48	0,08096	NM_001893	TMUB1
8143879	0,06	1,04	0,82451	0,60	1,51	0,32215	NM_001136044	S100P
8093950	0,06	1,04	0,74488	0,82	1,77	0,04270	NM_005980	LOC644714
8086538	0,06	1,04	0,94332	-1,94	-3,85	0,04071	BC047037 HMGN1	
8070389	0,06	1,04	0,90862	0,90	1,86	0,22434	NM_004965	N6AMT2
7970467	0,06	1,04	0,80782	0,10	1,07	0,82559	NM_174928	LENG8
8031176	0,06	1,04	0,71229	1,01	2,01	0,03847	NM_052925	GOLGA6L1
7981787	0,06	1,04	0,92227	-1,50	-2,84	0,03821	NM_001001413	---
8176574	0,06	1,04	0,87275	-0,51	-1,42	0,07762	--- PAPPA2	
7907572	0,06	1,04	0,84258	-1,10	-2,14	0,00196	NM_020318	RPAP1
7987675	0,06	1,04	0,92563	1,88	3,68	0,01850	NM_015540	SCN1B
8027719	0,06	1,04	0,78361	-0,02	-1,01	0,94372	NM_001037	MDFIC
8135568	0,06	1,04	0,78402	0,06	1,04	0,91503	NM_199072	---
8156112	0,06	1,04	0,87174	-0,55	-1,47	0,02417	--- RGS16	
7922717	0,06	1,04	0,88549	0,62	1,53	0,06971	NM_002928	TMEM212
8083897	0,06	1,04	0,76546	-1,20	-2,29	0,01114	NM_001164436	NOX4
7950933	0,06	1,04	0,87995	-1,48	-2,79	0,03403	NM_016931	TERF2IP
7997281	0,06	1,04	0,86315	-0,03	-1,02	0,94426	NM_018975	PLCD1
8086185	0,06	1,04	0,82323	0,39	1,31	0,30958	NM_006225	TMEM38A
8026579	0,06	1,04	0,88548	0,55	1,46	0,13294	NM_024074	NXT2
8169352	0,06	1,04	0,87843	0,65	1,57	0,01532	NM_018698	PWP2
8069003	0,06	1,04	0,87266	1,46	2,76	0,05138	NM_005049	LOC728498
7982284	0,06	1,04	0,96665	0,26	1,20	0,63899	ENST00000450802	CXCR7
8049471	0,06	1,04	0,86284	-0,13	-1,09	0,51413	NM_020311	---
8107468	0,06	1,04	0,75003	-0,19	-1,14	0,44498	--- CHFR	
7967841	0,06	1,04	0,88661	0,81	1,76	0,08826	NM_001161344	POFUT1
8061706	0,06	1,04	0,72540	1,43	2,70	0,00021	NM_015352	BCKDHB
8120860	0,06	1,04	0,94064	3,58	11,98	0,00002	NM_183050	RPUSD3
8085233	0,06	1,04	0,91999	3,85	14,40	0,00133	NM_173659	WHSC2
8098942	0,06	1,04	0,56229	0,81	1,76	0,05326	NM_005663	REPIN1
8137225	0,06	1,04	0,90579	1,20	2,30	0,05783	NM_013400	ZNF26
7960134	0,06	1,04	0,83266	1,18	2,27	0,00006	NM_019591	ITPRIPL2
7993622	0,06	1,04	0,89882	0,56	1,47	0,42296	NM_001034841	TP53RK
8066776	0,06	1,04	0,79557	-0,16	-1,12	0,64048	NM_033550	---
7913642	0,06	1,04	0,83952	-0,56	-1,47	0,05044	--- IL6R	
7905789	0,06	1,04	0,85036	1,89	3,71	0,01966	NM_000565	LOC654342
8053722	0,06	1,04	0,95080	2,13	4,38	0,00419	NR_027238	---
8122701	0,06	1,04	0,09670	0,11	1,08	0,61833	--- LOC143188	
7930533	0,06	1,04	0,89511	0,00	-1,00	0,99335	NR_015409	MIR503
8175261	0,05	1,04	0,85971	0,03	1,02	0,85672	NR_030228	RTN4IP1
8128606	0,05	1,04	0,84687	1,78	3,43	0,00107	NM_032730	TXNDC16
7979158	0,05	1,04	0,90567	0,77	1,71	0,06382	NM_020784	MRPS34
7998585	0,05	1,04	0,90417	1,17	2,25	0,04369	NM_023936	CXorf40B
8175642	0,05	1,04	0,89470	1,45	2,72	0,07599	NM_001013845	NCRNA00183
8168412	0,05	1,04	0,94149	0,74	1,67	0,07233	BC029480 PLEKHG2	
8028656	0,05	1,04	0,79507	0,81	1,76	0,04196	NM_022835	ZNF202
7952361	0,05	1,04	0,83159	1,65	3,15	0,00127	NM_003455	LYRM7
8107859	0,05	1,04	0,88581	0,35	1,28	0,20537	NM_181705	RNF220
7900979	0,05	1,04	0,92506	2,54	5,83	0,00030	NM_018150	YIPF4
8041273	0,05	1,04	0,78436	1,17	2,25	0,01099	NM_032312	UBAC2
7969776	0,05	1,04	0,89872	2,01	4,04	0,00516	NR_026644	TP53INP2
8062034	0,05	1,04	0,78278	-0,11	-1,08	0,90550	NM_021202	---
8095360	0,05	1,04	0,89304	1,27	2,41	0,04222	--- FLJ30430	
7925031	0,05	1,04	0,95241	-1,04	-2,06	0,00224	AK054992 PER2	
8059996	0,05	1,04	0,68729	-0,21	-1,15	0,59869	NM_022817	KIAA1712
8098348	0,05	1,04	0,85869	0,41	1,33	0,27060	NM_001040157	STARD9
7983145	0,05	1,04	0,80725	0,63	1,55	0,02918	NM_020759	---
8173609	0,05	1,04	0,81292	0,50	1,41	0,34600	--- METTL13	
7907353	0,05	1,04	0,92353	1,90	3,73	0,01487	NM_015935	ZRSR2
8166195	0,05	1,04	0,93934	-0,58	-1,50	0,47044	NM_005089	ARG2
7975268	0,05	1,04	0,85383	2,76	6,79	0,00306	NM_001172	ACAA1
8086201	0,05	1,04	0,84103	-0,70	-1,63	0,25120	NM_001607	ZNF284
8029368	0,05	1,04	0,73610	0,86	1,82	0,02109	NM_001037813	MELK
8155214	0,05	1,04	0,87207	1,92	3,79	0,00013	NM_014791	TPM3
7920472	0,05	1,04	0,76647	-0,63	-1,55	0,04595	NM_152263	---
8158212	0,05	1,04	0,92889	-0,34	-1,26	0,54976	--- RBM8A	
7904751	0,05	1,04	0,78944	0,16	1,12	0,67758	BC017770 OTUD6A	
8168083	0,05	1,04	0,93075	-1,18	-2,26	0,02205	NM_207320	PRDM9
8104634	0,05	1,03	0,80977	-0,88	-1,84	0,15608	NM_020227	XYLT1
7999754	0,05	1,03	0,88918	-1,35	-2,55	0,06252	NM_022166	GYPC

8045009	0,05	1,03	0,83093	-2,90	-7,48	0,00033	NM_002101	ERP29
7958819	0,05	1,03	0,92873	0,86	1,81	0,10498	NM_006817	---
8136161	0,05	1,03	0,85815	-1,36	-2,57	0,04016	--- SNORA55	
7915227	0,05	1,03	0,87551	-0,48	-1,40	0,36943	NR_002983	CACNB1
8014794	0,05	1,03	0,91895	2,00	4,01	0,00103	NM_199247	SLC25A20
8087224	0,05	1,03	0,90050	0,23	1,17	0,42137	NM_000387	ZSCAN12
8124574	0,05	1,03	0,89461	1,02	2,03	0,05310	NR_028077	HAO2
7904396	0,05	1,03	0,87418	-0,76	-1,69	0,00297	NM_001005783	FAM74A3
8155345	0,05	1,03	0,93728	-1,37	-2,59	0,00557	ENST00000377629	---
8151869	0,05	1,03	0,91483	0,05	1,04	0,73570	--- ODF2	
8158269	0,05	1,03	0,82392	0,62	1,54	0,07356	NM_002540	HIATL2
8162645	0,05	1,03	0,91293	1,47	2,76	0,02693	NR_002894	DMD
8171921	0,05	1,03	0,78339	-0,87	-1,83	0,01935	NM_000109	LOH12CR1
7954012	0,05	1,03	0,82962	0,78	1,72	0,12002	NM_058169	CATSPER2P1
7988208	0,05	1,03	0,92255	0,47	1,39	0,67396	NR_002318	C6orf141
8120176	0,05	1,03	0,93416	1,91	3,76	0,01942	NM_001145652	RNF144A
8040090	0,05	1,03	0,89117	1,30	2,46	0,00013	NM_014746	FAHD2A
8043564	0,05	1,03	0,91688	1,00	2,00	0,07422	NM_016044	EFCAB2
7911096	0,05	1,03	0,81578	0,78	1,72	0,07609	NR_026588	---
8022275	0,05	1,03	0,73921	-0,72	-1,65	0,00201	--- PPFIA2	
7965166	0,05	1,03	0,88585	-1,19	-2,28	0,04899	NM_003625	OAS1
7958884	0,05	1,03	0,72536	-1,77	-3,42	0,00091	NM_016816	TLN2
7984019	0,05	1,03	0,77881	-0,25	-1,19	0,52085	NM_015059	C10orf55
7934505	0,05	1,03	0,86199	-0,31	-1,24	0,04409	NM_001001791	GUSBL2
8127364	0,04	1,03	0,96409	2,54	5,83	0,02702	NR_003660	KLHL11
8015456	0,04	1,03	0,91440	0,91	1,88	0,01598	NM_018143	SCUBE2
7946454	0,04	1,03	0,73682	-0,21	-1,15	0,19905	NM_020974	DCK
8095574	0,04	1,03	0,94546	2,00	4,00	0,00165	NM_000788	---
7993774	0,04	1,03	0,90686	-0,56	-1,47	0,01170	--- ABCB4	
8140752	0,04	1,03	0,89556	-0,82	-1,77	0,00320	NM_000443	---
8133273	0,04	1,03	0,93687	0,08	1,05	0,88181	--- MRPS30	
8105181	0,04	1,03	0,89946	3,25	9,53	0,00003	NM_016640	KRTAP13-2
8069813	0,04	1,03	0,88211	-0,32	-1,25	0,20986	NM_181621	PRKD2
8037794	0,04	1,03	0,89299	0,95	1,93	0,06162	NM_016457	CENPP
8156341	0,04	1,03	0,80092	0,10	1,07	0,55845	NM_001012267	OR52I1
7937948	0,04	1,03	0,91559	-0,68	-1,61	0,08554	NM_001005169	ALAD
8163509	0,04	1,03	0,90056	-0,01	-1,01	0,97988	NM_000031	MRGPRF
7950005	0,04	1,03	0,77084	-1,17	-2,26	0,02065	NM_001098515	CMAS
7954407	0,04	1,03	0,91796	1,38	2,61	0,00088	NM_018686	MIRLET7A2
7952313	0,04	1,03	0,94355	-1,36	-2,57	0,00260	NR_029477	GTF2F1
8033135	0,04	1,03	0,91461	1,25	2,38	0,02974	NM_002096	---
7947989	0,04	1,03	0,92868	-0,73	-1,66	0,00143	--- C5	
8163839	0,04	1,03	0,90143	1,95	3,85	0,00497	NM_001735	MRPL10
8016366	0,04	1,03	0,92845	-0,19	-1,14	0,78629	NM_145255	PLEKHA8
8132045	0,04	1,03	0,87385	1,17	2,25	0,08396	NM_032639	HIC2
8071536	0,04	1,03	0,91632	0,33	1,26	0,53230	NM_015094	LOC390940
8029306	0,04	1,03	0,80713	-1,27	-2,42	0,03851	AK129550 LDLRAP1	
7899023	0,04	1,03	0,92754	0,20	1,15	0,68327	NM_015627	PUS7
8142061	0,04	1,03	0,81656	2,33	5,02	0,00624	NM_019042	ACOX3
8099304	0,04	1,03	0,88815	0,86	1,81	0,03491	NM_003501	RIC8B
7958331	0,04	1,03	0,93474	0,79	1,72	0,04118	NM_018157	SLC25A27
8120067	0,04	1,03	0,73208	-0,48	-1,39	0,09844	NM_004277	RG9MTD1
8081343	0,04	1,03	0,95646	0,81	1,76	0,01590	NM_017819	BRD4
8034983	0,04	1,03	0,85675	0,63	1,55	0,00518	NM_058243	METTL5
8056716	0,04	1,03	0,95462	1,10	2,14	0,01415	NM_014168	RNU4ATAC
8044961	0,04	1,03	0,79298	-1,25	-2,38	0,01898	NR_023343	JMJD4
7924823	0,04	1,03	0,92070	0,42	1,34	0,24515	NM_023007	C1orf77
7905589	0,04	1,03	0,91275	1,36	2,57	0,01704	NM_015607	CHRNA3
7990714	0,04	1,03	0,88420	-0,97	-1,96	0,01836	NM_000743	C2CD2L
7944418	0,04	1,03	0,91320	1,05	2,07	0,00856	NM_014807	CYP3A7
8141342	0,04	1,03	0,86529	-1,05	-2,08	0,00422	NM_000765	---
7951668	0,04	1,03	0,95910	-0,96	-1,95	0,10107	--- PINK1	
7913249	0,04	1,03	0,89586	0,28	1,21	0,37307	NM_032409	LAT
7994535	0,04	1,03	0,88861	-0,30	-1,23	0,46676	NM_014387	PNP
7973067	0,04	1,03	0,90418	3,37	10,32	0,00004	NM_000270	RPIA
8043413	0,04	1,03	0,93073	2,54	5,83	0,00070	NM_144563	PCYT2
8019778	0,04	1,03	0,92504	1,24	2,36	0,03107	NM_002861	C22orf33
8075850	0,04	1,03	0,92159	-1,03	-2,04	0,00068	NM_001163857	BANK1
8096617	0,04	1,03	0,78895	0,66	1,58	0,09906	NM_017935	WHAMML1
7986687	0,04	1,03	0,85764	0,91	1,88	0,02927	NR_003521	COL4A2
7970033	0,04	1,03	0,90813	-0,31	-1,24	0,38794	NM_001846	DOK6
8021695	0,04	1,03	0,93299	-1,25	-2,37	0,00404	NM_152721	PGAP3
8014871	0,04	1,03	0,94403	0,98	1,97	0,06356	NM_033419	RG9MTD2

8101916	0,04	1,03	0,89261	-0,33	-1,26	0,30322	NM_152292	RPL13AP3
7974531	0,04	1,03	0,65852	0,23	1,17	0,41690	NR_004844	SNORD46
7901048	0,04	1,03	0,92578	-0,11	-1,08	0,64913	NR_000024	ORAI3
7994981	0,04	1,03	0,93513	0,01	1,01	0,96155	NM_152288	CCT6B
8014172	0,04	1,03	0,85930	-0,36	-1,28	0,32531	NM_006584	ROBO2
8081001	0,04	1,03	0,93932	-1,73	-3,32	0,00507	NM_002942	RLIM
8168432	0,04	1,03	0,92758	0,02	1,01	0,97374	NM_016120	SNORA70E
7950751	0,04	1,03	0,89272	-1,28	-2,42	0,00700	NR_033347	VAMP8
8043197	0,04	1,03	0,93661	2,51	5,70	0,00029	NM_003761	AHRR
8104180	0,04	1,03	0,75438	0,33	1,25	0,26450	NM_020731	LPPR5
7917946	0,04	1,03	0,81966	-1,53	-2,89	0,00602	NM_001037317	RNF4
8093590	0,04	1,03	0,82992	1,30	2,47	0,00538	NM_002938	C11orf61
7952445	0,04	1,03	0,82737	0,63	1,54	0,07367	NM_024631	---
8088770	0,04	1,03	0,92983	-0,18	-1,14	0,26135	--- NUP85	
8009761	0,04	1,03	0,92905	2,18	4,52	0,00229	NM_024844	TRAF3
7977018	0,04	1,03	0,88821	1,74	3,34	0,00032	NM_145725	DCLK3
8086120	0,04	1,03	0,88692	-0,46	-1,37	0,03620	NM_033403	EXOSC5
8037023	0,04	1,03	0,92480	2,15	4,44	0,01077	NM_020158	GIPC1
8034827	0,04	1,02	0,93945	0,11	1,08	0,80935	NM_005716	PLXNA3
8170921	0,04	1,02	0,92640	0,79	1,73	0,03073	NM_017514	C22orf39
8074458	0,04	1,02	0,95251	2,35	5,10	0,00071	NM_173793	C15orf51
7986520	0,04	1,02	0,98666	-1,94	-3,83	0,03049	ENST00000423248	---
8159959	0,03	1,02	0,95506	-1,23	-2,34	0,01955	--- TOX3	
8001394	0,03	1,02	0,84099	-0,38	-1,30	0,33783	NM_001080430	TSPAN17
8110224	0,03	1,02	0,90191	1,83	3,57	0,01248	NM_012171	HSP90AB5P
8080987	0,03	1,02	0,85839	-1,16	-2,23	0,06962	AY956766 SIRT5	
8116956	0,03	1,02	0,93717	0,87	1,83	0,03412	NM_031244	GEMIN4
8010946	0,03	1,02	0,92241	1,49	2,81	0,00967	NM_015721	SNORA45
7938293	0,03	1,02	0,91974	-0,64	-1,55	0,19680	NR_002977	VDAC1
8114145	0,03	1,02	0,92432	0,67	1,59	0,09049	NM_003374	OCLM
7908347	0,03	1,02	0,86802	-1,01	-2,02	0,09569	NM_022375	PRPF31
8031102	0,03	1,02	0,88265	1,06	2,08	0,09535	NM_015629	THOC3
8115886	0,03	1,02	0,81332	1,33	2,51	0,00082	NM_032361	FBXO33
7978748	0,03	1,02	0,92076	0,94	1,92	0,06363	NM_203301	MYBBP1A
8011640	0,03	1,02	0,90730	1,80	3,47	0,02499	NM_014520	NCOA5
8066668	0,03	1,02	0,89329	1,31	2,49	0,00604	NM_020967	SCN3A
8056376	0,03	1,02	0,84340	-0,52	-1,44	0,10933	NM_006922	BRD9
8110734	0,03	1,02	0,87527	0,67	1,59	0,10143	NR_027633	HNRNPA1
7955890	0,03	1,02	0,96893	1,40	2,64	0,02965	NM_031157	---
7898535	0,03	1,02	0,84158	-0,10	-1,07	0,62785	--- MDN1	
8128138	0,03	1,02	0,80830	0,91	1,87	0,00374	NM_014611	TRMU
8073875	0,03	1,02	0,92544	0,36	1,29	0,13108	NM_018006	SNORD38A
7901050	0,03	1,02	0,88894	-0,12	-1,09	0,56252	NR_001456	FLJ40536
7936916	0,03	1,02	0,88971	-1,03	-2,05	0,00173	AK097855 ---	
8157191	0,03	1,02	0,97354	-1,87	-3,66	0,00614	--- C9orf70	
8154132	0,03	1,02	0,90839	-1,06	-2,08	0,01458	NR_026663	MAOB
8172204	0,03	1,02	0,92136	-0,43	-1,35	0,02731	NM_000898	ZNF618
8157362	0,03	1,02	0,91426	2,13	4,38	0,00075	NM_133374	CTSL3
8156240	0,03	1,02	0,91004	-0,57	-1,49	0,32879	NR_027917	WDR5B
8089993	0,03	1,02	0,95086	0,53	1,44	0,22524	NM_019069	RABGAP1L
7907492	0,03	1,02	0,91765	0,72	1,65	0,04518	NM_014857	SPAG7
8011765	0,03	1,02	0,94628	1,01	2,01	0,00033	NM_004890	KRTAP11-1
8069872	0,03	1,02	0,96300	-1,17	-2,25	0,01620	NM_175858	MGC27345
8142770	0,03	1,02	0,93290	0,55	1,46	0,01376	ENST00000424840	C1orf38
7899394	0,03	1,02	0,92779	1,47	2,77	0,02173	NM_001105556	SPEN
7898278	0,03	1,02	0,94990	2,63	6,17	0,00063	NM_015001	GTF2IRD1
8133477	0,03	1,02	0,91013	1,77	3,40	0,02670	NM_016328	CBFA2T2
8061919	0,03	1,02	0,94770	1,38	2,61	0,00028	NM_005093	APCDD1
8020141	0,03	1,02	0,95953	-0,80	-1,74	0,02081	NM_153000	MON1B
7997321	0,03	1,02	0,95562	1,65	3,15	0,00767	NM_014940	EPOR
8034217	0,03	1,02	0,90267	0,02	1,01	0,92732	NM_000121	MPHOSPH8
7967881	0,03	1,02	0,92907	0,92	1,89	0,03541	NM_017520	MBNL3
8175177	0,03	1,02	0,94295	0,41	1,32	0,11580	NM_018388	ZNF708 // ZNF708
8020377	0,03	1,02	0,92080	-0,48	-1,40	0,09805	ENST00000356929 // ENST00000356929	

7906175	0,03	1,02	0,93501	-1,29	-2,45	0,01126	--- ZNF830	
8006477	0,03	1,02	0,92624	1,27	2,42	0,06771	NM_052857	MFSD9
8054356	0,03	1,02	0,93659	1,26	2,39	0,07394	NM_032718	TM4SF4
8083301	0,03	1,02	0,92016	-0,21	-1,15	0,36398	NM_004617	KLHL23
8046186	0,03	1,02	0,92811	0,11	1,08	0,73948	NM_144711	PRSS27
7998843	0,03	1,02	0,95500	-0,41	-1,33	0,38042	NM_031948	ZNF749
8031748	0,03	1,02	0,94617	1,82	3,53	0,00591	NM_001023561	LY6G6D
8118242	0,03	1,02	0,94560	-0,38	-1,30	0,32078	NM_021246	RPPH1

7977507	0,03	1,02	0,96795	-2,33	-5,03	0,02723	NR_002312	ARHGEF10
8144281	0,03	1,02	0,81125	0,42	1,34	0,09614	NM_014629	---
7896738	0,03	1,02	0,95075	-1,42	-2,67	0,00080	---	RPL37AP8
7951660	0,03	1,02	0,92146	0,43	1,35	0,40795	ENST00000317197	RILPL2
7967456	0,03	1,02	0,90461	0,61	1,53	0,09400	NM_145058	LOC100131506
8040221	0,03	1,02	0,93280	-0,64	-1,56	0,02373	AF504647	MCTS1
8169701	0,03	1,02	0,94985	-0,30	-1,23	0,17883	NM_014060	TOP3A
8013222	0,03	1,02	0,97286	1,54	2,91	0,00464	NM_004618	RGS7BP
8105596	0,02	1,02	0,91771	-1,04	-2,06	0,08544	NM_001029875	POMT1
8158866	0,02	1,02	0,92997	0,86	1,82	0,20930	NM_001136113	KRT23
8015133	0,02	1,02	0,92080	-0,91	-1,88	0,16347	NM_015515	CMTM6
8085999	0,02	1,02	0,91364	0,55	1,47	0,28457	NM_017801	EMP2
7999387	0,02	1,02	0,96527	3,07	8,40	0,00423	NM_001424	SMYD4
8011200	0,02	1,02	0,90974	0,96	1,94	0,00057	NM_052928	ADCY2
8104394	0,02	1,02	0,93814	-0,59	-1,50	0,00604	NM_020546	LONRF3
8169603	0,02	1,02	0,91741	1,79	3,47	0,00045	NM_001031855	CCNL2
8039909	0,02	1,02	0,92896	1,49	2,82	0,00005	NM_030937	SH3RF3
8044295	0,02	1,02	0,97281	0,02	1,02	0,97870	NM_001099289	BAALC
8147756	0,02	1,02	0,93072	-1,75	-3,37	0,00429	NM_024812	HIST1H3I
8124531	0,02	1,02	0,96848	3,08	8,45	0,00038	NM_003533	DNAJA2
7995379	0,02	1,02	0,97265	-0,45	-1,36	0,07880	BC013044	HLA-DMB
8180078	0,02	1,02	0,96356	3,84	14,28	0,00023	NM_002118	---
8098165	0,02	1,02	0,79956	-0,60	-1,51	0,05807	---	---
8089036	0,02	1,02	0,95878	-0,05	-1,04	0,81183	---	OR52B6
7938010	0,02	1,01	0,97480	-2,16	-4,48	0,00635	NM_001005162	OR5D14
7939942	0,02	1,01	0,94920	-1,54	-2,91	0,00055	NM_001004735	KIAA1920
7985560	0,02	1,01	0,97120	0,32	1,25	0,58275	ENST00000456932	PPP2R4
8158523	0,02	1,01	0,95307	1,12	2,18	0,05245	NM_178001	CYP2S1
8028991	0,02	1,01	0,95383	1,07	2,10	0,04946	NM_030622	NFASC
7909027	0,02	1,01	0,90083	-1,35	-2,55	0,01276	NM_001005388	NR3C2
8103094	0,02	1,01	0,96657	0,41	1,33	0,01141	NM_000901	GAS8
7998103	0,02	1,01	0,97259	0,86	1,81	0,02969	NM_001481	CCL25
8025375	0,02	1,01	0,96892	-1,05	-2,07	0,03131	NM_005624	C11orf74
7939411	0,02	1,01	0,92367	-0,05	-1,03	0,81121	AK290833	RPL14
8078984	0,02	1,01	0,96437	0,86	1,82	0,00630	NM_003973	IL13
8107970	0,02	1,01	0,91080	-1,31	-2,49	0,00059	NM_002188	NDUFS6
8104298	0,02	1,01	0,96436	0,17	1,13	0,50844	NM_004553	DKK2
8102200	0,02	1,01	0,95936	-0,67	-1,59	0,01699	NM_014421	ZNF33A
7927062	0,02	1,01	0,93863	1,00	2,00	0,23640	NM_006954	USP5
7953483	0,02	1,01	0,97515	1,46	2,75	0,01479	NM_001098536	NAA20
8061262	0,02	1,01	0,96130	1,28	2,42	0,00226	NM_016100	MYF5
7957379	0,02	1,01	0,95961	-0,71	-1,64	0,05056	NM_005593	ZNF233
8029423	0,02	1,01	0,93579	-0,38	-1,30	0,21925	NM_181756	GUCY1B2
7971671	0,02	1,01	0,89856	0,40	1,32	0,14786	NR_003923	HN1L
7992337	0,02	1,01	0,97979	2,52	5,74	0,00377	NM_144570	---
7938074	0,02	1,01	0,94416	-1,25	-2,37	0,01238	---	PTK7
8119689	0,02	1,01	0,93704	1,08	2,11	0,06039	NM_002821	OXTR
8085138	0,02	1,01	0,94257	-0,30	-1,23	0,31435	NM_000916	MGC42105
8105146	0,02	1,01	0,92066	-0,88	-1,84	0,03794	NM_153361	ZXDB
8167942	0,02	1,01	0,97310	0,76	1,69	0,08048	NM_007157	PPIAL4A
7905051	0,02	1,01	0,97807	-1,63	-3,09	0,00188	NM_178230	PPIAL4A
7919162	0,02	1,01	0,97807	-1,63	-3,09	0,00188	NM_178230	ZNF445
8086482	0,02	1,01	0,95985	1,94	3,83	0,00087	NM_181489	TMEM171
8106170	0,02	1,01	0,96359	0,45	1,37	0,06588	NM_173490	OPN1SW
8142814	0,02	1,01	0,97336	-0,96	-1,94	0,01204	NM_001708	ZCCHC2
8021546	0,02	1,01	0,95586	1,09	2,13	0,03768	NM_017742	ULK1
7959957	0,02	1,01	0,95254	1,75	3,36	0,04605	NM_003565	INTS6
7971692	0,02	1,01	0,92268	0,66	1,58	0,03843	NM_012141	CYTSB
8005661	0,02	1,01	0,96358	1,42	2,67	0,00084	NM_001033553	S100A2
7920285	0,02	1,01	0,94897	-0,15	-1,11	0,64520	NM_005978	TTC15
8040018	0,02	1,01	0,97896	2,02	4,05	0,00011	NM_016030	WDR74
7948881	0,02	1,01	0,97272	3,42	10,73	0,01377	NM_018093	POLR3G
8106820	0,02	1,01	0,98565	1,05	2,08	0,01504	NM_006467	LMNA
7906085	0,01	1,01	0,94432	0,76	1,69	0,29654	NM_170707	TECTA
7944632	0,01	1,01	0,96593	-0,35	-1,27	0,37129	NM_005422	RBL1
8062347	0,01	1,01	0,92114	-0,76	-1,69	0,04544	NM_002895	LGR5
7957140	0,01	1,01	0,98249	-0,57	-1,49	0,07140	NM_003667	---
8022418	0,01	1,01	0,96761	0,48	1,39	0,10706	---	---
7974695	0,01	1,01	0,93416	-0,04	-1,02	0,88684	---	ELMOD1
7943562	0,01	1,01	0,95384	1,31	2,49	0,00892	NM_018712	NR4A3
8156848	0,01	1,01	0,93727	0,53	1,45	0,15190	NM_006981	ELK1
8172345	0,01	1,01	0,97687	-0,52	-1,43	0,08816	NM_001114123	LOC441666 // LOC441666

8045330	0,01	1,01	0,94192	-0,38	-1,30	0,18178	NR_024380 // NR_024380	LZTS2
7929911	0,01	1,01	0,97749	1,08	2,11	0,07301	NM_032429	MTERFD3
7966046	0,01	1,01	0,96039	0,22	1,17	0,35695	NM_001033050	SNORA2A
7962827	0,01	1,01	0,92123	-0,65	-1,57	0,04431	NR_002950	RANGRF
8004832	0,01	1,01	0,96422	0,00	-1,00	0,98733	NM_001177802	KIAA1539
8160981	0,01	1,01	0,97126	0,18	1,13	0,55798	BC004406 ZNF282	
8137101	0,01	1,01	0,95881	0,82	1,76	0,00595	NM_003575	---
8036363	0,01	1,01	0,98437	-1,09	-2,12	0,06609	--- NUDT2	
8154885	0,01	1,01	0,95203	0,30	1,23	0,01828	NM_001161	EFHA2
8144742	0,01	1,01	0,96633	-0,69	-1,61	0,13695	NM_181723	HSD17B7P2
7927082	0,01	1,01	0,98834	0,01	1,01	0,98429	NR_003086	---
8165684	0,01	1,01	0,99402	-3,82	-14,10	0,00308	--- MITD1	
8054192	0,01	1,01	0,97823	0,93	1,90	0,05154	NM_138798	C6orf1
8125818	0,01	1,01	0,97839	-0,31	-1,24	0,29131	NM_178508	C9orf89
8156404	0,01	1,01	0,98257	2,66	6,34	0,01868	NM_032310	PAPOLB
8137927	0,01	1,01	0,98900	-1,13	-2,19	0,02804	NM_020144	RPL32P3
8090559	0,01	1,01	0,97264	1,86	3,63	0,00614	NR_003111	RXRB
8180111	0,01	1,01	0,99057	1,97	3,93	0,02828	NM_021976	---
7969372	0,01	1,01	0,97470	-0,65	-1,57	0,00553	--- KRTAP5-5	
7937700	0,01	1,01	0,98463	-0,48	-1,40	0,17288	NM_001001480	CD177
8029280	0,01	1,01	0,98236	-1,23	-2,34	0,00179	NM_020406	HCST
8028104	0,01	1,01	0,98894	-0,70	-1,62	0,09528	NM_014266	CARHSP1
7999319	0,01	1,01	0,98229	0,56	1,47	0,54883	NM_014316	BHLHA15
8134452	0,01	1,01	0,98487	0,14	1,10	0,70361	NM_177455	BST2
8035304	0,01	1,01	0,98209	-0,70	-1,62	0,08209	NM_004335	NCKIPSD
8087182	0,01	1,01	0,98106	1,63	3,09	0,00399	NM_016453	LZIC
7912292	0,01	1,01	0,98577	1,06	2,09	0,02794	NM_032368	ZNF214
7946288	0,01	1,01	0,98751	-0,15	-1,11	0,42257	NM_013249	FHOD3
8020973	0,01	1,00	0,98706	1,26	2,39	0,01339	NM_025135	NR2F6
8035249	0,01	1,00	0,97773	0,49	1,41	0,40050	NM_005234	AMBRA1
7947652	0,01	1,00	0,96025	0,14	1,10	0,70557	NM_017749	BAHD1
7982738	0,01	1,00	0,93766	1,40	2,64	0,00417	NM_014952	F2RL1
8106403	0,01	1,00	0,97899	2,49	5,64	0,00037	NM_005242	NIPA1
7986675	0,01	1,00	0,98808	2,25	4,76	0,01886	NM_144599	HIST1H3B
8124388	0,01	1,00	0,97699	1,19	2,28	0,01404	NM_003537	VASN
7992967	0,01	1,00	0,97433	-0,73	-1,66	0,09114	NM_138440	C15orf51
7986517	0,01	1,00	0,99776	-1,79	-3,45	0,08731	ENST00000423248	C15orf51
7986522	0,01	1,00	0,99776	-1,79	-3,45	0,08731	ENST00000423248	ZNF649
8038913	0,01	1,00	0,98639	-0,84	-1,79	0,00119	NM_023074	---
7944333	0,01	1,00	0,99420	-1,57	-2,97	0,17888	--- ZFP30	
8036420	0,01	1,00	0,97800	0,33	1,26	0,07396	NM_014898	---
8169445	0,01	1,00	0,97928	-0,01	-1,01	0,96953	--- HOXA11	
8138765	0,01	1,00	0,98102	-0,93	-1,90	0,00025	NM_005523	TK1
8018849	0,00	1,00	0,99289	0,98	1,97	0,11260	NM_003258	HOXB2
8016438	0,00	1,00	0,97302	-0,50	-1,41	0,04726	NM_002145	GPR39
8045336	0,00	1,00	0,98751	3,86	14,56	0,00025	NM_001508	FSCB
7978756	0,00	1,00	0,99299	-0,26	-1,20	0,25337	NM_032135	ZNF75D
8175319	0,00	1,00	0,96822	0,69	1,62	0,01236	NM_007131	C2orf63
8052233	0,00	1,00	0,99045	-0,12	-1,09	0,78124	NM_152385	HSF1
8148824	0,00	1,00	0,98924	0,39	1,31	0,17237	NM_005526	UBAC1
8165064	0,00	1,00	0,99121	1,53	2,89	0,01297	NM_016172	SNORD116-18
7981984	0,00	1,00	0,99056	-0,75	-1,68	0,07910	NR_003333	MPHOSPH10
8042588	0,00	1,00	0,99196	0,31	1,24	0,32450	NM_005791	PHLPP1
8021565	0,00	1,00	0,98017	0,35	1,27	0,02302	NM_194449	SNORA42
7920873	0,00	1,00	0,99653	-1,69	-3,24	0,03632	NR_002974	---
8177046	0,00	1,00	0,99519	0,88	1,84	0,15807	--- IRS2	
7972745	0,00	1,00	0,99168	-0,19	-1,14	0,59439	NM_003749	EXO1
7910997	0,00	1,00	0,97803	1,85	3,61	0,00821	NM_130398	EXOC5
7974564	0,00	1,00	0,98466	-0,16	-1,11	0,35331	NM_006544	C7orf72
8132811	0,00	1,00	0,99480	-0,82	-1,76	0,04520	NM_001161834	UBE2L3
8071547	0,00	1,00	0,99486	-0,07	-1,05	0,83499	NR_028436	KCTD12
7972055	0,00	1,00	0,99615	-0,31	-1,24	0,23968	NM_138444	DNAJC17
7987554	0,00	1,00	0,99511	0,43	1,35	0,35408	NM_018163	SPR
8042696	0,00	1,00	0,99092	0,97	1,96	0,08251	NM_003124	IL18R1
8044035	0,00	1,00	0,98776	0,66	1,58	0,08965	NM_003855	NOV
8148049	0,00	1,00	0,99588	-0,03	-1,02	0,92240	NM_002514	TMEM68
8150846	0,00	1,00	0,99815	3,02	8,10	0,00016	NM_152417	PRDM8
8096032	0,00	1,00	0,99811	0,02	1,02	0,92844	NM_020226	LRP5
7942007	0,00	1,00	0,99763	2,34	5,06	0,00088	NM_002335	ENC1
8112615	0,00	1,00	0,99865	0,23	1,17	0,44670	NM_003633	FAM150A
8150751	0,00	1,00	0,99626	-0,49	-1,40	0,11238	NM_207413	ZNF225
8029385	0,00	1,00	0,99795	0,68	1,61	0,14425	NM_013362	ZNF229

8037467	0,00	1,00	0,99913	0,63	1,55	0,07105	NM_014518	RPS7 // RPS7 // RPS7 // RPS7
8005877	0,00	1,00	0,99955	1,54	2,91	0,00685	NM_001011 // NM_001011 // NM_001011 // NM_001011	MCM9
8129218	0,00	-1,00	0,99960	0,71	1,64	0,11248	NM_153255	HSPBAP1
8090030	0,00	-1,00	0,99939	-0,27	-1,20	0,23421	NM_024610	SHISA9
7993296	0,00	-1,00	0,99973	-1,35	-2,55	0,00319	NM_001145204	---
7927527	0,00	-1,00	0,99933	0,22	1,16	0,38786	---	---
7933593	0,00	-1,00	0,99933	0,22	1,16	0,38786	---	SOHLH2
7970975	0,00	-1,00	0,99789	0,75	1,69	0,04288	NM_017826	RCC1
7899462	0,00	-1,00	0,99706	1,72	3,29	0,00400	NM_001048194	PCYOX1L
8109149	0,00	-1,00	0,99655	2,33	5,03	0,03074	NM_024028	LOC100128816
7960362	0,00	-1,00	0,99819	0,08	1,06	0,78236	AY358109	MGAT1
8116484	0,00	-1,00	0,99768	-0,16	-1,12	0,73857	NM_001114618	DEFB121
8065563	0,00	-1,00	0,99597	-0,85	-1,80	0,00861	NM_001011878	RCBTB2
7971573	0,00	-1,00	0,99384	0,05	1,04	0,67300	NM_001268	---
8050225	0,00	-1,00	0,99057	-0,87	-1,83	0,00892	---	CRLS1
8060839	0,00	-1,00	0,99594	0,50	1,42	0,10347	NM_019095	RPL34
8096765	0,00	-1,00	0,99688	0,46	1,37	0,25147	NM_033625	SLC48A1
7955055	0,00	-1,00	0,99488	1,21	2,32	0,12441	NM_017842	TSPYL1
8129097	0,00	-1,00	0,99627	-0,50	-1,41	0,09557	NM_003309	HEATR3
7995456	0,00	-1,00	0,98510	0,42	1,34	0,32911	NM_182922	TASP1
8065018	0,00	-1,00	0,98838	1,22	2,32	0,00383	NM_017714	---
8171180	0,00	-1,00	0,99229	-0,52	-1,43	0,35210	---	DHX16
8178377	0,00	-1,00	0,99034	2,86	7,25	0,00048	NM_003587	GNL3L
8167797	0,00	-1,00	0,99333	1,81	3,51	0,00039	NM_019067	CNPY2
7964076	0,00	-1,00	0,99362	1,50	2,82	0,00785	NM_014255	CHST12
8131135	0,00	-1,00	0,99471	-0,35	-1,27	0,47311	NM_018641	FAU
7949377	0,00	-1,00	0,99054	0,46	1,38	0,18214	NM_001997	GOLGA6L1
7986598	0,00	-1,00	0,99615	-1,53	-2,88	0,01741	NM_001001413	---
8119196	0,00	-1,00	0,98803	-0,07	-1,05	0,43542	---	TMEM216
7940479	0,00	-1,00	0,99364	1,90	3,72	0,00010	NM_016499	FAM83D
8062571	0,00	-1,00	0,99015	1,65	3,14	0,07317	NM_030919	C1orf189
7920487	0,00	-1,00	0,98564	-0,65	-1,57	0,21994	BC127710	FBXO31
8003249	-0,01	-1,00	0,98292	0,47	1,38	0,22850	NM_024735	PHLDB1
7944302	-0,01	-1,00	0,98768	0,93	1,91	0,06206	NM_015157	ATF7IP2
7993167	-0,01	-1,00	0,97645	0,37	1,29	0,13657	NM_024997	SPSB1
7897449	-0,01	-1,00	0,98300	0,63	1,55	0,13930	NM_025106	HMBS
7944401	-0,01	-1,00	0,98586	1,87	3,65	0,10364	NM_000190	KIAA1841
8042168	-0,01	-1,00	0,98181	1,30	2,47	0,03399	NM_001129993	FBXO4
8105111	-0,01	-1,00	0,98418	1,73	3,33	0,01430	NM_012176	COL8A1
8081235	-0,01	-1,00	0,96479	-0,85	-1,80	0,00300	NM_001850	---
7901858	-0,01	-1,00	0,98703	-0,65	-1,57	0,02975	---	LOC646214 // LOC646214
7986603	-0,01	-1,00	0,97845	0,65	1,57	0,16775	NR_027053 // NR_027053	MID1IP1
8166797	-0,01	-1,00	0,99180	2,50	5,65	0,00767	NM_021242	EXTL3
8145624	-0,01	-1,00	0,98830	1,69	3,22	0,00485	NM_001440	YPEL1
8074780	-0,01	-1,00	0,98680	1,58	2,98	0,01114	NM_013313	ERV3
8139840	-0,01	-1,00	0,98257	2,02	4,05	0,00133	NM_001007253	LOC402644
8138797	-0,01	-1,00	0,97682	-0,39	-1,31	0,32326	NM_001126493	KIF21A
7962274	-0,01	-1,00	0,98165	2,70	6,50	0,00124	NM_001173464	ARID1B
8122971	-0,01	-1,00	0,99063	0,98	1,97	0,04000	NM_020732	SFRS16
8029592	-0,01	-1,00	0,97640	0,61	1,52	0,01950	NM_007056	HPS4
8075063	-0,01	-1,01	0,99282	1,86	3,62	0,00583	NM_022081	MCTP2
7986293	-0,01	-1,01	0,98039	4,19	18,22	0,00006	NM_018349	---
7971294	-0,01	-1,01	0,97818	-1,11	-2,15	0,00042	---	PRKRIP1
8135162	-0,01	-1,01	0,98276	0,98	1,97	0,01117	NM_024653	NIF3L1
8047356	-0,01	-1,01	0,97963	2,26	4,77	0,00013	NM_021824	ZNF542
8031632	-0,01	-1,01	0,96165	0,82	1,77	0,05054	NR_033418	ZNF221
8029331	-0,01	-1,01	0,97210	0,24	1,18	0,47408	NM_013359	---
8091861	-0,01	-1,01	0,97055	-0,81	-1,75	0,02998	---	SLC25A19
8018352	-0,01	-1,01	0,97437	1,42	2,68	0,00422	NM_001126121	---
8068494	-0,01	-1,01	0,96966	-0,48	-1,40	0,50051	---	LMNB1
8107706	-0,01	-1,01	0,98762	1,90	3,72	0,00104	NM_005573	TBCD
8010848	-0,01	-1,01	0,97070	1,43	2,70	0,03114	NM_005993	ZNF322A
8162669	-0,01	-1,01	0,97044	-0,11	-1,08	0,69051	NM_024639	WFDC11
8066569	-0,01	-1,01	0,96301	-0,25	-1,19	0,07924	NM_147197	PPIAL4A
7919407	-0,01	-1,01	0,98863	-1,41	-2,66	0,00157	NM_178230	SCN2A
8046020	-0,01	-1,01	0,98189	-1,19	-2,29	0,00216	NM_021007	C7orf34
8136832	-0,01	-1,01	0,96278	-0,64	-1,56	0,03702	NM_178829	DTX2
8133736	-0,01	-1,01	0,98521	0,32	1,25	0,31843	NM_020892	C17orf57
8007949	-0,01	-1,01	0,97623	0,00	-1,00	0,99377	NM_152347	PCK2
7973530	-0,01	-1,01	0,97889	1,43	2,69	0,03989	NM_004563	SNAPC5
7989924	-0,01	-1,01	0,97903	1,89	3,71	0,00612	NM_006049	SRY

8177038	-0,01	-1,01	0,97650	-1,57	-2,96	0,01179	NM_003140	CDCA4
7981525	-0,01	-1,01	0,96834	2,20	4,59	0,00482	NM_017955	HYAL3
8087596	-0,01	-1,01	0,85042	1,36	2,56	0,00546	NM_003549	RALGAPA1
7978653	-0,01	-1,01	0,98557	3,03	8,18	0,02244	NM_014990	NOL12
8072883	-0,01	-1,01	0,96262	1,52	2,87	0,00043	NM_024313	FAM60A
7962146	-0,01	-1,01	0,98628	3,08	8,45	0,00242	NM_001135811	CTRL
8002121	-0,01	-1,01	0,97435	-1,46	-2,76	0,00533	NM_001907	ERCC8
8112285	-0,01	-1,01	0,95004	0,02	1,01	0,96578	NM_000082	LOC338799
7967210	-0,01	-1,01	0,95167	0,84	1,79	0,03808	NR_002809	DCI
7998762	-0,01	-1,01	0,97924	1,98	3,95	0,00104	NM_001919	---
8116593	-0,01	-1,01	0,97596	-0,38	-1,30	0,43732	---	PCNXL2
7925089	-0,01	-1,01	0,94402	1,20	2,29	0,01499	NM_014801	CHRM4
7947649	-0,01	-1,01	0,95768	0,04	1,03	0,88867	NM_000741	ITPRIP
7936242	-0,01	-1,01	0,96913	0,58	1,50	0,17114	NM_033397	PTK2B
8145490	-0,01	-1,01	0,96641	0,30	1,23	0,56153	NM_173174	LRRC8B
7902861	-0,01	-1,01	0,95959	0,55	1,47	0,17549	NM_001134476	C19orf12
8035886	-0,01	-1,01	0,85252	0,82	1,77	0,07040	NM_031448	NLRX1
7944463	-0,01	-1,01	0,97282	1,22	2,33	0,14783	NM_024618	---
7923972	-0,01	-1,01	0,97056	-1,19	-2,28	0,00165	---	---
8114111	-0,01	-1,01	0,99161	-1,23	-2,34	0,03129	---	QPCT
8041508	-0,01	-1,01	0,97846	-0,23	-1,17	0,05422	NM_012413	CYP11B2
8153373	-0,01	-1,01	0,96411	-0,64	-1,56	0,03549	NM_000498	---
8114006	-0,01	-1,01	0,98062	-0,03	-1,02	0,91357	---	SPACA5
8167210	-0,01	-1,01	0,97116	-0,58	-1,49	0,14111	NM_205856	GLDN
7983704	-0,01	-1,01	0,97131	-0,78	-1,72	0,08754	NM_181789	NCAPG2
8144153	-0,01	-1,01	0,94717	2,29	4,88	0,00013	NM_017760	ZFYVE21
7977149	-0,01	-1,01	0,96779	1,73	3,31	0,04733	NM_024071	ABAT
7993126	-0,01	-1,01	0,95400	0,05	1,03	0,76990	NM_020686	WDTC1
7899289	-0,01	-1,01	0,98229	2,54	5,84	0,01191	NM_015023	ZNF182
8172369	-0,01	-1,01	0,97091	0,08	1,06	0,84619	NM_006962	ANO3
7939024	-0,02	-1,01	0,92898	-0,89	-1,85	0,00717	NM_031418	GORASP1
8086317	-0,02	-1,01	0,96775	0,96	1,94	0,10400	NM_031899	FAM108B1
8161727	-0,02	-1,01	0,89857	0,80	1,74	0,00711	NM_016014	C5orf35
8105456	-0,02	-1,01	0,96570	0,03	1,02	0,93632	NM_153706	TRIM49L
7943025	-0,02	-1,01	0,97411	-1,83	-3,55	0,00363	NM_001105522	TRIM49L
7950971	-0,02	-1,01	0,97411	-1,83	-3,55	0,00363	NM_001105522	---
7977435	-0,02	-1,01	0,98199	-1,18	-2,27	0,03841	---	SRGAP3
8085164	-0,02	-1,01	0,93078	-0,29	-1,23	0,10629	NM_014850	CRYGN
8143949	-0,02	-1,01	0,96523	-1,25	-2,37	0,05497	NM_144727	POP7
8134904	-0,02	-1,01	0,96156	0,81	1,75	0,01950	NM_005837	SFRS2
8018803	-0,02	-1,01	0,96460	2,08	4,23	0,00377	NM_003016	C4orf23
8094070	-0,02	-1,01	0,91167	0,68	1,61	0,01673	NM_152544	OR8G5
7944838	-0,02	-1,01	0,91604	-0,97	-1,96	0,03764	NM_001005198	ZNF250
8153920	-0,02	-1,01	0,96935	1,90	3,73	0,01439	NM_021061	PAK3
8169389	-0,02	-1,01	0,95758	-0,85	-1,80	0,11511	NM_002578	SNORD36B
8159006	-0,02	-1,01	0,89649	-0,33	-1,25	0,17666	NR_000017	ZUFSP
8129108	-0,02	-1,01	0,93761	0,46	1,37	0,19080	NM_145062	---
8108192	-0,02	-1,01	0,95420	-0,86	-1,82	0,14925	---	ZNF860
8078380	-0,02	-1,01	0,96620	0,25	1,19	0,47720	NM_001137674	MXD4
8098995	-0,02	-1,01	0,96927	0,40	1,32	0,36345	NM_006454	LSM6
8097679	-0,02	-1,01	0,91291	1,48	2,79	0,01909	NM_007080	GRHPR
8155250	-0,02	-1,01	0,91197	1,38	2,61	0,01668	NM_012203	MRPL55
7924853	-0,02	-1,01	0,94967	0,59	1,50	0,08888	NM_181462	BRIP1
8017262	-0,02	-1,01	0,96634	2,04	4,10	0,01559	NM_032043	IL4I1
8038487	-0,02	-1,01	0,96203	-0,02	-1,01	0,92521	NM_172374	FOXM1
7960340	-0,02	-1,01	0,97418	1,53	2,89	0,00352	NM_202002	TAS2R4
8136645	-0,02	-1,01	0,85119	-0,28	-1,22	0,09090	NM_016944	C16orf58
8001048	-0,02	-1,01	0,90210	-0,29	-1,22	0,73199	NM_022744	VIT
8041467	-0,02	-1,01	0,90326	-0,42	-1,34	0,11383	NM_053276	EWSR1
8072206	-0,02	-1,01	0,92871	-0,34	-1,26	0,23040	NM_013986	ZFP161
8022110	-0,02	-1,01	0,96454	1,77	3,42	0,00030	NM_001143823	SESN2
7899436	-0,02	-1,01	0,95209	1,24	2,36	0,06783	NM_031459	MTRF1
7971222	-0,02	-1,01	0,93615	1,02	2,02	0,00067	NM_004294	FANCB
8171381	-0,02	-1,01	0,95437	0,91	1,88	0,05386	NM_001018113	FABP7
8121784	-0,02	-1,01	0,95395	-1,13	-2,20	0,05921	NM_001446	UGT2A3
8095402	-0,02	-1,01	0,87861	-0,78	-1,72	0,22030	NM_024743	FGG
8103326	-0,02	-1,01	0,94315	-0,25	-1,19	0,03592	NM_021870	---
7965036	-0,02	-1,01	0,95923	1,15	2,22	0,08712	---	---
8072582	-0,02	-1,01	0,92501	-0,74	-1,67	0,14521	---	SRCAP
7994889	-0,02	-1,01	0,92084	2,61	6,09	0,00131	NM_006662	MIR107
7934959	-0,02	-1,01	0,92596	0,03	1,02	0,76044	NR_029524	---
7993451	-0,02	-1,01	0,97895	0,49	1,41	0,13433	---	---
7993586	-0,02	-1,01	0,97895	0,49	1,41	0,13433	---	---

7999594	-0,02	-1,01	0,97895	0,49	1,41	0,13433	---	---
7999750	-0,02	-1,01	0,97895	0,49	1,41	0,13433	---	ETF1
8114443	-0,02	-1,01	0,97818	1,46	2,75	0,02445	NM_004730	IFNA16
8160392	-0,02	-1,02	0,94563	-0,10	-1,07	0,71834	NM_002173	LOC441016
8095072	-0,02	-1,02	0,91472	-0,30	-1,23	0,30329	ENST00000312008	GOLGA8B
7987248	-0,02	-1,02	0,91722	0,92	1,90	0,00692	NR_027410	FAM74A3
8155354	-0,02	-1,02	0,95275	-1,47	-2,77	0,00609	ENST00000377629	ADPGK
7990231	-0,02	-1,02	0,97009	1,14	2,20	0,10304	NR_023318	SSX6
8167220	-0,02	-1,02	0,92881	-0,45	-1,37	0,32146	NR_028366	PRO3077
7932552	-0,02	-1,02	0,95510	-0,45	-1,37	0,04148	ENST00000451889	COL4A1
7972750	-0,02	-1,02	0,89584	-0,38	-1,30	0,04786	NM_001845	DGKB
8138310	-0,02	-1,02	0,91829	-0,98	-1,97	0,03199	NM_004080	C14orf37
7979400	-0,02	-1,02	0,95300	-0,69	-1,61	0,00409	AK292513 C2CD2	
8070538	-0,02	-1,02	0,93378	0,77	1,70	0,22757	NM_015500	JPH1
8151423	-0,02	-1,02	0,93775	1,67	3,19	0,00163	NM_020647	IRAK1BP1
8120826	-0,02	-1,02	0,97100	1,19	2,29	0,01340	NM_001010844	FASTK
8143863	-0,02	-1,02	0,96740	1,66	3,16	0,05937	NM_006712	DSERG1
8154128	-0,02	-1,02	0,93544	-0,44	-1,35	0,02911	DQ246450	HS3ST3B1
8005097	-0,02	-1,02	0,94426	-1,54	-2,90	0,00076	NM_006041	KRTAP5-4
7945657	-0,02	-1,02	0,97501	-1,09	-2,13	0,24962	NM_001012709	ARSD
8171148	-0,02	-1,02	0,95985	-1,27	-2,42	0,01463	NM_001669	PSKH1
7996720	-0,03	-1,02	0,88762	0,02	1,01	0,97187	NM_006742	---
8148619	-0,03	-1,02	0,98227	-1,55	-2,92	0,05791	---	NDUFC1
8102839	-0,03	-1,02	0,85437	0,53	1,44	0,02522	NM_002494	OR13C5
8162934	-0,03	-1,02	0,94824	-0,58	-1,49	0,05092	NM_001004482	---
8166569	-0,03	-1,02	0,97555	-0,96	-1,95	0,08277	---	C16orf57
7996174	-0,03	-1,02	0,91888	0,99	1,99	0,07433	NM_024598	ZNF90
8027258	-0,03	-1,02	0,92564	0,78	1,72	0,16213	AK298173 MAVS	
8060705	-0,03	-1,02	0,92383	0,40	1,32	0,25425	NM_020746	TPCN2
7942091	-0,03	-1,02	0,83597	0,36	1,28	0,40195	NM_139075	IFT27
8075828	-0,03	-1,02	0,93407	1,72	3,29	0,00566	NM_001177701	CCL20
8048864	-0,03	-1,02	0,86570	0,36	1,28	0,31664	NM_004591	NPRL2
8087669	-0,03	-1,02	0,92376	1,80	3,49	0,00078	NM_006545	---
8166824	-0,03	-1,02	0,84373	-0,68	-1,61	0,03484	---	DOT1L
8024391	-0,03	-1,02	0,94932	0,61	1,52	0,20730	NM_032482	DEM1
7900454	-0,03	-1,02	0,94536	0,02	1,02	0,94818	NM_022774	---
8117018	-0,03	-1,02	0,97115	-1,55	-2,93	0,02611	---	WFS1
8093906	-0,03	-1,02	0,93086	0,91	1,88	0,29398	NM_006005	PGBD1
8117675	-0,03	-1,02	0,84819	0,18	1,14	0,47818	NM_032507	ZNF133
8061154	-0,03	-1,02	0,87432	0,48	1,39	0,21764	NM_003434	SPANXN5
8172800	-0,03	-1,02	0,93504	-1,19	-2,29	0,00755	NM_001009616	SPACA5
8167228	-0,03	-1,02	0,94933	-0,77	-1,71	0,20155	NM_205856	PAFAH2
7913883	-0,03	-1,02	0,90978	0,82	1,77	0,11961	NM_000437	MECP2
8175998	-0,03	-1,02	0,94835	1,88	3,67	0,00299	NM_004992	MAP3K13
8084589	-0,03	-1,02	0,91559	0,20	1,15	0,38710	NM_004721	---
7910948	-0,03	-1,02	0,96866	-2,25	-4,76	0,15569	---	---
8065756	-0,03	-1,02	0,76854	0,49	1,41	0,13893	---	CYP3A4
8141317	-0,03	-1,02	0,93688	-1,21	-2,32	0,00211	NM_017460	KCNMB4
7957126	-0,03	-1,02	0,84075	0,97	1,96	0,01476	NM_014505	SNORA33
8122144	-0,03	-1,02	0,87835	-1,43	-2,70	0,03329	NR_002436	UPK2
7944341	-0,03	-1,02	0,94676	-0,21	-1,15	0,18557	NM_006760	PDGFA
8137670	-0,03	-1,02	0,87237	2,77	6,84	0,00013	NM_002607	ASB1
8049657	-0,03	-1,02	0,92079	1,68	3,21	0,00034	NM_001040445	---
8142448	-0,03	-1,02	0,84703	-0,70	-1,63	0,00187	---	CDADC1
7969096	-0,03	-1,02	0,94940	0,62	1,54	0,08878	NM_030911	ZNF385D
8085774	-0,03	-1,02	0,93692	-0,89	-1,86	0,00889	NM_024697	OR9A4
8136660	-0,03	-1,02	0,91125	-0,84	-1,79	0,01358	NM_001001656	---
8080960	-0,03	-1,02	0,80823	-0,46	-1,37	0,14577	---	DENND2C
7918751	-0,03	-1,02	0,85106	-0,26	-1,20	0,08893	BC063894 RNASE6	
7973101	-0,03	-1,02	0,89287	-0,90	-1,86	0,03595	NM_005615	---
8023935	-0,03	-1,02	0,94383	-1,34	-2,54	0,00367	---	WNT5A
8088180	-0,03	-1,02	0,91754	-0,32	-1,24	0,08345	NM_003392	ZNF324B
8031949	-0,03	-1,02	0,77562	-0,34	-1,26	0,06422	NM_207395	---
7985039	-0,03	-1,02	0,96651	-1,23	-2,34	0,23258	---	OR52E2
7946019	-0,03	-1,02	0,80448	-0,91	-1,88	0,01826	NM_001005164	SLITRK6
7972239	-0,03	-1,02	0,87201	-0,35	-1,28	0,03448	NM_032229	ARPC5L
8157828	-0,03	-1,02	0,84047	2,33	5,02	0,00161	NM_030978	EGFL6
8166079	-0,03	-1,02	0,88447	-0,34	-1,27	0,17342	NM_015507	DDX39
8034806	-0,04	-1,02	0,88462	0,96	1,95	0,01084	NM_005804	---
8169634	-0,04	-1,02	0,96610	-2,67	-6,37	0,01726	---	SLC37A1
8068810	-0,04	-1,02	0,87118	3,50	11,31	0,00081	NM_018964	C6orf57
8120579	-0,04	-1,02	0,83063	-0,18	-1,13	0,54459	NM_145267	TBC1D8
8054308	-0,04	-1,03	0,93951	2,51	5,70	0,00120	NM_001102426	GAK

8098789	-0,04	-1,03	0,92625	1,26	2,39	0,02986	NM_005255	---
8173206	-0,04	-1,03	0,96196	0,88	1,84	0,04688	---	OR6X1
7952373	-0,04	-1,03	0,93165	-1,65	-3,14	0,03764	NM_001005188	SLC6A8
7995292	-0,04	-1,03	0,87359	1,14	2,21	0,05958	NM_005629	GOLGA8B
7987279	-0,04	-1,03	0,77834	1,01	2,02	0,01041	NR_027410	ATP6V0D2
8147145	-0,04	-1,03	0,85041	-0,05	-1,04	0,84060	NM_152565	---
8044530	-0,04	-1,03	0,77486	-0,17	-1,12	0,49629	---	PALM
8024003	-0,04	-1,03	0,78831	0,34	1,26	0,44150	NM_002579	MIR195
8012004	-0,04	-1,03	0,84747	-0,65	-1,57	0,27501	NR_029712	MPHOSPH6
8003068	-0,04	-1,03	0,90808	0,10	1,07	0,48502	NM_005792	SAMD13
7902617	-0,04	-1,03	0,70938	-0,76	-1,69	0,01825	NM_001010971	GATA2
8090469	-0,04	-1,03	0,95523	-0,13	-1,09	0,19243	NM_032638	ARHGEF11
7921179	-0,04	-1,03	0,91428	2,61	6,12	0,00045	NM_198236	C17orf81
8004293	-0,04	-1,03	0,79780	2,64	6,25	0,00220	NM_203413	THRAP3
7900119	-0,04	-1,03	0,92949	2,36	5,13	0,01714	NM_005119	---
8144226	-0,04	-1,03	0,91980	0,10	1,07	0,50717	---	MIR214
7922326	-0,04	-1,03	0,95992	-1,04	-2,06	0,00120	NR_029627	TRIM35
8149907	-0,04	-1,03	0,92750	0,29	1,23	0,55308	NM_171982	MT1JP
7995803	-0,04	-1,03	0,94853	-0,06	-1,04	0,81347	AF348994	ACVRL1
7955562	-0,04	-1,03	0,71488	-0,87	-1,82	0,04834	NM_000020	KCND2
8135705	-0,04	-1,03	0,88650	-0,76	-1,69	0,00343	NM_012281	PPM1L
8083749	-0,04	-1,03	0,86970	0,04	1,03	0,87597	NM_139245	---
7974619	-0,04	-1,03	0,74540	-0,69	-1,62	0,00805	---	PPM1E
8008768	-0,04	-1,03	0,89137	0,99	1,99	0,02781	NM_014906	ZNF71
8031675	-0,04	-1,03	0,89753	0,98	1,98	0,12971	NM_021216	FAM71A
7909624	-0,04	-1,03	0,90255	-0,83	-1,78	0,00086	NM_153606	---
7908777	-0,04	-1,03	0,94544	2,24	4,74	0,00948	---	SCGB2A1
7940626	-0,04	-1,03	0,86851	-0,45	-1,37	0,23930	NM_002407	SLC26A6
8087119	-0,04	-1,03	0,89403	1,24	2,36	0,17480	NM_022911	KIAA1919
8121525	-0,04	-1,03	0,88349	1,33	2,52	0,00261	NM_153369	LOC100293211
7981728	-0,04	-1,03	0,97978	-4,84	-28,58	0,00016	ENST00000390601	ATRNL1
7930714	-0,04	-1,03	0,47800	-0,08	-1,06	0,66321	NM_207303	AIM1
8121277	-0,04	-1,03	0,85968	1,50	2,83	0,00216	NM_001624	MC1R
7998055	-0,04	-1,03	0,94072	-0,13	-1,10	0,84978	NM_002386	OR5AK3P
7939998	-0,04	-1,03	0,92530	-1,06	-2,08	0,04100	ENST00000326876	CXCL1
8095697	-0,04	-1,03	0,90801	1,17	2,24	0,04792	NM_001511	C5orf54
8115600	-0,04	-1,03	0,83470	0,28	1,22	0,38491	NM_022090	NFKBIB
8028552	-0,04	-1,03	0,88503	0,87	1,83	0,01565	NM_002503	DIP2C
7931683	-0,04	-1,03	0,90163	1,13	2,18	0,01840	NM_014974	MNT
8011262	-0,04	-1,03	0,88086	1,02	2,03	0,05822	NM_020310	GPRASP1
8168968	-0,04	-1,03	0,88750	-0,05	-1,03	0,87949	NM_014710	C6orf94
8122452	-0,04	-1,03	0,90737	-2,24	-4,71	0,00512	NM_001013623	SAE1
8029884	-0,04	-1,03	0,93178	0,91	1,88	0,16691	NR_027280	DNALI1
7900159	-0,04	-1,03	0,93600	-1,76	-3,39	0,00027	NM_003462	MBD6
7956470	-0,04	-1,03	0,90721	1,38	2,59	0,00231	NM_052897	OFD1
8166104	-0,04	-1,03	0,79293	0,95	1,93	0,02379	NM_003611	FAM74A3
8161467	-0,04	-1,03	0,91785	-1,37	-2,58	0,00164	ENST00000377629	ABHD14A
8080162	-0,04	-1,03	0,94113	2,58	5,97	0,00399	NM_015407	TCEAL7
8169015	-0,04	-1,03	0,94648	-1,65	-3,14	0,05675	NM_152278	MMP19
7963946	-0,04	-1,03	0,94229	-1,56	-2,94	0,02646	NM_002429	MLL2
7962951	-0,05	-1,03	0,78780	2,43	5,39	0,00171	NM_003482	KLC4
8119670	-0,05	-1,03	0,90467	1,29	2,45	0,01367	NM_201523	OR5AC2
8081192	-0,05	-1,03	0,75750	-1,06	-2,09	0,04993	NM_054106	ZIM3
8039625	-0,05	-1,03	0,91924	-0,84	-1,79	0,00063	NM_052882	C14orf183
7978917	-0,05	-1,03	0,73879	-1,72	-3,30	0,03060	NM_001014830	ZNF322A
8124459	-0,05	-1,03	0,84394	0,00	1,00	0,98624	NM_024639	---
8168779	-0,05	-1,03	0,58506	-0,57	-1,48	0,00610	---	FOXF1
7997726	-0,05	-1,03	0,82054	-1,67	-3,19	0,03072	NM_001451	CIR1
8056829	-0,05	-1,03	0,91192	-1,13	-2,19	0,02665	NM_004882	RRM2
8040223	-0,05	-1,03	0,90566	1,70	3,26	0,04737	NM_001165931	---
8174373	-0,05	-1,03	0,94304	-0,49	-1,41	0,09027	---	SETMAR
8077370	-0,05	-1,03	0,93920	-0,37	-1,29	0,28148	NM_006515	PDE4B
7902104	-0,05	-1,03	0,92440	-0,04	-1,03	0,79724	NM_002600	ORAOV1
7950016	-0,05	-1,03	0,90253	2,37	5,15	0,00018	NM_153451	MAOA
8166925	-0,05	-1,03	0,77864	-0,16	-1,11	0,53678	NM_000240	GFRAL
8120350	-0,05	-1,03	0,53859	-0,72	-1,65	0,01708	NM_207410	PYCARD
8001030	-0,05	-1,03	0,90919	-0,55	-1,47	0,04828	NM_013258	CDK1
7927710	-0,05	-1,03	0,90351	2,77	6,84	0,00043	NM_001786	RELA
7949465	-0,05	-1,03	0,88926	1,40	2,65	0,02564	NM_021975	TCF19
8179228	-0,05	-1,03	0,80716	0,52	1,44	0,16404	NM_007109	OR4N3P
7981785	-0,05	-1,03	0,81954	-0,66	-1,58	0,18468	NR_028067	DCAF11
7973545	-0,05	-1,03	0,76989	1,50	2,83	0,00839	NM_025230	GPATCH4
7921076	-0,05	-1,03	0,87721	2,36	5,15	0,00053	NM_182679	C20orf7

8061019	-0,05	-1,03	0,88372	0,98	1,97	0,00116	NR_029377	KRTAP20-3
8068164	-0,05	-1,03	0,69846	-0,78	-1,72	0,01256	NM_001128077	OR5C1
8157696	-0,05	-1,03	0,87253	-1,30	-2,46	0,14509	NM_001001923	SPIN2B
8173189	-0,05	-1,03	0,82587	-0,36	-1,29	0,42675	NM_001006681	PEX6
8126452	-0,05	-1,03	0,92493	1,13	2,19	0,05120	NM_000287	ACTC1
7987315	-0,05	-1,03	0,87648	0,44	1,36	0,35917	NM_005159	MIR382
7976842	-0,05	-1,03	0,87738	-1,05	-2,08	0,08284	NR_029874	C1orf63
7913824	-0,05	-1,03	0,78693	0,17	1,12	0,40531	NM_020317	NDUFB7
8034843	-0,05	-1,03	0,72264	0,00	1,00	0,99645	NM_004146	METT10D
8011275	-0,05	-1,03	0,78634	2,28	4,84	0,00039	NM_024086	TMEM81
7923707	-0,05	-1,03	0,85298	1,16	2,24	0,02755	NM_203376	FAM65A
7996571	-0,05	-1,03	0,89774	1,32	2,49	0,02829	NM_024519	EXOC3
8104201	-0,05	-1,04	0,93062	0,37	1,30	0,35751	NM_007277	CHRD
8084496	-0,05	-1,04	0,89236	-0,36	-1,28	0,26502	NM_003741	KHDC1
8127511	-0,05	-1,04	0,65659	-0,53	-1,45	0,04121	NM_030568	WRNIP1
8116595	-0,05	-1,04	0,90840	1,57	2,97	0,00030	NM_020135	ZDHHC24
7949645	-0,05	-1,04	0,76095	1,38	2,61	0,22473	NM_207340	MT1M
7995787	-0,05	-1,04	0,76324	1,26	2,40	0,06407	NM_176870	IL2
8102697	-0,05	-1,04	0,71978	-0,79	-1,73	0,08731	NM_000586	RBM10
8167042	-0,05	-1,04	0,73932	1,44	2,72	0,03383	NM_005676	NEK2
7924096	-0,05	-1,04	0,84498	1,32	2,50	0,02635	NM_002497	NDUFAF2
8050591	-0,05	-1,04	0,77362	-0,40	-1,32	0,01029	NM_174889	---
7921031	-0,05	-1,04	0,95879	-0,09	-1,06	0,90880	---	ANKRD6
8121095	-0,05	-1,04	0,89390	0,41	1,33	0,31108	NM_014942	---
8151215	-0,05	-1,04	0,83295	-1,05	-2,07	0,00043	---	KCNH7
8056303	-0,05	-1,04	0,88713	-1,06	-2,08	0,00257	NM_033272	MLF1
8083616	-0,05	-1,04	0,77069	1,05	2,07	0,03214	NM_001130157	NOM1
8137584	-0,05	-1,04	0,91729	0,89	1,86	0,09663	NM_138400	SLC6A8
8001082	-0,05	-1,04	0,80349	0,88	1,84	0,03550	NM_005629	UNC119B
7959212	-0,05	-1,04	0,57871	1,30	2,47	0,02466	NM_001080533	PPP1R3E
7977955	-0,05	-1,04	0,76384	0,72	1,64	0,01384	NR_026862	LSM5
8138912	-0,05	-1,04	0,76848	0,33	1,25	0,18719	NM_012322	ARMC2
8121370	-0,05	-1,04	0,89787	-0,72	-1,65	0,05837	NM_032131	HIST1H2BD
8117382	-0,05	-1,04	0,76335	1,87	3,65	0,05551	NM_021063	TPMT
8124134	-0,05	-1,04	0,83794	1,07	2,10	0,05897	NM_000367	---
7965871	-0,06	-1,04	0,83121	-1,83	-3,55	0,00391	---	RARG
7963631	-0,06	-1,04	0,88249	1,75	3,37	0,02083	NM_000966	LOC728819
8051864	-0,06	-1,04	0,81399	-0,39	-1,31	0,50003	NM_001101330	BRMS1L
7973948	-0,06	-1,04	0,80892	1,07	2,10	0,04066	NM_032352	CLCN7
7998510	-0,06	-1,04	0,92957	2,18	4,53	0,06078	NM_001287	OR1A1
8003804	-0,06	-1,04	0,78501	-0,55	-1,46	0,15784	NM_014565	---
7906808	-0,06	-1,04	0,88633	-1,60	-3,03	0,03101	---	MCART6
8174288	-0,06	-1,04	0,64938	0,20	1,15	0,33372	NM_001012755	RUVBL2
8030113	-0,06	-1,04	0,88840	1,68	3,20	0,00286	NM_006666	ZDHHC14
8122982	-0,06	-1,04	0,85962	1,46	2,75	0,06558	NM_024630	SNX8
8137833	-0,06	-1,04	0,91820	0,59	1,51	0,20468	NM_013321	DYNC2H1
7943498	-0,06	-1,04	0,80561	-0,03	-1,02	0,78547	NM_001377	RBM33
8137542	-0,06	-1,04	0,88658	1,38	2,60	0,00410	NM_053043	FLJ45248
8147721	-0,06	-1,04	0,86646	-0,80	-1,74	0,04245	AK127183	ACRV1
7952503	-0,06	-1,04	0,79944	-0,06	-1,04	0,87265	NM_001612	CLCF1
7949754	-0,06	-1,04	0,88621	1,34	2,54	0,06272	NM_013246	PSEN2
7910146	-0,06	-1,04	0,83850	0,95	1,94	0,16816	NM_000447	ASPHD2
8072108	-0,06	-1,04	0,87294	0,78	1,71	0,11675	NM_020437	CTSF
7949650	-0,06	-1,04	0,74263	2,19	4,57	0,03812	NM_003793	---
8001529	-0,06	-1,04	0,88391	-2,49	-5,62	0,00157	---	PGC
8126324	-0,06	-1,04	0,88373	-1,22	-2,32	0,03538	NM_002630	C1QTNF9B
7970595	-0,06	-1,04	0,82558	-1,08	-2,11	0,00815	NM_001007537	CDCP1
8086517	-0,06	-1,04	0,83788	3,43	10,77	0,00325	NM_022842	C11orf70
7943387	-0,06	-1,04	0,86203	-0,73	-1,66	0,08930	BC006128	POLA2
7941214	-0,06	-1,04	0,84898	1,96	3,90	0,00062	NM_002689	CES3
7996361	-0,06	-1,04	0,84030	-0,47	-1,39	0,31985	NM_024922	PCSK7
7951896	-0,06	-1,04	0,67009	0,27	1,21	0,54209	NM_004716	ALG12
8076851	-0,06	-1,04	0,82039	0,73	1,66	0,24692	NM_024105	AKD1
8128795	-0,06	-1,04	0,79318	0,57	1,49	0,14821	NM_001145128	GYPB
8102993	-0,06	-1,04	0,80787	-0,46	-1,37	0,11646	NM_002100	LIN28A
8065011	-0,06	-1,04	0,84296	-0,46	-1,38	0,02391	NM_024674	DHFRL1
8089029	-0,06	-1,04	0,58012	-0,01	-1,01	0,94631	NM_176815	SGOL1
8085754	-0,06	-1,04	0,89921	1,95	3,85	0,02235	NM_001012410	FAM171A2
8016033	-0,06	-1,04	0,80107	1,05	2,07	0,19721	NM_198475	C19orf44
8026568	-0,06	-1,04	0,86603	0,42	1,34	0,23565	NM_032207	---
8074059	-0,06	-1,04	0,53925	-0,08	-1,06	0,73911	---	BMPER
8132250	-0,06	-1,04	0,73159	-1,43	-2,70	0,00694	NM_133468	C14orf48
7976412	-0,06	-1,04	0,86124	0,06	1,04	0,60426	NR_024183	TRNAU1AP

7899486	-0,06	-1,04	0,77037	1,09	2,13	0,02162	NR_003109		C20orf96
8064322	-0,06	-1,04	0,76397	0,96	1,95	0,02592	NM_153269		PPP1R2P1
8125527	-0,06	-1,04	0,61579	-0,37	-1,29	0,12901	AF275684	PPP1R2P1	
8178882	-0,06	-1,04	0,61579	-0,37	-1,29	0,12901	AF275684	PPP1R2P1	
8180076	-0,06	-1,04	0,61579	-0,37	-1,29	0,12901	AF275684	FLJ38894	
7942771	-0,06	-1,04	0,76500	-1,17	-2,25	0,00373	AK096213	---	
8166667	-0,06	-1,04	0,86984	-0,45	-1,37	0,01701	---	TIGD1	
8059770	-0,06	-1,04	0,87652	1,73	3,32	0,00499	NM_145702		SLC47A1
8005603	-0,06	-1,04	0,84443	2,87	7,32	0,00338	NM_018242		---
8170633	-0,06	-1,04	0,89287	-0,25	-1,19	0,37858	---	---	
8175773	-0,06	-1,04	0,89287	-0,25	-1,19	0,37858	---	GLT25D2	
7922807	-0,06	-1,04	0,85928	0,90	1,87	0,03217	NM_015101		CD177
8037298	-0,06	-1,04	0,93014	-1,66	-3,16	0,06140	NM_020406		RPL23
8014749	-0,06	-1,04	0,74127	1,18	2,27	0,04764	NM_000978		LOC648987
8111922	-0,06	-1,04	0,76944	1,63	3,09	0,00312	ENST00000314957		GOLGA3
7967810	-0,06	-1,05	0,72293	0,63	1,55	0,10039	NM_005895		TAF4B
8020702	-0,06	-1,05	0,69175	2,74	6,67	0,00120	NM_005640		LOC100287934
7896754	-0,06	-1,05	0,74711	-0,45	-1,36	0,34232	AK290103	LNX1	
8100362	-0,06	-1,05	0,83311	-0,77	-1,71	0,00404	NM_001126328		C7orf25
8139228	-0,06	-1,05	0,90938	0,03	1,02	0,93992	NM_001099858		TNFSF13B
7969986	-0,06	-1,05	0,87864	-0,81	-1,75	0,00028	NM_006573		NIPAL4
8109563	-0,06	-1,05	0,74621	0,65	1,57	0,08246	NM_001099287		KLK12
8038747	-0,06	-1,05	0,82493	-0,54	-1,46	0,00281	NM_145894		KRT85
7963366	-0,06	-1,05	0,71078	-0,71	-1,64	0,12028	NM_002283		ZNF498
8134644	-0,06	-1,05	0,81922	0,02	1,01	0,95201	NM_145115		PDE12
8080676	-0,06	-1,05	0,85226	0,70	1,62	0,08441	NM_177966		---
8009253	-0,07	-1,05	0,86298	-0,56	-1,47	0,16340	---	TAOK2	
7994692	-0,07	-1,05	0,70381	1,09	2,13	0,01967	NM_016151		LMTK2
8134435	-0,07	-1,05	0,81947	1,94	3,85	0,00286	NM_014916		KDM4B
8024909	-0,07	-1,05	0,71826	0,39	1,31	0,25054	NM_015015		---
7953128	-0,07	-1,05	0,83878	-0,37	-1,29	0,22747	---	SCD5	
8101366	-0,07	-1,05	0,77187	0,60	1,51	0,00334	NM_001037582		HIST1H1D
8124430	-0,07	-1,05	0,75777	-0,56	-1,47	0,13720	NM_005320		GPR161
7922108	-0,07	-1,05	0,80662	-0,50	-1,41	0,02220	NM_153832		GREM2
7925452	-0,07	-1,05	0,78360	-0,85	-1,80	0,02364	NM_022469		---
8139365	-0,07	-1,05	0,52236	-0,86	-1,82	0,00491	---	SUDS3	
7959080	-0,07	-1,05	0,80482	0,41	1,33	0,14868	NM_022491		MIR222
8172268	-0,07	-1,05	0,73019	-0,74	-1,67	0,24758	NR_029636		CMC1
8078312	-0,07	-1,05	0,77800	-0,35	-1,28	0,48670	NM_182523		RIMKLB
7953765	-0,07	-1,05	0,89754	2,90	7,45	0,00019	NM_020734		LOC653113
7960889	-0,07	-1,05	0,90539	-0,13	-1,09	0,84568	NR_024254		C17orf101
8019494	-0,07	-1,05	0,86520	0,81	1,76	0,00515	NR_033265		RBM28
8142774	-0,07	-1,05	0,87779	1,68	3,20	0,00110	NM_018077		ANKRD16
7931888	-0,07	-1,05	0,85403	0,91	1,87	0,03604	NM_019046		JUP
8015412	-0,07	-1,05	0,83112	2,41	5,33	0,00398	NM_002230		ZNF692
7925773	-0,07	-1,05	0,78652	1,82	3,52	0,00066	NM_017865		ARMC4
7932744	-0,07	-1,05	0,88715	-0,19	-1,14	0,66631	NM_018076		ARAP1
7950197	-0,07	-1,05	0,75711	0,76	1,69	0,14588	NM_015242		TNXB
8125234	-0,07	-1,05	0,75807	-0,32	-1,25	0,24986	NM_019105		AMAC1L2
8144600	-0,07	-1,05	0,84992	-0,81	-1,76	0,10903	NM_054028		---
7924551	-0,07	-1,05	0,84520	-1,03	-2,04	0,15947	---	---	
7925523	-0,07	-1,05	0,84520	-1,03	-2,04	0,15947	---	---	
8132690	-0,07	-1,05	0,84520	-1,03	-2,04	0,15947	---	---	
8047401	-0,07	-1,05	0,86758	3,20	9,16	0,00034	---	---	
7964640	-0,07	-1,05	0,87294	-0,51	-1,42	0,02596	---	C12orf64	
7957351	-0,07	-1,05	0,74817	-0,72	-1,65	0,05761	NM_173591		C14orf138
7978923	-0,07	-1,05	0,81118	2,84	7,16	0,00146	NM_024558		---
7929114	-0,07	-1,05	0,86290	0,05	1,04	0,65442	---	---	
8101699	-0,07	-1,05	0,92592	-1,27	-2,41	0,02574	---	HSPB3	
8105323	-0,07	-1,05	0,78824	-1,48	-2,79	0,04318	NM_006308		WFDC6
8066536	-0,07	-1,05	0,87782	-1,32	-2,49	0,01577	NM_080827		LHFPL5
8118995	-0,07	-1,05	0,80100	-0,12	-1,08	0,73890	NM_182548		SORCS2
8093997	-0,07	-1,05	0,89794	-0,78	-1,72	0,10412	NM_020777		---
7987926	-0,07	-1,05	0,77759	-0,87	-1,82	0,03256	---	PTAFR	
7914184	-0,07	-1,05	0,90780	-0,95	-1,93	0,02971	NM_001164721		FLJ13197
8099817	-0,07	-1,05	0,83190	-0,59	-1,51	0,00223	NR_026804		---
7917528	-0,07	-1,05	0,71862	0,10	1,07	0,51721	---	LOC100130193	
7898568	-0,07	-1,05	0,85376	-0,77	-1,71	0,00169	AK096102	FLJ33996	
7955533	-0,07	-1,05	0,86428	1,16	2,24	0,01439	ENST00000423909		SPINK9
8109057	-0,07	-1,05	0,69960	-0,33	-1,25	0,02617	NM_001040433		P2RX7
7959251	-0,07	-1,05	0,91599	-0,99	-1,99	0,01565	NM_002562		SH3YL1
8049969	-0,07	-1,05	0,76939	1,18	2,26	0,01521	NM_015677		CYP39A1
8126770	-0,07	-1,05	0,87748	0,42	1,34	0,40723	NM_016593		---

8104727	-0,07	-1,05	0,86293	0,42	1,33	0,07240	---	RNF180
8105585	-0,07	-1,05	0,84221	-1,13	-2,19	0,03390	NM_001113561	PARS2
7916422	-0,07	-1,05	0,90162	1,31	2,48	0,00255	NM_152268	SNORD78
7922408	-0,07	-1,05	0,32940	1,58	3,00	0,00872	NR_003944	LOC100129312
8056810	-0,07	-1,05	0,69969	-1,51	-2,84	0,00017	AK095037	---
8051409	-0,07	-1,05	0,87463	-0,82	-1,76	0,00239	---	ADAMTS15
7945232	-0,07	-1,05	0,78492	1,49	2,81	0,00800	NM_139055	---
7991399	-0,07	-1,05	0,80009	-1,29	-2,44	0,00015	---	GNB5
7988859	-0,07	-1,05	0,54029	0,74	1,67	0,02765	NM_006578	RNF212
8098856	-0,07	-1,05	0,76862	4,38	20,76	0,00002	NM_001131034	KIF15
8079237	-0,07	-1,05	0,86748	2,07	4,21	0,01494	NM_020242	LY6G5C
8178554	-0,07	-1,05	0,63510	0,18	1,13	0,47532	NM_025262	---
8052733	-0,07	-1,05	0,74667	-0,50	-1,42	0,39681	---	LOC440173
8162183	-0,07	-1,05	0,87676	-1,67	-3,19	0,06919	NR_027471	CMTM1
7996290	-0,07	-1,05	0,71518	0,68	1,60	0,00041	NM_052999	---
8102350	-0,07	-1,05	0,85819	0,34	1,26	0,02803	---	METTL2B
8135922	-0,07	-1,05	0,90340	0,21	1,15	0,51573	NM_018396	---
8136578	-0,07	-1,05	0,75676	-0,76	-1,69	0,03496	---	SEPW1
8029969	-0,08	-1,05	0,68894	0,34	1,26	0,20310	NM_003009	PPM1M
8080198	-0,08	-1,05	0,86897	0,32	1,25	0,33064	NM_144641	FLJ34521
7945228	-0,08	-1,05	0,85653	-0,34	-1,27	0,59526	ENST00000318117	APOL2
8075720	-0,08	-1,05	0,89003	-0,36	-1,28	0,19282	NM_030882	RPS4Y2
8176730	-0,08	-1,05	0,72479	-0,21	-1,15	0,01980	NM_001039567	CLCN6
7897774	-0,08	-1,05	0,78429	1,62	3,06	0,00264	NM_001286	LINS1
7991566	-0,08	-1,05	0,82425	0,08	1,06	0,70179	NM_018148	XRCC2
8144036	-0,08	-1,05	0,73175	1,26	2,39	0,07071	NM_005431	KIAA1383
7910589	-0,08	-1,05	0,74634	0,22	1,17	0,46464	NM_019090	PAR5
7981943	-0,08	-1,05	0,49143	0,23	1,17	0,35647	NR_022008	SP2
8008052	-0,08	-1,06	0,86144	1,31	2,47	0,00031	NM_003110	USP1
7901867	-0,08	-1,06	0,75611	1,33	2,51	0,00318	NM_003368	LPCAT4
7987230	-0,08	-1,06	0,89058	2,80	6,94	0,00145	NM_153613	PLD4
7977319	-0,08	-1,06	0,87653	-1,47	-2,78	0,00238	NM_138790	STAG3L3
8133610	-0,08	-1,06	0,91241	1,59	3,01	0,00380	NM_001013739	GPR50
8170476	-0,08	-1,06	0,89469	-0,54	-1,46	0,31061	NM_004224	PTH1R
8079433	-0,08	-1,06	0,87525	0,11	1,08	0,26332	NM_000316	CSPG4
7990545	-0,08	-1,06	0,78145	-0,11	-1,08	0,73091	NM_001897	---
8041957	-0,08	-1,06	0,77708	-0,54	-1,46	0,08206	---	KLHL31
8127177	-0,08	-1,06	0,71707	-0,53	-1,44	0,14979	NM_001003760	WNT8A
8108287	-0,08	-1,06	0,69526	-1,14	-2,20	0,00093	NM_058244	FAM100B
8010050	-0,08	-1,06	0,72353	0,59	1,51	0,26267	NM_182565	ST3GAL4
7945110	-0,08	-1,06	0,62834	0,18	1,13	0,41874	NM_006278	ZNF33B
7933139	-0,08	-1,06	0,77130	1,40	2,64	0,02683	NM_006955	SRRT
8134992	-0,08	-1,06	0,87696	0,99	1,99	0,10499	NM_015908	NOL7
8123985	-0,08	-1,06	0,76177	-0,55	-1,46	0,08737	NM_016167	PPEF1
8166314	-0,08	-1,06	0,65500	0,48	1,39	0,08775	NM_006240	---
7991665	-0,08	-1,06	0,87705	-1,42	-2,68	0,00107	---	FLJ20712
8132245	-0,08	-1,06	0,78561	-0,73	-1,66	0,00103	ENST00000311067	ITGB1BP2
8168291	-0,08	-1,06	0,66072	-0,62	-1,54	0,04096	NM_012278	SMPD4
8045301	-0,08	-1,06	0,85122	1,85	3,60	0,02570	NM_017751	---
8166264	-0,08	-1,06	0,87950	-1,77	-3,42	0,03886	---	VIP
8122865	-0,08	-1,06	0,74255	-0,66	-1,58	0,05105	NM_003381	TCF25
7998033	-0,08	-1,06	0,87527	0,73	1,66	0,16425	NM_014972	C3orf42
8077815	-0,08	-1,06	0,72323	-0,24	-1,18	0,34664	NR_026829	---
8175332	-0,08	-1,06	0,77420	-0,37	-1,30	0,06166	---	POLR3F
8061171	-0,08	-1,06	0,77665	1,16	2,24	0,02246	NM_006466	PIK3R2
8026954	-0,08	-1,06	0,80079	0,44	1,35	0,24854	NM_005027	CISH
8087739	-0,08	-1,06	0,73148	0,14	1,10	0,65766	NM_013324	ZBTB17
7912675	-0,08	-1,06	0,83145	0,06	1,04	0,89568	NM_003443	---
8154388	-0,08	-1,06	0,90293	0,46	1,38	0,37363	---	NINL
8065485	-0,08	-1,06	0,82421	-0,11	-1,08	0,22565	NM_025176	TBL2
8140070	-0,08	-1,06	0,52137	0,36	1,28	0,23098	NM_012453	ARSE
8171161	-0,08	-1,06	0,74829	-1,01	-2,02	0,04648	NM_000047	GLUD2
8169711	-0,08	-1,06	0,81947	-0,25	-1,19	0,15854	NM_012084	KRTAP5-3
7945652	-0,08	-1,06	0,92078	-0,41	-1,33	0,53608	NM_001012708	MYB
8122202	-0,08	-1,06	0,82500	0,29	1,23	0,33238	NM_001130173	WDR60
8137639	-0,08	-1,06	0,82718	1,22	2,33	0,03341	NM_018051	---
8159848	-0,08	-1,06	0,49534	-0,45	-1,37	0,14171	---	BIRC3
7943413	-0,08	-1,06	0,54924	0,43	1,35	0,03440	NM_001165	SNORD116-25
7981998	-0,08	-1,06	0,72094	-0,77	-1,70	0,07772	NR_003339	S100A7L2
7920249	-0,08	-1,06	0,63211	-1,01	-2,02	0,01804	NM_001045479	FAM74A3
8161337	-0,08	-1,06	0,82062	-1,36	-2,56	0,00747	ENST00000377629	WDR31
8163491	-0,08	-1,06	0,80796	0,65	1,57	0,00568	NM_001012361	FBXW4
7935882	-0,08	-1,06	0,78966	1,26	2,39	0,05331	NM_022039	FAM117A

8016590	-0,08	-1,06	0,87026	2,04	4,12	0,00024	NM_030802	CCDC18
7903049	-0,08	-1,06	0,54923	-0,38	-1,31	0,09618	NM_206886	---
8029829	-0,08	-1,06	0,78053	-0,49	-1,40	0,02705	---	---
7967454	-0,08	-1,06	0,62818	-1,38	-2,61	0,03086	---	ABTB1
8082380	-0,08	-1,06	0,75442	-0,55	-1,47	0,09030	NM_172027	KIF27
8162019	-0,09	-1,06	0,47459	0,28	1,22	0,41993	NM_017576	C13orf38
7970989	-0,09	-1,06	0,49470	0,71	1,64	0,09706	NM_001144981	ZNF193
8117655	-0,09	-1,06	0,86654	1,51	2,84	0,00113	NM_006299	LYPLAL1
7909801	-0,09	-1,06	0,75926	0,69	1,61	0,04489	NM_138794	RASGRP1
7987405	-0,09	-1,06	0,64577	0,79	1,73	0,02393	NM_005739	---
7923582	-0,09	-1,06	0,92283	-0,26	-1,20	0,45112	---	KDM4D
7943282	-0,09	-1,06	0,59544	0,51	1,43	0,01808	NM_018039	---
8121275	-0,09	-1,06	0,78391	0,52	1,44	0,21895	---	OR6C3
7955991	-0,09	-1,06	0,82384	-1,31	-2,48	0,01268	NM_054104	B3GALT1
8046078	-0,09	-1,06	0,67406	0,28	1,22	0,30162	NM_020981	SFTA2
8124855	-0,09	-1,06	0,88165	-1,20	-2,30	0,00194	NM_205854	MED18
7899448	-0,09	-1,06	0,82856	0,05	1,04	0,45255	NM_017638	CLEC4G
8033433	-0,09	-1,06	0,84850	-0,68	-1,60	0,05600	NM_198492	ZNF483
8157193	-0,09	-1,06	0,76971	0,63	1,55	0,19375	NM_133464	RIN3
7976292	-0,09	-1,06	0,86621	0,09	1,07	0,72187	NM_024832	TMCC3
7965510	-0,09	-1,06	0,85288	1,13	2,19	0,05662	NM_020698	SAMD4B
8028624	-0,09	-1,06	0,43963	2,29	4,91	0,00156	NM_018028	SLC4A3
8048673	-0,09	-1,06	0,73441	-0,47	-1,39	0,41008	NM_201574	BCDIN3D
7963139	-0,09	-1,06	0,78271	1,61	3,05	0,00692	NM_181708	NFKB2
7930074	-0,09	-1,06	0,66810	2,38	5,22	0,00460	NM_002502	DSC3
8022692	-0,09	-1,06	0,74557	-0,13	-1,09	0,58448	NM_024423	PCDHGC5
8108757	-0,09	-1,06	0,48802	-0,60	-1,51	0,00253	NM_018929	---
8158684	-0,09	-1,06	0,86110	-0,18	-1,13	0,64296	---	ZNF154
8039674	-0,09	-1,06	0,89697	-0,97	-1,97	0,04964	NM_001085384	LOC442075
8077728	-0,09	-1,06	0,80747	1,33	2,52	0,00802	AK092352	ATRIP
8079637	-0,09	-1,06	0,63442	0,75	1,68	0,02103	NM_130384	ZNF704
8151496	-0,09	-1,06	0,78920	2,13	4,38	0,00831	NM_001033723	FTSJD1
8002523	-0,09	-1,06	0,72962	0,97	1,96	0,00120	NM_018348	MLST8
7992552	-0,09	-1,06	0,77742	-0,30	-1,23	0,42788	NM_022372	RBMY1A1
8177277	-0,09	-1,06	0,88910	-1,48	-2,79	0,02631	NM_005058	C19orf55
8028016	-0,09	-1,06	0,87386	1,22	2,33	0,01506	NM_001039887	ERICH1
8148985	-0,09	-1,06	0,70336	0,71	1,64	0,11459	NM_207332	RGS12
8093764	-0,09	-1,06	0,76446	-0,46	-1,38	0,05337	NM_198229	OSBPL6
8046646	-0,09	-1,06	0,15195	2,12	4,33	0,00118	NM_032523	---
7932909	-0,09	-1,07	0,49304	-0,52	-1,43	0,04821	---	---
7973752	-0,09	-1,07	0,36141	-0,72	-1,65	0,07349	---	MDM1
7964810	-0,09	-1,07	0,84743	0,14	1,10	0,65614	NM_017440	ATXN7L3B
7957242	-0,09	-1,07	0,57323	1,62	3,08	0,02268	NM_001136262	VEGFB
7940904	-0,09	-1,07	0,86878	1,82	3,54	0,01168	NM_003377	FLJ37035
7936925	-0,09	-1,07	0,80639	0,56	1,48	0,19633	AK094354	MAGEC2
8175562	-0,09	-1,07	0,85221	-0,41	-1,33	0,08531	NM_016249	LOC202181
8116128	-0,09	-1,07	0,68882	-0,48	-1,40	0,14269	NR_026921	TRIM49
7950955	-0,09	-1,07	0,90769	-2,11	-4,31	0,00009	NM_020358	RBM17
7926021	-0,09	-1,07	0,33273	1,40	2,63	0,00154	NM_001145547	GEMIN6
8041553	-0,09	-1,07	0,75411	0,46	1,37	0,00381	NM_024775	---
8101814	-0,09	-1,07	0,43407	-0,29	-1,22	0,11801	---	DOCK8
8153959	-0,09	-1,07	0,71116	1,05	2,07	0,00009	NM_203447	TMEM104
8009693	-0,09	-1,07	0,76703	1,01	2,01	0,10867	NM_017728	DLG4
8012054	-0,09	-1,07	0,84440	1,43	2,70	0,02850	NM_001365	CLEC1B
7961102	-0,09	-1,07	0,84949	-0,97	-1,96	0,00116	NM_016509	---
7952735	-0,09	-1,07	0,58152	-1,03	-2,04	0,13167	---	---
8165700	-0,09	-1,07	0,93086	-4,93	-30,45	0,00109	---	PRMT10
8103079	-0,09	-1,07	0,61740	0,86	1,82	0,09932	NM_138364	---
8177092	-0,09	-1,07	0,82637	-0,48	-1,40	0,03763	---	DGCR14
8074364	-0,09	-1,07	0,82163	-0,15	-1,11	0,67474	NM_022719	OPA3
8037608	-0,09	-1,07	0,67100	0,19	1,14	0,67032	NM_001017989	MGLL
8090433	-0,09	-1,07	0,76872	1,57	2,97	0,01416	NM_007283	ATP9B
8021832	-0,09	-1,07	0,82249	0,83	1,77	0,09959	NM_198531	---
8157036	-0,09	-1,07	0,72942	-0,89	-1,86	0,00594	---	NSUN6
7932433	-0,09	-1,07	0,81234	1,19	2,28	0,03918	NM_182543	FSD1L
8157058	-0,09	-1,07	0,62130	-0,08	-1,06	0,71712	NM_207647	CCDC134
8073513	-0,10	-1,07	0,54047	1,49	2,81	0,01570	NM_024821	ZNF461
8036341	-0,10	-1,07	0,75348	0,52	1,44	0,11131	NM_153257	LOC100129488
7993754	-0,10	-1,07	0,68029	-0,77	-1,71	0,06005	ENST00000438973	C3orf33
8091629	-0,10	-1,07	0,67450	0,96	1,94	0,05526	AK289890	---
7898854	-0,10	-1,07	0,72206	-0,63	-1,55	0,00034	---	C7orf31
8138647	-0,10	-1,07	0,61661	1,41	2,66	0,02627	NM_138811	DPH3B
8064003	-0,10	-1,07	0,75987	-0,07	-1,05	0,81825	NM_080750	AMBP

8163535	-0,10	-1,07	0,58104	-0,42	-1,34	0,05792	NM_001633	CREBZF
7950796	-0,10	-1,07	0,64061	0,32	1,25	0,38875	NM_001039618	DIAPH3
7971866	-0,10	-1,07	0,78753	1,09	2,12	0,00518	NM_001042517	LIPG
8021169	-0,10	-1,07	0,77739	-0,17	-1,13	0,59541	NM_006033	---
8053311	-0,10	-1,07	0,88591	-0,72	-1,65	0,02332	---	RASA3
7972946	-0,10	-1,07	0,83464	0,35	1,27	0,59087	NM_007368	---
8022434	-0,10	-1,07	0,91962	-2,58	-5,97	0,00059	---	C9orf7
8159078	-0,10	-1,07	0,85743	0,30	1,23	0,62978	NM_017586	LOC391358
8050906	-0,10	-1,07	0,82120	-0,61	-1,53	0,00558	XM_372921	---
8072272	-0,10	-1,07	0,90176	-0,78	-1,72	0,00945	---	---
8138862	-0,10	-1,07	0,71722	-0,05	-1,04	0,89504	---	MXRA7
8018786	-0,10	-1,07	0,71947	0,31	1,24	0,51998	NM_001008528	PRICKLE4
8119473	-0,10	-1,07	0,78967	-0,09	-1,06	0,76471	NM_013397	---
8161863	-0,10	-1,07	0,83129	-0,41	-1,33	0,42545	---	UPF1
8027074	-0,10	-1,07	0,80467	1,82	3,52	0,02026	NM_002911	C2
8178095	-0,10	-1,07	0,74734	-0,23	-1,17	0,26135	NM_000063	C2
8179331	-0,10	-1,07	0,74734	-0,23	-1,17	0,26135	NM_000063	SLC7A8
7977933	-0,10	-1,07	0,78305	-0,85	-1,80	0,00160	NM_012244	TJAP1
8119829	-0,10	-1,07	0,44238	0,85	1,81	0,08246	NM_001146018	MIF
8071737	-0,10	-1,07	0,39121	-0,50	-1,42	0,13889	NM_002415	FAM123B
8173232	-0,10	-1,07	0,72117	-0,22	-1,16	0,57316	NM_152424	PDK3
8166511	-0,10	-1,07	0,74766	1,75	3,36	0,00110	NM_005391	LY6G5C
8125007	-0,10	-1,07	0,46352	0,10	1,07	0,64369	NM_025262	---
8167428	-0,10	-1,07	0,69207	-0,75	-1,68	0,04493	---	ZNF425
8143702	-0,10	-1,07	0,73982	0,01	1,01	0,97324	NM_001001661	---
8143385	-0,10	-1,07	0,76055	-0,55	-1,47	0,09573	---	HTR3D
8084266	-0,10	-1,07	0,83741	-1,21	-2,31	0,09186	NM_182537	LOC91316
8074927	-0,10	-1,07	0,70866	1,39	2,61	0,03696	NR_024448	MIR220A
8174891	-0,10	-1,07	0,86319	-1,25	-2,38	0,00547	NR_029634	LOXL3
8053231	-0,10	-1,07	0,64820	-0,21	-1,16	0,46443	NM_032603	BIK
8073605	-0,10	-1,07	0,84308	-0,12	-1,09	0,66605	NM_001197	CIDECP
8085276	-0,10	-1,07	0,76300	0,82	1,76	0,04199	NR_002786	DLG3
8168179	-0,10	-1,07	0,68684	0,97	1,95	0,00427	NM_021120	CTAGE5
7974090	-0,10	-1,07	0,67398	0,97	1,96	0,00912	NM_005930	LRRK1
7986463	-0,10	-1,07	0,78715	1,44	2,72	0,01207	NM_024652	---
8072139	-0,10	-1,07	0,73199	-0,96	-1,95	0,02948	---	ACTR1B
8054077	-0,10	-1,07	0,77356	1,58	3,00	0,03494	NM_005735	CABC1
7910164	-0,10	-1,07	0,68411	0,76	1,69	0,16827	NM_020247	FFAR1
8027854	-0,10	-1,07	0,82452	-0,55	-1,46	0,34363	NM_005303	---
8135361	-0,10	-1,07	0,52412	-1,19	-2,27	0,00289	---	LPAL2
8130598	-0,10	-1,07	0,85866	-0,18	-1,13	0,87787	NR_028093	FBXO48
8052742	-0,10	-1,07	0,73472	-0,03	-1,02	0,85480	NM_001024680	RING1
8118634	-0,10	-1,07	0,84788	1,16	2,24	0,03236	NM_002931	---
8083445	-0,10	-1,07	0,88801	-0,86	-1,81	0,12992	---	SGEF
8083471	-0,10	-1,07	0,50903	3,10	8,55	0,00023	NM_015595	---
8055946	-0,10	-1,07	0,71948	-1,06	-2,08	0,06541	---	AAAS
7963646	-0,10	-1,07	0,83234	2,25	4,76	0,00646	NM_015665	EHD1
7949264	-0,10	-1,07	0,69176	0,52	1,43	0,14661	NM_006795	CLEC12B
7953914	-0,10	-1,07	0,75957	-0,88	-1,84	0,04702	NM_205852	IL10RA
7944152	-0,10	-1,07	0,64444	-0,26	-1,20	0,50348	NM_001558	TRY6
8136801	-0,10	-1,08	0,44746	-0,11	-1,08	0,65816	NR_001296	MBLAC2
8113059	-0,10	-1,08	0,78543	0,01	1,01	0,95239	NM_203406	SLC38A7
8001750	-0,10	-1,08	0,64361	1,07	2,10	0,02181	NM_018231	C8orf34
8146839	-0,10	-1,08	0,49179	-0,78	-1,72	0,00431	BC041961	PPFIBP2
7938231	-0,11	-1,08	0,68265	-0,09	-1,07	0,44777	NM_003621	NQO2
8123637	-0,11	-1,08	0,80657	0,03	1,02	0,94175	NM_000904	ADPRH
8081880	-0,11	-1,08	0,68738	-0,24	-1,18	0,54569	NM_001125	SHISA3
8094870	-0,11	-1,08	0,80165	-1,14	-2,20	0,12775	NM_001080505	NCDN
7899990	-0,11	-1,08	0,83992	2,55	5,86	0,00510	NM_014284	PI4K2B
8094378	-0,11	-1,08	0,61802	1,03	2,05	0,01946	NM_018323	GAL3ST2
8049934	-0,11	-1,08	0,65583	-0,57	-1,49	0,01745	NM_022134	---
8081501	-0,11	-1,08	0,54514	-0,54	-1,45	0,00861	---	TCHP
7958582	-0,11	-1,08	0,66715	0,27	1,20	0,31730	NM_032300	RBM14
7941702	-0,11	-1,08	0,34535	-0,20	-1,15	0,49359	NM_006328	VPS37B
7967331	-0,11	-1,08	0,81089	0,67	1,59	0,06601	NM_024667	PCDHB13
8108737	-0,11	-1,08	0,80353	0,42	1,33	0,04788	NM_018933	CDC42EP2
7941236	-0,11	-1,08	0,68748	-0,55	-1,46	0,37927	NM_006779	---
8096533	-0,11	-1,08	0,73168	-0,05	-1,03	0,74548	---	DENND2C
7918757	-0,11	-1,08	0,67682	-0,27	-1,20	0,42971	BC063894	AGXT2L2
8116177	-0,11	-1,08	0,57296	1,93	3,81	0,00008	NM_153373	HDAC8
8173531	-0,11	-1,08	0,69080	1,98	3,94	0,00043	NM_018486	CMTM8
8078397	-0,11	-1,08	0,51063	1,55	2,93	0,00351	NM_178868	---
7938368	-0,11	-1,08	0,90142	-0,60	-1,52	0,09882	---	FEZF2

8088485	-0,11	-1,08	0,53403	-1,33	-2,51	0,00460	NM_018008	C14orf33
7979351	-0,11	-1,08	0,73507	2,80	6,98	0,00622	NR_027123	LRRC8A
8158424	-0,11	-1,08	0,14585	2,61	6,09	0,00572	NM_001127244	KRT6B
7963406	-0,11	-1,08	0,82714	-1,20	-2,29	0,03545	NM_005555	N6AMT1
8069700	-0,11	-1,08	0,65725	-0,17	-1,13	0,66930	NM_013240	ZNF672
7911289	-0,11	-1,08	0,75704	0,89	1,85	0,01331	NM_024836	TCN1
7948444	-0,11	-1,08	0,42534	-0,86	-1,81	0,01711	NM_001062	SLFN12
8014241	-0,11	-1,08	0,53247	0,51	1,43	0,33601	NM_018042	PITPNM1
7949808	-0,11	-1,08	0,80330	0,98	1,97	0,20195	NM_004910	DLK2
8126556	-0,11	-1,08	0,78046	0,47	1,38	0,40161	NM_023932	PMS2CL
8131444	-0,11	-1,08	0,87822	-0,92	-1,89	0,11105	NR_002217	KIR2DL4
8039859	-0,11	-1,08	0,85100	-1,26	-2,40	0,00030	NM_002255	---
8126705	-0,11	-1,08	0,70752	-0,83	-1,77	0,00020	--- ---	
8025893	-0,11	-1,08	0,82558	-1,09	-2,12	0,00675	--- JUB	
7977854	-0,11	-1,08	0,62670	1,82	3,54	0,00465	NM_032876	CCL11
8006445	-0,11	-1,08	0,66120	-1,02	-2,02	0,12589	NM_002986	ANAPC1
8043322	-0,11	-1,08	0,75458	1,42	2,68	0,01580	NM_022662	UNG
7958455	-0,11	-1,08	0,66865	2,64	6,22	0,00124	NM_003362	SNORA54
7945801	-0,11	-1,08	0,49303	-1,18	-2,27	0,03333	NR_002982	---
8143594	-0,11	-1,08	0,79140	-0,78	-1,72	0,03758	--- FER1L6	
8152742	-0,11	-1,08	0,68787	-0,91	-1,88	0,00646	ENST00000399018	---
7950062	-0,11	-1,08	0,83907	-1,15	-2,22	0,03776	--- USP54	
7934411	-0,11	-1,08	0,60864	2,01	4,02	0,00988	NM_152586	ARID3B
7902592	-0,11	-1,08	0,64110	1,48	2,80	0,01072	NM_006465	C21orf94
8067836	-0,11	-1,08	0,87231	-0,01	-1,01	0,98099	NR_027246	RSPH4A
8121622	-0,11	-1,08	0,48869	-0,73	-1,65	0,00494	NM_001010892	PPP1R15A
8030128	-0,11	-1,08	0,49183	1,52	2,87	0,01088	NM_014330	---
8136589	-0,11	-1,08	0,78113	-0,35	-1,27	0,05866	--- ---	
7950442	-0,11	-1,08	0,46122	-0,80	-1,74	0,00832	--- B3GNTL1	
8019559	-0,11	-1,08	0,79895	-0,85	-1,80	0,04338	AK126018 ---	
7983771	-0,11	-1,08	0,75230	-2,01	-4,03	0,01960	--- MGAM	
8136662	-0,11	-1,08	0,63084	-0,78	-1,72	0,00429	NM_004668	---
8166659	-0,11	-1,08	0,61037	-0,08	-1,06	0,85012	--- HIST2H2AA3	
7905079	-0,11	-1,08	0,73296	0,48	1,39	0,38269	NM_003516	HIST2H2AA3
7919619	-0,11	-1,08	0,73296	0,48	1,39	0,38269	NM_003516	RNF5
8118535	-0,11	-1,08	0,74918	1,11	2,15	0,00661	NM_006913	RNF5
8179472	-0,11	-1,08	0,74918	1,11	2,15	0,00661	NM_006913	LOC100129566
8106352	-0,11	-1,08	0,80956	-0,59	-1,50	0,06181	XM_001722314	RCCD1
7986186	-0,11	-1,08	0,53884	1,58	2,99	0,01276	NM_033544	ZFYVE19
7982812	-0,12	-1,08	0,11772	2,27	4,84	0,00066	NM_001077268	TM4SF20
8059525	-0,12	-1,08	0,71176	-0,12	-1,09	0,39738	NM_024795	ZNF323
8124562	-0,12	-1,08	0,68459	-0,74	-1,67	0,08461	NM_001135215	STAG3L4
8133215	-0,12	-1,08	0,66852	2,49	5,61	0,00107	NM_022906	GRIPAP1
8172504	-0,12	-1,08	0,76597	0,65	1,57	0,07275	NM_020137	C1RL
7960757	-0,12	-1,08	0,83756	1,86	3,64	0,00692	NM_016546	OLAH
7926334	-0,12	-1,08	0,50773	-0,68	-1,60	0,02691	NM_018324	RBM34
7925184	-0,12	-1,08	0,79648	0,89	1,85	0,02673	NM_015014	ROGDI
7999217	-0,12	-1,08	0,73084	0,92	1,90	0,01475	NM_024589	---
7994570	-0,12	-1,08	0,89906	-2,63	-6,19	0,00023	--- THTPA	
7973427	-0,12	-1,08	0,70461	0,28	1,22	0,53550	NM_024328	SLC36A3
8115274	-0,12	-1,08	0,80390	-0,59	-1,50	0,02374	NM_001145017	HIST2H2AC
7905088	-0,12	-1,08	0,72371	-0,39	-1,31	0,02652	NM_003517	ELMO1
8139057	-0,12	-1,08	0,72800	-1,19	-2,28	0,05383	NM_014800	RNF8
8119184	-0,12	-1,08	0,75203	0,19	1,14	0,35733	NM_003958	SFTA2
8179709	-0,12	-1,08	0,84769	-1,27	-2,41	0,00062	NM_205854	FTHL3
8051133	-0,12	-1,08	0,84915	-0,17	-1,13	0,53742	NR_002201	PAQR3
8101237	-0,12	-1,08	0,73046	1,23	2,35	0,01119	NM_001040202	---
7931029	-0,12	-1,08	0,78981	-1,06	-2,08	0,00021	--- MIR15B	
8083737	-0,12	-1,08	0,60819	-0,80	-1,74	0,01132	NR_029663	PPAPDC1A
7930980	-0,12	-1,08	0,64567	-0,30	-1,23	0,20507	NM_001030059	CCDC34
7947189	-0,12	-1,08	0,66613	0,41	1,33	0,22553	NM_030771	CNTNAP3
8155460	-0,12	-1,09	0,62618	0,15	1,11	0,34356	NM_033655	LOC152217
8084982	-0,12	-1,09	0,62302	1,28	2,43	0,03887	NR_024388	LOC728606
8022653	-0,12	-1,09	0,48338	-0,57	-1,49	0,02850	NR_024259	PLEKHA4
8038225	-0,12	-1,09	0,66614	-1,28	-2,42	0,01946	NM_020904	RNF44
8115927	-0,12	-1,09	0,64426	2,02	4,06	0,00329	NM_014901	SETD8
7972973	-0,12	-1,09	0,83581	-0,46	-1,37	0,29306	AF287261 C1orf61	
7921003	-0,12	-1,09	0,84037	-0,57	-1,49	0,00585	NM_006365	C14orf93
7977868	-0,12	-1,09	0,55441	-0,72	-1,64	0,05443	NM_021944	C20orf191
8005733	-0,12	-1,09	0,88109	0,11	1,08	0,87500	NR_003678	FAM46C
7904361	-0,12	-1,09	0,83402	0,27	1,21	0,32524	NM_017709	ULBP2
8122720	-0,12	-1,09	0,90162	0,29	1,22	0,44825	NM_025217	MAP1S
8026821	-0,12	-1,09	0,47532	0,55	1,46	0,46391	NM_018174	EFNB1

8168045	-0,12	-1,09	0,66026	0,59	1,50	0,03991	NM_004429	DNA2
7934026	-0,12	-1,09	0,66323	1,80	3,48	0,01014	NM_001080449	---
7917180	-0,12	-1,09	0,89797	-0,47	-1,38	0,36017	---	KDM1B
8117081	-0,12	-1,09	0,63333	1,59	3,01	0,00056	NM_153042	ZNF560
8033767	-0,12	-1,09	0,53306	3,32	9,97	0,00011	NM_152476	LRRN1
8077366	-0,12	-1,09	0,58452	-0,62	-1,54	0,13053	NM_020873	DNAJC9
7934320	-0,12	-1,09	0,76418	-0,06	-1,04	0,90783	NM_015190	---
8089013	-0,12	-1,09	0,61290	0,30	1,23	0,20997	---	STAC
8078529	-0,12	-1,09	0,71477	-1,05	-2,07	0,00039	NM_003149	TTC5
7977482	-0,12	-1,09	0,23120	1,16	2,23	0,00295	NM_138376	B4GALT7
8110399	-0,12	-1,09	0,63019	-0,32	-1,25	0,23252	NM_007255	ARID3A
8024078	-0,12	-1,09	0,65538	-0,04	-1,02	0,93346	NM_005224	ASAP3
7913609	-0,12	-1,09	0,70315	-0,21	-1,16	0,57639	NM_017707	C14orf128
7978538	-0,12	-1,09	0,73869	2,12	4,35	0,00170	NR_027263	ZNF384
7953390	-0,12	-1,09	0,70932	-0,30	-1,23	0,48676	NM_001039916	PLEKHH1
7975238	-0,12	-1,09	0,59474	1,56	2,95	0,00314	NM_020715	PICK1
8072989	-0,12	-1,09	0,75864	2,31	4,96	0,00011	NM_012407	---
8003771	-0,12	-1,09	0,75959	-0,90	-1,86	0,00152	---	ULK3
7990400	-0,12	-1,09	0,81664	1,52	2,87	0,02883	NM_001099436	C1orf228
7900999	-0,12	-1,09	0,70785	0,25	1,19	0,37960	NM_001145636	B3GNT1
7949615	-0,12	-1,09	0,75765	1,31	2,47	0,16147	NM_006876	HSP90B3P
7902930	-0,12	-1,09	0,66388	-0,19	-1,14	0,74136	NR_003130	01. Mrz
8103508	-0,12	-1,09	0,72143	-0,80	-1,74	0,00917	NM_017923	GPS2
8012150	-0,12	-1,09	0,55290	-0,30	-1,23	0,51374	NM_004489	C19orf18
8039713	-0,12	-1,09	0,62911	-0,44	-1,35	0,16817	BC033933	SYTL4
8173955	-0,12	-1,09	0,28430	0,41	1,33	0,01687	NM_080737	PGLS
8026780	-0,12	-1,09	0,59713	-0,51	-1,43	0,09771	NM_012088	ARAP3
8114733	-0,13	-1,09	0,27471	0,00	1,00	0,99293	NM_022481	IMPG2
8089185	-0,13	-1,09	0,73551	-1,31	-2,47	0,03418	NM_016247	RACGAP1P // RACGAP1P // RACGAP1P // RACGAP1P // RACGAP1P // RACGAP1P // RACGAP1P // RACGAP1P // RACGAP1P
7962487	-0,13	-1,09	0,23412	1,01	2,01	0,04697	NR_026583 // NR_026583 // NR_026583 // NR_026583 // NR_026583 // NR_026583 // NR_026583 // NR_026583 // NR_026583	NDUFB6
8160587	-0,13	-1,09	0,79473	0,51	1,42	0,34525	NM_002493	TMX4
8064939	-0,13	-1,09	0,78034	0,48	1,39	0,27122	NM_021156	HMOX1
8072678	-0,13	-1,09	0,84514	0,03	1,02	0,92526	NM_002133	BAD
7949067	-0,13	-1,09	0,59835	0,27	1,20	0,59886	NM_004322	CDC45
8071212	-0,13	-1,09	0,75888	2,71	6,56	0,00514	NM_001178010	C7orf11
8139203	-0,13	-1,09	0,45542	0,34	1,27	0,32317	NM_138701	LCP1
7971461	-0,13	-1,09	0,72458	-0,88	-1,85	0,14289	NM_002298	---
7937913	-0,13	-1,09	0,80587	-1,40	-2,64	0,05070	---	OR5E1P
7938261	-0,13	-1,09	0,73345	-1,29	-2,45	0,00024	NR_027711	DUSP5
7930413	-0,13	-1,09	0,60919	2,16	4,46	0,06785	NM_004419	MCEE
8052934	-0,13	-1,09	0,75915	-0,33	-1,26	0,01016	NM_032601	---
7970563	-0,13	-1,09	0,22002	-0,29	-1,22	0,04805	---	RRAD
8001918	-0,13	-1,09	0,35032	-0,86	-1,81	0,00518	NM_001128850	SNX11
8008096	-0,13	-1,09	0,74127	0,49	1,40	0,26552	NM_152244	---
8050873	-0,13	-1,09	0,74042	-1,68	-3,21	0,00234	---	RNASE3
7973105	-0,13	-1,09	0,74838	-1,38	-2,60	0,06164	NM_002935	FLJ10661
8144498	-0,13	-1,09	0,74982	-0,41	-1,33	0,56921	NR_024362	POLR1A
8053496	-0,13	-1,09	0,71007	1,62	3,08	0,00565	NM_015425	SVOP
7966172	-0,13	-1,09	0,65190	-1,18	-2,26	0,02868	NM_018711	LRRC34
8092009	-0,13	-1,09	0,27736	1,28	2,42	0,07019	NM_001172779	TEAD4
7953150	-0,13	-1,09	0,44887	1,40	2,63	0,02736	NM_003213	---
8057931	-0,13	-1,09	0,60674	-0,57	-1,48	0,02888	---	C11orf31
7940070	-0,13	-1,09	0,74496	-1,06	-2,08	0,01109	NM_170746	SHPK
8011396	-0,13	-1,09	0,73261	0,99	1,99	0,02593	NM_013276	OR5P3
7946326	-0,13	-1,09	0,60741	-1,40	-2,65	0,04991	NM_153445	VPS33B
7991427	-0,13	-1,09	0,80529	1,72	3,30	0,00536	NM_018668	FMNL3
7963092	-0,13	-1,09	0,68665	-0,02	-1,01	0,93435	NM_175736	CFHR2
7908496	-0,13	-1,09	0,80040	-0,79	-1,73	0,14766	NM_005666	OR6S1
7977609	-0,13	-1,09	0,61310	-1,08	-2,12	0,00208	NM_001001968	LPHN3
8095303	-0,13	-1,09	0,22947	-1,06	-2,09	0,05395	NM_015236	MAN1B1
8159566	-0,13	-1,10	0,59481	-0,02	-1,01	0,97546	NM_016219	GOLGA6L1
7986741	-0,13	-1,10	0,87566	-1,51	-2,85	0,03605	NM_001001413	ZNF17
8031744	-0,13	-1,10	0,03482	0,20	1,15	0,34067	NM_006959	GAGE12C
8167560	-0,13	-1,10	0,93789	-3,79	-13,79	0,04517	NM_001098408	AKAP7
8122045	-0,13	-1,10	0,67899	-0,03	-1,02	0,86318	NM_016377	---
8061645	-0,13	-1,10	0,41271	-1,64	-3,12	0,00682	---	LOC100132147
7898407	-0,13	-1,10	0,84867	-1,21	-2,32	0,08563	BC036435	---
8074640	-0,13	-1,10	0,58032	-1,13	-2,19	0,00552	---	PKNOX2
7944955	-0,13	-1,10	0,72596	-1,27	-2,41	0,00221	NM_022062	KIAA1958
8157246	-0,13	-1,10	0,75767	2,42	5,35	0,00121	BC105048	GIMAP5
8137257	-0,13	-1,10	0,63494	-0,73	-1,66	0,01020	NM_018384	ZDHHC8

8071339	-0,13	-1,10	0,48928	1,19	2,28	0,06918	NM_013373	PAQR7
7913858	-0,13	-1,10	0,75532	-0,39	-1,31	0,24981	NM_178422	---
8068549	-0,13	-1,10	0,48425	-0,90	-1,87	0,12158	---	ARL6IP4
7959549	-0,13	-1,10	0,82784	0,63	1,54	0,09404	NM_001002252	---
8088915	-0,13	-1,10	0,74708	-0,38	-1,30	0,38437	---	---
8112892	-0,13	-1,10	0,80105	0,04	1,03	0,91324	---	TIGD2
8096411	-0,13	-1,10	0,76736	0,34	1,27	0,22331	NM_145715	FHIT
8088458	-0,13	-1,10	0,74270	1,19	2,29	0,07469	NM_002012	UBE2CBP
8127824	-0,13	-1,10	0,72395	0,79	1,73	0,00175	NM_198920	OTUD7A
7982294	-0,13	-1,10	0,75310	-0,32	-1,24	0,32072	NM_130901	HEXIM2
8007750	-0,13	-1,10	0,57534	1,19	2,28	0,00856	NM_144608	CATSPER3
8108182	-0,13	-1,10	0,53475	-0,21	-1,16	0,51015	NM_178019	PYCRL
8153459	-0,13	-1,10	0,74592	1,09	2,12	0,03293	NM_023078	ZNF7
8148941	-0,13	-1,10	0,48854	1,06	2,08	0,00677	NM_003416	TBC1D3P2
8017173	-0,13	-1,10	0,83496	0,69	1,62	0,24787	NR_027486	DBN1
8116051	-0,13	-1,10	0,45392	0,79	1,73	0,16992	NM_080881	ZBTB45
8039791	-0,13	-1,10	0,47104	0,63	1,55	0,04932	NM_032792	AGAP5
7927353	-0,13	-1,10	0,81825	2,45	5,46	0,00003	NM_001144000	AGAP5
7933331	-0,13	-1,10	0,81825	2,45	5,46	0,00003	NM_001144000	ADCY6
7962842	-0,13	-1,10	0,59134	1,27	2,41	0,07554	NM_015270	HELLS
7929438	-0,13	-1,10	0,54427	1,99	3,96	0,00008	NM_018063	KEAP1
8034034	-0,13	-1,10	0,46426	-0,25	-1,19	0,52957	NM_203500	PSMD9
7959322	-0,14	-1,10	0,68952	0,80	1,74	0,19955	NM_002813	FUNDC1
8172244	-0,14	-1,10	0,72094	0,32	1,24	0,26637	NM_173794	IFT172
8051135	-0,14	-1,10	0,36806	0,08	1,06	0,38272	NM_015662	STAG3L2
8140196	-0,14	-1,10	0,84467	1,39	2,63	0,01429	NM_001025202	---
8056794	-0,14	-1,10	0,50391	-0,68	-1,60	0,01966	---	CP
8091385	-0,14	-1,10	0,75105	0,62	1,54	0,15417	NM_000096	KAZ
7898084	-0,14	-1,10	0,50407	0,04	1,03	0,91226	NM_015209	PRPH2
8126436	-0,14	-1,10	0,46597	-1,30	-2,46	0,00910	NM_000322	ZNF606
8039719	-0,14	-1,10	0,29111	-0,32	-1,24	0,03784	NM_025027	LOC100127951
7997166	-0,14	-1,10	0,46493	-0,65	-1,56	0,02068	AY358233	NUDT9
8096251	-0,14	-1,10	0,56996	0,48	1,40	0,02153	NM_024047	SIP1
7974054	-0,14	-1,10	0,77805	0,68	1,60	0,13209	NM_003616	SUFU
7930120	-0,14	-1,10	0,63010	0,70	1,62	0,00443	NM_016169	OR4C45
7948039	-0,14	-1,10	0,68025	-1,25	-2,37	0,00095	NM_001005513	FASTKD1
8056693	-0,14	-1,10	0,17228	1,76	3,38	0,00200	NM_024622	LOC387790
7942647	-0,14	-1,10	0,86718	0,26	1,20	0,68678	ENST00000321844	SLC16A6
8017843	-0,14	-1,10	0,73318	0,88	1,85	0,04423	NM_001174166	---
8081452	-0,14	-1,10	0,56164	-0,77	-1,71	0,00256	---	HMGN5
8173812	-0,14	-1,10	0,54598	-0,60	-1,52	0,21516	NM_030763	---
7998835	-0,14	-1,10	0,67774	-1,26	-2,40	0,10935	---	MZF1
8039809	-0,14	-1,10	0,48208	0,33	1,26	0,59558	NM_198055	CFHR5
7908499	-0,14	-1,10	0,74839	-1,10	-2,15	0,03534	NM_030787	HSD17B3
8162570	-0,14	-1,10	0,20125	-0,17	-1,13	0,73239	NM_000197	SART1
7941478	-0,14	-1,10	0,40727	0,69	1,61	0,16084	NM_005146	PDXK
8068919	-0,14	-1,10	0,56658	1,19	2,29	0,04869	NM_003681	C14orf21
7973679	-0,14	-1,10	0,65336	-0,47	-1,38	0,52093	NM_174913	CXorf21
8171896	-0,14	-1,10	0,51492	-0,92	-1,89	0,01505	NM_025159	FLJ42393
8084739	-0,14	-1,10	0,71736	-1,69	-3,24	0,00694	NR_024413	PIGZ
8093166	-0,14	-1,10	0,50609	1,99	3,98	0,00135	NM_025163	EP400NL
7960054	-0,14	-1,10	0,59243	-0,55	-1,47	0,07613	NR_003290	L1CAM
8175871	-0,14	-1,10	0,48432	-0,78	-1,72	0,06432	NM_000425	MIR1247
7981326	-0,14	-1,10	0,63258	-1,75	-3,35	0,01505	NR_031649	URB2
7910416	-0,14	-1,10	0,74957	2,08	4,23	0,00002	NM_014777	---
7940110	-0,14	-1,10	0,62925	-0,98	-1,97	0,04941	---	---
8167198	-0,14	-1,10	0,59607	-0,19	-1,14	0,47599	---	C14orf43
7980051	-0,14	-1,10	0,50837	1,06	2,08	0,01798	NM_194278	GATSL3
8075322	-0,14	-1,10	0,24284	2,34	5,05	0,01018	NM_001037666	---
8035939	-0,14	-1,10	0,69286	-0,33	-1,26	0,09641	---	ARL5A
8055862	-0,14	-1,10	0,62780	0,40	1,32	0,31359	NM_012097	CDR2L
8009721	-0,14	-1,10	0,67899	0,75	1,68	0,19624	NM_014603	TRIM10
8178338	-0,14	-1,10	0,80003	-1,84	-3,58	0,00067	NM_006778	TTC30A
8056999	-0,14	-1,10	0,82709	0,36	1,29	0,31490	NM_152275	KLHDC8B
8079733	-0,14	-1,10	0,77340	0,67	1,59	0,42293	NM_173546	KIAA1328
8021001	-0,14	-1,10	0,43361	0,60	1,52	0,06617	NM_020776	GCFC1
8070046	-0,14	-1,10	0,69544	1,23	2,35	0,02926	NM_016631	---
8019958	-0,14	-1,10	0,24482	-0,99	-1,99	0,01779	---	AMY2B
7903407	-0,14	-1,10	0,75304	-0,47	-1,39	0,28590	NM_020978	---
7915329	-0,14	-1,10	0,67839	-1,12	-2,17	0,01008	---	NEUROD4
7955974	-0,14	-1,10	0,73917	-1,30	-2,46	0,01269	NM_021191	CTSS
7919800	-0,14	-1,10	0,61650	0,24	1,18	0,49220	NM_004079	ARHGAP6
8171313	-0,14	-1,10	0,61585	-1,69	-3,22	0,00179	NM_013427	PPP1R3A

8142435	-0,14	-1,10	0,53370	-0,88	-1,84	0,00316	NM_002711	HMMR
8109712	-0,14	-1,10	0,77067	1,35	2,54	0,01295	NM_001142556	OR56B1
7938049	-0,14	-1,10	0,58288	-1,11	-2,17	0,01058	NM_001005180	ADAMTS6
8112342	-0,14	-1,10	0,57482	-0,59	-1,51	0,03763	NM_197941	BSCL2
7948798	-0,14	-1,10	0,80297	0,08	1,06	0,75545	NM_001130702	C12orf51
7966488	-0,14	-1,10	0,67469	2,03	4,10	0,00029	NM_001109662	MAP3K3
8009183	-0,14	-1,10	0,64334	1,73	3,32	0,00508	NM_203351	NUDCD3
8139356	-0,14	-1,10	0,78772	0,99	1,99	0,03562	NM_015332	SERPIND1
8071420	-0,14	-1,10	0,53186	-1,54	-2,91	0,00823	NM_000185	MCHR1
8073296	-0,14	-1,10	0,66999	-0,80	-1,74	0,13384	NM_005297	---
8109424	-0,14	-1,10	0,36151	-0,60	-1,51	0,03991	---	OR52N1
7946107	-0,14	-1,10	0,83969	-1,45	-2,73	0,03734	NM_001001913	SLC9A4
8044067	-0,14	-1,10	0,50098	-0,66	-1,58	0,00856	NM_001011552	---
8067901	-0,14	-1,10	0,61028	-0,50	-1,41	0,28411	---	---
8149069	-0,14	-1,11	0,25000	-0,36	-1,28	0,01923	---	---
8133623	-0,14	-1,11	0,79762	-0,91	-1,87	0,02136	---	---
8139975	-0,14	-1,11	0,79762	-0,91	-1,87	0,02136	---	ZBTB2
8130185	-0,14	-1,11	0,74521	1,06	2,09	0,01933	NM_020861	C20orf106
8063526	-0,14	-1,11	0,71477	-1,08	-2,12	0,26433	NM_001012971	UIMC1
8115978	-0,14	-1,11	0,65067	0,92	1,89	0,00028	NM_016290	TFIP11
8075089	-0,14	-1,11	0,58617	0,39	1,31	0,18268	NM_001008697	---
8127999	-0,14	-1,11	0,51554	-0,52	-1,44	0,06936	---	TUBB2C
8165498	-0,15	-1,11	0,38205	-0,09	-1,07	0,85557	NM_006088	---
8122816	-0,15	-1,11	0,81623	-0,46	-1,38	0,04264	---	FHL3
7915147	-0,15	-1,11	0,46574	0,08	1,06	0,69666	NM_004468	SCAND2
7985587	-0,15	-1,11	0,48642	0,47	1,39	0,05369	NR_004859	IGHA1 // IGHA1
7981740	-0,15	-1,11	0,87926	-3,70	-12,96	0,00126	BC073771 // BC073771	CSRP2BP
8061138	-0,15	-1,11	0,50206	0,55	1,46	0,03743	NM_020536	---
8150373	-0,15	-1,11	0,15880	-0,75	-1,68	0,00448	---	INHBE
7956426	-0,15	-1,11	0,73789	0,51	1,43	0,00783	NM_031479	---
7971899	-0,15	-1,11	0,63353	-1,10	-2,15	0,00962	---	DYRK1B
8036763	-0,15	-1,11	0,80953	-0,84	-1,79	0,04085	NM_004714	C17orf69
8007803	-0,15	-1,11	0,64682	2,21	4,61	0,00107	NR_026905	ATP13A2
7912898	-0,15	-1,11	0,77664	1,60	3,03	0,03053	NM_022089	ZNF248
7933092	-0,15	-1,11	0,46646	0,36	1,29	0,22799	NM_021045	TDO2
8097991	-0,15	-1,11	0,77715	-0,59	-1,50	0,01543	NM_005651	EMCN
8101957	-0,15	-1,11	0,68290	-1,01	-2,01	0,02936	NM_016242	---
8168408	-0,15	-1,11	0,66244	-0,26	-1,19	0,14419	---	SNORA70B
8052524	-0,15	-1,11	0,65660	-0,48	-1,40	0,13726	NR_003707	HOXA5
8138735	-0,15	-1,11	0,65945	2,29	4,87	0,00202	NM_019102	C21orf87
8070386	-0,15	-1,11	0,69233	-0,93	-1,91	0,00078	AF426265	---
8135929	-0,15	-1,11	0,70723	-0,92	-1,89	0,18440	---	THAP10
7990086	-0,15	-1,11	0,67442	1,01	2,01	0,06455	NM_020147	ZC3H8
8054664	-0,15	-1,11	0,32885	1,28	2,42	0,00239	NM_032494	PCDHB2
8108683	-0,15	-1,11	0,84162	0,49	1,41	0,13736	NM_018936	DNAJC4
7940891	-0,15	-1,11	0,43885	0,30	1,24	0,27029	NM_005528	---
7965224	-0,15	-1,11	0,38691	-0,87	-1,83	0,04562	---	PLA2G4C
8037970	-0,15	-1,11	0,69312	0,75	1,68	0,02807	NM_003706	WASH1
7998119	-0,15	-1,11	0,87733	1,27	2,40	0,00689	NM_182905	ADRBK2
8072015	-0,15	-1,11	0,71268	2,50	5,64	0,00096	NM_005160	CELF2
7926127	-0,15	-1,11	0,82813	2,16	4,48	0,00064	NM_001025077	DFFB
7897210	-0,15	-1,11	0,54274	0,97	1,96	0,07222	NM_004402	KCNS3
8040458	-0,15	-1,11	0,60729	0,10	1,07	0,85914	NM_002252	TMEM99
8007108	-0,15	-1,11	0,61896	2,09	4,26	0,01356	NM_145274	C15orf39
7984952	-0,15	-1,11	0,69496	1,40	2,64	0,02861	NM_015492	TTLL12
8076569	-0,15	-1,11	0,52329	1,99	3,97	0,01791	NM_015140	---
7931403	-0,15	-1,11	0,69835	-1,04	-2,05	0,00133	---	TMEM22
8082905	-0,15	-1,11	0,23372	0,61	1,53	0,04690	NM_025246	---
7930919	-0,15	-1,11	0,64467	-0,26	-1,20	0,01403	---	ANKRD32
8106931	-0,15	-1,11	0,32350	0,25	1,19	0,34937	NM_032290	SS18L2
8079074	-0,15	-1,11	0,62354	1,41	2,65	0,05355	NM_016305	RAB6B
8090803	-0,15	-1,11	0,52602	1,77	3,41	0,01142	NM_016577	SLC35E4
8072374	-0,15	-1,11	0,56995	-0,18	-1,13	0,69345	NM_001001479	---
8106532	-0,15	-1,11	0,79460	-1,09	-2,13	0,00261	---	MRGPRX4
7938746	-0,15	-1,11	0,66172	-1,04	-2,05	0,00062	NM_054032	HTATSF1
8170166	-0,15	-1,11	0,66055	0,41	1,33	0,09436	NM_014500	BTBD9
8126121	-0,15	-1,11	0,58105	0,23	1,18	0,20722	NM_052893	AADAC
8083415	-0,15	-1,11	0,50006	-0,15	-1,11	0,63004	NM_001086	OTUB2
7976425	-0,15	-1,11	0,31701	1,82	3,53	0,02298	NM_023112	COX7A1
8036284	-0,15	-1,11	0,68453	-1,43	-2,69	0,00002	NM_001864	GRIA3
8169717	-0,15	-1,11	0,47457	-1,08	-2,12	0,00170	NM_007325	MFSD3
8148917	-0,15	-1,11	0,74214	1,84	3,59	0,06659	NM_138431	---
8058969	-0,15	-1,11	0,74323	-0,27	-1,20	0,17286	---	TUSC2

8087634	-0,15	-1,11	0,58699	0,71	1,63	0,16018	NM_007275	FAM8A1
8117071	-0,15	-1,11	0,06422	1,55	2,92	0,02580	NM_016255	CAPN5
7942697	-0,15	-1,11	0,56200	-0,48	-1,40	0,26605	NM_004055	C12orf10
7955777	-0,15	-1,11	0,82263	2,83	7,12	0,00104	NM_021640	LMF1
7998381	-0,15	-1,11	0,39512	0,98	1,98	0,17469	NM_022773	---
8164696	-0,15	-1,11	0,75856	-1,18	-2,27	0,06578	---	FOXRED2
8075785	-0,15	-1,11	0,54866	0,95	1,93	0,02152	NM_024955	CCDC68
8023401	-0,15	-1,11	0,33813	1,17	2,25	0,01305	NM_025214	ZNF318
8126542	-0,15	-1,11	0,52600	1,91	3,76	0,00095	NM_014345	DYX1C1
7989055	-0,15	-1,11	0,62969	-0,19	-1,14	0,52641	NM_130810	NOD1
8138842	-0,15	-1,11	0,46194	0,72	1,64	0,04114	NM_006092	OTUD5
8172478	-0,15	-1,11	0,72208	0,44	1,36	0,05398	NM_017602	---
7963863	-0,15	-1,11	0,68547	-1,35	-2,54	0,12797	---	DNAH14
7910030	-0,15	-1,11	0,63499	1,10	2,15	0,10750	NM_001145154	P2RX6
8071489	-0,15	-1,11	0,67508	-0,93	-1,91	0,15703	NM_005446	---
8044684	-0,15	-1,11	0,68773	-1,13	-2,19	0,03661	---	PYCR1
8019316	-0,15	-1,11	0,73543	2,02	4,06	0,02068	NM_006907	AEN
7985767	-0,15	-1,11	0,70779	0,81	1,75	0,20907	NM_022767	H2BFWT
8174283	-0,15	-1,11	0,60593	-0,50	-1,41	0,23696	NM_001002916	ATP7B
7971731	-0,15	-1,11	0,59074	-0,40	-1,32	0,44171	NM_000053	---
7954785	-0,15	-1,11	0,39137	-0,52	-1,44	0,00066	---	ZNF395
8149986	-0,15	-1,11	0,49327	1,38	2,60	0,00586	NM_018660	GOLGA2
8164398	-0,15	-1,11	0,41088	0,66	1,58	0,10332	NM_004486	---
7988442	-0,15	-1,11	0,39519	-1,34	-2,54	0,05385	---	GAGE1
8167573	-0,15	-1,11	0,89393	-0,31	-1,24	0,76257	NM_001468	GMPPB
8087461	-0,15	-1,11	0,65531	0,33	1,26	0,34694	NM_013334	MEGF8
8029273	-0,15	-1,11	0,72589	-0,40	-1,32	0,22365	NM_001410	CST6
7941505	-0,15	-1,11	0,73642	-0,13	-1,09	0,76770	NM_001323	SERPINB4
8023688	-0,15	-1,11	0,76726	-0,83	-1,78	0,09782	NM_002974	---
8097470	-0,15	-1,11	0,42423	-0,80	-1,74	0,03802	---	WDR91
8143070	-0,15	-1,11	0,34108	1,04	2,06	0,01347	NM_014149	GLRX5
7976515	-0,15	-1,11	0,75409	1,26	2,39	0,16913	NM_016417	MOSPD3
8134880	-0,15	-1,11	0,59442	0,63	1,55	0,01423	NM_023948	---
7969671	-0,15	-1,11	0,69223	-0,52	-1,43	0,16207	---	SLC22A4
8107909	-0,15	-1,11	0,73216	-0,25	-1,19	0,00090	NM_003059	EPGN
8095723	-0,15	-1,11	0,63055	-0,65	-1,57	0,05976	NM_001013442	HIST1H3D
8124416	-0,15	-1,11	0,30646	0,40	1,32	0,08097	NM_003530	DEFB129
8060320	-0,16	-1,11	0,50910	-1,00	-2,00	0,01776	NM_080831	---
8045007	-0,16	-1,11	0,45261	-0,79	-1,73	0,02976	---	AKR1B10
8136336	-0,16	-1,11	0,86019	-0,93	-1,91	0,00600	NM_020299	ABCC10
8119801	-0,16	-1,11	0,31455	2,05	4,13	0,00751	NM_033450	NFE2L3
8131944	-0,16	-1,11	0,61572	-0,30	-1,23	0,40102	NM_004289	KIAA0226
8093230	-0,16	-1,11	0,49872	2,49	5,63	0,00171	NM_001145642	---
8149315	-0,16	-1,11	0,56893	-0,82	-1,77	0,04433	---	SGPP1
7979574	-0,16	-1,11	0,50696	0,25	1,19	0,54351	NM_030791	PTRH1
8164235	-0,16	-1,11	0,51245	0,24	1,18	0,44058	NM_001002913	ZBTB7A
8032755	-0,16	-1,11	0,75029	1,27	2,41	0,05686	NM_015898	CDNF
7932177	-0,16	-1,11	0,62990	1,00	2,00	0,00379	NM_001029954	MTHFS
7990810	-0,16	-1,11	0,58805	1,50	2,83	0,00900	NM_006441	ENTPD6
8061428	-0,16	-1,11	0,76741	1,04	2,05	0,10917	NM_001247	NETO1
8023828	-0,16	-1,11	0,52348	-0,75	-1,68	0,00872	NM_138966	GTSF1
7963817	-0,16	-1,12	0,60600	-0,88	-1,84	0,03285	NM_144594	PILRB
8134789	-0,16	-1,12	0,11656	1,30	2,46	0,00180	NM_013440	EPHX2
8145532	-0,16	-1,12	0,74287	-0,25	-1,19	0,47837	NM_001979	LCE3D
7920185	-0,16	-1,12	0,64775	-1,19	-2,28	0,00751	NM_032563	---
8139765	-0,16	-1,12	0,70585	-1,03	-2,05	0,01780	---	LOC100128868
8135585	-0,16	-1,12	0,49269	0,28	1,22	0,42159	AY143171	---
8088908	-0,16	-1,12	0,22663	-1,05	-2,07	0,20189	---	CCDC22
8167457	-0,16	-1,12	0,54811	0,16	1,12	0,29256	NM_014008	HIST2H3D
7919589	-0,16	-1,12	0,80110	1,45	2,73	0,00104	NM_001123375	RPTOR
8010512	-0,16	-1,12	0,75973	1,41	2,66	0,02010	NM_020761	---
8154620	-0,16	-1,12	0,67343	-0,96	-1,95	0,02435	---	WDR34
8164440	-0,16	-1,12	0,58277	1,16	2,23	0,01587	NM_052844	TCF19
8118086	-0,16	-1,12	0,43164	0,37	1,29	0,23040	NM_007109	TCF19
8177947	-0,16	-1,12	0,43164	0,37	1,29	0,23040	NM_007109	WDR18
8024089	-0,16	-1,12	0,77225	1,75	3,36	0,04257	NM_024100	FAM183B
8139160	-0,16	-1,12	0,86162	-1,18	-2,26	0,25789	NR_028347	DLL4
7982854	-0,16	-1,12	0,71102	1,04	2,06	0,05038	NM_019074	CHD7
8146579	-0,16	-1,12	0,15591	2,51	5,69	0,00003	NM_017780	HMGN2
7899187	-0,16	-1,12	0,78583	-0,49	-1,40	0,11207	NM_005517	KRTAP20-4
8068157	-0,16	-1,12	0,80057	-0,88	-1,84	0,00206	NR_023342	---
8171295	-0,16	-1,12	0,82032	-1,52	-2,87	0,00391	---	NAIF1
8164357	-0,16	-1,12	0,53729	0,24	1,18	0,51042	NM_197956	MAGEA10

8175690	-0,16	-1,12	0,63695	-0,64	-1,56	0,04530	NM_001011543	ZNF587
8031827	-0,16	-1,12	0,66031	0,72	1,65	0,01151	NM_032828	TRPC2
7937878	-0,16	-1,12	0,54115	-1,18	-2,26	0,03204	NR_002720	SIGLEC1
8064716	-0,16	-1,12	0,55748	-0,58	-1,49	0,03773	NM_023068	hCG_1983896
7999550	-0,16	-1,12	0,46111	-0,47	-1,38	0,03540	ENST00000312019	LRRFIP1
8049542	-0,16	-1,12	0,61654	-0,45	-1,36	0,11698	NM_001137550	SOX13
7908993	-0,16	-1,12	0,68076	0,56	1,47	0,25963	NM_005686	---
7939374	-0,16	-1,12	0,66653	-0,62	-1,53	0,07180	---	MC3R
8063476	-0,16	-1,12	0,72480	-1,51	-2,84	0,02157	NM_019888	RNF7
8083119	-0,16	-1,12	0,82514	1,46	2,74	0,01422	NM_014245	LOC100287934
7909990	-0,16	-1,12	0,13442	-0,79	-1,73	0,21140	AK290103	CDCA7L
8138489	-0,16	-1,12	0,69266	3,33	10,09	0,00056	NM_018719	LRRC33
8084951	-0,16	-1,12	0,71818	-0,93	-1,90	0,00530	NM_198565	LOC84931
8054866	-0,16	-1,12	0,66667	-1,23	-2,34	0,00099	NR_027181	HIST1H2BJ
8124484	-0,16	-1,12	0,64641	0,52	1,43	0,38529	NM_021058	C19orf66
8025551	-0,16	-1,12	0,81177	0,14	1,10	0,79965	NM_018381	FAM194B
7971400	-0,16	-1,12	0,46122	-0,95	-1,93	0,00331	NM_182542	SNORD116-27
7982002	-0,16	-1,12	0,42601	-0,92	-1,89	0,02147	NR_003341	---
8066027	-0,16	-1,12	0,27485	-1,76	-3,40	0,30018	---	HSP90AA4P
8098707	-0,16	-1,12	0,36600	-0,49	-1,41	0,49379	AY956760	SNORA16A
7914216	-0,16	-1,12	0,65284	0,11	1,08	0,71828	NR_003035	OTOA
7993848	-0,16	-1,12	0,60805	-1,23	-2,35	0,01707	NM_144672	RPGR
8172056	-0,16	-1,12	0,49666	0,23	1,17	0,14632	NM_000328	ASB11
8171403	-0,16	-1,12	0,59687	-0,88	-1,84	0,01334	NM_080873	CCL7
8006440	-0,16	-1,12	0,31639	-1,91	-3,76	0,00416	NM_006273	DARS2
7907466	-0,16	-1,12	0,76590	1,84	3,57	0,00388	NM_018122	GNAT2
7918367	-0,16	-1,12	0,62759	-0,07	-1,05	0,81128	NM_005272	ATXN3L
8171348	-0,16	-1,12	0,66851	-0,43	-1,35	0,06719	NM_001135995	OR5K4
8081206	-0,17	-1,12	0,39517	-0,72	-1,65	0,02119	NM_001005517	C10orf131
7929525	-0,17	-1,12	0,12046	-0,43	-1,34	0,02392	AK294399	TLE6
8024537	-0,17	-1,12	0,69545	-0,10	-1,08	0,66644	NM_001143986	FGF11
8004408	-0,17	-1,12	0,62978	2,12	4,33	0,00000	NM_004112	ANKRD11
8175585	-0,17	-1,12	0,93015	1,67	3,18	0,00680	NM_013275	NBPF3
7898679	-0,17	-1,12	0,64807	0,18	1,14	0,41321	NM_032264	MCART1
8161249	-0,17	-1,12	0,78193	0,35	1,28	0,08130	NM_033412	SNORD116-11
7981970	-0,17	-1,12	0,37501	-0,81	-1,75	0,01805	NR_003326	FKBP8
8035494	-0,17	-1,12	0,79985	1,48	2,79	0,01087	NM_012181	---
8139113	-0,17	-1,12	0,54485	-0,77	-1,70	0,01927	---	SYN1
8172317	-0,17	-1,12	0,66661	-0,93	-1,90	0,00412	NM_006950	TOM1L1
8008564	-0,17	-1,12	0,57154	0,53	1,44	0,18139	AB065085	PLEKHJ1
8032455	-0,17	-1,12	0,34799	1,80	3,48	0,03246	NM_018049	KLRC1
7961187	-0,17	-1,12	0,24321	-1,04	-2,05	0,01555	NM_213658	CFHR3
7908481	-0,17	-1,12	0,53155	-0,60	-1,51	0,04016	NM_021023	ZNF667
8039593	-0,17	-1,12	0,67831	-0,54	-1,46	0,00630	NM_022103	SH3PXD2A
7936115	-0,17	-1,12	0,63737	-0,66	-1,58	0,00347	NM_014631	DDR1
8177867	-0,17	-1,12	0,74205	1,66	3,16	0,02859	NM_013993	IRG1
7969482	-0,17	-1,12	0,66858	-1,04	-2,05	0,07079	ENST00000377462	GNG5
7917232	-0,17	-1,12	0,66191	0,88	1,84	0,07074	NM_005274	IFIT1B
7929061	-0,17	-1,12	0,69340	-0,74	-1,67	0,08693	NM_001010987	---
7956908	-0,17	-1,12	0,72996	3,33	10,04	0,00454	---	LIMS1
8044258	-0,17	-1,12	0,88156	1,98	3,94	0,00559	NM_004987	C5orf53
8108475	-0,17	-1,12	0,40049	-0,01	-1,01	0,89533	NM_001007189	EID2B
8036744	-0,17	-1,12	0,64136	0,64	1,56	0,04913	NM_152361	DNAL1
7975616	-0,17	-1,12	0,67963	0,67	1,59	0,20365	NM_031427	KCNAB2
7897236	-0,17	-1,12	0,60808	1,86	3,62	0,00628	NM_003636	---
8053427	-0,17	-1,12	0,89411	-2,25	-4,77	0,02167	---	PGS1
8010271	-0,17	-1,12	0,49999	3,26	9,59	0,00490	NM_024419	NEIL3
8098423	-0,17	-1,12	0,72122	2,66	6,30	0,00982	NM_018248	XRCC4
8106730	-0,17	-1,12	0,75832	1,12	2,18	0,01403	NM_022550	---
8052121	-0,17	-1,12	0,49605	-0,87	-1,83	0,02324	---	LOC100131756
7915498	-0,17	-1,12	0,44499	-1,01	-2,01	0,03087	AK096139	NMNAT1
7897509	-0,17	-1,12	0,67630	0,34	1,27	0,41176	NM_022787	NPHP1
8054486	-0,17	-1,12	0,26062	0,03	1,02	0,80567	NM_000272	C4orf22
8096061	-0,17	-1,12	0,64447	-0,46	-1,38	0,06065	BC034296	---
7909102	-0,17	-1,12	0,66793	0,15	1,11	0,69536	---	FAM40B
8136115	-0,17	-1,12	0,62644	0,99	1,98	0,00254	NM_020704	FLG2
7920175	-0,17	-1,12	0,45725	-0,96	-1,95	0,00246	NM_001014342	SLC35E3
7956978	-0,17	-1,12	0,56617	1,01	2,01	0,00414	NM_018656	EBP
8167305	-0,17	-1,12	0,68810	2,96	7,77	0,00288	NM_006579	AMOT
8174576	-0,17	-1,13	0,43402	0,30	1,23	0,39170	NM_133265	MICAL3
8074286	-0,17	-1,13	0,51475	1,48	2,80	0,01341	NM_015241	---
7991155	-0,17	-1,13	0,16818	-1,12	-2,18	0,00111	---	ZNF781
8036430	-0,17	-1,13	0,46333	0,14	1,10	0,72015	NM_152605	NANP

8065510	-0,17	-1,13	0,64464	0,27	1,20	0,09898	NM_152667	FOXO1
7971177	-0,17	-1,13	0,68524	-0,42	-1,34	0,17182	NM_002015	TIAM2
8122933	-0,17	-1,13	0,01392	-0,35	-1,27	0,25513	NM_012454	ADAM30
7919088	-0,17	-1,13	0,57019	-0,86	-1,82	0,06456	NM_021794	MIR504
8175455	-0,17	-1,13	0,71236	-0,66	-1,58	0,04778	NR_030229	---
8040288	-0,17	-1,13	0,75418	-1,42	-2,68	0,01119	---	CXorf40A
8170393	-0,17	-1,13	0,56021	-0,51	-1,43	0,20955	NM_178124	LOC644538
8170007	-0,17	-1,13	0,69414	-1,25	-2,38	0,00261	NM_001163438	MIR1977
7911339	-0,17	-1,13	0,89294	-5,06	-33,30	0,00034	NR_031741	MIR1977
8165698	-0,17	-1,13	0,89294	-5,06	-33,30	0,00034	NR_031741	PEX7
8122242	-0,17	-1,13	0,57570	0,74	1,67	0,00541	NM_000288	SPPL2B
8024446	-0,17	-1,13	0,63166	1,00	2,00	0,15050	NM_001077238	SMEK3P
8171883	-0,17	-1,13	0,45874	-0,73	-1,66	0,03108	NR_002784	CDKL3
8114171	-0,17	-1,13	0,14052	-0,12	-1,09	0,60939	NM_001113575	OR4D9
7940189	-0,17	-1,13	0,43351	-1,25	-2,38	0,13548	NM_001004711	ZNF660
8079198	-0,17	-1,13	0,58694	-0,47	-1,38	0,12289	NM_173658	RAB15
7979663	-0,17	-1,13	0,53577	2,88	7,37	0,00083	NM_198686	WNT3
8016311	-0,17	-1,13	0,74098	0,89	1,86	0,00669	NM_030753	CCDC29
7970381	-0,17	-1,13	0,77822	0,90	1,87	0,07161	ENST00000340460	COMP
8035517	-0,17	-1,13	0,31636	-0,78	-1,72	0,02348	NM_000095	SNORA67
8004508	-0,17	-1,13	0,51279	0,77	1,70	0,05091	NR_002912	NELF
8165552	-0,17	-1,13	0,61620	1,76	3,39	0,00070	NM_001130969	LIF
8072314	-0,17	-1,13	0,62936	-0,39	-1,31	0,29190	NM_002309	SMUG1
7963741	-0,17	-1,13	0,69634	2,20	4,61	0,00508	NM_014311	GZMA
8105340	-0,17	-1,13	0,22442	-0,33	-1,26	0,22292	NM_006144	CNTN1
7954899	-0,17	-1,13	0,63429	3,35	10,19	0,01342	NM_001843	ZNF565
8036291	-0,17	-1,13	0,68582	-0,54	-1,46	0,00991	NM_001042474	---
8097934	-0,17	-1,13	0,65983	-0,54	-1,46	0,02001	---	ZNF699
8033746	-0,17	-1,13	0,69276	0,48	1,39	0,12558	NM_198535	NKAIN3
8146625	-0,17	-1,13	0,43633	-0,51	-1,42	0,00148	NM_173688	SCAND3
8124594	-0,17	-1,13	0,44964	0,19	1,14	0,30902	NM_052923	IGLV7-46
8071658	-0,17	-1,13	0,35211	-1,37	-2,58	0,00859	ENST00000390295	SEMA3E
8140650	-0,17	-1,13	0,68290	0,54	1,45	0,23098	NM_012431	KLRC2
7961182	-0,17	-1,13	0,45082	-1,18	-2,26	0,00399	NM_002260	ZBTB8B
7899797	-0,17	-1,13	0,46519	0,29	1,22	0,29266	NM_001145720	FAM105B
8104580	-0,18	-1,13	0,72704	1,56	2,96	0,00010	NM_138348	---
8069766	-0,18	-1,13	0,67777	-0,14	-1,10	0,38663	---	SLC44A3
7903144	-0,18	-1,13	0,42829	2,64	6,25	0,00051	NM_001114106	ZNF676
8035825	-0,18	-1,13	0,46469	-0,67	-1,59	0,07651	NM_001001411	TRIM49
7943036	-0,18	-1,13	0,82373	-2,28	-4,85	0,00003	NM_020358	---
7974164	-0,18	-1,13	0,43793	-0,42	-1,34	0,17738	---	DBF4B
8007651	-0,18	-1,13	0,31939	-0,14	-1,10	0,42177	NM_145663	C9orf95
8161839	-0,18	-1,13	0,60403	1,07	2,10	0,00756	NR_023352	FKSG83
8117567	-0,18	-1,13	0,48224	-0,83	-1,78	0,00748	NM_032030	HIST1H2AI
8117580	-0,18	-1,13	0,61540	0,46	1,38	0,42591	NM_003509	GOLGA6L1
7986736	-0,18	-1,13	0,83301	-1,57	-2,96	0,03797	NM_001001413	KIF3C
8050894	-0,18	-1,13	0,59482	2,19	4,57	0,00178	NM_002254	IL12A
8083690	-0,18	-1,13	0,67314	0,08	1,06	0,77638	NM_000882	---
8150842	-0,18	-1,13	0,50125	-0,43	-1,35	0,20842	---	TBC1D3P2
8017346	-0,18	-1,13	0,70850	0,52	1,43	0,36487	NR_027486	THSD7A
8138231	-0,18	-1,13	0,60731	-0,83	-1,78	0,04059	NM_015204	FANCM
7974166	-0,18	-1,13	0,65920	1,10	2,14	0,05189	NM_020937	TLR1
8099834	-0,18	-1,13	0,13881	-0,81	-1,75	0,04120	NM_003263	LOC100128288
8012464	-0,18	-1,13	0,83623	1,59	3,01	0,03727	NR_024447	ABHD12B
7974288	-0,18	-1,13	0,58256	-0,74	-1,67	0,00888	NM_181533	IFFO2
7912994	-0,18	-1,13	0,60190	1,88	3,69	0,01952	NM_001136265	PDZD11
8173373	-0,18	-1,13	0,83074	0,10	1,07	0,70529	NM_016484	CALML6
7897026	-0,18	-1,13	0,78187	-1,63	-3,09	0,00024	NM_138705	MOGAT2
7942603	-0,18	-1,13	0,60508	-1,05	-2,07	0,04071	NM_025098	---
8008491	-0,18	-1,13	0,58096	-0,99	-1,99	0,00025	---	C10orf68
7926936	-0,18	-1,13	0,43431	-0,64	-1,56	0,05819	NM_024688	HSP90AB4P
7989245	-0,18	-1,13	0,66366	0,30	1,23	0,14538	NR_002927	UBQLNL
7946082	-0,18	-1,13	0,52407	-1,07	-2,10	0,00718	NM_145053	ANKRD20B
8155591	-0,18	-1,13	0,62894	0,38	1,30	0,33477	NR_003366	NPM1
8109975	-0,18	-1,13	0,62339	-1,13	-2,19	0,11090	NM_001037738	C1orf156
7922243	-0,18	-1,13	0,17731	0,44	1,36	0,17832	NM_033418	SIGLEC14
8038885	-0,18	-1,13	0,70442	-2,04	-4,12	0,00051	NM_001098612	ZCWPW1
8141498	-0,18	-1,13	0,29123	-0,43	-1,35	0,04084	NM_017984	---
8111666	-0,18	-1,13	0,47872	-2,13	-4,37	0,04522	---	---
8167772	-0,18	-1,13	0,85522	-2,44	-5,42	0,02248	---	FERMT1
8064904	-0,18	-1,13	0,56030	2,54	5,83	0,00116	NM_017671	CLPB
7950142	-0,18	-1,13	0,76355	1,21	2,31	0,03538	NM_030813	---
8111253	-0,18	-1,13	0,53554	-1,19	-2,29	0,00068	---	---

8072344	-0,18	-1,13	0,72487	-0,19	-1,14	0,59169	---	LRTM1	
8088174	-0,18	-1,13	0,46690	-0,96	-1,94	0,00103	NM_020678		---
8068399	-0,18	-1,13	0,41691	-1,37	-2,59	0,00315	---	OMD	
8162388	-0,18	-1,13	0,42151	-1,07	-2,10	0,00078	NM_005014		---
7923501	-0,18	-1,13	0,84229	-1,01	-2,02	0,05959	---	ZNF41	
8172307	-0,18	-1,13	0,27318	0,42	1,34	0,02443	NM_007130		BHLHB9
8168984	-0,18	-1,13	0,18159	-0,07	-1,05	0,79075	NM_001142524		OR5F1
7948115	-0,18	-1,13	0,75568	-1,68	-3,20	0,05680	NM_003697		HS3ST5
8129062	-0,18	-1,13	0,38739	-0,53	-1,44	0,02936	NM_153612		ZNF607
8036436	-0,18	-1,13	0,51843	1,96	3,90	0,00122	NM_032689		CDC20
7900699	-0,18	-1,13	0,70914	2,27	4,83	0,00198	NM_001255		OR2C1
7992889	-0,18	-1,13	0,06733	-0,34	-1,27	0,15974	NM_012368		ZNF514
8053775	-0,18	-1,13	0,47678	-0,22	-1,16	0,56209	NM_032788		IQCE
8131155	-0,18	-1,13	0,73079	0,25	1,19	0,31111	NM_152558		NTF3
7953284	-0,18	-1,14	0,58379	-0,86	-1,81	0,00340	NM_002527		---
8022378	-0,18	-1,14	0,42253	-0,59	-1,51	0,02266	---	---	
8017391	-0,18	-1,14	0,32543	-0,30	-1,23	0,24045	---	LRRK2	
7954810	-0,18	-1,14	0,51766	-0,29	-1,23	0,40701	NM_198578		DPYSL4
7931439	-0,18	-1,14	0,06103	-0,79	-1,73	0,00060	NM_006426		---
8132705	-0,18	-1,14	0,43859	-1,28	-2,43	0,00535	---	---	
8062693	-0,18	-1,14	0,48274	0,03	1,02	0,82963	---	---	
8036881	-0,18	-1,14	0,74962	-1,98	-3,95	0,00069	---	TAAR8	
8122129	-0,18	-1,14	0,37730	-1,24	-2,35	0,02656	NM_053278		BCAM
8029489	-0,18	-1,14	0,29195	2,42	5,34	0,00890	NM_005581		C22orf40
8076749	-0,18	-1,14	0,72584	0,78	1,71	0,08618	NM_207327		NMBR
8129947	-0,18	-1,14	0,46356	-0,77	-1,71	0,03291	NM_002511		RNF31
7973584	-0,18	-1,14	0,60193	0,90	1,87	0,02214	NM_017999		AFMID
8010248	-0,18	-1,14	0,59722	1,23	2,35	0,03684	NM_001010982		ING5
8077226	-0,18	-1,14	0,58693	0,09	1,06	0,52815	NM_032329		SNRPF
7957649	-0,18	-1,14	0,34562	0,63	1,54	0,04004	NM_003095		---
8083061	-0,18	-1,14	0,69325	0,42	1,33	0,04413	---	LSM14B	
8063814	-0,18	-1,14	0,02757	0,16	1,12	0,62245	NM_144703		MIR135A2
7957735	-0,19	-1,14	0,13478	-0,73	-1,66	0,00131	NR_029678		PSMC3IP
8015642	-0,19	-1,14	0,49088	-0,32	-1,25	0,36108	NM_016556		LRWD1
8135181	-0,19	-1,14	0,52631	0,33	1,25	0,30410	NM_152892		HARBI1
7947676	-0,19	-1,14	0,55335	0,00	-1,00	0,99016	NM_173811		---
8095626	-0,19	-1,14	0,54776	-0,89	-1,85	0,12966	---	AQP9	
7983910	-0,19	-1,14	0,37330	-0,80	-1,74	0,00959	NM_020980		LOR
7905563	-0,19	-1,14	0,49074	-1,43	-2,70	0,00072	NM_000427		CEP110
8157534	-0,19	-1,14	0,47307	1,08	2,11	0,09267	NM_007018		ZNF75A
7992887	-0,19	-1,14	0,26175	0,82	1,77	0,04031	AK093979	OR3A3	
8003812	-0,19	-1,14	0,80674	-2,35	-5,08	0,00744	NM_012373		ARPM1
8092002	-0,19	-1,14	0,53523	-0,61	-1,52	0,11285	NM_032487		OR1S1
7940114	-0,19	-1,14	0,65039	-1,66	-3,16	0,00383	NM_001004458		---
8005829	-0,19	-1,14	0,46804	-0,60	-1,51	0,01284	---	---	
8085058	-0,19	-1,14	0,53635	0,13	1,10	0,84007	---	PARP11	
7960383	-0,19	-1,14	0,58331	-0,04	-1,03	0,90330	NM_020367		CCDC41
7965486	-0,19	-1,14	0,02091	0,69	1,61	0,00073	NM_016122		GOLGA4
8086183	-0,19	-1,14	0,72487	-0,80	-1,74	0,12664	NM_001172713		---
7970567	-0,19	-1,14	0,50845	-0,50	-1,42	0,04637	---	C8orf46	
8146703	-0,19	-1,14	0,32751	0,06	1,04	0,86159	NM_152765		TAF3
7926096	-0,19	-1,14	0,55798	0,02	1,02	0,89947	NM_031923		---
8122598	-0,19	-1,14	0,76944	-1,26	-2,39	0,00005	---	HMCN2	
8158637	-0,19	-1,14	0,62863	-1,39	-2,62	0,00134	AL834139	MUC1	
7920642	-0,19	-1,14	0,82944	1,17	2,24	0,02119	NM_001018016		ESRRB
7975876	-0,19	-1,14	0,36674	-1,21	-2,32	0,01173	NM_004452		C1QTNF9
7968052	-0,19	-1,14	0,57733	0,25	1,19	0,25181	NM_178540		PNKP
8038458	-0,19	-1,14	0,62709	-0,12	-1,09	0,73517	NM_007254		IKBKG
8170955	-0,19	-1,14	0,56542	1,04	2,05	0,10931	NM_001099857		GLRX5
8169709	-0,19	-1,14	0,90176	1,18	2,27	0,38149	NM_016417		HIST1H2BA
8117301	-0,19	-1,14	0,53878	-0,18	-1,13	0,08634	NM_170610		ULK4
8086372	-0,19	-1,14	0,27003	0,01	1,01	0,92500	NM_017886		SLC4A8
7955469	-0,19	-1,14	0,59351	0,08	1,06	0,56800	NM_001039960		ACVR1C
8055992	-0,19	-1,14	0,66809	1,12	2,17	0,01103	NM_145259		---
8052731	-0,19	-1,14	0,82702	-2,30	-4,92	0,02593	---	OR4M1	
7973008	-0,19	-1,14	0,81386	-1,29	-2,44	0,01269	NM_001005500		SCN5A
8086222	-0,19	-1,14	0,60411	-0,46	-1,37	0,04748	NM_001099404		IMPG1
8127666	-0,19	-1,14	0,29761	-1,13	-2,18	0,00031	NM_001563		SFXN2
7930148	-0,19	-1,14	0,27139	2,76	6,78	0,00247	NM_178858		VRK1
7976621	-0,19	-1,14	0,73916	2,06	4,18	0,00183	NM_003384		KIF5C
8045637	-0,19	-1,14	0,71186	1,40	2,65	0,00100	NM_004522		C9orf57
8161739	-0,19	-1,14	0,59020	-0,27	-1,20	0,27159	NM_001128618		---
8100310	-0,19	-1,14	0,93054	-6,44	-87,12	0,00277	---	C12orf45	

7958211	-0,19	-1,14	0,04424	0,07	1,05	0,86386	NM_152318	RASSF5
7909214	-0,19	-1,14	0,69435	0,33	1,26	0,35644	NM_182663	MIR16-1
7971659	-0,19	-1,14	0,41495	-0,57	-1,49	0,00234	NR_029486	HSPA1L
8125134	-0,19	-1,14	0,57060	1,24	2,36	0,01957	NM_005527	HSPA1L
8178650	-0,19	-1,14	0,57060	1,24	2,36	0,01957	NM_005527	HSPA1L
8179848	-0,19	-1,14	0,57060	1,24	2,36	0,01957	NM_005527	---
8163011	-0,19	-1,14	0,49375	-0,26	-1,20	0,12499	---	MARVELD2
8105899	-0,19	-1,14	0,37046	3,10	8,55	0,00134	NM_001038603	MARVELD2
8177498	-0,19	-1,14	0,37046	3,10	8,55	0,00134	NM_001038603	ATP4A
8036110	-0,19	-1,14	0,63569	-1,17	-2,24	0,02500	NM_000704	C12orf54
7955119	-0,19	-1,14	0,70934	-0,81	-1,76	0,00189	NM_152319	C14orf159
7976216	-0,19	-1,14	0,12308	-0,73	-1,65	0,01127	NM_001102366	FBXO39
8004195	-0,19	-1,14	0,61854	-1,29	-2,44	0,04963	NM_153230	NFATC4
7973709	-0,19	-1,14	0,55619	-0,12	-1,09	0,50073	NM_004554	SFRS5
7975368	-0,19	-1,14	0,30467	2,31	4,97	0,00001	NM_001039465	RILPL1
7967463	-0,19	-1,14	0,44401	2,04	4,12	0,01777	NM_178314	TMEM53
7915578	-0,19	-1,14	0,46880	1,29	2,45	0,00706	NM_024587	---
7918951	-0,19	-1,14	0,41732	-0,15	-1,11	0,63839	---	---
8133898	-0,19	-1,14	0,49694	-0,95	-1,93	0,00568	---	MIR17HG
7969576	-0,19	-1,14	0,32632	-0,66	-1,58	0,00583	NR_027350	---
7965587	-0,19	-1,14	0,53540	-0,98	-1,97	0,02114	---	GATC
7959157	-0,19	-1,14	0,19366	2,00	4,00	0,01685	NM_176818	SNORD37
8032749	-0,19	-1,14	0,09219	-0,56	-1,47	0,08452	NR_002602	PPM1K
8101701	-0,19	-1,14	0,51998	-0,38	-1,30	0,15093	NM_152542	TMEM93
8003840	-0,19	-1,14	0,68583	0,82	1,77	0,16064	NM_001014764	FKSG2
8145824	-0,19	-1,14	0,26334	-0,87	-1,82	0,01143	ENST00000402782	SNORD114-26
7976826	-0,19	-1,14	0,72528	-0,97	-1,95	0,01549	NR_003219	MYT1
8064277	-0,19	-1,14	0,61763	-0,69	-1,61	0,09136	NM_004535	CNTNAP3
8161288	-0,19	-1,14	0,20558	0,17	1,12	0,39795	NM_033655	GPC4
8175217	-0,19	-1,14	0,64177	-0,53	-1,44	0,01270	NM_001448	CYB561
8017378	-0,20	-1,14	0,58410	1,46	2,76	0,01092	NM_001915	KIR2DL4
8031346	-0,20	-1,14	0,73617	-1,41	-2,66	0,00012	NM_002255	THOC3
8110408	-0,20	-1,14	0,06276	1,15	2,22	0,01568	NM_032361	SPAG16
8048081	-0,20	-1,15	0,30660	0,07	1,05	0,79638	NM_024532	DDX28
8002211	-0,20	-1,15	0,51552	0,82	1,77	0,05598	NM_018380	EVI5L
8025328	-0,20	-1,15	0,50219	0,72	1,65	0,10327	NM_001159944	MRPL13
8152597	-0,20	-1,15	0,38088	0,03	1,02	0,93615	NM_014078	LOC80054
8027564	-0,20	-1,15	0,27531	-0,44	-1,36	0,17847	NR_026887	PYCR2
7924669	-0,20	-1,15	0,24988	1,80	3,49	0,00274	NM_013328	MIR517C
8031037	-0,20	-1,15	0,64670	-1,80	-3,48	0,03041	NR_030214	PCYT2
8019280	-0,20	-1,15	0,62584	1,06	2,09	0,03592	NM_002861	TFEB
8126312	-0,20	-1,15	0,56222	0,22	1,17	0,35046	NM_007162	GJD2
7987310	-0,20	-1,15	0,71950	-1,76	-3,38	0,01699	NM_020660	B4GALT3
7921806	-0,20	-1,15	0,23749	2,95	7,71	0,00125	NM_003779	---
7999356	-0,20	-1,15	0,58631	-0,46	-1,38	0,27151	---	C1orf131
7925033	-0,20	-1,15	0,57150	-0,19	-1,14	0,63640	NM_152379	C1orf161
7904244	-0,20	-1,15	0,49403	-2,37	-5,17	0,00003	NM_152367	---
8078260	-0,20	-1,15	0,49441	-0,46	-1,37	0,00469	---	NOSTRIN
8046099	-0,20	-1,15	0,31096	-0,78	-1,72	0,16114	NM_001039724	---
8063524	-0,20	-1,15	0,67025	-1,78	-3,44	0,00304	---	OPRL1
8064261	-0,20	-1,15	0,37527	-0,37	-1,29	0,36056	NM_182647	WDR66
7959330	-0,20	-1,15	0,58637	-0,20	-1,15	0,40119	NM_144668	TRMT11
8121895	-0,20	-1,15	0,19894	0,99	1,99	0,10516	NM_001031712	CHST3
7928291	-0,20	-1,15	0,70383	-0,85	-1,80	0,06758	NM_004273	---
8020347	-0,20	-1,15	0,43302	-1,08	-2,11	0,24955	---	C2
8118324	-0,20	-1,15	0,47677	-0,34	-1,26	0,07118	NM_000063	AXIN2
8017718	-0,20	-1,15	0,52856	-0,97	-1,96	0,08763	NM_004655	SDPR
8057797	-0,20	-1,15	0,71795	0,49	1,40	0,12631	NM_004657	PMEPA1
8067233	-0,20	-1,15	0,45135	2,30	4,91	0,00436	NM_020182	TLR6
8099841	-0,20	-1,15	0,59137	1,83	3,55	0,00694	NM_006068	DPP9
8032871	-0,20	-1,15	0,43955	3,78	13,73	0,00079	NM_139159	---
7919155	-0,20	-1,15	0,86006	-1,26	-2,40	0,00732	---	---
7919405	-0,20	-1,15	0,86006	-1,26	-2,40	0,00732	---	ANKRD36
8043687	-0,20	-1,15	0,79839	1,19	2,28	0,14364	NM_001164315	---
8053666	-0,20	-1,15	0,68437	-1,24	-2,37	0,00032	---	C8orf58
8145193	-0,20	-1,15	0,50540	-0,54	-1,45	0,19725	NM_001013842	ZBTB47
8079099	-0,20	-1,15	0,35543	-0,05	-1,03	0,87281	NM_145166	RPP25
7990442	-0,20	-1,15	0,31449	0,05	1,04	0,91779	NM_017793	SOCS2
7957551	-0,20	-1,15	0,59245	0,95	1,94	0,05132	NM_003877	TOP1MT
8153390	-0,20	-1,15	0,39174	1,46	2,75	0,01174	NM_052963	MATN1
7914260	-0,20	-1,15	0,32911	-0,78	-1,72	0,12554	NM_002379	ATP1B4
8169687	-0,20	-1,15	0,62203	-0,91	-1,88	0,01880	NM_001142447	LYVE1
7946579	-0,20	-1,15	0,29355	-0,84	-1,79	0,01523	NM_006691	GTF2H4

8118007	-0,20	-1,15	0,60679	1,88	3,68	0,00205	NM_001517	GTF2H4
8177885	-0,20	-1,15	0,60679	1,88	3,68	0,00205	NM_001517	GTF2H4
8179205	-0,20	-1,15	0,60679	1,88	3,68	0,00205	NM_001517	SEMA4B
7985934	-0,20	-1,15	0,55983	3,55	11,68	0,00384	NM_020210	RPA4
8168723	-0,20	-1,15	0,34763	-0,61	-1,52	0,04887	NM_013347	FZR1
8024637	-0,20	-1,15	0,37677	-0,04	-1,03	0,90508	NM_001136198	TMED8
7980381	-0,20	-1,15	0,64831	1,94	3,85	0,00317	NM_213601	14. Sep
8139727	-0,20	-1,15	0,59446	-0,49	-1,40	0,10047	NM_207366	---
7904953	-0,20	-1,15	0,07163	-0,20	-1,15	0,11981	---	TMEM191A
8071368	-0,20	-1,15	0,65997	-2,00	-4,00	0,03374	NR_026815	GPR21
8157727	-0,20	-1,15	0,76537	-0,99	-1,99	0,00810	NM_005294	DNAH17
8018902	-0,20	-1,15	0,49166	-0,02	-1,01	0,89768	NM_173628	---
8078448	-0,20	-1,15	0,38049	-1,15	-2,21	0,08527	---	KLHL1
7971937	-0,20	-1,15	0,29376	-0,75	-1,69	0,00003	NM_020866	TMCO6
8108579	-0,20	-1,15	0,68850	1,70	3,25	0,00690	NM_018502	---
8035868	-0,20	-1,15	0,69298	-0,63	-1,55	0,02672	---	ZNF439
8025964	-0,20	-1,15	0,67045	-0,77	-1,71	0,12189	NM_152262	LOC100131860
7991047	-0,20	-1,15	0,84785	-0,89	-1,86	0,30609	AK097109	NACAP1
8147693	-0,20	-1,15	0,40497	-0,20	-1,15	0,58727	NR_002182	STAG3L3
8139977	-0,20	-1,15	0,79118	1,23	2,34	0,01257	NM_001013739	---
7947674	-0,20	-1,15	0,24182	-0,24	-1,18	0,40247	---	ACSM3
7993756	-0,20	-1,15	0,30758	1,92	3,80	0,00524	NM_005622	---
7986763	-0,20	-1,15	0,38196	-1,02	-2,02	0,07116	---	GSG2
8003844	-0,20	-1,15	0,58368	1,48	2,79	0,00642	NM_031965	TMEM156
8099850	-0,20	-1,15	0,62272	4,57	23,73	0,00027	NM_024943	NSUN5P2
8139947	-0,20	-1,15	0,60793	0,77	1,71	0,19556	NR_033323	CRADD
7957560	-0,20	-1,15	0,37578	0,56	1,47	0,01408	NM_003805	HPGD
8103769	-0,20	-1,15	0,14436	-0,49	-1,41	0,00314	NM_000860	CER1
8160163	-0,20	-1,15	0,52585	-0,89	-1,85	0,06011	NM_005454	---
8149248	-0,21	-1,15	0,61596	-1,11	-2,16	0,02726	---	ZNF155
8029340	-0,21	-1,15	0,40918	0,01	1,01	0,94810	NM_003445	---
8085024	-0,21	-1,15	0,13861	-0,62	-1,54	0,13973	---	---
7968732	-0,21	-1,15	0,36313	-0,93	-1,90	0,06164	---	ZNF833
8025933	-0,21	-1,15	0,41769	-0,49	-1,41	0,07200	NR_028594	---
7901477	-0,21	-1,15	0,65364	-0,80	-1,74	0,01333	---	ATP6AP1L
8106722	-0,21	-1,15	0,35605	-0,68	-1,61	0,01665	NM_001017971	SUZ12P
8006183	-0,21	-1,15	0,68423	2,61	6,09	0,00119	NR_024187	FLJ43390
7974900	-0,21	-1,15	0,48390	-1,52	-2,87	0,00344	AK125380	CASR
8082046	-0,21	-1,15	0,72392	-1,32	-2,50	0,00974	NM_000388	KLHL21
7912102	-0,21	-1,15	0,70060	0,31	1,24	0,22380	NM_014851	SLC39A11
8018082	-0,21	-1,15	0,61715	1,87	3,65	0,04272	NM_001159770	FAM136A
8052866	-0,21	-1,15	0,74258	2,61	6,10	0,00303	NM_032822	---
8156599	-0,21	-1,15	0,69407	-0,05	-1,03	0,89263	---	---
7899600	-0,21	-1,16	0,46534	-1,56	-2,95	0,01326	---	TRIM56
8135064	-0,21	-1,16	0,51490	0,61	1,52	0,18917	NM_030961	TBX19
7907146	-0,21	-1,16	0,74188	0,64	1,56	0,23897	NM_005149	ZNF526
8029188	-0,21	-1,16	0,70433	1,49	2,82	0,01268	NM_133444	IL23R
7902189	-0,21	-1,16	0,25032	-0,67	-1,59	0,04021	NM_144701	PLEKHO2
7984217	-0,21	-1,16	0,29053	0,06	1,05	0,88806	NM_025201	OR10D4P
7952390	-0,21	-1,16	0,26258	-0,59	-1,50	0,04696	ENST00000334203	C20orf132
8066161	-0,21	-1,16	0,58686	-0,66	-1,57	0,01166	NM_152503	ARHGEF37
8109161	-0,21	-1,16	0,25091	0,11	1,08	0,44569	NM_001001669	KLF13
7982326	-0,21	-1,16	0,57171	1,60	3,04	0,02909	NM_015995	ZNF582
8039586	-0,21	-1,16	0,53286	0,34	1,26	0,29524	NM_144690	---
7939118	-0,21	-1,16	0,37617	-0,44	-1,36	0,03652	---	OR52A5
7946023	-0,21	-1,16	0,56739	-0,97	-1,96	0,06681	NM_001005160	---
8152666	-0,21	-1,16	0,31606	-0,87	-1,83	0,06113	---	FERMT3
7940869	-0,21	-1,16	0,58685	-0,20	-1,15	0,68125	NM_178443	TMEM9
7923319	-0,21	-1,16	0,42567	1,72	3,29	0,02720	NM_016456	PNLIPRP2
7930804	-0,21	-1,16	0,52190	-0,50	-1,41	0,05572	NM_005396	ABCG2
8101675	-0,21	-1,16	0,61584	1,17	2,25	0,00349	NM_004827	RNF145
8115562	-0,21	-1,16	0,39907	1,25	2,38	0,00003	NM_144726	RHD
7898998	-0,21	-1,16	0,32282	-0,19	-1,14	0,46589	NM_016124	---
7929130	-0,21	-1,16	0,30516	-0,45	-1,36	0,27761	---	ZNF786
8143697	-0,21	-1,16	0,50176	0,71	1,63	0,03631	NM_152411	SLC43A2
8011093	-0,21	-1,16	0,59415	0,32	1,25	0,24855	NM_152346	TTC18
7934334	-0,21	-1,16	0,27984	-0,27	-1,21	0,02044	NM_145170	---
8021540	-0,21	-1,16	0,43631	-1,03	-2,04	0,00870	---	SPIN4
8173208	-0,21	-1,16	0,60281	0,49	1,40	0,20139	NM_001012968	SYN3
8075616	-0,21	-1,16	0,70139	-0,73	-1,66	0,00533	NM_003490	---
8130181	-0,21	-1,16	0,41031	-1,46	-2,76	0,01711	---	FAM74A3
8155376	-0,21	-1,16	0,54782	-1,39	-2,62	0,00059	ENST00000377629	KLRG1
7953835	-0,21	-1,16	0,49875	0,55	1,47	0,13469	NM_005810	---

7910186	-0,21	-1,16	0,64579	-0,23	-1,17	0,31126	---	TGM6
8060447	-0,21	-1,16	0,66446	-0,99	-1,98	0,14010	NM_198994	CLSTN3
7953626	-0,21	-1,16	0,33640	1,20	2,29	0,01186	NM_014718	---
8131967	-0,21	-1,16	0,52880	1,34	2,52	0,03341	---	---
8172032	-0,21	-1,16	0,25948	-1,45	-2,74	0,04343	---	ANKRD36B
8054064	-0,21	-1,16	0,65582	-0,19	-1,14	0,65549	NM_025190	MORN3
7967193	-0,21	-1,16	0,52657	-1,06	-2,08	0,00098	NM_173855	KLF4
8163002	-0,21	-1,16	0,26481	1,36	2,57	0,00488	NM_004235	CCDC28B
7899719	-0,21	-1,16	0,37243	-1,09	-2,13	0,03368	NM_024296	LILRA5
8039236	-0,21	-1,16	0,27084	-0,40	-1,32	0,21727	NM_021250	ARSA
8077160	-0,21	-1,16	0,05261	0,94	1,91	0,09660	NM_000487	COL6A2
8069301	-0,21	-1,16	0,66211	-0,94	-1,92	0,12444	NM_001849	DIO1
7901565	-0,21	-1,16	0,08476	-0,27	-1,20	0,17062	NM_000792	---
8150157	-0,21	-1,16	0,34427	-1,40	-2,64	0,00431	---	INO80C
8022902	-0,21	-1,16	0,55020	0,19	1,14	0,68897	NM_001098817	CPA6
8151191	-0,21	-1,16	0,34414	-0,99	-1,99	0,00155	NM_020361	---
7944989	-0,21	-1,16	0,35811	-1,78	-3,44	0,00003	---	KRTAP4-7
8015218	-0,21	-1,16	0,92008	-4,03	-16,30	0,00119	NM_033061	APOA4
8077192	-0,21	-1,16	0,38542	-0,67	-1,59	0,00832	NM_000482	SNORD51
8047778	-0,21	-1,16	0,54131	-0,82	-1,77	0,09884	NR_002589	---
8149273	-0,21	-1,16	0,73696	-0,56	-1,48	0,14795	---	FLVCR2
7975799	-0,21	-1,16	0,60417	1,25	2,39	0,00031	NM_017791	IFNA13
8160419	-0,21	-1,16	0,71490	-0,42	-1,34	0,09638	NM_006900	MARK1
7909841	-0,21	-1,16	0,46024	2,47	5,52	0,00263	NM_018650	CALHM2
7936100	-0,21	-1,16	0,37106	-0,86	-1,82	0,22920	NM_015916	DAK
7940451	-0,21	-1,16	0,58820	2,22	4,66	0,01124	NM_015533	TP53I11
7947570	-0,22	-1,16	0,08901	-0,56	-1,47	0,26162	NM_001076787	RPS14
8115158	-0,22	-1,16	0,69928	3,95	15,41	0,00048	NM_001025071	CBLN3
7978331	-0,22	-1,16	0,47470	-0,35	-1,27	0,25111	NM_001039771	ZNF684
7900461	-0,22	-1,16	0,24033	0,12	1,09	0,49533	NM_152373	ZNF569
8036395	-0,22	-1,16	0,31333	0,91	1,88	0,07109	NM_152484	IL12B
8115570	-0,22	-1,16	0,51680	-1,03	-2,04	0,04786	NM_002187	SLED1
8103975	-0,22	-1,16	0,58581	-0,95	-1,94	0,00630	NR_003542	GCNT2
8116835	-0,22	-1,16	0,65880	1,22	2,33	0,00561	NM_145649	---
8047769	-0,22	-1,16	0,55667	-0,73	-1,66	0,01824	---	C10orf54
7934185	-0,22	-1,16	0,54402	0,54	1,45	0,18451	NM_022153	SLC37A4
7952132	-0,22	-1,16	0,64797	2,61	6,09	0,01416	NM_001164277	PLEKHA9
7962489	-0,22	-1,16	0,51758	0,78	1,71	0,06471	NM_015899	ROPN1L
8104492	-0,22	-1,16	0,60150	-0,87	-1,83	0,05333	NM_031916	CHODL
8067969	-0,22	-1,16	0,45505	-1,23	-2,35	0,02686	NM_024944	MNS1
7989146	-0,22	-1,16	0,23955	0,73	1,66	0,34055	NM_018365	ALDH5A1
8117207	-0,22	-1,16	0,27083	1,66	3,17	0,00140	NM_170740	ABHD1
8040815	-0,22	-1,16	0,42453	1,57	2,96	0,00535	NM_032604	CIC
8029193	-0,22	-1,16	0,55116	0,51	1,43	0,30402	NM_015125	SEMA4F
8042925	-0,22	-1,16	0,57257	0,06	1,04	0,84942	NM_004263	RXFP2
7968389	-0,22	-1,16	0,33829	-0,86	-1,82	0,02591	NM_130806	---
8110461	-0,22	-1,16	0,66303	-1,10	-2,15	0,01833	---	TAP2
8125483	-0,22	-1,16	0,13908	1,24	2,36	0,01437	NM_000544	ZNF70
8074931	-0,22	-1,16	0,42918	0,13	1,09	0,74649	NM_021916	COMMD5
8153911	-0,22	-1,16	0,43555	1,04	2,05	0,04806	NM_014066	IGSF22
7947052	-0,22	-1,16	0,50325	-0,38	-1,30	0,23931	NM_173588	LGI2
8099685	-0,22	-1,16	0,31066	0,98	1,97	0,05427	NM_018176	TUB
7938269	-0,22	-1,16	0,59953	0,30	1,23	0,17440	NM_003320	GMEB2
8067709	-0,22	-1,16	0,44204	0,04	1,03	0,91884	NM_012384	KIRREL3
7952577	-0,22	-1,16	0,37567	-1,31	-2,47	0,02087	NM_032531	C3orf35
8078605	-0,22	-1,16	0,48982	-0,62	-1,54	0,02567	NM_178339	C1orf122
7900192	-0,22	-1,16	0,55506	-0,31	-1,24	0,56852	NM_198446	C3orf21
8092922	-0,22	-1,16	0,56786	-0,03	-1,02	0,77363	NM_152531	MGAT2
7974207	-0,22	-1,16	0,53955	1,80	3,47	0,00703	NM_002408	LOC100287290
8083652	-0,22	-1,16	0,01447	0,02	1,01	0,92461	AY070437	---
8113689	-0,22	-1,16	0,35626	-0,42	-1,34	0,05121	---	EVI2A
8014066	-0,22	-1,16	0,00374	-0,96	-1,95	0,01900	NM_001003927	ACAD10
7958761	-0,22	-1,16	0,40454	0,77	1,70	0,22223	NM_001136538	TTYH3
8131179	-0,22	-1,16	0,73802	1,62	3,08	0,04687	NM_025250	CCDC8
8037762	-0,22	-1,16	0,52963	-0,69	-1,62	0,07295	NM_032040	C8orf38
8147424	-0,22	-1,16	0,37338	0,66	1,58	0,08658	NM_152416	C21orf122
8070863	-0,22	-1,17	0,53002	-0,79	-1,73	0,03487	NR_027292	---
7913799	-0,22	-1,17	0,50111	-1,38	-2,60	0,00158	---	FAM92B
8003193	-0,22	-1,17	0,40258	-1,18	-2,26	0,01373	BC093665	ZSCAN23
8124583	-0,22	-1,17	0,57713	-0,54	-1,46	0,08900	NM_001012455	SIAH2
8091485	-0,22	-1,17	0,67722	-0,48	-1,40	0,17020	NM_005067	GKAP1
8162006	-0,22	-1,17	0,24221	1,15	2,22	0,16671	NM_025211	TCF20
8076436	-0,22	-1,17	0,70219	1,09	2,13	0,00479	NM_005650	ZNF551

8031807	-0,22	-1,17	0,67749	0,42	1,34	0,16577	NM_138347	ZNF540
8028266	-0,22	-1,17	0,15548	-0,79	-1,74	0,00567	NM_001172225	TCOF1
8109254	-0,22	-1,17	0,48768	0,38	1,30	0,25377	NM_001135243	RTDR1
8074869	-0,22	-1,17	0,33324	0,09	1,06	0,88316	NM_014433	---
8055668	-0,22	-1,17	0,37626	-0,62	-1,54	0,05549	---	RPL22P15
8054477	-0,22	-1,17	0,69419	-0,18	-1,13	0,25327	CR590757	---
7948308	-0,22	-1,17	0,72602	-0,96	-1,94	0,08728	---	ATP8A1
8100026	-0,22	-1,17	0,37462	0,11	1,08	0,73253	NM_006095	---
7972546	-0,22	-1,17	0,49549	1,22	2,33	0,00654	---	ST8SIA4
8113358	-0,22	-1,17	0,24448	-0,78	-1,72	0,00804	NM_005668	OR51B5
7946071	-0,22	-1,17	0,41068	-1,06	-2,09	0,01679	NM_001005567	NUDT16P1
8082663	-0,22	-1,17	0,58125	2,05	4,13	0,00571	NR_027766	IMMP1L
7947332	-0,22	-1,17	0,31771	1,61	3,05	0,04943	NM_144981	HLA-DOB
8178833	-0,22	-1,17	0,38385	0,21	1,16	0,56523	NM_002120	UBTD1
7929649	-0,22	-1,17	0,60758	0,09	1,07	0,84498	NM_024954	---
8133189	-0,22	-1,17	0,04873	-0,42	-1,34	0,00086	---	INS-IGF2
7945688	-0,22	-1,17	0,37185	-0,35	-1,27	0,19231	NR_003512	MIR137
7917944	-0,22	-1,17	0,32904	-1,45	-2,74	0,00222	NR_029679	ILF3
8025697	-0,22	-1,17	0,43869	0,81	1,75	0,27060	NM_012218	OR2Y1
8116482	-0,22	-1,17	0,38176	-1,11	-2,15	0,01024	NM_001001657	HHIPL1
7976669	-0,22	-1,17	0,09675	-0,27	-1,20	0,04833	NM_001127258	BCAR1
8002854	-0,22	-1,17	0,50362	0,64	1,55	0,03543	NM_001170717	GPR75
8052143	-0,22	-1,17	0,43814	-0,28	-1,21	0,09207	NM_006794	SPOCD1
7914467	-0,22	-1,17	0,51238	-0,36	-1,28	0,16207	NM_144569	ZNF831
8063723	-0,22	-1,17	0,49476	-0,92	-1,90	0,02033	NM_178457	LOC100131508
8127776	-0,22	-1,17	0,12800	-1,04	-2,05	0,00869	ENST00000431141	---
7906303	-0,22	-1,17	0,36844	-1,03	-2,04	0,00136	---	---
8161442	-0,22	-1,17	0,86519	-0,36	-1,29	0,67045	---	CENPBD1
8003553	-0,22	-1,17	0,40288	1,75	3,37	0,00290	NM_145039	MYH7B
8062064	-0,22	-1,17	0,07505	-0,79	-1,73	0,03741	NM_020884	---
8063442	-0,22	-1,17	0,53135	-0,51	-1,42	0,08185	---	---
8008596	-0,22	-1,17	0,49019	-0,28	-1,21	0,01227	---	TEX15
8150138	-0,22	-1,17	0,56779	1,19	2,29	0,21431	NM_031271	PLK4
8097356	-0,22	-1,17	0,28985	3,54	11,61	0,00012	NM_014264	---
8139919	-0,22	-1,17	0,40705	-0,56	-1,48	0,09191	---	NR1H3
7939751	-0,23	-1,17	0,54960	0,02	1,01	0,82847	NM_005693	---
8115164	-0,23	-1,17	0,45712	-0,65	-1,57	0,27735	---	ZNF69
8025968	-0,23	-1,17	0,78724	-1,67	-3,18	0,06532	NM_021915	CRTC2
7920382	-0,23	-1,17	0,69368	3,07	8,43	0,00030	NM_181715	---
7926706	-0,23	-1,17	0,34012	-0,22	-1,17	0,07629	---	C7orf45
8136181	-0,23	-1,17	0,63540	-0,43	-1,35	0,25975	NM_145268	PPARGC1A
8099633	-0,23	-1,17	0,53582	-1,41	-2,65	0,00074	NM_013261	---
8113421	-0,23	-1,17	0,33058	-0,28	-1,22	0,26613	---	SNORD94
8043276	-0,23	-1,17	0,57179	-0,60	-1,52	0,05990	NR_004378	---
7992458	-0,23	-1,17	0,59746	-0,15	-1,11	0,67215	---	OR52N2
7938059	-0,23	-1,17	0,34667	-0,85	-1,80	0,09295	NM_001005174	ZNF354C
8110491	-0,23	-1,17	0,63255	0,09	1,07	0,76937	NM_014594	GNAT1
8079919	-0,23	-1,17	0,01902	-0,85	-1,80	0,13240	NM_144499	HGS
8010638	-0,23	-1,17	0,71412	0,58	1,49	0,43193	NM_004712	HAS3
7996883	-0,23	-1,17	0,62604	1,05	2,07	0,02336	NM_005329	PSMD3
8006984	-0,23	-1,17	0,45678	0,74	1,67	0,09992	NM_002809	---
7989307	-0,23	-1,17	0,41944	-0,58	-1,50	0,01583	---	ASAH2
7933597	-0,23	-1,17	0,41013	-0,41	-1,32	0,28260	NM_019893	---
8123949	-0,23	-1,17	0,12576	-1,00	-2,00	0,00950	---	TAF1A
7924476	-0,23	-1,17	0,39847	1,80	3,48	0,00326	NM_005681	---
7896748	-0,23	-1,17	0,85072	-3,80	-13,95	0,00645	---	SATL1
8173862	-0,23	-1,17	0,22515	-0,82	-1,77	0,02636	NM_001012980	INPP5J
8072436	-0,23	-1,17	0,49390	0,19	1,14	0,23856	NM_001002837	YIPF2
8034108	-0,23	-1,17	0,58983	1,74	3,33	0,07077	NM_024029	EPHA5
8100578	-0,23	-1,17	0,20850	-0,52	-1,43	0,10648	NM_004439	CES4
7995729	-0,23	-1,17	0,59089	-0,61	-1,52	0,00589	NR_003276	LOC645181
8106769	-0,23	-1,17	0,62306	0,26	1,20	0,48757	XR_038419	PLGLA
8044149	-0,23	-1,17	0,32692	0,05	1,04	0,77562	NR_003506	CCNB3
8167625	-0,23	-1,17	0,62990	0,74	1,68	0,05542	NM_033031	DDC
8139640	-0,23	-1,17	0,49909	-1,67	-3,17	0,00091	NM_000790	LSM14B
8063835	-0,23	-1,17	0,28698	1,05	2,07	0,05253	NM_144703	TRIM66
7946380	-0,23	-1,17	0,54411	-0,19	-1,14	0,35029	NM_014818	GAP43
8081810	-0,23	-1,17	0,72958	-0,47	-1,39	0,04673	NM_001130064	RASAL1
7966542	-0,23	-1,17	0,50986	-0,73	-1,66	0,19763	NM_004658	ZNF396
8022892	-0,23	-1,17	0,46400	-0,41	-1,33	0,18904	NM_145756	RAB20
7972805	-0,23	-1,17	0,54563	0,24	1,18	0,03902	NM_017817	PCID2
7972888	-0,23	-1,17	0,55962	-0,11	-1,08	0,47175	BC008975	CHCHD6
8082305	-0,23	-1,17	0,44686	0,22	1,16	0,31244	NM_032343	PPP4R4

7976451	-0,23	-1,17	0,36703	0,46	1,37	0,16116	NM_058237	THBS4
8106573	-0,23	-1,17	0,04416	-0,79	-1,73	0,01310	NM_003248	C7orf33
8137050	-0,23	-1,17	0,41636	-0,68	-1,61	0,00832	BC021251	LRRC8C
7902874	-0,23	-1,17	0,37392	0,35	1,28	0,10325	NM_032270	OR5M3
7948133	-0,23	-1,17	0,74879	-0,77	-1,71	0,14922	NM_001004742	G0S2
7909441	-0,23	-1,17	0,68739	0,17	1,13	0,74813	NM_015714	ABP1
8137271	-0,23	-1,17	0,22527	-0,51	-1,42	0,10046	NM_001091	MECOM
8091972	-0,23	-1,17	0,35970	0,38	1,30	0,04188	NM_001105077	FAM200A
8141311	-0,23	-1,17	0,27678	0,17	1,12	0,55104	NM_145111	KIF2C
7901010	-0,23	-1,17	0,47093	1,48	2,80	0,00232	NM_006845	CCL23
8014361	-0,23	-1,17	0,60905	-1,20	-2,30	0,00031	NM_005064	LOC222699
8124551	-0,23	-1,17	0,78805	1,43	2,70	0,01599	NR_002936	---
8106720	-0,23	-1,17	0,49268	-0,38	-1,30	0,29147	---	POLE3
8163525	-0,23	-1,17	0,27409	1,50	2,83	0,02306	NM_017443	RBM47
8099967	-0,23	-1,17	0,55609	1,53	2,89	0,00231	NM_001098634	G6PC
8007429	-0,23	-1,17	0,47830	-0,88	-1,84	0,00405	NM_000151	RNF128
8169174	-0,23	-1,17	0,53694	0,56	1,48	0,14404	NM_024539	GAB3
8176163	-0,23	-1,17	0,18358	-0,39	-1,31	0,23215	NM_001081573	ZNF101
8027233	-0,23	-1,17	0,35217	0,86	1,82	0,16055	NM_033204	MYST1
7995055	-0,23	-1,17	0,66482	0,90	1,87	0,02392	NM_182958	OR9H1P
7911215	-0,23	-1,17	0,22709	-1,31	-2,48	0,01362	AY358215	IL18BP
7942300	-0,23	-1,17	0,04127	-0,50	-1,41	0,06644	NM_173042	GATS
8141477	-0,23	-1,17	0,63635	0,92	1,89	0,00265	NR_028038	RAPGEF1
8164665	-0,23	-1,18	0,17393	2,68	6,43	0,00844	NM_005312	CNOT3
8031122	-0,23	-1,18	0,49518	1,20	2,29	0,00778	NM_014516	LOC646851
8076113	-0,23	-1,18	0,38002	0,37	1,30	0,39743	NM_001013647	RBP5
7960764	-0,23	-1,18	0,78674	-0,56	-1,47	0,12473	NM_031491	RMND5B
8110437	-0,23	-1,18	0,50522	1,36	2,56	0,03191	NM_022762	TMEM11
8013500	-0,23	-1,18	0,17996	0,27	1,20	0,62471	NM_003876	MIR29B2
7923976	-0,23	-1,18	0,03684	-0,69	-1,62	0,05575	NR_029518	---
8102979	-0,23	-1,18	0,66770	-1,59	-3,00	0,00006	---	LOC100130691
8056983	-0,23	-1,18	0,17178	0,84	1,80	0,04646	NR_026966	---
8169437	-0,23	-1,18	0,39305	-0,45	-1,37	0,05563	---	MTMR7
8149485	-0,23	-1,18	0,12194	-0,04	-1,03	0,69484	NM_004686	FANCC
8162514	-0,23	-1,18	0,58380	3,63	12,37	0,00007	NM_000136	11. Mrz
8111124	-0,23	-1,18	0,57168	-0,35	-1,28	0,10985	NM_001102562	SLC26A7
8147273	-0,23	-1,18	0,16983	-0,64	-1,56	0,17638	NM_052832	PGLYRP2
8035069	-0,23	-1,18	0,59690	-1,03	-2,04	0,00539	NM_052890	YRDC
7915084	-0,24	-1,18	0,74399	2,60	6,06	0,00430	NM_024640	SNORD24
8159004	-0,24	-1,18	0,52522	-0,25	-1,19	0,66549	NR_002447	---
7971860	-0,24	-1,18	0,51075	-0,64	-1,55	0,00264	---	---
7980350	-0,24	-1,18	0,51285	-0,99	-1,99	0,00099	---	CNGA1
8094936	-0,24	-1,18	0,69276	-0,57	-1,49	0,10053	NM_001142564	---
8097011	-0,24	-1,18	0,38446	0,39	1,31	0,35021	---	GRHL3
7898916	-0,24	-1,18	0,02937	-0,33	-1,25	0,26965	NM_198173	MDGA1
8126102	-0,24	-1,18	0,31212	-1,32	-2,50	0,01467	NM_153487	TGIF1
8019939	-0,24	-1,18	0,30226	0,03	1,02	0,88796	NM_170695	SNORD116-26
7982000	-0,24	-1,18	0,46052	-0,52	-1,43	0,18757	NR_003340	ZNF334
8066745	-0,24	-1,18	0,58013	-1,19	-2,28	0,01525	NM_199441	TTC7A
8041826	-0,24	-1,18	0,66246	0,67	1,59	0,03144	NM_020458	---
8122552	-0,24	-1,18	0,07328	-1,39	-2,62	0,03322	---	ACTR6
7957793	-0,24	-1,18	0,53879	0,64	1,56	0,16425	NM_022496	NKX2-2
8065334	-0,24	-1,18	0,52038	-0,85	-1,80	0,00362	NM_002509	PRSS8
8001007	-0,24	-1,18	0,72261	2,31	4,96	0,01646	NM_002773	PTPN20A
7933437	-0,24	-1,18	0,26327	1,66	3,16	0,00503	NM_001042389	DTNBP1
8124022	-0,24	-1,18	0,17348	-0,56	-1,47	0,07887	NM_183040	---
8055016	-0,24	-1,18	0,31702	-0,51	-1,42	0,03734	---	GBX1
8143885	-0,24	-1,18	0,43269	-0,42	-1,34	0,09725	NM_001098834	CNTNAP4
7997289	-0,24	-1,18	0,28604	-0,83	-1,78	0,06821	NM_033401	NLRP10
7946334	-0,24	-1,18	0,74421	-1,21	-2,32	0,01268	NM_176821	ZNHIT1
8135089	-0,24	-1,18	0,51353	-0,55	-1,47	0,08857	NM_006349	REPS2
8166243	-0,24	-1,18	0,32077	2,03	4,10	0,00062	NM_004726	C14orf174
7975971	-0,24	-1,18	0,36243	-0,50	-1,42	0,06418	NM_001010860	ING5
8049906	-0,24	-1,18	0,25817	0,47	1,39	0,03457	NM_032329	DDX4
8105384	-0,24	-1,18	0,44276	-0,30	-1,23	0,02375	NM_024415	---
7960861	-0,24	-1,18	0,43426	-1,68	-3,21	0,08095	---	HIC1
8003719	-0,24	-1,18	0,51021	-0,49	-1,40	0,42759	NM_001098202	ROCK1
8020382	-0,24	-1,18	0,41391	0,13	1,10	0,89494	NM_005406	---
7965478	-0,24	-1,18	0,71604	-1,41	-2,66	0,03884	---	C19orf45
8025169	-0,24	-1,18	0,57301	-0,29	-1,22	0,45202	NM_198534	ZDHHC18
7899253	-0,24	-1,18	0,66917	2,46	5,49	0,00551	NM_032283	ATG4D
8025659	-0,24	-1,18	0,55493	1,26	2,39	0,02733	NM_032885	SERF1A
8105949	-0,24	-1,18	0,50753	0,29	1,22	0,26825	NM_021967	SERF1A

8105997	-0,24	-1,18	0,50753	0,29	1,22	0,26825	NM_021967	SERF1A
8177658	-0,24	-1,18	0,50753	0,29	1,22	0,26825	NM_021967	LTF
8086607	-0,24	-1,18	0,64474	-1,40	-2,65	0,01381	NM_002343	RNF123
8079796	-0,24	-1,18	0,31589	0,45	1,37	0,15411	NM_022064	SCARNA7
8091778	-0,24	-1,18	0,64758	1,54	2,91	0,00664	NR_003001	NAGLU
8007250	-0,24	-1,18	0,45976	-0,05	-1,04	0,91902	NM_000263	BLID
7952309	-0,24	-1,18	0,59683	-0,88	-1,84	0,00731	NM_001001786	MRPL52
7973327	-0,24	-1,18	0,20630	0,22	1,17	0,24814	NM_181304	ATP13A1
8035737	-0,24	-1,18	0,35143	1,50	2,83	0,02295	NM_020410	---
7973865	-0,24	-1,18	0,28745	-1,82	-3,53	0,03327	---	TDGF1
8079422	-0,24	-1,18	0,39929	-0,59	-1,51	0,04286	NM_003212	FKBPL
8125316	-0,24	-1,18	0,61741	-0,99	-1,99	0,00919	NM_022110	FKBPL
8178749	-0,24	-1,18	0,61741	-0,99	-1,99	0,00919	NM_022110	---
8138487	-0,24	-1,18	0,72302	0,96	1,95	0,00903	---	CCDC144A
8013272	-0,24	-1,18	0,16920	0,57	1,49	0,25357	NM_014695	ULBP1
8122724	-0,24	-1,18	0,60493	1,98	3,95	0,00059	NM_025218	CASKIN2
8018379	-0,24	-1,18	0,51332	0,27	1,20	0,41981	NM_020753	C9orf152
8163181	-0,24	-1,18	0,36176	-1,05	-2,07	0,02490	NM_001012993	SPOCK2
7934215	-0,24	-1,18	0,57993	-0,40	-1,32	0,06470	NM_014767	---
8097036	-0,24	-1,18	0,59360	-0,51	-1,42	0,00001	---	ERBB2
8006906	-0,24	-1,18	0,48329	2,03	4,07	0,00624	NM_001005862	NUDT15
7968999	-0,24	-1,18	0,10333	-0,37	-1,30	0,21970	NM_018283	DUSP8
7945641	-0,24	-1,18	0,69430	0,07	1,05	0,90628	NM_004420	---
8100456	-0,24	-1,18	0,70281	0,06	1,04	0,88414	---	SPARCL1
8101659	-0,24	-1,18	0,42495	-0,93	-1,90	0,07962	NM_001128310	NCBP2L
8169247	-0,24	-1,18	0,17278	-0,45	-1,36	0,12972	ENST00000372379	C15orf58
7985959	-0,24	-1,18	0,64668	0,79	1,73	0,00939	NM_001013657	PTCH2
7915612	-0,24	-1,18	0,37314	-0,29	-1,22	0,10039	NM_001166292	---
8166817	-0,24	-1,18	0,27711	-0,56	-1,47	0,09231	---	tcag7.907
8097118	-0,24	-1,18	0,75879	-1,31	-2,49	0,20291	ENST00000415418	---
7972018	-0,24	-1,18	0,61406	-1,26	-2,39	0,00350	---	SLC2A11
8071783	-0,24	-1,18	0,63174	0,35	1,28	0,20972	NM_030807	TSEN2
8077914	-0,24	-1,18	0,07114	0,63	1,54	0,00971	NM_025265	GPM6A
8103789	-0,24	-1,18	0,45400	-0,11	-1,08	0,43154	NM_005277	ATP10B
8115623	-0,24	-1,18	0,38703	-0,54	-1,45	0,04732	NM_025153	---
8055664	-0,24	-1,18	0,28911	-0,66	-1,58	0,12371	---	CCDC149
8099670	-0,24	-1,18	0,23001	0,50	1,41	0,05914	NM_001130726	---
8022557	-0,24	-1,18	0,30455	-1,29	-2,44	0,03671	---	DAAM2
8119357	-0,24	-1,18	0,53146	-0,51	-1,42	0,02498	NM_015345	IMMP2L
8142332	-0,24	-1,18	0,29392	-0,35	-1,28	0,02017	NM_032549	---
8051666	-0,24	-1,18	0,51027	-0,88	-1,84	0,01178	---	EXOSC7
8079294	-0,24	-1,18	0,46025	1,38	2,61	0,00534	NM_015004	CARD6
8105077	-0,24	-1,18	0,27116	-0,03	-1,02	0,89206	NM_032587	SNORA74A
8108420	-0,24	-1,18	0,41705	-0,14	-1,10	0,54012	NR_002915	OR51V1
7946031	-0,24	-1,18	0,79980	-2,55	-5,85	0,00001	NM_001004760	---
8036025	-0,24	-1,18	0,21035	-1,12	-2,17	0,00869	---	PSG11
8037259	-0,24	-1,18	0,57222	-0,78	-1,71	0,06169	NM_203287	C1orf227
7924180	-0,24	-1,18	0,52307	-0,30	-1,23	0,02352	NM_001024601	PRR21
8060082	-0,24	-1,18	0,67223	-0,66	-1,58	0,06667	NM_001080835	LRP4
7947744	-0,24	-1,18	0,25992	0,41	1,33	0,45930	NM_002334	TRPM8
8049394	-0,25	-1,19	0,35064	-0,45	-1,36	0,22074	NM_024080	SLCO2B1
7942569	-0,25	-1,19	0,55903	-0,41	-1,32	0,03580	NM_001145211	---
8099438	-0,25	-1,19	0,65925	-1,78	-3,44	0,00016	---	NTHL1
7998692	-0,25	-1,19	0,18402	0,24	1,18	0,40410	NM_002528	ANO8
8035278	-0,25	-1,19	0,66406	0,69	1,61	0,23653	NM_020959	HIST1H3J
8124537	-0,25	-1,19	0,59015	0,09	1,07	0,35916	NM_003535	LSM4
8035449	-0,25	-1,19	0,70015	-0,32	-1,25	0,62488	NM_012321	TRIM75
8098175	-0,25	-1,19	0,31438	0,02	1,02	0,85458	ENST00000322402	---
8023059	-0,25	-1,19	0,51580	-0,10	-1,07	0,79401	---	PCDHB4
8108693	-0,25	-1,19	0,33136	-0,88	-1,84	0,08525	NM_018938	---
7922400	-0,25	-1,19	0,30233	-0,03	-1,02	0,94865	---	SDR39U1
7978335	-0,25	-1,19	0,51898	0,81	1,76	0,00843	NM_020195	---
8058201	-0,25	-1,19	0,77947	-1,42	-2,68	0,01156	---	SNORA38
8118207	-0,25	-1,19	0,40012	-0,40	-1,32	0,33842	NR_002971	RPL13P5
7953516	-0,25	-1,19	0,42801	0,96	1,95	0,01273	NR_002803	MOB2
7945631	-0,25	-1,19	0,24728	-0,65	-1,57	0,19315	NM_001172223	LOC643659
8065278	-0,25	-1,19	0,37272	-0,84	-1,78	0,12768	AK056971	BCL2L14
7953993	-0,25	-1,19	0,33072	-0,82	-1,77	0,15000	NM_030766	EPS8L2
7937443	-0,25	-1,19	0,16083	0,85	1,81	0,12861	NM_022772	C11orf85
7949327	-0,25	-1,19	0,38140	-0,91	-1,88	0,00158	BC106951	OR51L1
7937984	-0,25	-1,19	0,58148	-1,39	-2,62	0,00176	NM_001004755	---
8054870	-0,25	-1,19	0,83041	-0,98	-1,97	0,04229	---	PPRC1
7929990	-0,25	-1,19	0,31782	2,58	5,97	0,01772	NM_015062	IL24

7909271	-0,25	-1,19	0,26559	-0,14	-1,10	0,55469	NM_006850	NEK10
8085867	-0,25	-1,19	0,03642	-0,74	-1,67	0,00001	NM_199347	---
8162996	-0,25	-1,19	0,06888	-1,19	-2,27	0,02695	--- ---	
8062284	-0,25	-1,19	0,72251	-1,26	-2,39	0,03373	--- ---	
7971239	-0,25	-1,19	0,10426	-0,24	-1,18	0,50709	--- DDR1	
8117900	-0,25	-1,19	0,62220	1,30	2,47	0,05441	NM_013993	POLRMT
8032057	-0,25	-1,19	0,48107	1,07	2,10	0,00171	NM_005035	C8A
7901732	-0,25	-1,19	0,32703	-0,71	-1,64	0,06382	NM_000562	CYP2J2
7916616	-0,25	-1,19	0,43630	1,03	2,05	0,08257	NM_000775	GRIK4
7944603	-0,25	-1,19	0,51263	-0,37	-1,29	0,00327	NM_014619	XCL1
7907156	-0,25	-1,19	0,57362	-0,61	-1,52	0,21377	NM_002995	RBM15B
8080093	-0,25	-1,19	0,57422	0,48	1,40	0,23461	NM_013286	GJD4
7933073	-0,25	-1,19	0,57596	-1,09	-2,14	0,00175	NM_153368	POLG2
8017621	-0,25	-1,19	0,20717	0,74	1,67	0,07903	NM_007215	TRPC6
7951178	-0,25	-1,19	0,43126	-1,58	-2,99	0,00118	NM_004621	SPRYD5
7943019	-0,25	-1,19	0,74406	-1,32	-2,49	0,00220	NM_032681	SPRYD5
7950977	-0,25	-1,19	0,74406	-1,32	-2,49	0,00220	NM_032681	GPR64
8171624	-0,25	-1,19	0,48526	1,22	2,32	0,00022	NM_001079858	TMSB4Y
8176644	-0,25	-1,19	0,27684	-0,84	-1,79	0,07354	NM_004202	PLAC8L1
8114856	-0,25	-1,19	0,37166	-0,13	-1,10	0,79913	NM_001029869	AGXT2
8111474	-0,25	-1,19	0,22159	-1,33	-2,51	0,02133	NM_031900	CEP250
8062137	-0,25	-1,19	0,18970	0,22	1,17	0,36173	NM_007186	IGK@
8053690	-0,25	-1,19	0,76099	-1,64	-3,12	0,09837	BC032451 WWOX	
7997352	-0,25	-1,19	0,46235	-0,55	-1,47	0,03962	NM_016373	PARK2
8130645	-0,25	-1,19	0,22280	-0,67	-1,60	0,01733	NM_004562	LOC388796
8066247	-0,25	-1,19	0,18362	3,17	9,01	0,00469	NR_027241	POLL
7935865	-0,25	-1,19	0,49767	1,44	2,71	0,02441	NM_001174084	SNORD4A
8005953	-0,25	-1,19	0,07641	0,47	1,39	0,19973	NR_000010	KIAA0195
8009796	-0,25	-1,19	0,33945	2,62	6,15	0,00198	NM_014738	OR5L2
7939948	-0,25	-1,19	0,37105	-0,60	-1,52	0,05529	NM_001004739	MCM10
7926259	-0,25	-1,19	0,44117	2,52	5,75	0,01584	NM_182751	FBLN2
8077970	-0,25	-1,19	0,37597	-1,31	-2,47	0,03414	NM_001004019	NEXN
7917151	-0,25	-1,19	0,51194	-0,42	-1,34	0,39763	ENST00000334785	C2orf84
8040541	-0,25	-1,19	0,62225	-0,72	-1,64	0,00625	BC050462 PDRG1	
8065596	-0,25	-1,19	0,58307	1,53	2,88	0,00064	NM_030815	POTEA
8146307	-0,25	-1,19	0,51976	-0,50	-1,41	0,12366	NM_001005365	KIAA1407
8089659	-0,25	-1,19	0,35057	0,26	1,20	0,52414	AK302488 CCDC85A	
8052376	-0,25	-1,19	0,63776	-1,17	-2,25	0,00064	NM_001080433	SLC22A18AS
7945774	-0,25	-1,19	0,26724	-0,38	-1,30	0,08419	NM_007105	CATSPERG
8028341	-0,25	-1,19	0,62199	-0,18	-1,14	0,16849	NM_021185	---
8154357	-0,25	-1,19	0,68904	0,76	1,69	0,27203	--- ANKRD1	
7934979	-0,25	-1,19	0,33449	2,17	4,51	0,00526	NM_014391	DOCK3
8080028	-0,25	-1,19	0,34859	1,05	2,07	0,00820	NM_004947	SSX4
8172415	-0,25	-1,19	0,71321	-1,38	-2,61	0,00106	NM_005636	LPXN
7948332	-0,25	-1,19	0,58641	0,17	1,13	0,69770	NM_004811	APEX2
8167854	-0,25	-1,19	0,29831	0,64	1,56	0,17184	NM_014481	KLF5
7969414	-0,26	-1,19	0,26034	0,38	1,30	0,30402	NM_001730	---
8162868	-0,26	-1,19	0,26591	-0,97	-1,96	0,01211	--- C2orf40	
8044143	-0,26	-1,19	0,52545	-1,07	-2,10	0,00150	NM_032411	SBF1
8077042	-0,26	-1,19	0,55148	1,11	2,16	0,08645	NM_002972	EVL
7976726	-0,26	-1,19	0,55079	0,90	1,86	0,14855	NM_016337	ATF3
7909610	-0,26	-1,19	0,34894	0,30	1,23	0,07342	NM_001040619	CYorf15A
8176698	-0,26	-1,19	0,61921	0,67	1,60	0,08437	NM_001005852	ARHGEF10L
7898483	-0,26	-1,19	0,40032	-0,49	-1,41	0,13703	NM_018125	TCEAL5
8174235	-0,26	-1,19	0,25716	-0,88	-1,84	0,15403	NM_001012979	---
7904448	-0,26	-1,19	0,65732	-0,43	-1,34	0,40765	--- MGC16025	
8060061	-0,26	-1,19	0,27762	-1,34	-2,53	0,01763	NR_026664	---
8059687	-0,26	-1,19	0,76684	-0,53	-1,45	0,51254	--- FLJ39639	
7992867	-0,26	-1,19	0,47337	-0,02	-1,01	0,95586	AK096958 C21orf33	
8069026	-0,26	-1,20	0,26685	1,18	2,27	0,00215	NM_004649	NLRP14
7938195	-0,26	-1,20	0,41588	-0,72	-1,64	0,00661	NM_176822	RASSF1
8087640	-0,26	-1,20	0,65243	0,05	1,04	0,80641	NM_007182	CSNK1G1
7989657	-0,26	-1,20	0,54669	-0,08	-1,06	0,74137	AK301858 KHNYN	
7973732	-0,26	-1,20	0,23688	0,17	1,12	0,35913	NM_015299	---
7921358	-0,26	-1,20	0,69864	-0,66	-1,59	0,26511	--- ---	
8134676	-0,26	-1,20	0,42730	-0,71	-1,64	0,01036	--- RBM44	
8049552	-0,26	-1,20	0,20846	-0,63	-1,55	0,02001	NM_001080504	TOMM7
8138547	-0,26	-1,20	0,35808	-0,01	-1,01	0,97788	NM_019059	HPS1
7935588	-0,26	-1,20	0,46496	-0,01	-1,00	0,99099	NM_000195	PIGF
8051993	-0,26	-1,20	0,76945	0,75	1,68	0,31449	NM_173074	SULT1C2
8044212	-0,26	-1,20	0,55972	-0,77	-1,71	0,07619	NM_001056	C17orf73
8016735	-0,26	-1,20	0,25697	-0,97	-1,96	0,04287	NR_024626	CIDEA
8020211	-0,26	-1,20	0,46118	-0,36	-1,28	0,16308	NM_001279	HIST1H4L

8124534	-0,26	-1,20	0,41474	0,29	1,22	0,48091	NM_003546	ARL13A
8168781	-0,26	-1,20	0,09094	-0,77	-1,71	0,07448	NM_001162490	SV2B
7986195	-0,26	-1,20	0,42732	-1,02	-2,03	0,00097	NM_014848	B9D1
8013331	-0,26	-1,20	0,05160	-0,30	-1,23	0,07711	NM_015681	HOXD10
8046536	-0,26	-1,20	0,58329	-0,57	-1,49	0,43877	NM_002148	IGSF3
7918913	-0,26	-1,20	0,18564	3,09	8,51	0,00065	NM_001542	BMP6
8116818	-0,26	-1,20	0,40737	-0,05	-1,03	0,82989	NM_001718	ZNF2
8043512	-0,26	-1,20	0,19013	0,05	1,04	0,85063	NM_021088	SLC45A4
8153262	-0,26	-1,20	0,55073	1,75	3,36	0,01253	NM_001080431	KGFLP2
8161478	-0,26	-1,20	0,54439	-0,09	-1,06	0,75261	NR_003670	SLC5A4
8075569	-0,26	-1,20	0,51049	-0,34	-1,27	0,18745	NM_014227	EYS
8127391	-0,26	-1,20	0,45950	1,71	3,28	0,03126	NM_001142800	ERP27
7961524	-0,26	-1,20	0,42355	-0,91	-1,88	0,03116	NM_152321	GPR137
7940959	-0,26	-1,20	0,34183	1,42	2,68	0,03384	NM_001170880	PYGM
7949124	-0,26	-1,20	0,26513	-0,56	-1,48	0,00454	NM_005609	BAT2L1
8164649	-0,26	-1,20	0,21333	-0,04	-1,03	0,90740	NM_013318	UGT2A1
8100791	-0,26	-1,20	0,50198	-1,45	-2,73	0,00990	NM_006798	HSF2BP
8070689	-0,26	-1,20	0,14365	0,84	1,79	0,02441	NM_007031	SLC17A9
8064014	-0,26	-1,20	0,69947	-1,29	-2,44	0,00737	NM_022082	OR4A15
7939918	-0,26	-1,20	0,58742	-1,31	-2,48	0,00135	NM_001005275	IL1F6
8044548	-0,26	-1,20	0,39711	-1,28	-2,43	0,00046	NM_014440	---
8074897	-0,26	-1,20	0,42854	-0,64	-1,56	0,00082	--- FAM18B2	
8012924	-0,26	-1,20	0,04364	-0,88	-1,84	0,04359	NM_145301	TRMT61B
8051226	-0,26	-1,20	0,61271	2,92	7,56	0,00202	NM_017910	---
7937037	-0,26	-1,20	0,59623	-1,27	-2,42	0,08655	--- ---	
8067303	-0,26	-1,20	0,51018	-0,26	-1,20	0,31458	--- TC2N	
7980891	-0,26	-1,20	0,62388	1,77	3,41	0,01343	NM_001128596	MLLT6
8006788	-0,26	-1,20	0,40530	0,39	1,31	0,41111	NM_005937	ZNF564
8034401	-0,26	-1,20	0,66297	0,69	1,61	0,13693	NM_144976	C21orf59
8069998	-0,26	-1,20	0,22239	1,95	3,85	0,00019	AF282851 MEIG1	
7926330	-0,26	-1,20	0,44805	-0,72	-1,65	0,00821	NM_001080836	OR1L4
8157686	-0,26	-1,20	0,26001	-0,54	-1,46	0,02387	NM_001005235	C21orf56
8070953	-0,26	-1,20	0,56739	0,20	1,15	0,29151	NM_001142854	---
7957549	-0,26	-1,20	0,62683	2,19	4,55	0,00606	--- LPHN1	
8034783	-0,26	-1,20	0,56017	2,46	5,50	0,00601	NM_001008701	NLGN2
8004385	-0,26	-1,20	0,44458	0,87	1,82	0,18907	NM_020795	---
7952984	-0,26	-1,20	0,15282	-0,98	-1,97	0,07780	--- OR7E5P	
7948113	-0,26	-1,20	0,83647	-1,16	-2,24	0,13074	NR_027688	ING2
8098508	-0,26	-1,20	0,72944	0,21	1,16	0,27662	NM_001564	LY6G5B
8118228	-0,26	-1,20	0,21989	0,56	1,48	0,08202	NM_021221	TMEM91
8029056	-0,26	-1,20	0,50621	0,35	1,27	0,44903	NM_001042595	BARD1
8058695	-0,26	-1,20	0,69895	1,60	3,04	0,00084	NM_000465	PTGR2
7975632	-0,26	-1,20	0,38482	1,46	2,75	0,00409	NM_152444	---
8039210	-0,26	-1,20	0,13177	-0,93	-1,91	0,01180	--- SCARNA9	
7943160	-0,26	-1,20	0,23429	-0,59	-1,50	0,07269	NR_002569	---
8136654	-0,26	-1,20	0,79772	-3,55	-11,70	0,00011	--- ---	
7973820	-0,26	-1,20	0,04612	-0,64	-1,55	0,01395	--- CDC42EP4	
8018107	-0,26	-1,20	0,24464	0,44	1,36	0,25918	NM_012121	MAP2K6
8009476	-0,26	-1,20	0,04684	0,18	1,14	0,43140	NM_002758	FLJ44838
8019751	-0,26	-1,20	0,60737	-0,63	-1,55	0,07486	AK126788 ITPKC	
8028908	-0,26	-1,20	0,40899	0,23	1,17	0,44896	NM_025194	LY6G5B
8178059	-0,26	-1,20	0,15638	0,96	1,94	0,08994	NM_021221	CPXM2
7936835	-0,27	-1,20	0,27155	-1,27	-2,42	0,04013	NM_198148	ZNF419
8031750	-0,27	-1,20	0,49740	-0,40	-1,32	0,17552	NM_001098491	---
7906433	-0,27	-1,20	0,55337	-2,67	-6,38	0,00222	--- OR1D5	
8011320	-0,27	-1,20	0,73136	-2,99	-7,92	0,00915	NM_014566	PPP1R8
7914139	-0,27	-1,20	0,70560	0,19	1,14	0,70606	NM_014110	POLR3H
8076344	-0,27	-1,20	0,42792	0,24	1,18	0,48212	NM_001018050	TERF2
8002289	-0,27	-1,20	0,28331	0,79	1,73	0,03716	NM_005652	PBLD
7933982	-0,27	-1,20	0,25910	-0,09	-1,07	0,29385	NM_022129	ARHGDIG
7991809	-0,27	-1,20	0,37077	-0,15	-1,11	0,73861	NM_001176	CCRL1
8082745	-0,27	-1,20	0,57071	-1,47	-2,77	0,00094	NM_178445	C1orf101
7911056	-0,27	-1,20	0,44723	0,46	1,38	0,10295	NM_001130957	HLA-DOB
8125470	-0,27	-1,20	0,32290	0,21	1,15	0,60779	NM_002120	FAM154A
8160278	-0,27	-1,20	0,09246	-0,45	-1,37	0,06112	AK292245 ---	
8172272	-0,27	-1,20	0,29261	-0,70	-1,63	0,00238	--- MOCS3	
8063427	-0,27	-1,20	0,49885	0,38	1,30	0,18930	NM_014484	PLSCR2
8091318	-0,27	-1,20	0,34523	-0,73	-1,66	0,03565	NM_020359	---
8101235	-0,27	-1,20	0,75384	-2,46	-5,51	0,04480	--- DPP6	
8137485	-0,27	-1,20	0,53314	-0,60	-1,51	0,00432	NM_001039350	CIT
7966878	-0,27	-1,20	0,44958	1,87	3,66	0,00511	NM_007174	CAMK1G
7909425	-0,27	-1,20	0,50187	-0,88	-1,83	0,02165	NM_020439	PLCL1
8047248	-0,27	-1,20	0,56474	-1,01	-2,01	0,03435	NM_006226	PATZ1

8075493	-0,27	-1,20	0,28233	1,25	2,37	0,02860	NM_014323	NOX1
8173979	-0,27	-1,20	0,24659	-0,73	-1,66	0,01087	NM_007052	MAEA
8093462	-0,27	-1,20	0,26859	1,04	2,06	0,08870	NM_001017405	MYOZ2
8097086	-0,27	-1,20	0,46705	-0,84	-1,79	0,00465	NM_016599	MIR96
8142882	-0,27	-1,20	0,31188	-0,36	-1,28	0,02228	NR_029512	SPTB
7979615	-0,27	-1,20	0,33118	-0,13	-1,10	0,65293	NM_001024858	CDKL4
8051696	-0,27	-1,20	0,09050	-1,06	-2,09	0,07935	NM_001009565	FLJ46010
8105731	-0,27	-1,20	0,13669	-1,07	-2,11	0,05723	AK127903 GRIP1	
7964759	-0,27	-1,20	0,49457	0,42	1,34	0,15271	NM_021150	TGIF2
8062319	-0,27	-1,20	0,32579	0,47	1,39	0,17373	NM_021809	---
7955076	-0,27	-1,20	0,68863	-0,53	-1,44	0,13121	--- FAM74A7	
8161407	-0,27	-1,20	0,72429	-1,43	-2,69	0,00159	ENST00000452734	RHO
8082552	-0,27	-1,20	0,62157	-1,57	-2,98	0,00011	NM_000539	ZNF19
8002547	-0,27	-1,20	0,29615	-0,47	-1,38	0,00742	NM_006961	RHBG
7906163	-0,27	-1,21	0,59702	-0,45	-1,37	0,24114	NM_020407	---
8112258	-0,27	-1,21	0,74157	-0,72	-1,65	0,14296	--- DENND4B	
7920354	-0,27	-1,21	0,53650	2,53	5,76	0,00810	NM_014856	PLXNB1
8086908	-0,27	-1,21	0,54145	0,78	1,71	0,04715	NM_001130082	DHX16
8124775	-0,27	-1,21	0,52808	2,75	6,75	0,00067	NM_003587	---
8058912	-0,27	-1,21	0,38650	-0,66	-1,58	0,02699	--- OR2AP1	
7956005	-0,27	-1,21	0,05849	-0,44	-1,36	0,00858	ENST00000321688	SETD1B
7959314	-0,27	-1,21	0,18432	0,83	1,78	0,16083	NM_015048	TCTEX1D1
7902158	-0,27	-1,21	0,58703	-1,05	-2,07	0,07001	NM_152665	---
8054412	-0,27	-1,21	0,58118	-0,53	-1,44	0,00601	--- C11orf2	
7941136	-0,27	-1,21	0,63744	1,75	3,35	0,00827	NM_013265	ADAM29
8098368	-0,27	-1,21	0,41677	-1,34	-2,54	0,00449	NM_014269	---
8043570	-0,27	-1,21	0,52352	-0,50	-1,41	0,02624	--- ELP1P	
8052594	-0,27	-1,21	0,31078	-0,99	-1,99	0,00328	AF229803 SPIC	
7957962	-0,27	-1,21	0,50967	-0,87	-1,82	0,28377	NM_152323	SPATC1
8148703	-0,27	-1,21	0,51916	-1,17	-2,26	0,00144	NM_198572	KIF4B
8109484	-0,27	-1,21	0,00886	0,42	1,34	0,39566	NM_001099293	DUS4L
8135410	-0,27	-1,21	0,41990	1,73	3,32	0,00386	NM_181581	CCDC82
7951157	-0,27	-1,21	0,34095	-1,51	-2,85	0,01648	AK313893 GPR56	
7996081	-0,27	-1,21	0,58073	1,88	3,69	0,01417	NM_201524	ALDOB
8162884	-0,27	-1,21	0,18334	-0,61	-1,53	0,02122	NM_000035	---
8151927	-0,27	-1,21	0,75786	-1,06	-2,08	0,09546	--- CES2	
7996345	-0,27	-1,21	0,34583	1,22	2,33	0,02433	NM_003869	DGCR2
8074349	-0,27	-1,21	0,35413	1,65	3,13	0,02327	NM_005137	---
8047852	-0,27	-1,21	0,36175	-0,83	-1,78	0,03224	--- C6orf162	
8121002	-0,27	-1,21	0,26647	0,78	1,72	0,02371	NM_001042493	THEM5
7920100	-0,27	-1,21	0,39621	-1,23	-2,34	0,00314	NM_182578	IQSEC1
8085412	-0,27	-1,21	0,36147	1,74	3,34	0,03309	NM_001134382	LMOD2
8135821	-0,27	-1,21	0,07636	-0,86	-1,82	0,05895	NM_207163	C9orf53
8154654	-0,27	-1,21	0,59838	-1,09	-2,13	0,03193	NR_024274	---
8008963	-0,27	-1,21	0,50197	-0,96	-1,94	0,05940	--- C12orf47	
7966441	-0,27	-1,21	0,39173	0,63	1,55	0,22838	NR_015404	CST5
8065421	-0,27	-1,21	0,49520	-0,22	-1,17	0,14376	NM_001900	SKIV2L
8179364	-0,27	-1,21	0,56588	1,54	2,91	0,00218	NM_006929	ITGA10
7904761	-0,27	-1,21	0,21638	-1,38	-2,60	0,05599	NM_003637	SH3TC2
8114991	-0,27	-1,21	0,54130	-1,69	-3,23	0,01906	NM_024577	SNRPG
8052861	-0,27	-1,21	0,49822	0,03	1,02	0,90530	NM_003096	PM20D2
8121087	-0,27	-1,21	0,29016	3,44	10,87	0,00053	NM_001010853	---
8081034	-0,27	-1,21	0,19053	-0,86	-1,82	0,06022	--- DYRK3	
7909225	-0,27	-1,21	0,44487	0,49	1,41	0,10752	NM_001004023	KGFLP2
8161458	-0,27	-1,21	0,66354	-0,34	-1,26	0,14124	NR_003670	---
8039769	-0,27	-1,21	0,45439	-1,17	-2,25	0,11314	--- MIR183	
8142884	-0,27	-1,21	0,02482	-0,28	-1,21	0,14309	NR_029615	---
8085118	-0,27	-1,21	0,67329	-0,92	-1,89	0,01431	--- FTSJ1	
8167270	-0,27	-1,21	0,26654	1,36	2,57	0,05251	NM_177439	WDR13
8167322	-0,27	-1,21	0,34464	1,01	2,01	0,04797	NM_017883	GLTSCR2
7986323	-0,27	-1,21	0,60718	0,26	1,20	0,69003	NM_015710	GOLGA6B
7985221	-0,27	-1,21	0,87134	1,34	2,54	0,13132	NM_018652	MIP
7964177	-0,27	-1,21	0,28180	-1,23	-2,35	0,00625	NM_012064	C12orf59
7953936	-0,27	-1,21	0,34287	-0,94	-1,92	0,00072	NM_153022	MUSTN1
8088047	-0,27	-1,21	0,30622	-0,32	-1,25	0,33603	NM_205853	---
7965110	-0,27	-1,21	0,36843	-0,98	-1,97	0,13831	--- ALK	
8051241	-0,27	-1,21	0,41753	-1,38	-2,60	0,00506	NM_004304	CYB5D2
8003850	-0,28	-1,21	0,64872	0,76	1,70	0,17089	NM_144611	IFT122
8082523	-0,28	-1,21	0,49704	0,01	1,00	0,98349	NM_052985	NFYB
8089459	-0,28	-1,21	0,18624	-1,00	-2,00	0,00182	NM_006166	CHKB-CPT1B
8077123	-0,28	-1,21	0,25391	0,77	1,70	0,05678	NR_027928	ASB9
8171392	-0,28	-1,21	0,18947	-0,24	-1,18	0,39796	NM_024087	MUC20
8084895	-0,28	-1,21	0,32786	-0,99	-1,99	0,05357	NM_152673	DHX35

8062576	-0,28	-1,21	0,56941	2,47	5,55	0,00001	NM_021931	CYP4Z1
7901287	-0,28	-1,21	0,67172	-0,99	-1,99	0,01171	NM_178134	TTLL9
8061620	-0,28	-1,21	0,21645	-0,48	-1,39	0,30980	NM_001008409	FOLH1B
7942998	-0,28	-1,21	0,02049	-0,88	-1,84	0,02429	NM_153696	LOC554206
7994235	-0,28	-1,21	0,19817	0,68	1,60	0,02962	ENST00000426719	NINJ1
8162455	-0,28	-1,21	0,49544	1,91	3,75	0,00228	NM_004148	TIRAP
7945086	-0,28	-1,21	0,35621	0,70	1,62	0,07921	NM_001039661	CCDC21
7899134	-0,28	-1,21	0,36348	2,18	4,54	0,00031	NM_022778	DNAH5
8111019	-0,28	-1,21	0,07481	-0,39	-1,31	0,14933	NM_001369	PCBD2
8108174	-0,28	-1,21	0,33682	0,06	1,04	0,84241	NM_032151	ALDH3B2
7949882	-0,28	-1,21	0,49435	-0,98	-1,98	0,00850	NM_000695	---
8148313	-0,28	-1,21	0,16892	-0,71	-1,63	0,01642	--- SPZ1	
8106597	-0,28	-1,21	0,33609	-0,15	-1,11	0,09245	NM_032567	DDR1
8179184	-0,28	-1,21	0,57143	1,53	2,90	0,02975	NM_013993	IGKC
8043459	-0,28	-1,21	0,67970	-2,52	-5,74	0,00018	ENST00000390273	CASP9
7912646	-0,28	-1,21	0,70336	1,74	3,34	0,01648	NM_001229	DTYMK
8060286	-0,28	-1,21	0,32879	1,25	2,38	0,00784	NM_012145	DTYMK
8077262	-0,28	-1,21	0,32879	1,25	2,38	0,00784	NM_012145	TTF1
8164729	-0,28	-1,21	0,03482	-0,16	-1,12	0,63012	NM_007344	TRGV3
8139121	-0,28	-1,21	0,62116	-1,46	-2,76	0,10884	ENST00000390346	ZNF677
8039070	-0,28	-1,21	0,53205	0,79	1,73	0,08761	NM_182609	TARP
8139125	-0,28	-1,21	0,29369	-1,71	-3,28	0,06084	BC030554 JMJD1C	
7927784	-0,28	-1,21	0,65099	-1,06	-2,08	0,04805	NM_032776	FLJ25328
8026503	-0,28	-1,21	0,60790	-0,97	-1,96	0,08629	NR_024335	SETD4
8070215	-0,28	-1,21	0,51369	0,17	1,13	0,55021	NM_017438	---
7991465	-0,28	-1,21	0,75430	-1,30	-2,46	0,00358	--- OR8D1	
7952392	-0,28	-1,21	0,40321	-1,14	-2,20	0,01186	NM_001002917	DAPK3
8032718	-0,28	-1,21	0,47732	2,16	4,48	0,00544	NM_001348	VTRNA1-3
8108631	-0,28	-1,21	0,49565	-0,52	-1,43	0,23595	NR_026705	PLD6
8013068	-0,28	-1,21	0,46517	-0,56	-1,47	0,13236	NM_178836	---
8056792	-0,28	-1,21	0,80684	0,33	1,26	0,69384	--- MFSD4	
7909127	-0,28	-1,21	0,30858	0,00	-1,00	0,99597	NM_181644	RSPH1
8070603	-0,28	-1,21	0,34721	-0,47	-1,39	0,00639	NM_080860	SYBU
8152355	-0,28	-1,21	0,60214	0,50	1,41	0,24168	NM_001099750	AMELY
8177061	-0,28	-1,21	0,51138	-1,19	-2,29	0,02291	NM_001143	---
8069448	-0,28	-1,21	0,74774	0,09	1,07	0,87113	--- CDRT1	
8012928	-0,28	-1,21	0,09833	-0,64	-1,56	0,16029	AF337810 C21orf105	
8070629	-0,28	-1,21	0,04977	-0,82	-1,77	0,00133	BC005107 FAM128A	
8055287	-0,28	-1,21	0,70501	0,75	1,68	0,11661	NM_001085365	---
8107198	-0,28	-1,21	0,52402	-1,12	-2,18	0,05470	--- DOCK6	
8034151	-0,28	-1,21	0,50675	1,97	3,91	0,00419	NM_020812	ART3
8095806	-0,28	-1,21	0,55410	-0,90	-1,86	0,12658	NM_001130016	ADCY7
7995492	-0,28	-1,21	0,19276	1,66	3,16	0,02964	NM_001114	FMO6P
7907260	-0,28	-1,21	0,60202	-1,10	-2,14	0,00493	NR_002601	---
7948375	-0,28	-1,21	0,37873	-0,54	-1,46	0,00432	--- ---	
8043429	-0,28	-1,21	0,58545	-0,88	-1,84	0,03881	--- ---	
8053720	-0,28	-1,21	0,58545	-0,88	-1,84	0,03881	--- KIAA1024L	
8107847	-0,28	-1,21	0,47032	-1,08	-2,12	0,00191	ENST00000334562	---
7906950	-0,28	-1,22	0,14416	-1,30	-2,46	0,03817	--- CCDC158	
8101174	-0,28	-1,22	0,28968	-0,57	-1,48	0,01771	NM_001042784	USP35
7942798	-0,28	-1,22	0,38532	0,22	1,16	0,60317	NM_020798	SNORD117
8124940	-0,28	-1,22	0,39188	-0,82	-1,77	0,08730	NR_003140	PRPF40B
7955250	-0,28	-1,22	0,22296	-0,76	-1,69	0,21277	NM_012272	MDH1B
8058462	-0,28	-1,22	0,43094	-0,23	-1,17	0,29149	NM_001039845	SFXN5
8052979	-0,28	-1,22	0,38124	0,23	1,18	0,50594	NM_144579	PRKX
8171182	-0,28	-1,22	0,12689	0,43	1,35	0,21623	NM_005044	MUC6
7945595	-0,28	-1,22	0,34683	-1,32	-2,50	0,03621	NM_005961	OR5AS1
7939959	-0,28	-1,22	0,22505	-0,95	-1,94	0,01714	NM_001001921	CHRNB1
8004416	-0,28	-1,22	0,35360	4,31	19,77	0,00011	NM_000747	RXRB
8125638	-0,28	-1,22	0,66056	2,50	5,67	0,00077	NM_021976	CD96
8081564	-0,28	-1,22	0,16400	-1,42	-2,68	0,01584	NM_198196	TRPV3
8011354	-0,28	-1,22	0,18726	-0,15	-1,11	0,33354	NM_145068	---
8075160	-0,28	-1,22	0,56052	-0,50	-1,41	0,09381	--- OR10A7	
7955983	-0,28	-1,22	0,61746	-1,06	-2,09	0,00407	NM_001005280	---
8168079	-0,28	-1,22	0,77688	-1,18	-2,27	0,18390	--- LHFPL3	
8135262	-0,28	-1,22	0,34033	-0,70	-1,63	0,04493	NM_199000	SETBP1
8021047	-0,28	-1,22	0,36929	-2,09	-4,27	0,02715	NM_015559	PAGE2B
8167862	-0,28	-1,22	0,63980	-1,02	-2,02	0,03989	NM_001015038	---
7932786	-0,28	-1,22	0,59713	-2,07	-4,19	0,00132	--- DTX3	
7956539	-0,28	-1,22	0,49829	0,68	1,60	0,01483	NM_178502	SKIV2L
8118367	-0,28	-1,22	0,49423	1,47	2,78	0,00218	NM_006929	SKIV2L
8178136	-0,28	-1,22	0,49423	1,47	2,78	0,00218	NM_006929	---
7953934	-0,28	-1,22	0,33107	-0,87	-1,83	0,00223	--- NEIL2	

8144658	-0,28	-1,22	0,64241	0,88	1,85	0,09763	NM_145043	FAM86A
7999304	-0,28	-1,22	0,46025	1,09	2,13	0,13956	NM_201400	DPYS
8152264	-0,28	-1,22	0,58055	-1,26	-2,40	0,03362	NM_001385	CLDN17
8069790	-0,28	-1,22	0,56621	-1,43	-2,70	0,00357	NM_012131	SLC2A9
8099326	-0,28	-1,22	0,33104	-1,12	-2,17	0,00808	NM_020041	---
8017825	-0,28	-1,22	0,20367	-1,15	-2,22	0,02136	--- ZNF641	
7962794	-0,28	-1,22	0,27428	1,67	3,18	0,09270	NM_152320	CHCHD5
8044491	-0,28	-1,22	0,37259	-0,34	-1,27	0,14483	NM_032309	C2orf61
8052004	-0,28	-1,22	0,64132	-0,99	-1,99	0,00421	NM_173649	---
8114363	-0,28	-1,22	0,73764	-1,61	-3,06	0,23642	--- ENAM	
8095535	-0,29	-1,22	0,58400	-2,18	-4,52	0,00129	NM_031889	ZNF407
8021756	-0,29	-1,22	0,54696	0,27	1,21	0,55756	NM_017757	IFNA6
8160417	-0,29	-1,22	0,40947	-0,92	-1,89	0,00701	NM_021002	C9orf96
8159017	-0,29	-1,22	0,58492	-0,13	-1,09	0,51519	NM_153710	---
8047213	-0,29	-1,22	0,42012	-0,78	-1,71	0,00025	--- SLC10A5	
8151559	-0,29	-1,22	0,12944	3,56	11,76	0,00167	NM_001010893	FAM134B
8111136	-0,29	-1,22	0,02870	0,11	1,08	0,40667	NM_001034850	---
7962341	-0,29	-1,22	0,38681	-0,58	-1,49	0,00341	--- ZNF678	
7910198	-0,29	-1,22	0,49377	0,31	1,24	0,47998	NM_178549	WISP1
8148435	-0,29	-1,22	0,53583	-1,67	-3,18	0,00186	NM_003882	HLA-DQB1
8125461	-0,29	-1,22	0,41768	-0,46	-1,38	0,49189	AK097297 C4orf11	
8101359	-0,29	-1,22	0,58320	-1,19	-2,28	0,00281	ENST00000426551	APOF
7964142	-0,29	-1,22	0,50014	-0,73	-1,66	0,05136	NM_001638	---
8107131	-0,29	-1,22	0,40472	-1,94	-3,84	0,00516	--- GNL1	
8124742	-0,29	-1,22	0,24806	-0,06	-1,04	0,88231	NM_005275	SH3BP1
8075963	-0,29	-1,22	0,53286	-0,75	-1,69	0,06113	AY364241 ---	
8001455	-0,29	-1,22	0,58609	-1,67	-3,19	0,00012	--- SLC4A9	
8108483	-0,29	-1,22	0,48139	-0,29	-1,22	0,03981	NM_031467	KCNE4
8048749	-0,29	-1,22	0,55497	-1,25	-2,37	0,06396	NM_080671	CD163L1
7960771	-0,29	-1,22	0,35645	-0,85	-1,80	0,00030	NM_174941	KLC2
7941565	-0,29	-1,22	0,16232	0,80	1,74	0,15068	NM_022822	PDZD4
8175860	-0,29	-1,22	0,52360	-0,56	-1,47	0,28002	NM_032512	SLC1A5
8029854	-0,29	-1,22	0,67562	-0,44	-1,35	0,02274	NM_005628	XKRX
8173999	-0,29	-1,22	0,20703	-0,12	-1,09	0,22833	NM_212559	BPIL1
8061780	-0,29	-1,22	0,38290	-0,90	-1,87	0,00490	NM_025227	TBC1D10B
8000856	-0,29	-1,22	0,61241	1,36	2,56	0,00776	NM_015527	AQP7
8160663	-0,29	-1,22	0,61484	-1,34	-2,54	0,05978	NM_001170	ARAF2P
8133021	-0,29	-1,22	0,46855	-0,93	-1,91	0,01540	BC033982 ARAF2P	
8139804	-0,29	-1,22	0,46855	-0,93	-1,91	0,01540	BC033982 ---	
8042157	-0,29	-1,22	0,15462	-0,88	-1,84	0,01404	--- ART5	
7945882	-0,29	-1,22	0,30260	-1,18	-2,26	0,02955	NM_053017	TRIM41
8110649	-0,29	-1,22	0,27617	0,67	1,59	0,39458	NM_033549	VLDLR
8159850	-0,29	-1,22	0,31032	-1,19	-2,28	0,03413	NM_003383	H6PD
7897441	-0,29	-1,22	0,64021	1,95	3,86	0,01053	NM_004285	C11orf52
7943795	-0,29	-1,22	0,04163	0,35	1,28	0,25477	NM_080659	FUT6
8033043	-0,29	-1,22	0,27618	-0,86	-1,81	0,00004	NM_000150	FLJ46111
7938309	-0,29	-1,22	0,56935	-0,48	-1,39	0,20094	NR_027713	AWAT1
8168107	-0,29	-1,22	0,10880	-0,55	-1,47	0,12713	NM_001013579	GARNL3
8157976	-0,29	-1,22	0,22241	1,11	2,16	0,00611	NM_032293	---
8114843	-0,29	-1,22	0,21157	0,35	1,27	0,10359	--- MRPS28	
8151471	-0,29	-1,22	0,36277	1,40	2,64	0,01410	NM_014018	---
8085060	-0,29	-1,22	0,17004	-0,96	-1,95	0,02675	--- CRYM	
8000117	-0,29	-1,22	0,41043	-0,35	-1,27	0,47617	NM_001888	---
8174554	-0,29	-1,22	0,38130	-0,82	-1,76	0,11876	--- LPPR4	
7903214	-0,29	-1,22	0,27282	-1,68	-3,21	0,00002	NM_014839	SLC22A5
8107920	-0,29	-1,22	0,53395	2,33	5,04	0,00609	NM_003060	GFI1B
8158939	-0,29	-1,22	0,59914	-0,65	-1,57	0,06435	NM_004188	C4A
8118409	-0,29	-1,22	0,14903	-1,25	-2,38	0,02579	NM_007293	C4A
8118455	-0,29	-1,22	0,14903	-1,25	-2,38	0,02579	NM_007293	C4A
8179399	-0,29	-1,22	0,14903	-1,25	-2,38	0,02579	NM_007293	LEPREL2
7953450	-0,29	-1,22	0,40573	-0,87	-1,82	0,08669	NM_014262	SLC16A12
7934936	-0,29	-1,22	0,47810	-0,57	-1,48	0,08531	NM_213606	C5orf45
8116335	-0,29	-1,22	0,46362	0,94	1,92	0,03075	NM_016175	LOC284788
8065339	-0,29	-1,22	0,46066	-1,26	-2,40	0,14390	BC033532 NUDCD2	
8115666	-0,29	-1,22	0,47289	1,45	2,73	0,01875	NM_145266	PRAME
8074856	-0,29	-1,22	0,44402	3,33	10,03	0,00050	NM_206953	---
8160906	-0,29	-1,22	0,51120	-0,98	-1,98	0,12695	--- CEP63	
8082827	-0,29	-1,22	0,34856	-0,39	-1,31	0,04093	NM_025180	PEAR1
7906264	-0,29	-1,22	0,20410	1,82	3,53	0,00115	NM_001080471	SNRPA1
7991598	-0,29	-1,22	0,43958	-0,63	-1,55	0,05948	NM_003090	IDO1
8146092	-0,29	-1,22	0,47447	-1,05	-2,06	0,00292	NM_002164	RFX4
7958305	-0,29	-1,22	0,48262	-1,45	-2,73	0,00179	NM_213594	LOC729603
8123230	-0,29	-1,22	0,28624	-1,59	-3,01	0,00056	NR_003288	C1orf99

7908371	-0,29	-1,22	0,23472	-0,36	-1,28	0,15914	BC040856	---
8155081	-0,29	-1,22	0,22809	-0,89	-1,86	0,05774	---	CYHR1
8153823	-0,29	-1,23	0,56586	0,98	1,97	0,10988	NM_032687	---
8092312	-0,29	-1,23	0,22535	-0,58	-1,50	0,06019	---	LOC440313
7991577	-0,29	-1,23	0,60935	-0,70	-1,62	0,09031	AK127131	TXNDC2
8020123	-0,29	-1,23	0,55420	-0,72	-1,64	0,28196	NM_001098529	SNORD105
8025584	-0,29	-1,23	0,47385	-0,67	-1,59	0,04852	NR_004381	---
8130436	-0,29	-1,23	0,51305	-0,25	-1,19	0,59939	---	IL17RB
8080562	-0,29	-1,23	0,37694	0,98	1,98	0,05708	NM_018725	FLCN
8013071	-0,29	-1,23	0,58477	0,82	1,76	0,05482	NM_144997	LRRC18
7933484	-0,29	-1,23	0,62197	-1,63	-3,10	0,00023	NM_001006939	SNORD115-17
7982040	-0,29	-1,23	0,57472	-1,35	-2,55	0,00060	NR_003309	SNORD115-17
7982042	-0,29	-1,23	0,57472	-1,35	-2,55	0,00060	NR_003309	SNORD115-17
7982044	-0,29	-1,23	0,57472	-1,35	-2,55	0,00060	NR_003309	ACOT12
8112920	-0,29	-1,23	0,00170	-1,42	-2,67	0,00200	NM_130767	RPSAP52
7964733	-0,29	-1,23	0,47622	-0,83	-1,77	0,01988	NR_026825	---
7969364	-0,29	-1,23	0,09561	-1,03	-2,04	0,01288	---	CCR3
8079383	-0,29	-1,23	0,09501	-0,16	-1,12	0,34556	NM_001837	ZNF589
8079598	-0,29	-1,23	0,00558	0,72	1,65	0,00972	NM_016089	TM7SF4
8147830	-0,29	-1,23	0,34840	-0,92	-1,89	0,00631	NM_030788	TTC23L
8104812	-0,30	-1,23	0,30492	-0,14	-1,10	0,50684	NM_144725	ABCA2
8165345	-0,30	-1,23	0,48301	2,74	6,70	0,00560	NM_001606	MOBKL2A
8032410	-0,30	-1,23	0,34887	-0,07	-1,05	0,81934	NM_130807	CD24
8177222	-0,30	-1,23	0,21151	3,04	8,25	0,00122	NM_013230	ITFG1
8001233	-0,30	-1,23	0,10511	-0,54	-1,46	0,01053	AK309475	CECR5
8074227	-0,30	-1,23	0,26831	1,29	2,45	0,02333	NM_033070	RGS17
8130394	-0,30	-1,23	0,48565	0,11	1,08	0,71194	NM_012419	GPR116
8161418	-0,30	-1,23	0,74955	-1,85	-3,61	0,00449	NM_015234	DBR1
8090960	-0,30	-1,23	0,22487	0,48	1,40	0,18418	NM_016216	CPNE4
8090690	-0,30	-1,23	0,40446	-0,47	-1,38	0,33613	NM_130808	GPN2
7913978	-0,30	-1,23	0,17689	-0,17	-1,13	0,75782	NM_018066	C3orf65
8084627	-0,30	-1,23	0,47196	-0,34	-1,27	0,18092	NR_027317	SHISA6
8004955	-0,30	-1,23	0,54994	-0,94	-1,92	0,01205	NM_207386	C15orf38
7991367	-0,30	-1,23	0,66955	0,04	1,03	0,91664	NM_182616	OXNAD1
8078173	-0,30	-1,23	0,30473	2,29	4,89	0,00030	NM_138381	---
7998959	-0,30	-1,23	0,19640	-0,32	-1,25	0,18893	---	---
8014485	-0,30	-1,23	0,52316	-0,93	-1,90	0,18510	---	RHCE
7913835	-0,30	-1,23	0,25669	0,02	1,02	0,90450	NM_020485	MAN2C1
7990457	-0,30	-1,23	0,49017	0,44	1,35	0,48301	NM_006715	DMP1
8096274	-0,30	-1,23	0,35787	-1,32	-2,50	0,00038	NM_004407	COBRA1
8159654	-0,30	-1,23	0,52668	2,94	7,70	0,00400	NM_015456	PCDHB3
8108688	-0,30	-1,23	0,62221	-0,33	-1,26	0,50441	NM_018937	SEPX1
7998637	-0,30	-1,23	0,53076	2,91	7,53	0,00165	NM_016332	---
8144947	-0,30	-1,23	0,46439	-0,14	-1,11	0,26924	---	NR4A2
8055952	-0,30	-1,23	0,43844	3,11	8,63	0,00007	NM_006186	OR4A16
7939916	-0,30	-1,23	0,17481	-0,94	-1,91	0,01267	NM_001005274	---
7907847	-0,30	-1,23	0,56902	-1,06	-2,08	0,00059	---	CDKN2AIPNL
8114185	-0,30	-1,23	0,38407	-0,24	-1,18	0,45519	NM_080656	SNTN
8080863	-0,30	-1,23	0,33814	-0,41	-1,33	0,16058	NM_001080537	TNR
7922482	-0,30	-1,23	0,62548	-0,93	-1,90	0,13263	NM_003285	SNORD114-3
7976816	-0,30	-1,23	0,09238	-0,94	-1,92	0,00000	NR_003195	FAM87A
7896756	-0,30	-1,23	0,60044	-1,04	-2,05	0,02905	BC037297	---
7926934	-0,30	-1,23	0,61865	-0,76	-1,69	0,00078	---	SNORA71B
8066256	-0,30	-1,23	0,30638	-1,50	-2,82	0,01732	NR_002910	---
8153039	-0,30	-1,23	0,69888	-2,95	-7,74	0,00018	---	SLC24A5
7983553	-0,30	-1,23	0,61171	-0,99	-1,98	0,01215	NM_205850	SLC12A1
7983564	-0,30	-1,23	0,10507	-1,11	-2,16	0,00046	NM_000338	DDIT4L
8101952	-0,30	-1,23	0,34359	-0,67	-1,59	0,03011	NM_145244	LRRC20
7934145	-0,30	-1,23	0,29699	0,89	1,86	0,03834	NM_207119	ZG16B
7992732	-0,30	-1,23	0,51293	-1,56	-2,95	0,01565	NM_145252	MIR509-1
8175587	-0,30	-1,23	0,16623	-1,09	-2,12	0,00410	NR_030236	C4orf45
8103452	-0,30	-1,23	0,42289	-0,57	-1,49	0,00133	NM_152543	LOC100290146
8001104	-0,30	-1,23	0,80503	-3,25	-9,53	0,00211	AK289373	SNAP91
8127872	-0,30	-1,23	0,02977	-0,24	-1,18	0,36943	NR_026669	APOBEC1
7960821	-0,30	-1,23	0,53277	-1,18	-2,27	0,00263	NM_001644	PPFIA4
7908879	-0,30	-1,23	0,25046	-1,41	-2,65	0,00165	NM_015053	ERBB4
8058627	-0,30	-1,23	0,31461	-0,89	-1,86	0,02738	NM_005235	YY1
7976744	-0,30	-1,23	0,22015	-0,44	-1,35	0,12641	NM_003403	HIST1H4I
8117537	-0,30	-1,23	0,41729	-0,10	-1,07	0,57470	NM_003495	---
8075190	-0,30	-1,23	0,12419	-0,36	-1,28	0,09785	---	OR6Q1
7940108	-0,30	-1,23	0,17414	-0,58	-1,50	0,01726	NM_001005186	C1orf183
7918552	-0,30	-1,23	0,07455	-0,71	-1,64	0,02238	NM_019099	---
7976239	-0,30	-1,23	0,31569	-0,55	-1,46	0,07747	---	---

7929591	-0,30	-1,23	0,24809	-0,72	-1,65	0,04111	--- ADAMTS18	
8002941	-0,30	-1,23	0,40243	-1,32	-2,50	0,01822	NM_199355	LINGO2
8160546	-0,30	-1,23	0,64003	-0,88	-1,84	0,01764	NM_152570	ZNF20
8034334	-0,30	-1,23	0,48739	0,51	1,42	0,12102	NM_021143	DCUN1D2
7972912	-0,30	-1,23	0,45280	0,79	1,73	0,02356	NM_001014283	MEGF8
8029236	-0,30	-1,23	0,42409	0,41	1,32	0,33897	NM_001410	IGLJ3
7981730	-0,30	-1,23	0,85555	-4,22	-18,65	0,00014	AB001736 PSMC3	
7947867	-0,30	-1,23	0,37522	0,77	1,70	0,12517	NM_002804	---
8124086	-0,30	-1,23	0,55192	-0,73	-1,66	0,28234	--- C8orf86	
8150352	-0,30	-1,23	0,57934	-0,28	-1,21	0,11971	BC137511 OR2K2	
8163273	-0,30	-1,23	0,12933	0,01	1,01	0,97710	NM_205859	FBXW4P1
8071713	-0,30	-1,23	0,30646	-0,35	-1,28	0,22082	NR_033408	CCL8
8006453	-0,30	-1,23	0,61182	-0,91	-1,88	0,00097	NM_005623	---
8091099	-0,30	-1,23	0,08919	-0,37	-1,29	0,12124	--- SNORA40	
8045423	-0,30	-1,23	0,27746	-0,65	-1,56	0,06582	NR_002973	TCFL5
8067554	-0,30	-1,23	0,44382	0,84	1,79	0,07047	NM_006602	SNORD58A
8023259	-0,30	-1,23	0,24772	-0,70	-1,62	0,03824	NR_002571	CCL3
8014369	-0,30	-1,23	0,59209	-1,97	-3,91	0,00837	NM_002983	AREG
8095736	-0,30	-1,23	0,48032	4,48	22,35	0,00018	NM_001657	---
8082250	-0,30	-1,23	0,77508	-2,67	-6,38	0,00908	--- DALRD3	
8087236	-0,30	-1,23	0,30178	0,24	1,18	0,50037	NM_001009996	---
8131381	-0,30	-1,23	0,56791	-0,01	-1,01	0,94151	--- CCDC141	
8057377	-0,30	-1,24	0,18424	-0,65	-1,57	0,01683	NM_173648	---
7899517	-0,30	-1,24	0,15016	-0,92	-1,89	0,00950	--- IL31RA	
8105411	-0,30	-1,24	0,48131	1,96	3,90	0,00009	NM_139017	SERP2
7968892	-0,30	-1,24	0,58057	-1,07	-2,09	0,00094	NM_001010897	ASMT
8165773	-0,30	-1,24	0,36670	-0,15	-1,11	0,80553	NM_001171038	ASMT
8176342	-0,30	-1,24	0,36670	-0,15	-1,11	0,80553	NM_001171038	MIR410
7976856	-0,31	-1,24	0,04542	-0,59	-1,51	0,02941	NR_030156	SLC28A3
8162059	-0,31	-1,24	0,33298	-0,59	-1,51	0,06511	NM_022127	PCDHB5
8108697	-0,31	-1,24	0,26031	1,77	3,41	0,00144	NM_015669	OR7C2
8026388	-0,31	-1,24	0,43217	-1,23	-2,35	0,01909	NM_012377	TCEAL8
8174228	-0,31	-1,24	0,33851	-0,23	-1,18	0,51662	NM_153333	PABPC1P2
8045581	-0,31	-1,24	0,50017	0,39	1,31	0,04221	NR_026904	---
8006592	-0,31	-1,24	0,65632	-0,44	-1,36	0,09666	--- SLC38A3	
8079931	-0,31	-1,24	0,30923	-0,90	-1,87	0,06058	NM_006841	ZNF212
8137112	-0,31	-1,24	0,40709	0,04	1,03	0,84479	NM_012256	---
7967089	-0,31	-1,24	0,54971	-0,23	-1,17	0,56189	--- UBL3	
7970831	-0,31	-1,24	0,55512	0,64	1,55	0,02766	NM_007106	CSNK1G2
8024358	-0,31	-1,24	0,27607	1,84	3,57	0,00766	NM_001319	MARS2
8047243	-0,31	-1,24	0,27324	1,47	2,77	0,03626	NM_138395	SLC45A4
8153258	-0,31	-1,24	0,64165	1,10	2,15	0,39713	BC033223 NFATC2IP	
7994506	-0,31	-1,24	0,10063	0,90	1,86	0,12669	NM_032815	RNF186
7913206	-0,31	-1,24	0,41869	-1,29	-2,44	0,00364	NM_019062	LOC375010
7919139	-0,31	-1,24	0,45997	-0,11	-1,08	0,82380	AK090412 DEFB134	
8149350	-0,31	-1,24	0,32665	-0,36	-1,29	0,04749	NM_001033019	SPANXE
8175558	-0,31	-1,24	0,67161	-1,16	-2,24	0,19403	NM_145665	---
8099756	-0,31	-1,24	0,35494	-1,25	-2,38	0,00417	--- PABPC5	
8168650	-0,31	-1,24	0,47481	-0,88	-1,85	0,03160	NM_080832	PVRL1
7952274	-0,31	-1,24	0,29735	0,59	1,50	0,03384	NM_002855	KLRA1
7961198	-0,31	-1,24	0,20178	-0,48	-1,40	0,17030	NR_028045	ICA1
8138202	-0,31	-1,24	0,39827	1,52	2,86	0,02361	NM_004968	CIDEB
7978272	-0,31	-1,24	0,59655	-1,54	-2,92	0,00067	NM_014430	MAP2K5
7984408	-0,31	-1,24	0,44347	1,66	3,16	0,01022	NM_145160	---
8058855	-0,31	-1,24	0,59072	-0,54	-1,46	0,11202	--- ZSWIM5	
7915682	-0,31	-1,24	0,46398	1,04	2,05	0,05533	NM_020883	VKORC1L1
8133119	-0,31	-1,24	0,43522	-0,67	-1,59	0,18041	BC014249 CXCL16	
8011713	-0,31	-1,24	0,52458	3,62	12,33	0,00001	NM_022059	SMAD6
7984353	-0,31	-1,24	0,26060	0,34	1,27	0,50601	NR_027654	CHSY3
8107850	-0,31	-1,24	0,18081	-1,20	-2,30	0,00337	NM_175856	PDE6C
7929350	-0,31	-1,24	0,41659	-0,53	-1,45	0,03760	NM_006204	SPTA1
7921367	-0,31	-1,24	0,18733	-1,08	-2,11	0,00577	NM_003126	---
8131494	-0,31	-1,24	0,23236	-0,70	-1,63	0,07274	--- CHRNA1	
8056877	-0,31	-1,24	0,30707	-0,52	-1,44	0,15462	NM_001039523	NOC4L
7960068	-0,31	-1,24	0,02189	0,14	1,10	0,75151	NM_024078	BMP2
8060850	-0,31	-1,24	0,47901	-1,18	-2,27	0,02342	NM_001200	ZNF688
8000910	-0,31	-1,24	0,37190	-0,14	-1,10	0,29664	NM_145271	HNRNPA3P1
7933192	-0,31	-1,24	0,53762	-0,20	-1,15	0,60745	NR_002726	---
8174644	-0,31	-1,24	0,33693	-0,94	-1,92	0,11806	--- NT5DC4	
8044512	-0,31	-1,24	0,21755	-0,99	-1,99	0,02531	ENST00000327581	---
7985087	-0,31	-1,24	0,02178	-0,73	-1,66	0,00128	--- THNSL2	
8043393	-0,31	-1,24	0,58336	-0,55	-1,47	0,20096	NM_018271	ABCB6
8059111	-0,31	-1,24	0,38755	2,21	4,62	0,01221	NM_005689	NGFRAP1

8169028	-0,31	-1,24	0,45758	0,72	1,65	0,09117	NM_206917	LENG1
8039139	-0,31	-1,24	0,42863	-0,45	-1,37	0,23152	NM_024316	ILKAP
8059969	-0,31	-1,24	0,19086	0,15	1,11	0,52526	NM_030768	---
8094148	-0,31	-1,24	0,13954	0,23	1,17	0,17467	---	ZNF516
8023882	-0,31	-1,24	0,15802	0,19	1,14	0,45222	NM_014643	---
8089232	-0,31	-1,24	0,30185	-1,58	-2,99	0,00160	---	SLC26A11
8010405	-0,31	-1,24	0,15709	1,53	2,89	0,04306	NM_001166347	SEC14L2
8072328	-0,31	-1,24	0,41150	0,46	1,38	0,04293	NM_012429	ZNF705D
8144691	-0,31	-1,24	0,24515	-0,75	-1,68	0,00989	NM_001039615	---
7972426	-0,31	-1,24	0,51146	-1,33	-2,52	0,02736	---	---
8174887	-0,31	-1,24	0,12963	-0,59	-1,51	0,00359	---	HMGCLL1
8127180	-0,31	-1,24	0,44272	-1,53	-2,88	0,06269	NM_019036	P704P
8074170	-0,31	-1,24	0,54269	-1,99	-3,96	0,00554	NM_001145442	SERPINB8
8021653	-0,31	-1,24	0,06993	0,39	1,31	0,28004	NM_002640	TP53AIP1
7952631	-0,31	-1,24	0,33125	-0,95	-1,93	0,14864	NM_022112	CCDC15
7944913	-0,31	-1,24	0,21316	-0,44	-1,35	0,35538	NM_025004	APOBEC3D
8073072	-0,31	-1,24	0,50285	-0,60	-1,51	0,04287	NM_152426	TAS2R50
7961281	-0,31	-1,24	0,28465	-0,54	-1,46	0,21530	NM_176890	F11R
7921713	-0,31	-1,24	0,46216	4,25	19,03	0,00002	NM_016946	OR6M1
7952375	-0,31	-1,24	0,22460	-1,53	-2,89	0,01410	NM_001005325	C1QTNF3
8111443	-0,31	-1,24	0,37816	1,18	2,27	0,01411	NM_181435	B3GALT5
8068633	-0,31	-1,24	0,31584	-0,56	-1,48	0,03987	NM_033171	---
8053751	-0,31	-1,24	0,34221	-1,11	-2,16	0,06083	---	---
8161405	-0,31	-1,24	0,34221	-1,11	-2,16	0,06083	---	MIR136
7976806	-0,31	-1,24	0,43455	-0,60	-1,51	0,06783	NR_029699	LRRC66
8100312	-0,31	-1,24	0,24865	-0,72	-1,64	0,00972	NM_001024611	---
8111358	-0,31	-1,24	0,05578	-1,26	-2,39	0,01892	---	WDR73
7991126	-0,31	-1,24	0,51784	0,83	1,78	0,11858	NM_032856	ZNF80
8089694	-0,31	-1,24	0,25766	-0,85	-1,80	0,07670	NM_007136	IFITM5
7945368	-0,31	-1,24	0,61854	-0,89	-1,85	0,00261	NM_001025295	LOC154872
8142692	-0,31	-1,24	0,45388	-1,25	-2,39	0,00281	BC130604	C9orf135
8155747	-0,31	-1,24	0,28632	-1,55	-2,92	0,00145	NM_001010940	PLEC
8153568	-0,31	-1,24	0,06772	0,02	1,01	0,96770	NM_201384	SNORD115-2
7982010	-0,31	-1,24	0,04991	-0,59	-1,50	0,01272	NR_003294	KREMEN1
8072170	-0,31	-1,24	0,09943	1,34	2,54	0,01472	NM_032045	FMO5
7919314	-0,31	-1,24	0,16416	0,53	1,45	0,03650	NM_001461	KY
8090872	-0,31	-1,24	0,43931	-0,63	-1,55	0,07250	NM_178554	CYB5RL
7916393	-0,31	-1,24	0,11152	-0,53	-1,44	0,38894	NM_001031672	---
8009792	-0,31	-1,24	0,24647	-0,84	-1,79	0,13619	---	CCDC130
8026170	-0,31	-1,24	0,30077	1,47	2,77	0,02021	NM_030818	SNORD26
7948908	-0,31	-1,24	0,04743	0,23	1,17	0,07378	NR_002564	AG2
7939492	-0,31	-1,24	0,35594	-1,57	-2,96	0,00262	NM_001145033	URGCP
8139256	-0,31	-1,24	0,09500	2,12	4,36	0,02032	NM_017920	C3orf51
8088212	-0,31	-1,24	0,37165	-1,04	-2,05	0,09003	NR_024615	HIST1H3G
8124440	-0,31	-1,24	0,05271	0,19	1,14	0,66291	NM_003534	ENTPD1
7929511	-0,31	-1,24	0,09914	-1,68	-3,21	0,02432	NM_001776	PRMT2
8069450	-0,31	-1,24	0,64185	0,66	1,58	0,00921	NM_206962	CD160
7919243	-0,31	-1,24	0,28494	-0,93	-1,91	0,06235	NM_007053	GOLPH3
8111331	-0,31	-1,24	0,42435	0,87	1,83	0,00720	NM_022130	DEFB126
8060308	-0,31	-1,24	0,35952	-0,94	-1,91	0,00140	NM_030931	---
8053664	-0,31	-1,24	0,27659	-1,49	-2,81	0,00267	---	TRPC3
8102678	-0,31	-1,24	0,50165	-1,21	-2,32	0,02361	NM_001130698	CYSLTR1
8173745	-0,31	-1,24	0,63601	-0,49	-1,40	0,03318	NM_006639	LOC100129884
7971965	-0,32	-1,24	0,41237	-0,31	-1,24	0,15558	AK097079	---
8118319	-0,32	-1,24	0,21992	0,45	1,36	0,06952	---	PTTG3P
8151123	-0,32	-1,24	0,02321	-0,25	-1,19	0,21725	NR_002734	CEP152
7988537	-0,32	-1,24	0,28486	1,52	2,87	0,03605	NM_014985	LOC646513
8072353	-0,32	-1,24	0,30405	-0,59	-1,50	0,00070	AY358802	ARL4D
8007493	-0,32	-1,24	0,29561	0,38	1,30	0,37311	NM_001661	HOXA3
8138721	-0,32	-1,24	0,49812	0,69	1,62	0,02166	NM_153631	LY75
8056113	-0,32	-1,25	0,12827	-1,19	-2,28	0,00102	NM_002349	---
8097028	-0,32	-1,25	0,49711	-0,99	-1,98	0,01233	---	SYT7
7948588	-0,32	-1,25	0,03574	0,75	1,68	0,06831	NM_004200	CD1D
7906330	-0,32	-1,25	0,39568	-0,71	-1,64	0,00026	NM_001766	FAM72D
7919591	-0,32	-1,25	0,71316	2,97	7,81	0,00067	AB096683	DSPP
8096265	-0,32	-1,25	0,09936	-0,63	-1,54	0,02585	NM_014208	ZNF432
8038942	-0,32	-1,25	0,22018	-0,15	-1,11	0,20424	NM_014650	GLIPR1L1
7957245	-0,32	-1,25	0,09158	-0,36	-1,29	0,05014	NM_152779	MIR19B2
8175250	-0,32	-1,25	0,04227	-0,51	-1,43	0,11535	NR_029491	RBM14
7941707	-0,32	-1,25	0,09778	-0,58	-1,49	0,32771	NM_006328	TRIOBP
8072894	-0,32	-1,25	0,12930	0,63	1,54	0,09815	NM_001039141	SYT14L
8100699	-0,32	-1,25	0,11699	-1,07	-2,10	0,01889	NR_027094	LUZP2
7939007	-0,32	-1,25	0,26602	-0,99	-1,99	0,01198	NM_001009909	GOLGA6L5

7985472	-0,32	-1,25	0,70584	-0,13	-1,09	0,71770	NR_003246	C14orf143
7980690	-0,32	-1,25	0,04469	-0,08	-1,06	0,74336	NM_145231	---
8094499	-0,32	-1,25	0,41559	-0,87	-1,83	0,12507	---	---
8056570	-0,32	-1,25	0,52846	-0,98	-1,97	0,00159	---	---
8167893	-0,32	-1,25	0,70413	-1,60	-3,02	0,00129	---	DVL2
8012079	-0,32	-1,25	0,13626	1,12	2,18	0,01534	NM_004422	C11orf65
7951521	-0,32	-1,25	0,07653	-0,95	-1,93	0,04274	NM_152587	---
7966266	-0,32	-1,25	0,46846	-1,36	-2,57	0,01779	---	---
8079375	-0,32	-1,25	0,32677	-1,01	-2,01	0,01630	---	PIWIL2
8145097	-0,32	-1,25	0,10425	-0,62	-1,54	0,00700	NM_018068	ITK
8109507	-0,32	-1,25	0,25355	-1,09	-2,13	0,00900	NM_005546	CUL9
8119722	-0,32	-1,25	0,10129	1,53	2,89	0,00364	NM_015089	---
8017104	-0,32	-1,25	0,03363	-0,06	-1,05	0,78445	---	LOC643763
8146633	-0,32	-1,25	0,44307	-1,72	-3,29	0,00569	NR_027378	---
8139885	-0,32	-1,25	0,22966	-1,22	-2,34	0,00937	---	---
8015739	-0,32	-1,25	0,07767	-0,20	-1,15	0,18967	---	DNAH14
7910047	-0,32	-1,25	0,67811	1,37	2,58	0,11828	NM_001373	---
7932725	-0,32	-1,25	0,30776	-0,53	-1,44	0,03510	---	PTTG2
8094596	-0,32	-1,25	0,25362	-0,80	-1,74	0,09129	NM_006607	LTB4R
7973702	-0,32	-1,25	0,29003	0,04	1,03	0,78927	NM_001143919	PALMD
7903227	-0,32	-1,25	0,31102	-1,69	-3,22	0,03626	NM_017734	TMED1
8034101	-0,32	-1,25	0,52661	-0,92	-1,90	0,05392	NM_006858	RAB1C
8161238	-0,32	-1,25	0,43206	1,79	3,46	0,00974	U66622	TAF1L // TAF1L // TAF1L // TAF1L // TAF1L
8160597	-0,32	-1,25	0,42703	-0,59	-1,50	0,34284	NM_153809 // NM_153809 // NM_153809 // NM_153809 // NM_153809	---
7949717	-0,32	-1,25	0,28673	-0,97	-1,96	0,06908	---	FGFR2
7936734	-0,32	-1,25	0,21614	-0,07	-1,05	0,52455	NM_000141	---
8144699	-0,32	-1,25	0,54459	-1,14	-2,21	0,00107	---	NPFFR2
8095616	-0,32	-1,25	0,26532	-0,50	-1,41	0,01462	NM_004885	ARGLU1
7972723	-0,32	-1,25	0,09475	0,83	1,77	0,02815	NM_018011	---
8116904	-0,32	-1,25	0,51708	-0,77	-1,71	0,00610	---	IL10
7923907	-0,32	-1,25	0,43646	-1,21	-2,32	0,00598	NM_000572	C8orf22
8146393	-0,32	-1,25	0,26411	-0,34	-1,26	0,04672	NM_001007176	JSRP1
8032465	-0,32	-1,25	0,41750	-1,08	-2,11	0,04589	NM_144616	C9orf82
8160478	-0,32	-1,25	0,20510	-0,06	-1,04	0,71509	NM_024828	TMEM186
7999317	-0,32	-1,25	0,46089	0,33	1,26	0,38393	NM_015421	C17orf79
8014073	-0,32	-1,25	0,13797	-1,23	-2,34	0,10087	NM_018405	MIR200B
7896859	-0,32	-1,25	0,15602	-0,31	-1,24	0,39998	NR_029639	KLF9
8161648	-0,32	-1,25	0,36248	-1,36	-2,56	0,00278	NM_001206	---
7937890	-0,32	-1,25	0,59102	-1,08	-2,12	0,05920	---	SFRS14
8035581	-0,32	-1,25	0,20786	0,85	1,80	0,02397	NM_001017392	OR14A16
7925735	-0,32	-1,25	0,20773	-1,46	-2,75	0,03796	NM_001001966	RAB26
7992518	-0,32	-1,25	0,24301	2,23	4,69	0,00451	NM_014353	PATE2
7952517	-0,32	-1,25	0,55362	-0,45	-1,37	0,10015	NM_212555	ISLR2
7984802	-0,32	-1,25	0,54814	-1,54	-2,91	0,00049	NM_001130136	RND1
7962884	-0,32	-1,25	0,44896	0,42	1,34	0,15184	NM_014470	MTHFR
7912496	-0,32	-1,25	0,27531	0,64	1,56	0,14923	NM_005957	TMEM40
8085393	-0,32	-1,25	0,21246	0,19	1,14	0,44448	NM_018306	C1orf83
7901592	-0,32	-1,25	0,41551	0,91	1,88	0,00774	BC035374	TREX2
8175808	-0,32	-1,25	0,52132	-0,90	-1,86	0,34099	NM_080701	GPR19
7961381	-0,32	-1,25	0,17054	-0,28	-1,21	0,42215	NM_006143	---
8023380	-0,32	-1,25	0,51519	-0,47	-1,38	0,12915	---	TAP2
8180034	-0,32	-1,25	0,05168	1,17	2,26	0,03214	NM_000544	MIR218-1
8094340	-0,32	-1,25	0,50118	-1,48	-2,79	0,10097	NR_029631	---
8114812	-0,32	-1,25	0,25110	-0,98	-1,98	0,11403	---	C16orf82
7994267	-0,32	-1,25	0,57768	-1,50	-2,82	0,00242	NM_001145545	PTTG1 // PTTG1
8109639	-0,32	-1,25	0,35956	0,64	1,56	0,28077	NM_004219 // NM_004219	DBC1
8163716	-0,32	-1,25	0,41723	-1,13	-2,19	0,00337	NM_014618	FAIM
8083000	-0,32	-1,25	0,47908	0,20	1,15	0,62396	NM_001033030	ZNF45
8037433	-0,32	-1,25	0,28601	0,58	1,49	0,27148	NM_003425	SNORD35A
8030366	-0,32	-1,25	0,22366	1,05	2,06	0,03619	NR_000018	DAZL
8085676	-0,32	-1,25	0,21933	-1,02	-2,02	0,00115	NM_001351	HTRA2
8042905	-0,32	-1,25	0,42635	0,27	1,21	0,47901	NM_013247	CCDC148
8045857	-0,32	-1,25	0,46602	-1,10	-2,14	0,17169	NM_138803	ZNF29P
8005106	-0,32	-1,25	0,13910	-0,40	-1,32	0,01630	X52357	ENGASE
8010295	-0,32	-1,25	0,30408	0,02	1,02	0,94149	NM_001042573	SDAD1
8095802	-0,32	-1,25	0,05865	-0,65	-1,57	0,17774	NM_018115	C6orf58
8121936	-0,32	-1,25	0,19735	-1,00	-2,00	0,01709	AK303850	UNC5B
7928189	-0,32	-1,25	0,47884	-0,20	-1,15	0,40979	NM_170744	---
8103242	-0,32	-1,25	0,37186	-1,24	-2,36	0,00723	---	ZNF333
8026365	-0,32	-1,25	0,18574	-0,15	-1,11	0,82632	NM_032433	SLC34A1
8110347	-0,32	-1,25	0,59030	-0,71	-1,63	0,06368	NM_003052	OR10C1

8117727	-0,33	-1,25	0,56722	-0,82	-1,77	0,01042	NM_013941	OR6C4
7956003	-0,33	-1,25	0,52267	-0,39	-1,31	0,03209	NM_001005494	OR12D3
8178289	-0,33	-1,25	0,31417	-0,73	-1,65	0,00147	NM_030959	---
8154979	-0,33	-1,25	0,62137	-0,66	-1,58	0,06313	---	---
7933178	-0,33	-1,25	0,76114	-2,31	-4,95	0,00546	---	ARHGEF1
8029147	-0,33	-1,25	0,57248	-0,03	-1,02	0,91330	NM_199002	DNMT3A
8050813	-0,33	-1,25	0,21193	-0,20	-1,15	0,09843	NM_175629	OR2M5
7911263	-0,33	-1,25	0,61944	-1,00	-2,00	0,08294	NM_001004690	KRTAP5-10
7942267	-0,33	-1,25	0,15244	-0,29	-1,23	0,69557	NM_001012710	C8orf31
8148580	-0,33	-1,25	0,45730	0,67	1,59	0,03319	BC073830	FAM54A
8129763	-0,33	-1,25	0,22834	1,04	2,06	0,02416	NM_001099286	IFNA10
8160389	-0,33	-1,25	0,27617	-1,06	-2,08	0,05858	NM_002171	SNORD115-1
7982008	-0,33	-1,25	0,61331	-1,94	-3,85	0,00001	NR_001291	SNORD115-1
7982032	-0,33	-1,25	0,61331	-1,94	-3,85	0,00001	NR_001291	SNORD115-1
7982038	-0,33	-1,25	0,61331	-1,94	-3,85	0,00001	NR_001291	---
8102743	-0,33	-1,25	0,39243	0,16	1,11	0,55294	---	VNN2
8129637	-0,33	-1,25	0,17713	-1,24	-2,36	0,00326	NM_004665	LOC401387
8140869	-0,33	-1,25	0,39010	-1,23	-2,35	0,03013	NM_001161528	C21orf99
8067825	-0,33	-1,25	0,67342	-1,90	-3,73	0,00010	NR_026916	---
8113717	-0,33	-1,25	0,25656	-0,47	-1,38	0,27436	---	C19orf25
8032224	-0,33	-1,25	0,37906	-0,33	-1,26	0,13531	NM_152482	PRRG3
8170507	-0,33	-1,25	0,20011	-0,82	-1,77	0,11975	NR_033262	---
8096079	-0,33	-1,25	0,52378	-1,22	-2,34	0,06052	---	ZNF596
8144230	-0,33	-1,25	0,36869	-0,38	-1,30	0,11728	NM_001042416	AQP1
8132118	-0,33	-1,25	0,44345	-0,20	-1,15	0,56597	NM_198098	---
8043900	-0,33	-1,26	0,13631	-0,25	-1,19	0,34399	---	C9orf103
8156116	-0,33	-1,26	0,37214	0,94	1,91	0,00215	NM_001001551	GTPBP3
8026735	-0,33	-1,26	0,42635	0,66	1,57	0,20888	NM_133644	OR1A2
8003802	-0,33	-1,26	0,57916	-1,96	-3,89	0,04750	NM_012352	GIPC3
8024676	-0,33	-1,26	0,16451	0,00	-1,00	0,98766	NM_133261	---
8141138	-0,33	-1,26	0,26423	-0,79	-1,73	0,01490	---	ATG2A
7949277	-0,33	-1,26	0,29931	1,67	3,19	0,03380	NM_015104	CCDC36
8079719	-0,33	-1,26	0,54616	-0,96	-1,94	0,03355	NM_178173	FAM38A
8003357	-0,33	-1,26	0,02065	2,35	5,09	0,00321	NM_001142864	RASGRP3
8041422	-0,33	-1,26	0,39937	-0,67	-1,59	0,08108	NM_170672	TRIM45
7918925	-0,33	-1,26	0,43944	0,12	1,09	0,69803	NM_025188	TMEM180
7930106	-0,33	-1,26	0,30599	1,96	3,89	0,00603	NM_024789	RBM15
7903878	-0,33	-1,26	0,04038	0,35	1,27	0,18240	NM_022768	SERHL
8073585	-0,33	-1,26	0,19853	-0,34	-1,26	0,35771	NR_027786	LOC388692
7905043	-0,33	-1,26	0,51225	-0,84	-1,79	0,14676	NR_027002	ZNF550
8039655	-0,33	-1,26	0,23779	0,15	1,11	0,46944	NM_001039654	---
8113427	-0,33	-1,26	0,25825	-1,44	-2,71	0,00286	---	MGRN1
7992998	-0,33	-1,26	0,20575	-0,18	-1,14	0,51336	NM_001142290	---
7916775	-0,33	-1,26	0,28435	-0,60	-1,51	0,16148	---	RAPGEF4
8046428	-0,33	-1,26	0,15011	0,54	1,45	0,09901	NM_007023	RAD52
7960261	-0,33	-1,26	0,39522	0,52	1,44	0,02892	NM_134424	SBNO2
8032157	-0,33	-1,26	0,27562	1,18	2,26	0,11639	NM_014963	GPR157
7912239	-0,33	-1,26	0,48874	-0,02	-1,01	0,96007	NM_024980	VPS18
7982845	-0,33	-1,26	0,42302	0,78	1,71	0,23937	NM_020857	NUDT13
7928342	-0,33	-1,26	0,52447	-0,17	-1,12	0,64332	NM_015901	MLL4
8027956	-0,33	-1,26	0,07116	0,77	1,71	0,21569	NM_014727	ALX1
7957452	-0,33	-1,26	0,22635	0,26	1,20	0,57195	NM_006982	SDHC
7906746	-0,33	-1,26	0,69816	1,56	2,95	0,01928	NM_003001	OR10C1
8177704	-0,33	-1,26	0,52426	-0,77	-1,70	0,01978	NM_013941	OR10C1
8179007	-0,33	-1,26	0,52426	-0,77	-1,70	0,01978	NM_013941	ATP8A2
7968154	-0,33	-1,26	0,07497	-0,09	-1,06	0,73790	NM_016529	MAGEB16
8166669	-0,33	-1,26	0,20350	-1,81	-3,51	0,00331	NM_001099921	CDC25C
8114425	-0,33	-1,26	0,06058	1,71	3,28	0,00000	NM_001790	COCH
7973797	-0,33	-1,26	0,62663	3,31	9,92	0,00000	NM_001135058	PIN1
8025506	-0,33	-1,26	0,45047	1,01	2,02	0,16660	NM_006221	LOC100130713
8036936	-0,33	-1,26	0,58845	0,02	1,01	0,96156	AK096566	---
8041711	-0,33	-1,26	0,41698	-0,52	-1,44	0,01644	---	MRGPRX3
7938741	-0,33	-1,26	0,35745	-1,44	-2,71	0,04119	NM_054031	SDS
7966621	-0,33	-1,26	0,13658	-0,53	-1,45	0,02692	NM_006843	DLL1
8130939	-0,33	-1,26	0,30525	0,01	1,00	0,97932	NM_005618	GGA1
8072825	-0,33	-1,26	0,16035	1,46	2,75	0,01753	NM_013365	C7orf46
8131881	-0,33	-1,26	0,12699	3,03	8,18	0,00265	NM_199136	---
7963588	-0,33	-1,26	0,04313	-0,56	-1,47	0,00146	---	RINT1
8142079	-0,33	-1,26	0,06024	-0,72	-1,65	0,04488	NM_021930	FABP5
7948420	-0,33	-1,26	0,31720	1,52	2,87	0,00033	NM_001444	PECAM1
8017599	-0,33	-1,26	0,18863	-0,70	-1,63	0,04335	NM_000442	---
7926539	-0,33	-1,26	0,27382	-1,63	-3,10	0,01874	---	LOC360030
7953670	-0,33	-1,26	0,29086	-0,83	-1,78	0,01989	NM_001145465	ZNF879

8110486	-0,33	-1,26	0,19424	-0,12	-1,09	0,58852	NM_001136116	---
8121062	-0,33	-1,26	0,12901	-1,50	-2,83	0,00897	---	TRIM62
7914630	-0,33	-1,26	0,11048	0,80	1,74	0,06934	NM_018207	RHCG
7991283	-0,33	-1,26	0,28866	0,34	1,27	0,28622	NM_016321	OR12D3
8124645	-0,33	-1,26	0,25465	-0,58	-1,49	0,01044	NM_030959	---
7974337	-0,33	-1,26	0,20334	-0,87	-1,83	0,00986	---	IFLTD1
7961875	-0,33	-1,26	0,14799	-0,84	-1,79	0,03689	NM_001145728	---
7994343	-0,33	-1,26	0,34832	-0,47	-1,39	0,20400	---	CAPN13
8051275	-0,33	-1,26	0,22959	-1,27	-2,41	0,00663	NM_144575	KRT71
7963438	-0,33	-1,26	0,22895	-0,51	-1,43	0,01647	NM_033448	NAALAD2
7943051	-0,33	-1,26	0,14064	-0,73	-1,65	0,03650	NM_005467	RNF144B
8117106	-0,33	-1,26	0,30067	1,72	3,30	0,00084	NM_182757	COX19
8137693	-0,33	-1,26	0,25648	0,08	1,06	0,31278	NM_001031617	C1orf105
7907396	-0,33	-1,26	0,20795	-0,82	-1,77	0,01017	NM_139240	GCOM1
7983890	-0,33	-1,26	0,17891	-0,91	-1,87	0,06267	NM_001018100	OR52M1
7937944	-0,33	-1,26	0,40352	-1,15	-2,22	0,02777	NM_001004137	AURKC
8031700	-0,33	-1,26	0,38880	-0,64	-1,56	0,13007	NM_001015878	TNFSF18
7922337	-0,33	-1,26	0,37602	-0,97	-1,95	0,00056	NM_005092	KIF27
8156164	-0,33	-1,26	0,60254	2,44	5,41	0,00197	NM_017576	C10orf4
7935123	-0,33	-1,26	0,10120	-0,58	-1,49	0,02373	NM_145246	---
8128314	-0,33	-1,26	0,17213	-1,45	-2,73	0,01719	---	SNIP1
7915008	-0,33	-1,26	0,15204	0,34	1,27	0,10873	NM_024700	HAPLN1
8112971	-0,33	-1,26	0,02590	-0,65	-1,57	0,00068	NM_001884	P2RX1
8011499	-0,34	-1,26	0,19914	-1,07	-2,10	0,02145	NM_002558	---
8141148	-0,34	-1,26	0,33513	-1,03	-2,04	0,01623	---	PCDP1
8044818	-0,34	-1,26	0,11936	-0,49	-1,41	0,05898	NM_001029996	OR2M4
7911269	-0,34	-1,26	0,51308	-1,48	-2,79	0,00287	NM_017504	ANKRD34A
7904737	-0,34	-1,26	0,44546	-0,93	-1,90	0,10909	NM_001039888	KIAA1671
8071981	-0,34	-1,26	0,47065	0,50	1,41	0,48739	NM_001145206	GPR77
8029914	-0,34	-1,26	0,05604	-1,04	-2,06	0,01422	NM_018485	APOA4
7951859	-0,34	-1,26	0,13830	-0,75	-1,68	0,00272	NM_000482	L3MBTL4
8022145	-0,34	-1,26	0,27932	-0,64	-1,56	0,04812	NM_173464	MYBPC1
7957966	-0,34	-1,26	0,45342	-1,45	-2,74	0,00282	NM_002465	---
8099253	-0,34	-1,26	0,30979	-1,43	-2,69	0,00832	---	MIR31
8160439	-0,34	-1,26	0,64554	-0,61	-1,53	0,33479	NR_029505	C5orf36
8113113	-0,34	-1,26	0,18693	-0,21	-1,16	0,30982	NM_173665	CDK5RAP1
8065668	-0,34	-1,26	0,40789	0,73	1,66	0,08135	NM_016408	---
8163381	-0,34	-1,26	0,48752	-1,21	-2,31	0,02214	---	B3GALTL
7968370	-0,34	-1,26	0,01213	2,03	4,08	0,00002	NM_194318	---
8066212	-0,34	-1,26	0,14946	-0,82	-1,76	0,05315	---	ZPLD1
8081407	-0,34	-1,26	0,39368	-1,15	-2,21	0,01799	NM_175056	BCORL1
8169882	-0,34	-1,26	0,26786	1,07	2,10	0,00266	NM_021946	TXNDC8
8163193	-0,34	-1,26	0,21692	-0,54	-1,46	0,01194	NM_001003936	---
7935359	-0,34	-1,26	0,09718	-1,04	-2,06	0,06929	---	ANKH
8104590	-0,34	-1,26	0,39885	1,87	3,67	0,00587	NM_054027	SETD6
7996241	-0,34	-1,26	0,15556	1,27	2,41	0,02074	NM_001160305	---
8059876	-0,34	-1,26	0,23903	-1,16	-2,23	0,00156	---	LOC151009
8054517	-0,34	-1,26	0,60768	3,38	10,42	0,00001	AK095678	TBRG4
8139468	-0,34	-1,26	0,43822	1,21	2,32	0,00634	NM_004749	ST8SIA2
7986229	-0,34	-1,26	0,42533	-1,15	-2,22	0,00010	NM_006011	---
8052123	-0,34	-1,26	0,42814	-0,25	-1,19	0,28804	---	VTI1A
7930524	-0,34	-1,26	0,43107	1,09	2,12	0,08771	NM_145206	PSCA
8148548	-0,34	-1,26	0,40770	-0,33	-1,26	0,13721	NM_005672	---
7925087	-0,34	-1,26	0,03825	-0,67	-1,59	0,03015	---	HCN3
7905974	-0,34	-1,26	0,33831	1,02	2,03	0,04057	NM_020897	HOXC10
7955858	-0,34	-1,26	0,30488	0,62	1,54	0,20052	NM_017409	SPDYE3
8134785	-0,34	-1,26	0,57832	-1,10	-2,14	0,05584	NM_001004351	FLJ37543
8105517	-0,34	-1,26	0,18161	-0,80	-1,74	0,01591	NM_173667	TMEM232
8113483	-0,34	-1,26	0,34466	-0,62	-1,54	0,17164	NM_001039763	CAMK4
8107307	-0,34	-1,26	0,54718	-1,16	-2,24	0,00007	NM_001744	HSPB2
7943787	-0,34	-1,27	0,40258	-1,06	-2,08	0,01584	NM_001541	TSFM
7956639	-0,34	-1,27	0,58532	2,86	7,25	0,00393	NM_001172696	NSUN5P2
8133633	-0,34	-1,27	0,37940	0,59	1,50	0,16965	NR_033323	ADCY10
7922058	-0,34	-1,27	0,06344	-0,66	-1,59	0,07084	NM_018417	TUBB1
8063716	-0,34	-1,27	0,20537	-0,76	-1,70	0,03304	NM_030773	BHMT
8106504	-0,34	-1,27	0,14878	-1,16	-2,23	0,01930	NM_001713	TMEM132C
7959827	-0,34	-1,27	0,48003	-1,58	-3,00	0,00251	NM_001136103	---
7981215	-0,34	-1,27	0,60547	-1,86	-3,64	0,00383	---	IAPP
7954377	-0,34	-1,27	0,28753	-0,56	-1,47	0,19005	NM_000415	---
7979804	-0,34	-1,27	0,12705	-1,03	-2,05	0,02744	---	---
8056823	-0,34	-1,27	0,45922	-1,21	-2,31	0,11994	---	BEST1
7940582	-0,34	-1,27	0,07375	1,52	2,87	0,02775	NM_004183	---
7987135	-0,34	-1,27	0,79810	-1,18	-2,26	0,15569	---	---

8022506	-0,34	-1,27	0,40255	-1,81	-3,50	0,19802	---	SLC35F1
8121712	-0,34	-1,27	0,65432	-0,72	-1,64	0,09797	NM_001029858	LOC389936
7926117	-0,34	-1,27	0,45241	-1,46	-2,76	0,00300	AK131480	HAUS6
8132843	-0,34	-1,27	0,72478	1,56	2,94	0,03764	NM_017645	FSHB
7939080	-0,34	-1,27	0,40486	-1,03	-2,04	0,01732	NM_000510	HDAC9
8131631	-0,34	-1,27	0,02479	-1,17	-2,25	0,00213	NM_178423	EHMT1
8159702	-0,34	-1,27	0,22416	2,12	4,36	0,00242	NM_024757	NOTCH1
8165217	-0,34	-1,27	0,47712	1,80	3,47	0,03572	NM_017617	REG3A
8053341	-0,34	-1,27	0,50484	-1,18	-2,27	0,00651	NM_138938	RHEBL1
7963009	-0,34	-1,27	0,14725	1,42	2,68	0,00935	NM_144593	C6orf223
8119918	-0,34	-1,27	0,30153	-0,59	-1,51	0,03582	NM_153246	UCP1
8102904	-0,34	-1,27	0,38346	-0,87	-1,83	0,01655	NM_021833	RUNDC3B
8133961	-0,34	-1,27	0,23158	-0,05	-1,04	0,89106	NM_138290	OR10A6
7946330	-0,34	-1,27	0,00210	-0,52	-1,44	0,00185	NM_001004461	---
7929814	-0,34	-1,27	0,42628	-1,32	-2,49	0,00020	---	TPD52
8151475	-0,34	-1,27	0,12130	1,70	3,24	0,00372	NM_001025252	CLEC7A
7961120	-0,34	-1,27	0,14284	-1,42	-2,68	0,01304	NM_197947	APOL5
8072721	-0,34	-1,27	0,38696	-1,04	-2,05	0,00547	NM_030642	---
7932508	-0,34	-1,27	0,05461	-1,19	-2,29	0,12097	---	C10orf31
7932019	-0,34	-1,27	0,22532	-1,03	-2,04	0,00165	ENST00000379256	C17orf103
8013509	-0,34	-1,27	0,29646	-0,93	-1,90	0,03433	NM_152914	CASP10
8047403	-0,34	-1,27	0,01066	-0,17	-1,13	0,36263	NM_032977	EDN1
8116921	-0,34	-1,27	0,42738	1,02	2,03	0,06406	NM_001955	HAUS3
8098985	-0,34	-1,27	0,21622	-0,14	-1,10	0,70652	NM_024511	RNASE8
7973156	-0,34	-1,27	0,53949	-1,46	-2,75	0,20823	NM_138331	HELB
7956894	-0,34	-1,27	0,16175	1,07	2,10	0,02576	NM_033647	---
8174594	-0,34	-1,27	0,28143	-0,63	-1,54	0,02251	---	C20orf152
8062237	-0,34	-1,27	0,02761	-0,94	-1,92	0,00048	BC114945	ZNF620
8078999	-0,34	-1,27	0,17388	0,77	1,71	0,03275	NM_175888	FN3KRP
8010832	-0,34	-1,27	0,04565	0,99	1,99	0,03281	NM_024619	KIAA1609
8003158	-0,34	-1,27	0,31322	0,80	1,74	0,07343	NM_020947	OR52E6
7946109	-0,34	-1,27	0,41824	-1,64	-3,13	0,00031	NM_001005167	FURIN
7986092	-0,34	-1,27	0,38287	1,88	3,68	0,00142	NM_002569	GLYCTK
8080212	-0,34	-1,27	0,32929	-1,36	-2,57	0,00712	NM_145262	HIST1H4A
8117334	-0,34	-1,27	0,46090	-0,43	-1,34	0,07508	NM_003538	SNORA60
8062490	-0,34	-1,27	0,53807	-1,10	-2,14	0,06920	NR_002986	SCGB3A2
8108995	-0,35	-1,27	0,27828	-1,44	-2,72	0,02094	NM_054023	EPHB1
8090891	-0,35	-1,27	0,27128	-1,07	-2,09	0,00529	NM_004441	SRRM1
7898975	-0,35	-1,27	0,43061	0,31	1,24	0,22471	NM_005839	---
8063447	-0,35	-1,27	0,69831	-3,16	-8,94	0,03255	---	ARL5C
8014787	-0,35	-1,27	0,44261	-0,59	-1,51	0,05150	NM_001143968	ADAT2
8129963	-0,35	-1,27	0,28886	0,90	1,86	0,04093	NM_182503	GRIA2
8098021	-0,35	-1,27	0,38547	-0,86	-1,82	0,23889	NM_001083619	CHRM1
7948912	-0,35	-1,27	0,41998	-1,60	-3,02	0,00338	NM_000738	ATP5L2
8076493	-0,35	-1,27	0,22527	-0,96	-1,95	0,02800	NM_001165877	POLQ
8089875	-0,35	-1,27	0,17414	0,44	1,36	0,32033	NM_199420	---
8166047	-0,35	-1,27	0,03633	-0,73	-1,66	0,01973	---	SKI
7897068	-0,35	-1,27	0,26078	0,24	1,18	0,69863	NM_003036	GPR141
8132347	-0,35	-1,27	0,29333	-0,80	-1,74	0,00423	NM_181791	---
7981288	-0,35	-1,27	0,57995	-1,87	-3,65	0,00671	---	PPP2R5B
7941087	-0,35	-1,27	0,20955	0,97	1,96	0,07372	NM_006244	RXRB
8178903	-0,35	-1,27	0,63034	2,46	5,50	0,00054	NM_021976	UNC119
8013633	-0,35	-1,27	0,40549	0,21	1,15	0,33232	NM_054035	HLA-DQB1
8178826	-0,35	-1,27	0,09704	0,66	1,58	0,20537	AK296613	HLA-DQB1
8180022	-0,35	-1,27	0,09704	0,66	1,58	0,20537	AK296613	TMEM80
7937438	-0,35	-1,27	0,36618	1,25	2,39	0,05465	NM_174940	TSSC4
7937813	-0,35	-1,27	0,61574	-1,04	-2,06	0,00464	NM_005706	ZDHHC15
8173647	-0,35	-1,27	0,08523	-0,88	-1,85	0,04588	NM_144969	PELI2
7974533	-0,35	-1,27	0,38826	-1,84	-3,58	0,00098	NM_021255	ZNF709
8034395	-0,35	-1,27	0,01216	-0,78	-1,72	0,04073	NM_152601	---
8151411	-0,35	-1,27	0,44198	-0,75	-1,68	0,08470	---	FUBP3
8158686	-0,35	-1,27	0,36331	1,61	3,06	0,01274	NM_003934	HMGCS2
7919055	-0,35	-1,27	0,00194	-1,00	-2,00	0,09911	NM_005518	DENND3
8148476	-0,35	-1,27	0,09948	1,66	3,15	0,00304	NM_014957	---
7947127	-0,35	-1,27	0,04030	-0,30	-1,23	0,03813	---	OSTCL
8130522	-0,35	-1,27	0,06985	-0,27	-1,20	0,30930	NR_028496	NANOGP1
7953689	-0,35	-1,27	0,29175	-1,43	-2,69	0,05841	AY455283	KRT4
7963534	-0,35	-1,27	0,31645	0,58	1,49	0,01588	NM_002272	LOC100130428
7952451	-0,35	-1,27	0,11146	-0,34	-1,27	0,37607	BC040288	ATP8B1
8021365	-0,35	-1,27	0,58834	-0,34	-1,27	0,59274	NM_005603	DCHS2
8103260	-0,35	-1,27	0,05690	-0,96	-1,95	0,00146	NM_017639	KRTAP5-2
7945648	-0,35	-1,27	0,74937	-1,68	-3,21	0,05085	NM_001004325	CPA2
8136187	-0,35	-1,27	0,06073	-0,64	-1,56	0,00794	NM_001869	CPT1C

8030448	-0,35	-1,27	0,26795	-0,19	-1,14	0,60730	NM_001136052	FRG2C
8080994	-0,35	-1,27	0,40709	-0,71	-1,64	0,03187	NM_001124759	SNORD115-11
7982084	-0,35	-1,27	0,77126	-2,62	-6,13	0,00103	NR_003303	ADC
7899851	-0,35	-1,27	0,16969	1,31	2,49	0,09407	NM_052998	NFATC1
8021866	-0,35	-1,27	0,29012	0,42	1,34	0,42839	NM_172387	SNORD25
7948910	-0,35	-1,27	0,56195	3,69	12,87	0,00172	NR_002565	---
8072007	-0,35	-1,27	0,07682	-1,27	-2,42	0,06808	---	---
8166819	-0,35	-1,27	0,27462	-0,48	-1,40	0,00386	---	---
8022644	-0,35	-1,27	0,11401	-0,72	-1,65	0,08821	---	OR10G3
7977767	-0,35	-1,27	0,12655	-0,41	-1,33	0,00310	NM_001005465	TAZ
8170865	-0,35	-1,27	0,20250	0,66	1,58	0,08774	NM_000116	OR2A20P
8136983	-0,35	-1,27	0,67752	1,01	2,01	0,00914	NR_002158	ZNF324
8031956	-0,35	-1,27	0,55445	-1,17	-2,25	0,03862	NM_014347	HTR7
7934970	-0,35	-1,27	0,29754	-1,40	-2,63	0,03085	NM_019859	CYP1A1
7990391	-0,35	-1,27	0,41576	1,51	2,86	0,00370	NM_000499	FRMD8
7941260	-0,35	-1,27	0,25702	0,42	1,34	0,50176	NM_031904	LAMB4
8142232	-0,35	-1,27	0,26639	-0,28	-1,21	0,21598	NM_007356	GLS2
7964183	-0,35	-1,28	0,55860	1,04	2,05	0,02390	NM_013267	MTMR9L
7914516	-0,35	-1,28	0,42447	0,24	1,18	0,51444	NR_026850	ITIH5
7931977	-0,35	-1,28	0,33638	-0,82	-1,76	0,05441	NM_030569	C12orf36
7961413	-0,35	-1,28	0,34856	-0,71	-1,63	0,00000	NM_182558	---
7961420	-0,35	-1,28	0,29469	-0,39	-1,31	0,06402	---	FBXL12
8033813	-0,35	-1,28	0,56142	2,00	3,99	0,02501	NM_017703	ALS2CR12
8058203	-0,35	-1,28	0,38429	-0,32	-1,25	0,07428	NM_139163	CERKL
8057463	-0,35	-1,28	0,37771	-0,88	-1,84	0,03449	NM_201548	---
7928306	-0,35	-1,28	0,12569	0,28	1,22	0,55929	---	LGALS12
7940762	-0,35	-1,28	0,21583	-1,65	-3,13	0,00000	NM_001142535	AFP
8095646	-0,35	-1,28	0,03260	-0,96	-1,94	0,00083	NM_001134	---
8052141	-0,35	-1,28	0,31606	-1,92	-3,78	0,00926	---	---
8163017	-0,35	-1,28	0,11835	-1,67	-3,18	0,01143	---	NAT8L
8093578	-0,35	-1,28	0,04514	-0,02	-1,01	0,94644	NM_178557	CMTM4
8001830	-0,35	-1,28	0,36968	3,20	9,21	0,00042	NM_181521	TRDMT1
7932390	-0,35	-1,28	0,29906	0,06	1,04	0,86882	NM_004412	---
8062681	-0,35	-1,28	0,41352	-0,58	-1,50	0,13407	---	MAFB
8066266	-0,35	-1,28	0,07728	-1,39	-2,62	0,09104	NM_005461	EPB41L1
8062251	-0,35	-1,28	0,24999	-0,07	-1,05	0,83199	NM_012156	SNORD95
8116532	-0,35	-1,28	0,67000	0,58	1,50	0,61631	NR_002591	HIST1H1T
8124402	-0,35	-1,28	0,17456	-1,00	-2,01	0,00564	NM_005323	OR13C3
8162931	-0,35	-1,28	0,28913	-0,75	-1,68	0,02177	NM_001001961	---
8165903	-0,35	-1,28	0,27241	-1,05	-2,07	0,04183	---	SNORD7
8006540	-0,35	-1,28	0,05156	-0,38	-1,30	0,00034	NR_003037	---
8096661	-0,35	-1,28	0,29588	-0,61	-1,53	0,31784	---	PLEKHH3
8015665	-0,35	-1,28	0,56592	-1,13	-2,18	0,04323	NM_024927	---
8028323	-0,35	-1,28	0,12191	-0,83	-1,78	0,03102	---	SLCO4C1
8113369	-0,35	-1,28	0,13547	0,37	1,29	0,24308	NM_180991	---
8126016	-0,35	-1,28	0,17181	-0,79	-1,72	0,04307	---	---
8098163	-0,35	-1,28	0,67779	-0,58	-1,50	0,05231	---	CCDC105
8026390	-0,35	-1,28	0,14841	-0,97	-1,97	0,08225	NM_173482	PRH1
7961259	-0,35	-1,28	0,07961	0,25	1,19	0,55157	NM_006250	---
7972175	-0,35	-1,28	0,51996	-0,84	-1,79	0,41496	---	AVIL
7964555	-0,35	-1,28	0,06824	-0,43	-1,35	0,33613	NM_006576	---
8080619	-0,35	-1,28	0,40733	-0,60	-1,52	0,24071	---	---
8148047	-0,35	-1,28	0,06248	-0,89	-1,85	0,01682	---	LIG1
8037991	-0,35	-1,28	0,16599	-0,12	-1,08	0,50910	NM_000234	NLRP2
8031398	-0,35	-1,28	0,17308	-0,97	-1,95	0,00362	NM_017852	MOG
8179011	-0,35	-1,28	0,21210	-1,27	-2,41	0,00503	NM_206809	GIMAP7
8137240	-0,35	-1,28	0,33675	-0,84	-1,78	0,05725	NM_153236	OR6C1
7955989	-0,35	-1,28	0,20078	-0,19	-1,14	0,20196	NM_001005182	---
8038441	-0,35	-1,28	0,15298	-0,83	-1,78	0,00135	---	---
8124183	-0,36	-1,28	0,41431	-1,07	-2,10	0,06977	---	CCL3L1
8014391	-0,36	-1,28	0,49334	-0,94	-1,92	0,02378	NM_021006	CCL3L1
8014414	-0,36	-1,28	0,49334	-0,94	-1,92	0,02378	NM_021006	CCL3L1
8019731	-0,36	-1,28	0,49334	-0,94	-1,92	0,02378	NM_021006	PBXIP1
7920575	-0,36	-1,28	0,10273	0,06	1,04	0,87523	NM_020524	MAP2K3
8005707	-0,36	-1,28	0,23612	0,72	1,65	0,04763	NM_145109	SUCNR1
8083422	-0,36	-1,28	0,43959	-0,66	-1,58	0,05683	NM_033050	HEATR4
7980024	-0,36	-1,28	0,57159	0,82	1,76	0,21797	NM_203309	---
8050803	-0,36	-1,28	0,06526	-0,67	-1,59	0,00341	---	MS4A7
7940259	-0,36	-1,28	0,05229	-1,69	-3,23	0,00285	NM_021201	SNORA49
7960052	-0,36	-1,28	0,05470	-0,83	-1,78	0,20574	NR_002979	ZSCAN21
8134689	-0,36	-1,28	0,17657	-0,30	-1,23	0,16401	NM_145914	---
8002939	-0,36	-1,28	0,45165	-1,53	-2,88	0,02273	---	FAM13C
7933733	-0,36	-1,28	0,18126	-0,88	-1,84	0,00240	NM_198215	GOLGA8DP

7991695	-0,36	-1,28	0,46294	-0,40	-1,32	0,42355	NR_027407	MIR145
8109159	-0,36	-1,28	0,02633	-0,73	-1,65	0,03507	NR_029686	R3HCC1
8145272	-0,36	-1,28	0,19043	1,43	2,70	0,03122	NM_001136108	---
8022439	-0,36	-1,28	0,17649	-0,37	-1,30	0,10470	---	FAM187B
8036069	-0,36	-1,28	0,45466	-1,22	-2,33	0,05402	NM_152481	C19orf26
8032197	-0,36	-1,28	0,57034	-0,20	-1,14	0,70244	NM_152769	C8orf80
8149979	-0,36	-1,28	0,24897	-1,07	-2,10	0,14272	NM_001010906	ST6GALNAC1
8018774	-0,36	-1,28	0,46682	-0,48	-1,39	0,00427	NM_018414	---
8133038	-0,36	-1,28	0,72535	-3,70	-12,95	0,00016	---	---
8066557	-0,36	-1,28	0,28296	-0,28	-1,22	0,48157	---	---
8023545	-0,36	-1,28	0,27253	-0,72	-1,64	0,00113	---	MIR30C2
8127498	-0,36	-1,28	0,06133	-0,79	-1,72	0,06849	NR_029598	EFNB2
7972713	-0,36	-1,28	0,07268	-0,86	-1,82	0,24034	NM_004093	---
7959818	-0,36	-1,28	0,42655	-1,17	-2,25	0,01288	---	---
8145685	-0,36	-1,28	0,14069	-0,85	-1,80	0,01708	---	CHST9
8022666	-0,36	-1,28	0,15473	-0,95	-1,94	0,00045	NM_031422	---
8036989	-0,36	-1,28	0,31598	-1,23	-2,34	0,16850	---	SNORD42A
8005955	-0,36	-1,28	0,27912	-0,17	-1,13	0,53382	NR_000014	DCAF12L2
8174968	-0,36	-1,28	0,43912	-0,63	-1,55	0,06133	NM_001013628	ASPHD1
7994675	-0,36	-1,28	0,18614	0,19	1,14	0,17456	NM_181718	MAP2K7
8025351	-0,36	-1,28	0,14168	0,39	1,31	0,34467	NM_145185	ZIK1
8031768	-0,36	-1,28	0,49583	-0,47	-1,39	0,00298	NM_001010879	AARS2
8126681	-0,36	-1,28	0,29145	0,53	1,44	0,09326	NM_020745	ZWINT
7933707	-0,36	-1,28	0,30809	0,08	1,06	0,81475	NM_032997	GNRH1
8149849	-0,36	-1,28	0,09978	-0,88	-1,84	0,15724	NM_000825	---
8001691	-0,36	-1,28	0,30903	-0,40	-1,32	0,01464	---	MYO15B
8009875	-0,36	-1,28	0,39413	0,31	1,24	0,32596	NR_003587	---
8091340	-0,36	-1,28	0,33935	-0,70	-1,63	0,00248	---	---
8161854	-0,36	-1,28	0,54198	-1,40	-2,64	0,02206	---	FNDC1
8123104	-0,36	-1,28	0,26547	0,26	1,20	0,40138	NM_032532	C7orf47
8141517	-0,36	-1,28	0,05093	1,36	2,56	0,02008	BC026269	DCUN1D3
8000028	-0,36	-1,28	0,28459	1,34	2,53	0,27353	NM_173475	HSFY2
8176681	-0,36	-1,28	0,08038	-0,68	-1,60	0,01232	NR_003509	HSFY2
8177201	-0,36	-1,28	0,08038	-0,68	-1,60	0,01232	NR_003509	C19orf48
8038624	-0,36	-1,28	0,06456	1,87	3,66	0,02005	NM_199249	---
8066292	-0,36	-1,28	0,09350	-0,75	-1,68	0,20059	---	PNKD
8048257	-0,36	-1,28	0,45288	2,07	4,21	0,01468	NM_015488	LOC151009
8054513	-0,36	-1,28	0,66227	0,18	1,14	0,26842	NR_027244	---
7966148	-0,36	-1,28	0,14315	-1,01	-2,02	0,00102	---	OR1L6
8157688	-0,36	-1,28	0,26767	-1,28	-2,42	0,00560	NM_001004453	FFAR2
8027862	-0,36	-1,28	0,23522	-0,79	-1,72	0,06236	NM_005306	CD3EAP
8029688	-0,36	-1,28	0,18611	0,12	1,09	0,70947	NM_012099	CNGA1
8100202	-0,36	-1,28	0,23186	-0,15	-1,11	0,58269	NM_001142564	FAM78A
8164644	-0,36	-1,28	0,29908	-0,80	-1,74	0,06445	NM_033387	MIR24-1
8156573	-0,36	-1,28	0,02776	-0,93	-1,91	0,02793	NR_029496	KIFC1
8118669	-0,36	-1,28	0,42037	1,41	2,66	0,00544	NM_002263	---
8109401	-0,36	-1,28	0,23500	-1,39	-2,61	0,14336	---	---
8108376	-0,36	-1,28	0,39038	-1,82	-3,52	0,01992	---	AQP11
7942774	-0,36	-1,28	0,26478	0,23	1,17	0,10714	NM_173039	CCDC148
8056022	-0,36	-1,28	0,06734	-0,68	-1,60	0,02050	NM_138803	GAMT
8032214	-0,36	-1,28	0,17076	-0,17	-1,12	0,49504	NM_000156	C12orf65
7959563	-0,36	-1,28	0,49477	0,37	1,29	0,24727	NM_152269	TECPR2
7976976	-0,36	-1,28	0,04150	1,72	3,30	0,00090	NM_014844	MESDC1
7985349	-0,36	-1,28	0,57438	0,52	1,43	0,20100	NM_022566	LIN28B
8121251	-0,36	-1,28	0,44648	-0,98	-1,98	0,01732	NM_001004317	CENPQ
8120165	-0,36	-1,28	0,04339	0,89	1,85	0,19632	NM_018132	SLC13A3
8066757	-0,36	-1,28	0,38038	-0,85	-1,80	0,00821	NM_022829	SPATA1
7902645	-0,36	-1,28	0,12597	-0,74	-1,67	0,04646	NM_001081472	HNF1B
8014591	-0,36	-1,28	0,37495	3,28	9,73	0,00009	NM_000458	MSX1
8093852	-0,36	-1,28	0,33946	-1,16	-2,23	0,02346	NM_002448	---
8144878	-0,36	-1,28	0,08556	-0,62	-1,53	0,09452	---	SPHKAP
8059551	-0,36	-1,28	0,44513	-0,72	-1,64	0,00329	NM_001142644	---
7917972	-0,36	-1,28	0,18823	-0,48	-1,39	0,03410	---	GPR137C
7974372	-0,36	-1,29	0,29330	-0,17	-1,12	0,63861	NM_001099652	FAM128A
8045148	-0,36	-1,29	0,40034	0,04	1,03	0,91851	ENST00000427024	FAM128A
8055284	-0,36	-1,29	0,40034	0,04	1,03	0,91851	ENST00000427024	ZNF701
8030950	-0,36	-1,29	0,40951	-0,07	-1,05	0,85130	NM_018260	AGT
7924987	-0,36	-1,29	0,49368	-1,64	-3,12	0,00190	NM_000029	GEFT
7956551	-0,36	-1,29	0,26145	0,62	1,53	0,23353	NM_182947	---
7900508	-0,36	-1,29	0,32685	-0,24	-1,18	0,19494	---	---
8139196	-0,36	-1,29	0,15724	-0,53	-1,45	0,01864	---	NODAL
7934156	-0,36	-1,29	0,19601	-0,53	-1,45	0,08006	NM_018055	ERCC6L
8173506	-0,36	-1,29	0,24007	-0,06	-1,04	0,74242	NM_017669	---

7900633	-0,36	-1,29	0,10666	-1,31	-2,48	0,03261	---	C16orf91
7998485	-0,36	-1,29	0,60259	-0,27	-1,21	0,28577	NM_001010878	MFI2
8093171	-0,36	-1,29	0,37803	1,68	3,21	0,03021	NM_005929	LBP
8062461	-0,36	-1,29	0,38600	-0,93	-1,90	0,04328	NM_004139	ADCK2
8136591	-0,36	-1,29	0,48611	0,01	1,01	0,96965	NM_052853	OR5D18
7939946	-0,36	-1,29	0,34791	-1,39	-2,63	0,07961	NM_001001952	C9orf98
8164766	-0,36	-1,29	0,08408	0,02	1,01	0,91868	NM_152572	ZC3H12B
8167957	-0,36	-1,29	0,03879	-1,09	-2,13	0,09074	NM_001010888	TAF5
7930213	-0,36	-1,29	0,02568	1,39	2,62	0,00209	NM_006951	---
8113122	-0,36	-1,29	0,37537	-1,84	-3,58	0,01991	---	ALS2CR11
8058238	-0,36	-1,29	0,28995	-0,27	-1,21	0,37109	NM_001168221	---
7900336	-0,36	-1,29	0,22952	-0,56	-1,48	0,02359	---	C1orf59
7918223	-0,36	-1,29	0,17948	1,04	2,05	0,00317	NM_144584	VASP
8029710	-0,36	-1,29	0,34294	-0,77	-1,70	0,16978	NM_003370	DKFZp434H1419
8048171	-0,36	-1,29	0,43181	0,95	1,94	0,00817	AK125369	RPL29
8087830	-0,36	-1,29	0,72114	0,06	1,04	0,95537	NM_000992	TEX11
8173381	-0,36	-1,29	0,09086	-0,56	-1,47	0,10198	NM_001003811	RNF215
8075365	-0,36	-1,29	0,21096	-0,29	-1,22	0,47681	NM_001017981	LOC221442
8119423	-0,36	-1,29	0,45418	-1,23	-2,34	0,37814	NR_026938	---
7930775	-0,36	-1,29	0,25980	-2,07	-4,20	0,03050	---	OR56A4
7946117	-0,37	-1,29	0,47687	-1,38	-2,60	0,00008	NM_001005179	ERN1
8017555	-0,37	-1,29	0,06498	0,99	1,98	0,01932	NM_001433	CAD
8040843	-0,37	-1,29	0,08446	2,13	4,39	0,01261	NM_004341	DHODH
7997179	-0,37	-1,29	0,14374	1,05	2,07	0,01279	NM_001361	MYOM1
8022045	-0,37	-1,29	0,10102	0,09	1,06	0,48312	NM_003803	---
8078958	-0,37	-1,29	0,01334	-0,48	-1,39	0,07878	---	---
8061127	-0,37	-1,29	0,30184	-0,54	-1,45	0,10086	---	C1orf66
7906223	-0,37	-1,29	0,33554	2,21	4,61	0,00491	NM_015997	KRTAP5-9
7942261	-0,37	-1,29	0,52986	-0,45	-1,37	0,07694	NM_005553	---
7939996	-0,37	-1,29	0,33167	-0,68	-1,60	0,03581	---	C8orf51
8153405	-0,37	-1,29	0,10570	2,06	4,17	0,00050	NR_026785	C19orf73
8038314	-0,37	-1,29	0,29959	0,87	1,82	0,07757	NM_018111	JAKMIP1
8099200	-0,37	-1,29	0,42206	-0,75	-1,68	0,00958	NM_144720	---
8046500	-0,37	-1,29	0,30121	-0,92	-1,90	0,00390	---	---
7981714	-0,37	-1,29	0,66154	-2,34	-5,05	0,00028	---	GDF9
8114080	-0,37	-1,29	0,29946	0,16	1,11	0,72736	NM_005260	PTPRB
7964872	-0,37	-1,29	0,09992	-0,68	-1,60	0,00735	NM_001109754	---
7981523	-0,37	-1,29	0,57585	-0,82	-1,77	0,00500	---	PPFIA3
8030220	-0,37	-1,29	0,07869	-0,31	-1,24	0,22850	NM_003660	---
8174646	-0,37	-1,29	0,33031	-1,52	-2,87	0,03610	---	---
8167726	-0,37	-1,29	0,07205	-0,95	-1,94	0,00032	---	COL6A6
8082597	-0,37	-1,29	0,31377	-0,65	-1,57	0,05669	NM_001102608	hCG_1732469
8054465	-0,37	-1,29	0,68473	-0,82	-1,76	0,00099	ENST00000429128	---
8155944	-0,37	-1,29	0,32468	-0,96	-1,94	0,01670	---	TLCD1
8013771	-0,37	-1,29	0,46832	2,93	7,63	0,00060	NM_138463	FHOD1
8001981	-0,37	-1,29	0,21797	0,94	1,92	0,02945	NM_013241	---
7966181	-0,37	-1,29	0,08810	-0,58	-1,50	0,01431	---	PLG
8123259	-0,37	-1,29	0,03772	-1,01	-2,02	0,06896	NM_000301	SSX4
8167261	-0,37	-1,29	0,52689	-1,33	-2,51	0,00091	NM_005636	MAST3
8026926	-0,37	-1,29	0,09305	0,37	1,29	0,41262	NM_015016	---
7969675	-0,37	-1,29	0,25321	-1,74	-3,33	0,00627	---	G6PC2
8046116	-0,37	-1,29	0,29294	-0,86	-1,81	0,01194	NM_021176	---
7926563	-0,37	-1,29	0,18581	-0,67	-1,59	0,08991	---	LOC400464
7986428	-0,37	-1,29	0,40255	-0,82	-1,77	0,00046	AK127420	IRF3
8038416	-0,37	-1,29	0,41068	0,89	1,86	0,08640	NM_001571	MS4A6A
7948455	-0,37	-1,29	0,12665	-0,78	-1,72	0,15875	NM_152852	TMEM163
8055350	-0,37	-1,29	0,11361	0,63	1,55	0,13701	NM_030923	CLDN24
8103879	-0,37	-1,29	0,13755	-0,24	-1,18	0,09981	ENST00000329738	RBM43
8055697	-0,37	-1,29	0,27596	-0,70	-1,62	0,02198	NM_198557	SLC22A14
8078772	-0,37	-1,29	0,30709	-0,66	-1,58	0,02521	NM_004803	SNORA38B
8009380	-0,37	-1,29	0,24850	-1,23	-2,35	0,01442	NR_003706	ZNF716
8133018	-0,37	-1,29	0,41986	-1,33	-2,51	0,03827	NM_001159279	DNER
8059580	-0,37	-1,29	0,37336	-1,65	-3,13	0,00464	NM_139072	ZNF782
8162639	-0,37	-1,29	0,06388	-0,41	-1,33	0,02100	NM_001001662	FAM101B
8010915	-0,37	-1,29	0,53205	2,68	6,42	0,00164	NM_182705	---
8163255	-0,37	-1,29	0,31631	-0,74	-1,67	0,00653	---	---
7927595	-0,37	-1,29	0,15271	-0,76	-1,70	0,16662	---	KIAA1524
8089372	-0,37	-1,29	0,55570	1,74	3,34	0,00870	NM_020890	---
7962916	-0,37	-1,29	0,17048	-0,78	-1,72	0,02334	---	SPEF2
8104856	-0,37	-1,29	0,14319	-1,51	-2,86	0,01095	NM_024867	CPXM1
8064539	-0,37	-1,29	0,39269	-1,56	-2,96	0,01658	NM_019609	ADAMTS12
8111387	-0,37	-1,29	0,33171	-1,02	-2,03	0,05742	NM_030955	PAGE2
8167866	-0,37	-1,29	0,68890	-1,00	-2,00	0,18166	NM_207339	RAPGEF1

8164692	-0,37	-1,29	0,02880	-0,46	-1,38	0,03868	AF467069	RUNDC2C
7994576	-0,37	-1,29	0,39495	-0,22	-1,17	0,20230	NR_002939	OCLN
8105908	-0,37	-1,29	0,35075	4,66	25,27	0,00104	NM_002538	---
8115664	-0,37	-1,29	0,32937	-0,09	-1,06	0,84213	---	HIST1H2AH
8117543	-0,37	-1,29	0,54035	-0,95	-1,93	0,00436	NM_080596	C21orf82
8068363	-0,37	-1,29	0,14268	-0,63	-1,54	0,01052	NR_027267	ADAMTSL4
7905233	-0,37	-1,29	0,35899	0,88	1,84	0,07555	NM_019032	C8orf44
8146711	-0,37	-1,29	0,26039	0,08	1,06	0,64399	NM_019607	FGF21
8030105	-0,37	-1,29	0,28431	-0,21	-1,16	0,60108	NM_019113	CYP2C9
7929487	-0,37	-1,29	0,39616	-0,90	-1,87	0,00778	NM_000771	C9orf156
8162706	-0,37	-1,29	0,22228	0,30	1,23	0,26979	NM_016481	GPR151
8114896	-0,37	-1,29	0,05110	-1,31	-2,48	0,06329	NM_194251	COQ3
8128383	-0,37	-1,29	0,10161	1,43	2,69	0,00556	NM_017421	UNC93A
8123388	-0,37	-1,29	0,13054	-1,42	-2,68	0,00480	NM_018974	ESR1
8122840	-0,37	-1,29	0,20994	-1,04	-2,05	0,14577	NM_001122741	C6orf52
8123884	-0,37	-1,29	0,32661	-0,17	-1,12	0,26823	NR_026737	---
8157608	-0,37	-1,29	0,08959	-0,64	-1,56	0,00472	---	HTRA3
8094056	-0,37	-1,29	0,31667	-0,29	-1,22	0,27926	NM_053044	MCM2
8082350	-0,37	-1,29	0,48934	0,59	1,51	0,32511	NM_004526	QTRT1
8025728	-0,37	-1,29	0,27016	1,12	2,17	0,02524	NM_031209	SNORD116-13
7981974	-0,37	-1,29	0,00576	-0,28	-1,22	0,11548	NR_003328	RPL36AP40
7947152	-0,37	-1,30	0,21465	-0,83	-1,78	0,00029	BC058160	HIST3H2BB
7910369	-0,37	-1,30	0,08192	-0,50	-1,41	0,27673	NM_175055	C1orf175
7901634	-0,37	-1,30	0,51787	-1,52	-2,87	0,00618	NR_026782	---
8151252	-0,37	-1,30	0,11584	-0,15	-1,11	0,62145	---	UBE2O
8018708	-0,37	-1,30	0,25703	1,57	2,97	0,00135	NM_022066	TBX20
8138997	-0,37	-1,30	0,27136	1,14	2,20	0,11656	NM_001077653	---
8177273	-0,37	-1,30	0,53241	-0,73	-1,66	0,02675	---	SH3BGRL3
7899153	-0,37	-1,30	0,24155	-0,07	-1,05	0,78685	NM_031286	OR10A4
7938179	-0,37	-1,30	0,54303	-0,58	-1,49	0,00063	NM_207186	ACTR3B
8137474	-0,37	-1,30	0,34493	1,12	2,18	0,01275	NM_020445	CSK
7984871	-0,37	-1,30	0,11094	0,04	1,03	0,94908	NM_004383	TAS2R9
7961245	-0,37	-1,30	0,14528	-1,18	-2,26	0,03951	NM_023917	RNF5P1
8150356	-0,37	-1,30	0,10888	0,68	1,60	0,00556	NR_003129	A2ML1
7953775	-0,37	-1,30	0,01793	-0,91	-1,88	0,00186	NM_144670	LOC121952
7969955	-0,37	-1,30	0,36019	-1,24	-2,36	0,00761	NR_026965	ZNRD1
8179055	-0,37	-1,30	0,63637	3,84	14,33	0,00136	NM_170783	---
7989144	-0,38	-1,30	0,36810	-0,83	-1,78	0,00098	---	---
8042460	-0,38	-1,30	0,12889	-1,31	-2,48	0,00096	---	SNX15
7941111	-0,38	-1,30	0,21923	-0,31	-1,24	0,21521	NM_013306	EEF1DP3
7968411	-0,38	-1,30	0,36833	-0,82	-1,77	0,06235	NR_027062	CCDC150
8047187	-0,38	-1,30	0,00006	-0,42	-1,33	0,00610	NM_001080539	SERHL2
8073596	-0,38	-1,30	0,30179	-0,63	-1,55	0,05791	NM_014509	ODZ4
7950701	-0,38	-1,30	0,18118	-1,15	-2,23	0,00936	NM_001098816	SLCO1A2
7961626	-0,38	-1,30	0,20534	-0,65	-1,57	0,01219	NM_134431	---
7899909	-0,38	-1,30	0,68835	-0,25	-1,19	0,72264	---	C19orf41
8038532	-0,38	-1,30	0,43736	-1,25	-2,38	0,00618	NM_152358	HPR
7997192	-0,38	-1,30	0,30155	-1,06	-2,09	0,01074	NM_020995	ATP2A3
8011516	-0,38	-1,30	0,31418	0,65	1,57	0,33273	NM_174954	C16orf3
8003566	-0,38	-1,30	0,37578	-1,05	-2,07	0,01224	NM_001214	METTL7A
7955441	-0,38	-1,30	0,42157	-0,95	-1,93	0,02977	NM_014033	TM4SF19
8093104	-0,38	-1,30	0,44478	0,00	1,00	0,99129	NM_138461	ANKRD62
8020197	-0,38	-1,30	0,05518	-0,72	-1,64	0,00027	ENST00000314074	SNORD115-8
7982022	-0,38	-1,30	0,07432	-0,71	-1,63	0,00575	NR_003300	SERPINA13
7976506	-0,38	-1,30	0,17753	-0,44	-1,36	0,00573	NR_015340	---
8102513	-0,38	-1,30	0,31198	-0,65	-1,57	0,08734	---	---
8013533	-0,38	-1,30	0,52458	-1,45	-2,74	0,00193	---	GJA9
7915178	-0,38	-1,30	0,07419	-1,01	-2,02	0,00313	NM_030772	GINS3
7996211	-0,38	-1,30	0,57474	1,40	2,64	0,01271	NM_001126129	AKAP14
8169665	-0,38	-1,30	0,15064	-1,07	-2,11	0,00292	NM_178813	C17orf82
8008980	-0,38	-1,30	0,49824	-2,25	-4,76	0,00012	NM_203425	NCRNA00085
8030848	-0,38	-1,30	0,57470	-0,33	-1,25	0,27982	NR_024330	MSTN
8057713	-0,38	-1,30	0,24981	-0,51	-1,42	0,03049	NM_005259	RASL10A
8075211	-0,38	-1,30	0,34678	-1,16	-2,23	0,00031	NM_001007279	---
8168620	-0,38	-1,30	0,06653	-0,98	-1,97	0,08372	---	UBD
8178295	-0,38	-1,30	0,32815	-1,90	-3,73	0,00387	NM_006398	CCR1
8086600	-0,38	-1,30	0,03165	-0,87	-1,83	0,05153	NM_001295	ALAS2
8173135	-0,38	-1,30	0,53364	-0,57	-1,48	0,00079	NM_000032	CKS1B
7905826	-0,38	-1,30	0,37878	1,99	3,98	0,03063	NR_024163	PPAPDC3
8158825	-0,38	-1,30	0,32683	0,12	1,09	0,67501	NM_032728	CAMK2A
8115122	-0,38	-1,30	0,17460	-0,72	-1,65	0,11056	NM_015981	AMPH
8139131	-0,38	-1,30	0,44575	-0,42	-1,34	0,03710	NM_001635	ZNF840P
8063174	-0,38	-1,30	0,30198	-1,08	-2,11	0,20357	ENST00000442886	---

8169154	-0,38	-1,30	0,04803	-0,57	-1,49	0,09274	---	BMP3
8096070	-0,38	-1,30	0,19123	-1,21	-2,32	0,00020	NM_001201	PTPN18
8045182	-0,38	-1,30	0,11314	0,43	1,35	0,44801	NM_014369	---
8122875	-0,38	-1,30	0,16547	-0,56	-1,47	0,20241	---	---
8122240	-0,38	-1,30	0,12763	-1,27	-2,42	0,04632	---	GDA
8155802	-0,38	-1,30	0,46097	-1,05	-2,07	0,04918	NM_004293	P704P
7972983	-0,38	-1,30	0,50733	-1,90	-3,74	0,01296	NM_001145442	CDH20
8021484	-0,38	-1,30	0,11956	-1,34	-2,53	0,04213	NM_031891	CHRNA5
7985213	-0,38	-1,30	0,31666	5,10	34,37	0,00014	NM_000745	KIF9
8086729	-0,38	-1,30	0,30017	-1,00	-2,01	0,04552	NM_182902	FLJ39061
8047505	-0,38	-1,30	0,00058	-1,13	-2,19	0,01442	BC118982	DEF8
7998072	-0,38	-1,30	0,40735	0,25	1,19	0,51757	NM_207514	TMEM27
8171472	-0,38	-1,30	0,26368	0,16	1,11	0,66899	NM_020665	ARNT2
7985285	-0,38	-1,30	0,39371	0,34	1,26	0,12493	NM_014862	---
8151332	-0,38	-1,30	0,23692	-0,74	-1,67	0,04116	---	VILL
8078665	-0,38	-1,30	0,23711	-1,29	-2,44	0,00425	NM_015873	GOLGA6L9
7991722	-0,38	-1,30	0,71068	0,53	1,44	0,47042	NM_198181	IRAK2
8077786	-0,38	-1,30	0,43480	0,73	1,66	0,13852	NM_001570	---
7953871	-0,38	-1,30	0,34313	-0,73	-1,66	0,04459	---	ANXA2P3
7927801	-0,38	-1,30	0,16514	-0,19	-1,14	0,69991	NR_001446	GOLGA9P
7986947	-0,38	-1,30	0,49048	-1,00	-2,00	0,02771	NR_024074	---
8150862	-0,38	-1,30	0,21803	-1,03	-2,04	0,03683	---	RNF157
8018652	-0,38	-1,30	0,50912	3,55	11,70	0,00079	NM_052916	CSTL1
8061382	-0,38	-1,30	0,13525	-0,91	-1,88	0,11500	NM_138283	KIAA1967
8145201	-0,38	-1,30	0,23382	1,32	2,49	0,01208	NM_021174	SNAPC2
8025368	-0,38	-1,30	0,09724	-1,29	-2,45	0,14927	NM_003083	KIFC3
8001587	-0,38	-1,30	0,42856	1,04	2,05	0,13803	NM_005550	OR52E8
7946111	-0,38	-1,30	0,27438	-1,24	-2,36	0,02797	NM_001005168	TDRD1
7930631	-0,38	-1,30	0,14341	-0,82	-1,77	0,00038	NM_198795	PROL1
8095500	-0,38	-1,30	0,18650	-1,08	-2,11	0,00909	NM_021225	C1orf126
7912638	-0,38	-1,30	0,20071	0,22	1,17	0,11707	NR_027136	OR2F1
8136963	-0,38	-1,30	0,44531	-1,61	-3,06	0,00069	NM_012369	---
8109593	-0,38	-1,30	0,22116	-0,80	-1,74	0,02100	---	AGR2
8138381	-0,38	-1,30	0,14977	0,71	1,63	0,07658	NM_006408	KHK
8040802	-0,38	-1,30	0,27737	0,64	1,56	0,34014	NM_000221	DNMT3B
8061746	-0,38	-1,30	0,23954	0,50	1,42	0,34227	NM_006892	C17orf91
8011193	-0,38	-1,30	0,11768	-0,53	-1,45	0,12118	NR_028504	FAM190A
8096425	-0,38	-1,30	0,21942	-0,48	-1,40	0,00123	NM_001145065	---
7969298	-0,38	-1,30	0,43381	-2,22	-4,65	0,01498	---	---
8145827	-0,38	-1,30	0,13164	-0,91	-1,88	0,04734	---	CLN3
8000543	-0,38	-1,30	0,24915	0,70	1,62	0,07084	NM_000086	---
8104729	-0,38	-1,30	0,28099	-1,09	-2,12	0,01057	---	SLC4A10
8045974	-0,38	-1,30	0,00910	-1,14	-2,21	0,03968	NM_001178015	XKR9
8146900	-0,38	-1,30	0,00716	-0,62	-1,53	0,06591	NM_001011720	STARD6
8023394	-0,38	-1,30	0,06042	-0,82	-1,77	0,16212	NM_139171	MIR34A
7912248	-0,38	-1,30	0,17952	-0,57	-1,48	0,00431	NR_029610	TRHR
8147877	-0,38	-1,30	0,40004	-1,01	-2,01	0,04677	NM_003301	TRIM26
8179638	-0,38	-1,30	0,01087	0,99	1,99	0,07022	NM_003449	FLJ16124
8042346	-0,38	-1,30	0,38197	-0,29	-1,23	0,14736	BC033059	PADI4
7898448	-0,38	-1,30	0,10642	-0,85	-1,81	0,07977	NM_012387	PGM5
8155665	-0,38	-1,30	0,32979	-1,37	-2,59	0,01511	NM_021965	PAGE1
8172647	-0,38	-1,30	0,23173	-0,56	-1,47	0,03320	NM_003785	ARL10
8110158	-0,38	-1,31	0,46078	0,24	1,18	0,22311	NM_173664	PPOX
7906688	-0,38	-1,31	0,08151	0,76	1,70	0,12449	NM_001122764	---
8020802	-0,38	-1,31	0,13763	-0,63	-1,55	0,00391	---	---
8042333	-0,38	-1,31	0,20000	-0,14	-1,10	0,16547	---	ZNF713
8132917	-0,38	-1,31	0,26895	0,32	1,25	0,15992	NM_182633	BCHE
8091867	-0,38	-1,31	0,17790	-0,54	-1,46	0,10446	NM_000055	OR51M1
7938000	-0,38	-1,31	0,31965	-1,31	-2,49	0,17628	NM_001004756	EDDM3A
7973093	-0,38	-1,31	0,14810	-0,56	-1,48	0,03958	NM_006683	LOC151121
8055139	-0,38	-1,31	0,36949	-0,46	-1,37	0,03836	AK056598	---
8131707	-0,39	-1,31	0,05820	-0,85	-1,80	0,00151	---	---
8021666	-0,39	-1,31	0,36966	-0,70	-1,63	0,03440	---	C4orf12
8096147	-0,39	-1,31	0,41560	-1,10	-2,15	0,01340	AY250185	FLJ43879
8060020	-0,39	-1,31	0,18322	-1,20	-2,29	0,14158	AK125867	ZC3H15
8057561	-0,39	-1,31	0,41291	-0,74	-1,66	0,06090	NM_018471	PCDH19
8173933	-0,39	-1,31	0,41364	-0,93	-1,90	0,05996	NM_001105243	LOC100133299
8104568	-0,39	-1,31	0,51208	-0,81	-1,75	0,00585	AY358688	CD209
8033445	-0,39	-1,31	0,02155	-0,27	-1,20	0,28857	NR_026692	LIX1
8113278	-0,39	-1,31	0,31201	-0,98	-1,97	0,02660	NM_153234	TSPO
8073612	-0,39	-1,31	0,39942	1,66	3,17	0,07800	NM_000714	C4BPA
7909318	-0,39	-1,31	0,20750	-1,14	-2,21	0,04042	NM_000715	C20orf196
8060796	-0,39	-1,31	0,39294	1,67	3,17	0,00648	BC035800	ACBD6

7922656	-0,39	-1,31	0,17512	0,34	1,26	0,22443	NM_032360	GOLGA9P
7987027	-0,39	-1,31	0,57196	-0,20	-1,15	0,53516	NR_024074	ITGB6
8056184	-0,39	-1,31	0,05170	-0,33	-1,25	0,12731	NM_000888	---
8120824	-0,39	-1,31	0,12312	-1,08	-2,11	0,01021	---	RBP7
7897522	-0,39	-1,31	0,38431	1,83	3,55	0,01214	NM_052960	NUB1
8143937	-0,39	-1,31	0,28237	0,54	1,46	0,06093	NM_016118	OPRD1
7899528	-0,39	-1,31	0,30144	-1,19	-2,28	0,04329	NM_000911	SCAMP4
8024347	-0,39	-1,31	0,12900	0,86	1,82	0,01115	NM_079834	NLRP12
8039096	-0,39	-1,31	0,33321	0,50	1,42	0,10497	NM_144687	LOC100134868
8061483	-0,39	-1,31	0,38851	0,30	1,23	0,58929	NR_004846	CNTNAP3
8155359	-0,39	-1,31	0,09886	1,14	2,20	0,00032	NM_033655	---
8094439	-0,39	-1,31	0,17346	-0,64	-1,56	0,02224	---	OR5H2
8081204	-0,39	-1,31	0,20014	-0,48	-1,40	0,01037	NM_001005482	C19orf75
8030813	-0,39	-1,31	0,45937	-0,70	-1,62	0,01015	AK097554	RASL11B
8095043	-0,39	-1,31	0,08524	-1,23	-2,34	0,00371	NM_023940	TTC38
8073842	-0,39	-1,31	0,15790	1,89	3,71	0,00541	NM_017931	---
8002245	-0,39	-1,31	0,61830	-1,16	-2,23	0,00011	---	MS4A4A
7940237	-0,39	-1,31	0,00012	-0,92	-1,89	0,00572	NM_024021	RIMBP3
8074593	-0,39	-1,31	0,05306	-0,14	-1,10	0,46964	NM_015672	RPL32
8085407	-0,39	-1,31	0,00225	0,80	1,74	0,03399	NM_001007073	GABBR1
8124654	-0,39	-1,31	0,41377	1,00	2,00	0,12692	NM_001470	SNORD115-11
7982028	-0,39	-1,31	0,69780	-2,22	-4,67	0,00112	NR_003303	SNORD115-11
7982064	-0,39	-1,31	0,69780	-2,22	-4,67	0,00112	NR_003303	SNORD115-11
7982078	-0,39	-1,31	0,69780	-2,22	-4,67	0,00112	NR_003303	SNORD115-11
7982092	-0,39	-1,31	0,69780	-2,22	-4,67	0,00112	NR_003303	WFDC8
8066549	-0,39	-1,31	0,14381	-0,92	-1,89	0,00519	NM_130896	ROR2
8162283	-0,39	-1,31	0,06472	-1,35	-2,55	0,02589	NM_004560	---
8082605	-0,39	-1,31	0,44740	-1,29	-2,45	0,06036	---	PSMB10
8002133	-0,39	-1,31	0,13760	0,09	1,06	0,72946	NM_002801	DNAJC14
7963935	-0,39	-1,31	0,37669	1,83	3,55	0,00288	NM_032364	DNAH10
7959696	-0,39	-1,31	0,18248	-1,06	-2,08	0,00672	NM_207437	HHIP
8097628	-0,39	-1,31	0,26034	-1,30	-2,46	0,00371	NM_022475	GABBR1
8179595	-0,39	-1,31	0,26136	1,51	2,86	0,05994	NM_001470	---
8140907	-0,39	-1,31	0,01160	-1,19	-2,28	0,00264	---	LHX9
7908529	-0,39	-1,31	0,10497	-0,89	-1,86	0,11433	NM_020204	CDAN1
7987928	-0,39	-1,31	0,14279	0,49	1,40	0,12248	NM_138477	---
8020804	-0,39	-1,31	0,40218	-0,78	-1,72	0,01613	---	SLC6A18
8104281	-0,39	-1,31	0,30725	-1,47	-2,77	0,00723	NM_182632	CLDN8
8069795	-0,39	-1,31	0,20373	-0,90	-1,87	0,08134	NM_199328	OLFM1
8159220	-0,39	-1,31	0,36805	-1,30	-2,47	0,04361	NM_014279	C7orf43
8141445	-0,39	-1,31	0,10127	1,17	2,25	0,03533	BC015722	---
8107111	-0,39	-1,31	0,18499	-1,09	-2,13	0,01251	---	MYH7
7978021	-0,39	-1,31	0,00463	-1,00	-2,01	0,00324	NM_000257	CTU2
7997810	-0,39	-1,31	0,29667	0,53	1,44	0,39136	NM_001012759	SMTN
8075458	-0,39	-1,31	0,27029	-1,13	-2,18	0,10120	NM_134270	---
7932017	-0,39	-1,31	0,05977	-0,72	-1,65	0,00285	---	KDM6B
8004671	-0,39	-1,31	0,22139	1,35	2,56	0,05254	NM_001080424	RAPGEFL1
8007043	-0,39	-1,31	0,12288	1,75	3,35	0,00303	NM_016339	C2orf89
8053379	-0,39	-1,31	0,37197	-0,19	-1,14	0,56913	AK296608	---
8137044	-0,39	-1,31	0,28007	-1,12	-2,18	0,04115	---	SLC38A11
8056363	-0,39	-1,31	0,09976	-1,16	-2,24	0,00152	NM_173512	DGKZ
7939657	-0,39	-1,31	0,50722	-0,99	-1,99	0,30224	NM_001105540	UBD
8124650	-0,39	-1,31	0,30071	-1,87	-3,66	0,00369	NM_006398	TAL2
8157090	-0,39	-1,31	0,34067	-0,62	-1,53	0,03942	NM_005421	SIPA1L3
8028286	-0,39	-1,31	0,35390	1,93	3,81	0,00573	NM_015073	SUGT1P1
8160712	-0,39	-1,31	0,18817	0,70	1,63	0,10280	NR_003667	CAMKV
8087530	-0,39	-1,31	0,14974	-0,34	-1,27	0,23898	NM_024046	ZAR1L
7970887	-0,39	-1,31	0,14012	-0,82	-1,76	0,00045	NM_001136571	KRT9
8015357	-0,39	-1,31	0,42667	-0,47	-1,38	0,21532	NM_000226	HSF5
8017010	-0,39	-1,31	0,31946	-0,81	-1,75	0,01248	NM_001080439	WDR81
8003639	-0,39	-1,31	0,01125	0,45	1,36	0,42754	NM_001163809	FOXK2
8019521	-0,39	-1,31	0,22014	0,41	1,33	0,47764	NM_004514	AFAP1
8094030	-0,39	-1,31	0,03737	-1,26	-2,39	0,00399	NM_198595	NPM2
8144982	-0,39	-1,31	0,14330	-1,40	-2,64	0,01366	NM_182795	MGC16384
7967636	-0,39	-1,31	0,18228	-0,24	-1,18	0,29814	NR_026666	---
8046018	-0,39	-1,31	0,26164	-0,62	-1,53	0,29264	---	NXF5
8174105	-0,39	-1,31	0,26620	-0,89	-1,85	0,00607	NR_028089	KU-MEL-3
8116707	-0,39	-1,31	0,03185	-0,60	-1,51	0,02763	AY870321	VGLL1
8170179	-0,39	-1,31	0,52200	-1,71	-3,27	0,00386	NM_016267	ANAPC11
8019687	-0,39	-1,31	0,36116	0,29	1,22	0,26248	NM_001002244	---
7956044	-0,39	-1,31	0,16735	-0,45	-1,37	0,10581	---	LLPH
7964739	-0,39	-1,31	0,50405	-0,19	-1,14	0,67682	NM_032338	SCARNA1
7899392	-0,39	-1,31	0,10932	-1,54	-2,91	0,01014	NR_002997	SLC6A3

8110821	-0,39	-1,31	0,43319	-0,52	-1,43	0,11253	NM_001044	---
7951205	-0,39	-1,31	0,00507	-1,03	-2,05	0,03042	---	CR2
7909350	-0,39	-1,31	0,14128	0,30	1,23	0,37684	NM_001006658	PXMP2
7960117	-0,39	-1,31	0,29231	2,58	6,00	0,00919	NM_018663	COQ7
7993608	-0,39	-1,31	0,15006	0,56	1,48	0,23411	NM_016138	CHCHD8
7950294	-0,39	-1,31	0,03586	0,00	1,00	0,98972	NM_016565	OR10G4
7944797	-0,39	-1,31	0,29159	0,01	1,01	0,96711	NM_001004462	LIPE
8037186	-0,39	-1,31	0,33671	-0,82	-1,77	0,13981	NM_005357	FOXO3
8121365	-0,39	-1,31	0,63677	-2,41	-5,32	0,00291	NM_001455	BRS3
8170159	-0,39	-1,31	0,10757	-0,86	-1,82	0,00264	NM_001727	KRT17
8005449	-0,39	-1,31	0,30034	-0,77	-1,71	0,01176	NM_000422	ITGA7
7963880	-0,39	-1,31	0,40166	-0,77	-1,71	0,13473	NM_001144996	OLFML2A
8157804	-0,39	-1,31	0,49067	-0,84	-1,78	0,01125	NM_182487	IFNG
7964787	-0,39	-1,31	0,03851	-0,36	-1,29	0,17825	NM_000619	---
7983979	-0,39	-1,31	0,13147	-0,35	-1,27	0,08835	---	---
8090660	-0,39	-1,31	0,13214	-0,62	-1,53	0,09116	---	PARVB
8073662	-0,39	-1,31	0,30078	0,05	1,04	0,86259	NM_001003828	AGAP4
7933252	-0,39	-1,31	0,51861	2,01	4,02	0,00022	NM_133446	LRRC14
8148923	-0,39	-1,31	0,43576	0,38	1,30	0,28828	NM_014665	OR9G1
7939994	-0,39	-1,31	0,55348	-2,13	-4,37	0,00007	NM_001005213	NDUFS8
7941976	-0,39	-1,31	0,39284	0,55	1,47	0,19304	NM_002496	ADCYAP1R1
8132151	-0,39	-1,31	0,18569	-1,60	-3,04	0,02270	NM_001118	SCARNA4
7920875	-0,39	-1,31	0,56055	-0,21	-1,16	0,62732	NR_003005	---
8104731	-0,39	-1,31	0,28309	-1,34	-2,54	0,00896	---	---
8023462	-0,39	-1,31	0,62468	-1,36	-2,56	0,03435	---	SLN
7951479	-0,39	-1,31	0,12052	-0,84	-1,79	0,04797	NM_003063	---
8157580	-0,39	-1,31	0,12240	-0,94	-1,92	0,00812	---	STGC3
8079740	-0,39	-1,31	0,13616	-0,86	-1,81	0,13764	AY078383	TUBB3
7998063	-0,39	-1,31	0,31367	0,67	1,59	0,39495	NM_006086	BPESC1
8083025	-0,39	-1,31	0,24666	-1,16	-2,24	0,00995	NR_026783	---
8017597	-0,39	-1,31	0,43246	-1,43	-2,69	0,00080	---	TAF4
8067361	-0,39	-1,31	0,43800	0,43	1,35	0,33776	NM_003185	CD1B
7921346	-0,39	-1,31	0,27459	-1,93	-3,81	0,00001	NM_001764	OR4C13
7939900	-0,39	-1,31	0,71992	-1,43	-2,70	0,00526	NM_001001955	GH2
8017480	-0,40	-1,31	0,33239	-0,82	-1,77	0,01161	NM_022557	DDX51
7967711	-0,40	-1,32	0,18340	-0,73	-1,66	0,24475	NM_175066	ADORA1
7908907	-0,40	-1,32	0,18059	-0,28	-1,21	0,42624	NM_000674	SNAI1
8063382	-0,40	-1,32	0,12253	0,80	1,75	0,25530	NM_005985	VSNL1
8040430	-0,40	-1,32	0,19436	-1,34	-2,54	0,00911	NM_003385	CEBPZ
8051528	-0,40	-1,32	0,09616	0,15	1,11	0,44992	NM_005760	LPIN3
8062658	-0,40	-1,32	0,01082	-0,50	-1,42	0,11784	NM_022896	IGF2BP1
8008151	-0,40	-1,32	0,38356	-0,78	-1,72	0,00039	NM_006546	LOC642947
8156022	-0,40	-1,32	0,39692	-0,87	-1,82	0,22422	AK129705	GABBR1
8178298	-0,40	-1,32	0,27228	1,41	2,65	0,06593	NM_001470	DEFA6
8149104	-0,40	-1,32	0,24144	-1,83	-3,56	0,01292	NM_001926	---
7938625	-0,40	-1,32	0,33457	-0,76	-1,69	0,01902	---	PIGL
8005157	-0,40	-1,32	0,01852	0,35	1,27	0,31014	NM_004278	CLDN18
8082928	-0,40	-1,32	0,29600	-1,82	-3,53	0,00140	NM_001002026	LOC100128818
8076815	-0,40	-1,32	0,57402	-1,74	-3,33	0,02219	AY358194	---
8092931	-0,40	-1,32	0,51439	-1,28	-2,43	0,10015	---	C1orf158
7897966	-0,40	-1,32	0,00053	-1,16	-2,23	0,02639	NM_152290	LRRC7
7902235	-0,40	-1,32	0,13561	-1,55	-2,93	0,00172	NM_020794	---
8113707	-0,40	-1,32	0,22848	-1,15	-2,22	0,01987	---	LOC100128644
8085429	-0,40	-1,32	0,43142	-1,01	-2,02	0,00346	AY358240	PKP2
7962212	-0,40	-1,32	0,41357	3,31	9,95	0,00001	NM_004572	---
8096873	-0,40	-1,32	0,05785	-1,02	-2,02	0,05553	---	KCNK6
8028332	-0,40	-1,32	0,52219	-0,33	-1,26	0,30617	NM_004823	---
7979811	-0,40	-1,32	0,63917	-1,87	-3,67	0,10387	---	TMEM8C
8164931	-0,40	-1,32	0,22174	-1,66	-3,17	0,00580	NM_001080483	---
8090505	-0,40	-1,32	0,15122	-0,69	-1,61	0,00048	---	---
7979572	-0,40	-1,32	0,20284	-1,54	-2,91	0,07192	---	ASAH2
7933341	-0,40	-1,32	0,21217	-0,23	-1,17	0,53349	NM_019893	ETAA1
8042373	-0,40	-1,32	0,12315	0,32	1,25	0,23653	NM_019002	FAM69C
8023864	-0,40	-1,32	0,03523	-0,85	-1,81	0,03084	NM_001044369	CNR1
8128043	-0,40	-1,32	0,10745	-1,08	-2,12	0,00239	NM_016083	RAI1
8005267	-0,40	-1,32	0,00313	-0,35	-1,27	0,40907	NM_030665	GOLGA2B
7965746	-0,40	-1,32	0,23453	-0,54	-1,46	0,05908	NM_017600	LOC399898
7940116	-0,40	-1,32	0,29188	-0,78	-1,72	0,17446	AK128188	HSP90AA5P
8084299	-0,40	-1,32	0,42622	0,01	1,01	0,92672	AY956761	---
8165864	-0,40	-1,32	0,06883	-1,33	-2,52	0,01321	---	SNORD103A
7914322	-0,40	-1,32	0,23317	-1,11	-2,16	0,06416	NR_004054	SNORD103A
7914324	-0,40	-1,32	0,23317	-1,11	-2,16	0,06416	NR_004054	ZNF165
8117630	-0,40	-1,32	0,18861	1,04	2,06	0,15918	NM_003447	OR2AJ1

7911235	-0,40	-1,32	0,26697	-1,14	-2,21	0,12857	ENST00000318244	JDP2
7975787	-0,40	-1,32	0,25439	-0,12	-1,09	0,66916	NM_001135049	TMEM132B
7959807	-0,40	-1,32	0,38192	-1,20	-2,30	0,00235	NM_052907	ZFP41
8148597	-0,40	-1,32	0,36858	-0,59	-1,50	0,21467	NM_173832	SPATA22
8011343	-0,40	-1,32	0,25102	-0,78	-1,72	0,00068	NM_032598	SPRR2A
7920205	-0,40	-1,32	0,28664	-2,05	-4,13	0,01973	NM_005988	C6orf226
8126446	-0,40	-1,32	0,02981	-0,77	-1,71	0,18630	NM_001008739	TRPM3
8161654	-0,40	-1,32	0,07599	-0,89	-1,86	0,00000	NM_206946	IFNA4
8160377	-0,40	-1,32	0,32034	-1,42	-2,68	0,00145	NM_021068	JAKMIP2
8114938	-0,40	-1,32	0,21967	-0,94	-1,91	0,01733	NM_014790	RAD21L1
8060395	-0,40	-1,32	0,32995	-0,75	-1,68	0,03218	NM_001136566	KCNK16
8126173	-0,40	-1,32	0,19391	-1,23	-2,35	0,00091	NM_001135105	LRP1B
8055496	-0,40	-1,32	0,11104	-0,60	-1,51	0,00150	NM_018557	KLRF1
7953892	-0,40	-1,32	0,12786	1,67	3,18	0,11293	NM_016523	SIGLEC6
8038861	-0,40	-1,32	0,46008	-0,72	-1,65	0,04231	NM_001245	HMGB3L1
8065795	-0,40	-1,32	0,20368	-1,29	-2,44	0,02256	NR_002165	PNMAL1
8037767	-0,40	-1,32	0,22304	-1,01	-2,01	0,00656	NM_018215	FLJ00290
8148978	-0,40	-1,32	0,20231	-0,55	-1,47	0,19028	AK090404 ---	
7906467	-0,40	-1,32	0,25082	-0,79	-1,73	0,12469	--- CD40	
8063156	-0,40	-1,32	0,19455	-0,13	-1,09	0,58096	NM_001250	RTL1
7981322	-0,40	-1,32	0,22737	-0,81	-1,75	0,09863	NM_001134888	HIP1
8140319	-0,40	-1,32	0,02309	0,92	1,90	0,05064	NM_005338	CRYBA4
8072124	-0,40	-1,32	0,49838	-0,86	-1,81	0,13747	NM_001886	AP1B1
8075217	-0,40	-1,32	0,10471	1,86	3,64	0,00666	NM_001127	MIR106A
8175252	-0,40	-1,32	0,01651	-0,75	-1,68	0,00112	NR_029523	---
7932608	-0,40	-1,32	0,03540	-1,34	-2,52	0,00852	--- OR2T29	
7925747	-0,40	-1,32	0,09526	-0,94	-1,91	0,10344	NM_001004694	PCDH15
7933672	-0,40	-1,32	0,16112	-0,76	-1,69	0,00971	NM_001142763	HOXC6
7955873	-0,40	-1,32	0,18227	-0,14	-1,10	0,60626	NM_004503	PLCB3
7940924	-0,40	-1,32	0,15585	1,59	3,02	0,02381	NM_000932	PTPRU
7899562	-0,40	-1,32	0,41527	1,11	2,15	0,04308	NM_133178	---
8122969	-0,40	-1,32	0,07014	-1,00	-2,00	0,10284	--- KCNE2	
8068369	-0,40	-1,32	0,24818	-0,67	-1,59	0,03096	NM_172201	C3orf43
8093137	-0,40	-1,32	0,24024	-0,96	-1,95	0,02179	NM_001077657	PELP1
8011692	-0,40	-1,32	0,22217	0,11	1,08	0,85979	NM_014389	ENPP2
8152522	-0,40	-1,32	0,34402	0,87	1,83	0,05810	NM_006209	LOC100131217
8036862	-0,40	-1,32	0,13711	-0,77	-1,71	0,06899	AY358191 ---	
7942551	-0,40	-1,32	0,05914	-0,26	-1,19	0,17911	--- ---	
7918424	-0,40	-1,32	0,15154	-1,35	-2,55	0,03365	--- CAMP	
8079590	-0,40	-1,32	0,29352	-0,51	-1,42	0,04512	NM_004345	---
8083850	-0,40	-1,32	0,28379	-0,11	-1,08	0,83659	--- S100A14	
7920297	-0,40	-1,32	0,03428	-0,24	-1,18	0,35966	NM_020672	SPDYE4
8012528	-0,40	-1,32	0,43240	-1,90	-3,72	0,00027	NM_001128076	ANKRD37
8098604	-0,40	-1,32	0,21308	-1,01	-2,02	0,11688	NM_181726	C11orf41
7939215	-0,40	-1,32	0,37619	0,29	1,23	0,45538	NM_012194	BET3L
8129105	-0,40	-1,32	0,26392	-0,99	-1,99	0,07088	NM_001139444	CXorf58
8166481	-0,40	-1,32	0,24164	-0,73	-1,66	0,01219	NM_152761	SF1
7949146	-0,40	-1,32	0,10519	0,04	1,03	0,79177	NM_004630	---
7917695	-0,40	-1,32	0,19556	-0,78	-1,72	0,00409	--- ADAMTS4	
7921821	-0,40	-1,32	0,16076	-1,35	-2,55	0,10343	NM_005099	ADAM28
8145293	-0,40	-1,32	0,09683	-0,96	-1,95	0,00305	NM_014265	CCDC90B
7950753	-0,40	-1,32	0,13360	1,14	2,20	0,00873	NM_021825	ZNF821
8002618	-0,40	-1,32	0,00189	-0,13	-1,10	0,64613	NM_017530	OR7G2
8033734	-0,40	-1,32	0,36396	-0,83	-1,78	0,00032	NM_001005193	CRISP3
8126905	-0,40	-1,32	0,25581	-0,92	-1,89	0,05322	NM_006061	---
8111991	-0,40	-1,32	0,26817	-1,02	-2,02	0,01755	--- GALR2	
8010030	-0,40	-1,32	0,25344	-0,93	-1,90	0,19914	NM_003857	SAA4
7946977	-0,40	-1,32	0,27979	-0,35	-1,27	0,26632	NM_006512	ZNF556
8024527	-0,40	-1,32	0,23234	-0,91	-1,88	0,00097	NM_024967	FBXO40
8081997	-0,40	-1,32	0,10486	-0,66	-1,58	0,03552	NM_016298	---
7900197	-0,40	-1,32	0,52617	-0,70	-1,63	0,03816	--- UFM1	
7971124	-0,40	-1,32	0,34431	-0,44	-1,36	0,09445	NM_016617	TMEM59L
8027040	-0,40	-1,32	0,45914	-0,25	-1,19	0,57361	NM_012109	C1orf230
7905464	-0,41	-1,32	0,15885	-0,46	-1,37	0,11396	NM_001144956	C9orf117
8158081	-0,41	-1,32	0,30187	-0,95	-1,93	0,00115	BC141809 ---	
7986637	-0,41	-1,32	0,32087	-2,98	-7,88	0,02018	--- SALL3	
8021826	-0,41	-1,32	0,49425	-1,70	-3,25	0,00641	NM_171999	U2AF1L4
8036143	-0,41	-1,32	0,41062	1,85	3,62	0,07090	NM_001040425	SYNE2
7974920	-0,41	-1,32	0,01555	1,89	3,71	0,02401	NM_182914	MPP2
8015868	-0,41	-1,32	0,11351	0,82	1,77	0,03948	NM_005374	TOMM20L
7974647	-0,41	-1,33	0,00278	0,20	1,15	0,17635	NM_207377	MEGF10
8107722	-0,41	-1,33	0,28184	-1,32	-2,51	0,01404	NM_032446	LOC284023
8012282	-0,41	-1,33	0,41966	0,09	1,07	0,55022	NR_024349	---

7976808	-0,41	-1,33	0,32494	-0,72	-1,65	0,07943	---	AUTS2
8133233	-0,41	-1,33	0,04075	-0,86	-1,82	0,00784	NM_015570	BTBD12
7999008	-0,41	-1,33	0,09974	0,85	1,80	0,04628	NM_032444	---
8117077	-0,41	-1,33	0,04003	-0,95	-1,93	0,05579	---	MAGEB18
8166565	-0,41	-1,33	0,16044	-1,02	-2,03	0,00141	NM_173699	SNORD115-35
7982076	-0,41	-1,33	0,19509	-0,72	-1,64	0,02627	NR_003350	---
8143207	-0,41	-1,33	0,02110	-0,86	-1,81	0,00950	---	---
7915565	-0,41	-1,33	0,10310	-1,52	-2,86	0,01845	---	MUC19
7954864	-0,41	-1,33	0,14735	-0,97	-1,96	0,05669	AK093065	DMPK
8037657	-0,41	-1,33	0,04848	0,73	1,66	0,20480	NM_004409	LOC100288814
8165945	-0,41	-1,33	0,26234	-1,45	-2,74	0,00003	ENST00000445307	TRMT1
8034615	-0,41	-1,33	0,33880	0,34	1,27	0,27517	NM_017722	LOC401410
8143437	-0,41	-1,33	0,16528	-1,79	-3,45	0,10277	BX648692	FLJ45832
8065547	-0,41	-1,33	0,00460	-0,69	-1,62	0,00077	AK127732	OPTC
7908931	-0,41	-1,33	0,40731	-1,38	-2,59	0,02191	NM_014359	TMEM26
7933842	-0,41	-1,33	0,26527	-1,24	-2,37	0,00001	NM_178505	RABEPK
8157843	-0,41	-1,33	0,19830	0,98	1,97	0,00808	NM_005833	FLJ11710
8008700	-0,41	-1,33	0,39045	-0,75	-1,69	0,04313	AK021772	KRT34
8015268	-0,41	-1,33	0,44713	-0,69	-1,61	0,26178	NM_021013	SLC5A12
7947165	-0,41	-1,33	0,02019	-1,25	-2,38	0,00001	NM_178498	TRIM14
8162729	-0,41	-1,33	0,52054	1,09	2,12	0,00663	NM_014788	CCDC121
8051197	-0,41	-1,33	0,63208	-0,83	-1,77	0,27331	NM_024584	---
7988438	-0,41	-1,33	0,01670	-0,98	-1,97	0,04310	---	KBTBD7
7971218	-0,41	-1,33	0,01942	0,06	1,04	0,87178	NM_032138	ADAMTSL4
7919743	-0,41	-1,33	0,29802	-0,28	-1,22	0,31402	NM_019032	BBS12
8097252	-0,41	-1,33	0,30925	-0,53	-1,44	0,03962	NM_001178007	CBR3
8068413	-0,41	-1,33	0,17977	-0,31	-1,24	0,56340	NM_001236	---
7965787	-0,41	-1,33	0,04049	-0,50	-1,42	0,04078	---	RPS20P27
7950626	-0,41	-1,33	0,23684	0,54	1,45	0,10931	BC071734	CD27
7953333	-0,41	-1,33	0,33988	-0,73	-1,66	0,00596	NM_001242	RFXAP
7968653	-0,41	-1,33	0,22819	-0,44	-1,36	0,00253	NM_000538	ZNF664
7959751	-0,41	-1,33	0,18925	0,54	1,45	0,05224	NM_152437	SCN2B
7952011	-0,41	-1,33	0,34842	-1,67	-3,18	0,00949	NM_004588	---
7938055	-0,41	-1,33	0,20083	-1,21	-2,31	0,01076	---	CCDC62
7959484	-0,41	-1,33	0,18861	-1,24	-2,36	0,07317	NM_201435	IMPDH1
8142795	-0,41	-1,33	0,35224	0,21	1,16	0,10501	NM_000883	CROCCL2
7912784	-0,41	-1,33	0,06463	-0,20	-1,15	0,31084	NR_023386	PIM1
8119161	-0,41	-1,33	0,23745	0,56	1,47	0,22187	NM_002648	CLCN4
8165974	-0,41	-1,33	0,18225	0,57	1,49	0,02920	NM_001830	EGLN2
8028940	-0,41	-1,33	0,17694	-0,18	-1,13	0,34081	NM_080732	---
8170362	-0,41	-1,33	0,45551	-0,64	-1,55	0,13598	---	SNX21
8063057	-0,41	-1,33	0,01713	-0,67	-1,60	0,08168	NM_033421	ANKRD13D
7941822	-0,41	-1,33	0,16626	1,09	2,13	0,04412	NR_030767	ANKRD23
8053984	-0,41	-1,33	0,23408	-0,43	-1,35	0,28897	NM_144994	NEURL2
8066612	-0,41	-1,33	0,24806	-0,15	-1,11	0,52660	NM_080749	SORL1
7944667	-0,41	-1,33	0,00937	1,24	2,35	0,00564	NM_003105	NFATC2
8067040	-0,41	-1,33	0,29710	1,97	3,93	0,00196	NM_012340	RAG1
7939398	-0,41	-1,33	0,31945	-0,59	-1,50	0,05541	NM_000448	SPATA19
7952797	-0,41	-1,33	0,20883	-1,36	-2,57	0,00075	NM_174927	---
7928870	-0,41	-1,33	0,35101	-1,01	-2,01	0,01674	---	MLANA
8154285	-0,41	-1,33	0,23082	-1,06	-2,09	0,00383	NM_005511	CPXCR1
8168638	-0,41	-1,33	0,04414	-1,09	-2,12	0,00532	NM_033048	---
8097690	-0,41	-1,33	0,28075	-0,87	-1,83	0,00028	---	OR7E35P
8094154	-0,41	-1,33	0,48177	-0,85	-1,80	0,03207	AF073924	PRMT7
7996785	-0,41	-1,33	0,17232	0,50	1,42	0,14583	NM_019023	FOLH1
7948058	-0,41	-1,33	0,09995	-1,22	-2,32	0,00074	NM_004476	---
8116954	-0,41	-1,33	0,03217	-0,88	-1,84	0,00336	---	LOC100129345
7981237	-0,41	-1,33	0,01701	-1,13	-2,18	0,08021	ENST00000355909	---
8129454	-0,41	-1,33	0,73288	-2,40	-5,29	0,00631	---	LAMP3
8092348	-0,41	-1,33	0,31064	2,50	5,65	0,00151	NM_014398	SLC9A3R2
7992463	-0,41	-1,33	0,04516	-0,05	-1,04	0,89142	NM_001130012	ZNF778
7997896	-0,41	-1,33	0,32062	1,45	2,73	0,01855	NM_182531	---
8132243	-0,41	-1,33	0,00969	-0,40	-1,32	0,13000	---	VWA3B
8043747	-0,41	-1,33	0,06580	-0,91	-1,87	0,00467	NM_144992	LOC440792
8071357	-0,41	-1,33	0,32982	-1,42	-2,67	0,03448	AB051440	FAM55D
7951796	-0,41	-1,33	0,15991	-0,67	-1,59	0,04229	NM_001077639	UBXN2A
8040503	-0,41	-1,33	0,02912	0,61	1,52	0,03409	NM_181713	10. Mrz
8017365	-0,41	-1,33	0,17128	-0,50	-1,42	0,08263	NM_152598	---
8102726	-0,41	-1,33	0,23952	-1,11	-2,16	0,00670	---	---
7923965	-0,41	-1,33	0,08468	-0,44	-1,36	0,31922	---	LSMD1
8012274	-0,41	-1,33	0,50572	0,56	1,48	0,16128	NM_032356	ENPP3
8122071	-0,41	-1,33	0,13082	0,45	1,36	0,05173	NM_005021	BTN2A2
8117447	-0,41	-1,33	0,13688	-0,27	-1,20	0,32652	NM_006995	NAPRT1

8153430	-0,41	-1,33	0,21276	1,24	2,37	0,13511	NM_145201	---
8017096	-0,41	-1,33	0,10257	-0,78	-1,72	0,00169	---	IFFO1
7960559	-0,41	-1,33	0,29784	-0,98	-1,98	0,00102	NM_080730	OR1J2
8157668	-0,41	-1,33	0,41570	-0,89	-1,85	0,00828	NM_054107	SNORA56
8170992	-0,41	-1,33	0,31247	-0,92	-1,90	0,14610	NR_002984	TAB1
8073114	-0,41	-1,33	0,10675	0,27	1,21	0,16324	NM_006116	ZNF365
7927747	-0,41	-1,33	0,06765	-0,90	-1,87	0,00007	NM_014951	RNU5B-1
7984259	-0,41	-1,33	0,45103	-0,90	-1,86	0,13849	NR_002757	LCN1
8159255	-0,41	-1,33	0,59204	-1,91	-3,75	0,00082	NM_002297	CXorf42
8174761	-0,41	-1,33	0,25010	-0,75	-1,68	0,00378	NR_027131	CCDC147
7930320	-0,41	-1,33	0,26202	-0,74	-1,67	0,06037	NM_001008723	COG8
8002272	-0,41	-1,33	0,31399	0,22	1,17	0,73815	NM_032382	FLJ45949
8030964	-0,41	-1,33	0,14157	-1,41	-2,66	0,00036	AK127846	LYZL1
7926889	-0,41	-1,33	0,69962	-0,36	-1,28	0,30793	NM_032517	ACCN5
8103378	-0,41	-1,33	0,11359	-0,53	-1,44	0,06404	NM_017419	MUC21
8177931	-0,41	-1,33	0,53886	-1,56	-2,95	0,00024	NM_001010909	SNORD77
7922412	-0,41	-1,33	0,39457	-0,66	-1,58	0,01149	NR_003943	---
8071655	-0,41	-1,33	0,45193	-0,87	-1,83	0,21629	---	CCDC67
7943103	-0,41	-1,33	0,00396	-0,69	-1,62	0,07521	NM_181645	CSRNP1
8086330	-0,41	-1,33	0,04306	-0,57	-1,48	0,09077	NM_033027	---
8101224	-0,41	-1,33	0,25080	-0,40	-1,32	0,50498	---	CD1C
7906348	-0,41	-1,33	0,28061	-1,24	-2,37	0,00530	NM_001765	SNORD115-11
7982050	-0,42	-1,33	0,67068	-1,88	-3,69	0,00111	NR_003303	TMEM115
8087685	-0,42	-1,33	0,45319	2,46	5,49	0,05300	NM_007024	EPM2A
8130013	-0,42	-1,33	0,24274	-0,17	-1,12	0,15027	NM_005670	LMNB2
8032491	-0,42	-1,33	0,32315	2,00	4,01	0,03068	NM_032737	APITD1
7897632	-0,42	-1,33	0,22789	-0,30	-1,23	0,22754	NM_198544	ZNF671
8039680	-0,42	-1,33	0,24013	-0,44	-1,36	0,34569	NM_024833	---
7957604	-0,42	-1,33	0,37671	-0,29	-1,22	0,62241	---	CBX7
8076185	-0,42	-1,33	0,09465	1,78	3,42	0,01022	NM_175709	VMAC
8025000	-0,42	-1,33	0,03392	0,02	1,01	0,92930	NM_001017921	GTF3C1
8000425	-0,42	-1,33	0,13596	1,59	3,00	0,00459	NM_001520	TAS2R20
7961285	-0,42	-1,33	0,14192	-0,27	-1,21	0,01511	NM_176889	---
8098902	-0,42	-1,33	0,60281	-0,55	-1,47	0,10635	---	INSC
7938652	-0,42	-1,33	0,32142	-0,63	-1,55	0,02399	NM_001031853	---
8129835	-0,42	-1,33	0,22408	-0,94	-1,92	0,04782	---	CFD
8024062	-0,42	-1,33	0,31624	0,88	1,85	0,07924	NM_001928	IL1RL1
8044021	-0,42	-1,34	0,05852	0,47	1,39	0,12106	NM_016232	OR10A3
7946332	-0,42	-1,34	0,15379	-0,91	-1,87	0,02926	NM_001003745	---
7932548	-0,42	-1,34	0,10841	-0,94	-1,91	0,00809	---	EIF4E
8101839	-0,42	-1,34	0,21311	-0,40	-1,32	0,24363	NM_001968	FXYD6
7951977	-0,42	-1,34	0,45573	0,49	1,40	0,25260	NM_001164836	SLC15A5
7961571	-0,42	-1,34	0,14284	-1,04	-2,06	0,02915	NM_001170798	NLRP7
8039280	-0,42	-1,34	0,14983	-0,85	-1,81	0,05662	NM_001127255	ACACB
7958466	-0,42	-1,34	0,03479	1,05	2,07	0,00473	NM_001093	---
7943317	-0,42	-1,34	0,23844	-0,35	-1,27	0,06345	---	EMR1
8025103	-0,42	-1,34	0,20146	-1,02	-2,03	0,00002	NM_001974	---
8132830	-0,42	-1,34	0,69194	-0,35	-1,28	0,47415	---	---
8043833	-0,42	-1,34	0,12450	0,10	1,07	0,55947	---	TAAR5
8129604	-0,42	-1,34	0,18992	-1,43	-2,69	0,10659	NM_003967	---
8097645	-0,42	-1,34	0,14708	-0,81	-1,76	0,02242	---	FLJ25758
8025124	-0,42	-1,34	0,10900	-1,56	-2,96	0,00830	NR_024372	---
7971075	-0,42	-1,34	0,11338	-0,91	-1,87	0,00323	---	---
8032751	-0,42	-1,34	0,25898	-0,57	-1,48	0,02220	---	---
8162249	-0,42	-1,34	0,39026	-0,26	-1,20	0,10947	---	---
8012891	-0,42	-1,34	0,26752	-0,27	-1,21	0,13023	---	OR4C3
7939873	-0,42	-1,34	0,41475	-0,97	-1,96	0,00262	NM_001004702	HTR1D
7913566	-0,42	-1,34	0,32171	3,42	10,68	0,00658	NM_000864	COQ4
8158214	-0,42	-1,34	0,39923	0,46	1,38	0,13218	NM_016035	MTCP1
8176219	-0,42	-1,34	0,15445	1,12	2,18	0,01293	NM_001018025	SCEL
7969493	-0,42	-1,34	0,10909	2,31	4,97	0,01334	NM_144777	MYL10
8141737	-0,42	-1,34	0,11343	-0,88	-1,84	0,12600	NM_138403	SNORD34
8030364	-0,42	-1,34	0,09739	-0,79	-1,72	0,31682	NR_000019	KIF21B
7923233	-0,42	-1,34	0,11268	0,43	1,35	0,26239	NM_017596	RMI1
8156126	-0,42	-1,34	0,28799	1,03	2,05	0,05647	NM_024945	---
8168727	-0,42	-1,34	0,28323	-1,09	-2,13	0,02756	---	ADH6
8101862	-0,42	-1,34	0,13198	-1,02	-2,03	0,00002	NM_001102470	LOC100131613
7964868	-0,42	-1,34	0,38585	-1,08	-2,12	0,00179	ENST00000451128	---
8149322	-0,42	-1,34	0,14465	-1,28	-2,43	0,00946	---	LOC100131581
8144701	-0,42	-1,34	0,52139	-0,79	-1,73	0,15046	AK092544	TSSK1B
8113573	-0,42	-1,34	0,41558	-0,86	-1,81	0,00098	NM_032028	C17orf90
8019238	-0,42	-1,34	0,44010	0,84	1,79	0,08443	BC090923	TUBA3C
7970395	-0,42	-1,34	0,28419	-0,89	-1,85	0,00688	NM_006001	SOX9

8009517	-0,42	-1,34	0,37871	-0,45	-1,37	0,13845	NM_000346	IGSF1
8175121	-0,42	-1,34	0,13952	-0,83	-1,78	0,00479	NM_001555	OR4C12
7948103	-0,42	-1,34	0,26702	-0,96	-1,94	0,01068	NM_001005270	ANKRD55
8112159	-0,42	-1,34	0,39155	-1,33	-2,52	0,00009	NM_024669	CNTNAP2
8137010	-0,42	-1,34	0,00346	-0,34	-1,27	0,08190	NM_014141	APBA2
7982187	-0,42	-1,34	0,07170	1,22	2,33	0,01119	NM_005503	ABCA10
8009474	-0,42	-1,34	0,00358	-1,26	-2,40	0,02252	NM_080282	---
8117620	-0,42	-1,34	0,49223	-0,33	-1,26	0,04616	---	OR10J1
7906452	-0,42	-1,34	0,18453	-1,64	-3,11	0,01611	NM_012351	---
8052056	-0,42	-1,34	0,01786	-0,42	-1,34	0,08567	---	---
8129039	-0,42	-1,34	0,23062	-0,46	-1,37	0,29702	---	---
8022168	-0,42	-1,34	0,08738	-1,38	-2,60	0,00529	---	---
7912525	-0,42	-1,34	0,02312	-0,50	-1,41	0,00175	---	---
8092638	-0,42	-1,34	0,19214	-0,85	-1,80	0,00302	---	FXYD1
8027760	-0,42	-1,34	0,17062	-1,90	-3,74	0,00244	NM_005031	---
8121112	-0,42	-1,34	0,27381	-1,39	-2,61	0,03884	---	DAGLA
7940508	-0,42	-1,34	0,05988	1,09	2,13	0,01143	NM_006133	---
8017100	-0,42	-1,34	0,33139	-0,49	-1,40	0,01994	---	FKBP1B
8040530	-0,42	-1,34	0,32517	-0,35	-1,27	0,30831	NM_054033	OR4K2
7973012	-0,42	-1,34	0,34068	-1,19	-2,28	0,00917	NM_001005501	GABRG1
8100097	-0,42	-1,34	0,01933	-1,31	-2,48	0,00048	NM_173536	C8orf54
8152734	-0,42	-1,34	0,31811	-1,16	-2,23	0,00001	BC137357	COQ6
7980069	-0,42	-1,34	0,10091	-0,60	-1,51	0,05456	NM_182480	PPM1N
8029701	-0,42	-1,34	0,12165	0,09	1,07	0,71065	NM_001080401	LYAR
8099107	-0,42	-1,34	0,00011	1,13	2,19	0,05480	NM_017816	LCE2C
7905503	-0,42	-1,34	0,52499	-1,13	-2,20	0,17529	NM_178429	OR1J4
8157671	-0,42	-1,34	0,05251	-0,80	-1,75	0,00953	NM_001004452	CDC14C
8132803	-0,42	-1,34	0,32928	-1,18	-2,27	0,07341	NR_003595	FMO9P
7907012	-0,42	-1,34	0,35027	-0,46	-1,38	0,10516	NR_002925	OR2B6
8117622	-0,42	-1,34	0,36665	-0,83	-1,78	0,11818	NM_012367	TNIP1
8115210	-0,42	-1,34	0,31713	0,82	1,76	0,00926	NM_006058	PIN4
8168366	-0,42	-1,34	0,36879	0,26	1,20	0,44867	NM_006223	SSX2
8172787	-0,42	-1,34	0,53261	-1,58	-2,99	0,04214	NM_003147	OR6P1
7921362	-0,42	-1,34	0,38943	-2,53	-5,79	0,00192	NM_001160325	ATP5S
7974257	-0,42	-1,34	0,11664	0,09	1,06	0,88974	NM_015684	PIGO
8160953	-0,42	-1,34	0,38011	1,73	3,31	0,02636	NM_032634	TARBP2
7955829	-0,42	-1,34	0,06324	0,46	1,37	0,37652	NM_134323	---
7903876	-0,42	-1,34	0,26414	-0,73	-1,66	0,07711	---	---
8060741	-0,42	-1,34	0,31755	0,23	1,18	0,26633	---	RNASE10
7973078	-0,42	-1,34	0,30532	-0,67	-1,59	0,06836	NM_001012975	SCARNA10
7953383	-0,42	-1,34	0,16260	-0,34	-1,27	0,17427	NR_004387	FAM108A1
8074842	-0,42	-1,34	0,35658	0,85	1,81	0,18098	NM_001130111	LOC284581
7909144	-0,42	-1,34	0,24844	-2,12	-4,36	0,00287	AK094426	LOC93463
8059902	-0,42	-1,34	0,43448	-1,95	-3,87	0,00024	AL122100	---
7970392	-0,42	-1,34	0,85859	-5,69	-51,77	0,00193	---	C10orf71
7927474	-0,42	-1,34	0,02113	-0,94	-1,92	0,01280	NM_001135196	TLE3
7990033	-0,42	-1,34	0,12474	0,06	1,05	0,87786	NM_005078	EGFLAM
8105013	-0,42	-1,34	0,32272	-1,23	-2,35	0,00080	NM_152403	NR4A1
7955589	-0,42	-1,34	0,13227	1,43	2,69	0,01048	NM_002135	C7orf72
8132813	-0,42	-1,34	0,38156	-0,75	-1,68	0,02152	NM_001161834	---
7902787	-0,42	-1,34	0,35117	-0,69	-1,61	0,08103	---	OR2A9P
8143629	-0,42	-1,34	0,61110	1,39	2,63	0,00796	NR_002157	SLC16A13
8004266	-0,42	-1,34	0,08025	2,56	5,92	0,04086	NM_201566	DSCAM
8070421	-0,42	-1,34	0,09549	-1,52	-2,87	0,00188	NM_001389	---
7989489	-0,42	-1,34	0,05422	-1,23	-2,34	0,04346	---	TUBB2A
8123644	-0,42	-1,34	0,16146	-1,66	-3,17	0,00046	NM_001069	FLJ41484
7972932	-0,42	-1,34	0,40498	-0,85	-1,80	0,03280	AK092862	GDPD2
8168163	-0,42	-1,34	0,28117	-0,80	-1,74	0,05620	NM_001171192	DACT1
7974689	-0,42	-1,34	0,09310	-1,46	-2,76	0,00094	NM_016651	LAMC2
7908072	-0,42	-1,34	0,20479	2,67	6,35	0,00011	NM_005562	CCDC63
7958711	-0,43	-1,34	0,01257	-0,63	-1,55	0,05282	NM_152591	LRRIQ3
7917008	-0,43	-1,34	0,10858	-0,94	-1,92	0,00108	NM_001105659	GRHL2
8147697	-0,43	-1,34	0,10628	0,59	1,50	0,04093	NM_024915	APOLD1
7954055	-0,43	-1,34	0,24830	-0,53	-1,44	0,06607	NM_001130415	---
8127496	-0,43	-1,34	0,00004	-1,04	-2,05	0,04483	---	BBOX1
7939056	-0,43	-1,34	0,04559	-0,91	-1,87	0,00139	NM_003986	UBTFL1
8054075	-0,43	-1,34	0,34971	-1,31	-2,48	0,01211	NM_001143975	ABCG5
8051866	-0,43	-1,34	0,25844	-0,85	-1,80	0,01340	NM_022436	LIPC
7983928	-0,43	-1,34	0,06066	-1,04	-2,06	0,05547	NM_000236	PLAC8
8101429	-0,43	-1,34	0,14774	0,62	1,54	0,09009	NM_016619	MPZL2
7952046	-0,43	-1,34	0,21576	3,75	13,50	0,00215	NM_144765	CCDC159
8025868	-0,43	-1,34	0,36373	0,03	1,02	0,93166	NM_001080503	PRR7
8110382	-0,43	-1,34	0,24705	-0,05	-1,03	0,71954	NM_030567	OGDHL

7933537	-0,43	-1,34	0,42516	-0,34	-1,27	0,38202	NM_018245	---
7932792	-0,43	-1,34	0,25615	-1,05	-2,07	0,10086	---	ZCCHC5
8173752	-0,43	-1,34	0,17069	-0,66	-1,58	0,00980	NM_152694	---
7983661	-0,43	-1,34	0,53782	-1,20	-2,29	0,29407	---	ASB14
8088292	-0,43	-1,34	0,36831	-0,92	-1,89	0,03768	NM_130387	C6orf218
8123876	-0,43	-1,34	0,24142	-0,47	-1,39	0,07352	NR_027793	RDH5
7956018	-0,43	-1,34	0,31351	-0,96	-1,94	0,00536	NM_002905	CNTROB
8004741	-0,43	-1,34	0,23367	0,10	1,07	0,49581	NM_001037144	---
8171874	-0,43	-1,34	0,17040	-1,32	-2,49	0,02900	---	DGKE
8008629	-0,43	-1,34	0,24724	1,34	2,54	0,03513	NM_003647	TOP3B
8074817	-0,43	-1,34	0,11183	1,32	2,50	0,08343	NM_003935	---
7985023	-0,43	-1,34	0,56262	-1,32	-2,50	0,00087	---	PHACTR1
8116932	-0,43	-1,34	0,02785	-1,10	-2,15	0,00058	NM_030948	MYH6
7977987	-0,43	-1,34	0,00187	-1,01	-2,02	0,02157	NM_002471	LOC100291851
7941269	-0,43	-1,34	0,32544	1,01	2,02	0,03562	ENST00000309775	SCGB1D1
7940622	-0,43	-1,34	0,27116	-1,03	-2,04	0,00330	NM_006552	GPAA1
8148715	-0,43	-1,35	0,39690	2,45	5,45	0,01192	NM_003801	LOC646999
8132404	-0,43	-1,35	0,30271	-0,96	-1,94	0,00599	NR_024390	SLC6A14
8169504	-0,43	-1,35	0,00848	4,84	28,66	0,00004	NM_007231	HUS1B
8123558	-0,43	-1,35	0,11313	-0,36	-1,28	0,10252	NM_148959	IGFL1
8029779	-0,43	-1,35	0,48892	-1,53	-2,90	0,06397	NM_198541	HAAO
8051799	-0,43	-1,35	0,16667	-0,37	-1,29	0,39303	NM_012205	MMP15
7996185	-0,43	-1,35	0,17662	1,45	2,72	0,02344	NM_002428	MRM1
8006668	-0,43	-1,35	0,23209	1,66	3,15	0,01090	NM_024864	FAM89A
7925028	-0,43	-1,35	0,36602	-0,10	-1,07	0,80997	NM_198552	RSPO3
8121916	-0,43	-1,35	0,33456	1,02	2,03	0,06556	NM_032784	GNB3
7953469	-0,43	-1,35	0,40540	-0,55	-1,47	0,28577	NM_002075	LOC349196
8149218	-0,43	-1,35	0,74347	-1,46	-2,76	0,07754	NR_027000	LOC349196
8149220	-0,43	-1,35	0,74347	-1,46	-2,76	0,07754	NR_027000	LOC349196
8149222	-0,43	-1,35	0,74347	-1,46	-2,76	0,07754	NR_027000	LOC349196
8149224	-0,43	-1,35	0,74347	-1,46	-2,76	0,07754	NR_027000	LOC349196
8149226	-0,43	-1,35	0,74347	-1,46	-2,76	0,07754	NR_027000	SCAMP5
7984932	-0,43	-1,35	0,28426	0,87	1,82	0,15740	NM_138967	---
8117624	-0,43	-1,35	0,31281	-1,54	-2,90	0,00735	---	---
7987066	-0,43	-1,35	0,19937	-1,63	-3,10	0,00015	---	HIST1H2BG
8124423	-0,43	-1,35	0,12281	-0,25	-1,19	0,59734	NM_003518	---
7990090	-0,43	-1,35	0,13551	-0,49	-1,41	0,30587	---	PER1
8012349	-0,43	-1,35	0,01412	-0,22	-1,17	0,21907	NM_002616	KIF13B
8150036	-0,43	-1,35	0,34634	1,48	2,79	0,00008	NM_015254	SLC4A5
8053073	-0,43	-1,35	0,07211	-0,03	-1,02	0,89959	NM_133478	MYEOV
7942118	-0,43	-1,35	0,14459	1,62	3,08	0,03528	NM_138768	STX11
8122457	-0,43	-1,35	0,29058	0,05	1,03	0,94182	NM_003764	FLJ44874
7960436	-0,43	-1,35	0,18357	-1,34	-2,53	0,03549	AK126822	---
8122732	-0,43	-1,35	0,21071	0,27	1,21	0,29282	---	CDKN2B
8160452	-0,43	-1,35	0,24694	-0,47	-1,38	0,42104	NM_078487	LRRC36
7996546	-0,43	-1,35	0,40979	-0,38	-1,30	0,16492	NM_018296	C17orf66
8014298	-0,43	-1,35	0,02337	-0,77	-1,70	0,00051	NM_152781	FCGR2B
7906777	-0,43	-1,35	0,06817	-0,92	-1,89	0,04253	NM_004001	PRH2
7953967	-0,43	-1,35	0,32116	-0,37	-1,30	0,31101	NM_005042	PPP4R1L
8067248	-0,43	-1,35	0,16061	0,34	1,26	0,12402	NR_003505	HTR1E
8120983	-0,43	-1,35	0,43067	-0,79	-1,73	0,03153	NM_000865	SLC39A5
7956229	-0,43	-1,35	0,30073	-0,18	-1,13	0,34346	NM_001135195	---
8126145	-0,43	-1,35	0,11191	-1,16	-2,23	0,00737	---	---
8161000	-0,43	-1,35	0,21943	-1,48	-2,79	0,00379	---	CDA
7898655	-0,43	-1,35	0,36175	2,66	6,30	0,00232	NM_001785	GABRB1
8094901	-0,43	-1,35	0,14871	-1,05	-2,07	0,00078	NM_000812	---
8123340	-0,43	-1,35	0,36932	-0,53	-1,45	0,16203	---	---
8135216	-0,43	-1,35	0,11688	-1,06	-2,09	0,03368	---	INSRR
7921155	-0,43	-1,35	0,53246	-1,76	-3,39	0,00319	NM_014215	TAS2R8
7961243	-0,43	-1,35	0,07941	-1,13	-2,19	0,03492	NM_023918	DCT
7972259	-0,43	-1,35	0,10893	-1,16	-2,23	0,01828	NM_001922	RASL12
7989718	-0,43	-1,35	0,29857	-1,92	-3,79	0,01408	NM_016563	KCNMB3
8092241	-0,43	-1,35	0,51959	-0,86	-1,82	0,25601	NM_171830	OSTalpha
8084929	-0,43	-1,35	0,50399	-1,29	-2,45	0,01808	NM_152672	FAM23A
7926403	-0,43	-1,35	0,01152	-0,51	-1,42	0,15124	NM_001098844	FAM23A
7926445	-0,43	-1,35	0,01152	-0,51	-1,42	0,15124	NM_001098844	CCL4L1
8006621	-0,43	-1,35	0,20125	-0,84	-1,79	0,00319	NM_001001435	CCL4L1
8019651	-0,43	-1,35	0,20125	-0,84	-1,79	0,00319	NM_001001435	ETFB
8038792	-0,43	-1,35	0,02322	-0,46	-1,37	0,13331	NM_001985	RHOB
8040473	-0,43	-1,35	0,15805	0,03	1,02	0,95750	NM_004040	SH3GLB2
8164521	-0,43	-1,35	0,25673	2,22	4,67	0,00245	NM_020145	MAT1A
7934755	-0,43	-1,35	0,20235	-0,90	-1,86	0,00022	NM_000429	ANAPC1
8054437	-0,43	-1,35	0,27748	1,29	2,44	0,03342	NM_022662	C17orf47

8017057	-0,43	-1,35	0,32878	-1,24	-2,37	0,00566	BC022189	---	
7979808	-0,43	-1,35	0,20498	-0,46	-1,38	0,14622	---	STX19	
8089026	-0,43	-1,35	0,03623	-1,34	-2,54	0,03240	NM_001001850		---
7904082	-0,43	-1,35	0,10778	-0,19	-1,14	0,46399	---	---	
7904431	-0,43	-1,35	0,22089	-0,32	-1,25	0,35866	---	---	
8092183	-0,43	-1,35	0,15893	-1,68	-3,20	0,00418	---	PLEKHG1	
8122756	-0,43	-1,35	0,35248	0,50	1,42	0,02128	NM_001029884		MVD
8003332	-0,43	-1,35	0,24560	0,22	1,16	0,49609	NM_002461		GNAZ
8071671	-0,43	-1,35	0,38849	0,28	1,22	0,39337	NM_002073		LOC100131195
7933190	-0,43	-1,35	0,00378	-0,75	-1,68	0,02814	AK097743	---	
7983377	-0,43	-1,35	0,25901	-1,41	-2,66	0,01669	---	FGF13	
8175444	-0,43	-1,35	0,23568	-0,61	-1,53	0,01634	NM_004114		---
8040286	-0,43	-1,35	0,38621	-1,22	-2,34	0,00182	---	GPR52	
7907531	-0,43	-1,35	0,31820	-0,97	-1,96	0,01210	NM_005684		CDH24
7977888	-0,43	-1,35	0,28471	-0,04	-1,03	0,88403	NM_022478		GYPA
8102998	-0,43	-1,35	0,23715	-1,02	-2,03	0,04079	NM_002099		TLL1
8098214	-0,43	-1,35	0,26757	-0,66	-1,58	0,01174	NM_012464		ZNF544
8031884	-0,43	-1,35	0,44568	0,82	1,77	0,15008	NM_014480		AGAP5
7933427	-0,43	-1,35	0,35636	1,45	2,74	0,00793	NM_001144000		RPGRIP1
7973185	-0,43	-1,35	0,16472	-0,51	-1,43	0,01095	NM_020366		SPRY3
8171034	-0,43	-1,35	0,33416	-0,43	-1,35	0,00889	NM_005840		SPRY3
8176955	-0,43	-1,35	0,33416	-0,43	-1,35	0,00889	NM_005840		ATP6V1G3
7923164	-0,43	-1,35	0,01880	-0,46	-1,37	0,27929	NM_133326		OR8D4
7944793	-0,43	-1,35	0,02919	-1,38	-2,61	0,00721	NM_001005197		MUC12
8135037	-0,43	-1,35	0,08002	-0,50	-1,42	0,02750	NM_001164462		---
8172445	-0,43	-1,35	0,00731	-1,25	-2,38	0,00000	---	ZNF774	
7986004	-0,43	-1,35	0,09602	0,75	1,69	0,15737	NM_001004309		---
8085970	-0,43	-1,35	0,31732	-1,18	-2,27	0,00613	---	CLEC4E	
7960900	-0,43	-1,35	0,17643	-0,33	-1,26	0,12841	NM_014358		---
7904691	-0,43	-1,35	0,01342	-0,76	-1,70	0,10811	---	CHPF	
8059244	-0,43	-1,35	0,37531	0,55	1,46	0,48169	NM_024536		DNM1
8158183	-0,43	-1,35	0,28698	1,74	3,35	0,02495	NM_004408		---
7897520	-0,43	-1,35	0,07636	-0,14	-1,10	0,67925	---	---	
8048701	-0,43	-1,35	0,32306	-0,54	-1,45	0,14054	---	ASB15	
8135804	-0,43	-1,35	0,04872	-1,02	-2,02	0,03844	NM_080928		---
7991153	-0,43	-1,35	0,02613	-0,60	-1,52	0,03079	---	ARFRP1	
8067727	-0,44	-1,35	0,22659	0,05	1,03	0,78933	NM_003224		SHF
7988400	-0,44	-1,35	0,20007	-1,02	-2,03	0,11936	NM_138356		MUS81
7941425	-0,44	-1,35	0,02105	0,16	1,12	0,72825	NM_025128		CHRND
8049152	-0,44	-1,35	0,45227	-0,90	-1,87	0,01337	NM_000751		IGSF10
8091537	-0,44	-1,35	0,08059	-1,17	-2,25	0,00804	NM_178822		FLJ34690
8005043	-0,44	-1,35	0,33844	-1,74	-3,34	0,03321	AK092009	FLJ16126	
7912280	-0,44	-1,35	0,10397	-0,90	-1,86	0,00440	AK131225	WDR86	
8143941	-0,44	-1,35	0,11574	-0,98	-1,97	0,04685	NM_198285		FLJ37201
7934961	-0,44	-1,35	0,00618	0,28	1,22	0,45944	NR_026835		---
8094897	-0,44	-1,35	0,46496	-0,81	-1,75	0,00026	---	UBE2M	
8039805	-0,44	-1,35	0,52110	-0,27	-1,21	0,52823	NM_003969		FLT3LG
8030339	-0,44	-1,35	0,19674	-0,21	-1,16	0,47593	NM_001459		SNORA22
8133106	-0,44	-1,35	0,53558	-1,12	-2,17	0,25923	NR_002961		LOC339240
8005446	-0,44	-1,35	0,50550	-1,72	-3,29	0,00117	AK127974		---
8068014	-0,44	-1,35	0,16362	-1,11	-2,15	0,00186	---	FANK1	
7931281	-0,44	-1,35	0,17637	-0,85	-1,80	0,00386	NM_145235		FLJ13224
7954692	-0,44	-1,35	0,38677	-1,45	-2,73	0,00632	NR_026806		HIST1H2AI
8117583	-0,44	-1,35	0,17256	1,12	2,18	0,00928	NM_003509		NKX3-1
8149811	-0,44	-1,35	0,37566	1,00	2,00	0,06530	NM_006167		SLC7A7
7977786	-0,44	-1,35	0,13374	-1,46	-2,76	0,00509	NM_003982		BAGE2
8069487	-0,44	-1,35	0,29653	-1,23	-2,34	0,00313	NM_182482		ESX1
8174291	-0,44	-1,35	0,29513	-0,91	-1,88	0,05373	NM_153448		FCHSD1
8114709	-0,44	-1,35	0,05197	-0,31	-1,24	0,41639	NM_033449		PRSS35
8120932	-0,44	-1,35	0,12390	-1,70	-3,24	0,01222	NM_001170423		---
8168081	-0,44	-1,35	0,12006	-0,73	-1,66	0,02339	---	PTPN22	
7918657	-0,44	-1,35	0,09283	-0,26	-1,20	0,26148	NM_015967		MIR208A
7978019	-0,44	-1,35	0,20024	-1,21	-2,31	0,01703	NR_029595		GNAL
8020164	-0,44	-1,35	0,43585	1,55	2,92	0,01784	NM_182978		HDHD3
8163505	-0,44	-1,35	0,44949	0,31	1,24	0,48009	NM_031219		---
8042113	-0,44	-1,35	0,14628	-1,04	-2,06	0,00251	---	---	
7956668	-0,44	-1,35	0,56064	-0,62	-1,54	0,27178	---	ARHGEF16	
7897154	-0,44	-1,35	0,23175	-0,41	-1,33	0,16905	NM_014448		PIAS4
8024740	-0,44	-1,35	0,18134	-0,03	-1,02	0,95632	NM_015897		DNASE2B
7902623	-0,44	-1,35	0,29093	-0,76	-1,70	0,18391	NM_021233		FDPS
7920757	-0,44	-1,35	0,05800	0,14	1,10	0,58756	NM_002004		LOC401127
8094701	-0,44	-1,35	0,15185	-0,26	-1,20	0,10438	NR_026854		---
8159873	-0,44	-1,35	0,48498	-1,20	-2,30	0,02287	---	---	

8045341	-0,44	-1,35	0,23519	-0,47	-1,38	0,19472	---	SPHK2
8030078	-0,44	-1,35	0,21097	0,00	-1,00	0,98939	NM_020126	---
7968514	-0,44	-1,36	0,53298	-0,64	-1,56	0,00687	---	HIST1H2AJ
8124518	-0,44	-1,36	0,14156	-0,24	-1,18	0,65389	NM_021066	INTS3
7920337	-0,44	-1,36	0,16178	-0,24	-1,18	0,38249	NM_023015	LOC100129581
8050113	-0,44	-1,36	0,42139	-1,32	-2,50	0,00125	AK125905	WFIKKN1
7991927	-0,44	-1,36	0,23040	-1,80	-3,49	0,01395	NM_053284	DYRK2
7956930	-0,44	-1,36	0,11067	1,52	2,87	0,03392	NM_006482	IL17F
8127024	-0,44	-1,36	0,51317	-0,74	-1,66	0,03355	NM_052872	---
8150918	-0,44	-1,36	0,03146	-1,68	-3,20	0,00816	---	MIR186
7916984	-0,44	-1,36	0,10101	-0,49	-1,41	0,00330	NR_029707	LOC644714
8097474	-0,44	-1,36	0,22620	-1,21	-2,31	0,08699	BC047037	ZNF137
8030954	-0,44	-1,36	0,37679	-0,09	-1,06	0,48367	NR_023311	FAM26D
8121605	-0,44	-1,36	0,36863	-1,39	-2,62	0,01830	NM_153036	OR8K3
7939977	-0,44	-1,36	0,28800	-1,14	-2,20	0,03925	NM_001005202	PRIC285
8067680	-0,44	-1,36	0,26828	-0,25	-1,19	0,47407	NM_001037335	MRO
8023361	-0,44	-1,36	0,05154	-1,01	-2,01	0,00141	NM_031939	---
8174889	-0,44	-1,36	0,41130	-0,21	-1,16	0,41687	---	CENPB
8064762	-0,44	-1,36	0,39864	-0,26	-1,20	0,37655	NM_001810	SNORA15
8133072	-0,44	-1,36	0,40104	-1,21	-2,31	0,01621	NR_002957	SNORA15
8133108	-0,44	-1,36	0,40104	-1,21	-2,31	0,01621	NR_002957	CLEC4M
8025303	-0,44	-1,36	0,23735	-0,35	-1,27	0,24490	NR_026707	SOBP
8121319	-0,44	-1,36	0,31817	-0,50	-1,42	0,01641	NM_018013	---
7977784	-0,44	-1,36	0,04771	-0,64	-1,56	0,00055	---	HIST2H3D
7919612	-0,44	-1,36	0,41372	2,09	4,27	0,00156	NM_001123375	MIR146A
8109649	-0,44	-1,36	0,22907	-0,59	-1,51	0,03139	NR_029701	C4orf50
8099193	-0,44	-1,36	0,44434	-0,72	-1,65	0,16543	BC140710	PCP4
8068651	-0,44	-1,36	0,24045	-0,66	-1,58	0,11120	NM_006198	GPC1
8049670	-0,44	-1,36	0,60098	-0,02	-1,02	0,97101	NM_002081	CLEC2A
7961091	-0,44	-1,36	0,31532	-0,79	-1,73	0,00635	NM_001130711	GOLGA8DP
7986642	-0,44	-1,36	0,35315	-0,54	-1,46	0,22281	NR_027407	RNF152
8023598	-0,44	-1,36	0,13804	-0,81	-1,75	0,00557	NM_173557	---
7916783	-0,44	-1,36	0,12408	-0,83	-1,78	0,00301	---	L2HGDH
7978956	-0,44	-1,36	0,14006	2,25	4,77	0,00087	NM_024884	PRELID2
8114845	-0,44	-1,36	0,30667	0,15	1,11	0,65148	NM_182960	MIR25
8141419	-0,44	-1,36	0,42049	-0,50	-1,41	0,13119	NR_029498	BTN2A1
8117485	-0,44	-1,36	0,24199	0,00	1,00	0,99442	NM_078476	C21orf34
8067932	-0,44	-1,36	0,00458	-1,25	-2,39	0,02125	NR_027790	FABP5
8147049	-0,44	-1,36	0,21992	1,43	2,70	0,00039	NM_001444	C10orf93
7937135	-0,44	-1,36	0,28459	-0,73	-1,66	0,02249	NM_173572	LOC100129677
8004938	-0,44	-1,36	0,04033	-1,51	-2,85	0,00970	ENST00000420547	---
8120360	-0,44	-1,36	0,13174	-0,77	-1,70	0,01814	---	KBTBD8
8080911	-0,44	-1,36	0,01596	-1,56	-2,95	0,01003	NM_032505	---
8112329	-0,44	-1,36	0,01473	-1,31	-2,48	0,09505	---	MIB2
7896985	-0,44	-1,36	0,38309	-0,23	-1,17	0,56114	NM_080875	SPAG1
8147661	-0,44	-1,36	0,04711	0,87	1,83	0,08755	NM_003114	---
8152289	-0,44	-1,36	0,28339	-2,08	-4,22	0,00857	---	---
7909525	-0,44	-1,36	0,14483	0,00	-1,00	0,98486	---	FAM161A
8052554	-0,44	-1,36	0,38266	0,92	1,89	0,07991	NM_032180	SAMD5
8122634	-0,44	-1,36	0,06704	-0,58	-1,49	0,01296	NM_001030060	MST1
8087447	-0,44	-1,36	0,42109	-0,40	-1,32	0,13567	NM_020998	KRTAP19-2
8069827	-0,44	-1,36	0,11480	-0,24	-1,18	0,19150	NM_181608	---
7983523	-0,44	-1,36	0,19240	-0,82	-1,77	0,02718	---	RNASEL
7922707	-0,44	-1,36	0,05887	0,60	1,52	0,10362	NM_021133	---
8057574	-0,44	-1,36	0,25531	-1,47	-2,77	0,00156	---	LOC100130794
8013858	-0,44	-1,36	0,43721	-1,21	-2,31	0,13686	ENST00000437256	POU1F1
8088986	-0,44	-1,36	0,29287	-0,81	-1,75	0,00375	NM_001122757	LRMP
7954436	-0,44	-1,36	0,19472	-1,28	-2,42	0,04867	NM_006152	LOC100134868
8065537	-0,44	-1,36	0,10166	1,89	3,70	0,04712	NR_004846	EYS
8127408	-0,44	-1,36	0,36766	-0,90	-1,87	0,04399	NM_198283	TET3
8042811	-0,44	-1,36	0,36006	0,24	1,18	0,69509	NM_144993	CUEDC1
8016878	-0,44	-1,36	0,27544	-0,03	-1,02	0,92160	NM_017949	DLEU2L
7901948	-0,44	-1,36	0,04297	-1,05	-2,07	0,03101	NR_002771	---
8104490	-0,44	-1,36	0,03150	-1,79	-3,45	0,04439	---	LOC440337
7993108	-0,44	-1,36	0,43233	-1,59	-3,02	0,00094	AK094332	---
7924117	-0,44	-1,36	0,04641	-1,11	-2,15	0,00234	---	EGF
8096845	-0,44	-1,36	0,04951	0,76	1,69	0,00968	NM_001963	OR2V1
8116500	-0,44	-1,36	0,55468	-1,12	-2,18	0,00207	ENST00000329365	CD3G
7944185	-0,44	-1,36	0,34131	-1,23	-2,35	0,03744	NM_000073	EPB42
7988033	-0,44	-1,36	0,02971	-1,33	-2,52	0,04180	NM_000119	SCN4A
8017521	-0,44	-1,36	0,11130	-0,80	-1,74	0,00237	NM_000334	---
8115838	-0,44	-1,36	0,45871	-1,06	-2,09	0,06722	---	PLCG2
7997453	-0,44	-1,36	0,00524	-0,11	-1,08	0,69081	NM_002661	NTNG2

8158890	-0,44	-1,36	0,22974	-1,34	-2,54	0,00640	NM_032536	OR5AK2
7940000	-0,44	-1,36	0,34338	-0,85	-1,81	0,00189	NM_001005323	ABCC6P2
7999591	-0,45	-1,36	0,19146	0,26	1,20	0,51154	NR_023387	---
8099537	-0,45	-1,36	0,25778	-1,32	-2,50	0,00914	---	C4orf37
8101816	-0,45	-1,36	0,04681	-0,67	-1,59	0,00168	NM_174952	FEN1
7940561	-0,45	-1,36	0,12832	0,95	1,93	0,01955	NM_004111	CYP4X1
7901272	-0,45	-1,36	0,24638	-0,27	-1,21	0,06129	NM_178033	---
8058106	-0,45	-1,36	0,23414	-0,81	-1,75	0,06276	---	SEC31B
7935780	-0,45	-1,36	0,02466	-0,43	-1,35	0,14916	NM_015490	TGM4
8079279	-0,45	-1,36	0,18734	-0,24	-1,18	0,28076	NM_003241	ACTN2
7910727	-0,45	-1,36	0,17429	-0,99	-1,98	0,00602	NM_001103	GPER
8131069	-0,45	-1,36	0,14720	-0,60	-1,51	0,00608	NM_001039966	C17orf102
8014169	-0,45	-1,36	0,39363	-2,17	-4,50	0,00003	NM_207454	C14orf53
7975154	-0,45	-1,36	0,09387	-0,89	-1,85	0,02181	NR_024338	NEFH
8072229	-0,45	-1,36	0,08282	-1,31	-2,47	0,00000	NM_021076	C17orf77
8009662	-0,45	-1,36	0,19386	-0,35	-1,28	0,31150	NM_152460	GPR120
7929344	-0,45	-1,36	0,37640	1,33	2,52	0,03405	NM_181745	C18orf22
8021905	-0,45	-1,36	0,05411	-0,25	-1,19	0,55853	NM_024805	---
8128886	-0,45	-1,36	0,20159	-0,23	-1,18	0,42425	---	ACOT7
8099965	-0,45	-1,36	0,62875	-0,07	-1,05	0,92693	NM_007274	MRPS26
8060599	-0,45	-1,36	0,06443	1,31	2,49	0,00391	NM_030811	LOC401097
8083707	-0,45	-1,36	0,13728	0,18	1,14	0,47329	NM_001168214	---
8087545	-0,45	-1,36	0,04367	-1,37	-2,59	0,04552	---	KALRN
8082165	-0,45	-1,36	0,12110	-1,13	-2,19	0,00020	NM_001024660	ENPP4
8120061	-0,45	-1,36	0,26343	1,39	2,62	0,03931	NM_014936	RAB36
8071676	-0,45	-1,36	0,57198	-1,00	-1,99	0,09437	NM_004914	ZNF852
8086494	-0,45	-1,36	0,02338	0,66	1,58	0,23077	AK296954	---
8081454	-0,45	-1,36	0,58256	-1,66	-3,16	0,00739	---	FSHR
8052072	-0,45	-1,36	0,14592	-1,56	-2,95	0,04917	NM_000145	CENPT
8002104	-0,45	-1,36	0,23510	1,28	2,42	0,04234	NM_025082	UBASH3A
8068788	-0,45	-1,36	0,07276	-1,21	-2,32	0,06825	NM_018961	C14orf39
7979483	-0,45	-1,36	0,17271	-0,81	-1,75	0,00431	NM_174978	MIR103-1
8115689	-0,45	-1,36	0,30194	-0,68	-1,61	0,06420	NR_029520	TACC3
8093500	-0,45	-1,36	0,18798	1,09	2,13	0,04247	NM_006342	SAMD7
8083814	-0,45	-1,36	0,53173	-1,32	-2,50	0,02221	NM_182610	---
7980718	-0,45	-1,36	0,50697	-0,84	-1,80	0,15346	---	TMEM119
7966122	-0,45	-1,36	0,41969	-0,92	-1,90	0,01399	NM_181724	MIR15A
7971661	-0,45	-1,36	0,05002	-0,26	-1,19	0,39520	NR_029485	ZNF132
8039771	-0,45	-1,36	0,13676	-1,53	-2,90	0,00532	NM_003433	---
8173522	-0,45	-1,36	0,51452	-2,38	-5,19	0,15301	---	---
7905949	-0,45	-1,36	0,15296	-0,43	-1,34	0,09732	---	---
8176648	-0,45	-1,36	0,07943	-0,42	-1,34	0,20784	---	MIR128-1
8045453	-0,45	-1,36	0,07483	-0,93	-1,90	0,00566	NR_029672	DEFB110
8126939	-0,45	-1,36	0,02290	-0,58	-1,50	0,00259	NM_001037497	CDCA7
8046488	-0,45	-1,36	0,19279	2,45	5,47	0,00028	NM_031942	CHIT1
7923562	-0,45	-1,36	0,28173	-1,20	-2,30	0,03257	NM_003465	HAVCR1
8115455	-0,45	-1,36	0,18505	-0,50	-1,41	0,03407	NM_012206	ANKRD20B
7919146	-0,45	-1,36	0,41309	0,20	1,15	0,72484	NR_003366	ZNF695
7925662	-0,45	-1,36	0,37652	-0,34	-1,26	0,19778	NM_020394	C13orf18
7971486	-0,45	-1,36	0,35920	-0,86	-1,81	0,00148	NM_025113	DAB2IP
8157610	-0,45	-1,36	0,12203	0,74	1,67	0,09284	NM_032552	---
7919749	-0,45	-1,36	0,06965	-1,02	-2,03	0,00147	---	---
8054939	-0,45	-1,36	0,00898	-0,41	-1,33	0,08671	---	C14orf145
7980496	-0,45	-1,36	0,26736	0,39	1,31	0,30906	NM_152446	IQCF1
8087782	-0,45	-1,36	0,26515	-1,79	-3,45	0,00014	NM_152397	BIN1
8054945	-0,45	-1,36	0,04712	0,00	-1,00	0,99771	NM_139343	---
7912762	-0,45	-1,36	0,40732	0,11	1,08	0,11313	---	FAM135B
8153071	-0,45	-1,36	0,15558	-0,78	-1,71	0,00663	NM_015912	C3orf53
8085967	-0,45	-1,36	0,33341	-1,44	-2,72	0,02304	ENST00000317800	PTPRD
8160040	-0,45	-1,37	0,09391	-1,21	-2,31	0,00009	NM_002839	PIWIL4
7943240	-0,45	-1,37	0,18814	-0,53	-1,44	0,08362	NM_152431	KRT12
8015115	-0,45	-1,37	0,08760	-0,73	-1,65	0,02865	NM_000223	OR5M10
7948148	-0,45	-1,37	0,44855	-2,39	-5,25	0,04776	NM_001004741	PCLO
8140620	-0,45	-1,37	0,12579	0,87	1,82	0,01071	NM_033026	ABCD2
7962312	-0,45	-1,37	0,36803	-0,49	-1,40	0,00384	NM_005164	---
8132849	-0,45	-1,37	0,48658	-0,54	-1,46	0,20493	---	CCBP2
8079117	-0,45	-1,37	0,30088	-0,68	-1,60	0,02478	NM_001296	FLJ41309
8106727	-0,45	-1,37	0,16011	-1,13	-2,19	0,00332	AK123303	---
8048712	-0,45	-1,37	0,09035	-1,45	-2,74	0,00331	---	SNN
7993259	-0,45	-1,37	0,48495	-1,04	-2,06	0,01333	NM_003498	DEFB127
8060314	-0,45	-1,37	0,02040	-1,00	-2,00	0,00103	NM_139074	IGLON5
8030823	-0,45	-1,37	0,55748	0,70	1,62	0,14871	NM_001101372	UGT2B17
8100734	-0,45	-1,37	0,16891	-0,74	-1,67	0,04271	NM_001077	---

8112996	-0,45	-1,37	0,08568	-0,41	-1,33	0,11280	---	QRFPR
8102610	-0,45	-1,37	0,23882	0,07	1,05	0,73852	NM_198179	GRB2
8018364	-0,45	-1,37	0,02897	1,57	2,96	0,00319	NM_002086	CCDC142
8053171	-0,45	-1,37	0,08646	0,47	1,38	0,49134	NM_032779	NCRNA00028
8061539	-0,45	-1,37	0,52752	-1,80	-3,48	0,12386	NR_024358	F10
7970241	-0,45	-1,37	0,14009	-0,95	-1,93	0,03767	NM_000504	POF1B
8173869	-0,45	-1,37	0,36107	-0,80	-1,74	0,03194	NM_024921	SAPS1
8039413	-0,45	-1,37	0,10948	1,30	2,46	0,05055	NM_014931	FSIP2
8046824	-0,45	-1,37	0,11726	0,18	1,13	0,51828	AK092099	GSTA3
8127087	-0,45	-1,37	0,22999	-0,67	-1,59	0,01746	NM_000847	UNQ9370
7986291	-0,45	-1,37	0,23991	-0,12	-1,08	0,24477	AY358254	TBL1Y
8176442	-0,45	-1,37	0,11128	-0,47	-1,39	0,20426	NM_033284	OR6C76
7955997	-0,45	-1,37	0,09747	-0,95	-1,93	0,00406	NM_001005183	---
8051775	-0,45	-1,37	0,09726	-1,03	-2,04	0,00199	---	WFDC12
8066489	-0,45	-1,37	0,36277	-0,35	-1,27	0,31468	NM_080869	FBXO43
8152031	-0,45	-1,37	0,29284	0,94	1,92	0,00736	NM_001077528	PMM1
8076355	-0,45	-1,37	0,07406	0,93	1,90	0,02897	NM_002676	C6orf10
8125415	-0,45	-1,37	0,19669	-1,23	-2,34	0,00109	NM_006781	CSN3
8095477	-0,45	-1,37	0,19781	-1,07	-2,10	0,01286	NM_005212	MYLK2
8061605	-0,45	-1,37	0,10565	-0,90	-1,86	0,03518	NM_033118	C3orf49
8080868	-0,45	-1,37	0,04464	-0,78	-1,72	0,01143	NR_026866	LOC339524
7917466	-0,45	-1,37	0,43710	-1,13	-2,19	0,13267	NR_026988	PKHD1L1
8147891	-0,45	-1,37	0,08586	-0,45	-1,37	0,04872	NM_177531	C14orf43
7975626	-0,45	-1,37	0,29104	-0,70	-1,63	0,11547	NM_194278	---
7980452	-0,45	-1,37	0,30673	-1,93	-3,82	0,00030	---	PPP1R13B
7981460	-0,45	-1,37	0,57958	2,47	5,53	0,00218	NM_015316	BRD7P3
8121727	-0,45	-1,37	0,10366	-0,66	-1,58	0,00126	NR_002730	UBE2C
8063043	-0,45	-1,37	0,18194	0,78	1,72	0,02640	NM_181802	GOLGA9P
7981859	-0,45	-1,37	0,36511	-0,82	-1,77	0,07189	NR_024074	FGR
7914112	-0,45	-1,37	0,44231	-1,48	-2,80	0,03009	NM_005248	---
8089818	-0,45	-1,37	0,11967	-0,94	-1,91	0,06045	---	RCVRN
8012598	-0,45	-1,37	0,47808	-2,09	-4,27	0,00084	NM_002903	CPA5
8136217	-0,45	-1,37	0,01683	-1,18	-2,27	0,01027	NM_001127441	ZC3H10
7956162	-0,45	-1,37	0,18894	-1,23	-2,35	0,09464	NM_032786	TSPAN16
8025858	-0,45	-1,37	0,33150	-1,03	-2,04	0,01406	NM_012466	ZNF642
7900446	-0,45	-1,37	0,12634	0,14	1,10	0,60530	NM_198494	SNORD45C
7902396	-0,45	-1,37	0,07927	-0,23	-1,18	0,25320	NR_003042	---
8050213	-0,45	-1,37	0,52747	-2,14	-4,40	0,00335	---	---
8174634	-0,45	-1,37	0,07242	-1,14	-2,21	0,00547	---	VSTM2A
8132851	-0,45	-1,37	0,31573	-1,45	-2,73	0,00437	NM_182546	---
8082786	-0,45	-1,37	0,00999	-1,02	-2,03	0,02283	---	NLGN4Y
8176655	-0,45	-1,37	0,18718	-1,44	-2,72	0,00027	NR_028319	RRP7A
8076455	-0,45	-1,37	0,28238	0,35	1,27	0,53511	NM_015703	HOXC11
7955855	-0,45	-1,37	0,15189	-0,72	-1,64	0,10187	NM_014212	OR1L8
8163960	-0,45	-1,37	0,56526	-0,94	-1,92	0,00322	NM_001004454	KIFC1
8179564	-0,45	-1,37	0,41197	1,71	3,28	0,00899	NM_002263	KCNK5
8126153	-0,45	-1,37	0,26081	1,11	2,15	0,05385	NM_003740	---
8156058	-0,45	-1,37	0,11778	-0,78	-1,72	0,01407	---	CDH9
8111271	-0,45	-1,37	0,12479	-0,64	-1,56	0,00061	NM_016279	---
8050495	-0,45	-1,37	0,31657	-0,80	-1,74	0,04496	---	SH3BGR
8068620	-0,46	-1,37	0,00646	-0,55	-1,46	0,08922	NM_007341	AGPAT2
8165258	-0,46	-1,37	0,25328	1,79	3,46	0,01375	NM_006412	KRTAP19-5
8069838	-0,46	-1,37	0,27598	-1,51	-2,86	0,00142	NM_181611	---
8145797	-0,46	-1,37	0,23696	-1,08	-2,11	0,05906	---	---
7942781	-0,46	-1,37	0,05223	-0,57	-1,49	0,02934	---	C1orf84
7900710	-0,46	-1,37	0,04057	0,13	1,09	0,63113	NM_001012961	CST9L
8065396	-0,46	-1,37	0,17918	-0,74	-1,67	0,03402	NM_080610	GALNT9
7967727	-0,46	-1,37	0,06570	-1,27	-2,41	0,05753	NM_021808	---
7982495	-0,46	-1,37	0,21811	-0,92	-1,89	0,01194	---	CHAT
7927482	-0,46	-1,37	0,29436	-1,39	-2,62	0,00739	NM_001142933	FTSJ2
8137826	-0,46	-1,37	0,15628	0,27	1,21	0,44723	NM_013393	AGAP5
7934451	-0,46	-1,37	0,23499	1,30	2,46	0,01105	NM_001144000	---
7958844	-0,46	-1,37	0,24178	-0,86	-1,81	0,01254	---	C9orf128
8161154	-0,46	-1,37	0,18293	-1,18	-2,27	0,01488	NM_001012446	C19orf43
8034514	-0,46	-1,37	0,48709	-0,27	-1,21	0,55627	ENST00000242784	APOL4
8075709	-0,46	-1,37	0,20867	-0,66	-1,58	0,00219	NM_030643	---
7976261	-0,46	-1,37	0,05688	-0,81	-1,75	0,00081	---	---
7976126	-0,46	-1,37	0,07869	-0,38	-1,30	0,11436	---	C9orf142
8159541	-0,46	-1,37	0,51728	1,30	2,46	0,15124	BC002613	C6orf163
8121009	-0,46	-1,37	0,09076	-0,78	-1,72	0,04305	NM_001010868	---
7976576	-0,46	-1,37	0,05537	-0,86	-1,81	0,01186	---	MYO5B
8023267	-0,46	-1,37	0,34020	0,97	1,96	0,00034	NM_001080467	---
8085358	-0,46	-1,37	0,00712	-0,54	-1,46	0,12489	---	LY6G6D

8178070	-0,46	-1,37	0,28097	-0,78	-1,72	0,20810	NM_021246	LY6G6D
8179309	-0,46	-1,37	0,28097	-0,78	-1,72	0,20810	NM_021246	---
8053386	-0,46	-1,37	0,02218	-0,65	-1,57	0,07140	---	METTL1
7964548	-0,46	-1,37	0,28201	0,93	1,90	0,13859	NM_005371	GLYATL1
7940135	-0,46	-1,37	0,03839	-1,71	-3,28	0,00268	NM_080661	LOC441056
8176570	-0,46	-1,37	0,73693	-4,74	-26,67	0,00000	NM_001177376	BBS5
8046147	-0,46	-1,37	0,05495	-0,07	-1,05	0,75293	NM_152384	---
8118147	-0,46	-1,37	0,06871	-0,79	-1,73	0,19490	---	---
7946123	-0,46	-1,37	0,26448	-0,84	-1,78	0,00926	---	GPLD1
8124211	-0,46	-1,37	0,03528	-0,12	-1,09	0,55194	NM_001503	GRM3
8133904	-0,46	-1,37	0,45870	-0,94	-1,92	0,01336	NM_000840	C16orf55
7997976	-0,46	-1,37	0,32603	-0,36	-1,28	0,45302	AK303024	---
7939990	-0,46	-1,37	0,10864	-1,02	-2,02	0,00809	---	GK2
8101256	-0,46	-1,37	0,22216	-0,55	-1,46	0,18054	NM_033214	UGT2B10
8100768	-0,46	-1,37	0,45834	-1,40	-2,65	0,01014	NM_001075	ACTL8
7898516	-0,46	-1,37	0,15901	-0,79	-1,73	0,04683	NM_030812	LEAP2
8108006	-0,46	-1,37	0,27346	-0,43	-1,35	0,09411	NM_052971	---
8091188	-0,46	-1,37	0,03671	-1,05	-2,08	0,00745	---	PLA2G7
8126784	-0,46	-1,37	0,04149	-0,65	-1,57	0,08590	NM_001168357	MIR9-2
8113037	-0,46	-1,37	0,09947	-0,96	-1,95	0,00144	NR_030741	FAM172B
8081341	-0,46	-1,37	0,35975	1,02	2,03	0,10157	AK127584	RAB44
8119096	-0,46	-1,37	0,04408	-0,67	-1,59	0,08516	ENST00000355623	MYL1
8058604	-0,46	-1,37	0,11861	-0,69	-1,61	0,01941	NM_079420	ACSM1
7999981	-0,46	-1,37	0,13693	-0,95	-1,93	0,03081	NM_052956	ZFP82
8036309	-0,46	-1,37	0,26635	1,67	3,18	0,00475	NM_133466	CHMP4C
8147057	-0,46	-1,37	0,05909	3,06	8,32	0,00025	NM_152284	FAM75C1 // FAM75C1 // FAM75C1
8162214	-0,46	-1,38	0,24058	-1,96	-3,88	0,00210	NM_001145124 // NM_001145124 // NM_001145124	---
8084737	-0,46	-1,38	0,04364	-1,13	-2,19	0,08262	---	---
8005510	-0,46	-1,38	0,20133	-1,31	-2,48	0,00135	---	---
8013292	-0,46	-1,38	0,20133	-1,31	-2,48	0,00135	---	RPS17P5
8126946	-0,46	-1,38	0,28009	-0,97	-1,96	0,02181	ENST00000331146	KIAA0802
8020037	-0,46	-1,38	0,22232	0,10	1,07	0,76381	BC040542	FAM159B
8105607	-0,46	-1,38	0,16863	-0,77	-1,71	0,10274	NM_001164442	MIR141
7953592	-0,46	-1,38	0,15142	-1,12	-2,17	0,01361	NR_029682	GPR172B
8011808	-0,46	-1,38	0,31999	-0,94	-1,92	0,13837	NM_001104577	CD1A
7906339	-0,46	-1,38	0,32750	-1,36	-2,57	0,04273	NM_001763	OR5T3
7939973	-0,46	-1,38	0,19364	-0,50	-1,41	0,03497	NM_001004747	TIAM1
8069880	-0,46	-1,38	0,36519	2,73	6,65	0,00031	NM_003253	CENPA
8040712	-0,46	-1,38	0,28369	3,21	9,26	0,00157	NM_001809	---
7953198	-0,46	-1,38	0,00571	0,01	1,01	0,95533	---	C11orf66
7940493	-0,46	-1,38	0,23489	0,47	1,38	0,17874	NM_145017	ANKRD20A2
8155602	-0,46	-1,38	0,26643	-0,20	-1,15	0,43832	NM_001012421	---
8137925	-0,46	-1,38	0,07901	-1,21	-2,31	0,00846	---	KRTAP4-12
8015223	-0,46	-1,38	0,71778	-3,52	-11,46	0,00003	NM_031854	KRTAP4-12
8019565	-0,46	-1,38	0,71778	-3,52	-11,46	0,00003	NM_031854	PJA1
8173340	-0,46	-1,38	0,41534	-0,14	-1,10	0,41586	NM_145119	ORAI2
8135172	-0,46	-1,38	0,17408	0,59	1,51	0,20031	NM_001126340	GNG3
7940673	-0,46	-1,38	0,50362	-1,59	-3,00	0,01614	NM_012202	HIST1H2AB
8124391	-0,46	-1,38	0,20401	-0,02	-1,02	0,86844	NM_003513	RBPMS2
7989670	-0,46	-1,38	0,12259	2,52	5,74	0,00013	NM_194272	C1QTNF6
8075897	-0,46	-1,38	0,19081	1,14	2,20	0,01454	NM_031910	LOXHD1
8023080	-0,46	-1,38	0,05607	-0,75	-1,68	0,00278	NM_144612	MCM5
8072687	-0,46	-1,38	0,33331	1,88	3,69	0,01148	NM_006739	MIR196A2
7955863	-0,46	-1,38	0,37139	-0,57	-1,48	0,12535	NR_029617	---
7899375	-0,46	-1,38	0,02335	-0,60	-1,51	0,36000	---	C22orf46
8073470	-0,46	-1,38	0,00851	0,36	1,29	0,03053	NM_001142964	LSM14B
8067380	-0,46	-1,38	0,00491	-1,18	-2,27	0,11122	NM_144703	HAP1
8015396	-0,46	-1,38	0,14586	-0,35	-1,28	0,42926	NM_177977	HIST1H2AA
8124331	-0,46	-1,38	0,03978	-0,59	-1,51	0,07923	NM_170745	BRD3
8164995	-0,46	-1,38	0,30156	-0,27	-1,21	0,24864	NM_007371	CCL28
8111932	-0,46	-1,38	0,01281	2,35	5,11	0,00269	NM_148672	FLJ36840
8144228	-0,46	-1,38	0,37110	-0,01	-1,01	0,98013	AK094159	STARD9
7983143	-0,46	-1,38	0,31105	-0,10	-1,07	0,50405	NM_020759	ZNF619
8078991	-0,46	-1,38	0,37441	0,25	1,19	0,32621	NM_001145082	SLITRK2
8170307	-0,46	-1,38	0,27492	-0,95	-1,93	0,00150	NM_032539	SLC5A2
7995222	-0,46	-1,38	0,38569	-0,93	-1,90	0,02016	NM_003041	LRRC43
7959386	-0,46	-1,38	0,13234	-1,16	-2,24	0,03107	NM_001098519	LDHAL6B
7983985	-0,46	-1,38	0,00919	-1,41	-2,66	0,00123	NM_033195	LOC100128356
7931822	-0,46	-1,38	0,03030	-0,48	-1,39	0,01104	AY423624	CELSR2
7903632	-0,46	-1,38	0,33174	0,77	1,71	0,08189	NM_001408	PTPRH
8039389	-0,46	-1,38	0,09508	0,30	1,23	0,50545	NM_002842	MAP3K14

8016194	-0,46	-1,38	0,16807	0,78	1,72	0,10261	NM_003954	GJB3
7899932	-0,46	-1,38	0,04595	-0,42	-1,34	0,14930	NM_024009	LCE5A
7905483	-0,46	-1,38	0,45480	-1,33	-2,51	0,00240	NM_178438	YIF1B
8036483	-0,46	-1,38	0,34157	1,38	2,61	0,06291	NM_033557	MGC24125
8103906	-0,46	-1,38	0,24202	-1,53	-2,89	0,04136	AK097110 MIR205	
7909422	-0,46	-1,38	0,27200	-0,63	-1,55	0,02328	NR_029622	ERCC3
8054995	-0,46	-1,38	0,03684	-0,38	-1,30	0,00450	AK127469 MUC21	
8179224	-0,46	-1,38	0,55740	-1,32	-2,50	0,00093	NM_001010909	CAMTA2
8011774	-0,46	-1,38	0,15269	0,91	1,87	0,00461	NM_015099	CCDC23
7915468	-0,46	-1,38	0,42813	0,12	1,08	0,50557	NM_199342	ATP10A
7986789	-0,46	-1,38	0,21816	0,30	1,23	0,21170	NM_024490	---
8160084	-0,46	-1,38	0,03795	-0,35	-1,28	0,03329	--- ---	
8063408	-0,46	-1,38	0,21290	-0,09	-1,07	0,81978	--- BAI2	
7914433	-0,46	-1,38	0,06528	0,24	1,18	0,40743	NM_001703	---
7970082	-0,46	-1,38	0,30043	-1,08	-2,11	0,00579	--- KRTAP4-11	
8015221	-0,46	-1,38	0,81068	-3,66	-12,60	0,00121	NM_033059	PSCA
8153334	-0,46	-1,38	0,33310	-0,42	-1,33	0,11518	NM_005672	OR14J1
8117722	-0,46	-1,38	0,38721	-0,99	-1,99	0,01618	NM_030946	OR14J1
8177698	-0,46	-1,38	0,38721	-0,99	-1,99	0,01618	NM_030946	TSPYL4
8129089	-0,46	-1,38	0,33154	0,51	1,42	0,42652	NM_021648	SH2D1B
7921900	-0,46	-1,38	0,12966	-1,00	-2,00	0,03231	NM_053282	---
7972287	-0,47	-1,38	0,04277	-0,04	-1,03	0,78239	--- TM6SF2	
8035648	-0,47	-1,38	0,26174	-0,03	-1,02	0,93129	NM_001001524	ESR2
7979583	-0,47	-1,38	0,21356	-0,89	-1,86	0,00097	NM_001040275	KIAA1009
8127903	-0,47	-1,38	0,17852	-0,82	-1,76	0,00985	NM_014895	DNAH1
8080226	-0,47	-1,38	0,01537	0,19	1,14	0,35464	NM_015512	---
7937975	-0,47	-1,38	0,07914	-1,28	-2,42	0,00091	--- SNORD104	
8009241	-0,47	-1,38	0,29885	-0,62	-1,53	0,11226	NR_004380	C6orf142
8120300	-0,47	-1,38	0,00567	-0,89	-1,85	0,00066	NM_138569	AIFM2
7934101	-0,47	-1,38	0,25172	0,84	1,79	0,00769	NM_032797	---
7903389	-0,47	-1,38	0,38614	-1,57	-2,97	0,01167	--- SLC5A8	
7965769	-0,47	-1,38	0,05263	-0,88	-1,85	0,03488	NM_145913	---
8116908	-0,47	-1,38	0,05888	-1,15	-2,21	0,03198	--- RHPN2	
8035980	-0,47	-1,38	0,35505	3,77	13,66	0,00380	NM_033103	LOC400657
8023868	-0,47	-1,38	0,35432	-0,26	-1,19	0,38290	NR_024484	AGBL2
7947947	-0,47	-1,38	0,46476	1,30	2,47	0,07099	NM_024783	CAMK2B
8139330	-0,47	-1,38	0,07843	0,44	1,36	0,38811	NM_001220	AGAP6
7927552	-0,47	-1,38	0,35814	1,77	3,40	0,00319	NM_001077665	SNORD38B
7901052	-0,47	-1,38	0,22000	-0,28	-1,22	0,20370	NR_001457	POU6F1
7963265	-0,47	-1,38	0,31554	0,82	1,76	0,06417	NR_026893	OR11H1
7977450	-0,47	-1,38	0,41860	-2,81	-7,01	0,00468	NM_001005239	---
8001325	-0,47	-1,38	0,02890	-0,32	-1,25	0,04884	--- CCDC129	
8132167	-0,47	-1,38	0,16360	-1,36	-2,57	0,00395	NM_194300	DNAJB2
8048523	-0,47	-1,38	0,20547	-0,71	-1,63	0,01410	NM_006736	LOC255411
7955135	-0,47	-1,38	0,04118	-0,10	-1,07	0,56814	NR_029449	EFHC2
8172225	-0,47	-1,38	0,04031	-0,43	-1,34	0,14992	NM_025184	---
8040171	-0,47	-1,38	0,04938	-0,73	-1,66	0,00161	--- TREM1	
8126303	-0,47	-1,38	0,21350	-0,64	-1,56	0,01158	NM_018643	SLC46A1
8013616	-0,47	-1,38	0,32859	2,46	5,50	0,00788	NM_080669	---
8002018	-0,47	-1,38	0,11870	-0,46	-1,38	0,01330	--- EGR3	
8149720	-0,47	-1,38	0,14500	-0,90	-1,86	0,00279	NM_004430	PON3
8141066	-0,47	-1,38	0,13055	2,72	6,61	0,00784	NM_000940	IRF6
7924058	-0,47	-1,38	0,24901	0,86	1,81	0,09942	NM_006147	SLC39A2
7973135	-0,47	-1,38	0,07920	-1,35	-2,55	0,02684	NM_014579	GPR183
7972557	-0,47	-1,38	0,09308	-0,77	-1,71	0,03021	NM_004951	LOC349196
8144420	-0,47	-1,38	0,72402	-1,44	-2,72	0,06805	NR_027000	LOC349196
8144494	-0,47	-1,38	0,72402	-1,44	-2,72	0,06805	NR_027000	USP49
8126347	-0,47	-1,38	0,12742	-0,46	-1,38	0,20625	NM_018561	C13orf39
7972665	-0,47	-1,38	0,10176	-0,91	-1,88	0,00034	BC132748 KCTD6	
8080804	-0,47	-1,38	0,17553	-0,77	-1,71	0,19091	NM_153331	CRIP2
7977397	-0,47	-1,38	0,01471	-0,28	-1,22	0,22181	NM_001312	OVOS
7953873	-0,47	-1,38	0,16667	1,31	2,48	0,00376	BX647938 ---	
8170702	-0,47	-1,38	0,14285	-1,22	-2,33	0,15061	--- ---	
8068520	-0,47	-1,38	0,03470	-0,79	-1,73	0,05253	--- P2RY12	
8091530	-0,47	-1,38	0,14944	-1,56	-2,96	0,00667	NM_022788	---
7913803	-0,47	-1,38	0,01564	-1,09	-2,13	0,00159	--- ---	
8082236	-0,47	-1,39	0,09044	-1,12	-2,17	0,00540	--- LOC257358	
8109908	-0,47	-1,39	0,32095	-0,91	-1,88	0,02022	AK093725 SAA2	
7946983	-0,47	-1,39	0,57322	-1,53	-2,88	0,02819	NM_030754	TTLL11
8163916	-0,47	-1,39	0,43812	-0,04	-1,03	0,93581	NM_001139442	---
7973350	-0,47	-1,39	0,04376	-1,15	-2,22	0,04949	--- AKR7A3	
7913146	-0,47	-1,39	0,54535	-1,10	-2,14	0,18145	NM_012067	---
8097122	-0,47	-1,39	0,04419	-0,32	-1,25	0,06150	--- ---	

7938588	-0,47	-1,39	0,01023	-0,85	-1,80	0,00946	---	---
8014664	-0,47	-1,39	0,60706	-3,14	-8,79	0,00317	---	---
8019649	-0,47	-1,39	0,60706	-3,14	-8,79	0,00317	---	RPSAP15
8173912	-0,47	-1,39	0,13153	-0,82	-1,77	0,14614	AF284768	NOP16
8115907	-0,47	-1,39	0,23138	0,45	1,37	0,12479	NM_016391	SP140
8048898	-0,47	-1,39	0,00004	-1,09	-2,13	0,00003	NM_007237	---
8044791	-0,47	-1,39	0,40503	-0,96	-1,95	0,05816	---	FAM194A
8091469	-0,47	-1,39	0,26276	-0,62	-1,54	0,14573	NM_152394	FSTL3
8023995	-0,47	-1,39	0,56736	1,96	3,89	0,05673	NM_005860	CCDC29
8020349	-0,47	-1,39	0,32424	0,32	1,24	0,44070	ENST00000333394	ACAN
7985786	-0,47	-1,39	0,23196	-1,14	-2,20	0,00310	NM_013227	CNNM3
8043666	-0,47	-1,39	0,08385	0,63	1,54	0,07572	NM_017623	C9
8111757	-0,47	-1,39	0,13996	-0,65	-1,57	0,01393	NM_001737	NTRK3
7991186	-0,47	-1,39	0,16749	-1,33	-2,51	0,00045	NM_001007156	KCNQ3
8152946	-0,47	-1,39	0,25067	0,61	1,52	0,05429	NM_004519	---
7979800	-0,47	-1,39	0,20439	-0,83	-1,78	0,00224	---	MPND
8024829	-0,47	-1,39	0,07331	-0,31	-1,24	0,68754	NM_032868	---
8055948	-0,47	-1,39	0,23144	-0,74	-1,67	0,09598	---	C2orf73
8041987	-0,47	-1,39	0,20773	-0,53	-1,44	0,02525	NM_001100396	---
8097685	-0,47	-1,39	0,13804	-0,99	-1,99	0,00421	---	---
7954695	-0,47	-1,39	0,24135	-0,77	-1,71	0,05807	---	MURC
8156919	-0,47	-1,39	0,09528	-0,94	-1,91	0,02131	NM_001018116	UGT2B28
8095404	-0,47	-1,39	0,20528	-0,76	-1,69	0,02209	NM_053039	---
8119235	-0,47	-1,39	0,00604	-0,60	-1,52	0,10656	---	MAGOH
7916274	-0,47	-1,39	0,12408	-0,02	-1,01	0,93169	NM_002370	LOC374491
7968056	-0,47	-1,39	0,31818	0,14	1,10	0,72166	NR_002815	ABCB9
7967337	-0,47	-1,39	0,06623	0,07	1,05	0,86395	NM_019625	KCNK1
7910611	-0,47	-1,39	0,23396	0,56	1,48	0,11217	NM_002245	TAC1
8134420	-0,47	-1,39	0,06132	-0,75	-1,69	0,00238	NM_003182	HIST1H2BO
8117614	-0,47	-1,39	0,51952	-0,75	-1,68	0,03829	NM_003527	DUSP13
7934533	-0,47	-1,39	0,23617	-0,82	-1,76	0,04255	NM_001007271	CXorf59
8166690	-0,47	-1,39	0,14570	-0,32	-1,25	0,25010	NM_173695	TRMT2A
8074541	-0,47	-1,39	0,06671	0,38	1,30	0,56919	NM_022727	ANP32D
7955117	-0,47	-1,39	0,21533	-0,58	-1,49	0,01114	NM_012404	---
7914748	-0,47	-1,39	0,37862	-1,02	-2,03	0,01283	---	C3orf48
8085749	-0,47	-1,39	0,19607	-1,08	-2,12	0,03834	NR_027694	SNORA6
8078916	-0,47	-1,39	0,56556	-0,05	-1,03	0,92545	NR_002325	CYP11B1
8153363	-0,47	-1,39	0,37394	-1,69	-3,22	0,00004	NM_000497	---
8064188	-0,47	-1,39	0,59489	-1,91	-3,76	0,00155	---	---
7904572	-0,47	-1,39	0,51377	1,36	2,56	0,05053	---	---
7952733	-0,47	-1,39	0,47572	-1,49	-2,81	0,00341	---	KCTD16
8108905	-0,47	-1,39	0,47165	-0,43	-1,35	0,02093	NM_020768	SLC25A35
8012450	-0,47	-1,39	0,15262	0,92	1,90	0,03799	NM_201520	---
8113724	-0,47	-1,39	0,18353	-1,26	-2,39	0,05034	---	OR9A2
8143555	-0,47	-1,39	0,24617	-0,93	-1,91	0,00550	NM_001001658	RNU105C
8146456	-0,47	-1,39	0,23339	-0,43	-1,35	0,10258	NR_004385	---
7899955	-0,47	-1,39	0,71313	-3,40	-10,56	0,00094	---	CNNM4
8043657	-0,47	-1,39	0,07644	2,41	5,30	0,00020	NM_020184	---
7955140	-0,48	-1,39	0,04979	-0,77	-1,71	0,11535	---	EPHA3
8081081	-0,48	-1,39	0,07287	-1,28	-2,43	0,00407	NM_005233	C8orf12
8144621	-0,48	-1,39	0,09290	-0,37	-1,29	0,05314	NR_026814	---
8085529	-0,48	-1,39	0,08972	-1,24	-2,37	0,00631	---	RPA3
8138189	-0,48	-1,39	0,15591	-0,20	-1,15	0,75178	NM_002947	ZNF767
8143733	-0,48	-1,39	0,30302	0,66	1,58	0,26448	NR_027788	SERPINB13
8021603	-0,48	-1,39	0,39058	-0,90	-1,86	0,00035	NM_012397	KRTAP2-4
8015210	-0,48	-1,39	0,27953	-1,99	-3,98	0,00108	NM_033184	KRTAP2-4
8019576	-0,48	-1,39	0,27953	-1,99	-3,98	0,00108	NM_033184	GBA3
8094361	-0,48	-1,39	0,04196	-0,18	-1,13	0,14090	NM_020973	TMPRSS12
7955433	-0,48	-1,39	0,03566	-1,22	-2,34	0,07977	NM_182559	ATXN7L1
8142104	-0,48	-1,39	0,12861	0,56	1,48	0,27979	NM_152749	PRLR
8111490	-0,48	-1,39	0,43131	-1,48	-2,80	0,00905	NM_000949	---
8003769	-0,48	-1,39	0,03286	-1,19	-2,28	0,03645	---	SPINLW1
8066542	-0,48	-1,39	0,03705	-0,26	-1,19	0,02268	NM_020398	RNF166
8003348	-0,48	-1,39	0,30555	-0,33	-1,26	0,25365	NM_178841	---
7926297	-0,48	-1,39	0,57739	-1,30	-2,46	0,00513	---	MEX3B
7990873	-0,48	-1,39	0,22282	-0,56	-1,48	0,29630	NM_032246	RGS21
7908382	-0,48	-1,39	0,00657	-0,64	-1,56	0,00841	NM_001039152	FREM1
8160168	-0,48	-1,39	0,09689	-0,85	-1,80	0,00867	NM_144966	CSF2
8107887	-0,48	-1,39	0,43505	-1,07	-2,09	0,00754	NM_000758	C6orf97
8122827	-0,48	-1,39	0,09018	-0,89	-1,85	0,02720	NM_025059	LOC349196
8149165	-0,48	-1,39	0,72535	-1,31	-2,48	0,10388	NR_027000	LOC349196
8149167	-0,48	-1,39	0,72535	-1,31	-2,48	0,10388	NR_027000	FBLN7
8044440	-0,48	-1,39	0,51021	2,04	4,10	0,00223	NM_153214	HS1BP3

8050594	-0,48	-1,39	0,07016	0,06	1,04	0,82740	NM_022460	SCUBE1
8076586	-0,48	-1,39	0,39415	-0,85	-1,80	0,04467	NM_173050	LGR6
7908816	-0,48	-1,39	0,02628	0,96	1,94	0,01602	NM_001017403	C10orf82
7936507	-0,48	-1,39	0,42576	-1,51	-2,84	0,00006	BC021737	INTU
8097307	-0,48	-1,39	0,35416	0,95	1,93	0,04014	NM_015693	OBFC1
7936134	-0,48	-1,39	0,32737	0,55	1,47	0,06299	NM_024928	CSRP3
7947099	-0,48	-1,39	0,22281	-0,84	-1,80	0,01293	NM_003476	---
7912531	-0,48	-1,39	0,08420	-0,68	-1,60	0,00091	---	UNQ6975
8051956	-0,48	-1,39	0,13709	-1,13	-2,19	0,01217	AY358255	TRIM52
8116534	-0,48	-1,39	0,09301	0,47	1,38	0,06121	NM_032765	ZSCAN5B
8039574	-0,48	-1,39	0,12630	-1,37	-2,59	0,03083	NM_001080456	DNAH9
8004957	-0,48	-1,39	0,28785	-1,26	-2,40	0,00026	NM_001372	C14orf57
7979959	-0,48	-1,39	0,02987	-1,62	-3,07	0,00310	ENST00000416212	NELL2
7962455	-0,48	-1,39	0,19371	-0,52	-1,44	0,03742	NM_006159	OTUD7B
7919699	-0,48	-1,39	0,33294	0,20	1,15	0,54460	NM_020205	SERPINB2
8021635	-0,48	-1,39	0,06250	-1,50	-2,82	0,00059	NM_001143818	C20orf26
8061272	-0,48	-1,39	0,20474	-0,22	-1,17	0,43536	NM_015585	ESR1
8122843	-0,48	-1,39	0,18935	-1,22	-2,33	0,00031	NM_000125	NCRNA00204
8176155	-0,48	-1,39	0,09796	-0,29	-1,23	0,21408	AY168775	---
7968759	-0,48	-1,39	0,18409	-1,14	-2,21	0,01580	---	ALOX12
8004221	-0,48	-1,39	0,00721	-0,82	-1,77	0,09345	NM_000697	CLEC9A
7953924	-0,48	-1,39	0,17948	-0,77	-1,71	0,14864	NM_207345	---
7975309	-0,48	-1,39	0,16704	-0,40	-1,32	0,12328	---	NOP2
7960575	-0,48	-1,39	0,41475	1,35	2,55	0,02010	NM_001033714	---
7929956	-0,48	-1,39	0,15061	-1,22	-2,34	0,01404	---	LOC100131642
8065120	-0,48	-1,39	0,36293	-0,44	-1,36	0,09641	AF258585	---
8134678	-0,48	-1,39	0,02209	-1,17	-2,25	0,04691	---	LOC100288778
8171066	-0,48	-1,39	0,54395	0,90	1,87	0,04021	NR_028269	---
8133845	-0,48	-1,39	0,25613	-0,77	-1,70	0,03296	---	C19orf63
8038565	-0,48	-1,39	0,48133	-1,78	-3,43	0,00231	NM_206538	---
7919390	-0,48	-1,39	0,27799	0,01	1,01	0,97080	---	IL12RB2
7902205	-0,48	-1,39	0,13858	-0,27	-1,20	0,42447	NM_001559	---
8050761	-0,48	-1,39	0,12524	-1,43	-2,69	0,04330	---	CDKL1
7978970	-0,48	-1,39	0,17587	-0,23	-1,18	0,45674	NM_004196	CCIN
8155200	-0,48	-1,39	0,06951	-0,55	-1,46	0,03466	NM_005893	FUT7
8159549	-0,48	-1,39	0,43950	-0,26	-1,20	0,26973	NM_004479	CA14
7905131	-0,48	-1,39	0,22151	-1,29	-2,44	0,00099	NM_012113	LOC349196
8149216	-0,48	-1,39	0,71122	-1,41	-2,65	0,08430	NR_027000	ZNF665
8039062	-0,48	-1,39	0,23451	-1,00	-2,00	0,00910	NM_024733	SERTAD1
8036902	-0,48	-1,40	0,03007	0,07	1,05	0,91495	NM_013376	OR1B1
8163962	-0,48	-1,40	0,04568	-1,12	-2,17	0,00770	NM_001004450	PAX9
7973974	-0,48	-1,40	0,05454	0,98	1,97	0,05642	NM_006194	C3AR1
7960874	-0,48	-1,40	0,00190	-1,16	-2,24	0,03238	NM_004054	NCRNA00158
8069612	-0,48	-1,40	0,22817	-1,72	-3,29	0,00202	NR_024027	C14orf105
7979378	-0,48	-1,40	0,07108	-1,43	-2,69	0,00027	NM_018168	MRPS12
8028563	-0,48	-1,40	0,34255	0,98	1,97	0,03474	NM_021107	CD244
7921677	-0,48	-1,40	0,08757	-1,13	-2,19	0,00018	NM_016382	PMFBP1
8002667	-0,48	-1,40	0,18792	0,19	1,14	0,52962	NM_031293	---
8121298	-0,48	-1,40	0,25542	-1,61	-3,04	0,00050	---	SLC2A7
7912211	-0,48	-1,40	0,30891	-0,44	-1,35	0,13277	NM_207420	HOOK2
8034521	-0,48	-1,40	0,52431	1,00	2,00	0,01076	NM_013312	CSTA
8082058	-0,48	-1,40	0,28101	-1,86	-3,63	0,00039	NM_005213	FGF9
7967993	-0,48	-1,40	0,35930	-0,66	-1,58	0,01090	NM_002010	SPG7
7997909	-0,48	-1,40	0,05345	0,64	1,56	0,07627	NM_003119	C2orf77
8056710	-0,48	-1,40	0,06158	-1,06	-2,08	0,00034	NM_001085447	LOC283174
7952805	-0,48	-1,40	0,38846	0,38	1,30	0,05377	NR_024344	MLYCD
7997525	-0,48	-1,40	0,12684	1,25	2,38	0,01051	NM_012213	HORMAD1
7919787	-0,48	-1,40	0,07760	-0,56	-1,47	0,07928	NM_032132	SERPINA4
7976481	-0,48	-1,40	0,10138	-1,24	-2,37	0,00038	NM_006215	AGAP7
7933574	-0,48	-1,40	0,36938	1,68	3,21	0,00120	NM_001077685	---
8124305	-0,48	-1,40	0,26237	-1,18	-2,26	0,06343	---	NCRNA00161
8068057	-0,48	-1,40	0,31276	-1,27	-2,42	0,10821	NR_026552	NRM
8124806	-0,48	-1,40	0,02809	1,82	3,53	0,04970	NM_007243	KCND3
7918558	-0,48	-1,40	0,13675	-1,10	-2,14	0,04992	NM_004980	AXIN1
7998211	-0,48	-1,40	0,09583	1,37	2,58	0,07862	NM_003502	C1orf115
7909862	-0,48	-1,40	0,28281	1,04	2,06	0,01640	NM_024709	SNORA36A
8170990	-0,48	-1,40	0,26378	-0,80	-1,74	0,07415	NR_002969	LLGL1
8005407	-0,48	-1,40	0,06455	1,21	2,31	0,05592	NM_004140	LUZP3P
8079420	-0,48	-1,40	0,46350	-0,56	-1,47	0,03972	AJ312775	---
8035956	-0,48	-1,40	0,29816	-1,44	-2,71	0,09324	---	---
7928367	-0,48	-1,40	0,22742	-0,75	-1,68	0,38792	---	SPATA8
7986340	-0,48	-1,40	0,01052	-1,22	-2,32	0,00138	NM_173499	---
8083893	-0,48	-1,40	0,13379	-0,72	-1,65	0,00176	---	TNRC18

8132980	-0,48	-1,40	0,32141	0,22	1,16	0,77067	NM_001080495	---
7983987	-0,48	-1,40	0,12765	-1,42	-2,67	0,05001	---	MYH15
8089329	-0,48	-1,40	0,03759	-0,59	-1,50	0,01881	NM_014981	DPM3
7920633	-0,48	-1,40	0,17113	0,37	1,30	0,31260	NM_018973	MAB21L2
8097773	-0,48	-1,40	0,26907	-1,13	-2,19	0,02721	NM_006439	---
7970905	-0,48	-1,40	0,05388	-0,52	-1,44	0,05603	---	---
7917726	-0,48	-1,40	0,22992	-0,87	-1,82	0,04422	---	ZNF814
8039692	-0,48	-1,40	0,71408	-0,75	-1,68	0,10427	NM_001144989	---
7969701	-0,48	-1,40	0,00285	-0,82	-1,76	0,03138	---	---
7952795	-0,48	-1,40	0,48390	-1,18	-2,26	0,02557	---	TTTY8
8176553	-0,48	-1,40	0,02810	-1,09	-2,13	0,00000	NR_001533	TTTY8
8177048	-0,48	-1,40	0,02810	-1,09	-2,13	0,00000	NR_001533	---
8006834	-0,48	-1,40	0,17554	-1,43	-2,70	0,00898	---	GSG1L
8000467	-0,48	-1,40	0,32293	-0,86	-1,81	0,02323	NM_001109763	MIR181A2
8157800	-0,48	-1,40	0,07018	-0,84	-1,79	0,00767	NR_029611	SLC7A9
8035941	-0,48	-1,40	0,29657	-0,91	-1,88	0,02395	NM_014270	NMUR1
8059720	-0,48	-1,40	0,27839	-0,11	-1,08	0,59893	NM_006056	CTDP1
8021884	-0,48	-1,40	0,01942	-0,26	-1,20	0,31689	NM_004715	OAZ3
7905474	-0,48	-1,40	0,05357	-0,52	-1,43	0,12784	NM_016178	LOC100132005
8119237	-0,48	-1,40	0,41159	-0,85	-1,80	0,00101	AK092662	WFDC5
8066482	-0,48	-1,40	0,43080	-0,65	-1,57	0,29376	NM_145652	OR1J1
8163956	-0,48	-1,40	0,35716	-1,02	-2,03	0,02323	NM_001004451	ZMYND12
7915425	-0,48	-1,40	0,13979	-0,83	-1,78	0,00536	NM_032257	FAM160B2
8145027	-0,48	-1,40	0,02987	0,16	1,11	0,81106	NM_022749	KIAA1210
8174670	-0,48	-1,40	0,01818	-1,15	-2,23	0,04881	NM_020721	---
7942287	-0,48	-1,40	0,26576	-1,57	-2,96	0,03271	---	---
8044931	-0,48	-1,40	0,07610	-0,65	-1,57	0,00998	---	TECPR1
8141180	-0,49	-1,40	0,26382	0,01	1,00	0,98279	NM_015395	IL28RA
7913776	-0,49	-1,40	0,07234	-0,12	-1,09	0,76333	NM_170743	ANKRD20B
8155414	-0,49	-1,40	0,13546	0,28	1,21	0,40154	NR_003366	CCDC29
8161384	-0,49	-1,40	0,13546	0,28	1,21	0,40154	ENST00000340460	C3orf15
8081903	-0,49	-1,40	0,24641	-0,58	-1,49	0,06799	NM_033364	FAM71A
7924163	-0,49	-1,40	0,24928	-1,53	-2,89	0,02246	NM_153606	NLRC5
7995926	-0,49	-1,40	0,01075	-0,15	-1,11	0,54882	NM_032206	ACSBG2
8025011	-0,49	-1,40	0,05249	-0,92	-1,89	0,00990	NM_030924	S100A1
7905581	-0,49	-1,40	0,07228	-0,09	-1,07	0,57602	NM_006271	CEBPE
7977928	-0,49	-1,40	0,33383	-0,71	-1,63	0,18020	NM_001805	---
7997738	-0,49	-1,40	0,31414	-1,34	-2,52	0,00637	---	ALOX15
8011680	-0,49	-1,40	0,34941	-0,84	-1,78	0,10419	NM_001140	RPL36
8024966	-0,49	-1,40	0,05044	0,32	1,25	0,40750	NM_015414	LOC100128751
7907090	-0,49	-1,40	0,12275	-1,45	-2,73	0,01158	AY194294	LYPD3
8037301	-0,49	-1,40	0,19590	1,54	2,90	0,00731	NM_014400	---
8065122	-0,49	-1,40	0,27937	-0,20	-1,15	0,18386	---	---
7939418	-0,49	-1,40	0,13525	-1,27	-2,41	0,00782	---	---
8090341	-0,49	-1,40	0,09519	-0,70	-1,62	0,03060	---	MED12L
8083360	-0,49	-1,40	0,14719	1,00	1,99	0,00029	NM_053002	SLITRK1
7972231	-0,49	-1,40	0,08145	-1,23	-2,35	0,00437	NM_052910	---
8081331	-0,49	-1,40	0,10233	-0,67	-1,59	0,00036	---	GPR116
8126798	-0,49	-1,40	0,25428	-1,09	-2,13	0,01038	NM_015234	DOLPP1
8158513	-0,49	-1,40	0,20283	1,53	2,88	0,01962	NM_020438	MIR148B
7955906	-0,49	-1,40	0,05744	-1,09	-2,13	0,03583	NR_029894	AKAP5
7975066	-0,49	-1,40	0,08199	-0,28	-1,21	0,52550	NM_004857	SH2D1A
8169792	-0,49	-1,40	0,12569	-0,57	-1,48	0,04481	NM_002351	P2RY6
7942409	-0,49	-1,40	0,22137	-0,06	-1,04	0,79061	NM_176796	SNORD116-29
7982006	-0,49	-1,40	0,04857	-1,42	-2,67	0,00492	NR_003360	PRPS1L1 // PRPS1L1
8138435	-0,49	-1,40	0,01221	-0,83	-1,78	0,00158	NM_175886 // NM_175886	NUDT16
8082667	-0,49	-1,40	0,28612	-0,85	-1,80	0,14628	NR_033268	MGC2752
8031973	-0,49	-1,40	0,26610	-0,56	-1,48	0,28595	NR_026052	PKD1L3
8002629	-0,49	-1,40	0,08537	-0,81	-1,75	0,01410	NM_181536	GPR114
7996064	-0,49	-1,40	0,37415	-0,86	-1,82	0,01203	NM_153837	C16orf73
7998615	-0,49	-1,40	0,03180	-1,09	-2,13	0,04211	NM_001163560	---
8053360	-0,49	-1,40	0,43722	-1,84	-3,58	0,00143	---	SSX9
8172399	-0,49	-1,40	0,40181	-0,18	-1,14	0,22692	ENST00000376909	---
8001348	-0,49	-1,40	0,27317	-0,62	-1,54	0,00300	---	KRT83
7963359	-0,49	-1,40	0,39951	-0,45	-1,37	0,15359	NM_002282	SNORD60
7998722	-0,49	-1,40	0,35910	-0,28	-1,21	0,03836	NR_002736	TSGA10
8054166	-0,49	-1,40	0,20750	-0,32	-1,25	0,13619	NM_025244	---
8083322	-0,49	-1,40	0,06726	-1,18	-2,26	0,05715	---	---
8061881	-0,49	-1,40	0,44347	-1,40	-2,63	0,00225	---	---
8100143	-0,49	-1,40	0,06749	-0,88	-1,84	0,00454	---	C16orf85
8003318	-0,49	-1,40	0,17197	-0,37	-1,30	0,09181	AK127438	MUL1
7913242	-0,49	-1,40	0,30407	0,15	1,11	0,69262	NM_024544	---
7901314	-0,49	-1,40	0,40443	-1,34	-2,53	0,00814	---	---

7927708	-0,49	-1,40	0,38038	-0,72	-1,65	0,01133	---	C6orf134
8177834	-0,49	-1,40	0,12199	1,24	2,37	0,00184	NM_024909	SNORD14E
7952335	-0,49	-1,40	0,29959	-1,14	-2,20	0,04958	NR_003125	---
7922472	-0,49	-1,40	0,15933	-0,85	-1,80	0,05768	---	TRIM26
8124726	-0,49	-1,40	0,01143	0,71	1,64	0,11456	NM_003449	PCDH9
7971922	-0,49	-1,40	0,33631	-0,81	-1,75	0,01157	NM_203487	ARHGAP22
7933469	-0,49	-1,40	0,32410	-0,50	-1,42	0,09117	NM_021226	ABCG4
7944447	-0,49	-1,40	0,24127	0,63	1,54	0,19937	NM_022169	CRX
8029978	-0,49	-1,40	0,08626	-1,48	-2,79	0,00056	NM_000554	ABCB10
7924956	-0,49	-1,40	0,04788	0,67	1,59	0,12714	NM_012089	HPGDS
8101780	-0,49	-1,40	0,08773	-0,81	-1,75	0,04314	NM_014485	SLC14A2
8021058	-0,49	-1,40	0,32696	-1,17	-2,26	0,00032	NM_007163	---
8093126	-0,49	-1,40	0,10144	-0,75	-1,68	0,15385	---	ZNF418
8039698	-0,49	-1,40	0,18498	-0,45	-1,37	0,00368	NM_133460	EXD1
7987623	-0,49	-1,40	0,05418	-0,31	-1,24	0,30157	NM_152596	---
8046626	-0,49	-1,41	0,05873	-1,30	-2,46	0,03067	---	USP6
8004086	-0,49	-1,41	0,19542	-0,68	-1,60	0,00600	NM_004505	---
7910377	-0,49	-1,41	0,54013	-1,08	-2,11	0,24693	---	---
8092226	-0,49	-1,41	0,25500	-0,37	-1,29	0,01791	---	---
8128936	-0,49	-1,41	0,08405	-0,74	-1,67	0,00021	---	TAP2
8178841	-0,49	-1,41	0,00632	0,97	1,96	0,05401	NM_018833	LOC100132354
8119915	-0,49	-1,41	0,28847	-0,06	-1,04	0,52744	NR_024478	SNORD115-44
7982094	-0,49	-1,41	0,24781	-1,62	-3,07	0,04717	NR_003359	---
7937711	-0,49	-1,41	0,53184	-0,69	-1,62	0,03450	---	ZNF599
8036038	-0,49	-1,41	0,07850	0,05	1,04	0,84626	NM_001007248	---
8023640	-0,49	-1,41	0,04894	-1,06	-2,09	0,00145	---	MCRS1
7963064	-0,49	-1,41	0,20109	1,14	2,20	0,13185	NM_006337	HFM1
7917634	-0,49	-1,41	0,08523	-0,73	-1,66	0,00076	NM_001017975	KBTBD12
8082400	-0,49	-1,41	0,38544	-0,86	-1,82	0,20501	NM_207335	GPR45
8044124	-0,49	-1,41	0,37193	-1,24	-2,36	0,00804	NM_007227	ACTL6B
8141580	-0,49	-1,41	0,21608	-1,79	-3,47	0,00970	NM_016188	---
8067860	-0,49	-1,41	0,34415	0,31	1,24	0,46053	---	FAM149A
8098620	-0,49	-1,41	0,06561	-0,43	-1,35	0,02837	NM_015398	CRYGS
8092621	-0,49	-1,41	0,32047	-0,43	-1,34	0,00291	NM_017541	CA6
7897426	-0,49	-1,41	0,13413	-0,45	-1,37	0,04320	NM_001215	KAZ
7898102	-0,49	-1,41	0,44955	-0,76	-1,69	0,00492	NM_201628	NCLN
8024584	-0,49	-1,41	0,28052	2,51	5,68	0,01765	NM_020170	CUL7
8126486	-0,49	-1,41	0,17985	0,56	1,47	0,16861	NM_014780	DOK1
8042917	-0,49	-1,41	0,15486	-0,03	-1,02	0,75402	NM_001381	SMOX
8060745	-0,49	-1,41	0,21971	2,47	5,53	0,00134	NM_175839	PKDREJ
8076754	-0,49	-1,41	0,17099	-0,77	-1,71	0,00034	NM_006071	SLC25A37
8145281	-0,49	-1,41	0,17675	3,27	9,64	0,00191	NM_016612	TBX2
8008969	-0,49	-1,41	0,12760	-1,38	-2,61	0,02676	NM_005994	CA9
8155083	-0,49	-1,41	0,13137	-1,09	-2,12	0,08419	NM_001216	OR10H3
8026486	-0,49	-1,41	0,28319	-1,40	-2,64	0,01061	NM_013938	SARM1
8005865	-0,49	-1,41	0,02219	-0,02	-1,01	0,94499	NM_015077	KIAA1920
7985416	-0,49	-1,41	0,33494	0,09	1,07	0,82323	AB067507	KIAA1920
7985457	-0,49	-1,41	0,33494	0,09	1,07	0,82323	AB067507	KIAA1920
7985688	-0,49	-1,41	0,33494	0,09	1,07	0,82323	AB067507	KIAA1920
7990941	-0,49	-1,41	0,33494	0,09	1,07	0,82323	AB067507	SLC17A6
7938975	-0,49	-1,41	0,13512	-0,78	-1,72	0,00028	NM_020346	CA5A
8003313	-0,49	-1,41	0,25611	-1,19	-2,28	0,21300	NM_001739	SFRS13B
8128079	-0,49	-1,41	0,15587	0,69	1,62	0,01892	NM_080743	TGFB1I1
7995206	-0,49	-1,41	0,37037	-1,11	-2,16	0,05489	NM_001042454	ARID5A
8043621	-0,49	-1,41	0,15689	0,06	1,04	0,79292	NM_212481	TMEM8B
8155148	-0,49	-1,41	0,27632	-0,48	-1,40	0,42694	NM_016446	GUCY2F
8174448	-0,49	-1,41	0,04240	-1,77	-3,40	0,00189	NM_001522	RNU2-1
7948894	-0,49	-1,41	0,49548	-0,77	-1,71	0,36093	NR_002716	SEC16B
7922550	-0,49	-1,41	0,09961	-0,73	-1,65	0,05457	NM_033127	ZBTB3
7948829	-0,49	-1,41	0,20683	0,01	1,01	0,94115	NM_024784	---
8094357	-0,49	-1,41	0,08636	-0,43	-1,34	0,00154	---	CD86
8082035	-0,49	-1,41	0,04604	-1,06	-2,09	0,00784	NM_175862	C18orf62
8023876	-0,49	-1,41	0,38080	-0,67	-1,59	0,00511	NM_001037331	ST6GALNAC3
7902425	-0,49	-1,41	0,17973	-0,66	-1,58	0,09395	NM_152996	UNC5CL
8126248	-0,49	-1,41	0,04432	-0,02	-1,01	0,90302	NM_173561	---
8044107	-0,49	-1,41	0,26484	-1,05	-2,08	0,01501	---	RGS20
8146435	-0,49	-1,41	0,16950	-0,24	-1,18	0,35748	NM_170587	CNIH3
7910022	-0,49	-1,41	0,04918	-0,55	-1,46	0,10988	NM_152495	MARCKS
8121563	-0,50	-1,41	0,05214	-1,63	-3,10	0,00060	NM_002356	IL31
7967226	-0,50	-1,41	0,16407	-1,37	-2,58	0,01841	NM_001014336	---
8019960	-0,50	-1,41	0,04099	-1,28	-2,44	0,00349	---	ZSWIM2
8057563	-0,50	-1,41	0,00959	-0,88	-1,84	0,00084	NM_182521	ZIC1
8083233	-0,50	-1,41	0,04432	3,17	9,00	0,00144	NM_003412	---

8045931	-0,50	-1,41	0,14088	0,01	1,01	0,92822	---	---
8138948	-0,50	-1,41	0,37764	-0,99	-1,98	0,00000	---	---
8083848	-0,50	-1,41	0,12868	-0,56	-1,47	0,01909	---	---
8022623	-0,50	-1,41	0,52927	-0,75	-1,69	0,11310	---	---
8065196	-0,50	-1,41	0,08572	-0,80	-1,74	0,19880	---	C10orf62
7929674	-0,50	-1,41	0,07397	-0,59	-1,50	0,05356	NM_001009997	LOC285033
8043583	-0,50	-1,41	0,13376	-1,08	-2,11	0,09071	AK057419	ZNF643
7900438	-0,50	-1,41	0,26467	0,44	1,35	0,38428	NM_023070	---
8052231	-0,50	-1,41	0,36391	-0,79	-1,73	0,00329	---	FZD1
8134117	-0,50	-1,41	0,45528	-0,95	-1,93	0,01988	NM_003505	NLRP5
8031600	-0,50	-1,41	0,27183	-1,05	-2,07	0,03410	NM_153447	SNORA69
8174715	-0,50	-1,41	0,02068	-0,73	-1,66	0,08666	NR_002584	SLC17A2
8124365	-0,50	-1,41	0,26384	-0,76	-1,69	0,00035	NM_005835	ME3
7950864	-0,50	-1,41	0,10443	1,40	2,65	0,01443	NM_001014811	SLC44A4
8125149	-0,50	-1,41	0,35093	-1,38	-2,61	0,00395	NM_025257	SLC44A4
8178653	-0,50	-1,41	0,35093	-1,38	-2,61	0,00395	NM_025257	SLC44A4
8179861	-0,50	-1,41	0,35093	-1,38	-2,61	0,00395	NM_025257	---
7913801	-0,50	-1,41	0,34595	-0,54	-1,45	0,00969	---	CD5L
7921332	-0,50	-1,41	0,25886	-2,10	-4,29	0,00123	NM_005894	PARD6B
8063410	-0,50	-1,41	0,14301	2,64	6,25	0,02493	NM_032521	LONP1
8033002	-0,50	-1,41	0,07579	1,08	2,11	0,03615	NM_004793	TRIM29
7952290	-0,50	-1,41	0,01298	-0,68	-1,61	0,00864	NM_012101	METTL11A
8158544	-0,50	-1,41	0,14928	0,38	1,30	0,12055	NM_014064	SNORD115-26
7982058	-0,50	-1,41	0,62840	-2,27	-4,83	0,00013	NR_003343	---
8088891	-0,50	-1,41	0,35874	-1,71	-3,28	0,01156	---	PEX11A
7991323	-0,50	-1,41	0,18616	-0,32	-1,25	0,38224	NM_003847	---
8021714	-0,50	-1,41	0,18370	-0,56	-1,47	0,14986	---	---
8146458	-0,50	-1,41	0,18780	-0,35	-1,27	0,02324	---	MED25
8030498	-0,50	-1,41	0,15663	-0,49	-1,40	0,14413	NM_030973	ALB
8095628	-0,50	-1,41	0,08591	-0,34	-1,27	0,01229	NM_000477	---
7979999	-0,50	-1,41	0,25964	-0,44	-1,35	0,16570	---	FN3K
8010841	-0,50	-1,41	0,13298	0,69	1,61	0,25420	NM_022158	MRGPRX1
7947093	-0,50	-1,41	0,24606	-1,24	-2,37	0,00161	NM_147199	---
8084171	-0,50	-1,41	0,00242	-1,31	-2,49	0,17548	---	---
7901374	-0,50	-1,41	0,12164	-0,95	-1,93	0,00012	---	---
8068018	-0,50	-1,41	0,25757	-1,12	-2,17	0,00458	---	PIP5K1B
8155673	-0,50	-1,41	0,06991	-0,77	-1,70	0,00212	NM_003558	ZNF687
7905382	-0,50	-1,41	0,18073	0,99	1,98	0,16088	NM_020832	MYO5C
7988876	-0,50	-1,41	0,19757	3,06	8,31	0,00016	NM_018728	OR4S1
7939871	-0,50	-1,41	0,29802	-1,15	-2,22	0,00815	NM_001004725	MYO19
8014454	-0,50	-1,41	0,08666	3,07	8,38	0,00036	NM_001163735	THAP3
7897329	-0,50	-1,41	0,47321	-0,14	-1,10	0,74872	NM_138350	AMELX
8166006	-0,50	-1,41	0,28160	-1,43	-2,69	0,00120	NM_182680	SLC1A3
8104930	-0,50	-1,41	0,25830	-1,15	-2,22	0,00059	NM_004172	IL17RC
8077688	-0,50	-1,41	0,36624	-0,10	-1,07	0,77782	NM_153461	INPP5E
8165171	-0,50	-1,41	0,04756	0,68	1,60	0,45730	NM_019892	MCART2
8022763	-0,50	-1,41	0,01449	-0,31	-1,24	0,31498	NM_001034172	---
7981324	-0,50	-1,41	0,02041	-1,30	-2,46	0,03430	---	ZNRD1
8117822	-0,50	-1,41	0,15175	1,14	2,21	0,02468	NM_170783	LYSMD4
7991503	-0,50	-1,41	0,07483	0,22	1,16	0,41513	NM_152449	TNNI3K
7902317	-0,50	-1,41	0,04893	-0,90	-1,86	0,00095	NM_015978	CTAGEP
7931389	-0,50	-1,41	0,11365	-1,04	-2,05	0,00095	BC036527	---
8137900	-0,50	-1,41	0,57774	-2,19	-4,57	0,03490	---	---
8137903	-0,50	-1,41	0,57774	-2,19	-4,57	0,03490	---	SNORD54
8150877	-0,50	-1,41	0,14483	-0,87	-1,83	0,01121	NR_002437	TOR2A
8164243	-0,50	-1,41	0,24892	1,13	2,19	0,25204	NM_130459	LCE3A
7920191	-0,50	-1,41	0,50039	-0,29	-1,22	0,67663	NM_178431	GPHA2
7949320	-0,50	-1,41	0,17930	-0,04	-1,03	0,91978	NM_130769	---
8034326	-0,50	-1,41	0,30014	-0,97	-1,96	0,01344	---	SH2D5
7913290	-0,50	-1,41	0,08052	0,30	1,23	0,30598	NM_001103161	FBXW10
8005483	-0,50	-1,42	0,32491	-1,07	-2,10	0,00289	NM_031456	TROAP
7955195	-0,50	-1,42	0,16676	1,07	2,09	0,02028	NM_005480	PARD6A
7996647	-0,50	-1,42	0,33330	0,97	1,96	0,02532	NM_016948	NLRC4
8051396	-0,50	-1,42	0,08093	-0,61	-1,53	0,03299	NM_021209	DEFB123
8061519	-0,50	-1,42	0,05759	-1,11	-2,16	0,09459	NM_153324	MIR181A1
7923175	-0,50	-1,42	0,23537	-0,52	-1,43	0,10517	NR_029626	VSIG10L
8038785	-0,50	-1,42	0,36652	0,02	1,01	0,94887	NM_001163922	CRHR2
8138865	-0,50	-1,42	0,19482	-1,03	-2,05	0,02546	NM_001883	---
8005223	-0,50	-1,42	0,11767	-1,07	-2,10	0,00718	---	BAX
8030158	-0,50	-1,42	0,13737	-0,74	-1,67	0,06347	NR_027882	RNFT2
7959025	-0,50	-1,42	0,18982	1,31	2,49	0,02427	NM_001109903	C20orf108
8063478	-0,50	-1,42	0,11342	-0,70	-1,62	0,10536	NM_080821	C15orf33
7988625	-0,50	-1,42	0,27466	-0,31	-1,24	0,36545	NM_152647	UGT2A3

8100760	-0,50	-1,42	0,02961	-1,10	-2,14	0,11725	NM_024743	---
8057439	-0,50	-1,42	0,48161	-1,18	-2,27	0,01070	---	PDE1B
7955943	-0,50	-1,42	0,09420	-0,97	-1,96	0,00317	NM_000924	---
8050656	-0,50	-1,42	0,04326	-0,89	-1,86	0,01569	---	ACOXL
8044353	-0,50	-1,42	0,39124	-0,02	-1,01	0,92197	NM_001142807	MIR302A
8102404	-0,50	-1,42	0,12393	-0,94	-1,91	0,04076	NR_029835	TRPV5
8143518	-0,50	-1,42	0,46044	-0,96	-1,95	0,00233	NM_019841	HCRP1
8119525	-0,50	-1,42	0,16804	-1,22	-2,33	0,15158	AY465895	TH
7945712	-0,50	-1,42	0,08299	-0,81	-1,76	0,00164	NM_199292	CES8
7996377	-0,50	-1,42	0,02206	1,21	2,32	0,04367	NM_173815	HSD17B8
8118622	-0,50	-1,42	0,06834	-1,40	-2,63	0,00106	NM_014234	HSD17B8
8178234	-0,50	-1,42	0,06834	-1,40	-2,63	0,00106	NM_014234	HSD17B8
8179534	-0,50	-1,42	0,06834	-1,40	-2,63	0,00106	NM_014234	PPCDC
7984943	-0,50	-1,42	0,29002	-0,36	-1,29	0,05988	NM_021823	DDX55
7959604	-0,50	-1,42	0,41118	1,72	3,30	0,00040	NM_020936	ATP6V0A4
8143221	-0,50	-1,42	0,12984	-0,80	-1,74	0,02952	NM_020632	PDE4A
8025633	-0,50	-1,42	0,17687	0,45	1,37	0,11879	NM_001111307	EDEM1
8085116	-0,50	-1,42	0,07957	-1,43	-2,69	0,05853	NM_014674	MIR23B
8156569	-0,50	-1,42	0,41609	-1,08	-2,12	0,01817	NR_029664	---
8100003	-0,50	-1,42	0,14907	-0,55	-1,46	0,00388	---	---
7986346	-0,50	-1,42	0,06597	-1,26	-2,40	0,02904	---	ZCCHC24
7934690	-0,50	-1,42	0,31078	-0,43	-1,35	0,59132	NM_153367	---
7967709	-0,50	-1,42	0,36581	-0,17	-1,12	0,28743	---	ASB12
8173238	-0,50	-1,42	0,38563	1,96	3,90	0,00390	NM_130388	---
7900212	-0,50	-1,42	0,06140	-1,23	-2,34	0,03252	---	APOBEC3H
8073096	-0,50	-1,42	0,35011	-0,55	-1,47	0,01538	NM_001166003	WWC3
8165947	-0,50	-1,42	0,09820	0,26	1,20	0,51495	NM_015691	DNAH10
7959681	-0,50	-1,42	0,08992	-0,38	-1,30	0,11008	NM_207437	LOC349196
8149151	-0,50	-1,42	0,69309	-0,98	-1,97	0,15488	NR_027000	LOC349196
8149214	-0,50	-1,42	0,69309	-0,98	-1,97	0,15488	NR_027000	---
8051319	-0,50	-1,42	0,20334	-0,46	-1,37	0,11587	---	PDCL2
8100458	-0,50	-1,42	0,19427	-1,03	-2,04	0,00101	NM_152401	TSHB
7904203	-0,50	-1,42	0,18265	-1,06	-2,08	0,00107	NM_000549	HBE1
7946061	-0,50	-1,42	0,35020	-1,07	-2,11	0,07108	NM_005330	---
7983773	-0,50	-1,42	0,12892	-0,19	-1,14	0,53532	---	SMOC2
8123446	-0,50	-1,42	0,14640	-1,08	-2,12	0,00418	NM_022138	TMEM84
7984511	-0,50	-1,42	0,17309	-1,12	-2,17	0,01678	NR_026949	NMU
8100464	-0,50	-1,42	0,03302	0,47	1,39	0,45977	NM_006681	CCDC75
8041487	-0,50	-1,42	0,05269	-0,62	-1,53	0,04986	NM_174931	MEGF11
7989887	-0,50	-1,42	0,10866	-0,77	-1,70	0,08521	NM_032445	CXorf56
8174677	-0,50	-1,42	0,26419	-0,52	-1,43	0,52090	NM_022101	TP63
8084766	-0,50	-1,42	0,00691	-1,27	-2,42	0,09985	NM_003722	MPEG1
7948364	-0,50	-1,42	0,13895	-1,47	-2,76	0,00066	NM_001039396	PRR3
8117922	-0,50	-1,42	0,13269	0,59	1,51	0,08699	NM_025263	---
8092748	-0,50	-1,42	0,20510	-1,03	-2,04	0,01018	---	---
8020825	-0,50	-1,42	0,16349	-1,30	-2,46	0,00574	---	---
8059339	-0,50	-1,42	0,08722	-1,34	-2,52	0,00024	---	ANKRD32
8113124	-0,50	-1,42	0,06876	-0,20	-1,15	0,37812	NM_032290	EDDM3B
7973098	-0,50	-1,42	0,20010	-0,85	-1,80	0,00607	NM_022360	KCTD4
7971369	-0,50	-1,42	0,06922	-0,80	-1,74	0,00463	NM_198404	TRPM4
8030251	-0,50	-1,42	0,29767	-0,74	-1,67	0,02535	NM_017636	C2orf16
8040980	-0,50	-1,42	0,07815	-1,19	-2,28	0,01494	NM_032266	---
8175232	-0,50	-1,42	0,42877	-1,02	-2,03	0,04425	---	NPBWR2
8067818	-0,50	-1,42	0,24635	-1,89	-3,71	0,00232	NM_005286	---
8101697	-0,50	-1,42	0,11950	-1,05	-2,08	0,00591	---	ARL6
8081158	-0,50	-1,42	0,05675	-0,48	-1,39	0,23408	NM_032146	C12orf48
7958031	-0,51	-1,42	0,13785	1,20	2,30	0,02483	AK302724	SGSM3
8073259	-0,51	-1,42	0,05823	-0,21	-1,16	0,42905	NM_015705	ZNF23
8002533	-0,51	-1,42	0,26557	-0,79	-1,73	0,00355	NM_145911	C21orf122
8070867	-0,51	-1,42	0,44854	-1,19	-2,28	0,04618	ENST00000416043	C9orf144B
8160898	-0,51	-1,42	0,13958	-0,52	-1,43	0,00405	NM_001141917	FNDC4
8051187	-0,51	-1,42	0,24578	-0,63	-1,54	0,15280	NM_022823	KRT5
7963427	-0,51	-1,42	0,26434	-1,09	-2,13	0,06120	NM_000424	FREM2
7968678	-0,51	-1,42	0,11354	1,01	2,01	0,01497	NM_207361	PPP1R2P3
8109505	-0,51	-1,42	0,24953	-0,37	-1,29	0,10714	NR_002168	---
8035865	-0,51	-1,42	0,54585	-2,06	-4,17	0,02227	---	MRPL34
8026724	-0,51	-1,42	0,25621	1,38	2,61	0,00533	NM_023937	PCDH7
8094520	-0,51	-1,42	0,01817	-1,13	-2,18	0,00204	NM_032456	TTLL3
8077612	-0,51	-1,42	0,17279	0,46	1,38	0,00215	NM_001025930	COMMD4
7984989	-0,51	-1,42	0,07662	1,43	2,70	0,01378	NM_017828	FAM27A
8155521	-0,51	-1,42	0,72992	-0,28	-1,22	0,68598	NR_024060	FCGR1A
7905060	-0,51	-1,42	0,07869	-1,54	-2,91	0,00097	NM_000566	---
7972225	-0,51	-1,42	0,04140	-0,91	-1,88	0,05340	---	C17orf28

8018264	-0,51	-1,42	0,32929	1,67	3,17	0,00398	NM_030630	ZMAT1
8174119	-0,51	-1,42	0,18394	-0,77	-1,71	0,07920	NM_032441	TMEM149
8036136	-0,51	-1,42	0,31627	0,45	1,37	0,53056	NM_024660	CASZ1
7912343	-0,51	-1,42	0,54489	-0,10	-1,07	0,68936	NM_001079843	MAS1L
8124648	-0,51	-1,42	0,33595	-1,35	-2,55	0,00926	NM_052967	MAS1L
8178293	-0,51	-1,42	0,33595	-1,35	-2,55	0,00926	NM_052967	MAS1L
8179593	-0,51	-1,42	0,33595	-1,35	-2,55	0,00926	NM_052967	ELL3
7988212	-0,51	-1,42	0,02657	0,78	1,72	0,05327	NM_025165	RNASE12
7977606	-0,51	-1,42	0,33495	-0,97	-1,96	0,05156	NM_001024822	C5orf44
8105661	-0,51	-1,42	0,04999	0,07	1,05	0,78807	NR_003545	GJA10
8121132	-0,51	-1,42	0,20405	-0,46	-1,38	0,04923	NM_032602	MERTK
8044391	-0,51	-1,42	0,43177	2,15	4,44	0,01957	NM_006343	HEPACAM2
8140984	-0,51	-1,42	0,12732	-0,69	-1,62	0,06962	NM_001039372	SPANXN2
8175578	-0,51	-1,42	0,16632	-0,48	-1,39	0,16050	NM_001009615	---
8151306	-0,51	-1,42	0,12125	-0,99	-1,98	0,00031	---	TTC32
8050503	-0,51	-1,42	0,03100	-0,67	-1,59	0,03999	NM_001008237	SERINC2
7914350	-0,51	-1,42	0,22779	-0,92	-1,89	0,02811	NM_178865	ADAM21
7975406	-0,51	-1,42	0,12000	-0,17	-1,12	0,73311	NM_003813	RAB19
8136580	-0,51	-1,42	0,12655	0,07	1,05	0,60965	NM_001008749	TRIM65
8018502	-0,51	-1,42	0,24353	-0,44	-1,36	0,35458	NM_173547	DKKL1
8030292	-0,51	-1,42	0,23094	-0,43	-1,35	0,35888	NM_014419	---
8043446	-0,51	-1,42	0,05003	-1,06	-2,08	0,00079	---	PRODH
8074335	-0,51	-1,42	0,12908	0,25	1,19	0,52394	NM_016335	---
8006679	-0,51	-1,42	0,50892	-1,44	-2,70	0,03173	---	TSHZ2
8063437	-0,51	-1,42	0,05957	-1,48	-2,80	0,00345	NM_173485	SERINC4
7988227	-0,51	-1,42	0,21136	-0,67	-1,59	0,02740	NM_001033517	CBX2
8010320	-0,51	-1,42	0,25728	-0,15	-1,11	0,78539	NM_005189	---
8008912	-0,51	-1,42	0,09225	-0,57	-1,49	0,07460	---	HESX1
8088285	-0,51	-1,42	0,08232	-0,63	-1,55	0,13445	NM_003865	TMC6
8018823	-0,51	-1,42	0,20706	0,20	1,15	0,59129	NM_001127198	---
8012906	-0,51	-1,42	0,24279	-1,24	-2,36	0,01158	---	WDR49
8091922	-0,51	-1,42	0,17629	-0,79	-1,73	0,10702	NM_178824	---
7932832	-0,51	-1,42	0,12683	-0,81	-1,75	0,06905	---	SLC12A7
8110755	-0,51	-1,42	0,03365	2,46	5,49	0,00521	NM_006598	MUC13
8090180	-0,51	-1,42	0,15653	-0,63	-1,55	0,01353	NM_033049	C2orf80
8058542	-0,51	-1,42	0,11264	-0,86	-1,82	0,04419	NM_001099334	---
8102305	-0,51	-1,42	0,18658	-0,75	-1,68	0,01261	---	NXT1
8061368	-0,51	-1,42	0,33639	-1,46	-2,75	0,04714	NM_013248	CA2
8147132	-0,51	-1,42	0,11711	4,48	22,38	0,00000	NM_000067	ANTXRL
7927294	-0,51	-1,42	0,19222	-0,54	-1,45	0,00892	NR_003601	---
8099027	-0,51	-1,42	0,02934	-0,91	-1,88	0,03087	---	WDR8
7911839	-0,51	-1,42	0,14776	0,41	1,33	0,12357	NM_017818	MIR519A2
8031039	-0,51	-1,42	0,10978	-1,23	-2,34	0,03250	NR_030222	---
8044105	-0,51	-1,42	0,03001	-0,86	-1,81	0,02329	---	C7orf53
8135532	-0,51	-1,42	0,26081	-0,77	-1,71	0,01290	NM_182597	---
8162584	-0,51	-1,42	0,15808	-0,61	-1,53	0,03907	---	---
7902909	-0,51	-1,42	0,26660	-0,99	-1,99	0,11574	---	HOXA4
8138728	-0,51	-1,42	0,03397	-0,47	-1,38	0,05111	NM_002141	---
8127995	-0,51	-1,42	0,10641	-0,92	-1,89	0,03640	---	---
7943956	-0,51	-1,42	0,05850	-0,94	-1,92	0,10369	---	HEATR7B2
8111821	-0,51	-1,42	0,16365	-0,81	-1,76	0,00559	NM_173489	01. Dez
8157477	-0,51	-1,42	0,12527	-0,94	-1,92	0,00840	NM_017418	---
7955043	-0,51	-1,42	0,03370	-0,58	-1,49	0,02760	---	SNX10
8131957	-0,51	-1,42	0,20271	2,19	4,56	0,00076	NM_013322	HAPLN3
7991224	-0,51	-1,42	0,26530	-0,47	-1,38	0,24936	NM_178232	---
8143899	-0,51	-1,43	0,16511	-1,40	-2,64	0,05500	---	---
8163616	-0,51	-1,43	0,38833	-0,79	-1,73	0,03103	---	IQCC
7899727	-0,51	-1,43	0,28038	-0,19	-1,14	0,45851	NM_001160042	PDZD2
8104693	-0,51	-1,43	0,26763	-0,45	-1,36	0,05458	NM_178140	STARD8
8168028	-0,51	-1,43	0,03814	-0,49	-1,40	0,46884	NM_001142503	---
8023187	-0,51	-1,43	0,08359	-0,70	-1,63	0,00218	---	---
8078956	-0,51	-1,43	0,10064	-0,87	-1,83	0,00013	---	C20orf79
8061224	-0,51	-1,43	0,34040	-0,90	-1,87	0,07240	NM_178483	PPM1F
8074806	-0,51	-1,43	0,33474	-0,07	-1,05	0,68336	NM_014634	NDUFS7
8024273	-0,51	-1,43	0,20851	0,38	1,30	0,53816	NM_024407	FBXO6
7897728	-0,51	-1,43	0,07813	-0,39	-1,31	0,13225	NM_018438	IL5
8114023	-0,51	-1,43	0,06601	-0,90	-1,87	0,01564	NM_000879	---
8098498	-0,51	-1,43	0,17796	-0,73	-1,66	0,06075	---	---
8083492	-0,51	-1,43	0,12552	-1,12	-2,17	0,00112	---	---
8063449	-0,51	-1,43	0,33560	-2,29	-4,89	0,01160	---	---
7994093	-0,51	-1,43	0,25513	-1,21	-2,31	0,00085	---	---
8147501	-0,51	-1,43	0,07504	-1,09	-2,13	0,07203	---	KRTAP6-3
8068145	-0,51	-1,43	0,35501	-1,01	-2,02	0,02792	NM_181605	OR4X1

7939869	-0,51	-1,43	0,13655	-1,14	-2,21	0,01696	NM_001004726	---
8113274	-0,51	-1,43	0,58060	-1,28	-2,42	0,10943	---	NAGPA
7999279	-0,51	-1,43	0,15934	0,49	1,40	0,49006	NM_016256	FLJ25758
8033349	-0,51	-1,43	0,31565	-1,48	-2,79	0,06170	NR_024372	---
8083538	-0,51	-1,43	0,26996	-0,67	-1,59	0,00924	---	---
7993846	-0,51	-1,43	0,32282	-1,06	-2,09	0,01540	---	RANBP3L
8111569	-0,51	-1,43	0,28878	-0,55	-1,46	0,01033	NM_001161429	PDIK1L
7899087	-0,51	-1,43	0,09509	0,31	1,24	0,53222	NR_026686	TCEAL1
8169049	-0,51	-1,43	0,31140	-0,93	-1,90	0,00920	NM_004780	TMEM42
8079274	-0,51	-1,43	0,02724	0,06	1,04	0,80962	NM_144638	ZNF454
8110478	-0,51	-1,43	0,17937	-1,53	-2,89	0,05251	NM_182594	BRI3BP
7959777	-0,51	-1,43	0,02321	3,06	8,33	0,00503	NM_080626	---
8176817	-0,51	-1,43	0,17399	-0,93	-1,91	0,00909	---	---
8176943	-0,51	-1,43	0,17399	-0,93	-1,91	0,00909	---	---
8177389	-0,51	-1,43	0,17399	-0,93	-1,91	0,00909	---	ABCA10
8018006	-0,51	-1,43	0,07112	-0,50	-1,41	0,08526	NM_080282	TDGF3
8169385	-0,51	-1,43	0,40526	-0,73	-1,66	0,28503	NR_002718	LOC349196
8144410	-0,51	-1,43	0,68782	-1,32	-2,50	0,10562	NR_027000	---
8112424	-0,51	-1,43	0,06997	-1,05	-2,07	0,00101	---	FLJ40288
8136281	-0,51	-1,43	0,08810	-1,12	-2,17	0,03166	ENST00000332558	LOC100288871
8106929	-0,51	-1,43	0,15491	-0,84	-1,79	0,00379	XR_078322	GFRA2
8149629	-0,51	-1,43	0,41613	0,04	1,03	0,88570	NM_001495	ZNF557
8025132	-0,51	-1,43	0,07811	0,28	1,21	0,34044	NM_024341	C7orf10
8132439	-0,51	-1,43	0,14794	-0,28	-1,21	0,22292	NM_024728	---
8062689	-0,51	-1,43	0,22171	-0,87	-1,82	0,00211	---	SLC39A12
7926486	-0,51	-1,43	0,00902	-0,42	-1,34	0,01486	NM_001145195	LRRC39
7917996	-0,51	-1,43	0,01638	-0,36	-1,28	0,19341	NM_144620	MYLK4
8123584	-0,52	-1,43	0,01632	-0,29	-1,22	0,05530	NM_001012418	NEK5
7971757	-0,52	-1,43	0,28261	-0,79	-1,73	0,00895	NM_199289	---
8131412	-0,52	-1,43	0,32511	-0,39	-1,31	0,10604	---	KIAA1244
8122279	-0,52	-1,43	0,00213	0,72	1,65	0,04828	NM_020340	OR7G3
8033738	-0,52	-1,43	0,05656	-1,51	-2,84	0,04868	NM_001001958	ZFYVE27
7929692	-0,52	-1,43	0,33300	0,86	1,81	0,04642	NM_144588	MIR100
7952315	-0,52	-1,43	0,03885	-0,84	-1,79	0,00467	NR_029515	BEST2
8026035	-0,52	-1,43	0,05083	-0,46	-1,37	0,10566	NM_017682	FUT9
8121152	-0,52	-1,43	0,12597	-0,68	-1,60	0,00585	NM_006581	TRIM6-TRIM34
7938012	-0,52	-1,43	0,21169	0,13	1,10	0,54192	NM_001003819	DKFZp779M0652
7939587	-0,52	-1,43	0,37348	-2,26	-4,78	0,01971	BX640618	PRRT3
8085255	-0,52	-1,43	0,08656	-1,32	-2,50	0,00003	NM_207351	C1orf168
7916506	-0,52	-1,43	0,14703	-1,83	-3,56	0,00068	NM_001004303	ZNF689
8000924	-0,52	-1,43	0,37982	0,22	1,16	0,67136	NM_138447	LOC643008
8009929	-0,52	-1,43	0,41966	-0,09	-1,06	0,87417	NR_028439	EHMT2
8125172	-0,52	-1,43	0,01795	0,34	1,26	0,54579	NM_006709	EHMT2
8179884	-0,52	-1,43	0,01795	0,34	1,26	0,54579	NM_006709	SLC47A2
8013364	-0,52	-1,43	0,01236	-1,39	-2,62	0,00019	NM_152908	PNPLA2
7937485	-0,52	-1,43	0,02793	1,18	2,27	0,03758	NM_020376	---
7953761	-0,52	-1,43	0,09751	-1,44	-2,71	0,02087	---	CTF1
7994961	-0,52	-1,43	0,23247	-0,11	-1,08	0,73099	NM_001330	---
8027332	-0,52	-1,43	0,00140	-0,60	-1,52	0,06182	---	LOC729595
8110520	-0,52	-1,43	0,17741	-1,10	-2,15	0,02795	ENST00000451823	---
8100338	-0,52	-1,43	0,16345	0,60	1,52	0,07149	---	ZNF8
8031893	-0,52	-1,43	0,08196	0,80	1,75	0,03724	NM_021089	---
7956892	-0,52	-1,43	0,10893	-0,60	-1,52	0,11371	---	ACSS1
8065444	-0,52	-1,43	0,23293	-0,53	-1,44	0,19376	NM_032501	NPEPL1
8063650	-0,52	-1,43	0,39687	0,66	1,58	0,26673	NM_024663	---
8176815	-0,52	-1,43	0,26169	-0,65	-1,57	0,20665	---	---
8176941	-0,52	-1,43	0,26169	-0,65	-1,57	0,20665	---	---
8177393	-0,52	-1,43	0,26169	-0,65	-1,57	0,20665	---	PHKA2
8171587	-0,52	-1,43	0,03892	1,88	3,69	0,00044	NM_000292	---
8084126	-0,52	-1,43	0,05548	-0,83	-1,78	0,06060	---	---
8168841	-0,52	-1,43	0,09445	-0,74	-1,67	0,00185	---	CARD16
7951408	-0,52	-1,43	0,39965	-2,27	-4,81	0,00055	NM_052889	C12orf69
7961503	-0,52	-1,43	0,32023	-0,76	-1,70	0,00813	NM_001013698	LOC440957
8080416	-0,52	-1,43	0,07410	-0,06	-1,04	0,80792	NM_001124767	SDCCAG1
7974245	-0,52	-1,43	0,44059	-0,97	-1,95	0,02312	NM_004713	TRIM3
7946180	-0,52	-1,43	0,05969	-0,32	-1,25	0,18551	NM_006458	GPR110
8126820	-0,52	-1,43	0,17967	-0,43	-1,35	0,02924	NM_153840	TMPRSS11F
8100688	-0,52	-1,43	0,14984	-1,00	-2,01	0,00887	NM_207407	---
8042466	-0,52	-1,43	0,00974	-1,39	-2,61	0,03743	---	C12orf76
7966293	-0,52	-1,43	0,36305	-0,67	-1,59	0,10269	BC133010	FANCA
8003503	-0,52	-1,43	0,06421	1,03	2,05	0,01619	NM_000135	TAS2R46
7961293	-0,52	-1,43	0,06386	-1,43	-2,70	0,00039	NM_176887	PRR5L
7939383	-0,52	-1,43	0,19497	-0,59	-1,51	0,17676	NM_024841	---

7970511	-0,52	-1,43	0,12787	-0,65	-1,57	0,00595	---	GBGT1	
8164833	-0,52	-1,43	0,21364	-0,61	-1,52	0,15693	NM_021996		SPRR2E
7920214	-0,52	-1,43	0,24999	-1,70	-3,25	0,00132	NM_001024209		CYP4Z2P
7915896	-0,52	-1,43	0,54725	0,46	1,37	0,23218	NR_002788		MYADML
8051422	-0,52	-1,43	0,29325	-1,91	-3,77	0,00072	NR_003143		---
8008049	-0,52	-1,43	0,39036	-1,35	-2,55	0,01072	---	ACTA2	
7929026	-0,52	-1,43	0,07732	-0,55	-1,47	0,03378	NM_001141945		---
8161737	-0,52	-1,43	0,04441	-0,37	-1,29	0,25747	---	KCNIP1	
8109912	-0,52	-1,43	0,16976	-0,72	-1,65	0,08306	NM_001034837		ACSL6
8113938	-0,52	-1,43	0,39296	-1,17	-2,25	0,01612	NM_015256		ZNF789
8134613	-0,52	-1,43	0,13932	-0,06	-1,04	0,76607	NM_213603		CACNA1C
7953094	-0,52	-1,43	0,38348	-1,23	-2,34	0,00057	NM_199460		ECSIT
8034286	-0,52	-1,43	0,29451	-0,39	-1,31	0,21988	NM_016581		OR10Z1
7906372	-0,52	-1,43	0,13940	-2,02	-4,05	0,01141	NM_001004478		TP53TG5
8066521	-0,52	-1,43	0,11198	-1,07	-2,10	0,07985	NM_014477		---
8008130	-0,52	-1,43	0,01203	-1,15	-2,22	0,02295	---	CORIN	
8100154	-0,52	-1,43	0,26012	-0,53	-1,45	0,05420	NM_006587		---
8103693	-0,52	-1,43	0,03676	-0,20	-1,15	0,28123	---	SNORD27	
7948906	-0,52	-1,43	0,01589	-0,34	-1,27	0,22386	NR_002563		NOVA2
8037737	-0,52	-1,43	0,11275	-0,77	-1,71	0,08132	NM_002516		PP2672
7925170	-0,52	-1,43	0,12266	-1,30	-2,46	0,00744	AF193050	GRAMD2	
7990138	-0,52	-1,43	0,19731	0,70	1,63	0,00702	NM_001012642		HCG8
8124691	-0,52	-1,43	0,50527	-1,54	-2,90	0,00074	AY358246	SLC12A3	
7995868	-0,52	-1,43	0,22398	-1,80	-3,49	0,00894	NM_000339		SNORD115-12
7982016	-0,52	-1,43	0,59474	-2,31	-4,97	0,00093	NR_003304		SNORD115-12
7982024	-0,52	-1,43	0,59474	-2,31	-4,97	0,00093	NR_003304		SNORD115-12
7982030	-0,52	-1,43	0,59474	-2,31	-4,97	0,00093	NR_003304		---
7939875	-0,52	-1,43	0,01809	-0,79	-1,73	0,00019	---	C10orf90	
7936996	-0,52	-1,43	0,17120	-0,76	-1,69	0,02714	NM_001004298		---
7994339	-0,52	-1,43	0,09497	-1,19	-2,28	0,00192	---	---	
8107518	-0,52	-1,43	0,09191	-0,42	-1,33	0,11834	---	---	
7991770	-0,52	-1,44	0,02809	-0,59	-1,50	0,01050	---	IFRD2	
8087576	-0,52	-1,44	0,05935	0,75	1,68	0,07926	NM_006764		---
8055305	-0,52	-1,44	0,05096	-0,45	-1,36	0,02203	---	---	
7928746	-0,52	-1,44	0,10642	-0,02	-1,01	0,95695	---	---	
8020464	-0,52	-1,44	0,14514	-1,41	-2,66	0,00002	---	OR2J3	
8117714	-0,52	-1,44	0,49623	-1,11	-2,16	0,04335	NM_001005216		---
7915202	-0,52	-1,44	0,09033	-1,20	-2,30	0,05267	---	OCM2	
8141173	-0,52	-1,44	0,22555	-0,76	-1,69	0,05306	NM_006188		ACOT1
7975598	-0,52	-1,44	0,16655	0,63	1,55	0,19043	NM_001037161		CYP2D6
8076424	-0,52	-1,44	0,59174	-0,99	-1,99	0,25750	NM_000106		ACOT6
7975613	-0,52	-1,44	0,08626	-0,56	-1,48	0,10680	NM_001037162		---
8147715	-0,52	-1,44	0,01061	-1,01	-2,02	0,01712	---	C6orf191	
8129476	-0,52	-1,44	0,18303	-1,33	-2,52	0,03394	NM_001010876		KRT77
7963502	-0,52	-1,44	0,07121	-0,44	-1,36	0,47593	NM_175078		SLFN13
8014248	-0,52	-1,44	0,10971	2,35	5,10	0,00005	NM_144682		MYPN
7927827	-0,52	-1,44	0,09908	-0,92	-1,90	0,02391	NM_032578		LOC728477
7986001	-0,52	-1,44	0,53007	-0,35	-1,27	0,40202	ENST00000338706		---
8062404	-0,52	-1,44	0,22779	-1,30	-2,46	0,01858	---	RGS8	
7922727	-0,52	-1,44	0,14259	-0,87	-1,82	0,03888	NM_001102450		GPR128
8081298	-0,52	-1,44	0,25200	-0,92	-1,90	0,00530	NM_032787		TRIM47
8018494	-0,52	-1,44	0,15520	0,06	1,05	0,90067	NM_033452		ANKDD1A
7984227	-0,52	-1,44	0,23449	-0,43	-1,35	0,03728	NM_182703		DRD3
8089679	-0,52	-1,44	0,09640	-1,39	-2,62	0,02223	NM_000796		---
7933632	-0,52	-1,44	0,06447	-0,56	-1,47	0,02230	---	RNF165	
8021120	-0,52	-1,44	0,30017	-0,72	-1,65	0,13303	NM_152470		C6orf108
8126524	-0,52	-1,44	0,42039	4,43	21,62	0,00109	NM_199184		OTP
8112763	-0,52	-1,44	0,23390	-1,59	-3,00	0,00475	NM_032109		COL11A1
7918064	-0,52	-1,44	0,13187	-1,31	-2,48	0,00116	NM_001854		SCN1A
8056457	-0,52	-1,44	0,06200	-0,75	-1,69	0,00011	NM_001165963		MIR331
7957631	-0,52	-1,44	0,23716	-1,68	-3,21	0,00025	NR_029895		---
8167150	-0,52	-1,44	0,05697	-1,03	-2,05	0,02543	---	SH3BP2	
8093624	-0,52	-1,44	0,14748	0,72	1,64	0,14535	NM_001145856		TUT1
7948685	-0,52	-1,44	0,00262	0,41	1,33	0,24936	NM_022830		KRTAP6-2
8069848	-0,52	-1,44	0,17267	-1,64	-3,11	0,00977	NM_181604		NOL4
8022856	-0,52	-1,44	0,11698	-0,66	-1,58	0,13253	NM_003787		---
7904923	-0,52	-1,44	0,21193	-1,20	-2,31	0,01805	---	TTC31	
8042874	-0,52	-1,44	0,04548	0,50	1,41	0,37841	NM_022492		LOC84856
7927099	-0,52	-1,44	0,47710	-1,75	-3,37	0,00906	NR_026827		---
8139881	-0,52	-1,44	0,17981	-0,46	-1,37	0,33681	---	STAB2	
7958056	-0,52	-1,44	0,06250	-1,21	-2,32	0,02682	NM_017564		KRTAP10-6
8070786	-0,52	-1,44	0,35562	-1,45	-2,72	0,03400	NM_198688		MGC44328
8061167	-0,52	-1,44	0,31846	-0,51	-1,43	0,06053	BC033985	HOOK1	

7901765	-0,52	-1,44	0,00114	2,30	4,92	0,01340	NM_015888	RGSL1
7907986	-0,52	-1,44	0,06055	-0,78	-1,71	0,03536	NM_001137669	LOC151009 // LOC151009
8044346	-0,52	-1,44	0,37898	2,86	7,24	0,00018	NR_027244 // NR_027244	GOLGA9P
7987114	-0,52	-1,44	0,23437	-0,66	-1,58	0,06909	NR_024074	IGSF21
7898521	-0,52	-1,44	0,19306	-0,41	-1,33	0,08532	NM_032880	SNORA65
8164215	-0,52	-1,44	0,21830	-0,73	-1,66	0,01229	NR_002449	---
8107232	-0,52	-1,44	0,36444	-1,83	-3,56	0,01377	---	REP15
7954589	-0,52	-1,44	0,47726	-1,12	-2,17	0,00100	NM_001029874	C12orf72
7954701	-0,52	-1,44	0,09920	-0,56	-1,47	0,26053	NM_173802	LILRP2
8031253	-0,53	-1,44	0,32383	-1,25	-2,39	0,06986	NR_003061	SERPINA10
7981051	-0,53	-1,44	0,15205	-1,53	-2,89	0,00875	NM_016186	SPIN3
8173181	-0,53	-1,44	0,27212	1,22	2,32	0,00760	NR_027139	CACNA2D4
7960283	-0,53	-1,44	0,16408	-1,15	-2,22	0,00590	NM_172364	IFNA17
8160394	-0,53	-1,44	0,22039	-1,59	-3,01	0,00166	NM_021268	C16orf81
7997875	-0,53	-1,44	0,28065	-0,90	-1,86	0,00283	NR_024347	KIAA1486
8048763	-0,53	-1,44	0,08482	-0,34	-1,26	0,07986	NM_020864	B3GALT6
7896878	-0,53	-1,44	0,13210	-0,28	-1,21	0,56654	NM_080605	TMEM139
8136863	-0,53	-1,44	0,34375	-0,80	-1,74	0,23227	NM_153345	RIMBP3
8071532	-0,53	-1,44	0,09748	-0,16	-1,12	0,35748	NM_015672	NCRNA00204
8170963	-0,53	-1,44	0,09004	-0,34	-1,27	0,15706	AY168775	GPC5
7969596	-0,53	-1,44	0,02702	-0,59	-1,51	0,01023	NM_004466	---
8078153	-0,53	-1,44	0,36368	-0,50	-1,41	0,34370	---	---
7954098	-0,53	-1,44	0,03688	-0,73	-1,66	0,06812	---	FLJ39632
8074168	-0,53	-1,44	0,36511	-1,52	-2,87	0,02179	ENST00000400192	CDSN
8124862	-0,53	-1,44	0,58011	-1,61	-3,05	0,00984	NM_001264	TXK
8100210	-0,53	-1,44	0,10954	-0,39	-1,31	0,30433	NM_003328	---
8085052	-0,53	-1,44	0,04761	-1,10	-2,15	0,11621	---	SORBS2
8104066	-0,53	-1,44	0,22350	-1,65	-3,14	0,00717	AF090937	---
7943373	-0,53	-1,44	0,50590	-1,97	-3,92	0,01813	---	LEF1
8102232	-0,53	-1,44	0,32876	-0,10	-1,07	0,80859	NM_016269	---
7928316	-0,53	-1,44	0,16324	-0,80	-1,74	0,03291	---	NRM
8178399	-0,53	-1,44	0,15152	2,28	4,86	0,04395	NM_007243	NRM
8179683	-0,53	-1,44	0,15152	2,28	4,86	0,04395	NM_007243	NT5C3
8138941	-0,53	-1,44	0,20610	-0,60	-1,51	0,30058	NR_029372	WFDC1
7997582	-0,53	-1,44	0,28316	-1,71	-3,27	0,00019	NM_021197	RBP4
7935116	-0,53	-1,44	0,28165	0,50	1,42	0,03139	NM_006744	KRT18
8154725	-0,53	-1,44	0,75869	2,74	6,69	0,03020	NM_000224	CLP1
7940046	-0,53	-1,44	0,31645	0,87	1,82	0,00503	NM_006831	---
7928750	-0,53	-1,44	0,13983	-1,51	-2,86	0,02841	---	IL1RAPL2
8169094	-0,53	-1,44	0,02036	-1,27	-2,41	0,00003	NM_017416	GRIN2B
7961422	-0,53	-1,44	0,07215	-0,99	-1,99	0,02325	NM_000834	IQCF2
8080138	-0,53	-1,44	0,00546	-1,12	-2,17	0,04702	NM_203424	HRASLS
8084838	-0,53	-1,44	0,01440	-0,43	-1,35	0,39389	NM_020386	OVOS
7961026	-0,53	-1,44	0,16881	1,59	3,02	0,00085	BX647938	OLFM3
7918052	-0,53	-1,44	0,25768	-0,78	-1,71	0,00180	NM_058170	FRMPD4
8166028	-0,53	-1,44	0,05869	-0,92	-1,89	0,09333	NM_014728	SLC22A17
7977965	-0,53	-1,44	0,19398	-0,06	-1,04	0,92844	NM_016609	AMICA1
7952022	-0,53	-1,44	0,11355	-0,52	-1,43	0,01624	NM_001098526	NLRP1
8011884	-0,53	-1,44	0,16863	-0,50	-1,41	0,00856	NM_033004	C10orf108
7925813	-0,53	-1,44	0,06547	-1,38	-2,60	0,03304	NR_027152	RBM42
8027908	-0,53	-1,44	0,02723	0,58	1,50	0,05929	NM_024321	---
8015737	-0,53	-1,44	0,46860	-3,27	-9,66	0,02017	---	C17orf62
8019507	-0,53	-1,44	0,06044	1,19	2,28	0,06572	NM_001100407	GNA15
8024572	-0,53	-1,44	0,23979	0,00	1,00	0,99990	NM_002068	---
8076239	-0,53	-1,44	0,08512	-1,14	-2,20	0,00726	---	DNASE1L2
7992584	-0,53	-1,44	0,34299	-0,64	-1,55	0,01983	NM_001374	SPTBN4
8028806	-0,53	-1,44	0,10813	-0,87	-1,83	0,03975	NM_020971	PRDM15
8070503	-0,53	-1,44	0,00456	0,62	1,54	0,06067	NM_001040424	OR2AG1
7938170	-0,53	-1,44	0,32429	-1,42	-2,68	0,05057	NM_001004489	KCNIP2
7935930	-0,53	-1,44	0,35087	-0,93	-1,91	0,00152	NM_014591	C20orf197
8063785	-0,53	-1,44	0,09040	-0,58	-1,50	0,01479	AY358539	---
8091556	-0,53	-1,44	0,18093	-0,85	-1,80	0,00670	---	KIAA1656
8075363	-0,53	-1,44	0,09768	-1,23	-2,35	0,03702	CR456347	---
7979031	-0,53	-1,44	0,08187	-0,85	-1,80	0,06710	---	---
7928363	-0,53	-1,44	0,01712	-1,22	-2,32	0,04263	---	---
8120275	-0,53	-1,44	0,00802	-0,21	-1,16	0,20498	---	NDST3
8097038	-0,53	-1,44	0,02700	-0,98	-1,98	0,00707	NM_004784	---
8138485	-0,53	-1,44	0,20030	-0,44	-1,35	0,14261	---	LRIT2
7934779	-0,53	-1,44	0,37757	-0,85	-1,81	0,01151	NM_001017924	---
7970804	-0,53	-1,44	0,19344	-0,99	-1,98	0,00656	---	CYB561D2
8079993	-0,53	-1,44	0,06809	-0,30	-1,24	0,49069	NM_007022	LYZ
7957023	-0,53	-1,44	0,10537	-0,59	-1,50	0,02520	NM_000239	---

8173928	-0,53	-1,44	0,52811	-1,90	-3,73	0,08502	---	C19orf23
8032208	-0,53	-1,44	0,12367	-0,23	-1,17	0,34742	NR_027271	---
8055087	-0,53	-1,44	0,19987	-0,58	-1,50	0,01031	---	NLRP6
7937305	-0,53	-1,44	0,12252	-0,52	-1,43	0,16553	NM_138329	---
7979730	-0,53	-1,44	0,13013	-1,03	-2,04	0,00023	---	CXorf1
8170319	-0,53	-1,44	0,10330	-1,18	-2,27	0,03594	NM_004709	ZNF32
7933186	-0,53	-1,44	0,30989	0,78	1,71	0,04334	NM_006973	PLA2G4E
7987780	-0,53	-1,44	0,11391	-0,70	-1,62	0,04122	NM_001080490	---
7956419	-0,53	-1,44	0,03403	-1,29	-2,44	0,00937	---	---
8024001	-0,53	-1,44	0,31836	0,31	1,24	0,44332	---	---
7970509	-0,53	-1,45	0,14643	-1,49	-2,80	0,06688	---	DNAL4
8076161	-0,53	-1,45	0,37214	-0,65	-1,57	0,22813	NM_005740	KCNH4
8015554	-0,53	-1,45	0,20969	-0,93	-1,91	0,17086	NM_012285	EYS
8127393	-0,53	-1,45	0,12113	-1,24	-2,37	0,02779	NM_001142800	SNED1
8049752	-0,53	-1,45	0,08275	-0,31	-1,24	0,11112	NM_001080437	GJB6
7970448	-0,53	-1,45	0,25830	-0,89	-1,86	0,05498	NM_001110219	NKAIN4
8067602	-0,53	-1,45	0,11022	-0,82	-1,77	0,00972	NM_152864	---
8047441	-0,53	-1,45	0,69811	-2,12	-4,36	0,15675	---	GNLY
8043236	-0,53	-1,45	0,09800	-0,67	-1,60	0,00629	NM_012483	SCRN2
8016371	-0,53	-1,45	0,32664	0,76	1,70	0,02618	NM_001145023	CLYBL
7969815	-0,53	-1,45	0,14045	1,57	2,97	0,00259	NM_206808	ATP8B3
8032312	-0,53	-1,45	0,07342	1,03	2,04	0,05437	NM_138813	EFR3B
8040587	-0,53	-1,45	0,18083	0,16	1,12	0,21330	NM_014971	UMPS
8082229	-0,53	-1,45	0,21268	1,70	3,25	0,00049	NM_000373	CDKN2C
7901363	-0,53	-1,45	0,05497	-0,30	-1,23	0,33461	NM_001262	MEP1B
8020827	-0,53	-1,45	0,04661	-0,30	-1,23	0,02460	NM_005925	---
8015037	-0,53	-1,45	0,15837	0,13	1,09	0,84244	---	RPL22
7911989	-0,53	-1,45	0,24327	-0,79	-1,73	0,05125	NM_000983	SNORD47
7922402	-0,53	-1,45	0,29069	0,89	1,85	0,13467	NR_002746	---
7985266	-0,53	-1,45	0,17582	-0,82	-1,76	0,12832	---	BCORL2
8177229	-0,53	-1,45	0,14325	-1,30	-2,45	0,13367	NM_001173413	ARID3B
7984843	-0,53	-1,45	0,14909	-0,50	-1,41	0,29598	NM_006465	KDM2B
7967175	-0,53	-1,45	0,07897	2,22	4,65	0,00179	NM_032590	RCHY1
8095758	-0,53	-1,45	0,24293	-0,22	-1,16	0,03804	NM_015436	FLJ34503
8121569	-0,53	-1,45	0,19450	-1,07	-2,10	0,00373	AK091822	GPR12
7970691	-0,53	-1,45	0,22408	-0,91	-1,88	0,01500	NM_005288	CSMD1
8148992	-0,53	-1,45	0,01639	-1,05	-2,07	0,01264	NM_033225	---
7919594	-0,53	-1,45	0,17762	-0,77	-1,71	0,03165	---	PTPMT1
7939818	-0,53	-1,45	0,32838	1,72	3,29	0,03436	NM_175732	SYT2
7987010	-0,53	-1,45	0,38516	-1,67	-3,18	0,03823	NM_177402	MOG
8117748	-0,53	-1,45	0,07997	-1,40	-2,64	0,00382	NM_206814	CBS
8070632	-0,53	-1,45	0,05166	2,36	5,13	0,04936	NM_001178008	EPHA6
8081138	-0,53	-1,45	0,00302	-0,96	-1,95	0,00938	NM_001080448	DSCR9
8068543	-0,53	-1,45	0,08858	-0,56	-1,47	0,11773	AB066100	IRF8
7997712	-0,53	-1,45	0,11269	0,33	1,26	0,14551	NM_002163	F11
8098671	-0,53	-1,45	0,38045	-0,66	-1,58	0,02278	NM_000128	---
7983500	-0,53	-1,45	0,31148	-0,75	-1,68	0,00084	---	---
7976057	-0,53	-1,45	0,02762	-0,75	-1,68	0,01043	---	---
8055482	-0,53	-1,45	0,09695	-0,91	-1,88	0,02127	---	CRLF2
8171105	-0,53	-1,45	0,05806	-1,06	-2,08	0,08693	NM_022148	---
8131301	-0,53	-1,45	0,03779	-1,78	-3,44	0,00148	---	FAT2
8115302	-0,53	-1,45	0,11299	-0,79	-1,72	0,00517	NM_001447	GAFA1
7977592	-0,53	-1,45	0,16908	-1,86	-3,63	0,00066	AF190748	LTBP4
8028872	-0,53	-1,45	0,00777	1,47	2,77	0,08943	NM_001042544	NOS1AP
7906838	-0,53	-1,45	0,16568	-0,96	-1,94	0,00711	NM_014697	CLEC6A
7953737	-0,53	-1,45	0,16658	-1,15	-2,22	0,00043	NM_001007033	ERCC2
8037537	-0,53	-1,45	0,28866	1,99	3,97	0,01654	NM_000400	---
8069446	-0,54	-1,45	0,31350	-1,21	-2,31	0,00127	---	---
8013109	-0,54	-1,45	0,13780	-0,39	-1,31	0,37147	---	LOC646976
7922596	-0,54	-1,45	0,04189	-1,40	-2,64	0,01369	AK096082	ART4
7961507	-0,54	-1,45	0,22851	-0,99	-1,99	0,01207	NM_021071	AVPR2
8170794	-0,54	-1,45	0,13107	-1,13	-2,19	0,12575	NM_001146151	BRF1
7981573	-0,54	-1,45	0,01143	0,41	1,33	0,01123	NM_145685	ZNF799 // ZNF799
8034390	-0,54	-1,45	0,59076	0,09	1,06	0,46838	NM_001080821 // NM_001080821	MAPT
8007828	-0,54	-1,45	0,09967	1,28	2,43	0,05376	NM_016835	C2orf83
8059532	-0,54	-1,45	0,12923	-1,49	-2,81	0,01955	NM_020161	SPINK7
8109049	-0,54	-1,45	0,05282	-1,27	-2,41	0,06140	NM_032566	FETUB
8084657	-0,54	-1,45	0,05871	-0,81	-1,75	0,00736	NM_014375	CD300LF
8018196	-0,54	-1,45	0,21949	-1,52	-2,87	0,01723	NM_139018	---
8151585	-0,54	-1,45	0,08033	-1,29	-2,45	0,00095	---	TRIM31
8179617	-0,54	-1,45	0,23369	-0,86	-1,82	0,13002	NM_007028	RRAS2
7946697	-0,54	-1,45	0,51377	0,05	1,04	0,94781	NM_012250	OR4K14

7977476	-0,54	-1,45	0,34039	-1,71	-3,28	0,00658	NM_001004712	IL12RB1
8035380	-0,54	-1,45	0,32550	-1,34	-2,52	0,00362	NM_005535	TMEM219
7994683	-0,54	-1,45	0,03750	-0,16	-1,12	0,34463	NM_001083613	GDAP1
8146945	-0,54	-1,45	0,32393	0,83	1,78	0,00476	NM_018972	FLJ10357
7973158	-0,54	-1,45	0,02381	-0,68	-1,60	0,01224	NM_018071	---
8175297	-0,54	-1,45	0,06637	-0,57	-1,49	0,05910	---	PRO0611
7899602	-0,54	-1,45	0,22420	-0,62	-1,54	0,00085	NR_002762	FBLL1
8109819	-0,54	-1,45	0,43386	-1,84	-3,57	0,00352	NR_024356	---
7928314	-0,54	-1,45	0,04413	-0,26	-1,19	0,08344	---	SERPINB12
8021594	-0,54	-1,45	0,00409	-0,47	-1,39	0,03613	NM_080474	CSMD2
7914667	-0,54	-1,45	0,00938	-1,18	-2,27	0,00189	NM_052896	C18orf2
8021995	-0,54	-1,45	0,11585	-0,78	-1,72	0,03029	NR_023925	POLG
7991258	-0,54	-1,45	0,24906	-0,19	-1,14	0,52145	NM_002693	CLCA2
7902702	-0,54	-1,45	0,21317	-0,70	-1,62	0,07466	NM_006536	GRPR
8166202	-0,54	-1,45	0,00577	-0,29	-1,22	0,05534	NM_005314	LOC339240
8013473	-0,54	-1,45	0,38649	-1,42	-2,68	0,02936	AK127974	---
7936280	-0,54	-1,45	0,41751	-1,11	-2,16	0,09358	---	C9orf9
8158930	-0,54	-1,45	0,12520	-0,37	-1,30	0,51337	NM_018956	FRMPD2
7933446	-0,54	-1,45	0,35003	-1,81	-3,51	0,02585	NM_001018071	---
8037814	-0,54	-1,45	0,13706	-0,59	-1,51	0,07605	---	---
8143562	-0,54	-1,45	0,11020	-1,24	-2,36	0,01936	---	CCDC87
7949668	-0,54	-1,45	0,22346	-0,22	-1,17	0,45718	NM_018219	SLC15A3
7948493	-0,54	-1,45	0,09353	-0,86	-1,81	0,02634	NM_016582	LOC646813
7939902	-0,54	-1,45	0,27640	0,43	1,35	0,03037	NR_024504	LCE2D
7905500	-0,54	-1,45	0,39897	-1,25	-2,38	0,04657	NM_178430	FAM71B
8115481	-0,54	-1,45	0,01914	-1,45	-2,74	0,00305	NM_130899	---
8000930	-0,54	-1,45	0,20928	-0,74	-1,67	0,01216	---	---
7950895	-0,54	-1,45	0,01933	-1,12	-2,18	0,00218	---	---
7927745	-0,54	-1,45	0,09391	-1,03	-2,04	0,00662	---	---
8154392	-0,54	-1,45	0,23821	-1,45	-2,73	0,00719	---	PSG1
8037240	-0,54	-1,45	0,55063	-0,13	-1,10	0,49487	NM_006905	SCARNA9L
8171758	-0,54	-1,45	0,05275	-1,02	-2,03	0,02626	NR_023358	CDCA5
7949364	-0,54	-1,45	0,28914	0,92	1,89	0,07829	NM_080668	GFER
7992439	-0,54	-1,45	0,04873	0,61	1,53	0,03281	NM_005262	---
8056020	-0,54	-1,45	0,03891	-0,76	-1,69	0,00114	---	OR5D13
7939940	-0,54	-1,45	0,14829	-1,60	-3,02	0,01459	NM_001001967	---
8063522	-0,54	-1,45	0,01361	-0,77	-1,71	0,00391	---	TUBA3D
8045291	-0,54	-1,45	0,33193	-0,87	-1,83	0,03714	NM_080386	FLJ38723
7989476	-0,54	-1,45	0,17147	-0,39	-1,31	0,24382	AK096042	STK33
7946365	-0,54	-1,45	0,00714	1,59	3,01	0,00263	NM_030906	C1orf93
7897119	-0,54	-1,45	0,21597	-0,36	-1,28	0,31406	AF425266	OR2G2
7911207	-0,54	-1,45	0,42654	-1,05	-2,07	0,03920	NM_001001915	LOC100130264
8065248	-0,54	-1,45	0,23888	-1,37	-2,58	0,06963	AK097497	ABCD4
7980115	-0,54	-1,45	0,02868	1,02	2,03	0,00867	NM_005050	PMP2
8151525	-0,54	-1,45	0,01613	-1,32	-2,50	0,01551	NM_002677	TBC1D3G
8014437	-0,54	-1,45	0,42429	1,25	2,38	0,06724	NM_001040282	CEACAM16
8029458	-0,54	-1,45	0,01005	-0,94	-1,92	0,00661	NM_001039213	LRRTM4
8053315	-0,54	-1,45	0,02751	-0,50	-1,41	0,13017	NM_024993	C9orf43
8157308	-0,54	-1,45	0,04536	-0,19	-1,14	0,09973	BC026884	RPS6P6
8101357	-0,54	-1,45	0,29600	-0,60	-1,52	0,01843	BC052613	NSFL1C
8064438	-0,54	-1,45	0,13161	0,96	1,95	0,14445	NM_016143	NFKBIE
8126666	-0,54	-1,45	0,01354	-0,19	-1,14	0,74757	NM_004556	VPREB1
8071646	-0,54	-1,45	0,12076	-1,27	-2,42	0,00002	NM_007128	GPR26
7931199	-0,54	-1,45	0,15300	-0,94	-1,93	0,01291	NM_153442	---
7903115	-0,54	-1,45	0,07078	-1,27	-2,42	0,02816	---	C11orf16
7946428	-0,54	-1,45	0,18156	-1,11	-2,16	0,02766	NM_020643	SNORD79
7922406	-0,54	-1,46	0,10421	-0,50	-1,41	0,04962	NR_003939	TMEM57
7899018	-0,54	-1,46	0,11018	-0,24	-1,18	0,35098	NM_018202	ZNF546
8028770	-0,54	-1,46	0,15390	-0,15	-1,11	0,36106	NM_178544	AMT
8087419	-0,54	-1,46	0,06225	-0,16	-1,12	0,60916	NM_000481	LRRC3
8069122	-0,54	-1,46	0,09994	-0,94	-1,92	0,01827	NM_030891	DEFB131
8094139	-0,54	-1,46	0,00221	-0,50	-1,41	0,09209	NM_001040448	---
7898371	-0,54	-1,46	0,40643	-1,30	-2,46	0,00511	---	---
7912861	-0,54	-1,46	0,40643	-1,30	-2,46	0,00511	---	---
8079999	-0,54	-1,46	0,01416	-0,53	-1,45	0,21545	---	NAA30
7974576	-0,54	-1,46	0,01919	0,80	1,74	0,00414	NM_001011713	MSGN1
8040456	-0,54	-1,46	0,48887	-1,04	-2,05	0,03218	NM_001105569	---
8169965	-0,54	-1,46	0,07926	-0,10	-1,07	0,77757	---	CCDC29
8161415	-0,54	-1,46	0,12883	-0,11	-1,08	0,79474	ENST00000305962	SLC26A4
8135436	-0,54	-1,46	0,24637	-0,93	-1,90	0,09145	NM_000441	POU4F1
7972177	-0,54	-1,46	0,22311	-0,37	-1,29	0,23745	NM_006237	---
7974253	-0,54	-1,46	0,20795	-0,74	-1,67	0,13981	---	C17orf99
8010237	-0,54	-1,46	0,24295	-0,48	-1,39	0,24728	NM_001163075	---

7962199	-0,54	-1,46	0,26820	-0,31	-1,24	0,02867	---	OR6C6
7963865	-0,54	-1,46	0,01153	-1,17	-2,25	0,02640	NM_001005493	---
8139933	-0,54	-1,46	0,02271	-1,26	-2,40	0,01280	---	SNORD63
8114468	-0,54	-1,46	0,00998	-0,62	-1,53	0,00041	NR_002913	NUDT14
7981566	-0,54	-1,46	0,14169	0,76	1,69	0,18778	NM_177533	PCDHB18
8108749	-0,54	-1,46	0,21692	-0,61	-1,52	0,00061	NR_001281	HOXB5
8016457	-0,54	-1,46	0,28181	-0,55	-1,46	0,09452	NM_002147	TTLL6
8016494	-0,54	-1,46	0,22174	-0,78	-1,71	0,00374	NM_173623	---
8156194	-0,54	-1,46	0,30808	-1,57	-2,98	0,00567	---	DOCK2
8109843	-0,54	-1,46	0,07453	-1,41	-2,65	0,00927	NM_004946	METTL2A
8009008	-0,54	-1,46	0,00412	1,49	2,81	0,04694	NM_181725	---
8132501	-0,54	-1,46	0,08607	-1,02	-2,03	0,01219	---	LOC441666
8155634	-0,54	-1,46	0,00019	-0,46	-1,38	0,05584	NR_024380	HOXC8
7955869	-0,54	-1,46	0,18129	1,45	2,74	0,01656	NM_022658	FLT4
8116445	-0,54	-1,46	0,00135	0,66	1,58	0,05543	NM_182925	LONRF2
8054281	-0,54	-1,46	0,31382	0,79	1,72	0,02672	NM_198461	C1orf182
7906154	-0,54	-1,46	0,01937	-0,31	-1,24	0,18937	NM_144627	---
7986767	-0,54	-1,46	0,32822	-1,42	-2,67	0,04692	---	P2RY14
8091511	-0,54	-1,46	0,10887	-0,59	-1,50	0,07214	NM_014879	C10orf107
7927723	-0,54	-1,46	0,14468	-0,77	-1,70	0,01045	NM_173554	WDR72
7988990	-0,54	-1,46	0,14328	-1,13	-2,19	0,00859	NM_182758	IFI27
7976443	-0,54	-1,46	0,30598	-1,42	-2,67	0,00922	NM_001130080	OR6C65
7955995	-0,54	-1,46	0,26802	-0,93	-1,90	0,11466	NM_001005518	KLF8
8167912	-0,54	-1,46	0,02839	0,41	1,33	0,09806	NM_007250	TBXAS1
8136557	-0,54	-1,46	0,00277	-0,43	-1,35	0,00248	NM_001130966	---
7954699	-0,54	-1,46	0,01509	-0,91	-1,88	0,04317	---	---
7981720	-0,54	-1,46	0,73029	-3,63	-12,40	0,00529	---	---
7944525	-0,54	-1,46	0,48699	-1,72	-3,28	0,02396	---	SSX7
8172781	-0,54	-1,46	0,20812	-0,60	-1,51	0,04066	NM_173358	GPR65
7976080	-0,54	-1,46	0,01049	-1,31	-2,48	0,00607	NM_003608	CAMTA1
7897339	-0,54	-1,46	0,42298	1,18	2,27	0,08701	NM_015215	MAP2K2
8032761	-0,54	-1,46	0,46043	0,26	1,19	0,71389	NM_030662	GPSM1
8159318	-0,54	-1,46	0,27150	0,10	1,07	0,84331	NM_001145638	TRIM42
8083053	-0,55	-1,46	0,22102	-0,99	-1,98	0,00347	NM_152616	OR1G1
8011324	-0,55	-1,46	0,25689	-1,64	-3,12	0,04411	NM_003555	---
7926979	-0,55	-1,46	0,56715	-1,23	-2,34	0,01930	---	TCEB3CL
8023152	-0,55	-1,46	0,57135	-2,05	-4,13	0,00840	NM_001100817	KIF24
8160771	-0,55	-1,46	0,06324	0,02	1,01	0,80876	NM_194313	SLC4A2
8137352	-0,55	-1,46	0,00211	0,61	1,52	0,21654	NM_003040	USP17L6P
8094134	-0,55	-1,46	0,81536	-2,95	-7,74	0,00403	NR_027279	NCAPH
8043602	-0,55	-1,46	0,35211	1,20	2,30	0,01021	NM_015341	RTP1
8084726	-0,55	-1,46	0,17417	-0,85	-1,80	0,13733	NM_153708	OR2T5
7911285	-0,55	-1,46	0,04199	-1,03	-2,04	0,13423	NM_001004697	PELI3
7941610	-0,55	-1,46	0,06067	-0,16	-1,12	0,50145	NM_145065	SUN2
8076137	-0,55	-1,46	0,19151	-0,12	-1,08	0,82069	NM_015374	ACSF3
7997863	-0,55	-1,46	0,32302	0,25	1,19	0,56626	NM_174917	DNAH8
8119243	-0,55	-1,46	0,00905	-0,73	-1,65	0,00443	NM_001371	GABRR3
8089060	-0,55	-1,46	0,08695	-0,77	-1,70	0,03647	NM_001105580	C1orf200
7912254	-0,55	-1,46	0,31046	-1,66	-3,15	0,00039	NR_027045	TNIP2
8099029	-0,55	-1,46	0,13954	1,16	2,23	0,12632	NM_024309	---
8173338	-0,55	-1,46	0,27726	-0,93	-1,91	0,03907	---	SCGB1D2
7940630	-0,55	-1,46	0,11196	-1,06	-2,09	0,02421	NM_006551	FAM189A1
7986960	-0,55	-1,46	0,11624	-0,61	-1,53	0,05560	NM_015307	---
8006606	-0,55	-1,46	0,25042	-1,15	-2,21	0,02318	---	MRPL11
7949637	-0,55	-1,46	0,16663	0,84	1,79	0,15088	NM_170739	CYP2D7P1
8076431	-0,55	-1,46	0,19116	-1,63	-3,10	0,04822	NR_002570	TSTA3
8153474	-0,55	-1,46	0,32284	2,34	5,07	0,01521	NM_003313	PAGE4
8167592	-0,55	-1,46	0,11740	-0,72	-1,65	0,15614	NM_007003	CSN1S1
8095412	-0,55	-1,46	0,05412	-1,16	-2,24	0,02311	NM_001890	LOC387646
7932721	-0,55	-1,46	0,17108	1,06	2,09	0,05113	NR_003525	ABCB8
8137310	-0,55	-1,46	0,22755	0,28	1,21	0,43238	NM_007188	E4F1
7992568	-0,55	-1,46	0,10109	0,04	1,03	0,91213	NM_004424	GOLGA8E
7981874	-0,55	-1,46	0,24873	-0,36	-1,28	0,24354	NR_033350	---
7934968	-0,55	-1,46	0,18474	-1,05	-2,07	0,00016	---	LOC619207
7931623	-0,55	-1,46	0,21726	-1,20	-2,29	0,00122	NR_002934	PKD2L2
8108267	-0,55	-1,46	0,09042	-0,71	-1,63	0,06615	NM_014386	---
8101063	-0,55	-1,46	0,33226	-0,91	-1,88	0,06688	---	---
8063026	-0,55	-1,46	0,02763	-1,07	-2,11	0,04241	---	OR2J3
8177694	-0,55	-1,46	0,45695	-1,18	-2,26	0,00764	NM_001005216	OR2J3
8179003	-0,55	-1,46	0,45695	-1,18	-2,26	0,00764	NM_001005216	CXorf66
8175524	-0,55	-1,46	0,06553	-0,60	-1,51	0,06114	NM_001013403	IKZF1
8132819	-0,55	-1,46	0,06127	-1,80	-3,49	0,05955	NM_006060	C9orf125
8162902	-0,55	-1,46	0,01680	-0,60	-1,52	0,02825	NM_032342	BTBD6

7977340	-0,55	-1,46	0,08130	1,60	3,03	0,01389	NM_033271	CACNA2D3
8080578	-0,55	-1,46	0,29442	-0,66	-1,58	0,03289	NM_018398	OC90
8152924	-0,55	-1,46	0,18016	-1,66	-3,16	0,00386	NM_001080399	LOC391742 // LOC391742 // LOC391742 // LOC391742 // LOC391742 // LOC391742 // LOC391742 // LOC391742 // LOC391742 // LOC391742
8104613	-0,55	-1,46	0,49107	-2,04	-4,10	0,00066	ENST00000330110 // ENST00000330110 // ENST00000330110 // ENST00000330110 // ENST00000330110 // ENST00000330110 // ENST00000330110 // ENST00000330110 // ENST00000330110 // ENST00000330110	CCNI2
8107985	-0,55	-1,46	0,20882	-0,31	-1,24	0,69692	NM_001039780	---
8062117	-0,55	-1,46	0,00880	-1,06	-2,08	0,02586	---	ZNF793
8028254	-0,55	-1,46	0,02651	-0,52	-1,43	0,20574	NM_001013659	RPUSD1
7998367	-0,55	-1,46	0,04234	-0,11	-1,08	0,84743	NM_058192	---
8150974	-0,55	-1,46	0,04898	-1,15	-2,22	0,01857	---	OR51F1
7945999	-0,55	-1,46	0,04425	-1,61	-3,05	0,00255	NM_001004752	AFF2
8170364	-0,55	-1,46	0,23804	-1,40	-2,63	0,00024	NM_002025	HNF4G
8146986	-0,55	-1,46	0,26506	-0,65	-1,57	0,14997	NM_004133	KCTD19
8002005	-0,55	-1,46	0,02854	0,36	1,29	0,06841	NM_001100915	IL18RAP
8044049	-0,55	-1,46	0,01895	-0,76	-1,69	0,02065	NM_003853	---
8116829	-0,55	-1,46	0,04882	-1,24	-2,35	0,02598	---	SNORD1C
8010078	-0,55	-1,46	0,00765	-0,18	-1,13	0,40711	NR_004397	TAF7L
8174029	-0,55	-1,46	0,01716	0,50	1,41	0,04323	NM_001168474	MBD3L2
8033352	-0,55	-1,46	0,36416	-1,52	-2,87	0,00003	NM_144614	ATOH7
7933978	-0,55	-1,46	0,03678	-0,81	-1,75	0,04913	NM_145178	ALMS1P
8042761	-0,55	-1,46	0,15410	-0,62	-1,54	0,15745	NR_003683	OBSCN
7910265	-0,55	-1,46	0,04335	-0,12	-1,09	0,58114	NM_001098623	HOXA1
8138708	-0,55	-1,46	0,08187	2,79	6,94	0,00002	NM_005522	CCDC83
7942896	-0,55	-1,46	0,19332	-0,95	-1,93	0,02255	NM_173556	FLJ39632
7977452	-0,55	-1,46	0,34853	-1,48	-2,79	0,02544	ENST00000400192	C6orf134
8117965	-0,55	-1,46	0,08648	1,14	2,21	0,00116	NM_024909	C6orf134
8179149	-0,55	-1,46	0,08648	1,14	2,21	0,00116	NM_024909	ZNF750
8019541	-0,55	-1,46	0,05586	-0,88	-1,84	0,03641	NM_024702	FER1L4
8065920	-0,55	-1,46	0,25819	-0,94	-1,92	0,02157	NR_024377	CCDC24
7900944	-0,55	-1,46	0,34561	-1,01	-2,01	0,07436	NM_152499	---
8124549	-0,55	-1,46	0,04221	-1,15	-2,22	0,06689	---	SNORA31
7971386	-0,55	-1,46	0,06910	-0,80	-1,74	0,10493	NR_002967	TMEM31
8169056	-0,55	-1,46	0,13146	-1,67	-3,19	0,01002	NM_182541	SPINT4
8063019	-0,55	-1,46	0,03174	-0,88	-1,84	0,04053	NM_178455	IL15
8097553	-0,55	-1,46	0,07532	-0,69	-1,61	0,00702	NM_172174	---
8014142	-0,55	-1,46	0,27514	-0,72	-1,65	0,17170	---	ALPP
8049123	-0,55	-1,46	0,12981	-2,49	-5,61	0,00103	NM_001632	RPH3A
7958860	-0,55	-1,46	0,23492	-1,22	-2,33	0,01771	NM_001143854	CCDC97
8029050	-0,55	-1,46	0,04208	1,75	3,37	0,08768	NM_052848	---
8081053	-0,55	-1,46	0,11089	-1,17	-2,25	0,03506	---	LIPJ
7928967	-0,55	-1,46	0,43924	-0,85	-1,81	0,00029	NM_001010939	BPA-1
7902072	-0,55	-1,46	0,04689	-1,28	-2,43	0,04784	AB088847	LOC100289528
8161373	-0,55	-1,46	0,58950	-4,12	-17,43	0,00016	ENST00000438157	LOC100289528
8161554	-0,55	-1,46	0,58950	-4,12	-17,43	0,00016	ENST00000438157	---
7897439	-0,55	-1,46	0,05675	-1,27	-2,42	0,00076	---	---
8051411	-0,55	-1,46	0,30388	-1,70	-3,26	0,00056	---	---
8116479	-0,55	-1,46	0,05331	-1,03	-2,05	0,00017	---	LOC148696
7909418	-0,55	-1,46	0,28586	-1,13	-2,19	0,03637	NR_026817	KIF6
8126184	-0,55	-1,46	0,03097	-1,22	-2,33	0,05301	NM_145027	---
8035579	-0,55	-1,46	0,00123	-0,91	-1,88	0,00487	---	---
8055680	-0,55	-1,46	0,49136	-1,22	-2,33	0,06150	---	ERMAP
7900609	-0,55	-1,46	0,03280	-0,99	-1,99	0,11881	NM_001017922	LSM11
8109585	-0,55	-1,47	0,19103	-0,29	-1,22	0,31300	NM_173491	HTR1A
8112333	-0,55	-1,47	0,06216	-0,91	-1,88	0,01225	NM_000524	ASB18
8059868	-0,55	-1,47	0,27496	-2,05	-4,14	0,04203	NM_212556	---
7976156	-0,55	-1,47	0,12257	-1,42	-2,68	0,10599	---	HSD3B1
7904414	-0,55	-1,47	0,49686	-0,71	-1,64	0,00490	NM_000862	FGL1
8149521	-0,55	-1,47	0,02734	1,15	2,23	0,00606	NM_201553	---
8166997	-0,55	-1,47	0,08856	-0,46	-1,38	0,15827	---	GAS7
8012605	-0,55	-1,47	0,21534	-1,91	-3,76	0,00037	NM_201433	MTBP
8148124	-0,55	-1,47	0,17144	1,46	2,76	0,02607	NM_022045	MTP18
8072346	-0,55	-1,47	0,29841	-0,22	-1,17	0,70569	NM_016498	---
7974052	-0,55	-1,47	0,17104	-0,42	-1,34	0,02197	---	DMKN
8036079	-0,55	-1,47	0,05658	2,64	6,24	0,00025	NM_033317	RNF126
8032076	-0,55	-1,47	0,41093	1,72	3,30	0,03619	NM_194460	DKFZP779L1853
7955110	-0,55	-1,47	0,24239	-0,70	-1,62	0,09159	ENST00000454785	FOXA1
7978706	-0,55	-1,47	0,40059	2,97	7,83	0,00051	NM_004496	IL1RAPL1
8166593	-0,55	-1,47	0,11593	-1,23	-2,34	0,04504	NM_014271	SLC17A8
7957819	-0,55	-1,47	0,07552	0,67	1,59	0,16157	NM_139319	ART1
7937882	-0,55	-1,47	0,04718	-0,75	-1,68	0,01047	NM_004314	MARK2
7940798	-0,55	-1,47	0,10278	2,79	6,90	0,00003	NM_017490	ARHGAP15

8045563	-0,55	-1,47	0,33503	-0,77	-1,70	0,01267	NM_018460	OR9K2
7955981	-0,55	-1,47	0,11134	-1,38	-2,60	0,00187	NM_001005243	LOC153684
8105132	-0,55	-1,47	0,06000	1,22	2,33	0,00120	AK123995	FCRL6
7906475	-0,55	-1,47	0,05177	-0,60	-1,52	0,14288	NM_001004310	---
8144810	-0,55	-1,47	0,37531	-1,23	-2,35	0,01613	---	PIP5K1C
8032650	-0,55	-1,47	0,10569	-0,40	-1,32	0,53910	NM_012398	---
8121330	-0,55	-1,47	0,07994	-1,15	-2,23	0,01227	---	FLJ32063
8047257	-0,55	-1,47	0,19869	-0,96	-1,95	0,00174	NR_026830	NTRK1
7906244	-0,55	-1,47	0,08114	-1,13	-2,19	0,01895	NM_001007792	GBX2
8059864	-0,55	-1,47	0,29588	-0,85	-1,81	0,01790	NM_001485	CCL4L1
8006608	-0,55	-1,47	0,09019	-0,87	-1,82	0,00629	NM_001001435	PRDXDD1P
8042059	-0,55	-1,47	0,21360	0,55	1,47	0,00587	NR_027258	---
8040417	-0,55	-1,47	0,21136	-0,70	-1,63	0,03094	---	MFSD7
8098769	-0,55	-1,47	0,13263	-0,91	-1,87	0,10775	NM_032219	---
7974811	-0,55	-1,47	0,20007	-0,26	-1,19	0,06577	---	STOML1
7990273	-0,55	-1,47	0,12058	-0,79	-1,73	0,20534	NM_004809	---
8146391	-0,55	-1,47	0,23613	-0,90	-1,86	0,01360	---	CAPNS2
7995712	-0,55	-1,47	0,06139	-1,31	-2,47	0,00186	NM_032330	PRAMEF10 // PRAMEF10 // PRAMEF10
7912561	-0,55	-1,47	0,25438	-1,58	-2,99	0,00207	NM_001039361 // NM_001039361 // NM_001039361	KCNK18
7930833	-0,55	-1,47	0,17487	-1,42	-2,67	0,02522	NM_181840	C2orf27A
8045323	-0,55	-1,47	0,29979	0,00	1,00	0,99417	NM_013310	OR1E2
8011341	-0,55	-1,47	0,04695	-1,02	-2,03	0,00092	NM_003554	ANGPTL4
8025402	-0,55	-1,47	0,42749	-0,14	-1,10	0,46047	NM_139314	---
8011328	-0,55	-1,47	0,42714	-2,64	-6,22	0,00369	---	RNF113A
8174731	-0,55	-1,47	0,09887	-1,44	-2,71	0,00179	NM_006978	CAPN11
8119951	-0,55	-1,47	0,04159	-0,46	-1,38	0,02262	NM_007058	SNORD13P1
8076415	-0,55	-1,47	0,42352	-1,08	-2,11	0,00403	X58061	CXCR6
8079377	-0,55	-1,47	0,21841	-1,40	-2,63	0,02643	NM_006564	KIAA0664
8011293	-0,55	-1,47	0,07915	2,08	4,23	0,01770	NM_015229	MIR29B1
8142977	-0,56	-1,47	0,05243	-0,76	-1,69	0,02264	NR_029517	ATAD3B
7896961	-0,56	-1,47	0,13451	0,28	1,22	0,42265	NM_031921	MOBKL2B
8160521	-0,56	-1,47	0,22177	0,21	1,16	0,68301	NM_024761	CCDC13
8086434	-0,56	-1,47	0,24060	-1,07	-2,10	0,00233	NM_144719	OR4F6
7986530	-0,56	-1,47	0,03834	-1,83	-3,56	0,00464	NM_001005326	---
8116994	-0,56	-1,47	0,42186	-1,13	-2,20	0,02174	---	LCNL1
8159531	-0,56	-1,47	0,21659	-1,87	-3,67	0,00660	NM_207510	LOC100132099
7969794	-0,56	-1,47	0,40523	-1,06	-2,09	0,11525	AY358798	LOC652276
7992678	-0,56	-1,47	0,44857	-0,87	-1,82	0,16425	NR_015441	---
8018095	-0,56	-1,47	0,11690	-0,90	-1,86	0,01624	---	RPS10P7
7908692	-0,56	-1,47	0,12074	-0,24	-1,18	0,68182	NR_026667	PHLDB3
8037309	-0,56	-1,47	0,31433	-1,07	-2,10	0,02131	NM_198850	TDRD12
8027482	-0,56	-1,47	0,05770	-0,18	-1,13	0,18632	NM_001110822	---
8013448	-0,56	-1,47	0,00966	-0,71	-1,64	0,01085	---	THPO
8092501	-0,56	-1,47	0,18160	-1,43	-2,69	0,00142	NM_000460	DHRS12
7971713	-0,56	-1,47	0,16660	0,10	1,07	0,45558	NM_024705	---
7969360	-0,56	-1,47	0,13423	-1,62	-3,08	0,00070	---	LOC100291056
7981737	-0,56	-1,47	0,66401	-2,73	-6,62	0,03572	ENST00000390631	C16orf7
8003484	-0,56	-1,47	0,02261	-0,26	-1,20	0,29080	NM_004913	SNORA71A
8066258	-0,56	-1,47	0,05712	-1,97	-3,92	0,00058	NR_002911	SNORD8
7977732	-0,56	-1,47	0,00254	-0,70	-1,63	0,01532	NR_002916	---
8115836	-0,56	-1,47	0,02059	-1,20	-2,31	0,02852	---	ARPP21
8078497	-0,56	-1,47	0,03793	-0,63	-1,55	0,09040	NM_016300	ANKMY1
8060103	-0,56	-1,47	0,20399	-0,07	-1,05	0,76570	NM_016552	PRRT2
7994647	-0,56	-1,47	0,34687	-1,66	-3,16	0,00062	NM_145239	LOC349196
8144416	-0,56	-1,47	0,67739	-1,55	-2,92	0,05858	NR_027000	LOC349196
8144418	-0,56	-1,47	0,67739	-1,55	-2,92	0,05858	NR_027000	LOC349196
8144490	-0,56	-1,47	0,67739	-1,55	-2,92	0,05858	NR_027000	LOC349196
8144492	-0,56	-1,47	0,67739	-1,55	-2,92	0,05858	NR_027000	MUM1L1
8169145	-0,56	-1,47	0,19363	-0,35	-1,27	0,13862	NM_152423	CIB3
8035110	-0,56	-1,47	0,04411	-0,90	-1,87	0,16134	NM_054113	CHST1
7947599	-0,56	-1,47	0,12738	-0,48	-1,39	0,11071	NM_003654	HBII-52-24
7982054	-0,56	-1,47	0,15079	-0,67	-1,59	0,04012	NR_003495	---
8065252	-0,56	-1,47	0,22918	-0,84	-1,79	0,00153	---	SAMSN1
8069541	-0,56	-1,47	0,05732	-1,02	-2,03	0,00054	NM_022136	CARKD
7970084	-0,56	-1,47	0,15887	1,13	2,18	0,05459	NM_018210	C18orf34
8022831	-0,56	-1,47	0,08147	-1,14	-2,20	0,00702	NM_001105528	ALKBH2
7966183	-0,56	-1,47	0,01558	1,09	2,13	0,00675	NM_001145374	---
7954690	-0,56	-1,47	0,00225	-0,72	-1,65	0,15651	---	ANGPTL5
7951195	-0,56	-1,47	0,17907	-0,46	-1,38	0,23503	NM_178127	HEY1
8151457	-0,56	-1,47	0,11343	-0,27	-1,20	0,65942	NM_012258	SEMG1
8062933	-0,56	-1,47	0,37963	-1,33	-2,52	0,06489	NM_003007	CYTIP

8055980	-0,56	-1,47	0,15562	-1,01	-2,02	0,07133	NM_004288	MRC1
7926410	-0,56	-1,47	0,07896	-0,75	-1,68	0,00007	NM_002438	MRC1
7926451	-0,56	-1,47	0,07896	-0,75	-1,68	0,00007	NM_002438	---
8016429	-0,56	-1,47	0,38126	-1,41	-2,65	0,15672	---	CPSF4L
8018102	-0,56	-1,47	0,13940	-0,74	-1,67	0,00484	NM_001129885	---
8094163	-0,56	-1,47	0,04748	-0,77	-1,71	0,04852	---	NRBP2
8153550	-0,56	-1,47	0,21354	1,19	2,28	0,05787	NM_178564	---
8117192	-0,56	-1,47	0,01405	-1,33	-2,52	0,03557	---	---
8026989	-0,56	-1,47	0,16810	-1,59	-3,01	0,01669	---	ADAM21
7979904	-0,56	-1,47	0,08623	0,33	1,26	0,48149	NM_003813	SPTBN5
7983051	-0,56	-1,47	0,22409	-1,16	-2,24	0,00623	NM_016642	---
7937957	-0,56	-1,47	0,24564	-1,08	-2,12	0,02909	---	ADAM7
8145334	-0,56	-1,47	0,02898	-0,62	-1,54	0,04340	NM_003817	KRTAP9-8
8007130	-0,56	-1,47	0,48638	-2,03	-4,07	0,00020	NM_031963	FCRL2
7921298	-0,56	-1,47	0,12728	-1,08	-2,11	0,02686	NM_030764	UNC13D
8018449	-0,56	-1,47	0,04100	-0,50	-1,41	0,37946	NM_199242	ZNF423
8001337	-0,56	-1,47	0,03315	-0,88	-1,84	0,00052	NM_015069	---
7973611	-0,56	-1,47	0,15241	-0,71	-1,64	0,04994	---	BCAR3
7917754	-0,56	-1,47	0,06102	0,70	1,63	0,04662	NM_003567	IFT140
7998542	-0,56	-1,47	0,03029	0,10	1,07	0,77378	NM_014714	CXCR5
7944335	-0,56	-1,47	0,01925	-1,43	-2,70	0,06325	NM_001716	SMR3B
8095491	-0,56	-1,47	0,00429	-0,67	-1,59	0,10205	NM_006685	ZNF385A
7963774	-0,56	-1,47	0,02736	-0,21	-1,16	0,34298	NM_015481	NPVF
8138662	-0,56	-1,47	0,10059	-0,29	-1,23	0,27472	NM_022150	WIBG
7963965	-0,56	-1,47	0,09300	0,82	1,76	0,09428	NM_032345	PC
7949719	-0,56	-1,47	0,05048	1,07	2,11	0,06560	NM_001040716	---
7971711	-0,56	-1,47	0,09272	-0,78	-1,72	0,08278	---	TMEM120B
7959312	-0,56	-1,47	0,23598	2,72	6,59	0,00007	NM_001080825	---
8045577	-0,56	-1,47	0,00108	-1,20	-2,29	0,00194	---	GPR81
7967325	-0,56	-1,48	0,21158	-1,66	-3,15	0,00055	NM_032554	C1orf100
7911049	-0,56	-1,48	0,07720	-0,92	-1,89	0,02734	NM_001012970	BCAN
7906205	-0,56	-1,48	0,36798	-1,08	-2,11	0,00646	NM_021948	---
8151492	-0,56	-1,48	0,25916	-1,76	-3,40	0,00158	---	---
8143383	-0,56	-1,48	0,18215	-1,50	-2,83	0,00634	---	---
8094686	-0,56	-1,48	0,21958	-0,70	-1,62	0,03173	---	---
7939952	-0,56	-1,48	0,00170	-0,90	-1,86	0,00517	---	ZNF711
8168589	-0,56	-1,48	0,33527	0,03	1,02	0,92054	NM_021998	GPR172A
8148841	-0,56	-1,48	0,22823	0,43	1,35	0,42617	NM_024531	NEU3
7942562	-0,56	-1,48	0,07113	0,63	1,55	0,15336	NM_006656	tAKR
7931824	-0,56	-1,48	0,00499	-0,53	-1,45	0,14542	NR_026743	CRHBP
8106418	-0,56	-1,48	0,03591	-1,07	-2,10	0,04561	NM_001882	LOC100127950
8132493	-0,56	-1,48	0,25606	-1,22	-2,33	0,00160	AK093262	---
7907462	-0,56	-1,48	0,01834	-0,67	-1,59	0,00879	---	RSPH9
8119882	-0,56	-1,48	0,12905	-0,67	-1,59	0,04170	NM_152732	CCDC39
8092295	-0,56	-1,48	0,03978	-1,01	-2,02	0,05184	NM_181426	BCR
8071691	-0,56	-1,48	0,12083	0,60	1,51	0,25012	NM_004327	OR8A1
7944843	-0,56	-1,48	0,40536	-1,99	-3,99	0,00076	NM_001005194	CCDC42
8012520	-0,56	-1,48	0,08499	-1,37	-2,58	0,00450	NM_144681	KRTDAP
8036072	-0,56	-1,48	0,11180	-1,29	-2,44	0,00258	NM_207392	UNC45A
7986160	-0,56	-1,48	0,11809	0,57	1,49	0,42443	NM_001039675	POLR3GL
7919197	-0,56	-1,48	0,23126	0,53	1,44	0,23077	NM_032305	LDHC
7938788	-0,56	-1,48	0,00467	-1,27	-2,42	0,00170	NM_002301	---
8048715	-0,56	-1,48	0,10748	-0,71	-1,64	0,00122	---	C21orf81
8069511	-0,56	-1,48	0,08198	0,45	1,37	0,01253	NR_027270	GLYAT
7948344	-0,56	-1,48	0,04424	-1,49	-2,81	0,03188	NM_201648	STAP2
8032789	-0,56	-1,48	0,15817	-0,49	-1,40	0,03009	NM_017720	TPH2
7957205	-0,56	-1,48	0,07021	-0,75	-1,68	0,00321	NM_173353	RLBP1
7991246	-0,56	-1,48	0,12222	-1,45	-2,73	0,00811	NM_000326	---
7929986	-0,56	-1,48	0,08169	-1,18	-2,27	0,00538	---	RALYL
8147065	-0,56	-1,48	0,05923	-0,72	-1,64	0,03417	NM_173848	MIR492
7957608	-0,56	-1,48	0,28216	-0,21	-1,16	0,28715	NR_030171	---
8101203	-0,56	-1,48	0,24595	-0,68	-1,60	0,00570	---	HIST1H2AK
8124524	-0,56	-1,48	0,18369	-0,51	-1,42	0,23507	NM_003510	CYorf15B
8176709	-0,56	-1,48	0,05179	-0,09	-1,07	0,74179	NM_032576	---
7918811	-0,56	-1,48	0,23354	-1,27	-2,40	0,04631	---	ZNF366
8112584	-0,56	-1,48	0,10231	-1,22	-2,34	0,01368	NM_152625	TMCO2
7900409	-0,56	-1,48	0,08586	-0,90	-1,86	0,00691	NM_001008740	MBP
8023889	-0,56	-1,48	0,07523	0,28	1,21	0,30214	NM_001025101	---
8124636	-0,56	-1,48	0,06361	-0,81	-1,76	0,01144	---	---
8041638	-0,56	-1,48	0,14434	-0,71	-1,64	0,00352	---	GOLGA6L5
7985562	-0,56	-1,48	0,56037	-0,56	-1,48	0,08160	NR_003246	NLRP3
7911178	-0,56	-1,48	0,12802	-0,88	-1,84	0,00280	NM_004895	GYPE
8102988	-0,56	-1,48	0,06708	-1,21	-2,31	0,01057	NM_198682	---

7930452	-0,56	-1,48	0,25501	-1,09	-2,12	0,00242	---	C1orf120
7907972	-0,56	-1,48	0,09075	-0,94	-1,92	0,02668	BC140780	---
8116996	-0,56	-1,48	0,28846	-0,87	-1,83	0,12833	---	PRAMEL
8074836	-0,56	-1,48	0,00450	-0,64	-1,56	0,08066	AK131325	OSBPL7
8016336	-0,57	-1,48	0,27832	0,36	1,28	0,24158	NM_145798	C6orf47
8124994	-0,57	-1,48	0,03761	0,16	1,12	0,85011	NM_021184	ABCC2
7929779	-0,57	-1,48	0,06986	3,11	8,64	0,00010	NM_000392	PLCH1
8091600	-0,57	-1,48	0,01321	0,53	1,44	0,15341	NM_001130960	---
8102037	-0,57	-1,48	0,02002	-0,48	-1,40	0,01395	---	AADACL2
8083409	-0,57	-1,48	0,03385	-1,08	-2,11	0,00011	NM_207365	PROM1
8099476	-0,57	-1,48	0,03524	-0,82	-1,77	0,00824	NM_006017	---
8015944	-0,57	-1,48	0,33739	-1,88	-3,68	0,00365	---	---
7986627	-0,57	-1,48	0,06072	-1,07	-2,10	0,02091	---	---
7976724	-0,57	-1,48	0,13191	-0,67	-1,59	0,02234	---	TRIM22
7938057	-0,57	-1,48	0,32108	-1,60	-3,03	0,01509	AK308796	C21orf29
8070757	-0,57	-1,48	0,32994	-1,77	-3,41	0,00219	NM_144991	C2orf14
8055214	-0,57	-1,48	0,26205	-1,86	-3,63	0,00956	NR_023391	---
7932067	-0,57	-1,48	0,15523	-1,12	-2,18	0,02499	---	TSPAN11
7954645	-0,57	-1,48	0,14620	-0,58	-1,49	0,07330	NM_001080509	CRELD2
8073949	-0,57	-1,48	0,20541	0,59	1,50	0,12531	NM_001135101	MIR144
8013786	-0,57	-1,48	0,06956	-0,76	-1,70	0,00962	NR_029685	OR4X2
7939867	-0,57	-1,48	0,18217	-1,19	-2,28	0,00098	NM_001004727	HPN
8027728	-0,57	-1,48	0,03438	-1,56	-2,96	0,00196	NM_182983	---
7972237	-0,57	-1,48	0,22878	-0,88	-1,84	0,00025	---	---
7897424	-0,57	-1,48	0,11404	-0,60	-1,52	0,05243	---	FLJ45831
8012893	-0,57	-1,48	0,44019	-1,20	-2,29	0,01148	AK127731	C7orf52
8141669	-0,57	-1,48	0,06048	-0,18	-1,13	0,04944	NM_198571	C17orf108
8013567	-0,57	-1,48	0,57878	-0,10	-1,07	0,84458	NM_001076680	LOC285696
8111203	-0,57	-1,48	0,20061	-0,06	-1,04	0,83369	NR_027253	ANGPTL3
7901883	-0,57	-1,48	0,11219	-0,43	-1,35	0,11583	NM_014495	ZP4
7925419	-0,57	-1,48	0,20467	-1,21	-2,31	0,00426	NM_021186	---
7936902	-0,57	-1,48	0,08238	-0,76	-1,69	0,00007	---	---
8175440	-0,57	-1,48	0,07564	-1,01	-2,02	0,00067	---	---
8163015	-0,57	-1,48	0,36596	-1,15	-2,23	0,08265	---	MIRLET7D
8156523	-0,57	-1,48	0,14689	-1,32	-2,49	0,00243	NR_029481	LOC388152
7985402	-0,57	-1,48	0,00218	-0,03	-1,02	0,96229	NR_027001	ROCK1P1
8019804	-0,57	-1,48	0,56676	-2,35	-5,11	0,01076	BC041849	FLJ42280
8141127	-0,57	-1,48	0,04935	-1,07	-2,10	0,00069	ENST00000356686	---
7955462	-0,57	-1,48	0,15889	-0,86	-1,81	0,01221	---	---
8083775	-0,57	-1,48	0,15642	-1,05	-2,07	0,13935	---	APOBEC3A
8073056	-0,57	-1,48	0,01202	-1,39	-2,62	0,02914	NM_145699	---
8045812	-0,57	-1,48	0,17349	-0,40	-1,32	0,07034	---	FAM18A
7999412	-0,57	-1,48	0,20815	-0,74	-1,67	0,06898	NM_001079512	---
8119490	-0,57	-1,48	0,00978	-0,60	-1,52	0,02532	---	CCDC144NL
8013479	-0,57	-1,48	0,40996	-0,57	-1,49	0,32474	NM_001004306	DGCR14
8074371	-0,57	-1,48	0,21556	-0,13	-1,09	0,76755	NM_022719	---
8005433	-0,57	-1,48	0,32592	-0,53	-1,44	0,18220	---	CHRM5
7982497	-0,57	-1,48	0,11806	-0,25	-1,19	0,45678	NM_012125	ABRA
8152291	-0,57	-1,48	0,42013	-1,54	-2,91	0,00047	NM_139166	LOC440300
7985690	-0,57	-1,48	0,23802	-1,20	-2,30	0,02599	BX648930	OR6A2
7946280	-0,57	-1,48	0,21010	-0,77	-1,71	0,14696	NM_003696	SLC13A2
8005879	-0,57	-1,48	0,33168	-1,08	-2,11	0,03314	NR_027384	---
8152367	-0,57	-1,48	0,18508	-0,96	-1,95	0,01007	---	---
8148692	-0,57	-1,48	0,11381	-0,68	-1,60	0,08380	---	---
7996256	-0,57	-1,48	0,38346	-0,65	-1,56	0,34114	---	C21orf130
8068664	-0,57	-1,48	0,14767	-0,84	-1,79	0,00185	ENST00000433522	KBTBD13
7984253	-0,57	-1,48	0,00411	-1,35	-2,56	0,00691	NM_001101362	---
7931027	-0,57	-1,48	0,16619	-0,40	-1,32	0,06275	---	EREG
8095728	-0,57	-1,48	0,04981	2,07	4,19	0,00452	NM_001432	TDH
8144604	-0,57	-1,48	0,12200	-0,81	-1,75	0,05863	NR_001578	OR9I1
7948310	-0,57	-1,48	0,30606	-1,20	-2,30	0,00304	NM_001005211	PHRF1
7937415	-0,57	-1,48	0,13325	-0,38	-1,30	0,23390	NM_020901	MUCL1
7955963	-0,57	-1,48	0,38386	-1,20	-2,29	0,00034	NM_058173	---
7967698	-0,57	-1,48	0,45716	-1,50	-2,82	0,07269	---	CASQ1
7906552	-0,57	-1,48	0,18751	-0,25	-1,19	0,18013	NM_001231	MREG
8058824	-0,57	-1,48	0,12175	0,05	1,04	0,87848	NM_018000	CYP19A1
7988767	-0,57	-1,48	0,20742	-1,05	-2,06	0,03593	NM_031226	LY6G6E
8125038	-0,57	-1,48	0,21365	-0,26	-1,20	0,70352	NR_003673	LY6G6E
8178582	-0,57	-1,48	0,21365	-0,26	-1,20	0,70352	NR_003673	LY6G6E
8179810	-0,57	-1,48	0,21365	-0,26	-1,20	0,70352	NR_003673	LOC100131763
8049950	-0,57	-1,48	0,27465	-1,36	-2,56	0,00535	AK094921	FLJ42289
7991542	-0,57	-1,48	0,26113	-0,58	-1,49	0,03144	NR_028139	KCNAB1
8083546	-0,57	-1,48	0,00204	-0,77	-1,71	0,05731	NM_003471	FAM131B

8143564	-0,57	-1,48	0,22737	-1,57	-2,98	0,00032	NM_001031690	ABCC8
7946860	-0,57	-1,48	0,00842	-0,62	-1,54	0,01404	NM_000352	---
7934297	-0,57	-1,48	0,03762	-1,27	-2,41	0,01769	---	RASIP1
8038163	-0,57	-1,48	0,10471	-1,22	-2,33	0,00248	NM_017805	SNORD115-42
7982090	-0,57	-1,48	0,45232	-1,77	-3,41	0,00002	NR_003357	TEP1
7977511	-0,57	-1,48	0,20316	0,41	1,33	0,15506	NM_007110	GRASP
7955578	-0,57	-1,48	0,31128	-1,30	-2,45	0,04138	NM_181711	---
8022100	-0,57	-1,48	0,28584	-0,69	-1,61	0,12759	---	USP43
8004905	-0,57	-1,48	0,15288	-0,19	-1,14	0,28200	NM_153210	GOLGA8C
7981752	-0,57	-1,48	0,21885	-0,40	-1,32	0,33186	NR_027411	SERPINB3
8023696	-0,57	-1,48	0,12138	-0,93	-1,90	0,14570	NM_006919	C20orf185
8061815	-0,57	-1,48	0,16667	-1,28	-2,43	0,00631	NM_182658	C2orf78
8042769	-0,57	-1,49	0,02262	-0,74	-1,67	0,19425	NM_001080474	TNNC1
8087925	-0,57	-1,49	0,14771	0,11	1,08	0,62202	NM_003280	SHISA7
8039498	-0,57	-1,49	0,11094	0,35	1,28	0,17199	NM_001145176	LOC728437
7970806	-0,57	-1,49	0,06778	-1,64	-3,12	0,00117	BC027486	---
8113431	-0,57	-1,49	0,04704	-0,22	-1,17	0,02937	---	PLCXD1
8165711	-0,57	-1,49	0,30853	-0,14	-1,10	0,58057	NM_018390	KLHL33
7977567	-0,57	-1,49	0,22233	-1,31	-2,47	0,13934	NM_001109997	---
7925790	-0,57	-1,49	0,05732	-1,02	-2,02	0,00398	---	DNAH12
8088322	-0,57	-1,49	0,02282	-0,78	-1,72	0,09266	NM_198564	TMC2
8060461	-0,57	-1,49	0,27648	-0,85	-1,81	0,03504	NM_080751	PLCXD1
8176286	-0,57	-1,49	0,32250	-0,08	-1,06	0,72492	NM_018390	MUC21
8118069	-0,57	-1,49	0,40312	-1,67	-3,18	0,00001	NM_001010909	PRAMEF2
7897987	-0,57	-1,49	0,25032	-0,48	-1,40	0,08706	NM_023014	---
8173501	-0,57	-1,49	0,05598	-0,77	-1,71	0,00608	---	CD247
7922040	-0,57	-1,49	0,04456	-1,24	-2,36	0,01302	NM_198053	RHOXF1
8174755	-0,57	-1,49	0,05950	-0,82	-1,77	0,00732	NM_139282	SERPINA6
7981059	-0,57	-1,49	0,14010	-0,97	-1,96	0,00569	NM_001756	ERVFRDE1
8123929	-0,57	-1,49	0,19772	-2,22	-4,66	0,00382	NM_207582	---
8103204	-0,57	-1,49	0,14272	-0,31	-1,24	0,20535	---	SNRNP35
7959586	-0,57	-1,49	0,00103	-0,15	-1,11	0,42861	NM_022717	SRXN1
8064382	-0,57	-1,49	0,44445	-1,28	-2,44	0,03095	AK125343	---
8078688	-0,57	-1,49	0,17132	-1,07	-2,10	0,36456	---	MUSK
8157173	-0,57	-1,49	0,17464	-1,46	-2,75	0,00202	NM_005592	HERC2
7986863	-0,57	-1,49	0,12594	2,06	4,17	0,00096	NM_004667	TCEA2
8064245	-0,57	-1,49	0,14293	2,23	4,69	0,01704	NM_198723	IDUA
8093425	-0,57	-1,49	0,06116	0,66	1,58	0,18365	NM_000203	SIGLEC11
8038505	-0,57	-1,49	0,03891	-0,55	-1,46	0,00519	NM_052884	---
8121363	-0,57	-1,49	0,20211	-1,09	-2,12	0,00887	---	---
8029435	-0,57	-1,49	0,18059	-2,07	-4,20	0,00391	---	KCNV2
8154124	-0,57	-1,49	0,25521	-1,53	-2,89	0,00298	NM_133497	HOXB6
8016463	-0,57	-1,49	0,18234	0,80	1,74	0,08085	NM_018952	PBX4
8035694	-0,57	-1,49	0,30340	0,10	1,07	0,60969	NM_025245	TNP1
8058866	-0,57	-1,49	0,01440	-0,91	-1,87	0,01941	NM_003284	RG9MTD3
8155301	-0,57	-1,49	0,10737	-0,22	-1,16	0,52070	NM_144964	WDR17
8098379	-0,57	-1,49	0,03461	1,83	3,55	0,00345	NM_170710	ZNF358
8025179	-0,57	-1,49	0,03465	0,91	1,89	0,00723	NM_018083	FAM198A
8079131	-0,57	-1,49	0,12013	-0,65	-1,56	0,02934	NM_001129908	KRTAP4-12
8015230	-0,57	-1,49	0,72976	-4,05	-16,60	0,00065	NM_031854	---
7945997	-0,57	-1,49	0,11816	-1,31	-2,48	0,08172	---	TRBV23OR9-2
8154841	-0,57	-1,49	0,06168	-0,65	-1,57	0,29372	ENST00000390389	---
8040616	-0,57	-1,49	0,06284	-0,77	-1,70	0,04107	---	---
8086218	-0,57	-1,49	0,16553	-0,59	-1,51	0,00094	---	ITPK1
7980970	-0,57	-1,49	0,37941	1,85	3,60	0,01266	NM_014216	AKR1C4
7925939	-0,57	-1,49	0,08434	-0,74	-1,68	0,17837	NM_001818	CMYA5
8106556	-0,57	-1,49	0,11562	-0,81	-1,75	0,01145	NM_153610	GPR3
7899343	-0,57	-1,49	0,35104	-0,01	-1,00	0,99209	NM_005281	CDKN2A
8160441	-0,57	-1,49	0,20996	-0,04	-1,03	0,95682	NM_058197	NUDT9P1
7934993	-0,57	-1,49	0,27125	-1,03	-2,05	0,01353	NR_002779	FAM169A
8112649	-0,57	-1,49	0,00683	2,90	7,44	0,00197	NM_015566	TBL3
7992414	-0,57	-1,49	0,29298	0,70	1,62	0,05219	NM_006453	FLJ90680
8075846	-0,57	-1,49	0,02528	-1,14	-2,20	0,00721	AK075161	ITGAE
8011430	-0,57	-1,49	0,02799	-0,50	-1,41	0,02064	NM_002208	---
7963348	-0,58	-1,49	0,12883	-1,22	-2,33	0,01783	---	RLTPR
7996608	-0,58	-1,49	0,18484	-0,57	-1,48	0,12879	NM_001013838	---
8138795	-0,58	-1,49	0,00039	-1,20	-2,29	0,03242	---	---
8092275	-0,58	-1,49	0,26102	-1,50	-2,82	0,02429	---	FAM168A
7950271	-0,58	-1,49	0,22268	0,66	1,58	0,01123	EF363480	ELF3
7908793	-0,58	-1,49	0,03176	2,07	4,20	0,00122	NM_001114309	UBTFL1
7943047	-0,58	-1,49	0,29288	-1,33	-2,51	0,00985	NM_001143975	UBTFL1
7950951	-0,58	-1,49	0,29288	-1,33	-2,51	0,00985	NM_001143975	UBTFL1
8043581	-0,58	-1,49	0,29288	-1,33	-2,51	0,00985	NM_001143975	UBTFL1

8053785	-0,58	-1,49	0,29288	-1,33	-2,51	0,00985	NM_001143975	SH2D4B
7928723	-0,58	-1,49	0,23172	-0,84	-1,79	0,05564	NM_207372	KRTAP26-1
8069804	-0,58	-1,49	0,14925	-1,20	-2,30	0,00147	NM_203405	TEX14
8017061	-0,58	-1,49	0,04914	-0,40	-1,32	0,03434	NM_198393	FRMD5
7988260	-0,58	-1,49	0,11609	2,23	4,71	0,00448	NM_032892	IGHV1OR15-5
8067822	-0,58	-1,49	0,24434	-1,26	-2,39	0,02825	ENST00000302092	LOC643923
7943573	-0,58	-1,49	0,00650	-0,15	-1,11	0,68966	ENST00000299326	PATE1
7945033	-0,58	-1,49	0,30083	-0,70	-1,63	0,02540	NM_138294	SEL1L2
8065045	-0,58	-1,49	0,18190	-0,77	-1,70	0,07065	NM_025229	BTBD11
7958352	-0,58	-1,49	0,07011	0,85	1,81	0,02720	NM_001018072	C10orf32
7930170	-0,58	-1,49	0,07798	0,38	1,31	0,13498	NM_001136200	FOSB
8029693	-0,58	-1,49	0,10636	-1,49	-2,80	0,00372	NM_006732	RPL23AP71
7973894	-0,58	-1,49	0,13405	-0,94	-1,92	0,07169	CR595167 SPAG8	
8161133	-0,58	-1,49	0,03760	-1,13	-2,19	0,11597	NM_172312	HIST1H2BI
8117429	-0,58	-1,49	0,05691	-1,09	-2,13	0,07964	NM_003525	RSPO1
7915032	-0,58	-1,49	0,07501	-0,38	-1,30	0,15826	NM_001038633	---
8107162	-0,58	-1,49	0,13221	-0,58	-1,50	0,05968	---	CLEC4C
7960832	-0,58	-1,49	0,05215	-0,50	-1,42	0,07056	NM_130441	---
8144740	-0,58	-1,49	0,17821	-0,79	-1,72	0,03697	---	---
8040413	-0,58	-1,49	0,00392	-0,87	-1,82	0,00066	---	AKNA
8163569	-0,58	-1,49	0,05918	0,78	1,72	0,17919	NM_030767	PDCD7
7989725	-0,58	-1,49	0,01312	1,52	2,88	0,05152	NM_005707	---
7983841	-0,58	-1,49	0,01093	-0,85	-1,80	0,05113	---	---
7966343	-0,58	-1,49	0,11638	-0,80	-1,74	0,04804	---	ASF1B
8034772	-0,58	-1,49	0,00372	2,45	5,47	0,00050	NM_018154	FSIP1
7987426	-0,58	-1,49	0,03032	-1,10	-2,15	0,01787	NM_152597	---
8116846	-0,58	-1,49	0,28905	-1,44	-2,72	0,00032	---	DCST1
7905862	-0,58	-1,49	0,11550	-1,70	-3,26	0,02393	NM_152494	---
8132343	-0,58	-1,49	0,02764	-0,63	-1,54	0,03519	---	HMGB3
8170468	-0,58	-1,49	0,16762	0,62	1,54	0,16807	NM_005342	DKFZP434L187
7982252	-0,58	-1,49	0,28257	-1,24	-2,36	0,02345	NR_026771	LOC149134
7911170	-0,58	-1,49	0,10155	-1,05	-2,08	0,03764	NR_015422	---
7969977	-0,58	-1,49	0,34061	0,04	1,03	0,90827	---	TPSG1
7998427	-0,58	-1,49	0,35550	-1,01	-2,01	0,06003	NM_012467	---
7945952	-0,58	-1,49	0,22329	-0,95	-1,93	0,01346	---	LRCH4
8141535	-0,58	-1,49	0,05111	0,05	1,03	0,89261	NM_002319	GRWD1
8030035	-0,58	-1,49	0,18536	0,11	1,08	0,86981	NM_031485	PNRC1
8121076	-0,58	-1,49	0,01767	-0,69	-1,61	0,18090	NM_006813	RN7SL1
8040338	-0,58	-1,49	0,24612	-1,86	-3,63	0,02868	NR_002715	---
8176679	-0,58	-1,49	0,11875	-1,14	-2,20	0,01099	---	---
8177212	-0,58	-1,49	0,11875	-1,14	-2,20	0,01099	---	CD226
8023757	-0,58	-1,49	0,24680	-0,83	-1,78	0,10973	NM_006566	ZFP42
8098690	-0,58	-1,49	0,10872	2,13	4,38	0,00082	NM_174900	RGN
8167027	-0,58	-1,49	0,28403	-0,39	-1,31	0,25889	NM_152869	---
8028283	-0,58	-1,49	0,11410	-1,23	-2,34	0,00000	---	---
7987534	-0,58	-1,49	0,03816	-0,84	-1,79	0,09275	---	LOC100131497
7980481	-0,58	-1,49	0,19252	-0,38	-1,30	0,13035	BX248745 NXF4	
8168940	-0,58	-1,49	0,16224	-0,69	-1,61	0,00037	NR_002216	HSFX1
8170402	-0,58	-1,49	0,21954	-0,95	-1,93	0,04238	NM_016153	HSFX1
8175616	-0,58	-1,49	0,21954	-0,95	-1,93	0,04238	NM_016153	---
8004946	-0,58	-1,49	0,11612	-0,54	-1,45	0,18579	---	CTAGE4
8129560	-0,58	-1,50	0,21456	-0,30	-1,23	0,13489	NM_198495	GRIN3B
8024100	-0,58	-1,50	0,21373	-0,57	-1,49	0,20018	NM_138690	RIBC2
8073766	-0,58	-1,50	0,22849	0,69	1,62	0,01648	NM_015653	LRRC52
7906973	-0,58	-1,50	0,01922	-1,68	-3,21	0,01048	NM_001005214	---
8088337	-0,58	-1,50	0,16606	-0,37	-1,29	0,01119	---	---
7932211	-0,58	-1,50	0,40143	-2,05	-4,13	0,00942	---	PCDHB15
8108753	-0,58	-1,50	0,04721	-0,22	-1,17	0,57347	NM_018935	KCNJ1
7952617	-0,58	-1,50	0,27366	-1,28	-2,43	0,01530	NM_153767	CNBD1
8147194	-0,58	-1,50	0,01705	-1,19	-2,28	0,03171	NM_173538	GNAO1
7995739	-0,58	-1,50	0,02740	0,41	1,32	0,16041	NM_020988	ZNF487
7927169	-0,58	-1,50	0,44553	-0,85	-1,80	0,04053	NR_026693	SLC9A3R1
8009685	-0,58	-1,50	0,22106	1,31	2,48	0,05714	NM_004252	DNASE1
7992934	-0,58	-1,50	0,00091	0,19	1,14	0,28213	NM_005223	C8orf33
8148955	-0,58	-1,50	0,33643	2,02	4,05	0,00246	NM_023080	TMEM74
8152335	-0,58	-1,50	0,03538	1,31	2,47	0,00683	NM_153015	RAB38
7950899	-0,58	-1,50	0,03893	2,79	6,93	0,00002	NM_022337	C9orf93
8154416	-0,58	-1,50	0,04843	-0,45	-1,36	0,40325	AY422473 SIDT1	
8081710	-0,58	-1,50	0,13663	-1,76	-3,39	0,00748	NM_017699	ERMN
8055969	-0,58	-1,50	0,16160	-0,56	-1,47	0,01570	NM_001009959	CCL26
8140358	-0,58	-1,50	0,18727	-1,02	-2,03	0,00206	NM_006072	---
8105577	-0,58	-1,50	0,09815	-0,84	-1,79	0,06641	---	IL3RA
8165752	-0,58	-1,50	0,13398	-1,09	-2,13	0,00986	NM_002183	IL3RA

8176323	-0,58	-1,50	0,13398	-1,09	-2,13	0,00986	NM_002183	MYRIP
8078933	-0,58	-1,50	0,00466	-0,96	-1,95	0,02070	NM_015460	FASN
8019392	-0,58	-1,50	0,00589	1,95	3,86	0,01304	NM_004104	IRF2BP2
7925161	-0,58	-1,50	0,02457	0,21	1,16	0,66060	NM_182972	CXCL10
8101126	-0,58	-1,50	0,06393	-0,57	-1,49	0,01308	NM_001565	GNMT
8119620	-0,58	-1,50	0,15848	-0,84	-1,79	0,05003	NM_018960	C6orf64
8126147	-0,58	-1,50	0,26705	0,77	1,70	0,08799	BC022007	CDCP2
7916384	-0,58	-1,50	0,34508	-0,66	-1,58	0,04842	NM_201546	LUC7L
7998174	-0,58	-1,50	0,09871	0,00	1,00	0,99419	NM_018032	LRRC63
7968962	-0,58	-1,50	0,14953	-0,37	-1,30	0,48024	ENST00000446175	PTPN6
7953569	-0,58	-1,50	0,11553	2,57	5,93	0,00027	NM_080549	MYO18A
8013860	-0,58	-1,50	0,05275	0,69	1,61	0,08231	NM_078471	DIP2A
8069399	-0,58	-1,50	0,30266	1,44	2,71	0,01284	NM_015151	OR4A13P
7939920	-0,58	-1,50	0,18409	-1,05	-2,07	0,00041	ENST00000314689	NCF1
8133518	-0,58	-1,50	0,01679	-1,23	-2,35	0,00254	NM_000265	C1orf114
7922152	-0,58	-1,50	0,07968	-1,43	-2,69	0,00003	BC026073	---
7934777	-0,58	-1,50	0,00674	-0,62	-1,54	0,00216	---	SLC5A1
8072587	-0,58	-1,50	0,19796	-0,92	-1,89	0,00248	NM_000343	TPM1
7989491	-0,58	-1,50	0,08879	-1,73	-3,31	0,00111	AB209041	SNORD32B
8117746	-0,58	-1,50	0,00396	-0,95	-1,93	0,09766	NR_003049	SAMD14
8016615	-0,58	-1,50	0,12440	-1,12	-2,18	0,18586	NM_174920	MIR376C
7976838	-0,58	-1,50	0,05665	-0,70	-1,62	0,03904	NR_029861	SPINK4
8154779	-0,58	-1,50	0,04064	-1,01	-2,01	0,02324	NM_014471	COL8A2
7914880	-0,58	-1,50	0,48083	-1,53	-2,88	0,00074	NM_005202	---
7944791	-0,58	-1,50	0,11692	-0,28	-1,21	0,44358	---	OR10S1
7952384	-0,58	-1,50	0,39588	-0,85	-1,81	0,19484	NM_001004474	SHC2
8032037	-0,58	-1,50	0,12105	-1,54	-2,92	0,00090	NM_012435	SLC9A10
8089489	-0,58	-1,50	0,15228	-1,29	-2,44	0,04726	NM_183061	CCDC74A
8045309	-0,58	-1,50	0,02385	-1,03	-2,04	0,03718	NM_138770	LOC128102
7904429	-0,58	-1,50	0,52756	-2,11	-4,31	0,02664	AF252254	---
8015906	-0,58	-1,50	0,40287	-0,71	-1,63	0,03855	---	CORO2A
8162744	-0,58	-1,50	0,04294	2,08	4,23	0,00132	NM_003389	FASLG
7907430	-0,58	-1,50	0,24167	-1,65	-3,13	0,00017	NM_000639	---
8019081	-0,58	-1,50	0,17311	-1,16	-2,24	0,12284	---	LIMD1
8079334	-0,58	-1,50	0,14688	0,66	1,58	0,47680	NM_014240	POU6F2
8132387	-0,58	-1,50	0,24238	-1,49	-2,82	0,00022	NM_007252	LOC729040
8113071	-0,58	-1,50	0,11992	-2,23	-4,68	0,00041	ENST00000440769	CCR2
8079392	-0,58	-1,50	0,00448	-1,00	-2,00	0,05052	NM_001123396	---
8081405	-0,58	-1,50	0,01865	-0,63	-1,55	0,00492	---	---
8127991	-0,58	-1,50	0,12557	-1,00	-2,00	0,00461	---	OR51G1
7946013	-0,58	-1,50	0,37201	-0,58	-1,49	0,07637	NM_001005237	YY2
8166398	-0,59	-1,50	0,21861	-0,35	-1,28	0,30710	NM_206923	LAT
7994541	-0,59	-1,50	0,10612	-0,53	-1,44	0,18992	NM_014387	SLC27A4
8158224	-0,59	-1,50	0,29569	1,23	2,34	0,11659	NM_005094	TRDN
8129330	-0,59	-1,50	0,13763	-1,03	-2,04	0,00807	NM_006073	EHHADH
8092523	-0,59	-1,50	0,00970	0,98	1,97	0,03401	NM_001966	PRR14
7994858	-0,59	-1,50	0,38237	0,45	1,37	0,19627	NM_024031	---
8059475	-0,59	-1,50	0,16775	-0,16	-1,12	0,65187	---	ZNF335
8066641	-0,59	-1,50	0,01414	0,94	1,92	0,02546	NM_022095	LOC100129534
7911718	-0,59	-1,50	0,41202	0,14	1,10	0,63662	NR_024489	PTPN20B
7933379	-0,59	-1,50	0,04235	0,96	1,95	0,00427	NM_001042357	---
8136727	-0,59	-1,50	0,06741	-0,82	-1,77	0,00107	---	FYB
8105061	-0,59	-1,50	0,15609	-1,18	-2,26	0,00472	NM_001465	NSUN5
8140020	-0,59	-1,50	0,35766	2,99	7,97	0,00556	NM_018044	KRT6C
7963410	-0,59	-1,50	0,33915	-1,03	-2,04	0,11181	NM_173086	WASH1
8159790	-0,59	-1,50	0,47267	0,98	1,97	0,02062	NM_182905	C7orf54
8135902	-0,59	-1,50	0,00723	-0,75	-1,68	0,00671	NR_027330	NPY2R
8097938	-0,59	-1,50	0,13215	-2,08	-4,23	0,00002	NM_000910	---
8036954	-0,59	-1,50	0,01189	-0,87	-1,83	0,01689	---	USP18
8071155	-0,59	-1,50	0,40029	2,62	6,14	0,01808	NM_017414	HTR7P1
7954071	-0,59	-1,50	0,08253	-1,12	-2,18	0,00929	NR_002774	NOD2
7995539	-0,59	-1,50	0,05272	-0,68	-1,60	0,00844	NM_022162	TARP
8139107	-0,59	-1,50	0,27820	-1,13	-2,18	0,05402	NM_001003799	---
8156132	-0,59	-1,50	0,09387	-1,11	-2,16	0,08324	---	TLR5
7924499	-0,59	-1,50	0,02770	1,22	2,32	0,00974	NM_003268	CALCR
8140995	-0,59	-1,50	0,10146	-0,90	-1,87	0,01056	NM_001164737	---
8167871	-0,59	-1,50	0,19317	-0,39	-1,31	0,18456	---	ZNF434
7998967	-0,59	-1,50	0,04571	-0,38	-1,30	0,07996	NM_017810	LOC349196
8149153	-0,59	-1,50	0,65969	-1,48	-2,79	0,09931	NR_027000	LOC349196
8149157	-0,59	-1,50	0,65969	-1,48	-2,79	0,09931	NR_027000	GDF11
7956026	-0,59	-1,50	0,12847	-0,32	-1,25	0,56356	NM_005811	PNPLA7
8165575	-0,59	-1,50	0,01221	-0,42	-1,34	0,04842	NM_001098537	SPC25
8056572	-0,59	-1,50	0,42063	0,86	1,82	0,15804	NM_020675	RIMBP3

8074771	-0,59	-1,50	0,02390	-0,24	-1,18	0,20575	NM_015672	GPT2
7995362	-0,59	-1,50	0,11453	1,85	3,62	0,00554	NM_133443	LCN1
8158995	-0,59	-1,50	0,40958	-1,05	-2,07	0,04378	NM_002297	SNORD115-6
7982018	-0,59	-1,50	0,42470	-1,80	-3,49	0,00008	NR_003298	ITIH5L
8173106	-0,59	-1,50	0,21130	-0,94	-1,92	0,00722	NM_198510	---
7961889	-0,59	-1,50	0,10880	-1,82	-3,54	0,02243	---	AP3B2
7990987	-0,59	-1,50	0,07600	0,26	1,20	0,38790	NM_004644	MIR215
7924401	-0,59	-1,50	0,08908	-0,59	-1,50	0,04216	NR_029628	LRRC68
8029624	-0,59	-1,50	0,05659	0,63	1,55	0,15915	ENST00000421905	---
8070871	-0,59	-1,50	0,06232	-0,52	-1,43	0,10470	---	---
8106401	-0,59	-1,50	0,05579	-1,13	-2,19	0,04322	---	LRRC50
7997556	-0,59	-1,50	0,04797	-1,03	-2,04	0,03029	NM_178452	SORCS1
7936249	-0,59	-1,50	0,06101	-0,91	-1,88	0,00456	NM_052918	GSTZ1
7975956	-0,59	-1,50	0,23338	0,29	1,23	0,35155	NM_001513	LRFN5
7974109	-0,59	-1,50	0,03557	-2,02	-4,04	0,00265	NM_152447	KLHL32
8121193	-0,59	-1,50	0,02494	-0,81	-1,75	0,12384	NM_052904	---
8169443	-0,59	-1,50	0,18107	-0,90	-1,87	0,00050	---	MAP3K15
8171653	-0,59	-1,50	0,07823	0,94	1,92	0,00203	NM_001001671	LOC284542
7915949	-0,59	-1,50	0,55555	-1,67	-3,19	0,04152	AK090467	ANKRD11
8003448	-0,59	-1,50	0,02512	1,47	2,76	0,03524	NM_013275	OR1F1
7992863	-0,59	-1,50	0,30038	2,15	4,43	0,01984	NM_012360	---
8075162	-0,59	-1,50	0,02585	-1,07	-2,10	0,01188	---	FGF10
8111993	-0,59	-1,50	0,11540	-1,60	-3,03	0,02405	NM_004465	OR56B4
7938072	-0,59	-1,50	0,29430	-1,45	-2,72	0,00074	NM_001005181	PLEKHG4
7996516	-0,59	-1,50	0,11043	0,26	1,20	0,01664	NM_015432	ARHGAP9
7964436	-0,59	-1,50	0,11690	-0,07	-1,05	0,55722	NM_032496	BEND5
7915982	-0,59	-1,50	0,30635	-1,06	-2,09	0,11607	NM_024603	MED30
8148022	-0,59	-1,50	0,14292	-0,18	-1,13	0,48922	NM_080651	SNORA13
8107326	-0,59	-1,50	0,15967	-0,36	-1,28	0,25045	NR_002922	STAT4
8057771	-0,59	-1,50	0,06705	0,94	1,92	0,07884	NM_003151	HBII-52-27
7982060	-0,59	-1,50	0,03420	-0,47	-1,38	0,00914	NR_003496	---
8021961	-0,59	-1,50	0,04817	-0,88	-1,85	0,03861	---	OR8J3 // OR8J3
7948119	-0,59	-1,50	0,00911	-0,98	-1,97	0,00245	NM_001004064 // NM_001004064	

8140965	-0,59	-1,50	0,28869	-1,75	-3,37	0,02941	---	FERD3L
8138450	-0,59	-1,50	0,28721	-0,79	-1,73	0,01922	NM_152898	---
7907049	-0,59	-1,50	0,18975	-0,68	-1,60	0,01394	---	LOC349196
7953735	-0,59	-1,50	0,67870	-1,70	-3,26	0,08709	NR_027000	---
8037387	-0,59	-1,50	0,08379	-1,43	-2,70	0,00105	---	---
8144488	-0,59	-1,50	0,65917	-1,57	-2,97	0,16727	---	CDK20
8162194	-0,59	-1,50	0,07171	0,24	1,18	0,37031	NM_178432	---
8013570	-0,59	-1,50	0,20756	-0,69	-1,62	0,00288	---	OR5P2
7946323	-0,59	-1,50	0,07479	-1,37	-2,59	0,00798	NM_153444	MS4A10
7940341	-0,59	-1,51	0,40849	-1,49	-2,81	0,00578	NM_206893	DNAJA4
7985147	-0,59	-1,51	0,08390	1,19	2,28	0,01155	NM_018602	OBSL1
8059249	-0,59	-1,51	0,28855	0,10	1,07	0,44541	NM_015311	---
8142538	-0,59	-1,51	0,21595	-0,47	-1,38	0,09890	---	---
8160470	-0,59	-1,51	0,22641	-1,07	-2,10	0,02199	---	CAPN6
8174527	-0,59	-1,51	0,09776	-1,07	-2,10	0,00402	NM_014289	ZNF629
8000941	-0,59	-1,51	0,08945	-0,80	-1,74	0,22904	NM_001080417	MIR122
8021416	-0,59	-1,51	0,02725	-0,94	-1,92	0,00256	NR_029667	CCDC144A
8005204	-0,59	-1,51	0,05663	0,43	1,35	0,40461	NM_014695	---
8030826	-0,59	-1,51	0,00765	-0,87	-1,83	0,00953	---	UCP2
7950307	-0,59	-1,51	0,35009	1,94	3,85	0,00566	NM_003355	MIR9-3
7985871	-0,59	-1,51	0,18699	-0,94	-1,92	0,00077	NR_029692	GET4
8131044	-0,59	-1,51	0,30754	1,38	2,61	0,09683	NM_015949	NCF1
8140227	-0,59	-1,51	0,03362	-1,32	-2,50	0,00012	NM_000265	---
8076384	-0,59	-1,51	0,29952	-1,87	-3,67	0,00504	---	SEMA3B
8079966	-0,59	-1,51	0,23251	-0,15	-1,11	0,57696	NM_004636	KNG1
8084679	-0,59	-1,51	0,05678	-0,69	-1,62	0,01231	NM_000893	ACOT11
7901613	-0,59	-1,51	0,19149	-0,61	-1,53	0,00030	NM_147161	OR11H1
7972981	-0,59	-1,51	0,15203	-2,68	-6,40	0,00030	NM_001005239	OR11H1
8074194	-0,59	-1,51	0,15203	-2,68	-6,40	0,00030	NM_001005239	AGAP4
7933405	-0,59	-1,51	0,28567	1,34	2,54	0,01079	NM_133446	LOC100293553
8169231	-0,59	-1,51	0,26852	-1,41	-2,66	0,00437	ENST00000372451	OR4Q2
7973020	-0,59	-1,51	0,36335	-1,70	-3,25	0,00753	ENST00000357151	---
8169715	-0,59	-1,51	0,16326	-1,34	-2,53	0,01848	---	CEACAM3
8029107	-0,59	-1,51	0,20668	-1,60	-3,04	0,04717	NM_001815	CIB2
7990674	-0,59	-1,51	0,00984	-0,28	-1,22	0,26105	NM_006383	RP11-90M2.3
7971561	-0,59	-1,51	0,11956	-0,59	-1,50	0,01230	ENST00000438155	FAM71F1
8135945	-0,59	-1,51	0,32699	-1,04	-2,05	0,03921	NM_032599	---
8155024	-0,59	-1,51	0,00570	-1,17	-2,25	0,01452	---	---
8174302	-0,59	-1,51	0,22222	-0,62	-1,53	0,03222	---	OR2T11

7925755	-0,59	-1,51	0,23309	-1,53	-2,88	0,00048	NM_001001964	PARD6G
8023926	-0,59	-1,51	0,14601	-0,05	-1,04	0,62954	NM_032510	---
8136891	-0,59	-1,51	0,08762	-0,64	-1,56	0,02016	---	---
8125434	-0,59	-1,51	0,09711	-0,74	-1,67	0,11569	---	---
7936089	-0,59	-1,51	0,21875	-0,61	-1,53	0,07970	---	---
8126119	-0,59	-1,51	0,01295	-0,73	-1,65	0,06678	---	---
8050801	-0,59	-1,51	0,07276	-0,63	-1,55	0,09614	---	---
7945084	-0,59	-1,51	0,19229	-1,02	-2,02	0,02944	---	GPR83
7951040	-0,59	-1,51	0,24162	-1,36	-2,58	0,03576	NM_016540	HIRIP3
8000748	-0,59	-1,51	0,07219	-0,69	-1,61	0,04012	NM_003609	SLC10A6
8101609	-0,59	-1,51	0,27222	-1,36	-2,57	0,02895	NM_197965	---
8091627	-0,59	-1,51	0,03504	-0,85	-1,81	0,09060	---	TMPRSS13
7951987	-0,59	-1,51	0,06629	-0,17	-1,12	0,36373	NM_001077263	---
8139055	-0,59	-1,51	0,13856	-1,91	-3,76	0,02838	---	C16orf88
7999903	-0,59	-1,51	0,02949	0,36	1,29	0,39359	NM_001012991	TSPAN12
8142524	-0,59	-1,51	0,14914	0,28	1,22	0,31286	NM_012338	FLJ00049
8131140	-0,59	-1,51	0,04951	-0,42	-1,34	0,39901	AK024457	---
8084834	-0,59	-1,51	0,01543	-1,07	-2,09	0,01972	---	CLEC4D
7953749	-0,59	-1,51	0,06038	-0,66	-1,58	0,01589	NM_080387	ROM1
7940662	-0,59	-1,51	0,11382	-0,90	-1,86	0,00302	NM_000327	AMIGO1
7918359	-0,59	-1,51	0,18499	-1,24	-2,36	0,12049	NM_020703	---
7969914	-0,59	-1,51	0,03091	-0,77	-1,70	0,00017	---	HKR1
8028233	-0,59	-1,51	0,20363	0,18	1,13	0,69130	NM_181786	---
7979961	-0,59	-1,51	0,08090	-1,18	-2,26	0,01943	---	---
7951552	-0,59	-1,51	0,14726	-0,81	-1,76	0,03277	---	---
7993110	-0,59	-1,51	0,23187	-0,75	-1,69	0,01437	---	LOC554249
8161353	-0,59	-1,51	0,16066	0,32	1,25	0,02029	AK292642	HOXD12
8046527	-0,59	-1,51	0,10852	-0,98	-1,98	0,00671	NM_021193	C18orf1
8020308	-0,59	-1,51	0,23555	-1,33	-2,51	0,05601	NM_181481	PRM1
7999440	-0,60	-1,51	0,19788	-1,94	-3,84	0,00254	NM_002761	SLC15A1
7972461	-0,60	-1,51	0,06268	0,06	1,04	0,71246	NM_005073	---
7920795	-0,60	-1,51	0,11694	-0,94	-1,91	0,00419	---	MYH16
8134564	-0,60	-1,51	0,20881	-0,64	-1,55	0,04394	NR_002147	---
7977125	-0,60	-1,51	0,00112	-0,62	-1,53	0,00598	---	CCDC12
8086698	-0,60	-1,51	0,05088	-1,14	-2,20	0,03690	NM_144716	FLJ43860
8148512	-0,60	-1,51	0,14140	-0,50	-1,42	0,33823	NM_207414	C14orf166B
7975901	-0,60	-1,51	0,02586	-1,17	-2,24	0,03549	NM_194287	---
8102408	-0,60	-1,51	0,14593	-0,87	-1,83	0,00016	---	TAS2R19
7961287	-0,60	-1,51	0,20325	-0,34	-1,27	0,15190	NM_176888	HAVCR2
8115464	-0,60	-1,51	0,04884	-0,94	-1,92	0,01327	NM_032782	---
8096030	-0,60	-1,51	0,00514	-0,87	-1,83	0,02634	---	LOC400499
7999447	-0,60	-1,51	0,13792	-1,23	-2,34	0,00615	ENST00000344649	MAGEB5
8166576	-0,60	-1,51	0,02679	-0,65	-1,57	0,00586	ENST00000379029	---
8154447	-0,60	-1,51	0,11887	-0,46	-1,38	0,06049	---	---
8083229	-0,60	-1,51	0,07070	-1,05	-2,07	0,01757	---	ACOT11
7916412	-0,60	-1,51	0,20272	-1,98	-3,94	0,04926	NM_147161	---
8025474	-0,60	-1,51	0,15489	-1,33	-2,52	0,00747	---	ST6GALNAC4
8164314	-0,60	-1,51	0,04892	0,86	1,81	0,18181	NM_175039	---
8001656	-0,60	-1,51	0,27984	-1,34	-2,53	0,00464	---	AIPL1
8011912	-0,60	-1,51	0,19323	-1,02	-2,03	0,00104	NM_014336	PGA4
7940431	-0,60	-1,51	0,41090	-1,56	-2,96	0,02535	NM_001079808	RETNLB
8089394	-0,60	-1,51	0,31823	-0,97	-1,96	0,06248	NM_032579	STK32A
8108981	-0,60	-1,51	0,03519	3,01	8,08	0,00435	NM_001112724	LOC554207
7973182	-0,60	-1,51	0,29888	-1,04	-2,05	0,00209	ENST00000320322	---
7932086	-0,60	-1,51	0,04629	0,02	1,01	0,82214	---	GDPD4
7950560	-0,60	-1,51	0,01909	-1,25	-2,37	0,00035	NM_182833	MUC7
8095504	-0,60	-1,51	0,11338	-1,24	-2,35	0,00098	NM_001145006	IRF4
8116559	-0,60	-1,51	0,13544	-0,99	-1,98	0,06870	NM_002460	CABIN1
8071823	-0,60	-1,51	0,22074	0,27	1,20	0,60977	NM_012295	---
8087778	-0,60	-1,51	0,14621	-1,22	-2,33	0,00314	---	---
8031568	-0,60	-1,51	0,35427	-1,67	-3,17	0,00101	---	DAB1
7916566	-0,60	-1,51	0,01251	-1,09	-2,12	0,02470	AF263547	PDE6A
8115041	-0,60	-1,51	0,18723	-0,90	-1,87	0,00580	NM_000440	---
7953965	-0,60	-1,51	0,07716	-0,89	-1,85	0,03117	---	---
8062478	-0,60	-1,51	0,02297	-0,69	-1,62	0,00836	---	TK2
7996286	-0,60	-1,51	0,28561	-2,21	-4,63	0,00047	NM_004614	CDSN
8179716	-0,60	-1,51	0,51528	-1,56	-2,94	0,01353	NM_001264	ZNF697
7919051	-0,60	-1,51	0,00051	-1,16	-2,24	0,07276	NM_001080470	---
8135847	-0,60	-1,51	0,22041	-0,89	-1,85	0,01171	---	OR12D2
8177700	-0,60	-1,51	0,17351	-1,03	-2,04	0,05640	NM_013936	---
8146423	-0,60	-1,51	0,25646	-1,06	-2,08	0,00085	---	---
8095356	-0,60	-1,51	0,04093	-0,32	-1,25	0,21908	---	LOC349196
8149161	-0,60	-1,51	0,61782	-1,29	-2,44	0,11401	NR_027000	LOC349196

8149210	-0,60	-1,51	0,61782	-1,29	-2,44	0,11401	NR_027000	---
8130027	-0,60	-1,51	0,28089	-0,53	-1,44	0,02483	---	FLJ13744
8127754	-0,60	-1,51	0,06024	-1,63	-3,10	0,03103	BC070061	---
7905629	-0,60	-1,51	0,13365	-0,49	-1,40	0,01337	---	CLIP3
8036252	-0,60	-1,51	0,07845	-2,18	-4,54	0,00088	NM_015526	EFNA4
7905909	-0,60	-1,51	0,21782	1,49	2,81	0,02701	NM_005227	SLC10A2
7972692	-0,60	-1,51	0,19584	-1,17	-2,25	0,04281	NM_000452	PLIN4
8032829	-0,60	-1,51	0,01439	-0,36	-1,28	0,46941	NM_001080400	SNORD52
8118322	-0,60	-1,51	0,07048	-0,38	-1,30	0,36035	NR_002742	---
8083933	-0,60	-1,51	0,18614	0,16	1,12	0,28184	---	RASGRP4
8036503	-0,60	-1,51	0,06366	-1,71	-3,27	0,00006	NM_170604	LOC100130433
8156192	-0,60	-1,51	0,14920	-0,86	-1,81	0,13803	AK096255	CDH18
8111220	-0,60	-1,51	0,19490	-1,02	-2,03	0,00592	NM_004934	PRSS16
8117547	-0,60	-1,51	0,26857	1,49	2,82	0,00492	NM_005865	ASB7
7986433	-0,60	-1,51	0,10568	-0,09	-1,06	0,87206	NM_198243	IFNB1
8160360	-0,60	-1,51	0,16490	-0,47	-1,38	0,15073	NM_002176	TAS2R13
7961275	-0,60	-1,52	0,06256	-1,01	-2,01	0,06823	NM_023920	---
8144151	-0,60	-1,52	0,00484	-0,75	-1,68	0,17202	---	C21orf58
8071020	-0,60	-1,52	0,33461	-0,24	-1,18	0,19302	NM_058180	---
8021243	-0,60	-1,52	0,07421	-1,36	-2,57	0,02798	---	TBC1D3H
8014420	-0,60	-1,52	0,38751	1,25	2,38	0,05393	NM_001123392	SNORD115-4
7982014	-0,60	-1,52	0,14895	-1,22	-2,33	0,02493	NR_003296	CACNG6
8031090	-0,60	-1,52	0,16111	0,44	1,36	0,19120	NM_145814	CDH1
7996837	-0,60	-1,52	0,24297	3,76	13,51	0,00627	NM_004360	---
8137089	-0,60	-1,52	0,27625	-0,54	-1,45	0,00060	---	CHRM3
7910915	-0,60	-1,52	0,04882	-0,56	-1,47	0,06170	NM_000740	WDR27
8130891	-0,60	-1,52	0,03319	0,50	1,42	0,02870	NM_182552	ZNF613
8030871	-0,60	-1,52	0,00688	0,07	1,05	0,87529	NM_001031721	KAZALD1
7929932	-0,60	-1,52	0,02490	-0,67	-1,59	0,06973	NM_030929	FRG1B
8061497	-0,60	-1,52	0,19050	1,22	2,33	0,03620	NR_003579	TAS2R16
8142624	-0,60	-1,52	0,17915	-1,34	-2,52	0,02129	NM_016945	MAPK12
8076962	-0,60	-1,52	0,14961	0,19	1,14	0,66488	NM_002969	GLTPD1
8039937	-0,60	-1,52	0,48555	-0,88	-1,84	0,03816	NM_001029885	WDR52
8089606	-0,60	-1,52	0,07326	-0,78	-1,72	0,03967	NM_018338	GVIN1
7946275	-0,60	-1,52	0,04564	-1,55	-2,92	0,00033	NR_003945	---
8042115	-0,60	-1,52	0,22247	-1,49	-2,81	0,03443	---	C4orf21
8102389	-0,60	-1,52	0,04553	-0,61	-1,53	0,01271	NM_018392	MGC16703
8074695	-0,60	-1,52	0,22585	-0,82	-1,76	0,00413	NR_003608	C1orf211
7897277	-0,60	-1,52	0,20358	0,54	1,45	0,01910	BC030279	---
8083895	-0,60	-1,52	0,05687	-1,51	-2,84	0,00023	---	RNU2-1
8019802	-0,60	-1,52	0,66075	-2,59	-6,01	0,00688	NR_002716	ADCK4
8036938	-0,60	-1,52	0,15379	0,17	1,12	0,61218	NM_024876	GOLGA6L2
7986744	-0,60	-1,52	0,06484	-1,16	-2,23	0,01254	BC129821	ZSCAN16
8117640	-0,60	-1,52	0,09045	0,25	1,19	0,51264	NM_025231	FAM170A
8107554	-0,60	-1,52	0,13535	-0,62	-1,54	0,00843	NM_182761	MEX3D
8032265	-0,60	-1,52	0,36708	-0,12	-1,09	0,83381	NM_203304	LSP1
7968126	-0,60	-1,52	0,36406	-0,94	-1,92	0,07430	NM_002339	---
7946321	-0,60	-1,52	0,40601	-1,42	-2,67	0,00341	---	MMP12
7951297	-0,60	-1,52	0,16307	-2,03	-4,10	0,00003	NM_002426	EFNA3
7905918	-0,60	-1,52	0,00002	2,11	4,32	0,01789	NM_004952	---
8045345	-0,60	-1,52	0,00821	-0,73	-1,65	0,00418	---	CXCL17
8037197	-0,60	-1,52	0,15483	-0,78	-1,72	0,01858	NM_198477	AGAP2
7964499	-0,60	-1,52	0,24150	-1,71	-3,28	0,00088	NM_001122772	E2F1
8065710	-0,60	-1,52	0,14351	0,97	1,96	0,01716	NM_005225	LANCL3
8166714	-0,60	-1,52	0,13119	-1,01	-2,01	0,00536	NM_198511	CLNK
8099368	-0,60	-1,52	0,30120	-0,97	-1,96	0,00113	NM_052964	---
8151219	-0,60	-1,52	0,34742	-0,93	-1,91	0,00139	---	MIR216A
8052374	-0,60	-1,52	0,05961	-0,68	-1,60	0,01811	NR_029629	C1orf96
7924897	-0,60	-1,52	0,04809	-0,41	-1,33	0,25647	NM_145257	VWDE
8138258	-0,60	-1,52	0,03442	1,85	3,61	0,03222	NM_001135924	SMCP
7905530	-0,60	-1,52	0,15425	-1,44	-2,71	0,08981	NM_030663	TRPC5
8174556	-0,60	-1,52	0,02507	-1,39	-2,61	0,00044	NM_012471	RGS3
8157324	-0,60	-1,52	0,01777	-0,44	-1,36	0,09436	NM_144488	---
8094086	-0,60	-1,52	0,27287	-0,49	-1,41	0,03341	---	TMEM107
8012383	-0,60	-1,52	0,10984	0,69	1,61	0,02941	NM_032354	MIR134
7976844	-0,60	-1,52	0,09382	-1,69	-3,22	0,00182	NR_029698	C20orf46
8064415	-0,60	-1,52	0,01086	-1,15	-2,22	0,00192	NM_018354	NLGN3
8168264	-0,60	-1,52	0,02634	-0,48	-1,39	0,23400	NM_181303	HSP90B2P
7986407	-0,60	-1,52	0,21861	-0,26	-1,20	0,19576	AY956768	MST1R
8087547	-0,60	-1,52	0,02292	2,65	6,28	0,00297	NM_002447	LOC100288884
8071042	-0,60	-1,52	0,39796	-0,73	-1,66	0,04668	AK124122	TBC1D3C
8014397	-0,60	-1,52	0,31923	1,09	2,12	0,08591	NM_001001418	LOC100132147 // LOC100132147

7898350	-0,60	-1,52	0,38013	-0,55	-1,46	0,23238	BC126340 // BC126340	
	LOC100132147 // LOC100132147							
7912884	-0,60	-1,52	0,38013	-0,55	-1,46	0,23238	BC126340 // BC126340	CCDC84
7944365	-0,60	-1,52	0,23869	3,65	12,53	0,00011	NM_198489	CCDC136
8135969	-0,60	-1,52	0,12789	-0,46	-1,38	0,10177	NM_022742	---
8042825	-0,61	-1,52	0,20467	-0,27	-1,21	0,25798	---	OR1Q1
8157679	-0,61	-1,52	0,26855	-1,95	-3,88	0,00120	NM_012364	---
7965916	-0,61	-1,52	0,00069	-1,27	-2,42	0,00167	---	UGT3A1
8111512	-0,61	-1,52	0,07737	-1,41	-2,66	0,02588	NM_152404	SNORA68
8026875	-0,61	-1,52	0,00603	-0,49	-1,40	0,31899	NR_000012	ITIH4
8088020	-0,61	-1,52	0,32716	-1,31	-2,47	0,00077	NM_002218	KRT3
7963523	-0,61	-1,52	0,09974	-0,35	-1,27	0,25781	NM_057088	LCE1E
7905519	-0,61	-1,52	0,12802	-1,01	-2,01	0,09444	NM_178353	RGL2
8178955	-0,61	-1,52	0,14883	1,10	2,14	0,12822	NM_004761	RGL2
8180144	-0,61	-1,52	0,14883	1,10	2,14	0,12822	NM_004761	TRAF2
8159476	-0,61	-1,52	0,01773	0,73	1,66	0,12010	NM_021138	LOC100289612
7914878	-0,61	-1,52	0,10830	0,50	1,41	0,34772	AY605064	DCLRE1B
7904131	-0,61	-1,52	0,01454	0,21	1,16	0,54758	NM_022836	PSPH
8139737	-0,61	-1,52	0,20263	2,56	5,88	0,00804	NM_004577	LOC349196
8149228	-0,61	-1,52	0,65599	-1,72	-3,30	0,09856	NR_027000	RASGEF1A
7933149	-0,61	-1,52	0,29358	1,42	2,68	0,00226	NM_145313	C18orf18
8022106	-0,61	-1,52	0,01608	0,20	1,15	0,37937	NR_026849	FAM106A
8005687	-0,61	-1,52	0,31495	-0,93	-1,91	0,02399	NR_026809	---
8095703	-0,61	-1,52	0,11622	-1,65	-3,13	0,00753	---	SNORD116-16
7981980	-0,61	-1,52	0,14970	-0,45	-1,36	0,00375	NR_003331	INSL5
7916785	-0,61	-1,52	0,06902	-0,78	-1,72	0,05724	NM_005478	CHAC2
8041961	-0,61	-1,52	0,05395	1,57	2,97	0,04737	NM_001008708	VSX1
8065460	-0,61	-1,52	0,15863	-1,43	-2,70	0,00100	NM_014588	FOXO4
8168205	-0,61	-1,52	0,26239	-1,19	-2,29	0,05740	NM_005938	OR6F1
7925728	-0,61	-1,52	0,16777	-1,39	-2,62	0,04557	NM_001005286	DNAJB12
7934244	-0,61	-1,52	0,01824	-0,06	-1,04	0,58364	NM_001002762	C2orf14
8045210	-0,61	-1,52	0,28483	-1,78	-3,44	0,00529	NR_023391	C12orf44
7955606	-0,61	-1,52	0,04953	-1,08	-2,12	0,02117	NM_021934	SOCS7
8006768	-0,61	-1,52	0,10473	0,95	1,93	0,14517	NM_014598	PGLYRP3
7920220	-0,61	-1,52	0,27634	-2,37	-5,16	0,00036	NM_052891	FLJ37307
7971723	-0,61	-1,52	0,26192	-1,19	-2,29	0,01840	NR_027047	---
8089757	-0,61	-1,52	0,05032	-1,13	-2,18	0,00280	---	OR7E13P
7950883	-0,61	-1,52	0,08614	-1,37	-2,59	0,00133	AF238487	NCF1
8133314	-0,61	-1,52	0,02710	-1,30	-2,46	0,00081	NM_000265	---
8163084	-0,61	-1,52	0,08401	-1,45	-2,74	0,02781	---	TTC26
8136495	-0,61	-1,52	0,01898	0,60	1,52	0,08395	NM_024926	MIR33A
8073544	-0,61	-1,52	0,03212	-0,81	-1,75	0,00878	NR_029507	TMEM132E
8006466	-0,61	-1,52	0,30917	-0,90	-1,87	0,16392	NM_207313	---
8174592	-0,61	-1,52	0,01234	-0,81	-1,76	0,06539	---	OR2A12
8136971	-0,61	-1,52	0,03750	-0,42	-1,34	0,01356	NM_001004135	---
8088335	-0,61	-1,52	0,06948	-1,01	-2,01	0,02697	---	OR4C16
7939926	-0,61	-1,52	0,20220	-0,41	-1,33	0,00062	NM_001004701	---
8170235	-0,61	-1,52	0,05290	-1,30	-2,46	0,00115	---	RCE1
7941732	-0,61	-1,52	0,18356	2,56	5,90	0,01975	NM_005133	---
8108040	-0,61	-1,52	0,06945	-0,80	-1,74	0,01145	---	PTCD1
8141283	-0,61	-1,52	0,15552	-0,16	-1,12	0,51427	NM_015545	TECRL
8100557	-0,61	-1,52	0,02084	-0,98	-1,98	0,07270	NM_001010874	RNF32
8137566	-0,61	-1,52	0,07406	-1,26	-2,40	0,02188	NM_030936	---
7982514	-0,61	-1,52	0,51600	-1,67	-3,19	0,17771	---	RASA4
8141803	-0,61	-1,52	0,29481	-0,43	-1,35	0,40844	NM_006989	C1orf74
7924055	-0,61	-1,52	0,13868	0,39	1,31	0,55023	BC039719	---
8077777	-0,61	-1,52	0,16138	-0,98	-1,97	0,03946	---	---
7917591	-0,61	-1,52	0,08812	-1,10	-2,14	0,08162	---	TCEB3C
8023156	-0,61	-1,52	0,50984	-1,87	-3,65	0,01338	NM_145653	NHLRC1
8124129	-0,61	-1,52	0,18067	0,83	1,78	0,01345	NM_198586	---
8002027	-0,61	-1,52	0,14924	-1,17	-2,24	0,00008	---	MLXIPL
8140085	-0,61	-1,52	0,26122	0,92	1,90	0,21193	NM_032951	---
8100426	-0,61	-1,52	0,24295	-1,23	-2,35	0,02306	---	HIVEP3
7915392	-0,61	-1,52	0,16327	0,23	1,17	0,25201	NM_024503	USP26
8175206	-0,61	-1,52	0,00536	-0,52	-1,43	0,03625	NM_031907	CYP3A43
8134655	-0,61	-1,52	0,07903	-1,56	-2,95	0,00669	NM_022820	TRIM40
8177750	-0,61	-1,52	0,16976	-1,44	-2,72	0,00208	NM_138700	TRIM40
8179060	-0,61	-1,52	0,16976	-1,44	-2,72	0,00208	NM_138700	CENPI
8168794	-0,61	-1,52	0,01230	2,33	5,04	0,00111	NM_006733	GOLGA7B
7929711	-0,61	-1,52	0,08663	-0,01	-1,00	0,92657	NM_001010917	---
8020650	-0,61	-1,52	0,37066	-0,71	-1,64	0,12455	---	DND1
8114625	-0,61	-1,52	0,56177	-0,88	-1,84	0,04313	NM_194249	SPINK2
8100523	-0,61	-1,52	0,01810	0,11	1,08	0,66168	NM_021114	---

8172305	-0,61	-1,52	0,22365	-1,09	-2,13	0,01146	--- BACH2	
8128247	-0,61	-1,52	0,02478	-0,84	-1,79	0,02313	NM_021813	ARHGAP33
8028030	-0,61	-1,52	0,08599	-0,18	-1,14	0,36477	NM_001172630	OR10G8
7944801	-0,61	-1,53	0,04034	-1,39	-2,62	0,00110	NM_001004464	---
8040247	-0,61	-1,53	0,06852	-0,35	-1,28	0,48929	--- ---	
8053713	-0,61	-1,53	0,69392	-2,51	-5,69	0,00030	--- KRT80	
7963333	-0,61	-1,53	0,08495	2,32	4,99	0,00269	NM_182507	GRAMD1C
8081758	-0,61	-1,53	0,07981	1,99	3,98	0,00911	NM_017577	---
8139118	-0,61	-1,53	0,46227	0,17	1,12	0,55217	--- HS3ST4	
7994260	-0,61	-1,53	0,19810	-1,26	-2,39	0,02073	NM_006040	WDR38
8157818	-0,61	-1,53	0,13613	-1,82	-3,52	0,00641	NM_001045476	SIRT3
7945357	-0,61	-1,53	0,07571	2,17	4,52	0,00108	NM_012239	---
8147862	-0,61	-1,53	0,01732	-1,27	-2,42	0,02197	--- ---	
7955423	-0,61	-1,53	0,09145	-1,78	-3,44	0,00000	--- MAFA	
8153409	-0,61	-1,53	0,08244	-0,77	-1,71	0,03960	NM_201589	OR9Q1
7940103	-0,61	-1,53	0,10615	-2,00	-3,99	0,01420	NM_001005212	---
8125857	-0,61	-1,53	0,15324	-1,41	-2,65	0,00763	--- ADAM22	
8133983	-0,61	-1,53	0,00004	0,35	1,28	0,04901	NM_021723	hCG_1651160
7937934	-0,61	-1,53	0,30573	-1,02	-2,02	0,00806	ENST00000421628	hCG_1651160
7945954	-0,61	-1,53	0,30573	-1,02	-2,02	0,00806	ENST00000421628	MYO18B
8072039	-0,61	-1,53	0,08311	-1,05	-2,07	0,03454	NM_032608	RUNDC1
8007435	-0,61	-1,53	0,11812	0,87	1,83	0,00299	NM_173079	SPANXN3
8175572	-0,61	-1,53	0,00815	-0,33	-1,26	0,07087	NM_001009609	PTPN20A
7933263	-0,61	-1,53	0,02395	0,91	1,88	0,00557	NM_001042389	---
8113276	-0,61	-1,53	0,03593	-0,62	-1,54	0,01099	--- ---	
8084836	-0,61	-1,53	0,05555	-1,05	-2,07	0,00589	--- TIFA	
8102362	-0,61	-1,53	0,33487	1,18	2,27	0,05049	NM_052864	PPP1R16B
8062557	-0,61	-1,53	0,15743	-0,54	-1,45	0,12030	NM_015568	B4GALNT1
7964484	-0,61	-1,53	0,25536	1,89	3,71	0,03255	NM_001478	C6orf47
8178552	-0,61	-1,53	0,03122	0,26	1,20	0,69841	NM_021184	C6orf47
8179808	-0,61	-1,53	0,03122	0,26	1,20	0,69841	NM_021184	KRTAP9-2
8007123	-0,61	-1,53	0,26418	-1,14	-2,21	0,01326	NM_031961	ARHGEF10
8144315	-0,61	-1,53	0,05157	-0,65	-1,57	0,21371	BC026965 MMP10	
7951259	-0,61	-1,53	0,00222	-1,18	-2,27	0,00091	NM_002425	AKR7A2
7913156	-0,61	-1,53	0,02739	2,15	4,44	0,00743	NM_003689	C11orf40
7945974	-0,61	-1,53	0,08859	-1,05	-2,07	0,05069	NM_144663	ASCL4
7958396	-0,61	-1,53	0,17450	-1,22	-2,32	0,06816	NM_203436	INTS1
8137739	-0,61	-1,53	0,00742	1,00	2,00	0,12709	NM_001080453	---
7970542	-0,61	-1,53	0,04741	-1,16	-2,24	0,08659	--- C6orf81	
8118981	-0,61	-1,53	0,06643	-0,74	-1,67	0,02117	BC033033 CYB561D1	
7903688	-0,61	-1,53	0,28485	-0,30	-1,23	0,60873	NM_182580	FLJ40194
8008195	-0,61	-1,53	0,04168	-1,15	-2,22	0,01345	AK097513 TEX261	
8042566	-0,61	-1,53	0,19554	-0,44	-1,36	0,00479	NM_144582	AOAH
8139033	-0,61	-1,53	0,14926	0,11	1,08	0,78328	NM_001637	KERA
7965398	-0,61	-1,53	0,03583	-0,79	-1,73	0,04808	NM_007035	TBC1D3
8014633	-0,61	-1,53	0,33719	1,27	2,41	0,06147	NM_001123391	---
8176921	-0,61	-1,53	0,42432	-0,54	-1,45	0,14230	--- ---	
8177424	-0,61	-1,53	0,42432	-0,54	-1,45	0,14230	--- ---	
8075918	-0,61	-1,53	0,09294	-1,10	-2,14	0,01582	--- ---	
8020252	-0,61	-1,53	0,01103	-0,50	-1,41	0,17380	--- DNAH14	
7910054	-0,61	-1,53	0,00010	-0,23	-1,17	0,34363	NM_001373	ARVP6125
8082583	-0,61	-1,53	0,33266	-0,33	-1,25	0,49932	AY358247 BRD1	
8076836	-0,61	-1,53	0,03885	1,34	2,53	0,01315	NM_014577	FAM106C
8005231	-0,61	-1,53	0,26613	-0,85	-1,80	0,03622	NR_026810	TMC4
8039144	-0,61	-1,53	0,14374	1,57	2,97	0,02294	NM_001145303	---
8048118	-0,61	-1,53	0,02971	-1,42	-2,68	0,00481	--- SLCO1B1	
7954356	-0,61	-1,53	0,01179	-1,21	-2,31	0,01671	NM_006446	LOC220429
7969151	-0,61	-1,53	0,02430	-0,81	-1,75	0,04385	NR_003268	CD3E
7944179	-0,61	-1,53	0,11811	-1,67	-3,19	0,00266	NM_000733	ROPN1
8090152	-0,61	-1,53	0,10719	-0,65	-1,57	0,06954	NM_017578	MAGEE2
8173665	-0,61	-1,53	0,03615	-0,84	-1,79	0,03437	NM_138703	SLC25A21
7978692	-0,61	-1,53	0,19038	0,47	1,39	0,12660	NM_030631	ATP1A4
7906527	-0,61	-1,53	0,07590	-1,59	-3,01	0,00844	NM_144699	SEC14L3
8075375	-0,61	-1,53	0,34577	-0,29	-1,22	0,26790	NM_174975	---
8059341	-0,61	-1,53	0,12196	-0,35	-1,27	0,19442	--- FAM174B	
7991453	-0,61	-1,53	0,22547	0,07	1,05	0,83376	NM_207446	TNFSF12-TNFSF13
8004464	-0,61	-1,53	0,19134	1,40	2,63	0,01377	NM_172089	SGK2
8062728	-0,62	-1,53	0,05421	-0,29	-1,23	0,52776	NM_016276	HTN1
8095435	-0,62	-1,53	0,07530	-0,43	-1,35	0,00452	NM_002159	NCRNA00110
8068126	-0,62	-1,53	0,02113	-1,52	-2,87	0,01294	AY077697 MYOG	
7923528	-0,62	-1,53	0,28284	-1,70	-3,25	0,02508	NM_002479	SLC22A7
8119782	-0,62	-1,53	0,04784	-0,83	-1,78	0,00301	NM_006672	RASGRP2
7949104	-0,62	-1,53	0,34134	-0,70	-1,62	0,02137	NM_001098671	---

7989249	-0,62	-1,53	0,12418	-1,00	-2,00	0,08028	---	ICOS
8047702	-0,62	-1,53	0,00261	-0,66	-1,58	0,02330	NM_012092	PCNXL3
7941364	-0,62	-1,53	0,02623	1,74	3,34	0,01324	NM_032223	---
7943193	-0,62	-1,53	0,08494	-0,94	-1,92	0,00059	---	REG4
7919067	-0,62	-1,53	0,10288	-0,91	-1,87	0,03727	NM_001159352	---
8127660	-0,62	-1,53	0,21693	-1,32	-2,49	0,00022	---	---
7918953	-0,62	-1,53	0,03321	-0,72	-1,65	0,00381	---	---
8056993	-0,62	-1,53	0,22146	-1,36	-2,58	0,00881	---	DNM1P35
7985030	-0,62	-1,53	0,09171	-1,47	-2,76	0,00481	ENST00000428357	WRAP53
8004556	-0,62	-1,53	0,00911	1,14	2,21	0,00946	NM_018081	KRTAP17-1
8015252	-0,62	-1,53	0,19495	-1,34	-2,53	0,03484	NM_031964	TPRXL
8077989	-0,62	-1,53	0,15752	-0,99	-1,98	0,02713	NR_002223	ANKRD20B
8053737	-0,62	-1,53	0,13501	0,30	1,23	0,39631	NR_003366	---
8034905	-0,62	-1,53	0,11958	-1,60	-3,02	0,02306	---	FAM120C
8173041	-0,62	-1,53	0,14708	0,86	1,81	0,14224	NM_017848	ATPIF1
7899424	-0,62	-1,53	0,28161	-0,61	-1,53	0,03696	NM_178191	C9orf27
8163666	-0,62	-1,53	0,05993	-1,48	-2,79	0,03692	AB021923	PCDHB10
8108724	-0,62	-1,53	0,20094	-1,32	-2,49	0,00678	NM_018930	C9orf68
8159919	-0,62	-1,53	0,05622	-0,60	-1,52	0,13003	NM_001039395	RNASEH2C
7949482	-0,62	-1,53	0,04881	0,78	1,72	0,12627	NM_032193	MKL1
8076241	-0,62	-1,53	0,04263	0,31	1,24	0,35106	NM_020831	CCDC153
7952200	-0,62	-1,53	0,15583	-0,29	-1,22	0,53990	NM_001145018	MLLT6
8014702	-0,62	-1,53	0,33717	-0,10	-1,07	0,86915	NM_005937	GPR82
8166919	-0,62	-1,53	0,05664	-0,39	-1,31	0,01727	NM_080817	RPL12
8164210	-0,62	-1,53	0,26795	-0,75	-1,68	0,03015	NM_000976	RPS4P21
8036029	-0,62	-1,53	0,08459	-0,70	-1,62	0,07511	ENST00000334591	NCAM1
7943892	-0,62	-1,53	0,04860	-1,14	-2,21	0,02970	NM_181351	GOLGA9P
7982230	-0,62	-1,53	0,21525	-0,76	-1,69	0,02165	NR_024074	---
8098157	-0,62	-1,53	0,00091	-0,34	-1,26	0,12420	---	SOD3
8094372	-0,62	-1,53	0,14686	-2,02	-4,06	0,00138	NM_003102	GABBR2
8162777	-0,62	-1,53	0,22789	-1,11	-2,16	0,00390	NM_005458	OR4C6
7939932	-0,62	-1,53	0,29371	-1,68	-3,21	0,05837	NM_001004704	---
7957159	-0,62	-1,53	0,05074	-1,33	-2,52	0,00407	---	TMEM86A
7938812	-0,62	-1,53	0,06757	-0,25	-1,19	0,41013	NM_153347	OR6C68
7956001	-0,62	-1,53	0,02208	-0,95	-1,93	0,03137	NM_001005519	FAM50B
8116658	-0,62	-1,53	0,25429	1,36	2,56	0,04190	NM_012135	GRAP2
8073194	-0,62	-1,53	0,13354	-0,78	-1,71	0,01238	NM_004810	MIR377
7976852	-0,62	-1,53	0,08258	-0,77	-1,70	0,02321	NR_029869	IGKC
8043438	-0,62	-1,53	0,67851	-2,10	-4,29	0,00003	BC093097	TRMT12
8148263	-0,62	-1,53	0,29545	0,77	1,71	0,12499	NM_017956	---
8082252	-0,62	-1,53	0,28483	-1,73	-3,33	0,00888	---	ZFP14
8036304	-0,62	-1,53	0,18829	-0,32	-1,24	0,28263	NM_020917	LCN2
8158167	-0,62	-1,53	0,21051	2,20	4,59	0,00463	NM_005564	MOV10L1
8073962	-0,62	-1,53	0,04878	-0,84	-1,79	0,03868	NM_018995	ANKFN1
8008609	-0,62	-1,53	0,10623	-0,09	-1,07	0,74454	NM_153228	---
8159957	-0,62	-1,53	0,46642	-1,32	-2,49	0,00167	---	HLA-DPB1
8179519	-0,62	-1,53	0,33550	1,59	3,02	0,00627	NM_002121	TARP
8139100	-0,62	-1,54	0,30299	-1,09	-2,13	0,00289	M30894	ADAM20
7979927	-0,62	-1,54	0,13377	-1,22	-2,33	0,01249	NM_003814	GPR6
8121454	-0,62	-1,54	0,40775	-1,25	-2,39	0,00668	NM_005284	---
8173671	-0,62	-1,54	0,10793	-0,38	-1,31	0,06159	---	---
7942983	-0,62	-1,54	0,23146	-0,84	-1,79	0,01416	---	OR2D3
7938181	-0,62	-1,54	0,13037	-1,13	-2,18	0,00679	NM_001004684	DNASE1L3
8088371	-0,62	-1,54	0,15040	-0,63	-1,55	0,06614	NM_004944	MMRN2
7934842	-0,62	-1,54	0,07921	-0,37	-1,30	0,11072	NM_024756	PDDC1
7945521	-0,62	-1,54	0,07330	0,51	1,42	0,36255	NM_182612	---
8111197	-0,62	-1,54	0,00656	-0,55	-1,46	0,03226	---	---
8042958	-0,62	-1,54	0,05258	-1,10	-2,14	0,01134	---	LOC96610
8071597	-0,62	-1,54	0,15922	-1,22	-2,32	0,00513	NR_027293	GCRG224
7942830	-0,62	-1,54	0,11905	-0,66	-1,58	0,01809	ENST00000391393	SLC7A13
8151637	-0,62	-1,54	0,00059	-0,88	-1,84	0,00996	NM_138817	CLDN22
8103877	-0,62	-1,54	0,05234	-0,94	-1,91	0,02741	NM_001111319	---
8041568	-0,62	-1,54	0,00706	-0,68	-1,60	0,04104	---	SMAD5OS
8114270	-0,62	-1,54	0,34079	-0,78	-1,72	0,01331	AF071111	RFPL4A
8031573	-0,62	-1,54	0,23556	-0,90	-1,86	0,00273	NM_001145014	---
8083164	-0,62	-1,54	0,20056	-1,02	-2,03	0,00042	---	BIRC8
8039078	-0,62	-1,54	0,01725	-0,97	-1,96	0,01564	NM_033341	---
8139907	-0,62	-1,54	0,11041	-0,84	-1,79	0,00907	---	C10orf85
7930994	-0,62	-1,54	0,07576	-0,53	-1,44	0,14701	BC108662	---
8067899	-0,62	-1,54	0,00017	-0,90	-1,86	0,00511	---	---
8131515	-0,62	-1,54	0,08605	-0,88	-1,84	0,00017	---	SEMA5B
8090044	-0,62	-1,54	0,14373	-0,98	-1,97	0,01706	NM_001031702	---
7922389	-0,62	-1,54	0,08383	-1,93	-3,80	0,00019	---	---

7927852	-0,62	-1,54	0,16531	-1,47	-2,76	0,00184	---	TDRD9	
7977161	-0,62	-1,54	0,03365	-0,77	-1,71	0,00728	NM_153046		C9orf131
8154973	-0,62	-1,54	0,18705	-1,39	-2,63	0,00468	NM_203299		---
7969202	-0,62	-1,54	0,00033	-1,01	-2,02	0,14102	---	---	
8040528	-0,62	-1,54	0,01133	-0,64	-1,55	0,00162	---	CXCL5	
8100977	-0,62	-1,54	0,18967	-1,04	-2,06	0,04034	NM_002994		SNORD83A
8076221	-0,62	-1,54	0,11565	-1,62	-3,07	0,00048	NR_000027		MCM4
8146357	-0,62	-1,54	0,40917	2,61	6,09	0,00000	NM_005914		OGG1
8077635	-0,62	-1,54	0,02529	0,78	1,71	0,03597	NM_016819		KIAA1239
8094542	-0,62	-1,54	0,18841	-0,62	-1,54	0,05691	NM_001144990		CXCR1
8058905	-0,62	-1,54	0,08319	-1,03	-2,04	0,02030	NM_000634		NHSL2
8168359	-0,62	-1,54	0,30108	-0,64	-1,56	0,10468	NM_001013627		STK11
8024204	-0,62	-1,54	0,01778	0,56	1,47	0,12554	NM_000455		MOCS1
8126214	-0,62	-1,54	0,26573	-0,26	-1,20	0,26441	NM_005943		---
8081030	-0,62	-1,54	0,29029	-1,21	-2,31	0,00150	---	---	
8099082	-0,62	-1,54	0,27708	-0,98	-1,98	0,03681	---	WDR16	
8004889	-0,62	-1,54	0,05857	-0,89	-1,86	0,02772	NM_145054		---
8110880	-0,62	-1,54	0,17919	-0,84	-1,79	0,01408	---	---	
8133002	-0,62	-1,54	0,17919	-0,84	-1,79	0,01408	---	TBC1D3F	
8014603	-0,62	-1,54	0,34131	1,24	2,35	0,06290	NM_032258		TRIM36
8113577	-0,62	-1,54	0,07909	-0,54	-1,45	0,23250	NM_018700		---
8121943	-0,62	-1,54	0,10384	-1,33	-2,52	0,00217	---	SLC22A18	
7937852	-0,62	-1,54	0,03594	0,25	1,19	0,50504	NM_002555		RGS22
8151999	-0,62	-1,54	0,02408	-1,33	-2,51	0,00696	NM_015668		ZNF79
8158022	-0,62	-1,54	0,16151	0,26	1,20	0,35543	NM_007135		FAM3B
8068684	-0,62	-1,54	0,05358	0,10	1,07	0,55740	NM_058186		---
8133200	-0,62	-1,54	0,06660	-1,38	-2,60	0,00670	---	FGF20	
8149465	-0,62	-1,54	0,05133	-0,27	-1,20	0,01386	NM_019851		FAM131A
8084478	-0,62	-1,54	0,06846	-0,08	-1,06	0,74895	NM_001171093		---
8083144	-0,62	-1,54	0,04378	-0,71	-1,64	0,01156	---	TRIM31	
8178330	-0,62	-1,54	0,17731	-1,00	-1,99	0,08119	NM_007028		---
8040576	-0,62	-1,54	0,05443	-0,49	-1,41	0,05630	---	CDK11A	
7911578	-0,62	-1,54	0,37349	-0,73	-1,66	0,28902	NM_024011		FBXL18
8137964	-0,62	-1,54	0,18238	-0,92	-1,90	0,10813	NM_024963		C9orf114
8164481	-0,62	-1,54	0,00954	1,45	2,73	0,08316	NM_016390		KCNH5
7979529	-0,62	-1,54	0,17265	-0,78	-1,71	0,01335	NM_139318		MS4A13
7940307	-0,62	-1,54	0,01625	-0,74	-1,68	0,02192	NM_001012417		---
8157841	-0,62	-1,54	0,06006	-1,49	-2,80	0,00618	---	TNF	
8118142	-0,62	-1,54	0,23541	-1,96	-3,89	0,00481	NM_000594		TNF
8177983	-0,62	-1,54	0,23541	-1,96	-3,89	0,00481	NM_000594		TNF
8179263	-0,62	-1,54	0,23541	-1,96	-3,89	0,00481	NM_000594		ITPR3
8118734	-0,62	-1,54	0,00592	4,38	20,89	0,00065	NM_002224		RPL23AP64
7952126	-0,62	-1,54	0,35578	-0,46	-1,37	0,07764	NR_003040		---
7916341	-0,62	-1,54	0,12768	-0,63	-1,55	0,01332	---	---	
7963201	-0,62	-1,54	0,01558	-0,64	-1,56	0,02263	---	MIR153-1	
8059211	-0,62	-1,54	0,02703	-0,48	-1,39	0,00576	NR_029688		ARAP2
8099760	-0,62	-1,54	0,03703	1,57	2,97	0,00259	NM_015230		EMR3
8034851	-0,62	-1,54	0,03639	-1,05	-2,07	0,00373	NM_032571		RNF208
8165492	-0,62	-1,54	0,11458	-0,08	-1,06	0,85852	NM_031297		RNF208
8165637	-0,62	-1,54	0,11458	-0,08	-1,06	0,85852	NM_031297		---
7976059	-0,62	-1,54	0,03286	-1,02	-2,03	0,02249	---	HGD	
8089851	-0,62	-1,54	0,21700	-0,59	-1,51	0,04791	NM_000187		HGD
8093278	-0,62	-1,54	0,21700	-0,59	-1,51	0,04791	NM_000187		PXDNL
8150704	-0,62	-1,54	0,15897	-1,04	-2,06	0,00651	NM_144651		TSHR
7976037	-0,62	-1,54	0,02148	-1,05	-2,07	0,00286	NM_000369		KRT16
8015376	-0,62	-1,54	0,17731	-0,95	-1,94	0,30661	NM_005557		RBM11
8067869	-0,62	-1,54	0,07337	0,95	1,93	0,15831	NM_144770		GAA
8010354	-0,62	-1,54	0,19437	1,30	2,46	0,13684	NM_000152		C21orf62
8070073	-0,62	-1,54	0,00382	-0,43	-1,35	0,00148	NM_001162495		TSEN54
8009832	-0,62	-1,54	0,00311	1,06	2,08	0,07954	NM_207346		PDGFA
8130997	-0,62	-1,54	0,31234	-1,70	-3,26	0,00512	BC109246	DMWD	
8037679	-0,62	-1,54	0,15292	0,60	1,51	0,19889	NM_004943		---
8020025	-0,62	-1,54	0,30216	-0,13	-1,09	0,61563	---	CACYBP	
7907537	-0,62	-1,54	0,13979	-0,25	-1,19	0,20673	NM_014412		---
8171032	-0,62	-1,54	0,09123	-1,43	-2,69	0,00503	---	---	
8176243	-0,62	-1,54	0,09123	-1,43	-2,69	0,00503	---	---	
8093037	-0,62	-1,54	0,04521	-1,33	-2,51	0,03614	---	MYOT	
8108256	-0,63	-1,54	0,02049	-0,90	-1,87	0,02776	NM_006790		C7orf57
8132715	-0,63	-1,54	0,16743	-1,15	-2,21	0,00241	NM_001100159		---
7911092	-0,63	-1,54	0,32094	-0,05	-1,04	0,90653	---	---	
8152863	-0,63	-1,54	0,16225	-1,07	-2,10	0,01170	---	CLDN15	
8141708	-0,63	-1,54	0,10609	-0,22	-1,17	0,64490	NM_014343		HKDC1
7927998	-0,63	-1,54	0,34368	0,34	1,27	0,33292	NM_025130		ZBED1

8171139	-0,63	-1,54	0,09265	0,19	1,14	0,64939	NM_001171136	ZBED1
8177029	-0,63	-1,54	0,09265	0,19	1,14	0,64939	NM_001171136	OMG
8014057	-0,63	-1,54	0,03947	-0,90	-1,87	0,00227	NM_002544	---
8047690	-0,63	-1,54	0,00359	-0,85	-1,80	0,00024	---	OSM
8075316	-0,63	-1,54	0,22912	-1,08	-2,11	0,01787	NM_020530	---
8088126	-0,63	-1,54	0,00820	-1,14	-2,20	0,00444	---	GJB2
7970441	-0,63	-1,54	0,06602	-0,39	-1,31	0,42701	NM_004004	DIRAS3
7916853	-0,63	-1,54	0,06417	-0,77	-1,71	0,09898	NM_004675	TMEM37
8044813	-0,63	-1,54	0,30667	-0,29	-1,23	0,07838	NM_183240	TRIM31
8124707	-0,63	-1,54	0,16987	-1,11	-2,16	0,05813	NM_007028	SPATA2
8066953	-0,63	-1,54	0,11675	1,83	3,55	0,00451	NM_006038	ZC3H12A
7900146	-0,63	-1,54	0,15027	-0,10	-1,07	0,56778	NM_025079	VSIG1
8169263	-0,63	-1,54	0,19848	-0,75	-1,68	0,21741	NM_001170553	RGL2
8125687	-0,63	-1,54	0,09068	0,90	1,87	0,20176	NM_004761	---
7934318	-0,63	-1,54	0,19565	-1,90	-3,72	0,00000	---	TMEM225
7952377	-0,63	-1,54	0,23852	-1,16	-2,23	0,00088	NM_001013743	---
8022102	-0,63	-1,54	0,01174	-0,73	-1,66	0,00045	---	EDN3
8063729	-0,63	-1,54	0,08417	-1,21	-2,31	0,00026	NM_207032	KCNG3
8051785	-0,63	-1,54	0,15737	0,00	-1,00	0,99847	NM_133329	RERG
7961540	-0,63	-1,54	0,02687	-0,67	-1,59	0,01772	NM_032918	---
8069610	-0,63	-1,54	0,32817	-1,06	-2,09	0,00723	---	ZNF30
8027692	-0,63	-1,54	0,04666	0,90	1,86	0,04908	NR_024018	TUBB8
7931674	-0,63	-1,54	0,17621	-1,41	-2,67	0,16009	NM_177987	C17orf50
8006569	-0,63	-1,55	0,12309	-0,73	-1,66	0,01172	NM_145272	PCDHB8
8108713	-0,63	-1,55	0,20774	-0,49	-1,40	0,38463	NM_019120	---
7994341	-0,63	-1,55	0,01472	-0,58	-1,49	0,07593	---	---
7989832	-0,63	-1,55	0,11457	-0,64	-1,56	0,00111	---	---
8046520	-0,63	-1,55	0,04753	-0,96	-1,95	0,05635	---	---
8146525	-0,63	-1,55	0,20472	-1,42	-2,68	0,00604	---	HFM1
7917612	-0,63	-1,55	0,01724	-0,75	-1,68	0,08142	NM_001017975	GOLGA6B
7984662	-0,63	-1,55	0,33486	-0,81	-1,75	0,04151	NM_018652	---
8008868	-0,63	-1,55	0,31761	-1,43	-2,69	0,03255	---	---
7948369	-0,63	-1,55	0,04840	-1,66	-3,16	0,01798	---	---
8121884	-0,63	-1,55	0,24547	-1,50	-2,82	0,01367	---	KRT2
7963479	-0,63	-1,55	0,01948	-0,33	-1,25	0,11171	NM_000423	DGCR6L
8074565	-0,63	-1,55	0,13204	0,98	1,97	0,08443	NM_033257	NECAB2
7997542	-0,63	-1,55	0,36924	-0,35	-1,27	0,28653	NM_019065	---
8104070	-0,63	-1,55	0,02723	-0,15	-1,11	0,41790	---	BMS1P4
7927550	-0,63	-1,55	0,00852	-0,86	-1,81	0,01585	NR_026592	FLJ42842
8008540	-0,63	-1,55	0,36401	-1,19	-2,28	0,12886	AK124832	---
8132845	-0,63	-1,55	0,10136	-1,36	-2,56	0,03725	---	---
7979802	-0,63	-1,55	0,02181	-1,17	-2,24	0,01236	---	RPRD1B
8062433	-0,63	-1,55	0,16675	1,27	2,41	0,08634	NM_021215	LRRTM3
7927803	-0,63	-1,55	0,16129	-0,98	-1,98	0,01129	NM_178011	SERPINA11
7981078	-0,63	-1,55	0,11575	-1,03	-2,04	0,01460	NM_001080451	---
8047337	-0,63	-1,55	0,21843	-0,99	-1,99	0,00805	---	---
8101585	-0,63	-1,55	0,08210	-1,01	-2,01	0,21574	---	TTC6
7974029	-0,63	-1,55	0,03701	-0,21	-1,16	0,36420	BC103915	TNKS1BP1
7948176	-0,63	-1,55	0,04647	-0,08	-1,06	0,72386	NM_033396	ANKRD20B
8053741	-0,63	-1,55	0,39905	-0,91	-1,88	0,08861	NR_003366	---
7985257	-0,63	-1,55	0,05287	-0,95	-1,93	0,01274	---	GADL1
8085972	-0,63	-1,55	0,00052	-1,15	-2,21	0,01344	NM_207359	LYPLA2
8033458	-0,63	-1,55	0,53925	0,76	1,70	0,18265	NM_007260	TYR
7942991	-0,63	-1,55	0,00293	-1,01	-2,02	0,02854	NM_000372	---
7902439	-0,63	-1,55	0,15979	-0,99	-1,99	0,01442	---	ADAMDEC1
8145317	-0,63	-1,55	0,09259	-0,76	-1,69	0,00033	NM_014479	---
7932788	-0,63	-1,55	0,15889	-0,92	-1,89	0,04979	---	ESRRA
7940989	-0,63	-1,55	0,21673	-1,24	-2,36	0,12334	NM_004451	SNORD114-2
7976814	-0,63	-1,55	0,16869	-0,94	-1,92	0,00511	NR_003194	THAP5
8142324	-0,63	-1,55	0,38420	-0,24	-1,18	0,71638	NM_001130475	HSD17B13
8101637	-0,63	-1,55	0,13301	-0,86	-1,81	0,01326	NM_178135	GCK
8139314	-0,63	-1,55	0,12098	-0,73	-1,66	0,04267	NM_033508	HLA-DRA
8178193	-0,63	-1,55	0,04701	1,56	2,95	0,02184	NM_019111	GCDH
8026090	-0,63	-1,55	0,09228	0,27	1,21	0,41126	NM_000159	DKFZP434I0714
8097811	-0,63	-1,55	0,09186	-1,32	-2,49	0,01439	AL137273	LYZL6
8014334	-0,63	-1,55	0,18908	-1,08	-2,11	0,00212	NM_020426	KLHL26
8027049	-0,63	-1,55	0,04992	0,43	1,35	0,12444	NM_018316	OSCAR
8039120	-0,63	-1,55	0,07786	-1,46	-2,76	0,01294	NM_206818	ARHGEF5
8143610	-0,63	-1,55	0,06819	1,64	3,12	0,00269	NM_005435	TEKT2
7900076	-0,63	-1,55	0,02262	-0,90	-1,86	0,02345	NM_014466	ZNF434
7992895	-0,63	-1,55	0,01682	-1,33	-2,51	0,00480	NM_017810	---
7945156	-0,63	-1,55	0,21468	-0,79	-1,73	0,00371	---	CLIP2
8133459	-0,63	-1,55	0,06585	-0,59	-1,51	0,05127	NM_003388	CCDC60

7959108	-0,63	-1,55	0,08866	-0,67	-1,59	0,03477	NM_178499	DSCAML1
7951928	-0,63	-1,55	0,13810	-0,93	-1,91	0,01574	NM_020693	---
8152819	-0,63	-1,55	0,04450	-0,61	-1,52	0,04638	---	ASRGL1
7940643	-0,63	-1,55	0,25637	1,08	2,12	0,00339	NM_001083926	---
8113342	-0,63	-1,55	0,03423	-1,01	-2,01	0,00544	---	hCG_1659830
8023596	-0,63	-1,55	0,19532	-0,58	-1,50	0,13331	ENST00000426779	---
7967868	-0,63	-1,55	0,03621	-0,72	-1,65	0,02702	---	OR12D2
8117724	-0,63	-1,55	0,25336	-0,88	-1,84	0,04995	NM_013936	NMS
8043890	-0,63	-1,55	0,19897	-0,77	-1,70	0,09252	NM_001011717	IFNGR1
8122261	-0,63	-1,55	0,13783	-1,63	-3,10	0,01229	NM_000416	---
8053574	-0,63	-1,55	0,06201	-1,16	-2,24	0,08740	---	---
8036993	-0,63	-1,55	0,18804	-1,65	-3,13	0,00333	---	AMDHD1
7957654	-0,63	-1,55	0,05668	-0,39	-1,31	0,35420	NM_152435	OR4F15
7986532	-0,63	-1,55	0,24881	-1,24	-2,37	0,00038	NM_001001674	---
8169361	-0,63	-1,55	0,22375	-0,78	-1,71	0,07734	---	SNRPN
7981945	-0,63	-1,55	0,06476	-0,62	-1,54	0,03670	AB061718	CTXN3
8107764	-0,63	-1,55	0,17585	-0,76	-1,70	0,08955	NM_001048252	TMEM158
8086530	-0,63	-1,55	0,11902	-1,45	-2,74	0,00551	NM_015444	CXorf41
8169221	-0,63	-1,55	0,01248	-0,56	-1,48	0,00032	NM_001169154	RASGRF1
7990774	-0,63	-1,55	0,04858	-0,88	-1,84	0,00221	NM_002891	MIR516B2
8031031	-0,63	-1,55	0,04440	-0,94	-1,92	0,00541	NR_030207	---
7966688	-0,63	-1,55	0,24230	-1,19	-2,28	0,04442	---	GOLGA8G
7982131	-0,63	-1,55	0,15449	-0,67	-1,59	0,23928	NR_033353	GOLGA8G
7986922	-0,63	-1,55	0,15449	-0,67	-1,59	0,23928	NR_033353	---
8115828	-0,63	-1,55	0,00667	-0,97	-1,95	0,03799	---	---
8167038	-0,63	-1,55	0,06435	-1,56	-2,94	0,01454	---	---
7968913	-0,63	-1,55	0,21609	-0,63	-1,55	0,24312	---	OR2M7
7925745	-0,63	-1,55	0,07066	-1,01	-2,02	0,01462	NM_001004691	---
8020971	-0,63	-1,55	0,08781	-0,77	-1,70	0,08291	---	ZNF513
8051113	-0,63	-1,55	0,15705	-0,39	-1,31	0,42399	NM_144631	LOC253724
7965935	-0,63	-1,55	0,19396	-0,70	-1,62	0,04489	NR_027249	ITGB2
8070826	-0,63	-1,55	0,18776	0,67	1,59	0,09441	NM_000211	RGR
7928789	-0,63	-1,55	0,08670	-1,32	-2,49	0,01143	NM_002921	---
7994824	-0,63	-1,55	0,50357	-0,65	-1,57	0,04361	---	---
8056790	-0,63	-1,55	0,13770	-1,83	-3,56	0,01839	---	KLB
8094679	-0,63	-1,55	0,08173	1,27	2,41	0,12681	NM_175737	PDZD7
7935834	-0,63	-1,55	0,28865	-1,45	-2,74	0,00377	NM_024895	BGLAP
7906140	-0,63	-1,55	0,34251	1,96	3,89	0,00424	NM_199173	NEU2
8049243	-0,63	-1,55	0,13905	-1,79	-3,46	0,00068	NM_005383	GNG2
7979131	-0,63	-1,55	0,16445	-0,73	-1,66	0,12302	NM_053064	DAPL1
8045882	-0,63	-1,55	0,08723	-0,94	-1,92	0,06951	NM_001017920	GOLGA6B
7984961	-0,63	-1,55	0,37323	-0,93	-1,90	0,00298	NM_018652	LRRN4CL
7948794	-0,63	-1,55	0,19172	-2,25	-4,77	0,01009	NM_203422	TPRG1
8084757	-0,63	-1,55	0,11804	0,44	1,36	0,28521	NM_198485	RHOT2
7991989	-0,63	-1,55	0,03218	0,11	1,08	0,75197	NM_138769	TCEAL2
8168892	-0,64	-1,55	0,08455	-0,84	-1,79	0,11789	NM_080390	WFIKKN2
8008511	-0,64	-1,55	0,31140	-1,68	-3,21	0,02425	NM_175575	MAGEA11
8175629	-0,64	-1,55	0,20612	-1,07	-2,10	0,04582	NM_001011544	TAS2R5
8136647	-0,64	-1,55	0,03303	-0,08	-1,06	0,65597	NM_018980	---
8055909	-0,64	-1,55	0,39332	-1,46	-2,75	0,01192	---	PLA2G3
8075468	-0,64	-1,55	0,18796	-0,49	-1,40	0,28039	NM_015715	GAS2
7938989	-0,64	-1,55	0,08333	-1,34	-2,52	0,00096	NM_005256	CTAGE4
8136979	-0,64	-1,55	0,14587	-0,36	-1,29	0,00791	NM_198495	SPATA4
8103802	-0,64	-1,55	0,01043	-0,80	-1,74	0,03674	NM_144644	SNORA2B
7962829	-0,64	-1,55	0,13447	-0,66	-1,58	0,05891	NR_002951	OR4C5
7948042	-0,64	-1,55	0,16928	-0,98	-1,98	0,00124	ENST00000319813	C10orf72
7933488	-0,64	-1,55	0,07909	-0,93	-1,91	0,12433	NM_001031746	TMEM160
8037853	-0,64	-1,55	0,26347	-0,13	-1,09	0,71082	NM_017854	COL4A4
8059477	-0,64	-1,55	0,07101	-1,25	-2,38	0,00118	NM_000092	IL2RA
7931914	-0,64	-1,55	0,13310	-1,52	-2,86	0,00045	NM_000417	SLC2A2
8092083	-0,64	-1,55	0,17174	-0,41	-1,33	0,01208	NM_000340	OR5H14
8081196	-0,64	-1,55	0,12370	-0,94	-1,92	0,00030	NM_001005514	LGI1
7929373	-0,64	-1,55	0,03353	-1,37	-2,59	0,00571	NM_005097	SPRR2G
7920217	-0,64	-1,55	0,53675	-2,75	-6,73	0,00126	NM_001014291	---
8104648	-0,64	-1,55	0,06639	-1,28	-2,44	0,00235	---	ADAP2
8006214	-0,64	-1,56	0,08209	0,77	1,71	0,26460	NM_018404	---
7923905	-0,64	-1,56	0,03030	-1,66	-3,17	0,01053	---	FAM58A
8175811	-0,64	-1,56	0,21224	-1,37	-2,58	0,04812	NM_152274	---
8042694	-0,64	-1,56	0,00236	-1,54	-2,90	0,01091	---	LRRC19
8160504	-0,64	-1,56	0,01641	-1,15	-2,23	0,00055	NM_022901	---
7991182	-0,64	-1,56	0,01303	-0,14	-1,10	0,09471	---	DNHD1
7938133	-0,64	-1,56	0,13508	-0,30	-1,23	0,33556	NM_144666	OR5A1
7940173	-0,64	-1,56	0,12668	-2,37	-5,15	0,00948	NM_001004728	ANKRD20A1

8155574	-0,64	-1,56	0,29209	-0,62	-1,54	0,03100	NM_032250	---
7958015	-0,64	-1,56	0,19152	-1,66	-3,17	0,00301	---	CXCL13
8095886	-0,64	-1,56	0,04783	-0,78	-1,72	0,00272	NM_006419	CCDC144C
8005679	-0,64	-1,56	0,36217	0,53	1,44	0,19734	NR_023380	BAT4
8124998	-0,64	-1,56	0,21496	-0,16	-1,12	0,70213	NM_033177	SLC25A25
8158147	-0,64	-1,56	0,10260	0,15	1,11	0,72224	NM_052901	C10orf2
7929901	-0,64	-1,56	0,04421	1,74	3,34	0,00563	NM_001163812	LCN8
8165285	-0,64	-1,56	0,20343	-1,24	-2,36	0,01811	NM_178469	---
8151784	-0,64	-1,56	0,13195	-0,73	-1,66	0,00854	---	SOHLH1
8165038	-0,64	-1,56	0,10101	-0,65	-1,57	0,00806	NM_001012415	CGA
8128001	-0,64	-1,56	0,24602	-0,90	-1,87	0,08949	NM_000735	HCG9
8117809	-0,64	-1,56	0,01919	-1,09	-2,13	0,00081	NR_028032	HCG9
8177740	-0,64	-1,56	0,01919	-1,09	-2,13	0,00081	NR_028032	FAM19A4
8088671	-0,64	-1,56	0,04295	-1,30	-2,47	0,00506	NM_182522	TOR1AIP1
7922648	-0,64	-1,56	0,14807	-0,98	-1,97	0,00554	NM_015602	FCER1G
7906720	-0,64	-1,56	0,10255	0,00	1,00	0,99633	NM_004106	DDRGK1
8064601	-0,64	-1,56	0,17026	0,54	1,46	0,02344	NM_023935	C17orf68
8012423	-0,64	-1,56	0,05338	0,60	1,51	0,21986	NM_025099	TMPRSS2
8070467	-0,64	-1,56	0,00096	-0,46	-1,37	0,08080	NM_001135099	STON1
8041937	-0,64	-1,56	0,07588	-0,69	-1,61	0,04436	ENST00000406226	RRH
8096830	-0,64	-1,56	0,02259	-0,55	-1,46	0,00198	NM_006583	FLJ45964
8049667	-0,64	-1,56	0,22968	-0,15	-1,11	0,02755	ENST00000358775	ETV3
7921228	-0,64	-1,56	0,00566	0,10	1,07	0,89168	NM_001145312	---
7966876	-0,64	-1,56	0,05565	-1,02	-2,02	0,00651	---	---
7926819	-0,64	-1,56	0,06188	-1,48	-2,79	0,00017	---	---
8162084	-0,64	-1,56	0,29521	-0,58	-1,50	0,05212	---	MSX2P1
8008716	-0,64	-1,56	0,20403	-0,93	-1,91	0,14325	NR_002307	MMP26
7937961	-0,64	-1,56	0,07376	-0,52	-1,44	0,06747	NM_021801	PRO1768
7976148	-0,64	-1,56	0,13167	-1,20	-2,29	0,04994	NR_024620	---
8065386	-0,64	-1,56	0,24072	-0,91	-1,88	0,00537	---	CRYBB3
8071989	-0,64	-1,56	0,06558	-1,64	-3,11	0,00004	NM_004076	SEMG2
8062944	-0,64	-1,56	0,09245	-1,16	-2,23	0,01747	NM_003008	---
8151430	-0,64	-1,56	0,19260	-1,69	-3,23	0,00208	---	KRT24
8015060	-0,64	-1,56	0,29605	-1,37	-2,59	0,01547	NM_019016	SYTL1
7899323	-0,64	-1,56	0,14833	-0,54	-1,45	0,23375	NM_032872	---
7991564	-0,64	-1,56	0,06458	-0,75	-1,68	0,01768	---	---
7982335	-0,64	-1,56	0,26546	-1,68	-3,21	0,01057	---	---
8146353	-0,64	-1,56	0,00043	-0,69	-1,61	0,00331	---	C12orf42
7965900	-0,64	-1,56	0,07778	-1,83	-3,57	0,01975	NM_001099336	PIGR
7923929	-0,64	-1,56	0,13489	-1,29	-2,45	0,00029	NM_002644	ERN2
8000346	-0,64	-1,56	0,14437	-1,62	-3,07	0,00076	NM_033266	TCEB3CL
8023154	-0,64	-1,56	0,52111	-2,14	-4,41	0,00246	NM_001100817	CLCA4
7902738	-0,64	-1,56	0,10829	-1,06	-2,08	0,00344	NM_012128	C6orf154
8126566	-0,64	-1,56	0,31209	-1,35	-2,54	0,02675	BC108661	PRAMEF16
7898026	-0,64	-1,56	0,13075	-1,53	-2,89	0,01384	NM_001045480	KIF19
8018164	-0,64	-1,56	0,31643	-0,76	-1,70	0,09612	BC093631	---
8110112	-0,64	-1,56	0,07550	-0,78	-1,72	0,00118	---	AK7
7976578	-0,64	-1,56	0,02833	-0,06	-1,04	0,63259	NM_152327	AGAP11
7928890	-0,64	-1,56	0,04579	-0,26	-1,20	0,36623	NM_133447	C11orf35
7937404	-0,64	-1,56	0,03683	-0,21	-1,16	0,31565	NM_173573	CRH
8151092	-0,64	-1,56	0,32135	-1,31	-2,47	0,03352	NM_000756	TSSK2
8071196	-0,64	-1,56	0,13898	-0,71	-1,63	0,00602	NM_053006	PPM1J
7918606	-0,64	-1,56	0,13507	0,33	1,26	0,30377	NM_005167	C18orf26
8021297	-0,64	-1,56	0,13024	-1,34	-2,53	0,03954	NM_173629	---
8173579	-0,64	-1,56	0,21185	-0,75	-1,68	0,11977	---	ZPBP2
8006958	-0,64	-1,56	0,12177	-0,92	-1,89	0,00391	NM_199321	SH3GL3
7985507	-0,64	-1,56	0,06325	-0,97	-1,96	0,00061	NR_026799	GGT3P
8074716	-0,64	-1,56	0,05938	-1,33	-2,51	0,04255	NR_003267	BEGAIN
7981309	-0,64	-1,56	0,19157	-0,26	-1,19	0,14738	NM_020836	C3orf46
8082298	-0,64	-1,56	0,11453	0,19	1,14	0,51968	BC042038	SCARNA17
8021183	-0,64	-1,56	0,04976	0,22	1,17	0,00014	NR_003003	DHRS9
8046124	-0,64	-1,56	0,04286	-0,76	-1,69	0,08629	NM_005771	---
7897683	-0,64	-1,56	0,06141	-0,39	-1,31	0,00804	---	ENO3
8004043	-0,64	-1,56	0,16274	-0,34	-1,27	0,12107	NM_001976	GALNT6
7963313	-0,64	-1,56	0,04725	0,32	1,25	0,24754	NM_007210	FBXO41
8053009	-0,64	-1,56	0,08146	-0,73	-1,66	0,03971	NM_001080410	ZMIZ2
8132617	-0,64	-1,56	0,12165	1,00	2,00	0,05386	NM_031449	MSR1
8149448	-0,64	-1,56	0,12578	-0,85	-1,80	0,03464	NM_002445	HHATL
8086419	-0,64	-1,56	0,04305	-1,21	-2,31	0,08768	NR_027753	---
8119856	-0,64	-1,56	0,02987	-0,65	-1,57	0,00967	---	CSN1S2A
8095441	-0,64	-1,56	0,10955	-0,50	-1,42	0,04138	NR_003720	MRAP2
8120961	-0,64	-1,56	0,18234	-0,58	-1,49	0,01909	NM_138409	C13orf36
7968650	-0,64	-1,56	0,04806	-1,51	-2,84	0,00086	NM_203451	RHAG

8126878	-0,64	-1,56	0,19768	-0,86	-1,82	0,13411	NM_000324	WDR69
8048870	-0,64	-1,56	0,13613	-0,33	-1,25	0,02581	NM_178821	OR5W2
7948107	-0,64	-1,56	0,05731	-1,54	-2,90	0,00410	NM_001001960	---
8107472	-0,64	-1,56	0,12290	-1,47	-2,77	0,00633	---	CNGA3
8043782	-0,64	-1,56	0,32676	-0,81	-1,75	0,00182	NM_001298	C3orf57
8091799	-0,64	-1,56	0,03462	-0,49	-1,41	0,25128	NM_001040100	JOSD2
8038571	-0,64	-1,56	0,10659	-0,83	-1,77	0,03687	NM_138334	SRD5A2
8051361	-0,64	-1,56	0,08196	-1,53	-2,88	0,09356	NM_000348	---
7939961	-0,64	-1,56	0,14714	-1,00	-2,00	0,02382	---	LOC554174
8076744	-0,64	-1,56	0,12375	-0,86	-1,82	0,00628	BC009388	COLQ
8085581	-0,64	-1,56	0,03342	-0,56	-1,48	0,21915	NM_005677	---
8035080	-0,64	-1,56	0,03201	-1,32	-2,49	0,04711	---	C6orf118
8130664	-0,64	-1,56	0,02265	-0,71	-1,64	0,01104	NM_144980	PIK3C2G
7954208	-0,64	-1,56	0,02175	-0,44	-1,36	0,05454	NM_004570	CWH43
8094988	-0,64	-1,56	0,20220	-0,74	-1,67	0,00996	NM_025087	---
7940026	-0,65	-1,56	0,07629	-1,32	-2,49	0,01408	---	SMCR5
8013131	-0,65	-1,56	0,07596	-1,41	-2,65	0,01890	NR_024007	---
7973082	-0,65	-1,56	0,49669	-0,90	-1,87	0,04984	---	RASSF7
7937407	-0,65	-1,56	0,30563	-1,03	-2,04	0,07869	NM_003475	RXFP1
8098060	-0,65	-1,56	0,40899	-0,83	-1,78	0,00007	NM_021634	MICALL1
8072962	-0,65	-1,56	0,06377	0,42	1,34	0,24983	NM_033386	---
8162311	-0,65	-1,56	0,06724	-1,59	-3,02	0,01711	---	ADCK5
8148850	-0,65	-1,56	0,03625	-0,23	-1,17	0,60493	NM_174922	C10orf25
7933209	-0,65	-1,56	0,10894	0,16	1,12	0,26106	NM_001039380	---
8127542	-0,65	-1,56	0,50839	-0,92	-1,90	0,00467	---	PRG2
7948221	-0,65	-1,57	0,11465	-1,06	-2,08	0,00055	NM_002728	---
8116992	-0,65	-1,57	0,01377	-0,83	-1,78	0,24750	---	OR4D1
8008711	-0,65	-1,57	0,04835	-1,37	-2,58	0,04779	NM_012374	KCNA5
7953278	-0,65	-1,57	0,00039	-1,48	-2,79	0,06111	NM_002234	TSGA10IP
7941469	-0,65	-1,57	0,20626	-1,12	-2,17	0,00476	NM_152762	ABCB8
8137334	-0,65	-1,57	0,32945	-0,97	-1,95	0,04194	NM_007188	---
8137046	-0,65	-1,57	0,33557	-0,84	-1,79	0,00494	---	SLC27A2
7983650	-0,65	-1,57	0,17200	4,81	28,12	0,00003	NM_003645	OR51T1
7937979	-0,65	-1,57	0,08132	-0,50	-1,41	0,01249	NM_001004759	---
8004859	-0,65	-1,57	0,04716	-0,57	-1,49	0,00481	---	LOC100125556
8082254	-0,65	-1,57	0,12210	0,07	1,05	0,80114	NR_024251	---
8170117	-0,65	-1,57	0,01237	-0,30	-1,23	0,02390	---	PARP15
8082086	-0,65	-1,57	0,00084	-0,94	-1,92	0,00556	NM_001113523	---
7985999	-0,65	-1,57	0,41422	-1,31	-2,48	0,00309	---	UIMC1
7911343	-0,65	-1,57	0,48925	-4,14	-17,61	0,00382	AF284753	UIMC1
8165703	-0,65	-1,57	0,48925	-4,14	-17,61	0,00382	AF284753	WDR52
8089596	-0,65	-1,57	0,17648	-0,43	-1,35	0,36350	NM_001164496	LZTR1
8071466	-0,65	-1,57	0,21325	1,30	2,45	0,01100	NM_006767	---
8148018	-0,65	-1,57	0,11675	-1,23	-2,34	0,00072	---	FLJ25996
8084923	-0,65	-1,57	0,03788	-2,22	-4,65	0,00001	BC151150	---
8098261	-0,65	-1,57	0,12447	-0,71	-1,64	0,05590	---	TAS2R43 // TAS2R43
7961295	-0,65	-1,57	0,04838	-1,01	-2,02	0,01283	NM_176884 // NM_176884	LOC100130269
8072603	-0,65	-1,57	0,09128	-0,21	-1,16	0,02213	ENST00000414283	FOLR1
7942332	-0,65	-1,57	0,14168	0,11	1,08	0,65750	NM_016724	KCNJ8
7961702	-0,65	-1,57	0,19809	-0,74	-1,67	0,00588	NM_004982	---
8073293	-0,65	-1,57	0,22869	-0,48	-1,40	0,00461	---	MIR23A
8034698	-0,65	-1,57	0,01464	-0,81	-1,76	0,06429	NR_029495	RDH16
7964292	-0,65	-1,57	0,02026	-1,34	-2,54	0,03056	NM_003708	THRSP
7942793	-0,65	-1,57	0,15332	-1,02	-2,02	0,00087	NM_003251	MYH2
8012753	-0,65	-1,57	0,00670	-0,95	-1,93	0,00206	NM_017534	RGS13
7908397	-0,65	-1,57	0,01203	-0,92	-1,89	0,02527	NM_002927	---
8149809	-0,65	-1,57	0,34247	-0,59	-1,50	0,07668	---	---
7921431	-0,65	-1,57	0,00987	-0,92	-1,90	0,09447	---	MDGA2
7978801	-0,65	-1,57	0,00934	-0,32	-1,25	0,32550	NM_001113498	CTNND2
8110990	-0,65	-1,57	0,15489	-1,61	-3,05	0,04509	NM_001332	OR6N1
7921427	-0,65	-1,57	0,06037	-1,24	-2,37	0,01313	NM_001005185	---
7908728	-0,65	-1,57	0,07372	-0,81	-1,75	0,02268	---	FLJ45340
8135931	-0,65	-1,57	0,40966	-0,62	-1,54	0,14744	NR_024368	---
8079731	-0,65	-1,57	0,02631	-1,67	-3,18	0,01327	---	ETV7
8125993	-0,65	-1,57	0,14022	-0,41	-1,33	0,13634	NM_016135	FBRS
7994874	-0,65	-1,57	0,17419	-0,50	-1,41	0,07025	NM_001105079	BRMS1
7949603	-0,65	-1,57	0,25788	0,22	1,16	0,48103	NM_015399	CKMT1A
7983239	-0,65	-1,57	0,09079	0,52	1,43	0,11282	NM_001015001	CKMT1A
7983256	-0,65	-1,57	0,09079	0,52	1,43	0,11282	NM_001015001	SLC25A14
8169904	-0,65	-1,57	0,11504	-0,26	-1,19	0,25855	NM_003951	LOC285735
8122146	-0,65	-1,57	0,40561	-1,35	-2,54	0,01062	NR_026969	THAP9
8096091	-0,65	-1,57	0,01424	0,33	1,26	0,36881	NM_024672	TARS2

7905198	-0,65	-1,57	0,11389	1,05	2,06	0,12447	NM_025150	ANK1
8150439	-0,65	-1,57	0,03723	-0,74	-1,68	0,03651	NM_020476	EPHB4
8141625	-0,65	-1,57	0,30695	2,30	4,93	0,00734	NM_004444	SYT9
7938213	-0,65	-1,57	0,27978	-1,16	-2,24	0,00333	NM_175733	PLXDC1
8014768	-0,65	-1,57	0,09583	0,22	1,16	0,42931	NM_020405	CEND1
7945536	-0,65	-1,57	0,07248	-1,25	-2,38	0,00050	NM_016564	---
7927773	-0,65	-1,57	0,06476	-0,92	-1,89	0,01673	---	---
8170228	-0,65	-1,57	0,14221	-0,91	-1,88	0,03672	---	---
7945829	-0,65	-1,57	0,00334	-0,80	-1,74	0,04342	---	C8orf37
8151898	-0,65	-1,57	0,17235	2,12	4,35	0,00381	NM_177965	LOC283392
7964976	-0,65	-1,57	0,13522	-0,94	-1,92	0,08752	NR_026837	MAP3K6
7914042	-0,65	-1,57	0,12702	0,40	1,32	0,38797	NM_004672	CLLU1
7957508	-0,65	-1,57	0,00172	-1,16	-2,23	0,04768	NM_001025233	S100A13
7920303	-0,65	-1,57	0,04568	0,17	1,13	0,68607	NM_005979	C3orf67
8088436	-0,65	-1,57	0,11612	0,68	1,61	0,15948	NM_198463	HDAC4
8060030	-0,65	-1,57	0,03132	0,31	1,24	0,53405	NM_006037	---
8045028	-0,65	-1,57	0,02733	-0,81	-1,76	0,01166	---	FLJ30403
8038874	-0,65	-1,57	0,21360	-0,76	-1,70	0,01977	ENST00000301439	AHSG
8084648	-0,65	-1,57	0,11838	-1,13	-2,19	0,04253	NM_001622	PLB1
8041061	-0,65	-1,57	0,03402	-1,18	-2,26	0,00336	NM_153021	STBD1
8095826	-0,65	-1,57	0,34906	-0,45	-1,36	0,02708	NM_003943	---
7930376	-0,65	-1,57	0,06991	-0,31	-1,24	0,30315	---	LAMB3
7924029	-0,65	-1,57	0,05525	2,88	7,34	0,00226	NM_001017402	ICOSLG
8070720	-0,65	-1,57	0,09881	0,00	-1,00	0,98442	NM_015259	---
8133750	-0,65	-1,57	0,09280	-1,56	-2,94	0,00151	---	---
8135160	-0,65	-1,57	0,09280	-1,56	-2,94	0,00151	---	---
8139901	-0,65	-1,57	0,09280	-1,56	-2,94	0,00151	---	IGFL3
8037755	-0,65	-1,57	0,24785	-1,15	-2,23	0,00799	NM_207393	ZNF185
8170602	-0,65	-1,57	0,06014	1,26	2,39	0,01459	NM_007150	LEFTY1
7924663	-0,65	-1,57	0,17319	-0,64	-1,56	0,09031	NM_020997	NRN1L
7996715	-0,65	-1,57	0,24709	-0,56	-1,47	0,08887	NM_198443	WIPF2
8007058	-0,65	-1,57	0,16237	1,22	2,34	0,08276	NM_133264	IQGAP3
7921033	-0,65	-1,57	0,00116	1,40	2,63	0,00459	NM_178229	LOC401286
8130803	-0,65	-1,57	0,07196	-0,97	-1,96	0,06633	AK127120	LOC401286
8130976	-0,65	-1,57	0,07196	-0,97	-1,96	0,06633	AK127120	TNFRSF10A
8149762	-0,65	-1,57	0,14566	1,05	2,07	0,04848	NM_003844	---
8058514	-0,65	-1,57	0,12730	-0,79	-1,73	0,06139	---	SLC22A1
8123232	-0,65	-1,57	0,02817	-0,92	-1,89	0,14100	NM_003057	---
7921471	-0,65	-1,57	0,00691	-0,45	-1,37	0,00571	---	---
8163426	-0,65	-1,57	0,01449	-0,47	-1,39	0,03542	---	---
8053718	-0,65	-1,57	0,20649	-1,48	-2,79	0,14682	---	FAM71E2
8039477	-0,65	-1,57	0,06689	-1,14	-2,21	0,07077	BC031875	PYDC2
8084832	-0,65	-1,57	0,17723	-1,23	-2,35	0,14197	NM_001083308	---
7956269	-0,65	-1,57	0,04468	-1,03	-2,04	0,09963	---	POLR2C
7996051	-0,65	-1,57	0,41824	1,98	3,96	0,00073	NM_032940	GRM5
7950921	-0,65	-1,57	0,09418	-1,27	-2,41	0,00008	NM_001143831	HIST1H4F
8117422	-0,65	-1,57	0,12426	-0,12	-1,08	0,48816	NM_003540	HIST1H4B
8124385	-0,65	-1,57	0,11406	1,71	3,26	0,00007	NM_003544	KRT76
7963513	-0,65	-1,57	0,15357	-0,98	-1,97	0,04629	NM_015848	OR6Y1
7921360	-0,65	-1,57	0,04180	-1,60	-3,02	0,01482	NM_001005189	C16orf86
7996651	-0,65	-1,57	0,16637	-0,30	-1,23	0,16488	NM_001012984	IYD
8122744	-0,65	-1,57	0,10957	-0,83	-1,78	0,10073	NM_001164694	RAB9BP1
8107200	-0,65	-1,57	0,11953	-0,72	-1,65	0,00339	NR_000039	SNORD13P2
8131263	-0,65	-1,57	0,18182	-0,23	-1,17	0,33512	X58060	CXorf48
8175311	-0,65	-1,57	0,21527	-1,70	-3,24	0,07018	NM_001031705	---
8170208	-0,65	-1,57	0,07535	-1,51	-2,85	0,00929	---	LOC150197
8071355	-0,65	-1,57	0,19915	-1,63	-3,09	0,00283	NR_026919	GDPD5
7950501	-0,65	-1,57	0,08608	-0,53	-1,45	0,07793	NM_030792	---
8167910	-0,65	-1,57	0,26616	-0,86	-1,81	0,11357	---	HAO1
8064928	-0,65	-1,57	0,05159	-0,65	-1,57	0,00453	NM_017545	ACTRT1
8174979	-0,65	-1,57	0,06284	-1,55	-2,92	0,00211	NM_138289	TAS2R38
8143482	-0,65	-1,57	0,28743	-1,10	-2,14	0,00310	NM_176817	---
8177174	-0,65	-1,57	0,00046	-0,99	-1,99	0,02942	---	CALR3
8035146	-0,65	-1,57	0,18670	-1,17	-2,25	0,04021	NM_145046	IL20
7909261	-0,65	-1,57	0,10525	-1,33	-2,51	0,00174	NM_018724	OBP2A
8159259	-0,65	-1,57	0,32297	-1,92	-3,79	0,00431	NM_014582	NBEAL2
8079462	-0,65	-1,57	0,00783	1,92	3,80	0,00855	NM_015175	DNHD1
7938128	-0,65	-1,57	0,05902	0,19	1,14	0,64448	NM_144666	RP11-114H20.1
8167690	-0,65	-1,57	0,26577	-2,00	-3,99	0,00653	ENST00000375625	RP11-114H20.1
8172719	-0,65	-1,57	0,26577	-2,00	-3,99	0,00653	ENST00000375625	---
7914665	-0,65	-1,57	0,30565	-0,83	-1,77	0,03059	---	DEFB106A
8144452	-0,65	-1,57	0,15230	-0,30	-1,23	0,06393	NM_152251	DEFB106A
8149195	-0,65	-1,57	0,15230	-0,30	-1,23	0,06393	NM_152251	DEPDC4

7965760	-0,65	-1,57	0,19185	0,76	1,70	0,16636	NM_152317	KBTBD10
8046160	-0,65	-1,57	0,05878	-1,31	-2,48	0,04331	NM_006063	FAM106A
8013268	-0,65	-1,57	0,24236	-0,85	-1,81	0,06990	NR_026809	---
8102858	-0,66	-1,57	0,05790	-0,41	-1,33	0,11493	---	TMEM201
7897469	-0,66	-1,57	0,02547	0,57	1,49	0,25424	NM_001130924	MIR26A2
7964592	-0,66	-1,57	0,11105	-0,51	-1,42	0,21318	NR_029847	KISS1
7923621	-0,66	-1,57	0,15102	-1,43	-2,69	0,12632	NM_002256	THBD
8065353	-0,66	-1,57	0,26433	-1,08	-2,11	0,03367	NM_000361	BDH1
8093219	-0,66	-1,58	0,14339	-0,10	-1,07	0,80282	NM_203314	---
8147303	-0,66	-1,58	0,10151	0,15	1,11	0,31150	---	---
8101716	-0,66	-1,58	0,04943	-1,30	-2,46	0,00467	---	SLC5A10
8013309	-0,66	-1,58	0,09039	-0,95	-1,93	0,05231	NM_152351	C7orf55
8136535	-0,66	-1,58	0,00664	-0,77	-1,70	0,00898	NM_197964	---
8073343	-0,66	-1,58	0,26687	1,66	3,17	0,01514	---	TMEM143
8038069	-0,66	-1,58	0,11538	-0,79	-1,73	0,00999	NM_018273	---
8095005	-0,66	-1,58	0,71572	-5,94	-61,54	0,00041	---	MIR30A
8127500	-0,66	-1,58	0,30756	-0,30	-1,23	0,02538	NR_029504	FLJ43860
8153273	-0,66	-1,58	0,01650	-1,38	-2,60	0,00651	NM_207414	KRTAP10-11
8069156	-0,66	-1,58	0,53965	-2,06	-4,16	0,00019	NM_198692	PDE6B
8093360	-0,66	-1,58	0,03185	-0,57	-1,48	0,01295	NM_000283	SCARNA11
7960635	-0,66	-1,58	0,35119	-1,60	-3,03	0,01913	NR_003012	TTC9B
8036830	-0,66	-1,58	0,08487	-1,29	-2,45	0,00077	NM_152479	OR13C8
8157014	-0,66	-1,58	0,15260	-0,85	-1,80	0,00160	NM_001004483	OR10AG1
7948111	-0,66	-1,58	0,11017	-0,85	-1,81	0,01260	NM_001005491	KIAA1529
8156633	-0,66	-1,58	0,16482	0,29	1,23	0,35055	BC144521	EAF2
8082003	-0,66	-1,58	0,04981	-0,24	-1,18	0,13293	NM_018456	ZNF263
7992870	-0,66	-1,58	0,09266	0,87	1,83	0,07579	NM_005741	C11orf41
7939237	-0,66	-1,58	0,20351	-0,73	-1,65	0,01559	NM_012194	FLJ45721
8099754	-0,66	-1,58	0,09517	-1,14	-2,20	0,01737	AK127623	PAQR8
8120215	-0,66	-1,58	0,44651	0,58	1,49	0,07492	NM_133367	OR4S2
7939930	-0,66	-1,58	0,00441	-1,47	-2,77	0,03978	NM_001004059	---
7972733	-0,66	-1,58	0,00376	-0,91	-1,88	0,00499	---	CYP2B6
8028963	-0,66	-1,58	0,02000	-1,35	-2,55	0,00978	NM_000767	C15orf21
7983490	-0,66	-1,58	0,10209	-0,42	-1,34	0,24887	NR_022014	OR2A5
8136967	-0,66	-1,58	0,33468	-0,63	-1,55	0,10197	NM_012365	---
8106350	-0,66	-1,58	0,27320	-0,18	-1,13	0,45080	---	FOXP4
8119444	-0,66	-1,58	0,09833	0,81	1,75	0,03349	NM_001012426	CELA2B
7898184	-0,66	-1,58	0,12931	-1,12	-2,17	0,04248	NM_015849	GALE
7913667	-0,66	-1,58	0,09580	2,51	5,68	0,00489	NM_000403	---
7993335	-0,66	-1,58	0,26480	-1,83	-3,57	0,00222	---	KLHL4
8168622	-0,66	-1,58	0,05417	-0,58	-1,49	0,00227	NM_019117	A4GALT
8076497	-0,66	-1,58	0,04563	-0,08	-1,06	0,55951	NM_017436	ARHGEF4
8045229	-0,66	-1,58	0,07035	-0,14	-1,11	0,71325	NM_032995	ZCCHC18
8169083	-0,66	-1,58	0,26549	-0,58	-1,49	0,04066	NM_001143978	WASH3P
7986541	-0,66	-1,58	0,28807	0,77	1,71	0,01284	NR_003659	NCAPH2
8074082	-0,66	-1,58	0,07326	-1,13	-2,18	0,02673	NM_152299	---
8002102	-0,66	-1,58	0,13173	-1,69	-3,22	0,00005	---	PLA2G4F
7987815	-0,66	-1,58	0,05429	-1,04	-2,06	0,00792	NM_213600	ANKRD20A3
8155397	-0,66	-1,58	0,27703	-0,59	-1,51	0,05428	NM_001012419	ANKRD20A3
8161388	-0,66	-1,58	0,27703	-0,59	-1,51	0,05428	NM_001012419	---
7934432	-0,66	-1,58	0,14059	-0,83	-1,78	0,00249	---	BCL2L15
7918694	-0,66	-1,58	0,15784	-0,89	-1,85	0,01577	NM_001010922	C6orf174
8129392	-0,66	-1,58	0,07886	-0,29	-1,22	0,37018	NM_001012279	MS4A12
7940299	-0,66	-1,58	0,02861	-1,02	-2,03	0,06026	NM_017716	WNT5B
7953012	-0,66	-1,58	0,18956	-0,89	-1,85	0,13154	NM_032642	ZNF367
8162601	-0,66	-1,58	0,09348	-0,08	-1,05	0,84804	NM_153695	---
8138065	-0,66	-1,58	0,26247	-0,97	-1,96	0,02841	---	VSTM2L
8062427	-0,66	-1,58	0,14856	-0,68	-1,61	0,10425	NM_080607	---
8062687	-0,66	-1,58	0,10775	-0,71	-1,64	0,19719	---	---
8157677	-0,66	-1,58	0,16591	-1,59	-3,00	0,00008	---	---
7936639	-0,66	-1,58	0,12198	-1,63	-3,09	0,00116	---	STXBP5L
8081959	-0,66	-1,58	0,07110	-0,40	-1,32	0,01941	NM_014980	CRYGC
8058524	-0,66	-1,58	0,00420	-0,90	-1,87	0,04520	NM_020989	---
8148302	-0,66	-1,58	0,12378	-0,73	-1,66	0,03910	---	---
8152865	-0,66	-1,58	0,14758	-0,97	-1,96	0,03725	---	TMSB15A
8174189	-0,66	-1,58	0,10248	-1,22	-2,32	0,06985	NM_021992	---
8128618	-0,66	-1,58	0,10644	-0,69	-1,61	0,01004	---	SNORA70C
8163710	-0,66	-1,58	0,02972	-1,34	-2,53	0,00218	NR_003708	KIAA1654
8076561	-0,66	-1,58	0,13364	-0,80	-1,74	0,00276	AB051441	---
7922458	-0,66	-1,58	0,06000	-0,24	-1,18	0,15118	---	ZNF829
8036365	-0,66	-1,58	0,00707	-0,16	-1,12	0,34663	NM_001171979	SLC6A6
8078014	-0,66	-1,58	0,16194	2,68	6,40	0,00447	NM_003043	---
7909527	-0,66	-1,58	0,01084	-0,46	-1,38	0,43155	---	GRAMD4

8073890	-0,66	-1,58	0,01194	1,37	2,58	0,00362	NM_015124	KRTAP5-1
7945645	-0,66	-1,58	0,44138	-0,94	-1,92	0,17875	NM_001005922	QRICH2
8018673	-0,66	-1,58	0,06330	0,87	1,83	0,05328	NM_032134	GOLGA8F
7981895	-0,66	-1,58	0,13437	-0,34	-1,26	0,55114	NR_033351	BIRC5
8010260	-0,66	-1,58	0,14559	0,42	1,34	0,08688	NM_001168	TMEM52
7911634	-0,66	-1,58	0,32266	-1,02	-2,03	0,06440	NM_178545	RBM46
8097928	-0,66	-1,58	0,16603	-0,76	-1,70	0,00579	NM_144979	CYP4F8
8026442	-0,66	-1,58	0,10740	-1,17	-2,25	0,00941	NM_007253	GPR22
8135405	-0,66	-1,58	0,10376	-0,78	-1,72	0,00421	NM_005295	GOLGA9P
7982206	-0,66	-1,58	0,17194	-0,48	-1,40	0,01537	NR_024074	LOC440863
8041886	-0,66	-1,58	0,16090	-1,04	-2,05	0,01553	AK026773	TRIM40
8117840	-0,66	-1,58	0,13764	-1,58	-2,98	0,00464	NM_138700	---
8057416	-0,66	-1,58	0,03193	-1,05	-2,06	0,01117	---	---
8156525	-0,66	-1,58	0,01602	-1,07	-2,09	0,01256	---	DAOA
7969959	-0,66	-1,58	0,10482	-1,18	-2,26	0,00089	NM_172370	CYP8B1
8086457	-0,66	-1,58	0,02546	-0,99	-1,99	0,02209	NM_004391	---
8086536	-0,66	-1,58	0,10443	-0,83	-1,78	0,00105	---	RAD9A
7941865	-0,66	-1,58	0,05782	2,79	6,92	0,00203	NM_004584	NBPF7
7919080	-0,66	-1,58	0,00926	-0,75	-1,68	0,09226	NM_001047980	CDHR2
8110183	-0,66	-1,58	0,11102	-0,25	-1,19	0,48298	NM_017675	LENG9
8039269	-0,66	-1,58	0,03918	-0,34	-1,27	0,44775	NM_198988	DYDC1
7934767	-0,66	-1,58	0,01782	-0,97	-1,95	0,00927	NM_138812	CADM3
7906417	-0,66	-1,58	0,34438	-1,53	-2,89	0,00164	NM_021189	LY86
8116734	-0,66	-1,58	0,05703	-1,00	-2,01	0,00078	NM_004271	ITPKB
7924760	-0,66	-1,58	0,03331	0,25	1,19	0,34640	NM_002221	RXFP4
7906069	-0,66	-1,58	0,05415	-1,56	-2,95	0,02931	NM_181885	CXCL6
8095688	-0,66	-1,58	0,23825	-0,99	-1,99	0,04808	NM_002993	LRRIQ1
7957433	-0,66	-1,58	0,12206	-0,88	-1,84	0,02684	NM_032165	---
7959232	-0,66	-1,58	0,25090	-1,11	-2,15	0,00022	---	BFSP2
8082774	-0,66	-1,58	0,29913	-0,59	-1,50	0,22382	NM_003571	NPHP4
7911902	-0,66	-1,58	0,03531	0,01	1,01	0,94040	NM_015102	USP20
8158568	-0,66	-1,58	0,16265	1,05	2,06	0,00655	NM_006676	ZNF529
8036333	-0,66	-1,58	0,22323	-0,38	-1,30	0,49808	NM_020951	LIPI
8069521	-0,66	-1,58	0,04412	-0,66	-1,58	0,00050	NM_198996	FNDC7
7903530	-0,66	-1,58	0,23314	-0,50	-1,42	0,08647	NM_001144937	---
8104068	-0,66	-1,58	0,23032	-1,35	-2,56	0,01305	---	CLDN10
7969640	-0,66	-1,58	0,25616	0,62	1,53	0,17036	NM_182848	C2orf39
8040672	-0,66	-1,58	0,22237	-0,76	-1,69	0,04294	NM_145038	LOC440700
7906988	-0,66	-1,58	0,04764	-0,44	-1,36	0,06627	BC012108	SNCG
7928872	-0,66	-1,58	0,26826	-0,75	-1,68	0,19693	NM_003087	FLJ90757
8019083	-0,66	-1,58	0,02043	-0,22	-1,16	0,59491	NR_026857	CYBB
8166730	-0,66	-1,58	0,01739	-0,70	-1,63	0,01185	NM_000397	---
8094677	-0,66	-1,58	0,01983	-0,67	-1,59	0,13207	---	PSD2
8108453	-0,66	-1,58	0,01203	-1,37	-2,59	0,00200	NM_032289	---
7897995	-0,66	-1,58	0,18021	-0,56	-1,48	0,01565	---	---
7912589	-0,66	-1,58	0,18021	-0,56	-1,48	0,01565	---	C12orf28
7957092	-0,66	-1,58	0,02514	0,02	1,01	0,95785	BC143553	SYNM
7986385	-0,66	-1,58	0,26195	0,03	1,02	0,92604	NM_145728	---
7983677	-0,66	-1,58	0,29166	-0,82	-1,77	0,00167	---	C9orf38
8160020	-0,66	-1,58	0,04856	-1,17	-2,25	0,03804	AF090921	BCL2L12
8030429	-0,66	-1,58	0,36931	0,59	1,50	0,11702	NM_138639	PNPLA3
8073633	-0,66	-1,58	0,22096	0,25	1,19	0,59430	NM_025225	C16orf53
7994655	-0,66	-1,58	0,12978	0,36	1,28	0,16318	NM_024516	MLPH
8049487	-0,66	-1,59	0,02669	-0,36	-1,28	0,22953	NM_024101	C3orf39
8086462	-0,66	-1,59	0,11013	-0,16	-1,11	0,71773	NM_032806	FUNDC2P2
8043040	-0,66	-1,59	0,21448	-1,06	-2,08	0,02848	NR_003663	SYT6
7918716	-0,66	-1,59	0,19427	-1,54	-2,90	0,07148	NM_205848	ZNF536
8027431	-0,66	-1,59	0,01632	-0,60	-1,52	0,02470	NM_014717	---
8165709	-0,66	-1,59	0,56415	-3,99	-15,90	0,00899	---	GOLGA9P
7982271	-0,67	-1,59	0,18505	-1,20	-2,30	0,22471	NR_024074	---
7924461	-0,67	-1,59	0,21958	-1,36	-2,57	0,08634	---	---
8112611	-0,67	-1,59	0,04767	-0,74	-1,67	0,05982	---	DEFA4
8149109	-0,67	-1,59	0,10190	-0,79	-1,72	0,01026	NM_001925	SLCO1C1
7954312	-0,67	-1,59	0,04880	-1,34	-2,53	0,01970	NM_017435	LOC390956
8038668	-0,67	-1,59	0,18190	-1,41	-2,66	0,00866	ENST00000427643	---
8147691	-0,67	-1,59	0,01348	-1,08	-2,12	0,00061	---	BCO2
7943867	-0,67	-1,59	0,05319	-1,10	-2,14	0,02933	NM_031938	HOXD13
8046524	-0,67	-1,59	0,11737	-1,83	-3,56	0,00212	NM_000523	---
8001131	-0,67	-1,59	0,01142	-0,41	-1,33	0,01300	---	NR2E1
8121334	-0,67	-1,59	0,01384	-0,21	-1,16	0,08908	NM_003269	MLNR
7969093	-0,67	-1,59	0,44225	-0,80	-1,74	0,01753	NM_001507	MASP2
7912361	-0,67	-1,59	0,12076	-1,20	-2,30	0,01043	NM_006610	---
8126434	-0,67	-1,59	0,00273	-0,85	-1,80	0,03000	---	MIR200C

7953590	-0,67	-1,59	0,18317	-1,09	-2,12	0,03784	NR_029779	---
8136587	-0,67	-1,59	0,06251	-1,25	-2,37	0,02538	---	VAV3
7918157	-0,67	-1,59	0,12737	-0,75	-1,68	0,05924	NM_006113	---
7903889	-0,67	-1,59	0,31029	-2,30	-4,92	0,01822	---	---
8102777	-0,67	-1,59	0,00035	-0,76	-1,70	0,00310	---	ARMC6
8027117	-0,67	-1,59	0,36236	0,41	1,33	0,49079	NM_033415	---
8146326	-0,67	-1,59	0,16556	-1,67	-3,17	0,00399	---	---
7934566	-0,67	-1,59	0,20726	-2,10	-4,27	0,00070	---	LDB3
7928838	-0,67	-1,59	0,06242	-1,10	-2,15	0,01136	NM_007078	C2orf88
8047062	-0,67	-1,59	0,06923	-0,83	-1,78	0,00457	NM_001042519	ANKRD19
8156360	-0,67	-1,59	0,03910	-0,62	-1,54	0,14117	NR_026868	PDZRN4
7954926	-0,67	-1,59	0,04164	-1,24	-2,36	0,00124	NM_013377	NCRNA00116
8054511	-0,67	-1,59	0,28785	0,08	1,06	0,70544	BC064430	PITPNM2
7967358	-0,67	-1,59	0,10815	0,70	1,62	0,09147	NM_020845	RPL22P15
8044351	-0,67	-1,59	0,15739	-0,43	-1,35	0,15870	CR590757	---
7952319	-0,67	-1,59	0,06262	-0,89	-1,85	0,00388	---	TRPM2
8069085	-0,67	-1,59	0,06720	-1,02	-2,02	0,03161	NM_003307	STK31
8131890	-0,67	-1,59	0,23544	-0,05	-1,03	0,82775	NM_032944	FLJ35816
8093453	-0,67	-1,59	0,07235	-1,06	-2,08	0,11043	AK093135	ZG16
7994615	-0,67	-1,59	0,13036	-0,60	-1,51	0,00968	NM_152338	MIR323
7976832	-0,67	-1,59	0,20076	-1,51	-2,85	0,06554	NR_029890	---
8118005	-0,67	-1,59	0,10558	-0,30	-1,23	0,01929	---	ADAMTS14
7928147	-0,67	-1,59	0,24234	-1,23	-2,34	0,00382	NM_139155	---
7922667	-0,67	-1,59	0,08213	-1,26	-2,39	0,00129	---	CPNE7
7997942	-0,67	-1,59	0,13069	0,56	1,47	0,16065	NM_014427	IGHA1
7995263	-0,67	-1,59	0,61667	-3,29	-9,77	0,00136	AF067420	MAP6
7950524	-0,67	-1,59	0,21571	-0,75	-1,68	0,20052	NM_207577	CEACAM1
8037205	-0,67	-1,59	0,03383	-0,78	-1,72	0,00608	NM_001712	---
8044880	-0,67	-1,59	0,20302	-1,02	-2,03	0,00162	---	---
8167251	-0,67	-1,59	0,24364	-0,79	-1,73	0,05141	---	SPP2
8049425	-0,67	-1,59	0,03589	-1,35	-2,55	0,01765	NM_006944	---
8081356	-0,67	-1,59	0,00891	-0,95	-1,93	0,05948	---	AICDA
7960910	-0,67	-1,59	0,37100	-1,76	-3,39	0,00153	NM_020661	THAP7
8074688	-0,67	-1,59	0,18265	1,26	2,39	0,15258	NM_030573	PLEKHA7
7946781	-0,67	-1,59	0,21596	1,05	2,07	0,06822	NM_175058	LOC729866
7921342	-0,67	-1,59	0,11109	-0,57	-1,49	0,18465	AK057458	ZSCAN20
7899884	-0,67	-1,59	0,02891	-0,10	-1,07	0,79193	NM_145238	ESAM
7952436	-0,67	-1,59	0,02086	-0,79	-1,73	0,09984	NM_138961	PRSS38
7910211	-0,67	-1,59	0,00405	-1,42	-2,68	0,00575	NM_183062	PAIP2B
8052940	-0,67	-1,59	0,11861	1,42	2,67	0,02407	NM_020459	C10orf125
7937208	-0,67	-1,59	0,38568	0,11	1,08	0,79973	NM_198472	---
8060835	-0,67	-1,59	0,01622	-1,01	-2,02	0,00016	---	HERC5
8096361	-0,67	-1,59	0,14834	1,03	2,04	0,08035	NM_016323	---
8016607	-0,67	-1,59	0,05600	-1,48	-2,79	0,00027	---	MYO7A
7942717	-0,67	-1,59	0,12177	-0,74	-1,66	0,01909	NM_000260	GTSF1L
8066384	-0,67	-1,59	0,00629	-0,41	-1,33	0,00684	NM_176791	---
8148640	-0,67	-1,59	0,19393	-0,42	-1,34	0,00486	---	DENND2C
7918747	-0,67	-1,59	0,05430	-0,87	-1,82	0,03780	BC063894	EDA
8168062	-0,67	-1,59	0,05937	-0,96	-1,94	0,00274	NM_001399	HDC
7988672	-0,67	-1,59	0,04209	-1,25	-2,38	0,03103	NM_002112	---
8123336	-0,67	-1,59	0,08010	-0,72	-1,64	0,05773	---	TRPM6
8161774	-0,67	-1,59	0,02821	-0,63	-1,54	0,01158	NM_017662	---
8040111	-0,67	-1,59	0,11017	-0,55	-1,46	0,00453	---	HTR3C
8084275	-0,67	-1,59	0,24992	-0,92	-1,89	0,02493	NM_130770	ZC3H18
7997779	-0,67	-1,59	0,14598	0,29	1,22	0,29920	NM_144604	ANKRD2
7929653	-0,67	-1,59	0,02897	-0,79	-1,72	0,04584	NM_020349	ACE2
8171449	-0,67	-1,59	0,17994	-1,00	-2,00	0,02766	NM_021804	---
8139857	-0,67	-1,59	0,07360	-1,65	-3,13	0,00086	---	PFAS
8004804	-0,67	-1,59	0,13173	0,99	1,99	0,02572	NM_012393	---
7935056	-0,67	-1,59	0,01045	-0,87	-1,82	0,04251	---	GIPC2
7902518	-0,67	-1,59	0,17990	-0,90	-1,86	0,00286	NM_017655	ITGAM
7995096	-0,67	-1,59	0,14687	-1,33	-2,51	0,01719	NM_001145808	SNORD115-15
7982036	-0,67	-1,59	0,15663	-1,14	-2,20	0,03256	NR_003307	ELOVL7
8112274	-0,67	-1,59	0,01640	2,81	7,02	0,00041	NM_024930	TLR7
8166059	-0,67	-1,59	0,01604	-1,04	-2,06	0,00044	NM_016562	---
7964981	-0,67	-1,59	0,06906	-1,51	-2,84	0,01710	---	SNORD1A
8010082	-0,67	-1,59	0,00068	-1,24	-2,36	0,00660	NR_004395	EFS
7977978	-0,67	-1,59	0,06675	-0,98	-1,97	0,06181	NM_005864	HYDIN
8002492	-0,67	-1,59	0,20462	-0,56	-1,48	0,12865	NM_032821	SLC25A45
7949400	-0,67	-1,59	0,08482	1,06	2,09	0,01729	NM_182556	MIR155
8068022	-0,67	-1,59	0,36770	-0,89	-1,85	0,00079	NR_030784	ASB16
8007584	-0,67	-1,59	0,39209	-1,52	-2,86	0,00530	NM_080863	CEMP1
7998817	-0,67	-1,59	0,19037	-0,31	-1,24	0,04615	NM_001048212	DNHD1

7938139	-0,67	-1,59	0,15927	0,51	1,42	0,04809	NM_144666	IFNA14
8160383	-0,67	-1,59	0,12130	-0,85	-1,81	0,13210	NM_002172	CLEC4A
7953723	-0,67	-1,59	0,07177	-0,29	-1,22	0,07896	NM_016184	---
7993146	-0,67	-1,59	0,10871	-0,88	-1,84	0,00389	---	PRAMEF22
7898002	-0,67	-1,59	0,11835	-1,40	-2,63	0,01193	NM_001100631	OR1E1
8011339	-0,67	-1,59	0,03592	-0,72	-1,64	0,04033	NM_003553	---
8043018	-0,67	-1,59	0,32116	-1,48	-2,80	0,00007	---	NCRNA00164
8055297	-0,67	-1,59	0,26655	-0,33	-1,26	0,30815	NR_027020	BAIAP3
7992219	-0,67	-1,59	0,02607	0,22	1,16	0,50568	NM_003933	BREA2
8148671	-0,67	-1,60	0,33705	-0,51	-1,42	0,10607	NR_015445	---
8021712	-0,67	-1,60	0,13263	-0,51	-1,42	0,13235	---	C20orf117
8066091	-0,67	-1,60	0,03777	-0,44	-1,36	0,17051	NM_080627	SFTPD
7934719	-0,67	-1,60	0,22841	-1,22	-2,33	0,00402	NM_003019	OR11H1
7977472	-0,67	-1,60	0,22528	-3,11	-8,61	0,00057	NM_001005239	---
7949744	-0,67	-1,60	0,53403	-1,52	-2,86	0,10011	---	CNDP1
8021741	-0,67	-1,60	0,01628	-0,70	-1,62	0,04928	NM_032649	---
8068397	-0,67	-1,60	0,05761	1,78	3,44	0,00762	---	---
7927031	-0,67	-1,60	0,00505	-0,60	-1,51	0,00009	---	BIN2
7963289	-0,67	-1,60	0,11761	-1,51	-2,85	0,00011	NM_016293	---
8091095	-0,67	-1,60	0,00616	-0,99	-1,99	0,00882	---	---
8118544	-0,67	-1,60	0,21079	-1,98	-3,94	0,00955	---	TM6SF1
7985493	-0,67	-1,60	0,00139	1,27	2,41	0,00987	NM_023003	OR4D10
7940177	-0,67	-1,60	0,11663	-1,47	-2,78	0,01024	NM_001004705	TRNP1
7899284	-0,67	-1,60	0,36134	-0,62	-1,54	0,24645	NM_001013642	BMP15
8167642	-0,67	-1,60	0,03696	-1,04	-2,05	0,01590	NM_005448	PANX3
7944845	-0,68	-1,60	0,11526	-1,68	-3,20	0,01952	NM_052959	---
8062691	-0,68	-1,60	0,05951	-1,41	-2,65	0,00450	---	RRAGA
8154523	-0,68	-1,60	0,06402	-0,08	-1,06	0,76296	NM_006570	IL2RG
8173444	-0,68	-1,60	0,19840	-1,49	-2,81	0,00000	NM_000206	KRTAP21-2
8069855	-0,68	-1,60	0,20842	-1,37	-2,59	0,10837	NM_181617	HIST1H1B
8124527	-0,68	-1,60	0,10458	1,25	2,38	0,00116	NM_005322	SLITRK5
7969569	-0,68	-1,60	0,11189	-1,17	-2,25	0,02782	NM_015567	KPNA7
8141263	-0,68	-1,60	0,15818	1,98	3,93	0,00219	NM_001145715	SPAG4
8062190	-0,68	-1,60	0,03898	-0,21	-1,15	0,42816	NM_003116	BTK
8174051	-0,68	-1,60	0,09818	-0,89	-1,85	0,05187	NM_000061	CHDH
8088142	-0,68	-1,60	0,05071	0,94	1,91	0,03455	NM_018397	---
7910383	-0,68	-1,60	0,07376	0,96	1,95	0,16555	---	PLXNA2
7923991	-0,68	-1,60	0,00408	-0,35	-1,28	0,04319	NM_025179	HOXA10
8138757	-0,68	-1,60	0,10135	-0,12	-1,09	0,58335	NM_018951	---
7981333	-0,68	-1,60	0,00260	-0,99	-1,98	0,00836	---	---
8104854	-0,68	-1,60	0,27458	-1,30	-2,46	0,00681	---	C12orf63
7957688	-0,68	-1,60	0,01030	-0,75	-1,68	0,04091	NM_198520	CCDC19
7921473	-0,68	-1,60	0,16622	-0,45	-1,37	0,11605	NM_012337	C12orf37
7957503	-0,68	-1,60	0,27871	-0,95	-1,93	0,00041	AK058107	---
8150866	-0,68	-1,60	0,07283	-1,62	-3,07	0,00168	---	---
8030879	-0,68	-1,60	0,17552	-1,17	-2,25	0,04773	---	COL24A1
7917370	-0,68	-1,60	0,00045	-0,92	-1,89	0,01193	NM_152890	---
7940987	-0,68	-1,60	0,11019	-2,25	-4,75	0,00003	---	---
8040547	-0,68	-1,60	0,16114	-1,14	-2,20	0,01892	---	VCX3A
8171216	-0,68	-1,60	0,31021	-1,17	-2,25	0,00130	NM_016379	OR10G2
7977771	-0,68	-1,60	0,29048	-2,56	-5,88	0,00248	NM_001005466	NCOR2
7959772	-0,68	-1,60	0,17909	-0,42	-1,34	0,09713	NM_006312	---
8150586	-0,68	-1,60	0,13523	-0,80	-1,74	0,00644	---	---
7982375	-0,68	-1,60	0,03759	-1,05	-2,07	0,06039	---	---
8052089	-0,68	-1,60	0,05892	-1,12	-2,18	0,07141	---	GRIK1
8069770	-0,68	-1,60	0,04356	-0,79	-1,73	0,00108	NM_175611	RTP3
8079415	-0,68	-1,60	0,12116	-0,78	-1,72	0,00507	NM_031440	---
8115074	-0,68	-1,60	0,04876	-0,67	-1,59	0,01889	---	DEFB109P1B
8149380	-0,68	-1,60	0,43340	-1,08	-2,12	0,13508	NR_003668	LCE1B
7905525	-0,68	-1,60	0,02102	-0,89	-1,86	0,00144	NM_178349	SLC15A2
8082012	-0,68	-1,60	0,22012	-0,86	-1,82	0,01741	NM_021082	---
7910138	-0,68	-1,60	0,04960	-0,99	-1,99	0,07403	---	ICAM3
8033987	-0,68	-1,60	0,04043	0,64	1,56	0,01452	NM_002162	C3orf24
8085283	-0,68	-1,60	0,36537	-1,22	-2,33	0,02639	NM_173472	---
7990078	-0,68	-1,60	0,08120	-1,38	-2,60	0,08069	---	---
8146377	-0,68	-1,60	0,14290	-1,01	-2,02	0,00096	---	OR6N2
7921429	-0,68	-1,60	0,47267	-1,97	-3,91	0,00828	NM_001005278	---
8177269	-0,68	-1,60	0,49522	-0,65	-1,56	0,07740	---	ABCA12
8058708	-0,68	-1,60	0,03280	-0,72	-1,65	0,00447	NM_173076	---
8036589	-0,68	-1,60	0,07547	-0,45	-1,36	0,02520	---	FAM27C
8155497	-0,68	-1,60	0,51026	-1,94	-3,84	0,01378	NR_027421	---
8107348	-0,68	-1,60	0,07337	-0,86	-1,81	0,00055	---	TJP3
8024687	-0,68	-1,60	0,17219	0,58	1,49	0,33599	NM_014428	---

8065134	-0,68	-1,60	0,05653	-0,62	-1,54	0,04055	---	---
8124057	-0,68	-1,60	0,01875	-1,02	-2,03	0,02277	---	DZIP1L
8090938	-0,68	-1,60	0,05764	-1,49	-2,81	0,00665	NM_173543	RET
7927120	-0,68	-1,60	0,03577	-0,74	-1,67	0,00942	NM_020975	LOC388946
8041804	-0,68	-1,60	0,03582	-1,06	-2,08	0,04191	NM_001145051	GRM8
8142718	-0,68	-1,60	0,33978	-1,19	-2,28	0,00107	NM_001127323	EFCAB3
8008995	-0,68	-1,60	0,04597	-1,25	-2,38	0,00957	NM_173503	HIST1H2BL
8124510	-0,68	-1,60	0,13261	1,88	3,67	0,00619	NM_003519	TBC1D2B
7985224	-0,68	-1,60	0,52482	0,12	1,09	0,88245	NM_144572	CHRFAM7A
7987012	-0,68	-1,60	0,05399	0,21	1,16	0,54199	NM_139320	GALT
8154916	-0,68	-1,60	0,14012	0,04	1,03	0,79545	NM_000155	TBCC
8126442	-0,68	-1,60	0,14644	0,04	1,03	0,92647	NM_003192	---
7921930	-0,68	-1,60	0,17387	-0,62	-1,53	0,02600	---	COL21A1
8127201	-0,68	-1,60	0,09156	-1,22	-2,33	0,00057	NM_030820	PDZK1
7904843	-0,68	-1,60	0,01476	-0,87	-1,83	0,02598	NM_002614	---
7988175	-0,68	-1,60	0,04871	-1,04	-2,05	0,06134	---	---
7988206	-0,68	-1,60	0,04871	-1,04	-2,05	0,06134	---	ZNF276
7998002	-0,68	-1,60	0,03157	1,97	3,91	0,00025	NM_152287	HTN3
8095430	-0,68	-1,60	0,14689	-1,47	-2,78	0,01778	NM_000200	C1QTNF2
8115594	-0,68	-1,60	0,02046	-0,40	-1,32	0,23357	NM_031908	SLC45A3
7923792	-0,68	-1,60	0,22559	-1,15	-2,21	0,01279	NM_033102	BAHCC1
8010590	-0,68	-1,60	0,32540	-0,13	-1,10	0,74779	NM_001080519	MMP28
8014282	-0,68	-1,60	0,05572	-0,86	-1,81	0,07880	NM_024302	NKX2-5
8115840	-0,68	-1,60	0,03521	-0,72	-1,65	0,21310	NM_004387	CACNG2
8075820	-0,68	-1,60	0,09711	-0,54	-1,45	0,00461	NM_006078	---
7988410	-0,68	-1,60	0,05357	-0,68	-1,61	0,00919	---	---
8092220	-0,68	-1,60	0,00117	-1,34	-2,54	0,00839	---	CD6
7940391	-0,68	-1,60	0,11298	-0,80	-1,74	0,00036	NM_006725	C20orf107
8063531	-0,68	-1,60	0,21282	0,31	1,24	0,03873	BC105792	LCN10
8165271	-0,68	-1,60	0,21545	-0,39	-1,31	0,49454	NM_001001712	CCR5
8079401	-0,68	-1,60	0,02721	-0,93	-1,90	0,01182	NM_000579	CCR5
8093298	-0,68	-1,60	0,02721	-0,93	-1,90	0,01182	NM_000579	GNL1
8178346	-0,68	-1,60	0,13943	-0,43	-1,35	0,45614	NM_005275	DOCK6
8025850	-0,68	-1,60	0,26738	-0,77	-1,70	0,00052	NM_020812	LOC100131232
7949568	-0,68	-1,60	0,24184	-2,20	-4,60	0,00673	AK090761	---
8009515	-0,68	-1,60	0,00065	-1,07	-2,09	0,04024	---	BTBD16
7931062	-0,68	-1,60	0,08907	-0,62	-1,54	0,02800	NM_144587	LGALS9C
8005458	-0,68	-1,60	0,07166	-1,52	-2,87	0,04372	NM_001040078	---
7934991	-0,68	-1,60	0,00273	-1,24	-2,36	0,02608	---	IGKC // IGKC
8043436	-0,68	-1,60	0,41023	-0,88	-1,84	0,03605	BC073772 // BC073772	MOG
8177709	-0,68	-1,60	0,03368	-1,49	-2,81	0,00527	NM_206809	MED9
8005260	-0,68	-1,60	0,01320	-1,24	-2,36	0,00614	NM_018019	---
7911681	-0,68	-1,60	0,07039	-0,82	-1,76	0,02248	---	KRT32
8015293	-0,68	-1,60	0,15091	-0,81	-1,75	0,05536	NM_002278	CCDC89
7950804	-0,68	-1,60	0,19745	-1,32	-2,50	0,02468	NM_152723	---
8020421	-0,68	-1,60	0,11331	-0,28	-1,21	0,33536	---	ATF5
8030557	-0,68	-1,60	0,06988	0,51	1,42	0,16944	NM_012068	CYTH2
8030064	-0,68	-1,60	0,27147	-0,17	-1,13	0,71503	NM_017457	CLEC3B
8079305	-0,68	-1,60	0,10976	-1,58	-2,98	0,00240	NM_003278	C6orf124
8130817	-0,68	-1,60	0,08629	-0,26	-1,19	0,10384	NR_027906	MANEAL
7900183	-0,68	-1,60	0,14130	1,66	3,16	0,00445	NM_001031740	CHIC1
8168399	-0,68	-1,60	0,00025	-0,44	-1,36	0,12373	NM_001039840	GALNT3
8056408	-0,68	-1,60	0,21672	0,73	1,65	0,01383	NM_004482	FAM163B
8164937	-0,68	-1,60	0,13233	-0,76	-1,70	0,07115	BC146946	---
8089255	-0,68	-1,60	0,33812	-0,69	-1,61	0,19501	---	---
8047767	-0,68	-1,60	0,01668	-0,99	-1,98	0,08862	---	---
8043038	-0,68	-1,60	0,04532	-0,61	-1,52	0,01544	---	C9orf50
8164562	-0,68	-1,60	0,27388	-1,34	-2,52	0,05149	NM_199350	TMEM127
8053901	-0,68	-1,60	0,09771	0,84	1,79	0,09864	NM_017849	---
7917647	-0,68	-1,61	0,19201	-0,50	-1,41	0,22559	---	NDST4
8102450	-0,68	-1,61	0,11782	-0,85	-1,80	0,00087	NM_022569	KIAA0556
7994308	-0,68	-1,61	0,15434	-0,05	-1,04	0,64505	NM_015202	PTPRC
7908553	-0,68	-1,61	0,04213	-1,02	-2,03	0,00283	NM_002838	DCDC1
7947322	-0,68	-1,61	0,10295	-0,54	-1,45	0,00450	NM_181807	OR1F2P // OR1F2P // OR1F2P // OR1F2P
7992865	-0,68	-1,61	0,45425	1,11	2,16	0,01105	NR_002169 // NR_002169 // NR_002169 // NR_002169	---
7908591	-0,68	-1,61	0,37226	-1,28	-2,42	0,05818	---	EARS2
8000310	-0,68	-1,61	0,03236	0,02	1,01	0,96330	NM_001083614	GOLGA6L10
7990894	-0,68	-1,61	0,54048	-1,00	-1,99	0,02131	NM_001164465	SYT2
7923442	-0,68	-1,61	0,07320	-1,61	-3,06	0,00177	NM_177402	---
8137582	-0,68	-1,61	0,06715	-0,98	-1,97	0,00099	---	RP11-429E11.3
8067358	-0,68	-1,61	0,08825	-0,88	-1,84	0,01518	ENST00000317652	SEMA4D

8162236	-0,68	-1,61	0,14482	0,59	1,51	0,13774	NM_006378	LRRC9
7974737	-0,68	-1,61	0,08671	-1,21	-2,31	0,03202	ENST00000254271	---
8114579	-0,68	-1,61	0,01235	-0,49	-1,41	0,03843	---	OR5K1
8081210	-0,68	-1,61	0,04533	-1,01	-2,01	0,02478	NM_001004736	---
7922992	-0,68	-1,61	0,13120	-1,26	-2,39	0,01751	---	DDX43
8120679	-0,68	-1,61	0,03313	0,46	1,38	0,07260	NM_018665	ZC3H3
8153411	-0,68	-1,61	0,06546	0,50	1,41	0,34028	NM_015117	FYB
8111739	-0,68	-1,61	0,04477	-1,17	-2,25	0,00847	NM_001465	IL3
8107878	-0,68	-1,61	0,03118	-0,76	-1,69	0,00040	NM_000588	DSG1
8020724	-0,68	-1,61	0,06546	-0,49	-1,40	0,02045	NM_001942	OR4P4
7939928	-0,68	-1,61	0,05549	-0,61	-1,53	0,00937	NM_001004124	SHISA6
8004948	-0,68	-1,61	0,23550	-1,18	-2,27	0,00225	NM_207386	---
7924840	-0,68	-1,61	0,08379	-1,16	-2,23	0,13889	---	OR11H4
7973034	-0,68	-1,61	0,06511	-1,03	-2,04	0,00213	NM_001004479	HIGD2B
7990227	-0,68	-1,61	0,11669	-1,06	-2,08	0,01053	NR_002780	NICN1
8087433	-0,68	-1,61	0,26786	-0,12	-1,09	0,41444	NM_032316	ANKRD45
7922382	-0,68	-1,61	0,02895	-0,80	-1,74	0,01008	NM_198493	CXorf50B
8168391	-0,68	-1,61	0,13819	0,45	1,37	0,39855	NR_026594	OR10D1P
7944825	-0,68	-1,61	0,26457	-1,63	-3,11	0,03115	X64981	RNU5A
7984257	-0,68	-1,61	0,24971	-1,50	-2,83	0,02799	NR_002756	CDHR1
7928770	-0,69	-1,61	0,01177	0,05	1,04	0,55227	NM_033100	GRIA4
7943530	-0,69	-1,61	0,03258	-1,15	-2,21	0,00639	NM_000829	SNORD115-21
7982048	-0,69	-1,61	0,00118	-1,64	-3,11	0,01348	NR_003313	GGT3P
8074316	-0,69	-1,61	0,07289	-1,30	-2,47	0,10331	NR_003267	---
8132463	-0,69	-1,61	0,06281	-1,46	-2,74	0,00093	---	SDR42E1
8003060	-0,69	-1,61	0,01421	2,24	4,73	0,00113	NM_145168	LILRB3
8039196	-0,69	-1,61	0,21972	-0,59	-1,51	0,07957	NM_001081450	---
7983648	-0,69	-1,61	0,36853	-1,01	-2,02	0,05726	---	---
8006028	-0,69	-1,61	0,03822	-0,30	-1,23	0,04229	---	OR5B12
7948328	-0,69	-1,61	0,07418	-1,28	-2,43	0,00506	NM_001004733	ZNF397OS
8022872	-0,69	-1,61	0,14793	0,72	1,65	0,12047	NM_001112734	CPNE9
8077572	-0,69	-1,61	0,01240	-0,48	-1,39	0,04063	NM_153635	GADD45B
8024485	-0,69	-1,61	0,07138	1,10	2,14	0,01124	NM_015675	BTN1A1
8117498	-0,69	-1,61	0,09777	-1,01	-2,01	0,01683	NM_001732	HCRTR2
8120342	-0,69	-1,61	0,00171	-0,77	-1,70	0,01409	NM_001526	---
7979365	-0,69	-1,61	0,20864	-1,17	-2,26	0,01926	---	TMEM204
7992293	-0,69	-1,61	0,03146	-1,13	-2,20	0,00185	NM_024600	LOC100190938
8015706	-0,69	-1,61	0,08358	-1,29	-2,44	0,00031	NR_024461	---
7923424	-0,69	-1,61	0,09930	-0,67	-1,59	0,00035	---	IL1RL2
8044008	-0,69	-1,61	0,02515	-0,84	-1,79	0,04224	NM_003854	---
7911341	-0,69	-1,61	0,38878	-4,65	-25,16	0,00189	---	SLC22A25
7948957	-0,69	-1,61	0,11176	-2,00	-4,01	0,00030	NM_199352	KRT31
8015273	-0,69	-1,61	0,08944	-1,62	-3,08	0,06172	NM_002277	CNTNAP3
8155540	-0,69	-1,61	0,01039	0,69	1,62	0,09786	NM_033655	CNTNAP3
8161460	-0,69	-1,61	0,01039	0,69	1,62	0,09786	NM_033655	OR2AG2
7946278	-0,69	-1,61	0,20491	-0,82	-1,76	0,05221	NM_001004490	VAX1
7936552	-0,69	-1,61	0,23511	-1,20	-2,29	0,01784	NM_199131	ADAD1
8097236	-0,69	-1,61	0,13644	-1,38	-2,60	0,00210	NM_139243	---
7936318	-0,69	-1,61	0,02749	-0,82	-1,76	0,00083	---	HLA-DPB1
8118594	-0,69	-1,61	0,09127	1,25	2,39	0,01724	NM_002121	PIM2
8172471	-0,69	-1,61	0,12588	1,71	3,27	0,05093	NM_006875	IRS1
8059470	-0,69	-1,61	0,01307	1,19	2,28	0,13212	NM_005544	PNPLA1
8119052	-0,69	-1,61	0,06993	-1,34	-2,53	0,00063	NM_173676	PRAMEF17
7898048	-0,69	-1,61	0,16107	-1,74	-3,34	0,00133	NM_001099851	SMPDL3B
7899407	-0,69	-1,61	0,11302	2,93	7,60	0,00523	NM_014474	---
7927874	-0,69	-1,61	0,32863	-1,08	-2,11	0,00353	---	---
7927265	-0,69	-1,61	0,01070	-1,09	-2,13	0,00060	---	---
7927517	-0,69	-1,61	0,01070	-1,09	-2,13	0,00060	---	---
7933595	-0,69	-1,61	0,01070	-1,09	-2,13	0,00060	---	SNORA46
8001746	-0,69	-1,61	0,11037	-0,17	-1,12	0,18511	NR_002978	ARL9
8095246	-0,69	-1,61	0,03562	-1,15	-2,22	0,01588	NM_206919	CHRNB2
7905817	-0,69	-1,61	0,09245	-1,64	-3,11	0,00244	NM_000748	---
7969477	-0,69	-1,61	0,27480	-1,24	-2,36	0,00543	---	SBSN
8036103	-0,69	-1,61	0,08195	0,16	1,12	0,82127	NM_001166034	---
7909999	-0,69	-1,61	0,00335	-0,86	-1,81	0,00802	---	---
7967872	-0,69	-1,61	0,08883	-0,51	-1,42	0,08069	---	---
8142017	-0,69	-1,61	0,07648	-0,83	-1,78	0,09734	---	CLEC1A
7961111	-0,69	-1,61	0,00830	-1,01	-2,02	0,00019	NM_016511	NCRNA00029
8067588	-0,69	-1,61	0,09670	-1,47	-2,76	0,02495	NR_028295	VWC2L
8048112	-0,69	-1,61	0,12291	-0,95	-1,93	0,05675	NM_001080500	KCNK2
7909730	-0,69	-1,61	0,09612	-0,66	-1,58	0,00070	NM_001017425	EMID2
8135099	-0,69	-1,61	0,24561	-1,88	-3,68	0,01288	NM_133457	---
8068608	-0,69	-1,61	0,05160	-0,85	-1,80	0,00440	---	FAM65C

8066985	-0,69	-1,61	0,01585	-0,99	-1,98	0,04547	AK299337	MIR154
7976850	-0,69	-1,61	0,04510	-0,22	-1,17	0,19737	NR_029704	RAD9B
7958677	-0,69	-1,61	0,05683	-0,67	-1,59	0,02806	NM_152442	BPY2
8176821	-0,69	-1,61	0,15080	-1,28	-2,42	0,06026	NM_004678	BPY2
8176867	-0,69	-1,61	0,15080	-1,28	-2,42	0,06026	NM_004678	BPY2
8177449	-0,69	-1,61	0,15080	-1,28	-2,42	0,06026	NM_004678	RAB9B
8174271	-0,69	-1,61	0,22937	-1,28	-2,42	0,00367	NM_016370	---
8016016	-0,69	-1,61	0,45457	-1,59	-3,01	0,00058	---	C3orf34
8093145	-0,69	-1,61	0,07646	-0,26	-1,19	0,38205	NM_032898	C16orf13
7998251	-0,69	-1,61	0,11108	-0,61	-1,53	0,16505	NM_032366	C17orf78
8006706	-0,69	-1,61	0,06035	-0,76	-1,69	0,07365	NM_173625	DUSP4
8150076	-0,69	-1,61	0,01824	0,51	1,42	0,14945	NM_001394	---
7980889	-0,69	-1,61	0,02584	-0,94	-1,91	0,00969	---	GOLGA9P
7982256	-0,69	-1,61	0,39052	-0,86	-1,82	0,00420	NR_024074	TBR1
8045962	-0,69	-1,61	0,06057	-2,02	-4,07	0,00315	NM_006593	---
7976646	-0,69	-1,61	0,13660	-0,41	-1,33	0,03502	---	---
8091279	-0,69	-1,61	0,09305	-0,57	-1,48	0,07659	---	XIRP1
8086341	-0,69	-1,61	0,21946	-1,81	-3,50	0,00184	NM_194293	CNNM1
7929732	-0,69	-1,61	0,03430	0,95	1,93	0,01607	NM_020348	FLJ40453
8109995	-0,69	-1,61	0,05486	-1,26	-2,39	0,02110	AK097772	---
8081427	-0,69	-1,61	0,12400	-0,70	-1,62	0,00021	---	---
8043682	-0,69	-1,61	0,42251	-0,11	-1,08	0,90280	---	C12orf55
7957673	-0,69	-1,61	0,04177	-0,62	-1,54	0,10835	ENST00000298953	PLA2G12B
7934271	-0,69	-1,61	0,05387	-0,76	-1,69	0,04537	NM_032562	C2orf57
8049079	-0,69	-1,61	0,10346	-1,07	-2,10	0,08815	NM_152614	MAGEA9
8170407	-0,69	-1,61	0,12967	-1,10	-2,14	0,01403	NM_005365	MAGEA9
8175611	-0,69	-1,61	0,12967	-1,10	-2,14	0,01403	NM_005365	---
8086350	-0,69	-1,61	0,09624	-0,83	-1,78	0,04905	---	---
8147038	-0,69	-1,61	0,00490	-0,63	-1,54	0,08056	---	SPATA24
8114518	-0,69	-1,61	0,26703	-0,74	-1,67	0,02925	NM_194296	BFSP1
8065124	-0,69	-1,61	0,13981	-0,32	-1,25	0,29182	NM_001195	---
8108566	-0,69	-1,61	0,17071	-0,61	-1,53	0,00609	---	ALS2CL
8086627	-0,69	-1,61	0,02833	-0,37	-1,29	0,49030	NM_147129	HRK
7966746	-0,69	-1,61	0,33207	0,97	1,96	0,01628	NM_003806	ZNF691
7900624	-0,69	-1,61	0,02399	0,33	1,26	0,24202	NM_015911	KLRC4
7961166	-0,69	-1,61	0,03596	-0,93	-1,91	0,09133	NM_013431	SEMA4G
7929882	-0,69	-1,61	0,16033	1,21	2,32	0,02070	NM_017893	---
8116400	-0,69	-1,61	0,01865	-0,70	-1,63	0,02649	---	RLN1
8159981	-0,69	-1,61	0,00714	-0,43	-1,35	0,00298	NM_006911	---
7938681	-0,69	-1,61	0,01753	-0,68	-1,60	0,29909	---	PTGDS
8159521	-0,69	-1,61	0,24773	-1,74	-3,34	0,00238	NM_000954	A1CF
7933640	-0,69	-1,61	0,05429	-1,03	-2,04	0,02037	NM_138933	---
7950532	-0,69	-1,61	0,09462	-1,26	-2,40	0,00015	---	RNASE2
7973110	-0,69	-1,61	0,04652	-1,70	-3,24	0,05817	NM_002934	S100Z
8106411	-0,69	-1,61	0,01187	-0,58	-1,50	0,00828	NM_130772	---
8062204	-0,69	-1,61	0,24072	-2,26	-4,80	0,00175	---	---
7906465	-0,69	-1,61	0,21989	-0,38	-1,30	0,25645	---	PAX2
7929840	-0,69	-1,61	0,10040	-1,00	-2,00	0,00612	NM_003987	SP3P
7972247	-0,69	-1,61	0,15681	-1,31	-2,48	0,00008	BC036697	ARHGEF5
8136987	-0,69	-1,61	0,03143	0,79	1,73	0,05520	NM_005435	ADAM33
8064686	-0,69	-1,61	0,21697	-0,53	-1,45	0,07028	NM_025220	MIR129-1
8135907	-0,69	-1,61	0,01898	-1,18	-2,26	0,00720	NR_029596	FOXR1
7944351	-0,69	-1,61	0,05046	-1,16	-2,23	0,06715	NM_181721	POTEE
8045257	-0,69	-1,61	0,09726	-1,72	-3,30	0,00070	NM_001083538	ANKK1
7943943	-0,69	-1,61	0,17824	-1,02	-2,02	0,03958	NM_178510	---
8120878	-0,69	-1,61	0,10634	-1,09	-2,13	0,01329	---	HOXD3
8046560	-0,69	-1,61	0,22124	-0,95	-1,93	0,06931	NM_006898	CXorf57
8169158	-0,69	-1,61	0,05652	-0,84	-1,79	0,00167	NM_018015	PCSK2
8061094	-0,69	-1,61	0,02164	-1,15	-2,22	0,00291	NM_002594	---
8117170	-0,69	-1,61	0,04030	-1,16	-2,24	0,04825	---	GPIHBP1
8148591	-0,69	-1,61	0,15783	-0,51	-1,42	0,09204	NM_178172	---
7989126	-0,69	-1,61	0,07070	-0,66	-1,58	0,01155	---	HEXDC
8010787	-0,69	-1,61	0,06740	-0,69	-1,61	0,00601	NM_173620	C1orf173
7917019	-0,69	-1,61	0,08124	-0,72	-1,65	0,03962	NM_001002912	---
8142444	-0,69	-1,61	0,06477	-1,07	-2,10	0,00426	---	REG1A
8042986	-0,69	-1,61	0,12515	-0,89	-1,86	0,02484	NM_002909	C1orf69
7910261	-0,69	-1,61	0,04128	-0,69	-1,61	0,00129	NM_001010867	KIAA0040
7922474	-0,69	-1,62	0,03971	1,27	2,42	0,00020	NM_014656	PHLDB3
8037315	-0,69	-1,62	0,19405	-1,09	-2,13	0,02695	NM_198850	OR5M11
7948144	-0,69	-1,62	0,31333	-1,71	-3,28	0,00427	NM_001005245	TAT
8002556	-0,69	-1,62	0,04580	-0,92	-1,89	0,02943	NM_000353	HPSE
8101449	-0,69	-1,62	0,05724	0,80	1,74	0,03719	NM_006665	RNF151
7992409	-0,69	-1,62	0,32850	-0,87	-1,83	0,01736	NM_174903	---

8054295	-0,69	-1,62	0,20540	-1,05	-2,08	0,10742	---	---
7969366	-0,69	-1,62	0,09399	-0,98	-1,97	0,01936	---	UBTFL1
7939893	-0,69	-1,62	0,26020	-1,05	-2,07	0,00213	NM_001143975	AFM
8095663	-0,69	-1,62	0,18787	-0,86	-1,81	0,02331	NM_001133	---
8056972	-0,69	-1,62	0,04551	-0,91	-1,87	0,02444	---	F12
8116033	-0,69	-1,62	0,07971	-0,19	-1,14	0,50140	NM_000505	ZNF586
8031821	-0,69	-1,62	0,05031	-0,53	-1,44	0,11753	NM_017652	PRAMEF10 // PRAMEF10 // PRAMEF10
7898020	-0,69	-1,62	0,15175	-1,64	-3,11	0,00697	NM_001039361 // NM_001039361 // NM_001039361	PRAMEF10 // PRAMEF10 // PRAMEF10
7898034	-0,69	-1,62	0,15175	-1,64	-3,11	0,00697	NM_001039361 // NM_001039361 // NM_001039361	SLC26A9
7923850	-0,69	-1,62	0,04957	-0,04	-1,03	0,78065	NM_052934	MMP8
7951246	-0,69	-1,62	0,05246	-1,89	-3,71	0,00528	NM_002424	LOC392364
8162191	-0,69	-1,62	0,36118	-1,66	-3,17	0,00025	BC086877	---
7957954	-0,69	-1,62	0,05973	-0,92	-1,89	0,00012	---	---
8132415	-0,69	-1,62	0,05585	-0,99	-1,99	0,00788	---	SCARNA16
8010137	-0,69	-1,62	0,14426	-0,33	-1,26	0,39833	NR_003013	---
8048173	-0,69	-1,62	0,37999	-2,26	-4,79	0,00001	---	---
8177280	-0,69	-1,62	0,05216	-0,98	-1,97	0,00568	---	MIR30D
8153067	-0,69	-1,62	0,03924	-1,20	-2,29	0,01201	NR_029599	---
7943917	-0,69	-1,62	0,03342	-1,25	-2,38	0,02258	---	RFNG
8019367	-0,69	-1,62	0,09360	0,94	1,91	0,10454	NM_002917	TMEM207
8092735	-0,69	-1,62	0,00737	-1,34	-2,54	0,00066	NM_207316	C6orf122
8130934	-0,69	-1,62	0,00982	-1,18	-2,26	0,00826	NR_026781	ZNF562
8033801	-0,69	-1,62	0,18189	0,26	1,20	0,33771	NM_001130031	DDO
8128843	-0,69	-1,62	0,00326	-2,09	-4,25	0,00339	NM_003649	C22orf23
8075981	-0,69	-1,62	0,19192	-0,07	-1,05	0,62417	CR456440	GHRHR
8132130	-0,69	-1,62	0,17391	-1,94	-3,83	0,03163	NM_000823	RPS9
8031152	-0,69	-1,62	0,28905	0,87	1,82	0,12584	NM_001013	GBP5
7917576	-0,69	-1,62	0,08262	-0,84	-1,79	0,02001	NM_052942	KRT27
8015087	-0,69	-1,62	0,05458	-0,93	-1,90	0,01950	NM_181537	IL21
8102707	-0,69	-1,62	0,02086	-0,59	-1,51	0,03530	NM_021803	GSTTP1
8074988	-0,69	-1,62	0,40671	-0,93	-1,91	0,28811	NR_003081	GABRA2
8100109	-0,69	-1,62	0,00654	-0,87	-1,83	0,05247	NM_000807	COL9A1
8127446	-0,69	-1,62	0,10335	-1,41	-2,65	0,00697	NM_001851	SLAMF6
7921625	-0,69	-1,62	0,04778	-2,08	-4,22	0,00043	NM_052931	PI16
8119124	-0,69	-1,62	0,24931	-1,33	-2,52	0,00372	NM_153370	ECHDC2
7916229	-0,69	-1,62	0,25142	0,19	1,14	0,38710	NM_018281	F13A1
8123744	-0,69	-1,62	0,08613	3,53	11,56	0,00035	NM_000129	INSL6
8159971	-0,70	-1,62	0,08266	-1,13	-2,20	0,02923	NM_007179	psiTPTE22
8071063	-0,70	-1,62	0,00500	-1,03	-2,04	0,00321	NR_001591	---
8040068	-0,70	-1,62	0,17890	-0,97	-1,96	0,04045	---	SLAIN1
7969533	-0,70	-1,62	0,00185	2,39	5,23	0,01434	NM_001040153	KRT16P3
8013465	-0,70	-1,62	0,34648	-1,72	-3,29	0,00434	NR_029393	ARRDC1
8159692	-0,70	-1,62	0,23067	2,22	4,67	0,00687	NM_152285	TRBV21OR9-2
8154838	-0,70	-1,62	0,17396	-1,58	-2,98	0,00500	ENST00000331828	GABRA3
8175696	-0,70	-1,62	0,03238	-0,95	-1,93	0,00098	NM_000808	---
8122259	-0,70	-1,62	0,56574	-1,14	-2,20	0,46393	---	---
8154572	-0,70	-1,62	0,05654	-1,16	-2,24	0,00157	---	METT5D1
7939072	-0,70	-1,62	0,28429	-0,30	-1,23	0,51208	NM_152636	OR51A7
7937982	-0,70	-1,62	0,19468	-0,87	-1,82	0,00378	NM_001004749	---
8112196	-0,70	-1,62	0,02499	-1,34	-2,53	0,02768	---	---
8171723	-0,70	-1,62	0,24802	-1,18	-2,27	0,00446	---	KLF16
8032365	-0,70	-1,62	0,05175	1,26	2,40	0,14096	NM_031918	TMEM129
8098916	-0,70	-1,62	0,08646	1,60	3,03	0,15688	NM_001127266	C9orf3
8156549	-0,70	-1,62	0,03598	-0,32	-1,25	0,11451	NM_032823	---
8115812	-0,70	-1,62	0,04270	-1,01	-2,01	0,01278	---	RFPL4A
8031570	-0,70	-1,62	0,27336	-0,91	-1,88	0,00399	NM_001145014	C1orf129
7907232	-0,70	-1,62	0,17661	-1,50	-2,84	0,10092	NM_025063	---
8141358	-0,70	-1,62	0,18249	-0,60	-1,51	0,03389	---	LCT
8055406	-0,70	-1,62	0,06788	-0,66	-1,58	0,02300	NM_002299	---
7958209	-0,70	-1,62	0,05807	-0,89	-1,86	0,07263	---	LAMC3
8158739	-0,70	-1,62	0,06728	-0,17	-1,13	0,71530	NM_006059	CYP2B7P1
8028955	-0,70	-1,62	0,06505	-0,16	-1,12	0,61322	NR_001278	OR7A10
8034897	-0,70	-1,62	0,06239	-1,19	-2,29	0,01474	NM_001005190	DENND2A
8143397	-0,70	-1,62	0,13998	-1,60	-3,04	0,00144	NM_015689	---
7983989	-0,70	-1,62	0,01595	-1,05	-2,07	0,00329	---	TLL2
7935296	-0,70	-1,62	0,01303	-0,37	-1,30	0,16581	NM_012465	FAM153B
8116130	-0,70	-1,62	0,06908	-0,67	-1,59	0,08944	NM_001079529	TUBB4
8025051	-0,70	-1,62	0,14185	-1,07	-2,11	0,03367	NM_006087	OR9Q2
7940112	-0,70	-1,62	0,28605	-1,75	-3,37	0,01542	NM_001005283	FAM70A
8174767	-0,70	-1,62	0,00809	-0,84	-1,79	0,00860	NM_017938	LECT1

7971838	-0,70	-1,62	0,05299	-0,34	-1,27	0,02432	NM_007015	---
8153035	-0,70	-1,62	0,03083	-1,26	-2,39	0,00396	---	TBC1D3
8006746	-0,70	-1,62	0,35114	1,41	2,65	0,04535	NM_001123391	KIAA0125
7977440	-0,70	-1,62	0,13272	-1,73	-3,31	0,00830	NR_026800	MKRN9P
7965245	-0,70	-1,62	0,10078	-0,85	-1,80	0,02139	NR_033410	GABRR2
8128099	-0,70	-1,62	0,19047	-1,35	-2,55	0,00409	NM_002043	CCDC73
7947375	-0,70	-1,62	0,12169	-1,18	-2,26	0,00696	NM_001008391	RAD54L
7901192	-0,70	-1,62	0,08262	0,25	1,19	0,57441	NM_003579	---
8030840	-0,70	-1,62	0,00557	-1,04	-2,06	0,02527	---	HRH4
8020684	-0,70	-1,62	0,05873	-1,27	-2,42	0,03381	NM_021624	---
8131438	-0,70	-1,62	0,20970	-0,32	-1,25	0,51228	---	CCDC54
8081462	-0,70	-1,62	0,09588	-0,87	-1,83	0,01575	NM_032600	---
8103238	-0,70	-1,62	0,26150	-0,83	-1,78	0,06717	---	---
7969424	-0,70	-1,62	0,00106	-0,88	-1,84	0,02886	---	OR52N5
7946105	-0,70	-1,62	0,40619	-1,91	-3,76	0,00144	NM_001001922	SCARF2
8074617	-0,70	-1,62	0,12976	-1,37	-2,59	0,02871	NM_153334	LY6G6C
8125042	-0,70	-1,62	0,19070	-1,14	-2,21	0,12595	NM_025261	---
7937876	-0,70	-1,62	0,46156	-0,93	-1,91	0,01072	---	TMEM71
8152976	-0,70	-1,62	0,03674	-1,17	-2,26	0,00003	NM_144649	IFNA5
8160401	-0,70	-1,62	0,10151	-1,02	-2,03	0,00242	NM_002169	WASH2P
8044649	-0,70	-1,62	0,31951	0,93	1,91	0,00936	NR_024077	RS1
8171579	-0,70	-1,62	0,08644	-0,78	-1,72	0,03033	NM_000330	KRT25
8015070	-0,70	-1,62	0,23603	-1,32	-2,49	0,00044	NM_181534	---
8145529	-0,70	-1,62	0,19188	-0,96	-1,94	0,11016	---	BAI1
8148517	-0,70	-1,62	0,27020	-0,47	-1,39	0,08434	NM_001702	FAM177B
7909946	-0,70	-1,62	0,01302	-0,47	-1,38	0,01233	NM_207468	C2orf46
8050115	-0,70	-1,62	0,06365	-0,78	-1,71	0,01139	AK127578	---
8158145	-0,70	-1,62	0,29437	-0,69	-1,61	0,01994	---	---
8105603	-0,70	-1,62	0,08694	-1,40	-2,64	0,00002	---	DNAI1
8154892	-0,70	-1,62	0,02269	-0,65	-1,57	0,00972	NM_012144	JMJD5
8000411	-0,70	-1,62	0,33910	0,50	1,42	0,25769	NM_001145348	---
8104926	-0,70	-1,62	0,01971	-1,03	-2,04	0,00092	---	LIPK
7928994	-0,70	-1,62	0,06704	-0,52	-1,43	0,14423	NM_001080518	UGT3A2
8111524	-0,70	-1,62	0,07239	-0,69	-1,61	0,21902	NM_174914	C4orf6
8093872	-0,70	-1,62	0,03230	-1,56	-2,95	0,00645	NM_005750	---
8061068	-0,70	-1,62	0,15652	-1,11	-2,16	0,01963	---	---
8054393	-0,70	-1,62	0,01614	-0,90	-1,86	0,00436	---	CSMD3
8152376	-0,70	-1,62	0,03835	-1,20	-2,30	0,00031	NM_198124	RBP2
8091071	-0,70	-1,62	0,09106	-0,70	-1,63	0,00142	NM_004164	---
8088903	-0,70	-1,62	0,45496	-2,73	-6,64	0,01039	---	C20orf165
8066609	-0,70	-1,62	0,22473	-0,39	-1,31	0,17909	NM_080608	SNHG11
8062480	-0,70	-1,62	0,03778	0,22	1,16	0,52542	NR_003239	TBC1D3G
8019716	-0,70	-1,62	0,36113	1,29	2,44	0,07112	NM_001040282	---
8112664	-0,70	-1,62	0,00511	-0,62	-1,53	0,01667	---	OR51G2
7946011	-0,70	-1,62	0,10309	-1,71	-3,28	0,00007	NM_001005238	VCX3B
8165890	-0,70	-1,62	0,27442	-1,05	-2,07	0,00288	NM_001001888	LOC349196
8144412	-0,70	-1,62	0,60334	-1,47	-2,77	0,10267	NR_027000	LOC349196
8144414	-0,70	-1,62	0,60334	-1,47	-2,77	0,10267	NR_027000	GREB1
8040292	-0,70	-1,62	0,04868	-0,17	-1,12	0,40219	NM_014668	SNORD115-14
7982034	-0,70	-1,62	0,07785	-0,88	-1,84	0,02472	NR_003306	NLRC3
7998983	-0,70	-1,63	0,12118	-1,54	-2,91	0,00330	NM_178844	SLCO6A1
8113385	-0,70	-1,63	0,00729	-1,09	-2,13	0,01348	NM_173488	SULT1A3
7994781	-0,70	-1,63	0,11735	0,82	1,77	0,03909	NM_003166	PTPN23
8079532	-0,70	-1,63	0,11619	1,06	2,08	0,04315	NM_015466	MIR140
7997008	-0,70	-1,63	0,05334	-1,26	-2,40	0,02977	NR_029681	OR5M1
7948150	-0,70	-1,63	0,12005	-1,26	-2,40	0,00189	NM_001004740	---
8176482	-0,70	-1,63	0,40135	-1,45	-2,73	0,10712	---	FAM138D
7960172	-0,70	-1,63	0,11155	-1,82	-3,52	0,00776	AY341951	FAM110A
8060370	-0,70	-1,63	0,05755	0,14	1,10	0,79589	NM_031424	SSX2
8167728	-0,70	-1,63	0,28006	-1,70	-3,25	0,01839	NM_003147	RFPL3
8072605	-0,70	-1,63	0,18961	-0,21	-1,16	0,47512	NM_001098535	MED26
8035187	-0,70	-1,63	0,01324	-0,30	-1,23	0,27291	NM_004831	OCM
8131349	-0,70	-1,63	0,13095	-0,54	-1,46	0,03523	NM_001097622	---
8040614	-0,70	-1,63	0,20225	-1,26	-2,40	0,01397	---	PSTPIP1
7985099	-0,70	-1,63	0,00189	-0,87	-1,83	0,00433	NM_003978	LCE2B
7905505	-0,70	-1,63	0,24903	-1,12	-2,18	0,03000	NM_014357	---
7901383	-0,70	-1,63	0,02125	-0,90	-1,86	0,00572	---	OR4H12P
7973006	-0,70	-1,63	0,28760	-2,77	-6,83	0,00622	ENST00000316004	ADSSL1
7977273	-0,70	-1,63	0,22021	1,18	2,27	0,01008	NM_152328	---
7971359	-0,70	-1,63	0,01581	-1,21	-2,31	0,07983	---	---
8110104	-0,70	-1,63	0,06368	-0,80	-1,74	0,10663	---	FTMT
8107576	-0,70	-1,63	0,06431	-1,03	-2,04	0,02397	NM_177478	FDXR
8018236	-0,70	-1,63	0,02192	-0,02	-1,01	0,96991	NM_024417	LRP11

8130142	-0,70	-1,63	0,30409	0,93	1,91	0,04821	NM_032832	PRL
8124185	-0,70	-1,63	0,14875	-1,11	-2,15	0,00804	NM_000948	ABHD11
8140129	-0,70	-1,63	0,06763	0,64	1,55	0,30428	NR_026912	SMC1B
8076704	-0,70	-1,63	0,00297	-0,50	-1,42	0,08994	NM_148674	---
8130626	-0,70	-1,63	0,49127	-2,49	-5,62	0,02352	---	EPHA1
8143575	-0,70	-1,63	0,04904	-0,12	-1,09	0,58334	NM_005232	TNMD
8168737	-0,70	-1,63	0,23868	-0,55	-1,46	0,03926	NM_022144	LRSAM1
8158028	-0,70	-1,63	0,17175	0,16	1,12	0,57914	NM_138361	UBN1
7993035	-0,70	-1,63	0,07555	0,20	1,15	0,23603	NM_016936	SC65
8015432	-0,70	-1,63	0,01306	-0,11	-1,08	0,79391	NM_006455	DEFB135
8144687	-0,70	-1,63	0,00333	-1,05	-2,08	0,02597	NM_001033017	HRCT1
8155167	-0,70	-1,63	0,04063	-0,15	-1,11	0,16159	NM_001039792	GNGT1
8134252	-0,70	-1,63	0,09768	-1,05	-2,08	0,10390	NM_021955	METT11D1
7973116	-0,70	-1,63	0,01114	1,77	3,42	0,00933	NM_001029991	LY6G6C
8179814	-0,70	-1,63	0,17141	-1,27	-2,41	0,09890	NM_025261	ANAPC2
8165462	-0,70	-1,63	0,03263	0,33	1,26	0,55826	NM_013366	LRFN3
8028112	-0,70	-1,63	0,10387	-0,26	-1,20	0,37584	NM_024509	CLDN16
8084788	-0,70	-1,63	0,11479	0,64	1,55	0,11753	NM_006580	TEKT1
8011990	-0,70	-1,63	0,02875	-0,50	-1,41	0,07071	NM_053285	---
8128445	-0,70	-1,63	0,47933	-1,26	-2,40	0,07190	---	---
8048116	-0,70	-1,63	0,10359	0,68	1,60	0,21196	---	---
8173605	-0,70	-1,63	0,16048	-0,89	-1,86	0,00205	---	---
8124335	-0,70	-1,63	0,22765	-0,67	-1,59	0,06467	---	HHEX
7929282	-0,70	-1,63	0,21247	-0,89	-1,85	0,15363	NM_002729	FLYWCH2
7992737	-0,70	-1,63	0,18240	0,01	1,01	0,95710	NM_138439	TBC1D16
8019031	-0,70	-1,63	0,11823	-0,70	-1,63	0,26198	NM_019020	HSP90AB6P
7972420	-0,70	-1,63	0,06636	-1,14	-2,21	0,03409	AY956767	---
8098437	-0,70	-1,63	0,01469	-1,17	-2,25	0,02415	---	FSCN3
8135865	-0,70	-1,63	0,00514	-0,88	-1,84	0,04728	NM_020369	TTTY10
8177261	-0,70	-1,63	0,18795	-0,19	-1,14	0,12691	NR_001542	FER1L6
8148220	-0,70	-1,63	0,00321	-1,17	-2,25	0,00013	NM_001039112	CDO1
8113641	-0,70	-1,63	0,00640	-0,96	-1,95	0,01079	NM_001801	PCNT
8069348	-0,70	-1,63	0,00566	0,24	1,18	0,12862	NM_006031	MGC45800
8103853	-0,70	-1,63	0,17142	-1,89	-3,70	0,00136	NR_027107	KIAA0319
8124240	-0,70	-1,63	0,12196	0,13	1,09	0,71053	NM_014809	FCRL1
7921319	-0,70	-1,63	0,04528	-1,46	-2,75	0,00779	NM_052938	---
8104305	-0,70	-1,63	0,37398	-2,43	-5,39	0,00325	---	XKRY
8176675	-0,70	-1,63	0,01970	-0,66	-1,58	0,00219	NM_004677	XKRY
8177180	-0,70	-1,63	0,01970	-0,66	-1,58	0,00219	NM_004677	FAM84B
8152812	-0,70	-1,63	0,01891	1,44	2,72	0,01931	NM_174911	LOC168474 // LOC168474 // LOC168474
8139826	-0,70	-1,63	0,17490	-0,30	-1,23	0,26976	NR_002789 // NR_002789 // NR_002789	---
7900405	-0,70	-1,63	0,04378	-1,35	-2,54	0,00035	---	MIR30C1
7900490	-0,71	-1,63	0,06766	-1,10	-2,14	0,00610	NR_029833	LOC100129112
8017344	-0,71	-1,63	0,11306	1,68	3,21	0,01279	AK123839	---
7901359	-0,71	-1,63	0,00543	-0,66	-1,58	0,00731	---	---
8113664	-0,71	-1,63	0,09638	-0,85	-1,80	0,02095	---	TBC1D26
8005117	-0,71	-1,63	0,29639	-1,46	-2,75	0,05390	NM_178571	AGFG2
8134834	-0,71	-1,63	0,10553	-0,76	-1,70	0,05407	NM_006076	CCM2
8132646	-0,71	-1,63	0,04279	1,00	2,00	0,02270	NM_031443	C5orf46
8114970	-0,71	-1,63	0,03221	-1,47	-2,78	0,00004	NM_206966	---
8171311	-0,71	-1,63	0,14822	-2,11	-4,32	0,02661	---	C1orf137
7904281	-0,71	-1,63	0,06383	-1,30	-2,47	0,00558	ENST00000369482	MACROD2
8061035	-0,71	-1,63	0,05828	-1,06	-2,09	0,00127	NM_080676	LOC441177
8123334	-0,71	-1,63	0,04484	-1,49	-2,81	0,00224	NR_027284	---
7925128	-0,71	-1,63	0,19264	-0,52	-1,44	0,13405	---	LOC348840
8093272	-0,71	-1,63	0,00810	-1,05	-2,07	0,02390	NR_003291	PLGLB2
8043367	-0,71	-1,63	0,15674	0,10	1,07	0,51796	NM_002665	PLGLB2
8053602	-0,71	-1,63	0,15674	0,10	1,07	0,51796	NM_002665	---
8155246	-0,71	-1,63	0,20018	-1,07	-2,10	0,04744	---	SLC7A14
8092055	-0,71	-1,63	0,07535	-2,26	-4,77	0,00196	NM_020949	UTS2
7912136	-0,71	-1,63	0,00093	-0,91	-1,89	0,02424	NM_021995	TRAF1
8163825	-0,71	-1,63	0,03253	-0,41	-1,33	0,06075	NM_005658	RHBDL3
8006367	-0,71	-1,63	0,22206	-1,07	-2,09	0,06715	NM_138328	C9orf173
8159646	-0,71	-1,63	0,13494	-1,14	-2,20	0,02264	NM_001004353	CXorf24
8167163	-0,71	-1,63	0,11520	-0,42	-1,34	0,17899	ENST00000357412	ESYT3
8082974	-0,71	-1,63	0,02462	0,91	1,88	0,00159	NM_031913	TMEM14E
8091546	-0,71	-1,63	0,01810	-0,57	-1,48	0,03267	NM_001123228	TUBA8
8071147	-0,71	-1,63	0,07143	0,17	1,13	0,72438	NM_018943	C14orf177
7976635	-0,71	-1,63	0,05477	-0,75	-1,69	0,11011	NM_182560	NCF2
7922773	-0,71	-1,63	0,03695	-1,19	-2,28	0,00248	NM_000433	---
7969358	-0,71	-1,63	0,09299	-0,63	-1,55	0,01449	---	GPR32

8030733	-0,71	-1,63	0,00567	-1,11	-2,16	0,00559	NM_001506	PITPNM3
8011924	-0,71	-1,63	0,02361	-0,60	-1,52	0,10210	NM_031220	ACADS
7959220	-0,71	-1,63	0,06492	-0,43	-1,35	0,40196	NM_000017	RP1
8146468	-0,71	-1,63	0,07181	-1,06	-2,09	0,10776	NM_006269	PGM5
8044640	-0,71	-1,63	0,34200	-1,00	-2,00	0,00067	NM_021965	KRBA2
8012466	-0,71	-1,63	0,08779	-1,36	-2,56	0,02562	NM_213597	CYP2A13
8028973	-0,71	-1,63	0,10835	-1,90	-3,73	0,00977	NM_000766	GTSCR1
8023816	-0,71	-1,63	0,02256	-1,17	-2,25	0,02867	AY262164 ---	
7926354	-0,71	-1,63	0,02760	-1,31	-2,48	0,01382	---	C20orf114
8061894	-0,71	-1,63	0,06570	-0,30	-1,23	0,09702	NM_033197	TERT
8110783	-0,71	-1,63	0,25942	-0,38	-1,30	0,46842	NM_198253	HOMER2
7991034	-0,71	-1,63	0,09786	0,98	1,97	0,01940	NM_199330	CDCA8
7900167	-0,71	-1,63	0,01967	1,29	2,45	0,02628	NM_018101	---
7938047	-0,71	-1,63	0,01084	-1,63	-3,09	0,00161	---	SIGLEC15
8021091	-0,71	-1,63	0,10603	-0,87	-1,82	0,06439	NM_213602	KIAA1024
7985248	-0,71	-1,63	0,06408	0,95	1,93	0,02045	NM_015206	IGHV4-31
7981601	-0,71	-1,63	0,07793	-1,40	-2,63	0,00020	AK301335 HLA-DQB1	
8125447	-0,71	-1,63	0,00230	-0,18	-1,13	0,71603	NM_002123	CLDND2
8038804	-0,71	-1,63	0,36331	-0,88	-1,84	0,23341	NM_152353	HIST1H2BE
8117389	-0,71	-1,63	0,01085	-0,88	-1,84	0,11399	NM_003523	NFE2
7963760	-0,71	-1,63	0,12257	-0,52	-1,43	0,04756	NM_001136023	FAM48B2
8166509	-0,71	-1,63	0,42184	-0,49	-1,40	0,29720	NM_001136233	FAM48B2
8171844	-0,71	-1,63	0,42184	-0,49	-1,40	0,29720	NM_001136233	SNTG2
8039977	-0,71	-1,63	0,08517	-1,24	-2,37	0,03886	NM_018968	ZFHX2
7978059	-0,71	-1,63	0,00371	-0,32	-1,25	0,38126	BC131533 SNORA1	
7951032	-0,71	-1,63	0,03815	-1,92	-3,80	0,02580	NR_003026	OR5M3
7939988	-0,71	-1,63	0,12163	-1,86	-3,63	0,16012	NM_001004742	---
8149590	-0,71	-1,64	0,06674	-1,64	-3,11	0,11412	---	---
8147650	-0,71	-1,64	0,05135	-1,15	-2,21	0,04436	---	NOTCH3
8034940	-0,71	-1,64	0,09984	0,64	1,56	0,23438	NM_000435	CNGB1
8001615	-0,71	-1,64	0,06781	-1,14	-2,21	0,02009	NM_001297	CCNF
7992594	-0,71	-1,64	0,03400	0,62	1,54	0,30228	NM_001761	MIR124-2
8146643	-0,71	-1,64	0,11809	-1,67	-3,19	0,05320	NR_029669	FAM186B
7963084	-0,71	-1,64	0,06260	-0,41	-1,33	0,11304	NM_032130	DSP
8116780	-0,71	-1,64	0,00023	2,67	6,37	0,00056	NM_004415	OLFM4
7969288	-0,71	-1,64	0,03487	-1,15	-2,23	0,02460	NM_006418	---
7928298	-0,71	-1,64	0,00291	-0,74	-1,67	0,16275	---	PLCB2
7987475	-0,71	-1,64	0,01409	-0,76	-1,70	0,06184	NM_004573	GJA8
7904927	-0,71	-1,64	0,04907	-1,93	-3,80	0,00025	NM_005267	---
7974212	-0,71	-1,64	0,09856	-1,18	-2,26	0,00398	---	---
8065853	-0,71	-1,64	0,01958	-0,70	-1,63	0,00243	---	WSCD2
7958398	-0,71	-1,64	0,07871	-0,72	-1,65	0,02448	NM_014653	MIR34B
7943736	-0,71	-1,64	0,22939	-0,27	-1,21	0,11371	NR_029839	---
8002301	-0,71	-1,64	0,43892	-2,02	-4,07	0,00088	---	PREX2
8146794	-0,71	-1,64	0,04952	-1,00	-2,00	0,05269	NM_024870	SLC22A13
8078759	-0,71	-1,64	0,09445	-1,59	-3,01	0,01190	NM_004256	SLC35E2
7911591	-0,71	-1,64	0,22106	1,20	2,31	0,07584	NM_001110781	MSTO2P
7906056	-0,71	-1,64	0,24734	1,78	3,43	0,01796	NR_024117	TMEM35
8168790	-0,71	-1,64	0,10962	-1,76	-3,40	0,05754	NM_021637	SCN10A
8086255	-0,71	-1,64	0,00556	-0,99	-1,99	0,01176	NM_006514	LHX8
7902353	-0,71	-1,64	0,01501	-1,26	-2,39	0,00882	NM_001001933	LOC728855
7919394	-0,71	-1,64	0,12373	-0,71	-1,64	0,08171	NR_024510	SIRPG
8064485	-0,71	-1,64	0,07206	-1,54	-2,90	0,00047	NM_018556	CTAGE6
8136936	-0,71	-1,64	0,11000	-0,59	-1,51	0,03297	NM_178561	SPRR3
7905548	-0,71	-1,64	0,05290	-1,08	-2,11	0,00095	NM_005416	TACC2
7931031	-0,71	-1,64	0,05942	0,30	1,23	0,25879	NM_206862	---
7993935	-0,71	-1,64	0,01676	-0,72	-1,65	0,08729	---	ZNF615
8038925	-0,71	-1,64	0,13096	0,07	1,05	0,78995	NM_198480	PRSS3
8154848	-0,71	-1,64	0,26522	-0,52	-1,43	0,02881	NM_007343	ARL4A
8131573	-0,71	-1,64	0,13589	-1,25	-2,37	0,00151	NM_212460	FHDC1
8097829	-0,71	-1,64	0,19342	-0,06	-1,05	0,77401	NM_033393	S1PR3
8156278	-0,71	-1,64	0,13863	-1,70	-3,26	0,00005	NM_005226	LOC730441
8143494	-0,71	-1,64	0,11962	-0,87	-1,83	0,04536	BC039387 ---	
8151766	-0,71	-1,64	0,09217	-1,49	-2,81	0,00001	---	C4orf17
8096568	-0,71	-1,64	0,14925	-0,93	-1,90	0,02425	NM_032149	---
7919303	-0,71	-1,64	0,10745	-1,92	-3,78	0,00356	---	---
8020682	-0,71	-1,64	0,01034	-0,82	-1,77	0,00009	---	IL22RA2
8129848	-0,71	-1,64	0,14506	-1,28	-2,42	0,00316	NM_052962	---
8023375	-0,71	-1,64	0,00387	-0,56	-1,47	0,21009	---	MS4A1
7940287	-0,71	-1,64	0,00211	-1,26	-2,40	0,01575	NM_152866	GFOD2
8002082	-0,71	-1,64	0,05116	0,46	1,37	0,11890	NM_030819	NEURL
7930264	-0,71	-1,64	0,19290	-0,05	-1,03	0,83729	NM_004210	HIST1H2AL
8117608	-0,71	-1,64	0,04765	-0,04	-1,03	0,81467	NM_003511	---

8056043	-0,71	-1,64	0,06107	-1,22	-2,32	0,00017	---	CNO
8093957	-0,71	-1,64	0,07288	-0,79	-1,73	0,00687	NM_018366	BARHL1
8158912	-0,71	-1,64	0,14229	-1,97	-3,91	0,00697	NM_020064	IQCJ
8083673	-0,71	-1,64	0,01007	-1,67	-3,18	0,00584	NM_001042706	RECQL4
8153838	-0,71	-1,64	0,02243	-0,59	-1,50	0,30637	NM_004260	---
8128954	-0,71	-1,64	0,01371	-0,79	-1,73	0,00147	---	DACH2
8168604	-0,71	-1,64	0,09910	-1,20	-2,30	0,02320	NM_053281	FLJ37396
8121405	-0,71	-1,64	0,13548	0,97	1,95	0,13893	AK094715	HIP1R
7959500	-0,71	-1,64	0,00033	0,71	1,64	0,07676	NM_003959	ZNF645
8166433	-0,71	-1,64	0,02499	-0,77	-1,70	0,09939	NM_152577	LOC400986
8053797	-0,71	-1,64	0,25591	1,24	2,37	0,04296	ENST00000456556	GLP1R
8119338	-0,71	-1,64	0,02265	-0,49	-1,41	0,19261	NM_002062	CYP2C19
7929478	-0,71	-1,64	0,01170	-0,93	-1,90	0,01283	NM_000769	---
8165255	-0,71	-1,64	0,37968	-1,36	-2,57	0,00282	---	LOC100049716
7952950	-0,71	-1,64	0,22104	1,80	3,48	0,00445	ENST00000318291	C6orf155
8127502	-0,71	-1,64	0,08206	-0,41	-1,33	0,41001	NR_026807	TYSND1
7934114	-0,71	-1,64	0,03477	-0,06	-1,04	0,81392	NM_173555	FSIP2
8046836	-0,71	-1,64	0,02028	-0,93	-1,91	0,01068	BX648733	MSMB
7927529	-0,71	-1,64	0,01872	-0,79	-1,72	0,00210	NM_002443	---
8110629	-0,71	-1,64	0,04076	-0,98	-1,97	0,01449	---	PYHIN1
7906386	-0,71	-1,64	0,00716	-1,51	-2,84	0,00774	NM_152501	ALLC
8040053	-0,71	-1,64	0,04057	-1,18	-2,27	0,00155	NM_018436	IGLV6-57
8071642	-0,71	-1,64	0,30822	-1,11	-2,15	0,00003	ENST00000390285	GAFA3
8099807	-0,71	-1,64	0,07341	-0,96	-1,95	0,01575	AF220235	ACTBL2
8112198	-0,71	-1,64	0,20973	-0,50	-1,42	0,07970	NM_001017992	GAGE12G
8167577	-0,71	-1,64	0,54556	-1,89	-3,70	0,01873	NM_001098409	GAGE12G
8167584	-0,71	-1,64	0,54556	-1,89	-3,70	0,01873	NM_001098409	---
7935114	-0,71	-1,64	0,13090	-1,18	-2,26	0,01416	---	C13orf30
7968866	-0,71	-1,64	0,19338	-1,46	-2,74	0,00010	NM_182508	TAS2R39
8136844	-0,71	-1,64	0,24155	-1,12	-2,18	0,00178	NM_176881	LOC339524
7902808	-0,71	-1,64	0,02340	-0,51	-1,42	0,02067	NR_026989	ASZ1
8142480	-0,71	-1,64	0,05314	-0,63	-1,55	0,02562	NR_023315	TP53I3
8050702	-0,71	-1,64	0,07241	0,16	1,12	0,60475	NM_004881	C2orf51
8043407	-0,71	-1,64	0,07693	-1,31	-2,47	0,00533	NM_152670	CHIA
7903945	-0,72	-1,64	0,04400	-0,88	-1,84	0,01808	NM_201653	RDH12
7975284	-0,72	-1,64	0,21702	-1,38	-2,60	0,00044	NM_152443	APBA1
8161618	-0,72	-1,64	0,08536	-0,21	-1,16	0,41111	NM_001163	CCDC137
8010629	-0,72	-1,64	0,20201	-0,56	-1,48	0,24018	NM_199287	CENPW
8121911	-0,72	-1,64	0,28353	-0,49	-1,41	0,08821	NM_001012507	KDR
8100393	-0,72	-1,64	0,02515	-0,66	-1,58	0,04281	NM_002253	C5orf39
8111925	-0,72	-1,64	0,07009	-1,08	-2,11	0,01027	NM_001014279	OR5T1
7939975	-0,72	-1,64	0,11283	-0,69	-1,62	0,00241	NM_001004745	SLC39A4
8153762	-0,72	-1,64	0,09366	-1,02	-2,02	0,00556	NM_017767	CNTFR
8160823	-0,72	-1,64	0,27234	-1,22	-2,33	0,00036	NM_147164	---
7988091	-0,72	-1,64	0,15369	-1,42	-2,67	0,00036	---	YDJC
8074773	-0,72	-1,64	0,10337	0,25	1,19	0,74110	NM_001017964	C6orf165
8121015	-0,72	-1,64	0,21593	-0,68	-1,60	0,00773	NM_001031743	---
7944832	-0,72	-1,64	0,01576	-1,56	-2,94	0,00548	---	PWWP2A
8115580	-0,72	-1,64	0,01688	-0,12	-1,08	0,85802	NM_052927	EFHB
8085732	-0,72	-1,64	0,00173	-0,42	-1,34	0,01961	NM_144715	ATP8B5P
8155026	-0,72	-1,64	0,02259	-0,65	-1,57	0,02762	NR_003581	GBP7
7917548	-0,72	-1,64	0,09574	-0,77	-1,71	0,00520	NM_207398	CGN
7905406	-0,72	-1,64	0,08960	0,34	1,27	0,46453	NM_020770	SUSD4
7924508	-0,72	-1,64	0,05966	-0,51	-1,43	0,10711	NM_017982	OCEL1
8026687	-0,72	-1,64	0,14629	0,64	1,56	0,20716	NM_024578	---
8170286	-0,72	-1,64	0,04849	-0,95	-1,93	0,09314	---	OR2T10
7925751	-0,72	-1,64	0,04984	-1,04	-2,05	0,00061	NM_001004693	FCGR1B
7919133	-0,72	-1,64	0,02518	-1,36	-2,56	0,00186	NM_001017986	---
8117628	-0,72	-1,64	0,17903	-0,92	-1,89	0,03586	---	WNK4
8007363	-0,72	-1,64	0,03471	-1,20	-2,30	0,18375	NM_032387	STATH
8095422	-0,72	-1,64	0,00267	-0,88	-1,85	0,02166	NM_003154	RP11-408E5.4
7967876	-0,72	-1,64	0,17635	-1,66	-3,15	0,00424	ENST00000382988	---
8103409	-0,72	-1,64	0,00757	-1,21	-2,32	0,05448	---	TAS2R31
7961291	-0,72	-1,64	0,11503	-0,83	-1,78	0,01927	NM_176885	HDAC11
8077958	-0,72	-1,64	0,03231	0,74	1,67	0,07437	NM_024827	ZNF443
8034393	-0,72	-1,64	0,17408	-1,27	-2,41	0,01060	NM_005815	---
8089986	-0,72	-1,64	0,03403	-1,07	-2,10	0,01229	---	---
8080509	-0,72	-1,64	0,05034	-2,09	-4,27	0,00422	---	SCRT1
8153937	-0,72	-1,64	0,15969	-1,15	-2,22	0,01374	NM_031309	PRAMEF3
7912585	-0,72	-1,64	0,12318	-2,06	-4,17	0,00078	NM_001013692	GABRG2
8109677	-0,72	-1,64	0,15204	-1,22	-2,33	0,02208	NM_198904	WNT8B
7929822	-0,72	-1,64	0,01827	-1,29	-2,45	0,02107	NM_003393	---
8169441	-0,72	-1,64	0,11152	-0,54	-1,45	0,04795	---	---

7931152	-0,72	-1,64	0,06313	-1,52	-2,87	0,03510	---	OR2J3
8117718	-0,72	-1,64	0,51951	-1,40	-2,64	0,06512	NM_001005216	DEFB119
8065555	-0,72	-1,64	0,14381	-1,46	-2,76	0,02549	NM_173460	ST8SIA3
8021349	-0,72	-1,64	0,04053	-0,92	-1,89	0,11376	NM_015879	---
8008817	-0,72	-1,64	0,21580	-1,70	-3,26	0,05024	---	TEKT4
8053753	-0,72	-1,65	0,35580	-0,55	-1,47	0,33205	NM_144705	---
8020760	-0,72	-1,65	0,23302	-1,10	-2,14	0,06592	---	OR8J1
7939981	-0,72	-1,65	0,09248	-1,37	-2,58	0,00015	NM_001005205	MTNR1A
8104074	-0,72	-1,65	0,08592	-0,17	-1,12	0,32610	NM_005958	MYH4
8012696	-0,72	-1,65	0,01307	-0,89	-1,86	0,00609	NM_017533	SYT10
7962226	-0,72	-1,65	0,09063	-1,29	-2,45	0,00374	NM_198992	LHX4
7907849	-0,72	-1,65	0,10008	-1,11	-2,17	0,01847	NM_033343	NOBOX
8143635	-0,72	-1,65	0,07652	-1,44	-2,72	0,04312	NM_001080413	---
8136078	-0,72	-1,65	0,42899	-0,60	-1,51	0,44468	---	COL28A1
8138157	-0,72	-1,65	0,11417	-0,52	-1,44	0,20309	NM_001037763	---
8105605	-0,72	-1,65	0,14096	-0,80	-1,74	0,00126	---	DSG3
8020762	-0,72	-1,65	0,18899	-0,60	-1,52	0,01036	NM_001944	---
8130011	-0,72	-1,65	0,15511	-0,86	-1,81	0,00053	---	ARHGAP36
8169931	-0,72	-1,65	0,07253	-1,89	-3,70	0,00087	NM_144967	LOC100130857
7982339	-0,72	-1,65	0,15671	-1,19	-2,27	0,00408	AK097050	FAM176A
8053278	-0,72	-1,65	0,00818	-1,58	-2,98	0,00221	NM_001135032	HOXA13
8138770	-0,72	-1,65	0,03535	-1,53	-2,89	0,00061	NM_000522	XAGE2
8167693	-0,72	-1,65	0,14742	-0,86	-1,81	0,00474	NM_130777	XAGE2
8172749	-0,72	-1,65	0,14742	-0,86	-1,81	0,00474	NM_130777	---
7904693	-0,72	-1,65	0,27392	-0,72	-1,64	0,13070	---	MIR32
8163107	-0,72	-1,65	0,01118	-0,44	-1,35	0,20483	NR_029506	TMEM20
7929383	-0,72	-1,65	0,21100	1,45	2,74	0,00232	NM_001134658	---
7956747	-0,72	-1,65	0,33313	-0,87	-1,82	0,06518	---	INTS5
7948772	-0,72	-1,65	0,09648	-0,39	-1,31	0,27135	NM_030628	---
8050472	-0,72	-1,65	0,07335	-0,68	-1,61	0,01405	---	MGC15705
8072484	-0,72	-1,65	0,09466	-0,87	-1,83	0,03344	ENST00000425084	---
8100754	-0,72	-1,65	0,14248	-1,36	-2,58	0,00060	---	ADAMTS20
7962384	-0,72	-1,65	0,03844	-1,24	-2,36	0,00046	NM_025003	UBL4A
8176098	-0,72	-1,65	0,03458	0,51	1,42	0,21705	NM_014235	NFKBIL1
8118127	-0,72	-1,65	0,05673	-0,27	-1,20	0,42623	NM_005007	NFKBIL1
8177967	-0,72	-1,65	0,05673	-0,27	-1,20	0,42623	NM_005007	NFKBIL1
8179249	-0,72	-1,65	0,05673	-0,27	-1,20	0,42623	NM_005007	---
8108399	-0,72	-1,65	0,02149	-1,58	-2,99	0,00095	---	---
8083467	-0,72	-1,65	0,08320	-0,72	-1,65	0,00316	---	CPNE6
7973510	-0,72	-1,65	0,35758	-1,79	-3,46	0,00250	NM_006032	---
7907968	-0,72	-1,65	0,06789	-0,52	-1,43	0,00070	---	TTTY11
8177085	-0,72	-1,65	0,20631	-0,90	-1,87	0,01023	NR_001548	---
7911349	-0,72	-1,65	0,11065	-0,72	-1,64	0,00777	---	CDY1
8176926	-0,72	-1,65	0,13843	-1,39	-2,62	0,01062	NM_170723	CDY1
8177405	-0,72	-1,65	0,13843	-1,39	-2,62	0,01062	NM_170723	TNFRSF13C
8076387	-0,72	-1,65	0,04031	-1,17	-2,25	0,00200	NM_052945	ELFN2
8075921	-0,72	-1,65	0,00954	-0,63	-1,55	0,04842	NM_052906	NRCAM
8142270	-0,72	-1,65	0,04103	-0,88	-1,84	0,00093	NM_001037132	PPIEL
7915204	-0,72	-1,65	0,10302	-1,37	-2,58	0,00244	AK093659	PCDHB12
8108733	-0,72	-1,65	0,16678	-1,45	-2,73	0,02838	NM_018932	MVK
7958565	-0,72	-1,65	0,09179	-0,32	-1,24	0,40390	NM_000431	BPI
8062444	-0,72	-1,65	0,00178	-1,26	-2,40	0,00274	NM_001725	TLR9
8087852	-0,72	-1,65	0,09137	-1,29	-2,44	0,07727	NM_017442	DPF3
7979963	-0,72	-1,65	0,05817	-1,61	-3,05	0,02168	NM_012074	FGD5
8078066	-0,72	-1,65	0,07156	-1,49	-2,81	0,00720	NM_152536	RRP9
8087790	-0,72	-1,65	0,14938	0,75	1,68	0,05191	NM_004704	SLC24A1
7984276	-0,72	-1,65	0,12979	0,94	1,92	0,09269	NM_004727	---
8129037	-0,72	-1,65	0,00424	-0,65	-1,57	0,10269	---	---
8091560	-0,72	-1,65	0,19270	-0,61	-1,53	0,00016	---	OR8D2
7952394	-0,72	-1,65	0,14844	-0,99	-1,99	0,00165	NM_001002918	PRSS37
8143463	-0,72	-1,65	0,00082	-1,02	-2,02	0,03129	NM_001008270	THSD1
7971813	-0,72	-1,65	0,03467	-0,75	-1,68	0,00777	NM_018676	C1orf86
7911683	-0,72	-1,65	0,08164	-1,07	-2,09	0,04465	NM_182533	PHLDB2
8089472	-0,72	-1,65	0,13904	-0,73	-1,66	0,00303	NM_001134438	UNC13A
8035318	-0,72	-1,65	0,04563	-1,25	-2,38	0,00069	NM_001080421	KIAA0748
7963851	-0,72	-1,65	0,02875	-0,47	-1,38	0,02303	NM_001098815	TRIM7
8116504	-0,72	-1,65	0,14614	-0,13	-1,09	0,76864	NM_203293	FAM123C
8045224	-0,72	-1,65	0,10880	-0,94	-1,92	0,03108	NM_152698	---
8027341	-0,72	-1,65	0,19691	-0,98	-1,97	0,00052	---	RASD2
8072705	-0,72	-1,65	0,20060	0,47	1,38	0,09163	NM_014310	TAGLN3
8081620	-0,72	-1,65	0,11167	-0,70	-1,63	0,17045	NM_013259	SDR9C7
7964285	-0,72	-1,65	0,17191	-1,46	-2,76	0,00356	NM_148897	HYDIN
8002446	-0,72	-1,65	0,13440	-1,35	-2,56	0,00227	NM_032821	---

7944300	-0,72	-1,65	0,05412	-0,88	-1,84	0,07957	---	---
8048887	-0,72	-1,65	0,01304	-0,72	-1,65	0,09228	---	LYPLA2
7898902	-0,72	-1,65	0,13556	-0,41	-1,33	0,41510	NM_007260	DENND2C
7918755	-0,72	-1,65	0,09544	-1,06	-2,08	0,01526	BC063894	TBC1D3B
8014376	-0,72	-1,65	0,32399	1,23	2,35	0,07475	NM_001001417	TMEM155
8102636	-0,72	-1,65	0,03105	-1,00	-2,01	0,04091	NM_152399	---
7996759	-0,72	-1,65	0,04495	-0,61	-1,53	0,05063	---	VASH1
7975889	-0,72	-1,65	0,07024	-0,05	-1,04	0,80186	NM_014909	CCKAR
8099746	-0,72	-1,65	0,03082	-1,49	-2,81	0,04487	NM_000730	ABCC11
8001271	-0,72	-1,65	0,14661	-0,59	-1,51	0,18873	NM_033151	C19orf22
8032127	-0,72	-1,65	0,05950	1,19	2,28	0,13371	NM_138774	---
8081429	-0,72	-1,65	0,25220	-0,53	-1,44	0,06258	---	EPHB3
8084524	-0,72	-1,65	0,02603	0,09	1,06	0,84292	NM_004443	SH2D3A
8033319	-0,72	-1,65	0,02501	0,07	1,05	0,91457	NM_005490	SLC30A10
7924342	-0,72	-1,65	0,10434	-0,61	-1,52	0,17193	NM_018713	EFCAB2
7911112	-0,72	-1,65	0,24265	-0,93	-1,91	0,00019	NR_026586	SNORA21
8014755	-0,72	-1,65	0,00624	0,39	1,31	0,05969	NR_002576	CAMKK1
8011480	-0,72	-1,65	0,04183	-0,48	-1,39	0,27282	NM_032294	IER3IP1
8021147	-0,72	-1,65	0,12955	-0,47	-1,38	0,24328	NM_016097	BCMO1
7997401	-0,72	-1,65	0,02724	-0,76	-1,70	0,01248	NM_017429	PRSS53
8000978	-0,72	-1,65	0,03642	-1,14	-2,20	0,00219	NM_001039503	OR4K13
7977478	-0,72	-1,65	0,17890	-1,46	-2,75	0,04446	NM_001004714	OVCH1
7962029	-0,72	-1,65	0,10602	-1,04	-2,06	0,15813	NM_183378	---
8047465	-0,72	-1,65	0,03758	-0,71	-1,63	0,05425	---	FAM123A
7970648	-0,72	-1,65	0,25951	-1,21	-2,31	0,00505	NM_152704	FSD2
7991019	-0,72	-1,65	0,14262	-1,16	-2,23	0,00537	NM_001007122	FAM113B
7955045	-0,72	-1,65	0,05055	0,32	1,25	0,33749	BC008360	TLR10
8099826	-0,72	-1,65	0,05751	-1,10	-2,15	0,01696	NM_030956	---
7990377	-0,72	-1,65	0,04259	-0,55	-1,46	0,00081	---	---
8122858	-0,72	-1,65	0,00752	-1,68	-3,21	0,00637	---	ANKRD62
8074201	-0,73	-1,65	0,08651	-0,47	-1,39	0,00009	ENST00000418274	RPRM
8055941	-0,73	-1,65	0,07417	-1,14	-2,21	0,09254	NM_019845	FLJ33360
8110882	-0,73	-1,65	0,04498	-0,94	-1,92	0,00823	NR_028351	OR1D4
8003806	-0,73	-1,65	0,68371	-2,70	-6,52	0,01852	NM_003552	SYT16
7974882	-0,73	-1,65	0,24298	-1,40	-2,64	0,01158	NM_031914	BICD2
8162438	-0,73	-1,65	0,06918	1,46	2,75	0,02041	NM_001003800	ATG4B
8049888	-0,73	-1,65	0,12623	1,27	2,42	0,06511	NM_013325	OR52N4
7938053	-0,73	-1,65	0,18660	-0,60	-1,52	0,07416	NM_001005175	C19orf34
8032375	-0,73	-1,65	0,07378	-0,67	-1,59	0,10165	NR_033400	---
8168553	-0,73	-1,65	0,04318	-0,46	-1,38	0,12162	---	---
7979042	-0,73	-1,65	0,02031	-2,28	-4,86	0,02192	---	KRTAP12-2
8070791	-0,73	-1,65	0,23465	-1,62	-3,07	0,00050	NM_181684	---
7931928	-0,73	-1,65	0,01344	-0,89	-1,86	0,00214	---	---
8116662	-0,73	-1,65	0,02731	-0,48	-1,40	0,28610	---	DNAH12
8088315	-0,73	-1,65	0,06422	-0,53	-1,44	0,01219	NM_178504	POLD4
7949746	-0,73	-1,65	0,10121	1,50	2,83	0,01294	NM_021173	---
8156024	-0,73	-1,65	0,11784	-1,23	-2,35	0,00698	---	MAL2
8148040	-0,73	-1,65	0,17520	4,97	31,36	0,00276	NM_052886	---
8029615	-0,73	-1,65	0,16884	-0,90	-1,86	0,01086	---	DMC1
8076094	-0,73	-1,65	0,14399	-0,87	-1,83	0,00096	NM_007068	---
8069768	-0,73	-1,65	0,06919	-2,09	-4,25	0,00507	---	LRRC53
7917015	-0,73	-1,65	0,09550	-0,70	-1,63	0,02694	ENST00000416014	TRAIP
8087513	-0,73	-1,65	0,06472	0,42	1,34	0,01413	NM_005879	---
7904715	-0,73	-1,65	0,08711	-2,67	-6,38	0,00041	---	GJB7
8128007	-0,73	-1,65	0,08483	1,78	3,43	0,01035	NM_198568	DLX6
8134407	-0,73	-1,65	0,28950	-0,79	-1,73	0,03673	NM_005222	SYNPR
8080855	-0,73	-1,65	0,01444	-0,60	-1,52	0,00246	NM_144642	---
8095297	-0,73	-1,65	0,01067	-1,40	-2,65	0,01033	---	---
7948367	-0,73	-1,65	0,16375	-0,41	-1,32	0,09060	---	MN1
8075126	-0,73	-1,65	0,04742	0,02	1,01	0,94007	NM_002430	GPR88
7903355	-0,73	-1,65	0,47575	-2,31	-4,95	0,00086	NM_022049	FDX1L
8033960	-0,73	-1,65	0,01283	0,67	1,59	0,11732	NM_001031734	JAG2
7981538	-0,73	-1,65	0,02803	1,62	3,06	0,11786	NM_002226	PNMA1
7980044	-0,73	-1,66	0,03245	1,37	2,59	0,07557	NM_006029	---
8051394	-0,73	-1,66	0,10437	-1,26	-2,39	0,00145	---	---
8081113	-0,73	-1,66	0,15888	-1,03	-2,05	0,01410	---	GPR44
7948470	-0,73	-1,66	0,35922	-1,69	-3,23	0,00595	NM_004778	---
8083320	-0,73	-1,66	0,13661	-0,28	-1,22	0,03017	---	---
7932306	-0,73	-1,66	0,33461	-1,40	-2,64	0,01564	---	---
8076513	-0,73	-1,66	0,07991	-1,72	-3,30	0,00217	---	---
8117161	-0,73	-1,66	0,02184	-1,20	-2,29	0,00207	---	HRAS
7945436	-0,73	-1,66	0,04124	-0,77	-1,71	0,11797	NM_176795	CT45A2
8170060	-0,73	-1,66	0,23024	-1,65	-3,14	0,00412	NM_152582	---

8094257	-0,73	-1,66	0,14186	-0,96	-1,95	0,02938	---	SPEM1
8004394	-0,73	-1,66	0,04869	-0,61	-1,52	0,06867	NM_199339	GLIPR1L2
7957253	-0,73	-1,66	0,01255	0,04	1,03	0,83147	NM_152436	SKCG-1
7943707	-0,73	-1,66	0,08563	-0,67	-1,59	0,04672	AY662656	ITGAL
7994826	-0,73	-1,66	0,03961	-1,28	-2,43	0,00006	NM_002209	---
8061165	-0,73	-1,66	0,19909	-1,74	-3,34	0,02864	---	---
7962238	-0,73	-1,66	0,33970	-2,07	-4,21	0,00456	---	MAGIX
8167443	-0,73	-1,66	0,06644	-0,96	-1,95	0,07318	NM_024859	C6orf105
8123951	-0,73	-1,66	0,00337	-0,33	-1,26	0,16424	NM_001143948	SLC5A5
8026877	-0,73	-1,66	0,02098	-0,77	-1,70	0,01230	NM_000453	LOC554249
8155393	-0,73	-1,66	0,07474	0,40	1,32	0,06076	AK292642	LOC554249
8161451	-0,73	-1,66	0,07474	0,40	1,32	0,06076	AK292642	TNFRSF17
7993267	-0,73	-1,66	0,00895	-1,35	-2,54	0,04627	NM_001192	C6orf59
8130641	-0,73	-1,66	0,09851	-1,07	-2,10	0,00372	NR_024277	---
8084215	-0,73	-1,66	0,20352	-1,15	-2,22	0,10760	---	CD8B
8053594	-0,73	-1,66	0,06344	-1,30	-2,47	0,02199	NM_172101	C2orf68
8053458	-0,73	-1,66	0,15907	-1,15	-2,21	0,02171	NM_001013649	H2AFX
7952179	-0,73	-1,66	0,31882	0,51	1,43	0,17859	NM_002105	FLJ16734
8137483	-0,73	-1,66	0,15062	-1,74	-3,35	0,00551	AK131514	ATP12A
7968062	-0,73	-1,66	0,08689	-0,18	-1,13	0,24550	NM_001676	DUOXA2
7983405	-0,73	-1,66	0,00588	-0,63	-1,54	0,05844	NM_207581	ANO9
7945394	-0,73	-1,66	0,04308	1,67	3,18	0,08491	NM_001012302	WDFY4
7927425	-0,73	-1,66	0,06256	-1,14	-2,20	0,00010	NM_020945	---
7971669	-0,73	-1,66	0,04578	-1,06	-2,08	0,00010	---	ANKRD58
8169657	-0,73	-1,66	0,21171	-1,80	-3,47	0,01617	NM_001105576	DUSP28
8049684	-0,73	-1,66	0,04355	0,21	1,16	0,48945	NM_001033575	MYO15B
8009913	-0,73	-1,66	0,11245	-0,42	-1,33	0,10637	NR_003587	---
8068044	-0,73	-1,66	0,12989	-1,11	-2,16	0,00302	---	PRSS29P
7998444	-0,73	-1,66	0,17550	-1,36	-2,57	0,00034	ENST00000440800	---
7955168	-0,73	-1,66	0,09069	-1,32	-2,50	0,01401	---	CHMP6
8010550	-0,73	-1,66	0,03906	0,56	1,47	0,20188	NM_024591	MIR181B1
7923173	-0,73	-1,66	0,05810	-0,54	-1,45	0,03603	NR_029612	OR8B3
7952400	-0,73	-1,66	0,01203	-1,28	-2,42	0,00499	NM_001005467	LRRIQ3
7917004	-0,73	-1,66	0,16148	-1,09	-2,13	0,01701	NM_001105659	HECW2
8057898	-0,73	-1,66	0,10039	0,10	1,07	0,64301	NM_020760	PMVK
7920567	-0,73	-1,66	0,08408	-0,49	-1,40	0,06840	NM_006556	TRAJ17
7973221	-0,73	-1,66	0,01910	-1,15	-2,21	0,00107	AK301287	ELAVL4
7901342	-0,73	-1,66	0,16059	-1,04	-2,06	0,00305	NM_021952	TIMD4
8115445	-0,73	-1,66	0,11931	-0,67	-1,59	0,01129	NM_138379	NRL
7978107	-0,73	-1,66	0,00898	-1,32	-2,49	0,00441	NM_006177	IL9
8114256	-0,73	-1,66	0,07701	-0,91	-1,88	0,01597	NM_000590	SLC1A2
7947496	-0,73	-1,66	0,22936	-0,86	-1,81	0,05487	NM_004171	---
8087746	-0,73	-1,66	0,07620	-0,22	-1,17	0,57323	---	ATP5D
8024219	-0,73	-1,66	0,05467	0,56	1,48	0,29065	NM_001687	GYS2
7961673	-0,73	-1,66	0,02138	-0,85	-1,80	0,00388	NM_021957	NHP2
8116168	-0,73	-1,66	0,29079	1,99	3,98	0,00442	NM_017838	MBD3L1
8025452	-0,73	-1,66	0,08757	-0,88	-1,84	0,08833	NM_145208	FMR1NB
8170353	-0,73	-1,66	0,12177	-0,77	-1,70	0,04553	NM_152578	HPCAL4
7915245	-0,73	-1,66	0,07853	-0,38	-1,30	0,09068	NM_016257	---
7939070	-0,73	-1,66	0,02108	-0,74	-1,67	0,00269	---	LOC146880
8017671	-0,73	-1,66	0,15415	-0,01	-1,00	0,99542	NR_027487	ZNF770
7982527	-0,73	-1,66	0,01220	-0,37	-1,30	0,26435	NM_014106	SORCS3
7930341	-0,73	-1,66	0,17669	-0,62	-1,53	0,01128	NM_014978	SIAH3
7971419	-0,73	-1,66	0,03461	-1,64	-3,12	0,06558	NM_198849	RXRG
7921955	-0,73	-1,66	0,24340	-1,54	-2,91	0,00250	NM_006917	ELN
8133372	-0,73	-1,66	0,05622	-1,13	-2,20	0,00469	NM_000501	AVPR1A
7964660	-0,73	-1,66	0,14421	-1,33	-2,51	0,00047	NM_000706	---
7994350	-0,73	-1,66	0,22559	-1,15	-2,21	0,01941	---	PNLIP
7930777	-0,73	-1,66	0,11917	-0,70	-1,62	0,00726	NM_000936	RUNDC2C
7993825	-0,73	-1,66	0,08299	0,03	1,02	0,83748	NR_002939	---
7920045	-0,73	-1,66	0,02312	-0,71	-1,63	0,00197	---	FAM115A
8143597	-0,73	-1,66	0,01719	-1,47	-2,77	0,02496	ENST00000307248	GCG
8056250	-0,73	-1,66	0,11627	-0,89	-1,86	0,01463	NM_002054	SERPINB5
8021584	-0,73	-1,66	0,01855	0,42	1,33	0,06751	NM_002639	LOC100133130
7933261	-0,73	-1,66	0,00679	-0,73	-1,66	0,02860	ENST00000317346	LOC100133130
7933572	-0,73	-1,66	0,00679	-0,73	-1,66	0,02860	ENST00000317346	---
7927924	-0,73	-1,66	0,01080	-0,90	-1,87	0,00817	---	GPR148
8045216	-0,73	-1,66	0,12660	-1,34	-2,53	0,01031	NM_207364	---
7996569	-0,73	-1,66	0,19834	0,08	1,06	0,85287	---	ANKRD24
8024768	-0,73	-1,66	0,19756	-1,06	-2,09	0,03587	NM_133475	DAPP1
8096602	-0,73	-1,66	0,04253	0,51	1,42	0,10348	NM_014395	---
8061517	-0,73	-1,66	0,01473	-1,44	-2,72	0,00000	---	TMEM75
8152815	-0,73	-1,66	0,20301	-0,85	-1,80	0,02933	BC137383	---

8045537	-0,73	-1,66	0,00029	-1,11	-2,17	0,01408	---	---
8173364	-0,73	-1,66	0,34763	-0,85	-1,80	0,08751	---	SCAND1
8066031	-0,73	-1,66	0,06126	-1,44	-2,71	0,01778	NM_016558	OR2W3
7911229	-0,73	-1,66	0,09818	-0,74	-1,67	0,09351	NM_001001957	OR2W3
7911231	-0,73	-1,66	0,09818	-0,74	-1,67	0,09351	NM_001001957	PSD
7935990	-0,73	-1,66	0,03530	-0,43	-1,35	0,36491	NM_002779	AFG3L1
7998083	-0,73	-1,66	0,15067	-0,40	-1,32	0,16498	NR_003228	ANKRD31
8112672	-0,73	-1,66	0,01583	-0,35	-1,27	0,18664	NM_001164443	JMJD5
7994269	-0,73	-1,66	0,23179	-0,64	-1,56	0,01748	NM_024773	SCNN1B
7994074	-0,73	-1,66	0,13797	-1,15	-2,21	0,00505	NM_000336	EPCAM
8041853	-0,73	-1,66	0,00064	5,74	53,51	0,00047	NM_002354	C17orf44
8012416	-0,73	-1,66	0,05734	-0,77	-1,71	0,01847	NR_026951	---
8104441	-0,73	-1,66	0,07579	-1,89	-3,70	0,01113	---	PSMB8
8125500	-0,73	-1,66	0,10548	-0,14	-1,10	0,79808	NM_004159	PSMB8
8178855	-0,73	-1,66	0,10548	-0,14	-1,10	0,79808	NM_004159	PSMB8
8180049	-0,73	-1,66	0,10548	-0,14	-1,10	0,79808	NM_004159	CCDC85A
8042079	-0,73	-1,66	0,01298	-1,25	-2,38	0,01934	NM_001080433	ZNHIT2
7949373	-0,73	-1,66	0,23475	-1,34	-2,53	0,17475	NM_014205	AMIGO3
8087453	-0,73	-1,66	0,14041	-0,08	-1,06	0,87580	NM_198722	VN1R3
8001064	-0,73	-1,66	0,10934	-0,86	-1,81	0,11390	BC107074	---
7968000	-0,73	-1,66	0,00063	-1,09	-2,13	0,01681	---	PEX26
8071136	-0,73	-1,66	0,03980	0,71	1,64	0,13914	NM_001127649	---
8168689	-0,73	-1,66	0,00280	-0,79	-1,73	0,00242	---	KCNJ13
8059776	-0,73	-1,66	0,08317	-0,70	-1,63	0,01372	NM_002242	SEC14L4
8072355	-0,73	-1,66	0,07009	-0,81	-1,76	0,00795	NM_174977	AGAP3
8137381	-0,73	-1,66	0,13444	0,26	1,19	0,55060	NM_031946	ELF5
7947481	-0,73	-1,66	0,08830	-1,67	-3,18	0,00428	NM_198381	---
7939938	-0,73	-1,66	0,15640	-1,98	-3,93	0,00139	---	ATP1A2
7906501	-0,73	-1,66	0,24536	-0,40	-1,32	0,15359	NM_000702	PADI6
7898466	-0,73	-1,66	0,08282	-0,57	-1,48	0,18191	NM_207421	CYTH4
8072798	-0,73	-1,66	0,09336	-0,62	-1,53	0,07554	NM_013385	IL1F9
8044541	-0,73	-1,66	0,03495	-2,07	-4,20	0,00513	NM_019618	---
8053646	-0,73	-1,66	0,13166	-0,70	-1,63	0,04278	---	DNAJC6
7902043	-0,73	-1,66	0,04547	0,95	1,93	0,06657	NM_014787	PGA5
7940441	-0,73	-1,66	0,26854	-1,41	-2,66	0,03651	NM_014224	EXPH5
7951545	-0,73	-1,66	0,04505	2,52	5,75	0,00406	NM_015065	AKD1
8128788	-0,73	-1,66	0,08341	-1,01	-2,01	0,01468	NM_001145128	TTC28
8072133	-0,73	-1,66	0,03618	-1,23	-2,34	0,02127	NM_001145418	LOC149620
7903935	-0,73	-1,66	0,15384	-1,03	-2,04	0,00003	NR_003928	---
8079588	-0,74	-1,66	0,00143	-1,56	-2,96	0,00170	---	FZD5
8058498	-0,74	-1,66	0,12264	1,25	2,39	0,00393	NM_003468	---
8009349	-0,74	-1,66	0,08369	-1,02	-2,02	0,02116	---	C12orf43
7967109	-0,74	-1,66	0,01639	-0,06	-1,04	0,81685	BC014661	KRTAP10-5
8070782	-0,74	-1,66	0,06164	-0,51	-1,43	0,16070	NM_198694	ACOT7
7912012	-0,74	-1,66	0,01821	-0,98	-1,97	0,05066	NM_007274	---
7972334	-0,74	-1,67	0,01951	-1,24	-2,36	0,00036	---	---
8100756	-0,74	-1,67	0,38679	-2,50	-5,64	0,00025	---	ZNF496
7925705	-0,74	-1,67	0,03361	-0,23	-1,17	0,60076	NM_032752	CADM2
8081036	-0,74	-1,67	0,09496	-1,08	-2,12	0,01623	NM_001167674	---
8164808	-0,74	-1,67	0,12037	-1,70	-3,24	0,01271	---	C4orf40
8095451	-0,74	-1,67	0,08175	-1,51	-2,84	0,00187	NM_214711	MAK
8123893	-0,74	-1,67	0,05962	-0,87	-1,83	0,01367	NM_005906	SLC22A6
7948920	-0,74	-1,67	0,19103	-1,05	-2,07	0,00470	NM_004790	CALB1
8151730	-0,74	-1,67	0,07734	-1,56	-2,95	0,00482	NM_004929	SNORD12C
8063345	-0,74	-1,67	0,07567	-0,58	-1,50	0,00881	NR_002433	PRDM16
7897132	-0,74	-1,67	0,00604	-0,74	-1,67	0,02094	NM_022114	C22orf37
8071134	-0,74	-1,67	0,01158	-2,22	-4,65	0,00138	ENST00000444596	---
7930612	-0,74	-1,67	0,18781	-1,16	-2,23	0,01782	---	SNORD115-20
7982046	-0,74	-1,67	0,40518	-1,83	-3,56	0,00003	NR_003312	SAA3P
7946972	-0,74	-1,67	0,16350	-0,60	-1,52	0,04301	NR_026576	HEMK1
8080001	-0,74	-1,67	0,12571	0,30	1,23	0,24561	NM_016173	ZNF846
8033809	-0,74	-1,67	0,08506	-0,39	-1,31	0,16652	NM_001077624	C17orf54
8018156	-0,74	-1,67	0,01596	-0,89	-1,85	0,05013	AK097638	PPP1R12C
8039316	-0,74	-1,67	0,13194	-0,69	-1,61	0,08800	NM_017607	---
8054790	-0,74	-1,67	0,18370	-1,00	-2,01	0,02374	---	---
8154414	-0,74	-1,67	0,00997	-1,25	-2,38	0,00925	---	SLC16A10
8121515	-0,74	-1,67	0,34216	1,37	2,58	0,03707	NM_018593	CD83
8116983	-0,74	-1,67	0,10077	-0,22	-1,16	0,37901	NM_004233	---
7965650	-0,74	-1,67	0,09524	-0,78	-1,72	0,02287	---	WDR62
8028119	-0,74	-1,67	0,09248	0,55	1,47	0,06803	NM_001083961	PTH2R
8047910	-0,74	-1,67	0,06156	-1,19	-2,28	0,00000	NM_005048	DEFB4A
8144481	-0,74	-1,67	0,19051	-1,53	-2,88	0,00183	NM_004942	DEFB4A
8149169	-0,74	-1,67	0,19051	-1,53	-2,88	0,00183	NM_004942	OR10T2

7921354	-0,74	-1,67	0,02941	-0,95	-1,93	0,16485	NM_001004475	TRAPPC5
8025296	-0,74	-1,67	0,18295	1,27	2,41	0,02511	NM_174894	SPIRE2
7998015	-0,74	-1,67	0,04086	-0,16	-1,12	0,72771	NM_032451	GSTM2P1
8128890	-0,74	-1,67	0,07632	-1,44	-2,72	0,00117	NR_002932	C3orf79
8083463	-0,74	-1,67	0,24974	-0,76	-1,69	0,01950	NM_001101337	CENPH
8105842	-0,74	-1,67	0,13178	0,21	1,15	0,38300	NM_022909	---
8066029	-0,74	-1,67	0,00951	-0,89	-1,86	0,00287	---	OR52H1
7946087	-0,74	-1,67	0,20648	-1,55	-2,93	0,00064	NM_001005289	---
8054090	-0,74	-1,67	0,01547	-0,49	-1,40	0,01036	---	SPDYA
8041138	-0,74	-1,67	0,06965	-0,90	-1,86	0,01298	NM_182756	A1BG
8039748	-0,74	-1,67	0,41214	-0,24	-1,18	0,13347	NM_130786	C1orf116
7923958	-0,74	-1,67	0,16755	2,11	4,31	0,00234	NM_023938	FCRL5
7921237	-0,74	-1,67	0,16372	-1,24	-2,36	0,00866	NM_031281	LOC442132
8110916	-0,74	-1,67	0,48619	-1,62	-3,08	0,00933	ENST00000382550	ZNF48
7994813	-0,74	-1,67	0,05117	1,12	2,18	0,04412	NM_152652	FLJ14107
8149718	-0,74	-1,67	0,16225	-1,85	-3,60	0,01284	NR_027715	---
8064926	-0,74	-1,67	0,07208	-1,31	-2,48	0,00787	---	CCDC79
8001858	-0,74	-1,67	0,18350	-1,02	-2,02	0,00166	NM_001136505	HDAC5
8015914	-0,74	-1,67	0,04221	0,19	1,14	0,41414	NM_005474	---
7953685	-0,74	-1,67	0,00007	-1,28	-2,42	0,00347	---	---
8172658	-0,74	-1,67	0,05696	-1,33	-2,51	0,01222	---	GAL3ST1
8075401	-0,74	-1,67	0,13533	1,39	2,62	0,01393	NM_004861	HLA-DRB5
8125436	-0,74	-1,67	0,05262	-0,47	-1,39	0,08274	NM_002125	FAM98C
8028397	-0,74	-1,67	0,15332	0,37	1,29	0,40486	NM_174905	AHSA2
8042195	-0,74	-1,67	0,04624	1,23	2,34	0,02191	NM_152392	BAAT
8162870	-0,74	-1,67	0,12112	-1,24	-2,35	0,00254	NM_001701	TRIM67
7910503	-0,74	-1,67	0,25000	-1,76	-3,38	0,02185	NM_001004342	OR5AP2
7948155	-0,74	-1,67	0,06469	-0,93	-1,91	0,00037	NM_001002925	ANXA9
7905283	-0,74	-1,67	0,03267	-0,46	-1,37	0,02476	NM_003568	BOLA1
7905092	-0,74	-1,67	0,18473	1,68	3,21	0,02834	NM_016074	---
7906233	-0,74	-1,67	0,13774	-0,88	-1,85	0,25100	---	04. Sep
8017039	-0,74	-1,67	0,09062	-0,33	-1,26	0,05424	NM_080415	OR1N2
8157674	-0,74	-1,67	0,23118	-1,51	-2,85	0,07047	NM_001004457	---
8059714	-0,74	-1,67	0,36798	-0,70	-1,62	0,25738	---	---
7992954	-0,74	-1,67	0,11183	-0,72	-1,65	0,02832	---	FRAT2
7935421	-0,74	-1,67	0,03533	-1,08	-2,12	0,06188	NM_012083	ZMAT4
8150419	-0,74	-1,67	0,12781	-1,95	-3,87	0,00071	NM_024645	MIR494
7976834	-0,74	-1,67	0,01117	-0,94	-1,93	0,11600	NR_030174	STK32B
8093858	-0,74	-1,67	0,09732	-0,77	-1,71	0,00066	NM_018401	GUCA1C
8089402	-0,74	-1,67	0,08752	-1,01	-2,02	0,12679	NM_005459	BMP8A
7900340	-0,74	-1,67	0,07722	0,37	1,29	0,41851	NM_181809	RUNX3
7913805	-0,74	-1,67	0,12718	0,00	1,00	0,99634	NM_001031680	---
7968632	-0,74	-1,67	0,06388	-1,12	-2,17	0,00659	---	---
8094946	-0,74	-1,67	0,06822	-0,91	-1,88	0,01022	---	PTPRE
7931353	-0,74	-1,67	0,07138	2,09	4,26	0,01397	NM_006504	---
8019517	-0,74	-1,67	0,07596	-1,19	-2,28	0,00270	---	PLEKHG4B
8104141	-0,74	-1,67	0,22403	0,42	1,34	0,33703	NM_052909	ZNF527
8028241	-0,74	-1,67	0,14168	-0,98	-1,97	0,12272	NM_032453	---
8050687	-0,74	-1,67	0,10551	-0,77	-1,70	0,08660	---	---
8057557	-0,74	-1,67	0,24069	-0,73	-1,66	0,00092	---	TMPRSS5
7951717	-0,74	-1,67	0,00418	-1,15	-2,22	0,00192	NM_030770	FAAH
7901229	-0,74	-1,67	0,10708	0,17	1,12	0,75689	NM_001441	MMP7
7951217	-0,74	-1,67	0,00030	-1,16	-2,23	0,00446	NM_002423	C11orf68
7949540	-0,74	-1,67	0,13386	0,19	1,14	0,77729	NM_001135635	---
8067981	-0,74	-1,67	0,06268	-0,95	-1,93	0,00553	---	---
8133110	-0,74	-1,67	0,14789	-0,37	-1,29	0,39097	---	C10orf129
7929497	-0,74	-1,67	0,00051	-0,93	-1,91	0,01572	NM_207321	MYH13
8012625	-0,74	-1,67	0,04283	-0,97	-1,96	0,03021	NM_003802	IGKC // IGKC // IGKC // IGKC // IGKC // IGKC
8043465	-0,74	-1,67	0,27115	-1,39	-2,63	0,00159	BC029444 // BC029444 // BC029444 // BC029444 // BC029444 // BC029444	OR2L2
7911252	-0,74	-1,67	0,23107	-1,75	-3,37	0,03817	NM_001004686	KCNAB3
8012285	-0,74	-1,67	0,00346	-0,76	-1,69	0,04414	NM_004732	ERLIN2
8150214	-0,74	-1,67	0,19562	-0,58	-1,49	0,21988	NM_007175	---
8009073	-0,74	-1,67	0,17737	-0,98	-1,97	0,02953	---	ZNF653
8034276	-0,74	-1,67	0,03331	-0,67	-1,59	0,07364	NM_138783	OR10H2
8026483	-0,74	-1,67	0,18963	-1,24	-2,36	0,01807	NM_013939	GCNT7
8067178	-0,74	-1,67	0,19206	-0,90	-1,86	0,13370	NM_080615	TYRP1
8154367	-0,74	-1,67	0,16882	-0,66	-1,57	0,02012	NM_000550	NCAN
8027152	-0,74	-1,67	0,16169	-0,81	-1,75	0,02405	NM_004386	LRRC67
8151127	-0,74	-1,67	0,04408	-0,91	-1,88	0,00723	NM_001013626	TRAV8-3
7973298	-0,74	-1,67	0,11122	-1,21	-2,32	0,00040	X58769	CASQ2
7918878	-0,74	-1,67	0,28239	-1,23	-2,34	0,01161	NM_001232	TMEM95

8004364	-0,74	-1,67	0,07682	-0,86	-1,81	0,04622	NM_198154	ZDHHC8P1
8074892	-0,74	-1,67	0,16321	-1,70	-3,25	0,00923	NR_003950	MIR211
7987097	-0,74	-1,67	0,02600	-1,37	-2,59	0,01180	NR_029624	SLAMF8
7906486	-0,74	-1,67	0,03125	-1,00	-1,99	0,00318	NM_020125	---
8065013	-0,74	-1,67	0,08081	-1,27	-2,41	0,02814	---	PSMB11
7973369	-0,74	-1,67	0,20152	-0,83	-1,78	0,06352	NM_001099780	ABCA11P
8098752	-0,74	-1,67	0,03330	-0,84	-1,80	0,01028	NR_002451	---
7934568	-0,74	-1,67	0,00092	-1,00	-2,00	0,00042	---	---
7916018	-0,74	-1,67	0,02425	-0,86	-1,81	0,02726	---	---
8146892	-0,74	-1,67	0,09016	-1,33	-2,52	0,00121	---	LOC100133315
7950082	-0,74	-1,67	0,12968	2,65	6,30	0,00006	NR_029192	SUMO1P1
8067121	-0,74	-1,67	0,02245	-0,32	-1,25	0,26028	NR_002189	ZBTB11
8081358	-0,74	-1,67	0,22848	-0,65	-1,56	0,08339	NM_014415	RAB39B
8176230	-0,74	-1,67	0,15898	-0,13	-1,09	0,61362	NM_171998	---
8103892	-0,74	-1,67	0,08876	-0,48	-1,40	0,02845	---	FBXO15
8023843	-0,74	-1,67	0,17774	-0,34	-1,27	0,13584	NM_152676	POLE
7967736	-0,74	-1,67	0,03045	1,83	3,55	0,00080	NM_006231	SNORD115-37
7982080	-0,74	-1,68	0,03831	-0,90	-1,86	0,00160	NR_003352	DPPA4
8089448	-0,74	-1,68	0,04500	-1,03	-2,05	0,00720	NM_018189	SSX5
8172387	-0,74	-1,68	0,08137	-0,59	-1,51	0,02820	NM_021015	MST1P2
7898357	-0,74	-1,68	0,03077	-0,27	-1,21	0,48139	NR_027504	FAM133A
8168678	-0,74	-1,68	0,22453	-0,33	-1,26	0,02348	NM_001171110	LRRC10
7964846	-0,74	-1,68	0,08356	-1,40	-2,64	0,00066	NM_201550	KCNIP4
8099593	-0,74	-1,68	0,13418	-1,21	-2,31	0,00519	NM_147182	PPBP
8100971	-0,74	-1,68	0,08669	-1,28	-2,43	0,09315	NM_002704	TRPM1
7987068	-0,74	-1,68	0,00379	-0,99	-1,99	0,00129	NM_002420	---
7944952	-0,74	-1,68	0,19508	-0,41	-1,33	0,17033	---	CDSN
8178442	-0,74	-1,68	0,49039	-1,72	-3,29	0,00896	NM_001264	LBR
7910096	-0,74	-1,68	0,06060	-1,19	-2,29	0,00088	NM_002296	ALKBH6
8036242	-0,74	-1,68	0,17321	0,78	1,72	0,20326	NM_032878	EIF4E1B
8110218	-0,74	-1,68	0,17765	-0,76	-1,69	0,00062	NM_001099408	FJX1
7939365	-0,75	-1,68	0,18820	0,70	1,62	0,00318	NM_014344	---
8050654	-0,75	-1,68	0,29853	-0,74	-1,68	0,00006	---	---
8099091	-0,75	-1,68	0,30420	-0,96	-1,95	0,18551	---	PTPRZ1
8135774	-0,75	-1,68	0,00322	-0,57	-1,48	0,05979	NM_002851	CCDC102A
8001576	-0,75	-1,68	0,08109	-0,44	-1,35	0,29709	NM_033212	CD300LG
8007537	-0,75	-1,68	0,04361	-1,87	-3,65	0,00015	NM_145273	---
7950117	-0,75	-1,68	0,24410	-0,61	-1,52	0,03817	---	BIRC5
8018860	-0,75	-1,68	0,00226	-0,71	-1,63	0,01284	NM_001168	WNT9A
7924832	-0,75	-1,68	0,13942	-0,49	-1,40	0,15145	NM_003395	HYDIN
8002470	-0,75	-1,68	0,17922	-1,55	-2,93	0,00106	NM_032821	LY6G6C
8178586	-0,75	-1,68	0,16311	-1,16	-2,24	0,09593	NM_025261	XKR8
7899417	-0,75	-1,68	0,02790	1,35	2,54	0,02316	NM_018053	---
8163105	-0,75	-1,68	0,00065	-1,33	-2,51	0,01038	---	---
7958826	-0,75	-1,68	0,05763	-1,19	-2,28	0,00414	---	TM4SF18
8091402	-0,75	-1,68	0,01815	-1,54	-2,92	0,00653	NM_138786	MND1
8097857	-0,75	-1,68	0,02697	-0,07	-1,05	0,74094	NM_032117	OR5I1
7948109	-0,75	-1,68	0,29992	-2,83	-7,09	0,00255	NM_006637	---
8088474	-0,75	-1,68	0,01700	-0,56	-1,47	0,03765	---	SLC26A10
7956573	-0,75	-1,68	0,25011	-0,05	-1,03	0,78994	NM_133489	OR5V1
8178287	-0,75	-1,68	0,06282	-1,02	-2,03	0,00342	NM_030876	BAG1
8160647	-0,75	-1,68	0,34907	1,61	3,04	0,00494	NM_004323	---
8173412	-0,75	-1,68	0,14968	-0,53	-1,44	0,10900	---	CDH19
8023710	-0,75	-1,68	0,11243	-1,13	-2,20	0,01461	NM_021153	THEMIS
8129410	-0,75	-1,68	0,02163	-0,88	-1,84	0,00009	NM_001164685	NTRK2
8156134	-0,75	-1,68	0,05623	-1,12	-2,17	0,02169	NM_006180	---
7975987	-0,75	-1,68	0,06428	-0,79	-1,72	0,07814	---	OR51E2
7945991	-0,75	-1,68	0,25050	-1,90	-3,74	0,00462	NM_030774	MYOM3
7913727	-0,75	-1,68	0,03899	-0,65	-1,57	0,00210	NM_152372	SIM2
8068496	-0,75	-1,68	0,15608	1,00	2,00	0,05133	NM_009586	KRT40
8015152	-0,75	-1,68	0,07680	-1,18	-2,26	0,03051	NM_182497	KRT40
8019604	-0,75	-1,68	0,07680	-1,18	-2,26	0,03051	NM_182497	---
7969768	-0,75	-1,68	0,04883	-0,80	-1,74	0,00184	---	TFAP4
7999102	-0,75	-1,68	0,19709	0,05	1,03	0,73868	NM_003223	AP4S1
7973813	-0,75	-1,68	0,09053	-0,74	-1,67	0,13375	NM_007077	---
8082163	-0,75	-1,68	0,52142	-2,19	-4,57	0,00333	---	C20orf62
8066411	-0,75	-1,68	0,11994	-1,50	-2,83	0,00383	ENST00000372910	MMP25
7992811	-0,75	-1,68	0,19142	-0,80	-1,74	0,06194	NM_022468	ST8SIA6
7932407	-0,75	-1,68	0,09992	-1,08	-2,11	0,02929	NM_001004470	LOC440297
7985418	-0,75	-1,68	0,11464	-1,28	-2,43	0,01367	ENST00000342039	LOC440297
7985459	-0,75	-1,68	0,11464	-1,28	-2,43	0,01367	ENST00000342039	LOC440297
7990928	-0,75	-1,68	0,11464	-1,28	-2,43	0,01367	ENST00000342039	SLC26A5
8141922	-0,75	-1,68	0,19083	-1,25	-2,39	0,00880	NM_198999	FCRL4

7921259	-0,75	-1,68	0,12022	-0,78	-1,71	0,15140	NM_031282	ASB2
7981020	-0,75	-1,68	0,05406	-1,75	-3,38	0,01186	NM_016150	---
8112041	-0,75	-1,68	0,05749	-0,51	-1,42	0,00218	---	---
8121347	-0,75	-1,68	0,19304	-2,30	-4,93	0,00115	---	---
8017257	-0,75	-1,68	0,16525	-0,94	-1,93	0,00649	---	RAB17
8059955	-0,75	-1,68	0,00626	0,28	1,22	0,39562	NM_022449	CCDC135
7996117	-0,75	-1,68	0,20927	-1,72	-3,30	0,00665	NM_032269	CARD9
8165107	-0,75	-1,68	0,06434	-0,19	-1,14	0,56446	NM_052813	EVC2
8099144	-0,75	-1,68	0,01521	-0,17	-1,12	0,58214	NM_147127	GRID2
8096440	-0,75	-1,68	0,00022	-1,19	-2,28	0,00400	NM_001510	GRIN2A
7999364	-0,75	-1,68	0,00015	-0,70	-1,62	0,00624	NM_001134407	GGCT
8138857	-0,75	-1,68	0,00616	1,17	2,25	0,02986	NM_024051	UPK1B
8081826	-0,75	-1,68	0,18933	-1,13	-2,19	0,00152	NM_006952	OR13F1
8157012	-0,75	-1,68	0,24202	-1,79	-3,45	0,02942	NM_001004485	AAA1
8138958	-0,75	-1,68	0,00127	-1,29	-2,45	0,00000	AY312368	LOC100128988
8062601	-0,75	-1,68	0,06647	-1,21	-2,32	0,08391	AK023614	RAMP2
8007348	-0,75	-1,68	0,01701	-1,24	-2,36	0,05667	NM_005854	XCL2
7922127	-0,75	-1,68	0,00944	-1,13	-2,19	0,03264	NM_003175	SUN5
8065652	-0,75	-1,68	0,12767	-1,95	-3,86	0,00060	NM_080675	---
8035144	-0,75	-1,68	0,01296	-1,15	-2,21	0,13699	---	LRFN2
8119390	-0,75	-1,68	0,15395	-1,12	-2,18	0,00196	NM_020737	C7orf49
8143065	-0,75	-1,68	0,04026	0,78	1,72	0,31212	NR_024185	CHCHD7
8146517	-0,75	-1,68	0,26314	2,00	3,99	0,00219	NM_001011667	CYSLTR2
7969050	-0,75	-1,68	0,10055	-1,00	-2,01	0,01419	NM_020377	GOT1L1
8150244	-0,75	-1,68	0,30027	-0,46	-1,37	0,07747	NM_152413	LOC440570 // LOC440570
7919580	-0,75	-1,68	0,47749	-3,34	-10,12	0,00113	AK125737 // AK125737	LOC440570 // LOC440570
7919596	-0,75	-1,68	0,47749	-3,34	-10,12	0,00113	AK125737 // AK125737	PIH1D2
7951672	-0,75	-1,68	0,03093	-0,37	-1,29	0,21806	NM_138789	---
8122740	-0,75	-1,68	0,29900	-1,22	-2,33	0,20599	---	---
8131942	-0,75	-1,68	0,06060	-0,89	-1,86	0,12721	---	GIGYF1
8141595	-0,75	-1,68	0,02701	-0,34	-1,27	0,09991	NM_022574	MAGEE1
8168458	-0,75	-1,68	0,10854	-0,81	-1,76	0,02453	NM_020932	ZNF469
7997765	-0,75	-1,68	0,26225	-1,98	-3,96	0,00028	NM_001127464	---
8058568	-0,75	-1,68	0,02943	-1,22	-2,33	0,02080	---	---
8173922	-0,75	-1,68	0,12234	-0,60	-1,51	0,00127	---	FHL5
8121181	-0,75	-1,68	0,01518	-0,67	-1,59	0,02899	NM_020482	KRTAP4-9
8007115	-0,75	-1,68	0,60381	-3,29	-9,78	0,00293	NM_001146041	NANOG
7953675	-0,75	-1,68	0,12357	-1,32	-2,49	0,03867	NM_024865	---
8014113	-0,75	-1,68	0,02943	-1,06	-2,08	0,00222	---	ELAVL3
8034263	-0,75	-1,68	0,01613	-1,08	-2,11	0,00780	NM_001420	COMMD9
7947528	-0,75	-1,68	0,12559	0,08	1,06	0,72144	NM_014186	SUN3
8139606	-0,75	-1,69	0,12469	-0,61	-1,52	0,06448	NM_001030019	---
7912209	-0,75	-1,69	0,13125	-0,77	-1,71	0,33850	---	---
7965838	-0,75	-1,69	0,00043	-0,58	-1,49	0,04137	---	CCDC26
8152821	-0,75	-1,69	0,06897	-1,24	-2,35	0,02006	BC070152	LOC440993
8084917	-0,75	-1,69	0,05865	-0,10	-1,07	0,54289	AK128346	C9orf163
8159350	-0,75	-1,69	0,06162	-0,94	-1,91	0,03352	NM_152571	FLJ39653
8094251	-0,75	-1,69	0,09804	-0,23	-1,17	0,59537	NR_027696	---
8157227	-0,75	-1,69	0,06194	-1,47	-2,78	0,00548	---	FCGR3A
7921873	-0,75	-1,69	0,00208	-1,11	-2,15	0,01382	NM_000569	RAB39
7943577	-0,75	-1,69	0,05362	-0,76	-1,69	0,01939	NM_017516	PRSS48
8097794	-0,75	-1,69	0,14030	-1,77	-3,41	0,00029	NM_183375	CCNYL2
7933121	-0,75	-1,69	0,17041	-0,51	-1,42	0,03903	ENST00000426433	---
8107857	-0,75	-1,69	0,16609	-1,11	-2,16	0,01601	---	FBXW12
8079615	-0,75	-1,69	0,02731	-1,25	-2,38	0,00007	NM_207102	FBXO9
8120271	-0,75	-1,69	0,02380	-1,12	-2,17	0,04871	AK095307	OR52L1
7946115	-0,75	-1,69	0,00312	-1,24	-2,36	0,00788	NM_001005173	IGSF9B
7952813	-0,75	-1,69	0,07719	0,46	1,37	0,10974	NM_014987	GABRP
8109926	-0,75	-1,69	0,14876	-0,75	-1,69	0,04585	NM_014211	---
7930179	-0,75	-1,69	0,03150	-1,23	-2,35	0,00002	---	---
8175246	-0,75	-1,69	0,35262	-1,65	-3,14	0,00423	---	FLJ39061
8047492	-0,75	-1,69	0,03829	-0,37	-1,29	0,08547	AK297413	---
8084810	-0,75	-1,69	0,00545	-1,97	-3,93	0,00015	---	OR7E87P
7942274	-0,75	-1,69	0,62778	-2,97	-7,85	0,00597	ENST00000329336	---
8052016	-0,75	-1,69	0,01186	-1,17	-2,25	0,01589	---	ZNRF3
8072160	-0,75	-1,69	0,01299	0,26	1,19	0,28714	NM_032173	TPTE2
7970404	-0,75	-1,69	0,02163	-1,78	-3,44	0,00323	NM_130785	---
8061916	-0,75	-1,69	0,13188	-0,54	-1,46	0,00230	---	ERC2
8088192	-0,75	-1,69	0,02119	-0,69	-1,61	0,02378	NM_015576	---
7988623	-0,76	-1,69	0,12623	-0,64	-1,56	0,00889	---	HCN1
8111998	-0,76	-1,69	0,06509	-1,68	-3,21	0,00809	NM_021072	HNF1A

7959234	-0,76	-1,69	0,15057	-1,26	-2,40	0,03692	NM_000545	SCN7A
8056518	-0,76	-1,69	0,02500	-0,84	-1,78	0,00647	NM_002976	---
8133187	-0,76	-1,69	0,00004	-0,38	-1,30	0,17650	---	---
8142536	-0,76	-1,69	0,14509	-1,15	-2,22	0,00002	---	PAX4
8142747	-0,76	-1,69	0,16045	-1,66	-3,15	0,00142	NM_006193	CRIPAK
8093494	-0,76	-1,69	0,04195	0,79	1,73	0,09883	NM_175918	MIR29A
8142975	-0,76	-1,69	0,02939	-1,54	-2,90	0,00268	NR_029503	---
8151991	-0,76	-1,69	0,00360	-0,47	-1,38	0,05043	---	GNL1
8179648	-0,76	-1,69	0,00902	-0,31	-1,24	0,48875	NM_005275	OR4C46
7948044	-0,76	-1,69	0,20144	-1,58	-3,00	0,01253	NM_001004703	---
8146324	-0,76	-1,69	0,08367	-0,89	-1,86	0,04331	---	ARHGAP39
8153876	-0,76	-1,69	0,00445	-0,28	-1,21	0,36898	NM_025251	CLEC18A
7997010	-0,76	-1,69	0,27376	-0,96	-1,95	0,07237	NM_182619	SNORA19
7936637	-0,76	-1,69	0,07739	-0,95	-1,93	0,00240	NR_002917	---
7971243	-0,76	-1,69	0,07388	-0,93	-1,90	0,01555	---	PIGQ
7991904	-0,76	-1,69	0,05402	1,16	2,24	0,07616	NM_004204	SIGLEC5
8038877	-0,76	-1,69	0,40062	-0,23	-1,18	0,40128	NM_003830	---
7989220	-0,76	-1,69	0,08213	-0,99	-1,98	0,00029	---	OR10V1
7948418	-0,76	-1,69	0,17622	-0,47	-1,38	0,23493	NM_001005324	VIPR2
8144213	-0,76	-1,69	0,06797	-0,57	-1,48	0,04201	NM_003382	FPR1
8038899	-0,76	-1,69	0,02253	-0,66	-1,58	0,20942	NM_002029	ACAP1
8004331	-0,76	-1,69	0,03412	-0,75	-1,68	0,07502	NM_014716	RELT
7942439	-0,76	-1,69	0,00856	-0,04	-1,03	0,85563	NM_032871	---
8175664	-0,76	-1,69	0,00021	-1,43	-2,69	0,00224	---	CHST5
8002891	-0,76	-1,69	0,24445	-1,06	-2,09	0,00388	NM_024533	---
7922460	-0,76	-1,69	0,00350	-1,00	-2,01	0,01203	---	FCGR2A
7906757	-0,76	-1,69	0,41681	-1,11	-2,16	0,00130	NM_001136219	---
8152090	-0,76	-1,69	0,14795	-0,57	-1,48	0,03841	---	SCARNA15
7985480	-0,76	-1,69	0,08095	-1,48	-2,79	0,00023	NR_003011	IGLL3
8072004	-0,76	-1,69	0,29652	-1,25	-2,37	0,00796	NR_029395	MORN4
7935510	-0,76	-1,69	0,11054	1,63	3,10	0,00021	NM_178832	C19orf57
8034700	-0,76	-1,69	0,02935	-0,20	-1,15	0,56552	BC119719	LOC29034
8048071	-0,76	-1,69	0,18000	-0,96	-1,94	0,00436	NR_002763	B3GAT2
8127484	-0,76	-1,69	0,06335	-0,88	-1,84	0,10388	NM_080742	WSCD1
8004152	-0,76	-1,69	0,15329	0,52	1,43	0,38719	NM_015253	CNTN6
8077299	-0,76	-1,69	0,10081	-0,80	-1,74	0,00056	NM_014461	C21orf57
8069340	-0,76	-1,69	0,09173	0,21	1,16	0,56861	NM_058181	CLLU1OS
7965431	-0,76	-1,69	0,00311	-0,52	-1,43	0,04449	NM_001025232	ULBP3
8130176	-0,76	-1,69	0,13144	0,23	1,18	0,46934	NM_024518	---
8106512	-0,76	-1,69	0,02133	-0,45	-1,36	0,11461	---	---
8094772	-0,76	-1,69	0,18955	-1,60	-3,02	0,00063	---	SHANK2
7950042	-0,76	-1,69	0,00348	0,98	1,97	0,03094	NM_012309	FHAD1
7898124	-0,76	-1,69	0,04928	-0,77	-1,70	0,00422	NM_052929	ACOT2
7975602	-0,76	-1,69	0,20760	0,33	1,26	0,65407	NM_006821	SMAD7
8023220	-0,76	-1,69	0,03686	-0,88	-1,84	0,01010	NM_005904	ISM1
8061013	-0,76	-1,69	0,09696	-0,55	-1,46	0,17925	NM_080826	GALP
8031622	-0,76	-1,69	0,14585	-0,72	-1,64	0,08557	NM_033106	---
7971013	-0,76	-1,69	0,43972	-1,48	-2,78	0,11979	---	CCDC65
7955156	-0,76	-1,69	0,10152	-0,91	-1,88	0,01490	NM_033124	SNORA16B
7909601	-0,76	-1,69	0,01147	-1,13	-2,19	0,01383	NR_004389	ANKS1B
7965686	-0,76	-1,69	0,00961	0,12	1,09	0,51334	NM_152788	NPTX2
8134463	-0,76	-1,69	0,21857	-0,91	-1,88	0,11756	NM_002523	PXT1
8126007	-0,76	-1,69	0,11194	-1,24	-2,36	0,07448	NM_152990	PRND
8060765	-0,76	-1,69	0,15672	-1,05	-2,07	0,10258	NM_012409	ATG4B
8077211	-0,76	-1,69	0,13170	1,61	3,06	0,02812	NM_013325	ZNF511
7931561	-0,76	-1,69	0,10635	0,94	1,92	0,03356	NM_145806	CST4
8065410	-0,76	-1,69	0,38561	-0,02	-1,02	0,97051	NM_001899	OR52J3
7937986	-0,76	-1,69	0,19586	-1,88	-3,67	0,03647	NM_001001916	C15orf59
7990269	-0,76	-1,69	0,15954	-0,44	-1,36	0,29901	BC111368	---
7910587	-0,76	-1,69	0,12014	-1,00	-2,00	0,08330	---	---
8146647	-0,76	-1,69	0,15744	-2,03	-4,07	0,02250	---	TDRD6
8120082	-0,76	-1,69	0,19373	-1,41	-2,65	0,00117	NM_001010870	CKMT2
8106689	-0,76	-1,69	0,29756	-1,50	-2,82	0,03440	NM_001825	---
7930559	-0,76	-1,69	0,03061	-0,63	-1,55	0,03408	---	ZNF204P
8124498	-0,76	-1,69	0,24191	2,33	5,02	0,00682	NR_002722	LOC150622
8040073	-0,76	-1,69	0,08530	-0,72	-1,65	0,13322	NR_026832	CCDC40
8010328	-0,76	-1,69	0,07017	-0,77	-1,70	0,09708	NM_017950	---
8157520	-0,76	-1,69	0,09298	-1,06	-2,08	0,00273	---	LETM1
8098924	-0,76	-1,69	0,05846	1,32	2,50	0,07078	NM_012318	EML5
7980636	-0,76	-1,69	0,00684	0,90	1,86	0,00480	NM_183387	---
7964625	-0,76	-1,69	0,05017	-0,94	-1,91	0,01382	---	KRT39
8015144	-0,76	-1,69	0,05267	-1,11	-2,16	0,00900	NM_213656	KRT39
8019614	-0,76	-1,69	0,05267	-1,11	-2,16	0,00900	NM_213656	PIN1L

7902267	-0,76	-1,69	0,08017	-0,85	-1,81	0,13024	NR_023916	---
7907351	-0,76	-1,69	0,03229	0,14	1,11	0,55205	---	KCNA2
7918449	-0,76	-1,69	0,14732	-1,50	-2,82	0,02549	NM_004974	PRAMEF12
7897974	-0,76	-1,69	0,31581	-1,12	-2,18	0,00354	NM_001080830	---
8089295	-0,76	-1,69	0,10983	-1,24	-2,36	0,07336	---	MAGEB1
8166619	-0,76	-1,69	0,23451	-0,79	-1,72	0,06874	NM_002363	ZNF783
8137118	-0,76	-1,69	0,04193	-0,51	-1,42	0,17138	ENST00000378052	CATSPER2
7988163	-0,76	-1,69	0,21765	-0,59	-1,51	0,10897	NM_054020	MIR130A
7940044	-0,76	-1,69	0,06955	-0,95	-1,93	0,00008	NR_029673	---
7963263	-0,76	-1,69	0,10012	-0,86	-1,82	0,00060	---	UBXN10
7898623	-0,76	-1,69	0,19304	-0,66	-1,58	0,00203	NM_152376	FILIP1
8127646	-0,76	-1,69	0,02310	-1,03	-2,05	0,00081	NM_015687	ZNF576
8029311	-0,76	-1,69	0,10933	-0,49	-1,40	0,00586	NM_024327	---
8045525	-0,76	-1,69	0,22266	-1,01	-2,02	0,00004	---	---
8074615	-0,76	-1,69	0,14039	-1,15	-2,22	0,00307	---	OPALIN
7935288	-0,76	-1,69	0,03520	-0,76	-1,70	0,00836	NM_033207	ITIH2
7926061	-0,76	-1,69	0,02020	-0,89	-1,86	0,00135	NM_002216	FCGR1A
7905047	-0,76	-1,69	0,04714	-1,37	-2,59	0,00314	NM_000566	---
8106919	-0,76	-1,69	0,01588	-0,66	-1,58	0,09023	---	CT45A3
8170068	-0,76	-1,69	0,21800	-1,75	-3,36	0,01305	NM_001017435	MAZ
7994637	-0,76	-1,70	0,04150	0,86	1,82	0,03798	NM_001042539	MGC24103
8160274	-0,76	-1,70	0,07051	-1,00	-1,99	0,11355	AK021795	GPR34
8166906	-0,76	-1,70	0,09604	-0,80	-1,75	0,01345	NM_001097579	CREB3L3
8024754	-0,76	-1,70	0,30886	-1,20	-2,30	0,00766	NM_032607	ZNF777
8143714	-0,76	-1,70	0,00269	0,58	1,49	0,04985	NM_015694	SLC26A8
8125959	-0,76	-1,70	0,14938	-1,01	-2,01	0,04660	NM_052961	HIST1H3H
8117589	-0,76	-1,70	0,11628	1,25	2,37	0,10414	NM_003536	SNORA14A
8133688	-0,76	-1,70	0,13954	-0,14	-1,10	0,53812	NR_002955	GKN2
8052753	-0,76	-1,70	0,14727	-0,61	-1,53	0,05674	NM_182536	---
8000700	-0,76	-1,70	0,04704	-0,98	-1,98	0,00381	---	---
8003599	-0,76	-1,70	0,04704	-0,98	-1,98	0,00381	---	USHBP1
8035254	-0,76	-1,70	0,01151	-1,26	-2,39	0,01176	NM_031941	LOC401052
8085272	-0,76	-1,70	0,22093	-1,15	-2,22	0,00872	AK022260	LMX1A
7921936	-0,76	-1,70	0,21542	-1,66	-3,16	0,02172	NM_177398	OR8B2
7952398	-0,76	-1,70	0,01208	-1,21	-2,31	0,00050	NM_001005468	GNGT2
8016532	-0,76	-1,70	0,00678	-0,72	-1,65	0,03008	NM_031498	---
8170300	-0,76	-1,70	0,05693	-0,71	-1,63	0,03074	---	CLCN2
8092473	-0,76	-1,70	0,19096	-0,27	-1,21	0,65742	NM_004366	VIL1
8048319	-0,76	-1,70	0,03123	-0,50	-1,42	0,00351	NM_007127	---
7926594	-0,76	-1,70	0,20911	-0,74	-1,67	0,02132	---	CXCL11
8101131	-0,76	-1,70	0,08909	-0,49	-1,40	0,24211	NM_005409	---
8151099	-0,76	-1,70	0,24886	-0,76	-1,69	0,01733	---	C1orf88
7903959	-0,76	-1,70	0,04875	-0,59	-1,51	0,07296	NM_181643	---
8041900	-0,76	-1,70	0,07554	0,92	1,90	0,08510	---	LOC338797
7959921	-0,76	-1,70	0,29006	-1,03	-2,04	0,04342	AK098523	LHX2
8157751	-0,76	-1,70	0,24123	0,86	1,82	0,09531	NM_004789	BRD7P3
8121725	-0,76	-1,70	0,02200	-1,03	-2,05	0,04541	NR_002730	CORO7
7999120	-0,76	-1,70	0,07789	0,88	1,85	0,07140	NM_024535	OR1N1
8163958	-0,76	-1,70	0,17790	-0,87	-1,83	0,00338	NM_012363	---
8131335	-0,76	-1,70	0,00499	-0,84	-1,79	0,02509	---	HLA-DOA
8125545	-0,76	-1,70	0,05576	-2,37	-5,18	0,00560	NM_002119	PRIMA1
7981013	-0,76	-1,70	0,32848	-0,83	-1,78	0,01774	NM_178013	CD14
8114612	-0,76	-1,70	0,08140	-0,79	-1,72	0,01139	NM_000591	FAM75A7
8156253	-0,76	-1,70	0,23822	-1,23	-2,35	0,30559	NM_015667	ARL4C
8059854	-0,76	-1,70	0,02421	-1,38	-2,61	0,01429	NM_005737	LRRC45
8010719	-0,76	-1,70	0,00951	-0,08	-1,05	0,75379	NM_144999	---
7942645	-0,76	-1,70	0,26614	-1,03	-2,04	0,00970	---	---
7961173	-0,76	-1,70	0,44314	-1,73	-3,32	0,19432	---	---
7951163	-0,76	-1,70	0,07163	-0,96	-1,95	0,01792	---	---
7985997	-0,76	-1,70	0,01577	1,20	2,30	0,03364	---	---
7925874	-0,76	-1,70	0,04916	-1,48	-2,78	0,00080	---	MYCBPAP
8008350	-0,76	-1,70	0,11159	-0,59	-1,50	0,13070	NM_032133	KRTAP5-11
7950078	-0,76	-1,70	0,05240	-2,11	-4,33	0,00285	NM_001005405	RFTN1
8177072	-0,76	-1,70	0,08006	-1,62	-3,07	0,00398	BC041884	SPO11
8063551	-0,76	-1,70	0,00386	-0,84	-1,79	0,00001	NM_012444	EMR4P
8033332	-0,76	-1,70	0,00978	-0,99	-1,98	0,01710	NR_024075	KRT33B
8015262	-0,76	-1,70	0,23030	-1,64	-3,11	0,01329	NM_002279	NEB
8055711	-0,76	-1,70	0,02395	-0,91	-1,88	0,00007	NM_001164507	---
7926484	-0,76	-1,70	0,02614	-0,63	-1,55	0,07940	---	STOML3
7971126	-0,76	-1,70	0,17631	-1,15	-2,22	0,01804	NM_145286	---
8147445	-0,76	-1,70	0,01631	-1,07	-2,10	0,01590	---	---
8079058	-0,76	-1,70	0,05182	-0,98	-1,97	0,00068	---	PMCH
7965867	-0,76	-1,70	0,08294	-0,72	-1,65	0,01770	NM_002674	PRAMEF13

7912595	-0,76	-1,70	0,13951	-1,30	-2,45	0,01906	NM_001024661	PRAMEF13
7912610	-0,76	-1,70	0,13951	-1,30	-2,45	0,01906	NM_001024661	---
8054039	-0,76	-1,70	0,13113	-0,86	-1,81	0,00675	---	---
7913556	-0,76	-1,70	0,00032	-0,83	-1,78	0,00393	---	---
7943777	-0,76	-1,70	0,04052	-0,77	-1,70	0,00591	---	OR2H1
8117732	-0,76	-1,70	0,00786	-1,03	-2,04	0,00001	NM_030883	CAPZA3
7954240	-0,76	-1,70	0,18158	-0,81	-1,75	0,07929	NM_033328	---
8175436	-0,76	-1,70	0,03338	-0,52	-1,44	0,06631	---	CPO
8047829	-0,76	-1,70	0,25635	-1,26	-2,39	0,00072	NM_173077	C2orf54
8060187	-0,77	-1,70	0,05206	-1,10	-2,14	0,02365	NM_001085437	SNORD21
7903022	-0,77	-1,70	0,04385	-0,59	-1,51	0,01059	NR_000006	---
8138287	-0,77	-1,70	0,10852	-1,08	-2,12	0,00778	---	AATK
8019090	-0,77	-1,70	0,09823	-1,42	-2,68	0,01840	NM_001080395	RNPS1
7998774	-0,77	-1,70	0,08811	0,02	1,01	0,97137	NM_080594	---
8089727	-0,77	-1,70	0,05614	-0,51	-1,43	0,01364	---	C15orf51
7986503	-0,77	-1,70	0,39895	-1,84	-3,58	0,00634	NR_003260	CDH26
8063761	-0,77	-1,70	0,00271	-0,85	-1,81	0,00003	NM_177980	TAAR1
8129615	-0,77	-1,70	0,10107	-1,25	-2,37	0,03291	NM_138327	SETD1A
7994985	-0,77	-1,70	0,01707	0,33	1,26	0,22253	NM_014712	---
8167638	-0,77	-1,70	0,13686	-0,94	-1,92	0,00122	---	BCL3
8029465	-0,77	-1,70	0,01183	1,43	2,69	0,06725	NM_005178	FRMPD2
7933279	-0,77	-1,70	0,07261	-1,03	-2,04	0,01129	NM_001018071	FRMPD2
7933394	-0,77	-1,70	0,07261	-1,03	-2,04	0,01129	NM_001018071	FREM3
8102986	-0,77	-1,70	0,04629	-0,70	-1,63	0,00034	NM_001168235	TREM2
8126279	-0,77	-1,70	0,12300	-0,71	-1,63	0,05744	NM_018965	UGT2B15
8100737	-0,77	-1,70	0,05983	-1,03	-2,04	0,00072	NM_001076	UGT2B15
8100746	-0,77	-1,70	0,05983	-1,03	-2,04	0,00072	NM_001076	AQP7P1
8155554	-0,77	-1,70	0,22483	-1,98	-3,94	0,00373	NR_002817	SPATS1
8120011	-0,77	-1,70	0,00628	-0,95	-1,93	0,00173	NM_145026	TMEM65
8152750	-0,77	-1,70	0,14695	0,95	1,94	0,01287	NM_194291	---
7935863	-0,77	-1,70	0,11341	-1,96	-3,88	0,00338	---	PDZD3
7944478	-0,77	-1,70	0,17899	-1,14	-2,20	0,00078	NR_033122	CCHCR1
8178448	-0,77	-1,70	0,04432	-0,39	-1,31	0,12635	NM_001105564	---
8080989	-0,77	-1,70	0,13260	-1,26	-2,39	0,00059	---	HDAC6
8167369	-0,77	-1,70	0,01960	0,78	1,71	0,01484	NM_006044	FAM136B // FAM136B
8116620	-0,77	-1,70	0,24094	-0,78	-1,72	0,04062	NM_001012983 // NM_001012983	OK/SW-CL.36
8101501	-0,77	-1,70	0,03022	-1,24	-2,36	0,01126	AB064670	RIMS3
7915333	-0,77	-1,70	0,03428	0,10	1,07	0,73765	NM_014747	NCRNA00185
8177214	-0,77	-1,70	0,03303	-0,23	-1,17	0,08042	NR_001544	C1orf49
7907633	-0,77	-1,70	0,22996	-1,83	-3,57	0,00790	NM_032126	ACCSL
7939494	-0,77	-1,70	0,22134	-1,31	-2,48	0,03688	NM_001031854	MIR27A
8034696	-0,77	-1,70	0,31266	-1,10	-2,14	0,00039	NR_029501	CT45A5
8175344	-0,77	-1,70	0,20322	-1,50	-2,83	0,00357	NM_001007551	---
7967452	-0,77	-1,70	0,15720	-1,54	-2,91	0,02659	---	LRP5L
8075052	-0,77	-1,70	0,07856	-0,65	-1,57	0,20267	NM_182492	SCG3
7983718	-0,77	-1,70	0,11971	-1,01	-2,02	0,00155	NM_013243	PRODH2
8036155	-0,77	-1,70	0,04062	-1,61	-3,06	0,00321	NM_021232	ANKRD34B
8112896	-0,77	-1,70	0,01388	0,10	1,08	0,38211	NM_001004441	DMBT1
7931108	-0,77	-1,70	0,11816	-1,46	-2,75	0,00076	NM_007329	PHPT1
8159441	-0,77	-1,70	0,18255	0,14	1,10	0,83509	NM_001135861	CLEC18B
7997032	-0,77	-1,70	0,18476	-1,25	-2,37	0,05895	NM_001011880	CHRNB4
7990729	-0,77	-1,70	0,04081	-0,13	-1,09	0,81064	NM_000750	HLA-DRA
8179481	-0,77	-1,70	0,01415	1,18	2,26	0,06059	NM_019111	SPINK5
8109001	-0,77	-1,70	0,01138	-0,05	-1,03	0,89836	NM_001127698	CARD14
8010379	-0,77	-1,70	0,01000	-1,33	-2,52	0,00419	NM_024110	ASB17
7917082	-0,77	-1,70	0,00521	-0,89	-1,85	0,01479	NM_080868	ZNRF4
8024934	-0,77	-1,70	0,30823	-1,18	-2,27	0,00242	NM_181710	---
7990672	-0,77	-1,70	0,15976	-1,15	-2,22	0,00595	---	IMPA2
8020183	-0,77	-1,70	0,03667	0,75	1,69	0,28965	NM_014214	IL1R2
8043981	-0,77	-1,70	0,18207	-0,94	-1,92	0,00274	NM_004633	FLJ44006
8054607	-0,77	-1,70	0,13762	-0,82	-1,77	0,07586	AK125994	---
8141239	-0,77	-1,70	0,12476	-1,31	-2,47	0,01455	---	SHMT1
8013243	-0,77	-1,70	0,10714	0,59	1,50	0,18437	NM_004169	TIGD7
7998952	-0,77	-1,70	0,05567	-0,12	-1,08	0,51864	NM_033208	---
8042308	-0,77	-1,70	0,17631	-1,39	-2,62	0,01619	---	---
8171890	-0,77	-1,70	0,09838	-1,09	-2,13	0,00469	---	---
7951108	-0,77	-1,70	0,03696	0,25	1,19	0,56912	---	PTCHD3
7932727	-0,77	-1,70	0,02061	-1,07	-2,11	0,02720	NM_001034842	AGR3
8138392	-0,77	-1,70	0,19227	-0,81	-1,75	0,03772	NM_176813	C12orf27
7967107	-0,77	-1,70	0,13595	-1,66	-3,16	0,01840	NR_024345	TTPA
8151042	-0,77	-1,70	0,00352	-0,63	-1,54	0,00031	NM_000370	KLHDC9

7906642	-0,77	-1,70	0,00097	-0,66	-1,58	0,09279	NM_152366	ATAD3C
7896937	-0,77	-1,70	0,08710	0,00	1,00	0,99953	NM_001039211	---
8139941	-0,77	-1,70	0,00366	-1,10	-2,15	0,03028	---	---
8137042	-0,77	-1,70	0,04082	-1,20	-2,30	0,00108	---	SAG
8049301	-0,77	-1,70	0,11341	-0,70	-1,62	0,00821	NM_000541	CCDC86
7940349	-0,77	-1,70	0,02453	-0,43	-1,34	0,22672	NM_024098	---
8138956	-0,77	-1,70	0,30052	-1,13	-2,19	0,01909	---	ZNF77
8032544	-0,77	-1,70	0,11759	0,56	1,47	0,34282	NM_021217	ENTPD3
8078971	-0,77	-1,70	0,03843	0,13	1,09	0,54285	NM_001248	---
7986325	-0,77	-1,70	0,02077	-1,41	-2,65	0,02058	---	WDR65
7900639	-0,77	-1,71	0,01712	-0,98	-1,97	0,01692	NR_030778	DBH
8159096	-0,77	-1,71	0,04286	-1,58	-2,99	0,00018	NM_000787	SLC12A9
8134965	-0,77	-1,71	0,02237	0,29	1,22	0,55856	NM_020246	---
7932528	-0,77	-1,71	0,18139	-0,61	-1,53	0,23606	---	ASB10
8143887	-0,77	-1,71	0,01662	-1,56	-2,96	0,01247	NM_001142459	GRIN2C
8018220	-0,77	-1,71	0,09975	-1,23	-2,34	0,02071	NM_000835	---
8097743	-0,77	-1,71	0,34181	-0,12	-1,09	0,59765	---	KIF18B
8016139	-0,77	-1,71	0,02334	2,08	4,21	0,00080	NM_001080443	---
8136457	-0,77	-1,71	0,02851	-1,21	-2,32	0,04622	---	ENPEP
8096875	-0,77	-1,71	0,02760	1,12	2,18	0,05861	NM_001977	ZNF114
8030002	-0,77	-1,71	0,10138	1,29	2,45	0,00158	NM_153608	CTSG
7978351	-0,77	-1,71	0,24243	-1,25	-2,38	0,00619	NM_001911	C12orf60
7954132	-0,77	-1,71	0,00106	-0,55	-1,47	0,10718	NM_175874	PIP5K1P1
8116827	-0,77	-1,71	0,03664	-0,90	-1,87	0,17211	NR_027712	BTNL8
8116537	-0,77	-1,71	0,05471	-1,59	-3,02	0,00077	NM_024850	MGMT
7931379	-0,77	-1,71	0,04307	0,24	1,18	0,04633	NM_002412	---
8068731	-0,77	-1,71	0,16482	-1,11	-2,16	0,08792	---	MLKL
8002778	-0,77	-1,71	0,00160	-0,16	-1,12	0,61716	NM_152649	ODZ1
8174937	-0,77	-1,71	0,01669	-0,74	-1,66	0,02751	NM_001163278	C6orf222
8125980	-0,77	-1,71	0,03064	-0,90	-1,87	0,00744	NM_001010903	FLJ42418
8050095	-0,77	-1,71	0,00653	-1,51	-2,85	0,00030	AK124409	---
8080962	-0,77	-1,71	0,26007	-0,66	-1,58	0,02585	---	FLJ14100
7911699	-0,77	-1,71	0,35795	-0,66	-1,58	0,11625	AK024162	---
8130401	-0,77	-1,71	0,04529	-0,77	-1,70	0,00052	---	GRM2
8080128	-0,77	-1,71	0,07095	-1,63	-3,10	0,00128	NM_000839	C9orf167
8159667	-0,77	-1,71	0,07024	0,62	1,54	0,25153	NM_017723	ARMCX4
8168855	-0,77	-1,71	0,05745	-0,58	-1,50	0,07639	NR_028407	CCS
7941685	-0,77	-1,71	0,10165	0,91	1,88	0,05382	NM_005125	---
8035234	-0,77	-1,71	0,04883	-0,99	-1,99	0,03294	---	---
7970702	-0,77	-1,71	0,00628	-1,01	-2,02	0,02470	---	---
7921623	-0,77	-1,71	0,21161	-1,01	-2,01	0,16817	---	TBC1D30
7956826	-0,77	-1,71	0,09846	2,26	4,79	0,00096	AB449914	ATOH8
8043244	-0,77	-1,71	0,20501	-1,29	-2,45	0,11876	NM_032827	CGNL1
7983867	-0,77	-1,71	0,01647	-0,75	-1,68	0,00643	NM_032866	BTF3L1
7969479	-0,77	-1,71	0,03516	-0,68	-1,60	0,12147	NR_026983	GPR84
7963770	-0,77	-1,71	0,06721	-0,46	-1,38	0,03106	NM_020370	CD84
7921637	-0,77	-1,71	0,04175	-1,01	-2,02	0,01239	NM_003874	---
7995629	-0,77	-1,71	0,01902	-1,17	-2,25	0,00219	---	POU2F2
8037123	-0,77	-1,71	0,21107	-1,87	-3,65	0,01016	NM_002698	IFNE
8160435	-0,77	-1,71	0,00456	-0,58	-1,50	0,02562	NM_176891	RGSL1
7907977	-0,77	-1,71	0,01872	-1,13	-2,18	0,00869	NM_001137669	---
7926834	-0,77	-1,71	0,14532	-0,93	-1,91	0,02373	---	SUV39H1
8167347	-0,77	-1,71	0,02141	1,00	1,99	0,04263	NM_003173	MYT1L
8050031	-0,77	-1,71	0,05076	-1,22	-2,33	0,01493	NM_015025	C9orf130
8162562	-0,77	-1,71	0,06658	-0,79	-1,73	0,06892	NR_023389	FLJ46120
8091595	-0,77	-1,71	0,12382	-1,13	-2,19	0,00800	AK128002	ROPN1B
8082261	-0,77	-1,71	0,04089	-0,57	-1,48	0,04678	NM_001012337	---
7924967	-0,77	-1,71	0,03187	-1,14	-2,21	0,00139	---	C4orf31
8102587	-0,77	-1,71	0,13400	-0,62	-1,53	0,00897	NM_024574	OSTN
8084814	-0,77	-1,71	0,07293	-0,85	-1,80	0,03266	NM_198184	SLC38A10
8019149	-0,77	-1,71	0,15633	-0,43	-1,34	0,34190	NM_001037984	ACTL7A
8157141	-0,77	-1,71	0,10749	-0,64	-1,56	0,03262	NM_006687	PPP1R16A
8153835	-0,77	-1,71	0,20025	-0,14	-1,11	0,76209	NM_032902	TRPV6
8143499	-0,77	-1,71	0,05245	-0,48	-1,39	0,04997	NM_018646	DHRS4
7973448	-0,77	-1,71	0,00963	0,63	1,55	0,25290	NM_021004	TEKT3
8012908	-0,77	-1,71	0,00081	-0,56	-1,47	0,03242	NM_031898	---
8090418	-0,77	-1,71	0,13668	-1,11	-2,16	0,00225	---	TMPRSS11D
8100664	-0,77	-1,71	0,05426	-0,81	-1,76	0,02551	NM_004262	NAT8
8053025	-0,77	-1,71	0,14107	-1,86	-3,63	0,00268	NM_003960	---
8132915	-0,77	-1,71	0,05407	-0,43	-1,35	0,01251	---	ARHGAP27
8016215	-0,77	-1,71	0,04480	0,52	1,44	0,34609	NM_199282	MYOM2
8144322	-0,77	-1,71	0,04873	-0,93	-1,91	0,00462	NM_003970	ZMYND17
7934384	-0,77	-1,71	0,05146	-0,67	-1,60	0,13725	NM_001024593	FGF16

8168463	-0,77	-1,71	0,07369	-0,95	-1,93	0,00015	NM_003868	SLC13A4
8143110	-0,77	-1,71	0,02324	-1,18	-2,27	0,00155	NM_012450	ZNF474
8107591	-0,77	-1,71	0,00880	-1,14	-2,21	0,00084	NM_207317	SLC22A8
7948937	-0,77	-1,71	0,13687	-0,65	-1,57	0,02323	NM_004254	KLKP1
8038653	-0,77	-1,71	0,03754	-1,14	-2,21	0,02418	NR_002948	ZNF646
7995033	-0,77	-1,71	0,14035	0,17	1,13	0,67037	NM_014699	TMCC2
7909088	-0,78	-1,71	0,01219	-0,77	-1,70	0,00780	NM_014858	ANKRD18B
8154823	-0,78	-1,71	0,28436	2,52	5,75	0,00039	ENST00000290943	FLJ41649
8126240	-0,78	-1,71	0,02687	-0,51	-1,42	0,09364	AK123643	C10orf126
7926882	-0,78	-1,71	0,14040	-1,69	-3,22	0,00103	ENST00000375520	ZCWPW2
8078319	-0,78	-1,71	0,08141	-1,16	-2,23	0,01903	NM_001040432	OR2M2
7911265	-0,78	-1,71	0,01028	-1,20	-2,29	0,00032	NM_001004688	TDRD5
7907749	-0,78	-1,71	0,05226	-0,37	-1,29	0,14596	NM_173533	FBLIM1
7898263	-0,78	-1,71	0,01831	-0,07	-1,05	0,80912	NM_017556	---
8137472	-0,78	-1,71	0,15846	-0,73	-1,66	0,17166	---	---
8124156	-0,78	-1,71	0,02157	-1,08	-2,12	0,02133	---	GPR171
8091503	-0,78	-1,71	0,09313	-1,29	-2,45	0,00072	NM_013308	SAGE1
8170076	-0,78	-1,71	0,16330	-0,71	-1,64	0,01293	NM_018666	---
7969638	-0,78	-1,71	0,14564	-1,99	-3,97	0,00005	---	P2RY10
8168524	-0,78	-1,71	0,07638	-0,54	-1,45	0,14770	NM_014499	BMPR1B
8096511	-0,78	-1,71	0,00447	1,77	3,41	0,00455	NM_001203	NCRNA00094
8159109	-0,78	-1,71	0,17077	-0,31	-1,24	0,62813	NR_015427	---
8150212	-0,78	-1,71	0,04333	-1,29	-2,44	0,00277	---	SNORD115-30
7982066	-0,78	-1,71	0,18562	-2,05	-4,14	0,00110	NR_003345	CXorf65
8173437	-0,78	-1,71	0,07407	-0,91	-1,88	0,01056	NM_001025265	CTAGE3
7971727	-0,78	-1,71	0,19074	-0,29	-1,22	0,03517	AF338231	SEMA6C
7919898	-0,78	-1,71	0,10320	-1,46	-2,76	0,00071	NM_001178061	TUBGCP6
8076909	-0,78	-1,71	0,00640	-0,72	-1,64	0,01558	NM_020461	BHMT2
8106494	-0,78	-1,71	0,28556	-1,45	-2,73	0,08929	NM_017614	SPANXN1
8170302	-0,78	-1,71	0,17451	-0,84	-1,79	0,05389	NM_001009614	MGC13053
7952065	-0,78	-1,71	0,32445	-1,33	-2,52	0,00055	ENST00000313693	BMX
8166157	-0,78	-1,71	0,00189	-1,13	-2,19	0,00566	NM_203281	---
7978626	-0,78	-1,71	0,14977	-0,59	-1,50	0,23442	---	HAL
7965606	-0,78	-1,71	0,00549	-0,63	-1,55	0,00141	NM_002108	GRK7
8083129	-0,78	-1,71	0,00748	-1,14	-2,21	0,00576	NM_139209	ANKRD30A
7927033	-0,78	-1,71	0,01067	-1,13	-2,20	0,04292	NM_052997	KCNN3
7920552	-0,78	-1,71	0,01697	-1,04	-2,06	0,11206	NM_002249	RRP12
7935425	-0,78	-1,71	0,01687	1,73	3,32	0,00131	NM_015179	ECE2
8084397	-0,78	-1,71	0,00419	-0,33	-1,26	0,10088	NM_014693	MMP24
8062123	-0,78	-1,71	0,00325	0,37	1,29	0,28110	NM_006690	COX11
8117696	-0,78	-1,71	0,30254	1,58	2,99	0,06565	NM_004375	POTEF
8055153	-0,78	-1,71	0,12907	-1,46	-2,75	0,00071	NM_001099771	---
8104652	-0,78	-1,71	0,03729	-0,76	-1,70	0,02620	---	CASP12
7951363	-0,78	-1,71	0,03751	-1,52	-2,87	0,00337	NR_000035	AQP7P1
8161488	-0,78	-1,71	0,19774	-2,02	-4,07	0,00488	NR_002817	PUSL1
7896908	-0,78	-1,71	0,06920	0,45	1,36	0,38943	NM_153339	---
8037535	-0,78	-1,71	0,29935	0,12	1,08	0,79679	---	ELAVL2
8160459	-0,78	-1,71	0,27566	-0,99	-1,99	0,02190	NM_004432	---
8113703	-0,78	-1,71	0,02821	-1,77	-3,41	0,00027	---	DOK7
8093807	-0,78	-1,71	0,13871	-1,08	-2,11	0,05655	NM_173660	ITGB1BP3
8024728	-0,78	-1,71	0,08211	-1,13	-2,19	0,02986	NM_170678	SHARPIN
8153652	-0,78	-1,71	0,05922	0,10	1,07	0,84552	NM_030974	---
7982550	-0,78	-1,71	0,02308	-0,57	-1,49	0,00701	---	SLC16A9
7933750	-0,78	-1,71	0,02258	-0,75	-1,68	0,01037	NM_194298	ARSI
8115144	-0,78	-1,72	0,13271	-0,72	-1,65	0,13432	NM_001012301	DCAF4L1
8094843	-0,78	-1,72	0,12014	-1,02	-2,03	0,00106	NM_001029955	MIR9-1
7921012	-0,78	-1,72	0,05298	-0,48	-1,40	0,00041	NR_029691	C21orf2
8069083	-0,78	-1,72	0,08807	0,27	1,21	0,55684	AB209578	---
7937971	-0,78	-1,72	0,17248	-0,99	-1,98	0,06699	---	PTH
7946687	-0,78	-1,72	0,06741	-0,85	-1,80	0,00267	NM_000315	LCK
7899753	-0,78	-1,72	0,04507	-1,17	-2,26	0,00285	NM_005356	EFNB3
8004571	-0,78	-1,72	0,11983	-0,10	-1,07	0,78778	NM_001406	SEMA3F
8079896	-0,78	-1,72	0,03028	-0,22	-1,17	0,53261	NM_004186	BEND2
8171545	-0,78	-1,72	0,01558	-1,76	-3,38	0,00305	NM_153346	TBC1D3B
8019655	-0,78	-1,72	0,34841	1,29	2,45	0,07507	NM_001001417	VCX2
8171240	-0,78	-1,72	0,23673	-1,03	-2,04	0,01085	NM_016378	TTTY13
8177282	-0,78	-1,72	0,14497	-1,75	-3,37	0,00275	NR_001537	LOC440925
8056730	-0,78	-1,72	0,03151	-0,81	-1,75	0,00097	NR_027433	---
8044572	-0,78	-1,72	0,25104	-1,11	-2,16	0,01457	---	---
8124634	-0,78	-1,72	0,02334	-1,12	-2,17	0,00364	---	OR2T27
7925759	-0,78	-1,72	0,10506	-1,80	-3,48	0,00581	NM_001001824	C12orf70
7954551	-0,78	-1,72	0,01846	-0,64	-1,56	0,01065	NM_001145010	---
8019347	-0,78	-1,72	0,25016	-0,99	-1,98	0,01714	---	OR4A5

7948099	-0,78	-1,72	0,03052	-1,04	-2,05	0,03329	NM_001005272	CDY1
8177186	-0,78	-1,72	0,05305	-1,20	-2,30	0,00898	NM_170723	---
8115039	-0,78	-1,72	0,14129	-1,35	-2,54	0,00093	---	MPO
8016932	-0,78	-1,72	0,14440	-0,48	-1,39	0,02112	NM_000250	---
8020321	-0,78	-1,72	0,05093	-1,45	-2,74	0,01315	---	PLCD4
8048350	-0,78	-1,72	0,12870	-0,56	-1,48	0,03898	NM_032726	ETV3L
7921222	-0,78	-1,72	0,05964	-1,35	-2,55	0,00655	NM_001004341	FPGS
8158123	-0,78	-1,72	0,04252	1,15	2,21	0,09992	NM_004957	---
8073924	-0,78	-1,72	0,04274	-1,51	-2,85	0,00143	---	HLA-DRB4
8178811	-0,78	-1,72	0,17985	0,99	1,99	0,00429	AK293020	KCNA3
7918457	-0,78	-1,72	0,03655	-1,42	-2,68	0,00232	NM_002232	---
7915561	-0,78	-1,72	0,11042	-1,22	-2,33	0,01011	---	FKSG73
8001070	-0,78	-1,72	0,07254	-1,45	-2,74	0,05193	NR_027714	---
8165888	-0,78	-1,72	0,09695	-0,91	-1,88	0,14527	---	LOC100131112
8149084	-0,78	-1,72	0,10470	-0,59	-1,51	0,00061	ENST00000438484	---
7933008	-0,78	-1,72	0,09287	-0,87	-1,83	0,02916	---	IL27RA
8026272	-0,78	-1,72	0,07055	1,35	2,56	0,08104	NM_004843	LOC388692
7898353	-0,78	-1,72	0,53661	-1,74	-3,33	0,00303	NR_027002	RLN2
8159977	-0,78	-1,72	0,11031	-0,38	-1,30	0,23773	NM_134441	---
7906751	-0,78	-1,72	0,00129	-0,85	-1,81	0,00365	---	VSTM1
8039109	-0,78	-1,72	0,12513	-1,68	-3,21	0,00344	NM_198481	ALDH1L1
8090314	-0,78	-1,72	0,01074	-0,89	-1,85	0,02687	NM_012190	APOA5
7951853	-0,78	-1,72	0,03438	-1,14	-2,21	0,05704	NM_052968	PKD1L1
8139534	-0,78	-1,72	0,07278	-0,95	-1,93	0,00405	NM_138295	---
8105144	-0,78	-1,72	0,24682	-0,29	-1,22	0,35438	---	---
8098326	-0,78	-1,72	0,20364	-0,85	-1,80	0,04074	---	HEMGN
8162719	-0,78	-1,72	0,13409	-0,68	-1,60	0,05197	NM_018437	---
7978403	-0,78	-1,72	0,09231	-1,37	-2,58	0,00196	---	FAM75A7
8155383	-0,78	-1,72	0,12666	-1,53	-2,89	0,00646	NM_015667	IQCA1
8059878	-0,78	-1,72	0,15070	-0,28	-1,21	0,07599	NM_024726	---
8111885	-0,78	-1,72	0,18131	-1,82	-3,53	0,00075	---	TMPRSS7
8081631	-0,78	-1,72	0,04829	-0,83	-1,78	0,00333	NM_001042575	CDC20B
8112053	-0,78	-1,72	0,02104	-0,91	-1,88	0,00773	NM_152623	MYPOP
8037728	-0,78	-1,72	0,04422	-0,69	-1,62	0,04265	NM_001012643	C1orf217
7908966	-0,78	-1,72	0,07932	-1,22	-2,32	0,02128	BC000988	ACRC
8168345	-0,78	-1,72	0,09275	-1,60	-3,03	0,00270	NM_052957	---
8121755	-0,78	-1,72	0,01561	-0,58	-1,49	0,13249	---	C1orf135
7913852	-0,78	-1,72	0,05824	0,27	1,20	0,21330	NM_024037	CT45A1
8170052	-0,78	-1,72	0,16792	-1,73	-3,32	0,00620	NM_001017417	FAM183B
8139163	-0,78	-1,72	0,14111	-0,82	-1,77	0,00216	NR_028347	---
7996391	-0,78	-1,72	0,08157	-1,22	-2,33	0,00021	---	FAM27A
8161503	-0,78	-1,72	0,43937	-2,01	-4,02	0,00860	NR_024060	MIIP
7897849	-0,78	-1,72	0,13051	0,42	1,34	0,21385	NM_021933	---
8102936	-0,78	-1,72	0,28634	-2,26	-4,78	0,01489	---	---
8167951	-0,78	-1,72	0,17772	-2,03	-4,08	0,00008	---	LOC391742
8104615	-0,78	-1,72	0,53620	-1,40	-2,65	0,00040	ENST00000330110	ADCY1
8132667	-0,78	-1,72	0,05136	0,82	1,77	0,03220	NM_021116	---
7897368	-0,78	-1,72	0,01381	-1,06	-2,09	0,02289	---	SILV
7963970	-0,78	-1,72	0,09091	-0,58	-1,49	0,27589	NM_006928	---
7926443	-0,78	-1,72	0,10269	-0,66	-1,58	0,04887	---	IGHMBP2
7942073	-0,78	-1,72	0,09714	0,52	1,44	0,08868	NM_002180	LIPF
7928982	-0,78	-1,72	0,00030	-0,73	-1,66	0,00442	NM_004190	---
7966068	-0,78	-1,72	0,18694	-1,94	-3,85	0,00101	---	GEMIN7
8029617	-0,78	-1,72	0,05601	1,01	2,02	0,03912	NM_024707	KLRD1
7953949	-0,78	-1,72	0,02867	-0,59	-1,51	0,00222	NM_002262	CDK2AP2
7949836	-0,78	-1,72	0,01941	0,00	1,00	0,98925	NM_005851	EEF1A1
8127544	-0,78	-1,72	0,17353	-0,09	-1,06	0,18968	NM_001402	---
8134347	-0,78	-1,72	0,09133	-1,63	-3,09	0,00266	---	MGC10814
8035551	-0,78	-1,72	0,08557	-1,44	-2,72	0,05095	BC004943	ABCB8
8137332	-0,78	-1,72	0,18393	-0,57	-1,49	0,29476	NM_007188	FAM81B
8106950	-0,78	-1,72	0,00485	-1,32	-2,50	0,04879	NM_152548	RHBDF2
8018731	-0,78	-1,72	0,10505	1,45	2,73	0,05160	NM_024599	CYP2E1
7931643	-0,78	-1,72	0,00692	-0,10	-1,07	0,54801	NM_000773	LOC100129455
8056963	-0,78	-1,72	0,23848	-1,24	-2,36	0,00322	ENST00000313266	---
8020451	-0,78	-1,72	0,21584	-0,79	-1,73	0,00046	---	KCNJ16
8009493	-0,78	-1,72	0,11577	-1,08	-2,11	0,03982	NM_170742	FAM75A7
8155371	-0,78	-1,72	0,12960	-1,52	-2,87	0,00718	NM_015667	TRIM10
8124716	-0,78	-1,72	0,02570	-1,21	-2,31	0,04335	NM_006778	TRIM10
8179628	-0,78	-1,72	0,02570	-1,21	-2,31	0,04335	NM_006778	KRTAP3-2
8015169	-0,78	-1,72	0,03275	-0,89	-1,85	0,00941	NM_031959	KRTAP3-2
8019597	-0,78	-1,72	0,03275	-0,89	-1,85	0,00941	NM_031959	C1orf162
7903980	-0,78	-1,72	0,30656	-0,73	-1,66	0,05834	NM_174896	MIR204
8161695	-0,78	-1,72	0,18270	-1,53	-2,89	0,00020	NR_029621	SLC24A6

7966600	-0,78	-1,72	0,01937	0,31	1,24	0,57264	NM_024959	---
8155896	-0,78	-1,72	0,06679	-1,32	-2,49	0,04526	---	TBX15
7919028	-0,78	-1,72	0,00441	-0,57	-1,48	0,11871	NM_152380	---
7945418	-0,78	-1,72	0,14775	-0,07	-1,05	0,84395	---	SOLH
7991877	-0,78	-1,72	0,01156	-0,09	-1,06	0,81051	NM_005632	---
8105799	-0,78	-1,72	0,16960	-0,86	-1,82	0,01294	---	---
7983379	-0,78	-1,72	0,17738	-1,43	-2,69	0,00803	---	ANKZF1
8048489	-0,78	-1,72	0,05173	0,69	1,61	0,07825	NM_018089	FABP12
8151542	-0,78	-1,72	0,12434	-1,51	-2,85	0,00100	NM_001105281	KCNK17
8126163	-0,78	-1,72	0,04886	-1,30	-2,46	0,00206	NM_031460	KRTAP20-2
8068161	-0,78	-1,72	0,10949	-0,80	-1,74	0,02709	NM_181616	SYCP2
8067305	-0,78	-1,72	0,02140	-0,63	-1,55	0,01037	NM_014258	---
8149269	-0,79	-1,72	0,00141	-1,47	-2,76	0,06276	---	MIR412
7976854	-0,79	-1,72	0,08738	-1,64	-3,11	0,00716	NR_030155	---
8007603	-0,79	-1,72	0,03177	-0,37	-1,29	0,18486	---	PKD1L2
8003007	-0,79	-1,72	0,00103	-0,55	-1,46	0,00169	NM_052892	CCDC88C
7980828	-0,79	-1,72	0,08241	2,83	7,09	0,01531	NM_001080414	---
8160784	-0,79	-1,72	0,23717	-1,25	-2,37	0,01080	---	---
8035901	-0,79	-1,72	0,09808	-0,60	-1,51	0,01466	---	---
7905054	-0,79	-1,72	0,00147	-1,20	-2,30	0,02031	---	---
7910583	-0,79	-1,72	0,01505	-1,08	-2,12	0,00120	---	DPYSL5
8040725	-0,79	-1,72	0,00985	0,41	1,32	0,13425	NM_020134	---
8117707	-0,79	-1,72	0,09332	-1,45	-2,73	0,01012	---	FBN3
8033487	-0,79	-1,72	0,02238	-0,85	-1,81	0,02417	NM_032447	BTG4
7951604	-0,79	-1,72	0,00450	-0,83	-1,78	0,06135	NM_017589	ORM2
8157450	-0,79	-1,72	0,06147	-1,32	-2,50	0,09433	NM_000608	RASGEF1B
8101304	-0,79	-1,72	0,03784	-0,82	-1,77	0,00427	NM_152545	SNORD114-6
7976818	-0,79	-1,72	0,02446	-1,31	-2,49	0,01183	NR_003198	---
7956739	-0,79	-1,72	0,00053	-0,91	-1,87	0,00140	---	---
8129313	-0,79	-1,72	0,01864	-0,86	-1,82	0,06104	---	LOC57399
8112306	-0,79	-1,72	0,00291	-1,04	-2,06	0,00599	AF264626	RLN3
8026265	-0,79	-1,72	0,10994	-0,40	-1,32	0,13786	NM_080864	SPAG17
7918973	-0,79	-1,72	0,00135	-0,75	-1,69	0,01202	NM_206996	---
8176665	-0,79	-1,72	0,25268	-1,82	-3,54	0,00207	---	---
8177193	-0,79	-1,72	0,25268	-1,82	-3,54	0,00207	---	IL20RB
8082916	-0,79	-1,72	0,05000	2,90	7,47	0,00041	NM_144717	---
7956399	-0,79	-1,72	0,03106	-0,85	-1,80	0,00233	---	SLC39A3
8032525	-0,79	-1,72	0,10009	0,89	1,85	0,06481	NM_213568	---
8172193	-0,79	-1,72	0,06299	-0,69	-1,61	0,04676	---	PLEK2
7979710	-0,79	-1,73	0,06063	3,51	11,36	0,00061	NM_016445	GGTA1
8163908	-0,79	-1,73	0,04804	-1,03	-2,05	0,00495	NR_003191	MMP20
7951224	-0,79	-1,73	0,06410	-1,14	-2,21	0,02520	NM_004771	---
8175418	-0,79	-1,73	0,01435	-0,61	-1,53	0,00993	---	OR6V1
8136837	-0,79	-1,73	0,17091	-1,22	-2,32	0,01309	NM_001001667	KCNN2
8107408	-0,79	-1,73	0,10827	-1,22	-2,33	0,00018	NM_021614	FAM27E3
8161499	-0,79	-1,73	0,23965	0,91	1,88	0,00059	BC032035	TBC1D17
8030539	-0,79	-1,73	0,05561	-0,06	-1,04	0,90939	NM_024682	EPB41L4B
8163116	-0,79	-1,73	0,03024	3,47	11,08	0,00012	NM_019114	OR5H1
8081194	-0,79	-1,73	0,07724	-0,83	-1,78	0,00123	NM_001005338	SLC6A12
7960177	-0,79	-1,73	0,03249	-0,97	-1,96	0,01498	NM_003044	FOXF2
8116582	-0,79	-1,73	0,12699	-2,08	-4,24	0,00037	NM_001452	CLDN20
8122966	-0,79	-1,73	0,01980	-0,69	-1,61	0,08333	NM_001001346	GPCRLTM7
7986536	-0,79	-1,73	0,13501	-1,04	-2,06	0,02591	AY792621	C8orf8
8149318	-0,79	-1,73	0,01737	-1,11	-2,15	0,00043	ENST00000443854	---
8084943	-0,79	-1,73	0,01434	-1,52	-2,86	0,01920	---	NXF2B
8174149	-0,79	-1,73	0,06556	-0,56	-1,48	0,02150	NM_001099686	LOC100128374
8131098	-0,79	-1,73	0,16177	-1,28	-2,43	0,03578	ENST00000402221	TTC29
8103064	-0,79	-1,73	0,03233	-0,69	-1,61	0,23335	NM_031956	OXGR1
7972428	-0,79	-1,73	0,08882	-1,31	-2,49	0,00743	NM_080818	---
8044698	-0,79	-1,73	0,09357	-0,86	-1,82	0,00293	---	GGT1 // GGT1 // GGT1
8074569	-0,79	-1,73	0,16716	-1,60	-3,02	0,01802	BC128239 // BC128239 // BC128239	POM121L1P
8074714	-0,79	-1,73	0,33265	-2,16	-4,46	0,00019	NR_024591	C1orf223
7901253	-0,79	-1,73	0,13340	-0,64	-1,56	0,12287	BC144026	TERC
8092000	-0,79	-1,73	0,03121	-0,87	-1,83	0,08113	NR_001566	FGF17
8144995	-0,79	-1,73	0,04987	-1,12	-2,17	0,04385	NM_003867	LOC100131170
8019088	-0,79	-1,73	0,06056	-0,05	-1,04	0,56592	AK096606	---
8145609	-0,79	-1,73	0,03794	-0,85	-1,80	0,02446	---	NRXN2
7949080	-0,79	-1,73	0,20200	-0,99	-1,99	0,14565	NM_015080	WFDC10B
8066574	-0,79	-1,73	0,25409	-0,39	-1,31	0,13009	NM_172006	TCL6
7976523	-0,79	-1,73	0,03999	-1,49	-2,82	0,02753	ENST00000332130	KRT15
8015337	-0,79	-1,73	0,14539	-0,58	-1,50	0,01054	NM_002275	SLC25A11
8011747	-0,79	-1,73	0,02363	1,13	2,19	0,10864	NM_003562	---

8079368	-0,79	-1,73	0,00155	-0,45	-1,36	0,22045	---	SELL	
7922219	-0,79	-1,73	0,00251	-0,81	-1,75	0,02267	NM_000655		---
8097749	-0,79	-1,73	0,17604	-1,73	-3,32	0,00763	---	---	
8114581	-0,79	-1,73	0,11096	-0,66	-1,58	0,00007	---	KIAA0284	
7977299	-0,79	-1,73	0,06657	0,54	1,46	0,33099	NM_001112726		MBD3L2
8025129	-0,79	-1,73	0,23664	-1,84	-3,58	0,00040	NM_144614		C16orf93
8000932	-0,79	-1,73	0,02729	-0,80	-1,74	0,08028	NM_001014979		KIF25
8123437	-0,79	-1,73	0,15089	-0,82	-1,77	0,24422	NM_030615		---
7918911	-0,79	-1,73	0,06425	-0,73	-1,66	0,02012	---	HSPBP1	
8039440	-0,79	-1,73	0,30374	0,23	1,18	0,68808	NM_012267		LOC100288105
8114645	-0,79	-1,73	0,25218	0,17	1,12	0,29294	ENST00000319589		ASGR1
8012043	-0,79	-1,73	0,06221	-1,30	-2,46	0,02931	NM_001671		SPACA3
8006423	-0,79	-1,73	0,22812	-0,95	-1,93	0,05248	NM_173847		---
7941863	-0,79	-1,73	0,06140	-1,11	-2,16	0,00473	---	FAM46D	
8168546	-0,79	-1,73	0,04057	-0,79	-1,73	0,00070	NM_001170574		CLEC18B
8002713	-0,79	-1,73	0,15790	-1,27	-2,41	0,03662	NM_001011880		HES5
7911750	-0,79	-1,73	0,09334	-1,53	-2,89	0,04709	NM_001010926		TSPAN1
7901175	-0,79	-1,73	0,05741	3,42	10,71	0,00015	NM_005727		GDF6
8151906	-0,79	-1,73	0,22132	-1,39	-2,63	0,00277	NM_001001557		---
7967418	-0,79	-1,73	0,00999	-0,56	-1,47	0,01158	---	NEUROG3	
7934083	-0,79	-1,73	0,20817	-1,88	-3,69	0,03438	NM_020999		ABCD1
8170704	-0,79	-1,73	0,05381	0,56	1,47	0,47791	NM_000033		KRI1
8034055	-0,79	-1,73	0,12810	0,34	1,27	0,43425	NM_023008		RAB3D
8034202	-0,79	-1,73	0,07184	1,00	2,00	0,03530	NM_004283		OR5H15
8081198	-0,79	-1,73	0,07736	-0,94	-1,92	0,00170	NM_001005515		OR8B8
7952404	-0,79	-1,73	0,44995	-1,90	-3,74	0,00001	NM_012378		MAFG
8019308	-0,79	-1,73	0,02089	1,16	2,23	0,02150	NM_032711		---
7978704	-0,79	-1,73	0,01117	-1,33	-2,51	0,02054	---	LOC494127	
8162187	-0,79	-1,73	0,04967	-0,97	-1,96	0,00056	BC068978	---	
8022007	-0,79	-1,73	0,17886	-1,89	-3,70	0,00767	---	VCX	
8165881	-0,79	-1,73	0,27995	-0,93	-1,90	0,00298	NM_013452		CETN3
8113050	-0,79	-1,73	0,19598	-0,38	-1,30	0,17869	NM_004365		FAM84A
8040374	-0,79	-1,73	0,19082	-1,40	-2,63	0,02722	NM_145175		ZNF584
8031939	-0,79	-1,73	0,18778	0,81	1,75	0,14447	NM_173548		---
8079613	-0,79	-1,73	0,13590	-1,01	-2,01	0,00044	---	MIR455	
8157444	-0,79	-1,73	0,09189	-0,92	-1,90	0,02076	NR_030255		---
8146559	-0,79	-1,73	0,03286	-1,24	-2,37	0,08900	---	---	
8157139	-0,79	-1,73	0,28531	-0,06	-1,04	0,84711	---	LYG1	
8054209	-0,79	-1,73	0,17112	-0,64	-1,56	0,09845	NM_174898		FAM156A
8167749	-0,79	-1,73	0,11004	1,47	2,77	0,00632	NM_014138		---
8042221	-0,79	-1,73	0,32341	-1,29	-2,44	0,02180	---	RYR2	
7910792	-0,79	-1,73	0,02610	-0,64	-1,56	0,00441	NM_001035		---
8005685	-0,79	-1,73	0,03877	-1,42	-2,67	0,04316	---	GPR152	
7949796	-0,79	-1,73	0,11584	-1,73	-3,33	0,01356	NM_206997		ATP6V1E2
8051989	-0,79	-1,73	0,13795	0,35	1,27	0,26455	NM_080653		---
7925703	-0,79	-1,73	0,20193	-1,06	-2,08	0,00307	---	---	
8149345	-0,79	-1,73	0,59325	-3,87	-14,64	0,00004	---	---	
8097124	-0,79	-1,73	0,08682	-1,50	-2,84	0,01106	---	RNF213	
8019071	-0,79	-1,73	0,00935	-0,54	-1,45	0,03878	NM_020914		CD164L2
7914084	-0,79	-1,73	0,26227	0,93	1,91	0,03350	NM_207397		SLC44A5
7917052	-0,79	-1,73	0,02004	-0,55	-1,46	0,16724	NM_152697		FLJ45974
8139699	-0,79	-1,73	0,00660	-1,01	-2,01	0,04975	AK127870	C14orf4	
7980338	-0,79	-1,73	0,26473	1,30	2,47	0,08103	NM_024496		---
8064864	-0,79	-1,73	0,07909	-0,53	-1,44	0,14690	---	FLJ45340	
7945344	-0,79	-1,73	0,34768	-2,21	-4,63	0,01657	NR_024368		GPD1
7955348	-0,79	-1,73	0,25868	-1,15	-2,22	0,00740	NM_005276		---
8136289	-0,79	-1,73	0,18999	-0,80	-1,74	0,09465	---	FAM75A7	
8161471	-0,79	-1,73	0,13158	-1,57	-2,96	0,00680	NM_015667		RFESD
8106978	-0,79	-1,73	0,33960	-0,62	-1,54	0,13597	NM_001131065		KRTAP10-8
8069148	-0,79	-1,73	0,16267	-1,72	-3,30	0,00007	NM_198695		---
7959078	-0,79	-1,73	0,21517	0,76	1,69	0,29482	---	---	
7970426	-0,79	-1,73	0,13146	-1,55	-2,92	0,00235	---	TLX1	
7929947	-0,79	-1,73	0,08249	-0,66	-1,58	0,09349	NM_005521		CCR4
8078442	-0,79	-1,73	0,07748	-1,25	-2,38	0,03586	NM_005508		SUSD2
8071861	-0,79	-1,73	0,08482	2,02	4,06	0,01218	NM_019601		---
7919024	-0,79	-1,73	0,24477	-0,95	-1,93	0,00118	---	LOC100127980	
8036302	-0,79	-1,73	0,08867	0,17	1,13	0,69847	AK090827	---	
7948141	-0,80	-1,74	0,08508	-1,13	-2,19	0,02050	---	---	
8146118	-0,80	-1,74	0,13643	-0,98	-1,97	0,04191	---	---	
7997164	-0,80	-1,74	0,17435	-1,02	-2,02	0,05830	---	---	
7916781	-0,80	-1,74	0,21400	-1,41	-2,65	0,01930	---	MYO3A	
7926728	-0,80	-1,74	0,00693	1,93	3,82	0,01006	NM_017433		---
8102728	-0,80	-1,74	0,02226	0,11	1,08	0,39338	---	PCDHAC1	

8108633	-0,80	-1,74	0,01106	-0,47	-1,38	0,13485	NM_018898		CNTD1
8007388	-0,80	-1,74	0,06277	-1,10	-2,14	0,16847	NM_173478		C6orf103
8122561	-0,80	-1,74	0,04468	-0,96	-1,95	0,00431	NM_024694		---
8121138	-0,80	-1,74	0,06676	-1,25	-2,39	0,05581	---	ATP1A3	
8037079	-0,80	-1,74	0,19468	1,83	3,56	0,01095	NM_152296		---
8115395	-0,80	-1,74	0,00641	-1,54	-2,90	0,02152	---	---	
7991184	-0,80	-1,74	0,00182	-0,55	-1,47	0,01838	---	ZFHX2	
7978056	-0,80	-1,74	0,01703	-0,08	-1,05	0,54344	BC144336	C4BPB	
7909306	-0,80	-1,74	0,16268	-1,48	-2,80	0,01379	NM_000716		TSEN34
8031145	-0,80	-1,74	0,19002	-0,02	-1,02	0,96585	NM_024075		---
7965484	-0,80	-1,74	0,02943	-1,18	-2,26	0,00141	---	ITM2A	
8173755	-0,80	-1,74	0,13654	-0,75	-1,68	0,00181	NM_004867		CLIC5
8126729	-0,80	-1,74	0,02291	-1,09	-2,13	0,00037	NM_001114086		ACCS
7939507	-0,80	-1,74	0,18388	-0,17	-1,13	0,57753	NM_032592		MIR127
7976804	-0,80	-1,74	0,02569	-0,45	-1,37	0,18439	NR_029696		TBX6
8000779	-0,80	-1,74	0,05456	-0,43	-1,34	0,15550	NM_004608		MS4A2
7940226	-0,80	-1,74	0,13005	-0,81	-1,75	0,00536	NM_000139		OR8U1
7939983	-0,80	-1,74	0,09520	-0,78	-1,72	0,00486	NM_001005204		LOC441795
8007310	-0,80	-1,74	0,04349	-1,23	-2,35	0,00876	ENST00000447408		FLJ46300
7937073	-0,80	-1,74	0,08397	-1,59	-3,02	0,00153	ENST00000341866		---
7907700	-0,80	-1,74	0,22844	-1,21	-2,31	0,03925	---	---	
8157189	-0,80	-1,74	0,12018	-0,24	-1,18	0,32503	---	OR56A5	
7946113	-0,80	-1,74	0,03093	-0,92	-1,90	0,00035	NM_001146033		KCNV1
8152369	-0,80	-1,74	0,05600	-1,33	-2,52	0,00919	NM_014379		---
8120462	-0,80	-1,74	0,12894	-0,76	-1,70	0,00560	---	FOXR2	
8167895	-0,80	-1,74	0,00464	-0,64	-1,55	0,00901	NM_198451		LOC100128501
8158542	-0,80	-1,74	0,23576	-1,95	-3,86	0,00015	AK092192	---	
8099924	-0,80	-1,74	0,26627	-1,15	-2,21	0,05384	---	ANKLE1	
8026712	-0,80	-1,74	0,00057	-0,51	-1,42	0,26404	NM_152363		---
8045134	-0,80	-1,74	0,00037	-1,25	-2,39	0,01658	---	CCDC157	
8072316	-0,80	-1,74	0,17726	-0,89	-1,85	0,06887	NM_001017437		CLCNKA
7898300	-0,80	-1,74	0,06959	-0,10	-1,07	0,65250	NM_004070		DNAJC22
7955211	-0,80	-1,74	0,04067	-0,30	-1,23	0,31543	NM_024902		TTTY12
8176471	-0,80	-1,74	0,07549	-0,83	-1,78	0,00277	NR_001551		SNORD109A
7981947	-0,80	-1,74	0,12484	-0,05	-1,04	0,70648	NR_001295		SNORD109A
7982098	-0,80	-1,74	0,12484	-0,05	-1,04	0,70648	NR_001295		C9orf153
8162132	-0,80	-1,74	0,00356	-0,77	-1,71	0,00991	BC108665	---	
7989243	-0,80	-1,74	0,01364	-0,86	-1,81	0,00250	---	---	
8127532	-0,80	-1,74	0,20460	-1,90	-3,74	0,00370	---	---	
7971415	-0,80	-1,74	0,02327	-0,55	-1,47	0,04275	---	KPRP	
7905515	-0,80	-1,74	0,03444	-1,79	-3,45	0,01820	NM_001025231		UBQLN3
7946078	-0,80	-1,74	0,20160	-0,90	-1,87	0,04083	NM_017481		---
8127997	-0,80	-1,74	0,09945	-0,49	-1,41	0,00337	---	HP	
7997188	-0,80	-1,74	0,36691	-1,19	-2,29	0,12178	NM_005143		KRT17
8013042	-0,80	-1,74	0,11050	-1,53	-2,89	0,01305	NM_000422		PRKAR1B
8137675	-0,80	-1,74	0,11670	-0,57	-1,48	0,18158	NM_002735		GOLGA6L9
7985409	-0,80	-1,74	0,41858	-0,06	-1,05	0,91497	NM_198181		GOLGA6L9
7985450	-0,80	-1,74	0,41858	-0,06	-1,05	0,91497	NM_198181		MMP11
8071758	-0,80	-1,74	0,21520	-0,01	-1,01	0,97391	NM_005940		RP9P
8138930	-0,80	-1,74	0,05753	0,16	1,12	0,18975	NR_003500		EPB41L4A
8113512	-0,80	-1,74	0,00033	0,47	1,39	0,22951	NM_022140		ZNF606
8031843	-0,80	-1,74	0,15067	-0,92	-1,89	0,01973	NM_025027		---
7965038	-0,80	-1,74	0,30920	-1,59	-3,02	0,02016	---	SYNGR3	
7992447	-0,80	-1,74	0,11172	0,04	1,03	0,88590	NM_004209		EMR2
8034873	-0,80	-1,74	0,00500	0,72	1,65	0,02926	NM_013447		---
7981779	-0,80	-1,74	0,33418	-0,97	-1,95	0,06170	---	MIR106B	
8141423	-0,80	-1,74	0,20927	-0,94	-1,91	0,02201	NR_029831		RGS6
7975482	-0,80	-1,74	0,15786	0,79	1,73	0,16766	NM_004296		SHROOM1
8114068	-0,80	-1,74	0,22960	-1,21	-2,32	0,01504	NM_133456		PLA2G16
7948987	-0,80	-1,74	0,07731	-0,24	-1,18	0,37518	NM_007069		---
8123060	-0,80	-1,74	0,01601	-0,95	-1,93	0,00627	---	F5	
7922174	-0,80	-1,74	0,05518	-1,18	-2,27	0,00043	NM_000130		PROCA1
8013747	-0,80	-1,74	0,11810	-0,76	-1,69	0,08777	NM_152465		IP6K3
8125780	-0,80	-1,74	0,01040	-0,97	-1,96	0,14300	NM_054111		---
8093451	-0,80	-1,74	0,30610	-0,20	-1,15	0,76474	---	ZACN	
8010036	-0,80	-1,74	0,07830	-1,16	-2,24	0,02226	NM_180990		ITGB7
7963614	-0,80	-1,74	0,08528	-1,48	-2,79	0,00012	NM_000889		SRC
8062377	-0,80	-1,74	0,03807	-0,11	-1,08	0,78427	NM_005417		MIR99B
8030842	-0,80	-1,74	0,03209	-1,24	-2,37	0,11202	NR_029843		SNORD115-33
7982072	-0,80	-1,74	0,02541	-1,31	-2,47	0,00116	NR_003348		POM121L1P
8071530	-0,80	-1,74	0,38626	-1,01	-2,02	0,33263	NR_024591		POM121L1P
8074712	-0,80	-1,74	0,38626	-1,01	-2,02	0,33263	NR_024591		---
7910674	-0,80	-1,74	0,10200	-1,33	-2,51	0,03218	---	CDHR3	

8135341	-0,80	-1,74	0,04253	-1,13	-2,19	0,01588	NM_152750		LOH3CR2A
8077499	-0,80	-1,74	0,02311	-0,56	-1,47	0,08014	AF086709	KCNG1	
8067029	-0,80	-1,74	0,03855	-0,45	-1,37	0,49161	NM_002237		TTYH2
8009568	-0,80	-1,74	0,13136	2,39	5,24	0,00498	NM_032646		ANGPT2
8149071	-0,80	-1,74	0,04436	-1,07	-2,09	0,00815	NM_001147		GOLGA8F
7991668	-0,80	-1,74	0,14576	-0,61	-1,52	0,25358	NR_033351		INA
7930208	-0,80	-1,74	0,12289	-1,08	-2,11	0,01940	NM_032727		APCS
7906458	-0,80	-1,74	0,12647	-1,73	-3,31	0,00114	NM_001639		QTRTD1
8081786	-0,80	-1,74	0,04750	1,70	3,25	0,00000	NM_024638		GTSE1
8073858	-0,80	-1,74	0,00314	1,04	2,05	0,01215	NM_016426		---
7962242	-0,80	-1,74	0,02446	-0,91	-1,88	0,11596	---	PTK6	
8067662	-0,80	-1,74	0,07551	-0,20	-1,15	0,69092	NM_005975		SULT1A3
7994582	-0,80	-1,74	0,02542	1,14	2,20	0,01141	NM_003166		KIAA0415
8131265	-0,80	-1,74	0,30127	-0,28	-1,21	0,33230	NM_014855		---
8174279	-0,80	-1,74	0,04913	-0,70	-1,63	0,03287	---	---	
7926981	-0,80	-1,74	0,09361	-0,66	-1,58	0,01574	---	---	
8140855	-0,80	-1,74	0,04794	-0,59	-1,51	0,00376	---	PPP1R9B	
8016628	-0,80	-1,75	0,00057	0,02	1,02	0,94670	NM_032595		SLC22A24
7948951	-0,80	-1,75	0,11106	-1,42	-2,68	0,00017	NM_001136506		UPK3A
8073743	-0,80	-1,75	0,06563	-0,39	-1,31	0,40733	NM_006953		FLJ44790
8063869	-0,80	-1,75	0,20803	-0,28	-1,22	0,13248	AK126744	SLC7A10	
8035993	-0,80	-1,75	0,16919	0,28	1,22	0,39584	NM_019849		MICALL2
8137715	-0,80	-1,75	0,00084	0,47	1,38	0,37463	NM_182924		PLEKHG3
7975095	-0,80	-1,75	0,02565	-0,05	-1,04	0,79021	NM_015549		---
8121214	-0,80	-1,75	0,33287	-1,32	-2,49	0,10359	---	---	
8094739	-0,80	-1,75	0,06688	-1,02	-2,02	0,03130	---	---	
8023591	-0,80	-1,75	0,00975	0,24	1,18	0,56028	---	---	
7914805	-0,80	-1,75	0,11172	-1,58	-2,98	0,01208	---	NCRNA00205	
8070891	-0,80	-1,75	0,15054	-2,25	-4,75	0,00140	AF426264	HYAL4	
8135827	-0,80	-1,75	0,09972	-0,86	-1,81	0,00502	NM_012269		AIDA
7924491	-0,80	-1,75	0,23924	-1,22	-2,33	0,00884	NM_022831		DPRX
8031013	-0,80	-1,75	0,24520	-0,53	-1,44	0,12687	NM_001012728		HHIPL2
7924465	-0,80	-1,75	0,08817	-1,23	-2,35	0,00158	NM_024746		LOC100131496
8063209	-0,80	-1,75	0,25765	-1,34	-2,54	0,00317	NR_024594		---
8022502	-0,80	-1,75	0,05682	-2,66	-6,32	0,00223	---	ZSCAN18	
8039730	-0,80	-1,75	0,15825	-0,53	-1,45	0,09586	NM_023926		POLN
8098958	-0,80	-1,75	0,18296	-0,88	-1,84	0,03934	NM_181808		---
8043020	-0,80	-1,75	0,13462	-1,15	-2,22	0,00027	---	FLJ43826	
8006836	-0,80	-1,75	0,04602	-1,59	-3,02	0,00031	BC111725	KIAA1257	
8090518	-0,80	-1,75	0,09865	-0,91	-1,88	0,00030	ENST00000265068		C1orf50
7900603	-0,81	-1,75	0,09287	-0,68	-1,60	0,00768	AK299874	TBX4	
8008982	-0,81	-1,75	0,04980	2,15	4,44	0,00009	NM_018488		ARHGDIG
7991800	-0,81	-1,75	0,02510	0,10	1,07	0,55354	NM_001176		CBLN1
8001329	-0,81	-1,75	0,02316	-0,57	-1,49	0,06309	NM_004352		ZIC4
8091342	-0,81	-1,75	0,01268	-1,21	-2,32	0,02533	NM_032153		ISG15
7896817	-0,81	-1,75	0,04343	0,00	-1,00	0,99305	NM_005101		---
8151052	-0,81	-1,75	0,16052	-1,69	-3,22	0,01963	---	---	
8020664	-0,81	-1,75	0,03908	-1,08	-2,11	0,05456	---	---	
8014861	-0,81	-1,75	0,25232	-1,37	-2,59	0,00874	---	A3GALT2	
7914643	-0,81	-1,75	0,02028	-0,97	-1,96	0,00101	ENST00000442999		SNORD15B
7942594	-0,81	-1,75	0,04942	-0,73	-1,66	0,04670	NR_000025		GMIP
8035714	-0,81	-1,75	0,17029	-0,76	-1,69	0,13434	NM_016573		---
8100553	-0,81	-1,75	0,07256	-1,43	-2,70	0,00040	---	NOS1	
7966779	-0,81	-1,75	0,02993	-0,97	-1,96	0,04642	NM_000620		---
8100068	-0,81	-1,75	0,11865	-1,26	-2,39	0,00234	---	SULT1A2	
8000582	-0,81	-1,75	0,03870	-0,15	-1,11	0,67938	NM_001054		CYLC2
8156971	-0,81	-1,75	0,00217	-0,64	-1,56	0,00248	NM_001340		---
7922324	-0,81	-1,75	0,04132	-0,54	-1,45	0,08358	---	---	
7916667	-0,81	-1,75	0,13419	-0,79	-1,73	0,00563	---	---	
7932676	-0,81	-1,75	0,01336	-1,61	-3,06	0,03287	---	CR1	
7909371	-0,81	-1,75	0,08245	-0,65	-1,57	0,01735	NM_000573		---
8121041	-0,81	-1,75	0,02959	-0,75	-1,68	0,00315	---	NPY5R	
8098146	-0,81	-1,75	0,00377	-0,58	-1,50	0,00104	NM_006174		HTR3A
7943969	-0,81	-1,75	0,00509	-0,97	-1,96	0,08682	NM_213621		FLJ37786
8053731	-0,81	-1,75	0,31383	-1,98	-3,95	0,00594	AK095105	RAB11FIP5	
8052994	-0,81	-1,75	0,14093	-0,89	-1,85	0,04800	NM_015470		MIR30E
7900488	-0,81	-1,75	0,01574	-0,20	-1,15	0,46183	NR_029846		RPE65
7916882	-0,81	-1,75	0,01078	-1,13	-2,19	0,00016	NM_000329		SVOPL
8143209	-0,81	-1,75	0,00830	-1,16	-2,23	0,06553	NM_001139456		LOC388152
7985444	-0,81	-1,75	0,00031	-0,19	-1,14	0,75316	NR_027001		HSD3B7
7995007	-0,81	-1,75	0,02801	0,24	1,18	0,45593	NM_025193		SYCP1
7904168	-0,81	-1,75	0,05317	-0,74	-1,67	0,00546	NM_003176		RNU5F
7915590	-0,81	-1,75	0,06914	-0,46	-1,38	0,02420	NR_002753		SLC36A2

8115287	-0,81	-1,75	0,06351	-1,36	-2,57	0,00970	NM_181776	OR10P1
7956007	-0,81	-1,75	0,15414	-2,24	-4,72	0,00069	NM_206899	STX1B
8000963	-0,81	-1,75	0,01930	-0,55	-1,46	0,03365	NM_052874	NANOS3
8026214	-0,81	-1,75	0,16940	-1,55	-2,93	0,02976	NM_001098622	GSDMC
8152828	-0,81	-1,75	0,03181	-0,35	-1,28	0,01834	NM_031415	---
7957602	-0,81	-1,75	0,02497	-0,95	-1,94	0,03054	---	FAM83A
8148184	-0,81	-1,75	0,10786	2,96	7,78	0,00151	NM_032899	TAS2R10
7961249	-0,81	-1,75	0,05409	-0,39	-1,31	0,12657	NM_023921	---
7917904	-0,81	-1,75	0,08097	-0,70	-1,62	0,05485	---	EXD3
8165512	-0,81	-1,75	0,06462	-0,77	-1,71	0,10318	NM_017820	---
8148147	-0,81	-1,75	0,05190	-0,97	-1,96	0,05728	---	---
8175153	-0,81	-1,75	0,00198	-1,17	-2,25	0,00030	---	PPBPL2
8100990	-0,81	-1,75	0,02395	-1,01	-2,02	0,03071	NR_026769	---
8168461	-0,81	-1,75	0,18467	-0,81	-1,75	0,00042	---	---
8176284	-0,81	-1,75	0,18467	-0,81	-1,75	0,00042	---	C19orf29
8032637	-0,81	-1,75	0,23377	0,06	1,04	0,83847	NM_001080543	KHDRBS2
8127370	-0,81	-1,75	0,14340	-1,82	-3,52	0,00058	NM_152688	DEDD2
8037144	-0,81	-1,75	0,00315	0,65	1,57	0,20590	NM_133328	TBC1D21
7984759	-0,81	-1,75	0,07407	-1,31	-2,48	0,02072	NM_153356	---
8067834	-0,81	-1,75	0,07841	-1,45	-2,73	0,00063	---	KCNMB1
8115756	-0,81	-1,75	0,11995	-1,43	-2,69	0,00072	NM_004137	---
8112014	-0,81	-1,75	0,28039	-0,20	-1,15	0,09308	---	C20orf70
8061847	-0,81	-1,75	0,22653	-1,35	-2,55	0,00763	NM_080574	CD200R1
8089568	-0,81	-1,75	0,02170	-1,27	-2,41	0,01559	NM_138806	BLK
8144625	-0,81	-1,75	0,03677	-1,34	-2,52	0,00127	NM_001715	PIWIL3
8075026	-0,81	-1,75	0,09595	-1,21	-2,32	0,00214	NM_001008496	RBM19
7966638	-0,81	-1,75	0,10625	0,61	1,53	0,20010	NM_016196	GUCA2B
7900531	-0,81	-1,75	0,15771	-1,36	-2,56	0,00714	NM_007102	CLDN9
7992782	-0,81	-1,75	0,12227	-1,11	-2,17	0,00693	NM_020982	CENPL
7922391	-0,81	-1,75	0,03072	0,10	1,07	0,81375	NM_001127181	---
7953098	-0,81	-1,75	0,14228	-1,06	-2,08	0,08565	---	CCDC33
7984819	-0,81	-1,75	0,12294	-0,76	-1,69	0,13665	NM_025055	POU3F4
8168567	-0,81	-1,75	0,04894	-1,69	-3,24	0,00433	NM_000307	MAPKBP1
7982985	-0,81	-1,75	0,01723	0,04	1,03	0,92922	NM_014994	NPY1R
8103494	-0,81	-1,75	0,09106	-0,21	-1,16	0,67042	NM_000909	ABCG8
8041696	-0,81	-1,75	0,03787	-1,28	-2,43	0,03255	NM_022437	SLC8A2
8037888	-0,81	-1,75	0,04310	-1,19	-2,28	0,06478	NM_015063	GPR150
8106976	-0,81	-1,75	0,18250	-2,73	-6,63	0,00263	NM_199243	TRO
8167835	-0,81	-1,75	0,22490	-1,76	-3,40	0,01217	NM_001039705	BARX2
7945162	-0,81	-1,75	0,16999	-0,22	-1,16	0,52810	NM_003658	SLC4A1
8015991	-0,81	-1,75	0,06562	-1,01	-2,02	0,00558	NM_000342	---
8122670	-0,81	-1,75	0,04759	-0,51	-1,43	0,13382	---	C19orf24
8024246	-0,81	-1,75	0,09869	0,23	1,17	0,55101	NM_017914	---
8061092	-0,81	-1,75	0,11914	-0,70	-1,62	0,02525	---	SCGN
8117288	-0,81	-1,75	0,03061	-0,95	-1,94	0,01615	NM_006998	CCK
8086391	-0,81	-1,75	0,12763	-1,98	-3,93	0,00007	NM_000729	KIAA0087
8138686	-0,81	-1,75	0,06954	-1,11	-2,16	0,01073	NR_022006	FLJ33544
8060790	-0,81	-1,75	0,06751	-0,59	-1,51	0,16849	AK090863 LGI3	
8149685	-0,81	-1,75	0,00905	0,10	1,07	0,76561	NM_139278	MIR219-2
8164438	-0,81	-1,75	0,22929	-0,31	-1,24	0,19985	NR_029837	STX1A
8140113	-0,81	-1,76	0,23705	1,27	2,42	0,02356	NM_004603	RUNDC3A
8007607	-0,81	-1,76	0,00136	-0,86	-1,82	0,04419	NM_006695	---
8144512	-0,81	-1,76	0,11767	-1,04	-2,06	0,01298	---	EFCAB4B
7960365	-0,81	-1,76	0,25288	2,14	4,41	0,01987	NM_001144958	SLC5A7
8044191	-0,81	-1,76	0,01125	-0,87	-1,83	0,00770	NM_021815	TXNDC6
8090972	-0,81	-1,76	0,16560	-1,12	-2,18	0,02780	NM_178130	MIR148A
8138668	-0,81	-1,76	0,04262	-1,34	-2,53	0,00385	NR_029597	---
8176568	-0,81	-1,76	0,15016	-0,46	-1,37	0,10337	---	JUND
8035445	-0,81	-1,76	0,06424	-0,39	-1,31	0,53968	NM_005354	POTEG
7977456	-0,81	-1,76	0,13705	-2,11	-4,32	0,00609	NR_027480	PRDM7
8003571	-0,81	-1,76	0,13296	-0,76	-1,69	0,31373	NM_001098173	LOC100128262
8084921	-0,81	-1,76	0,17054	-1,07	-2,11	0,00161	AK094115 C15orf55	
7982516	-0,81	-1,76	0,05437	-1,09	-2,13	0,01178	NM_175741	MEPCE
8134821	-0,81	-1,76	0,01097	0,18	1,13	0,77168	NM_019606	OR2V2
8110647	-0,81	-1,76	0,22215	-0,87	-1,82	0,07718	NM_206880	---
7945894	-0,81	-1,76	0,30999	-0,09	-1,06	0,70040	---	CHMP1A
8003467	-0,81	-1,76	0,04751	0,62	1,54	0,38033	NM_001083314	---
7969286	-0,81	-1,76	0,01143	-0,92	-1,89	0,00430	---	DGKZ
7968890	-0,81	-1,76	0,00403	0,43	1,35	0,32099	NM_003646	---
7916489	-0,81	-1,76	0,04737	-0,98	-1,97	0,00007	---	MPZ
7921852	-0,81	-1,76	0,38733	-0,58	-1,50	0,00938	NM_000530	TREX2
8175802	-0,81	-1,76	0,09116	-1,23	-2,35	0,10859	NM_080701	---
8105338	-0,81	-1,76	0,04766	-1,40	-2,64	0,00527	---	DYSF

8042637	-0,81	-1,76	0,01051	1,64	3,11	0,00244	NM_001130978	HK2
8042942	-0,81	-1,76	0,10106	-0,14	-1,10	0,83198	NM_000189	GPR132
7981530	-0,81	-1,76	0,17840	-0,39	-1,31	0,48490	NM_013345	DNAJB13
7950299	-0,81	-1,76	0,01863	-1,09	-2,13	0,00254	NM_153614	SERPINB7
8021623	-0,81	-1,76	0,05481	-0,60	-1,51	0,04578	NM_003784	LGSN
8127380	-0,81	-1,76	0,04489	1,96	3,89	0,00852	NM_016571	TRIM9
7979108	-0,81	-1,76	0,01256	-0,91	-1,88	0,00421	NM_052978	OVCH2
7946306	-0,81	-1,76	0,08420	-1,83	-3,55	0,00068	NM_198185	SYCP2L
8116874	-0,81	-1,76	0,02986	0,51	1,43	0,06130	NM_001040274	RIMS4
8066451	-0,81	-1,76	0,06622	-0,47	-1,38	0,23924	NM_182970	RRP7B
8076463	-0,81	-1,76	0,00150	-0,80	-1,74	0,08414	BC014647 KCNJ15	
8068583	-0,81	-1,76	0,01075	-1,18	-2,27	0,00243	NM_002243	---
7981235	-0,81	-1,76	0,02650	-1,60	-3,02	0,00053	--- MAMDC4	
8159448	-0,81	-1,76	0,06420	-0,96	-1,95	0,16085	NM_206920	ACER1
8033151	-0,81	-1,76	0,11068	-0,61	-1,53	0,04437	NM_133492	---
8073341	-0,81	-1,76	0,04525	-0,67	-1,59	0,00214	--- ---	
8002264	-0,81	-1,76	0,05055	-1,21	-2,31	0,10105	--- ASCL2	
7945730	-0,81	-1,76	0,13393	-1,11	-2,15	0,08890	NM_005170	---
8000600	-0,81	-1,76	0,10963	-1,27	-2,40	0,03465	--- ZNF428	
8037355	-0,81	-1,76	0,02524	-0,96	-1,95	0,00022	NM_182498	---
8113481	-0,82	-1,76	0,13795	-0,89	-1,85	0,04872	--- MIR29C	
7923974	-0,82	-1,76	0,00868	-1,14	-2,21	0,01042	NR_029832	KCNH1
7924071	-0,82	-1,76	0,00099	1,31	2,47	0,04173	NM_172362	DNAH6
8043055	-0,82	-1,76	0,00190	-0,82	-1,76	0,00283	NM_001370	ACTL7B
8163019	-0,82	-1,76	0,27509	-1,10	-2,14	0,00392	NM_006686	---
8012839	-0,82	-1,76	0,00609	-1,22	-2,33	0,03150	--- PTGIR	
8037775	-0,82	-1,76	0,00748	-0,92	-1,89	0,09396	NM_000960	PPY2
8005836	-0,82	-1,76	0,23806	-1,27	-2,41	0,00933	NR_002181	TRAF3IP3
7909455	-0,82	-1,76	0,05006	-1,17	-2,25	0,00210	NM_025228	LOC51145
8164196	-0,82	-1,76	0,03081	-1,16	-2,23	0,04878	AF159054 ---	
8161482	-0,82	-1,76	0,15166	-1,60	-3,03	0,00468	--- ---	
8161566	-0,82	-1,76	0,15166	-1,60	-3,03	0,00468	--- LOC729173	
7944527	-0,82	-1,76	0,07729	-0,77	-1,71	0,05896	ENST00000319763	---
8134431	-0,82	-1,76	0,23619	-0,51	-1,42	0,29641	--- ---	
8069517	-0,82	-1,76	0,10210	-1,07	-2,10	0,01354	--- ---	
7974027	-0,82	-1,76	0,21873	-1,72	-3,29	0,00509	--- ZNF530	
8031778	-0,82	-1,76	0,00706	-0,77	-1,71	0,05101	NM_020880	CELSR3
8087145	-0,82	-1,76	0,02014	-0,07	-1,05	0,88463	NM_001407	RTN1
7979455	-0,82	-1,76	0,28681	-0,83	-1,78	0,01934	NM_021136	OR2A1
8143627	-0,82	-1,76	0,11223	-0,73	-1,66	0,01826	NM_001005287	---
7922887	-0,82	-1,76	0,15960	-0,99	-1,99	0,01010	--- NELL1	
7938925	-0,82	-1,76	0,00560	-0,84	-1,79	0,01709	NM_006157	COL4A3
8048782	-0,82	-1,76	0,02681	-0,89	-1,86	0,00604	NM_000091	C6orf132
8126382	-0,82	-1,76	0,03691	0,35	1,27	0,34061	NM_001164446	---
7988258	-0,82	-1,76	0,04611	-0,89	-1,86	0,14511	--- DGKQ	
8098822	-0,82	-1,76	0,14310	-1,18	-2,26	0,04691	NM_001347	---
7982337	-0,82	-1,76	0,10729	-0,64	-1,55	0,05985	--- WIT1	
7939131	-0,82	-1,76	0,00508	-1,28	-2,43	0,02740	NR_023920	LINGO3
8032478	-0,82	-1,76	0,37300	-0,67	-1,60	0,16776	NM_001101391	SLC1A6
8034907	-0,82	-1,76	0,12143	-1,44	-2,71	0,00859	NM_005071	---
8080617	-0,82	-1,76	0,13635	-1,77	-3,41	0,00573	--- RNF148	
8142622	-0,82	-1,76	0,00670	-1,60	-3,03	0,00668	NM_198085	---
8100549	-0,82	-1,76	0,06800	-0,99	-1,99	0,00225	--- ---	
8049665	-0,82	-1,76	0,07567	-1,65	-3,15	0,00119	--- RPS6KA4	
7941039	-0,82	-1,76	0,17154	-0,50	-1,42	0,22367	NM_003942	---
8011732	-0,82	-1,76	0,02097	-0,92	-1,89	0,01685	--- FABP2	
8102523	-0,82	-1,76	0,00969	-0,73	-1,66	0,02859	NM_000134	KCNB2
8146908	-0,82	-1,76	0,03540	-1,21	-2,31	0,00930	NM_004770	CADM4
8037363	-0,82	-1,76	0,13860	0,04	1,03	0,89895	NM_145296	UNK
8018445	-0,82	-1,76	0,06272	-0,56	-1,48	0,00212	NM_001080419	---
8168674	-0,82	-1,76	0,25761	1,24	2,37	0,04589	--- ---	
7929812	-0,82	-1,76	0,02832	-1,51	-2,85	0,00483	--- HBCBP	
7956285	-0,82	-1,76	0,10457	-0,82	-1,76	0,01260	AF529371 ---	
7954697	-0,82	-1,76	0,23901	-1,74	-3,34	0,10854	--- BEST3	
7964852	-0,82	-1,76	0,01129	-0,81	-1,75	0,00053	NM_032735	CCR7
8015031	-0,82	-1,76	0,23840	-1,69	-3,23	0,00145	NM_001838	TRIM71
8078435	-0,82	-1,76	0,18990	-1,53	-2,89	0,01156	NM_001039111	SYP
8172573	-0,82	-1,76	0,00449	-0,56	-1,47	0,07494	NM_003179	---
8175365	-0,82	-1,76	0,05200	-1,12	-2,18	0,02003	--- GBP6	
7902850	-0,82	-1,76	0,01363	-1,10	-2,15	0,07448	NM_198460	CXCR3
8173493	-0,82	-1,76	0,04290	-1,95	-3,87	0,00194	NM_001142797	TPPP2
7973142	-0,82	-1,76	0,08859	-1,64	-3,11	0,00098	NM_173846	FGD3
8156373	-0,82	-1,77	0,16858	-0,93	-1,91	0,09067	NM_001083536	CES7

8001464	-0,82	-1,77	0,13959	-1,34	-2,53	0,01559	NM_001143685	---
8080142	-0,82	-1,77	0,01555	-1,97	-3,91	0,01016	---	C9orf86
8159415	-0,82	-1,77	0,01810	0,55	1,46	0,30958	NM_024718	---
8028192	-0,82	-1,77	0,04492	-0,99	-1,98	0,01703	---	OR2L8
7911241	-0,82	-1,77	0,40730	-3,10	-8,57	0,00023	NM_001001963	OR2M3
7911267	-0,82	-1,77	0,33790	-1,18	-2,27	0,06843	NM_001004689	CTNNBL1
8062409	-0,82	-1,77	0,01108	1,02	2,03	0,00530	NM_030877	GPR162
7953442	-0,82	-1,77	0,02782	-0,96	-1,95	0,00466	NM_019858	C8orf79
8144712	-0,82	-1,77	0,17967	-0,33	-1,26	0,11337	NM_001099677	SPATA21
7912764	-0,82	-1,77	0,03712	-0,69	-1,61	0,02748	NM_198546	SLC26A3
8142171	-0,82	-1,77	0,03939	-1,06	-2,09	0,11840	NM_000111	CYP2C8
7935169	-0,82	-1,77	0,00119	-0,49	-1,40	0,12716	NM_000770	MYH3
8012787	-0,82	-1,77	0,02790	-1,24	-2,37	0,00224	NM_002470	---
7981373	-0,82	-1,77	0,00309	-1,24	-2,37	0,00688	---	PI3
8062927	-0,82	-1,77	0,20787	-0,01	-1,01	0,97409	NM_002638	IGHA1
7981722	-0,82	-1,77	0,63930	-4,74	-26,78	0,00014	AK128476	KLRK1
7961151	-0,82	-1,77	0,00779	-0,89	-1,85	0,02782	NM_007360	NHLH2
7918891	-0,82	-1,77	0,09062	-0,51	-1,43	0,04301	NM_005599	EXOC3L2
8037513	-0,82	-1,77	0,08674	-1,16	-2,23	0,08529	NM_138568	RADIL
8137906	-0,82	-1,77	0,17778	-1,09	-2,13	0,05580	NM_018059	---
7953747	-0,82	-1,77	0,01413	-0,51	-1,42	0,02795	---	TAS2R14
7961279	-0,82	-1,77	0,11803	-0,50	-1,42	0,36746	NM_023922	---
8072131	-0,82	-1,77	0,07713	-1,22	-2,33	0,00434	---	LOC645261
8112998	-0,82	-1,77	0,16089	-0,95	-1,93	0,18402	AF258587	SLFN14
8014259	-0,82	-1,77	0,04644	-1,49	-2,80	0,01320	NM_001129820	---
8060736	-0,82	-1,77	0,09607	-0,82	-1,76	0,05498	---	MRPL4
8025586	-0,82	-1,77	0,12416	0,44	1,36	0,37213	NM_146388	C11orf83
7940669	-0,82	-1,77	0,10628	0,57	1,49	0,08903	NM_001085372	SMPD3
8002249	-0,82	-1,77	0,11779	-0,99	-1,99	0,03132	NM_018667	OR4F16
7896744	-0,82	-1,77	0,66272	-2,19	-4,58	0,00469	NM_001005277	OR4F16
7911345	-0,82	-1,77	0,66272	-2,19	-4,58	0,00469	NM_001005277	OR4F16
8110672	-0,82	-1,77	0,66272	-2,19	-4,58	0,00469	NM_001005277	KIAA2013
7912511	-0,82	-1,77	0,27944	-0,23	-1,17	0,66089	NM_138346	CRABP1
7985159	-0,82	-1,77	0,04970	0,12	1,09	0,66025	NM_004378	NCALD
8152119	-0,82	-1,77	0,05499	-0,69	-1,62	0,03033	NM_001040624	---
8115037	-0,82	-1,77	0,15576	-1,20	-2,29	0,00590	---	---
8099025	-0,82	-1,77	0,01299	-0,70	-1,63	0,00736	---	COL10A1
8129082	-0,82	-1,77	0,07165	-1,81	-3,51	0,00066	NM_000493	LOC613206
8164062	-0,82	-1,77	0,03456	-0,85	-1,80	0,01079	AY567967	ZNF81
8167201	-0,82	-1,77	0,02025	-0,38	-1,30	0,21310	NM_007137	---
8129271	-0,82	-1,77	0,06142	-0,79	-1,73	0,00300	---	LECT2
8114263	-0,82	-1,77	0,13625	-0,84	-1,79	0,04997	NM_002302	LOC388387
8015730	-0,82	-1,77	0,00376	-1,07	-2,10	0,03450	NR_027254	TAS2R40
8136846	-0,82	-1,77	0,00108	-0,77	-1,71	0,07584	NM_176882	---
8175096	-0,82	-1,77	0,00146	-0,91	-1,88	0,00029	---	CLECL1
7961069	-0,82	-1,77	0,06104	-1,26	-2,39	0,02614	NM_172004	LOC100132167
8155503	-0,82	-1,77	0,02467	-0,61	-1,52	0,12534	BC006438	RIBC1
8167774	-0,82	-1,77	0,10922	-0,83	-1,77	0,20251	NM_001031745	FAM158A
7978114	-0,82	-1,77	0,00883	0,46	1,38	0,14690	BC002491	ACADVL
8004271	-0,82	-1,77	0,19337	1,45	2,74	0,06596	NM_000018	ZP1
7940358	-0,82	-1,77	0,25695	-0,96	-1,94	0,00151	NM_207341	---
8138600	-0,82	-1,77	0,02862	-1,25	-2,37	0,00014	---	DUOX2
7988350	-0,82	-1,77	0,03522	1,11	2,15	0,01301	NM_014080	ZNF512B
8067773	-0,82	-1,77	0,05313	0,50	1,41	0,32357	NM_020713	POLR2J
8141762	-0,82	-1,77	0,02816	-0,66	-1,59	0,20995	NM_006234	INE1
8167099	-0,82	-1,77	0,18182	-0,77	-1,70	0,00014	NR_024616	COX8C
7976402	-0,82	-1,77	0,00367	-0,82	-1,76	0,06359	NM_182971	---
8002247	-0,82	-1,77	0,10541	-0,71	-1,63	0,00409	---	SLC35F4
7979387	-0,82	-1,77	0,18250	-0,63	-1,55	0,36334	NM_001080455	---
8047485	-0,82	-1,77	0,00178	-0,57	-1,48	0,02444	---	TMPRSS11A
8100675	-0,82	-1,77	0,09186	-1,15	-2,23	0,02390	NM_182606	LOC339524
7902799	-0,82	-1,77	0,03955	-0,46	-1,38	0,04981	NR_026985	---
8175215	-0,82	-1,77	0,13210	-1,22	-2,34	0,01498	---	RAB41
8168135	-0,82	-1,77	0,08967	-1,04	-2,05	0,00690	NM_001032726	---
7911337	-0,82	-1,77	0,17274	-3,22	-9,32	0,00045	---	---
7973871	-0,82	-1,77	0,17274	-3,22	-9,32	0,00045	---	---
8165696	-0,82	-1,77	0,17274	-3,22	-9,32	0,00045	---	NECAB1
8147244	-0,82	-1,77	0,17808	-1,39	-2,62	0,01147	NM_022351	KCNH6
8009145	-0,82	-1,77	0,04936	-1,09	-2,13	0,09676	NM_030779	SMYD1
8043381	-0,82	-1,77	0,12833	-1,12	-2,18	0,00651	NM_198274	---
8151421	-0,82	-1,77	0,05170	-0,66	-1,58	0,08363	---	OR1K1
8157698	-0,82	-1,77	0,19507	-1,31	-2,49	0,00131	NM_080859	---
8136612	-0,82	-1,77	0,01191	-1,39	-2,61	0,00152	---	IL11RA

8154934	-0,82	-1,77	0,09691	-0,09	-1,06	0,61728	NM_147162	ZBTB10
8147040	-0,82	-1,77	0,06488	0,84	1,79	0,05171	NM_001105539	SEMA4A
7906107	-0,82	-1,77	0,06682	-1,58	-2,98	0,00194	NM_022367	CASS4
8063497	-0,82	-1,77	0,04738	-1,45	-2,73	0,00575	NM_001164116	TNNI1
7923360	-0,82	-1,77	0,16564	-1,74	-3,33	0,00813	NM_003281	RTN4RL1
8011214	-0,82	-1,77	0,00047	-0,80	-1,74	0,04804	NM_178568	TAF6L
7940698	-0,83	-1,77	0,14925	-0,16	-1,12	0,57042	NM_006473	HNRNPCL1
7912552	-0,83	-1,77	0,12042	-1,14	-2,20	0,15986	NM_001013631	RFX2
8033097	-0,83	-1,77	0,01894	-0,24	-1,18	0,58297	NM_000635	---
8176415	-0,83	-1,77	0,00773	-1,11	-2,17	0,00274	--- ---	
8150590	-0,83	-1,77	0,17812	-0,77	-1,70	0,00043	--- OR6J1	
7977782	-0,83	-1,77	0,06764	-1,05	-2,07	0,03692	ENST00000312039	TSPAN33
8136067	-0,83	-1,77	0,04842	1,99	3,97	0,00326	NM_178562	OR2W5
7911197	-0,83	-1,77	0,03835	-1,21	-2,31	0,00002	NM_001004698	SLC5A10
8005529	-0,83	-1,77	0,09923	-0,80	-1,75	0,01551	NM_152351	CT45A4
8175336	-0,83	-1,77	0,17920	-1,49	-2,82	0,01658	NM_001017436	TLR8
8166065	-0,83	-1,77	0,01121	-0,74	-1,67	0,07919	NM_138636	---
8145566	-0,83	-1,77	0,09245	-0,82	-1,77	0,13520	--- CDH8	
8001784	-0,83	-1,77	0,11592	-1,23	-2,34	0,08809	NM_001796	CRISP2
8126891	-0,83	-1,77	0,06447	-1,08	-2,11	0,00670	NM_003296	C1orf146
7902984	-0,83	-1,77	0,09982	-1,11	-2,17	0,00653	NM_001012425	ANXA13
8152719	-0,83	-1,77	0,01703	-0,90	-1,86	0,00920	NM_001003954	---
7963696	-0,83	-1,77	0,09418	-0,89	-1,86	0,01262	--- ---	
8103847	-0,83	-1,77	0,53692	-0,94	-1,92	0,12008	--- S100A9	
7905571	-0,83	-1,77	0,03886	-1,29	-2,45	0,00026	NM_002965	ABCA3
7998784	-0,83	-1,77	0,00020	1,15	2,22	0,05093	NM_001089	PDC
7922969	-0,83	-1,77	0,08165	-1,07	-2,10	0,01392	NM_002597	PLA2G5
7898609	-0,83	-1,77	0,11062	-1,53	-2,89	0,02529	NM_000929	CPAMD8
8035201	-0,83	-1,77	0,11268	-0,64	-1,56	0,00908	NM_015692	RGS18
7908376	-0,83	-1,77	0,00759	-1,38	-2,60	0,00002	NM_130782	SAMD3
8129482	-0,83	-1,77	0,09576	-1,15	-2,22	0,03475	NM_001017373	C10orf113
7932492	-0,83	-1,77	0,02162	-0,36	-1,28	0,03315	NM_001010896	PTGDR
7974363	-0,83	-1,78	0,00687	-1,07	-2,10	0,02716	NM_000953	ABCB11
8056583	-0,83	-1,78	0,24953	-0,68	-1,60	0,05516	NM_003742	TBC1D25
8172443	-0,83	-1,78	0,03001	-0,85	-1,80	0,08257	NM_002536	CEP164
7944113	-0,83	-1,78	0,00139	0,29	1,22	0,18178	NM_014956	CCL14-CCL15
8014349	-0,83	-1,78	0,03133	-1,92	-3,79	0,00252	NR_027921	ANKRD11
7997907	-0,83	-1,78	0,00738	-1,08	-2,12	0,02164	NM_013275	ANKRD11
8170322	-0,83	-1,78	0,00738	-1,08	-2,12	0,02164	NM_013275	C11orf34
7951695	-0,83	-1,78	0,04163	-1,51	-2,85	0,00494	NM_001145024	ILDR2
7922018	-0,83	-1,78	0,03423	-1,05	-2,07	0,01555	NM_199351	PAX6
7947338	-0,83	-1,78	0,02327	0,03	1,02	0,92410	NM_000280	MAFF
8073007	-0,83	-1,78	0,22947	-0,95	-1,93	0,02984	NM_012323	GDPD3
8000799	-0,83	-1,78	0,20418	0,62	1,54	0,25421	NM_024307	SYK
8156321	-0,83	-1,78	0,12813	0,67	1,59	0,16359	NM_003177	AHDC1
7914103	-0,83	-1,78	0,12348	-0,99	-1,98	0,08603	NM_001029882	FAM27A
8155510	-0,83	-1,78	0,41731	-2,20	-4,59	0,00243	NR_024060	SIRT6
8032770	-0,83	-1,78	0,01054	0,80	1,74	0,08563	NM_016539	NRN1
8123739	-0,83	-1,78	0,02906	1,05	2,07	0,00765	NM_016588	TRIML1
8098697	-0,83	-1,78	0,02021	-1,35	-2,55	0,00467	NM_178556	PRSS1
8136795	-0,83	-1,78	0,12126	-0,68	-1,60	0,01591	NM_002769	CD72
8161004	-0,83	-1,78	0,08006	-0,34	-1,27	0,41342	NM_001782	STK11IP
8048647	-0,83	-1,78	0,14871	-0,21	-1,16	0,68895	NM_052902	KCNMB2
8084002	-0,83	-1,78	0,10879	-1,45	-2,73	0,00895	NM_005832	FOXD4L6
8161533	-0,83	-1,78	0,21738	-1,18	-2,26	0,01053	NM_001085476	OR8H2
7939967	-0,83	-1,78	0,34595	-1,29	-2,45	0,01225	NM_001005200	---
8114203	-0,83	-1,78	0,19386	-1,41	-2,66	0,07123	--- TF	
8082797	-0,83	-1,78	0,08082	-1,37	-2,58	0,04521	NM_001063	RAB3C
8105487	-0,83	-1,78	0,09084	-0,54	-1,45	0,02045	NM_138453	PPAN-P2RY11
8025563	-0,83	-1,78	0,00181	-0,12	-1,08	0,53395	NM_001040664	KCNJ6
8070279	-0,83	-1,78	0,03579	-0,77	-1,71	0,03332	NM_002240	CD22
8027837	-0,83	-1,78	0,07896	-1,07	-2,09	0,03430	NM_001771	TRABD
8074003	-0,83	-1,78	0,28552	-0,27	-1,21	0,36708	NM_025204	---
7933310	-0,83	-1,78	0,11968	-0,48	-1,39	0,40061	--- ABCC13	
8067877	-0,83	-1,78	0,03570	-1,28	-2,43	0,00332	NR_003087	ESPL1
7955736	-0,83	-1,78	0,02563	1,28	2,43	0,02596	NM_012291	---
8099805	-0,83	-1,78	0,06198	-0,98	-1,97	0,00430	--- ATP2B3	
8170671	-0,83	-1,78	0,09976	-0,44	-1,35	0,06909	NM_021949	---
8046644	-0,83	-1,78	0,11683	-0,50	-1,41	0,05239	--- TMEM63C	
7975932	-0,83	-1,78	0,00359	1,37	2,59	0,00076	NM_020431	CNGA2
8170520	-0,83	-1,78	0,20930	-1,17	-2,25	0,00969	NM_005140	CDK18
7909104	-0,83	-1,78	0,07159	-0,11	-1,08	0,89180	NM_212503	---
7943550	-0,83	-1,78	0,00070	-0,66	-1,58	0,00662	--- DES	

8048541	-0,83	-1,78	0,03979	-1,46	-2,74	0,01001	NM_001927	C10orf122
7936918	-0,83	-1,78	0,00943	-0,43	-1,35	0,23469	NM_001128202	HTR6
7898594	-0,83	-1,78	0,15065	-1,47	-2,76	0,01740	NM_000871	SURF2
8159008	-0,83	-1,78	0,07547	0,15	1,11	0,89914	NM_017503	C21orf104
8070328	-0,83	-1,78	0,17064	-2,45	-5,46	0,00324	ENST00000442121	TMEM187
8170803	-0,83	-1,78	0,04336	0,22	1,17	0,15655	NM_003492	GPR35
8049722	-0,83	-1,78	0,10355	-1,45	-2,73	0,00274	NM_005301	SCARNA23
8166563	-0,83	-1,78	0,00374	-1,11	-2,15	0,02505	NR_003007	---
7969143	-0,83	-1,78	0,23394	-1,38	-2,61	0,02002	---	hCG_1651160
7945956	-0,83	-1,78	0,21605	-0,93	-1,90	0,01864	ENST00000421628	SSH3
7941843	-0,83	-1,78	0,24027	0,81	1,75	0,25569	NM_017857	SIX2
8051949	-0,83	-1,78	0,06202	-2,11	-4,33	0,01822	NM_016932	SNORA27
7968234	-0,83	-1,78	0,03342	-0,86	-1,82	0,01146	NR_002575	BAI3
8120468	-0,83	-1,78	0,04256	-0,85	-1,81	0,00428	NM_001704	SASH3
8169859	-0,83	-1,78	0,04981	-1,07	-2,10	0,01290	NM_018990	PRDM6
8107646	-0,83	-1,78	0,08160	-1,49	-2,82	0,03668	NM_001136239	CLSTN2
8083034	-0,83	-1,78	0,01405	-1,42	-2,67	0,01183	NM_022131	KRT222
8015049	-0,83	-1,78	0,00128	-0,82	-1,77	0,08547	NM_152349	---
8000945	-0,83	-1,78	0,01646	-0,44	-1,35	0,59504	---	KRTAP13-3
8069818	-0,83	-1,78	0,13242	-1,34	-2,53	0,04444	NM_181622	GSTTP1
8071801	-0,83	-1,78	0,22968	-1,23	-2,34	0,08800	NR_003081	LRP11
8122717	-0,83	-1,78	0,02547	-0,89	-1,85	0,11861	NM_032832	TMEM121
7977432	-0,83	-1,78	0,22201	-1,24	-2,36	0,08888	NM_025268	MIR16-2
8083739	-0,83	-1,78	0,07452	-0,80	-1,75	0,08324	NR_029525	---
7932983	-0,83	-1,78	0,11076	-0,57	-1,48	0,19326	---	OR52B4
7945958	-0,83	-1,78	0,10151	-1,46	-2,75	0,00166	NM_001005161	ACAD9
8090511	-0,83	-1,78	0,04605	-0,84	-1,78	0,17008	NM_014049	SELO
8074020	-0,83	-1,78	0,06498	0,67	1,60	0,30143	NM_031454	---
8023373	-0,83	-1,78	0,02527	-1,10	-2,15	0,06407	---	MUTYH
7915695	-0,83	-1,78	0,13426	-0,38	-1,31	0,07396	NM_012222	C9orf116
8165024	-0,83	-1,78	0,00700	-0,19	-1,14	0,76946	NM_001048265	ZKSCAN4
8124553	-0,83	-1,78	0,05454	0,67	1,59	0,05837	NM_019110	CYP3A5
8141328	-0,83	-1,78	0,13904	-0,93	-1,91	0,05231	NM_000777	TREML2
8126288	-0,84	-1,78	0,09783	-1,30	-2,46	0,01686	NM_024807	KLHL25
7991173	-0,84	-1,78	0,04688	-0,67	-1,59	0,01449	NM_022480	FLJ10213
8080980	-0,84	-1,78	0,15271	-0,87	-1,83	0,00045	NM_018029	INF2
7977241	-0,84	-1,78	0,07332	1,85	3,60	0,05667	NM_022489	RAD21L1
8060400	-0,84	-1,78	0,02105	-0,90	-1,87	0,04013	NM_001136566	---
8161852	-0,84	-1,78	0,16141	-0,42	-1,34	0,22601	---	---
8088472	-0,84	-1,78	0,05495	-1,15	-2,22	0,00144	---	---
8122194	-0,84	-1,78	0,26401	-0,19	-1,14	0,40136	---	TBC1D10C
7941879	-0,84	-1,78	0,05399	-0,94	-1,91	0,02637	NM_198517	CASP5
7951385	-0,84	-1,78	0,04160	-0,39	-1,31	0,34246	NM_004347	TTC39A
7916024	-0,84	-1,78	0,01193	1,72	3,28	0,00344	NM_001144832	---
7922293	-0,84	-1,78	0,12761	-1,62	-3,08	0,00160	---	INSIG2
8044766	-0,84	-1,78	0,14908	0,38	1,30	0,18158	NM_016133	FANCG
8160935	-0,84	-1,79	0,02233	0,89	1,85	0,02267	NM_004629	ANKRD20B
8053744	-0,84	-1,79	0,13168	-0,98	-1,97	0,07107	NR_003366	SELE
7922229	-0,84	-1,79	0,03822	-0,83	-1,78	0,04165	NM_000450	DAPK2
7989596	-0,84	-1,79	0,01859	-0,94	-1,92	0,00383	NM_014326	PSORS1C1
8118076	-0,84	-1,79	0,02164	0,66	1,58	0,19799	NM_014068	PIPSL
7935139	-0,84	-1,79	0,31876	0,22	1,17	0,04113	NR_002319	SUGT1L1
7971191	-0,84	-1,79	0,05125	-0,59	-1,50	0,29455	NR_003365	BMP7
8067185	-0,84	-1,79	0,12375	0,32	1,24	0,11857	NM_001719	SERPINA7
8174304	-0,84	-1,79	0,07950	-0,86	-1,82	0,03903	NM_000354	UBE2K
8099918	-0,84	-1,79	0,23566	-1,03	-2,04	0,04839	AK291454	LOC646508
8030974	-0,84	-1,79	0,24961	-0,99	-1,99	0,00074	ENST00000338885	TRIM69
7983365	-0,84	-1,79	0,08777	-0,44	-1,35	0,09764	NM_182985	MIR153-2
8144149	-0,84	-1,79	0,07139	-1,20	-2,30	0,02514	NR_029689	TMPRSS3
8070584	-0,84	-1,79	0,02841	-0,47	-1,39	0,03974	NM_024022	LOC338579
7927207	-0,84	-1,79	0,00568	-1,10	-2,14	0,01265	AK098688	NCRNA00052
7985752	-0,84	-1,79	0,12001	-1,90	-3,74	0,00128	NR_026869	TAC4
8016600	-0,84	-1,79	0,29090	-1,38	-2,59	0,00306	NM_170685	MAMSTR
8038150	-0,84	-1,79	0,15082	-1,83	-3,56	0,00031	NM_182574	HEPACAM
7944911	-0,84	-1,79	0,04347	-0,51	-1,42	0,02859	NM_152722	GGT1
8071927	-0,84	-1,79	0,00452	-0,98	-1,98	0,07258	NM_005265	---
8043433	-0,84	-1,79	0,39727	-2,62	-6,14	0,00000	---	RELB
8029580	-0,84	-1,79	0,15632	-0,47	-1,39	0,10238	NM_006509	---
8123338	-0,84	-1,79	0,00953	-0,98	-1,97	0,01088	---	SP8
8138474	-0,84	-1,79	0,10636	-1,47	-2,77	0,00176	NM_198956	SNORA15
8132962	-0,84	-1,79	0,22245	-1,23	-2,35	0,00705	NR_002957	C5orf52
8109572	-0,84	-1,79	0,14985	-0,80	-1,74	0,14386	NM_001145132	FAM5C
7922994	-0,84	-1,79	0,09545	-1,20	-2,30	0,00447	NM_199051	PWWP2B

7931469	-0,84	-1,79	0,04356	-0,63	-1,55	0,01154	NM_138499		---
8169156	-0,84	-1,79	0,21738	-2,16	-4,47	0,02983	---	C1orf141	
7916822	-0,84	-1,79	0,05370	-1,19	-2,28	0,01343	BC047053	GPR31	
8130785	-0,84	-1,79	0,02267	-1,81	-3,51	0,00050	NM_005299		XKR7
8061647	-0,84	-1,79	0,12115	-1,00	-1,99	0,12351	NM_001011718		LSP1
7937735	-0,84	-1,79	0,00797	-0,95	-1,93	0,03419	NM_002339		---
7978536	-0,84	-1,79	0,12628	-1,27	-2,42	0,01054	---	SLC32A1	
8062539	-0,84	-1,79	0,02058	-1,23	-2,34	0,00606	NM_080552		PREX1
8066848	-0,84	-1,79	0,03333	0,32	1,25	0,45788	NM_020820		C13orf35
7970158	-0,84	-1,79	0,12174	-1,69	-3,22	0,00100	NM_207440		---
8100095	-0,84	-1,79	0,08754	-1,05	-2,07	0,00745	---	CORO1B	
7949776	-0,84	-1,79	0,05233	0,12	1,09	0,72238	NM_020441		FLJ40852
8143448	-0,84	-1,79	0,09383	-0,78	-1,72	0,05622	NR_015392		---
8133500	-0,84	-1,79	0,09460	-1,41	-2,65	0,02135	---	KRTAP20-1	
8068154	-0,84	-1,79	0,26524	-0,79	-1,72	0,01160	NM_181615		---
7999560	-0,84	-1,79	0,00887	-1,50	-2,82	0,00986	---	KNDC1	
7931519	-0,84	-1,79	0,00776	-0,26	-1,19	0,52810	NM_152643		---
8114548	-0,84	-1,79	0,09521	-1,69	-3,24	0,05988	---	---	
8141281	-0,84	-1,79	0,06633	-0,91	-1,88	0,03200	---	---	
7926205	-0,84	-1,79	0,01733	-1,19	-2,28	0,00327	---	GFRA1	
7936494	-0,84	-1,79	0,34100	-1,35	-2,55	0,00012	NM_005264		SYCP3
7965800	-0,84	-1,79	0,04021	-1,13	-2,19	0,00386	NM_001177949		NOL6
8160682	-0,84	-1,79	0,00121	1,72	3,30	0,00729	NM_022917		---
8143205	-0,84	-1,79	0,10408	-1,31	-2,48	0,01430	---	CRB1	
7908508	-0,84	-1,79	0,00021	-0,56	-1,47	0,04782	NM_201253		---
8053404	-0,84	-1,79	0,02263	-1,82	-3,52	0,01088	---	---	
7977474	-0,84	-1,79	0,13730	-1,66	-3,15	0,02361	---	IL1RN	
8044574	-0,84	-1,79	0,13462	-1,06	-2,09	0,01023	NM_173842		GP1BB
8071274	-0,84	-1,79	0,68528	-1,58	-3,00	0,35150	L20860	SLC5A9	
7901316	-0,84	-1,79	0,13237	-1,35	-2,55	0,02730	NM_001135181		---
8092883	-0,84	-1,79	0,28888	-1,16	-2,24	0,11236	---	SIM1	
8128459	-0,84	-1,79	0,01864	-1,54	-2,91	0,00083	NM_005068		GOLGA6A
7990283	-0,84	-1,79	0,25447	-1,04	-2,05	0,00731	NM_001038640		RGS1
7908388	-0,84	-1,79	0,00543	-1,02	-2,03	0,02377	NM_002922		---
7960357	-0,84	-1,79	0,32541	0,26	1,20	0,32121	---	SNORD115-7	
7982020	-0,84	-1,79	0,06182	-1,79	-3,45	0,00267	NR_003299		NRG3
7928736	-0,84	-1,79	0,12842	-1,18	-2,26	0,00349	NM_001010848		GLYATL2
7948354	-0,84	-1,79	0,00134	-1,43	-2,70	0,01599	NM_145016		ST18
8150722	-0,84	-1,79	0,27149	-1,27	-2,41	0,00883	NM_014682		BLM
7986068	-0,84	-1,79	0,02602	2,51	5,69	0,00239	NM_000057		CD101
7904303	-0,84	-1,79	0,11199	-1,09	-2,13	0,02672	NM_004258		11. Mrz
8111127	-0,84	-1,79	0,02373	-0,17	-1,13	0,45286	NM_001102562		TTC39C
8020647	-0,84	-1,79	0,10011	0,35	1,28	0,48224	NR_024232		MPP3
8015846	-0,84	-1,79	0,03220	1,04	2,06	0,16446	NM_001932		SSX1
8167254	-0,84	-1,79	0,10686	-1,16	-2,23	0,01307	NM_005635		RNF17
7968085	-0,84	-1,79	0,00969	-0,90	-1,87	0,00007	NM_031277		---
8031615	-0,84	-1,79	0,09203	-1,65	-3,13	0,00179	---	MRGPRX2	
7947096	-0,84	-1,79	0,06543	-0,33	-1,26	0,38490	NM_054030		FUZ
8038443	-0,84	-1,79	0,14939	-0,59	-1,51	0,10925	NM_025129		ZNF674
8172274	-0,84	-1,79	0,13790	-0,32	-1,25	0,42435	NM_001039891		---
7986534	-0,84	-1,79	0,04975	-1,40	-2,64	0,04791	---	---	
8113701	-0,84	-1,79	0,14317	-0,55	-1,47	0,21235	---	MIR485	
7976846	-0,84	-1,79	0,03501	-1,23	-2,35	0,07342	NR_030160		---
8066960	-0,84	-1,79	0,13885	-0,64	-1,56	0,05660	---	IL19	
7909250	-0,84	-1,79	0,05635	-1,23	-2,35	0,00420	NM_153758		CHD5
7911941	-0,84	-1,79	0,14964	-1,02	-2,03	0,02771	NM_015557		CD274
8154233	-0,84	-1,79	0,16737	-0,03	-1,02	0,93396	NM_014143		ACTR5
8062545	-0,84	-1,79	0,01703	2,09	4,26	0,00100	NM_024855		ENKUR
7932598	-0,84	-1,79	0,16506	-1,05	-2,06	0,04282	NM_145010		AQP2
7955290	-0,84	-1,79	0,06869	-1,75	-3,37	0,02614	NM_000486		---
8122803	-0,84	-1,79	0,03239	-0,57	-1,49	0,10076	---	---	
7924325	-0,84	-1,79	0,09349	-0,75	-1,68	0,06480	---	FSIP2	
8046833	-0,84	-1,79	0,06685	-0,60	-1,51	0,24968	AK126104	CCL16	
8014342	-0,84	-1,79	0,00220	-0,40	-1,32	0,37641	NM_004590		GIP
8016523	-0,84	-1,79	0,04625	-0,95	-1,94	0,03228	NM_004123		---
7951591	-0,84	-1,79	0,11260	-0,46	-1,38	0,13237	---	LRP2	
8056611	-0,84	-1,79	0,05052	-1,19	-2,28	0,00230	NM_004525		LOC150527
8045205	-0,84	-1,79	0,06003	-1,36	-2,56	0,07776	NR_027313		LOC150527
8055236	-0,84	-1,79	0,06003	-1,36	-2,56	0,07776	NR_027313		SNORD30
7948900	-0,84	-1,79	0,13336	-0,90	-1,87	0,02265	NR_002561		---
8097790	-0,84	-1,79	0,05777	-1,01	-2,01	0,02270	---	---	
7944516	-0,84	-1,79	0,16626	-1,34	-2,53	0,07007	---	MIR128-2	
8078527	-0,84	-1,79	0,02777	-1,43	-2,69	0,05718	NR_029824		NR1I2

8081925	-0,84	-1,79	0,00123	-1,41	-2,65	0,00082	NM_003889	SLFNL1
7915351	-0,84	-1,79	0,09161	-0,71	-1,63	0,07289	NM_001168247	ADCY5
8090070	-0,84	-1,79	0,03908	-0,89	-1,86	0,12726	NM_183357	CATSPERB
7980861	-0,84	-1,79	0,08075	-0,50	-1,41	0,00118	NM_024764	LOC440563
7912580	-0,84	-1,79	0,13403	-1,04	-2,06	0,03084	NM_001136561	GOLGA6L5
7991110	-0,84	-1,79	0,46927	-0,23	-1,17	0,63235	NR_003246	CCDC115
8055208	-0,84	-1,79	0,00886	-0,79	-1,73	0,08031	NM_032357	SUMO1P3
7906574	-0,84	-1,79	0,25806	-0,44	-1,35	0,27921	NR_002190	ALDH4A1
7912975	-0,84	-1,79	0,02022	-0,30	-1,23	0,29294	NM_003748	CDC42BPG
7949227	-0,84	-1,79	0,05098	-0,67	-1,59	0,10616	NM_017525	FAM184A
8129231	-0,84	-1,79	0,03066	-0,54	-1,46	0,00467	NM_024581	---
8050236	-0,84	-1,79	0,06617	-0,87	-1,83	0,09506	---	---
8081941	-0,84	-1,79	0,15186	-0,95	-1,94	0,05836	---	MIR487A
7976840	-0,84	-1,79	0,16171	-0,65	-1,56	0,00304	NR_030162	EPB49
8145005	-0,84	-1,79	0,03565	1,09	2,13	0,03646	NM_001978	DPPA2
8089438	-0,84	-1,79	0,08804	-0,36	-1,28	0,00173	NM_138815	OTOL1
8083770	-0,84	-1,79	0,14422	-0,77	-1,71	0,15706	NM_001080440	PAR4
7982052	-0,84	-1,79	0,00649	-0,89	-1,86	0,01413	NR_022010	EME1
8008310	-0,84	-1,79	0,04395	-0,30	-1,23	0,24333	NM_001166131	C10orf79
7936201	-0,84	-1,79	0,08126	-0,30	-1,23	0,23007	NM_025145	---
8168687	-0,84	-1,79	0,14991	-0,70	-1,62	0,20896	---	PTPRT
8066347	-0,84	-1,80	0,11625	-1,25	-2,38	0,00084	NM_133170	---
7901416	-0,84	-1,80	0,02058	-1,03	-2,04	0,07873	---	---
8069444	-0,84	-1,80	0,20574	-0,86	-1,82	0,09168	---	---
7946029	-0,84	-1,80	0,11303	-0,96	-1,95	0,04369	---	---
7980477	-0,84	-1,80	0,42668	-2,03	-4,10	0,05638	---	---
8121557	-0,84	-1,80	0,04121	-1,50	-2,83	0,00556	---	NKAIN2
8121814	-0,84	-1,80	0,23108	-0,44	-1,36	0,14900	NM_001040214	LRFN4
7941743	-0,84	-1,80	0,24222	-0,67	-1,60	0,10181	NM_024036	PNLIPRP3
7930760	-0,84	-1,80	0,10171	-1,12	-2,17	0,00709	NM_001011709	RASGEF1C
8116384	-0,84	-1,80	0,00851	-0,52	-1,43	0,13762	NM_175062	ARX
8171867	-0,84	-1,80	0,03797	-1,62	-3,07	0,00439	NM_139058	AQP8
7994252	-0,84	-1,80	0,19188	-1,17	-2,25	0,07507	NM_001169	VAC14
8002444	-0,84	-1,80	0,00661	-0,51	-1,42	0,11575	AK128439	---
8168672	-0,84	-1,80	0,05921	-1,36	-2,57	0,01221	---	C11orf72
7949851	-0,84	-1,80	0,10782	-1,49	-2,80	0,00089	AK075315	NOG
8008627	-0,85	-1,80	0,01104	-1,49	-2,81	0,00324	NM_005450	---
8110585	-0,85	-1,80	0,22702	-1,64	-3,12	0,00020	---	MGC5590
7971345	-0,85	-1,80	0,03569	-0,56	-1,47	0,11838	ENST00000400419	OR2A1
8136985	-0,85	-1,80	0,17032	-0,74	-1,67	0,02717	NM_001005287	GJA5
7919340	-0,85	-1,80	0,09094	-1,35	-2,56	0,02869	NM_005266	VTCN1
7918936	-0,85	-1,80	0,00768	-1,09	-2,13	0,08825	NM_024626	---
8156450	-0,85	-1,80	0,10711	-1,28	-2,43	0,01327	---	C3orf56
8082347	-0,85	-1,80	0,08418	-1,28	-2,44	0,00051	BC137154	LOC387720
7937014	-0,85	-1,80	0,09250	-1,59	-3,00	0,07184	AK127642	GALR1
8021818	-0,85	-1,80	0,03210	-1,37	-2,59	0,03123	NM_001480	---
7901714	-0,85	-1,80	0,02313	-0,73	-1,66	0,04256	---	KRTAP23-1
8069811	-0,85	-1,80	0,20177	-1,51	-2,84	0,00570	NM_181624	HES1
8084880	-0,85	-1,80	0,06098	0,78	1,72	0,06357	NM_005524	MORC1
8089407	-0,85	-1,80	0,07120	-0,92	-1,90	0,00927	NM_014429	---
8094774	-0,85	-1,80	0,04292	-1,07	-2,10	0,02528	---	---
8096554	-0,85	-1,80	0,08425	-0,71	-1,64	0,07335	---	NCRNA00087
8175308	-0,85	-1,80	0,02259	-0,94	-1,92	0,07321	NR_024493	OR5A2
7948377	-0,85	-1,80	0,20334	-0,48	-1,39	0,08003	NM_001001954	CSPG4PY1
8176923	-0,85	-1,80	0,04802	-0,27	-1,21	0,08052	NR_001554	CSPG4PY1
8177410	-0,85	-1,80	0,04802	-0,27	-1,21	0,08052	NR_001554	USP17L2 // USP17L2
8149243	-0,85	-1,80	0,52679	-2,55	-5,86	0,00047	NM_201402 // NM_201402	AADACL4
7897955	-0,85	-1,80	0,09672	-1,76	-3,38	0,00521	NM_001013630	---
8130701	-0,85	-1,80	0,00213	-1,38	-2,60	0,00039	---	---
7943156	-0,85	-1,80	0,02611	-1,99	-3,98	0,00615	---	---
8043129	-0,85	-1,80	0,08630	-1,11	-2,16	0,00634	---	REM1
8061529	-0,85	-1,80	0,16550	-1,29	-2,44	0,03011	NM_014012	IRX6
7995674	-0,85	-1,80	0,12199	-1,42	-2,68	0,03975	NM_024335	TIMELESS
7964145	-0,85	-1,80	0,00215	2,05	4,15	0,00104	NM_003920	SLC13A1
8142628	-0,85	-1,80	0,08435	-1,17	-2,25	0,04546	NM_022444	FLJ36000
8005743	-0,85	-1,80	0,01065	-1,33	-2,52	0,00003	NR_027084	---
7996917	-0,85	-1,80	0,03542	-1,37	-2,58	0,24268	---	ZNF385B
8057418	-0,85	-1,80	0,00556	-0,21	-1,15	0,45343	NM_152520	---
8084213	-0,85	-1,80	0,04568	-1,97	-3,92	0,01206	---	---
8022801	-0,85	-1,80	0,00582	-1,06	-2,09	0,00405	---	SUSD3
8156393	-0,85	-1,80	0,03039	-0,57	-1,49	0,06136	NM_145006	---
8150868	-0,85	-1,80	0,02670	-1,28	-2,43	0,02133	---	---
8121561	-0,85	-1,80	0,00128	-0,51	-1,43	0,06672	---	MAGEB10

8166587	-0,85	-1,80	0,07122	-0,94	-1,92	0,02008	NM_182506	---
8112801	-0,85	-1,80	0,16372	-0,35	-1,28	0,03209	---	PIP
8136839	-0,85	-1,80	0,14000	-1,22	-2,32	0,01771	NM_002652	SERPINB10
8021645	-0,85	-1,80	0,00222	-0,86	-1,81	0,00245	NM_005024	COL4A5
8169294	-0,85	-1,80	0,07894	-0,64	-1,56	0,04728	NM_000495	TRIM39
8117869	-0,85	-1,80	0,00307	1,08	2,12	0,00594	NM_172016	TRIM39
8177770	-0,85	-1,80	0,00307	1,08	2,12	0,00594	NM_172016	TRIM39
8179088	-0,85	-1,80	0,00307	1,08	2,12	0,00594	NM_172016	AQP7P1
8155442	-0,85	-1,80	0,18041	-2,07	-4,19	0,00347	NR_002817	---
8113936	-0,85	-1,80	0,19101	-1,20	-2,30	0,09838	---	---
8027746	-0,85	-1,80	0,23794	0,08	1,06	0,78940	---	C11orf71
7951781	-0,85	-1,80	0,11512	0,43	1,35	0,09197	BC071695	C16orf59
7992614	-0,85	-1,80	0,15834	-0,89	-1,85	0,00628	NM_025108	---
8121140	-0,85	-1,80	0,23077	-0,90	-1,86	0,11495	---	LOC441426
8155455	-0,85	-1,80	0,52731	-3,66	-12,64	0,00021	AK126863	ACSBG1
7990683	-0,85	-1,80	0,00822	-0,85	-1,80	0,00543	NM_015162	FAM75C1 // FAM75C1
8156257	-0,85	-1,80	0,12322	-2,07	-4,21	0,00975	NM_001145124 // NM_001145124	FLJ31713
8163533	-0,85	-1,80	0,15728	-0,87	-1,82	0,08400	AK056275	---
8103559	-0,85	-1,80	0,04108	-0,74	-1,67	0,01366	---	L3MBTL2
8073379	-0,85	-1,80	0,01609	0,64	1,56	0,02652	NM_031488	---
7922684	-0,85	-1,80	0,20726	-2,66	-6,31	0,00285	---	---
8118611	-0,85	-1,80	0,26365	-1,69	-3,22	0,02677	---	LOC643406
8060792	-0,85	-1,80	0,16860	-0,59	-1,50	0,01651	NR_029405	---
8021683	-0,85	-1,80	0,00938	-0,55	-1,47	0,00175	---	PZP
7960984	-0,85	-1,80	0,04698	-0,95	-1,93	0,00061	NM_002864	SFI1
8072494	-0,85	-1,80	0,01927	-0,78	-1,72	0,04630	NM_001007467	TMPRSS6
8075865	-0,85	-1,80	0,02049	-1,56	-2,95	0,00019	NM_153609	LOC100128922
7915951	-0,85	-1,80	0,01721	-0,81	-1,75	0,00293	AF251047	RASGRF1
7985233	-0,85	-1,80	0,05973	-0,72	-1,65	0,12403	NM_002891	---
8150195	-0,85	-1,80	0,03231	-1,51	-2,85	0,00720	---	BTNL8
8110606	-0,85	-1,80	0,03300	-1,55	-2,93	0,00021	NM_024850	HIST1H2AG
8117535	-0,85	-1,80	0,01244	0,83	1,78	0,22019	NM_021064	SLC26A1
8098848	-0,85	-1,80	0,11470	-1,41	-2,66	0,02655	NM_213613	---
7970622	-0,85	-1,80	0,00195	-1,29	-2,45	0,00049	---	---
7971996	-0,85	-1,80	0,25262	-1,28	-2,43	0,03636	---	KIAA1409
7976350	-0,85	-1,80	0,00301	-1,01	-2,02	0,02473	NM_020818	ANKRD54
8075971	-0,85	-1,80	0,11602	-0,12	-1,08	0,84535	NM_138797	UPK3B
8133741	-0,85	-1,80	0,14057	-0,39	-1,31	0,51396	NM_030570	---
8128888	-0,85	-1,80	0,12463	-1,19	-2,28	0,00479	---	INPP4B
8097568	-0,85	-1,80	0,00850	-1,27	-2,42	0,00002	NM_003866	---
8171491	-0,85	-1,80	0,05822	-1,65	-3,14	0,02038	---	TRPC7
8114273	-0,85	-1,80	0,24229	-1,24	-2,36	0,00279	NM_020389	CELA3A
7898725	-0,85	-1,80	0,04228	-1,64	-3,12	0,04845	NM_005747	TTR
8020795	-0,85	-1,80	0,22575	-1,62	-3,07	0,00081	NM_000371	IL2RB
8075886	-0,85	-1,80	0,10384	-0,13	-1,09	0,78574	NM_000878	MAP6D1
8092404	-0,85	-1,80	0,05670	-0,85	-1,80	0,03452	NM_024871	---
8083939	-0,85	-1,80	0,01071	-0,85	-1,80	0,00971	---	C1QC
7898799	-0,85	-1,80	0,02996	-2,16	-4,46	0,00020	NM_001114101	ZBED3
8112740	-0,85	-1,80	0,11775	-0,11	-1,08	0,85658	NM_032367	---
7944970	-0,85	-1,80	0,50361	-0,78	-1,72	0,11822	---	ABHD4
7977780	-0,85	-1,80	0,46186	-1,16	-2,23	0,02982	NM_022060	---
7908730	-0,85	-1,80	0,20772	-1,10	-2,14	0,00031	---	PRF1
7934161	-0,85	-1,81	0,11087	-2,10	-4,29	0,01016	NM_005041	PRKAG3
8058997	-0,85	-1,81	0,08283	-0,79	-1,73	0,08141	NM_017431	---
8053325	-0,85	-1,81	0,22826	3,26	9,56	0,00050	---	---
7990031	-0,85	-1,81	0,05327	-1,04	-2,05	0,00994	---	CPB1
8083246	-0,85	-1,81	0,19058	-0,72	-1,65	0,00436	NM_001871	SCNN1D
7896882	-0,85	-1,81	0,05453	-1,00	-2,00	0,03021	NM_001130413	---
8171533	-0,85	-1,81	0,03364	-1,09	-2,13	0,00206	---	DEFB136
8149347	-0,85	-1,81	0,02516	-1,58	-2,99	0,00403	NM_001033018	C10orf99
7928766	-0,85	-1,81	0,11094	-1,41	-2,66	0,05408	NM_207373	OR10A5
7938172	-0,85	-1,81	0,19952	-0,94	-1,93	0,03404	NM_178168	STAC3
7964400	-0,85	-1,81	0,02034	-0,14	-1,10	0,59770	NM_145064	---
8058460	-0,85	-1,81	0,06996	-1,76	-3,38	0,00070	---	MBOAT4
8150099	-0,85	-1,81	0,03598	-0,48	-1,39	0,09563	NM_001100916	RNASE11
7977598	-0,85	-1,81	0,02447	-0,50	-1,41	0,00277	NM_145250	---
8014071	-0,85	-1,81	0,02726	-1,67	-3,18	0,02118	---	MUC3B
8135015	-0,85	-1,81	0,17641	-0,68	-1,60	0,16514	AB038783	IL17D
7967969	-0,85	-1,81	0,24196	-0,83	-1,78	0,02608	NM_138284	GPR97
7996100	-0,85	-1,81	0,02039	-1,54	-2,91	0,00463	NM_170776	CCND1
7950012	-0,85	-1,81	0,02655	-0,58	-1,50	0,07777	NM_053056	GAGE12C

8167482	-0,85	-1,81	0,45675	-1,95	-3,86	0,01458	NM_001098408	GAGE12C
8167562	-0,85	-1,81	0,45675	-1,95	-3,86	0,01458	NM_001098408	RELL2
8108822	-0,85	-1,81	0,12127	-0,78	-1,72	0,01094	NM_173828	SNAP25
8060963	-0,85	-1,81	0,00724	0,20	1,15	0,67073	NM_003081	SLC1A7
7916248	-0,85	-1,81	0,02405	-0,35	-1,27	0,11038	NM_006671	DEFB115
8061504	-0,85	-1,81	0,04535	-1,17	-2,25	0,00929	NM_001037730	---
7912527	-0,85	-1,81	0,23398	-0,59	-1,50	0,12200	---	---
8111269	-0,85	-1,81	0,06437	-1,18	-2,26	0,01810	---	CSRNP3
8046048	-0,85	-1,81	0,03344	-0,40	-1,32	0,03948	NM_001172173	DCX
8174543	-0,85	-1,81	0,01120	-0,74	-1,67	0,00031	NM_000555	TCL1A
7981183	-0,85	-1,81	0,00142	-1,65	-3,14	0,01026	NM_021966	---
8048978	-0,85	-1,81	0,00879	-0,72	-1,65	0,03865	---	SLC30A3
8051050	-0,85	-1,81	0,22622	-0,45	-1,37	0,45429	NM_003459	FLJ37453
7912672	-0,85	-1,81	0,15637	-0,85	-1,80	0,09667	NR_024279	FKSG73
8071058	-0,85	-1,81	0,04568	-1,26	-2,39	0,00082	NR_027714	B3GNT7
8049075	-0,85	-1,81	0,49429	-0,97	-1,96	0,30067	NM_145236	---
7964627	-0,86	-1,81	0,00884	-0,80	-1,75	0,00041	---	OIP5
7987636	-0,86	-1,81	0,03854	2,05	4,14	0,00134	NM_007280	SNORA14B
7925182	-0,86	-1,81	0,04565	-0,32	-1,25	0,05668	NR_002956	LOC100130924
8077877	-0,86	-1,81	0,01155	-1,74	-3,33	0,01747	AK094424	MIR125B2
8067946	-0,86	-1,81	0,09125	-1,42	-2,68	0,01242	NR_029694	---
7952781	-0,86	-1,81	0,06532	-1,14	-2,21	0,00839	---	ACAD8
7945283	-0,86	-1,81	0,17112	1,60	3,02	0,08263	NM_014384	NEBL
7932453	-0,86	-1,81	0,02204	0,67	1,59	0,04833	NM_006393	KRTAP13-4
8068136	-0,86	-1,81	0,15990	-0,89	-1,86	0,12806	NM_181600	OR13C4
8162929	-0,86	-1,81	0,19501	-0,64	-1,55	0,07033	NM_001001919	IL17A
8120210	-0,86	-1,81	0,05220	-1,45	-2,72	0,00821	NM_002190	RPS6KA5
7980744	-0,86	-1,81	0,12118	2,62	6,16	0,00268	NM_004755	---
8145687	-0,86	-1,81	0,00180	-1,42	-2,68	0,00261	---	C6orf15
8124859	-0,86	-1,81	0,17065	-1,38	-2,60	0,00627	NM_014070	GPBAR1
8048249	-0,86	-1,81	0,07271	-0,61	-1,53	0,08914	NM_001077191	ZNF598
7998679	-0,86	-1,81	0,03143	-0,61	-1,52	0,28361	NM_178167	SPATA12
8080640	-0,86	-1,81	0,21749	-0,07	-1,05	0,82812	NM_181727	C1QTNF7
8094184	-0,86	-1,81	0,18996	-1,67	-3,18	0,00701	NM_001135170	FAM36A
7911085	-0,86	-1,81	0,12944	-1,28	-2,43	0,08382	NM_198076	POM121L2
8124495	-0,86	-1,81	0,09676	-1,04	-2,05	0,03833	NM_033482	ING1
7970096	-0,86	-1,81	0,05280	-0,20	-1,15	0,64577	NM_005537	CNPY1
8144072	-0,86	-1,81	0,11724	-0,96	-1,95	0,09403	NM_001103176	DCDC5
7947282	-0,86	-1,81	0,03598	-0,93	-1,90	0,00867	AK128035	RIPK4
8070489	-0,86	-1,81	0,10635	0,63	1,55	0,03619	NM_020639	CCDC74A
8055173	-0,86	-1,81	0,03170	-0,72	-1,64	0,01689	NM_138770	LOC100130507
7952882	-0,86	-1,81	0,13312	-0,90	-1,86	0,01475	ENST00000442954	C13orf29
7972826	-0,86	-1,81	0,06383	-0,78	-1,72	0,13867	NR_027701	RIC3
7946340	-0,86	-1,81	0,02174	-0,96	-1,95	0,02395	NM_024557	---
8133872	-0,86	-1,81	0,16182	-1,14	-2,21	0,00208	---	CPNE5
8126035	-0,86	-1,81	0,10994	-0,63	-1,54	0,00908	NM_020939	FTHL17
8171917	-0,86	-1,81	0,11811	-2,18	-4,54	0,00103	NM_031894	ZNF180
8037474	-0,86	-1,81	0,09208	1,65	3,15	0,00082	NM_013256	PSMA8
8020691	-0,86	-1,81	0,02293	-0,83	-1,77	0,03528	NM_144662	HSPB9
8007208	-0,86	-1,81	0,11768	-1,39	-2,63	0,01360	NM_033194	---
8085912	-0,86	-1,81	0,17292	-1,06	-2,08	0,00757	---	---
7947915	-0,86	-1,81	0,02073	-1,32	-2,50	0,04096	---	---
7946561	-0,86	-1,81	0,25313	-0,96	-1,95	0,06350	---	TFDP3
8175209	-0,86	-1,81	0,08509	-0,11	-1,08	0,24806	NM_016521	CLDN7
8012126	-0,86	-1,81	0,01843	4,80	27,92	0,00016	NM_001307	LOC100131541
7951140	-0,86	-1,81	0,21283	-2,32	-4,98	0,00256	AY358248	C9orf31
8163892	-0,86	-1,81	0,04435	-1,14	-2,21	0,01714	AF220263	OR14A2
7925730	-0,86	-1,81	0,07460	-1,00	-2,00	0,02489	ENST00000366485	USP50
7988705	-0,86	-1,81	0,01787	-0,80	-1,74	0,00838	NM_203494	---
7953977	-0,86	-1,81	0,22640	-1,06	-2,09	0,00713	---	TRIM48
7939912	-0,86	-1,81	0,04646	-1,60	-3,03	0,00050	NM_024114	ZNF670
7925672	-0,86	-1,81	0,02079	0,98	1,97	0,05039	NM_033213	---
8129067	-0,86	-1,81	0,06467	-0,94	-1,92	0,01037	---	RNU5D // RNU5D
7915592	-0,86	-1,81	0,00596	-0,78	-1,71	0,00764	NR_002755 // NR_002755	TAF8
8119503	-0,86	-1,81	0,00197	0,67	1,60	0,04336	NM_138572	SNORD45A
7902398	-0,86	-1,81	0,18882	0,19	1,14	0,57822	NR_002749	DLX5
8141140	-0,86	-1,81	0,00022	-1,65	-3,13	0,00104	NM_005221	---
8108470	-0,86	-1,81	0,24471	-1,91	-3,75	0,01592	---	---
8133858	-0,86	-1,81	0,00106	-1,39	-2,62	0,00121	---	OR5AU1
7977655	-0,86	-1,81	0,11554	-1,77	-3,41	0,00018	NM_001004731	PLA2G4D
7987792	-0,86	-1,81	0,16821	-1,36	-2,57	0,00145	NM_178034	ADAM3A
8150375	-0,86	-1,81	0,08366	-0,86	-1,81	0,00065	NR_024107	OR4Q3
7973004	-0,86	-1,81	0,34368	-1,16	-2,24	0,04994	NM_172194	AGER

8179967	-0,86	-1,81	0,19676	-0,22	-1,16	0,44754	NM_001136	ZNF668
8000974	-0,86	-1,81	0,03917	-1,32	-2,50	0,03392	NM_024706	GPR98
8106827	-0,86	-1,81	0,01802	-0,87	-1,82	0,00005	NM_032119	GPR18
7972548	-0,86	-1,81	0,02170	-1,54	-2,92	0,01122	NM_005292	ACY3
7949873	-0,86	-1,81	0,05524	-0,95	-1,93	0,01568	NM_080658	FAM156A
8172813	-0,86	-1,81	0,06290	1,40	2,65	0,00212	NM_014138	CNTN5
7943319	-0,86	-1,81	0,07556	-0,97	-1,95	0,00019	NM_014361	VCY
8176650	-0,86	-1,81	0,19522	-1,46	-2,74	0,05838	NM_004679	VCY
8177169	-0,86	-1,81	0,19522	-1,46	-2,74	0,05838	NM_004679	KLHL14
8022817	-0,86	-1,81	0,02911	-1,68	-3,20	0,00807	NM_020805	OR11G2
7973028	-0,86	-1,81	0,20792	-1,07	-2,11	0,03385	NM_001005503	WDFY4
7927405	-0,86	-1,81	0,08513	-1,19	-2,28	0,00021	NM_020945	OR2T1
7911278	-0,86	-1,81	0,09902	-2,01	-4,04	0,00210	NM_030904	CDKL2
8101031	-0,86	-1,82	0,00484	0,08	1,05	0,77691	NM_003948	TGM1
7978222	-0,86	-1,82	0,03935	-0,95	-1,93	0,00282	NM_000359	F13B
7923073	-0,86	-1,82	0,03039	-1,21	-2,31	0,00786	NM_001994	CPN2
8092836	-0,86	-1,82	0,17150	-2,00	-4,01	0,00023	NM_001080513	MNDA
7906377	-0,86	-1,82	0,04897	-0,98	-1,97	0,05836	NM_002432	LOC120824
7948048	-0,86	-1,82	0,08295	-1,31	-2,48	0,00080	ENST00000437540	FANCE
8118963	-0,86	-1,82	0,26136	1,83	3,56	0,00422	NM_021922	OR5L1
7939944	-0,86	-1,82	0,06224	-0,66	-1,58	0,01320	NM_001004738	VSIG4
8173287	-0,86	-1,82	0,00280	-0,33	-1,26	0,07859	NM_007268	RNASE9
7977596	-0,86	-1,82	0,01232	-1,61	-3,04	0,00637	NM_001110359	LYNX1
8153346	-0,86	-1,82	0,00812	-1,37	-2,58	0,00033	NM_023946	---
8022277	-0,86	-1,82	0,01414	-2,41	-5,31	0,00180	---	ATAD5
8006187	-0,86	-1,82	0,01236	0,19	1,14	0,17358	NM_024857	DGKK
8172670	-0,86	-1,82	0,03599	-0,74	-1,68	0,00547	NM_001013742	SNORD45B
7902400	-0,86	-1,82	0,01084	-0,87	-1,83	0,00393	NR_002748	SNORD31
7948898	-0,86	-1,82	0,11916	-0,32	-1,25	0,14882	NR_002560	---
7905742	-0,86	-1,82	0,13995	-0,65	-1,57	0,00122	---	ARGFX
8081989	-0,86	-1,82	0,04518	-0,83	-1,78	0,01543	NM_001012659	PRY
8176806	-0,86	-1,82	0,09886	-1,43	-2,69	0,00569	NM_004676	CA5BP
8166179	-0,86	-1,82	0,22042	-0,59	-1,51	0,37581	NR_026551	---
7909780	-0,86	-1,82	0,00198	-1,02	-2,03	0,01976	---	EPHX3
8034974	-0,86	-1,82	0,08939	0,91	1,87	0,12512	NM_024794	---
8146896	-0,86	-1,82	0,52679	-3,00	-8,02	0,00403	---	LOC390282
7960434	-0,86	-1,82	0,01425	-0,50	-1,41	0,02182	ENST00000439470	LRG1
8032834	-0,86	-1,82	0,03275	-0,90	-1,86	0,07016	NM_052972	PCDH12
8114780	-0,86	-1,82	0,11061	-1,29	-2,44	0,00015	NM_016580	C13orf26
7968361	-0,86	-1,82	0,03374	-0,76	-1,70	0,01005	BC030277	POM121L8P
8071170	-0,86	-1,82	0,30642	-1,36	-2,57	0,00170	NR_024583	---
8022994	-0,86	-1,82	0,19660	-0,42	-1,34	0,09573	---	KEL
8143534	-0,86	-1,82	0,04861	-1,10	-2,15	0,00733	NM_000420	PURG
8150143	-0,86	-1,82	0,05584	-0,67	-1,59	0,03730	NM_001015508	HEATR7A
8148783	-0,86	-1,82	0,00668	1,55	2,92	0,10145	NM_032450	BMP10
8052747	-0,86	-1,82	0,24879	-1,12	-2,18	0,00533	NM_014482	OR4F21
8148962	-0,86	-1,82	0,64955	-2,39	-5,23	0,00999	NM_001005504	C8G
8159491	-0,86	-1,82	0,09079	-1,41	-2,66	0,06479	NM_000606	OR3A2
8011331	-0,86	-1,82	0,19036	-1,57	-2,97	0,06633	NM_002551	ORC6L
7995354	-0,86	-1,82	0,02368	-0,30	-1,23	0,44262	NM_014321	MIR135A1
8087883	-0,86	-1,82	0,00717	-0,77	-1,70	0,00111	NR_029677	---
8119799	-0,86	-1,82	0,13808	-0,86	-1,82	0,00436	---	USP17L2 // USP17L2
8144395	-0,86	-1,82	0,53494	-2,46	-5,51	0,00115	NM_201402 // NM_201402	OR4N4
7981783	-0,86	-1,82	0,04358	-1,14	-2,21	0,36423	NM_001005241	LOC100287948
7907907	-0,86	-1,82	0,01653	-1,15	-2,22	0,00216	AF387616	LOC440896
8161526	-0,86	-1,82	0,06702	-1,78	-3,44	0,00301	AK127288	GPR174
8168531	-0,86	-1,82	0,15113	-0,99	-1,98	0,01048	NM_032553	SNORD116-28
7982004	-0,86	-1,82	0,07138	-0,67	-1,59	0,09998	NR_003361	SLC25A41
8033179	-0,86	-1,82	0,17015	-0,93	-1,90	0,09722	NM_173637	---
7985553	-0,86	-1,82	0,00748	-0,93	-1,90	0,06805	---	ABCG1
8068761	-0,86	-1,82	0,11417	-0,04	-1,02	0,82460	NM_207627	SNORD115-38
7982082	-0,86	-1,82	0,00660	-0,91	-1,88	0,00039	NR_003353	SI
8091811	-0,86	-1,82	0,10483	-1,07	-2,10	0,00135	NM_001041	USP51
8173174	-0,86	-1,82	0,00103	-1,35	-2,55	0,01921	NM_201286	NSUN7
8094759	-0,86	-1,82	0,05888	1,43	2,70	0,00092	NM_024677	LOC100131726
8152642	-0,86	-1,82	0,20466	-0,36	-1,28	0,32154	NR_024479	MEPE
8096290	-0,86	-1,82	0,05753	-0,92	-1,89	0,00105	NM_020203	MC2R
8022412	-0,86	-1,82	0,08530	-1,22	-2,34	0,03745	NM_000529	PTPRN
8059186	-0,86	-1,82	0,18176	-1,37	-2,58	0,00276	NM_002846	---
7922821	-0,86	-1,82	0,02730	-1,81	-3,50	0,00168	---	ABLIM2
8099279	-0,86	-1,82	0,25531	-0,58	-1,50	0,00139	NM_001130083	KRTAP15-1
8068139	-0,86	-1,82	0,00679	-0,98	-1,97	0,02020	NM_181623	ZBTB22
8178988	-0,86	-1,82	0,04578	-0,05	-1,03	0,79047	NM_005453	GAGE12J

8167526	-0,86	-1,82	0,40438	-1,92	-3,78	0,00884	NM_001098406		FAM75A7
8155349	-0,86	-1,82	0,11961	-1,53	-2,90	0,00346	NM_015667		PDIA2
7991815	-0,86	-1,82	0,01309	-0,55	-1,46	0,38180	NM_006849		RORC
7920082	-0,86	-1,82	0,03212	-1,06	-2,09	0,01953	NM_005060		MDS2
7898869	-0,86	-1,82	0,29168	-1,92	-3,78	0,01392	ENST00000374555		---
8047036	-0,86	-1,82	0,00818	-1,45	-2,73	0,00020	---	---	
8083745	-0,87	-1,82	0,00537	-0,86	-1,82	0,04144	---	---	
8045334	-0,87	-1,82	0,17444	-0,83	-1,78	0,17652	---	---	
7961418	-0,87	-1,82	0,20445	-3,41	-10,64	0,00002	---	KCNG1	
8067033	-0,87	-1,82	0,01211	-0,68	-1,60	0,12829	NM_002237		C14orf148
7980390	-0,87	-1,82	0,04214	0,08	1,06	0,55022	NM_001113475		ZBTB25
7979611	-0,87	-1,82	0,08827	0,68	1,60	0,21568	NM_006977		AMPD1
7918794	-0,87	-1,82	0,00015	-0,70	-1,63	0,03683	NM_000036		RSPO2
8152314	-0,87	-1,82	0,02452	-1,32	-2,49	0,00669	NM_178565		UPK1A
8027938	-0,87	-1,82	0,00232	1,04	2,06	0,06593	NM_007000		---
8134077	-0,87	-1,82	0,02328	-1,14	-2,21	0,00000	---	---	
8173308	-0,87	-1,82	0,02177	-1,35	-2,55	0,01045	---	---	
7934977	-0,87	-1,82	0,06741	-1,42	-2,67	0,00816	---	CCDC107	
8155073	-0,87	-1,82	0,00605	-1,19	-2,28	0,00094	NM_174923		---
7908157	-0,87	-1,82	0,15318	-0,90	-1,86	0,07635	---	---	
7933840	-0,87	-1,82	0,03283	-1,07	-2,10	0,00002	---	---	
8110216	-0,87	-1,82	0,20482	-1,80	-3,48	0,00054	---	VPS37C	
7948504	-0,87	-1,82	0,01180	0,76	1,69	0,02467	NM_017966		---
7979982	-0,87	-1,82	0,07693	-0,06	-1,04	0,83362	---	---	
8106960	-0,87	-1,82	0,09017	-1,13	-2,19	0,01899	---	EEF1A2	
8067652	-0,87	-1,82	0,02635	1,03	2,05	0,02579	NM_001958		CT45A6
8175353	-0,87	-1,82	0,15983	-1,60	-3,04	0,00005	NM_001017438		OSTBETA
7984245	-0,87	-1,82	0,02014	-1,17	-2,25	0,01242	NM_178859		MAP4K2
7949172	-0,87	-1,82	0,04525	1,15	2,22	0,11598	NM_004579		---
8041223	-0,87	-1,82	0,01664	-0,77	-1,70	0,00168	---	ZBTB12	
8125201	-0,87	-1,82	0,06523	-1,27	-2,41	0,02247	NM_181842		ZBTB12
8178683	-0,87	-1,82	0,06523	-1,27	-2,41	0,02247	NM_181842		IL22
7964803	-0,87	-1,82	0,02669	-1,32	-2,50	0,01003	NM_020525		---
8088546	-0,87	-1,82	0,01089	-0,90	-1,86	0,01645	---	---	
8145887	-0,87	-1,82	0,14300	-0,94	-1,92	0,00695	---	ADAM2	
8150395	-0,87	-1,82	0,00780	-0,60	-1,51	0,00082	NM_001464		TRIM11
7924868	-0,87	-1,82	0,04036	0,06	1,05	0,87475	NM_145214		MIR194-1
7924403	-0,87	-1,82	0,03702	-0,88	-1,84	0,07930	NR_029711		PLAC2
8032965	-0,87	-1,82	0,24510	-1,34	-2,52	0,06276	BC036545	FAM75A7	
8161341	-0,87	-1,82	0,09507	-1,57	-2,97	0,00864	NM_015667		---
8120466	-0,87	-1,82	0,01519	-1,16	-2,23	0,00014	---	SARS2	
8036656	-0,87	-1,82	0,12009	0,83	1,78	0,12498	NM_017827		DLEC1
8078690	-0,87	-1,82	0,01345	-0,77	-1,71	0,00331	NM_007335		---
8077511	-0,87	-1,82	0,01511	-0,74	-1,67	0,14311	---	---	
8014197	-0,87	-1,82	0,05428	-1,18	-2,27	0,01279	---	---	
8136469	-0,87	-1,82	0,02321	-1,40	-2,63	0,00001	---	---	
8042976	-0,87	-1,82	0,00292	-0,71	-1,63	0,00025	---	PRTG	
7989073	-0,87	-1,83	0,00728	3,40	10,56	0,00084	NM_173814		---
8017102	-0,87	-1,83	0,03121	-1,33	-2,52	0,00038	---	UNC45B	
8006509	-0,87	-1,83	0,00149	-0,56	-1,47	0,05779	NM_173167		---
8083996	-0,87	-1,83	0,01289	-1,06	-2,08	0,00034	---	MBD3L5	
8025126	-0,87	-1,83	0,22632	-1,84	-3,59	0,01328	NM_001136507		---
7978690	-0,87	-1,83	0,00681	-1,08	-2,12	0,03853	---	---	
8166182	-0,87	-1,83	0,05735	-0,91	-1,88	0,01957	---	---	
7955417	-0,87	-1,83	0,06093	-0,57	-1,48	0,00138	---	GP1BB	
8071272	-0,87	-1,83	0,49518	-0,75	-1,68	0,00653	L20860	---	
8158993	-0,87	-1,83	0,06676	-1,52	-2,87	0,00425	---	NMRAL1	
7999149	-0,87	-1,83	0,03796	0,11	1,08	0,84842	NM_020677		HEPH
8167973	-0,87	-1,83	0,04957	-0,76	-1,69	0,03051	NM_138737		POU3F1
7915156	-0,87	-1,83	0,22908	-1,93	-3,80	0,01462	NM_002699		SNORD28
7948904	-0,87	-1,83	0,01361	1,54	2,92	0,13373	NR_002562		LRRIQ4
8083808	-0,87	-1,83	0,01102	-0,73	-1,65	0,00059	NM_001080460		NAG18
8031840	-0,87	-1,83	0,12955	-1,22	-2,33	0,01466	AF210651	---	
8146401	-0,87	-1,83	0,07481	-0,97	-1,96	0,00062	---	OLIG3	
8129872	-0,87	-1,83	0,13459	-1,70	-3,24	0,00525	NM_175747		NKAPL
8117663	-0,87	-1,83	0,11841	-1,15	-2,22	0,01645	NM_001007531		---
8052945	-0,87	-1,83	0,03227	-1,98	-3,94	0,00000	---	DSC2	
8022711	-0,87	-1,83	0,06422	3,24	9,42	0,00045	NM_024422		SLC12A5
8063129	-0,87	-1,83	0,14884	-0,73	-1,66	0,00671	NM_020708		COL29A1
8082585	-0,87	-1,83	0,00234	-1,26	-2,40	0,00111	NR_022012		---
7939922	-0,87	-1,83	0,17005	-2,39	-5,24	0,00049	---	PPP1R2P9	
8172197	-0,87	-1,83	0,16254	-1,90	-3,74	0,04255	NR_002191		C9orf144
8160900	-0,87	-1,83	0,10043	-0,67	-1,59	0,16400	NR_024481		EVX1

8131970	-0,87	-1,83	0,00614	-1,12	-2,17	0,00146	NM_001989	---
8094355	-0,87	-1,83	0,00582	-0,80	-1,74	0,08960	---	TACR3
8102127	-0,87	-1,83	0,07826	-0,80	-1,74	0,05044	NM_001059	AGXT2L1
8102249	-0,87	-1,83	0,03235	-0,63	-1,55	0,02696	NM_031279	RASEF
8161945	-0,87	-1,83	0,12162	1,21	2,31	0,00956	NM_152573	CPLX3
7984908	-0,87	-1,83	0,05567	-1,73	-3,32	0,08925	NM_001030005	KCNT1
8159283	-0,87	-1,83	0,05089	-0,86	-1,81	0,02645	NM_020822	---
7944033	-0,87	-1,83	0,00133	-1,28	-2,42	0,00570	---	C14orf38
7979444	-0,87	-1,83	0,00232	-1,01	-2,01	0,02422	NM_001164399	HLA-DRB3
8180003	-0,87	-1,83	0,10254	0,66	1,58	0,03977	NM_022555	RAPGEF5
8138504	-0,87	-1,83	0,07096	0,85	1,81	0,14027	NM_012294	SIRT2
8036636	-0,87	-1,83	0,09409	0,24	1,18	0,60514	NM_012237	---
8060609	-0,87	-1,83	0,28250	-1,12	-2,17	0,10847	---	CBLN2
8023822	-0,87	-1,83	0,03503	-1,08	-2,11	0,00479	NM_182511	---
8007902	-0,87	-1,83	0,03852	-0,95	-1,94	0,05092	---	C4orf35
8095484	-0,87	-1,83	0,02421	-1,04	-2,06	0,03069	NM_033122	---
8088636	-0,87	-1,83	0,07893	-0,73	-1,66	0,07829	---	SEZ6L
8072088	-0,87	-1,83	0,09385	-1,22	-2,32	0,05858	NM_021115	OPRM1
8122887	-0,87	-1,83	0,01788	-0,82	-1,77	0,00002	NM_000914	A2BP1
7993083	-0,87	-1,83	0,05008	-1,24	-2,36	0,00018	NM_018723	C1orf194
7918294	-0,87	-1,83	0,13716	-0,98	-1,98	0,02854	NM_001122961	C12orf68
7955107	-0,87	-1,83	0,00804	-0,18	-1,14	0,11088	NM_001013635	HMGB4
7899905	-0,87	-1,83	0,14778	-0,90	-1,86	0,02328	NM_145205	C15orf34
7984631	-0,87	-1,83	0,05849	-1,48	-2,80	0,00042	NR_027262	SLC24A4
7976263	-0,87	-1,83	0,00352	-0,60	-1,52	0,05703	NM_153646	ADRA2A
7930482	-0,87	-1,83	0,06849	-2,54	-5,81	0,00026	NM_000681	TFAP2B
8120194	-0,87	-1,83	0,03562	-2,02	-4,05	0,00109	NM_003221	OR52E5
7938063	-0,87	-1,83	0,16672	-1,27	-2,42	0,05169	ENST00000316698	FLJ44674
7995440	-0,87	-1,83	0,03925	-1,60	-3,03	0,00286	AK128747	---
7899073	-0,87	-1,83	0,08756	-0,89	-1,85	0,09003	---	---
8082118	-0,87	-1,83	0,05993	-1,75	-3,35	0,03814	---	CACNA1A
8034643	-0,87	-1,83	0,04727	-1,03	-2,04	0,00667	NM_000068	---
8161904	-0,87	-1,83	0,04536	-1,06	-2,08	0,05255	---	VSIG7
7986561	-0,87	-1,83	0,06070	-2,01	-4,02	0,00639	ENST00000338912	HLA-DRA
8118548	-0,87	-1,83	0,04886	0,96	1,94	0,09159	NM_019111	AKAP4
8172660	-0,87	-1,83	0,02988	-1,01	-2,01	0,01784	NM_003886	IFNA1
8154627	-0,87	-1,83	0,05818	-1,47	-2,77	0,03807	NM_024013	STAB1
8080344	-0,87	-1,83	0,05568	-1,24	-2,36	0,00049	NM_015136	AGER
8178771	-0,87	-1,83	0,20631	-0,22	-1,17	0,49746	NM_001136	OR6C74
7955985	-0,87	-1,83	0,02044	-1,21	-2,31	0,04539	NM_001005490	KIF5A
7956488	-0,87	-1,83	0,02750	-0,41	-1,33	0,06825	NM_004984	---
8114285	-0,87	-1,83	0,08977	-1,10	-2,14	0,02163	---	GKN1
8042431	-0,87	-1,83	0,00518	-0,50	-1,41	0,11373	NM_019617	C15orf43
7983381	-0,87	-1,83	0,05153	-0,42	-1,34	0,06510	BC029537	---
7902365	-0,87	-1,83	0,03993	-1,04	-2,05	0,17116	---	---
8046012	-0,87	-1,83	0,03350	-1,09	-2,14	0,01581	---	KLF15
8090343	-0,87	-1,83	0,07201	-0,68	-1,60	0,26266	NM_014079	FNDC8
8006504	-0,87	-1,83	0,16911	-1,85	-3,60	0,00044	NM_017559	---
8035682	-0,87	-1,83	0,04998	-1,00	-1,99	0,03124	---	NYX
8166899	-0,87	-1,83	0,16577	-1,15	-2,23	0,01884	NM_022567	---
7975630	-0,87	-1,83	0,02806	-0,82	-1,77	0,00037	---	MOBP
8078920	-0,87	-1,83	0,13503	-1,52	-2,87	0,00031	NR_003090	GLI1
7956430	-0,87	-1,83	0,02401	-1,31	-2,47	0,06261	NM_005269	SEC14L5
7993054	-0,87	-1,83	0,07991	-1,31	-2,47	0,02289	NM_014692	PLXNB3
8170716	-0,87	-1,83	0,01015	-0,86	-1,81	0,02523	NM_005393	C8orf17
8148463	-0,87	-1,83	0,01800	-1,60	-3,03	0,01354	AF220264	LOC100287671
8105189	-0,87	-1,83	0,01452	-1,96	-3,89	0,00799	BC137195	KAAG1
8117189	-0,87	-1,83	0,01078	-1,25	-2,38	0,11357	NM_181337	ZDHHC23
8081779	-0,87	-1,83	0,11344	1,44	2,72	0,08874	NM_173570	ANKRD30B
8069499	-0,87	-1,83	0,00687	-0,96	-1,94	0,00425	NM_001145029	LRFN1
8036707	-0,87	-1,83	0,21400	-1,18	-2,27	0,02969	NM_020862	---
8157103	-0,87	-1,83	0,02247	-1,13	-2,18	0,01915	---	---
8134316	-0,87	-1,83	0,01687	-0,52	-1,43	0,01810	---	---
8018906	-0,87	-1,83	0,22238	-0,10	-1,07	0,68114	---	TEDDM1
7922703	-0,87	-1,83	0,15326	-0,73	-1,66	0,04823	NM_172000	TTTY2
8176558	-0,87	-1,83	0,12886	-0,60	-1,51	0,04810	NR_001536	TTTY2
8177053	-0,87	-1,83	0,12886	-0,60	-1,51	0,04810	NR_001536	---
8078382	-0,87	-1,83	0,00959	-0,87	-1,83	0,06166	---	GCET2
8089478	-0,87	-1,83	0,10722	-1,38	-2,60	0,02697	NM_001008756	ACTA1
7924910	-0,87	-1,83	0,05918	-1,24	-2,36	0,00244	NM_001100	---
8142897	-0,87	-1,83	0,02134	-0,75	-1,68	0,00173	---	---
7919131	-0,87	-1,83	0,19275	-2,51	-5,69	0,00721	---	---
7974912	-0,87	-1,83	0,03133	-1,26	-2,40	0,04187	---	BEND3

8128620	-0,87	-1,83	0,00339	0,99	1,98	0,09296	NM_001080450	FAM180B
7939835	-0,87	-1,83	0,13284	-1,02	-2,03	0,06889	NM_001164379	PROZ
7970251	-0,88	-1,83	0,10776	-1,22	-2,33	0,02264	NM_003891	CCR6
8123362	-0,88	-1,83	0,00787	-0,78	-1,72	0,13428	BX647686	MRPL38
8018511	-0,88	-1,83	0,07808	-0,16	-1,12	0,66697	NM_032478	SH3D20
8016232	-0,88	-1,83	0,29068	-1,82	-3,53	0,00030	NM_174919	IGSF5
8068644	-0,88	-1,83	0,00336	-0,72	-1,64	0,14942	NM_001080444	C2orf52
8059716	-0,88	-1,84	0,03803	-0,26	-1,20	0,55026	BC033054	---
7900214	-0,88	-1,84	0,05840	-0,74	-1,67	0,00003	---	USP29
8031686	-0,88	-1,84	0,04811	-0,98	-1,98	0,07206	NM_020903	---
7904048	-0,88	-1,84	0,19191	-0,95	-1,94	0,00172	---	FKSG73
7927097	-0,88	-1,84	0,01081	-1,14	-2,21	0,00022	NR_027714	PIPOX
8006005	-0,88	-1,84	0,20295	-0,25	-1,19	0,10800	NM_016518	WBSCR16
8140220	-0,88	-1,84	0,02274	0,00	1,00	0,99718	NM_030798	STYK1
7961215	-0,88	-1,84	0,00377	0,63	1,55	0,00251	NM_018423	---
7960259	-0,88	-1,84	0,11741	-1,21	-2,32	0,03484	---	---
7930450	-0,88	-1,84	0,20638	-2,60	-6,07	0,00089	---	OR2A25
8136969	-0,88	-1,84	0,00285	-1,40	-2,63	0,00332	NM_001004488	LAMA5
8067409	-0,88	-1,84	0,01815	1,65	3,15	0,03561	NM_005560	APOBEC2
8119403	-0,88	-1,84	0,15265	-0,79	-1,73	0,00186	NM_006789	---
8029937	-0,88	-1,84	0,10678	-0,97	-1,96	0,00088	---	---
8086139	-0,88	-1,84	0,03306	-1,06	-2,09	0,00661	---	---
8042970	-0,88	-1,84	0,06437	-1,61	-3,06	0,01485	---	NPHS2
7922627	-0,88	-1,84	0,02898	-0,89	-1,86	0,01055	NM_014625	CDH7
8021668	-0,88	-1,84	0,00115	-0,89	-1,85	0,00780	NM_033646	NCRNA00189
8068100	-0,88	-1,84	0,06662	-1,06	-2,08	0,20722	AF490769	NRK
8169115	-0,88	-1,84	0,02338	-1,03	-2,05	0,03796	NM_198465	---
7941608	-0,88	-1,84	0,21260	-0,97	-1,96	0,00020	---	ARL11
7969145	-0,88	-1,84	0,09196	-1,19	-2,28	0,05107	NM_138450	CYP7A1
8150920	-0,88	-1,84	0,08742	-0,74	-1,67	0,00278	NM_000780	C8orf55
8148559	-0,88	-1,84	0,00716	0,54	1,46	0,33803	NM_016647	CCRL2
8093304	-0,88	-1,84	0,09555	-1,09	-2,12	0,11116	NM_003965	MIR10A
8016455	-0,88	-1,84	0,00827	-1,14	-2,21	0,01390	NR_029608	---
7972735	-0,88	-1,84	0,03755	-1,24	-2,36	0,00497	---	SSX3
8172406	-0,88	-1,84	0,24537	-1,15	-2,21	0,01873	NM_021014	HSN2
7952986	-0,88	-1,84	0,03856	1,16	2,24	0,00438	NM_213655	---
8019962	-0,88	-1,84	0,01195	-1,53	-2,88	0,00932	---	NAPSA
8038556	-0,88	-1,84	0,00505	1,45	2,74	0,02618	NM_004851	---
8144724	-0,88	-1,84	0,09091	-1,79	-3,46	0,01754	---	PRO2964
8057559	-0,88	-1,84	0,05008	-0,59	-1,51	0,05163	ENST00000424540	---
7948502	-0,88	-1,84	0,29104	-1,31	-2,49	0,00161	---	TAS1R3
7896921	-0,88	-1,84	0,17554	-1,42	-2,68	0,01487	NM_152228	PEX14
7897648	-0,88	-1,84	0,01790	-0,96	-1,94	0,14813	NM_004565	---
8160767	-0,88	-1,84	0,02702	-0,94	-1,92	0,00271	---	RIMS2
8147796	-0,88	-1,84	0,01718	1,04	2,05	0,03762	NM_001100117	FAM58B
7908612	-0,88	-1,84	0,14142	-0,03	-1,02	0,95609	NM_001105517	PROX2
7980213	-0,88	-1,84	0,12562	-1,27	-2,41	0,01360	NM_001080408	RNF182
8116980	-0,88	-1,84	0,01494	-0,11	-1,08	0,56210	NM_001165032	---
8161433	-0,88	-1,84	0,34490	-2,38	-5,21	0,00031	---	---
8131925	-0,88	-1,84	0,00379	-0,71	-1,63	0,00105	---	RPS6KA1
7899192	-0,88	-1,84	0,02083	2,26	4,78	0,00051	NM_002953	OR4A47
7939877	-0,88	-1,84	0,21300	-0,85	-1,80	0,04428	NM_001005512	DLGAP2
8144253	-0,88	-1,84	0,14864	-1,18	-2,26	0,00028	NM_004745	FAM9B
8171271	-0,88	-1,84	0,22902	-0,63	-1,55	0,01920	NM_205849	MAGEB2
8166611	-0,88	-1,84	0,00884	-0,94	-1,92	0,01670	NM_002364	CAGE1
8123780	-0,88	-1,84	0,00364	-1,05	-2,07	0,00551	NM_001170692	CYP4B1
7901256	-0,88	-1,84	0,03146	-1,40	-2,63	0,00277	NM_001099772	HEPHL1
7943197	-0,88	-1,84	0,07128	-1,32	-2,50	0,01234	NM_001098672	SNORD1B
8010080	-0,88	-1,84	0,01039	-0,91	-1,88	0,00021	NR_004396	MIR101-1
7916777	-0,88	-1,84	0,05727	-0,91	-1,88	0,01652	NR_029516	---
8097062	-0,88	-1,84	0,07177	-1,67	-3,19	0,01707	---	CDH5
7996264	-0,88	-1,84	0,02338	-0,91	-1,88	0,02992	NM_001795	ADAM5P
8146051	-0,88	-1,84	0,01554	-1,05	-2,07	0,00353	NR_001448	BMP5
8127193	-0,88	-1,84	0,03138	-0,80	-1,74	0,00860	NM_021073	---
7992131	-0,88	-1,84	0,10257	-1,30	-2,47	0,08301	---	---
8128258	-0,88	-1,84	0,04089	-0,94	-1,91	0,02315	---	LAX1
7908968	-0,88	-1,84	0,01269	-1,09	-2,13	0,03132	NM_017773	LOC200261
8065363	-0,88	-1,84	0,12027	-0,42	-1,34	0,15206	AF258576	---
8019937	-0,88	-1,84	0,21774	-1,43	-2,69	0,02675	---	---
7938748	-0,88	-1,84	0,07151	0,07	1,05	0,75591	---	FBP1
8162502	-0,88	-1,84	0,00511	0,19	1,14	0,60039	NM_000507	NTSR1
8063942	-0,88	-1,84	0,01476	-1,26	-2,40	0,03439	NM_002531	OR4D6
7940175	-0,88	-1,84	0,17759	-0,96	-1,95	0,00771	NM_001004708	C10orf53

7927505	-0,88	-1,84	0,02375	-1,32	-2,49	0,09281	NM_182554	CACNA1I
8073152	-0,88	-1,84	0,06303	-0,86	-1,81	0,08808	NM_021096	---
8104504	-0,88	-1,84	0,04171	-0,94	-1,92	0,03162	---	IHH
8059067	-0,88	-1,84	0,00861	-1,45	-2,73	0,01418	NM_002181	DNAH11
8131719	-0,88	-1,84	0,00110	-0,90	-1,87	0,00099	NM_003777	---
8111627	-0,88	-1,84	0,01952	-1,88	-3,67	0,02269	---	HLA-DRB3
8178802	-0,88	-1,84	0,03010	0,94	1,92	0,02487	NM_022555	---
7924340	-0,88	-1,84	0,00084	-0,94	-1,92	0,05547	---	FAM9A
8171260	-0,88	-1,84	0,02471	-0,89	-1,86	0,00283	NM_174951	IQCD
7966596	-0,88	-1,84	0,00569	-0,47	-1,38	0,16882	NM_138451	C16orf46
8002992	-0,88	-1,84	0,02339	-0,84	-1,79	0,13190	NM_152337	---
7908595	-0,88	-1,84	0,14201	-0,65	-1,56	0,00638	---	---
7923179	-0,88	-1,84	0,12547	-0,92	-1,90	0,01520	---	OR5M9
7948131	-0,88	-1,84	0,02983	-0,90	-1,87	0,04524	NM_001004743	FAM189A2
8155734	-0,88	-1,84	0,02821	1,11	2,15	0,00470	NM_004816	KIAA1683
8035435	-0,88	-1,84	0,19226	-0,40	-1,32	0,21882	NM_001145304	CLC
8036755	-0,88	-1,84	0,09483	-1,34	-2,54	0,00120	NM_001828	CD200R1L
8089559	-0,88	-1,84	0,32833	-1,28	-2,43	0,02934	NM_001008784	---
8059799	-0,88	-1,84	0,02397	-2,68	-6,41	0,00002	---	---
8121085	-0,88	-1,84	0,04827	-1,26	-2,39	0,00072	---	C9orf45
8164008	-0,88	-1,84	0,12523	1,01	2,02	0,16764	NR_026677	CTAGE6
8143601	-0,88	-1,84	0,06656	-0,76	-1,69	0,00012	NM_178561	CA10
8016808	-0,88	-1,84	0,01387	-1,20	-2,30	0,09320	NM_001082533	PHKG1
8139746	-0,88	-1,84	0,05984	-1,13	-2,19	0,10859	NM_006213	REG1B
8053330	-0,88	-1,84	0,04008	-1,38	-2,61	0,00905	NM_006507	MUM1
8024255	-0,88	-1,84	0,08101	1,39	2,63	0,04411	NM_032853	---
8044760	-0,88	-1,84	0,00950	-1,35	-2,55	0,02262	---	---
8123714	-0,88	-1,84	0,14463	-1,33	-2,52	0,01102	---	OR52K2
7937938	-0,88	-1,84	0,01516	-1,15	-2,22	0,00970	NM_001005172	---
8138440	-0,88	-1,84	0,18903	-0,95	-1,93	0,00270	---	---
8121332	-0,88	-1,84	0,10608	0,03	1,02	0,81255	---	---
8169465	-0,88	-1,84	0,15375	-0,91	-1,88	0,04565	---	NEIL1
7985001	-0,88	-1,84	0,04602	-0,11	-1,08	0,72398	NM_024608	---
7903028	-0,88	-1,84	0,09445	-0,77	-1,71	0,03357	---	MAGEB17
8166211	-0,88	-1,84	0,15573	-1,46	-2,76	0,03185	ENST00000400004	---
7981726	-0,88	-1,84	0,17185	-0,93	-1,91	0,02599	---	HLA-DPB2
8118607	-0,88	-1,84	0,01264	-0,42	-1,34	0,22473	NR_001435	---
7958128	-0,88	-1,84	0,00085	-1,08	-2,11	0,02533	---	---
7937959	-0,88	-1,84	0,09686	-1,28	-2,43	0,00273	---	IGHM // IGHM // IGHM // IGHM // IGHM // IGHM
7981718	-0,88	-1,84	0,36995	-3,26	-9,58	0,00001	BC020240 // BC020240 // BC020240 // BC020240 // BC020240 // BC020240	CST7
8061416	-0,88	-1,84	0,22054	-1,59	-3,01	0,04716	NM_003650	---
7982390	-0,88	-1,84	0,10004	-0,97	-1,96	0,05707	---	NUP62CL
8174351	-0,88	-1,84	0,11144	2,04	4,12	0,00224	NM_017681	---
8006227	-0,88	-1,84	0,07374	-1,21	-2,31	0,00133	---	---
8139017	-0,88	-1,84	0,23134	-0,70	-1,63	0,12521	---	STRA13
8019350	-0,88	-1,84	0,08311	1,02	2,03	0,04341	NM_144998	RESP18
8059213	-0,88	-1,85	0,11406	-1,22	-2,32	0,00340	NM_001007089	---
8097116	-0,88	-1,85	0,07992	-0,58	-1,49	0,00520	---	GGT6
8011671	-0,88	-1,85	0,09673	-1,31	-2,47	0,00024	NM_001122890	SOSTDC1
8138363	-0,88	-1,85	0,03069	-2,01	-4,04	0,00261	NM_015464	PRSS2
8136807	-0,88	-1,85	0,00600	0,27	1,21	0,42956	NM_002770	OR6B2
8060080	-0,88	-1,85	0,33748	-1,44	-2,71	0,11137	NM_001005853	MIR181C
8026212	-0,88	-1,85	0,11268	-0,92	-1,89	0,07922	NR_029613	CD320
8033554	-0,88	-1,85	0,07799	0,04	1,03	0,92401	NM_016579	---
7941380	-0,88	-1,85	0,06482	-0,43	-1,35	0,29352	---	---
8055639	-0,88	-1,85	0,31506	-1,40	-2,64	0,01014	---	DLX2
8056784	-0,88	-1,85	0,04644	-1,06	-2,09	0,01280	NM_004405	TYK2
8033996	-0,88	-1,85	0,01238	0,96	1,94	0,13479	NM_003331	---
8083998	-0,88	-1,85	0,00703	-1,05	-2,07	0,00080	---	ISY1
8090533	-0,89	-1,85	0,23841	0,35	1,27	0,10695	NM_020701	ANKRD33
7955551	-0,89	-1,85	0,09914	-1,45	-2,73	0,03205	NM_001130015	---
7933731	-0,89	-1,85	0,00867	-1,10	-2,14	0,01248	---	---
8023642	-0,89	-1,85	0,00469	-0,90	-1,87	0,00123	---	IL5RA
8085062	-0,89	-1,85	0,04175	-1,01	-2,01	0,00024	NM_000564	SNORD83B
8076219	-0,89	-1,85	0,30992	-2,28	-4,87	0,01442	NR_000028	---
7950469	-0,89	-1,85	0,10850	-1,53	-2,88	0,06007	---	---
8169636	-0,89	-1,85	0,00376	-1,13	-2,19	0,00002	---	FAM55B
7944023	-0,89	-1,85	0,00581	-1,26	-2,40	0,00362	NM_182495	PLEKHF1
8027398	-0,89	-1,85	0,13270	-0,95	-1,94	0,02726	NM_024310	FAM104B
8173148	-0,89	-1,85	0,29105	-0,13	-1,09	0,70730	NR_030722	GALNTL5
8137437	-0,89	-1,85	0,00894	-1,30	-2,45	0,00521	NR_033169	SAA1

7946986	-0,89	-1,85	0,00010	-1,35	-2,56	0,00273	NM_000331	SERPINA9
7981084	-0,89	-1,85	0,10772	-1,49	-2,80	0,00745	NM_175739	NR1I3
7921840	-0,89	-1,85	0,06657	-1,52	-2,88	0,01372	NM_001077482	MIR223
8167971	-0,89	-1,85	0,04143	-1,14	-2,20	0,02572	NR_029637	MIR138-2
7995866	-0,89	-1,85	0,05274	-1,46	-2,75	0,00553	NR_029680	IGSF11
8089729	-0,89	-1,85	0,01372	-0,80	-1,74	0,02207	NM_001015887	---
8046512	-0,89	-1,85	0,02805	-0,35	-1,27	0,07627	---	SLC7A4
8074701	-0,89	-1,85	0,05651	-1,36	-2,56	0,01257	NM_004173	LOC284232
7970388	-0,89	-1,85	0,00799	-1,08	-2,12	0,02657	NR_027995	---
8068660	-0,89	-1,85	0,03012	-0,98	-1,97	0,03952	---	GLTSCR1
8029939	-0,89	-1,85	0,05288	-0,85	-1,80	0,02784	NM_015711	NADK
7911600	-0,89	-1,85	0,01210	0,92	1,90	0,05250	NM_023018	USP17L2 // USP17L2
8149356	-0,89	-1,85	0,53783	-2,48	-5,58	0,00126	NM_201402 // NM_201402	---
8081079	-0,89	-1,85	0,06648	-0,73	-1,66	0,08055	---	---
7943345	-0,89	-1,85	0,01582	-0,68	-1,60	0,05721	---	MGAT5B
8010113	-0,89	-1,85	0,02591	-0,26	-1,20	0,11376	NM_144677	LGALS2
8075956	-0,89	-1,85	0,14579	-1,41	-2,65	0,03113	NM_006498	OR2B2
8124547	-0,89	-1,85	0,14933	-1,00	-2,00	0,01373	NM_033057	DECR2
7991846	-0,89	-1,85	0,01526	-0,25	-1,19	0,60246	NM_020664	MAGEC1
8170273	-0,89	-1,85	0,03171	-0,87	-1,82	0,04832	NM_005462	---
8081333	-0,89	-1,85	0,03433	-1,23	-2,35	0,03110	---	DNAH2
8004580	-0,89	-1,85	0,01024	-0,57	-1,48	0,00244	NM_020877	OAF
7944530	-0,89	-1,85	0,00530	-0,86	-1,82	0,26461	NM_178507	EYS
8127399	-0,89	-1,85	0,05247	-0,66	-1,58	0,02036	NM_001142800	FBXL17
8113445	-0,89	-1,85	0,10216	-1,04	-2,06	0,05150	NM_001163315	IGDCC4
7989785	-0,89	-1,85	0,03632	0,50	1,42	0,25335	NM_020962	BTLA
8089519	-0,89	-1,85	0,21638	-1,07	-2,10	0,02618	NM_181780	PLIN1
7991313	-0,89	-1,85	0,10346	-1,27	-2,41	0,00420	NM_002666	---
7910012	-0,89	-1,85	0,04695	-0,67	-1,59	0,01322	---	BNC1
7991080	-0,89	-1,85	0,00360	2,51	5,68	0,00129	NM_001717	---
8142230	-0,89	-1,85	0,01483	-0,89	-1,86	0,08521	---	MGC29506
8114511	-0,89	-1,85	0,01335	-0,50	-1,42	0,01746	NM_016459	KRT19
8015349	-0,89	-1,85	0,03763	1,10	2,15	0,08451	NM_002276	---
7928748	-0,89	-1,85	0,10961	-1,44	-2,71	0,00008	---	PKD2L1
7935756	-0,89	-1,85	0,05278	-0,87	-1,83	0,00190	NM_016112	OR5AR1
7939992	-0,89	-1,85	0,25832	-0,78	-1,72	0,01467	NM_001004730	IL17REL
8076865	-0,89	-1,85	0,09392	-1,38	-2,60	0,00054	NM_001001694	ZNF705A
7953733	-0,89	-1,85	0,09706	-0,42	-1,34	0,34868	NM_001004328	CSF1R
8115076	-0,89	-1,85	0,00968	-0,39	-1,31	0,06172	NM_005211	ELSPBP1
8029988	-0,89	-1,85	0,12279	-1,15	-2,21	0,00007	NM_022142	C17orf104
8007643	-0,89	-1,85	0,07708	2,32	5,00	0,00533	NM_001145080	SAFB2
8032974	-0,89	-1,85	0,04900	0,43	1,35	0,32491	NM_014649	HIPK4
8036883	-0,89	-1,85	0,03625	-1,19	-2,29	0,00986	NM_144685	PNLDC1
8123155	-0,89	-1,85	0,00022	-1,12	-2,17	0,01929	NM_173516	WDR93
7985898	-0,89	-1,85	0,06555	-0,56	-1,48	0,01425	NM_020212	KCNQ1DN
7937847	-0,89	-1,85	0,04857	-2,12	-4,35	0,00347	NR_024627	---
7919886	-0,89	-1,85	0,00077	-0,76	-1,69	0,00323	---	FLJ30403
8030838	-0,89	-1,85	0,07960	0,45	1,37	0,10565	ENST00000301439	GPR160
8083839	-0,89	-1,85	0,05968	1,17	2,25	0,01605	NM_014373	---
8164560	-0,89	-1,85	0,04874	-0,63	-1,55	0,12365	---	FLJ46020
8068350	-0,89	-1,85	0,18135	-0,66	-1,58	0,18508	AK127913 WDR64	
7910971	-0,89	-1,85	0,00887	-1,11	-2,15	0,00025	NM_144625	RAB36
8074880	-0,89	-1,85	0,11960	-0,94	-1,91	0,04902	NM_004914	FXYD2
7944147	-0,89	-1,85	0,02250	-1,93	-3,81	0,00519	NM_001680	TEX101
8029289	-0,89	-1,85	0,06416	-1,38	-2,60	0,11535	NM_031451	TESC
7966749	-0,89	-1,85	0,06193	0,77	1,70	0,05991	NR_031766	---
7981011	-0,89	-1,85	0,53472	-1,98	-3,95	0,00515	---	TRMT61A
7977105	-0,89	-1,85	0,00664	-0,16	-1,11	0,13203	NM_152307	CD300A
8009653	-0,89	-1,85	0,19121	-0,46	-1,37	0,28929	NM_007261	TYROBP
8036224	-0,89	-1,85	0,13668	-1,54	-2,90	0,00698	NM_003332	PRSS54
8001682	-0,89	-1,85	0,02978	-1,33	-2,51	0,00604	NM_001080492	MMRN1
8096415	-0,89	-1,85	0,12794	-1,15	-2,22	0,05683	NM_007351	TSHZ2
8063444	-0,89	-1,85	0,05303	-1,17	-2,26	0,05745	ENST00000371497	---
7970947	-0,89	-1,85	0,04816	-0,36	-1,28	0,02339	---	---
7990892	-0,89	-1,85	0,01657	-1,92	-3,79	0,00069	---	CDT1
7997839	-0,89	-1,86	0,00841	-0,55	-1,46	0,16539	NM_030928	NKX2-6
8149820	-0,89	-1,86	0,04346	-2,17	-4,49	0,00861	NM_001136271	GRIK1
8068120	-0,89	-1,86	0,09978	-0,42	-1,34	0,13982	NM_000830	TRYX3
8143486	-0,89	-1,86	0,12488	-0,49	-1,40	0,05389	NM_001001317	---
7971482	-0,89	-1,86	0,07219	-1,33	-2,52	0,02675	---	CSAG2
8170580	-0,89	-1,86	0,01748	-0,79	-1,73	0,00574	NM_001080848	---
8156969	-0,89	-1,86	0,00020	-0,44	-1,35	0,25044	---	---
8113299	-0,89	-1,86	0,19029	-1,28	-2,43	0,00072	---	WEE1

7938364	-0,89	-1,86	0,06265	-1,08	-2,11	0,00991	BX641032	---	
8028309	-0,89	-1,86	0,00403	-0,43	-1,35	0,07950	---	---	
8133165	-0,89	-1,86	0,03403	-0,65	-1,57	0,03259	---	NXF2	
8168900	-0,89	-1,86	0,05386	-0,73	-1,65	0,00661	NM_017809		---
8165862	-0,89	-1,86	0,12417	-0,68	-1,61	0,00838	---	FLJ45244	
7976512	-0,89	-1,86	0,07706	-0,45	-1,37	0,00041	NR_015415		---
8062569	-0,89	-1,86	0,00627	-0,76	-1,70	0,05875	---	LCE1C	
7920193	-0,89	-1,86	0,43346	-0,95	-1,93	0,20818	NM_178351		SNORD73A
8097792	-0,89	-1,86	0,04544	-0,44	-1,35	0,01044	NR_000007		CAPN9
7910466	-0,89	-1,86	0,05294	-1,08	-2,11	0,00213	NM_006615		AMPD2
7903719	-0,89	-1,86	0,04944	0,50	1,41	0,36325	NM_004037		---
8046790	-0,89	-1,86	0,05864	-1,01	-2,01	0,01550	---	GLI4	
8148607	-0,89	-1,86	0,01473	-0,12	-1,09	0,77043	NM_138465		---
8027377	-0,89	-1,86	0,12363	-1,72	-3,30	0,04188	---	OR4C15	
7939924	-0,89	-1,86	0,08936	-0,81	-1,75	0,00725	NM_001001920		OR5M8
7948137	-0,89	-1,86	0,07918	-1,81	-3,51	0,00024	NM_001005282		SLC22A15
7904226	-0,89	-1,86	0,00924	0,13	1,09	0,73013	NM_018420		---
7965976	-0,89	-1,86	0,00050	0,11	1,08	0,64893	---	TTN	
8057056	-0,89	-1,86	0,00215	-1,07	-2,10	0,00001	NM_133378		LAIR1
8039257	-0,89	-1,86	0,01629	-1,26	-2,39	0,00997	NM_002287		SIX3
8041759	-0,89	-1,86	0,16851	-0,58	-1,50	0,16573	NM_005413		ADAMTS16
8104321	-0,89	-1,86	0,06280	-0,43	-1,35	0,45139	NM_139056		SNX31
8152062	-0,89	-1,86	0,05284	-0,61	-1,52	0,08519	NM_152628		OR52R1
7946001	-0,89	-1,86	0,08757	-0,70	-1,62	0,02424	NM_001005177		PLAC1L
7940210	-0,89	-1,86	0,12520	-1,18	-2,27	0,00162	NM_173801		---
8145083	-0,89	-1,86	0,15578	-1,00	-2,00	0,05000	---	POTEE	
8055222	-0,89	-1,86	0,02602	-1,84	-3,57	0,00069	NM_001083538		TBC1D10A
8075332	-0,89	-1,86	0,01978	0,13	1,10	0,78794	NM_031937		CLCA3P
7902754	-0,89	-1,86	0,01023	-1,37	-2,58	0,00027	NR_024604		---
8120163	-0,89	-1,86	0,05349	-0,41	-1,33	0,02487	---	HIST3H2A	
7924888	-0,89	-1,86	0,04210	-0,90	-1,87	0,10759	NM_033445		SCRT2
8064384	-0,89	-1,86	0,04023	-2,29	-4,90	0,00115	NM_033129		ADAMTSL1
8154512	-0,89	-1,86	0,13994	-0,95	-1,93	0,00126	NM_001040272		---
7922652	-0,89	-1,86	0,10132	-1,43	-2,70	0,00357	---	ENTPD8	
8165538	-0,90	-1,86	0,00861	-0,10	-1,07	0,76322	NM_001033113		RTEL1
8064111	-0,90	-1,86	0,04823	-0,14	-1,10	0,61492	NM_032957		---
8118820	-0,90	-1,86	0,18818	-1,25	-2,38	0,01071	---	OR13J1	
8161164	-0,90	-1,86	0,15105	-1,43	-2,70	0,02021	NM_001004487		KIAA1804
7910600	-0,90	-1,86	0,02383	1,67	3,17	0,01138	NM_032435		CABP1
7959195	-0,90	-1,86	0,03522	-1,82	-3,54	0,00007	NM_001033677		GAGE12J
8167508	-0,90	-1,86	0,43224	-2,00	-4,01	0,01240	NM_001098406		---
8174668	-0,90	-1,86	0,00159	-0,92	-1,89	0,07406	---	---	
8166657	-0,90	-1,86	0,02464	-1,53	-2,89	0,00260	---	CASKIN1	
7998724	-0,90	-1,86	0,03913	-0,51	-1,42	0,03927	NM_020764		UGT1A1
8049349	-0,90	-1,86	0,00277	-0,85	-1,80	0,01351	NM_000463		---
8138822	-0,90	-1,86	0,05944	-1,39	-2,63	0,09052	---	AP1G2	
7978066	-0,90	-1,86	0,02185	1,23	2,34	0,04221	NM_003917		C14orf181
7979820	-0,90	-1,86	0,02280	-1,25	-2,37	0,04808	NM_207442		---
8046014	-0,90	-1,86	0,04363	-1,47	-2,78	0,01080	---	LOC100132147	
7912804	-0,90	-1,86	0,35032	-3,21	-9,27	0,00033	BC036435	IL1F8	
8054731	-0,90	-1,86	0,00173	-1,00	-2,00	0,03596	NM_014438		CD40LG
8170187	-0,90	-1,86	0,00637	-0,45	-1,36	0,07641	NM_000074		PRY
8177323	-0,90	-1,86	0,07575	-1,42	-2,68	0,00548	NM_004676		---
7926123	-0,90	-1,86	0,05338	-0,85	-1,80	0,25891	---	CD80	
8089771	-0,90	-1,86	0,00643	-1,11	-2,15	0,05287	NM_005191		---
8099758	-0,90	-1,86	0,02041	-0,71	-1,64	0,02089	---	BNIPL	
7905308	-0,90	-1,86	0,09297	-0,33	-1,26	0,14704	NM_001159642		SNORD116-21
7981990	-0,90	-1,86	0,05625	-0,08	-1,06	0,87614	NR_003335		---
8074789	-0,90	-1,86	0,01161	-1,26	-2,39	0,00022	---	---	
8115863	-0,90	-1,86	0,08124	-1,06	-2,09	0,12485	---	PDE10A	
8130674	-0,90	-1,86	0,07885	-1,54	-2,91	0,02120	NM_006661		CDRT1
8012931	-0,90	-1,86	0,22768	-0,88	-1,84	0,17474	NM_006382		---
8147652	-0,90	-1,86	0,00893	-0,87	-1,82	0,00020	---	---	
7927639	-0,90	-1,86	0,06543	-1,75	-3,37	0,00180	---	---	
8151508	-0,90	-1,86	0,03946	-1,75	-3,37	0,00227	---	SCLY	
8049582	-0,90	-1,86	0,01959	1,16	2,24	0,05561	NM_016510		C3orf25
8090567	-0,90	-1,86	0,02502	-1,10	-2,14	0,00302	BC066975	---	
8140033	-0,90	-1,86	0,04873	-1,03	-2,04	0,00016	---	MIR142	
8016980	-0,90	-1,86	0,00944	-1,14	-2,20	0,00466	NR_029683		C2orf15
8043835	-0,90	-1,86	0,00315	2,10	4,29	0,00824	BC021264	CEACAM21	
8029076	-0,90	-1,86	0,00108	-0,91	-1,88	0,03252	NM_001098506		CCDC64
7959131	-0,90	-1,86	0,02570	0,71	1,64	0,21955	NM_207311		---
8089926	-0,90	-1,86	0,15934	-1,26	-2,39	0,01707	---	SHROOM4	

8172698	-0,90	-1,86	0,02787	-0,87	-1,83	0,05529	NR_027121	AQP7P1
8161558	-0,90	-1,86	0,22195	-2,07	-4,21	0,01852	NR_002817	OR13H1
8169947	-0,90	-1,86	0,17933	-0,41	-1,33	0,17548	NM_001004486	---
8096331	-0,90	-1,86	0,03463	-1,77	-3,42	0,00880	---	hCG_2011852
7972670	-0,90	-1,86	0,06388	-0,90	-1,87	0,00055	ENST00000322527	RERGL
7961595	-0,90	-1,86	0,00498	-0,56	-1,48	0,22185	NM_024730	EMX2
7930857	-0,90	-1,86	0,13006	-1,63	-3,09	0,01253	NM_004098	ZCCHC13
8168420	-0,90	-1,86	0,23845	-0,47	-1,38	0,21443	NM_203303	LOC100128003
7897066	-0,90	-1,86	0,04725	-1,07	-2,09	0,07189	NR_024445	STOX1
7927915	-0,90	-1,86	0,00846	2,85	7,20	0,00037	NM_152709	DGCR6
8071179	-0,90	-1,86	0,08010	-1,20	-2,29	0,28682	NM_005675	SRL
7999093	-0,90	-1,86	0,15818	-0,79	-1,74	0,02025	NM_001098814	FBXO16
8150002	-0,90	-1,86	0,00627	2,68	6,43	0,00304	NM_172366	---
8083790	-0,90	-1,86	0,07113	-1,18	-2,26	0,00029	---	LOC645339
7925058	-0,90	-1,86	0,08583	-0,89	-1,85	0,05905	BC014600	P2RY13
8091523	-0,90	-1,87	0,07923	-1,11	-2,16	0,01118	NM_176894	---
7963758	-0,90	-1,87	0,12408	-0,95	-1,94	0,01543	---	---
7943709	-0,90	-1,87	0,07912	-0,99	-1,98	0,00354	---	AGRN
7896822	-0,90	-1,87	0,06140	1,86	3,64	0,06465	NM_198576	---
8092199	-0,90	-1,87	0,07337	-0,76	-1,69	0,00049	---	NOTCH4
8125383	-0,90	-1,87	0,05758	-1,66	-3,16	0,00044	NM_004557	PRAMEF15
7898012	-0,90	-1,87	0,31289	-1,04	-2,05	0,01302	NM_001098376	PRAMEF15
7898040	-0,90	-1,87	0,31289	-1,04	-2,05	0,01302	NM_001098376	PON1
8141052	-0,90	-1,87	0,05338	0,00	-1,00	0,99976	NM_000446	BOP1
8153664	-0,90	-1,87	0,01344	1,29	2,44	0,02231	NM_015201	TTC25
8007176	-0,90	-1,87	0,02573	-0,34	-1,27	0,48128	NM_031421	SLC22A23
8123658	-0,90	-1,87	0,03496	-0,13	-1,09	0,75833	NM_015482	SLC30A2
7913896	-0,90	-1,87	0,04951	-1,58	-2,98	0,00634	NM_001004434	FLYWCH1
7992744	-0,90	-1,87	0,01098	-0,55	-1,46	0,12125	NM_032296	---
8164426	-0,90	-1,87	0,05112	-1,53	-2,88	0,02427	---	---
7924443	-0,90	-1,87	0,00100	-1,45	-2,72	0,00252	---	ALOX5
7927215	-0,90	-1,87	0,08511	-0,44	-1,36	0,13197	NM_000698	AGAP4
7927231	-0,90	-1,87	0,17720	1,32	2,49	0,03688	NM_133446	JMJD7-PLA2G4B
7983021	-0,90	-1,87	0,03773	-0,40	-1,32	0,21330	NR_015346	C16orf54
8000702	-0,90	-1,87	0,03748	-1,29	-2,44	0,03729	NM_175900	C16orf54
8003601	-0,90	-1,87	0,03748	-1,29	-2,44	0,03729	NM_175900	NBPF22P
8106771	-0,90	-1,87	0,29391	-0,61	-1,53	0,52188	NR_003719	---
8070295	-0,90	-1,87	0,18434	-1,44	-2,72	0,00236	---	RAMP3
8132660	-0,90	-1,87	0,02220	-0,97	-1,96	0,01060	NM_005856	---
7915657	-0,90	-1,87	0,07337	-0,97	-1,96	0,04592	---	---
7998249	-0,90	-1,87	0,00817	-1,55	-2,92	0,00213	---	---
8004219	-0,90	-1,87	0,14966	-1,49	-2,80	0,02235	---	ADORA3
7918533	-0,90	-1,87	0,11391	-1,30	-2,47	0,00128	NM_020683	FLJ43390
7974895	-0,90	-1,87	0,01880	-1,26	-2,40	0,06755	NR_015358	---
7959014	-0,90	-1,87	0,08087	-0,96	-1,95	0,01086	---	TRMT5
7974814	-0,90	-1,87	0,05266	-1,27	-2,40	0,00975	NM_020810	SGSM2
8003733	-0,90	-1,87	0,09887	1,14	2,20	0,00970	NM_014853	CHST4
7997152	-0,90	-1,87	0,12835	-1,02	-2,03	0,00765	NM_001166395	---
7948371	-0,90	-1,87	0,00339	-1,07	-2,09	0,00491	---	VWF
7960464	-0,90	-1,87	0,01887	-1,27	-2,40	0,00002	NM_000552	CCL18
8006594	-0,90	-1,87	0,00953	-0,99	-1,99	0,00087	NM_002988	---
8029375	-0,90	-1,87	0,08057	-1,44	-2,71	0,02614	---	CDH23
7928218	-0,90	-1,87	0,03961	-1,17	-2,24	0,00235	NM_022124	LOC131055
8083777	-0,90	-1,87	0,00676	-0,65	-1,57	0,09543	ENST00000407723	---
7917737	-0,90	-1,87	0,34562	-0,43	-1,35	0,05691	---	ZSCAN5A
8039577	-0,90	-1,87	0,02960	-0,27	-1,20	0,17651	NM_024303	---
7948416	-0,90	-1,87	0,18938	-1,09	-2,13	0,00060	---	---
7978822	-0,90	-1,87	0,06098	-0,87	-1,82	0,07938	---	RIPPLY2
8120937	-0,90	-1,87	0,13576	-0,58	-1,49	0,05419	NM_001009994	---
7997724	-0,90	-1,87	0,09841	-1,00	-2,00	0,03228	---	INSL4
8154229	-0,90	-1,87	0,00307	-1,06	-2,09	0,00255	NM_002195	MIR519A2
8031045	-0,90	-1,87	0,03026	-0,84	-1,79	0,00216	NR_030222	---
8109967	-0,90	-1,87	0,00641	-2,42	-5,35	0,01687	---	---
7937800	-0,90	-1,87	0,21410	-1,84	-3,59	0,01744	---	LOC283693
7991017	-0,90	-1,87	0,17969	-0,95	-1,93	0,02685	BC020234	ARR3
8168117	-0,90	-1,87	0,01057	-1,22	-2,33	0,00901	NM_004312	RGS14
8110327	-0,90	-1,87	0,13375	-0,64	-1,56	0,12617	NM_006480	DSCR4
8070286	-0,90	-1,87	0,09490	-1,63	-3,10	0,00016	NM_005867	TAS2R1
8110960	-0,90	-1,87	0,04292	-1,07	-2,11	0,02042	NM_019599	ASXL3
8020844	-0,90	-1,87	0,07862	-1,52	-2,86	0,01136	NM_030632	RP1L1
8149283	-0,90	-1,87	0,09300	-0,97	-1,96	0,05236	NM_178857	PDE6H
7954136	-0,90	-1,87	0,22503	-1,78	-3,44	0,00221	NM_006205	ZP2
8000097	-0,90	-1,87	0,02047	-0,93	-1,91	0,00773	NM_003460	OR5B3

7948324	-0,90	-1,87	0,03284	-0,91	-1,88	0,00201	NM_001005469	---
7981872	-0,90	-1,87	0,31089	-1,56	-2,95	0,01314	---	KRTAP19-6
8069841	-0,90	-1,87	0,00008	-1,41	-2,66	0,01003	NM_181612	SPACA1
8121066	-0,90	-1,87	0,00684	-1,03	-2,04	0,00942	NM_030960	CUBN
7932326	-0,90	-1,87	0,06333	-0,59	-1,50	0,09673	NM_001081	---
7962557	-0,90	-1,87	0,01303	-1,20	-2,29	0,00187	---	LY9
7906622	-0,90	-1,87	0,02432	0,13	1,09	0,36484	NM_002348	FAM38A
7997827	-0,91	-1,87	0,01333	-0,66	-1,58	0,08000	NM_001142864	C6orf15
8178439	-0,91	-1,87	0,13214	-1,30	-2,47	0,00251	NM_014070	FAM95B1
8155418	-0,91	-1,87	0,13141	-1,40	-2,63	0,03462	AL833349	---
8074192	-0,91	-1,87	0,30238	-4,08	-16,92	0,00187	---	MST1P9
7912863	-0,91	-1,87	0,05808	0,47	1,38	0,28946	NR_002729	AGMAT
7912659	-0,91	-1,87	0,00467	-0,92	-1,89	0,09019	NM_024758	FPR2
8030860	-0,91	-1,87	0,18550	-1,33	-2,51	0,00237	NM_001462	OR52D1
7938008	-0,91	-1,87	0,08542	-1,69	-3,22	0,01009	NM_001005163	SNORD59B
7964246	-0,91	-1,87	0,07033	-0,51	-1,43	0,11031	NR_003046	---
8137470	-0,91	-1,87	0,08218	-0,99	-1,98	0,02535	---	ZCCHC12
8169598	-0,91	-1,87	0,11686	-1,92	-3,78	0,00006	NM_173798	PYDC1
8001037	-0,91	-1,87	0,04741	-1,40	-2,64	0,04719	NM_152901	ANKRD34C
7985238	-0,91	-1,87	0,21722	-0,91	-1,88	0,02365	NM_001146341	---
7917910	-0,91	-1,87	0,17300	-1,42	-2,68	0,00376	---	RASSF9
7965226	-0,91	-1,87	0,00183	-0,67	-1,59	0,01446	NM_005447	---
8082238	-0,91	-1,87	0,24225	-2,61	-6,10	0,00432	---	---
8174281	-0,91	-1,87	0,01888	-0,99	-1,98	0,02964	---	OR7E125P
8149148	-0,91	-1,87	0,56559	-3,34	-10,16	0,00002	ENST00000420198	ODF4
8004861	-0,91	-1,87	0,10529	-1,27	-2,42	0,00880	NM_153007	MYL3
8086689	-0,91	-1,87	0,09132	-0,68	-1,61	0,14743	NM_000258	VSIG6
7986639	-0,91	-1,87	0,15260	-2,28	-4,85	0,01475	ENST00000338567	AGER
8125341	-0,91	-1,87	0,17864	-0,20	-1,15	0,51740	NM_001136	SOX15
8012220	-0,91	-1,87	0,05034	-0,94	-1,91	0,00706	NM_006942	KIAA1751
7911659	-0,91	-1,87	0,03475	-0,73	-1,66	0,05110	ENST00000270720	---
8149823	-0,91	-1,88	0,00653	-1,18	-2,26	0,05537	---	ANAPC11
8010686	-0,91	-1,88	0,09113	-0,11	-1,08	0,60206	NM_001002244	C10orf114
7932495	-0,91	-1,88	0,12256	-0,42	-1,34	0,15806	NM_001010911	DHX38
7997197	-0,91	-1,88	0,00469	0,42	1,34	0,36961	NM_014003	DAZ2
8176832	-0,91	-1,88	0,02650	-1,38	-2,60	0,00141	NM_020363	DAZ1
8176878	-0,91	-1,88	0,02650	-1,38	-2,60	0,00141	NM_004081	DAZ1
8177354	-0,91	-1,88	0,02650	-1,38	-2,60	0,00141	NM_004081	OR7E125P
8144422	-0,91	-1,88	0,54106	-3,33	-10,04	0,00003	ENST00000420198	NXNL2
8156274	-0,91	-1,88	0,12612	-1,98	-3,96	0,00694	NM_145283	KIAA1161
8160786	-0,91	-1,88	0,02026	1,37	2,58	0,00347	NM_020702	DMBX1
7901247	-0,91	-1,88	0,19256	-1,40	-2,63	0,01696	NM_172225	RENBP
8175933	-0,91	-1,88	0,01316	-1,16	-2,24	0,05037	NM_002910	---
8058386	-0,91	-1,88	0,11922	-1,00	-2,00	0,04691	---	APOH
8017766	-0,91	-1,88	0,00815	-0,51	-1,42	0,06785	NM_000042	---
7957833	-0,91	-1,88	0,00438	-1,00	-2,00	0,01927	---	ESRP1
8147351	-0,91	-1,88	0,03438	4,52	23,02	0,00001	NM_017697	BCL2L2
7973377	-0,91	-1,88	0,23652	0,08	1,06	0,68500	NM_004050	CYP7B1
8151056	-0,91	-1,88	0,00337	-1,48	-2,78	0,00266	NM_004820	KIR2DL1
8031297	-0,91	-1,88	0,22518	-2,17	-4,51	0,00050	NM_014218	DNAH6
8043071	-0,91	-1,88	0,00014	-0,96	-1,95	0,01243	NM_001370	EFTUD1
7985555	-0,91	-1,88	0,42205	2,27	4,81	0,00250	NM_024580	NKX6-2
7937106	-0,91	-1,88	0,00913	-0,75	-1,68	0,03595	NM_177400	OR7A5
8034892	-0,91	-1,88	0,00538	-1,10	-2,14	0,01065	NM_017506	FBXO47
8014757	-0,91	-1,88	0,00747	-0,73	-1,65	0,01987	NM_001008777	---
8038023	-0,91	-1,88	0,05276	-0,91	-1,88	0,01303	---	CLRN1
8091491	-0,91	-1,88	0,03367	-0,81	-1,76	0,00038	NM_052995	DNAH3
8000034	-0,91	-1,88	0,00745	-0,67	-1,59	0,00386	NM_017539	---
8106168	-0,91	-1,88	0,00100	-1,08	-2,12	0,00540	---	---
8171535	-0,91	-1,88	0,05430	-0,45	-1,37	0,21308	---	C17orf46
8016187	-0,91	-1,88	0,01580	-1,39	-2,62	0,02408	NM_152343	EXOSC6
8002344	-0,91	-1,88	0,13576	-1,31	-2,48	0,00251	NM_058219	PHYHIPL
7927694	-0,91	-1,88	0,17432	-0,44	-1,35	0,31269	NM_032439	---
7975770	-0,91	-1,88	0,00144	-0,86	-1,81	0,11190	---	---
8096077	-0,91	-1,88	0,21610	-0,58	-1,50	0,09242	---	LOC400965
8043320	-0,91	-1,88	0,05719	-0,97	-1,96	0,04181	AK098018	C17orf76
8013022	-0,91	-1,88	0,03393	-0,36	-1,29	0,19757	NM_001113567	SLC9A11
7922353	-0,91	-1,88	0,00277	-1,22	-2,34	0,00719	NM_178527	---
8066275	-0,91	-1,88	0,00471	-0,66	-1,58	0,00835	---	---
7971901	-0,91	-1,88	0,01229	-1,21	-2,32	0,00581	---	PIK3R6
8012539	-0,91	-1,88	0,10733	-1,32	-2,49	0,09347	NM_001010855	STAC2
8014812	-0,91	-1,88	0,05037	-0,44	-1,35	0,26510	NM_198993	MIR34C
7943738	-0,91	-1,88	0,04862	-0,87	-1,83	0,01160	NR_029840	MS4A8B

7940323	-0,91	-1,88	0,00042	-1,48	-2,78	0,00247	NM_031457	PKMYT1
7998886	-0,91	-1,88	0,03070	0,20	1,15	0,67792	NM_182687	C6orf221
8120675	-0,91	-1,88	0,00664	-0,98	-1,97	0,05621	NM_001017361	---
7983938	-0,91	-1,88	0,02036	0,25	1,19	0,67697	---	OR13C2
8162936	-0,91	-1,88	0,19187	-0,54	-1,45	0,22045	NM_001004481	TFAP2A
8123864	-0,91	-1,88	0,23782	3,74	13,37	0,00146	NM_003220	GSG1
7961399	-0,91	-1,88	0,06993	-0,80	-1,74	0,00125	NM_001080554	---
8016504	-0,91	-1,88	0,15237	-0,79	-1,73	0,00007	---	---
8022970	-0,91	-1,88	0,02559	-0,83	-1,78	0,00417	---	HRNBP3
8018993	-0,91	-1,88	0,04091	-1,22	-2,34	0,00313	NM_001082575	OR8B12
7952406	-0,91	-1,88	0,07219	-0,78	-1,72	0,02867	NM_001005195	WT1
7947363	-0,91	-1,88	0,29009	-1,70	-3,25	0,00157	NM_024424	PRAMEF3
7897997	-0,91	-1,88	0,06675	-2,15	-4,44	0,00031	NM_001013692	FNDC5
7914580	-0,91	-1,88	0,01798	-0,13	-1,10	0,44753	NM_001171941	IL1F7
8044532	-0,91	-1,88	0,12192	-1,67	-3,18	0,00266	NM_014439	NOX3
8130447	-0,91	-1,88	0,08612	-1,55	-2,92	0,01043	NM_015718	---
8132847	-0,91	-1,88	0,11388	-2,21	-4,62	0,03057	---	GRHL1
8040190	-0,91	-1,88	0,14597	0,81	1,76	0,04762	NM_198182	DDX53
8166440	-0,91	-1,88	0,00928	-1,58	-2,99	0,00202	NM_182699	DOK2
8149638	-0,91	-1,88	0,02518	-0,80	-1,74	0,01041	NM_003974	CHRNA6
8150550	-0,91	-1,88	0,08016	-0,41	-1,33	0,05677	NM_004198	---
8052303	-0,91	-1,88	0,04231	-1,71	-3,27	0,00665	---	KIAA1920
7991106	-0,91	-1,88	0,12885	-1,25	-2,38	0,04836	AB067507	---
8055670	-0,91	-1,88	0,00183	-0,80	-1,74	0,03487	---	DNAH17
8018908	-0,91	-1,88	0,01865	-0,81	-1,76	0,05495	NM_173628	NCRNA00173
7959016	-0,91	-1,88	0,03697	-1,28	-2,43	0,00839	NR_027345	---
8078565	-0,91	-1,88	0,08445	-0,70	-1,63	0,07531	---	---
8115541	-0,91	-1,88	0,03316	-0,89	-1,85	0,01530	---	ILVBL
8034920	-0,91	-1,88	0,00724	0,37	1,29	0,54223	NM_006844	OR2L5
7911248	-0,91	-1,88	0,02880	-2,12	-4,33	0,00002	ENST00000355281	UGT2B4
8100784	-0,91	-1,88	0,20539	-0,70	-1,62	0,00712	NM_021139	PASK
8060205	-0,91	-1,88	0,08729	-0,36	-1,28	0,10726	NM_015148	SNORD115-48
7982096	-0,91	-1,88	0,03972	-0,38	-1,30	0,00512	NR_003362	---
8046279	-0,91	-1,88	0,15690	0,44	1,36	0,43997	---	SOX30
8115516	-0,91	-1,88	0,04854	-0,72	-1,65	0,11919	NM_178424	CYP21A2
8178177	-0,91	-1,88	0,06457	-0,64	-1,56	0,23855	NM_000500	CYP21A2
8179440	-0,91	-1,88	0,06457	-0,64	-1,56	0,23855	NM_000500	LOC100129648
8076327	-0,91	-1,88	0,06933	-0,81	-1,76	0,00697	AK097637	TUBBP5
8159786	-0,91	-1,88	0,07894	0,64	1,56	0,23491	NR_027156	ALOXE3
8012326	-0,91	-1,88	0,04222	0,19	1,14	0,33283	NM_001165960	C1QTNF5
7952227	-0,91	-1,88	0,04918	-1,18	-2,26	0,01933	NM_015645	TFAP2A
8116831	-0,91	-1,88	0,04546	1,87	3,65	0,01616	NM_003220	---
8171798	-0,91	-1,88	0,05105	-1,34	-2,54	0,00552	---	MIA2
7974080	-0,91	-1,88	0,02052	-0,26	-1,20	0,00607	NM_054024	DEFB108B
7942284	-0,91	-1,88	0,37586	-2,89	-7,39	0,00202	NM_001002035	DEFB108B
8094136	-0,91	-1,88	0,37586	-2,89	-7,39	0,00202	NM_001002035	---
7904252	-0,91	-1,88	0,00821	-1,70	-3,25	0,00007	---	---
7931838	-0,91	-1,88	0,05228	-1,33	-2,51	0,00605	---	---
8139128	-0,91	-1,88	0,09169	-0,52	-1,43	0,54948	---	ASCL3
7946436	-0,91	-1,88	0,00861	-1,08	-2,11	0,01569	NM_020646	TMEM44
8092888	-0,91	-1,88	0,02872	0,37	1,29	0,16886	NM_138399	SLC10A1
7979878	-0,91	-1,89	0,01318	-1,09	-2,13	0,04314	NM_003049	---
8055402	-0,91	-1,89	0,04654	-1,49	-2,81	0,00484	---	NPC1L1
8139367	-0,91	-1,89	0,04633	-1,63	-3,09	0,00004	NM_013389	FAM47E
8095819	-0,91	-1,89	0,01321	-0,60	-1,51	0,15400	NM_001136570	---
7907568	-0,91	-1,89	0,03035	-1,66	-3,16	0,00892	---	---
8168565	-0,92	-1,89	0,02747	-0,93	-1,91	0,00124	---	LOC441046
8097626	-0,92	-1,89	0,04498	0,51	1,42	0,37896	NR_003675	OR1C1
7925732	-0,92	-1,89	0,13575	-2,32	-5,00	0,02086	NM_012353	MS4A3
7940216	-0,92	-1,89	0,01313	-1,12	-2,17	0,00004	NM_006138	CHCHD10
8074939	-0,92	-1,89	0,00766	1,24	2,36	0,01443	NM_213720	TAS2R42
7961298	-0,92	-1,89	0,06343	-0,71	-1,63	0,00380	NM_181429	TEX12
7943859	-0,92	-1,89	0,02377	-1,01	-2,02	0,00086	NM_031275	SPRR2B
7920210	-0,92	-1,89	0,16228	-1,55	-2,94	0,02145	NM_001017418	OR2Z1
8025450	-0,92	-1,89	0,04042	-1,22	-2,33	0,00558	NM_001004699	---
8173581	-0,92	-1,89	0,10490	-0,89	-1,85	0,00081	---	---
7942071	-0,92	-1,89	0,17085	-1,00	-2,00	0,09245	---	WDR85
8165610	-0,92	-1,89	0,23290	0,75	1,68	0,02030	NM_138778	SCGB1D4
7948662	-0,92	-1,89	0,00284	-1,08	-2,11	0,01525	NM_206998	KRTAP4-4
8015236	-0,92	-1,89	0,43873	-2,26	-4,80	0,02274	NM_032524	---
8169291	-0,92	-1,89	0,03963	-0,35	-1,27	0,11955	---	SPANXB1
8170241	-0,92	-1,89	0,01887	-1,22	-2,33	0,00010	NM_032461	SPANXB1
8170244	-0,92	-1,89	0,01887	-1,22	-2,33	0,00010	NM_032461	TNFRSF8

7897860	-0,92	-1,89	0,07245	-1,43	-2,69	0,00229	NM_001243	DAZ2
8177426	-0,92	-1,89	0,02453	-1,31	-2,49	0,00088	NM_020363	---
8138086	-0,92	-1,89	0,07336	-1,08	-2,11	0,00001	---	UNC5D
8145799	-0,92	-1,89	0,10123	-1,29	-2,45	0,00036	NM_080872	---
7991171	-0,92	-1,89	0,05617	-1,30	-2,46	0,00108	---	C14orf23
7973748	-0,92	-1,89	0,03854	-0,97	-1,96	0,00436	NR_026731	PRKCQ
7931930	-0,92	-1,89	0,01635	1,44	2,72	0,02992	NM_006257	AMTN
8095508	-0,92	-1,89	0,00054	-1,21	-2,31	0,00519	NM_212557	---
8160661	-0,92	-1,89	0,11187	-1,18	-2,26	0,00102	---	C2orf66
8057954	-0,92	-1,89	0,03737	-2,27	-4,81	0,00048	AY358249	---
8047370	-0,92	-1,89	0,01013	-0,85	-1,80	0,03069	---	---
7912196	-0,92	-1,89	0,01496	-0,96	-1,95	0,00481	---	KLRB1
7961059	-0,92	-1,89	0,07569	-1,20	-2,30	0,00001	NM_002258	GPA33
7922029	-0,92	-1,89	0,00765	-0,85	-1,81	0,01820	NM_005814	---
7907308	-0,92	-1,89	0,15913	-1,63	-3,09	0,00250	---	AP1M2
8034084	-0,92	-1,89	0,07082	1,59	3,02	0,00357	NM_005498	KCNK10
7980605	-0,92	-1,89	0,02896	-0,74	-1,67	0,06017	NM_021161	---
8129087	-0,92	-1,89	0,03935	-1,47	-2,77	0,01376	---	FCRLA
7906786	-0,92	-1,89	0,02265	-1,21	-2,31	0,01198	NM_032738	GH1
8017505	-0,92	-1,89	0,00999	-1,21	-2,31	0,00011	NM_000515	---
7966594	-0,92	-1,89	0,04840	-1,11	-2,15	0,01647	---	---
8121130	-0,92	-1,89	0,14274	-0,17	-1,12	0,12392	---	FLJ37786
7905018	-0,92	-1,89	0,16723	-1,70	-3,26	0,00197	AK095105	OR13D1
8157016	-0,92	-1,89	0,00598	-0,71	-1,63	0,14456	NM_001004484	---
7916582	-0,92	-1,89	0,11568	-0,61	-1,53	0,00261	---	---
7923317	-0,92	-1,89	0,07325	-1,19	-2,27	0,02614	---	MIR196A1
8016482	-0,92	-1,89	0,20764	-0,41	-1,33	0,16470	NR_029582	DNAH6
8043043	-0,92	-1,89	0,04384	-1,05	-2,07	0,01558	NM_001370	TUBB2B
8123651	-0,92	-1,89	0,11888	-1,16	-2,24	0,00583	NM_178012	C1orf125
7907719	-0,92	-1,89	0,03940	-0,62	-1,53	0,02190	NM_144696	ADRA1A
8149885	-0,92	-1,89	0,16458	-0,79	-1,73	0,00197	NM_000680	LRRC46
8008040	-0,92	-1,89	0,03662	-0,74	-1,67	0,26055	NM_033413	RASA4
8141768	-0,92	-1,89	0,17692	-0,79	-1,73	0,09979	NM_006989	OR52A4
7946021	-0,92	-1,89	0,07052	-0,88	-1,85	0,04651	NM_001005222	ADAMTSL2
8159086	-0,92	-1,89	0,06991	-2,23	-4,68	0,00888	NM_014694	PTGER4
8105067	-0,92	-1,89	0,00560	0,04	1,03	0,88606	NM_000958	TRIM15
8117849	-0,92	-1,89	0,03237	-1,17	-2,26	0,00377	NM_033229	TRIM15
8177760	-0,92	-1,89	0,03237	-1,17	-2,26	0,00377	NM_033229	TRIM15
8179070	-0,92	-1,89	0,03237	-1,17	-2,26	0,00377	NM_033229	---
8135943	-0,92	-1,89	0,30523	-2,05	-4,14	0,00270	---	---
8146157	-0,92	-1,89	0,16358	-0,94	-1,91	0,11648	---	PADI1
7898413	-0,92	-1,89	0,01250	-0,74	-1,67	0,11611	NM_013358	SPRYD5
7939954	-0,92	-1,89	0,18989	-0,28	-1,21	0,04310	NM_032681	---
7973303	-0,92	-1,89	0,24907	-2,05	-4,14	0,00578	---	---
7911211	-0,92	-1,89	0,32651	-0,89	-1,85	0,00631	---	PTP4A3
8148501	-0,92	-1,89	0,11339	1,29	2,44	0,09836	NM_032611	IBSP
8096281	-0,92	-1,89	0,12374	-0,94	-1,92	0,00016	NM_004967	LEKR1
8083576	-0,92	-1,89	0,01750	-0,70	-1,63	0,04080	NM_001004316	REXO1L1
8151609	-0,92	-1,89	0,60224	-5,31	-39,80	0,00005	NM_172239	SCARNA22
8093576	-0,92	-1,89	0,08947	-0,89	-1,85	0,02911	NR_003004	NPHS1
8036176	-0,92	-1,89	0,02447	-1,59	-3,00	0,00137	NM_004646	KSR2
7966810	-0,92	-1,89	0,01999	-0,37	-1,29	0,06661	NM_173598	KRTAP19-1
8069822	-0,92	-1,89	0,16075	-1,84	-3,59	0,00021	NM_181607	TICAM1
8032899	-0,92	-1,89	0,09489	1,22	2,32	0,00943	NM_182919	---
7925976	-0,92	-1,89	0,01948	-1,94	-3,84	0,01004	---	---
8068020	-0,92	-1,89	0,03892	-1,23	-2,34	0,00264	---	KAZ
7912629	-0,92	-1,89	0,04170	-0,94	-1,92	0,01306	NM_201628	---
7978534	-0,92	-1,89	0,07096	-0,56	-1,48	0,03663	---	FER1L5
8043632	-0,92	-1,89	0,16880	-1,58	-2,99	0,02725	NM_001113382	LOC100129150
8144526	-0,92	-1,89	0,26483	-2,63	-6,20	0,01316	ENST00000416972	---
7906948	-0,92	-1,89	0,08801	-0,93	-1,91	0,26566	---	TBC1D28
8013294	-0,92	-1,89	0,14800	-0,74	-1,67	0,03103	NM_001039397	USP17L2
8144397	-0,92	-1,89	0,57483	-2,28	-4,84	0,00150	NM_201402	USP17L2
8149241	-0,92	-1,89	0,57483	-2,28	-4,84	0,00150	NM_201402	QRFPR
8102608	-0,92	-1,89	0,04882	-1,22	-2,32	0,04893	NM_198179	ZNF574
8029182	-0,92	-1,89	0,00799	0,83	1,77	0,01715	NM_022752	TBX22
8168534	-0,92	-1,89	0,03905	-1,30	-2,46	0,00726	NM_016954	CSAG2
8175710	-0,92	-1,89	0,01873	-0,82	-1,76	0,00336	NM_001080848	---
7929589	-0,92	-1,89	0,02134	-1,40	-2,64	0,02653	---	OR8K5
7948121	-0,92	-1,89	0,25892	-1,08	-2,12	0,03711	NM_001004058	HPD
7967212	-0,92	-1,89	0,09728	-1,22	-2,33	0,01243	NM_002150	---
8168026	-0,92	-1,90	0,24011	-1,45	-2,72	0,00294	---	GPR123
7931500	-0,92	-1,90	0,05737	-0,91	-1,88	0,01670	NM_001083909	LOC100133469

7977447	-0,92	-1,90	0,09965	-1,03	-2,04	0,01097	NR_027457		SNORD56B
7975453	-0,92	-1,90	0,04460	-0,93	-1,91	0,01370	NR_001276		---
8161431	-0,92	-1,90	0,11066	-0,97	-1,96	0,07948	---	---	
8061414	-0,92	-1,90	0,22605	-0,44	-1,36	0,01623	---	ZFYVE28	
8099008	-0,92	-1,90	0,01253	-0,60	-1,52	0,20325	NM_020972		AKR1D1
8136459	-0,92	-1,90	0,00378	-1,17	-2,25	0,00031	NM_005989		---
7938070	-0,92	-1,90	0,00381	-1,29	-2,45	0,08862	---	ZNF283	
8029321	-0,92	-1,90	0,14005	-0,15	-1,11	0,58517	NM_181845		SNORD115-40
7982086	-0,92	-1,90	0,11945	-1,54	-2,91	0,00168	NR_003355		---
7961624	-0,92	-1,90	0,01279	-1,29	-2,44	0,00037	---	LYPD4	
8037061	-0,92	-1,90	0,09680	-1,53	-2,89	0,00933	NM_173506		YIPF7
8100076	-0,92	-1,90	0,24461	-1,18	-2,27	0,00084	NM_182592		---
8169348	-0,92	-1,90	0,01146	-1,38	-2,60	0,00535	---	NOTUM	
8019334	-0,92	-1,90	0,04949	0,31	1,24	0,34740	NM_178493		FAM75A7
8155340	-0,92	-1,90	0,12454	-1,65	-3,15	0,00312	NM_015667		---
8140532	-0,92	-1,90	0,03639	-1,45	-2,74	0,07264	---	MYL4	
8007921	-0,92	-1,90	0,00820	-0,95	-1,93	0,17778	NM_001002841		---
7986836	-0,92	-1,90	0,13267	-0,55	-1,47	0,16078	---	RNF31	
7973613	-0,92	-1,90	0,02310	-0,32	-1,25	0,39065	NM_017999		C1QTNF1
8010287	-0,92	-1,90	0,10191	-0,99	-1,98	0,08012	NM_030968		ACTN3
7941662	-0,92	-1,90	0,07825	-0,88	-1,85	0,04534	NM_001104		PPY
8015884	-0,92	-1,90	0,04143	-1,61	-3,06	0,00187	NM_002722		C17orf59
8004802	-0,92	-1,90	0,09583	-1,43	-2,69	0,01166	NM_017622		AASDHPPT
7951435	-0,92	-1,90	0,23065	-0,82	-1,77	0,01323	NM_015423		---
7984215	-0,92	-1,90	0,00868	-0,95	-1,93	0,03399	---	psiTPTE22	
8071061	-0,92	-1,90	0,35197	-3,60	-12,10	0,00086	AK097082	ZSCAN2	
7985577	-0,92	-1,90	0,08309	-0,15	-1,11	0,45106	NM_181877		RAPGEF3
7962625	-0,92	-1,90	0,12889	-1,19	-2,28	0,06901	NM_001098531		SERPINB11
8021614	-0,92	-1,90	0,04783	-0,41	-1,33	0,00820	NM_080475		---
7921220	-0,92	-1,90	0,04584	-0,94	-1,91	0,01666	---	---	
8016412	-0,92	-1,90	0,02401	-1,77	-3,42	0,00400	---	FLJ33534	
8050293	-0,92	-1,90	0,03332	-1,70	-3,24	0,00009	AK090853	---	
8112888	-0,92	-1,90	0,10337	-1,12	-2,17	0,10836	---	---	
8007139	-0,93	-1,90	0,08980	-0,74	-1,67	0,01092	---	SNORA64	
7998666	-0,93	-1,90	0,08582	-0,36	-1,28	0,43680	NR_002326		---
8078310	-0,93	-1,90	0,00719	-0,77	-1,70	0,03011	---	DUS3L	
8033025	-0,93	-1,90	0,00334	0,92	1,89	0,14714	NM_020175		TSPO2
8119396	-0,93	-1,90	0,03677	-1,56	-2,94	0,00799	NM_001010873		CCR8
8078888	-0,93	-1,90	0,07147	-0,98	-1,98	0,02771	NM_005201		---
8097505	-0,93	-1,90	0,06649	-1,72	-3,29	0,00235	---	KMO	
7910950	-0,93	-1,90	0,00066	-0,82	-1,77	0,00641	NM_003679		---
7985037	-0,93	-1,90	0,08109	-0,84	-1,79	0,00110	---	OR10G7	
7952388	-0,93	-1,90	0,18144	-1,43	-2,70	0,00366	NM_001004463		MCF2L2
8092358	-0,93	-1,90	0,00905	0,20	1,15	0,42306	NM_015078		---
8073941	-0,93	-1,90	0,14350	-1,65	-3,15	0,00102	---	COL16A1	
7914361	-0,93	-1,90	0,14101	-2,16	-4,47	0,00043	NM_001856		CEACAM19
8029446	-0,93	-1,90	0,21733	-1,22	-2,33	0,00263	NM_001127893		FRAT1
7929616	-0,93	-1,90	0,02280	-0,10	-1,07	0,50235	NM_005479		FADS6
8018251	-0,93	-1,90	0,13620	-2,23	-4,68	0,00003	NM_178128		LOC100129318
7992996	-0,93	-1,90	0,01548	-0,93	-1,91	0,14390	AY358225	TTTY9A	
8176692	-0,93	-1,90	0,03000	-1,87	-3,66	0,00054	NR_001530		TTTY9A
8177195	-0,93	-1,90	0,03000	-1,87	-3,66	0,00054	NR_001530		OR6K6
7906374	-0,93	-1,90	0,02029	-1,60	-3,04	0,01057	NM_001005184		---
8149833	-0,93	-1,90	0,08016	-2,13	-4,38	0,00132	---	---	
8047536	-0,93	-1,90	0,04488	-0,65	-1,57	0,06443	---	TACR1	
8053266	-0,93	-1,90	0,03054	-1,59	-3,00	0,00517	NM_001058		---
7899849	-0,93	-1,90	0,08068	-0,83	-1,78	0,02582	---	C4orf33	
8097435	-0,93	-1,90	0,13425	1,17	2,25	0,02408	NM_173487		WDR24
7998280	-0,93	-1,90	0,05232	0,10	1,07	0,77130	NM_032259		MYBPC3
7947828	-0,93	-1,90	0,07716	-1,61	-3,05	0,00078	NM_000256		NBPF4
7903478	-0,93	-1,90	0,04082	-1,42	-2,68	0,00320	NM_001143989		CD52
7899160	-0,93	-1,90	0,13768	-0,35	-1,27	0,30376	NM_001803		---
8073631	-0,93	-1,90	0,00693	-1,00	-2,01	0,00560	---	KLHL31	
8120277	-0,93	-1,90	0,06419	-0,81	-1,76	0,00975	ENST00000407079		LOC347376
8167640	-0,93	-1,90	0,05395	-1,12	-2,17	0,00624	NR_003933		---
8151888	-0,93	-1,90	0,00669	-1,38	-2,61	0,04388	---	LOC145757	
7986444	-0,93	-1,90	0,01455	1,18	2,26	0,01591	AK056534	SLC6A16	
8038326	-0,93	-1,90	0,02294	1,10	2,14	0,00068	NM_014037		CATSPER4
7899124	-0,93	-1,90	0,00906	-0,84	-1,79	0,01620	NM_198137		C12orf56
7964687	-0,93	-1,90	0,00699	-0,43	-1,35	0,29402	NM_001170633		C21orf15
8069505	-0,93	-1,90	0,35398	-1,02	-2,03	0,00795	BX648100	---	
8137524	-0,93	-1,90	0,00114	-1,25	-2,37	0,02772	---	C11orf64	
7940315	-0,93	-1,90	0,00725	-1,41	-2,66	0,00168	NR_026946		---

8119159	-0,93	-1,90	0,11972	-1,08	-2,11	0,08005	---	---
8019561	-0,93	-1,90	0,10477	-0,84	-1,80	0,00463	---	MIR30B
8153065	-0,93	-1,90	0,03077	-0,88	-1,85	0,05636	NR_029666	MAST1
8026063	-0,93	-1,90	0,02413	-0,14	-1,10	0,63044	NM_014975	PLXNA4
8142997	-0,93	-1,90	0,04540	-1,05	-2,07	0,02139	NM_020911	PRAMEF11
7912547	-0,93	-1,90	0,26338	-1,05	-2,06	0,00635	NM_001146344	FAM150B
8049990	-0,93	-1,90	0,15456	-2,47	-5,54	0,00151	NM_001002919	---
8038137	-0,93	-1,90	0,10251	-1,24	-2,35	0,06130	---	DNAH12
8088299	-0,93	-1,90	0,00102	-0,82	-1,77	0,01912	NM_178504	LOC400696
8028744	-0,93	-1,90	0,04440	-0,62	-1,53	0,10929	NM_207646	KCNA10
7918444	-0,93	-1,90	0,18567	-1,95	-3,86	0,00567	NM_005549	C3orf36
8090817	-0,93	-1,90	0,15471	-1,51	-2,85	0,00370	NM_025041	EPHX4
7902957	-0,93	-1,91	0,11858	1,34	2,52	0,00538	NM_173567	CNTNAP5
8044976	-0,93	-1,91	0,08800	-1,24	-2,35	0,00227	NM_130773	KRT38
8015287	-0,93	-1,91	0,00702	-0,57	-1,48	0,20125	NM_006771	KIAA0125
7977445	-0,93	-1,91	0,17774	-1,43	-2,69	0,06055	NR_026800	---
8120247	-0,93	-1,91	0,03628	-0,92	-1,90	0,00027	---	---
7975386	-0,93	-1,91	0,02603	-1,57	-2,97	0,00258	---	PRAMEF15
7912582	-0,93	-1,91	0,30856	-1,13	-2,19	0,09278	NM_001098376	---
8091998	-0,93	-1,91	0,11239	-1,86	-3,62	0,03842	---	---
8016431	-0,93	-1,91	0,09031	-1,28	-2,43	0,01815	---	B3GNT9
8001932	-0,93	-1,91	0,06152	-0,56	-1,47	0,36943	NM_033309	---
8102592	-0,93	-1,91	0,02418	-1,02	-2,03	0,03026	---	---
7955248	-0,93	-1,91	0,12087	-0,93	-1,90	0,00987	---	---
7924731	-0,93	-1,91	0,03182	-0,99	-1,98	0,00148	---	IL25
7973393	-0,93	-1,91	0,05374	-1,07	-2,10	0,00124	NM_022789	ATP2A1
7994463	-0,93	-1,91	0,01694	-0,80	-1,74	0,00906	NM_004320	MS4A5
7940279	-0,93	-1,91	0,04412	-0,67	-1,59	0,00053	NM_023945	RECQL5
8018400	-0,93	-1,91	0,04096	-0,39	-1,31	0,15688	NM_004259	GPR68
7980765	-0,93	-1,91	0,02306	-1,80	-3,48	0,00116	NM_003485	---
7900407	-0,93	-1,91	0,05237	-1,15	-2,21	0,00137	---	PSORS1C1
8177938	-0,93	-1,91	0,07501	0,99	1,99	0,10230	NM_014068	ID2B
8088480	-0,93	-1,91	0,00437	-0,75	-1,68	0,01127	NR_026582	---
7911331	-0,93	-1,91	0,21407	-1,82	-3,54	0,04991	---	---
7924463	-0,93	-1,91	0,21407	-1,82	-3,54	0,04991	---	---
7927089	-0,93	-1,91	0,21407	-1,82	-3,54	0,04991	---	---
7945347	-0,93	-1,91	0,21407	-1,82	-3,54	0,04991	---	---
7998115	-0,93	-1,91	0,21407	-1,82	-3,54	0,04991	---	---
8031997	-0,93	-1,91	0,21407	-1,82	-3,54	0,04991	---	---
8102530	-0,93	-1,91	0,21407	-1,82	-3,54	0,04991	---	---
8137668	-0,93	-1,91	0,21407	-1,82	-3,54	0,04991	---	---
7899071	-0,93	-1,91	0,03953	-0,98	-1,98	0,01520	---	---
8165993	-0,93	-1,91	0,01247	-0,93	-1,91	0,03993	---	---
7919578	-0,93	-1,91	0,29077	-3,11	-8,63	0,00122	---	POTEB
7986605	-0,93	-1,91	0,11711	-1,63	-3,09	0,03544	NM_207355	---
8043468	-0,93	-1,91	0,09237	-1,38	-2,61	0,00486	---	TRIM61
8103520	-0,93	-1,91	0,27442	-1,36	-2,57	0,02765	NM_001012414	CXorf18
8175537	-0,93	-1,91	0,40056	-1,17	-2,24	0,18120	AK093505	---
8107125	-0,93	-1,91	0,07383	-0,75	-1,68	0,12849	---	PLEK
8042391	-0,93	-1,91	0,03423	-0,80	-1,74	0,01586	NM_002664	FGFRL1
8093440	-0,93	-1,91	0,21620	-0,99	-1,99	0,24752	NM_001004356	PAPLN
7975562	-0,93	-1,91	0,14296	-0,50	-1,41	0,44670	NM_173462	---
7999884	-0,93	-1,91	0,08225	-0,95	-1,93	0,02247	---	C21orf90
8069135	-0,93	-1,91	0,07550	-1,38	-2,59	0,07660	NR_026547	---
8099130	-0,93	-1,91	0,17744	-1,33	-2,52	0,00002	---	TRAT1
8081537	-0,93	-1,91	0,00429	-0,81	-1,75	0,03456	NM_016388	RAC3
8010737	-0,93	-1,91	0,06070	0,48	1,40	0,25399	NM_005052	---
8173823	-0,93	-1,91	0,04055	-0,94	-1,92	0,01501	---	TMOD4
7919929	-0,93	-1,91	0,01739	-1,22	-2,34	0,06394	NM_013353	BPIL2
8075600	-0,93	-1,91	0,00974	-1,11	-2,16	0,00201	NM_174932	---
8151279	-0,93	-1,91	0,00434	-1,50	-2,83	0,00009	---	---
8088634	-0,93	-1,91	0,04401	-0,98	-1,97	0,00580	---	NCRNA00207
8073698	-0,93	-1,91	0,03947	-1,21	-2,31	0,00536	NR_028409	---
8175589	-0,93	-1,91	0,08578	-0,84	-1,79	0,08317	---	---
8040465	-0,93	-1,91	0,09232	-1,41	-2,66	0,03538	---	---
8138043	-0,93	-1,91	0,04390	-0,77	-1,71	0,07873	---	ZNF503
7928529	-0,93	-1,91	0,13104	-1,72	-3,29	0,05493	NM_032772	PIK3CG
8135363	-0,93	-1,91	0,01522	-1,84	-3,59	0,00373	NM_002649	MAGEA5
8175685	-0,93	-1,91	0,05991	-1,37	-2,59	0,00634	NM_021049	---
8022281	-0,93	-1,91	0,00251	-0,98	-1,98	0,09937	---	ITPA
8060611	-0,93	-1,91	0,04027	0,41	1,33	0,20107	NM_033453	C15orf51
7986426	-0,93	-1,91	0,12848	-1,90	-3,73	0,02765	NR_003260	FAM19A3
7904073	-0,93	-1,91	0,00909	-1,03	-2,04	0,01754	NM_001004440	TMEM145

8029219	-0,93	-1,91	0,02217	0,25	1,19	0,50856	NM_173633		RAP1GAP
7913385	-0,93	-1,91	0,06944	-0,32	-1,24	0,49436	NM_001145657		OR5D3P
7939936	-0,94	-1,91	0,08101	-1,72	-3,28	0,00507	ENST00000333984		---
7975813	-0,94	-1,91	0,03059	-1,43	-2,70	0,01437	---	GLT1D1	
7959834	-0,94	-1,91	0,09543	-0,90	-1,86	0,03809	NM_144669		---
8151234	-0,94	-1,91	0,07712	-1,63	-3,09	0,00083	---	GC	
8100853	-0,94	-1,91	0,09327	-0,88	-1,84	0,01558	NM_000583		TNNT2
7923332	-0,94	-1,91	0,11234	-0,08	-1,06	0,76540	NM_000364		---
8095251	-0,94	-1,91	0,00709	-0,50	-1,42	0,24369	---	GOLGA9P	
7982350	-0,94	-1,91	0,34561	-0,89	-1,86	0,04075	NR_024074		GOLGA9P
7987139	-0,94	-1,91	0,34561	-0,89	-1,86	0,04075	NR_024074		SARDH
8164940	-0,94	-1,91	0,03478	-1,62	-3,08	0,00007	NM_001134707		DHX34
8029918	-0,94	-1,91	0,04240	0,65	1,57	0,30105	NM_014681		FLI1
7945132	-0,94	-1,91	0,00478	-1,26	-2,39	0,00216	NM_002017		MS4A6E
7940253	-0,94	-1,91	0,09282	-0,90	-1,86	0,09684	NM_139249		ZNF157
8167152	-0,94	-1,91	0,08261	-1,76	-3,38	0,00537	NM_003446		PGP
7998759	-0,94	-1,91	0,03698	0,16	1,12	0,54152	NM_001042371		SIRPD
8064464	-0,94	-1,91	0,02218	-1,26	-2,40	0,00456	NM_178460		LRRC16B
7973468	-0,94	-1,91	0,02186	-0,91	-1,88	0,11209	NM_138360		MAMDC2
8155754	-0,94	-1,91	0,01502	-0,85	-1,80	0,04405	NM_153267		OK/SW-CL.58
7970828	-0,94	-1,91	0,15217	-1,70	-3,24	0,09600	AB064667	---	
7945226	-0,94	-1,91	0,02186	-1,36	-2,57	0,00430	---	---	
8166498	-0,94	-1,91	0,04365	-1,08	-2,12	0,00441	---	ZNF485	
7927173	-0,94	-1,91	0,07205	0,37	1,29	0,66759	NM_145312		FAM164C
7975772	-0,94	-1,91	0,03214	-0,28	-1,22	0,08009	NM_024643		GRM6
8116253	-0,94	-1,91	0,09065	-1,08	-2,11	0,02090	NM_000843		RHOV
7987574	-0,94	-1,91	0,12004	0,06	1,04	0,89554	NM_133639		CR1L
7909390	-0,94	-1,91	0,33403	-0,71	-1,64	0,00153	NM_175710		SNORA5A
8139482	-0,94	-1,91	0,05508	-0,30	-1,23	0,28037	NR_002919		C1orf172
7914003	-0,94	-1,92	0,05524	1,81	3,51	0,00023	NM_152365		RDH13
8039306	-0,94	-1,92	0,00151	-0,07	-1,05	0,84721	NM_001145971		---
8091456	-0,94	-1,92	0,07195	-1,76	-3,39	0,04127	---	---	
8106763	-0,94	-1,92	0,05938	-1,16	-2,24	0,00130	---	ACRBP	
7960642	-0,94	-1,92	0,04458	-0,95	-1,93	0,02922	NM_032489		CDKN1C
7945767	-0,94	-1,92	0,14175	-1,11	-2,15	0,13901	NM_000076		RGL3
8034227	-0,94	-1,92	0,03249	1,03	2,05	0,05420	NM_001161616		GSTA5
8127079	-0,94	-1,92	0,15062	-1,09	-2,13	0,07434	NM_153699		SLC30A8
8148003	-0,94	-1,92	0,00410	-0,88	-1,84	0,01656	NM_001172813		MIR103-2
8060734	-0,94	-1,92	0,04491	-0,58	-1,49	0,04797	NR_029519		ID4
8117120	-0,94	-1,92	0,00315	-2,09	-4,25	0,00101	NM_001546		CCRL2
8079407	-0,94	-1,92	0,10992	-1,10	-2,14	0,04702	NM_003965		C21orf2
8070744	-0,94	-1,92	0,03429	-1,28	-2,43	0,04308	NM_004928		---
8118979	-0,94	-1,92	0,02302	-0,47	-1,38	0,02584	---	LOC51152	
7904478	-0,94	-1,92	0,64523	-4,62	-24,55	0,00040	AF172850	TMEM202	
7984635	-0,94	-1,92	0,01617	-1,01	-2,02	0,00541	NM_001080462		---
7958017	-0,94	-1,92	0,05206	-0,84	-1,79	0,00012	---	---	
7913972	-0,94	-1,92	0,15492	-0,98	-1,97	0,00517	---	PKD1	
7998700	-0,94	-1,92	0,00185	0,08	1,06	0,89325	NM_001009944		LOC100129831
8024444	-0,94	-1,92	0,01329	-0,98	-1,98	0,05970	AY358234	LRRC61	
8137215	-0,94	-1,92	0,00025	0,57	1,48	0,24795	NM_001142928		---
7961593	-0,94	-1,92	0,01360	-0,87	-1,82	0,06356	---	---	
8173977	-0,94	-1,92	0,05179	-1,06	-2,08	0,00891	---	---	
7944767	-0,94	-1,92	0,03784	-1,21	-2,31	0,00784	---	HIST1H2AM	
8124540	-0,94	-1,92	0,00383	-0,17	-1,12	0,65192	NM_003514		---
8065200	-0,94	-1,92	0,04878	-0,54	-1,45	0,00165	---	CCT8L2	
8074196	-0,94	-1,92	0,17696	-1,00	-2,01	0,05651	NM_014406		PTCHD1
8166447	-0,94	-1,92	0,17482	-1,17	-2,25	0,02387	NM_173495		PLCZ1
7961604	-0,94	-1,92	0,04523	-0,52	-1,43	0,00292	NM_033123		ACE
8009096	-0,94	-1,92	0,00255	-0,86	-1,82	0,07405	NM_000789		RIT2
8022976	-0,94	-1,92	0,00353	-0,99	-1,98	0,07867	NM_002930		LOC100129503
8010560	-0,94	-1,92	0,02519	-0,07	-1,05	0,85467	AF258550	OR51B4	
7946067	-0,94	-1,92	0,06854	-1,47	-2,77	0,00222	NM_033179		VN1R2
8030972	-0,94	-1,92	0,02322	-1,23	-2,34	0,00323	NM_173856		NARFL
7998336	-0,94	-1,92	0,00562	-0,59	-1,51	0,35585	NM_022493		---
8121747	-0,94	-1,92	0,01177	-1,14	-2,20	0,04132	---	---	
8038960	-0,94	-1,92	0,09759	-1,14	-2,20	0,01568	---	LOC100128292	
7928545	-0,94	-1,92	0,05569	-0,45	-1,37	0,30530	NR_024585		SPINK6
8109044	-0,94	-1,92	0,04150	-0,94	-1,92	0,00542	NM_205841		HS3ST3A1
8012883	-0,94	-1,92	0,08555	-1,75	-3,37	0,00245	NM_006042		CASZ1
7912316	-0,94	-1,92	0,06986	0,21	1,15	0,31420	NM_001079843		LIN28A
7899167	-0,94	-1,92	0,06051	-1,13	-2,20	0,04741	NM_024674		OR2A7
8143633	-0,94	-1,92	0,13214	1,48	2,79	0,01045	NM_001005328		ZNF648
7922686	-0,94	-1,92	0,04431	-1,52	-2,87	0,00002	NM_001009992		---

8129311	-0,94	-1,92	0,02201	-1,15	-2,22	0,00630	---	FAM83C
8065880	-0,94	-1,92	0,06299	-1,20	-2,31	0,00061	NM_178468	XAGE3
8172806	-0,94	-1,92	0,08414	-0,73	-1,65	0,01666	NM_133179	NTN1
8004880	-0,94	-1,92	0,09663	-0,25	-1,19	0,56800	NM_004822	CRP
7921449	-0,94	-1,92	0,00659	-1,51	-2,85	0,00014	NM_000567	---
8039672	-0,94	-1,92	0,10391	-0,44	-1,35	0,00346	---	---
7954775	-0,94	-1,92	0,04546	-1,26	-2,40	0,00004	---	LOC729444
8074597	-0,94	-1,92	0,32340	-2,38	-5,20	0,00158	AK302783	GIMAP2
8137250	-0,94	-1,92	0,03452	-1,12	-2,17	0,01283	NM_015660	TRPV1
8011375	-0,94	-1,92	0,02488	-0,39	-1,31	0,44612	NM_080704	DKFZp434F142
8133112	-0,94	-1,92	0,24657	-0,44	-1,35	0,15072	AL136837	EPHB1
8082846	-0,94	-1,92	0,05044	-0,73	-1,65	0,00379	NM_004441	LOC439914
7941059	-0,94	-1,92	0,07629	-1,37	-2,59	0,00165	ENST00000316124	KIR3DL2
8031328	-0,94	-1,92	0,15396	-1,17	-2,25	0,00174	NM_006737	KCNH8
8078196	-0,94	-1,92	0,05086	-1,25	-2,37	0,00113	NM_144633	FAM71D
7975191	-0,94	-1,92	0,19287	-0,54	-1,45	0,18468	NM_173526	HK3
8115957	-0,94	-1,92	0,00260	-0,54	-1,46	0,08143	NM_002115	SLC17A3
8124351	-0,94	-1,92	0,00833	-0,46	-1,38	0,01761	NM_001098486	LPL
8144917	-0,94	-1,92	0,06387	-1,39	-2,62	0,00486	NM_000237	---
8023310	-0,94	-1,92	0,00105	-1,52	-2,86	0,00542	---	MIR199A2
7922328	-0,94	-1,92	0,03483	-1,17	-2,25	0,06661	NR_029618	HSF4
7996430	-0,94	-1,92	0,03591	0,13	1,09	0,73439	NM_001040667	---
8010963	-0,94	-1,92	0,00102	-1,03	-2,04	0,02024	---	---
7945950	-0,94	-1,92	0,01998	-0,47	-1,39	0,20633	---	C20orf103
8060940	-0,94	-1,92	0,03139	-1,37	-2,59	0,01529	NM_012261	GPATCH3
7913990	-0,94	-1,92	0,16924	-1,07	-2,10	0,00317	NM_022078	CCHCR1
8124868	-0,94	-1,92	0,00908	-0,81	-1,75	0,01925	NM_001105564	C11orf87
7943711	-0,94	-1,92	0,12286	-1,72	-3,29	0,02587	NM_207645	FRMPD1
8155284	-0,94	-1,92	0,04460	-0,92	-1,89	0,02163	NM_014907	C16orf89
7999291	-0,94	-1,92	0,04838	-0,79	-1,73	0,04613	NM_152459	LOC93432
8136709	-0,94	-1,92	0,10003	-1,06	-2,09	0,01378	NR_003715	UCP3
7950321	-0,94	-1,92	0,07325	-0,96	-1,94	0,00074	NM_003356	FLJ40504
8013574	-0,94	-1,92	0,12015	-0,23	-1,17	0,34799	NR_028334	LRRC14B
8104163	-0,94	-1,92	0,08655	-0,34	-1,27	0,41481	NM_001080478	TEKT5
7999396	-0,94	-1,92	0,01371	-0,22	-1,17	0,56456	NM_144674	---
8115366	-0,94	-1,92	0,17701	-1,08	-2,11	0,00842	---	MTRF1L
8098705	-0,94	-1,92	0,03793	-0,51	-1,42	0,25947	NM_019041	HSD3B2
7904408	-0,94	-1,92	0,05988	-1,74	-3,34	0,00022	NM_000198	TPRX1
8037947	-0,94	-1,92	0,11998	-0,71	-1,64	0,17459	NM_198479	CCL19
8160879	-0,94	-1,92	0,07935	-1,30	-2,47	0,01693	NM_006274	AGBL3
8136370	-0,94	-1,93	0,03317	-0,84	-1,79	0,01234	NM_178563	---
7972565	-0,94	-1,93	0,02986	-0,85	-1,81	0,01815	---	MCF2
8175457	-0,95	-1,93	0,00842	-0,97	-1,96	0,09695	NM_001171876	RGS7
7925457	-0,95	-1,93	0,01135	-1,64	-3,11	0,00544	NM_002924	---
8045793	-0,95	-1,93	0,00927	-1,20	-2,30	0,01736	---	RIMBP3C
8074769	-0,95	-1,93	0,01444	-0,25	-1,19	0,53110	ENST00000331505	---
7917670	-0,95	-1,93	0,07910	-1,24	-2,37	0,01043	---	ZNF847P
7924821	-0,95	-1,93	0,22020	-1,25	-2,38	0,07002	AF387613	HMG20B
8024660	-0,95	-1,93	0,03268	0,86	1,82	0,28879	NM_006339	PRG3
7948213	-0,95	-1,93	0,13443	-1,07	-2,09	0,05038	NM_006093	---
8091676	-0,95	-1,93	0,02322	-0,81	-1,76	0,17239	---	---
7969370	-0,95	-1,93	0,13645	-0,72	-1,65	0,10921	---	CHAD
8016718	-0,95	-1,93	0,01172	-1,14	-2,20	0,04696	NM_001267	CCRN4L
8097461	-0,95	-1,93	0,02860	0,12	1,09	0,74914	NM_012118	---
8010210	-0,95	-1,93	0,00486	-1,36	-2,56	0,00385	---	SGK223
8149250	-0,95	-1,93	0,04099	1,05	2,08	0,01532	NM_001080826	TNNT3
7937749	-0,95	-1,93	0,25672	-1,50	-2,84	0,03513	NM_006757	---
8119896	-0,95	-1,93	0,09052	-0,59	-1,51	0,08133	---	---
7946676	-0,95	-1,93	0,23451	-1,60	-3,04	0,07723	---	---
7902615	-0,95	-1,93	0,07969	-0,85	-1,80	0,01519	---	RPS6KL1
7980219	-0,95	-1,93	0,03241	0,06	1,04	0,82963	NM_031464	---
8140530	-0,95	-1,93	0,03302	-1,21	-2,31	0,00178	---	GRM7
8077472	-0,95	-1,93	0,16030	-1,29	-2,45	0,01558	NM_181874	GPR32
8030731	-0,95	-1,93	0,46602	-0,99	-1,99	0,00904	NM_001506	MIR101-2
8154176	-0,95	-1,93	0,08459	-1,10	-2,14	0,01351	NR_029836	LOC100130876
8010978	-0,95	-1,93	0,41021	-2,01	-4,03	0,04546	AK130278	---
7984317	-0,95	-1,93	0,04772	-0,84	-1,79	0,00140	---	---
8016521	-0,95	-1,93	0,00664	-1,24	-2,35	0,00094	---	GGNBP1
8118726	-0,95	-1,93	0,05235	-1,32	-2,50	0,06825	NR_028361	OR6C2
7955999	-0,95	-1,93	0,00973	-1,37	-2,59	0,00430	NM_054105	---
7898721	-0,95	-1,93	0,21051	-1,01	-2,02	0,02359	---	GATA3
7926105	-0,95	-1,93	0,02168	-1,31	-2,49	0,04345	NM_001002295	ZDHHC19
8093074	-0,95	-1,93	0,08960	-1,62	-3,07	0,03339	NM_001039617	RPL13AP17

8133847	-0,95	-1,93	0,03297	-0,95	-1,94	0,00561	NR_003680	FCRL3
7921275	-0,95	-1,93	0,03275	-1,47	-2,77	0,01487	NM_052939	TMEM220
8012830	-0,95	-1,93	0,08322	-1,07	-2,09	0,00132	NM_001004313	ITIH1
8080445	-0,95	-1,93	0,02102	-0,81	-1,76	0,08858	NM_002215	SMOC1
7975390	-0,95	-1,93	0,17014	-0,01	-1,01	0,97419	NM_001034852	CPA3
8083260	-0,95	-1,93	0,10365	-1,07	-2,10	0,04185	NM_001870	KCNQ4
7900492	-0,95	-1,93	0,20751	-0,06	-1,04	0,90821	NM_004700	APOM
8118209	-0,95	-1,93	0,00011	-0,65	-1,57	0,18535	NM_019101	APOM
8178043	-0,95	-1,93	0,00011	-0,65	-1,57	0,18535	NM_019101	APOM
8179291	-0,95	-1,93	0,00011	-0,65	-1,57	0,18535	NM_019101	HABP2
7930561	-0,95	-1,93	0,03409	-1,95	-3,86	0,00008	NM_004132	CACNA2D2
8087691	-0,95	-1,93	0,04485	2,25	4,74	0,00889	NM_001005505	PRODH2
8036170	-0,95	-1,93	0,06462	-1,54	-2,90	0,00343	ENST00000443172	---
7951091	-0,95	-1,93	0,05097	-1,91	-3,75	0,03901	---	TKTL2
8103503	-0,95	-1,93	0,00897	-0,99	-1,98	0,00292	NM_032136	NWD1
8026587	-0,95	-1,93	0,04688	-0,05	-1,04	0,72285	NM_001007525	---
8066459	-0,95	-1,93	0,05213	0,11	1,08	0,54641	---	---
7922295	-0,95	-1,93	0,00252	-1,32	-2,50	0,03628	---	CHST2
8083214	-0,95	-1,93	0,04328	-1,78	-3,42	0,00400	NM_004267	LRRTM2
8114470	-0,95	-1,93	0,08710	-1,30	-2,46	0,03513	NM_015564	FLJ32742
8166585	-0,95	-1,93	0,08657	-2,26	-4,80	0,00100	AK057304	GSDMD
8148642	-0,95	-1,93	0,05853	-0,45	-1,36	0,48842	NM_024736	---
7938388	-0,95	-1,93	0,00915	-0,67	-1,59	0,08948	---	PCDHB1
8108679	-0,95	-1,93	0,00326	-0,71	-1,64	0,02699	NM_013340	OR10A2
7938176	-0,95	-1,93	0,22332	-1,85	-3,62	0,02055	NM_001004460	CD207
8052916	-0,95	-1,93	0,28117	-1,21	-2,31	0,00972	NM_015717	MRGPRG
7945857	-0,95	-1,93	0,13267	-1,06	-2,08	0,00215	NM_001164377	---
8146709	-0,95	-1,93	0,00901	-1,06	-2,09	0,02096	---	03. Sep
8073548	-0,95	-1,93	0,01657	3,67	12,73	0,00259	NM_019106	PAH
7965884	-0,95	-1,93	0,10876	-1,29	-2,45	0,00280	NM_000277	TRAM1L1
8102464	-0,95	-1,93	0,11132	-0,99	-1,98	0,00346	NM_152402	IQSEC2
8172858	-0,95	-1,93	0,12298	-0,29	-1,23	0,45093	NM_001111125	WDR78
7916789	-0,95	-1,93	0,03267	-0,21	-1,16	0,24129	NM_024763	RNF112
8005586	-0,95	-1,93	0,00007	-0,41	-1,33	0,18639	NM_007148	TRIML2
8104107	-0,95	-1,93	0,01095	-0,83	-1,77	0,03783	NM_173553	WAS
8167334	-0,95	-1,93	0,13657	0,60	1,51	0,02371	NM_000377	CCDC17
7915743	-0,95	-1,93	0,12739	-0,94	-1,92	0,07586	NM_001114938	---
8084887	-0,95	-1,93	0,08506	-1,13	-2,19	0,00666	---	MEP1A
8120088	-0,95	-1,93	0,09748	-1,13	-2,19	0,00462	NM_005588	---
8098058	-0,95	-1,93	0,02498	-1,20	-2,30	0,00003	---	FLJ32955
8055682	-0,95	-1,93	0,09643	-1,18	-2,26	0,00107	AK057517	OTC
8166769	-0,95	-1,93	0,07217	-1,45	-2,74	0,00003	NM_000531	ADORA2A
8071899	-0,95	-1,93	0,06338	-0,21	-1,16	0,46226	NM_000675	UPF3A
7970370	-0,95	-1,93	0,42389	-1,09	-2,13	0,03760	NM_023011	MLC1
8076894	-0,95	-1,93	0,13492	-1,98	-3,94	0,03487	NM_015166	OR51J1
7938002	-0,95	-1,93	0,12864	-1,32	-2,50	0,02912	ENST00000332043	GLTPD1
7896917	-0,95	-1,93	0,09549	-0,68	-1,61	0,03123	NM_001029885	SPNS3
8003861	-0,95	-1,93	0,02696	-0,76	-1,69	0,12785	NM_182538	LOC646057
7986563	-0,95	-1,93	0,02006	-1,34	-2,53	0,02337	ENST00000451896	OR11H6
7973030	-0,95	-1,94	0,03871	-1,86	-3,62	0,01202	NM_001004480	C19orf59
8025285	-0,95	-1,94	0,03591	-0,60	-1,51	0,01630	NM_174918	SCGB3A1
8116439	-0,95	-1,94	0,07858	-1,19	-2,28	0,07541	NM_052863	---
7949672	-0,95	-1,94	0,04766	-1,74	-3,34	0,00636	---	MRPL23
7937763	-0,95	-1,94	0,10826	-1,93	-3,80	0,00064	NM_021134	EPS8L3
7918394	-0,95	-1,94	0,07706	-1,74	-3,35	0,00664	NM_139053	---
8026005	-0,95	-1,94	0,14934	-0,64	-1,56	0,19571	---	FLJ39632
7973002	-0,95	-1,94	0,05034	-1,47	-2,77	0,00177	AK096951	---
8040101	-0,95	-1,94	0,05454	-0,96	-1,95	0,00124	---	C7orf62
8140852	-0,95	-1,94	0,08132	-0,36	-1,28	0,03418	BC028365	---
7965677	-0,95	-1,94	0,11893	-0,94	-1,93	0,00768	---	HRNBP3
8019005	-0,95	-1,94	0,10661	-0,66	-1,58	0,10174	NM_001082575	PROC
8045018	-0,95	-1,94	0,04286	-0,47	-1,38	0,31581	NM_000312	FCGBP
8036787	-0,95	-1,94	0,09611	-0,80	-1,74	0,19970	NM_003890	C9orf62
8159239	-0,95	-1,94	0,01321	-1,57	-2,98	0,02728	BC034752	CD8A
8053584	-0,95	-1,94	0,13495	-1,57	-2,96	0,00114	NM_001768	---
8072960	-0,95	-1,94	0,00666	-2,38	-5,21	0,00015	---	PAFAH1B3
8037170	-0,95	-1,94	0,18078	2,50	5,66	0,02697	NM_002573	ITGAX
7995128	-0,95	-1,94	0,00306	-0,91	-1,88	0,09648	NM_000887	UBE2U
7901982	-0,95	-1,94	0,16444	-0,49	-1,40	0,09054	NM_152489	LOC644714
8161943	-0,95	-1,94	0,02775	-1,53	-2,88	0,07180	BC047037	---
8078284	-0,95	-1,94	0,02863	-1,47	-2,77	0,01060	---	CTNNA3
7933904	-0,95	-1,94	0,12859	-1,00	-1,99	0,12941	NM_013266	P2RY4
8173366	-0,95	-1,94	0,16416	-2,23	-4,70	0,00386	NM_002565	SCN11A

8086283	-0,95	-1,94	0,00324	-1,10	-2,15	0,00601	NM_014139		FPR3
8030866	-0,95	-1,94	0,00550	-1,14	-2,21	0,01032	NM_002030		RCSD1
7907079	-0,95	-1,94	0,10883	-1,59	-3,01	0,00231	NM_052862		---
7913663	-0,95	-1,94	0,03981	-1,54	-2,91	0,02025	---	---	
7925609	-0,95	-1,94	0,01445	-1,16	-2,23	0,03043	---	---	
7967702	-0,95	-1,94	0,26315	-1,09	-2,13	0,00151	---	CLCNKB	
7898314	-0,95	-1,94	0,02642	-1,20	-2,29	0,14385	NM_000085		LTB4R2
7973694	-0,96	-1,94	0,02848	-0,92	-1,89	0,03725	NM_001164692		SNORD61
8175432	-0,96	-1,94	0,00714	-1,21	-2,31	0,04549	NR_002735		---
8164848	-0,96	-1,94	0,05753	-1,50	-2,82	0,01225	---	NXF3	
8174207	-0,96	-1,94	0,04828	-0,66	-1,59	0,01543	NM_022052		RFX1
8034722	-0,96	-1,94	0,03862	-0,19	-1,14	0,56881	NM_002918		---
8073013	-0,96	-1,94	0,26546	-0,60	-1,52	0,04169	---	---	
7919560	-0,96	-1,94	0,14451	-1,83	-3,56	0,00183	---	CYP11A1	
7990333	-0,96	-1,94	0,15497	-0,66	-1,58	0,03982	NM_000781		---
8093974	-0,96	-1,94	0,05659	-1,14	-2,21	0,02631	---	SOX4	
8117165	-0,96	-1,94	0,09127	-0,93	-1,91	0,06598	NM_003107		GSX2
8095074	-0,96	-1,94	0,30907	-1,56	-2,95	0,00097	NM_133267		C5orf47
8110068	-0,96	-1,94	0,08042	-0,71	-1,64	0,01813	NM_001144954		KLHL6
8092392	-0,96	-1,94	0,08662	-0,92	-1,90	0,04006	NM_130446		OR8B4
7952402	-0,96	-1,94	0,14432	-1,11	-2,15	0,05778	NM_001005196		PSG9
8037290	-0,96	-1,94	0,04406	-2,63	-6,19	0,01006	NM_002784		C21orf121
8068735	-0,96	-1,94	0,09476	-1,19	-2,28	0,00333	NR_027273		C14orf49
7981161	-0,96	-1,94	0,03916	-0,40	-1,32	0,46184	NM_152592		CNFN
8037179	-0,96	-1,94	0,00966	0,11	1,08	0,77973	NM_032488		---
7952396	-0,96	-1,94	0,12433	-0,81	-1,76	0,15074	---	---	
7957275	-0,96	-1,94	0,04518	-1,20	-2,30	0,04136	---	GLRA4	
8174263	-0,96	-1,94	0,03901	-1,11	-2,16	0,08023	NM_001024452		---
8005155	-0,96	-1,94	0,00932	-1,23	-2,35	0,00737	---	CXCR4	
8055465	-0,96	-1,94	0,00080	0,78	1,71	0,10223	NM_001008540		CD300LB
8018169	-0,96	-1,94	0,02449	-1,67	-3,19	0,01170	NM_174892		ZNF345
8028206	-0,96	-1,94	0,00917	-0,27	-1,20	0,48300	NM_003419		SULT4A1
8076644	-0,96	-1,94	0,02721	0,36	1,29	0,43318	NM_014351		KRT86
7955624	-0,96	-1,94	0,05428	-0,40	-1,32	0,39539	NM_002284		---
7933086	-0,96	-1,94	0,01292	-1,33	-2,52	0,00815	---	MAGEA8	
8170412	-0,96	-1,94	0,12101	-1,92	-3,79	0,00070	NM_001166400		SSPO
8137183	-0,96	-1,94	0,05861	-1,81	-3,52	0,00195	NM_198455		---
8097415	-0,96	-1,94	0,01004	-0,51	-1,42	0,03302	---	RXRA	
8159127	-0,96	-1,94	0,08429	1,70	3,25	0,02342	NM_002957		---
8119827	-0,96	-1,94	0,00546	-0,79	-1,73	0,00031	---	---	
7971373	-0,96	-1,94	0,05472	-0,77	-1,71	0,05769	---	---	
8104392	-0,96	-1,94	0,01578	-0,67	-1,60	0,08253	---	GTPBP5	
8063857	-0,96	-1,94	0,02487	0,62	1,54	0,20576	NM_015666		AP1S2
8171481	-0,96	-1,94	0,01264	-0,95	-1,93	0,00616	NM_003916		FAM82A1
8041519	-0,96	-1,94	0,11854	-0,29	-1,23	0,34570	NM_001170792		---
7982661	-0,96	-1,94	0,00809	-1,47	-2,78	0,00006	---	KIF19	
8009602	-0,96	-1,94	0,01276	-0,48	-1,39	0,31878	NM_153209		ITPRIPL1
8043597	-0,96	-1,94	0,35012	-1,33	-2,52	0,00150	NM_178495		PCSK4
8032232	-0,96	-1,94	0,00288	-0,99	-1,99	0,04473	NM_017573		ATP6V0E2
8137208	-0,96	-1,94	0,01816	0,14	1,10	0,54463	NM_145230		MAPK13
8119016	-0,96	-1,94	0,02530	1,74	3,35	0,00437	NM_002754		AFAP1
8094028	-0,96	-1,94	0,01835	1,00	2,00	0,01017	NM_198595		---
8166353	-0,96	-1,94	0,10963	-1,14	-2,20	0,01643	---	CCDC151	
8034249	-0,96	-1,94	0,14603	-0,75	-1,68	0,01006	NM_145045		KIAA1522
7899821	-0,96	-1,94	0,00177	0,69	1,62	0,22782	NM_020888		---
8119894	-0,96	-1,94	0,07660	-1,14	-2,20	0,00208	---	C19orf46	
8036232	-0,96	-1,94	0,11682	-0,38	-1,31	0,02334	NM_001039876		CYP27C1
8054967	-0,96	-1,94	0,05250	-0,38	-1,30	0,16823	NM_001001665		---
7930954	-0,96	-1,94	0,03846	-1,47	-2,77	0,02857	---	PTGES2	
8164362	-0,96	-1,94	0,05816	-0,41	-1,33	0,40969	NM_025072		PANK4
7911730	-0,96	-1,94	0,16886	0,24	1,18	0,52352	NM_018216		ATP13A5
8092769	-0,96	-1,94	0,01361	-0,88	-1,83	0,03802	NM_198505		RNU11
7899502	-0,96	-1,94	0,11309	-1,22	-2,33	0,02176	NR_004407		PGK2
8126916	-0,96	-1,94	0,08380	-1,19	-2,28	0,01184	NM_138733		GPHB5
7979548	-0,96	-1,94	0,26785	-1,96	-3,90	0,00417	NM_145171		DCD
7963845	-0,96	-1,94	0,06437	-1,59	-3,01	0,00452	NM_053283		---
8053710	-0,96	-1,94	0,08055	-1,17	-2,26	0,00260	---	MUC4	
8092978	-0,96	-1,94	0,00989	-0,94	-1,91	0,02643	NM_018406		HOXC5
7955887	-0,96	-1,94	0,04022	0,58	1,50	0,10193	NM_018953		---
8023448	-0,96	-1,94	0,02868	-0,88	-1,84	0,04698	---	OR52A1	
7946025	-0,96	-1,94	0,18625	-1,11	-2,15	0,01105	NM_012375		---
8083227	-0,96	-1,95	0,07732	-1,19	-2,28	0,00015	---	CYP1A2	
7984862	-0,96	-1,95	0,03009	-0,74	-1,67	0,00478	NM_000761		---

7965836	-0,96	-1,95	0,01204	-1,18	-2,27	0,00001	---	LIPH
8092541	-0,96	-1,95	0,08575	4,74	26,68	0,00008	NM_139248	TMC1
8155824	-0,96	-1,95	0,10539	-0,65	-1,57	0,00024	NM_138691	GRB14
8056327	-0,96	-1,95	0,00637	0,71	1,63	0,31221	NM_004490	POTED
8067844	-0,96	-1,95	0,03037	-0,97	-1,96	0,01801	NM_174981	MYLK3
8001163	-0,96	-1,95	0,01383	-1,21	-2,31	0,01186	NM_182493	---
8047172	-0,96	-1,95	0,00285	-1,16	-2,24	0,03978	---	09. Mrz
7956623	-0,96	-1,95	0,03005	1,14	2,21	0,00119	NM_138396	FGA
8103311	-0,96	-1,95	0,00898	0,94	1,92	0,01980	NM_000508	---
8080956	-0,96	-1,95	0,20159	-1,20	-2,30	0,00567	---	HPCA
7899841	-0,96	-1,95	0,24429	0,17	1,12	0,24250	NM_002143	---
8060368	-0,96	-1,95	0,05172	-1,20	-2,30	0,00106	---	KRTAP22-1
8068151	-0,96	-1,95	0,00564	-0,61	-1,52	0,07586	NM_181620	PAX1
8061357	-0,96	-1,95	0,08812	-1,39	-2,62	0,00856	NM_006192	---
8172030	-0,96	-1,95	0,10219	-1,59	-3,01	0,03274	---	SNORA62
8078918	-0,96	-1,95	0,31597	-0,76	-1,70	0,24611	NR_002324	WISP3
8121532	-0,96	-1,95	0,00321	-1,10	-2,15	0,00734	NM_198239	---
7922908	-0,96	-1,95	0,01011	-0,80	-1,75	0,01471	---	C1orf127
7912349	-0,96	-1,95	0,14783	-1,68	-3,21	0,06073	NM_001170754	SPTBN5
7987701	-0,96	-1,95	0,02609	-0,77	-1,71	0,00665	NM_016642	EMILIN2
8019912	-0,96	-1,95	0,04424	-1,07	-2,10	0,02658	NM_032048	P4HTM
8079677	-0,96	-1,95	0,00183	0,00	-1,00	0,99212	NM_177938	MICAL3
8074274	-0,96	-1,95	0,01868	1,21	2,31	0,03272	NM_015241	LOC134466
8115205	-0,96	-1,95	0,01674	-1,36	-2,56	0,06838	NR_026867	TNK2
8093013	-0,96	-1,95	0,08137	-0,55	-1,46	0,20044	NM_005781	CA8
8150978	-0,96	-1,95	0,14795	-0,59	-1,50	0,00292	NM_004056	CXorf22
8166671	-0,96	-1,95	0,06034	-1,32	-2,50	0,00444	NM_152632	SLC6A1
8077833	-0,96	-1,95	0,01776	-0,79	-1,73	0,00010	NM_003042	PRSS45
8086669	-0,96	-1,95	0,07547	-0,97	-1,96	0,02812	NM_199183	---
8133959	-0,96	-1,95	0,10012	-1,66	-3,17	0,00278	---	OR5AN1
7940171	-0,96	-1,95	0,11712	-0,77	-1,71	0,00500	NM_001004729	CLDN2
8169210	-0,96	-1,95	0,07180	-0,03	-1,02	0,90244	NM_020384	SREBF1
8013135	-0,96	-1,95	0,01880	0,69	1,61	0,22954	NM_001005291	CD37
8030277	-0,96	-1,95	0,13653	-1,23	-2,34	0,00242	NM_001774	SPANXN4
8170282	-0,96	-1,95	0,11977	-0,95	-1,93	0,00072	NM_001009613	SLC10A3
8176109	-0,96	-1,95	0,07487	-1,56	-2,95	0,04165	NM_019848	MIR199B
8164396	-0,96	-1,95	0,14024	-1,82	-3,54	0,04270	NR_029619	BEX5
8174141	-0,96	-1,95	0,02651	-0,72	-1,64	0,00310	NM_001012978	CX3CR1
8086344	-0,96	-1,95	0,20128	-0,56	-1,47	0,11149	NM_001337	NINJ2
7960253	-0,96	-1,95	0,18634	-0,96	-1,94	0,06335	NM_016533	SURF6
8164862	-0,96	-1,95	0,00953	-0,74	-1,67	0,02750	NM_006753	RHBDL2
7915184	-0,96	-1,95	0,00555	-0,49	-1,40	0,03009	NM_017821	---
8091281	-0,96	-1,95	0,00491	-0,95	-1,93	0,00957	---	LOC100128840
7967700	-0,96	-1,95	0,02103	-0,91	-1,87	0,06980	ENST00000415058	PFKFB1
8173120	-0,96	-1,95	0,03938	-0,79	-1,73	0,17106	NM_002625	EHF
7939314	-0,96	-1,95	0,01658	1,85	3,62	0,00572	NM_012153	PPP1R13L
8037563	-0,96	-1,95	0,01423	0,63	1,55	0,21369	NM_006663	PKHD1
8126954	-0,97	-1,95	0,00134	-0,97	-1,95	0,02366	NM_138694	UGT2B10
8095390	-0,97	-1,95	0,05389	-0,70	-1,62	0,14435	NM_001075	---
8141726	-0,97	-1,95	0,34909	-1,37	-2,59	0,03722	---	CARD17
7951413	-0,97	-1,95	0,03488	-1,00	-2,01	0,02784	NM_001007232	RP11-93B14.6
8067543	-0,97	-1,95	0,24275	-1,47	-2,78	0,00011	ENST00000370520	SLC45A2
8111417	-0,97	-1,95	0,00833	-0,71	-1,63	0,00347	NM_016180	---
8086389	-0,97	-1,95	0,11786	-0,81	-1,75	0,08496	---	SNORA24
8097056	-0,97	-1,95	0,02342	-0,71	-1,63	0,00545	NR_002963	AGRP
8002051	-0,97	-1,95	0,13418	-0,59	-1,51	0,19929	NM_001138	CYP21A2
8118498	-0,97	-1,95	0,07274	-0,63	-1,54	0,24235	NM_000500	OR2L13
7911237	-0,97	-1,95	0,04219	-1,33	-2,52	0,00117	NM_175911	---
8100308	-0,97	-1,95	0,59761	-5,91	-60,31	0,00070	---	DISC1
7910559	-0,97	-1,95	0,00156	-0,73	-1,65	0,03147	NM_001164537	DMRTA1
8154656	-0,97	-1,95	0,00379	-1,06	-2,09	0,01768	NM_022160	SYCE1
7937230	-0,97	-1,95	0,00129	-1,19	-2,29	0,00004	NM_130784	---
7985200	-0,97	-1,95	0,00006	-0,91	-1,88	0,00909	---	ZSCAN10
7998921	-0,97	-1,95	0,02084	-1,41	-2,65	0,04942	NM_032805	HPSE2
7935613	-0,97	-1,95	0,12294	-1,10	-2,14	0,00033	NM_021828	C14orf50
7975081	-0,97	-1,95	0,03098	-0,67	-1,59	0,00229	BC068989 CLDN6	
7998898	-0,97	-1,95	0,16430	-1,77	-3,42	0,00612	NM_021195	MFSD2B
8040516	-0,97	-1,95	0,13173	-0,66	-1,58	0,22132	NM_001080473	MUC15
7947156	-0,97	-1,95	0,06795	-1,40	-2,65	0,00689	NM_001135091	E2F8
7947110	-0,97	-1,95	0,11634	2,05	4,15	0,00683	NM_024680	NLRP11
8039545	-0,97	-1,95	0,01758	-0,95	-1,93	0,04191	NM_145007	FLJ46121 // FLJ46121
8001108	-0,97	-1,95	0,05416	-2,09	-4,26	0,00185	AK128823 // AK128823	SELP
7922200	-0,97	-1,95	0,04532	-1,07	-2,10	0,05368	NM_003005	---

8126093	-0,97	-1,96	0,10404	-1,86	-3,63	0,03301	---	ANGPTL7
7897675	-0,97	-1,96	0,08423	-1,50	-2,83	0,03551	NM_021146	MSH4
7902402	-0,97	-1,96	0,09538	-1,41	-2,66	0,02442	NM_002440	ASMTL
8177011	-0,97	-1,96	0,01752	-0,39	-1,31	0,38059	NM_004192	---
8175570	-0,97	-1,96	0,01117	-0,93	-1,91	0,00031	---	IGHV7-81
7981745	-0,97	-1,96	0,11090	-1,35	-2,54	0,09128	ENST00000420380	RALGPS1
8157949	-0,97	-1,96	0,00653	1,35	2,54	0,00332	NM_014636	DKK4
8150529	-0,97	-1,96	0,15752	-1,18	-2,27	0,04760	NM_014420	---
7975282	-0,97	-1,96	0,06841	-1,56	-2,96	0,01525	---	CLEC4F
8052908	-0,97	-1,96	0,13858	-1,25	-2,38	0,00047	NM_173535	LOC100129979
7982348	-0,97	-1,96	0,02344	-1,49	-2,81	0,02611	ENST00000416633	---
8128075	-0,97	-1,96	0,09129	-2,11	-4,32	0,00047	---	TRPV4
7966234	-0,97	-1,96	0,06942	-0,56	-1,48	0,16854	NM_021625	ARVCF
8074522	-0,97	-1,96	0,19461	-0,80	-1,74	0,20438	NM_001670	DNMT3L
8070730	-0,97	-1,96	0,02713	-1,49	-2,80	0,00514	NM_013369	TMEM146
8024971	-0,97	-1,96	0,01918	-1,47	-2,77	0,00182	NM_152784	SHBG
8004534	-0,97	-1,96	0,02712	-1,14	-2,20	0,02034	NM_001040	OR2A4
8129558	-0,97	-1,96	0,16777	1,28	2,43	0,01486	NM_030908	---
8172803	-0,97	-1,96	0,13661	-0,40	-1,32	0,00198	---	LHX6
8163936	-0,97	-1,96	0,06637	-0,27	-1,20	0,68464	NM_014368	---
7944361	-0,97	-1,96	0,66942	-1,96	-3,88	0,07523	---	AQP6
7955305	-0,97	-1,96	0,04347	-1,38	-2,60	0,00011	NM_001652	KRT35
8015301	-0,97	-1,96	0,05346	-1,22	-2,33	0,01251	NM_002280	---
7976642	-0,97	-1,96	0,46622	-5,07	-33,59	0,00123	---	---
8171577	-0,97	-1,96	0,09358	-1,09	-2,13	0,00123	---	OGFOD2
7959535	-0,97	-1,96	0,01633	0,57	1,48	0,29036	NM_024623	OFCC1
8123843	-0,97	-1,96	0,04726	-0,97	-1,96	0,01243	AF520800	---
8162080	-0,97	-1,96	0,00756	-1,10	-2,15	0,07061	---	CMTM5
7973403	-0,97	-1,96	0,03825	-1,44	-2,71	0,00220	NM_138460	---
8110587	-0,97	-1,96	0,10441	-1,09	-2,13	0,02266	---	KRTAP19-7
8069845	-0,97	-1,96	0,11914	-0,24	-1,18	0,15461	NM_181614	MIR519A2
8031027	-0,97	-1,96	0,02352	-0,95	-1,94	0,00678	NR_030222	AIM1L
7913938	-0,97	-1,96	0,05069	-1,40	-2,65	0,02679	NM_001039775	---
8084605	-0,97	-1,96	0,11629	-2,28	-4,85	0,00090	---	LOC284861
8074734	-0,97	-1,96	0,29377	-3,56	-11,76	0,02281	AK128837	PPP1R16A
8148888	-0,97	-1,96	0,04536	0,20	1,15	0,61622	NM_032902	C15orf28
7990027	-0,97	-1,96	0,14137	-0,87	-1,83	0,05510	NR_026808	CACNG3
7994152	-0,97	-1,96	0,01914	-1,30	-2,46	0,01901	NM_006539	GCM1
8127130	-0,97	-1,96	0,06198	-0,92	-1,90	0,04979	NM_003643	---
8101778	-0,97	-1,96	0,00529	-1,59	-3,01	0,05200	---	---
7901338	-0,97	-1,96	0,00752	-0,85	-1,81	0,00417	---	---
8055643	-0,97	-1,96	0,01150	-1,25	-2,38	0,04812	---	FLJ44124
8131042	-0,97	-1,96	0,09976	-1,28	-2,43	0,00043	AK126112	GPR135
7979426	-0,97	-1,96	0,04171	-0,69	-1,62	0,04515	NM_022571	DEFB128
8064319	-0,97	-1,96	0,06063	-1,14	-2,20	0,00472	NM_001037732	VWA5B2
8084382	-0,97	-1,96	0,00296	-0,47	-1,39	0,21439	NM_138345	BEND4
8100015	-0,97	-1,96	0,04586	-0,11	-1,08	0,73298	NM_207406	C14orf102
7980702	-0,97	-1,96	0,02317	-0,02	-1,01	0,96363	NM_017970	C1orf191
7901611	-0,97	-1,96	0,04515	-0,44	-1,36	0,04150	ENST00000361350	PP14571
8060101	-0,97	-1,96	0,11692	-1,85	-3,60	0,00067	AK092442	MBD3L3
8033356	-0,97	-1,96	0,18157	-1,92	-3,80	0,00933	NM_001164425	MBD3L3
8033359	-0,97	-1,96	0,18157	-1,92	-3,80	0,00933	NM_001164425	S100A7
7920252	-0,97	-1,96	0,14770	-2,25	-4,75	0,00091	NM_002963	SLCO1B3
7954330	-0,97	-1,96	0,00028	2,42	5,34	0,00868	NM_019844	BAIAP2L2
8076007	-0,97	-1,96	0,10679	-0,02	-1,01	0,96819	NM_025045	---
8095035	-0,97	-1,96	0,11329	-0,88	-1,84	0,01026	---	TUBA4B
8048518	-0,97	-1,96	0,09986	-1,32	-2,50	0,00800	NR_003063	---
7948303	-0,97	-1,96	0,08551	-1,25	-2,38	0,00729	---	DRD5
8094156	-0,97	-1,96	0,15113	-1,67	-3,18	0,02636	NM_000798	SLC6A7
8109236	-0,97	-1,96	0,06202	-1,72	-3,30	0,00004	NM_014228	TAS2R7
7961239	-0,97	-1,96	0,00263	-0,81	-1,76	0,04543	NM_023919	---
8075059	-0,97	-1,96	0,05473	-1,17	-2,24	0,04247	---	KCNC4
7903867	-0,97	-1,96	0,01907	-0,29	-1,22	0,21098	NM_004978	PCDHB11
8108729	-0,97	-1,96	0,01076	-0,61	-1,53	0,00043	NM_018931	DDX12
7953844	-0,97	-1,96	0,12181	0,63	1,55	0,26781	NR_033399	---
8121116	-0,97	-1,96	0,02564	-1,16	-2,24	0,07242	---	ACMSD
8045368	-0,97	-1,96	0,02808	-1,32	-2,50	0,01352	NM_138326	---
8085813	-0,97	-1,96	0,05424	-0,51	-1,43	0,03199	---	TLX3
8109969	-0,97	-1,96	0,15865	-1,64	-3,12	0,01427	NM_021025	---
8131441	-0,97	-1,96	0,46860	0,56	1,48	0,30113	---	AGTR1
8083240	-0,97	-1,96	0,10037	-0,92	-1,89	0,01405	NM_031850	PRPH
7955184	-0,97	-1,96	0,02484	-0,73	-1,65	0,02587	NM_006262	---
7933176	-0,97	-1,96	0,03013	-1,80	-3,48	0,00039	---	CORO6

8013947	-0,97	-1,96	0,11803	-0,25	-1,19	0,50957	NM_032854	FAM70B
7972936	-0,97	-1,96	0,06979	-1,37	-2,58	0,01232	NM_182614	SLC16A3
8010770	-0,97	-1,96	0,14102	0,35	1,28	0,39072	NM_001042422	---
8042501	-0,97	-1,96	0,08844	-0,76	-1,69	0,09838	---	---
8098287	-0,97	-1,96	0,01154	-1,08	-2,11	0,01272	---	KLF17
7900957	-0,97	-1,96	0,03411	-1,89	-3,70	0,00601	NM_173484	FLJ25694
7971915	-0,97	-1,96	0,10211	-0,94	-1,92	0,04461	AK127969	C12orf33
7960942	-0,97	-1,96	0,00050	-0,36	-1,28	0,03164	BC028195	RAG2
7947548	-0,97	-1,96	0,02920	-0,85	-1,81	0,01897	NM_000536	CELA3B
7898713	-0,97	-1,96	0,18795	-2,34	-5,05	0,01408	NM_007352	---
8093683	-0,97	-1,97	0,03710	-1,43	-2,70	0,01916	---	LOC100129278
8040483	-0,97	-1,97	0,00334	-0,83	-1,78	0,05499	ENST00000405799	ZNF785
8000917	-0,97	-1,97	0,04860	-0,73	-1,66	0,18086	NM_152458	BSN
8079759	-0,97	-1,97	0,04706	-1,19	-2,28	0,00120	NM_003458	NDUFAF2
8105499	-0,97	-1,97	0,13970	-0,56	-1,48	0,06419	NM_174889	FOLR3
7942328	-0,98	-1,97	0,16120	-1,90	-3,73	0,00153	NM_000804	OR5T2
7948125	-0,98	-1,97	0,05889	-0,96	-1,94	0,08091	NM_001004746	OR6T1
7952382	-0,98	-1,97	0,02015	-1,31	-2,48	0,02575	NM_001005187	---
7989309	-0,98	-1,97	0,00962	-0,81	-1,75	0,03125	---	---
8026292	-0,98	-1,97	0,01548	-0,82	-1,77	0,03802	---	HIST2H2BF
7919604	-0,98	-1,97	0,10538	-3,27	-9,66	0,00412	AK092418	---
8112647	-0,98	-1,97	0,06329	-1,24	-2,37	0,01967	---	---
8081028	-0,98	-1,97	0,08551	-1,05	-2,08	0,01046	---	NFKBID
8036207	-0,98	-1,97	0,00997	-0,21	-1,16	0,29525	NM_139239	TMEM191A
8071541	-0,98	-1,97	0,21427	-2,13	-4,37	0,00668	NR_026815	SLC17A4
8117304	-0,98	-1,97	0,04253	-1,19	-2,28	0,00058	NM_005495	TUBAL3
7931852	-0,98	-1,97	0,05023	-1,21	-2,31	0,00731	NM_024803	RNF175
8103244	-0,98	-1,97	0,18711	-1,26	-2,40	0,00409	NM_173662	KRT78
7963555	-0,98	-1,97	0,00295	-0,89	-1,85	0,09529	NM_173352	C9orf11
8160511	-0,98	-1,97	0,01887	-0,72	-1,65	0,00154	NM_020641	---
7917597	-0,98	-1,97	0,05445	-1,19	-2,28	0,00088	---	IGLL1
8074909	-0,98	-1,97	0,10059	-1,99	-3,97	0,00150	NM_020070	MIR301A
8017139	-0,98	-1,97	0,10509	-0,59	-1,51	0,01089	NR_029842	---
8052678	-0,98	-1,97	0,01936	-0,98	-1,97	0,00822	---	ZNF775
8137228	-0,98	-1,97	0,02830	-1,38	-2,59	0,00021	NM_173680	RFX8
8054344	-0,98	-1,97	0,05894	-1,63	-3,09	0,00022	NM_001145664	TMEM90A
7980142	-0,98	-1,97	0,02853	-0,81	-1,76	0,09594	NM_001105579	SPN
7994603	-0,98	-1,97	0,17361	-0,92	-1,89	0,04993	NM_001030288	---
8036865	-0,98	-1,97	0,16295	-0,73	-1,66	0,04308	---	FGF18
8109981	-0,98	-1,97	0,06426	-1,98	-3,96	0,00160	NM_003862	MIR7-3
8024896	-0,98	-1,97	0,13990	-1,48	-2,79	0,00227	NR_029607	MIR190
7984077	-0,98	-1,97	0,02417	-0,83	-1,78	0,00190	NR_029709	GPAT2
8053842	-0,98	-1,97	0,10478	-1,43	-2,70	0,02240	NM_207328	IFNA8
8154622	-0,98	-1,97	0,09137	-1,12	-2,17	0,02296	NM_002170	GIPR
8029728	-0,98	-1,97	0,03403	-0,06	-1,04	0,76094	NM_000164	LHX5
7966631	-0,98	-1,97	0,06700	-0,43	-1,35	0,06896	NM_022363	C20orf54
8064388	-0,98	-1,97	0,07040	-0,33	-1,25	0,53701	NM_033409	SH2B1
7994445	-0,98	-1,97	0,01519	0,38	1,30	0,24856	NM_015503	UBE2MP1 // UBE2MP1 // UBE2MP1 // UBE2MP1
8001111	-0,98	-1,97	0,03691	-1,05	-2,06	0,02587	NR_002837 // NR_002837 // NR_002837 // NR_002837	ZDHHC11
8110721	-0,98	-1,97	0,15904	-1,06	-2,09	0,20172	NM_024786	PFN3
8116031	-0,98	-1,97	0,08698	-0,28	-1,22	0,52363	NM_001029886	POTEC
8022428	-0,98	-1,97	0,11473	-2,31	-4,97	0,00566	NM_001137671	OR1M1
8025456	-0,98	-1,97	0,22019	-1,32	-2,50	0,08096	NM_001004456	---
7984255	-0,98	-1,97	0,01032	-0,56	-1,47	0,00497	---	KRT13
8015323	-0,98	-1,97	0,00902	-1,16	-2,24	0,00348	NM_153490	AZI1
8019108	-0,98	-1,97	0,01218	-0,83	-1,78	0,03723	NM_014984	---
8098161	-0,98	-1,97	0,03178	-0,68	-1,60	0,00144	---	OR14I1
7925761	-0,98	-1,97	0,13220	-0,70	-1,63	0,27415	NM_001004734	D2HGDH
8077238	-0,98	-1,97	0,06082	-0,41	-1,33	0,37305	NM_152783	OR2H2
8177706	-0,98	-1,97	0,08757	-1,59	-3,01	0,01000	NM_007160	OR2H2
8179009	-0,98	-1,97	0,08757	-1,59	-3,01	0,01000	NM_007160	SERPINI2
8091910	-0,98	-1,97	0,02365	-1,43	-2,70	0,00022	NM_006217	LOC100129827
7938416	-0,98	-1,97	0,00464	-0,77	-1,70	0,18154	ENST00000324928	IFI27L1
7976436	-0,98	-1,97	0,05222	1,00	1,99	0,16618	NM_145249	C2orf19
8059965	-0,98	-1,97	0,00932	-0,77	-1,70	0,00913	BC031945	OR10AD1
7962792	-0,98	-1,97	0,05818	-1,76	-3,39	0,03671	NM_001004134	DEFB109P1B
8149358	-0,98	-1,97	0,54967	-0,74	-1,67	0,39957	NR_003668	DEFB104A
8144457	-0,98	-1,97	0,01607	-1,25	-2,38	0,00033	NM_080389	DEFB104A
8149192	-0,98	-1,97	0,01607	-1,25	-2,38	0,00033	NM_080389	C3P1
8025522	-0,98	-1,97	0,00580	-1,38	-2,59	0,01282	NR_027300	GAR1
8096821	-0,98	-1,97	0,07405	-0,12	-1,08	0,55121	NM_018983	PFN4

8050713	-0,98	-1,97	0,04876	0,98	1,97	0,00477	NM_199346		TCAP
8006896	-0,98	-1,97	0,09000	-2,42	-5,37	0,00010	NM_003673		VNN1
8129618	-0,98	-1,97	0,01525	-1,23	-2,34	0,00698	NM_004666		TPM4
8026513	-0,98	-1,97	0,00425	-1,37	-2,59	0,00042	NM_001145160		---
8138007	-0,98	-1,97	0,14391	-0,74	-1,68	0,01159	---	TECTB	
7930487	-0,98	-1,97	0,04986	-0,76	-1,69	0,00384	NM_058222		PSORS1C3
8124897	-0,98	-1,97	0,10602	-1,95	-3,86	0,00056	AY484516	PSORS1C3	
8179727	-0,98	-1,97	0,10602	-1,95	-3,86	0,00056	AY484516	TCTEX1D4	
7915609	-0,98	-1,97	0,03652	-1,65	-3,15	0,02924	NM_001013632		ST8SIA5
8023121	-0,98	-1,97	0,04252	-1,15	-2,21	0,01062	NM_013305		PROKR1
8042413	-0,98	-1,97	0,08530	-1,61	-3,06	0,04209	NM_138964		CAND2
8077944	-0,98	-1,97	0,02889	-1,73	-3,33	0,01287	NM_001162499		TMEM86B
8039409	-0,98	-1,97	0,07244	-0,97	-1,96	0,01855	NM_173804		STON2
7980537	-0,98	-1,97	0,05523	-0,41	-1,33	0,04438	NM_033104		KIAA1530
8093476	-0,98	-1,97	0,01676	-0,26	-1,20	0,25145	NM_020894		FAM154B
7985398	-0,98	-1,97	0,02248	-0,84	-1,79	0,13022	AK304339	AQPEP	
8107429	-0,98	-1,97	0,02105	-0,85	-1,80	0,02243	NM_173800		---
8074210	-0,98	-1,97	0,09140	-1,35	-2,54	0,00656	---	TRIM58	
7911218	-0,98	-1,97	0,04238	-1,43	-2,70	0,00121	NM_015431		C16orf71
7993024	-0,98	-1,97	0,03670	-1,04	-2,06	0,01725	NM_139170		EFCAB5
8006085	-0,98	-1,97	0,02575	-0,83	-1,77	0,01803	NM_198529		---
8101141	-0,98	-1,97	0,02211	-0,32	-1,24	0,04015	---	TBC1D29	
8006161	-0,98	-1,98	0,18696	-0,72	-1,64	0,00788	NM_015594		ALDH3A1
8013384	-0,98	-1,98	0,18015	-0,86	-1,81	0,02187	NM_000691		---
8138598	-0,98	-1,98	0,08804	-1,62	-3,07	0,00033	---	---	
7904016	-0,98	-1,98	0,02086	-1,22	-2,33	0,01770	---	---	
7903391	-0,98	-1,98	0,07720	-1,14	-2,20	0,01163	---	ZNF500	
7999196	-0,98	-1,98	0,04492	-0,29	-1,22	0,19776	NM_021646		UMOD
7999936	-0,98	-1,98	0,07019	-1,13	-2,19	0,03043	NM_003361		LOC503543
7910184	-0,98	-1,98	0,01267	-0,47	-1,39	0,12077	BC062736	SIPA1	
7941311	-0,98	-1,98	0,00676	0,04	1,03	0,93169	NM_153253		CYP2F1
8028984	-0,98	-1,98	0,17945	-1,52	-2,87	0,06645	NM_000774		PODXL
8142981	-0,98	-1,98	0,08702	1,51	2,85	0,00041	NM_001018111		---
8122805	-0,98	-1,98	0,09336	-1,23	-2,34	0,00305	---	12. Sep	
7999206	-0,98	-1,98	0,04233	0,53	1,45	0,06108	NM_144605		SPEG
8048551	-0,98	-1,98	0,05922	-1,08	-2,12	0,00595	NM_005876		APRT
8003401	-0,98	-1,98	0,00162	0,35	1,27	0,60023	NM_000485		TTLL8
8076882	-0,98	-1,98	0,07633	-2,19	-4,58	0,00346	NM_001080447		HOXB7
8016468	-0,98	-1,98	0,02246	-0,15	-1,11	0,53567	NM_004502		---
7974916	-0,98	-1,98	0,04363	-1,41	-2,66	0,00005	---	---	
8057929	-0,98	-1,98	0,06128	-1,32	-2,49	0,01785	---	---	
7957629	-0,98	-1,98	0,14450	-0,99	-1,98	0,00515	---	---	
7924203	-0,98	-1,98	0,00271	-0,81	-1,75	0,01930	---	CCL1	
8014160	-0,98	-1,98	0,21266	-0,83	-1,77	0,00912	NM_002981		EML2
8037621	-0,98	-1,98	0,05827	0,63	1,55	0,09327	NM_012155		SEC1
8030092	-0,98	-1,98	0,08970	-2,45	-5,45	0,02286	NR_004401		OR5B17
7948322	-0,98	-1,98	0,07393	-1,28	-2,42	0,04022	NM_001005489		FAM95B1
8155627	-0,98	-1,98	0,14970	-0,94	-1,92	0,06652	AL833349	FAM95B1	
8161381	-0,98	-1,98	0,14970	-0,94	-1,92	0,06652	AL833349	---	
8098940	-0,98	-1,98	0,10197	-1,30	-2,46	0,00138	---	ORAI1	
7959294	-0,98	-1,98	0,05271	1,43	2,69	0,04329	NM_032790		---
8143365	-0,98	-1,98	0,23242	-0,46	-1,37	0,05248	---	---	
8116573	-0,98	-1,98	0,00455	-1,20	-2,30	0,00405	---	GSTTP1	
8074972	-0,98	-1,98	0,09045	-1,57	-2,96	0,00018	NR_003081		---
8008702	-0,98	-1,98	0,02506	-2,64	-6,23	0,00034	---	---	
8139697	-0,98	-1,98	0,00754	-1,27	-2,41	0,00571	---	OR7E125P	
8144496	-0,98	-1,98	0,51868	-3,43	-10,77	0,00001	ENST00000420198		LAT2
8133442	-0,98	-1,98	0,12506	-0,16	-1,12	0,64217	NM_032464		IL17B
8115014	-0,99	-1,98	0,05764	-1,04	-2,06	0,08577	NM_014443		LOC401433
8143939	-0,99	-1,98	0,07304	-0,25	-1,19	0,58595	AK127717	CYP2A6	
8036969	-0,99	-1,98	0,02185	-1,69	-3,23	0,00258	NM_000762		KRT75
7963396	-0,99	-1,98	0,12876	-1,63	-3,09	0,00013	NM_004693		---
8115344	-0,99	-1,98	0,02634	-1,77	-3,40	0,00074	---	MAGEB6	
8166571	-0,99	-1,98	0,01013	-1,09	-2,13	0,01875	NM_173523		---
7932862	-0,99	-1,98	0,08980	-0,62	-1,54	0,05618	---	IGDCC3	
7989770	-0,99	-1,98	0,02400	-0,77	-1,70	0,06037	NM_004884		GUCY2C
7961455	-0,99	-1,98	0,10086	-1,26	-2,40	0,00032	NM_004963		AMDHD2
7992656	-0,99	-1,98	0,03307	-1,49	-2,81	0,04073	NM_015944		NT5C
8018297	-0,99	-1,98	0,01576	-0,73	-1,65	0,03164	NM_014595		---
7928543	-0,99	-1,98	0,11004	-1,06	-2,08	0,10566	---	PQLC1	
8023914	-0,99	-1,98	0,04663	-0,10	-1,07	0,83683	NM_025078		NAP1L2
8173600	-0,99	-1,98	0,00989	-0,60	-1,51	0,06437	NM_021963		PPARGC1B
8109179	-0,99	-1,98	0,03023	-0,07	-1,05	0,85347	NM_133263		---

8002967	-0,99	-1,98	0,01851	-1,64	-3,11	0,01600	---	---
8111812	-0,99	-1,98	0,17989	-1,07	-2,09	0,00475	---	---
8102829	-0,99	-1,98	0,18050	-0,71	-1,64	0,08269	---	LRIT3
8096839	-0,99	-1,98	0,00071	-0,81	-1,76	0,05986	NM_198506	---
7943049	-0,99	-1,98	0,02545	-1,70	-3,24	0,01726	---	APOB
8050619	-0,99	-1,98	0,06865	-2,04	-4,12	0,01423	NM_000384	---
7968796	-0,99	-1,98	0,01938	-1,39	-2,62	0,05948	---	---
7904014	-0,99	-1,98	0,04681	-1,25	-2,38	0,00179	---	---
8146322	-0,99	-1,98	0,11615	-0,92	-1,89	0,06611	---	SCARNA14
7989922	-0,99	-1,98	0,00595	-1,58	-3,00	0,01997	NR_004388	MYL2
7966379	-0,99	-1,98	0,04858	-0,91	-1,88	0,00210	NM_000432	C14orf165
7978103	-0,99	-1,98	0,04998	-1,22	-2,32	0,00016	NR_024081	GPR55
8059674	-0,99	-1,98	0,00180	-0,73	-1,66	0,00348	NM_005683	IRS4
8174444	-0,99	-1,98	0,08513	-1,32	-2,50	0,02019	NM_003604	PAQR6
7920928	-0,99	-1,98	0,05710	0,21	1,16	0,45157	NM_024897	COL4A6
8174389	-0,99	-1,98	0,01587	-0,91	-1,87	0,00157	NM_033641	MACROD1
7949033	-0,99	-1,98	0,07902	-0,78	-1,71	0,03534	NM_014067	MIR202
7937148	-0,99	-1,98	0,05433	-0,94	-1,92	0,02100	NR_030170	---
8167786	-0,99	-1,98	0,00609	-0,64	-1,56	0,00315	---	ISM2
7980425	-0,99	-1,98	0,20119	-0,11	-1,08	0,70899	NM_199296	RFX6
8121649	-0,99	-1,98	0,11053	-0,74	-1,67	0,00172	NM_173560	C3orf47
8082517	-0,99	-1,98	0,11309	-1,40	-2,63	0,00179	NR_026991	---
8117626	-0,99	-1,98	0,20170	-1,92	-3,79	0,01457	---	OR8G2
7944834	-0,99	-1,98	0,35899	-2,33	-5,03	0,00043	NM_001007249	RN28S1
7942791	-0,99	-1,98	0,08722	-3,82	-14,14	0,00001	NR_003287	---
7971913	-0,99	-1,98	0,00129	-1,21	-2,31	0,12975	---	FAM124B
8059387	-0,99	-1,98	0,02439	-1,14	-2,21	0,02419	NM_024785	MIR26B
8048317	-0,99	-1,98	0,04172	-1,13	-2,18	0,01767	NR_029500	H1FOO
8082560	-0,99	-1,98	0,03862	-1,59	-3,00	0,00426	NM_153833	---
8100454	-0,99	-1,98	0,06300	-0,56	-1,48	0,00585	---	C12orf40
7954794	-0,99	-1,98	0,05528	-1,00	-2,00	0,00081	NM_001031748	---
7989130	-0,99	-1,98	0,01335	-1,00	-2,00	0,00011	---	TCTE1
8126674	-0,99	-1,98	0,00041	0,17	1,12	0,60158	NM_182539	---
7955074	-0,99	-1,98	0,12924	-0,71	-1,64	0,00920	---	MYO7B
8045030	-0,99	-1,98	0,01028	-0,99	-1,99	0,03606	NM_001080527	SNX32
7941408	-0,99	-1,98	0,06240	-0,71	-1,63	0,04540	NM_152760	TMEM229B
7979721	-0,99	-1,99	0,15652	0,01	1,01	0,98281	NM_182526	---
7977773	-0,99	-1,99	0,12606	-1,75	-3,37	0,03822	---	DNAJC30
8140107	-0,99	-1,99	0,16535	-1,56	-2,96	0,02163	NM_032317	OR2J1
8117712	-0,99	-1,99	0,00678	-1,92	-3,78	0,00239	ENST00000377171	OR7C1
8034890	-0,99	-1,99	0,00209	-1,01	-2,01	0,00419	NM_198944	MIR92A2
8175248	-0,99	-1,99	0,06341	-1,01	-2,02	0,00650	NR_029509	XAGE-4
8173176	-0,99	-1,99	0,06129	-0,92	-1,89	0,00816	AJ318895	TMIE
8079426	-0,99	-1,99	0,02266	-0,20	-1,15	0,12231	NM_147196	---
7997700	-0,99	-1,99	0,04746	-1,07	-2,10	0,01123	---	C6orf15
8179713	-0,99	-1,99	0,09394	-1,25	-2,38	0,00728	NM_014070	NANOG
7987365	-0,99	-1,99	0,06934	-1,41	-2,65	0,00594	NM_024865	LDHD
8002830	-0,99	-1,99	0,20328	-0,44	-1,36	0,02385	NM_153486	MSH5
8118259	-0,99	-1,99	0,00990	0,88	1,85	0,02854	NM_002441	PAX8
8054740	-0,99	-1,99	0,03405	-0,95	-1,94	0,01564	NM_003466	C1orf110
7921909	-0,99	-1,99	0,02992	-1,01	-2,02	0,00505	NM_178550	FTCD
8070933	-0,99	-1,99	0,00464	-1,20	-2,30	0,00070	NM_006657	---
7974314	-0,99	-1,99	0,01763	-1,34	-2,53	0,03701	---	---
8111327	-0,99	-1,99	0,07115	-1,04	-2,05	0,03603	---	FUT3
8033054	-0,99	-1,99	0,13309	-0,72	-1,64	0,06069	NM_000149	MAP3K9
7979943	-0,99	-1,99	0,05357	0,78	1,72	0,06506	NM_033141	DCC
8021245	-0,99	-1,99	0,00940	-1,62	-3,07	0,01792	NM_005215	C2CD4A
7984016	-0,99	-1,99	0,20998	-0,73	-1,66	0,12213	NM_207322	---
8161986	-0,99	-1,99	0,00331	-0,99	-1,98	0,00024	---	---
8097478	-0,99	-1,99	0,00285	-1,35	-2,54	0,00060	---	---
8016213	-0,99	-1,99	0,21968	-0,89	-1,85	0,09530	---	FAIM3
7923917	-0,99	-1,99	0,01542	-0,76	-1,69	0,04478	NM_005449	LOC283588
7980742	-0,99	-1,99	0,02627	-0,09	-1,06	0,68265	ENST00000433659	LOC554249
8155490	-0,99	-1,99	0,09934	0,14	1,10	0,65752	AK292642	CIITA
7993195	-0,99	-1,99	0,07630	-1,28	-2,43	0,00049	NM_000246	LOC442421
8155547	-0,99	-1,99	0,39800	-1,39	-2,63	0,04748	NR_024496	NPDC1
8165406	-0,99	-1,99	0,22772	-0,74	-1,67	0,06895	NM_015392	CRIP1
7977409	-0,99	-1,99	0,07314	-0,64	-1,56	0,08553	NM_001311	MIR451
8013784	-0,99	-1,99	0,00252	-1,10	-2,14	0,01211	NR_029970	---
8117239	-0,99	-1,99	0,15876	-2,18	-4,52	0,00626	---	CARD11
8137874	-0,99	-1,99	0,01598	-1,00	-2,00	0,02945	NM_032415	C1orf157
7923584	-0,99	-1,99	0,11894	-1,53	-2,88	0,00645	NR_027902	ZNF575
8029301	-0,99	-1,99	0,08091	-1,73	-3,31	0,00065	NM_174945	NES

7921088	-0,99	-1,99	0,00624	-1,31	-2,47	0,01345	NM_006617	---
8136977	-0,99	-1,99	0,14120	-0,67	-1,59	0,05165	---	MUC5B
7937612	-0,99	-1,99	0,09481	-1,15	-2,22	0,03722	NM_002458	LOC100131373
8040469	-0,99	-1,99	0,11150	-1,41	-2,66	0,02960	AY189285	---
8104661	-0,99	-1,99	0,01661	-1,38	-2,60	0,02657	---	SYN2
8077879	-0,99	-1,99	0,31324	-1,05	-2,07	0,03754	NM_133625	MACC1
8138466	-0,99	-1,99	0,05792	-0,33	-1,25	0,32692	NM_182762	---
8046084	-0,99	-1,99	0,00269	-0,45	-1,37	0,02002	---	---
7957958	-0,99	-1,99	0,08883	-0,72	-1,65	0,00125	---	C22orf31
8075192	-0,99	-1,99	0,00866	-0,71	-1,64	0,02220	BC126262	FADD
7942168	-0,99	-1,99	0,00837	0,54	1,45	0,32239	NM_003824	PTPRR
7964907	-0,99	-1,99	0,02412	-1,13	-2,19	0,02430	NM_002849	TFAP2C
8063536	-0,99	-1,99	0,14023	2,72	6,57	0,00002	NM_003222	SEMA3G
8087907	-0,99	-1,99	0,04242	-0,66	-1,58	0,18615	NM_020163	EGLN3
7978544	-0,99	-1,99	0,01410	0,74	1,67	0,28621	NM_022073	---
8139915	-0,99	-1,99	0,00304	-0,95	-1,93	0,01906	---	---
8007673	-0,99	-1,99	0,05798	-0,61	-1,53	0,15924	---	CCDC114
8038048	-0,99	-1,99	0,01370	-0,21	-1,16	0,29986	NM_144577	MAP7D2
8171725	-0,99	-1,99	0,07567	-1,03	-2,04	0,16979	NM_001168465	---
7916743	-0,99	-1,99	0,06228	-1,30	-2,47	0,02310	---	---
8066264	-0,99	-1,99	0,05648	-1,30	-2,46	0,06445	---	C7orf27
8137847	-0,99	-1,99	0,14526	-0,31	-1,24	0,30106	NM_152743	RNF125
8020806	-0,99	-1,99	0,00484	1,87	3,65	0,00027	NM_017831	IGSF9
7921492	-0,99	-1,99	0,06704	-0,86	-1,81	0,23583	NM_001135050	GRID1
7934789	-0,99	-1,99	0,13036	-1,26	-2,40	0,00582	NM_017551	---
7932418	-0,99	-1,99	0,04484	-1,08	-2,11	0,00419	---	GDF1
8035539	-0,99	-1,99	0,00998	-0,11	-1,08	0,81923	NM_001492	SLC6A5
7938907	-0,99	-1,99	0,09114	-0,91	-1,87	0,01905	NM_004211	ZBTB22
8125731	-0,99	-1,99	0,06541	-0,27	-1,21	0,33537	NM_005453	LYG2
8054200	-0,99	-1,99	0,01315	-1,19	-2,29	0,00103	NM_175735	SH3BP1
8072847	-0,99	-1,99	0,00132	-0,54	-1,46	0,12693	NM_018957	SNORD96A
8116530	-0,99	-1,99	0,04675	-0,91	-1,88	0,21003	NR_002592	FKSG73
8043487	-1,00	-1,99	0,00704	-1,22	-2,33	0,00152	NR_027714	RIPPLY1
8174317	-1,00	-1,99	0,13381	-1,39	-2,62	0,02842	NM_138382	WEE1
7938366	-1,00	-1,99	0,02351	-0,32	-1,25	0,01301	BX641032	---
7919160	-1,00	-1,99	0,00641	-1,02	-2,03	0,02476	---	---
7919166	-1,00	-1,99	0,00641	-1,02	-2,03	0,02476	---	OR5J2
7939971	-1,00	-1,99	0,02306	-1,09	-2,13	0,01186	NM_001005492	---
8003228	-1,00	-1,99	0,11516	-1,10	-2,14	0,00854	---	GPR156
8089820	-1,00	-1,99	0,01455	-0,09	-1,06	0,72447	NM_153002	PYY
8015890	-1,00	-1,99	0,04099	1,45	2,73	0,01159	NM_004160	B9D2
8037018	-1,00	-1,99	0,09727	-1,16	-2,23	0,00903	NM_030578	---
8008991	-1,00	-1,99	0,08561	-0,75	-1,68	0,00887	---	---
8047782	-1,00	-1,99	0,08059	-0,60	-1,52	0,31640	---	CKM
8037525	-1,00	-1,99	0,04950	-1,84	-3,58	0,09311	NM_001824	OR8H3
7939969	-1,00	-1,99	0,08735	-1,70	-3,24	0,09314	NM_001005201	KCNA4
7947270	-1,00	-1,99	0,01078	-0,66	-1,58	0,13061	NM_002233	UNC93B1
8138147	-1,00	-1,99	0,19725	-0,23	-1,17	0,78042	NM_030930	---
8149271	-1,00	-1,99	0,01249	-1,68	-3,21	0,00011	---	---
7906952	-1,00	-1,99	0,02334	-0,83	-1,77	0,01693	---	OR4K15
7973018	-1,00	-1,99	0,05179	-0,95	-1,93	0,03890	NM_001005486	---
7968926	-1,00	-1,99	0,09426	-1,85	-3,61	0,00337	---	ZFP92
8170644	-1,00	-2,00	0,00360	-0,42	-1,34	0,12961	NM_001136273	---
8106818	-1,00	-2,00	0,04286	-0,60	-1,51	0,08482	---	TTC9
7975412	-1,00	-2,00	0,06902	1,74	3,34	0,00104	NM_015351	LRRC48
8005289	-1,00	-2,00	0,00113	-0,93	-1,90	0,14177	NM_001130090	LILRB1
8031223	-1,00	-2,00	0,08836	-2,41	-5,33	0,00046	NM_006669	CRTAM
7944739	-1,00	-2,00	0,01700	-1,03	-2,04	0,00313	NM_019604	DMRTC2
8029117	-1,00	-2,00	0,08215	-1,71	-3,27	0,00054	NM_001040283	AQP10
7905746	-1,00	-2,00	0,00308	-1,81	-3,50	0,00823	NM_080429	SCRT1
8153704	-1,00	-2,00	0,00207	-1,68	-3,20	0,00094	NM_031309	SYT17
7993624	-1,00	-2,00	0,00962	3,94	15,35	0,00028	NM_016524	MYH14
8030582	-1,00	-2,00	0,02328	-0,14	-1,10	0,69275	NM_001145809	MIR516B1
8031035	-1,00	-2,00	0,04419	-0,77	-1,71	0,21759	NR_030212	ABCA4
7917798	-1,00	-2,00	0,02850	-0,95	-1,93	0,10961	NM_000350	GAB4
8074212	-1,00	-2,00	0,02565	-1,24	-2,36	0,02600	NM_001037814	---
8099447	-1,00	-2,00	0,00791	-0,59	-1,51	0,05127	---	---
8020375	-1,00	-2,00	0,00512	-1,37	-2,59	0,04354	---	TMEM184A
8137783	-1,00	-2,00	0,04721	0,32	1,25	0,41845	NM_001097620	KPTN
8037898	-1,00	-2,00	0,01513	-0,74	-1,67	0,05453	NM_007059	C10orf67
7932542	-1,00	-2,00	0,03046	-0,90	-1,87	0,00422	NM_153714	VASH2
7909642	-1,00	-2,00	0,00973	-1,07	-2,10	0,01112	NM_024749	GPM6B
8171359	-1,00	-2,00	0,12509	-0,38	-1,30	0,07648	NM_001001995	LOC554249

8155550	-1,00	-2,00	0,05902	0,05	1,04	0,88469	AK292642	SCTR
8054846	-1,00	-2,00	0,02179	-1,50	-2,82	0,01107	NM_002980	---
8135849	-1,00	-2,00	0,03125	-1,07	-2,10	0,02613	---	FABP1
8053654	-1,00	-2,00	0,06518	-0,88	-1,84	0,00259	NM_001443	---
7937988	-1,00	-2,00	0,13568	-1,08	-2,11	0,18235	---	FGF4
7950030	-1,00	-2,00	0,03215	-1,23	-2,35	0,00391	ENST00000425152	CITED4
7915347	-1,00	-2,00	0,01047	-0,55	-1,47	0,33743	NM_133467	NMNAT3
8091087	-1,00	-2,00	0,07069	0,10	1,07	0,75889	NM_178177	MNX1
8144112	-1,00	-2,00	0,03816	-0,59	-1,50	0,02449	NM_005515	KCNH2
8143825	-1,00	-2,00	0,05277	-0,69	-1,62	0,19080	NM_000238	TRIB3
8060344	-1,00	-2,00	0,07203	2,26	4,79	0,00309	NM_021158	SIT1
8161017	-1,00	-2,00	0,03041	-0,65	-1,57	0,05448	NM_014450	FAM71C
7957790	-1,00	-2,00	0,25220	-1,24	-2,36	0,00553	BC031221	SLCO2A1
8090823	-1,00	-2,00	0,04689	2,96	7,77	0,00042	NM_005630	---
7954206	-1,00	-2,00	0,07240	-1,26	-2,40	0,00006	---	ZNF726
8027363	-1,00	-2,00	0,03919	-1,15	-2,22	0,04633	BC046415	DEFB1
8149097	-1,00	-2,00	0,03011	-0,90	-1,87	0,07983	NM_005218	CCNYL3
7995312	-1,00	-2,00	0,16663	-0,60	-1,51	0,04830	ENST00000407030	CLDN4
8133360	-1,00	-2,00	0,08402	1,55	2,92	0,01200	NM_001305	---
8105318	-1,00	-2,00	0,00081	-1,07	-2,09	0,05536	---	MATN1
7899595	-1,00	-2,00	0,00928	-0,86	-1,81	0,02016	NM_002379	XIRP2
8046062	-1,00	-2,00	0,03735	-1,41	-2,67	0,00216	NM_152381	---
8055294	-1,00	-2,00	0,25451	-1,06	-2,09	0,05241	---	IDI2
7931748	-1,00	-2,00	0,00107	-0,90	-1,87	0,04344	NM_033261	PCDH1
8114767	-1,00	-2,00	0,01679	-1,87	-3,66	0,00034	NM_032420	RND2
8007454	-1,00	-2,00	0,00302	1,53	2,89	0,05515	NM_005440	---
7917431	-1,00	-2,00	0,03579	-1,16	-2,23	0,00564	---	DLGAP4
8062293	-1,00	-2,00	0,05269	-1,03	-2,04	0,08716	NM_014902	MFSD2A
7900365	-1,00	-2,00	0,10433	0,33	1,25	0,17954	NM_001136493	ATG16L2
7942381	-1,00	-2,00	0,14150	-0,23	-1,17	0,47534	NM_033388	NUDT10
8167645	-1,00	-2,00	0,06847	-1,91	-3,75	0,00040	NM_153183	LOC100133207
7976554	-1,00	-2,00	0,01807	-1,52	-2,87	0,00168	ENST00000420674	EMILIN3
8066294	-1,00	-2,00	0,01079	-1,05	-2,07	0,00337	NM_052846	KCNN4
8037408	-1,00	-2,00	0,02817	0,13	1,10	0,41125	NM_002250	PROKR2
8064859	-1,00	-2,00	0,14227	-1,21	-2,31	0,05609	NM_144773	KRTAP13-1
8068130	-1,00	-2,00	0,05000	-1,32	-2,49	0,00656	NM_181599	NRG2
8114550	-1,00	-2,00	0,07975	-0,73	-1,66	0,03036	NM_004883	SH3GL2
8154476	-1,00	-2,00	0,02229	0,14	1,10	0,65778	NM_003026	C5orf60
8116313	-1,00	-2,00	0,21381	1,12	2,17	0,01814	NM_001142306	---
8086417	-1,00	-2,00	0,30930	-1,17	-2,25	0,05252	---	---
7930137	-1,00	-2,00	0,00516	-0,93	-1,91	0,00616	---	C1orf65
7909965	-1,00	-2,00	0,09351	-1,61	-3,05	0,00041	NM_152610	MIR27B
8156571	-1,00	-2,00	0,05335	-0,45	-1,37	0,01775	NR_029665	ZBTB7C
8023212	-1,00	-2,00	0,04816	-1,27	-2,42	0,00046	NM_001039360	---
7947926	-1,00	-2,00	0,02439	-1,24	-2,36	0,00233	---	GPR158
7926715	-1,00	-2,00	0,03127	-0,86	-1,81	0,01495	NM_020752	---
8173890	-1,00	-2,00	0,05348	-0,98	-1,97	0,00301	---	---
8035863	-1,00	-2,00	0,18007	-1,26	-2,39	0,00952	---	---
8164194	-1,00	-2,00	0,09456	-0,47	-1,39	0,14980	---	---
7962236	-1,00	-2,00	0,03000	-1,21	-2,32	0,00234	---	---
8098496	-1,00	-2,00	0,10173	-1,22	-2,33	0,00256	---	---
7972979	-1,00	-2,00	0,11489	-1,15	-2,22	0,00010	---	---
8163712	-1,00	-2,00	0,09295	-1,41	-2,65	0,00014	---	TRIM64
7943031	-1,00	-2,00	0,18573	-1,05	-2,07	0,13454	NM_001136486	TRIM64
7950965	-1,00	-2,00	0,18573	-1,05	-2,07	0,13454	NM_001136486	RBM38
8067203	-1,00	-2,01	0,07089	-0,83	-1,78	0,02370	NM_017495	SCARNA8
8160295	-1,00	-2,01	0,07479	-1,72	-3,30	0,01040	NR_003009	---
7963233	-1,00	-2,01	0,00650	-0,54	-1,45	0,00432	---	GPRIN2
7927277	-1,00	-2,01	0,15879	-1,09	-2,13	0,16349	NM_014696	JPH3
7997746	-1,00	-2,01	0,03933	-1,54	-2,90	0,01115	NM_020655	ZNF572
8148276	-1,00	-2,01	0,08993	-0,48	-1,40	0,38458	NM_152412	---
7911213	-1,00	-2,01	0,03465	-0,82	-1,76	0,12868	---	RIMBP3B
8074591	-1,00	-2,01	0,07466	0,24	1,18	0,60967	ENST00000357029	AKT1S1
8038477	-1,00	-2,01	0,05630	-0,53	-1,45	0,41431	NM_032375	PARP10
8153609	-1,00	-2,01	0,00182	-1,14	-2,20	0,03888	NM_032789	RRP1
8068938	-1,01	-2,01	0,00460	-0,02	-1,02	0,95926	NM_003683	GOLGA2P2
8176910	-1,01	-2,01	0,05999	-0,58	-1,49	0,00483	NR_001555	GOLGA2P2
8177413	-1,01	-2,01	0,05999	-0,58	-1,49	0,00483	NR_001555	NHLRC4
7991898	-1,01	-2,01	0,01031	-1,21	-2,31	0,03690	NM_176677	ZBP1
8067221	-1,01	-2,01	0,05690	-1,11	-2,15	0,00089	NM_030776	DLG2
7950764	-1,01	-2,01	0,02816	-1,30	-2,46	0,00032	NM_001364	SUV420H2
8031466	-1,01	-2,01	0,08076	-0,17	-1,13	0,83884	NM_032701	DDX54
7966570	-1,01	-2,01	0,02194	0,62	1,54	0,31212	NM_001111322	---

7946008	-1,01	-2,01	0,19498	-1,46	-2,75	0,00195	---	MIR1-1
8063921	-1,01	-2,01	0,00712	-1,16	-2,24	0,02118	NR_029780	---
8094899	-1,01	-2,01	0,17319	-0,90	-1,87	0,07954	---	GABRA6
8109651	-1,01	-2,01	0,04070	-1,37	-2,58	0,00420	NM_000811	---
8049042	-1,01	-2,01	0,09604	-0,99	-1,99	0,02717	---	FBXL6
8153709	-1,01	-2,01	0,00720	0,03	1,02	0,96979	NM_012162	GSTA1
8127072	-1,01	-2,01	0,01645	-0,95	-1,94	0,00238	NM_145740	C21orf74
8068006	-1,01	-2,01	0,02373	-0,70	-1,62	0,06133	AY077696	---
8155596	-1,01	-2,01	0,53165	-5,40	-42,18	0,00005	---	---
8092132	-1,01	-2,01	0,03734	-0,55	-1,46	0,28544	---	SDK2
8018114	-1,01	-2,01	0,00605	-0,58	-1,50	0,02339	NM_001144952	PLA2G2C
7913235	-1,01	-2,01	0,04260	-1,82	-3,54	0,00765	NM_001105572	PSPN
8033159	-1,01	-2,01	0,03361	0,48	1,40	0,29980	NM_004158	AVPR1B
7909155	-1,01	-2,01	0,09450	-0,94	-1,92	0,11242	NM_000707	IGKC
8043476	-1,01	-2,01	0,17476	-2,18	-4,52	0,00022	ENST00000390273	SLC9A2
8044080	-1,01	-2,01	0,05294	1,01	2,01	0,00393	NM_003048	SSTR4
8061366	-1,01	-2,01	0,13339	-1,16	-2,24	0,02331	NM_001052	EIF4E3
8088803	-1,01	-2,01	0,05314	0,78	1,72	0,03740	NM_173359	SCARNA9L
8171760	-1,01	-2,01	0,04277	-0,44	-1,36	0,31028	NR_023358	---
8011112	-1,01	-2,01	0,07836	-1,66	-3,16	0,00412	---	CCDC81
7942941	-1,01	-2,01	0,04761	-0,97	-1,96	0,01378	NM_001156474	---
7998399	-1,01	-2,01	0,01411	-0,62	-1,53	0,02561	---	VMO1
8011724	-1,01	-2,01	0,02894	-0,26	-1,20	0,40066	NM_182566	AFAP1L2
7936439	-1,01	-2,01	0,05938	0,18	1,13	0,66559	NM_001001936	C6orf114
8123981	-1,01	-2,01	0,00156	-1,23	-2,34	0,01108	AF264036	---
8042159	-1,01	-2,01	0,01083	-1,19	-2,27	0,03686	---	OR6K3
7921422	-1,01	-2,01	0,05762	-1,09	-2,13	0,10731	NM_001005327	CLEC14A
7978712	-1,01	-2,01	0,11210	-0,88	-1,84	0,03127	NM_175060	---
8006323	-1,01	-2,01	0,03936	-1,60	-3,02	0,00192	---	PPP1R9A
8134351	-1,01	-2,01	0,05489	1,18	2,26	0,03160	NM_001166160	ZSWIM4
8026198	-1,01	-2,01	0,05493	-0,73	-1,66	0,20780	NM_023072	FLJ43859
8156098	-1,01	-2,01	0,00216	-1,43	-2,69	0,01466	NM_001145197	FAM27E3
8161446	-1,01	-2,01	0,09507	0,75	1,69	0,01065	BC032035	---
8093152	-1,01	-2,01	0,00213	-0,89	-1,85	0,00126	---	GHDC
8015575	-1,01	-2,01	0,03961	-0,93	-1,91	0,01048	NR_024573	---
8016042	-1,01	-2,01	0,34650	-2,16	-4,47	0,08307	---	---
8129315	-1,01	-2,01	0,13558	-1,84	-3,58	0,01154	---	LPA
8130608	-1,01	-2,01	0,03074	-0,94	-1,92	0,08204	NM_005577	---
7909813	-1,01	-2,01	0,07249	-1,30	-2,46	0,00703	---	RNU2-1
8019631	-1,01	-2,01	0,46387	-2,57	-5,92	0,00443	NR_002716	RNU2-1
8019633	-1,01	-2,01	0,46387	-2,57	-5,92	0,00443	NR_002716	RNU2-1
8019635	-1,01	-2,01	0,46387	-2,57	-5,92	0,00443	NR_002716	RNU2-1
8019637	-1,01	-2,01	0,46387	-2,57	-5,92	0,00443	NR_002716	RNU2-1
8019639	-1,01	-2,01	0,46387	-2,57	-5,92	0,00443	NR_002716	RNU2-1
8019641	-1,01	-2,01	0,46387	-2,57	-5,92	0,00443	NR_002716	RNU2-1
8019703	-1,01	-2,01	0,46387	-2,57	-5,92	0,00443	NR_002716	RNU2-1
8019705	-1,01	-2,01	0,46387	-2,57	-5,92	0,00443	NR_002716	RNU2-1
8019707	-1,01	-2,01	0,46387	-2,57	-5,92	0,00443	NR_002716	C8B
7916526	-1,01	-2,01	0,01425	-0,87	-1,83	0,00700	NM_000066	PCDH11Y
8176400	-1,01	-2,01	0,05335	-1,28	-2,42	0,00079	NM_032971	BRDT
7902934	-1,01	-2,01	0,02339	3,03	8,16	0,00221	NM_207189	---
7931387	-1,01	-2,01	0,22743	-1,37	-2,59	0,00363	---	RIPK3
7978312	-1,01	-2,01	0,00607	-1,45	-2,73	0,01525	NM_006871	MSLN
7992071	-1,01	-2,01	0,07287	-1,97	-3,92	0,00601	NM_005823	IL16
7985364	-1,01	-2,01	0,11266	-1,36	-2,56	0,00870	NM_172217	TYR
7948085	-1,01	-2,01	0,05241	-1,34	-2,53	0,00042	NM_000372	C18orf12
8021152	-1,01	-2,01	0,00566	-1,73	-3,31	0,00248	AB027121	---
8065016	-1,01	-2,01	0,00273	-1,62	-3,08	0,00225	---	---
7972563	-1,01	-2,01	0,00006	-1,26	-2,40	0,01381	---	OXER1
8051793	-1,01	-2,01	0,13485	-0,66	-1,58	0,12621	NM_148962	KCNJ5
7945146	-1,01	-2,01	0,06791	-1,13	-2,19	0,00056	NM_000890	---
8124455	-1,01	-2,01	0,20543	-0,70	-1,63	0,00109	---	FOXS1
8065576	-1,01	-2,01	0,06141	-1,65	-3,14	0,00068	NM_004118	---
8093035	-1,01	-2,01	0,11771	-1,59	-3,02	0,00437	---	IGKC // IGKC
8043431	-1,01	-2,01	0,31639	-3,02	-8,09	0,01142	AF113887 // AF113887	CCDC108
8059028	-1,01	-2,02	0,01299	-1,29	-2,44	0,00305	NM_194302	HTR2C
8169447	-1,01	-2,02	0,00136	-1,03	-2,04	0,09419	NM_000868	KRT20
8015124	-1,01	-2,02	0,03014	-0,97	-1,95	0,00796	NM_019010	ATOH1
8096461	-1,01	-2,02	0,24882	-1,66	-3,15	0,00043	NM_005172	---
8092239	-1,01	-2,02	0,03059	-1,29	-2,44	0,04012	---	MIF4GD
8018343	-1,01	-2,02	0,03028	2,24	4,72	0,00122	NM_020679	---
8156088	-1,01	-2,02	0,08368	-0,45	-1,37	0,01308	---	---
8055967	-1,01	-2,02	0,10035	-1,05	-2,07	0,06304	---	---

7910492	-1,01	-2,02	0,05384	-1,02	-2,02	0,00294	---	RABEP2
8000616	-1,01	-2,02	0,00671	-1,49	-2,81	0,00434	NM_024816	SLC22A3
8123246	-1,01	-2,02	0,02181	0,86	1,81	0,06055	NM_021977	S100A12
7920238	-1,01	-2,02	0,08343	-1,50	-2,83	0,05311	NM_005621	GABRA1
8109663	-1,01	-2,02	0,02453	-1,01	-2,01	0,00145	NM_000806	CDKN2D
8034075	-1,01	-2,02	0,09084	-1,61	-3,05	0,01289	NM_001800	CST8
8061389	-1,01	-2,02	0,01032	-1,31	-2,48	0,00134	NM_005492	TFAP2E
7900001	-1,01	-2,02	0,05653	-1,47	-2,78	0,02827	NM_178548	C1orf150
7911199	-1,01	-2,02	0,03991	-1,44	-2,72	0,01266	NM_145278	---
7955987	-1,01	-2,02	0,47796	-1,47	-2,77	0,00084	---	---
7938878	-1,01	-2,02	0,05411	-1,34	-2,54	0,02057	---	HIST2H3A
7905085	-1,01	-2,02	0,01844	0,45	1,37	0,15749	NM_001005464	HIST2H3A
7919614	-1,01	-2,02	0,01844	0,45	1,37	0,15749	NM_001005464	DNAJB8
8090464	-1,01	-2,02	0,04417	-1,24	-2,37	0,04173	NM_153330	ADRA1B
8109612	-1,01	-2,02	0,01454	-0,35	-1,27	0,26097	NM_000679	---
8162529	-1,01	-2,02	0,11752	-1,61	-3,05	0,00286	---	---
8166661	-1,01	-2,02	0,07004	-0,53	-1,45	0,09447	---	---
8018814	-1,01	-2,02	0,06134	-1,18	-2,27	0,05181	---	HEATR7A
8148808	-1,01	-2,02	0,13360	-0,13	-1,09	0,83645	NM_032450	GUCA1B
8126387	-1,01	-2,02	0,01614	-0,68	-1,60	0,30459	NM_002098	---
8021438	-1,01	-2,02	0,05905	-0,59	-1,51	0,07449	---	---
8169439	-1,01	-2,02	0,05239	-0,59	-1,51	0,12041	---	GSTA2
8127065	-1,01	-2,02	0,16289	-0,25	-1,19	0,04509	NM_000846	LRTM2
7953032	-1,01	-2,02	0,02162	-1,30	-2,47	0,01924	NM_001039029	---
8057819	-1,01	-2,02	0,09400	-0,87	-1,83	0,04041	---	---
7944339	-1,01	-2,02	0,02279	-0,84	-1,78	0,08147	---	EIF4A2
8084704	-1,01	-2,02	0,03559	-1,38	-2,60	0,02209	AB209021	SNORD116-22
7981992	-1,01	-2,02	0,01924	-0,55	-1,46	0,29914	NR_003336	TDRG1
8119386	-1,01	-2,02	0,09365	-1,81	-3,50	0,00364	DQ168992	---
7956279	-1,01	-2,02	0,12738	-1,19	-2,28	0,00853	---	GNB1L
8074481	-1,01	-2,02	0,01039	-1,42	-2,67	0,00670	NM_053004	---
8021482	-1,01	-2,02	0,00964	-1,36	-2,56	0,00729	---	HIST1H2BN
8117600	-1,01	-2,02	0,15315	0,55	1,46	0,16543	NM_003520	TELO2
7992271	-1,01	-2,02	0,14198	-0,58	-1,50	0,18974	NM_016111	NCRNA00200
7925870	-1,01	-2,02	0,01109	-1,70	-3,25	0,00021	NR_015376	R3HDML
8062813	-1,01	-2,02	0,05192	-0,67	-1,59	0,11963	NM_178491	---
8037003	-1,01	-2,02	0,09084	-1,60	-3,02	0,04212	---	SLC2A4RG
8064175	-1,01	-2,02	0,03655	-0,45	-1,36	0,13171	NM_020062	FAM9C
8171338	-1,01	-2,02	0,00776	-1,67	-3,18	0,01337	NM_174901	FSTL5
8103466	-1,01	-2,02	0,08730	-0,74	-1,66	0,01113	NM_020116	SGSM1
8071953	-1,01	-2,02	0,16439	-0,85	-1,80	0,00149	NM_001039948	CD70
8033241	-1,01	-2,02	0,15996	-0,90	-1,87	0,04183	NM_001252	SNX18
8105328	-1,01	-2,02	0,04421	-1,11	-2,16	0,00884	NM_052870	---
8109973	-1,01	-2,02	0,00531	0,49	1,41	0,21090	---	---
8119580	-1,01	-2,02	0,09086	-0,67	-1,59	0,00384	---	NIPAL1
8094938	-1,01	-2,02	0,04631	1,13	2,19	0,05508	NM_207330	---
7915731	-1,02	-2,02	0,00984	-0,57	-1,49	0,01472	---	CTCFL
8067208	-1,02	-2,02	0,05315	-0,97	-1,96	0,00095	NM_080618	ATP13A4
8092800	-1,02	-2,02	0,01311	-0,66	-1,58	0,00033	NM_032279	RTP2
8092686	-1,02	-2,02	0,18809	-1,15	-2,21	0,04337	NM_001004312	FAM78B
7922001	-1,02	-2,02	0,00406	-1,40	-2,63	0,01807	NM_001017961	DNAJB7
8076292	-1,02	-2,02	0,08993	-1,40	-2,64	0,00002	NM_145174	WNT1
7955170	-1,02	-2,02	0,12929	-1,19	-2,28	0,12038	NM_005430	---
7980576	-1,02	-2,02	0,08060	-1,47	-2,77	0,01182	---	CHRNB3
8146230	-1,02	-2,02	0,00466	-1,36	-2,56	0,04304	NM_000749	CXorf50B
8168387	-1,02	-2,02	0,08173	-0,09	-1,06	0,85995	NR_026594	MIR133A1
8022498	-1,02	-2,02	0,14778	-1,35	-2,55	0,04147	NR_029675	FAM27E3
8155514	-1,02	-2,02	0,12593	0,86	1,81	0,00171	BC032035	PPM1H
7964646	-1,02	-2,02	0,00870	0,36	1,28	0,52084	NM_020700	---
7993632	-1,02	-2,02	0,01529	-1,46	-2,75	0,04599	---	C16orf47
8002706	-1,02	-2,02	0,07087	-1,57	-2,97	0,01565	AK129695	KCNK3
8040695	-1,02	-2,02	0,04422	0,25	1,19	0,73093	NM_002246	TAS1R1
7897295	-1,02	-2,02	0,03277	-1,49	-2,82	0,00288	NM_138697	PCSK1
8113234	-1,02	-2,02	0,00361	-0,69	-1,61	0,05027	NM_000439	TIGD5
8148655	-1,02	-2,02	0,02079	0,64	1,55	0,19828	NM_032862	STAP1
8095343	-1,02	-2,02	0,00716	-0,97	-1,96	0,00075	NM_012108	---
8017141	-1,02	-2,02	0,00884	-0,84	-1,79	0,00168	---	---
8001102	-1,02	-2,02	0,16885	-1,36	-2,57	0,00008	---	AFF3
8054254	-1,02	-2,02	0,01283	0,01	1,01	0,95874	NM_002285	FZD10
7959850	-1,02	-2,02	0,04218	-1,52	-2,86	0,00880	NM_007197	---
7959249	-1,02	-2,02	0,10473	-0,75	-1,68	0,11798	---	FRG2
8104124	-1,02	-2,02	0,20159	-2,06	-4,17	0,00412	NM_001005217	OR2G3
7911209	-1,02	-2,02	0,19753	-1,09	-2,12	0,02767	NM_001001914	BLNK

7935270	-1,02	-2,02	0,01350	-0,98	-1,98	0,04231	NM_013314	ANKRD36B
8053834	-1,02	-2,02	0,11914	1,71	3,28	0,03053	NM_025190	FAM90A10
8144448	-1,02	-2,03	0,47047	-2,17	-4,49	0,00817	NM_001164447	---
8081105	-1,02	-2,03	0,00120	-1,35	-2,55	0,06129	---	---
7927597	-1,02	-2,03	0,02664	-0,65	-1,57	0,03761	---	CALML5
7931859	-1,02	-2,03	0,01379	-1,32	-2,50	0,05707	NM_017422	PLEKHG7
7957514	-1,02	-2,03	0,04125	-0,70	-1,62	0,02731	NM_001004330	---
7969609	-1,02	-2,03	0,00538	-0,50	-1,41	0,03786	---	GOLGA6L10
7990943	-1,02	-2,03	0,40386	-0,39	-1,31	0,43710	NM_001164465	ACCN1
8014144	-1,02	-2,03	0,01792	-1,86	-3,62	0,01491	NM_183377	CLPP
8025028	-1,02	-2,03	0,09767	0,38	1,30	0,24319	NM_006012	DUSP5P
7910379	-1,02	-2,03	0,07291	0,46	1,38	0,03119	NR_002834	C19orf36
8024373	-1,02	-2,03	0,13209	-0,56	-1,48	0,06102	NM_001039846	---
7965721	-1,02	-2,03	0,00644	-0,86	-1,81	0,03700	---	C8orf74
8144577	-1,02	-2,03	0,05077	-2,02	-4,05	0,00293	NM_001040032	C1orf130
7898967	-1,02	-2,03	0,04809	-0,31	-1,24	0,57099	NM_001010980	EFCAB1
8150691	-1,02	-2,03	0,09368	-0,57	-1,48	0,02704	NM_024593	---
8160256	-1,02	-2,03	0,02682	-1,05	-2,07	0,00114	---	C11orf42
7938078	-1,02	-2,03	0,00721	-1,70	-3,25	0,03754	NM_173525	CDY1
8176671	-1,02	-2,03	0,07292	-1,36	-2,56	0,00314	NM_170723	AGBL1
7985741	-1,02	-2,03	0,00608	-0,63	-1,55	0,01167	NM_152336	FAM75A7
8161409	-1,02	-2,03	0,06022	-1,70	-3,25	0,00168	NM_015667	DDI1
7943521	-1,02	-2,03	0,06932	-1,30	-2,46	0,00145	NM_001001711	DNAJC4
7949058	-1,02	-2,03	0,00647	-1,07	-2,10	0,10087	NM_005528	---
7899988	-1,02	-2,03	0,01063	-0,66	-1,58	0,00389	---	RIMBP2
7967660	-1,02	-2,03	0,03590	-0,67	-1,59	0,23852	NM_015347	TMC3
7990848	-1,02	-2,03	0,01596	-2,05	-4,14	0,00195	NM_001080532	---
7972661	-1,02	-2,03	0,06434	-1,31	-2,48	0,00679	---	FLJ42102
7950064	-1,02	-2,03	0,06917	-0,57	-1,48	0,42625	ENST00000331301	ENPP7
8010313	-1,02	-2,03	0,05439	-2,11	-4,31	0,00167	NM_178543	GPC3
8175234	-1,02	-2,03	0,07063	-1,07	-2,10	0,12009	NM_001164617	LHFPL1
8174568	-1,02	-2,03	0,01989	-1,36	-2,56	0,05979	NM_178175	---
8102948	-1,02	-2,03	0,07713	-0,79	-1,73	0,01815	---	---
8068662	-1,02	-2,03	0,00720	-0,91	-1,87	0,00336	---	OSGIN1
7997533	-1,02	-2,03	0,10576	-1,61	-3,05	0,01832	NM_013370	MTMR8
8173245	-1,02	-2,03	0,16223	0,18	1,13	0,69796	NM_017677	---
8020027	-1,02	-2,03	0,20143	-1,36	-2,58	0,00379	---	---
8080985	-1,02	-2,03	0,12461	-1,33	-2,52	0,00076	---	SOX14
8082926	-1,02	-2,03	0,06383	-1,53	-2,89	0,00130	NM_004189	---
7919852	-1,02	-2,03	0,30402	-0,51	-1,43	0,02207	---	FAM22A
7928915	-1,02	-2,03	0,06192	-0,81	-1,75	0,07715	NM_001099338	---
8123737	-1,02	-2,03	0,05020	-0,97	-1,96	0,08722	---	---
7948161	-1,02	-2,03	0,10994	-1,68	-3,20	0,00578	---	EEFSEC
8082422	-1,02	-2,03	0,02562	0,62	1,54	0,09196	NM_021937	NAPSB
8038547	-1,02	-2,03	0,01019	-0,90	-1,87	0,05457	NR_002798	FAM186A
7963203	-1,02	-2,03	0,09026	-1,47	-2,77	0,01556	NM_001145475	STXBP2
8025255	-1,02	-2,03	0,04532	0,96	1,95	0,08713	NM_006949	SMO
8136080	-1,02	-2,03	0,08938	-0,46	-1,37	0,21307	NM_005631	---
7983525	-1,02	-2,03	0,04249	-0,35	-1,27	0,27054	---	---
7974617	-1,02	-2,03	0,02167	-0,72	-1,64	0,00837	---	SLC10A4
8094960	-1,02	-2,03	0,00049	0,04	1,03	0,92007	NM_152679	TG
8148385	-1,02	-2,03	0,00373	-1,17	-2,24	0,01821	NM_003235	SFRP5
7935528	-1,02	-2,03	0,15994	-1,41	-2,66	0,00139	NM_003015	---
8036027	-1,02	-2,03	0,01723	-0,70	-1,62	0,16200	---	---
8005245	-1,02	-2,03	0,02025	-1,42	-2,67	0,00185	---	DNAH6
8043059	-1,02	-2,03	0,00575	-0,99	-1,99	0,00266	NM_001370	MIR194-2
7949275	-1,02	-2,03	0,12499	-0,99	-1,99	0,07633	NR_029829	---
8124181	-1,02	-2,03	0,01846	-1,09	-2,13	0,00560	---	APOC4
8029547	-1,02	-2,03	0,07832	-1,39	-2,63	0,01565	NM_001646	SLA2
8066038	-1,02	-2,03	0,05950	-0,98	-1,98	0,00395	NM_032214	---
7924497	-1,02	-2,03	0,02079	-1,24	-2,37	0,00867	---	PCSK6
7991602	-1,02	-2,03	0,00621	1,31	2,47	0,00643	NM_002570	---
8173611	-1,02	-2,03	0,00643	-0,71	-1,64	0,07022	---	GALNTL4
7946641	-1,02	-2,03	0,03004	-0,52	-1,44	0,21603	NM_198516	---
7976069	-1,02	-2,03	0,28994	-0,77	-1,70	0,02143	---	TCP11
8125870	-1,02	-2,03	0,12098	-1,51	-2,84	0,00777	NM_001093728	---
8032273	-1,02	-2,03	0,00165	-0,95	-1,93	0,02389	---	---
7906784	-1,02	-2,03	0,01526	-1,01	-2,01	0,00013	---	CLDN3
8140140	-1,02	-2,03	0,19373	1,40	2,63	0,00269	NM_001306	RSPH10B
8131452	-1,02	-2,03	0,03704	-0,57	-1,49	0,12506	NM_173565	SPINT3
8066530	-1,02	-2,03	0,00682	-1,15	-2,21	0,02460	NM_006652	---
8109591	-1,02	-2,03	0,11756	-1,00	-2,00	0,00594	---	---
8099539	-1,02	-2,03	0,15906	-1,53	-2,89	0,01046	---	DGAT2L6

8168098	-1,02	-2,03	0,00179	-1,12	-2,17	0,00276	NM_198512	C15orf56
7987472	-1,02	-2,03	0,13005	-0,88	-1,84	0,01200	NM_001039905	CSF2RB
8072757	-1,02	-2,03	0,04781	-1,60	-3,02	0,00034	NM_000395	LOC100132178
8031566	-1,02	-2,03	0,00700	-1,06	-2,08	0,03343	ENST00000413847	---
7982388	-1,02	-2,03	0,00007	-1,20	-2,29	0,00875	---	RGL4
8071717	-1,02	-2,03	0,02890	-1,30	-2,46	0,04532	NM_153615	KRT36
8015312	-1,02	-2,03	0,00010	-1,18	-2,27	0,00542	NM_003771	MIRLET7C
8067944	-1,02	-2,03	0,01835	-1,44	-2,72	0,00048	NR_029480	---
7923177	-1,03	-2,04	0,00023	-0,73	-1,66	0,00744	---	CCDC103
8007707	-1,03	-2,04	0,01966	-0,37	-1,29	0,28121	NM_213607	RPL22L1
8092067	-1,03	-2,04	0,14266	-0,11	-1,08	0,27163	NM_001099645	MDP1
7978192	-1,03	-2,04	0,00222	-0,42	-1,34	0,08208	NM_138476	NUDT16L1
7993019	-1,03	-2,04	0,00425	-0,57	-1,48	0,19539	NM_032349	PP13439
8092162	-1,03	-2,04	0,07155	-1,93	-3,82	0,00221	ENST00000331659	LOC157740
8149320	-1,03	-2,04	0,07747	-0,83	-1,78	0,07628	AJ291676	---
7898294	-1,03	-2,04	0,13367	-1,10	-2,14	0,00467	---	---
8022340	-1,03	-2,04	0,03629	-1,53	-2,89	0,03213	---	TSPAN18
7939559	-1,03	-2,04	0,11818	1,01	2,02	0,02729	NM_130783	CCDC57
8019437	-1,03	-2,04	0,00367	-1,04	-2,06	0,01967	NM_198082	MCM3APAS
8069332	-1,03	-2,04	0,01151	-0,05	-1,03	0,85737	NR_002776	KCNE1
8070173	-1,03	-2,04	0,18016	-1,53	-2,90	0,02717	NM_000219	FXYD4
7927155	-1,03	-2,04	0,02132	-1,36	-2,57	0,00568	NM_173160	SLC38A8
8003077	-1,03	-2,04	0,02268	-2,20	-4,59	0,00306	NM_001080442	CXCL9
8101118	-1,03	-2,04	0,05882	-1,18	-2,26	0,01268	NM_002416	ATHL1
7937314	-1,03	-2,04	0,00904	0,02	1,01	0,96980	NM_025092	HTR1F
8081067	-1,03	-2,04	0,01523	-1,11	-2,16	0,01291	NM_000866	---
8152115	-1,03	-2,04	0,03627	-1,19	-2,28	0,00009	---	TBPL2
7979341	-1,03	-2,04	0,21465	-1,36	-2,57	0,00314	NM_199047	---
7920256	-1,03	-2,04	0,06371	-1,13	-2,20	0,15274	---	FAM167A
8149324	-1,03	-2,04	0,04714	0,19	1,14	0,55692	NM_053279	PACS2
7977371	-1,03	-2,04	0,06964	-1,22	-2,34	0,04831	NM_001100913	---
8008704	-1,03	-2,04	0,07275	-2,14	-4,42	0,00275	---	---
7955132	-1,03	-2,04	0,07000	-1,36	-2,56	0,01042	---	CHCHD4
8085481	-1,03	-2,04	0,02984	-0,49	-1,40	0,22815	NM_144636	PHACTR3
8063739	-1,03	-2,04	0,07458	-0,77	-1,70	0,00759	NM_080672	---
8113035	-1,03	-2,04	0,04508	-0,33	-1,26	0,03202	---	HAUS7
8175782	-1,03	-2,04	0,04748	-0,40	-1,32	0,13567	NM_017518	KLKB1
8098654	-1,03	-2,04	0,03665	-0,78	-1,72	0,07563	NM_000892	GRAP
8013319	-1,03	-2,04	0,01688	-1,81	-3,50	0,01912	NM_006613	DNAI2
8009585	-1,03	-2,04	0,05481	-1,13	-2,19	0,04781	NM_023036	GPR87
8091515	-1,03	-2,04	0,04319	0,30	1,23	0,50663	NM_023915	C11orf84
7940790	-1,03	-2,04	0,09703	1,00	2,00	0,11228	BC056402	INCENP
7940600	-1,03	-2,04	0,00414	0,15	1,11	0,40984	NM_001040694	---
7942326	-1,03	-2,04	0,05563	-1,25	-2,38	0,00820	---	SLC23A3
8059081	-1,03	-2,04	0,00141	-0,89	-1,86	0,00563	NM_001144889	---
8065321	-1,03	-2,04	0,05782	-0,97	-1,96	0,09227	---	PHLDA2
7945781	-1,03	-2,04	0,01177	-0,57	-1,48	0,06649	NM_003311	---
8051862	-1,03	-2,04	0,02663	-0,76	-1,69	0,00050	---	PABPC1L
8062890	-1,03	-2,04	0,06122	2,12	4,34	0,00116	NM_001124756	STK38
8119076	-1,03	-2,04	0,14366	-0,29	-1,22	0,49993	NM_007271	PNMA3
8170622	-1,03	-2,04	0,03266	-1,67	-3,19	0,03000	NM_013364	---
8088998	-1,03	-2,04	0,03494	-0,79	-1,73	0,02119	---	---
8105629	-1,03	-2,04	0,09160	-1,28	-2,43	0,02448	---	CCL13
8006459	-1,03	-2,04	0,01314	-0,77	-1,70	0,03929	NM_005408	ST6GAL2
8054439	-1,03	-2,04	0,12392	-0,95	-1,93	0,01827	NM_032528	LOC441242
8139845	-1,03	-2,04	0,20409	-1,56	-2,94	0,04536	ENST00000439845	---
8150489	-1,03	-2,04	0,03352	-0,76	-1,70	0,02419	---	DEFB116
8065552	-1,03	-2,04	0,03422	-1,52	-2,88	0,00447	NM_001037731	LOC401010
8055281	-1,03	-2,04	0,27478	-0,63	-1,55	0,23925	NR_002826	---
8157018	-1,03	-2,04	0,14948	-1,10	-2,14	0,01367	---	MAEL
7907032	-1,03	-2,04	0,00139	-0,74	-1,67	0,00364	NM_032858	---
7965429	-1,03	-2,04	0,39567	-0,67	-1,59	0,09459	---	KLC3
8029671	-1,03	-2,04	0,02763	-1,60	-3,03	0,00006	NM_177417	---
8148156	-1,03	-2,04	0,10570	-1,74	-3,35	0,01462	---	---
8088828	-1,03	-2,04	0,02119	-0,99	-1,98	0,03432	---	VIPR1
8079060	-1,03	-2,04	0,02765	-0,91	-1,87	0,06695	NM_004624	SNORD115-10
7982026	-1,03	-2,04	0,01544	-0,92	-1,89	0,09197	NR_003302	FAM25B
7927363	-1,03	-2,04	0,25678	-1,78	-3,45	0,04140	NM_001137556	FAM25B
7933327	-1,03	-2,04	0,25678	-1,78	-3,45	0,04140	NM_001137556	FAM25B
7933423	-1,03	-2,04	0,25678	-1,78	-3,45	0,04140	NM_001137556	---
7916880	-1,03	-2,04	0,01195	-1,14	-2,20	0,00619	---	PXN
7967021	-1,03	-2,04	0,08003	-0,53	-1,44	0,38315	BC052611	FAM193B
8116113	-1,03	-2,04	0,11846	-0,76	-1,69	0,13102	NR_024019	---

8075505	-1,03	-2,04	0,05875	-2,02	-4,05	0,00883	---	B3GNT4	
7959402	-1,03	-2,04	0,02790	-0,42	-1,34	0,19198	NM_030765		SNORD115-31
7982068	-1,03	-2,04	0,04001	-0,89	-1,85	0,03387	NR_003346		FBF1
8018522	-1,03	-2,04	0,00758	-0,26	-1,20	0,08802	NM_001080542		RBMY1B
8177303	-1,03	-2,04	0,01650	-1,19	-2,28	0,00459	NM_001006121		C14orf19
7973900	-1,03	-2,04	0,08514	0,54	1,46	0,15921	NR_002937		NEUROG2
8102368	-1,03	-2,04	0,00991	-0,16	-1,12	0,55659	NM_024019		BTNL3
8110620	-1,03	-2,04	0,02040	-0,94	-1,92	0,02490	NM_197975		---
7970922	-1,03	-2,04	0,09254	-1,49	-2,80	0,00089	---	IGK@	
8043449	-1,03	-2,04	0,18404	-1,19	-2,29	0,02351	BC030813	---	
8123889	-1,03	-2,04	0,00339	-0,87	-1,83	0,00389	---	---	
7939934	-1,03	-2,04	0,03514	-1,77	-3,42	0,00005	---	SLC24A2	
8160321	-1,03	-2,04	0,03195	-1,11	-2,16	0,00331	NM_020344		PCNXL3
7941350	-1,03	-2,04	0,00591	1,36	2,57	0,02494	NM_032223		---
7962893	-1,03	-2,04	0,65761	-0,22	-1,16	0,46472	---	DYSFIP1	
8019243	-1,03	-2,04	0,05578	-1,55	-2,93	0,00127	NM_001007533		PGA3
7940421	-1,03	-2,04	0,25886	-1,76	-3,39	0,02944	NM_001079807		---
8144042	-1,03	-2,04	0,38775	-0,98	-1,97	0,08004	---	VPS72	
7919940	-1,03	-2,04	0,17040	2,56	5,88	0,00890	NM_005997		FCAR
8031374	-1,03	-2,04	0,03087	-1,61	-3,05	0,00273	NM_002000		CHST8
8027574	-1,03	-2,04	0,01017	-1,11	-2,16	0,04271	NM_022467		---
8124446	-1,03	-2,04	0,06513	-0,36	-1,29	0,11642	---	MIR429	
7896863	-1,03	-2,04	0,06267	-1,22	-2,33	0,02197	NR_029957		---
8143268	-1,03	-2,04	0,04536	-1,03	-2,04	0,00005	---	RPH3AL	
8010903	-1,03	-2,04	0,10925	-0,43	-1,35	0,33519	NM_006987		POLD1
8030641	-1,03	-2,04	0,02945	0,12	1,09	0,80369	NM_002691		---
8122885	-1,03	-2,04	0,07791	-1,65	-3,15	0,00661	---	FES	
7986110	-1,03	-2,04	0,02039	-0,73	-1,65	0,00431	NM_002005		---
8114352	-1,03	-2,04	0,01705	-0,73	-1,66	0,00380	---	PTCHD2	
7897691	-1,03	-2,04	0,03243	-1,43	-2,70	0,11206	NM_020780		TMEM17
8052585	-1,03	-2,05	0,05007	1,29	2,45	0,00113	NM_198276		TRPM5
7945742	-1,03	-2,05	0,02708	-0,70	-1,62	0,04093	NM_014555		SLC24A3
8061227	-1,03	-2,05	0,00325	-1,47	-2,78	0,00561	NM_020689		MTTP
8096580	-1,03	-2,05	0,01125	-0,93	-1,90	0,00352	NM_000253		LOC641367
8027320	-1,03	-2,05	0,01276	-1,14	-2,20	0,01704	NR_024523		ANKRD7
8135697	-1,03	-2,05	0,05994	-1,02	-2,03	0,01562	NM_019644		---
8055666	-1,03	-2,05	0,00080	-0,82	-1,77	0,07824	---	---	
7919049	-1,03	-2,05	0,00499	-1,31	-2,49	0,00038	---	---	
8093110	-1,03	-2,05	0,07958	-1,02	-2,03	0,00009	---	---	
7963739	-1,03	-2,05	0,05001	-2,82	-7,06	0,00130	---	FAM3D	
8088425	-1,03	-2,05	0,05198	-1,72	-3,30	0,00662	NM_138805		TMEM78
7910396	-1,03	-2,05	0,05743	-1,41	-2,66	0,00293	ENST00000323223		OR2M1P
7911260	-1,03	-2,05	0,44285	-2,37	-5,17	0,05640	NR_002141		CGREF1
8051001	-1,03	-2,05	0,00038	1,11	2,16	0,08801	NM_006569		SNORA70G
7964830	-1,03	-2,05	0,10731	-0,43	-1,35	0,00220	NR_033335		GAD1
8046283	-1,03	-2,05	0,01093	-0,17	-1,13	0,47440	NM_000817		C6orf132
8126379	-1,03	-2,05	0,00870	-0,95	-1,93	0,03776	NM_001164446		---
7969426	-1,03	-2,05	0,00009	-1,45	-2,72	0,05070	---	---	
8116952	-1,03	-2,05	0,35576	-2,65	-6,26	0,00101	---	PNMA2	
8149877	-1,03	-2,05	0,03373	-0,25	-1,19	0,59305	NM_007257		CABP5
8037962	-1,03	-2,05	0,16392	-0,80	-1,75	0,13509	NM_019855		---
8116295	-1,03	-2,05	0,05970	-1,36	-2,56	0,02747	---	LAIR2	
8031196	-1,03	-2,05	0,11273	-1,43	-2,70	0,00063	NM_002288		C12orf74
7957510	-1,03	-2,05	0,02362	-1,87	-3,65	0,05927	BC107813	NCRNA00083	
7922590	-1,03	-2,05	0,03150	-1,98	-3,94	0,01034	AK125993	BATF3	
7924166	-1,03	-2,05	0,07012	-0,48	-1,39	0,21081	NM_018664		CCDC106
8031526	-1,03	-2,05	0,03394	-0,05	-1,04	0,88649	NM_013301		CTBP2
7936904	-1,03	-2,05	0,07141	0,07	1,05	0,77033	NM_022802		CHMP4A
7978187	-1,04	-2,05	0,00660	-0,78	-1,71	0,05455	NM_014169		---
7942809	-1,04	-2,05	0,06208	-0,25	-1,19	0,27234	---	GRM1	
8122537	-1,04	-2,05	0,00428	-1,47	-2,78	0,04018	NM_001114329		SGK494
8013622	-1,04	-2,05	0,14100	1,02	2,03	0,02563	NM_001174103		---
8175151	-1,04	-2,05	0,06138	-0,95	-1,93	0,17432	---	PEX16	
7947609	-1,04	-2,05	0,03232	-1,33	-2,52	0,00995	NM_004813		HHLA1
8152938	-1,04	-2,05	0,07835	-1,40	-2,64	0,00119	NM_001145095		---
7937936	-1,04	-2,05	0,01500	-1,73	-3,32	0,06293	---	FOXD4L2	
8155440	-1,04	-2,05	0,12544	-1,26	-2,39	0,03493	NM_001099279		FOXD4L2
8161583	-1,04	-2,05	0,12544	-1,26	-2,39	0,03493	NM_001099279		ROBO4
7952453	-1,04	-2,05	0,04064	-1,76	-3,38	0,00960	NM_019055		RUSC1
7905999	-1,04	-2,05	0,06755	0,08	1,06	0,90536	NM_001105203		---
8105583	-1,04	-2,05	0,00080	-1,44	-2,72	0,02220	---	---	
8158057	-1,04	-2,05	0,06008	-1,73	-3,31	0,02197	---	RFPL4B	
8121553	-1,04	-2,05	0,00150	-0,72	-1,64	0,01564	NM_001013734		---

8117739	-1,04	-2,05	0,04400	-1,79	-3,46	0,00004	---	HCRTR1	
7899643	-1,04	-2,05	0,03617	-1,85	-3,60	0,03337	NM_001525		LOC100132167
8155516	-1,04	-2,05	0,03209	-0,96	-1,95	0,08366	BC006438	OR7E125P	
8149208	-1,04	-2,05	0,50883	-3,16	-8,95	0,00002	ENST00000420198		ZNRF1
7997247	-1,04	-2,05	0,00748	0,02	1,01	0,96655	NM_032268		PRSS55
8144571	-1,04	-2,05	0,03520	-1,19	-2,28	0,00106	NM_198464		---
8005397	-1,04	-2,05	0,07045	-1,15	-2,22	0,01099	---	---	
8115675	-1,04	-2,05	0,00209	-0,96	-1,95	0,05442	---	TMPRSS15	
8069582	-1,04	-2,05	0,00042	-1,18	-2,27	0,04998	NM_002772		GPR15
8081214	-1,04	-2,05	0,12264	-1,20	-2,29	0,00027	NM_005290		---
8157101	-1,04	-2,05	0,06445	-2,31	-4,97	0,01874	---	CXorf36	
8172254	-1,04	-2,05	0,00691	-1,18	-2,27	0,00022	NM_176819		HPDL
7901087	-1,04	-2,05	0,13325	-0,11	-1,08	0,76587	NM_032756		KIR3DL1
8031311	-1,04	-2,05	0,12015	-1,63	-3,10	0,00521	NM_013289		CTNNA2
8042993	-1,04	-2,05	0,02879	-1,35	-2,54	0,00847	NM_004389		TPPP
8110708	-1,04	-2,05	0,06840	-1,47	-2,76	0,00331	NM_007030		OR4K5
7973014	-1,04	-2,05	0,05022	-1,00	-2,00	0,02325	NM_001005483		---
8017619	-1,04	-2,05	0,01320	-1,11	-2,15	0,01599	---	---	
8117720	-1,04	-2,05	0,17303	-2,06	-4,17	0,01093	---	CCT8L1	
8137468	-1,04	-2,05	0,00370	-0,74	-1,67	0,05491	ENST00000021776		SEMA6B
8032839	-1,04	-2,05	0,04782	0,23	1,18	0,59656	NM_032108		CEACAM21
8037043	-1,04	-2,05	0,14199	-1,77	-3,41	0,00157	AK023602	---	
7969563	-1,04	-2,05	0,03938	-1,18	-2,26	0,00470	---	MAGEA4	
8170531	-1,04	-2,05	0,14704	-1,30	-2,46	0,00575	NM_001011548		---
8080683	-1,04	-2,05	0,03863	-1,34	-2,52	0,03780	---	OR1I1	
8026405	-1,04	-2,05	0,04993	-1,84	-3,58	0,00202	NM_001004713		SDR16C5
8150889	-1,04	-2,05	0,00133	-0,03	-1,02	0,45741	NM_138969		---
7950862	-1,04	-2,05	0,28041	-0,96	-1,95	0,00362	---	MAGEL2	
7986755	-1,04	-2,05	0,03928	-2,27	-4,82	0,00342	NM_019066		FAM115C
8136954	-1,04	-2,05	0,02331	-1,17	-2,26	0,00127	NM_001130026		---
8121223	-1,04	-2,05	0,00065	-0,98	-1,97	0,02791	---	---	
8121255	-1,04	-2,05	0,01000	-1,01	-2,01	0,03567	---	HOXB4	
8016452	-1,04	-2,05	0,05159	-1,31	-2,47	0,00010	NM_024015		PHOX2B
8100007	-1,04	-2,05	0,04008	-0,36	-1,28	0,17279	NM_003924		GAL3ST3
7949570	-1,04	-2,05	0,07571	-1,79	-3,47	0,01864	NM_033036		P2RX2
7960099	-1,04	-2,06	0,01404	-1,60	-3,03	0,01983	NM_170683		ECHDC3
7926152	-1,04	-2,06	0,06543	0,75	1,68	0,22706	NM_024693		---
7999023	-1,04	-2,06	0,00290	-1,47	-2,76	0,01118	---	C5orf27	
8106999	-1,04	-2,06	0,01416	-1,18	-2,26	0,00981	NR_026936		OR2T3
7911283	-1,04	-2,06	0,37231	-2,00	-4,01	0,04901	NM_001005495		---
7916841	-1,04	-2,06	0,09682	-1,11	-2,16	0,00594	---	MAG	
8027824	-1,04	-2,06	0,01754	-1,96	-3,88	0,00031	NM_080600		---
8003800	-1,04	-2,06	0,07216	-1,12	-2,17	0,02805	---	OR2F2	
8136961	-1,04	-2,06	0,12174	-1,60	-3,03	0,00396	NM_001004685		C9orf172
8159439	-1,04	-2,06	0,05753	-0,86	-1,81	0,10796	NM_001080482		---
8044764	-1,04	-2,06	0,01444	-1,34	-2,53	0,05106	---	FLJ44881	
8021777	-1,04	-2,06	0,14645	-1,52	-2,87	0,08075	BC119776	HECA	
8122343	-1,04	-2,06	0,09991	0,64	1,55	0,06318	NM_016217		HAND2
8103745	-1,04	-2,06	0,08541	-1,51	-2,85	0,00025	NM_021973		XAGE1A
8167701	-1,04	-2,06	0,09588	2,00	4,00	0,00040	NR_033253		XAGE1A
8167710	-1,04	-2,06	0,09588	2,00	4,00	0,00040	NR_033253		XAGE1A
8172739	-1,04	-2,06	0,09588	2,00	4,00	0,00040	NR_033253		XAGE1A
8172757	-1,04	-2,06	0,09588	2,00	4,00	0,00040	NR_033253		XAGE1A
8172766	-1,04	-2,06	0,09588	2,00	4,00	0,00040	NR_033253		MYOZ1
7934434	-1,04	-2,06	0,02280	-1,08	-2,11	0,00039	NM_021245		---
8101427	-1,04	-2,06	0,06593	-0,93	-1,91	0,00091	---	JAKMIP3	
7931417	-1,04	-2,06	0,02616	-0,37	-1,29	0,07450	NM_001105521		---
7999886	-1,04	-2,06	0,11753	-0,60	-1,52	0,20897	---	---	
8042062	-1,04	-2,06	0,03798	-1,73	-3,33	0,02704	---	GPR173	
8167758	-1,04	-2,06	0,01481	-0,81	-1,75	0,02535	NM_018969		OPN5
8120151	-1,04	-2,06	0,00309	-1,49	-2,80	0,01963	NM_181744		AWAT2
8173349	-1,04	-2,06	0,01578	-1,41	-2,65	0,02399	NM_001002254		---
8171537	-1,04	-2,06	0,00663	-1,48	-2,79	0,03559	---	---	
8156748	-1,04	-2,06	0,23529	-2,46	-5,49	0,00121	---	---	
7923005	-1,04	-2,06	0,01187	-1,64	-3,12	0,00016	---	LSM10	
7914917	-1,04	-2,06	0,07797	0,07	1,05	0,79265	NM_032881		RHOH
8094743	-1,04	-2,06	0,01237	-0,59	-1,50	0,08581	NM_004310		---
8024304	-1,04	-2,06	0,07968	-0,72	-1,65	0,15297	---	---	
8104117	-1,04	-2,06	0,00942	-1,10	-2,14	0,00722	---	---	
7948117	-1,04	-2,06	0,35779	-1,21	-2,31	0,01003	---	CXorf64	
8169808	-1,04	-2,06	0,06689	-1,52	-2,87	0,03695	NM_001122716		FLJ23865
8076193	-1,04	-2,06	0,08911	-1,04	-2,06	0,05193	AK074445	---	
8098101	-1,04	-2,06	0,00690	-1,87	-3,64	0,01597	---	---	

8081339	-1,04	-2,06	0,02894	-1,88	-3,67	0,00831	---	UNC93B1	
7949904	-1,04	-2,06	0,16038	1,54	2,90	0,01885	NM_030930		GPR113
8050924	-1,04	-2,06	0,01547	-1,31	-2,48	0,00803	NM_001145168		PPEF2
8101065	-1,04	-2,06	0,02365	-1,07	-2,10	0,00318	NM_006239		CNGB3
8151659	-1,04	-2,06	0,01771	-1,23	-2,34	0,00394	NM_019098		---
7933145	-1,04	-2,06	0,01228	-0,99	-1,98	0,02786	---	FOXB1	
7984011	-1,04	-2,06	0,05220	-1,54	-2,91	0,00523	NM_012182		LOC100129484
8137962	-1,04	-2,06	0,11481	-0,98	-1,97	0,14277	AK125308	SHROOM3	
8095834	-1,04	-2,06	0,05897	0,53	1,44	0,02476	NM_020859		TMEM155
8097126	-1,04	-2,06	0,00081	-0,31	-1,24	0,42041	NM_152399		WASH7P
7911309	-1,04	-2,06	0,05192	-0,19	-1,14	0,52039	NR_024540		---
8048024	-1,04	-2,06	0,00051	-1,47	-2,77	0,04688	---	LOC641515	
7908863	-1,04	-2,06	0,03858	-1,50	-2,82	0,00020	BC032956	CSF3R	
7914950	-1,04	-2,06	0,00140	-1,23	-2,34	0,06953	NM_156039		---
8040428	-1,04	-2,06	0,04052	-0,67	-1,59	0,01057	---	CCDC122	
7971335	-1,04	-2,06	0,07534	-0,22	-1,17	0,55605	NM_144974		---
7916745	-1,04	-2,06	0,00022	-1,07	-2,10	0,00060	---	CIB4	
8050993	-1,04	-2,06	0,04929	-0,76	-1,70	0,01333	NM_001029881		CHRNG
8049166	-1,04	-2,06	0,19687	-1,55	-2,93	0,00602	NM_005199		---
7944359	-1,04	-2,06	0,00013	-0,82	-1,77	0,12024	---	FCER2	
8033420	-1,04	-2,06	0,02848	-1,44	-2,72	0,00594	NM_002002		KRT73
7963471	-1,04	-2,06	0,00956	-1,44	-2,72	0,00017	NM_175068		---
8020088	-1,04	-2,06	0,01628	-1,33	-2,52	0,03793	---	---	
7944601	-1,04	-2,06	0,09410	-1,03	-2,05	0,10601	---	---	
8086075	-1,04	-2,06	0,04841	-1,06	-2,08	0,01061	---	---	
8131512	-1,04	-2,06	0,24148	-0,48	-1,40	0,39091	---	ANKDD1B	
8106325	-1,04	-2,06	0,00833	-0,15	-1,11	0,27630	ENST00000344149		---
8080674	-1,04	-2,06	0,09680	-0,46	-1,38	0,00751	---	ASPG	
7977203	-1,04	-2,06	0,03204	-1,04	-2,06	0,03177	NM_001080464		PKP1
7908672	-1,04	-2,06	0,10104	-1,45	-2,72	0,00321	NM_000299		NLGN4X
8171205	-1,04	-2,06	0,04150	-0,76	-1,69	0,01405	NM_020742		---
8022247	-1,04	-2,06	0,02728	-1,26	-2,40	0,01342	---	---	
8127509	-1,04	-2,06	0,01243	-1,51	-2,85	0,02007	---	EDAR	
8054451	-1,04	-2,06	0,10290	-1,61	-3,06	0,00014	NM_022336		C9orf100
8161026	-1,04	-2,06	0,01169	1,20	2,30	0,00119	NM_032818		ZNF169
8156529	-1,04	-2,06	0,04073	-0,69	-1,62	0,21134	NM_194320		FAM99A
7937707	-1,04	-2,06	0,18387	-1,70	-3,25	0,15580	NR_026643		ASCL1
7958051	-1,04	-2,06	0,17576	-0,78	-1,71	0,05153	NM_004316		---
7972001	-1,04	-2,06	0,02398	-1,01	-2,02	0,00000	---	STARD9	
7983132	-1,04	-2,06	0,02342	-0,30	-1,23	0,34574	NM_020759		---
8077768	-1,05	-2,06	0,10785	-0,73	-1,66	0,01291	---	KRTAP27-1	
8069808	-1,05	-2,06	0,03147	-0,68	-1,60	0,22208	NM_001077711		---
7929332	-1,05	-2,06	0,12721	-1,83	-3,56	0,05885	---	---	
7931975	-1,05	-2,06	0,01074	-1,43	-2,69	0,02859	---	CA4	
8008900	-1,05	-2,06	0,07001	-1,37	-2,58	0,00095	NM_000717		---
8176669	-1,05	-2,06	0,01893	-2,04	-4,11	0,00737	---	---	
8177189	-1,05	-2,06	0,01893	-2,04	-4,11	0,00737	---	---	
8123642	-1,05	-2,06	0,13866	-1,59	-3,02	0,00195	---	POLM	
8139281	-1,05	-2,06	0,03135	-0,19	-1,14	0,64738	NM_013284		---
8095037	-1,05	-2,06	0,05910	-1,34	-2,53	0,00418	---	---	
8130783	-1,05	-2,06	0,10367	-1,09	-2,12	0,01823	---	ARG1	
8122058	-1,05	-2,06	0,05926	-1,06	-2,08	0,00395	NM_000045		---
8044909	-1,05	-2,06	0,00778	-0,89	-1,85	0,00483	---	ABCB5	
8131682	-1,05	-2,07	0,04457	-0,86	-1,81	0,05303	NM_178559		SIRPB2
8064451	-1,05	-2,07	0,03872	-1,55	-2,92	0,00012	NM_001122962		OR10H4
8026494	-1,05	-2,07	0,14772	-2,37	-5,17	0,00118	NM_001004465		---
8100868	-1,05	-2,07	0,02183	-1,28	-2,43	0,00228	---	---	
8097305	-1,05	-2,07	0,00288	-0,90	-1,87	0,01518	---	C5orf60	
8110518	-1,05	-2,07	0,09973	0,26	1,20	0,03648	NM_001142306		CCDC29
8069508	-1,05	-2,07	0,12588	-0,25	-1,19	0,64253	ENST00000333394		STAR
8150253	-1,05	-2,07	0,04872	-1,46	-2,75	0,00130	NM_000349		CYYR1
8069668	-1,05	-2,07	0,00887	-1,67	-3,19	0,02555	NM_052954		RNF133
8142618	-1,05	-2,07	0,14688	-1,37	-2,59	0,01263	NM_139175		MPP4
8058273	-1,05	-2,07	0,11212	-1,87	-3,65	0,06299	NM_033066		LGALS13
8028737	-1,05	-2,07	0,06944	-2,15	-4,43	0,03196	NM_013268		C1orf53
7908525	-1,05	-2,07	0,03514	-0,50	-1,41	0,05272	NM_001024594		CD180
8112428	-1,05	-2,07	0,09747	-0,42	-1,34	0,10577	NM_005582		CACNG4
8009334	-1,05	-2,07	0,03103	-0,41	-1,33	0,47179	NM_014405		RAET1G
8130163	-1,05	-2,07	0,06004	-1,22	-2,32	0,03860	NM_001001788		---
8084169	-1,05	-2,07	0,15153	-1,39	-2,62	0,01336	---	---	
8103542	-1,05	-2,07	0,00200	-1,75	-3,37	0,00689	---	NLRP4	
8031576	-1,05	-2,07	0,01478	-0,68	-1,60	0,00872	NM_134444		C18orf23
8021129	-1,05	-2,07	0,01927	-1,05	-2,07	0,08828	BC093850	ACCN3	

8137336	-1,05	-2,07	0,09053	-1,06	-2,09	0,00084	NM_004769	MIR320C1
8020419	-1,05	-2,07	0,15313	-0,97	-1,95	0,05581	NR_031565	---
8114546	-1,05	-2,07	0,05810	-1,11	-2,16	0,02115	---	TAAR2
8129610	-1,05	-2,07	0,15177	-0,79	-1,73	0,01873	NM_001033080	LOC400891
8071504	-1,05	-2,07	0,01630	-1,09	-2,12	0,01836	NR_027006	FCN1
8165011	-1,05	-2,07	0,02947	-1,43	-2,69	0,04567	NM_002003	---
8129456	-1,05	-2,07	0,06798	-0,80	-1,75	0,00251	---	---
8123959	-1,05	-2,07	0,07864	-1,28	-2,43	0,00332	---	SERPINC1
7922420	-1,05	-2,07	0,01613	-0,15	-1,11	0,64937	NM_000488	SIGIRR
7945377	-1,05	-2,07	0,00825	0,12	1,09	0,77079	NM_001135053	---
8112407	-1,05	-2,07	0,03053	-0,87	-1,83	0,05341	---	---
7898112	-1,05	-2,07	0,17982	0,86	1,82	0,27153	---	GRTP1
7972890	-1,05	-2,07	0,10795	1,04	2,06	0,08724	NM_024719	PTX4
7998538	-1,05	-2,07	0,01065	-1,46	-2,74	0,01953	NM_001013658	TSPAN11
7954653	-1,05	-2,07	0,08469	-0,52	-1,44	0,09472	NM_001080509	YPEL4
7948282	-1,05	-2,07	0,01204	-1,27	-2,42	0,00117	NM_145008	OR4D11
7940187	-1,05	-2,07	0,10245	-1,61	-3,06	0,00069	NM_001004706	FOXB2
8155942	-1,05	-2,07	0,07087	-1,59	-3,01	0,00157	NM_001013735	---
8115012	-1,05	-2,07	0,02628	-1,23	-2,34	0,05620	---	MARCO
8044773	-1,05	-2,07	0,03407	-0,97	-1,96	0,00354	NM_006770	LOC100130815
7981190	-1,05	-2,07	0,06397	-1,48	-2,78	0,00922	AL834311	KCNJ11
7946853	-1,05	-2,07	0,01121	-0,50	-1,42	0,21298	NM_000525	RAB4B
8028930	-1,05	-2,07	0,02153	0,29	1,22	0,60139	NM_016154	CD38
8094240	-1,05	-2,07	0,01390	3,05	8,29	0,00002	NM_001775	C1orf95
7910142	-1,05	-2,07	0,03427	-1,03	-2,04	0,12179	NM_001003665	OR51F2
7937973	-1,05	-2,07	0,05512	-2,05	-4,14	0,04415	NM_001004753	IGHE
7981708	-1,05	-2,07	0,08858	-1,47	-2,77	0,06053	ENST00000390541	ASMTL
8171119	-1,05	-2,07	0,03551	-0,75	-1,68	0,25442	NM_004192	---
8050238	-1,05	-2,07	0,00048	-0,91	-1,88	0,03423	---	OR4B1
7939865	-1,05	-2,07	0,18883	-1,54	-2,90	0,00109	NM_001005470	MAP1D
8046360	-1,05	-2,07	0,05283	0,00	-1,00	0,99205	NM_199227	FAM22F
8162479	-1,05	-2,07	0,04329	-1,35	-2,54	0,05828	NM_017561	FBXL14
7960280	-1,05	-2,07	0,10269	-0,28	-1,21	0,36245	NM_152441	FAM108A1
7904869	-1,05	-2,07	0,04558	0,77	1,71	0,13937	NM_001130111	FAM108A1
7904948	-1,05	-2,07	0,04558	0,77	1,71	0,13937	NM_001130111	---
7898673	-1,05	-2,07	0,02129	-0,80	-1,74	0,04166	---	SLC28A1
7985636	-1,05	-2,07	0,01779	-0,93	-1,91	0,00047	NM_004213	---
7900144	-1,05	-2,07	0,03739	-0,69	-1,61	0,00145	---	REXO1L2P
8151631	-1,05	-2,07	0,58921	-6,02	-64,88	0,00002	NR_003594	KRT79
7963545	-1,05	-2,07	0,11264	-1,72	-3,30	0,00059	NM_175834	SYNGAP1
8118696	-1,05	-2,07	0,02044	-0,30	-1,23	0,29081	NM_006772	RALGDS
8164810	-1,05	-2,07	0,34600	2,43	5,38	0,00269	NM_006266	IKBKE
7923885	-1,05	-2,07	0,00945	-1,27	-2,41	0,00078	NM_014002	LOC400931
8073816	-1,05	-2,07	0,01155	-0,96	-1,94	0,03940	NR_027033	HMHB1
8108900	-1,05	-2,07	0,02229	-1,60	-3,03	0,00617	NM_021182	---
7906015	-1,05	-2,07	0,10156	-1,11	-2,16	0,10228	---	RGS11
7998190	-1,05	-2,07	0,00317	-0,30	-1,23	0,31728	NM_183337	MPL
7900683	-1,05	-2,07	0,06848	-1,01	-2,01	0,02724	NM_005373	---
7911335	-1,05	-2,07	0,06692	-2,97	-7,82	0,00483	---	---
8165694	-1,05	-2,07	0,06692	-2,97	-7,82	0,00483	---	GRIN1
8159583	-1,05	-2,07	0,01801	-0,78	-1,72	0,01109	NM_007327	---
8092455	-1,05	-2,07	0,01400	-2,25	-4,76	0,00277	---	---
8149279	-1,05	-2,07	0,00146	-1,42	-2,67	0,00799	---	ZNF668
7995030	-1,05	-2,07	0,05948	-1,53	-2,88	0,02302	NM_001172668	---
7978389	-1,05	-2,07	0,05602	-2,31	-4,97	0,00276	---	---
8084124	-1,05	-2,07	0,00770	-1,13	-2,19	0,03265	---	HDGFRP2
8024864	-1,05	-2,07	0,08588	0,07	1,05	0,88209	NM_001001520	CETP
7995907	-1,05	-2,07	0,03576	-1,39	-2,62	0,00193	NM_000078	CPB2
7971444	-1,05	-2,07	0,08636	-1,23	-2,34	0,00092	NM_001872	CLEC12A
7953901	-1,05	-2,07	0,07429	-1,10	-2,15	0,00485	NM_138337	CHRNA9
8094751	-1,05	-2,07	0,00217	-0,89	-1,85	0,09977	NM_017581	TEKT4
8043491	-1,05	-2,07	0,00876	-0,80	-1,74	0,00275	NM_144705	CA1
8151592	-1,05	-2,07	0,04317	-1,42	-2,67	0,02219	NM_001738	GABRA4
8100131	-1,05	-2,07	0,00337	-1,15	-2,21	0,00160	NM_000809	OPN1LW
8170811	-1,05	-2,07	0,15367	-0,65	-1,57	0,11413	NM_020061	HIGD1B
8007701	-1,05	-2,07	0,04941	-1,64	-3,11	0,00042	NM_016438	FRMD7
8175155	-1,05	-2,07	0,00423	-0,76	-1,69	0,03318	NM_194277	GMCL1L
8116174	-1,05	-2,07	0,00189	-0,89	-1,86	0,00767	NR_003281	GOLGA6A
7990654	-1,05	-2,07	0,41387	-0,04	-1,03	0,97600	NM_001038640	NRXN1
8052091	-1,05	-2,07	0,01693	-1,19	-2,28	0,02591	NM_004801	---
8109618	-1,05	-2,07	0,02343	-1,69	-3,22	0,00071	---	CD2BP2
8000848	-1,05	-2,07	0,02877	-0,47	-1,38	0,44583	NM_006110	WBSCR17
8133258	-1,05	-2,08	0,04653	-1,52	-2,87	0,00861	NM_022479	---

7975404	-1,05	-2,08	0,08496	-1,48	-2,78	0,03918	---	GADD45G
8156309	-1,05	-2,08	0,01384	-0,31	-1,24	0,43907	NM_006705	---
8137131	-1,05	-2,08	0,04755	-2,15	-4,44	0,03664	---	---
8165856	-1,05	-2,08	0,17020	-1,23	-2,35	0,00653	---	---
7935401	-1,05	-2,08	0,02651	-0,88	-1,84	0,02484	---	SLCO4A1
8067546	-1,05	-2,08	0,07947	-1,25	-2,38	0,01355	NM_016354	LYZL4
8086400	-1,05	-2,08	0,01511	-1,56	-2,95	0,02454	NM_144634	IGSF6
8000184	-1,05	-2,08	0,01491	-1,49	-2,81	0,00196	NM_005849	ZXDC
8090364	-1,05	-2,08	0,04230	-0,92	-1,89	0,00869	AK093796	C7
8105084	-1,05	-2,08	0,07892	-1,27	-2,42	0,00060	NM_000587	SHH
8144078	-1,05	-2,08	0,02101	-1,14	-2,20	0,03126	NM_000193	---
8059576	-1,05	-2,08	0,33925	-3,43	-10,80	0,00033	---	ZPBP
8139622	-1,05	-2,08	0,06191	-0,71	-1,63	0,04035	NM_007009	TSPAN16
8034199	-1,05	-2,08	0,04767	-1,92	-3,79	0,00095	NM_012466	AGAP5
7927305	-1,05	-2,08	0,02072	0,85	1,80	0,02110	NM_001144000	TPD52L3
8154307	-1,06	-2,08	0,01811	-1,13	-2,19	0,00956	NM_033516	---
8140317	-1,06	-2,08	0,01515	-1,70	-3,25	0,00228	---	---
7905129	-1,06	-2,08	0,17779	-1,20	-2,29	0,02011	---	CECR2
8071086	-1,06	-2,08	0,03075	1,43	2,70	0,00119	NM_031413	C9orf106
8158539	-1,06	-2,08	0,03812	-1,82	-3,52	0,01142	NM_001012715	FAM115C
8136940	-1,06	-2,08	0,02058	-0,94	-1,91	0,00353	NM_001130026	---
8106530	-1,06	-2,08	0,19177	-0,37	-1,29	0,14777	---	CDH15
7997880	-1,06	-2,08	0,04182	-0,69	-1,62	0,12534	NM_004933	---
8152451	-1,06	-2,08	0,00007	-1,59	-3,01	0,00436	---	NT5C1B
8050480	-1,06	-2,08	0,00697	-1,05	-2,07	0,00260	NM_001002006	ARHGEF35
8136981	-1,06	-2,08	0,01338	-0,18	-1,14	0,61553	NM_001003702	---
7976154	-1,06	-2,08	0,00054	-0,66	-1,58	0,00091	---	MBL2
7933665	-1,06	-2,08	0,12774	-1,11	-2,16	0,01542	NM_000242	SYT4
8022986	-1,06	-2,08	0,03917	-0,82	-1,77	0,01118	NM_020783	PRY
8176935	-1,06	-2,08	0,04724	-1,41	-2,65	0,00834	NM_004676	PRY
8177395	-1,06	-2,08	0,04724	-1,41	-2,65	0,00834	NM_004676	HTRA4
8145989	-1,06	-2,08	0,00392	-1,34	-2,53	0,01041	NM_153692	---
8128714	-1,06	-2,08	0,00538	-1,06	-2,08	0,00294	---	DBX2
7962481	-1,06	-2,08	0,00429	-0,67	-1,59	0,04074	NM_001004329	KREMEN2
7992758	-1,06	-2,08	0,03432	-1,68	-3,19	0,00205	NM_172229	---
8091731	-1,06	-2,08	0,06556	-2,23	-4,70	0,00356	---	KRT72
7963459	-1,06	-2,08	0,00168	-1,45	-2,72	0,00089	NM_080747	OR6C75
7955993	-1,06	-2,08	0,07109	-1,45	-2,73	0,01995	NM_001005497	TMEM125
7900654	-1,06	-2,08	0,02140	-1,61	-3,04	0,00190	NM_144626	GDAP1L1
8062796	-1,06	-2,08	0,05208	-1,33	-2,51	0,00552	NM_024034	ADARB2
7931764	-1,06	-2,08	0,05264	-1,19	-2,28	0,00859	NM_018702	DNAJB3
8059832	-1,06	-2,08	0,03222	-1,10	-2,14	0,04588	NM_001001394	DKFZp761E198
7949490	-1,06	-2,08	0,09376	-0,53	-1,45	0,22706	NM_138368	SNORA35
8169459	-1,06	-2,08	0,11614	-1,51	-2,84	0,03700	NR_002993	INHBC
7956423	-1,06	-2,08	0,12449	-0,30	-1,23	0,26471	NM_005538	CASC1
7961844	-1,06	-2,08	0,03825	-1,03	-2,04	0,00405	NM_018272	---
8106816	-1,06	-2,08	0,06275	-0,71	-1,64	0,00139	---	VHLL
7920980	-1,06	-2,08	0,00894	-1,16	-2,23	0,01678	NM_001004319	GPR153
7912004	-1,06	-2,08	0,05949	-0,77	-1,70	0,15806	NM_207370	PRSS36
8001014	-1,06	-2,08	0,14867	-1,38	-2,60	0,06620	NM_173502	SLC16A5
8009746	-1,06	-2,08	0,01735	1,17	2,25	0,05352	NM_004695	PNMT
8006900	-1,06	-2,08	0,02315	-0,99	-1,98	0,02777	NM_002686	ZMAT5
8075276	-1,06	-2,08	0,01264	-1,25	-2,39	0,03270	NM_019103	---
7924495	-1,06	-2,08	0,00885	-0,56	-1,48	0,00659	---	SPDYE8P
8140211	-1,06	-2,08	0,29955	-2,12	-4,36	0,00138	NR_003664	C12orf53
7960682	-1,06	-2,08	0,06284	-0,62	-1,54	0,21421	AK289648	KLK4
8038655	-1,06	-2,08	0,01418	-0,83	-1,78	0,06091	NM_004917	APOC4
8029541	-1,06	-2,08	0,00028	-1,69	-3,22	0,00010	NM_001646	DUSP15
8065580	-1,06	-2,08	0,04777	-1,81	-3,51	0,08842	NM_080611	AKNAD1
7918235	-1,06	-2,08	0,13670	-1,11	-2,15	0,10592	NM_152763	ZNF534
8030939	-1,06	-2,08	0,02017	-1,04	-2,06	0,02667	NM_001143938	---
8165682	-1,06	-2,08	0,17478	-3,16	-8,91	0,00021	---	---
8114191	-1,06	-2,08	0,00899	-1,58	-3,00	0,00101	---	LOC494141
7938750	-1,06	-2,08	0,01804	-0,83	-1,77	0,00717	NR_026541	CSNK1A1P
7987381	-1,06	-2,08	0,01691	-1,27	-2,42	0,01276	NR_027320	---
7937977	-1,06	-2,08	0,00804	-0,86	-1,81	0,02237	---	---
7970672	-1,06	-2,08	0,02975	-1,03	-2,05	0,00271	---	---
7937798	-1,06	-2,09	0,06969	-1,42	-2,67	0,02202	---	OR11L1
7925737	-1,06	-2,09	0,03024	-1,64	-3,13	0,00274	NM_001001959	KCNQ1
7937823	-1,06	-2,09	0,03690	-0,36	-1,28	0,22742	NM_000218	---
8170198	-1,06	-2,09	0,01045	0,09	1,07	0,81111	---	HRH2
8110106	-1,06	-2,09	0,00951	-0,59	-1,51	0,30348	NM_001131055	ADRA2B
8053869	-1,06	-2,09	0,20475	-1,32	-2,49	0,05203	NM_000682	CHGB

8060805	-1,06	-2,09	0,06044	-1,08	-2,11	0,00088	NM_001819	F7
7970232	-1,06	-2,09	0,00055	-1,09	-2,13	0,00156	NM_000131	KLHL34
8171786	-1,06	-2,09	0,03310	-1,39	-2,62	0,00216	NM_153270	---
8020466	-1,06	-2,09	0,01658	-1,34	-2,54	0,00047	---	IL21R
7994292	-1,06	-2,09	0,02291	-1,70	-3,24	0,01859	NM_181078	RBM12B
8151788	-1,06	-2,09	0,04510	-0,44	-1,36	0,16010	NM_203390	CLRN3
7937016	-1,06	-2,09	0,00112	0,00	1,00	0,98825	NM_152311	RYR3
7982392	-1,06	-2,09	0,02231	-1,29	-2,44	0,00009	NM_001036	---
7984622	-1,06	-2,09	0,00011	-0,28	-1,21	0,19567	---	DPP10
8044700	-1,06	-2,09	0,01365	-0,63	-1,55	0,00235	NM_020868	---
8010184	-1,06	-2,09	0,19215	-2,72	-6,58	0,07478	---	FOXD4L2
8161571	-1,06	-2,09	0,15214	-1,29	-2,44	0,02355	NM_001099279	---
8151490	-1,06	-2,09	0,06106	-1,43	-2,69	0,00308	---	RSPH10B
8138009	-1,06	-2,09	0,02708	-0,58	-1,49	0,08320	NM_173565	DNAH17
8018872	-1,06	-2,09	0,04125	-1,26	-2,40	0,00346	NM_173628	KIF26A
7977216	-1,06	-2,09	0,02241	-1,12	-2,17	0,03919	NM_015656	---
8097120	-1,06	-2,09	0,16369	-0,92	-1,89	0,00027	---	COL25A1
8102263	-1,06	-2,09	0,11436	-1,04	-2,06	0,00300	NM_198721	ALDH8A1
8129695	-1,06	-2,09	0,00247	-1,39	-2,62	0,07154	NM_022568	C17orf87
8011826	-1,06	-2,09	0,01802	-1,43	-2,69	0,02467	NM_207103	OR10K2
7921356	-1,06	-2,09	0,18576	-1,37	-2,59	0,00602	NM_001004476	NTS
7957458	-1,06	-2,09	0,02390	-0,98	-1,97	0,00121	NM_006183	---
8035903	-1,06	-2,09	0,04149	-2,11	-4,31	0,00356	---	FLJ45139
8070321	-1,06	-2,09	0,01845	-0,86	-1,82	0,12097	AK127082	GRB7
8006940	-1,06	-2,09	0,03351	0,86	1,82	0,20522	NM_001030002	---
8157729	-1,06	-2,09	0,23263	-2,54	-5,82	0,00004	---	---
7944162	-1,06	-2,09	0,07923	-1,28	-2,43	0,00461	---	---
8001392	-1,06	-2,09	0,03072	-0,83	-1,77	0,00614	---	---
7992197	-1,06	-2,09	0,05920	-1,56	-2,94	0,00933	---	GOLGA6L10
7990922	-1,06	-2,09	0,39168	-0,44	-1,36	0,37154	NM_001164465	LOC152586
8102894	-1,06	-2,09	0,11454	-0,96	-1,94	0,00045	ENST00000388762	TRIM55
8146669	-1,06	-2,09	0,00505	-1,06	-2,08	0,00836	NM_033058	ABHD15
8013919	-1,06	-2,09	0,04871	0,32	1,25	0,58828	NM_198147	KCNN1
8026900	-1,06	-2,09	0,12190	-0,54	-1,45	0,05367	NM_002248	---
8056100	-1,06	-2,09	0,09841	-0,09	-1,06	0,45528	---	---
8054792	-1,06	-2,09	0,01056	-0,91	-1,88	0,02411	---	MAGEA1
8175775	-1,06	-2,09	0,00733	-1,20	-2,30	0,03806	NM_004988	TM4SF5
8003939	-1,06	-2,09	0,03562	-1,05	-2,08	0,18384	NM_003963	WFDC13
8063014	-1,06	-2,09	0,04943	-1,60	-3,03	0,00988	NM_172005	TUSC5
8003635	-1,06	-2,09	0,05552	-1,82	-3,52	0,01523	NM_172367	GDNF
8111670	-1,06	-2,09	0,07852	-1,40	-2,64	0,00044	NM_000514	APBB1IP
7926786	-1,06	-2,09	0,01251	-0,56	-1,48	0,04696	NM_019043	FLJ44313
8021774	-1,06	-2,09	0,07394	-0,38	-1,30	0,34679	ENST00000405150	CCKBR
7938090	-1,06	-2,09	0,01172	-1,83	-3,54	0,00986	NM_176875	RYR1
8028407	-1,06	-2,09	0,01632	-0,66	-1,58	0,00323	NM_000540	SLC34A2
8094441	-1,06	-2,09	0,10539	3,76	13,56	0,00054	NM_006424	STELLAR
7973962	-1,06	-2,09	0,01785	-1,11	-2,15	0,23263	BC062480	EXOC3L
8001953	-1,06	-2,09	0,07772	-1,63	-3,09	0,02256	NM_178516	---
7904394	-1,06	-2,09	0,16921	-1,43	-2,69	0,00397	---	---
8088917	-1,06	-2,09	0,08524	-1,69	-3,22	0,00032	---	CD163
7960794	-1,06	-2,09	0,03295	-1,71	-3,27	0,00303	NM_004244	KRTAP3-3 // KRTAP3-3
8015163	-1,06	-2,09	0,02817	-1,18	-2,27	0,11529	NM_033185 // NM_033185	KRTAP3-3 // KRTAP3-3
8019599	-1,06	-2,09	0,02817	-1,18	-2,27	0,11529	NM_033185 // NM_033185	MYL7
8139307	-1,07	-2,09	0,15331	-1,30	-2,47	0,05042	NM_021223	BANP
7997755	-1,07	-2,09	0,12946	-1,07	-2,10	0,12708	NM_079837	MIR126
8159371	-1,07	-2,09	0,00278	-1,09	-2,13	0,00168	NR_029695	UGT2B7
8095395	-1,07	-2,09	0,07765	-1,53	-2,89	0,00418	NM_001074	---
8151989	-1,07	-2,09	0,00122	-1,08	-2,11	0,09587	---	FAM64A
8004167	-1,07	-2,09	0,14588	0,71	1,64	0,41594	NM_019013	---
8090934	-1,07	-2,09	0,01171	-1,33	-2,52	0,01698	---	RASSF6
8100943	-1,07	-2,09	0,01353	-0,54	-1,45	0,00083	NM_201431	PCSK9
7901696	-1,07	-2,09	0,05574	-1,05	-2,07	0,04841	NM_174936	HEJ1
7903401	-1,07	-2,09	0,01115	-0,66	-1,58	0,00760	NR_033424	FBXL13
8141846	-1,07	-2,09	0,20322	-0,86	-1,82	0,09798	NM_145032	PCDHB6
8108703	-1,07	-2,09	0,00998	-0,51	-1,42	0,07793	NM_018939	SLC22A2
8130582	-1,07	-2,09	0,04492	-1,11	-2,17	0,10506	NM_003058	---
8045339	-1,07	-2,09	0,11032	-0,95	-1,93	0,00602	---	IGSF8
7921538	-1,07	-2,09	0,17509	0,84	1,79	0,38982	NM_052868	---
8161646	-1,07	-2,09	0,01435	-1,13	-2,19	0,01150	---	RNF39
8124695	-1,07	-2,09	0,04975	-0,20	-1,15	0,68794	NM_025236	RNF39
8178326	-1,07	-2,09	0,04975	-0,20	-1,15	0,68794	NM_025236	LRRC27

7931455	-1,07	-2,09	0,10039	-1,57	-2,97	0,00002	NM_001143757	HHLA2
8081488	-1,07	-2,09	0,01849	-0,81	-1,75	0,01537	NM_007072	LOC100128233
7979691	-1,07	-2,09	0,03767	-0,27	-1,21	0,21984	BX161428	FRMD4B
8088745	-1,07	-2,09	0,07925	0,36	1,29	0,25378	NM_015123	LPO
8008736	-1,07	-2,09	0,02794	-1,51	-2,85	0,03813	NM_006151	TMEM88
8004691	-1,07	-2,09	0,05935	0,29	1,22	0,63731	NM_203411	SNORA70F
8056359	-1,07	-2,09	0,00529	-1,56	-2,95	0,01225	NR_033309	---
7944262	-1,07	-2,10	0,04673	-1,62	-3,08	0,00948	---	MGAT4C
7965231	-1,07	-2,10	0,02431	-1,49	-2,82	0,00141	NM_013244	ZNF804B
8134018	-1,07	-2,10	0,01057	-1,55	-2,93	0,00190	NM_181646	---
8084941	-1,07	-2,10	0,03436	-2,44	-5,43	0,00297	---	SCML4
8128638	-1,07	-2,10	0,01851	-1,38	-2,60	0,02079	NM_198081	CREG2
8054338	-1,07	-2,10	0,00688	-1,45	-2,73	0,00341	NM_153836	---
7950893	-1,07	-2,10	0,12503	-1,00	-2,00	0,06014	---	WBSCR28
8133366	-1,07	-2,10	0,03474	0,58	1,50	0,01767	NM_182504	SPINK14
8109039	-1,07	-2,10	0,01701	-1,08	-2,12	0,04132	NM_001001325	---
8052087	-1,07	-2,10	0,15660	-0,45	-1,37	0,26094	---	---
8090235	-1,07	-2,10	0,05203	-1,57	-2,96	0,03855	---	RAPSN
7947881	-1,07	-2,10	0,02337	-1,41	-2,65	0,00834	NM_005055	---
8172086	-1,07	-2,10	0,01018	-0,79	-1,73	0,01931	---	FBXO9
8120269	-1,07	-2,10	0,02001	-1,36	-2,56	0,02811	AK095315	---
8152278	-1,07	-2,10	0,06205	-1,11	-2,15	0,10522	---	C11orf88
7943740	-1,07	-2,10	0,06175	-1,11	-2,15	0,00645	NM_207430	---
8132960	-1,07	-2,10	0,00348	-0,99	-1,99	0,02163	---	TAF1C
8003125	-1,07	-2,10	0,01064	0,06	1,05	0,87060	NM_005679	POM121L10P
8075024	-1,07	-2,10	0,14284	-2,44	-5,42	0,00451	NR_024593	LOC644950
8089457	-1,07	-2,10	0,05157	-0,99	-1,98	0,05003	ENST00000414444	DEFB133
8126931	-1,07	-2,10	0,03761	-1,72	-3,29	0,03095	NM_001166478	LOC284837
8070708	-1,07	-2,10	0,14486	-0,82	-1,76	0,18122	NR_026961	---
7920470	-1,07	-2,10	0,10724	-1,38	-2,61	0,02819	---	GPR101
8175434	-1,07	-2,10	0,06896	-0,88	-1,84	0,00349	NM_054021	ANO1
7942135	-1,07	-2,10	0,02702	-0,64	-1,56	0,04083	NM_018043	SNORA29
8130580	-1,07	-2,10	0,01199	-1,02	-2,03	0,00729	NR_002965	---
8103309	-1,07	-2,10	0,04425	-0,89	-1,85	0,01177	---	---
7925739	-1,07	-2,10	0,24911	-2,31	-4,96	0,10172	---	C1orf177
7901675	-1,07	-2,10	0,00362	-0,29	-1,22	0,21784	NM_152607	OTOS
8060094	-1,07	-2,10	0,06281	-1,71	-3,27	0,00298	NM_148961	TGM7
7988064	-1,07	-2,10	0,06538	-1,25	-2,38	0,03078	NM_052955	---
7991086	-1,07	-2,10	0,02574	-0,68	-1,60	0,01207	---	F9
8170215	-1,07	-2,10	0,00290	-1,40	-2,64	0,00000	NM_000133	ALKBH7
8025035	-1,07	-2,10	0,05235	-1,11	-2,15	0,06626	NM_032306	HPCAL1
8040238	-1,07	-2,10	0,07691	0,67	1,59	0,29140	NM_134421	---
8103458	-1,07	-2,10	0,13640	-0,90	-1,86	0,01557	---	DAO
7958425	-1,07	-2,10	0,15559	-1,57	-2,96	0,00845	NM_001917	DNAJC5G
8040889	-1,07	-2,10	0,04086	-0,33	-1,26	0,34168	NM_173650	---
8063240	-1,07	-2,10	0,09797	-0,58	-1,50	0,12268	---	---
8177068	-1,07	-2,10	0,05298	-0,90	-1,86	0,15636	---	ZNF554
8024511	-1,07	-2,10	0,04719	-0,71	-1,64	0,00089	NM_001102651	---
8081254	-1,07	-2,10	0,00141	-1,03	-2,04	0,04211	---	LOC646870
7922351	-1,07	-2,10	0,06492	-1,54	-2,91	0,06118	AK130460	FLJ42291
8144044	-1,07	-2,10	0,10097	-1,51	-2,85	0,03010	AK124285	OTOG
7938707	-1,07	-2,10	0,04010	-1,28	-2,43	0,00354	ENST00000342528	NANOS1
7930872	-1,07	-2,10	0,02911	-0,23	-1,17	0,30262	NM_199461	LOC648913
7986565	-1,07	-2,10	0,20003	-2,67	-6,36	0,00183	XM_001126277	---
8011110	-1,07	-2,10	0,02605	-1,97	-3,92	0,00711	---	XK
8166723	-1,07	-2,10	0,00866	1,41	2,65	0,00992	NM_021083	ITLN2
7921702	-1,07	-2,10	0,08732	-1,00	-2,00	0,00495	NM_080878	LOC100129038
7993339	-1,07	-2,10	0,06076	-0,62	-1,54	0,02705	ENST00000391432	---
8161915	-1,07	-2,10	0,06146	-1,36	-2,57	0,00036	---	ALPI
8049137	-1,07	-2,10	0,01927	-0,77	-1,70	0,06315	NM_001631	PAK6
7982688	-1,07	-2,10	0,08999	-0,26	-1,19	0,53017	NM_020168	SERPINA12
7981098	-1,07	-2,10	0,00681	-0,85	-1,80	0,00310	NM_173850	MYBL2
8062766	-1,07	-2,10	0,01001	2,27	4,81	0,00111	NM_002466	GABRG3
7982117	-1,07	-2,10	0,00361	-0,37	-1,29	0,17824	NM_033223	SPDYE2
8135197	-1,07	-2,10	0,40862	-2,12	-4,35	0,00121	NM_001031618	C21orf88
8070411	-1,07	-2,10	0,01250	-1,23	-2,34	0,01871	NR_026543	GRP
8021461	-1,07	-2,10	0,08384	-1,85	-3,61	0,00496	NM_002091	DSC1
8022728	-1,07	-2,10	0,07128	-1,27	-2,41	0,00375	NM_024421	TIGD6
8115068	-1,07	-2,10	0,00139	-0,39	-1,31	0,39815	NM_030953	INO80B
8042859	-1,07	-2,10	0,00847	-0,92	-1,90	0,09640	NM_031288	---
8171489	-1,07	-2,10	0,00417	-1,58	-2,98	0,00094	---	UCN3
7925950	-1,07	-2,10	0,03078	-2,20	-4,59	0,00278	NM_053049	SNORD55
7901046	-1,07	-2,10	0,11837	-2,04	-4,10	0,00012	NR_000015	UPP2

8045848	-1,07	-2,10	0,01078	-2,13	-4,37	0,00091	NM_001135098	TAS2R60
8136930	-1,07	-2,10	0,14220	-1,17	-2,26	0,06099	NM_177437	---
8020086	-1,07	-2,10	0,13833	-1,21	-2,32	0,00205	---	KIAA0913
7928408	-1,07	-2,10	0,03099	-1,96	-3,90	0,00183	NM_015037	HLA-DQB2
8125463	-1,07	-2,10	0,09932	-0,15	-1,11	0,57714	NR_003937	---
8175367	-1,07	-2,10	0,05923	-1,14	-2,21	0,01465	---	---
8103875	-1,07	-2,10	0,01068	-1,35	-2,56	0,00581	---	NCRNA00169
7993821	-1,07	-2,10	0,02365	0,11	1,08	0,60211	NR_026675	IL17C
7997801	-1,07	-2,10	0,07691	-1,51	-2,85	0,00385	NM_013278	GNRHR2
7919208	-1,07	-2,10	0,02984	-1,17	-2,24	0,14976	NR_002328	CCL17
7996034	-1,07	-2,11	0,12804	-1,50	-2,82	0,09197	NM_002987	---
8143657	-1,07	-2,11	0,08722	-1,51	-2,84	0,01012	---	---
8114136	-1,07	-2,11	0,01060	-1,16	-2,23	0,03025	---	01. Sep
8000869	-1,07	-2,11	0,10316	-0,57	-1,48	0,22988	NM_052838	MOP-1
8101322	-1,07	-2,11	0,02472	-1,25	-2,37	0,00253	AB014771	---
7907008	-1,07	-2,11	0,00369	-1,13	-2,18	0,00491	---	OR3A1
8011337	-1,07	-2,11	0,18004	-1,49	-2,81	0,04779	NM_002550	ATF4
8073148	-1,07	-2,11	0,07113	-0,38	-1,30	0,04037	NM_001675	FAM81A
7983991	-1,07	-2,11	0,01696	0,17	1,12	0,32075	NM_152450	SAA1
7938758	-1,07	-2,11	0,07622	-1,81	-3,49	0,00535	NM_000331	FAM125A
8026751	-1,07	-2,11	0,00015	1,42	2,67	0,05107	NM_138401	ACR
8074153	-1,07	-2,11	0,31077	-0,29	-1,23	0,47479	NM_001097	C1QA
7898793	-1,07	-2,11	0,06423	-1,66	-3,16	0,00422	NM_015991	OR7E87P
7960896	-1,07	-2,11	0,45544	-3,18	-9,07	0,00036	ENST00000329336	PDE4C
8035408	-1,07	-2,11	0,03053	-1,78	-3,44	0,01406	NM_000923	---
8173926	-1,07	-2,11	0,09004	-2,06	-4,16	0,00009	---	EDN2
7915385	-1,08	-2,11	0,00550	-0,52	-1,43	0,35974	NM_001956	CRYBB2
8071997	-1,08	-2,11	0,05131	-1,73	-3,32	0,01341	NM_000496	OR5H14
8081200	-1,08	-2,11	0,13322	-1,25	-2,38	0,08762	ENST00000341450	---
8029845	-1,08	-2,11	0,14114	-1,62	-3,06	0,01148	---	NEURL3
8053944	-1,08	-2,11	0,03702	-0,96	-1,94	0,00499	NR_026875	ARMC5
7995193	-1,08	-2,11	0,02247	-1,80	-3,49	0,00103	NM_024742	F2
7939706	-1,08	-2,11	0,00859	-1,07	-2,10	0,00001	NM_000506	---
8144993	-1,08	-2,11	0,10770	-1,72	-3,30	0,00012	---	---
7979129	-1,08	-2,11	0,07331	-0,63	-1,55	0,19643	---	ATP2C2
7997593	-1,08	-2,11	0,00498	-0,26	-1,20	0,49887	NM_014861	---
7966070	-1,08	-2,11	0,05106	-0,58	-1,49	0,00445	---	OR14C36
7911271	-1,08	-2,11	0,05741	-1,89	-3,71	0,00095	NM_001001918	TCF23
8040827	-1,08	-2,11	0,13734	-1,55	-2,93	0,00101	NM_175769	FAM83E
8038109	-1,08	-2,11	0,09810	-1,20	-2,30	0,00503	NM_017708	---
8174664	-1,08	-2,11	0,41301	-0,39	-1,31	0,44095	---	CALN1
8139921	-1,08	-2,11	0,00313	-1,24	-2,37	0,01721	NM_001017440	IVL
7905533	-1,08	-2,11	0,01434	-0,89	-1,86	0,09234	NM_005547	---
8144514	-1,08	-2,11	0,00442	-0,62	-1,54	0,09904	---	IER5
7907904	-1,08	-2,11	0,06613	-2,12	-4,34	0,00252	NM_016545	VGLL2
8121672	-1,08	-2,11	0,03566	-1,53	-2,88	0,00177	NM_182645	ANKRD57
8044301	-1,08	-2,11	0,03030	0,70	1,63	0,10412	NM_023016	---
8070930	-1,08	-2,11	0,09200	-1,55	-2,93	0,00887	---	RPS2P45
7996879	-1,08	-2,11	0,01780	-1,15	-2,21	0,00054	AK022393	ZYG11A
7901497	-1,08	-2,11	0,13408	1,95	3,87	0,00285	NM_001004339	OR4K17
7973024	-1,08	-2,11	0,09550	-0,92	-1,89	0,07139	NM_001004715	FAM169B
7991469	-1,08	-2,11	0,07960	-1,67	-3,18	0,00601	NM_182562	MGC34796
7901802	-1,08	-2,11	0,08917	-1,57	-2,98	0,05344	BC034822	INSL3
8035345	-1,08	-2,11	0,08540	2,07	4,21	0,00139	NM_005543	PDXP
8072870	-1,08	-2,11	0,07874	-0,92	-1,89	0,28728	NM_020315	C11orf86
7941746	-1,08	-2,11	0,05814	-1,18	-2,26	0,00719	AK026328	SPATA9
8113202	-1,08	-2,11	0,00064	-1,17	-2,25	0,00486	NM_031952	OR6C70
7963878	-1,08	-2,11	0,04714	-1,05	-2,06	0,03394	NM_001005499	HCG27
8118093	-1,08	-2,11	0,14481	-1,37	-2,59	0,17521	NR_026791	---
8142446	-1,08	-2,11	0,15584	-1,22	-2,32	0,02265	---	CITED1
8173524	-1,08	-2,11	0,03178	-1,19	-2,28	0,00182	NM_001144886	---
7899484	-1,08	-2,11	0,00143	-1,03	-2,04	0,00042	---	ANKRD22
7934898	-1,08	-2,11	0,11444	-0,35	-1,27	0,24958	NM_144590	CD5
7940409	-1,08	-2,11	0,07869	-1,68	-3,20	0,00025	NM_014207	MMP23B
7897006	-1,08	-2,11	0,10491	-2,53	-5,79	0,00119	NM_006983	---
8067963	-1,08	-2,11	0,00179	-1,75	-3,37	0,00021	---	RNU2-1
8019709	-1,08	-2,11	0,39872	-2,51	-5,71	0,00550	NR_002716	EMILIN1
8040792	-1,08	-2,11	0,01925	-1,33	-2,51	0,01223	NM_007046	PGLYRP4
7920228	-1,08	-2,11	0,02203	-0,73	-1,65	0,02972	NM_020393	SLURP1
8153336	-1,08	-2,11	0,24632	-1,26	-2,39	0,02806	NM_020427	---
8042974	-1,08	-2,11	0,00878	-1,34	-2,52	0,03222	---	---
8095543	-1,08	-2,11	0,00905	-1,78	-3,43	0,00194	---	MTMR11
7919669	-1,08	-2,11	0,01054	0,01	1,01	0,97352	NM_001145862	---

8114918	-1,08	-2,11	0,21420	-2,08	-4,23	0,00049	---	---
8168468	-1,08	-2,11	0,08754	-1,09	-2,12	0,03825	---	---
8176280	-1,08	-2,11	0,08754	-1,09	-2,12	0,03825	---	---
7942611	-1,08	-2,11	0,07577	-2,40	-5,27	0,00024	---	---
8137433	-1,08	-2,11	0,05168	-0,36	-1,28	0,15546	---	---
8124482	-1,08	-2,11	0,04122	-1,13	-2,18	0,00632	---	---
8017148	-1,08	-2,11	0,05804	-1,18	-2,27	0,00438	---	KIR3DL3
8039820	-1,08	-2,11	0,14936	-2,27	-4,84	0,00081	NM_153443	TNFRSF1B
7897877	-1,08	-2,11	0,00001	-1,46	-2,75	0,01728	NM_001066	GLOD5
8167356	-1,08	-2,11	0,03561	-1,54	-2,91	0,00482	NM_001080489	MIR24-2
8034694	-1,08	-2,11	0,07138	-0,77	-1,70	0,00366	NR_029497	OR2T34
7925749	-1,08	-2,11	0,37048	-2,17	-4,50	0,03414	NM_001001821	AMHR2
7955797	-1,08	-2,11	0,01470	-1,36	-2,57	0,00321	NM_020547	PVRL4
7921773	-1,08	-2,11	0,02811	-0,53	-1,44	0,11432	NM_030916	FRS3
8126337	-1,08	-2,11	0,06786	-0,92	-1,90	0,12592	NM_006653	SERPINA5
7976488	-1,08	-2,12	0,02736	-1,83	-3,56	0,00558	NM_000624	MIR125B1
7952307	-1,08	-2,12	0,08079	-1,15	-2,22	0,00016	NR_029671	LOC441178
8130807	-1,08	-2,12	0,01713	-0,75	-1,68	0,06232	AL832737	LOC441178
8130980	-1,08	-2,12	0,01713	-0,75	-1,68	0,06232	AL832737	ZNF444
8031617	-1,08	-2,12	0,03818	-1,45	-2,73	0,00750	NM_018337	---
8021045	-1,08	-2,12	0,01458	-0,81	-1,76	0,12554	---	CUZD1
7936798	-1,08	-2,12	0,00303	2,91	7,53	0,00047	NM_022034	---
8015757	-1,08	-2,12	0,29814	-1,22	-2,32	0,01077	---	LOC341912
7980306	-1,08	-2,12	0,03348	-1,07	-2,09	0,07415	ENST00000238638	RPTN
7920146	-1,08	-2,12	0,01031	-2,05	-4,13	0,00076	NM_001122965	NCKAP1L
7955908	-1,08	-2,12	0,00253	-1,19	-2,28	0,06763	NM_005337	---
8016787	-1,08	-2,12	0,25935	-1,91	-3,75	0,01690	---	FITM1
7973580	-1,08	-2,12	0,11994	-1,67	-3,18	0,00104	NM_203402	APOA1
7951865	-1,08	-2,12	0,05864	-1,46	-2,74	0,00038	NM_000039	FLJ43950
8156090	-1,08	-2,12	0,30178	-2,10	-4,28	0,00725	NR_026851	C16orf78
7995442	-1,08	-2,12	0,07114	-1,38	-2,60	0,00371	BC021718	ASGR2
8012028	-1,08	-2,12	0,02131	-0,61	-1,53	0,02734	NM_080912	VARS2
8118028	-1,08	-2,12	0,03913	0,55	1,46	0,19452	NM_020442	PPP2R3B
8171087	-1,08	-2,12	0,02229	-0,01	-1,01	0,98681	NM_013239	---
7989195	-1,08	-2,12	0,30378	-2,44	-5,42	0,02383	---	POC1A
8087839	-1,08	-2,12	0,01080	0,53	1,44	0,07568	NM_015426	PRR5
8073705	-1,08	-2,12	0,01334	0,26	1,20	0,52867	NM_181333	ADH7
8101904	-1,08	-2,12	0,15393	-1,54	-2,90	0,00085	NM_000673	TAC3
7964303	-1,08	-2,12	0,03192	-1,91	-3,77	0,00072	NR_033654	---
8081818	-1,08	-2,12	0,01656	-1,84	-3,59	0,00080	---	CCDC38
7965589	-1,08	-2,12	0,00697	-0,79	-1,73	0,01348	NM_182496	---
7941563	-1,08	-2,12	0,25991	-1,18	-2,27	0,04195	---	WNT10A
8048452	-1,08	-2,12	0,01060	-0,55	-1,46	0,03867	NM_025216	---
8173549	-1,08	-2,12	0,03667	-2,09	-4,25	0,00172	---	MYL5
8093386	-1,08	-2,12	0,03271	-0,91	-1,88	0,01671	NM_002477	C15orf54
7982593	-1,08	-2,12	0,02786	-1,18	-2,26	0,00737	NM_207445	---
7899251	-1,08	-2,12	0,06430	-1,23	-2,35	0,00241	---	LAG3
7953418	-1,08	-2,12	0,04881	-1,28	-2,43	0,00115	NM_002286	PAX7
7898537	-1,08	-2,12	0,05551	-1,22	-2,32	0,02531	NM_002584	HRH3
8067394	-1,08	-2,12	0,14783	-2,07	-4,20	0,01791	NM_007232	TAGAP
8130539	-1,08	-2,12	0,10149	-1,61	-3,04	0,00034	NM_054114	OR6K2
7921420	-1,08	-2,12	0,00412	-2,07	-4,21	0,01891	NM_001005279	MIR224
8175683	-1,08	-2,12	0,11048	-1,06	-2,08	0,01152	NR_029638	SV2C
8106336	-1,08	-2,12	0,06667	-1,52	-2,87	0,00540	NM_014979	FLJ38109
8109426	-1,08	-2,12	0,00678	-0,55	-1,46	0,00683	AK090581	---
8086181	-1,08	-2,12	0,09688	-1,79	-3,45	0,00165	---	S100G
8166213	-1,08	-2,12	0,00043	-0,74	-1,67	0,07868	NM_004057	---
8122196	-1,08	-2,12	0,03476	-0,47	-1,38	0,15524	---	VARS2
8177901	-1,08	-2,12	0,01494	0,49	1,41	0,20711	NM_020442	L3MBTL
8062705	-1,08	-2,12	0,11349	-0,30	-1,23	0,26173	NM_032107	EPN1
8031550	-1,09	-2,12	0,00149	0,25	1,19	0,46556	NM_001130071	CACNA1E
7907911	-1,09	-2,12	0,00357	-1,36	-2,57	0,00015	NM_000721	---
7907788	-1,09	-2,12	0,05870	-1,49	-2,81	0,05331	---	TTC21A
8078857	-1,09	-2,12	0,00840	-0,65	-1,56	0,00186	NM_145755	GDF10
7933372	-1,09	-2,12	0,05561	-1,24	-2,37	0,03425	NM_004962	C15orf26
7985352	-1,09	-2,12	0,01903	-1,72	-3,29	0,00118	AK302120	SFTPB
8053467	-1,09	-2,12	0,08554	1,80	3,48	0,01629	NM_000542	DUSP27
7907053	-1,09	-2,12	0,00138	-1,62	-3,08	0,00468	NM_001080426	OR8I2
7939965	-1,09	-2,12	0,01452	-1,29	-2,45	0,00000	NM_001003750	TXNDC3
8132349	-1,09	-2,12	0,01548	-0,83	-1,78	0,06623	NM_016616	OR51I2
7938006	-1,09	-2,12	0,04368	-1,07	-2,10	0,01549	NM_001004754	NEUROD6
8138882	-1,09	-2,12	0,00778	-1,48	-2,79	0,01035	NM_022728	CCDC116
8071554	-1,09	-2,12	0,11830	-0,93	-1,90	0,14401	NM_152612	---

7953695	-1,09	-2,12	0,06094	-1,21	-2,32	0,00208	---	SHD	
8024808	-1,09	-2,12	0,03003	-1,62	-3,08	0,00005	NM_020209		FAM27A
8155572	-1,09	-2,12	0,30965	-0,31	-1,24	0,25128	NR_024060		---
8081032	-1,09	-2,12	0,12476	-2,00	-4,01	0,00087	---	LOC254028	
7973080	-1,09	-2,12	0,13300	-1,42	-2,68	0,10120	AK093210	REEP1	
8053551	-1,09	-2,12	0,02711	0,03	1,02	0,89943	NM_022912		CD69
7961075	-1,09	-2,12	0,04063	-1,17	-2,26	0,01460	NM_001781		---
7967586	-1,09	-2,12	0,08050	-1,67	-3,19	0,00991	---	---	
8145912	-1,09	-2,12	0,12895	-1,33	-2,51	0,03794	---	---	
8104609	-1,09	-2,12	0,09201	-1,78	-3,43	0,01046	---	---	
8129899	-1,09	-2,12	0,02564	-0,33	-1,26	0,06266	---	KCNG2	
8021900	-1,09	-2,12	0,04117	-0,58	-1,50	0,21425	NM_012283		C1orf46
7905496	-1,09	-2,12	0,15611	-1,33	-2,52	0,13162	AF005082	---	
8042530	-1,09	-2,13	0,09864	-1,19	-2,28	0,00828	---	TMEM108	
8082767	-1,09	-2,13	0,00307	0,42	1,34	0,07408	NM_023943		---
8059862	-1,09	-2,13	0,10862	-1,43	-2,69	0,00379	---	---	
8129693	-1,09	-2,13	0,09932	-1,22	-2,33	0,00535	---	---	
8053735	-1,09	-2,13	0,36184	-2,52	-5,74	0,00028	---	COL20A1	
8064059	-1,09	-2,13	0,05952	-0,65	-1,56	0,12065	NM_020882		---
7927704	-1,09	-2,13	0,00147	-0,93	-1,90	0,05493	---	TRIP6	
8134981	-1,09	-2,13	0,00537	-0,63	-1,54	0,17937	NM_003302		CD53
7903893	-1,09	-2,13	0,00699	-0,38	-1,30	0,10244	NM_000560		SCRIB
8153497	-1,09	-2,13	0,01154	-0,21	-1,16	0,53210	NM_182706		DBP
8038117	-1,09	-2,13	0,07005	-0,23	-1,17	0,58878	NM_001352		ZNF491
8025951	-1,09	-2,13	0,01466	-0,78	-1,72	0,01202	NM_152356		ADAM18
8146069	-1,09	-2,13	0,05940	-0,61	-1,53	0,01123	NM_014237		GLRA2
8166127	-1,09	-2,13	0,00402	-1,25	-2,37	0,04767	NM_002063		CARTPT
8106088	-1,09	-2,13	0,02748	-1,54	-2,91	0,00123	NM_004291		CARTPT
8177620	-1,09	-2,13	0,02748	-1,54	-2,91	0,00123	NM_004291		---
8052119	-1,09	-2,13	0,06588	-1,15	-2,22	0,02619	---	---	
7948135	-1,09	-2,13	0,04426	-1,44	-2,71	0,00540	---	CDHR5	
7945475	-1,09	-2,13	0,10630	-2,02	-4,07	0,00049	NM_021924		SNORA28
7977075	-1,09	-2,13	0,01704	-0,88	-1,85	0,00121	NR_002964		C9orf169
8165639	-1,09	-2,13	0,10013	-1,29	-2,45	0,00958	NM_199001		SLC28A2
7983447	-1,09	-2,13	0,00164	-0,85	-1,80	0,02036	NM_004212		SNORD82
8059712	-1,09	-2,13	0,06482	-0,98	-1,97	0,00325	NR_004398		SMCR7
8005435	-1,09	-2,13	0,02471	0,55	1,47	0,15009	NM_148886		SIGLEC7
8030789	-1,09	-2,13	0,01468	-1,06	-2,08	0,00084	NM_014385		SNORD20
8059710	-1,09	-2,13	0,00381	-1,13	-2,19	0,00107	NR_002908		NPCDR1
8088468	-1,09	-2,13	0,13531	-2,08	-4,23	0,00805	AF134979	FMNL1	
8007757	-1,09	-2,13	0,00630	2,18	4,55	0,00190	NM_005892		C9orf79
8156247	-1,09	-2,13	0,05076	-1,19	-2,27	0,01859	NM_178828		KCNJ3
8045795	-1,09	-2,13	0,05230	-1,47	-2,76	0,01132	NM_002239		GPX5
8117700	-1,09	-2,13	0,14753	-0,99	-1,98	0,04068	NM_001509		DPY19L2
7964665	-1,09	-2,13	0,27585	-0,98	-1,97	0,04877	NM_173812		BTN2A3
8117470	-1,09	-2,13	0,01906	-1,07	-2,10	0,15138	NR_027795		REXO1L2P
8151615	-1,09	-2,13	0,57159	-5,85	-57,59	0,00006	NR_003594		---
8083592	-1,09	-2,13	0,24067	-1,52	-2,88	0,03142	---	FOXD4L1	
8044634	-1,09	-2,13	0,08626	-1,70	-3,25	0,00807	NM_012184		OR5R1
7948129	-1,09	-2,13	0,00225	-1,35	-2,55	0,00366	NM_001004744		KIR3DL3
8031260	-1,09	-2,13	0,10598	-2,02	-4,07	0,00003	NM_153443		DNAH7
8057821	-1,09	-2,13	0,01115	-0,98	-1,97	0,03811	NM_018897		---
8092968	-1,09	-2,13	0,03158	-0,58	-1,50	0,10746	---	TFR2	
8141560	-1,09	-2,13	0,08159	-0,97	-1,96	0,26042	NM_003227		MAPK8IP3
7992347	-1,09	-2,13	0,08032	0,28	1,21	0,31713	NM_015133		TSPYL6
8052200	-1,09	-2,13	0,07513	-1,72	-3,30	0,03998	NM_001003937		---
8021725	-1,09	-2,13	0,00701	-1,87	-3,65	0,01429	---	DHRS7C	
8012591	-1,09	-2,13	0,10472	-1,56	-2,96	0,01269	NM_001105571		TCL1B
7976544	-1,09	-2,13	0,00215	-1,32	-2,50	0,00083	NM_199206		NPR1
7905606	-1,09	-2,13	0,05062	-0,71	-1,64	0,10816	NM_000906		CNTN2
7909064	-1,09	-2,13	0,05088	-1,39	-2,62	0,00267	NM_005076		C8orf79
8144705	-1,09	-2,13	0,05013	-0,61	-1,53	0,02512	NM_020844		CYP2A7
8036981	-1,09	-2,13	0,19361	-0,81	-1,75	0,09327	NM_000764		LUZP4
8169467	-1,09	-2,13	0,07495	-1,29	-2,45	0,00022	NM_016383		LRGUK
8136315	-1,09	-2,13	0,04087	-0,65	-1,56	0,19397	NM_144648		GZMK
8105331	-1,09	-2,13	0,00164	-1,27	-2,42	0,02802	NM_002104		LHCGR
8052058	-1,09	-2,13	0,01500	-0,70	-1,62	0,00931	NM_000233		---
8064500	-1,09	-2,13	0,00842	-0,92	-1,89	0,01717	---	AKD1	
8128777	-1,09	-2,13	0,01112	-0,96	-1,94	0,06258	NM_001145128		PDZD9
8000229	-1,09	-2,13	0,00040	-1,12	-2,18	0,01336	NM_173806		CRAMP1L
7992302	-1,09	-2,13	0,01081	-1,18	-2,27	0,00617	NM_020825		FLJ26850
8030578	-1,09	-2,13	0,20767	-1,34	-2,53	0,00072	ENST00000377006		ERG
8070297	-1,09	-2,13	0,06704	-1,74	-3,34	0,00814	NM_001136154		PIM3

8073960	-1,09	-2,13	0,03765	-1,15	-2,22	0,01881	NM_001001852	MMP9
8063115	-1,09	-2,13	0,09196	-1,51	-2,84	0,00722	NM_004994	---
8143142	-1,09	-2,13	0,01211	-1,31	-2,47	0,00062	---	FLT3
7970737	-1,09	-2,13	0,00916	-0,81	-1,75	0,01218	NM_004119	REXO1
8032347	-1,09	-2,13	0,00448	0,31	1,24	0,54172	NM_020695	KIR2DL2
8039829	-1,09	-2,13	0,17996	-2,15	-4,43	0,00005	NM_014219	MIR520C // MIR520C // MIR520C // MIR520C // MIR520C
8031029	-1,09	-2,13	0,04651	-1,29	-2,45	0,02687	NR_030198 // NR_030198 // NR_030198 // NR_030198 // NR_030198	MIR193A
8006321	-1,09	-2,13	0,20250	-1,39	-2,62	0,00656	NR_029710	CCDC37
8082270	-1,09	-2,13	0,00095	-1,11	-2,16	0,00296	NM_182628	MIR1826
7995308	-1,09	-2,13	0,15934	-1,04	-2,06	0,00663	NR_031727	ADIG
8062527	-1,09	-2,13	0,02055	-2,57	-5,96	0,00016	NM_001018082	OTOR
8061082	-1,09	-2,13	0,05379	-1,22	-2,33	0,00802	NM_020157	---
7904166	-1,09	-2,13	0,31563	-3,42	-10,72	0,00083	---	FLG
7920165	-1,09	-2,13	0,11075	-1,27	-2,42	0,01316	NM_002016	C20orf187
8060986	-1,09	-2,13	0,01468	-1,09	-2,12	0,02558	ENST00000378252	---
8146120	-1,09	-2,13	0,03462	-1,05	-2,07	0,07317	---	---
7938590	-1,09	-2,13	0,04916	-1,96	-3,90	0,00024	---	---
7970735	-1,09	-2,13	0,19920	-0,48	-1,40	0,08144	---	SFTPA1
7928602	-1,09	-2,13	0,09252	-1,27	-2,42	0,00057	NM_005411	SFTPA1
7928632	-1,09	-2,13	0,09252	-1,27	-2,42	0,00057	NM_005411	OTUD7A
7987099	-1,09	-2,14	0,08905	-1,03	-2,04	0,01752	NM_130901	LOC390561
7982299	-1,09	-2,14	0,14029	-0,63	-1,54	0,12525	ENST00000220188	HTR5A
8137517	-1,09	-2,14	0,05731	-2,04	-4,11	0,00546	NM_024012	C9orf84
8163348	-1,09	-2,14	0,00110	0,08	1,05	0,71580	NM_173521	CD79B
8017511	-1,09	-2,14	0,02588	-1,77	-3,41	0,00746	NM_000626	---
8005104	-1,09	-2,14	0,01556	-0,96	-1,95	0,02551	---	---
8109177	-1,09	-2,14	0,00466	-1,08	-2,12	0,06271	---	---
8096312	-1,09	-2,14	0,08798	-1,14	-2,20	0,00040	---	KIAA1984
8159385	-1,09	-2,14	0,00112	-0,52	-1,44	0,14418	NM_001039374	BSPH1
8037959	-1,09	-2,14	0,00134	-0,93	-1,91	0,00524	NM_001128326	CWC25
8014738	-1,10	-2,14	0,04362	-0,28	-1,21	0,33984	NM_017748	---
7918876	-1,10	-2,14	0,03632	-1,62	-3,07	0,00659	---	CYTL1
8099132	-1,10	-2,14	0,09455	-2,37	-5,18	0,00823	NM_018659	CCDC78
7998307	-1,10	-2,14	0,02070	-1,44	-2,72	0,00508	NM_001031737	---
7898572	-1,10	-2,14	0,04023	-1,28	-2,42	0,02986	---	PPP1R3D
8067351	-1,10	-2,14	0,00358	-1,14	-2,20	0,00801	NM_006242	---
8104632	-1,10	-2,14	0,03468	-0,97	-1,97	0,01563	---	OPRK1
8150785	-1,10	-2,14	0,05777	-1,26	-2,39	0,00341	NM_000912	---
8105409	-1,10	-2,14	0,22116	-0,61	-1,53	0,14508	---	APOB48R
7994353	-1,10	-2,14	0,01358	-1,59	-3,01	0,00146	NM_018690	S100B
8071036	-1,10	-2,14	0,08088	-1,29	-2,45	0,01652	NM_006272	PDILT
7999952	-1,10	-2,14	0,04868	-1,49	-2,82	0,02587	NM_174924	LOC100131792
8107720	-1,10	-2,14	0,10516	-1,34	-2,53	0,01023	AK093561	SPATA16
8092187	-1,10	-2,14	0,26242	-1,62	-3,08	0,00942	NM_031955	SNORD75
7922416	-1,10	-2,14	0,06112	-1,39	-2,62	0,00345	NR_003941	FAM90A13
8144424	-1,10	-2,14	0,44681	-2,43	-5,40	0,00547	NM_001164456	FAM90A13
8144440	-1,10	-2,14	0,44681	-2,43	-5,40	0,00547	NM_001164456	FBXO24
8134856	-1,10	-2,14	0,03210	-0,20	-1,15	0,21279	NM_033506	PPP2R3B
8176986	-1,10	-2,14	0,01342	0,09	1,07	0,86391	NM_013239	OR11A1
8124639	-1,10	-2,14	0,00213	-1,09	-2,14	0,00808	NM_013937	C15orf53
7982587	-1,10	-2,14	0,05774	-1,50	-2,83	0,03763	NM_207444	REG3G
8042978	-1,10	-2,14	0,06972	-0,89	-1,85	0,00046	NM_001008387	SOCS1
7999423	-1,10	-2,14	0,04832	-1,87	-3,64	0,00592	NM_003745	IRF5
8136045	-1,10	-2,14	0,00034	-0,31	-1,24	0,37148	NM_001098629	---
8108299	-1,10	-2,14	0,00845	-1,72	-3,29	0,01501	---	C21orf45
8069933	-1,10	-2,14	0,00078	2,02	4,07	0,00784	NM_018944	---
8113782	-1,10	-2,14	0,00774	-0,85	-1,80	0,03003	---	---
7927872	-1,10	-2,14	0,04517	-0,75	-1,69	0,16451	---	---
7961024	-1,10	-2,14	0,13408	-2,31	-4,96	0,03786	---	ABCA7
8024120	-1,10	-2,14	0,00470	0,47	1,38	0,22755	NM_019112	FBXL19
7994967	-1,10	-2,14	0,03589	-0,75	-1,69	0,03553	NM_001099784	HLA-DQA2
8118564	-1,10	-2,14	0,03675	-2,25	-4,75	0,00260	NM_020056	ITIH3
8080466	-1,10	-2,14	0,04355	-1,47	-2,77	0,01130	NM_002217	HYI
7915529	-1,10	-2,14	0,04552	-0,42	-1,34	0,04371	NM_031207	---
7926059	-1,10	-2,14	0,07852	-0,82	-1,77	0,03651	---	---
8146898	-1,10	-2,14	0,12953	-1,36	-2,57	0,01841	---	---
7942910	-1,10	-2,14	0,10059	-1,36	-2,56	0,00017	---	OVOL1
7941401	-1,10	-2,14	0,00096	0,26	1,20	0,33816	NM_004561	MMP21
7936928	-1,10	-2,14	0,00947	-1,33	-2,52	0,00132	NM_147191	CDH3
7996819	-1,10	-2,14	0,00073	3,92	15,09	0,00023	NM_001793	---
8112724	-1,10	-2,14	0,09203	-0,87	-1,83	0,08024	---	IRGQ

8037352	-1,10	-2,14	0,00249	-1,06	-2,09	0,02665	NM_001007561	---
8161240	-1,10	-2,14	0,02272	-1,16	-2,24	0,00056	---	SNORD115-32
7982070	-1,10	-2,14	0,01174	-0,73	-1,65	0,01623	NR_003347	---
8147305	-1,10	-2,14	0,02438	-0,78	-1,72	0,04945	---	DMRTC1
8173583	-1,10	-2,14	0,03185	-0,57	-1,49	0,18288	NM_033053	---
7972561	-1,10	-2,14	0,37660	-1,10	-2,15	0,02110	---	---
8048466	-1,10	-2,14	0,03126	-1,09	-2,12	0,00533	---	ALOX12B
8012309	-1,10	-2,14	0,00531	-0,51	-1,42	0,07596	NM_001139	---
8045602	-1,10	-2,14	0,01097	-1,36	-2,56	0,00983	---	CABP4
7941917	-1,10	-2,14	0,06085	-1,78	-3,45	0,00371	NM_145200	SLCO5A1
8151223	-1,10	-2,14	0,02324	0,29	1,23	0,11562	NM_030958	LMAN1L
7984892	-1,10	-2,14	0,00295	-1,18	-2,26	0,03996	NM_021819	RNU6ATAC // RNU6ATAC // RNU6ATAC // RNU6ATAC // RNU6ATAC
8112176	-1,10	-2,14	0,00947	-1,55	-2,93	0,00251	NR_023344 // NR_023344 // NR_023344 // NR_023344 // NR_023344	---
8006317	-1,10	-2,14	0,00328	-0,91	-1,88	0,00200	---	FBXL22
7984152	-1,10	-2,14	0,00707	-1,68	-3,20	0,00266	NM_203373	MICALCL
7938519	-1,10	-2,14	0,08613	-0,83	-1,78	0,12208	NM_032867	SPRR1A
7905544	-1,10	-2,14	0,03363	-2,13	-4,37	0,02066	NM_005987	PRKCB
7994131	-1,10	-2,14	0,00667	-1,32	-2,50	0,00004	NM_002738	NLE1
8014214	-1,10	-2,14	0,00018	0,37	1,29	0,12858	NM_001014445	---
8104650	-1,10	-2,15	0,00810	-1,31	-2,49	0,01572	---	---
8085606	-1,10	-2,15	0,07856	-1,56	-2,95	0,01164	---	BRSK2
7937667	-1,10	-2,15	0,07606	-0,31	-1,24	0,11650	NM_003957	POU4F2
8097687	-1,10	-2,15	0,00876	-1,74	-3,34	0,01870	NM_004575	ARF6
7974249	-1,10	-2,15	0,36618	-0,60	-1,51	0,63965	NM_001663	NCRNA00175
8070905	-1,10	-2,15	0,05126	-0,55	-1,46	0,05393	NR_028082	SYT3
8038587	-1,10	-2,15	0,07432	-0,38	-1,30	0,50864	NM_032298	TGFA
8052872	-1,10	-2,15	0,02579	0,68	1,61	0,10682	NM_003236	TOMM40
8029521	-1,10	-2,15	0,01049	-0,43	-1,35	0,22053	NM_001128917	---
8151434	-1,10	-2,15	0,08162	-0,88	-1,84	0,01054	---	OBSCN
7924863	-1,10	-2,15	0,11595	1,70	3,26	0,00013	NM_052843	CRABP1
7990712	-1,10	-2,15	0,07431	-0,79	-1,73	0,01491	NM_004378	C15orf27
7985066	-1,10	-2,15	0,02793	-0,02	-1,01	0,97815	NM_152335	---
7924386	-1,10	-2,15	0,00981	-2,38	-5,21	0,00000	---	HSD11B1L
8024956	-1,10	-2,15	0,03229	-0,75	-1,69	0,07536	NM_198706	---
7943191	-1,10	-2,15	0,36313	-0,72	-1,64	0,02441	---	---
8005992	-1,10	-2,15	0,00129	-1,04	-2,06	0,02925	---	PRICKLE3
8172561	-1,10	-2,15	0,00100	0,26	1,20	0,59228	NM_006150	GGTLC2
8071662	-1,10	-2,15	0,14620	-0,70	-1,63	0,10223	NM_199127	DEFB124
8065566	-1,10	-2,15	0,19573	-0,65	-1,57	0,36201	NM_001037500	C20orf186
8061831	-1,10	-2,15	0,01305	-0,82	-1,76	0,00080	NM_182519	MRPS2
8159249	-1,10	-2,15	0,10362	2,74	6,70	0,01026	NM_016034	ESPN
7897288	-1,10	-2,15	0,07956	-0,63	-1,55	0,06182	NM_031475	PTGES
8164580	-1,10	-2,15	0,01338	-0,01	-1,00	0,98892	NM_004878	NPY6R
8108251	-1,10	-2,15	0,10481	-1,29	-2,45	0,02950	NR_002713	CPN1
7935679	-1,10	-2,15	0,00771	-1,29	-2,44	0,00512	NM_001308	VN1R5
7911174	-1,10	-2,15	0,10012	-1,35	-2,55	0,00051	NM_173858	---
8112894	-1,10	-2,15	0,15959	-2,53	-5,76	0,02886	---	TNRC18
8137942	-1,10	-2,15	0,05506	-0,15	-1,11	0,85858	NM_001080495	CST11
8065388	-1,10	-2,15	0,00574	-0,58	-1,50	0,03889	NM_130794	ISG20
7985777	-1,10	-2,15	0,00531	-0,21	-1,16	0,55693	NM_002201	C1orf180
7917251	-1,10	-2,15	0,00022	-0,53	-1,44	0,00219	NR_027379	TFAP2D
8120184	-1,10	-2,15	0,01785	-0,52	-1,43	0,02604	NM_172238	GNG4
7925250	-1,10	-2,15	0,00875	1,73	3,31	0,00614	NM_001098721	---
8112607	-1,10	-2,15	0,09725	-2,31	-4,97	0,02354	---	---
8094776	-1,10	-2,15	0,09536	-0,68	-1,60	0,03492	---	---
7926894	-1,10	-2,15	0,07155	-0,77	-1,71	0,05633	---	CYP4A11
7915902	-1,10	-2,15	0,09331	-1,44	-2,70	0,04837	NM_000778	---
7939986	-1,10	-2,15	0,00306	-1,20	-2,30	0,00213	---	TIGIT
8081799	-1,10	-2,15	0,09020	-0,47	-1,38	0,26836	NM_173799	---
7950530	-1,10	-2,15	0,10602	-1,34	-2,53	0,00014	---	VNN3
8129627	-1,10	-2,15	0,00622	-1,34	-2,53	0,00176	NR_028290	TNFAIP2
7977046	-1,10	-2,15	0,00058	1,76	3,39	0,00790	NM_006291	KLHDC4
8003283	-1,10	-2,15	0,03120	0,14	1,10	0,64492	NM_017566	SNORD42B
8005951	-1,11	-2,15	0,03219	-1,16	-2,24	0,04539	NR_000013	GMFG
8036710	-1,11	-2,15	0,04657	-0,97	-1,96	0,00011	NM_004877	---
7953975	-1,11	-2,15	0,00755	-1,27	-2,41	0,03867	---	GTPBP6
8171079	-1,11	-2,15	0,12037	-1,27	-2,42	0,03821	ENST00000326153	NPFF
7963689	-1,11	-2,15	0,07209	-1,04	-2,05	0,05979	NM_003717	KRTAP4-11 // KRTAP4-11
8019570	-1,11	-2,15	0,21297	-2,92	-7,59	0,00099	NM_033059 // NM_033059	OTOP3
8009713	-1,11	-2,15	0,04126	-1,21	-2,31	0,00954	NM_178233	DLX4

8008228	-1,11	-2,15	0,07887	-0,33	-1,26	0,54215	NM_138281		---
8074888	-1,11	-2,15	0,05848	0,71	1,64	0,16156	---	TSNAXIP1	
7996654	-1,11	-2,15	0,00852	-0,83	-1,78	0,00941	NM_018430		SPAG11B
8144460	-1,11	-2,15	0,01094	-1,06	-2,09	0,00444	NM_016512		GFI1
7917697	-1,11	-2,15	0,03221	-0,62	-1,53	0,36231	NM_005263		HSPA12B
8060660	-1,11	-2,15	0,00735	-1,60	-3,02	0,00003	NM_052970		---
8081995	-1,11	-2,15	0,10180	-1,25	-2,38	0,00004	---	PRAMEF4	
7912555	-1,11	-2,15	0,16717	-0,92	-1,89	0,01504	NM_001009611		C4orf19
8094550	-1,11	-2,15	0,03548	-1,14	-2,21	0,00694	NM_001104629		OR13A1
7933219	-1,11	-2,15	0,04382	-1,60	-3,04	0,00165	NM_001004297		---
8053840	-1,11	-2,15	0,02400	-1,74	-3,34	0,00516	---	HMGA2	
7964736	-1,11	-2,15	0,08193	-1,05	-2,07	0,11934	AY601863	MYCL1	
7915277	-1,11	-2,15	0,06201	-1,13	-2,18	0,07870	NM_005376		GALNTL6
8098307	-1,11	-2,15	0,15126	-1,45	-2,74	0,00222	NM_001034845		ARHGAP25
8042416	-1,11	-2,15	0,00001	0,28	1,22	0,35335	NM_014882		FAM22A
7928653	-1,11	-2,16	0,11421	-0,85	-1,80	0,08393	NM_001099338		DYDC2
7928685	-1,11	-2,16	0,05160	-0,66	-1,58	0,00301	NM_032372		LSR
8027793	-1,11	-2,16	0,00311	1,97	3,92	0,00705	NM_205834		SLC2A5
7912224	-1,11	-2,16	0,02465	-1,96	-3,90	0,00960	NM_003039		SP6
8016382	-1,11	-2,16	0,03183	-0,22	-1,17	0,63089	NM_199262		C13orf16
7970139	-1,11	-2,16	0,00149	-0,84	-1,78	0,31978	BC029889	TTLL2	
8123398	-1,11	-2,16	0,08234	-1,59	-3,00	0,03086	NM_031949		PROM2
8043522	-1,11	-2,16	0,00617	-0,81	-1,75	0,19797	NM_001165978		---
8096075	-1,11	-2,16	0,03024	-1,17	-2,25	0,00010	---	EPS8L1	
8031414	-1,11	-2,16	0,02227	-0,40	-1,32	0,27295	NM_133180		---
7965150	-1,11	-2,16	0,00962	-0,97	-1,96	0,00714	---	CTSL2	
8162652	-1,11	-2,16	0,08817	0,56	1,47	0,05881	NM_001333		LOC439938
8124500	-1,11	-2,16	0,04141	0,49	1,40	0,42608	AK095259	DSG4	
8020740	-1,11	-2,16	0,00756	-1,33	-2,51	0,00358	NM_001134453		PTGER3
7916944	-1,11	-2,16	0,00553	-1,37	-2,58	0,00007	NR_028294		PUS1
7959986	-1,11	-2,16	0,00879	0,66	1,58	0,36926	NM_025215		MGC39545
7944972	-1,11	-2,16	0,02720	-1,41	-2,65	0,01669	BC036197	GML	
8148565	-1,11	-2,16	0,02357	-0,77	-1,71	0,01672	NM_002066		ZIC2
7969830	-1,11	-2,16	0,02813	-0,06	-1,04	0,94643	NM_007129		B3GNT3
8026861	-1,11	-2,16	0,12129	-0,16	-1,12	0,61254	NM_014256		---
7919570	-1,11	-2,16	0,00214	-1,08	-2,11	0,16623	---	ANKS4B	
7993815	-1,11	-2,16	0,02121	-1,30	-2,47	0,00046	NM_145865		GPR179
8014666	-1,11	-2,16	0,04545	-1,56	-2,94	0,00493	NM_001004334		---
7927056	-1,11	-2,16	0,19907	-2,23	-4,71	0,00878	---	CD3D	
7952056	-1,11	-2,16	0,02206	-0,69	-1,61	0,00117	NM_000732		---
7936994	-1,11	-2,16	0,00199	-1,30	-2,47	0,00464	---	KLHL36	
7997626	-1,11	-2,16	0,00636	1,13	2,19	0,15853	NM_024731		RAB25
7906079	-1,11	-2,16	0,08977	1,01	2,01	0,07060	NM_020387		---
8094531	-1,11	-2,16	0,03099	-1,13	-2,19	0,00046	---	---	
8013917	-1,11	-2,16	0,04222	-1,32	-2,50	0,00011	---	---	
7930915	-1,11	-2,16	0,06645	-0,12	-1,09	0,06411	---	ZNF519	
8022420	-1,11	-2,16	0,00196	-0,51	-1,43	0,01128	NM_145287		---
8085370	-1,11	-2,16	0,11488	-0,57	-1,48	0,48018	---	OR8G2	
7944829	-1,11	-2,16	0,00883	-1,55	-2,93	0,00026	ENST00000318460		NMUR2
8115368	-1,11	-2,16	0,01620	-1,16	-2,23	0,00529	NM_020167		FGF12
8092750	-1,11	-2,16	0,06327	1,03	2,05	0,04996	NM_021032		FIGNL2
7963328	-1,11	-2,16	0,03671	-0,56	-1,48	0,23665	NM_001013690		---
8109828	-1,11	-2,16	0,01071	-0,87	-1,83	0,03846	---	---	
7908775	-1,11	-2,16	0,14175	-0,63	-1,55	0,04467	---	LOC100132356	
8111930	-1,11	-2,16	0,16522	-1,52	-2,86	0,00000	AK075204	LOC100289181	
8055275	-1,11	-2,16	0,05218	-1,01	-2,01	0,04178	XR_078263		NDP
8172220	-1,11	-2,16	0,00639	-1,28	-2,43	0,00296	NM_000266		D4S234E
8093839	-1,11	-2,16	0,05015	-0,65	-1,57	0,09119	NM_014392		ARHGEF38
8096682	-1,11	-2,16	0,00835	-0,82	-1,77	0,03920	NM_017700		IL1F10
8044563	-1,11	-2,16	0,04412	-1,52	-2,87	0,00096	NM_032556		SNORD44
7922410	-1,11	-2,16	0,00247	-0,37	-1,29	0,22793	NR_002750		CILP2
8027222	-1,11	-2,16	0,04813	-2,19	-4,55	0,00535	NM_153221		DUOXA1
7988380	-1,11	-2,16	0,01089	-0,28	-1,21	0,44463	NM_144565		---
7966299	-1,11	-2,16	0,00958	-0,81	-1,76	0,04361	---	DUXA	
8039635	-1,11	-2,16	0,17635	-1,33	-2,51	0,00279	NM_001012729		C20orf27
8064739	-1,11	-2,16	0,00628	-0,73	-1,65	0,34799	NM_001039140		AFAP1L1
8109120	-1,11	-2,16	0,00954	-0,40	-1,32	0,15259	NM_152406		MORN5
8157632	-1,11	-2,16	0,05218	-1,52	-2,86	0,00182	NM_198469		---
8112437	-1,11	-2,16	0,01279	-1,75	-3,37	0,00582	---	CXCL14	
8114249	-1,11	-2,16	0,06408	-0,76	-1,69	0,05838	NM_004887		---
8154660	-1,11	-2,16	0,00394	-0,86	-1,82	0,01422	---	---	
7962588	-1,11	-2,16	0,00125	-1,48	-2,79	0,00067	---	C11orf44	
7945241	-1,11	-2,16	0,10456	-0,82	-1,76	0,17238	AK096377	C4orf32	

8096899	-1,11	-2,16	0,01850	-0,62	-1,54	0,19619	NM_152400	OR4C13
7948046	-1,11	-2,16	0,03766	-1,44	-2,72	0,00035	NM_001001955	---
7970507	-1,11	-2,16	0,00850	-1,67	-3,18	0,00640	---	PRG4
7908312	-1,11	-2,16	0,01631	-1,27	-2,41	0,03355	NM_005807	OR3A4
8003808	-1,11	-2,16	0,04316	-1,21	-2,31	0,02053	NR_024128	TRH
8082574	-1,11	-2,16	0,05099	-0,94	-1,92	0,02815	NM_007117	CDS1
8096130	-1,11	-2,16	0,00258	1,31	2,48	0,03531	NM_001263	---
7958273	-1,11	-2,16	0,00218	-0,62	-1,54	0,08901	---	GZMB
7978366	-1,11	-2,16	0,03698	-1,03	-2,04	0,00067	NM_004131	SPERT
7968957	-1,11	-2,16	0,01985	-1,95	-3,86	0,00347	NM_152719	LOC51152
8071049	-1,11	-2,17	0,57991	-4,57	-23,83	0,00027	AF172850	---
8048993	-1,11	-2,17	0,10945	-0,98	-1,97	0,11963	---	---
8116245	-1,11	-2,17	0,00138	-1,04	-2,05	0,00010	---	---
8142534	-1,11	-2,17	0,00593	-1,00	-1,99	0,00605	---	COL22A1
8153101	-1,11	-2,17	0,01488	-1,33	-2,51	0,02233	NM_152888	TGM3
8060432	-1,11	-2,17	0,06815	-1,80	-3,48	0,00140	NM_003245	---
8017702	-1,11	-2,17	0,22402	-0,38	-1,30	0,13586	---	---
7993255	-1,11	-2,17	0,15243	-1,83	-3,56	0,01746	---	BEX2
8174239	-1,12	-2,17	0,01379	-1,18	-2,27	0,01041	NM_032621	NR5A1
8164077	-1,12	-2,17	0,01819	-0,88	-1,84	0,02684	NM_004959	KL
7968556	-1,12	-2,17	0,06547	-0,45	-1,36	0,14261	NM_004795	DUSP9
8170695	-1,12	-2,17	0,00200	-0,83	-1,78	0,07833	NM_001395	LOC731797
8027744	-1,12	-2,17	0,10378	-1,60	-3,04	0,02372	ENST00000313865	TNFRSF11A
8021528	-1,12	-2,17	0,09733	-1,83	-3,56	0,00025	NM_003839	---
7922868	-1,12	-2,17	0,03237	-1,75	-3,36	0,00785	---	GJC3
8141371	-1,12	-2,17	0,11070	-0,83	-1,78	0,00705	NM_181538	LCN6
8165277	-1,12	-2,17	0,09232	-1,08	-2,11	0,00139	NM_198946	---
8035801	-1,12	-2,17	0,14262	-1,57	-2,98	0,02702	---	IKZF3
8014891	-1,12	-2,17	0,00432	-0,70	-1,63	0,00027	NM_012481	---
7904450	-1,12	-2,17	0,01621	-1,17	-2,25	0,04195	---	---
7919195	-1,12	-2,17	0,01621	-1,17	-2,25	0,04195	---	LGALS4
8036591	-1,12	-2,17	0,05143	-1,89	-3,69	0,00607	NM_006149	C1QB
7898805	-1,12	-2,17	0,04248	-1,55	-2,92	0,00189	NM_000491	GPSM3
8125373	-1,12	-2,17	0,14095	-0,93	-1,91	0,00045	NM_022107	GPSM3
8179987	-1,12	-2,17	0,14095	-0,93	-1,91	0,00045	NM_022107	ENTHD1
8076231	-1,12	-2,17	0,15023	-0,83	-1,77	0,02494	NM_152512	---
7910140	-1,12	-2,17	0,08249	-1,74	-3,35	0,01352	---	---
7985117	-1,12	-2,17	0,30822	-2,13	-4,38	0,02897	---	---
8024494	-1,12	-2,17	0,01273	-1,42	-2,67	0,00522	---	NCR1
8031387	-1,12	-2,17	0,02661	-1,52	-2,87	0,00128	NM_004829	EBF4
8060505	-1,12	-2,17	0,01765	-1,00	-2,00	0,15548	NM_001110514	---
7907349	-1,12	-2,17	0,12495	-1,61	-3,06	0,00063	---	DAOA
7972699	-1,12	-2,17	0,02643	-1,59	-3,00	0,00306	NM_001161812	---
7987648	-1,12	-2,17	0,00825	-1,18	-2,26	0,00062	---	ADAMTS7
7990736	-1,12	-2,17	0,20506	-1,28	-2,42	0,04384	NM_014272	PHYHIP
8149699	-1,12	-2,17	0,07766	-0,84	-1,80	0,00894	NM_001099335	ZNF663
8066739	-1,12	-2,17	0,05166	-0,89	-1,85	0,11130	ENST00000400371	MIR217
8052372	-1,12	-2,17	0,00047	-0,91	-1,88	0,12018	NR_029630	PRR10
7945125	-1,12	-2,17	0,02408	-1,75	-3,35	0,01001	BC113593	C10orf111
7932221	-1,12	-2,17	0,04017	-1,33	-2,51	0,09104	BC029034	SLC22A10
7940737	-1,12	-2,17	0,00095	-0,99	-1,99	0,00073	NM_001039752	PRSS41
7992716	-1,12	-2,17	0,04552	-1,08	-2,11	0,00341	NM_001135086	CLCA1
7902721	-1,12	-2,17	0,02792	-0,95	-1,94	0,02117	NM_001285	---
8049655	-1,12	-2,17	0,02262	-1,01	-2,01	0,01657	---	---
8044682	-1,12	-2,17	0,17255	-1,83	-3,54	0,00174	---	SNORD102
7968232	-1,12	-2,17	0,15864	-1,30	-2,47	0,00453	NR_002574	PPAP2C
8031999	-1,12	-2,17	0,14002	2,40	5,27	0,00313	NM_003712	---
8167218	-1,12	-2,17	0,01097	-0,43	-1,35	0,38714	---	DPEP2
8002194	-1,12	-2,17	0,00857	-1,81	-3,52	0,00107	NM_022355	CLCN1
8136893	-1,12	-2,17	0,01552	-1,41	-2,66	0,00024	NM_000083	FAM22A
7928619	-1,12	-2,17	0,09271	-0,76	-1,69	0,07963	NM_001099338	---
7968911	-1,12	-2,17	0,16121	-1,38	-2,60	0,01744	---	FLJ46257 // FLJ46257
8076819	-1,12	-2,17	0,07085	-0,58	-1,50	0,01162	NR_033377 // NR_033377	TMCO5A
7982552	-1,12	-2,17	0,02675	-1,21	-2,31	0,02019	NM_152453	FOXA3
8029754	-1,12	-2,17	0,07873	-1,10	-2,15	0,03200	NM_004497	GJB1
8168274	-1,12	-2,17	0,19756	-0,99	-1,99	0,05619	NM_000166	---
8095185	-1,12	-2,17	0,04472	-1,38	-2,59	0,01350	---	BHLHA9
8003633	-1,12	-2,17	0,04366	-2,36	-5,15	0,01273	NM_001164405	CC2D1A
8026217	-1,12	-2,17	0,00310	0,17	1,12	0,62209	NM_017721	OMP
7942715	-1,12	-2,17	0,00258	-0,27	-1,20	0,25286	NM_006189	---
8094391	-1,12	-2,17	0,19936	-0,64	-1,56	0,02138	---	---
7931214	-1,12	-2,17	0,12601	-2,01	-4,04	0,00009	---	NUP210L
7920426	-1,12	-2,17	0,02738	-1,39	-2,62	0,00615	NM_207308	NLRP13

8039557	-1,12	-2,17	0,01302	-1,09	-2,13	0,00282	NM_176810	---
8030197	-1,12	-2,17	0,02552	-1,17	-2,24	0,01994	---	OR2T33
7925741	-1,12	-2,17	0,27550	-2,82	-7,04	0,00155	NM_001004695	HLA-DQA1
8118556	-1,12	-2,17	0,09922	-0,51	-1,42	0,12287	NM_002122	ANKRD26P1
8001113	-1,12	-2,17	0,01389	-1,65	-3,13	0,02355	NR_026556	OR2J2
8179005	-1,12	-2,17	0,10760	-1,79	-3,47	0,00216	NM_030905	HSD17B1
8007263	-1,12	-2,17	0,15121	-1,50	-2,84	0,00393	NM_000413	---
7961998	-1,12	-2,17	0,01346	-2,80	-6,99	0,00355	---	---
8011678	-1,12	-2,17	0,04299	-0,68	-1,61	0,12057	---	RAB40C
7991917	-1,12	-2,17	0,10905	0,49	1,40	0,45004	NM_021168	T
8130703	-1,12	-2,17	0,01963	-1,58	-2,99	0,00164	NM_003181	---
8109503	-1,12	-2,18	0,01618	-1,41	-2,66	0,00511	---	KIF5A
7956522	-1,12	-2,18	0,02661	-1,08	-2,11	0,08308	AF063608	TAS2R41
8136934	-1,12	-2,18	0,11224	-1,08	-2,11	0,00859	NM_176883	SLC17A1
8124337	-1,12	-2,18	0,00313	-1,61	-3,06	0,00024	NM_005074	BHLHE22
8146645	-1,12	-2,18	0,04100	-0,80	-1,74	0,06319	NM_152414	SOX2
8084165	-1,12	-2,18	0,06633	-1,11	-2,16	0,00942	NM_003106	---
8027884	-1,12	-2,18	0,25349	-2,58	-5,97	0,00070	---	TINAG
8120315	-1,12	-2,18	0,03003	-0,75	-1,68	0,00396	NM_014464	KANK4
7916654	-1,12	-2,18	0,08716	-0,91	-1,88	0,04653	NM_181712	MKRN3
7981911	-1,12	-2,18	0,05625	-1,26	-2,40	0,06257	NM_005664	KATNB1
7996137	-1,12	-2,18	0,03385	-0,03	-1,02	0,95877	NM_005886	WFDC10A
8063011	-1,12	-2,18	0,05471	-0,72	-1,65	0,07045	NM_080753	MIR124-1
8149277	-1,12	-2,18	0,14056	-1,15	-2,22	0,00018	NR_029668	MAB21L1
7970949	-1,12	-2,18	0,00950	-2,06	-4,17	0,00263	NM_005584	---
8092185	-1,12	-2,18	0,10487	-0,99	-1,99	0,01525	---	---
8146894	-1,12	-2,18	0,01341	-1,00	-2,00	0,01488	---	LOC100293539
8145795	-1,12	-2,18	0,02754	-0,08	-1,06	0,94482	XM_002346094	ZNF99
8035836	-1,12	-2,18	0,07195	-1,40	-2,64	0,01893	BC021822	HBG1
7937990	-1,12	-2,18	0,49856	-2,03	-4,08	0,03254	NM_000559	HBG2
7937993	-1,12	-2,18	0,49856	-2,03	-4,08	0,03254	NM_000184	---
8127993	-1,12	-2,18	0,00083	-1,46	-2,75	0,00011	---	---
7928216	-1,12	-2,18	0,26268	-1,63	-3,09	0,03487	---	OR8K1
7939979	-1,12	-2,18	0,00651	-1,12	-2,18	0,00449	NM_001002907	FUT1
8038192	-1,12	-2,18	0,03306	0,32	1,25	0,11776	NM_000148	LDLRAD1
7916364	-1,12	-2,18	0,05838	-0,68	-1,60	0,00216	NM_001010978	CRCT1
7905486	-1,12	-2,18	0,01979	-1,21	-2,32	0,03230	NM_019060	---
8164153	-1,12	-2,18	0,12261	-1,16	-2,24	0,05055	---	PABPC1L2A
8173596	-1,12	-2,18	0,03081	-0,65	-1,57	0,01780	NM_001012977	---
8172191	-1,12	-2,18	0,02042	-0,69	-1,62	0,03649	---	---
7930680	-1,12	-2,18	0,01157	-1,18	-2,26	0,00291	---	REXO1L2P
8151603	-1,12	-2,18	0,54135	-5,99	-63,35	0,00001	NR_003594	REXO1L2P
8151607	-1,12	-2,18	0,54135	-5,99	-63,35	0,00001	NR_003594	REXO1L2P
8151623	-1,12	-2,18	0,54135	-5,99	-63,35	0,00001	NR_003594	KRTAP9-4
8007134	-1,12	-2,18	0,24516	-1,30	-2,46	0,00117	NM_033191	GOLGA6A
7985034	-1,12	-2,18	0,23498	0,01	1,01	0,99049	NM_001038640	C21orf93
8070896	-1,12	-2,18	0,00646	-1,02	-2,03	0,11684	AF427488	---
8043185	-1,12	-2,18	0,12993	-1,17	-2,26	0,02930	---	---
8118216	-1,12	-2,18	0,12143	-1,21	-2,31	0,02274	---	SNORD115-34
7982074	-1,12	-2,18	0,01377	-1,21	-2,32	0,01382	NR_003349	---
7903117	-1,12	-2,18	0,04973	-0,53	-1,44	0,33507	---	REXO1L2P
8151619	-1,12	-2,18	0,53767	-6,05	-66,32	0,00001	NR_003594	PTPN5
7947076	-1,12	-2,18	0,26172	-1,35	-2,55	0,00006	NM_006906	GPR78
8094093	-1,12	-2,18	0,05801	-1,30	-2,47	0,05555	NM_080819	FOXD1
8112592	-1,12	-2,18	0,00980	0,56	1,48	0,11660	NM_004472	PRAMEF5
7912567	-1,12	-2,18	0,15856	-1,02	-2,03	0,00557	NM_001013407	IRX3
8001449	-1,12	-2,18	0,12970	-1,22	-2,34	0,06526	NM_024336	---
8147479	-1,12	-2,18	0,04237	-1,29	-2,45	0,00022	---	---
7962137	-1,12	-2,18	0,01394	-1,25	-2,38	0,00834	---	IER5L
8164554	-1,12	-2,18	0,12166	-1,50	-2,83	0,01093	NM_203434	LACRT
7963837	-1,12	-2,18	0,02431	-0,89	-1,86	0,01278	NM_033277	---
8016866	-1,12	-2,18	0,00410	-1,14	-2,20	0,00068	---	---
8176663	-1,13	-2,18	0,05089	-1,97	-3,91	0,00003	---	---
8177176	-1,13	-2,18	0,05089	-1,97	-3,91	0,00003	---	PDCD11
7936096	-1,13	-2,18	0,08107	-0,91	-1,88	0,11269	NM_014976	GRIK3
7914974	-1,13	-2,18	0,02281	-1,35	-2,55	0,00250	NM_000831	KIF18B
8016147	-1,13	-2,18	0,12945	0,74	1,67	0,06901	NM_001080443	MUC12
8135031	-1,13	-2,18	0,56012	-3,29	-9,80	0,00011	NM_001164462	---
7992136	-1,13	-2,18	0,07290	-1,71	-3,27	0,00011	---	CORO1A
7994769	-1,13	-2,18	0,00589	-0,75	-1,68	0,12359	NM_007074	---
7904417	-1,13	-2,18	0,00117	-0,89	-1,86	0,01366	---	LGALS14
8028750	-1,13	-2,18	0,12658	-1,09	-2,13	0,02365	NM_203471	MYBPHL
7918313	-1,13	-2,18	0,00251	-1,55	-2,93	0,00002	NM_001010985	IDO2

8146105	-1,13	-2,18	0,09129	-0,96	-1,95	0,07271	NM_194294	SSC5D
8031499	-1,13	-2,18	0,08264	-2,03	-4,08	0,00038	NM_001144950	CLRN2
8094253	-1,13	-2,18	0,00497	-1,28	-2,44	0,04897	NM_001079827	LOC389791
8158164	-1,13	-2,18	0,11166	-1,11	-2,16	0,00205	NR_024425	GNG7
8032509	-1,13	-2,18	0,01947	1,47	2,77	0,00047	NM_052847	TTLL13
7985981	-1,13	-2,18	0,13730	-0,33	-1,26	0,15847	ENST00000438251	C3orf77
8079167	-1,13	-2,18	0,16081	-0,41	-1,33	0,12047	NM_001145030	---
8087780	-1,13	-2,18	0,02466	-1,05	-2,08	0,10481	---	TNFRSF17
7999547	-1,13	-2,18	0,10528	-0,96	-1,94	0,06103	NM_001192	04. Mrz
8058849	-1,13	-2,18	0,08679	-2,02	-4,05	0,00278	NM_020814	LST-3TM12
7954344	-1,13	-2,18	0,02531	-0,14	-1,10	0,70197	NM_001009562	---
7932790	-1,13	-2,18	0,09166	-0,87	-1,83	0,15506	---	TTC36
7944271	-1,13	-2,18	0,03382	-0,80	-1,74	0,01335	NM_001080441	REXO1L2P
8151627	-1,13	-2,18	0,54523	-6,21	-74,18	0,00001	NR_003594	SAMD1
8034754	-1,13	-2,18	0,06133	-0,97	-1,96	0,14589	NM_138352	---
8174933	-1,13	-2,18	0,02165	-1,81	-3,50	0,00517	---	EPHA8
7898773	-1,13	-2,19	0,01045	-1,02	-2,03	0,00316	NM_020526	---
8107940	-1,13	-2,19	0,21120	-0,67	-1,59	0,23262	---	SCN3B
7952350	-1,13	-2,19	0,03344	-1,83	-3,57	0,00176	NM_018400	KCNG4
8003149	-1,13	-2,19	0,04037	-1,25	-2,37	0,03076	NM_172347	KIAA1644
8076668	-1,13	-2,19	0,02179	-0,58	-1,49	0,33429	BC104183	DLEU2
7971653	-1,13	-2,19	0,07844	0,18	1,13	0,66688	NR_002612	PDYN
8064495	-1,13	-2,19	0,06370	-1,50	-2,83	0,00156	NM_024411	LOC100132686
7943890	-1,13	-2,19	0,00937	-0,70	-1,63	0,04723	ENST00000416328	GPR119
8175098	-1,13	-2,19	0,18352	-2,13	-4,36	0,00220	NM_178471	---
8119241	-1,13	-2,19	0,27611	-1,07	-2,09	0,12200	---	HBG1
7946048	-1,13	-2,19	0,01969	-1,27	-2,41	0,08390	NM_000559	HBG1
7946054	-1,13	-2,19	0,01969	-1,27	-2,41	0,08390	NM_000559	---
8127656	-1,13	-2,19	0,00549	-1,02	-2,03	0,01848	---	SNORA36C
8052826	-1,13	-2,19	0,02323	-1,49	-2,81	0,01799	NR_003705	---
8113489	-1,13	-2,19	0,04032	-2,28	-4,85	0,00755	---	HIST1H4G
8124433	-1,13	-2,19	0,05279	-1,09	-2,13	0,02939	NM_003547	CSAD
7963590	-1,13	-2,19	0,02127	-0,63	-1,54	0,09177	NM_015989	PROK1
7903884	-1,13	-2,19	0,09687	-1,72	-3,30	0,00025	NM_032414	FLJ44385
8073939	-1,13	-2,19	0,00369	-1,21	-2,32	0,03272	AK126356	---
8064900	-1,13	-2,19	0,04260	-0,76	-1,69	0,01279	---	LILRA3
8039226	-1,13	-2,19	0,23667	-2,09	-4,25	0,01469	NM_006865	MAGEA12
8175724	-1,13	-2,19	0,00311	-1,24	-2,36	0,04289	NM_001166386	LOC284379
8039086	-1,13	-2,19	0,12113	-0,96	-1,95	0,01592	NR_002938	C8orf85
8148000	-1,13	-2,19	0,12378	-1,19	-2,28	0,03885	NM_001025357	---
7978329	-1,13	-2,19	0,00652	-0,48	-1,39	0,00382	---	OVGP1
7918504	-1,13	-2,19	0,05625	-0,62	-1,54	0,03291	NM_002557	UNKL
7998466	-1,13	-2,19	0,07377	-1,30	-2,47	0,00010	NM_001037125	---
8022104	-1,13	-2,19	0,05565	-0,42	-1,34	0,02046	---	LRRC70
8105579	-1,13	-2,19	0,00382	-1,07	-2,10	0,02036	NM_181506	---
7905462	-1,13	-2,19	0,04304	-0,96	-1,94	0,01651	---	PMCHL1
8104627	-1,13	-2,19	0,45248	-0,86	-1,82	0,04616	NR_003921	MC4R
8023593	-1,13	-2,19	0,02281	-1,50	-2,82	0,00518	NM_005912	METTL7B
7956009	-1,13	-2,19	0,02461	1,64	3,12	0,00988	NM_152637	C1orf229
7925687	-1,13	-2,19	0,18941	-0,79	-1,73	0,02640	NM_207401	SERPINA2
7981065	-1,13	-2,19	0,08471	-1,27	-2,41	0,01759	ENST00000238576	SFRP2
8103254	-1,13	-2,19	0,04806	-1,55	-2,92	0,00007	NM_003013	---
7957956	-1,13	-2,19	0,13046	-1,75	-3,35	0,00001	---	C6orf27
8125072	-1,13	-2,19	0,00418	-0,46	-1,38	0,27935	NM_025258	C14orf80
7977418	-1,13	-2,19	0,02935	-0,56	-1,48	0,33885	NM_001134875	SYCE2
8034583	-1,13	-2,19	0,03691	-0,64	-1,56	0,17876	NM_001105578	MYO15A
8005328	-1,13	-2,19	0,00832	-1,23	-2,34	0,01044	NM_016239	RNF183
8163485	-1,13	-2,19	0,01145	-1,95	-3,85	0,01494	NM_145051	WBSCR27
8140144	-1,13	-2,19	0,00919	-0,23	-1,17	0,38749	NM_152559	SPRR2B
7920201	-1,13	-2,19	0,08593	-2,22	-4,66	0,00325	NM_001017418	FOXD4L3
8155661	-1,13	-2,19	0,18483	-1,21	-2,32	0,02506	NM_199135	---
8155323	-1,13	-2,19	0,07384	-1,45	-2,72	0,00960	---	PIGT
8066528	-1,13	-2,19	0,02827	-0,83	-1,78	0,02991	ENST00000425680	LOC100128185
8039975	-1,13	-2,19	0,00650	-1,00	-2,00	0,00456	AF274944	FAM22A
7928645	-1,13	-2,19	0,10719	-0,92	-1,89	0,03914	NM_001099338	CNTN3
8088866	-1,13	-2,19	0,10586	-1,44	-2,71	0,00059	NM_020872	---
7952766	-1,13	-2,19	0,10455	-1,10	-2,14	0,04874	---	KRT6A
7963421	-1,13	-2,19	0,21433	-1,27	-2,42	0,01490	NM_005554	PRMT8
7953181	-1,13	-2,19	0,00874	-1,57	-2,98	0,04376	NM_019854	GLB1L3
7945296	-1,13	-2,19	0,00072	-0,29	-1,22	0,18358	NM_001080407	CCR6
8123364	-1,13	-2,19	0,19510	-1,18	-2,27	0,00071	NM_031409	OR7D2
8025470	-1,13	-2,19	0,04229	-1,53	-2,90	0,00092	NM_175883	HEATR7B1
8049375	-1,13	-2,19	0,03313	-1,50	-2,83	0,00088	ENST00000389758	FCAMR

7923942	-1,13	-2,19	0,19213	-1,39	-2,62	0,00234	NM_001170631	KCNS1
8066475	-1,13	-2,19	0,01736	-1,51	-2,86	0,00545	NM_002251	ORC1L
7916167	-1,13	-2,19	0,01843	0,22	1,16	0,46101	NM_004153	ZNF746
8143725	-1,13	-2,19	0,08342	-0,76	-1,69	0,13423	NM_001163474	PCDH11X
8168657	-1,13	-2,19	0,02119	-1,27	-2,40	0,00108	NM_032967	---
7969975	-1,13	-2,19	0,23021	-1,36	-2,56	0,02075	---	UPP1
8132725	-1,13	-2,19	0,06042	0,57	1,48	0,02506	NM_003364	---
7966227	-1,13	-2,19	0,05532	-0,88	-1,84	0,00094	---	---
8051202	-1,13	-2,19	0,01795	-1,79	-3,47	0,00169	---	FOXJ1
8018646	-1,13	-2,19	0,00648	-0,71	-1,64	0,00789	NM_001454	PDE11A
8057004	-1,13	-2,19	0,00825	-0,24	-1,18	0,54469	NM_001077197	MCF2L
7970194	-1,13	-2,19	0,04766	-0,70	-1,63	0,04419	NM_001112732	WDR88
8027543	-1,13	-2,19	0,01575	-0,55	-1,46	0,28490	NM_173479	GJD4
7927028	-1,13	-2,19	0,07323	-2,28	-4,87	0,00449	NM_153368	ALPK3
7985620	-1,13	-2,20	0,00922	-1,19	-2,28	0,00243	NM_020778	ZSCAN22
8031908	-1,13	-2,20	0,17643	-0,01	-1,01	0,98281	NM_181846	L1TD1
7901860	-1,13	-2,20	0,00601	-1,35	-2,55	0,00077	NM_001164835	---
8084927	-1,13	-2,20	0,00215	-0,75	-1,68	0,02426	---	KRTAP10-4
8069142	-1,13	-2,20	0,40666	-2,99	-7,97	0,00048	NM_198687	---
8176737	-1,13	-2,20	0,00612	-1,47	-2,78	0,03661	---	PAX5
8161211	-1,14	-2,20	0,04402	-1,66	-3,16	0,02663	NM_016734	C16orf75
7993248	-1,14	-2,20	0,05244	-0,57	-1,49	0,25942	NM_152308	INPP5D
8049246	-1,14	-2,20	0,00830	-0,44	-1,36	0,21083	NM_001017915	SNORA5C
8139484	-1,14	-2,20	0,19872	-1,35	-2,54	0,01919	NR_002991	---
7999384	-1,14	-2,20	0,10704	-1,92	-3,79	0,00352	---	APCDD1L
8067270	-1,14	-2,20	0,04400	-1,88	-3,69	0,00026	NM_153360	C1orf94
7899911	-1,14	-2,20	0,12477	-1,18	-2,26	0,00676	NM_001134734	TP53I13
8006052	-1,14	-2,20	0,08468	-0,75	-1,68	0,20416	NM_138349	UNC80
8047965	-1,14	-2,20	0,00168	-1,10	-2,15	0,00504	NM_032504	---
8174195	-1,14	-2,20	0,05456	-1,20	-2,30	0,04458	---	DSCR8
8068570	-1,14	-2,20	0,13799	-1,61	-3,05	0,06425	NR_026839	---
8098421	-1,14	-2,20	0,19473	-1,86	-3,63	0,03579	---	OR4D10
7940182	-1,14	-2,20	0,25359	-4,31	-19,89	0,00010	NM_001004705	---
8043480	-1,14	-2,20	0,37054	-3,10	-8,59	0,00050	---	FER1L4
8065948	-1,14	-2,20	0,00277	-0,58	-1,50	0,23386	NR_024377	EPN3
8008372	-1,14	-2,20	0,06518	-1,64	-3,12	0,02867	NM_017957	TTLL10
7896865	-1,14	-2,20	0,08305	-1,70	-3,25	0,00008	NM_153254	FOLR2
7942372	-1,14	-2,20	0,02497	-1,57	-2,97	0,00013	NM_000803	LOC158696
8175442	-1,14	-2,20	0,06295	-1,87	-3,67	0,02676	NR_026935	---
8047963	-1,14	-2,20	0,20454	-1,26	-2,40	0,00052	---	WIPF3
8132036	-1,14	-2,20	0,01922	0,77	1,71	0,24859	NM_001080529	RAB11FIP3
7991860	-1,14	-2,20	0,00416	-0,57	-1,48	0,20157	NM_014700	---
8117286	-1,14	-2,20	0,10103	-0,99	-1,99	0,07667	---	CPLX3
7984914	-1,14	-2,20	0,01683	-0,45	-1,37	0,13805	NM_001030005	---
8172084	-1,14	-2,20	0,07401	-1,11	-2,17	0,00499	---	---
8141765	-1,14	-2,20	0,07214	-1,23	-2,35	0,00423	---	---
8136213	-1,14	-2,20	0,11929	-1,45	-2,73	0,00259	---	SLC8A3
7979888	-1,14	-2,20	0,00767	-2,17	-4,50	0,00024	NM_033262	GAB1
8102982	-1,14	-2,20	0,11441	-1,22	-2,33	0,01821	BC064848	NKG7
8038809	-1,14	-2,20	0,15159	-1,79	-3,47	0,05323	NM_005601	SPHK1
8010061	-1,14	-2,20	0,01017	-0,18	-1,14	0,63694	NM_182965	RGAG1
8169378	-1,14	-2,20	0,02297	-1,13	-2,19	0,02061	NM_020769	CDH16
8001898	-1,14	-2,20	0,01360	-0,96	-1,94	0,02333	NM_004062	---
7977438	-1,14	-2,20	0,14357	-0,73	-1,65	0,19230	---	NUDT17
7919217	-1,14	-2,20	0,02376	0,86	1,81	0,09308	NM_001012758	SLC3A1
8041727	-1,14	-2,20	0,03402	-0,95	-1,93	0,00247	NM_000341	NR2E3
7984606	-1,14	-2,20	0,01837	-0,49	-1,40	0,14345	NM_014249	NEUROD1
8057480	-1,14	-2,20	0,00779	-1,45	-2,73	0,00374	NM_002500	RHPN1
8148621	-1,14	-2,20	0,10470	0,40	1,32	0,51323	NM_052924	RNF113B
7972438	-1,14	-2,20	0,06081	-1,55	-2,93	0,03831	NM_178861	NDOR1
8159616	-1,14	-2,20	0,02079	0,68	1,60	0,18486	NM_001144026	LCE2A
7905507	-1,14	-2,20	0,19197	-2,74	-6,69	0,00007	NM_178428	GPR4
8037614	-1,14	-2,20	0,03008	-0,57	-1,48	0,04015	NM_005282	---
7902474	-1,14	-2,20	0,25284	-1,12	-2,17	0,00013	---	C6orf123
8130811	-1,14	-2,20	0,06410	-1,38	-2,60	0,01230	AB016901	C6orf123
8130982	-1,14	-2,20	0,06410	-1,38	-2,60	0,01230	AB016901	OR2T4
7911273	-1,14	-2,20	0,01854	-1,12	-2,18	0,00582	NM_001004696	OR5K3
8081208	-1,14	-2,20	0,05137	-1,21	-2,31	0,03342	NM_001005516	GPX2
7979658	-1,14	-2,20	0,02994	0,53	1,44	0,35100	NM_002083	PNO1
8052719	-1,14	-2,20	0,03273	-1,82	-3,54	0,04738	NM_020143	---
7960898	-1,14	-2,20	0,37769	-3,23	-9,41	0,00002	---	ACP5
8034304	-1,14	-2,20	0,00135	-1,29	-2,45	0,01240	NM_001111035	C1orf192
7921862	-1,14	-2,20	0,03739	-1,28	-2,43	0,00173	NM_001013625	NFKBIL2

8153790	-1,14	-2,20	0,07171	-1,02	-2,02	0,00254	NM_013432	APOA1
8077185	-1,14	-2,20	0,05367	-1,50	-2,82	0,00025	NM_000039	RBMY1B
8176753	-1,14	-2,20	0,03040	-1,25	-2,37	0,02380	NM_001006121	RBMY1B
8177290	-1,14	-2,20	0,03040	-1,25	-2,37	0,02380	NM_001006121	---
7922125	-1,14	-2,20	0,06782	-1,29	-2,45	0,03179	---	SNORD3A
8005547	-1,14	-2,20	0,27591	-1,05	-2,06	0,02146	NR_006880	SNORD3A
8005553	-1,14	-2,20	0,27591	-1,05	-2,06	0,02146	NR_006880	SNORD3A
8013323	-1,14	-2,20	0,27591	-1,05	-2,06	0,02146	NR_006880	SNORD3A
8013325	-1,14	-2,20	0,27591	-1,05	-2,06	0,02146	NR_006880	SNORD3A
8013329	-1,14	-2,20	0,27591	-1,05	-2,06	0,02146	NR_006880	C6orf201
8116696	-1,14	-2,20	0,00219	-1,49	-2,81	0,00200	NM_001085401	---
8086797	-1,14	-2,20	0,11285	-1,50	-2,82	0,03012	---	FOXL2
8091032	-1,14	-2,20	0,03800	-1,21	-2,31	0,01318	NM_023067	---
8140857	-1,14	-2,20	0,01818	-1,62	-3,08	0,00186	---	---
8113699	-1,14	-2,20	0,11231	-1,37	-2,58	0,00049	---	PDGFB
8076195	-1,14	-2,20	0,10388	0,46	1,38	0,51953	NM_002608	FRMD3
8161964	-1,14	-2,20	0,02547	-1,37	-2,59	0,01986	NM_174938	---
8095299	-1,14	-2,20	0,40133	-1,68	-3,20	0,03189	---	NKPD1
8037502	-1,14	-2,20	0,03853	-1,50	-2,83	0,02810	ENST00000317951	BEAN
7996281	-1,14	-2,20	0,05496	1,40	2,64	0,00253	NM_001178020	C6
8111864	-1,14	-2,20	0,01356	-1,41	-2,65	0,06199	NM_001115131	LIPN
7928999	-1,14	-2,20	0,10971	-0,91	-1,87	0,07427	NM_001102469	FLJ35220
8010499	-1,14	-2,20	0,01197	-0,78	-1,71	0,18993	NM_173627	SH3RF2
8108912	-1,14	-2,20	0,00170	-0,05	-1,04	0,81511	NM_152550	SNORD115-41
7982088	-1,14	-2,20	0,04202	-2,06	-4,16	0,00951	NR_003356	AIM2
7921434	-1,14	-2,20	0,07409	-1,41	-2,66	0,00702	NM_004833	PRKACG
8161615	-1,14	-2,20	0,00333	-1,82	-3,54	0,00307	NM_002732	MAF
8002969	-1,14	-2,20	0,01483	-1,55	-2,93	0,00120	NM_001031804	---
8113467	-1,14	-2,20	0,04404	-1,13	-2,18	0,03424	---	C11orf45
7952626	-1,14	-2,20	0,00076	0,06	1,05	0,68764	NM_145013	KIAA0664P3
7995242	-1,14	-2,20	0,02440	0,34	1,27	0,43443	BC021555	---
8024882	-1,14	-2,20	0,03338	-1,25	-2,37	0,00384	---	---
8085056	-1,14	-2,21	0,06548	-1,48	-2,80	0,03460	---	SNORD116-12
7981972	-1,14	-2,21	0,00684	-0,81	-1,75	0,00014	NR_003327	---
8065082	-1,14	-2,21	0,06584	-1,15	-2,21	0,01686	---	---
7950292	-1,14	-2,21	0,00610	-1,27	-2,42	0,00045	---	SCNN1A
7960529	-1,14	-2,21	0,03264	2,00	4,00	0,00217	NM_001038	OR2J2
8117716	-1,14	-2,21	0,09544	-1,94	-3,83	0,00127	NM_030905	OR2J2
8177696	-1,14	-2,21	0,09544	-1,94	-3,83	0,00127	NM_030905	RAVER1
8033969	-1,14	-2,21	0,05784	-0,81	-1,75	0,15101	NM_133452	GRK4
8093665	-1,14	-2,21	0,00004	-0,69	-1,61	0,01308	NM_182982	CBLN4
8067157	-1,14	-2,21	0,05771	-1,52	-2,87	0,00001	NM_080617	AQP12A
8060126	-1,14	-2,21	0,10983	-1,83	-3,57	0,00090	NM_198998	FAM92A3
8098465	-1,14	-2,21	0,00782	-1,08	-2,11	0,00661	NR_003612	OR7E91P
8042568	-1,14	-2,21	0,10127	-1,73	-3,33	0,01462	NR_002185	MYO1H
7958524	-1,14	-2,21	0,00277	-0,79	-1,73	0,13276	NM_001101421	---
8078225	-1,14	-2,21	0,16089	-0,63	-1,55	0,05399	---	STRA8
8136391	-1,14	-2,21	0,02538	-1,15	-2,22	0,00081	NM_182489	ONECUT1
7988987	-1,14	-2,21	0,06294	-0,75	-1,68	0,24660	NM_004498	DHDH
8030148	-1,14	-2,21	0,00343	-0,10	-1,07	0,71992	NM_014475	FLJ46365
8146388	-1,14	-2,21	0,05683	-0,91	-1,88	0,04663	AK128232	---
8022020	-1,14	-2,21	0,00303	-1,53	-2,89	0,01437	---	---
8079149	-1,14	-2,21	0,02738	-0,98	-1,97	0,04852	---	ZMYND15
8003926	-1,14	-2,21	0,02935	-1,42	-2,67	0,01611	NM_001136046	SLAMF7
7906613	-1,14	-2,21	0,00223	0,37	1,29	0,12949	NM_021181	AKAP3
7960427	-1,14	-2,21	0,00041	-0,36	-1,28	0,26394	NM_006422	---
7933976	-1,14	-2,21	0,21545	-1,84	-3,58	0,03005	---	---
8062555	-1,14	-2,21	0,10625	-1,01	-2,01	0,06974	---	MS4A15
7940333	-1,14	-2,21	0,03414	-0,40	-1,32	0,34306	NM_001098835	BTBD8
7902965	-1,14	-2,21	0,08394	-0,47	-1,38	0,06744	NM_183242	OR4F17
7896740	-1,14	-2,21	0,19631	-1,43	-2,69	0,00520	NM_001005240	OR4F17
7991663	-1,14	-2,21	0,19631	-1,43	-2,69	0,00520	NM_001005240	OR4F17
8023937	-1,14	-2,21	0,19631	-1,43	-2,69	0,00520	NM_001005240	SLAMF1
7921652	-1,14	-2,21	0,03551	-1,53	-2,90	0,00056	NM_003037	---
8046199	-1,14	-2,21	0,04883	-0,82	-1,77	0,00768	---	---
7971122	-1,14	-2,21	0,00339	-2,26	-4,78	0,00099	---	---
8002342	-1,14	-2,21	0,05906	-2,10	-4,29	0,00001	---	RAET1E
8130151	-1,14	-2,21	0,01759	-0,80	-1,74	0,00303	NM_139165	DCAF8L1
8171885	-1,14	-2,21	0,00378	-0,94	-1,92	0,00152	NM_001017930	OR51A2
7946017	-1,14	-2,21	0,02699	-1,04	-2,05	0,00503	NM_001004748	---
8088772	-1,14	-2,21	0,01762	-0,34	-1,26	0,44782	---	---
7914994	-1,14	-2,21	0,03348	-1,08	-2,11	0,02843	---	LRRC4
8142765	-1,14	-2,21	0,01775	-0,95	-1,93	0,00700	NM_022143	CYP4A22

7901293	-1,14	-2,21	0,27226	-1,49	-2,80	0,02575	NM_001010969	KCTD15
8027584	-1,14	-2,21	0,11583	-0,39	-1,31	0,54554	NM_024076	PTPN3
8163149	-1,14	-2,21	0,00470	3,39	10,49	0,00003	NM_002829	SULT1C3
8044204	-1,14	-2,21	0,04094	-1,19	-2,28	0,01337	NM_001008743	SAMD10
8067792	-1,14	-2,21	0,10099	0,63	1,55	0,06904	NM_080621	GRK1
7970325	-1,14	-2,21	0,00306	-1,10	-2,14	0,00958	NM_002929	HOXC9
7955865	-1,14	-2,21	0,00758	-0,87	-1,83	0,06688	NM_006897	LOC158572
8172654	-1,14	-2,21	0,06022	-1,31	-2,48	0,03305	NR_026742	OR56A1
7946119	-1,14	-2,21	0,01285	-0,94	-1,92	0,00808	NM_001001917	TKTL1
8170834	-1,14	-2,21	0,01301	-0,66	-1,58	0,01090	NM_012253	USP17
8094122	-1,14	-2,21	0,51681	-3,23	-9,38	0,00028	NM_001105662	OR52K1
7937942	-1,14	-2,21	0,16033	-1,58	-2,99	0,09363	NM_001005171	MTHFR
7897753	-1,15	-2,21	0,07183	-1,19	-2,29	0,02313	ENST00000376592	SPDYE8P
8133582	-1,15	-2,21	0,23452	-2,52	-5,74	0,00168	NR_003664	SPRR2D
7920196	-1,15	-2,21	0,00264	-1,27	-2,41	0,04699	NM_006945	FZD3
8145611	-1,15	-2,21	0,00528	3,37	10,31	0,00066	NM_017412	ZBTB7B
7905854	-1,15	-2,21	0,08461	-0,58	-1,49	0,05721	NM_015872	---
8063347	-1,15	-2,21	0,04615	-1,30	-2,46	0,04159	---	CETN1
8019828	-1,15	-2,21	0,11345	-1,14	-2,21	0,03970	NM_004066	MEOX2
8138353	-1,15	-2,21	0,01322	-1,99	-3,97	0,00043	NM_005924	DNTT
7929574	-1,15	-2,21	0,04040	-0,72	-1,65	0,08243	NM_004088	---
8166609	-1,15	-2,21	0,01378	-1,13	-2,19	0,00380	---	IFNA21
8160371	-1,15	-2,21	0,00753	-1,31	-2,48	0,04284	NM_002175	ACCN4
8048611	-1,15	-2,21	0,00248	-0,86	-1,82	0,01492	NM_018674	---
8083852	-1,15	-2,21	0,00731	-1,37	-2,58	0,00139	---	ARTN
7900878	-1,15	-2,21	0,01062	-0,84	-1,79	0,13393	NM_057091	VDAC2
7928524	-1,15	-2,21	0,04151	-1,21	-2,32	0,04274	NM_003375	ACADL
8058591	-1,15	-2,21	0,05546	-1,30	-2,46	0,01916	NM_001608	DACH1
7971950	-1,15	-2,21	0,00087	-0,76	-1,69	0,00009	NM_080759	GUCY2D
8004763	-1,15	-2,21	0,07811	-1,55	-2,92	0,01665	NM_000180	AVP
8064575	-1,15	-2,21	0,13342	-1,64	-3,11	0,00014	NM_000490	PRAMEF5
7898007	-1,15	-2,22	0,13792	-0,94	-1,91	0,00898	NM_001013407	PRAMEF5
7912575	-1,15	-2,22	0,13792	-0,94	-1,91	0,00898	NM_001013407	HS3ST6
7998634	-1,15	-2,22	0,11123	-1,47	-2,78	0,01714	NM_001009606	SYNPO
8109305	-1,15	-2,22	0,04235	-0,24	-1,18	0,52029	NM_007286	CSHL1
8017499	-1,15	-2,22	0,11166	-1,94	-3,85	0,00896	NM_022579	MTNR1B
7943098	-1,15	-2,22	0,05900	-1,20	-2,30	0,06135	NM_005959	---
7971484	-1,15	-2,22	0,01184	-1,70	-3,26	0,00342	---	MAP1LC3C
7925504	-1,15	-2,22	0,14956	-1,41	-2,66	0,00906	NM_001004343	NUDT8
7949857	-1,15	-2,22	0,01116	-0,11	-1,08	0,84861	NM_181843	FER1L5
8043639	-1,15	-2,22	0,02625	-1,06	-2,08	0,05383	NM_001077400	---
8090674	-1,15	-2,22	0,07547	-0,99	-1,99	0,00465	---	KCNA6
7953264	-1,15	-2,22	0,06502	-1,00	-2,01	0,06711	NM_002235	CD1E
7906355	-1,15	-2,22	0,03953	-2,36	-5,13	0,00819	NM_030893	FBXO17
8036676	-1,15	-2,22	0,00709	2,08	4,22	0,03652	NM_024907	FAM22G
8156620	-1,15	-2,22	0,02229	-1,47	-2,77	0,04562	NM_001170741	ANKS3
7999177	-1,15	-2,22	0,01838	-1,25	-2,38	0,04692	NM_133450	OR9G4
7948157	-1,15	-2,22	0,03343	-1,76	-3,38	0,01608	NM_001005284	---
8028789	-1,15	-2,22	0,00027	-0,86	-1,81	0,00767	---	ITGAD
7995161	-1,15	-2,22	0,06175	-1,01	-2,01	0,03027	NM_005353	---
8042513	-1,15	-2,22	0,20578	-1,74	-3,33	0,04557	---	SYT5
8039378	-1,15	-2,22	0,12143	-1,67	-3,18	0,01080	NM_003180	ARL14
8083743	-1,15	-2,22	0,01949	-0,43	-1,34	0,38765	NM_025047	PVALB
8075838	-1,15	-2,22	0,08241	-1,60	-3,04	0,00744	NM_002854	---
8076329	-1,15	-2,22	0,24833	-1,14	-2,20	0,03059	---	---
8129943	-1,15	-2,22	0,05780	-0,58	-1,50	0,05203	---	---
8160555	-1,15	-2,22	0,00638	-1,06	-2,08	0,00354	---	---
8119078	-1,15	-2,22	0,01641	-1,37	-2,58	0,00126	---	---
8067867	-1,15	-2,22	0,17496	-1,54	-2,90	0,00130	---	C1orf187
7897737	-1,15	-2,22	0,11567	-0,92	-1,89	0,12996	NM_198545	FKBP6
8140291	-1,15	-2,22	0,13868	-1,89	-3,70	0,00084	NM_003602	OR51B6
7937996	-1,15	-2,22	0,00688	-1,46	-2,76	0,01246	NM_001004750	---
7902685	-1,15	-2,22	0,01462	-1,12	-2,18	0,04259	---	TGIF2LY
8176397	-1,15	-2,22	0,04633	-1,58	-2,99	0,01558	NM_139214	---
8124920	-1,15	-2,22	0,08022	-2,51	-5,71	0,00611	---	LOC442293
8139015	-1,15	-2,22	0,00860	-0,55	-1,46	0,02766	NR_033246	OR2AT4
7950471	-1,15	-2,22	0,08646	-1,86	-3,63	0,00735	NM_001005285	LOC100128653
8137781	-1,15	-2,22	0,09903	-1,27	-2,41	0,03733	ENST00000443349	ADAT3
8024356	-1,15	-2,22	0,13024	-0,04	-1,02	0,94692	NM_138422	C15orf60
7984736	-1,15	-2,22	0,00731	-0,77	-1,70	0,10784	NM_001042367	PYROXD2
7935572	-1,15	-2,22	0,01128	-0,93	-1,91	0,03690	NM_032709	FCER1A
7906443	-1,15	-2,22	0,00211	-1,53	-2,89	0,00704	NM_002001	SRPK3
8170753	-1,15	-2,22	0,01914	-1,22	-2,33	0,00771	NM_014370	REG1P

8053337	-1,15	-2,22	0,05408	-1,36	-2,57	0,00447	NR_002714	ZNF818P
8030969	-1,15	-2,22	0,05236	-1,67	-3,19	0,00001	ENST00000344759	C2orf50
8040270	-1,15	-2,22	0,01416	-0,94	-1,91	0,01834	NM_182500	HOXD4
8046555	-1,15	-2,22	0,10688	-1,84	-3,58	0,01424	NM_014621	PTPRO
7954143	-1,15	-2,22	0,00321	-0,81	-1,75	0,01166	NM_030667	---
8147689	-1,15	-2,22	0,01641	-1,10	-2,15	0,13000	---	C14orf162
7979870	-1,15	-2,22	0,09908	-1,70	-3,26	0,01099	NR_024630	ADAM6 // ADAM6
7986629	-1,15	-2,22	0,03657	-2,00	-4,01	0,00445	NR_002224 // NR_002224	LMOD3
8088739	-1,15	-2,22	0,01171	-0,93	-1,91	0,02578	NM_198271	ALG1L
8090291	-1,15	-2,22	0,17952	2,20	4,60	0,00810	NM_001015050	GJB5
7899921	-1,15	-2,22	0,00257	-1,09	-2,13	0,06295	NM_005268	HIST1H3C
8117339	-1,15	-2,22	0,04932	-1,46	-2,75	0,00005	NM_003531	LCN15
8165298	-1,15	-2,22	0,00680	-1,87	-3,66	0,01166	NM_203347	DEF6
8118932	-1,15	-2,22	0,02244	-1,52	-2,88	0,00456	NM_022047	SLC25A42
8027128	-1,15	-2,22	0,02756	-0,70	-1,62	0,07292	NM_178526	WTIP
8027665	-1,15	-2,22	0,06377	-1,09	-2,13	0,06219	NM_001080436	---
7964577	-1,15	-2,22	0,04075	-0,78	-1,72	0,10831	---	SPC24
8034122	-1,15	-2,22	0,04885	-0,16	-1,12	0,67889	NM_182513	---
8097476	-1,15	-2,23	0,07665	-1,10	-2,14	0,00044	---	---
8067858	-1,15	-2,23	0,00194	-0,56	-1,47	0,13576	---	BTNL2
8125426	-1,15	-2,23	0,13317	-1,60	-3,04	0,00615	NM_019602	BTNL2
8178795	-1,15	-2,23	0,13317	-1,60	-3,04	0,00615	NM_019602	BTNL2
8179996	-1,15	-2,23	0,13317	-1,60	-3,04	0,00615	NM_019602	LOC100292301
8102781	-1,15	-2,23	0,08956	-0,68	-1,61	0,09128	ENST00000330122	TMPRSS11B
8100701	-1,15	-2,23	0,00792	-0,87	-1,82	0,00036	NM_182502	RPL21P44
8100378	-1,15	-2,23	0,04240	-0,79	-1,73	0,00763	NR_027153	FAM36A
7925561	-1,16	-2,23	0,07480	-0,18	-1,13	0,55991	NM_198076	OVOS2
7962139	-1,16	-2,23	0,00492	-0,29	-1,23	0,13221	ENST00000266524	---
8009491	-1,16	-2,23	0,02015	-1,57	-2,96	0,00902	---	NLRP8
8031586	-1,16	-2,23	0,01216	-1,30	-2,45	0,00181	NM_176811	EPYC
7965390	-1,16	-2,23	0,06111	-0,90	-1,87	0,00585	NM_004950	---
7969531	-1,16	-2,23	0,05352	-0,85	-1,80	0,03615	---	VWDE
8138277	-1,16	-2,23	0,10417	0,47	1,38	0,34748	NM_001135924	CTSW
7941444	-1,16	-2,23	0,06182	-1,16	-2,24	0,00504	NM_001335	---
8148309	-1,16	-2,23	0,07419	-1,11	-2,16	0,00831	---	LOC440419
8005777	-1,16	-2,23	0,00680	-1,01	-2,02	0,03220	BC112354	PTMS
7953409	-1,16	-2,23	0,04893	0,32	1,25	0,30647	NM_002824	CEACAM7
8037053	-1,16	-2,23	0,03091	-1,31	-2,48	0,03540	NM_006890	ADAMTSL5
8032249	-1,16	-2,23	0,12110	0,56	1,48	0,38282	NM_213604	---
7936492	-1,16	-2,23	0,05786	-1,71	-3,27	0,01981	---	NCRNA00174
8139873	-1,16	-2,23	0,01149	-0,44	-1,36	0,58072	NR_026873	---
8100599	-1,16	-2,23	0,01506	-2,24	-4,73	0,02365	---	C7orf63
8134051	-1,16	-2,23	0,00329	-1,19	-2,28	0,07086	NM_001039706	CSTT
8061396	-1,16	-2,23	0,04789	-1,15	-2,22	0,02583	NR_001279	---
8044731	-1,16	-2,23	0,02457	-1,60	-3,03	0,03897	---	---
8104723	-1,16	-2,23	0,01525	-1,48	-2,79	0,00244	---	LAPTM5
7914270	-1,16	-2,23	0,03877	-0,87	-1,83	0,03612	NM_006762	C19orf39
8025888	-1,16	-2,23	0,10345	-0,32	-1,25	0,42773	NM_175871	GUCA1A
8119515	-1,16	-2,23	0,03968	-1,10	-2,14	0,00271	NM_000409	UMODL1
8068740	-1,16	-2,23	0,00993	-0,14	-1,10	0,69234	NM_001004416	---
8089436	-1,16	-2,23	0,00608	-0,74	-1,68	0,05876	---	NDUFA4L2
7964388	-1,16	-2,23	0,02966	-1,29	-2,44	0,00237	NM_020142	FFAR3
8027856	-1,16	-2,23	0,00140	-1,24	-2,36	0,02915	NM_005304	WWC1
8109773	-1,16	-2,23	0,00749	2,74	6,69	0,00045	NM_001161661	FAM182A
8065527	-1,16	-2,23	0,24630	-2,19	-4,55	0,00011	NR_026713	CHD9
8001402	-1,16	-2,23	0,02314	-1,19	-2,28	0,04119	NM_025134	---
7923810	-1,16	-2,23	0,02196	-1,21	-2,32	0,00022	---	---
8025996	-1,16	-2,23	0,00191	-1,05	-2,07	0,03052	---	CHAF1A
8024843	-1,16	-2,23	0,01310	0,28	1,22	0,28292	NM_005483	---
8122405	-1,16	-2,23	0,00571	-1,01	-2,01	0,17803	---	LFNG
8131143	-1,16	-2,23	0,02390	-1,16	-2,24	0,00867	NM_001040167	LEMD2
8125788	-1,16	-2,23	0,16283	1,36	2,56	0,07111	NM_181336	PILRA
8134814	-1,16	-2,23	0,09550	-1,37	-2,58	0,06078	NM_013439	SSTR2
8009526	-1,16	-2,23	0,04313	-0,86	-1,81	0,00558	NM_001050	SPEF1
8064751	-1,16	-2,23	0,07135	-1,93	-3,82	0,00522	NM_015417	IFRD2
8079988	-1,16	-2,23	0,01530	-1,81	-3,52	0,00036	NM_006764	SCGB1A1
7940654	-1,16	-2,23	0,02866	-1,28	-2,43	0,04024	NM_003357	LOC399900
7940598	-1,16	-2,23	0,07407	-0,96	-1,95	0,02155	AK093779	CCDC27
7897196	-1,16	-2,23	0,02608	-0,77	-1,70	0,00259	NM_152492	TMEM68
8146480	-1,16	-2,23	0,03640	-1,67	-3,17	0,02742	BC043417	---
7916926	-1,16	-2,24	0,01431	-0,88	-1,84	0,00002	---	---
7902265	-1,16	-2,24	0,03335	-1,13	-2,19	0,03303	---	LGALS16
8028742	-1,16	-2,24	0,05771	-1,43	-2,70	0,00808	FJ613352	GAFA2

8003056	-1,16	-2,24	0,01834	-1,63	-3,09	0,00160	AF220234	TCP10L	
8069991	-1,16	-2,24	0,07681	-1,21	-2,31	0,05543	NM_144659		ANKRD53
8042558	-1,16	-2,24	0,02695	-0,83	-1,78	0,00966	NM_024933		C10orf41
7928531	-1,16	-2,24	0,07911	-2,59	-6,02	0,00487	NR_024421		FBP2
8162492	-1,16	-2,24	0,01206	-1,90	-3,73	0,00427	NM_003837		PLAC4
8070458	-1,16	-2,24	0,01359	-2,06	-4,16	0,00507	NM_182832		HES6
8059989	-1,16	-2,24	0,06394	-1,47	-2,78	0,00456	NM_018645		C22orf27
8072400	-1,16	-2,24	0,00680	-1,00	-2,01	0,09021	NR_026920		---
8143294	-1,16	-2,24	0,01158	-1,57	-2,97	0,02809	---	---	
7925753	-1,16	-2,24	0,10576	-1,44	-2,71	0,02240	---	---	
7932635	-1,16	-2,24	0,08742	-1,21	-2,31	0,02724	---	---	
7934269	-1,16	-2,24	0,11203	-1,95	-3,86	0,04395	---	ATP6V0E2	
8143759	-1,16	-2,24	0,00085	-0,46	-1,38	0,13952	NM_145230		DCAF4L2
8151679	-1,16	-2,24	0,04591	-1,59	-3,02	0,00099	NM_152418		TSSK4
7973652	-1,16	-2,24	0,02033	-2,05	-4,14	0,00877	NM_174944		HECW1
8132465	-1,16	-2,24	0,05118	-0,10	-1,08	0,74160	NM_015052		---
8156844	-1,16	-2,24	0,02356	-1,07	-2,10	0,00744	---	---	
8121136	-1,16	-2,24	0,05180	-1,27	-2,42	0,00080	---	KBTBD5	
8079107	-1,16	-2,24	0,07739	-1,57	-2,96	0,00120	NM_152393		---
7975410	-1,16	-2,24	0,03482	-1,76	-3,39	0,05430	---	ZNF862	
8137202	-1,16	-2,24	0,00998	-0,50	-1,41	0,04436	NM_001099220		OR51D1
7937950	-1,16	-2,24	0,09565	-1,46	-2,75	0,00025	NM_001004751		CD300E
8018189	-1,16	-2,24	0,13697	-1,90	-3,72	0,00004	NM_181449		---
8163614	-1,16	-2,24	0,00448	-1,41	-2,67	0,01357	---	APBA3	
8032670	-1,16	-2,24	0,05245	-0,16	-1,12	0,74038	NM_004886		---
7934706	-1,16	-2,24	0,16987	-1,35	-2,54	0,00581	---	---	
7934717	-1,16	-2,24	0,16987	-1,35	-2,54	0,00581	---	ASTN2	
8163678	-1,16	-2,24	0,08572	-0,76	-1,70	0,04787	NM_198186		CXorf67
8167650	-1,16	-2,24	0,00531	-1,71	-3,27	0,00620	BC046248	ZNF581	
8031522	-1,17	-2,24	0,01155	-0,42	-1,34	0,43659	NM_016535		ANKRD56
8101207	-1,17	-2,24	0,05635	-0,22	-1,17	0,36610	NM_001029870		P2RY1
8083447	-1,17	-2,24	0,01494	-0,87	-1,83	0,03150	NM_002563		C1orf109
7915045	-1,17	-2,24	0,00992	0,62	1,53	0,00840	BC018109	NPBWR1	
8146429	-1,17	-2,24	0,01576	-2,46	-5,49	0,00003	NM_005285		D2HGDH
8049919	-1,17	-2,24	0,01422	-0,70	-1,63	0,15822	NM_152783		---
8172195	-1,17	-2,24	0,37350	-2,33	-5,04	0,00087	---	LOC401097	
8091735	-1,17	-2,24	0,10698	-1,25	-2,37	0,00405	NM_001168214		---
7990452	-1,17	-2,24	0,24046	-1,12	-2,17	0,11966	---	NTN5	
8038139	-1,17	-2,24	0,04751	-1,53	-2,89	0,00248	NM_145807		---
8079964	-1,17	-2,24	0,07372	-1,01	-2,02	0,00448	---	NOS2	
8013536	-1,17	-2,24	0,08228	-0,75	-1,68	0,05004	NM_000625		DKK3
7938481	-1,17	-2,24	0,15069	-0,98	-1,98	0,00480	NM_015881		LOC440742
7911045	-1,17	-2,24	0,04409	-0,57	-1,49	0,06627	ENST00000452677		KCNS2
8147566	-1,17	-2,24	0,00118	-1,45	-2,73	0,00329	NM_020697		---
8098193	-1,17	-2,24	0,15051	-0,17	-1,12	0,34666	---	SULT6B1	
8051520	-1,17	-2,24	0,00768	-1,28	-2,43	0,00088	NM_001032377		RNASE1
7977615	-1,17	-2,24	0,03092	-1,22	-2,33	0,09193	NM_198232		SNORD101
8122142	-1,17	-2,24	0,08178	-1,33	-2,52	0,00005	NR_002434		ROS1
8129134	-1,17	-2,24	0,00756	-0,72	-1,65	0,00668	NM_002944		ACSM5
7993726	-1,17	-2,25	0,04680	-1,37	-2,58	0,04856	NM_017888		GLRA1
8115355	-1,17	-2,25	0,10322	-2,09	-4,25	0,01635	NM_001146040		VWA3A
7993898	-1,17	-2,25	0,05163	-1,42	-2,67	0,00243	NM_173615		AMBN
8095519	-1,17	-2,25	0,09267	-1,53	-2,88	0,00564	NM_016519		OR10K1
7906365	-1,17	-2,25	0,19294	-2,93	-7,60	0,00337	NM_001004473		PSG2
8037267	-1,17	-2,25	0,03929	-0,77	-1,70	0,16795	NM_031246		TCHH
7920141	-1,17	-2,25	0,00800	-1,54	-2,91	0,00121	NM_007113		CNIH2
7941587	-1,17	-2,25	0,01264	-0,87	-1,83	0,01909	NM_182553		---
8046281	-1,17	-2,25	0,05000	-0,91	-1,88	0,00319	---	SPDYE8P	
8140445	-1,17	-2,25	0,23303	-2,24	-4,73	0,00127	NR_003664		GAL
7942064	-1,17	-2,25	0,00248	2,31	4,96	0,00014	NM_015973		---
7978793	-1,17	-2,25	0,00040	-0,90	-1,87	0,02708	---	GIMAP6	
8143781	-1,17	-2,25	0,07148	-0,97	-1,96	0,00178	NM_024711		CACNA1F
8172580	-1,17	-2,25	0,02539	-0,59	-1,51	0,00121	NM_005183		---
8127688	-1,17	-2,25	0,16472	-1,06	-2,09	0,00017	---	---	
8050111	-1,17	-2,25	0,03150	-0,59	-1,51	0,00251	---	OR2T6	
7911276	-1,17	-2,25	0,02023	-1,07	-2,10	0,00016	NM_001005471		C9orf29
8163341	-1,17	-2,25	0,00372	-0,92	-1,89	0,06365	BC031236	---	
8051569	-1,17	-2,25	0,00411	-1,28	-2,43	0,02197	---	LOC100131138	
7958724	-1,17	-2,25	0,01599	-1,22	-2,32	0,00035	ENST00000331096		---
7959012	-1,17	-2,25	0,02116	-0,67	-1,59	0,05575	---	FLJ43315	
8077198	-1,17	-2,25	0,12880	-0,90	-1,87	0,00186	BC057848	---	
8060625	-1,17	-2,25	0,01426	-0,94	-1,92	0,00102	---	FAAH2	
8167930	-1,17	-2,25	0,03478	0,40	1,32	0,35042	NM_174912		---

8007503	-1,17	-2,25	0,00328	-1,63	-3,11	0,00317	---	GOLGA6A
7984985	-1,17	-2,25	0,31607	-0,67	-1,59	0,46611	NM_001038640	C14orf56
7979957	-1,17	-2,25	0,01367	-1,21	-2,32	0,01741	AK024445	OCR1
7908861	-1,17	-2,25	0,02640	-0,69	-1,61	0,14160	AF314543	MYH1
8012726	-1,17	-2,25	0,01710	-1,23	-2,35	0,00815	NM_005963	---
8084014	-1,17	-2,25	0,05910	-1,38	-2,61	0,00009	---	SLIT1
7935361	-1,17	-2,25	0,02381	-0,31	-1,24	0,19411	NM_003061	EML3
7948718	-1,17	-2,25	0,01433	0,57	1,49	0,25675	NM_153265	HIST1H3F
8124437	-1,17	-2,25	0,08974	-0,67	-1,59	0,06331	NM_021018	LOC728675
8007641	-1,17	-2,25	0,02117	0,67	1,59	0,39500	ENST00000434166	PRSS42
8086683	-1,17	-2,25	0,03164	-1,67	-3,17	0,00028	NM_182702	---
7945972	-1,17	-2,25	0,00191	-0,75	-1,68	0,00033	---	---
8019908	-1,17	-2,25	0,08331	-2,09	-4,27	0,00017	---	---
8100601	-1,17	-2,25	0,00029	-0,99	-1,98	0,05778	---	FLJ40606
8066637	-1,17	-2,25	0,01459	-2,07	-4,21	0,00908	AK097925	---
8121485	-1,17	-2,25	0,00179	-0,67	-1,59	0,00333	---	---
7998629	-1,17	-2,25	0,03855	-1,70	-3,25	0,01307	---	PRR19
8029214	-1,17	-2,25	0,00110	-0,47	-1,39	0,05491	NM_199285	PLEKHG6
7953303	-1,17	-2,25	0,05578	-0,42	-1,34	0,16034	NM_018173	RAP1GAP2
8003773	-1,17	-2,25	0,02385	1,56	2,95	0,00926	NM_015085	LTK
7987650	-1,17	-2,25	0,04127	-1,91	-3,77	0,00002	NM_002344	CCBL1
8164496	-1,17	-2,25	0,00530	0,05	1,04	0,87051	NM_004059	C2orf65
8053248	-1,17	-2,25	0,00568	-1,20	-2,29	0,06591	BC025997	C19orf52
8025783	-1,17	-2,25	0,18411	-0,58	-1,50	0,17818	BC011833	CMTM2
7996313	-1,17	-2,25	0,05932	-0,46	-1,37	0,08014	NM_144673	NPAS4
7941599	-1,17	-2,25	0,04695	-1,50	-2,83	0,00013	NM_178864	FKBP2
7940914	-1,17	-2,25	0,07215	-0,68	-1,61	0,15443	NM_004470	---
8137863	-1,17	-2,25	0,04219	-1,50	-2,84	0,02737	---	ENTPD2
8165416	-1,17	-2,25	0,02553	-0,40	-1,32	0,22236	NM_203468	---
7961453	-1,17	-2,25	0,00497	-1,29	-2,44	0,00022	---	---
8120979	-1,17	-2,25	0,01998	-1,07	-2,09	0,00148	---	---
8147242	-1,17	-2,25	0,08012	-0,82	-1,77	0,07084	---	OR6B3
8060084	-1,17	-2,25	0,04540	-0,97	-1,96	0,06827	NM_173351	---
8116577	-1,17	-2,25	0,00202	-1,23	-2,35	0,01434	---	NCRNA00105
8165765	-1,17	-2,25	0,04794	-1,37	-2,59	0,00097	NR_026711	CRYGB
8058530	-1,17	-2,25	0,05928	-1,19	-2,29	0,00092	NM_005210	B3GNT6
7942693	-1,17	-2,25	0,01498	-1,70	-3,26	0,00081	NM_138706	FBXO46
8037647	-1,17	-2,25	0,03935	-0,72	-1,64	0,08864	NM_001080469	SLC27A3
7905664	-1,17	-2,25	0,00401	-0,86	-1,81	0,15760	NM_024330	LGI4
8036055	-1,17	-2,25	0,05915	-1,28	-2,44	0,04188	NM_139284	PLP1
8169061	-1,17	-2,26	0,04069	-1,44	-2,72	0,00074	NM_000533	TCHHL1
7920135	-1,17	-2,26	0,04200	-0,93	-1,90	0,00797	NM_001008536	---
7966684	-1,17	-2,26	0,01251	-1,19	-2,28	0,01691	---	---
8121247	-1,17	-2,26	0,05616	-0,97	-1,96	0,06000	---	DHRS4L2
7973458	-1,17	-2,26	0,03969	0,96	1,95	0,15131	NM_198083	---
8148194	-1,17	-2,26	0,02594	-0,49	-1,41	0,00552	---	NHLH1
7906597	-1,17	-2,26	0,04686	-1,25	-2,37	0,02416	NM_005598	ASL
8133122	-1,17	-2,26	0,09813	-0,70	-1,63	0,24853	NM_001024943	---
7989659	-1,17	-2,26	0,06281	-1,39	-2,63	0,00911	---	MPG
7991742	-1,17	-2,26	0,03185	-1,03	-2,04	0,00143	NM_002434	ZNF408
7939699	-1,17	-2,26	0,01517	-0,44	-1,36	0,14960	NM_024741	NCRNA00164
8055303	-1,17	-2,26	0,01224	-1,77	-3,41	0,00026	BC029821	KIAA1614
7907882	-1,17	-2,26	0,05327	-1,09	-2,13	0,01869	NM_020950	---
7982292	-1,17	-2,26	0,02007	-0,83	-1,78	0,00163	---	MGC16075
8132707	-1,17	-2,26	0,02496	-0,97	-1,96	0,07540	BC007354	---
7957528	-1,17	-2,26	0,00696	-1,47	-2,77	0,01645	---	A2LD1
7972570	-1,17	-2,26	0,01230	-0,69	-1,61	0,08511	NM_033110	---
8089108	-1,17	-2,26	0,06348	-1,38	-2,59	0,00239	---	TMEM79
7906146	-1,17	-2,26	0,01859	-0,39	-1,31	0,21091	NR_026678	FAM170B
7933498	-1,17	-2,26	0,07707	-1,75	-3,35	0,00750	NM_001164484	FOXD4
8159808	-1,17	-2,26	0,08048	-1,56	-2,96	0,01779	NM_207305	---
8113871	-1,17	-2,26	0,02737	-1,27	-2,41	0,01673	---	MYH8
8012663	-1,17	-2,26	0,01784	-1,39	-2,62	0,00911	NM_002472	---
8051234	-1,18	-2,26	0,01165	-1,12	-2,18	0,00223	---	C18orf56
8021963	-1,18	-2,26	0,00678	0,37	1,29	0,39470	NM_001012716	HCG27
8179235	-1,18	-2,26	0,14721	-2,56	-5,91	0,07115	NR_026791	TUBB4
8033233	-1,18	-2,26	0,01563	1,00	2,00	0,04575	NM_006087	SPAG11B
8149180	-1,18	-2,26	0,02806	-1,35	-2,54	0,00306	NM_016512	---
8105218	-1,18	-2,26	0,01515	-1,02	-2,02	0,02384	---	LRIT1
7934783	-1,18	-2,26	0,06931	-1,16	-2,23	0,05187	NM_015613	ZMYND10
8087653	-1,18	-2,26	0,08181	-1,57	-2,97	0,01811	NM_015896	COX4I2
8061572	-1,18	-2,26	0,01819	-1,30	-2,45	0,01982	NM_032609	CTRC
7898167	-1,18	-2,26	0,14919	-0,99	-1,98	0,14588	NM_007272	RBMY1A3P

8177096	-1,18	-2,26	0,00455	-1,14	-2,21	0,09656	NR_001547	PRSS21
7992722	-1,18	-2,26	0,03343	-1,30	-2,46	0,07593	NM_006799	LOC100128175
8081808	-1,18	-2,26	0,04151	-0,92	-1,90	0,01332	AF119886	C21orf129
8070482	-1,18	-2,26	0,01404	-1,24	-2,36	0,03123	NR_027272	HMCN2
8158666	-1,18	-2,26	0,09556	-1,90	-3,72	0,00368	AK128680	FAM83H
8153488	-1,18	-2,26	0,02954	0,54	1,45	0,48895	NM_198488	ROBO3
7944882	-1,18	-2,26	0,01866	-0,45	-1,37	0,20670	NM_022370	EID3
7958200	-1,18	-2,26	0,06069	-0,86	-1,81	0,00344	NM_001008394	CALB2
7997139	-1,18	-2,26	0,07371	0,42	1,34	0,22343	NM_001740	ANKRD20A5
8020343	-1,18	-2,26	0,15889	-0,77	-1,71	0,16737	BC127726	ZNF703
8145829	-1,18	-2,26	0,02611	-2,45	-5,48	0,00001	NM_025069	B4GALNT3
7952927	-1,18	-2,26	0,05221	0,46	1,37	0,34034	NM_173593	---
8022570	-1,18	-2,26	0,01260	-1,05	-2,08	0,16924	---	SOX12
8060334	-1,18	-2,26	0,19496	-1,00	-1,99	0,16656	NM_006943	---
8091554	-1,18	-2,26	0,12669	-0,71	-1,64	0,01109	---	---
8003810	-1,18	-2,26	0,14564	-1,45	-2,74	0,02256	---	---
8114205	-1,18	-2,26	0,08830	-1,33	-2,52	0,00066	---	KRTAP4-5
8015232	-1,18	-2,26	0,07658	-1,66	-3,16	0,00445	NM_033188	ADPRHL1
7972902	-1,18	-2,26	0,14084	-0,35	-1,28	0,12812	NM_138430	TNFSF11
7968851	-1,18	-2,26	0,03253	-1,64	-3,11	0,00012	NM_003701	ISX
8072636	-1,18	-2,26	0,02537	-1,03	-2,04	0,00212	NM_001008494	KIF12
8163548	-1,18	-2,26	0,03616	-1,12	-2,18	0,00077	NM_138424	TNIP3
8102594	-1,18	-2,26	0,00475	-1,07	-2,10	0,00005	NM_024873	LOC100291336
8039584	-1,18	-2,26	0,07908	-1,00	-2,00	0,03141	XM_002347980	KLK14
8038770	-1,18	-2,26	0,15109	-1,61	-3,05	0,00953	NM_022046	FAM167B
7899750	-1,18	-2,26	0,11730	-1,69	-3,22	0,04214	NM_032648	LRRC8E
8025347	-1,18	-2,26	0,02362	-0,52	-1,44	0,19953	NM_025061	GPR149
8091588	-1,18	-2,26	0,08150	-1,46	-2,76	0,00104	NM_001038705	MYO16
7969995	-1,18	-2,26	0,01973	-1,72	-3,29	0,00273	NM_015011	TMIGD1
8014025	-1,18	-2,26	0,06888	-0,80	-1,74	0,11766	NM_206832	---
8148515	-1,18	-2,27	0,03614	-2,12	-4,33	0,00082	---	OIT3
7928330	-1,18	-2,27	0,02381	-1,65	-3,13	0,00460	NM_152635	DERL3
8074944	-1,18	-2,27	0,10374	-0,66	-1,58	0,23998	NM_198440	MGC14436
7966229	-1,18	-2,27	0,01604	-0,11	-1,08	0,49690	NR_026661	---
8139083	-1,18	-2,27	0,21236	-0,96	-1,95	0,01489	---	LOC220077
7950140	-1,18	-2,27	0,04015	-1,34	-2,53	0,00077	AF229166	OPLAH
8153625	-1,18	-2,27	0,02607	0,43	1,35	0,35491	NM_017570	JAK3
8035351	-1,18	-2,27	0,00206	-0,75	-1,68	0,21361	NM_000215	LRRC3B
8078305	-1,18	-2,27	0,03016	-1,36	-2,58	0,00497	NM_052953	---
8045005	-1,18	-2,27	0,00020	-0,97	-1,95	0,06868	---	C20orf200
8067530	-1,18	-2,27	0,02097	-1,81	-3,50	0,00746	NR_033263	CYP4F3
8026456	-1,18	-2,27	0,02286	-1,05	-2,06	0,05917	NM_000896	SLC18A2
7930837	-1,18	-2,27	0,00163	1,08	2,11	0,09131	NM_003054	---
7938685	-1,18	-2,27	0,04684	-0,10	-1,07	0,72005	---	---
7952737	-1,18	-2,27	0,01774	-0,90	-1,87	0,00877	---	LRRC25
8035456	-1,18	-2,27	0,09771	-1,14	-2,21	0,00729	NM_145256	KLK2
8030768	-1,18	-2,27	0,04815	-0,77	-1,71	0,01381	NM_001002231	CRB2
8157731	-1,18	-2,27	0,07820	-1,17	-2,25	0,00057	NM_173689	DTX4
7940160	-1,18	-2,27	0,03591	-0,42	-1,33	0,38592	NM_015177	C21orf30
8069131	-1,18	-2,27	0,00306	-0,56	-1,47	0,31393	ENST00000443689	HLA-DQA2
8178205	-1,18	-2,27	0,04961	-2,23	-4,70	0,00079	NM_020056	---
7975222	-1,18	-2,27	0,00033	-1,07	-2,10	0,03810	---	MIR129-2
7939463	-1,18	-2,27	0,07592	-2,11	-4,32	0,00420	NR_029697	ELMO3
7996468	-1,18	-2,27	0,08105	0,39	1,31	0,46282	NM_024712	ISOC2
8039511	-1,18	-2,27	0,02808	-0,40	-1,32	0,05646	NM_024710	---
8107127	-1,18	-2,27	0,08303	-1,70	-3,25	0,00479	---	SLC22A12
7941074	-1,18	-2,27	0,00788	-1,15	-2,22	0,00191	NM_144585	---
8047185	-1,18	-2,27	0,12817	-1,03	-2,05	0,01219	---	---
8121945	-1,18	-2,27	0,04039	-1,42	-2,68	0,00066	---	TSPAN15
7928046	-1,18	-2,27	0,00918	2,39	5,23	0,00773	NM_012339	FOXG1
7973745	-1,18	-2,27	0,06110	-2,55	-5,84	0,00824	NM_005249	---
8056215	-1,18	-2,27	0,04294	-1,36	-2,57	0,00814	---	ERVWE1
8140909	-1,18	-2,27	0,07588	-1,99	-3,98	0,00561	NM_014590	SEMA4D
8162231	-1,18	-2,27	0,05416	-0,02	-1,02	0,93479	NM_001142287	NDRG2
7977621	-1,18	-2,27	0,03369	1,03	2,05	0,00900	NM_201540	TUBB4Q
8104119	-1,18	-2,27	0,26215	-0,89	-1,85	0,01846	NM_020040	OR1S2
7948312	-1,18	-2,27	0,00405	-1,56	-2,94	0,04785	NM_001004459	DEFB112
8126943	-1,18	-2,27	0,00075	-1,13	-2,19	0,00533	NM_001037498	OR2C3
7925720	-1,18	-2,27	0,00285	-1,55	-2,93	0,00053	NM_198074	FRMD1
8130837	-1,18	-2,27	0,01209	-1,37	-2,58	0,01760	NM_024919	C20orf135
8064203	-1,18	-2,27	0,07015	-1,53	-2,88	0,00546	NM_080622	NUBP2
7992388	-1,18	-2,27	0,02535	-0,42	-1,34	0,31700	NM_012225	OR10W1
7948316	-1,18	-2,27	0,01245	-1,14	-2,21	0,00146	NM_207374	TSPY1

8176494	-1,18	-2,27	0,07678	-1,34	-2,54	0,00017	NM_003308	SYT12
7941749	-1,18	-2,27	0,02611	-0,40	-1,32	0,29243	NM_177963	S100A7A
7905576	-1,18	-2,27	0,20742	-1,84	-3,59	0,00211	NM_176823	---
7958207	-1,18	-2,27	0,14896	-1,21	-2,31	0,03043	---	AIF1
8118158	-1,18	-2,27	0,03144	-1,10	-2,15	0,03498	NM_004847	AIF1
8177996	-1,18	-2,27	0,03144	-1,10	-2,15	0,03498	NM_004847	AIF1
8179276	-1,18	-2,27	0,03144	-1,10	-2,15	0,03498	NM_004847	LRRTM1
8053349	-1,18	-2,27	0,05205	-1,19	-2,28	0,00034	NM_178839	TAF6L
7948834	-1,18	-2,27	0,04897	-0,89	-1,86	0,04637	NM_006473	---
8006083	-1,18	-2,27	0,02796	-1,62	-3,08	0,01650	---	---
8101990	-1,18	-2,27	0,01637	-0,42	-1,34	0,02605	---	DDX12
7961031	-1,18	-2,27	0,04086	0,44	1,35	0,39380	NR_033399	---
7957627	-1,18	-2,27	0,01395	-1,32	-2,50	0,19530	---	---
8003605	-1,18	-2,27	0,64287	-2,55	-5,84	0,03332	---	ZBED2
8089467	-1,18	-2,27	0,02211	-0,83	-1,77	0,29926	NM_024508	FLJ46836
8164627	-1,19	-2,27	0,01898	-1,24	-2,37	0,00758	ENST00000358748	---
7932610	-1,19	-2,27	0,06273	-0,96	-1,95	0,00276	---	HSD3B1
7904421	-1,19	-2,27	0,00164	-1,26	-2,40	0,00111	NM_000862	---
8100389	-1,19	-2,27	0,03576	-0,73	-1,66	0,03381	---	---
7903024	-1,19	-2,27	0,11585	-1,63	-3,08	0,00010	---	---
8089462	-1,19	-2,27	0,00985	-2,01	-4,03	0,00121	---	---
7932130	-1,19	-2,27	0,00569	-1,88	-3,67	0,00067	---	---
8137635	-1,19	-2,27	0,11218	-1,43	-2,69	0,06337	---	KRTAP3-1
8015173	-1,19	-2,27	0,02144	-1,39	-2,61	0,00450	NM_031958	KRTAP3-1
8019593	-1,19	-2,27	0,02144	-1,39	-2,61	0,00450	NM_031958	OR13C9
8162938	-1,19	-2,27	0,04043	-1,49	-2,80	0,03187	NM_001001956	---
8004165	-1,19	-2,27	0,00575	-0,87	-1,83	0,05669	---	NDUFB1
7980955	-1,19	-2,27	0,01691	-0,62	-1,53	0,04448	NM_004545	RUFY4
8048211	-1,19	-2,28	0,00574	-0,45	-1,37	0,04715	NM_198483	---
8010059	-1,19	-2,28	0,00337	-0,97	-1,96	0,00023	---	RAD51L3
8014199	-1,19	-2,28	0,00272	0,53	1,45	0,19509	NM_002878	HMGB4
7899898	-1,19	-2,28	0,01456	-0,81	-1,75	0,03661	NM_145205	TTC16
8158094	-1,19	-2,28	0,01794	-1,76	-3,38	0,01444	NM_144965	TSPY2
8176419	-1,19	-2,28	0,07307	-1,46	-2,74	0,00085	NM_022573	CTSE
7909164	-1,19	-2,28	0,11315	-0,97	-1,96	0,04496	NM_001910	ZCCHC3
8060330	-1,19	-2,28	0,01331	-0,34	-1,27	0,29678	NM_033089	---
8142401	-1,19	-2,28	0,01902	-0,82	-1,77	0,00365	---	---
8158991	-1,19	-2,28	0,00085	-0,95	-1,93	0,06586	---	OR51B2
7946069	-1,19	-2,28	0,12224	-1,59	-3,00	0,02551	NM_033180	KLK7
8038695	-1,19	-2,28	0,01037	-1,36	-2,56	0,00053	NM_139277	ANGPT4
8064396	-1,19	-2,28	0,00200	-0,85	-1,80	0,09345	NM_015985	SNORD96B
8174507	-1,19	-2,28	0,06603	-1,23	-2,35	0,00245	NR_004379	---
8082242	-1,19	-2,28	0,03251	-1,73	-3,32	0,00550	---	APOA2
7921834	-1,19	-2,28	0,01350	-1,12	-2,17	0,00902	NM_001643	XAGE5
8167742	-1,19	-2,28	0,00196	-1,17	-2,26	0,06809	NM_130775	USP17
8094116	-1,19	-2,28	0,50819	-3,29	-9,78	0,00040	NM_001105662	USP17
8094118	-1,19	-2,28	0,50819	-3,29	-9,78	0,00040	NM_001105662	USP17
8094120	-1,19	-2,28	0,50819	-3,29	-9,78	0,00040	NM_001105662	USP17
8094124	-1,19	-2,28	0,50819	-3,29	-9,78	0,00040	NM_001105662	USP17
8094126	-1,19	-2,28	0,50819	-3,29	-9,78	0,00040	NM_001105662	USP17
8094128	-1,19	-2,28	0,50819	-3,29	-9,78	0,00040	NM_001105662	USP17
8094132	-1,19	-2,28	0,50819	-3,29	-9,78	0,00040	NM_001105662	STOX2
8098549	-1,19	-2,28	0,01869	0,18	1,13	0,56616	NM_020225	C10orf120
7936793	-1,19	-2,28	0,15533	-1,42	-2,67	0,02626	BC140922	IGFL4
8037750	-1,19	-2,28	0,05795	-1,51	-2,85	0,00920	NM_001002923	BANF2
8061122	-1,19	-2,28	0,00079	-0,81	-1,75	0,06555	NM_178477	COX7B2
8100127	-1,19	-2,28	0,00470	-1,07	-2,09	0,00739	NM_130902	CNGA4
7938082	-1,19	-2,28	0,00362	-0,47	-1,39	0,16005	NM_001037329	CDCA3
7960702	-1,19	-2,28	0,00707	1,55	2,93	0,00366	NM_031299	LOC283711
7982333	-1,19	-2,28	0,00765	-0,72	-1,65	0,06883	AK127052	SNORD62A
8158862	-1,19	-2,28	0,05793	-0,89	-1,86	0,05409	NR_002914	SNORD62A
8158864	-1,19	-2,28	0,05793	-0,89	-1,86	0,05409	NR_002914	STH
8007846	-1,19	-2,28	0,04184	-0,48	-1,39	0,00474	NM_001007532	C10orf27
7934166	-1,19	-2,28	0,02852	-0,61	-1,53	0,03109	NM_152710	PTF1A
7926674	-1,19	-2,28	0,13536	-1,44	-2,72	0,02711	NM_178161	ARRDC2
8026915	-1,19	-2,28	0,01294	-0,10	-1,08	0,85351	NM_015683	MXD3
8116012	-1,19	-2,28	0,06288	-0,57	-1,48	0,28559	NM_031300	---
8174277	-1,19	-2,28	0,00110	-1,13	-2,19	0,07925	---	ACTR3C
8143766	-1,19	-2,28	0,01215	-1,76	-3,38	0,01771	NM_001164458	SIRPB1
8064471	-1,19	-2,28	0,03264	-0,51	-1,42	0,15053	NM_006065	---
8007152	-1,19	-2,28	0,14151	-0,97	-1,96	0,02242	---	---
8133900	-1,19	-2,28	0,06174	-1,43	-2,70	0,03785	---	---
8051236	-1,19	-2,28	0,01711	-1,57	-2,97	0,02103	---	C22orf45

8075005	-1,19	-2,28	0,06815	-1,75	-3,37	0,00174	NR_028484	NOS3
8137280	-1,19	-2,28	0,04283	-1,80	-3,49	0,00094	NM_000603	---
7988412	-1,19	-2,28	0,00351	-1,59	-3,02	0,00283	---	PRODH2
8036172	-1,19	-2,28	0,03865	-2,41	-5,31	0,00243	ENST00000443172	---
8107530	-1,19	-2,28	0,07606	-2,80	-6,95	0,00155	---	THOP1
8024497	-1,19	-2,28	0,00838	-0,27	-1,21	0,42494	NM_003249	ZSCAN4
8031799	-1,19	-2,28	0,00971	-1,09	-2,13	0,00168	NM_152677	CRMP1
8099172	-1,19	-2,28	0,05346	-1,48	-2,79	0,00067	NM_001313	TMED6
8002283	-1,19	-2,28	0,00861	-0,82	-1,76	0,06136	NM_144676	CCR9
8079370	-1,19	-2,28	0,05415	-1,39	-2,63	0,04222	NM_031200	FGD2
8119132	-1,19	-2,28	0,03347	-1,34	-2,52	0,00977	NM_173558	---
8095159	-1,19	-2,28	0,30818	-2,99	-7,95	0,00436	---	ACAA1
8086216	-1,19	-2,28	0,00891	-2,02	-4,06	0,00684	AK127051	---
8151583	-1,19	-2,28	0,00132	-0,84	-1,79	0,00286	---	---
8112043	-1,19	-2,28	0,08382	-1,14	-2,21	0,01425	---	---
8169233	-1,19	-2,28	0,02033	-1,38	-2,61	0,14121	---	PEX10
7911720	-1,19	-2,28	0,02755	0,38	1,30	0,35774	NM_153818	PHOX2A
7950136	-1,19	-2,28	0,07149	-1,71	-3,28	0,00013	NM_005169	MIR339
8137707	-1,19	-2,28	0,24043	-2,05	-4,14	0,03699	NR_029898	---
7936966	-1,19	-2,28	0,00388	-0,75	-1,69	0,00276	---	---
8035175	-1,19	-2,28	0,00397	-0,92	-1,90	0,01802	---	---
7944021	-1,19	-2,28	0,00275	-1,19	-2,28	0,00748	---	ASPRV1
8052828	-1,19	-2,29	0,07531	-0,52	-1,43	0,17163	NM_152792	FBXL15
7930099	-1,19	-2,29	0,00153	-0,80	-1,74	0,06188	NM_024326	ASTL
8053872	-1,19	-2,29	0,02116	-1,87	-3,66	0,00005	NM_001002036	FAM109A
7966389	-1,19	-2,29	0,11977	-0,59	-1,51	0,27655	NM_001177996	---
8000369	-1,19	-2,29	0,09488	-0,99	-1,99	0,01035	---	SLC23A1
8114491	-1,19	-2,29	0,11223	-0,41	-1,32	0,16280	NM_005847	---
8123763	-1,19	-2,29	0,03371	-1,13	-2,19	0,02115	---	KIF17
7913271	-1,19	-2,29	0,01646	-1,41	-2,66	0,00351	NM_020816	BCAS1
8067125	-1,19	-2,29	0,00959	-1,73	-3,32	0,02882	NM_003657	MBD1
8023323	-1,19	-2,29	0,08683	-0,20	-1,15	0,63599	NM_015846	MIR150
8038393	-1,19	-2,29	0,04592	-1,47	-2,76	0,01715	NR_029703	CYLC1
8168569	-1,19	-2,29	0,12714	-1,42	-2,68	0,00200	NM_021118	---
7925927	-1,19	-2,29	0,14195	-1,15	-2,22	0,07037	---	---
7931830	-1,19	-2,29	0,14195	-1,15	-2,22	0,07037	---	SIK1
8070665	-1,19	-2,29	0,02187	2,80	6,98	0,00437	NM_173354	---
8080909	-1,19	-2,29	0,17268	-2,24	-4,72	0,01374	---	HBA2
7991762	-1,19	-2,29	0,06905	-1,97	-3,92	0,00741	NM_000517	HBA1
7991766	-1,19	-2,29	0,06905	-1,97	-3,92	0,00741	NM_000558	DEFB114
8126934	-1,19	-2,29	0,10049	-1,05	-2,06	0,00085	NM_001037499	---
8162247	-1,19	-2,29	0,15756	-0,56	-1,47	0,03393	---	LLGL2
8009844	-1,19	-2,29	0,04647	1,51	2,85	0,07366	NM_001031803	---
8109824	-1,19	-2,29	0,12586	-1,24	-2,37	0,00020	---	ISL2
7985080	-1,19	-2,29	0,02483	-1,35	-2,55	0,00837	NM_145805	C10orf92
7937112	-1,19	-2,29	0,01364	-1,32	-2,49	0,00483	BC034223	---
8002758	-1,19	-2,29	0,01595	-1,02	-2,03	0,00891	---	KRTAP4-7
8007112	-1,19	-2,29	0,32065	-3,64	-12,45	0,00027	NM_033061	CEACAM8
8037222	-1,19	-2,29	0,18782	-1,59	-3,01	0,02941	NM_001816	SLC46A2
8163437	-1,19	-2,29	0,05252	-1,48	-2,78	0,00734	NM_033051	---
8075479	-1,19	-2,29	0,13276	-0,76	-1,69	0,00331	---	---
8112178	-1,20	-2,29	0,00331	-1,46	-2,76	0,01755	---	TNS4
8015016	-1,20	-2,29	0,00225	2,58	5,96	0,00012	NM_032865	FAM173A
7992043	-1,20	-2,29	0,01261	-0,75	-1,69	0,29266	BC001181	---
7989768	-1,20	-2,29	0,00028	-0,81	-1,75	0,05652	---	CENPVL1
8167652	-1,20	-2,29	0,03414	-1,42	-2,67	0,02566	AY730278	CENPVL1
8172715	-1,20	-2,29	0,03414	-1,42	-2,67	0,02566	AY730278	---
8123332	-1,20	-2,29	0,02166	-1,71	-3,27	0,00319	---	RNU105B
8060895	-1,20	-2,29	0,04748	-1,56	-2,94	0,03399	NR_004386	ABCB8
8137330	-1,20	-2,29	0,10689	-2,25	-4,74	0,01672	ENST00000356058	---
8129179	-1,20	-2,29	0,00449	-1,21	-2,31	0,00026	---	---
8018093	-1,20	-2,29	0,12294	-0,36	-1,28	0,07198	---	---
8049197	-1,20	-2,29	0,05115	-1,66	-3,17	0,03277	---	---
8110674	-1,20	-2,29	0,03152	-1,03	-2,04	0,05191	---	TREML3
8126296	-1,20	-2,29	0,08879	-1,76	-3,38	0,00104	NR_027256	TMEM132D
7967641	-1,20	-2,29	0,02170	-0,99	-1,98	0,01422	NM_133448	---
8118724	-1,20	-2,29	0,00967	-1,10	-2,14	0,14431	---	---
7919747	-1,20	-2,29	0,01873	-0,76	-1,69	0,00039	---	C5orf38
8104307	-1,20	-2,29	0,03697	-1,89	-3,71	0,00172	NM_178569	C14orf178
7975995	-1,20	-2,29	0,00524	-1,46	-2,76	0,06006	NM_174943	FLJ32756
8076813	-1,20	-2,29	0,09598	-0,77	-1,71	0,10457	BC041833	SCARNA5
8049297	-1,20	-2,29	0,01577	-1,05	-2,07	0,02289	NR_003008	---
8066198	-1,20	-2,29	0,00840	-0,88	-1,84	0,00739	---	FAM169A

8106278	-1,20	-2,29	0,00867	-0,51	-1,42	0,01832	NM_015566	PEX5L
8092277	-1,20	-2,29	0,04041	-1,42	-2,67	0,00069	NM_016559	SNORD116-10
7981968	-1,20	-2,29	0,01599	-1,04	-2,06	0,00042	NR_003325	ATP4B
7972923	-1,20	-2,29	0,08596	-1,74	-3,33	0,00211	NM_000705	MMP27
7951235	-1,20	-2,29	0,00346	-1,29	-2,44	0,00140	NM_022122	NCKAP5L
7963121	-1,20	-2,29	0,01263	-0,80	-1,74	0,06977	NM_001037806	MIR181B2
8157802	-1,20	-2,29	0,05530	-1,49	-2,82	0,00785	NR_029782	NR0B2
7914000	-1,20	-2,29	0,01511	-1,33	-2,51	0,03300	NM_021969	NNAT
8062395	-1,20	-2,29	0,05564	-1,64	-3,12	0,00031	NM_005386	ANXA8L2
7927307	-1,20	-2,29	0,02361	-0,96	-1,94	0,00132	NM_001630	LOC643332
7973108	-1,20	-2,29	0,02344	-1,50	-2,83	0,00057	ENST00000258817	RP11-410N8.4
8061742	-1,20	-2,29	0,01393	-2,35	-5,10	0,00117	ENST00000375670	---
8136576	-1,20	-2,29	0,00962	-1,96	-3,89	0,00154	---	LRRC24
8153862	-1,20	-2,29	0,04705	-1,92	-3,78	0,00810	NM_001024678	TMEM82
7898256	-1,20	-2,29	0,07345	-0,93	-1,91	0,12764	NM_001013641	---
8106189	-1,20	-2,29	0,06016	-0,65	-1,57	0,00213	---	C3orf54
8079839	-1,20	-2,29	0,01016	-1,29	-2,45	0,01351	NM_203370	FLJ45983
7932014	-1,20	-2,29	0,00836	-1,95	-3,87	0,00196	NR_024256	---
8035935	-1,20	-2,30	0,09053	-0,81	-1,75	0,02760	---	---
8176779	-1,20	-2,30	0,03466	-2,00	-4,00	0,00059	---	SERPINF2
8003656	-1,20	-2,30	0,06644	-0,74	-1,67	0,17059	NM_001165920	MIR130B
8071564	-1,20	-2,30	0,00310	-0,87	-1,82	0,02655	NR_029845	---
8037760	-1,20	-2,30	0,04373	-0,97	-1,96	0,00242	---	OSBP2
8072382	-1,20	-2,30	0,01795	0,32	1,25	0,27610	NM_030758	MIR143
8109157	-1,20	-2,30	0,05406	-0,88	-1,84	0,00013	NR_029684	KLHDC7A
7898533	-1,20	-2,30	0,21987	-1,82	-3,52	0,02828	NM_152375	---
7916020	-1,20	-2,30	0,03266	-0,48	-1,39	0,15067	---	---
7901854	-1,20	-2,30	0,04449	-1,54	-2,92	0,01522	---	---
8022410	-1,20	-2,30	0,00476	-0,84	-1,79	0,09459	---	OR6W1P
8143557	-1,20	-2,30	0,00312	-0,99	-1,98	0,00029	NR_002140	---
8112582	-1,20	-2,30	0,04872	-0,66	-1,58	0,01814	---	BSPRY
8157300	-1,20	-2,30	0,00892	1,16	2,23	0,01064	NM_017688	---
8062235	-1,20	-2,30	0,00342	-0,86	-1,82	0,00843	---	---
8098259	-1,20	-2,30	0,23481	-1,35	-2,55	0,00966	---	HS6ST3
7969665	-1,20	-2,30	0,06098	-1,32	-2,50	0,00673	NM_153456	C8orf42
8148973	-1,20	-2,30	0,03460	0,25	1,19	0,39793	NM_175075	COBL
8139680	-1,20	-2,30	0,02873	3,45	10,95	0,00036	NM_015198	---
8081337	-1,20	-2,30	0,03363	-1,64	-3,12	0,00028	---	AFARP1
7904084	-1,20	-2,30	0,00390	-0,49	-1,40	0,00970	NR_002796	MYO1G
8139433	-1,20	-2,30	0,04201	-0,78	-1,72	0,00379	NM_033054	C11orf36
7937868	-1,20	-2,30	0,01025	-1,19	-2,29	0,01353	NR_027138	FLJ46358
7970597	-1,20	-2,30	0,00008	-1,54	-2,92	0,05134	AK128225	BCL2
8023646	-1,20	-2,30	0,00321	-0,97	-1,95	0,02204	NM_000633	POU5F1P4 // POU5F1P4
7906017	-1,20	-2,30	0,02507	-2,14	-4,41	0,00115	GU480875 // GU480875	GIMAP8
8137232	-1,20	-2,30	0,00144	-1,26	-2,39	0,00394	NM_175571	---
8146943	-1,20	-2,30	0,02079	-1,65	-3,15	0,00014	---	CELA2A
7898176	-1,20	-2,30	0,03794	-1,33	-2,51	0,00089	NM_033440	---
8132856	-1,20	-2,30	0,19124	-0,89	-1,85	0,00299	---	MIR206
8120206	-1,20	-2,30	0,17174	-1,83	-3,55	0,00130	NR_029713	---
8139843	-1,20	-2,30	0,03318	-1,35	-2,54	0,00000	---	CLDN5
8074473	-1,20	-2,30	0,01075	-1,10	-2,14	0,03183	NM_001130861	MGC34800
7995310	-1,20	-2,30	0,09451	-1,52	-2,88	0,04693	ENST00000319817	XKR5
8149086	-1,20	-2,30	0,01160	-0,64	-1,56	0,07315	NM_207411	BSND
7901691	-1,20	-2,30	0,01963	-1,27	-2,41	0,05458	NM_057176	C1orf216
7914847	-1,20	-2,31	0,01623	-1,57	-2,97	0,00135	BC026909	TIMM17B
8172453	-1,20	-2,31	0,04825	-1,08	-2,11	0,06202	NM_001167947	OR7E18P
8033742	-1,21	-2,31	0,02244	-1,41	-2,66	0,00577	X89673	MYF6
7957373	-1,21	-2,31	0,02304	-0,84	-1,80	0,01383	NM_002469	C7orf29
8137219	-1,21	-2,31	0,16492	-0,98	-1,97	0,03889	NM_138434	---
8017310	-1,21	-2,31	0,00458	-0,91	-1,88	0,00016	---	---
8007917	-1,21	-2,31	0,12540	-3,01	-8,03	0,00531	---	---
8113784	-1,21	-2,31	0,02730	-2,33	-5,03	0,00298	---	KCNC2
7964987	-1,21	-2,31	0,00552	-1,36	-2,57	0,00015	NM_139136	---
8046822	-1,21	-2,31	0,03037	-0,93	-1,91	0,03740	---	MEI1
8073474	-1,21	-2,31	0,04292	-1,17	-2,25	0,00083	NM_152513	FLJ45079
8018818	-1,21	-2,31	0,00529	-1,18	-2,27	0,00054	NR_028337	---
8025826	-1,21	-2,31	0,00539	-1,54	-2,90	0,00250	---	PACSIN1
8118804	-1,21	-2,31	0,02648	-0,94	-1,92	0,07402	NM_020804	---
7971161	-1,21	-2,31	0,01211	-1,10	-2,14	0,00340	---	SNORA58
8090688	-1,21	-2,31	0,05756	-0,33	-1,25	0,02703	NR_002985	---
8061912	-1,21	-2,31	0,01619	-1,06	-2,08	0,08406	---	TTTY6
8176782	-1,21	-2,31	0,01468	-1,24	-2,36	0,02385	NR_001527	TTTY6

8177347	-1,21	-2,31	0,01468	-1,24	-2,36	0,02385	NR_001527	IL17RE
8077668	-1,21	-2,31	0,10593	-1,47	-2,77	0,00770	NM_153480	LOC93622
8093943	-1,21	-2,31	0,05952	-0,53	-1,44	0,10951	NR_015433	LZTS1
8149612	-1,21	-2,31	0,09488	-1,45	-2,73	0,01508	NM_021020	---
8103483	-1,21	-2,31	0,25531	-4,04	-16,47	0,00237	---	---
8113344	-1,21	-2,31	0,25531	-4,04	-16,47	0,00237	---	---
8114207	-1,21	-2,31	0,25531	-4,04	-16,47	0,00237	---	ZIC3
8170200	-1,21	-2,31	0,00152	-1,75	-3,37	0,01531	NM_003413	---
7965154	-1,21	-2,31	0,06140	-2,06	-4,16	0,01169	---	GRAMD1B
7944769	-1,21	-2,31	0,04339	3,21	9,26	0,00009	NM_020716	---
8142343	-1,21	-2,31	0,05749	-1,68	-3,20	0,03206	---	TTC22
7916425	-1,21	-2,31	0,03942	-0,43	-1,34	0,41115	NM_017904	ARHGAP10
8103092	-1,21	-2,31	0,00095	-1,58	-3,00	0,00522	NM_024605	---
8120059	-1,21	-2,31	0,01351	-1,02	-2,02	0,04469	---	FA2H
8002792	-1,21	-2,31	0,01474	-0,01	-1,00	0,98416	NM_024306	KRTAP4-2
8015242	-1,21	-2,31	0,21224	-2,15	-4,44	0,05215	NM_033062	ZNF213
7992854	-1,21	-2,31	0,01988	-1,19	-2,29	0,03351	NM_004220	PRAMEF1
7897978	-1,21	-2,31	0,06564	-1,44	-2,71	0,02046	NM_023013	TSNARE1
8153304	-1,21	-2,31	0,06426	0,00	-1,00	0,98960	NM_145003	LAYN
7951612	-1,21	-2,31	0,10550	-2,02	-4,06	0,00032	AK293317	CEACAM5
8029086	-1,21	-2,31	0,01443	-0,13	-1,10	0,64214	NM_004363	GPRC5C
8009639	-1,21	-2,31	0,08830	0,61	1,53	0,08484	NM_022036	FAM90A1
7960881	-1,21	-2,31	0,05841	-1,78	-3,44	0,02652	NM_018088	---
8045086	-1,21	-2,31	0,00709	-1,50	-2,83	0,00326	---	MSLNL
7998351	-1,21	-2,31	0,07939	-1,82	-3,53	0,00880	NM_001025190	HMX2
7931184	-1,21	-2,31	0,03250	-0,70	-1,62	0,06299	NM_005519	HBZ
7991754	-1,21	-2,31	0,12743	-1,65	-3,14	0,01213	NM_005332	P2RX3
7940005	-1,21	-2,31	0,16270	-1,39	-2,62	0,00127	NM_002559	---
8089723	-1,21	-2,31	0,02083	-0,68	-1,60	0,05417	---	---
7979741	-1,21	-2,31	0,17782	-2,23	-4,68	0,00137	---	POU3F2
8121209	-1,21	-2,31	0,03928	-1,12	-2,18	0,00277	NM_005604	PADI3
7898431	-1,21	-2,31	0,02908	-1,30	-2,45	0,00079	NM_016233	BCR
8074730	-1,21	-2,31	0,10893	-1,56	-2,96	0,00532	NM_004327	NDRG4
7996219	-1,21	-2,31	0,00846	-0,58	-1,49	0,13536	NM_020465	TYMP
8077103	-1,21	-2,31	0,05029	-0,18	-1,13	0,65612	NM_001113756	---
8123313	-1,21	-2,31	0,01015	-1,20	-2,30	0,02140	---	---
8021993	-1,21	-2,31	0,01713	-0,98	-1,97	0,00027	---	GPR27
8080958	-1,21	-2,31	0,04714	-1,01	-2,02	0,06055	NM_018971	SLC37A2
7944931	-1,21	-2,31	0,01619	-1,13	-2,19	0,04453	NM_198277	---
8119107	-1,21	-2,31	0,05428	-1,61	-3,05	0,00001	---	KIFC2
8148867	-1,21	-2,31	0,00837	-0,78	-1,72	0,20203	NM_145754	GUCY1A2
7951437	-1,21	-2,31	0,02625	-1,69	-3,23	0,00068	NM_000855	ALOX15B
8004784	-1,21	-2,32	0,01679	-0,75	-1,68	0,10403	NM_001141	---
8170237	-1,21	-2,32	0,14185	-1,41	-2,65	0,00030	---	UNKL
7998457	-1,21	-2,32	0,02914	-0,30	-1,23	0,62474	NM_023076	FAM116B
8077036	-1,21	-2,32	0,04414	-1,12	-2,17	0,02258	NM_001001794	EBF2
8149865	-1,21	-2,32	0,01364	-1,36	-2,56	0,00252	NM_022659	FLJ45743
8023437	-1,21	-2,32	0,02894	-1,04	-2,06	0,00773	ENST00000382897	---
7896736	-1,21	-2,32	0,60614	-3,79	-13,84	0,03163	---	---
8023933	-1,21	-2,32	0,60614	-3,79	-13,84	0,03163	---	---
8124160	-1,21	-2,32	0,01258	-1,25	-2,38	0,00620	---	CATSPER1
7949553	-1,21	-2,32	0,04088	-1,35	-2,55	0,00146	NM_053054	---
8127690	-1,21	-2,32	0,05259	-0,76	-1,70	0,17864	---	C21orf111
8070900	-1,21	-2,32	0,07561	-1,59	-3,01	0,00014	ENST00000418846	ASB4
8134376	-1,21	-2,32	0,03971	-0,15	-1,11	0,59748	NM_016116	---
7954965	-1,21	-2,32	0,00512	-0,95	-1,93	0,00897	---	C15orf48
7983478	-1,21	-2,32	0,01382	1,35	2,55	0,00452	NM_032413	LOC100130071
7897286	-1,21	-2,32	0,06266	-1,02	-2,03	0,00357	AY358179	QRFP
8164630	-1,21	-2,32	0,05259	-1,61	-3,05	0,00120	NM_198180	APOL3
8075695	-1,21	-2,32	0,00011	-1,25	-2,38	0,00299	NR_027833	---
8112016	-1,21	-2,32	0,04136	-0,92	-1,89	0,00974	---	TMEM176B
8143790	-1,21	-2,32	0,04007	-1,83	-3,54	0,00050	NM_014020	---
7999433	-1,21	-2,32	0,02391	-1,18	-2,26	0,13017	---	FLJ27243
8001816	-1,21	-2,32	0,29595	-2,52	-5,72	0,00136	ENST00000456054	IRX4
8110865	-1,21	-2,32	0,02318	-0,95	-1,93	0,05685	NM_016358	---
8136610	-1,21	-2,32	0,01775	-0,88	-1,83	0,00209	---	CD48
7921667	-1,21	-2,32	0,00246	-1,13	-2,19	0,00768	NM_001778	---
8147373	-1,21	-2,32	0,04822	-0,72	-1,65	0,04128	---	LOC440905
8055143	-1,21	-2,32	0,06081	-0,74	-1,67	0,02648	BC144438	HCN4
7990244	-1,21	-2,32	0,03455	-0,48	-1,39	0,09320	NM_005477	FRG2B
7937251	-1,21	-2,32	0,14218	-2,17	-4,50	0,00555	NM_001080998	---
8051185	-1,21	-2,32	0,03869	-1,40	-2,63	0,00111	---	IL4
8107977	-1,21	-2,32	0,07886	-1,22	-2,33	0,00007	NM_000589	DDX54

7958948	-1,21	-2,32	0,05900	-2,89	-7,43	0,00036	NM_001111322		KRT81
7963353	-1,21	-2,32	0,11307	1,51	2,86	0,09215	NM_002281		---
8152211	-1,21	-2,32	0,05861	-0,55	-1,46	0,00493	---	---	
7932304	-1,21	-2,32	0,03718	-1,80	-3,48	0,05447	---	---	
7995377	-1,21	-2,32	0,15675	-2,75	-6,73	0,00099	---	KLHL30	
8049610	-1,21	-2,32	0,00771	-1,27	-2,41	0,01494	NM_198582		PRRG2
8030393	-1,22	-2,32	0,13028	-0,42	-1,34	0,09318	NM_000951		RNF187
7910372	-1,22	-2,32	0,03086	0,50	1,41	0,55181	NM_001010858		SH2D3C
8164252	-1,22	-2,32	0,00812	-1,24	-2,36	0,02151	NM_170600		NGEF
8059783	-1,22	-2,32	0,00100	-0,63	-1,54	0,00483	NM_019850		SOAT2
7955702	-1,22	-2,32	0,00407	-1,10	-2,15	0,01427	NM_003578		TSPAN32
7937782	-1,22	-2,32	0,01201	-1,50	-2,82	0,00025	NM_139022		NOXO1
7998668	-1,22	-2,32	0,03210	-1,64	-3,12	0,00931	NM_144603		NANOS2
8037732	-1,22	-2,32	0,00850	-1,81	-3,50	0,00883	NM_001029861		PAK4
8028583	-1,22	-2,32	0,03867	1,28	2,42	0,06730	NM_001014831		FAM196A
7937007	-1,22	-2,32	0,00256	-1,15	-2,22	0,06539	NM_001039762		CYP17A1
7936050	-1,22	-2,32	0,00510	-1,00	-1,99	0,04848	NM_000102		FAM5B
7907601	-1,22	-2,32	0,00335	-1,96	-3,90	0,00053	NM_021165		---
7988703	-1,22	-2,32	0,08138	-0,51	-1,43	0,09873	---	---	
8009794	-1,22	-2,32	0,01661	-0,73	-1,66	0,00156	---	---	
8156577	-1,22	-2,32	0,00479	-1,20	-2,29	0,08291	---	CACNA1D	
8080511	-1,22	-2,32	0,02185	-0,57	-1,49	0,00072	NM_000720		FUT2
8030094	-1,22	-2,33	0,00953	0,32	1,25	0,13157	NM_000511		NGB
7980352	-1,22	-2,33	0,01053	-1,30	-2,46	0,00526	NM_021257		---
8020209	-1,22	-2,33	0,00414	-0,96	-1,95	0,01000	---	---	
8050441	-1,22	-2,33	0,21042	-2,68	-6,43	0,01590	---	---	
8013177	-1,22	-2,33	0,00051	-1,38	-2,60	0,00601	---	PDE6G	
8019231	-1,22	-2,33	0,13398	-1,32	-2,50	0,06575	NM_002602		---
7901445	-1,22	-2,33	0,03538	-0,47	-1,38	0,33565	---	---	
7988465	-1,22	-2,33	0,06320	-1,42	-2,68	0,01233	---	NXPH1	
8131506	-1,22	-2,33	0,01956	-1,51	-2,84	0,00454	NM_152745		C5orf50
8109990	-1,22	-2,33	0,02209	-1,86	-3,64	0,00165	ENST00000330910		CD8B
8044154	-1,22	-2,33	0,06301	-1,38	-2,61	0,01077	NM_172099		---
8174045	-1,22	-2,33	0,02940	-0,68	-1,60	0,07632	---	WRAP53	
8012270	-1,22	-2,33	0,04954	0,93	1,91	0,03546	NM_001143990		NPTX1
8019074	-1,22	-2,33	0,03321	0,18	1,13	0,61281	NM_002522		---
8168884	-1,22	-2,33	0,02820	-0,76	-1,69	0,01183	---	---	
8056975	-1,22	-2,33	0,01632	-1,00	-2,00	0,01633	---	PLS1	
8083146	-1,22	-2,33	0,00810	3,14	8,82	0,00011	NM_001172312		DEFB105A
8144401	-1,22	-2,33	0,06434	-1,59	-3,01	0,00060	NM_152250		DEFB105A
8149235	-1,22	-2,33	0,06434	-1,59	-3,01	0,00060	NM_152250		---
7903292	-1,22	-2,33	0,00150	-0,80	-1,74	0,15250	---	TMEM131	
8043745	-1,22	-2,33	0,03921	-0,46	-1,38	0,27388	ENST00000429765		DHX37
7967588	-1,22	-2,33	0,09797	0,47	1,39	0,36707	NM_032656		DEFA3
8149116	-1,22	-2,33	0,11337	-1,70	-3,25	0,00061	NM_005217		DEFA3
8149126	-1,22	-2,33	0,11337	-1,70	-3,25	0,00061	NM_005217		DEFA3
8149137	-1,22	-2,33	0,11337	-1,70	-3,25	0,00061	NM_005217		LOC100131826
8095868	-1,22	-2,33	0,00153	-0,67	-1,59	0,01826	AY358789	CYP24A1	
8067140	-1,22	-2,33	0,02202	4,64	24,85	0,00003	NM_000782		TNNI2
7937728	-1,22	-2,33	0,03326	-1,61	-3,05	0,00993	NM_001145829		ACBD4
8007730	-1,22	-2,33	0,04798	-1,25	-2,38	0,05418	NM_001135704		VSIG8
7921462	-1,22	-2,33	0,06071	-0,94	-1,92	0,00278	NM_001013661		DEFB103A
8144473	-1,22	-2,33	0,02397	-0,68	-1,60	0,04124	NM_018661		DEFB103A
8149172	-1,22	-2,33	0,02397	-0,68	-1,60	0,04124	NM_018661		KLHL35
7950492	-1,22	-2,33	0,08216	0,04	1,03	0,94905	NM_001039548		---
8065903	-1,22	-2,33	0,00789	-1,20	-2,30	0,00013	---	TMPRSS4	
7944164	-1,22	-2,33	0,07968	-1,03	-2,04	0,00379	NM_019894		---
8040630	-1,22	-2,33	0,04902	-1,27	-2,41	0,03974	---	RBMY1A1	
8176789	-1,22	-2,33	0,03108	-1,26	-2,40	0,00229	NM_005058		OR2D2
7946286	-1,22	-2,33	0,01074	-0,77	-1,70	0,01141	NM_003700		KCNB1
8066921	-1,22	-2,33	0,02084	-1,01	-2,02	0,06727	NM_004975		---
7971539	-1,22	-2,33	0,00371	-1,07	-2,10	0,04532	---	SCXA	
8148796	-1,22	-2,33	0,07777	-1,12	-2,17	0,00254	NM_001008271		SCXA
8148821	-1,22	-2,33	0,07777	-1,12	-2,17	0,00254	NM_001008271		TNNC2
8066590	-1,22	-2,33	0,03610	-1,53	-2,89	0,03424	NM_003279		FCN3
7914075	-1,22	-2,33	0,00199	-0,59	-1,50	0,06412	NM_003665		C17orf106
8010017	-1,22	-2,33	0,00075	0,09	1,06	0,77459	NM_001113324		LOC100129858
8102871	-1,22	-2,33	0,00545	0,53	1,45	0,13459	AK095554	OR2A2	
8136973	-1,22	-2,33	0,01417	-1,64	-3,12	0,00005	NM_001005480		GHRH
8066189	-1,22	-2,33	0,04428	-1,52	-2,87	0,00014	NM_021081		OR7E19P
8033744	-1,22	-2,33	0,04662	-1,48	-2,79	0,03333	X89668	ABCC12	
8001235	-1,22	-2,33	0,00250	-1,04	-2,05	0,00032	NM_033226		KCNE3
7950409	-1,22	-2,34	0,00742	-2,01	-4,03	0,00138	NM_005472		ODF3B

8077116	-1,22	-2,34	0,11086	-1,35	-2,55	0,02697	NM_001014440	EHD4
7987772	-1,22	-2,34	0,00036	1,71	3,28	0,02223	NM_139265	---
7909140	-1,22	-2,34	0,07021	-1,51	-2,84	0,01883	---	NMNAT2
7922756	-1,22	-2,34	0,01189	1,25	2,37	0,04387	NM_015039	GPRIN3
8101757	-1,22	-2,34	0,00246	-1,62	-3,07	0,03165	NM_198281	ANXA8
7927367	-1,22	-2,34	0,03421	-0,95	-1,94	0,00253	NM_001040084	FLJ45256
8000371	-1,22	-2,34	0,03956	-0,91	-1,88	0,02459	AK127191	GNG8
8037785	-1,22	-2,34	0,04299	-1,84	-3,57	0,00018	NM_033258	---
8113759	-1,22	-2,34	0,00081	-1,46	-2,75	0,00004	---	ANKRD43
8107992	-1,22	-2,34	0,04234	-1,85	-3,61	0,00106	NM_175873	OR7A2P
8034899	-1,22	-2,34	0,07781	-1,55	-2,93	0,00073	ENST00000304105	ANO7
8049799	-1,22	-2,34	0,05582	-1,47	-2,78	0,03928	NM_001001891	MYOZ3
8109315	-1,23	-2,34	0,13295	-1,15	-2,21	0,00229	NM_001122853	DPY19L2P4
8134026	-1,23	-2,34	0,05986	-1,13	-2,20	0,00319	NR_003551	---
7989313	-1,23	-2,34	0,33545	-2,49	-5,63	0,04371	---	GLRA3
8103778	-1,23	-2,34	0,00049	-1,36	-2,57	0,00648	NM_006529	---
8111989	-1,23	-2,34	0,03440	-0,15	-1,11	0,61693	---	USH2A
7924233	-1,23	-2,34	0,00081	-1,31	-2,48	0,00001	NM_206933	PHF21B
8076677	-1,23	-2,34	0,00624	-1,40	-2,63	0,00134	NM_138415	WNT7B
8076734	-1,23	-2,34	0,03315	0,79	1,73	0,33425	NM_058238	TIMP2
8018972	-1,23	-2,34	0,08707	-0,66	-1,58	0,05462	AK057217	FAM129C
8026787	-1,23	-2,34	0,25706	-1,61	-3,05	0,05510	NM_173544	FGFBP2
8099471	-1,23	-2,34	0,04432	-0,63	-1,55	0,03831	NM_031950	RAB40AL
8169006	-1,23	-2,34	0,06144	-1,41	-2,65	0,09563	NM_001031834	FSCN2
8010614	-1,23	-2,34	0,03081	-0,71	-1,63	0,40240	NM_012418	C19orf28
8032608	-1,23	-2,34	0,08511	2,23	4,68	0,02384	NM_174983	HCLS1
8089911	-1,23	-2,34	0,01367	-1,07	-2,10	0,00056	NM_005335	---
8031439	-1,23	-2,34	0,02268	-1,40	-2,63	0,07375	---	TCF7
8108050	-1,23	-2,34	0,00012	-0,42	-1,34	0,19033	NM_003202	UGT8
8097017	-1,23	-2,34	0,00888	3,36	10,29	0,00010	NM_001128174	---
8097541	-1,23	-2,34	0,04955	-1,25	-2,38	0,00457	---	CCDC96
8099242	-1,23	-2,34	0,00042	-1,02	-2,02	0,02052	NM_153376	---
7972414	-1,23	-2,34	0,04054	-0,98	-1,97	0,01155	---	---
8105987	-1,23	-2,34	0,36173	-3,77	-13,59	0,00346	---	SAMHD1
8062339	-1,23	-2,34	0,04388	-1,21	-2,31	0,00926	NM_015474	XCR1
8086595	-1,23	-2,34	0,04523	-2,12	-4,36	0,00322	NM_005283	PROX1
7909681	-1,23	-2,34	0,07033	-1,39	-2,62	0,00112	NM_002763	---
7910010	-1,23	-2,34	0,01627	-1,89	-3,70	0,00140	---	---
8074890	-1,23	-2,34	0,09098	-0,97	-1,95	0,09332	---	WISP2
8062864	-1,23	-2,34	0,03133	-0,25	-1,19	0,70896	NM_003881	LRP2BP
8103986	-1,23	-2,34	0,00016	-0,82	-1,76	0,05919	NM_018409	---
7968195	-1,23	-2,34	0,05293	-1,26	-2,39	0,01746	---	---
8074057	-1,23	-2,34	0,01955	-0,90	-1,87	0,13791	---	---
7985308	-1,23	-2,34	0,02297	-1,95	-3,87	0,00002	---	SNORD50B
8127989	-1,23	-2,34	0,02125	-1,35	-2,56	0,03333	NR_003044	C22orf26
8076739	-1,23	-2,34	0,03459	-0,90	-1,86	0,01421	NM_018280	MUC16
8033674	-1,23	-2,34	0,03418	1,42	2,67	0,00064	NM_024690	TAAR3
8129608	-1,23	-2,34	0,03641	-0,50	-1,41	0,09241	NR_028511	---
8176951	-1,23	-2,34	0,00470	-0,73	-1,66	0,12697	---	MTSS1L
7997135	-1,23	-2,34	0,07995	-1,46	-2,75	0,00312	NM_138383	---
8121487	-1,23	-2,35	0,05159	-0,84	-1,78	0,02235	---	H2AFB1
8170994	-1,23	-2,35	0,00215	-0,58	-1,49	0,05403	NM_001017990	H2AFB1
8176249	-1,23	-2,35	0,00215	-0,58	-1,49	0,05403	NM_001017990	KIF1A
8060134	-1,23	-2,35	0,00426	0,11	1,08	0,79348	NM_004321	MAS1
8123176	-1,23	-2,35	0,00246	-0,89	-1,85	0,00449	NM_002377	---
8137006	-1,23	-2,35	0,15123	-1,01	-2,02	0,00051	---	PCK1
8063590	-1,23	-2,35	0,01849	-1,19	-2,28	0,01007	NM_002591	OR10R3P
7906370	-1,23	-2,35	0,02315	-1,87	-3,65	0,01083	AK122667	C3orf20
8078046	-1,23	-2,35	0,00569	-1,01	-2,01	0,05012	BC038406	XDH
8051322	-1,23	-2,35	0,01997	-0,90	-1,87	0,05394	NM_000379	THSD7B
8045470	-1,23	-2,35	0,04436	-1,16	-2,24	0,00008	NM_001080427	SPDYE8P
8139996	-1,23	-2,35	0,20400	-2,45	-5,48	0,00034	NR_003664	---
7993606	-1,23	-2,35	0,00728	-0,76	-1,70	0,03774	---	---
8008967	-1,23	-2,35	0,14376	-1,61	-3,05	0,01897	---	LHX1
8006681	-1,23	-2,35	0,02726	0,08	1,06	0,85271	NM_005568	OR4K1
7973016	-1,23	-2,35	0,02371	-1,03	-2,04	0,07776	NM_001004063	---
7928819	-1,23	-2,35	0,07971	-1,42	-2,67	0,00603	---	ST8SIA1
7961755	-1,23	-2,35	0,05119	-1,11	-2,16	0,00033	NM_003034	ATP2B2
8085311	-1,23	-2,35	0,03163	-1,63	-3,08	0,01237	NM_001001331	MOS
8150879	-1,23	-2,35	0,09195	-1,53	-2,89	0,09304	NM_005372	KRTAP4-1
8015247	-1,23	-2,35	0,10348	-1,39	-2,61	0,00625	NM_033060	HEPACAM
7952475	-1,23	-2,35	0,04598	-1,72	-3,29	0,00298	NM_152722	---
7982533	-1,23	-2,35	0,01943	-0,88	-1,83	0,01108	---	CRISP1

8126920	-1,23	-2,35	0,01771	-1,79	-3,46	0,00036	NM_001131	---
7928556	-1,23	-2,35	0,01810	-2,00	-4,00	0,00517	---	SSTR1
7974047	-1,23	-2,35	0,05131	-2,57	-5,93	0,00026	NM_001049	REC8
7973629	-1,23	-2,35	0,00112	-1,19	-2,29	0,00064	NM_001048205	HAMP
8027819	-1,23	-2,35	0,11723	-1,42	-2,67	0,02828	NM_021175	---
7899560	-1,23	-2,35	0,24791	-1,62	-3,08	0,00272	---	---
7947394	-1,23	-2,35	0,01642	-1,57	-2,98	0,00037	---	ZNF662
8079124	-1,23	-2,35	0,00627	-1,38	-2,60	0,00002	NM_207404	FLJ41170
7976828	-1,23	-2,35	0,04575	-1,51	-2,86	0,00208	AK123165	C19orf70
8032996	-1,23	-2,35	0,03377	-1,22	-2,33	0,00513	NM_205767	KIR2DL3
8031277	-1,23	-2,35	0,16583	-2,45	-5,46	0,00009	NM_015868	BRSK1
8031441	-1,23	-2,35	0,01311	-0,25	-1,19	0,46083	NM_032430	TUSC1
8160472	-1,23	-2,35	0,06357	-1,38	-2,60	0,00604	NM_001004125	CD2
7904287	-1,23	-2,35	0,00048	-1,03	-2,04	0,00030	NM_001767	DUSP21
8166950	-1,23	-2,35	0,00164	-0,93	-1,91	0,00679	NM_022076	GPC2
8141466	-1,23	-2,35	0,02102	-1,94	-3,84	0,00057	NM_152742	WNT16
8135763	-1,23	-2,35	0,03456	-0,75	-1,68	0,10443	NM_057168	OPN1MW2
8170825	-1,23	-2,35	0,02002	-1,47	-2,78	0,00870	NM_001048181	HS6ST1
8055130	-1,23	-2,35	0,00743	-0,60	-1,51	0,31018	NM_004807	ALOX12P2
8004201	-1,23	-2,35	0,03850	0,39	1,31	0,20056	NR_002710	GINS4
8146130	-1,23	-2,35	0,03149	-0,86	-1,81	0,02784	NM_032336	PALM3
8034744	-1,23	-2,35	0,00056	-1,04	-2,05	0,05021	NM_001145028	LOC100131796
7989951	-1,24	-2,35	0,00225	-0,97	-1,96	0,23214	ENST00000380078	C20orf173
8065911	-1,24	-2,35	0,04425	-1,19	-2,28	0,01678	BC112060	GCGR
8019669	-1,24	-2,35	0,00855	-1,69	-3,22	0,00607	NM_000160	PACSIN3
7947801	-1,24	-2,36	0,01559	-0,22	-1,17	0,65314	NM_016223	ZNF580
8031516	-1,24	-2,36	0,01813	-0,70	-1,63	0,12305	NM_016202	PRB4
7961269	-1,24	-2,36	0,02296	-1,16	-2,23	0,01240	NM_002723	---
8052305	-1,24	-2,36	0,06650	-1,07	-2,10	0,02022	---	GSDMB
8014903	-1,24	-2,36	0,00510	-0,36	-1,29	0,39566	NM_001165958	SHE
7920506	-1,24	-2,36	0,02911	-1,50	-2,83	0,00183	NM_001010846	IRGC
8029318	-1,24	-2,36	0,02460	-1,18	-2,26	0,00231	NM_019612	CD7
8019478	-1,24	-2,36	0,04651	-1,15	-2,22	0,10590	NM_006137	---
8095562	-1,24	-2,36	0,00442	-1,10	-2,15	0,01665	---	RBM38
8063583	-1,24	-2,36	0,01691	-1,05	-2,08	0,04328	NM_017495	---
8014863	-1,24	-2,36	0,01359	-0,67	-1,59	0,08116	---	---
7997137	-1,24	-2,36	0,00431	-1,66	-3,16	0,02806	---	FRMPD3
8169235	-1,24	-2,36	0,04077	-0,04	-1,03	0,82785	ENST00000276185	---
8175193	-1,24	-2,36	0,05009	-1,06	-2,09	0,07086	---	DHRS2
7973433	-1,24	-2,36	0,00582	-1,01	-2,02	0,01236	NM_182908	ZAN
8134915	-1,24	-2,36	0,02755	-0,89	-1,85	0,00637	NM_003386	ITPKA
7982927	-1,24	-2,36	0,02376	-0,97	-1,96	0,09108	NM_002220	ADAM8
7937150	-1,24	-2,36	0,04355	-0,54	-1,45	0,29043	NM_001109	LST1
8118149	-1,24	-2,36	0,04998	-0,24	-1,18	0,52424	NM_205837	LST1
8177988	-1,24	-2,36	0,04998	-0,24	-1,18	0,52424	NM_205837	LST1
8179268	-1,24	-2,36	0,04998	-0,24	-1,18	0,52424	NM_205837	HTR4
8114977	-1,24	-2,36	0,00866	-1,59	-3,00	0,00020	NM_001040172	LBX1
7935855	-1,24	-2,36	0,11528	-1,58	-2,99	0,00418	NM_006562	---
8000649	-1,24	-2,36	0,04255	-2,39	-5,25	0,00042	---	---
8000690	-1,24	-2,36	0,04255	-2,39	-5,25	0,00042	---	SPIN2A
8173198	-1,24	-2,36	0,14144	-1,86	-3,63	0,00549	NM_019003	UNC5A
8110237	-1,24	-2,36	0,01107	0,64	1,56	0,14804	NM_133369	---
8065067	-1,24	-2,36	0,00155	-1,24	-2,36	0,00049	---	CEP72
8104220	-1,24	-2,36	0,13046	1,05	2,07	0,08477	NM_018140	---
8053447	-1,24	-2,36	0,10384	-2,06	-4,16	0,00436	---	MYO3B
8046238	-1,24	-2,36	0,03612	-0,53	-1,44	0,11797	NM_138995	PNMA5
8175763	-1,24	-2,36	0,03037	-1,18	-2,26	0,06226	NM_001103151	PIK3CD
7897482	-1,24	-2,36	0,00655	-0,66	-1,58	0,25925	NM_005026	HOMER3
8035566	-1,24	-2,36	0,03337	-1,18	-2,27	0,03349	NM_004838	SIGLEC10
8038824	-1,24	-2,36	0,06291	-1,48	-2,80	0,02338	NM_033130	SLC25A31
8097327	-1,24	-2,36	0,00124	-0,84	-1,79	0,03148	NM_031291	FAM181B
7950726	-1,24	-2,36	0,06540	-1,99	-3,97	0,00001	NM_175885	ARHGAP4
8175900	-1,24	-2,36	0,05106	-0,98	-1,97	0,00969	NM_001164741	---
8092763	-1,24	-2,36	0,00228	-1,21	-2,31	0,01262	---	C22orf36
8075016	-1,24	-2,36	0,02400	-1,38	-2,61	0,06688	NM_207644	KRT84
7963375	-1,24	-2,36	0,04469	-1,20	-2,29	0,09129	NM_033045	ACHE
8141648	-1,24	-2,36	0,00037	-1,16	-2,24	0,00206	NM_015831	CARKD
7972808	-1,24	-2,36	0,02220	-1,16	-2,24	0,00661	NM_018210	CD300C
8018177	-1,24	-2,36	0,02717	-0,89	-1,85	0,04371	NM_006678	OR10X1
7921364	-1,24	-2,36	0,01394	-0,83	-1,77	0,00161	NM_001004477	NEUROG1
8114245	-1,24	-2,36	0,04965	-2,70	-6,50	0,00291	NM_006161	CPLX4
8023554	-1,24	-2,36	0,02975	-1,61	-3,05	0,00083	NM_181654	---
8083407	-1,24	-2,36	0,00167	-1,46	-2,75	0,02101	---	PGLYRP1

8037742	-1,24	-2,36	0,06387	-1,77	-3,42	0,00315	NM_005091		C4orf38
8103867	-1,24	-2,36	0,04641	-1,21	-2,32	0,11699	NR_024008		---
8094068	-1,24	-2,36	0,00774	-1,61	-3,04	0,00309	---	---	
8151494	-1,24	-2,36	0,08240	-1,89	-3,71	0,00482	---	ABHD8	
8035271	-1,24	-2,36	0,01150	-0,30	-1,23	0,32509	NM_024527		KCNK7
7949444	-1,24	-2,36	0,00188	-1,00	-1,99	0,05485	NM_005714		REM2
7973363	-1,24	-2,36	0,00693	0,02	1,02	0,94067	NM_173527		---
8105179	-1,24	-2,37	0,01954	-2,17	-4,51	0,00026	---	KRTAP5-6	
7937709	-1,24	-2,37	0,05981	-0,95	-1,94	0,17934	NM_001012416		C21orf118
8068046	-1,24	-2,37	0,00066	-1,07	-2,10	0,00935	AF304442	FBXW9	
8034469	-1,24	-2,37	0,00905	-0,93	-1,90	0,01888	NM_032301		---
7921425	-1,24	-2,37	0,01815	-1,99	-3,96	0,00881	---	TSPY1	
8176508	-1,24	-2,37	0,06301	-1,34	-2,52	0,00024	NM_003308		CHP2
7994123	-1,24	-2,37	0,00685	-1,00	-2,00	0,03743	NM_022097		C15orf5
7990636	-1,24	-2,37	0,03897	-0,87	-1,82	0,09199	NR_026813		PAPL
8028570	-1,24	-2,37	0,00267	-0,68	-1,60	0,12815	NM_001004318		---
8096002	-1,24	-2,37	0,02090	-0,77	-1,71	0,00359	---	DNAJB13	
7942489	-1,24	-2,37	0,00293	-1,03	-2,05	0,01025	NM_153614		OR1L1
8157681	-1,24	-2,37	0,01382	-1,46	-2,75	0,00769	NM_001005236		SLC6A11
8077817	-1,24	-2,37	0,07750	0,93	1,90	0,01003	NM_014229		WDR90
7991939	-1,24	-2,37	0,00342	-1,52	-2,86	0,00077	NM_145294		TMC5
7993638	-1,24	-2,37	0,00706	3,60	12,16	0,00002	NM_001105248		KRTAP24-1
8069800	-1,24	-2,37	0,26084	-2,09	-4,25	0,01483	NM_001085455		H1FNT
7955112	-1,24	-2,37	0,05358	-1,50	-2,82	0,00098	NM_181788		CMA1
7978343	-1,24	-2,37	0,01735	-1,10	-2,15	0,01905	NM_001836		LRRFIP1
8049532	-1,24	-2,37	0,00546	-1,01	-2,02	0,03387	NM_001137550		LEFTY2
7924682	-1,24	-2,37	0,16084	-1,66	-3,17	0,03317	NM_003240		ALKBH4
8141757	-1,24	-2,37	0,02806	-1,70	-3,26	0,00638	NM_017621		RAB37
8009666	-1,24	-2,37	0,01043	-0,59	-1,51	0,18433	NM_175738		SNORA30
7994926	-1,24	-2,37	0,00028	-1,31	-2,49	0,00892	NR_002966		SULT1E1
8100808	-1,25	-2,37	0,00117	-0,59	-1,51	0,19421	NM_005420		FAM73B
8158492	-1,25	-2,37	0,01261	-0,20	-1,15	0,64515	BC009114	FXYD2	
7951966	-1,25	-2,37	0,06767	-1,34	-2,53	0,00771	NM_001127489		KLHL10
8007169	-1,25	-2,37	0,00890	-1,08	-2,12	0,00246	NM_152467		---
7976146	-1,25	-2,37	0,27084	-0,72	-1,64	0,20761	---	MARVELD3	
7997158	-1,25	-2,37	0,01336	-0,31	-1,24	0,44624	NM_001017967		GPR112
8170135	-1,25	-2,37	0,06039	-1,17	-2,26	0,03476	NM_153834		GALNT8
7953252	-1,25	-2,37	0,03663	-0,93	-1,90	0,01320	NM_017417		CLVS1
8146618	-1,25	-2,37	0,02498	-1,36	-2,56	0,00857	NM_173519		KRTAP19-4
8069836	-1,25	-2,37	0,00252	-0,89	-1,86	0,04527	NM_181610		KIAA1274
7928126	-1,25	-2,37	0,01284	0,10	1,07	0,86060	NM_014431		---
7934365	-1,25	-2,37	0,03613	-1,16	-2,24	0,00069	---	PKLR	
7920744	-1,25	-2,37	0,05752	-2,20	-4,59	0,00081	NM_000298		LOC391769
8111216	-1,25	-2,37	0,14161	-2,73	-6,65	0,00068	ENST00000426411		BEST4
7915598	-1,25	-2,37	0,00037	-1,03	-2,05	0,02455	NM_153274		HBB
7946033	-1,25	-2,37	0,02262	-1,72	-3,29	0,00304	NM_000518		LOC286359
8162674	-1,25	-2,37	0,06194	-0,63	-1,55	0,01301	NR_026847		CBFA2T3
8003425	-1,25	-2,37	0,02238	-1,00	-2,00	0,00062	NM_005187		---
8007259	-1,25	-2,37	0,30864	-1,29	-2,44	0,01309	---	TEX28	
8176018	-1,25	-2,37	0,03139	-1,93	-3,80	0,00116	NM_001586		PEBP4
8149725	-1,25	-2,37	0,00203	0,38	1,30	0,28839	NM_144962		ODAM
8095456	-1,25	-2,37	0,07244	-1,09	-2,13	0,00457	NM_017855		CORL2
8023184	-1,25	-2,37	0,06262	-2,15	-4,44	0,00051	AY669508	MIR192	
7949273	-1,25	-2,38	0,08819	-1,05	-2,08	0,09388	NR_029578		MEIS3
8037878	-1,25	-2,38	0,09411	-1,64	-3,12	0,00281	NM_020160		LMF2
8077082	-1,25	-2,38	0,00280	0,46	1,37	0,43877	NM_033200		GRIN3A
8162908	-1,25	-2,38	0,00044	-1,46	-2,76	0,00509	NM_133445		RBMY1A1
8176766	-1,25	-2,38	0,02529	-1,27	-2,42	0,00893	NM_005058		PASD1
8170488	-1,25	-2,38	0,04516	-0,82	-1,76	0,01854	NM_173493		RN28S1
7917645	-1,25	-2,38	0,02793	-3,12	-8,70	0,00084	NR_003287		---
8095137	-1,25	-2,38	0,02015	-1,53	-2,88	0,00151	---	---	
8122125	-1,25	-2,38	0,01216	-0,81	-1,75	0,00242	---	DTHD1	
8094533	-1,25	-2,38	0,01653	-1,37	-2,59	0,00394	NM_001136536		FLJ44896
8110676	-1,25	-2,38	0,06831	-1,15	-2,22	0,23726	AK126844	ANXA8L1	
7933312	-1,25	-2,38	0,11294	-1,19	-2,29	0,00095	NM_001098845		GABRQ
8170538	-1,25	-2,38	0,00906	1,02	2,03	0,04977	NM_018558		STMN1
7913864	-1,25	-2,38	0,00398	-0,60	-1,51	0,13498	NM_001145454		---
7948123	-1,25	-2,38	0,08994	-0,98	-1,98	0,06547	---	---	
7993112	-1,25	-2,38	0,00272	-1,64	-3,11	0,00017	---	C2CD4B	
7989473	-1,25	-2,38	0,07369	-1,45	-2,73	0,02946	NM_001007595		---
7985918	-1,25	-2,38	0,27681	-0,55	-1,47	0,12660	---	C5orf48	
8107702	-1,25	-2,38	0,00155	-0,74	-1,67	0,12878	BC130495	C14orf25	
7974008	-1,25	-2,38	0,01244	-0,39	-1,31	0,00138	BC038110	OR2H2	

8117741	-1,25	-2,38	0,05645	-2,04	-4,11	0,00061	NM_007160	RPL3L
7998643	-1,25	-2,38	0,18959	-2,48	-5,59	0,00091	NM_005061	ZIC5
7972567	-1,25	-2,38	0,00396	-1,17	-2,25	0,06982	NM_033132	CALML4
7989968	-1,25	-2,38	0,02622	-0,14	-1,10	0,75299	NM_033429	MRGPRD
7950003	-1,25	-2,38	0,00437	-1,94	-3,83	0,02686	NM_198923	---
8119527	-1,25	-2,38	0,00654	-0,81	-1,75	0,02079	---	---
8045319	-1,25	-2,38	0,15133	-2,58	-5,99	0,00072	---	---
8055234	-1,25	-2,38	0,15133	-2,58	-5,99	0,00072	---	---
7957495	-1,25	-2,38	0,00734	-2,35	-5,10	0,00073	---	---
8121159	-1,25	-2,38	0,14205	-0,63	-1,55	0,00272	---	---
8045347	-1,25	-2,38	0,06235	-0,91	-1,88	0,00282	---	FGFR4
8110265	-1,25	-2,38	0,01120	1,23	2,35	0,15960	NM_213647	---
8099240	-1,25	-2,38	0,07008	-2,48	-5,58	0,04799	---	---
7923162	-1,25	-2,38	0,00932	-1,49	-2,80	0,00658	---	VWA2
7930663	-1,25	-2,38	0,00305	-1,69	-3,22	0,00144	NM_198496	EIF5A
8004325	-1,25	-2,38	0,08150	-1,82	-3,53	0,01327	NM_001143760	TSPY1
8176484	-1,25	-2,38	0,05058	-1,36	-2,57	0,00011	NM_003308	STK32C
7937089	-1,25	-2,38	0,00688	0,39	1,31	0,23650	NM_173575	PNCK
8175815	-1,25	-2,38	0,01690	-1,75	-3,37	0,01022	NM_001039582	ANKRD30B
8020357	-1,25	-2,38	0,13503	-0,91	-1,88	0,00432	NM_001145029	HOXD9
8046542	-1,25	-2,38	0,03666	-1,10	-2,14	0,02502	NM_014213	LMO2
7947450	-1,25	-2,38	0,02054	-1,12	-2,17	0,01379	NM_005574	ZAP70
8043725	-1,25	-2,38	0,01090	-1,05	-2,07	0,02277	NM_001079	---
7902700	-1,25	-2,38	0,02023	-1,43	-2,69	0,00683	---	EFCAB4B
7960370	-1,25	-2,38	0,02822	-1,52	-2,88	0,02387	NM_032680	C15orf32
7986239	-1,25	-2,38	0,01001	-1,11	-2,16	0,00133	BC109252	LOC642864
7903561	-1,25	-2,38	0,00378	-1,20	-2,30	0,00813	AY312144	GPR139
7999916	-1,25	-2,38	0,11602	-1,52	-2,87	0,00480	NM_001002911	MTG1
7931607	-1,25	-2,38	0,03308	0,16	1,12	0,72683	NM_138384	USP17
8094130	-1,25	-2,38	0,48324	-3,40	-10,59	0,00025	NM_001105662	USH1C
7946903	-1,25	-2,38	0,01649	0,60	1,52	0,02023	NM_153676	RBPJL
8062948	-1,25	-2,38	0,02591	-1,08	-2,11	0,05884	NM_014276	---
8019875	-1,25	-2,39	0,00501	-1,44	-2,71	0,00530	---	CMKLR1
7966089	-1,25	-2,39	0,01404	-0,99	-1,99	0,12012	NM_001142343	TEX28
8176006	-1,25	-2,39	0,02282	-1,90	-3,72	0,00132	NM_001586	TEX28
8176012	-1,25	-2,39	0,02282	-1,90	-3,72	0,00132	NM_001586	SLC16A11
8012006	-1,25	-2,39	0,04124	-0,88	-1,85	0,07056	NM_153357	CD79A
8029136	-1,25	-2,39	0,00018	-0,39	-1,31	0,11894	NM_001783	FOXI2
7931348	-1,26	-2,39	0,01373	-1,60	-3,03	0,00158	NM_207426	TRIM63
7913907	-1,26	-2,39	0,02838	-0,73	-1,66	0,04856	NM_032588	---
8070712	-1,26	-2,39	0,00062	-1,39	-2,62	0,02425	---	KIAA0895L
8001946	-1,26	-2,39	0,05089	1,58	2,98	0,00602	NM_001040715	---
8006319	-1,26	-2,39	0,12986	-1,49	-2,80	0,15755	---	PMCHL2
8106019	-1,26	-2,39	0,00080	-1,62	-3,08	0,06636	NR_003922	---
8154283	-1,26	-2,39	0,00545	-1,30	-2,46	0,00335	---	LOC728800
7986336	-1,26	-2,39	0,03037	-1,42	-2,67	0,01116	ENST00000444787	TAAR9
8122127	-1,26	-2,39	0,00857	-1,48	-2,78	0,01506	NM_175057	C11orf35
7945445	-1,26	-2,39	0,16571	-1,17	-2,26	0,10229	NM_173573	CHRNA10
7945896	-1,26	-2,39	0,01351	-1,67	-3,18	0,01624	NM_020402	---
7981375	-1,26	-2,39	0,01537	-0,66	-1,58	0,02119	---	C8orf47
8147543	-1,26	-2,39	0,00163	-1,12	-2,17	0,02338	NM_173549	---
8045497	-1,26	-2,39	0,00004	-2,02	-4,04	0,00437	---	---
8090446	-1,26	-2,39	0,10962	-1,78	-3,44	0,00062	---	PLA2G6
8076025	-1,26	-2,39	0,00822	-1,08	-2,12	0,00122	NM_003560	LOC401296
8137802	-1,26	-2,39	0,06542	-1,68	-3,20	0,01289	ENST00000382528	GLP2R
8004922	-1,26	-2,39	0,00783	-0,89	-1,86	0,00211	NM_004246	WNK2
8156415	-1,26	-2,39	0,04383	-0,23	-1,17	0,61213	NM_006648	---
7935861	-1,26	-2,39	0,02664	-0,85	-1,80	0,00952	---	OR6B1
8136965	-1,26	-2,39	0,03821	-1,33	-2,52	0,05213	NM_001005281	ADAD2
8003145	-1,26	-2,39	0,14683	-0,68	-1,60	0,00328	NM_139174	---
8045585	-1,26	-2,39	0,00806	-0,87	-1,83	0,02520	---	C6orf168
8128371	-1,26	-2,39	0,01783	1,33	2,51	0,00054	NM_032511	HLA-DOA
8180093	-1,26	-2,39	0,02033	-2,46	-5,49	0,00043	NM_002119	TSPY1
8176517	-1,26	-2,39	0,07990	-1,16	-2,24	0,00042	NM_003308	---
8176566	-1,26	-2,39	0,19157	-2,70	-6,48	0,02091	---	RBMY1A1
8177331	-1,26	-2,39	0,02238	-1,27	-2,40	0,00376	NM_005058	FAM163A
7907769	-1,26	-2,39	0,08465	-2,00	-4,00	0,01872	NM_173509	COL5A1
8159142	-1,26	-2,39	0,05995	-2,03	-4,09	0,00061	NM_000093	SLC9A5
7996498	-1,26	-2,39	0,05987	0,73	1,66	0,05789	NM_004594	---
8122658	-1,26	-2,40	0,00435	-1,67	-3,18	0,00004	---	---
7974585	-1,26	-2,40	0,07917	-1,48	-2,80	0,02640	---	NR6A1
8164087	-1,26	-2,40	0,00280	1,62	3,07	0,00406	NM_033334	PITX2
8102352	-1,26	-2,40	0,03973	-1,37	-2,59	0,00002	NM_153426	---

8003859	-1,26	-2,40	0,16426	-2,53	-5,78	0,00001	---	PGBD5	
7924977	-1,26	-2,40	0,00183	-1,16	-2,24	0,00153	NM_024554		ACD
8002057	-1,26	-2,40	0,04341	0,27	1,21	0,48977	NM_001082486		---
7950269	-1,26	-2,40	0,05928	-0,69	-1,61	0,35207	---	SLC13A5	
8011975	-1,26	-2,40	0,04725	-1,19	-2,28	0,01232	NM_177550		RTN2
8037594	-1,26	-2,40	0,01797	-0,37	-1,29	0,08775	NM_005619		C11orf21
7945734	-1,26	-2,40	0,01920	-1,91	-3,76	0,01945	AB029488	SLC9A3	
8110688	-1,26	-2,40	0,00907	-0,56	-1,47	0,17696	NM_004174		FAM109B
8073573	-1,26	-2,40	0,07247	0,77	1,70	0,03126	NM_001002034		OR7E87P
7942277	-1,26	-2,40	0,21633	-2,11	-4,31	0,00096	ENST00000454336		DKFZP547L112
7981775	-1,26	-2,40	0,00848	-1,77	-3,41	0,00501	ENST00000435482		ARSF
8165843	-1,26	-2,40	0,05953	-0,82	-1,76	0,00490	NM_004042		NCF4
8072744	-1,26	-2,40	0,10560	-1,08	-2,11	0,00011	NM_013416		---
7925450	-1,26	-2,40	0,02378	-0,67	-1,59	0,00166	---	RAPGEF3	
7962623	-1,26	-2,40	0,06588	-1,60	-3,04	0,00128	NM_001098531		---
8124162	-1,26	-2,40	0,00151	-1,87	-3,66	0,00001	---	SPAG6	
7926622	-1,26	-2,40	0,00010	-1,05	-2,07	0,00318	NM_012443		---
8095301	-1,26	-2,40	0,00296	-1,04	-2,06	0,01935	---	---	
8043572	-1,26	-2,40	0,05240	-3,32	-10,02	0,00002	---	SDCBP2	
8064418	-1,26	-2,40	0,17370	-1,57	-2,97	0,02335	NM_080489		C11orf53
7943729	-1,26	-2,40	0,14832	-1,48	-2,78	0,08515	BC039669	SNORD59A	
7964248	-1,26	-2,40	0,02188	-0,63	-1,55	0,02136	NR_002737		CYP4F2
8035083	-1,26	-2,40	0,04785	-1,26	-2,40	0,00266	NM_001082		---
8150976	-1,26	-2,40	0,01106	-1,36	-2,57	0,01122	---	MDFI	
8119466	-1,26	-2,40	0,05947	-0,27	-1,21	0,44447	NM_005586		ITGB4
8009951	-1,26	-2,40	0,00774	2,59	6,01	0,00527	NM_000213		FBXO9
8127128	-1,26	-2,40	0,22441	-1,04	-2,05	0,09819	NM_033480		SRRM3
8133704	-1,26	-2,40	0,04444	-0,94	-1,93	0,01185	NM_001110199		TUBB8
7911355	-1,26	-2,40	0,11820	-2,19	-4,55	0,00283	NM_177987		LENEP
7905848	-1,26	-2,40	0,04648	-1,29	-2,44	0,10758	NM_018655		---
8154719	-1,26	-2,40	0,00527	-1,19	-2,28	0,02001	---	---	
8112310	-1,26	-2,40	0,15476	-0,52	-1,44	0,00773	---	NRG4	
7990555	-1,26	-2,40	0,01711	3,37	10,36	0,00010	NM_138573		---
8104996	-1,26	-2,40	0,00236	-0,96	-1,94	0,01773	---	TREH	
7952086	-1,26	-2,40	0,00306	-0,60	-1,52	0,02655	NM_007180		TNFRSF14
7911754	-1,26	-2,40	0,04290	-1,56	-2,94	0,00725	NM_003820		---
8060837	-1,26	-2,40	0,04488	-1,51	-2,85	0,00355	---	IFNK	
8154721	-1,26	-2,40	0,02989	-0,78	-1,72	0,04376	NM_020124		ZZEF1
8003846	-1,26	-2,40	0,02273	-0,57	-1,48	0,11891	NM_015113		CHST13
8082289	-1,27	-2,40	0,01139	-1,16	-2,23	0,00795	NM_152889		MT3
7995776	-1,27	-2,40	0,08688	-1,23	-2,35	0,03823	NM_005954		CDK5R1
8006409	-1,27	-2,40	0,01876	-0,57	-1,48	0,37195	NM_003885		KIAA0649
8159243	-1,27	-2,40	0,06805	-1,22	-2,33	0,05096	NM_014811		---
8139798	-1,27	-2,40	0,01172	-1,41	-2,65	0,00144	---	LOC732275	
8003230	-1,27	-2,40	0,12336	-1,79	-3,45	0,03474	NR_024406		MIR423
8006119	-1,27	-2,40	0,09795	-1,94	-3,83	0,04594	NR_029945		MYCNOS
8050423	-1,27	-2,40	0,11936	-1,20	-2,30	0,03179	NR_026766		PSRC1
7918300	-1,27	-2,41	0,00967	-0,07	-1,05	0,86773	NM_032636		CDH17
8151795	-1,27	-2,41	0,00831	-1,02	-2,03	0,00206	NM_004063		HTR1B
8127692	-1,27	-2,41	0,07964	-0,96	-1,94	0,00295	NM_000863		PRR20A
7969300	-1,27	-2,41	0,30445	-4,31	-19,87	0,00000	NM_198441		PRR20A
7969306	-1,27	-2,41	0,30445	-4,31	-19,87	0,00000	NM_198441		PRR20A
7969312	-1,27	-2,41	0,30445	-4,31	-19,87	0,00000	NM_198441		PRR20A
7969318	-1,27	-2,41	0,30445	-4,31	-19,87	0,00000	NM_198441		PRR20A
7969324	-1,27	-2,41	0,30445	-4,31	-19,87	0,00000	NM_198441		TNN
7907549	-1,27	-2,41	0,00926	-0,62	-1,53	0,05879	NM_022093		---
8054794	-1,27	-2,41	0,03488	-2,14	-4,42	0,01319	---	FLJ20464	
8072486	-1,27	-2,41	0,03204	-0,59	-1,51	0,17900	CR456348	C7orf50	
8137700	-1,27	-2,41	0,07603	-1,34	-2,53	0,09074	NM_032350		LOC100129476
7912133	-1,27	-2,41	0,12266	-1,53	-2,90	0,00187	ENST00000439923		---
8092081	-1,27	-2,41	0,01458	-0,74	-1,67	0,03972	---	---	
7900181	-1,27	-2,41	0,00827	-2,00	-3,99	0,00003	---	---	
8072573	-1,27	-2,41	0,05397	-1,48	-2,79	0,01547	---	---	
8005265	-1,27	-2,41	0,05577	-1,53	-2,89	0,00171	---	---	
7972173	-1,27	-2,41	0,00968	-1,37	-2,59	0,00577	---	DNAJC5B	
8146660	-1,27	-2,41	0,02284	-1,81	-3,51	0,00016	NM_033105		---
7996288	-1,27	-2,41	0,00031	-1,17	-2,25	0,00175	---	DPF1	
8036460	-1,27	-2,41	0,00080	0,41	1,33	0,26513	NM_004647		ERBB3
7956120	-1,27	-2,41	0,00201	2,17	4,51	0,00483	NM_001982		---
8097015	-1,27	-2,41	0,02883	-1,24	-2,37	0,02890	---	OR2B3	
8124632	-1,27	-2,41	0,00548	-1,89	-3,72	0,00373	NM_001005226		OR2B3
8178285	-1,27	-2,41	0,00548	-1,89	-3,72	0,00373	NM_001005226		OR2B3
8179589	-1,27	-2,41	0,00548	-1,89	-3,72	0,00373	NM_001005226		---

7936461	-1,27	-2,41	0,01102	-0,92	-1,89	0,00203	---	SLC6A20
8086540	-1,27	-2,41	0,09240	-0,66	-1,58	0,10040	NM_020208	LYPD6B
8045664	-1,27	-2,41	0,04346	0,14	1,10	0,76312	NM_177964	LOC645212
7988283	-1,27	-2,41	0,00382	0,00	1,00	0,99015	ENST00000313807	---
8117241	-1,27	-2,41	0,05970	-0,40	-1,32	0,14772	---	---
8127507	-1,27	-2,41	0,14625	-0,93	-1,90	0,00584	---	---
8013089	-1,27	-2,41	0,08475	-0,61	-1,53	0,12465	---	FCGR3A
7921868	-1,27	-2,41	0,00748	-1,07	-2,10	0,14155	NM_000569	CXorf27
8166745	-1,27	-2,41	0,03369	-1,03	-2,05	0,02017	NM_012274	ESPNP
7912854	-1,27	-2,41	0,04162	-2,23	-4,71	0,08037	NR_026567	PRR15
8132031	-1,27	-2,41	0,03187	-0,97	-1,95	0,17586	NM_175887	ADAMTS10
8033635	-1,27	-2,41	0,00060	0,37	1,29	0,50370	NM_030957	TCP10
8130787	-1,27	-2,41	0,02529	-1,21	-2,31	0,00004	NM_004610	C15orf62
7982810	-1,27	-2,41	0,07270	-0,04	-1,03	0,93156	NM_001130448	---
7980401	-1,27	-2,41	0,10826	-0,48	-1,40	0,11442	---	ZNF768
8000899	-1,27	-2,41	0,02033	0,56	1,47	0,35233	NM_024671	HUNK
8068180	-1,27	-2,41	0,01612	-0,61	-1,53	0,03154	NM_014586	OLIG2
8068231	-1,27	-2,41	0,00279	-1,06	-2,08	0,00237	NM_005806	SLC22A9
7940749	-1,27	-2,41	0,08273	-1,06	-2,09	0,00713	NM_080866	CCDC85C
7981266	-1,27	-2,41	0,10799	-0,25	-1,19	0,63615	NM_001144995	ANKRD18A
8161270	-1,27	-2,41	0,00225	1,29	2,44	0,02218	NM_147195	---
7904164	-1,27	-2,41	0,01809	-1,25	-2,39	0,00467	---	NRIP2
7960331	-1,27	-2,41	0,09526	-1,22	-2,32	0,13726	NM_031474	SPACA4
8030076	-1,27	-2,41	0,03221	-0,65	-1,57	0,03855	NM_133498	NFYC
7915345	-1,27	-2,41	0,01866	-0,93	-1,91	0,20658	NM_001142588	C8orf30A
8148799	-1,27	-2,41	0,01973	-0,07	-1,05	0,88956	NM_016458	---
8048368	-1,27	-2,41	0,04795	-1,09	-2,13	0,00117	---	PF4V1
8095694	-1,27	-2,41	0,06121	-1,28	-2,43	0,03910	NM_002620	---
8166945	-1,27	-2,41	0,14192	-2,25	-4,75	0,00083	---	LOC100129726
8041640	-1,27	-2,41	0,04240	-0,76	-1,69	0,08432	NR_027251	HRASLS5
7948973	-1,27	-2,42	0,00992	-2,22	-4,66	0,00032	NM_054108	ODF3
7937263	-1,27	-2,42	0,01119	-1,21	-2,32	0,00075	NM_053280	---
7986501	-1,27	-2,42	0,01918	-1,95	-3,86	0,03580	---	KLHDC8A
7923762	-1,27	-2,42	0,00301	-1,13	-2,19	0,00879	NM_018203	C1QTNF4
7947928	-1,27	-2,42	0,01535	-1,70	-3,25	0,00696	NM_031909	FGFBP3
7935006	-1,27	-2,42	0,04838	-1,95	-3,86	0,00305	NM_152429	LRFN2
8126244	-1,27	-2,42	0,01950	-0,64	-1,56	0,27126	NM_020737	ZAR1
8094968	-1,27	-2,42	0,02247	-1,09	-2,12	0,04475	NM_175619	TNP2
7999427	-1,27	-2,42	0,10111	-1,29	-2,45	0,00808	NM_005425	SULT1B1
8100798	-1,27	-2,42	0,03311	-0,96	-1,95	0,02271	NM_014465	C2orf86
8052620	-1,27	-2,42	0,03203	-0,63	-1,55	0,00340	ENST00000448143	---
8158404	-1,27	-2,42	0,01663	-1,64	-3,11	0,00077	---	DEFB118
8061509	-1,27	-2,42	0,08171	-2,09	-4,26	0,01172	NM_054112	---
8090637	-1,27	-2,42	0,30807	-3,44	-10,84	0,00240	---	CTLA4
8047692	-1,27	-2,42	0,00711	-1,27	-2,40	0,02051	NM_005214	---
8013507	-1,27	-2,42	0,18301	-1,15	-2,23	0,09523	---	IGF2AS
7937774	-1,27	-2,42	0,03781	-2,18	-4,54	0,00329	NR_028044	ZNF764
8000906	-1,27	-2,42	0,22131	-1,20	-2,29	0,23025	NM_033410	TMEM102
8004404	-1,27	-2,42	0,11109	-1,22	-2,34	0,00776	NM_178518	SULT2A1
8037949	-1,27	-2,42	0,00715	-1,24	-2,37	0,05013	NM_003167	LOC113230
8026294	-1,27	-2,42	0,02518	0,00	1,00	0,99471	NR_024282	CFP
8172333	-1,27	-2,42	0,13045	-1,91	-3,77	0,01339	NM_002621	---
7925587	-1,28	-2,42	0,03144	-1,89	-3,70	0,09594	---	SLC17A7
8038367	-1,28	-2,42	0,06631	-0,65	-1,57	0,25107	NM_020309	---
7917514	-1,28	-2,42	0,00048	-1,55	-2,94	0,00244	---	PCDH8
7971850	-1,28	-2,42	0,02845	-1,86	-3,62	0,00032	NM_002590	PTCH2
7915614	-1,28	-2,42	0,06724	-0,80	-1,75	0,02576	NM_003738	ZG16
8003578	-1,28	-2,42	0,01839	-1,31	-2,49	0,00076	NM_152338	OR2T12
7925743	-1,28	-2,42	0,21676	-3,01	-8,06	0,00021	NM_001004692	RAB11FIP4
8006298	-1,28	-2,42	0,00207	0,31	1,24	0,40944	NM_032932	---
7915198	-1,28	-2,42	0,01341	-1,53	-2,88	0,00825	---	NKD1
7995525	-1,28	-2,42	0,00635	0,13	1,09	0,70768	NM_033119	CTAG1A
8170965	-1,28	-2,42	0,05299	-0,77	-1,71	0,02058	NM_139250	CTAG1A
8176149	-1,28	-2,42	0,05299	-0,77	-1,71	0,02058	NM_139250	---
7927643	-1,28	-2,42	0,10831	-1,53	-2,88	0,00314	---	OR4D5
7944795	-1,28	-2,42	0,00668	-1,05	-2,06	0,00276	NM_001001965	HSCB
8072143	-1,28	-2,42	0,01181	-0,30	-1,23	0,46083	NM_172002	COMTD1
7934544	-1,28	-2,42	0,01942	-1,12	-2,18	0,03893	NM_144589	KRT74
7963448	-1,28	-2,42	0,00614	-1,21	-2,32	0,03654	NM_175053	XRCC3
7981447	-1,28	-2,42	0,00015	-0,57	-1,48	0,10410	NM_001100119	GP1BB
8071268	-1,28	-2,42	0,00314	-0,72	-1,64	0,13417	NM_000407	---
7955448	-1,28	-2,42	0,03507	-1,03	-2,04	0,12103	---	GPR111
8120124	-1,28	-2,42	0,01330	-1,54	-2,90	0,00069	NM_153839	FAM90A20

8144388	-1,28	-2,42	0,30080	-2,54	-5,81	0,00173	NM_001164453	PLUNC
8061883	-1,28	-2,42	0,01763	-1,16	-2,24	0,08032	NM_130852	NBPF4
7918203	-1,28	-2,42	0,01489	-1,63	-3,10	0,00025	NM_001143989	UROC1
8090366	-1,28	-2,42	0,05879	-0,68	-1,61	0,13389	NM_144639	PRAC
8016484	-1,28	-2,43	0,01351	-1,35	-2,56	0,00065	NM_032391	---
8072482	-1,28	-2,43	0,01581	-1,04	-2,06	0,05279	---	---
8080894	-1,28	-2,43	0,00348	-1,76	-3,38	0,00003	---	---
8110668	-1,28	-2,43	0,03660	-1,49	-2,81	0,03894	---	LRRC56
7937391	-1,28	-2,43	0,05366	-1,11	-2,16	0,00641	NM_198075	C1orf89
7912743	-1,28	-2,43	0,01772	-1,03	-2,04	0,09098	NM_030907	GAPT
8105481	-1,28	-2,43	0,00839	-1,66	-3,16	0,00163	NM_152687	PABPC1L2B
8168393	-1,28	-2,43	0,04020	-1,01	-2,02	0,01170	NM_001042506	---
8055641	-1,28	-2,43	0,02584	-0,76	-1,69	0,01246	---	NEK8
8005959	-1,28	-2,43	0,02266	-0,46	-1,37	0,07453	NM_178170	AJAP1
7897227	-1,28	-2,43	0,00528	-0,76	-1,69	0,13682	NM_018836	KCNQ2
8067626	-1,28	-2,43	0,00076	-1,21	-2,31	0,00252	NM_172107	CTRB2
8002845	-1,28	-2,43	0,07027	-1,98	-3,94	0,00076	NM_001025200	C12orf61
7964642	-1,28	-2,43	0,01448	-1,63	-3,09	0,00672	NM_175895	MIR188
8167623	-1,28	-2,43	0,01582	-1,77	-3,41	0,00235	NR_029708	C20orf195
8064108	-1,28	-2,43	0,03018	-1,46	-2,76	0,04328	BC000912	CHST6
8002882	-1,28	-2,43	0,03077	1,34	2,52	0,03215	NM_021615	---
8062685	-1,28	-2,43	0,00150	-1,23	-2,35	0,00502	---	MYBPC2
8030677	-1,28	-2,43	0,00655	-0,93	-1,90	0,03457	NM_004533	BASE
8061859	-1,28	-2,43	0,03252	-1,21	-2,32	0,01495	NR_026760	MIR191
8087252	-1,28	-2,43	0,01474	-1,22	-2,32	0,08235	NR_029690	---
7987634	-1,28	-2,43	0,15022	-1,03	-2,04	0,00648	---	---
8056045	-1,28	-2,43	0,03639	-0,81	-1,75	0,14188	---	---
7954995	-1,28	-2,43	0,05839	-1,09	-2,13	0,02238	---	---
7965767	-1,28	-2,43	0,04451	-0,73	-1,66	0,08429	---	PM20D1
7923837	-1,28	-2,43	0,03245	-1,76	-3,38	0,00389	NM_152491	DEFB130
8149353	-1,28	-2,43	0,11108	-1,30	-2,47	0,04009	NM_001037804	DEFB130
8149377	-1,28	-2,43	0,11108	-1,30	-2,47	0,04009	NM_001037804	ZBTB16
7943984	-1,28	-2,43	0,01691	-1,57	-2,97	0,02602	NM_006006	SFN
7899265	-1,28	-2,43	0,08646	1,12	2,17	0,03005	NM_006142	C6orf54
8130827	-1,28	-2,43	0,05201	-2,03	-4,07	0,01281	AB016899	DQX1
8053200	-1,28	-2,43	0,05501	-0,63	-1,55	0,46421	NM_133637	ZRSR1
8107353	-1,28	-2,43	0,27618	-0,92	-1,89	0,12061	BC104811	ELP2P
8003619	-1,28	-2,43	0,11172	-0,71	-1,63	0,03081	NR_024120	HIF3A
8029784	-1,28	-2,43	0,02077	-1,39	-2,63	0,01987	NM_152794	---
8083994	-1,28	-2,43	0,02787	-0,77	-1,71	0,01810	---	ERF
8037166	-1,28	-2,43	0,00579	-0,57	-1,48	0,02011	NM_006494	HLX
7909890	-1,28	-2,43	0,00254	-1,13	-2,19	0,07990	NM_021958	---
7948159	-1,28	-2,43	0,07482	-1,30	-2,46	0,00107	---	NPPA
7912515	-1,28	-2,43	0,14651	-2,10	-4,29	0,00186	NM_006172	HILS1
8016640	-1,28	-2,43	0,01463	-1,89	-3,71	0,00717	NR_024193	---
8021342	-1,28	-2,43	0,07579	-0,66	-1,58	0,07209	---	---
7977653	-1,28	-2,43	0,00044	-1,22	-2,33	0,00000	---	---
8042219	-1,28	-2,44	0,00113	-1,27	-2,41	0,03002	---	EFHD1
8049187	-1,28	-2,44	0,14291	0,22	1,17	0,76989	NM_025202	SIGLEC16
8030563	-1,28	-2,44	0,11324	-2,23	-4,69	0,07756	NR_002825	SDF2L1
8071559	-1,28	-2,44	0,04739	1,56	2,95	0,05597	NM_022044	---
8134372	-1,28	-2,44	0,00382	-0,74	-1,67	0,00063	---	---
8119336	-1,28	-2,44	0,02541	-1,55	-2,92	0,00732	---	TRIM60
8098170	-1,28	-2,44	0,02384	-0,93	-1,91	0,02389	NM_152620	MIR26A1
8078663	-1,28	-2,44	0,03423	-0,94	-1,92	0,00655	NR_029499	CST9
8065400	-1,28	-2,44	0,04814	-1,55	-2,93	0,00049	NM_001008693	FAM90A13
8149204	-1,29	-2,44	0,31689	-2,37	-5,16	0,00587	NM_001164456	---
8149065	-1,29	-2,44	0,01371	-1,12	-2,18	0,10152	---	C18orf16
8020717	-1,29	-2,44	0,18666	-1,95	-3,87	0,08245	AK055069	HRC
8038319	-1,29	-2,44	0,01045	-1,68	-3,20	0,01752	NM_002152	TTTY14
8177217	-1,29	-2,44	0,02661	-1,34	-2,53	0,00396	NR_001543	---
7909888	-1,29	-2,44	0,05423	-1,00	-2,00	0,03394	---	CALY
7937200	-1,29	-2,44	0,00624	-2,10	-4,28	0,00251	NM_015722	FLJ45340
8031992	-1,29	-2,44	0,34377	-2,77	-6,80	0,00062	NR_024368	SPSB3
7998590	-1,29	-2,44	0,03808	-1,76	-3,40	0,00156	NM_080861	C16orf42
7998449	-1,29	-2,44	0,01016	0,31	1,24	0,33251	NM_001001410	---
7997372	-1,29	-2,44	0,08166	-1,37	-2,58	0,00213	---	RNF126P1
8008662	-1,29	-2,44	0,09414	-1,47	-2,77	0,00511	NR_002818	SLC19A1
8070912	-1,29	-2,44	0,00120	0,80	1,74	0,13655	NM_194255	GABRB2
8115651	-1,29	-2,44	0,00142	-1,61	-3,06	0,00304	NM_021911	TRIM54
8040898	-1,29	-2,44	0,01115	0,16	1,11	0,39741	NM_032546	C19orf33
8028325	-1,29	-2,44	0,05704	1,62	3,07	0,01064	NM_033520	---
8137081	-1,29	-2,44	0,02068	-1,74	-3,34	0,00086	---	---

8065370	-1,29	-2,44	0,02197	-0,88	-1,84	0,13387	---	---	
7965675	-1,29	-2,44	0,03063	-1,25	-2,38	0,05630	---	DENND2C	
7918753	-1,29	-2,44	0,00004	-0,41	-1,33	0,00173	BC063894	---	
7966686	-1,29	-2,44	0,01649	-2,10	-4,28	0,00415	---	UNC93B1	
8094144	-1,29	-2,44	0,13084	-0,42	-1,34	0,61124	NM_030930		---
8078960	-1,29	-2,44	0,02316	-1,12	-2,17	0,00972	---	RNF222	
8012473	-1,29	-2,44	0,14159	-1,73	-3,32	0,02669	NM_001146684		COASY
8007272	-1,29	-2,44	0,05526	0,82	1,77	0,12508	NM_025233		---
7973972	-1,29	-2,44	0,00491	0,33	1,26	0,63529	---	PRAMEF18	
7912601	-1,29	-2,44	0,04793	-1,11	-2,16	0,00045	NM_001099850		PRAMEF18
7912617	-1,29	-2,44	0,04793	-1,11	-2,16	0,00045	NM_001099850		LRDD
7945550	-1,29	-2,44	0,14739	-0,89	-1,85	0,12265	NM_018494		KCNA7
8038309	-1,29	-2,44	0,03417	-1,17	-2,26	0,05057	NM_031886		---
7991425	-1,29	-2,44	0,02510	-2,17	-4,49	0,00015	---	MAP7D1	
7900095	-1,29	-2,44	0,04838	-0,70	-1,63	0,15075	NM_018067		---
7983254	-1,29	-2,44	0,00024	-1,04	-2,05	0,05255	---	---	
7983272	-1,29	-2,44	0,00024	-1,04	-2,05	0,05255	---	PMCHL2	
8177554	-1,29	-2,44	0,00106	-1,65	-3,15	0,06554	NR_003922		---
8047503	-1,29	-2,44	0,07953	-2,29	-4,87	0,00754	---	TSPY1	
8176544	-1,29	-2,44	0,05860	-1,28	-2,43	0,00032	NM_003308		---
8065323	-1,29	-2,44	0,01032	-0,77	-1,71	0,04447	---	---	
8046755	-1,29	-2,44	0,14494	-2,24	-4,74	0,00326	---	OR52I2	
7937946	-1,29	-2,45	0,01426	-0,92	-1,89	0,07606	NM_001005170		LOC100132169
7991731	-1,29	-2,45	0,03616	0,27	1,21	0,61888	BC032901	---	
7984660	-1,29	-2,45	0,12121	-2,56	-5,89	0,00741	---	GRK6	
8110362	-1,29	-2,45	0,03766	-0,05	-1,03	0,93935	NM_001004106		---
8176933	-1,29	-2,45	0,01782	-0,38	-1,30	0,25058	---	---	
8177401	-1,29	-2,45	0,01782	-0,38	-1,30	0,25058	---	BCR	
8071516	-1,29	-2,45	0,05285	-1,42	-2,68	0,01359	NM_004327		DRD1
8115875	-1,29	-2,45	0,01504	-1,08	-2,12	0,00129	NM_000794		SNRNP70
8030199	-1,29	-2,45	0,05948	-0,82	-1,77	0,13277	NM_003089		SLC27A1
8026763	-1,29	-2,45	0,00874	-1,18	-2,26	0,00776	NM_198580		CSF2RA
8165735	-1,29	-2,45	0,01289	0,11	1,08	0,47740	NM_001161531		CSF2RA
8176306	-1,29	-2,45	0,01289	0,11	1,08	0,47740	NM_001161531		---
8143788	-1,29	-2,45	0,04053	-1,12	-2,17	0,12137	---	DEFB125	
8060303	-1,29	-2,45	0,01672	-0,97	-1,96	0,06332	NM_153325		MB
8075659	-1,29	-2,45	0,01484	-0,24	-1,18	0,62469	NM_203377		LCE3C
7905490	-1,29	-2,45	0,00240	-0,72	-1,64	0,03527	NM_178434		---
7989311	-1,29	-2,45	0,03203	-1,55	-2,92	0,01888	---	---	
8029726	-1,29	-2,45	0,04360	-1,22	-2,33	0,00547	---	TWIST1	
8138442	-1,29	-2,45	0,02790	-1,93	-3,81	0,00713	NM_000474		ADD2
8052882	-1,29	-2,45	0,01896	3,28	9,73	0,00031	NM_017488		LPPR1
8156923	-1,29	-2,45	0,07893	-1,30	-2,45	0,00002	NM_207299		RUNDC2A
7993274	-1,29	-2,45	0,00720	-0,19	-1,14	0,31623	NM_032167		ADIPOQ
8084710	-1,29	-2,45	0,02159	-2,31	-4,97	0,00018	NM_001177800		MIR105-1
8175706	-1,29	-2,45	0,00088	-0,85	-1,81	0,02252	NR_029521		---
7957534	-1,29	-2,45	0,11213	-1,28	-2,43	0,00083	---	ZNF771	
7994817	-1,29	-2,45	0,03692	-1,10	-2,15	0,08930	NM_016643		XKR6
8149307	-1,29	-2,45	0,02806	-1,45	-2,72	0,02244	NM_173683		C8orf56
8152252	-1,29	-2,45	0,08911	-0,69	-1,61	0,01993	NR_027071		LOC100131943
8005659	-1,29	-2,45	0,00331	-0,99	-1,99	0,01719	AY358202	SYT8	
7937713	-1,29	-2,45	0,00335	-1,24	-2,36	0,00936	NM_138567		PUF60
8153536	-1,29	-2,45	0,02339	0,60	1,51	0,54801	NM_001136033		---
8091097	-1,29	-2,45	0,00258	-1,11	-2,17	0,09074	---	CACNG1	
8009343	-1,29	-2,45	0,03201	-1,59	-3,02	0,01426	NM_000727		YSK4
8055361	-1,29	-2,45	0,02252	-1,49	-2,81	0,00286	NM_025052		SNORA80
8069985	-1,29	-2,45	0,08925	-1,83	-3,57	0,00229	NR_002996		PRKCZ
7897044	-1,29	-2,45	0,00620	1,79	3,46	0,00074	NM_002744		GATM
7988414	-1,29	-2,45	0,02193	4,62	24,52	0,00048	NM_001482		---
8026383	-1,29	-2,45	0,01145	-1,85	-3,60	0,00301	---	---	
7959891	-1,30	-2,45	0,24191	-3,06	-8,32	0,00105	---	TFF2	
8070574	-1,30	-2,45	0,03603	-1,05	-2,07	0,04851	NM_005423		CCL24
8140367	-1,30	-2,46	0,00280	-0,75	-1,68	0,02748	NM_002991		CXXC4
8102135	-1,30	-2,46	0,05461	-1,66	-3,15	0,04227	NM_025212		---
7946027	-1,30	-2,46	0,07186	-1,97	-3,92	0,04776	---	---	
7970031	-1,30	-2,46	0,07901	-1,09	-2,12	0,00165	---	FAM128B	
8045146	-1,30	-2,46	0,06963	-0,27	-1,21	0,24008	AK024408	---	
7992140	-1,30	-2,46	0,02217	-1,57	-2,96	0,04704	---	ZNF552	
8039687	-1,30	-2,46	0,07265	-1,45	-2,73	0,00141	NM_024762		C2orf48
8040231	-1,30	-2,46	0,00246	-0,43	-1,35	0,11067	BC093999	GFOD1	
8123976	-1,30	-2,46	0,00758	-0,89	-1,86	0,09914	NM_018988		---
8056361	-1,30	-2,46	0,03306	-1,55	-2,92	0,07189	---	SOX3	
8175528	-1,30	-2,46	0,06287	-2,31	-4,97	0,00216	NM_005634		---

7934070	-1,30	-2,46	0,00040	-1,38	-2,60	0,00399	---	---
8127752	-1,30	-2,46	0,15627	-1,53	-2,89	0,00155	---	DSCR10
8068578	-1,30	-2,46	0,04697	-1,03	-2,04	0,00066	NR_027695	ProSAPiP1
8064591	-1,30	-2,46	0,01772	-1,63	-3,09	0,00591	NM_014731	GRIN2D
8030022	-1,30	-2,46	0,01650	-0,89	-1,85	0,12606	NM_000836	PPP1R1B
8006865	-1,30	-2,46	0,02243	-1,79	-3,46	0,00059	NM_032192	LOC100130581
8015802	-1,30	-2,46	0,04065	-1,29	-2,45	0,02197	AK127427	LOC645188
8170257	-1,30	-2,46	0,05910	-1,34	-2,53	0,00006	BC042039	MIR124-3
8064029	-1,30	-2,46	0,01048	-1,90	-3,72	0,00991	NR_029670	C9orf16
8158177	-1,30	-2,46	0,04808	-1,80	-3,48	0,00242	NM_024112	CELA1
7963304	-1,30	-2,46	0,04227	-1,50	-2,82	0,03188	NM_001971	ZNF852
8079187	-1,30	-2,46	0,01698	-0,87	-1,83	0,01991	AK296954	LINGO1
7990643	-1,30	-2,46	0,05459	-1,34	-2,54	0,00210	NM_032808	PBOV1
8129876	-1,30	-2,46	0,00046	-1,20	-2,30	0,05769	NM_021635	DKFZP434K028
7948599	-1,30	-2,46	0,01473	-1,34	-2,53	0,00137	ENST00000244906	C21orf49
8068220	-1,30	-2,46	0,08587	-1,24	-2,36	0,00810	NR_024622	OR11A1
8178291	-1,30	-2,46	0,01904	-1,01	-2,01	0,07108	NM_013937	OR11A1
8179591	-1,30	-2,46	0,01904	-1,01	-2,01	0,07108	NM_013937	C15orf63
7983298	-1,30	-2,46	0,01805	-0,16	-1,12	0,48480	NM_016400	CDHR4
8087481	-1,30	-2,46	0,00175	-0,71	-1,64	0,00385	NM_001007540	---
7988603	-1,30	-2,46	0,02186	-0,84	-1,79	0,01079	---	MATK
8032682	-1,30	-2,46	0,01256	-0,96	-1,95	0,00848	NM_139355	ARMC3
7926638	-1,30	-2,46	0,02838	-0,34	-1,27	0,08476	NM_173081	TNNT1
8039340	-1,30	-2,46	0,25346	0,42	1,33	0,09246	NM_003283	ZNF219
7977646	-1,30	-2,46	0,02088	-0,55	-1,47	0,37536	NM_016423	---
8023906	-1,30	-2,46	0,08491	-0,84	-1,79	0,00065	---	CEACAM20
8037482	-1,30	-2,46	0,00001	-0,65	-1,57	0,15194	NM_001102597	NPAS1
8029870	-1,30	-2,46	0,02069	-1,42	-2,67	0,00092	NM_002517	PAK7
8064952	-1,30	-2,46	0,04660	-1,29	-2,44	0,01114	NM_020341	RFPL2
8075564	-1,30	-2,46	0,13364	-1,03	-2,05	0,00906	NM_001098527	MED22
8164872	-1,30	-2,46	0,01754	0,75	1,68	0,32464	NM_181491	NT5M
8005247	-1,30	-2,46	0,02501	-1,08	-2,11	0,01827	NM_020201	C2orf53
8051024	-1,30	-2,47	0,01722	-1,86	-3,64	0,00054	NM_178553	---
8148327	-1,30	-2,47	0,01640	-1,59	-3,01	0,00182	---	APLN
8175016	-1,30	-2,47	0,09470	-0,76	-1,69	0,14167	NM_017413	C17orf74
8004400	-1,30	-2,47	0,05936	-1,27	-2,41	0,00265	NM_175734	DCAF12L1
8174972	-1,30	-2,47	0,01278	-2,06	-4,17	0,00001	NM_178470	C10orf140
7932498	-1,30	-2,47	0,02672	-0,73	-1,66	0,01143	NM_207371	---
8167953	-1,30	-2,47	0,00053	-1,18	-2,27	0,05300	---	IRF7
7945462	-1,30	-2,47	0,06955	-0,66	-1,58	0,14274	NM_004031	---
7898607	-1,30	-2,47	0,02319	-0,99	-1,99	0,00413	---	---
8159917	-1,30	-2,47	0,05299	-1,06	-2,08	0,00327	---	ODF3L1
7985025	-1,30	-2,47	0,00884	-2,17	-4,50	0,00954	NM_175881	---
7952599	-1,30	-2,47	0,05947	-1,56	-2,95	0,00017	---	ECEL1P2
8059744	-1,30	-2,47	0,09811	-1,34	-2,53	0,02387	NR_028501	LGALS8
7925361	-1,30	-2,47	0,01589	-1,16	-2,23	0,04631	NM_006499	---
8117138	-1,30	-2,47	0,13516	-1,62	-3,07	0,01597	---	---
8113232	-1,30	-2,47	0,07477	-0,98	-1,97	0,02808	---	---
7925607	-1,30	-2,47	0,01703	-1,18	-2,27	0,00110	---	PDE2A
7950162	-1,30	-2,47	0,03277	-0,56	-1,47	0,01755	NM_002599	LOC643837
7896759	-1,30	-2,47	0,04986	0,70	1,62	0,30521	AK096570	GP2
7999920	-1,30	-2,47	0,00358	-0,97	-1,96	0,01015	NM_001007240	DFNB31
8163599	-1,30	-2,47	0,01058	-0,45	-1,36	0,43310	NM_015404	HOXD8
8046546	-1,30	-2,47	0,03441	-0,73	-1,66	0,02701	NM_019558	RAET1L
8130173	-1,30	-2,47	0,01616	0,38	1,30	0,56755	NM_130900	TYRO3
7982938	-1,30	-2,47	0,01962	1,93	3,80	0,02001	NM_006293	CNKSR1
7899101	-1,30	-2,47	0,03545	2,42	5,35	0,00221	NM_006314	PRAM1
8033587	-1,31	-2,47	0,03100	-1,96	-3,90	0,00003	NM_032152	CEACAM6
8029098	-1,31	-2,47	0,07816	0,43	1,34	0,23357	NM_002483	---
8100476	-1,31	-2,47	0,03363	-1,09	-2,13	0,04702	---	DLL3
8028719	-1,31	-2,47	0,03415	-0,54	-1,46	0,04208	NM_016941	HORMAD2
8072304	-1,31	-2,47	0,03452	-0,09	-1,07	0,77359	NM_152510	---
8129761	-1,31	-2,47	0,04050	-1,28	-2,43	0,00262	---	CLVS2
8121803	-1,31	-2,47	0,01354	-1,85	-3,62	0,00239	NM_001010852	CTAGE1
8022508	-1,31	-2,47	0,05850	-1,04	-2,05	0,01534	NM_172241	MEGF6
7911799	-1,31	-2,47	0,01907	-1,05	-2,06	0,11644	NM_001409	PHLDA3
7923372	-1,31	-2,47	0,00594	-1,06	-2,09	0,04822	NM_012396	SPDYC
7941172	-1,31	-2,47	0,04892	-1,61	-3,06	0,00151	NM_001008778	FLJ27255
8130553	-1,31	-2,47	0,06413	-1,44	-2,71	0,06133	AK130765	---
7928815	-1,31	-2,47	0,01628	-1,64	-3,12	0,00080	---	KIR2DS2
8039842	-1,31	-2,48	0,09745	-2,43	-5,38	0,00058	NM_012312	---
7947228	-1,31	-2,48	0,00351	-1,14	-2,21	0,01479	---	FZD8
7933075	-1,31	-2,48	0,02587	-0,91	-1,88	0,03310	NM_031866	---

7965671	-1,31	-2,48	0,00249	-1,51	-2,85	0,00434	---	---
8175591	-1,31	-2,48	0,03967	-0,79	-1,73	0,01668	---	NPSR1
8132270	-1,31	-2,48	0,05358	-1,39	-2,62	0,01115	NM_207172	PSD4
8044584	-1,31	-2,48	0,01307	-0,57	-1,48	0,13509	NM_012455	---
7972748	-1,31	-2,48	0,17819	-1,38	-2,60	0,02541	---	DUOX1
7983413	-1,31	-2,48	0,05635	0,12	1,08	0,77734	NM_017434	CRLF1
8035506	-1,31	-2,48	0,05949	-0,97	-1,96	0,10351	NM_004750	---
7919556	-1,31	-2,48	0,09227	-1,89	-3,72	0,00014	---	CDK5R2
8048460	-1,31	-2,48	0,01132	-0,70	-1,63	0,26599	NM_003936	DEAF1
7945503	-1,31	-2,48	0,00467	0,01	1,01	0,97296	NM_021008	---
8022745	-1,31	-2,48	0,03108	-1,62	-3,07	0,00839	---	MAPK8IP1
7939613	-1,31	-2,48	0,03665	-0,31	-1,24	0,62899	NM_005456	TNFSF9
8025053	-1,31	-2,48	0,01039	1,40	2,63	0,18017	NM_003811	NKAIN1
7914326	-1,31	-2,48	0,02700	-0,19	-1,14	0,77251	NM_024522	PP13004
8132302	-1,31	-2,48	0,09977	-1,48	-2,79	0,13855	ENST00000381493	C20orf151
8067506	-1,31	-2,48	0,02390	-1,32	-2,49	0,00046	NM_080833	CDH4
8063796	-1,31	-2,48	0,06764	-0,74	-1,66	0,00256	NM_001794	---
8105479	-1,31	-2,48	0,11406	-1,45	-2,74	0,00293	---	---
8134403	-1,31	-2,48	0,34085	-1,83	-3,56	0,06412	---	CELF4
8022952	-1,31	-2,48	0,05343	-0,86	-1,82	0,04464	NM_020180	KIF7
7991296	-1,31	-2,48	0,00113	-0,50	-1,41	0,05694	NM_198525	---
8065194	-1,31	-2,48	0,09607	-2,02	-4,06	0,00638	---	MPST
8072777	-1,31	-2,48	0,01744	-1,12	-2,17	0,04843	NR_024038	UBE2J2
7911444	-1,31	-2,48	0,16719	-0,47	-1,39	0,07936	NM_194458	---
7901334	-1,31	-2,48	0,04580	-1,19	-2,28	0,00104	---	FATE1
8170513	-1,31	-2,48	0,00593	-1,14	-2,20	0,00161	NM_033085	C5AR1
8029907	-1,31	-2,48	0,01338	0,13	1,09	0,40755	NM_001736	CT47B1
8174831	-1,31	-2,48	0,10844	-1,64	-3,12	0,02949	NM_001145718	HOXB9
8016476	-1,31	-2,48	0,12035	0,77	1,70	0,28221	NM_024017	ARID3C
8160849	-1,31	-2,48	0,00920	-1,44	-2,71	0,00233	NM_001017363	---
7905077	-1,31	-2,48	0,01821	0,22	1,17	0,64898	---	---
8124082	-1,31	-2,48	0,16283	-1,02	-2,02	0,00438	---	LOC100133746
7990827	-1,31	-2,48	0,01094	-1,03	-2,04	0,01060	ENST00000447682	C20orf203
8065630	-1,31	-2,48	0,20307	-1,70	-3,26	0,01285	NM_182584	LCAT
8002143	-1,31	-2,48	0,00502	-0,85	-1,80	0,15163	NM_000229	---
8140737	-1,31	-2,48	0,25837	-3,45	-10,94	0,00088	---	---
8166712	-1,31	-2,48	0,05675	-1,66	-3,15	0,00855	---	LOC100288144
7981488	-1,31	-2,48	0,10139	-2,58	-5,99	0,01365	AF462446	---
8142661	-1,31	-2,48	0,05781	-0,90	-1,86	0,01820	---	SYT13
7947590	-1,31	-2,48	0,00380	-0,85	-1,80	0,02092	NM_020826	ITLN1
7921690	-1,31	-2,48	0,08539	-1,02	-2,02	0,00901	NM_017625	APOBEC4
7922804	-1,31	-2,48	0,00498	-1,47	-2,77	0,00255	NM_203454	---
8063024	-1,31	-2,49	0,01675	-1,19	-2,28	0,00053	---	---
8086368	-1,31	-2,49	0,02940	-1,75	-3,35	0,00950	---	---
8112519	-1,31	-2,49	0,33838	-3,77	-13,68	0,00243	---	---
8066962	-1,31	-2,49	0,01401	-1,34	-2,54	0,04279	---	LOC728690
7911657	-1,31	-2,49	0,05263	-0,66	-1,58	0,01234	AK094642	OR10R2
7906367	-1,31	-2,49	0,09896	-1,77	-3,41	0,00854	NM_001004472	LOC146429
8003444	-1,31	-2,49	0,02014	-0,73	-1,66	0,17642	ENST00000333222	---
7978662	-1,31	-2,49	0,00640	-1,92	-3,78	0,01070	---	---
8136215	-1,31	-2,49	0,00376	-1,06	-2,09	0,00343	---	TNFRSF9
7912145	-1,31	-2,49	0,00192	1,31	2,48	0,00064	NM_001561	LOC407835
8136065	-1,31	-2,49	0,00438	-0,98	-1,98	0,09163	NR_002144	ALDH1A2
7989199	-1,31	-2,49	0,01931	-1,07	-2,11	0,02325	NM_003888	GPX6
8124588	-1,31	-2,49	0,04318	-1,11	-2,16	0,02118	NM_182701	TTTY5
8177344	-1,31	-2,49	0,10952	-1,97	-3,93	0,00033	NR_001541	C6orf25
8178074	-1,32	-2,49	0,00455	-1,79	-3,45	0,00862	NM_138277	HCRT
8015572	-1,32	-2,49	0,07989	-1,73	-3,31	0,00015	NM_001524	---
7966568	-1,32	-2,49	0,13200	-1,47	-2,77	0,00865	---	---
8122277	-1,32	-2,49	0,00294	-1,03	-2,04	0,03086	---	IRX2
8110872	-1,32	-2,49	0,04604	-2,03	-4,10	0,00063	NM_033267	HCG27
8177951	-1,32	-2,49	0,11821	-2,00	-4,01	0,07417	NR_026791	C16orf74
8003210	-1,32	-2,49	0,08477	0,12	1,08	0,83434	NM_206967	---
8099142	-1,32	-2,49	0,01190	-1,06	-2,09	0,00119	---	---
8065392	-1,32	-2,49	0,01595	-1,64	-3,11	0,00481	---	---
8001798	-1,32	-2,49	0,10022	-1,46	-2,75	0,00857	---	NFAM1
8076441	-1,32	-2,49	0,00653	-1,04	-2,05	0,08999	NM_145912	TGIF2LX
8168646	-1,32	-2,49	0,01196	-1,47	-2,78	0,01067	NM_138960	---
8066444	-1,32	-2,49	0,12460	0,02	1,02	0,95529	---	---
8120464	-1,32	-2,49	0,03004	-2,17	-4,49	0,00346	---	---
8082406	-1,32	-2,49	0,20797	-0,74	-1,67	0,17056	---	CSN2
8100819	-1,32	-2,49	0,01787	-0,68	-1,60	0,00224	NM_001891	LOC494150
7963261	-1,32	-2,49	0,01864	-0,23	-1,17	0,33527	AK292677	PITX1

8114215	-1,32	-2,49	0,00435	-0,35	-1,27	0,24878	NM_002653		TAAR6
8122131	-1,32	-2,49	0,02205	-0,61	-1,53	0,00059	NM_175067		---
8121741	-1,32	-2,49	0,02408	-0,82	-1,76	0,01621	---	CCL5	
8014316	-1,32	-2,49	0,00425	-1,22	-2,33	0,00207	NM_002985		---
8059648	-1,32	-2,49	0,16541	-1,47	-2,78	0,03641	---	---	
8033460	-1,32	-2,49	0,01971	-1,73	-3,32	0,00848	---	SPDEF	
8125843	-1,32	-2,49	0,04350	-0,62	-1,54	0,14297	NM_012391		C16orf48
8002072	-1,32	-2,49	0,01259	-1,53	-2,88	0,07355	BC008284	FAM27L	
8005739	-1,32	-2,49	0,16426	-1,46	-2,75	0,00279	NR_028336		SEMA4F
8053263	-1,32	-2,49	0,08973	-1,09	-2,13	0,01856	NM_004263		---
7950372	-1,32	-2,50	0,01371	-1,01	-2,01	0,01915	---	CCR6	
8123372	-1,32	-2,50	0,14351	-2,21	-4,62	0,00006	BX647686	DDT	
8071806	-1,32	-2,50	0,00068	-0,18	-1,13	0,54270	NM_001355		RGS9BP
8027480	-1,32	-2,50	0,03064	-0,69	-1,61	0,00063	NM_207391		OR8S1
7955129	-1,32	-2,50	0,01484	-1,45	-2,74	0,01983	NM_001005203		POU5F1
8124889	-1,32	-2,50	0,01337	-1,89	-3,72	0,00008	NM_002701		POU5F1
8179719	-1,32	-2,50	0,01337	-1,89	-3,72	0,00008	NM_002701		LOC100288797
8060533	-1,32	-2,50	0,01917	-0,87	-1,83	0,01121	NM_001167670		PLA2G10
7999588	-1,32	-2,50	0,13655	-0,46	-1,37	0,42971	NM_003561		SPDYE1
8132531	-1,32	-2,50	0,17507	-2,44	-5,44	0,00001	NM_175064		---
8175662	-1,32	-2,50	0,03960	-0,94	-1,92	0,04452	---	OR1L3	
8157684	-1,32	-2,50	0,20436	-1,36	-2,57	0,01818	NM_001005234		---
8163000	-1,32	-2,50	0,27619	-1,39	-2,63	0,03603	---	KCNC1	
7938738	-1,32	-2,50	0,02499	-1,75	-3,35	0,00059	NM_004976		---
7899811	-1,32	-2,50	0,06049	-1,03	-2,04	0,01102	---	KISS1R	
8024070	-1,32	-2,50	0,01982	-0,04	-1,03	0,90992	NM_032551		PTPRN2
8144121	-1,32	-2,50	0,00179	-1,36	-2,58	0,05931	NM_002847		C3orf30
8081822	-1,32	-2,50	0,01740	-1,46	-2,75	0,02399	NM_152539		---
7948139	-1,32	-2,50	0,23756	-1,64	-3,12	0,00278	---	UBQLN4	
8091352	-1,32	-2,50	0,11133	-0,53	-1,45	0,05811	NM_020131		---
8169461	-1,32	-2,50	0,03427	-1,12	-2,17	0,04684	---	PHLPP1	
8023644	-1,32	-2,50	0,00623	-1,29	-2,45	0,03465	ENST00000420025		ADRB2
8109086	-1,32	-2,50	0,00083	-0,53	-1,44	0,14749	NM_000024		---
7995419	-1,32	-2,50	0,00805	-1,76	-3,39	0,01101	---	---	
8063547	-1,32	-2,50	0,02120	-1,61	-3,06	0,00089	---	---	
7917468	-1,32	-2,50	0,24303	-1,03	-2,05	0,08915	---	SCGB2A2	
7940636	-1,32	-2,50	0,04504	-1,90	-3,74	0,00856	NM_002411		SYNPO2L
7934442	-1,32	-2,50	0,00256	-0,33	-1,26	0,01498	NM_001114133		MIR95
8099302	-1,32	-2,50	0,01174	-1,01	-2,01	0,00704	NR_029511		---
8021478	-1,32	-2,50	0,03630	-0,47	-1,38	0,22304	---	---	
7942379	-1,32	-2,50	0,24162	-1,33	-2,51	0,00555	---	---	
8097751	-1,32	-2,50	0,07656	-1,25	-2,38	0,06649	---	ZXDA	
8173201	-1,32	-2,50	0,04111	-3,13	-8,78	0,00023	NM_007156		OR52B2
7946125	-1,32	-2,50	0,05240	-1,75	-3,36	0,00612	NM_001004052		TPRN
8165478	-1,32	-2,50	0,03269	-1,09	-2,13	0,03415	NM_173691		MIR375
8059026	-1,32	-2,50	0,11334	-1,35	-2,56	0,01785	NR_029867		LOC100130274
8153486	-1,32	-2,50	0,06234	-1,23	-2,35	0,01762	NM_001162914		BTC
8101002	-1,32	-2,50	0,00442	-0,28	-1,21	0,26961	NM_001729		---
8111284	-1,32	-2,50	0,08553	-2,47	-5,56	0,00567	---	C19orf63	
8030706	-1,32	-2,50	0,12557	-1,18	-2,27	0,02637	NM_175063		PAGE3
8173168	-1,32	-2,50	0,21086	-0,62	-1,53	0,00331	NR_033460		INGX
8173486	-1,32	-2,50	0,07796	-0,19	-1,14	0,54380	NR_002226		---
8141661	-1,32	-2,51	0,01062	-1,21	-2,32	0,01226	---	---	
7903181	-1,32	-2,51	0,05047	-1,10	-2,14	0,05947	---	ABI3	
8008185	-1,32	-2,51	0,00108	-1,10	-2,14	0,00077	NM_016428		LOC151760
8089464	-1,32	-2,51	0,00392	-1,12	-2,17	0,01850	ENST00000383686		H2AFB1
8171026	-1,33	-2,51	0,00591	-0,73	-1,66	0,00581	NM_001017990		LOC401557
8159235	-1,33	-2,51	0,02742	-1,40	-2,65	0,02439	AK127261	---	
8111122	-1,33	-2,51	0,02073	-1,96	-3,89	0,00110	---	SLC6A4	
8013989	-1,33	-2,51	0,05313	-1,43	-2,69	0,00097	NM_001045		---
8155877	-1,33	-2,51	0,02582	-1,35	-2,55	0,03104	---	CHADL	
8076302	-1,33	-2,51	0,08777	-1,67	-3,17	0,00533	NM_138481		LRRFIP1
8049536	-1,33	-2,51	0,10118	-2,29	-4,88	0,01285	NM_001137550		C5orf20
8114239	-1,33	-2,51	0,04090	-1,09	-2,13	0,05784	NM_130848		ARHGEF15
8004842	-1,33	-2,51	0,01808	-1,31	-2,49	0,01067	NM_173728		MUC12
8135033	-1,33	-2,51	0,51198	-3,62	-12,29	0,00122	NM_001164462		LYZL2
7932864	-1,33	-2,51	0,06429	-1,19	-2,29	0,06971	NM_183058		SPATA3
8049007	-1,33	-2,51	0,10185	-1,78	-3,44	0,01323	NM_139073		WNT4
7913547	-1,33	-2,51	0,06952	-1,00	-2,00	0,04464	NM_030761		IL22RA1
7913768	-1,33	-2,51	0,02459	-0,17	-1,13	0,77272	NM_021258		---
8086876	-1,33	-2,51	0,00399	-1,97	-3,91	0,00480	---	DRGX	
7933501	-1,33	-2,51	0,02650	-1,51	-2,85	0,00031	NM_001080520		TCERG1L
7937059	-1,33	-2,51	0,00275	-1,86	-3,63	0,00053	NM_174937		C17orf106

8010012	-1,33	-2,51	0,01984	0,54	1,46	0,30498	NM_001113324	KRT26
8015078	-1,33	-2,51	0,02875	-0,56	-1,47	0,06386	NM_181539	SCARF1
8011114	-1,33	-2,51	0,01011	-1,07	-2,10	0,00298	NR_028076	---
8150685	-1,33	-2,51	0,00121	-1,22	-2,33	0,08459	---	A4GNT
8090955	-1,33	-2,51	0,00108	-0,94	-1,92	0,00384	NM_016161	VSIG6
7981732	-1,33	-2,51	0,08823	-2,22	-4,64	0,00289	ENST00000338567	---
8106120	-1,33	-2,51	0,10227	-0,04	-1,03	0,96915	---	BCL6B
8004255	-1,33	-2,51	0,04962	-1,11	-2,15	0,00275	NM_181844	---
8136063	-1,33	-2,51	0,04554	-1,65	-3,13	0,00019	---	KIAA1045
8154953	-1,33	-2,51	0,01626	-1,74	-3,34	0,00087	NM_015297	RBM33
8137558	-1,33	-2,51	0,02745	-0,19	-1,14	0,33642	NM_053043	---
8168422	-1,33	-2,51	0,04421	-0,84	-1,79	0,18748	---	CYP2W1
8131056	-1,33	-2,51	0,02313	-1,66	-3,15	0,00138	NM_017781	PRR15L
8016387	-1,33	-2,51	0,09325	-1,08	-2,12	0,08174	NM_024320	---
7973968	-1,33	-2,51	0,02694	-1,02	-2,03	0,01208	---	---
7960848	-1,33	-2,51	0,00026	-1,34	-2,53	0,00023	---	UNQ5830
8050227	-1,33	-2,51	0,06489	-1,50	-2,83	0,05902	AY358123	CABP7
8072266	-1,33	-2,51	0,10824	-1,10	-2,15	0,00228	NM_182527	---
8144582	-1,33	-2,51	0,00685	-1,22	-2,33	0,00195	---	SLC5A11
7994216	-1,33	-2,51	0,05827	-1,16	-2,23	0,00216	NM_052944	THAP8
8036270	-1,33	-2,51	0,02842	-1,00	-2,00	0,00909	NM_152658	ZNF573
8036444	-1,33	-2,51	0,03534	-0,93	-1,90	0,00794	NM_152360	UBXN11
7913918	-1,33	-2,51	0,02559	-0,68	-1,61	0,04385	NM_183008	OTOP2
8009705	-1,33	-2,51	0,07250	-1,38	-2,60	0,00086	NM_178160	NAT2
8144866	-1,33	-2,51	0,00062	-0,56	-1,47	0,04442	NM_000015	GJC2
7910257	-1,33	-2,51	0,06060	-1,38	-2,61	0,07046	NM_020435	CLEC10A
8012013	-1,33	-2,51	0,00638	-1,53	-2,88	0,00890	NM_182906	---
8168115	-1,33	-2,51	0,11832	-1,48	-2,78	0,00074	---	LOC646359
8022426	-1,33	-2,51	0,03148	-1,16	-2,23	0,00995	ENST00000342224	---
8168555	-1,33	-2,51	0,01094	-1,52	-2,87	0,01034	---	MIR1-2
8022500	-1,33	-2,52	0,04116	-1,01	-2,02	0,00221	NR_029662	MON1A
8087568	-1,33	-2,52	0,00362	-0,46	-1,38	0,02929	NM_032355	SPDYE2
8141750	-1,33	-2,52	0,28930	-2,16	-4,46	0,00025	NM_001031618	FAM83F
8073207	-1,33	-2,52	0,02028	-1,12	-2,18	0,18238	NM_138435	---
8043405	-1,33	-2,52	0,00545	-1,51	-2,86	0,06596	---	TBC1D24
7992639	-1,33	-2,52	0,00220	0,08	1,06	0,89728	NM_020705	MIR132
8011218	-1,33	-2,52	0,02928	-1,17	-2,25	0,01172	NR_029674	APOE
8029530	-1,33	-2,52	0,01799	-1,03	-2,04	0,10171	NM_000041	LOC440131
7968323	-1,33	-2,52	0,00135	-1,22	-2,34	0,03377	ENST00000400540	DPPA3
7953665	-1,33	-2,52	0,24062	-1,79	-3,45	0,05807	NM_199286	CHRNA2
8149918	-1,33	-2,52	0,02147	-1,20	-2,30	0,00033	NM_000742	OR7E87P // OR7E87P
7949896	-1,33	-2,52	0,33962	-3,53	-11,54	0,00040	ENST00000454336 // ENST00000454336	UPK1A
8036133	-1,33	-2,52	0,00327	-0,36	-1,29	0,31638	NM_007000	---
8104611	-1,33	-2,52	0,00152	-1,95	-3,87	0,00055	---	SPDYE8P
8133590	-1,33	-2,52	0,19782	-2,54	-5,82	0,00089	NR_003664	EVPL
8018579	-1,33	-2,52	0,04569	-0,53	-1,44	0,23471	NM_001988	HTR3B
7943958	-1,33	-2,52	0,04026	-1,06	-2,09	0,02731	NM_006028	TSPY1
8176524	-1,33	-2,52	0,05033	-1,22	-2,32	0,00002	NM_003308	HDLBP
8049825	-1,33	-2,52	0,07523	-0,87	-1,82	0,04201	NM_005336	---
8043441	-1,33	-2,52	0,13570	-1,76	-3,39	0,01092	---	---
7986338	-1,33	-2,52	0,02046	-1,83	-3,56	0,00020	---	---
8065550	-1,34	-2,52	0,30997	-3,76	-13,56	0,00004	---	GCHFR
7982805	-1,34	-2,52	0,05505	0,19	1,14	0,63537	NM_005258	CAPN12
8036563	-1,34	-2,52	0,00746	-0,64	-1,56	0,06347	NM_144691	---
7961067	-1,34	-2,52	0,02460	-1,21	-2,31	0,00310	---	C20orf141
8060528	-1,34	-2,52	0,00612	-2,46	-5,49	0,00342	NM_080739	MIR200A
7896861	-1,34	-2,52	0,02979	-0,67	-1,59	0,03481	NR_029834	TCP10
8123374	-1,34	-2,52	0,00891	-1,16	-2,24	0,00003	NM_004610	ADAMTS8
7952752	-1,34	-2,52	0,00012	-1,55	-2,93	0,00472	NM_007037	---
8172656	-1,34	-2,52	0,00460	-1,73	-3,33	0,00064	---	ELOVL3
7930025	-1,34	-2,52	0,06199	-0,38	-1,30	0,22717	NM_152310	OR4C12
7948090	-1,34	-2,52	0,00369	-1,52	-2,86	0,00477	NM_001005270	CARD18
7951417	-1,34	-2,52	0,03482	-1,54	-2,92	0,00563	NM_021571	PACRG
8123303	-1,34	-2,52	0,00045	-0,87	-1,82	0,02382	NM_152410	HOXC13
7955845	-1,34	-2,52	0,01327	-0,09	-1,06	0,83455	NM_017410	STMN4
8149898	-1,34	-2,52	0,00268	-1,02	-2,02	0,00276	NM_030795	---
8112157	-1,34	-2,53	0,00256	-1,49	-2,81	0,00096	---	TFCP2L1

8054872	-1,34	-2,53	0,03139	0,89	1,86	0,06537	NM_014553	SLC11A1
8048283	-1,34	-2,53	0,00259	-0,90	-1,86	0,01065	NM_000578	---
7963768	-1,34	-2,53	0,09625	-0,92	-1,89	0,13391	---	FAM178B
8054020	-1,34	-2,53	0,02155	-1,00	-2,01	0,00697	NM_001122646	KATNAL2
8021131	-1,34	-2,53	0,00109	-0,23	-1,18	0,41133	NM_031303	C16orf5
7999160	-1,34	-2,53	0,09064	-1,39	-2,62	0,00181	NM_013399	CYP27B1
7964535	-1,34	-2,53	0,03675	-0,09	-1,06	0,75047	NM_000785	NCRNA00105
8176336	-1,34	-2,53	0,00681	-1,25	-2,38	0,00435	NR_026711	TRPT1
7949046	-1,34	-2,53	0,08049	-0,27	-1,20	0,63934	NM_001033678	MCCD1
8177964	-1,34	-2,53	0,14049	-1,59	-3,01	0,03237	NM_001011700	MCCD1
8179246	-1,34	-2,53	0,14049	-1,59	-3,01	0,03237	NM_001011700	TMEM231
8002897	-1,34	-2,53	0,08966	-1,14	-2,20	0,07165	NM_001077419	ZFPM1
7997767	-1,34	-2,53	0,02325	-2,14	-4,42	0,00038	NM_153813	LDHAL6A
7938800	-1,34	-2,53	0,00246	-1,25	-2,38	0,00409	NM_144972	HOXB8
8016473	-1,34	-2,53	0,00629	-0,84	-1,79	0,04862	NM_024016	---
8081065	-1,34	-2,53	0,01106	-0,63	-1,55	0,01062	---	SIGLEC12
8038852	-1,34	-2,53	0,00378	-1,48	-2,79	0,00003	NM_053003	BZRAP1
8016947	-1,34	-2,53	0,01319	-1,69	-3,22	0,00152	NM_004758	MOGAT1
8048725	-1,34	-2,53	0,05229	-1,01	-2,01	0,06305	NM_058165	LYPD5
8037422	-1,34	-2,53	0,08346	-1,54	-2,91	0,00646	NM_001031749	C19orf21
8024013	-1,34	-2,53	0,01313	1,23	2,35	0,05243	BC052236	KIR2DS4
8031358	-1,34	-2,53	0,10533	-2,68	-6,39	0,00022	NM_012314	LOC100130236
7919301	-1,34	-2,53	0,22235	-2,84	-7,14	0,00801	AK128227	GZMM
8023977	-1,34	-2,53	0,03668	-1,96	-3,90	0,03376	NM_005317	PIK3AP1
7935337	-1,34	-2,53	0,02867	0,23	1,17	0,03354	NM_152309	RIN1
7949592	-1,34	-2,53	0,00411	-0,63	-1,55	0,10000	NM_004292	ILDR1
8089970	-1,34	-2,53	0,02700	-0,07	-1,05	0,84259	NM_175924	---
7922291	-1,34	-2,53	0,00016	-1,13	-2,19	0,05136	---	PCDH20
7971905	-1,34	-2,53	0,05512	-2,09	-4,26	0,00020	NM_022843	KRTAP10-7
8069146	-1,34	-2,53	0,01667	-1,61	-3,04	0,08159	NM_198689	CDRT15
8012887	-1,34	-2,53	0,10855	-0,66	-1,58	0,01983	NM_001007530	---
8013013	-1,34	-2,53	0,01782	-1,35	-2,55	0,03819	---	---
7908155	-1,34	-2,53	0,06611	-2,89	-7,41	0,00386	---	GAD2
7926766	-1,34	-2,53	0,00921	-1,51	-2,86	0,02104	NM_000818	FLJ30679
7997731	-1,34	-2,53	0,05873	-1,45	-2,73	0,00698	NR_026674	C15orf37
7985253	-1,34	-2,53	0,00800	-1,68	-3,20	0,00168	NR_028330	CAPN10
8049702	-1,34	-2,53	0,00278	-1,04	-2,05	0,00730	NM_023083	SLC22A16
8128850	-1,34	-2,53	0,00304	-1,30	-2,45	0,00510	NM_033125	MAPT
8016263	-1,34	-2,53	0,00703	-1,49	-2,80	0,00458	NM_016835	LILRB4
8031238	-1,34	-2,53	0,02165	-1,43	-2,69	0,00237	NM_006847	DDX11
7954655	-1,34	-2,53	0,03280	0,11	1,08	0,81117	NM_030653	LIMS2
8055038	-1,34	-2,53	0,01253	-1,93	-3,80	0,00220	NM_017980	---
7908839	-1,34	-2,53	0,03809	-2,40	-5,29	0,00045	---	ADRA1D
8064779	-1,34	-2,53	0,04423	-1,47	-2,77	0,05830	NM_000678	CCDC51
8086949	-1,34	-2,53	0,00834	-0,89	-1,86	0,06691	NM_024661	CC2D2B
7929541	-1,34	-2,54	0,02236	-0,28	-1,22	0,04271	NM_001159747	---
8147828	-1,34	-2,54	0,02526	-1,32	-2,50	0,05335	---	NLRP9
8039533	-1,34	-2,54	0,10187	-0,87	-1,83	0,00783	NM_176820	---
7904872	-1,34	-2,54	0,02099	-1,15	-2,22	0,00001	---	---
7904951	-1,34	-2,54	0,02099	-1,15	-2,22	0,00001	---	HDGFL1
8117172	-1,34	-2,54	0,01022	-1,82	-3,53	0,00098	NM_138574	CRYBA1
8006019	-1,34	-2,54	0,00630	-1,47	-2,78	0,02593	NM_005208	IGK@
8043360	-1,34	-2,54	0,05969	-1,35	-2,55	0,02509	BC030813	---
[truncated: 257,222 more chars]
